# Thyroid Hormone Receptor Is Essential for Larval Epithelial Apoptosis and Adult Epithelial Stem Cell Development but Not Adult Intestinal Morphogenesis during *Xenopus tropicalis* Metamorphosis

**DOI:** 10.3390/cells10030536

**Published:** 2021-03-03

**Authors:** Yuki Shibata, Yuta Tanizaki, Hongen Zhang, Hangnoh Lee, Mary Dasso, Yun-Bo Shi

**Affiliations:** 1 Section on Molecular Morphogenesis, Eunice Kennedy Shriver National Institute of Child Health and Human Development, National Institutes of Health, Bethesda, MD 20892, USA; yuki.shibata@nih.gov (Y.S.); yuta.tanizaki@nih.gov (Y.T.); 2 Bioinformatics and Scientific Programming Core, Eunice Kennedy Shriver National Institute of Child Health and Human Development (NICHD), National Institutes of Health (NIH), Bethesda, MD 20892, USA; hzhang@mail.nih.gov; 3 Section on Cell Cycle Regulation, Eunice Kennedy Shriver National Institute of Child Health and Human Development, National Institutes of Health, Bethesda, MD 20892, USA; Hangnoh.Lee@som.umaryland.edu (H.L.); dassom@mail.nih.gov (M.D.); 4 Department of Medicine, University of Maryland School of Medicine, Baltimore, MD 21201, USA

**Keywords:** *Xenopustropicalis*, thyroid hormone receptor, transcriptional regulation, amphibian metamorphosis, intestinal remodeling, apoptosis, adult stem cells

## Abstract

Vertebrate postembryonic development is regulated by thyroid hormone (T3). Of particular interest is anuran metamorphosis, which offers several unique advantages for studying the role of T3 and its two nuclear receptor genes, *TR*α and *TRβ*, during postembryonic development. We have recently generated TR double knockout (TRDKO) *Xenopus tropicalis* animals and reported that TR is essential for the completion of metamorphosis. Furthermore, TRDKO tadpoles are stalled at the climax of metamorphosis before eventual death. Here we show that TRDKO intestine lacked larval epithelial cell death and adult stem cell formation/proliferation during natural metamorphosis. Interestingly, TRDKO tadpole intestine had premature formation of adult-like epithelial folds and muscle development. In addition, T3 treatment of premetamorphic TRDKO tadpoles failed to induce any metamorphic changes in the intestine. Furthermore, RNA-seq analysis revealed that TRDKO altered the expression of many genes in biological pathways such as Wnt signaling and the cell cycle that likely underlay the inhibition of larval epithelial cell death and adult stem cell development caused by removing both TR genes. Our data suggest that liganded TR is required for larval epithelial cell degeneration and adult stem cell formation, whereas unliganded TR prevents precocious adult tissue morphogenesis such as smooth-muscle development and epithelial folding.

## 1. Introduction

Plasma thyroid hormone (T3) levels reach a peak around birth, known as the postembryonic period, during human development, when many organs/tissues such as intestine mature into their adult forms. Insufficiency in T3 or inhibition of T3 signaling during postembryonic development causes major developmental defects, including neurological disorders and skeletal abnormalities a [1,2,3] However, the mechanism underlying these defects is still unclear, in part due to the maternal influence and the difficulty to manipulate uterus-enclosed embryos in mammals. Anuran metamorphosis is the most dramatic morphological transformation regulated by T3 but without maternal influence. The formation of many adult organs during metamorphosis resembles that during postembryonic development in mammals [4,5] Of particular interest is intestinal remodeling, which involves degeneration of larval epithelium via apoptosis and de novo formation of adult intestinal stem cells, followed by their proliferation and differentiation to form a self-renewing adult intestinal epithelium as seen in mammals [6,7,8].

The effect of T3 is mediated mainly through transcriptional regulation via thyroid hormone receptors (TRs), which form heterodimers with retinoic X receptors (RXRs) and recruit cofactor complexes such as those containing steroid receptor coactivators (SRCs) or protein arginine methyltransferase 1 (PRMT1) to target genes [5,9,10,11,12,13,14]. In vertebrates including pseudo-tetraploid species *Xenopus laevis* and diploid species *Xenopus tropicalis*, two TR genes, *TRα* and *TR®*, exist and have distinct spatiotemporal expression patterns. Earlier studies in *Xenopus laevis* led to a dual-function model for TRs during anuran development [15,16]. That is, during premetamorphosis (up to stage 54), when there is little or no T3, TR/RXR heterodimers recruit corepressor complexes to repress T3-inducible genes and prevent precocious development of adult tissues, whereas in the presence of T3 during metamorphosis (between stage 54 and stage 66, the end of metamorphosis when tail is completely resorbed), TRs bind to T3 and liganded TRs activate T3 target genes and induce metamorphosis. This model has subsequently been substantiated through various molecular and transgenic studies in *Xenopus laevis* [16,17,18,19,20,21,22,23,24,25,26,27,28,29,30,31].

With the development of gene-editing technologies and the advancement in genome annotation for diploid *Xenopus tropicalis,* we and others have generated single (*TR*α or *TR®*) or double TR knockout (TRDKO) animals that have not only supported the dual-function model, but also provided novel details on the role of TRs during *Xenopus* development [32,33,34,35,36,37,38,39,40,41,42,43,44,45,46]. Interestingly, single TR knockout tadpoles could complete metamorphosis with delayed developmental progression of different organs, suggesting compensation between *TRα* and *TR®*. Surprisingly, analyses of TRDKO tadpoles have shown that TRs are not required for tadpole development up to stage 61, the climax of metamorphosis [42], indicating that many metamorphic events can occur without TR. On the other hand, such tadpoles are then developmentally stalled at stage 61 and die after about two weeks, while wild-type tadpoles at stage 61 complete metamorphosis within one week, indicating an essential role of TR for the completion of metamorphosis [42]. The TRDKO tadpoles precociously develop most, if not all, adult tissues, such as limbs. Surprisingly, larval organ degeneration, e.g., tail and gill resorption, is blocked or inhibited in TRDKO tadpoles.

While these earlier analyses, which focused on external organs, revealed interesting effects of TR knockouts, particularly on larval tissue degeneration, it remains unclear if TRs are required for metamorphosis of internal organs, particularly those that exist in both tadpoles and frogs. To address this, we have analyzed the effects of TR double knockout on the intestine, an internal organ where both larval tissue degeneration and adult tissue development take place, mostly during the climax of metamorphosis between stages 58-66 [6]. We show that in the intestine of TRDKO tadpoles, larval epithelial cell death is also inhibited. Interestingly, a multiply folded, adult-like intestinal epithelium is precociously formed during metamorphosis despite the lack of de novo formation of adult epithelial stem cells and their subsequent proliferation. RNA-seq analysis of the intestine of wild-type and TRDKO tadpoles at premetamorphic stage 54 and climax stage 61 (before the death of TRKDO animals, likely due to failure of some key organs resulted from the inability to degenerate larval tissues [42]) suggests that the precocious adult epithelial fold formation in premetamorphic TRDKO tadpole intestine was mediated at least in part via increased expression of extracellular matrix organization-related genes, likely due to their de-repression caused by TR knockout. On the other hand, failure to upregulate genes related to cell death and stem cells in response to T3 might be responsible for the lack of cell death or adult stem-cell development in TRDKO tadpole intestine. Our data suggest that unliganded TR prevents precocious maturation of adult intestinal morphology, while liganded TR is essential for larval tissue degeneration and adult stem cell development during intestinal metamorphosis.

## 2. Materials and Methods

### 2.1. Experimental Animals

Wild-type adult *Xenopus tropicalis* were purchased from Nasco (Fort Atkinson, WI, USA). Embryos and tadpoles were staged according to [47]. All animal care and treatments were performed as approved by the Animal Use and Care Committee of the Eunice Kennedy Shriver National Institute of Child Health and Human Development.

### 2.2. Generation of TR Double Knockout Xenopus tropicalis Animals and Genotyping

TRDKO animals (*TR*α*^(−/−)^TR® ^(−/−)^*) were generated from *TR*α*^(−/−)^TR® ^(^^+^^/^^−)^* male and female frogs [42]. In our previous study, we used one mutant line that contained a 19-base out-of-frame deletion in the *TR®* gene, and we subsequently obtained another line with a 29-base deletion in the *TR®* gene, in the same *TR*α knockout background. Both lines were used in the current study. Briefly, female and male mutant frogs were primed with 20 U of human chorionic gonadotropin (hCG: Novarel; FerringPharmaceuticals Inc., Parsippany, NJ, USA) one day before egg laying. They were then boosted with another injection of 200 U of human chorionic gonadotropin on the second day for natural mating. The resulting fertilized eggs/embryos were collected and reared for 3–4 days at 25 °C to reach the onset feeding (stage 45). The tadpoles were then transferred to a 4-L container and fed.

Tadpoles were anesthetized with MS222 (Ethyl 3-aminobenzonate methanesulfonate, Sigma-Aldrich, St. Louis, MO, USA) for photography, tail clipping, and body-length measurement. For genotyping, tadpole tail tip (about 5 mm or less) was clipped and lysed in 20 μL QuickExtract DNA extraction solution (EPICENTRE Biotechnologies, Madison, WI, USA) at 65 °C for 20 min. After incubating at 95 °C for 5 min, 1 μL of the DNA extraction solution was immediately used for genotyping by PCR and sequencing [42]. The genotyping of *TRβ* were done by PCR with forward primer 2, 5′-GGACAACATTAGATCTTTCTTTCTTTG-3′ and reverse primer 2, 5′-CACACCACGCATAGCTCATC-3′ for the 19-base deletion in the *TRβ* gene [42]; or with forward primer 2, 5′- TCAATGGAACCCTTTGGAGCTG -3′ and reverse primer 2, 5′- ACAGTTACAGGCATTTCCAGGC -3′ for the 29-base deletion in the *TRβ* gene (Table S1), for 33 cycles of 94 °C for 30 s, 60 °C for 30 s, and 72 °C for 20 s. The PCR products were analyzed by gel electrophoresis.

### 2.3. T3 Treatment

For long-term T3 treatment, 5 tadpoles of different genotypes at stage 54 were pooled together in 4-L plastic containers and treated with or without 5 nM T3 for 5 days at 25 °C, with half of the rearing water replaced every day with water containing 5 nM T3. The tadpole tail tip (about 5 mm or less) was cut for genotyping, and the intestine of each animal was fixed with 4% PFA/PBS at 4 °C overnight, then transferred to 70% ethanol. The fixed intestine samples of the same genotype were mounted together in paraffin block for immunohistochemistry.

### 2.4. RNA Extraction and qRT-PCR

Total RNA was isolated with RNeasy^®^ Mini Kit 250 (QIAGEN, Hilden, Germany). The homogenates of individual tissues from at least five animals, or five whole animals, of each genotype were combined together for RNA extraction. The RNA concentration was measured by using a NanoDrop (Thermo Fisher Scientific, Waltham, MA, USA). The RNA from each genotype was reverse-transcribed with the QuantiTect reverse transcription kit (QIAGEN). The cDNA was analyzed by using the SYBR Green-based qPCR method. The PCR primers for the internal control gene *rpl8* were described previously [36,37]. All expression data were normalized against that of the internal control gene *rpl8*. The expression analyses were performed at least twice, with consistent findings. The primer sequences are listed in Tables S2 and S3.

### 2.5. 5-Ethynyl-2-Deoxyuridine (EdU) Labeling

EdU staining was done as before [48]. Briefly, EdU was injected into tadpoles; 30 min after injection, the tadpoles were sacrificed, and the intestine was fixed in 4% PFA/PBS and processed for paraffin-sectioning. Tissue sections cut at 5 μm were subjected to EdU staining by using the Click-iT Plus EdU Alexa Fluor 594 Imaging kit (Thermo Fisher Scientific, Waltham, MA, USA). EdU positive areas in epithelium were measured by using ImageJ software (National Institutes of Health, Bethesda, MD, USA).

### 2.6. TUNEL Assays

TUNEL (terminal deoxyribonucleotidyl transferase-mediated dUTP-biotin nick end labeling) assays were done by using the In Situ Cell Death Detection Kit (Roche Diagnostics, Mannheim, Germany) [49]. The fluorescent pictures for different colors and different sections were taken under the same settings and then analyzed by using ImageJ software at the same settings to measure the TUNEL-positive cell area.

### 2.7. Methyl Green-Pyronin Y (MGPY) Staining

Tissue sections were stained with MGPY (Muto, Tokyo, Japan), a mixture of methyl green, which binds strongly to DNA, and pyronin Y, which binds strongly to RNA, for 5 min at room temperature according to the supplier’s instructions. Adult epithelial stem/progenitor cells were intensely stained red because of their RNA-rich cytoplasm [48].

### 2.8. Immunohistochemistry

Paraffin-embedded intestinal sections from wild-type tadpoles at stage 54, 58, 61, and 66 or TRDKO tadpoles at stage 54, 58, and 61 were deparaffinized in xylene, rehydrated in a series of different concentrations of ethanol, and microwaved in the sodium citrate antigen retrieve buffer (10 mM sodium citrate, 0.05% Tween 20, pH 6.0) for 10 min. After 15 min incubation at room temperature, the sections were washed in 0.7 × PBS and then incubated with mouse anti-smooth muscle actin antibody (diluted 1:200; Sigma-Aldrich) for 1 h at room temperature. After washing in 0.7 × PBS several times, the sections were incubated with Alexa flour 568 goat anti-mouse IgG (diluted 1:1000; Molecular probe, Eugene, OR, USA) and Hoechst 33342 (diluted 1:1000; Invitrogen, Carlsbad, CA, USA) for 1 h at room temperature and mounted with mount medium (Vector Laboratories Inc., Burlingame, CA, USA). The fluorescence pictures for different colors and different sections were taken under the same settings.

### 2.9. Whole Transcriptome Sequencing (RNA-Seq)

Total RNA of the intestine at stage 54 and stage 61 from *TR*α*^(+/+)^TR® ^(+/+)^* and *TR*α*^(−/−)^TR® ^(−/−)^* animals was extracted with RNeasy^®^ Mini Kit 250 (QIAGEN) and submitted to NICHD Molecular Genomics Core for library construction of poly-A-selected fragments of the entire transcriptome with the TruSeq Stranded Total RNA Library Prep Kit (Illumina Inc., San Diego, CA, USA). Three biological replica RNA samples were prepared for RNA-seq analysis on a HiSeq2500 (Illumina).

The demultiplexed and adapter-removed short reads were mapped with START software (version 2.6.1c) to the *Xenopus tropicalis* genome (Ensemble_release_v94). *Xenopus tropicalis*.JGI_4.2.93.gtf was used for extracting splice junctions to improve accuracy of the mapping. All advanced parameters used the default. The read counts for each gene/exon were obtained with the featureCounts tool of Subread software (version 1.6.3). The raw fastq data were deposited to the NCBI GEO database with accession number GSE161714. Ensembl *Xenopus* IDs were changed into human IDs to increase the hit counts [50], and DESeq2 [51] was used for differential gene-expression analysis (FDR < 0.05). Bioinformatics analysis, gene ontology (GO) and KEGG pathway were performed with the DAVID bioinformatics resource (https://david.ncifcrf.gov, accessed on 25 February 2021) [52]. The R “pathview” package (v1.22.3) was used to visualize the KEGG pathway [53].

To compare the transcript expression levels among the samples by heatmap, the reads for each transcript were normalized to yield the number of sequenced fragments per kilobase of transcript sequence per million base pairs sequenced (FPKM) for each sample. Log10(FPKM + 1) values were used for each tested transcript with MultiExperiment Viewer (MeV v4.9.0) software [54].

### 2.10. Statistical Analysis

Data are presented as mean ± SE. The significance of differences between groups was evaluated by one-way ANOVA test followed by Bonferroni multiple-test correction or Student’s *t*-test by using Prism 5 (GraphPad Software, La Jolla, CA, USA).

## 3. Results

### 3.1. TR Double Knockout Blocks Intestinal Length Reduction During Metamorphosis

**Figure 1:**
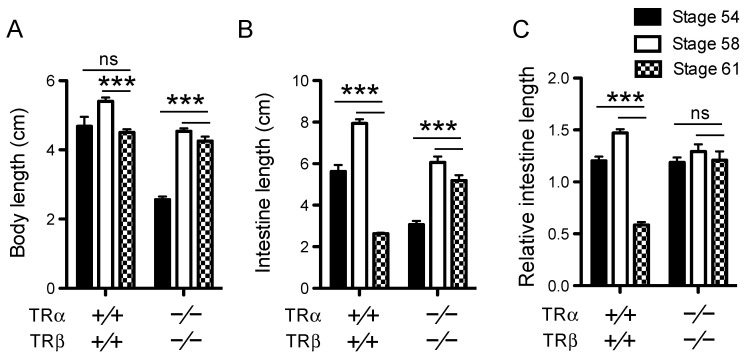
TR double knockout inhibits natural intestine shortening at the climax of metamorphosis. The total body length (**A**) and the intestine length (**B**) were measured for wild-type and TRDKO tadpoles at the indicated stages. The relative intestine length (**C**) was obtained by normalizing the intestine length against the total body length at the indicated stages. At least 4 tadpoles were analyzed for each sample. Asterisks (***) indicate a significant difference vs. stage 58 for the 2 genotypes (*p* < 0.001, Bonfferoni comparison test). ns: No significant difference.

One of the most dramatic organ transformations during anuran metamorphosis is intestinal remodeling. The larval *Xenopus* intestine is a simple tubular structure made mostly of a single layer of larval epithelium surrounded by thin layers of connective tissue and muscles [6,55]. During metamorphosis, the larval epithelial cells undergo apoptotic degeneration and concurrently, adult epithelial stem cells are formed de novo, accompanied by a up to 90% reduction in the length of the small intestine [6,48,55,56,57]. The stem cells actively proliferate around the climax stages 61–63 and subsequently differentiate to form a multiply folded adult epithelium surrounded by thick layers of connective tissue and muscles. We have recently generated *Xenopus tropicalis* animals lacking both TR genes, and such animals could develop to stage 61 [42] (Figure S1). Since much of the intestinal remodeling has begun by stage 61 in wild type animals, including larval epithelial cell death and intestinal length reduction [6,48,55,56], we analyzed the effects of TR double knockout on intestinal remodeling. We first compared the length of the small intestine (duodenum + ileum) of wild-type and TRDKO tadpoles at stages 54–61. As shown in Figure 1, the intestine in the wild type tadpoles was expectedly the longest at stage 58 but reduced 2–3-fold by stage 61. In contrast, the length of the intestine of the TRDKO tadpoles changed little from stage 54 to stage 61. In addition to this TRDKO line, which has a 19 bp out-of-frame deletion in *TRβ*, we also analyzed an independent line of TRDKO animals with a 29 bp out-of-frame deletion in *TRβ* with the same finding (Figure S1D). Thus, intestinal length reduction, the most noticeable change during intestinal metamorphosis, requires TR-mediated signaling.

### 3.2. Abnormal Intestinal Development in TRDKO Xenopus tropicalis Tadpoles

**Figure 2:**
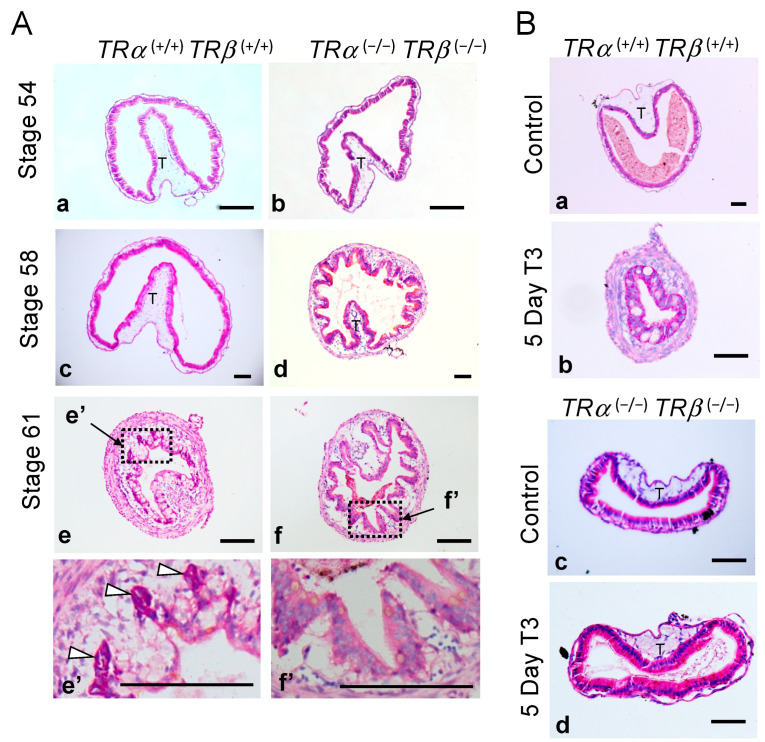
TRDKO tadpoles have abnormal intestinal morphology with apparent adult type epithelial folding by stage 58. (**A**) Abnormal intestinal morphology in TRDKO tadpoles. Cross-sections of the intestine isolated from tadpoles at the indicated stages were stained with MGPY (methyl green-pyronin Y). (**a**,**c**,**e**): Wild-type *TRα ^(+/+)^TRβ ^(+/+)^*; and (**b**,**d**,**f**): TR double knockout (TRDKO*) TRα ^(−/−)^TRβ ^(−/−)^*. Dashed boxes in **e** and **f** for stage 61 are shown in higher magnification in **e’** and **f’**, respectively. White arrowheads point to the clusters of proliferating adult epithelial stem cells adjacent to/underlying the degenerating larval epithelium (vacuole-like, poorly stained) at the climax of metamorphosis (stage 61) in wild-type animals. Note that the knockout tadpoles lacked such clusters at stage 61 and the epithelium appeared to be uniform without any obvious degeneration throughout the stages, but with numerous folds by stage 58. T: typhlosole. Bars: 100 μm. (**B**) TRDKO intestine does not have T3-induced tissue remodeling. Cross-sections of the intestine isolated from stage 54 tadpoles with or without 5 days T3 treatment were stained with MGPY. (**a**,**b**): *TRα ^(+/+)^TRβ ^(+/+)^*; and (**c**,**d**): *TRα ^(−/−)^TRβ ^(−/−)^*. Note that after T3 treatment, the wild-type intestine resembled that at stage 61 (climax of intestinal remodeling), while no T3-induced changes were present in the TRDKO tadpole intestine. T: typhlosole. Bars: 100 μm.

We next investigated intestinal morphology by staining cross-sections of the small intestine with methyl green pyronin Y (MGPY), a mixture of methyl green, which binds to DNA, and pyronin Y, which binds to RNA and thus labels the proliferating cells very strongly but the apoptotic cells poorly [48,58,59]. The wild-type intestine at stages 54 and 58 was mainly composed of a monolayer of larval epithelial cells surrounded by thin connecting tissue and muscles, with only a single epithelial fold, the typhlosole (Figure 2A, a,c).

At stage 61, as the larval epithelial cells underwent apoptosis, they became poorly labeled, while clusters of strongly labeled proliferating adult stem cells (white arrowheads in Figure 2A, e,e’) were located between the connective tissue and dying larval epithelial cells [48]. The intestine of TRDKO tadpoles at stage 54, while shorter than wild-type intestine (Figure 1), had a cross-section morphology that resembled that in the wild-type stage 54 tadpoles (Figure 2A, b vs. a). However, by stage 58, the intestine in TRDKO tadpoles had numerous epithelial folds and its connective tissue was also thicker than that in the wild-type intestine at stage 58 (Figure 2A, d vs. c). This morphological difference was also observed in another TRDKO line (TRDKOΔ29) (Figure S1E). At stage 61, the intestine in TRDKO tadpoles had even more developed epithelial folds and connective tissue even though intestinal length reduction was inhibited in the TRDKO animals compare to wild-type ones (Figure 1). In addition, it lacked any identifiable adult stem cell clusters as seen in the wildtype stage 61 tadpoles (Figure 2A, f,f’). Such an intestinal morphology resembles the intestine of wild-type tadpoles at the end of metamorphosis [48,55,59].

To examine the effect of TR knockout on T3-induced metamorphosis, premetamorphic tadpoles at stage 54 were treated with exogenous T3 for 5 days and the intestine was analyzed. As expected, we observed T3-induced intestinal remodeling in the wild-type tadpoles, including increased thickness of the connective tissue and muscle layers, and presence of intensely stained clusters of adult stem cells and vacuole-like, poorly stained apoptotic larval epithelial cells (Figure 2B, b vs. a). However, no T3-induced changes were observed in the TRDKO tadpoles (Figure 2B, d vs. c), indicating an essential role of TR to mediate T3 response in the intestine. It is also worth pointing out that the intestinal cross-section became smaller after T3-treatment of the wild-type tadpoles (Figure 2B, b vs. a; note the different scale bars), similar to what happened during natural metamorphosis (Figure 2A, c vs. e; again note the different scale bars), likely due to larval epithelial degeneration and muscle contraction. Such changes were also absent in the TRDKO tadpoles (Figure 2).

**Figure 3:**
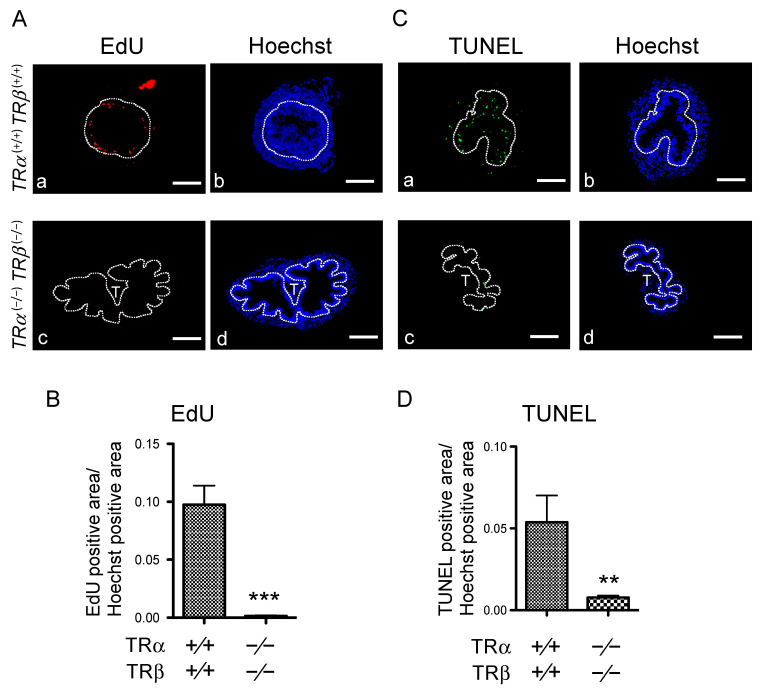
TRDKO tadpoles have little or no cell proliferation or apoptosis at the climax of metamorphosis. (**A**,**B**) TRDKO inhibits epithelial cell proliferation. (**A**) Cross-sections of the intestine isolated from wild type (**a**,**b**) and TRDKO (**c**,**d**) tadpoles at stage 61 preinjected with EdU to label proliferating cells were stained for the presence of EdU. The dotted lines depict the epithelium–mesenchyme boundary, drawn based on morphological differences between epithelial cells and mesenchyme cells in the pictures of the stained tissues. EdU, red-color (**a**,**c**) and Hoechst, blue-color (**b**,**d**). T: typhlosole. Bars: 100 μm. (**B**) Quantification of the EdU positive area from at least 3 tadpoles for each genotype. The statistical significance of differences was determined by Student’s *t*-test. ***, *p* < 0.0001. (**C**,**D**) TRDKO inhibits larval epithelial cell death. (**C**) Cross-sections of the intestine isolated from wild type (**a**,**b**) and TRDKO (**c**,**d**) tadpoles at stage 61 were subjected to TUNEL labeling for apoptotic cells and Hoechst 33342 staining for DNA. The dotted lines depict the epithelium–mesenchyme boundary, drawn based on morphological differences between epithelial cells and mesenchyme cells in the pictures of the stained tissues. TUNEL: green, and Hoechst: blue. T: typhlosole. Bars: 100 μm. (**D**) Quantification of TUNEL-positive area from at least 3 tadpoles for each genotype. The statistical significance of differences was determined by Student’s *t*-test. **, *p* < 0.001.

To further investigate the effects of TR double knockout, we analyzed larval epithelial cell death and adult stem cell proliferation in the intestine during metamorphosis. When we carried out EdU staining to detect proliferating cells at stage 61, we observed little EdU staining in the epithelium of TRDKO intestine, while strong cell-proliferation signals were detected in the wild-type intestine (Figure 3A,B). In addition, TUNEL-labeling for apoptotic cells revealed numerous dying cells in the wild-type intestine, but few in the TRDKO intestine at stage 61 (Figure 3C,D). These findings are consistent with the morphological analysis with MGPY-staining above (Figure 2), and indicate that both larval epithelial cell death and adult stem cell formation/proliferation require TR-dependent T3-signaling.

**Figure 4:**
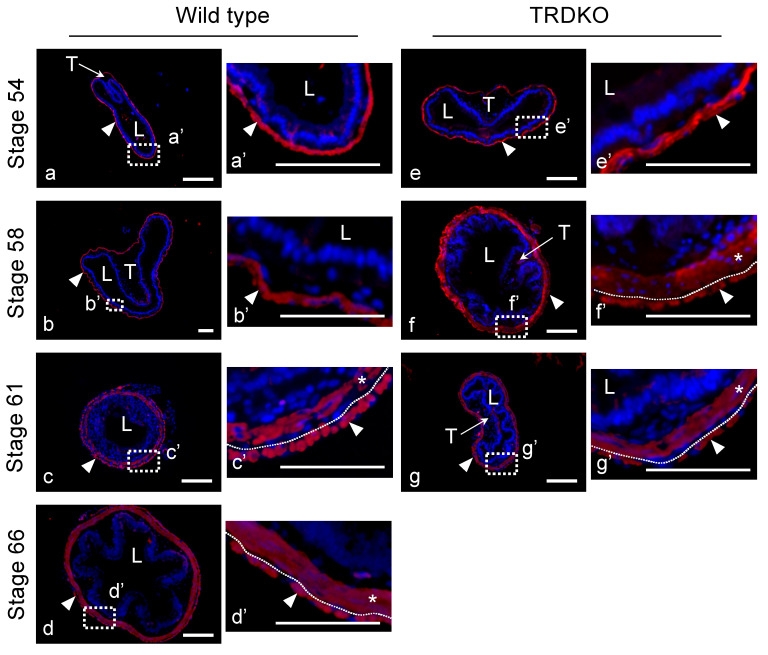
TRDKO accelerates intestinal muscle development during metamorphosis. Cross-sections of the intestine isolated from wild type (**a**–**d**) and TRDKO (**e**–**g**) tadpoles at stage 54 (**a** and **e**), stage 58 (**b** and **f**), stage 61 (**c** and **g**), and stage 66 (**d**) were stained with α-smooth actin (α-SMA, red) to label muscle cells in the nonepithelial cell layer. Dashed boxes in the left panels are shown at higher magnification in the right panels for each genotype, respectively. The dotted lines depict the muscularis–serosa boundary, drawn based on morphological differences between muscle cells and serosa cells in the pictures of the stained tissues. α-SMA, red-color and Hoechst, blue-color for DNA staining. T: typhlosole and L: lumen. Arrowheads: serosa, and asterisks: Muscularis. Shown are representative from at least 3 tadpoles analyzed for each genotype at different stages. Note that the precocious muscle development, indicated by *, in TRDKO tadpoles by stage 58 compared to the wild-type tadpoles. Bars: 100 μm.

The morphological analysis above indicated premature formation of the adult epithelial folds in the intestine of TRDKO animals, with an obvious premature increase in the connective tissue under the folds. Furthermore, immunohistochemical staining for α-smooth actin showed that smooth muscle under the connective tissue was easily detected by stage 61 in both wild-type and knockout animals (Figure 4). Interestingly, it could be detected as early as stage 58 in TRDKO tadpoles, but not in the wild-type animals (Figure 4b’,f’), indicating premature development of the smooth muscle due to TR double knockout. Thus, removing TRs led to premature epithelial folding and development of the connective tissue and muscles, but prevented larval epithelial cell death and adult epithelial stem cell development.

### 3.3. Gene Regulation Programs Underlying the Observed Abnormal Intestinal Development in TRDKO Tadpoles

**Figure 5:**
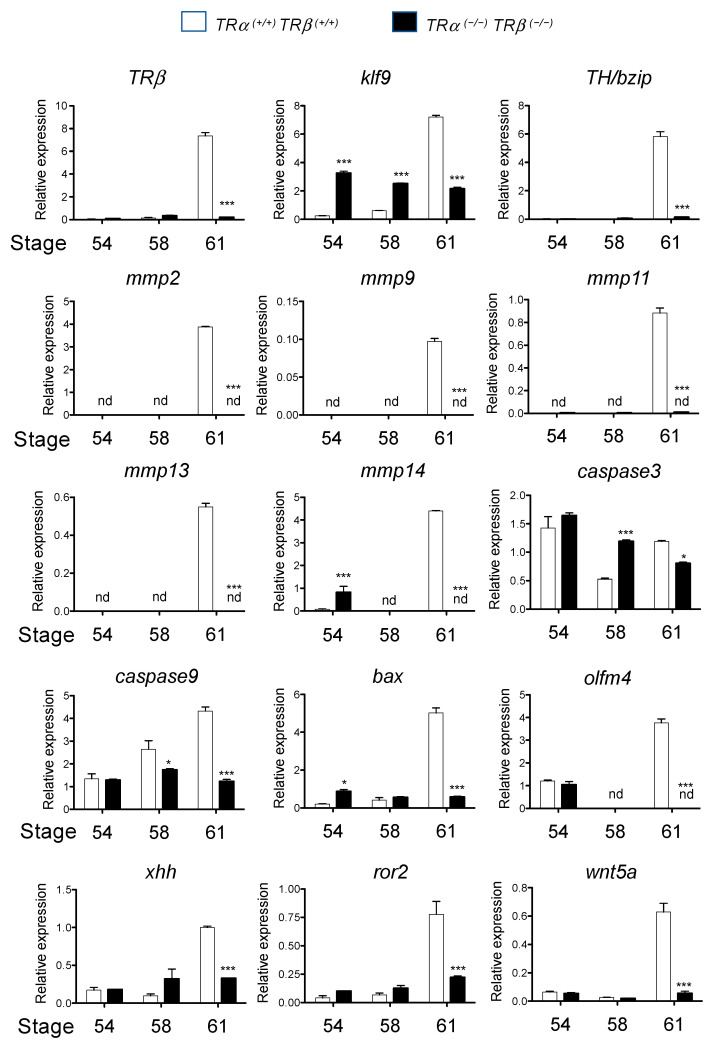
T3 response genes fail to be upregulated in the intestine of TRDKO tadpoles during metamorphosis. Total RNA was isolated from the intestine of wild-type and TRDKO tadpoles at stage 54, 58, and 61, and used for real-time RT-PCR analysis of the expression of known T3 direct-target genes: *TR®*, *klf9*, and *TH/bzip*; genes related to extracellular matrix remodeling: *mmp2*, *mmp9*, *mmp11*, *mmp13,* and *mmp14*; apoptosis-related genes: *caspase 3*, *caspase 9,* and *bax*; adult stem cell marker genes: sonic hedgehog (*xhh*) and *olfm4*, and cell dedifferentiation-related genes: *ror2* and *wnt5a*. The mRNA levels were normalized against that of *rpl8*. The groups included 5 wild-type and 8 TRKDO animals. Note that the expression of all genes that are regulated during intestinal remodeling in the wild-type animals was essentially unchanged in the TRDKO animals. Asterisks (* and ***) indicate a significant difference determined by Bonfferoni comparison test (*p* < 0.05 and *p* < 0.001). nd: Not detected.

To investigate the gene-expression programs that underly the morphological differences due to TR double knockout, we first analyzed the expression of some known T3 response genes during intestinal metamorphosis. As shown in Figure 5, T3-upregulated genes, including several transcription factor genes (*TR®*, *klf9,* and *TH/bzip*) and matrix metalloproteinase (MMP) genes (*mmp2*, *mmp9*, *mmp11*, *mmp13,* and *mmp14*), which encode proteases capable of cleaving extracellular matrix components [60,61], were not upregulated in the intestine of TRDKO tadpoles at stage 61, in contrast to the strong upregulation observed at the climax of metamorphosis (stage 61) in wild-type tadpoles (Figure 5). 

Analyses of several apoptosis-related genes (*caspase 3*, *caspase 9,* and *bax*), adult stem cell-associated genes (*xhh* and *olfm4*), and dedifferentiation-related genes (*ror2* and *wnt5a*) showed that they were also not upregulated in the intestine of TRDKO tadpoles in contrast to their upregulation in the wild-type intestine during metamorphosis (Figure 5). These results suggest that gene activation by liganded TR is critical for adult epithelial stem cell development and larval epithelial apoptosis.

**Figure 6:**
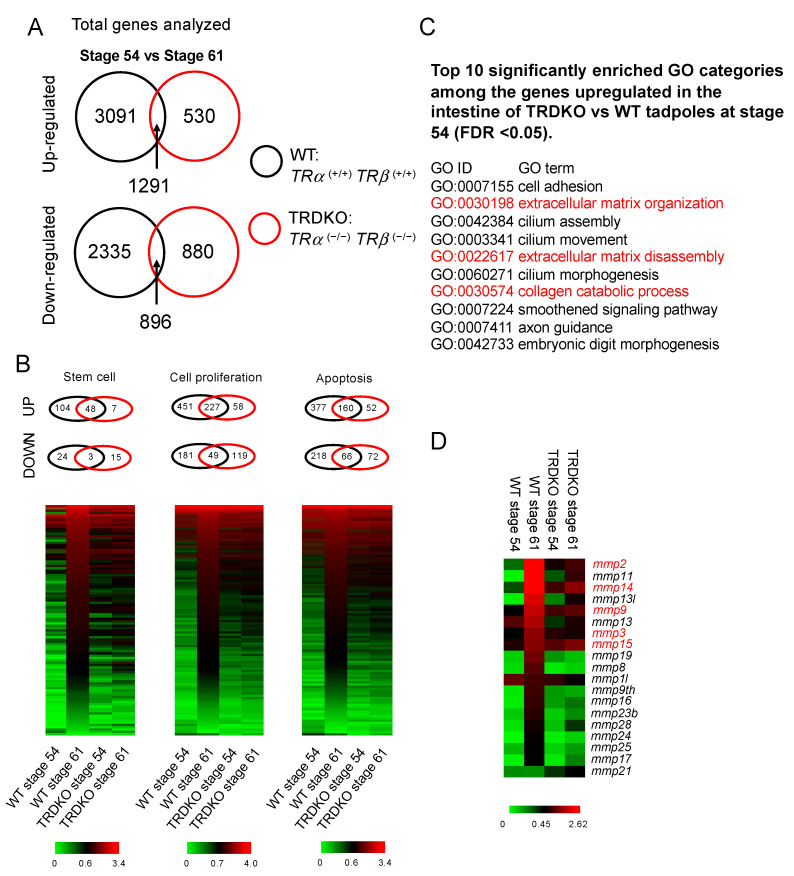
TR double knockout inhibits the upregulation of genes in the GO categories involved in major intestinal remodeling processes: stem cell, cell proliferation, and apoptosis, at stage 61 during metamorphosis, but derepresses genes in ECM remodeling at premetamorphic stage 54. (**A**) Venn diagram analysis of genes regulated during intestinal metamorphosis in wild-type and TRDKO tadpoles shows that the vast majority of the regulated genes are different between the wild-type and TRDKO animals. RNA-seq was carried out on intestinal RNA from wild-type and TRDKO tadpoles at premetamorphic stage 54 and climax stage 61. Genes up- or down-regulated at stage 61 compared to stage 54 (2 folds or more) were identified for each genotype and compared. Note that there were much fewer genes regulated in the TRDKO animals. (**B**) Venn diagrams and heat maps reveal that TRDKO tadpoles have drastically reduced number of regulated genes at stage 61 in the three GO categories critical for intestinal remodeling: stem cell, cell proliferation, and apoptosis. Top: All genes in each of the three GO categories were identified from the up- and down-regulated genes in each genotype as found in (**A**) and compared by Venn diagram analysis between the two genotypes. Note that again most of the genes did not overlap between the two genotypes. Bottom: Heat maps of the expression of all genes in each GO category at the two stages for the two genotypes. Note the coordinated upregulation of most of the genes in each GO category at stage 61 compared to stage 54 in the wild-type intestine but not in the TRDKO intestine. (**C**) GO analysis reveals that GO categories related to ECM remodeling (shown in red) are significantly enriched among genes expressed at higher levels in the stage 54 intestine of TRDKO tadpoles compared to the wild-type ones. The RNA-seq data from (A) were used to identify genes that were expressed at higher levels (2-fold or more) in the stage 54 intestine of TRDKO tadpoles compared to the wild type ones. The 3187 genes thus identified were subjected to GO analysis, and the top 10 enriched GO terms are shown. (**D**) Heat maps of the expression of MMP genes at the two developmental stages (54, or premetamorphosis, and 61, or metamorphic climax) for the two genotypes. Note that essentially all MMPs genes were upregulated at stage 61 compared to stage 54 in wild-type but not TRDKO intestine and many of the MMPs genes were expressed at higher levels (2-fold or more) in the TRDKO intestine compared the wild-type intestine at stage 54 (genes in red), suggesting that they were derepressed upon TR knockout.

We next carried out a genome-wide RNA-seq analysis by using whole intestine samples from wild-type and TRDKO tadpoles at stage 54 (a premetamorphic stage) and stage 61 (climax of metamorphosis). We identified a total of 19,921 transcripts assembled against Ensembl genome database of *Xenopus tropicalis* (Table S4). The assembled transcripts’ Ensembl IDs were converted into human Ensembl genome IDs (85% or 16,293/19,921 of transcripts were converted) for GO and KEGG pathway analyses. Pair-wise comparisons between stage 54 and stage 61 of either genotypes were performed, leading to the identification of 4382 or 3231 genes significantly up- or down-regulated between stage 54 and stage 61, respectively, in the wild-type tadpoles (FDR < 0.05, log 2 > ±1) (Figure 6A, Table S5). For the TRDKO tadpoles, 1821 or 1776 genes were found to be significantly up- or down-regulated, respectively, between stage 54 and stage 61 (FDR <0.05, log 2 > ±1) (Figure 6A, Table S5). To validate the RNA-seq data, we selected 7 genes with changes of 10 folds or more during metamorphosis in wild-type or TRDKO animals for RT-PCR analysis and found that the RT-PCR data for all were consistent with the RNA-seq data (Figures S2 and S3).

Among these developmentally regulated genes, 1291 upregulated and 896 downregulated genes were common between the two genotypes (Figure 6A). These results showed that 3091 upregulated genes and 2335 downregulated genes were unique to the wild-type tadpoles and that their regulation between stage 54 and stage 61 required T3 signaling via TRs.

Given that major transformations at the climax of intestinal metamorphosis (stage 61) involve larval epithelial cell death, de novo formation of the adult epithelial stem cells and their subsequent proliferation [6], we next analyzed the genes in gene ontology (GO) categories related to stem cells, cell proliferation, and apoptosis. Venn diagram analyses showed that many more genes in these GO categories were upregulated in the wild-type animals than TRDKO tadpoles and only 31.6% (48/152, stem cell), 33.5% (227/678, cell proliferation), and 29.8% (160/537, apoptosis) of these upregulated genes in the three GO categories, respectively, in the wild-type animals were upregulated in TRDKO tadpoles (Figure 6B). These findings suggest that the failure in the upregulation of genes in these GO categories underlie the observed lack of larval cell death, stem cell clusters, and cell proliferation in the TRDKO animals during metamorphosis.

The premature formation of the adult intestine-like structure by stage 58–61, i.e., the folding of the intestinal epithelium and the development of the connective tissue and muscles (Figure 2 and Figure 3), suggests that some intestinal metamorphic processes took place precociously in the knockout animals. To investigate the underlying gene regulation programs, we compared the gene expression profiles in the intestine of wild-type and TRDKO tadpoles at stage 54 or stage 61 (Table S6). We found that 1834 or 3187 genes were significantly downregulated or upregulated, respectively, in the stage 54 intestine of TRDKO tadpoles compared to the wild-type ones (FDR < 0.05, log 2 > ±1) (Table S6). Since TR double-knockout was expected to cause derepression of T3-inducible genes and thus premature metamorphosis, we focused on genes that were upregulated in the knockout animals compared to wild-type animals at stage 54. GO analysis revealed the enrichment of GO categories related to intestinal remodeling, particularly cell proliferation and ECM remodeling, among these upregulated genes (Table S7). Among the most significantly enriched GO categories were those related to the extracellular matrix (ECM), e.g., ECM organization, ECM disassembly, and collagen catabolic process, etc. (Table S7, Figure 6C). Of particular interest were the genes encoding ECM remodeling/degrading enzymes, i.e., the matrix metalloproteases (MMPs), which were highly upregulated during intestinal metamorphosis in wild-type animals at stage 61 (Figure 6D). Several of them were expressed at higher levels in stage 54 knockout tadpole intestine (Figure 6D), suggesting that they, together with other genes associated with ECM GO categories, may participate in the premature ECM remodeling associated with formation of the adult intestine-like structure in the knockout animals by stages 58–61.

**Figure 7:**
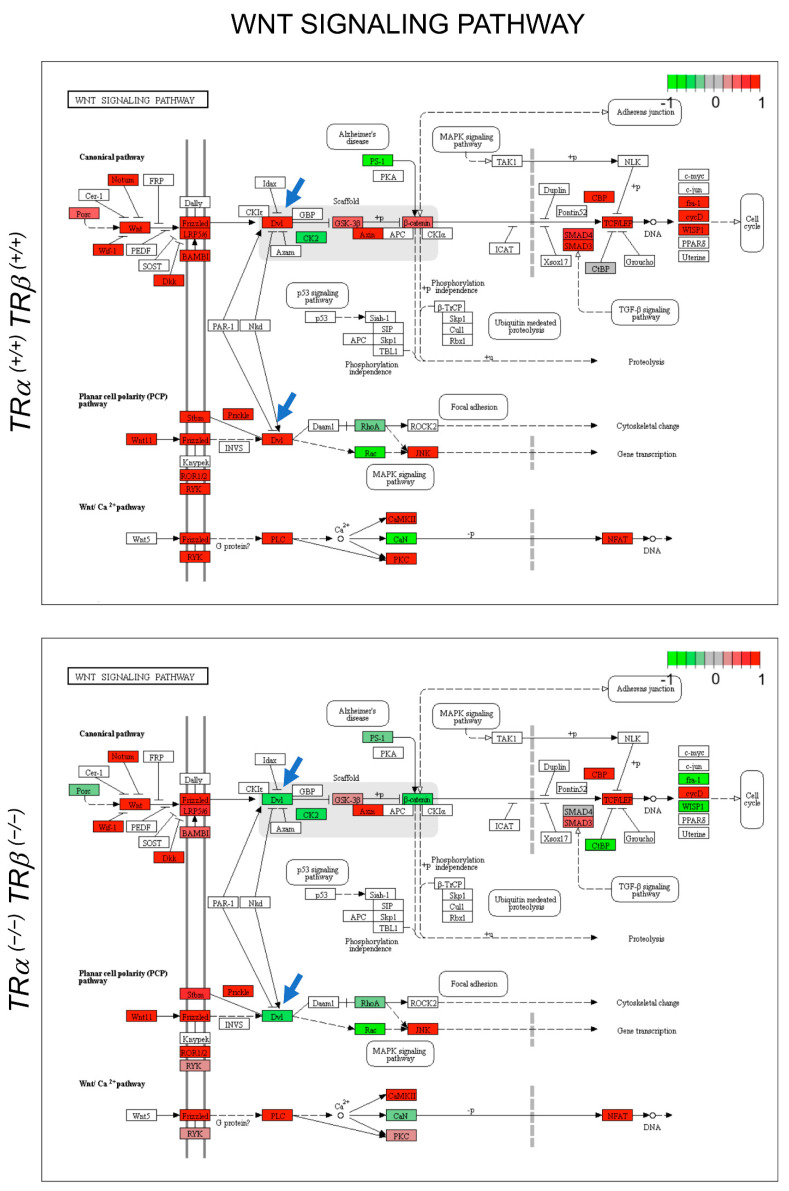
TR-dependent regulation of genes in the Wnt signaling pathway, a representative pathway likely involved in the development of adult intestinal stem cells, as revealed by RNA-seq analysis. Up- and down-regulated genes in the intestine at stage 61 compared to stage 54 in each genotype are shown in red and green, respectively (color keys, log2 fold change). Note that many more genes were upregulated (red) at stage 61 in the wild-type tadpoles (Top) compared to TRDKO animals, and the reverse was true for down-regulated genes (green). Two blue arrows point to *dvl* genes, which are known as the hub of Wnt signaling critical for relaying Wnt signal from receptors to downstream effectors [62] that were upregulated at stage 61 in the wild-type animals (*dvl2* and *dvl3*), but downregulated at stage 61 in the TRDKO animals (*dvl1*).

When we carried out GO analyses of genes that were regulated between stage 54 and stage 61 in WT animals, we found that genes in the GO categories such as cell adhesion/ECM organization, cell cycle/proliferation, stem cells, etc., were enriched among the up-regulated genes, while those associated with GO categories such as translational initiation, translation, rRNA processing, and nuclear-transcribed mRNA catabolic processes, nonsense-mediated decay, etc., were significantly enriched among the downregulated genes (Table S8). These findings are consistent with the metamorphic changes, i.e., larval epithelial degeneration, development/proliferation of adult stem cells, and epithelial morphogenesis/folding, that take place during intestinal remodeling. In the TRDKO animals, cell adhesion/ECM-related GO categories were also enriched among genes upregulated at stage 61, likely contributing to the epithelial folding that still occurred in the intestine. However, many other GO categories, particularly cell cycle/proliferation-related ones, were not enriched among the upregulated genes (Table S8). Interestingly, a number of cell cycle/proliferation-related GO categories were enriched among genes downregulated at stage 61 in the TRDKO animals (Table S8). All these are consistent with the lack of larval epithelial degeneration and adult epithelial stem cell development and proliferation during intestinal metamorphosis in the TRDKO tadpoles. In addition, KEGG pathway analyses also revealed distinct pathways enriched among genes that were up- or down-regulated during metamorphosis in the wild-type or knockout animals (Table S9). Of interest were a few significantly up-regulated pathways in the wild-type animals, including the Wnt signaling pathway (Figure 7) and Notch signaling pathway (Figure S4), where many more genes were up-regulated and fewer genes were down-regulated in the wild-type animals than those in the TRDKO animals. Such pathways are very likely important for adult stem cell formation/proliferation during intestinal metamorphosis, and the effects on these pathways by TR double knockout may be responsible for the lack of stem cell formation/proliferation in the knockout animals.

To further examine the effect of TRDKO on intestinal gene expression program during metamorphosis, we carried out GO analysis on the genes expressed at higher levels in wild-type than on TRDKO intestine at stage 61. Again, we found that many cell-cycle related GO categories were enriched among these genes (Table S10). KEGG pathway analysis also showed that the cell-cycle pathway was enriched among genes expressed at higher levels in the wildtype intestine at stage 61 (Table S11, Figure S5), supporting the involvement of TR-dependent upregulation of the cell-cycle program in stem-cell proliferation during intestinal metamorphosis.

## 4. Discussion

Frog metamorphosis has long been used as an excellent model to investigate vertebrate development, including organ formation and adult stem cell development, largely because it is controlled by T3 without maternal influence [4,63,64]. Of particular special interest is intestinal remodeling, which involves degeneration of the larval epithelium via apoptosis and de novo formation of the adult intestinal stem cells in an organ autonomous, T3-dependent manner, thus offering an opportunity to study how adult organ-specific stem cells are formed during vertebrate development. Recent knockout studies on individual *Xenopus tropicalis* TR genes, *TR*α or *TR®*, have revealed important gene- and organ-specific roles during *Xenopus* metamorphosis [32,33,34,35,36,37,38,39,40,41,43,46]. Interestingly, the two TR genes can compensate for each other to enable tadpoles to complete metamorphosis in the absence of either TR gene despite their distinct expression profiles during natural development. More recently, we have shown that knocking out both TR genes leads to precocious initiation of metamorphosis, and that animals without TR can develop to the climax of metamorphosis when many organ-remodeling processes take place [42]. These double-knockout tadpoles are subsequently stalled at the climax stage 61 before eventual death without any significant tail resorption [42]. We have reported here the first analysis of the effects of TR double knockout on the natural metamorphosis of an internal organ. Our results indicate that both liganded and unliganded TRs are essential for temporal coordination of the transformations of different tissues during intestinal remodeling.

### 4.1. TRα and TR® Are the Only Receptors Mediating Intestinal Epithelial Remodeling by T3 in Xenopus tropicalis

During intestinal metamorphosis, the vast majority of the larval epithelial cells undergo apoptosis, starting around stage 60 [6,48,56]. Concurrently, adult epithelial stem cells develop de novo around stage 60–62, likely through dedifferentiation of some larval epithelial cells, followed by their subsequent proliferation and differentiation to form a multifolded epithelium surrounded by elaborate connective tissue and muscles [48,65,66,67,68]. During this period, the intestinal length also shortens dramatically in response to endogenous T3. Interestingly, in the TRDKO tadpoles, the intestine shortening was absent in two independent TRDKO lines with different out-of-frame mutations in the *TR®* gene (Figure 1A, Figure S1D). In addition, unlike wild-type intestine at the climax of metamorphosis, the intestine in the TRDKO tadpoles had little or no larval epithelial cell death or adult epithelial cell proliferation at the climax of metamorphosis (stage 61). Furthermore, T3-treatment of premetamorphic tadpoles failed to induce any changes in the TRDKO intestine, but induced intestinal remodeling in the wild-type animals. Thus, *TR*α and *TR®* are the only TRs for mediating the metamorphic effects of T3 in the intestine.

### 4.2. Liganded TR Induces Larval Epithelial Cell Death and Adult Stem Cell Development

Our earlier *TR*α or *TR®* single-knockout studies showed that T3-induced apoptosis and adult stem cell proliferation were drastically reduced when either TR gene was knocked out [43,44], suggesting both TR genes are important for intestinal remodeling. Consistent with an essential role of TRs for intestinal remodeling, both cell death and adult epithelial cell proliferation were essentially absent in the intestine of TRDKO tadpoles at stage 61 when they were at or near peak levels in the wild-type intestine. Molecularly, we found that the expression of genes involved in apoptosis and stem-cell formation and/or proliferation was strongly upregulated at stage 61, the climax of metamorphosis, in the intestine of wild-type but not TRDKO tadpoles. Furthermore, our RNA-seq analyses also revealed that GO categories related to stem cells, cell proliferation, and apoptosis were highly enriched among the genes up-regulated between stage 54 (premetamorphosis) and stage 61 (metamorphic climax) in the wild-type intestine. Most of these genes, however, were not regulated during metamorphosis in the TRDKO tadpoles. KEGG pathway analyses similarly showed that the regulation of many genes in pathways known to be important for stem cells, such as Wnt and Notch pathways, or both stem cells and apoptosis, such as cell-cycle pathways [44], are highly regulated in the wild-type but not or in opposite fashion in the TRDKO animals during metamorphosis. For example, *dvl*, a key player in the Wnt pathway, is up-regulated at the climax of metamorphosis (stage 61) in the wild-type animals but is down-regulated in the TRDKO tadpoles (Figure 7). Previous studies have consistently reported that inhibition of Wnt and Notch signaling inhibits *Xenopus* intestinal metamorphosis in *Xenopus laevis* [69,70]. Clearly, it will be interesting to investigate the roles of these pathways in regulating larval cell death and/or adult stem cell development. In addition, it remains to be seen whether TR knockout affects the development of adult stem cells in other tissues/organs.

### 4.3. Unliganded TR Prevents Precocious Adult Epithelial Morphogenesis and Smooth Muscle Development During Intestinal Remodeling

According to the dual-function model for TRs during anuran development, unliganded TRs repress target gene expression by recruiting co-repressors such as NCOR and SMRT to prevent precocious metamorphosis [15,16,71]. Not surprisingly, TR double knockout in *Xenopus tropicalis* causes increased expression of many T3-target genes in premetamorphic tadpoles and precocious initiation of metamorphosis, as revealed largely based on limb development [42]. Our analysis here showed that intestine, an internal organ, also underwent some precocious changes upon knocking out both TR genes. The most noticeable changes are the premature folding of the epithelium and development of smooth muscles as revealed by immunohistochemical staining of α-smooth muscle actin. These adult-like features were well developed by stage 58 in the TRDKO animals, similar to what was observed at stage 61 in the wild-type animals. In addition, the connective tissue and muscle layers were also thicker and more developed in the TRDKO animals than in the wild-type animals by stage 58.

Mechanistically, our RNA-seq analyses revealed that many genes in the GO categories related to ECM remodeling, which is likely required for epithelial folding, were de-repressed in the TRDKO tadpoles at stage 54 (Table S9). In addition, we have previously shown that treatment of tadpoles with cyclopamine, an inhibitor of signaling pathway induced by the T3-direct target gene sonic hedgehog (*xhh*), leads to longer intestine, i.e., reduced intestinal length shortening, and strongly inhibits muscle development during intestinal remodeling [72]. Thus, in addition to ECM remodeling, the derepression of the sonic hedgehog gene due to TR double knockout likely contributes the precocious muscle development via hedgehog signaling pathways. Clearly, unliganded TRs are critical to maintain normal tadpole development and prevent premature metamorphosis in different organs/tissues.

## 5. Conclusions

Our findings here have revealed an essential role of TRs for intestinal remodeling during anuran metamorphosis. It was shown earlier that single *TR*α or *TR®* knockouts lead to delayed progression of intestinal remodeling, including reduced larval cell death and adult epithelial cell proliferation [32,33,34,35,36,37,38]. Importantly, our studies here show that TR double knockout completely blocks intestinal length shortening, larval cell death, and adult stem cell development, demonstrating a requirement for TR in both larval tissue cell death and adult intestinal development. Furthermore, TR double knockout also induces precocious development of the non-epithelial tissues and adult epithelial morphogenesis. Our RNA-seq analysis has revealed that the activation of a broad spectrum of pathways related to the cell cycle, notch signaling, sonic hedgehog signaling, and Wnt signaling is blocked by TR double knockout at the climax of metamorphosis and may be collectively responsible for the lack of larval epithelial cell death and adult epithelial stem cell development and proliferation in the knockout tadpoles. On the other hand, the precocious upregulation of extracellular matrix remodeling pathways and derepression of the hedgehog signaling in premetamorphic TRDKO tadpoles may underlie their premature adult epithelial morphogenesis and muscle development. These findings further strengthen the dual-function model for TRs during *Xenopus* metamorphosis by revealing a critical role of unliganded TR in repressing premature adult tissue development and an essential role of liganded TR in larval tissue degeneration and adult stem cell formation. In addition, the role of T3 signaling in intestinal development may be conserved since mouse intestinal maturation also takes place during postembryonic development when plasma T3 levels peak [8,73,74,75], and TR mutations and T3 deficiency lead to intestinal defects in the adult, including reduced stem cell proliferation [49,76,77,78,79,80], suggesting that TR is also important for intestinal maturation in mammals.

## Data Availability

RNA-seq data were deposited at Gene Expression Omnibus (GEO), NCBI (GSE161714).

## Supplementary Materials

The following are available at https://www.mdpi.com/2073-4409/10/3/536/s1. Figure S1. TR double-knockout tadpoles with a homozygous 29 base-out-frame deletion (14 base in exon and 15 base in intron) of *TR®* gene locus shows similar intestinal remodeling defects as TR double-knockout tadpoles with a homozygous 19 base-out-frame mutation of *TR®* gene locus. Figure S2. Validation of RNA-seq data for the upregulated genes. Figure S3. Validation of RNA-seq data for the down-regulated genes. Figure S4. A representative Notch signaling pathway likely involved in the development of adult intestinal stem cells as revealed by RNA-seq analysis. Figure S5. A representative cell-cycle signaling pathway likely involved in the proliferation of adult intestinal stem cells as revealed by RNA-seq analysis. Table S1. Primers for genotyping to detect a 29 base-out-frame (14-base deletion in exon and 15-base deletion in the intron) mutation at the *TR®* locus. Table S2. Primers used in qRT-PCR. Table S3. Primers used in qRT-PCR validation of the Xenopus RNA-seq data. Table S4A. Ensembl RNA-seq data. Table S4B. RNA-seq data in FPKM. Table S5A. Genes upregulated between stages 54 and 61 in TRDKO animals. Table S5B. Genes down-regulated between stages 54 and 61 in TRDKO animals. Table S5C. Genes down-regulated between stages 54 and 61 in wild type animals. Table S5D. Genes up-regulated between stages 54 and 61 in wild-type animals. Table S6A. Genes expressed at higher level in the wild type than TRDKO at stages 54. Table S6B. Genes expressed at higher level in the TRDKO than wild type at stages 54. Table S6C. Genes expressed at higher level in the TRDKO than wild type at stages 61. Table S6D. Genes expressed at higher level in the wild type than TRDKO at stages 61. Table S7. Gene ontology (GO) categories enriched among genes expressed at higher levels in TRDKO than wild-type intestine at stage 54 (*p* < 0.05). Table S8A. Gene ontology (GO) categories enriched among genes up-regulated between stages 54 and 61 in wild-type intestine (*p* < 0.05). Table S8B. Gene ontology (GO) categories enriched among genes down-regulated between stages 54 and 61 in wild-type intestine (*p* < 0.05). Table S8C. Gene ontology (GO) categories enriched among genes upregulated between stages 54 and 61 in TRDKO intestine (*p* < 0.05). Table S8D. Gene ontology (GO) categories enriched among genes down-regulated between stages 54 and 61 in TRDKO intestine (*p* < 0.05). Table S9A. Enriched KEGG pathways among genes up-regulated between stages 54 and 61 in wild-type intestine (*p* < 0.05). Table S9B. Enriched KEGG pathways among genes down-regulated between stages 54 and 61 in wild-type intestine (*p* < 0.05). Table S9C. Enriched KEGG pathways among genes up-regulated between stages 54 and 61 in TRDKO intestine (*p* < 0.05). Table S9D. Enriched KEGG pathways among genes down-regulated between stages 54 and 61 in TRDKO intestine (*p* < 0.05). Table S10. Gene ontology (GO) categories enriched among genes expressed at higher levels in wild-type than TRDKO intestine at stage 61 (*p* < 0.05). Table S11. Enriched KEGG pathways among genes expressed at higher levels in wild-type than TRDKO intestine at stage 61 (*p* < 0.05).

## References

[1] Hetzel B.S. (1989). The story of Iodine Deficiency: An. International Challenge in Nutrition.

[2] Vermiglio F., Presti V.P.L., Moleti M., Sidoti M., Tortorella G., Scaffidi G., Castagna M.G., Mattina F., Violi M.A., Crisà A. (2004). Attention Deficit and Hyperactivity Disorders in the Offspring of Mothers Exposed to Mild-Moderate Iodine Deficiency: A Possible Novel Iodine Deficiency Disorder in Developed Countries. J. Clin. Endocrinol. Metab..

[3] de Escobar G.M., Obregon M.J., del Rey F.E. (2007). Iodine deficiency and brain development in the first half of pregnancy. Public Health Nutr..

[4] Tata J.R. (1993). Gene expression during metamorphosis: An ideal model for post-embryonic development. Bioessays.

[5] Shi Y.-B. (1999). Amphibian Metamorphosis: From Morphology to Molecular Biology.

[6] Shi Y.-B., Ishizuya-Oka A. (1996). Biphasic intestinal development in amphibians: Embryogensis and remodeling during metamorphosis. Curr. Top. Dev. Biol..

[7] Bao L., Shi B., Shi Y.-B. (2020). Intestinal homeostasis: A communication between life and death. Cell Biosci..

[8] Sun G., Shi Y.-B. (2012). Thyroid Hormone Regulation of Adult Intestinal Stem Cell Development: Mechanisms and Evolutionary Conservations. Int. J. Biol. Sci..

[9] Wong J., Shi Y.B., Wolffe A.P. (1995). A role for nucleosome assembly in both silencing and activation of the Xenopus TR beta A gene by the thyroid hormone receptor. Genes Dev..

[10] Lazar M.A. (1993). Thyroid hormone receptors: Multiple forms, multiple possibilities. Endocr. Rev..

[11] Yen P.M. (2001). Physiological and Molecular Basis of Thyroid Hormone Action. Physiol. Rev..

[12] Evans R.M. (1988). The steroid and thyroid hormone receptor superfamily. Science.

[13] Tsai M.J., O’Malley B.W. (1994). Molecular mechanisms of action of steroid/thyroid receptor superfamily members. Ann. Rev. Biochem..

[14] Laudet V., Gronemeyer H. (2002). The Nuclear Receptor Facts Book.

[15] Shi Y.-B., Wong J., Puzianowska-Kuznicka M., Stolow M.A. (1996). Tadpole competence and tissue-specific temporal regulation of amphibian metamorphosis: Roles of thyroid hormone and its receptors. BioEssays.

[16] Sachs L.M., Damjanovski S., Jones P.L., Li Q., Amano T., Ueda S., Shi Y.-B., Ishizuya-Oka A. (2000). Dual functions of thyroid hormone receptors during Xenopus development. Comp. Biochem. Physiol. Part. B Biochem. Mol. Biol..

[17] Schreiber A.M., Das B., Huang H., Marsh-Armstrong N., Brown D.D. (2001). Diverse developmental programs of Xenopus laevis metamorphosis are inhibited by a dominant negative thyroid hormone receptor. Proc. Natl. Acad. Sci. USA.

[18] Brown D.D., Cai L. (2007). Amphibian metamorphosis. Dev. Biol..

[19] Buchholz D.R., Hsia S.-C.V., Fu L., Shi Y.-B. (2003). A Dominant-Negative Thyroid Hormone Receptor Blocks Amphibian Metamorphosis by Retaining Corepressors at Target Genes. Mol. Cell. Biol..

[20] Buchholz D.R., Tomita A., Fu L., Paul B.D., Shi Y.-B. (2004). Transgenic Analysis Reveals that Thyroid Hormone Receptor Is Sufficient To Mediate the Thyroid Hormone Signal in Frog Metamorphosis. Mol. Cell. Biol..

[21] Buchholz D.R., Paul B.D., Fu L., Shi Y.-B. (2006). Molecular and developmental analyses of thyroid hormone receptor function in Xenopus laevis, the African clawed frog. Gen. Comp. Endocrinol..

[22] Shi Y.-B. (2009). Dual Functions of Thyroid Hormone Receptors in Vertebrate Development: The Roles of Histone-Modifying Cofactor Complexes. Thyroid.

[23] Nakajima K., Yaoita Y. (2003). Dual mechanisms governing muscle cell death in tadpole tail during amphibian metamorphosis. Dev. Dyn..

[24] Denver R.J., Hu F., Scanlan T.S., Furlow J.D. (2009). Thyroid hormone receptor subtype specificity for hormone-dependent neurogenesis in Xenopus laevis. Dev. Biol..

[25] Bagamasbad P., Howdeshell K.L., Sachs L.M., Demeneix B.A., Denver R.J. (2008). A role for basic transcription element-binding protein 1 (BTEB1) in the autoinduction of thyroid hormone receptor beta. J. Biol. Chem..

[26] Schreiber A.M., Mukhi S., Brown D.D. (2009). Cell–cell interactions during remodeling of the intestine at metamorphosis in Xenopus laevis. Dev. Biol..

[27] Shi Y.-B. (1994). Molecular biology of amphibian metamorphosis: A new approach to an old problem. Trends Endocrinol. Metab..

[28] Shi Y.-B., Matsuura K., Fujimoto K., Wen L., Fu L. (2012). Thyroid hormone receptor actions on transcription in amphibia: The roles of histone modification and chromatin disruption. Cell Biosci..

[29] Grimaldi A., Buisine N., Miller T., Shi Y.-B., Sachs L.M. (2013). Mechanisms of thyroid hormone receptor action during development: Lessons from amphibian studies. Biochim. Biophys. Acta—Gen. Subj..

[30] Puzianowska-Kuznicka M., Damjanovski S., Shi Y.B. (1997). Both thyroid hormone and 9-cis retinoic acid receptors are required to efficiently mediate the effects of thyroid hormone on embryonic development and specific gene regulation in Xenopus laevis. Mol. Cell. Biol..

[31] Sachs L.M., Shi Y.-B. (2000). Targeted chromatin binding and histone acetylation in vivo by thyroid hormone receptor during amphibian development. Proc. Natl. Acad. Sci. USA.

[32] Choi J., Ishizuya-Oka A., Buchholz D.R. (2017). Growth, Development, and Intestinal Remodeling Occurs in the Absence of Thyroid Hormone Receptor α in Tadpoles of Xenopus tropicalis. Endocrinology.

[33] Choi J., Suzuki K.I., Sakuma T., Shewade L., Yamamoto T., Buchholz D.R. (2015). Unliganded thyroid hormone receptor alpha regulates developmental timing via gene repression as revealed by gene disruption in Xenopus tropicalis. Endocrinology.

[34] Sakane Y., Iida M., Hasebe T., Fujii S., Buchholz D.R., Ishizuya-Oka A., Yamamoto T., Suzuki K.-I.T. (2018). Functional analysis of thyroid hormone receptor beta in Xenopus tropicalis founders using CRISPR-Cas. Biol. Open.

[35] Nakajima K., Tazawa I., Yaoita Y. (2018). Thyroid Hormone Receptor alpha- and beta-Knockout Xenopus tropicalis Tadpoles Reveal Subtype-Specific Roles During Development. Endocrinology.

[36] Wen L., Shi Y.-B. (2015). Unliganded Thyroid Hormone Receptor α Controls Developmental Timing in Xenopus tropicalis. Endocrinol..

[37] Wen L., Shibata Y., Su D., Fu L., Luu N., Shi Y.-B. (2017). Thyroid Hormone Receptor α Controls Developmental Timing and Regulates the Rate and Coordination of Tissue-Specific Metamorphosis in Xenopus tropicalis. Endocrinology.

[38] Wen L., Shi Y.B. (2016). Regulation of growth rate and developmental timing by Xenopus thyroid hormone receptor alpha. Dev. Growth Differ..

[39] Sachs L.M. (2015). Unliganded Thyroid Hormone Receptor Function: Amphibian Metamorphosis Got TALENs. Endocrinology.

[40] Yen P.M. (2015). Unliganded TRs regulate growth and developmental timing during early embryogenesis: Evidence for a dual function mechanism of TR action. Cell Biosci..

[41] Nakajima K., Tazawa I., Shi Y.B. (2019). A unique role of thyroid hormone receptor beta in regulating notochord resorption during Xenopus metamorphosis. Gen. Comp. Endocrinol..

[42] Shibata Y., Wen L., Okada M., Shi Y.-B. (2020). Organ-Specific Requirements for Thyroid Hormone Receptor Ensure Temporal Coordination of Tissue-Specific Transformations and Completion ofXenopusMetamorphosis. Thyroid.

[43] Shibata Y., Tanizaki Y., Shi Y.-B. (2020). Thyroid hormone receptor beta is critical for intestinal remodeling during Xenopus tropicalis metamorphosis. Cell Biosci..

[44] Tanizaki Y., Shibata Y., Zhang H., Shi Y.-B. (2021). Analysis of Thyroid Hormone Receptor α-Knockout Tadpoles Reveals That the Activation of Cell Cycle Program Is Involved in Thyroid Hormone-Induced Larval Epithelial Cell Death and Adult Intestinal Stem Cell Development During Xenopus tropicalis Metamorphosis. Thyroid.

[45] Tanizaki Y., Bao L., Shi B., Shi Y.-B. (2020). A role of endogenous histone acetyltransferase steroid hormone receptor coactivator (SRC) 3 in thyroid hormone signaling during Xenopus intestinal metamorphosis. Thyroid.

[46] Shi Y.-B. (2021). Life without thyroid hormone receptor. Endocrinology.

[47] Nieuwkoop P.D., Faber J. (1965). Normal Table of Xenopus laevis.

[48] Okada M., Wen L., Miller T.C., Su D., Shi Y.-B. (2015). Molecular and cytological analyses reveal distinct transformations of intestinal epithelial cells during Xenopus metamorphosis. Cell Biosci..

[49] Bao L., Roediger J., Park S., Fu L., Shi B., Cheng S.-Y., Shi Y.-B. (2019). Thyroid Hormone Receptor Alpha Mutations Lead to Epithelial Defects in the Adult Intestine in a Mouse Model of Resistance to Thyroid Hormone. Thyroid.

[50] Nagasawa K., Tanizaki Y., Okui T., Watarai A., Ueda S., Kato T. (2013). Significant modulation of the hepatic proteome induced by exposure to low temperature in Xenopus laevis. Biol. Open.

[51] Love M.I., Huber W., Anders S. (2014). Moderated estimation of fold change and dispersion for RNA-seq data with DESeq2. Genome Biol..

[52] Huang Da W., Sherman B.T., Lempicki R.A. (2009). Systematic and integrative analysis of large gene lists using DAVID bioinformatics resources. Nat. Protoc..

[53] Luo W., Brouwer C. (2013). Pathview: An R/Bioconductor package for pathway-based data integration and visualization. Bioinformatics.

[54] Howe E.A., Sinha R., Schlauch D., Quackenbush J. (2011). RNA-Seq analysis in MeV. Bioinformatics.

[55] Sterling J., Fu L., Matsuura K., Shi Y.-B. (2012). Cytological and Morphological Analyses Reveal Distinct Features of Intestinal Development during Xenopus tropicalis Metamorphosis. PLoS ONE.

[56] Shi Y.-B., Ishizuya-Oka A. (2000). Thyroid hormone regulation of apoptotic tissue remodeling: Implications from molecular analysis of amphibian metamorphosis. Prog. Nucleic Acid Res. Mol. Biol..

[57] Schreiber A.M., Cai L., Brown D.D. (2005). Remodeling of the intestine during metamorphosis of Xenopus laevis. Proc. Natl. Acad. Sci. USA.

[58] Ishizuya-Oka A., Ueda S. (1996). Apoptosis and cell proliferation in the Xenopus small intestine during metamorphosis. Cell Tissue Res..

[59] Ishizuya-Oka A., Ueda S., Damjanovski S., Li Q., Liang V.C., Shi Y.-B. (1997). Anteroposterior gradient of epithelial transformation during amphibian intestinal remodeling: Immunohistochemical detection of intestinal fatty acid-binding protein. Dev. Biol..

[60] Page-McCaw A., Ewald A.J., Werb Z. (2007). Matrix metalloproteinases and the regulation of tissue remodelling. Nat. Rev. Mol. Cell Biol..

[61] Fu L., Hasebe T., Ishizuya-Oka A., Shi Y.-B. (2007). Roles of Matrix Metalloproteinases and ECM Remodeling during Thyroid Hormone-Dependent Intestinal Metamorphosis in Xenopus laevis. Organogenesis.

[62] Gao C., Chen Y.-G. (2010). Dishevelled: The hub of Wnt signaling. Cell. Signal..

[63] Morvan-Dubois G., Demeneix B.A., Sachs L.M. (2008). Xenopus laevis as a model for studying thyroid hormone signalling: From development to metamorphosis. Mol. Cell. Endocrinol..

[64] Takashi H., Hasebe T., Fu L., Fujimoto K., Ishizuya-Oka A. (2011). The development of the adult intestinal stem cells: Insights from studies on thyroid hormone-dependent amphibian metamorphosis. Cell Biosci..

[65] Ishizuya-Oka A., Hasebe T., Buchholz D.R., Kajita M., Fu L., Shi Y. (2009). Origin of the adult intestinal stem cells induced by thyroid hormone in Xenopus laevis. FASEB J..

[66] Ishizuya-Oka A., Hasebe T. (2013). Establishment of Intestinal Stem Cell Niche During Amphibian Metamorphosis. Curr. Top. Dev. Biol..

[67] Okada M., Shi Y.-B. (2018). The balance of two opposing factors Mad and Myc regulates cell fate during tissue remodeling. Cell Biosci..

[68] Sun G., Fu L., Shi Y.-B. (2014). Epigenetic regulation of thyroid hormone-induced adult intestinal stem cell development during anuran metamorphosis. Cell Biosci..

[69] Ishizuya-Oka A., Kajita M., Hasebe T. (2014). Thyroid Hormone-Regulated Wnt5a/Ror2 Signaling Is Essential for Dedifferentiation of Larval Epithelial Cells into Adult Stem Cells in the Xenopus laevis Intestine. PLoS ONE.

[70] Hasebe T., Fujimoto K., Kajita M., Fu L., Shi Y., Ishizuya-Oka A. (2017). Thyroid Hormone-Induced Activation of Notch Signaling is Required for Adult Intestinal Stem Cell Development DuringXenopus LaevisMetamorphosis. Stem Cells.

[71] Buchholz D.R., Shi Y.-B. (2018). Dual function model revised by thyroid hormone receptor alpha knockout frogs. Gen. Comp. Endocrinol..

[72] Wen L., Hasebe T., Miller T.C., Ishizuya-Oka A., Shi Y.-B. (2015). A requirement for hedgehog signaling in thyroid hormone-induced postembryonic intestinal remodeling. Cell Biosci..

[73] Muncan V., Heijmans J., Krasinski S.D., Büller N.V., Wildenberg M.E., Meisner S., Radonjic M., Stapleton K.A., Lamers W.H., Biemond I. (2011). Blimp1 regulates the transition of neonatal to adult intestinal epithelium. Nat. Commun..

[74] Harper J., Mould A., Andrews R.M., Bikoff E.K., Robertson E.J. (2011). The transcriptional repressor Blimp1/Prdm1 regulates postnatal reprogramming of intestinal enterocytes. Proc. Natl. Acad. Sci. USA.

[75] Matsuda H., Shi Y.-B. (2010). An Essential and Evolutionarily Conserved Role of Protein Arginine Methyltransferase 1 for Adult Intestinal Stem Cells During Postembryonic Development. Stem Cells.

[76] Plateroti M., Gauthier K., Domon-Dell C., Freund J.N., Samarut J., Chassande O. (2001). Functional interference between thyroid hormone receptor alpha (TRalpha) and natural truncated TRDeltaalpha isoforms in the control of intestine development. Mol. Cell Biol..

[77] Flamant F., Poguet A.L., Plateroti M., Chassande O., Gauthier K., Streichenberger N., Mansouri A., Samarut J. (2002). Congenital hypothyroid Pax8(-/-) mutant mice can be rescued by inactivating the TRalpha gene. Mol. Endocrinol..

[78] Kress E., Rezza A., Nadjar J., Samarut J., Plateroti M. (2009). The frizzled-related sFRP2 gene is a target of thyroid hormone receptor alpha1 and activates beta-catenin signaling in mouse intestine. J. Biol. Chem..

[79] Plateroti M., Chassande O., Fraichard A., Gauthier K., Freund J.N., Samarut J., Kedinger M. (1999). Involvement of T3Ralpha- and beta-receptor subtypes in mediation of T3 functions during postnatal murine intestinal development. Gastroenterology.

[80] Plateroti M., Kress E., Mori J.I., Samarut J. (2006). Thyroid hormone receptor alpha1 directly controls transcription of the beta-catenin gene in intestinal epithelial cells. Mol. Cell Biol..

