# Supplementary material for: Thyroid Hormone Receptor Is Essential for Larval Epithelial Apoptosis and Adult Epithelial Stem Cell Development but Not Adult Intestinal Morphogenesis during Xenopus tropicalis Metamorphosis
Source: Cells. 2021 Mar 3;10(3):536. doi: 10.3390/cells10030536 (PMC8000126; doi:10.3390/cells10030536)
Supplement: Supplementary file 1 [file cells-10-00536-s001.pdf]

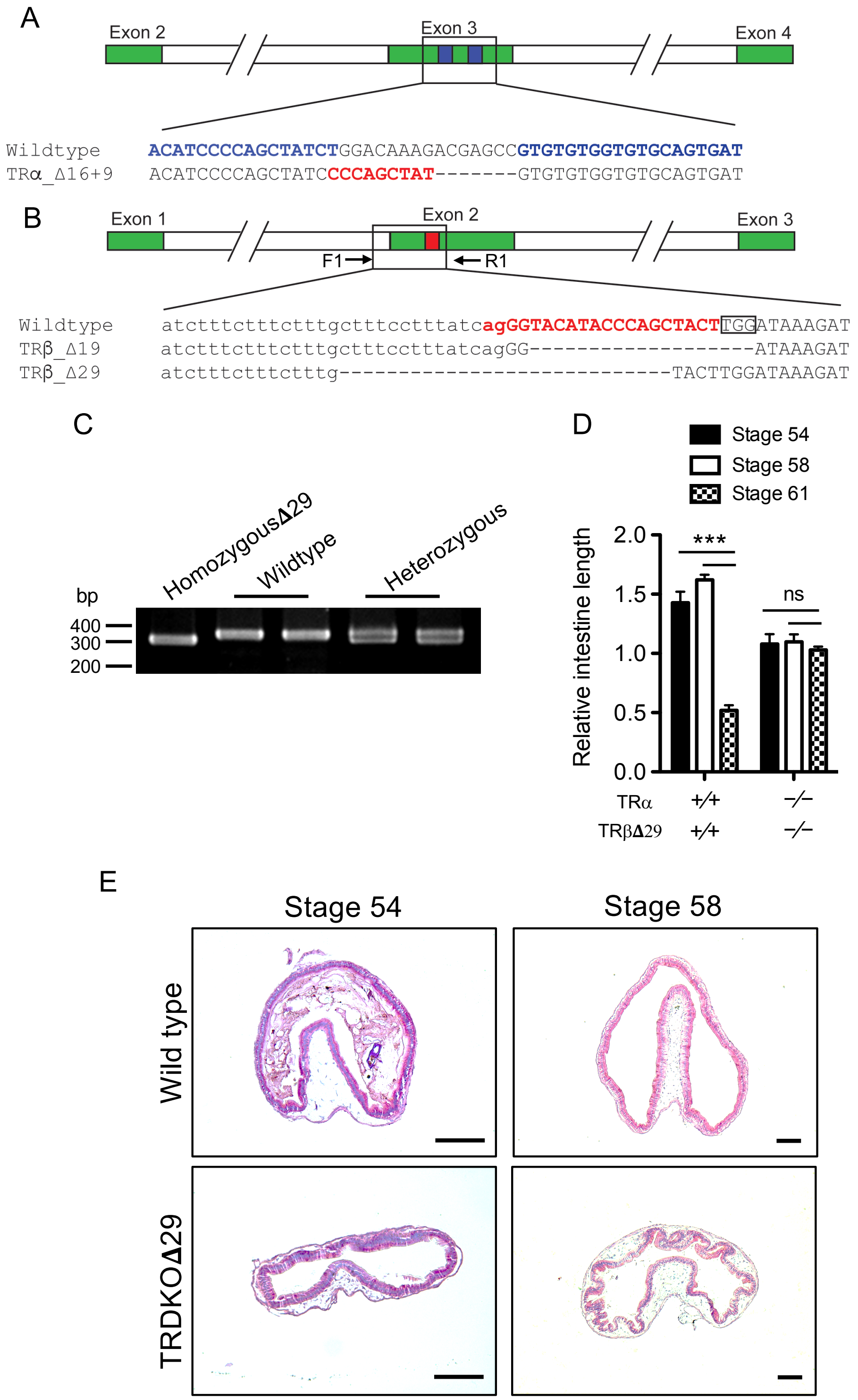

Supplemental Figure 1. TR double knockout tadpoles with a homozygous 29 base-out-frame deletion (14 base in exon and 15 base in intron) of TRβ gene locus shows similar intestinal remodeling defects as TR double knockout tadpoles with a homozygous 19 base-out-frame mutation of TRβ gene locus.

(A) Schematic diagram depicting the sequence of the TALEN-target region in wild type and TRα mutants (16 base-deletion and 9 base-insertion) animals. All TR double knockout animals have this TRα mutation. The protein coding exons are shown as numbered, colored boxes. The TALEN-recognized sequences are shown as blue letters whereas the insertion sequences described as red letters.

(B) Schematic diagram depicting the sequence of the CRISPR sgRNA-targeted region in wild type and TRβ mutants (19 base-deletion or 29 base-deletion) animals. The protein coding exons are shown as numbered, colored boxes. The CRISPR short guide RNA (sgRNA)-recognized sequence is shown as red box (top) or red letters (below). Exon and intron sequences are shown in capital or lower-case letters, respectively.

(C) Representative genotyping by PCR. Genotyping PCR was carried out on genomic DNA by using a common primer set, primer F and primer R. The presence of only upper (105 bp) or lower (86 bp) band in the gel indicates a wild type or homozygous mutant animal, respectively, while the presence of both upper and lower bands indicates a heterozygous mutant.

(D) TR double knockout with a 29 base out-of-frame mutation in TRβ locus (TRDKOΔ29) inhibits intestinal shortening at the climax of metamorphosis. The intestine length was measured and normalized against total body length at indicated stages. At least 4 tadpoles were analyzed for each sample. Asterisks (***) indicate a significant difference vs stage 58, for the 2 genotypes ($P < 0.001$).

(E) Abnormal intestinal morphology in TRDKOΔ29 tadpoles. Cross-sections of the intestine isolated from tadpoles were stained with methyl green-pyronin Y. (a and b): TRα $^{(+/+)}$TRβ $^{(+/+)}$; and (c and d): TRα $^{(-/-)}$TRβ $^{(-/-)\Delta 29}$. Note the precocious folding of the epithelium in the TRDKOΔ29 tadpoles by as early as stage 58, similar as TRDKOΔ19 tadpoles (see text). T: typhlosole. Bars: 100 μm.

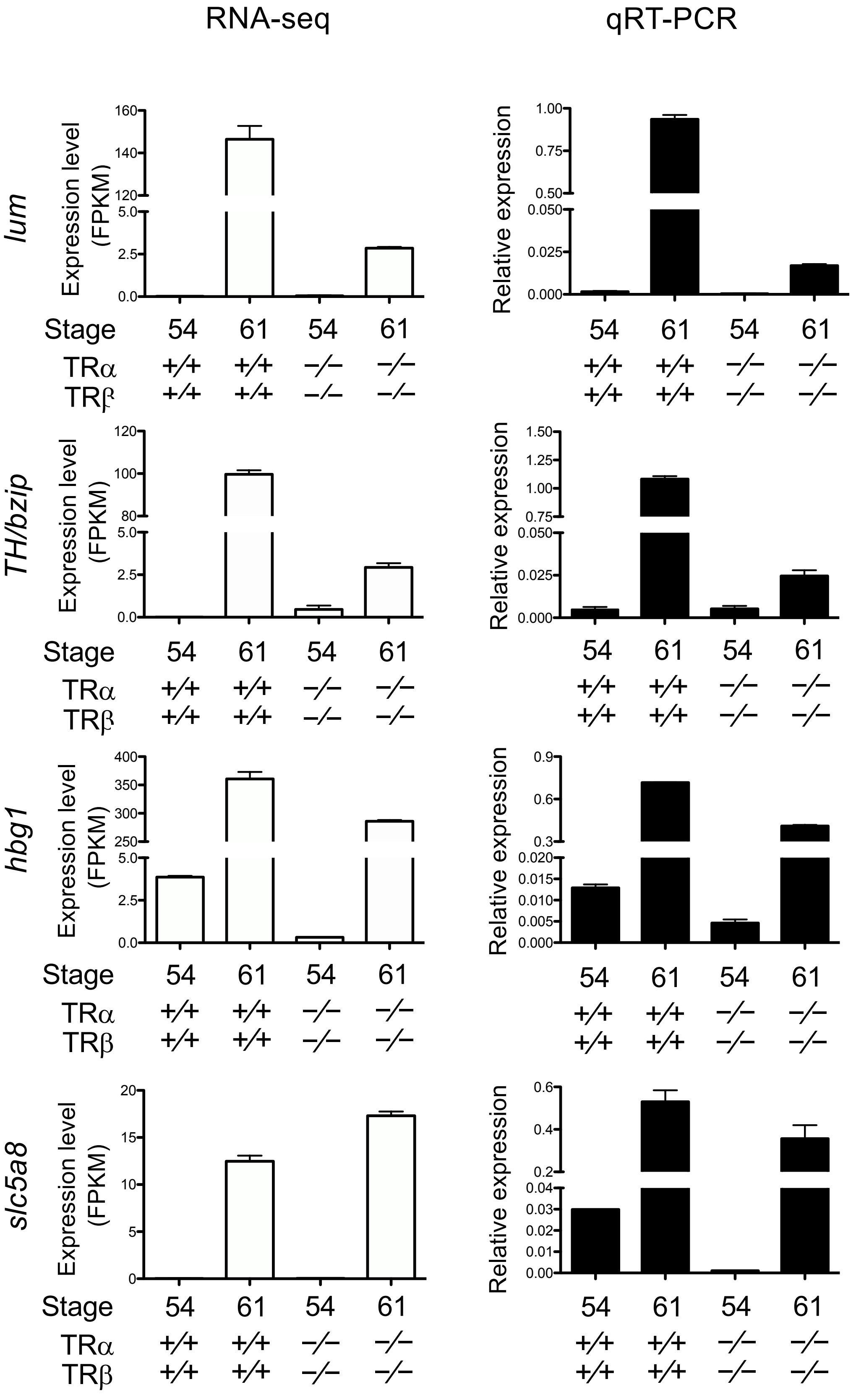

Supplemental Figure 2. Validation of RNA-seq data for the upregulated genes.

A number of up-regulated genes identified from the RNA-seq were selected for qRT-PCR analysis. The results from the RNA-seq (left) and qRT-PCR (right) were all consistent. Shown here are the expression patterns for 4 representative genes, *lum*, *TH/bzip*, *hbg1* and *slc5a8*. All expression data for RNA-seq and qRT-PCR were shown in FPKM and relatively expression normalized against that of *rpl8*, respectively.

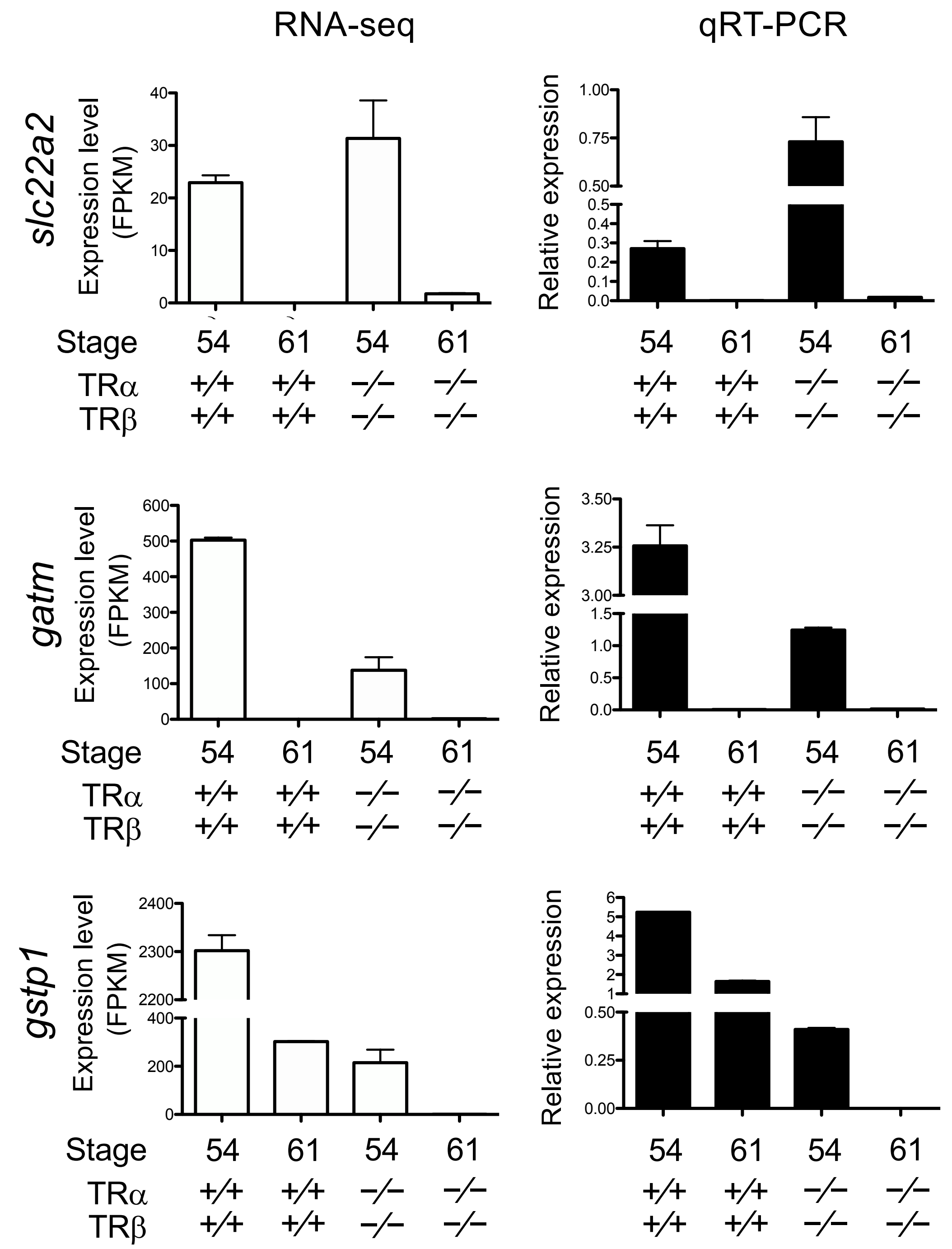

Supplemental Figure 3. Validation of RNA-seq data for the downregulated genes.

A number of down-regulated genes identified from the RNA-seq were selected for qRT-PCR analysis. The results from the RNA-seq (left) and qRT-PCR (right) were all consistent. Shown here are the expression patterns for 3 representative genes, *slc22a2*, *gatm* and *gstp1*. All expression data for RNA-seq and qRT-PCR were shown in FPKM and relatively expression normalized against that of *rpl8*, respectively.

Notch signaling pathway

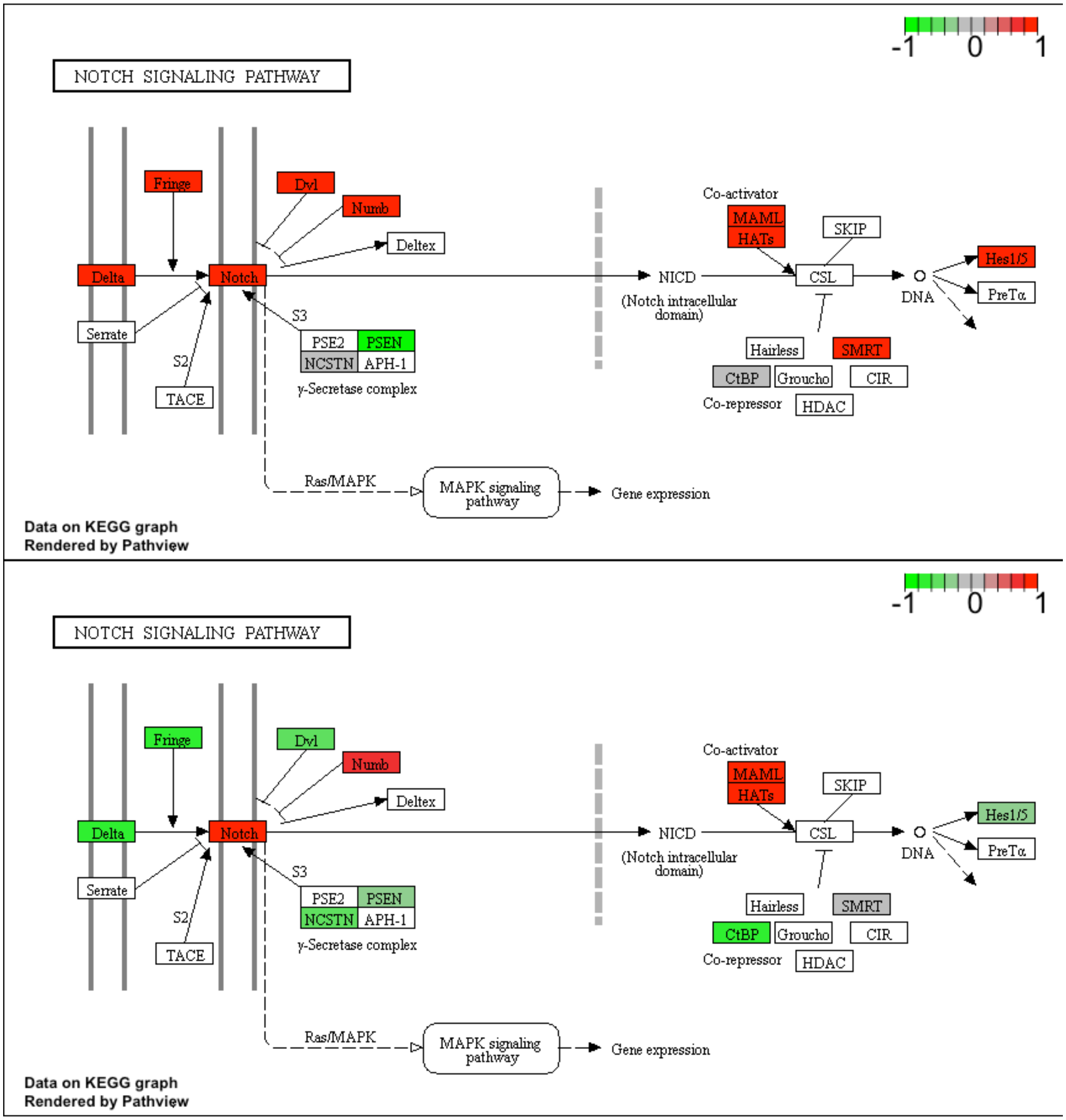

Supplemental Figure 4. A representative Notch signaling pathway likely involved in the development of adult intestinal stem cells as revealed by RNA-seq analysis.

Note that many more genes were upregulated (red) at stage 61 in the wild type tadpoles (Top) compared to the $TR\alpha^{(-/-)}TR\beta^{(-/-)}$ animals and the reverse was true for downregulated genes (green). The color bars for gene expression are in log2 scale.

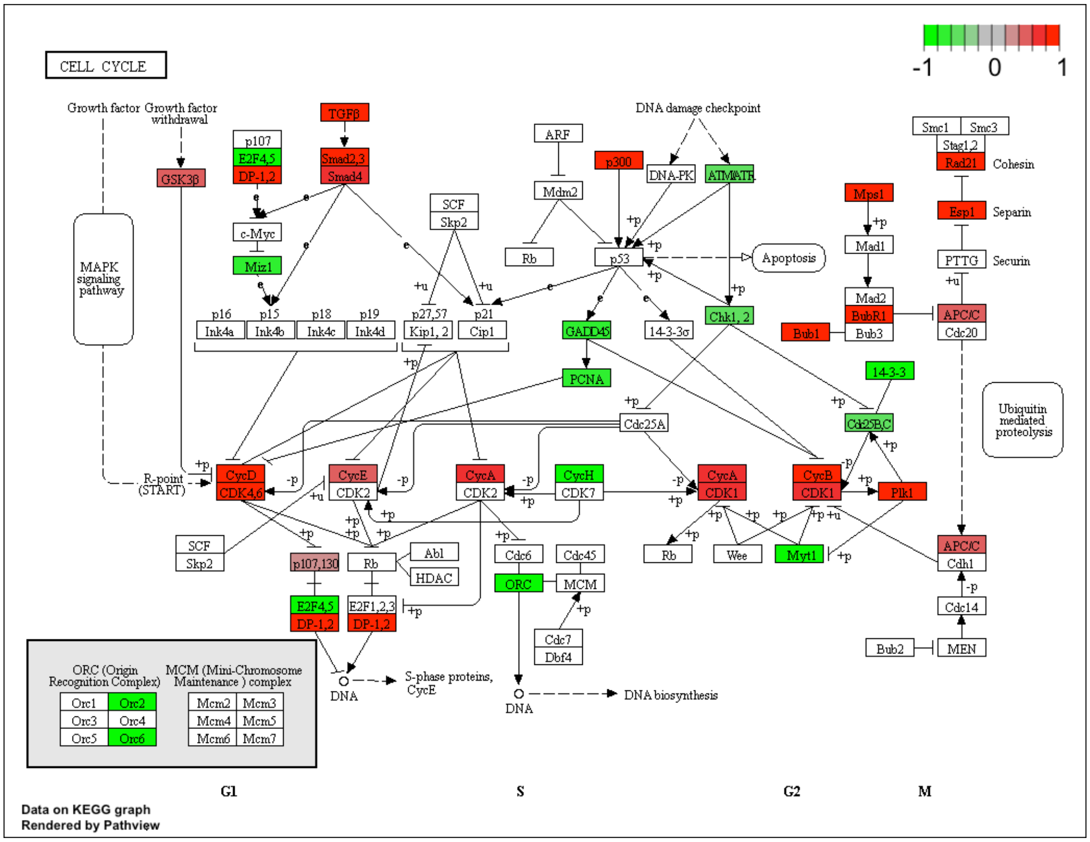

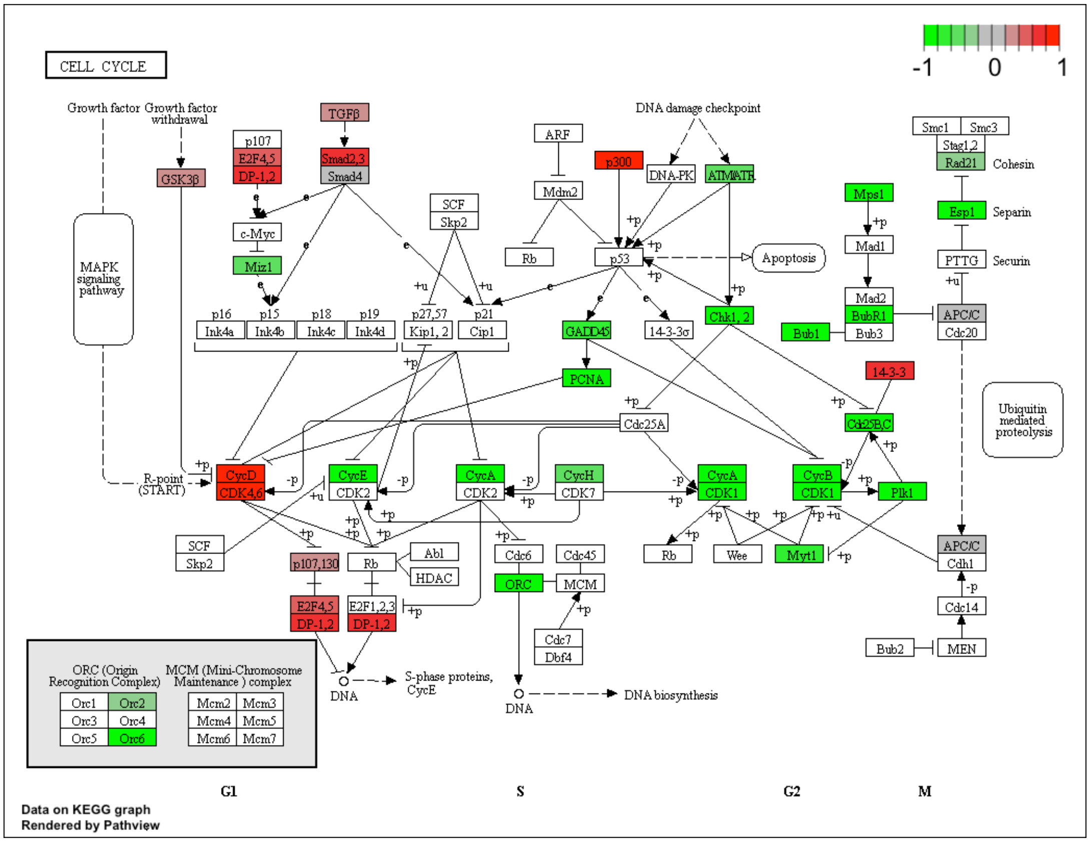

Supplemental Figure 5. A representative cell cycle signaling pathway likely involved in the proliferation of adult intestinal stem cells as revealed by RNA-seq analysis.

Note that many more mitosis related genes (right side) and G2-related genes (middle right) were upregulated (red) at stage 61 in the wild type tadpoles (Top) compared to the $TR\alpha^{(-/-)}TR\beta^{(-/-)}$ animals. Conversely, many more downregulated genes (green) were present among the S- (middle left) and G2 (middle right)-related genes in the $TR\alpha^{(-/-)}TR\beta^{(-/-)}$ animals. G1: Gap 1 phase, S: S phase, G2: Gap2 phase and M: Mitosis. The color bars for gene expression are in log2 scale.

Supplemental Table 1. Primers for genotyping to detect a 29 base-out-frame (14-base deletion in exon and 15-base deletion in the intron) mutation at the TRβ locus

Name	Primer sequence
CRISPR sgRNA	5′-TAATACGACTCACTATAGGGTACATACCCAGCT ACTGTTTTAGAGCTAGAAATAGCAAG -3′
CRISPR-3´primer	5′-AAAAGCACCGACTCGGTGCCACTTTTTCAAGTTGATAA CGGACTAGCCTTATTTTAACTTGCTATTTCTAGCTCTAAAAC-3′
Forward primer 1	5′- TCAATGGAACCCTTTGGAGCTG -3′
Reverse primer 1	5′- ACAGTTACAGGCATTTCCAGGC -3′

Supplemental Table 2. Primers used in qRT-PCR

Gene	Forward	Reverse
TRβ	5′- CTCTAGCAGCTGATGGTGGG -3′	5′- TGGGTTTTGTTCCTGGTGCT -3′
klf9	5′- GGCACAGGTGTCCTTATGCT -3′	5′- AAGGGCGTTCACCTGTATGG -3′
TH/bzip	5′- CCAAGGGAAACGGGTGGCTT -3′	5′- GTGCCACCTCTGCGGAAAGT -3′
rpl8	5′- AGAAGGTCATCTCATCTGCAAACAG -3′	5′- CAATACGACCACCACCAGCAA -3′
mmp2	5′- TAGAGCACTCTCAAGATCCTGGAG -3′	5′- AGGTCCAGGACGTGGTTTTTCCTT -3′
mmp9	5′- CTTTGTTCGATGGGTCACTCTGCT -3′	5′- ACGTTTCTGCATCCAGGTTG -3′
mmp11	5′- GGTTATGTGTGGCGCCTTCG -3′	5′- AATGGGAAAGGGCCCAGAGG -3′
mmp13	5′- TTCTAGCTCATGCCTTTGCTCCAG -3′	5′- TAGAGTGGTCTAATCCCAGCGAGT -3′
mmp14	5′- TGGAGATTTGCGGACACACACCTT -3′	5′- ATTTGTCAGGGACTCCACATCGAG -3′
casp3	5′- AGCCCAAACTATTCTTCATCCA -3′	5′- GATGAACCAGGAACCATTCATT -3′
casp 9	5′- GAGGAGTTTACGGAACAGATGG -3′	5′- AGTCACCGAACATCCTTTGTCT -3′
bax	5′- CTGCAGAGGAGAATTGAGTGTG -3′	5′- CATGGTCCAGTTAATGATGGTG -3′
xhh	5′- GCAGACCGACTCATGACTCA -3′	5′- CATCCTCATCCCAACCCTCC -3′
olfm4	5′- GATGAGCATGGAGTCTGTCAGTGT -3′	5′- AGCTTCTGCAGGCATCCTGTTAGT -3′
ror2	5′- CCCACTGGGACAAATGGATA -3′	5′- TCCTGACACTTTGCAGTGGA -3′
wnt5a	5′- CATCTCTCTCTGCTGCCTGA -3′	5′- GCTGAGCCCCAATGATATAAA -3′

Supplemental Table 3. Primers used in qRT-PCR validation of the *Xenopus* RNA-seq data.

			Primer sequence	
	GenBank	Gene	Forward	Reverse
The upregulated genes				
	XM_018092557	*TH/bzip*	5′- CCAAGGGAAACGGGTGGCTT -3′	5′- GTGCCACCTCTGCGGAAAGT -3′
	NM_001011006	*lum*	5′- TCCAGCTGTGAATGAAGGTCT -3′	5′- TGGAGTATTCCAGTGGTCCAA -3′
	NM_203528	*hbg1*	5′- GCAAAGACCTTGGGAAAACA -3′	5′- GGCTTCGATACTGCTGAGGT -3′
	NM_001364951	*slc5a8*	5′- CAGACTGGGCATCACTAGCA -3′	5′- GAAAAGTATGGTGCCGAGGA -3′
The downregulated genes				
	XM_002933862	*slc22a2*	5′- CACCGGATCATCCCTTGTAA -3′	5′- GATCAAGCCGCCAATAAAGA -3′
	NM_203640	*gatm*	5′- GGTGTGTGAGAGGAGGAAGC -3′	5′- CTCTGGAAACTTCGCTGCAC -3′
	NM_001016641	*gstp1*	5′- CGGGGAAAGATGACAGAAAG -3′	5′- TTACGCCCCAGATACCTCAG -3′
House-keeping gene				
	NM_203594	*rpl8*	5′- AGAAGGTCATCTCATCTGCAAACAG -3′	5′- CAATACGACCACCACCAGCAA -3′

Supplemental Table 4A. Ensembl RNA-seq data

Geneid	Length	St54-WT1_S1_Ensem	St54-WT2_S2_Ensem	St54-WT3_S3_Ensem	St54-TRDKO1	St54-TRDKO2	St54-TRDKO3	St61-WT1_S	St61-WT2_S	St61-WT3_S	St61-TRDKO1	St61-TRDKO2	St61-TRDKO3	Gene Symbol
ENSXETG00000000002	764	449	686	622	304	239	414	638	671	481	206	148	166	eaf2
ENSXETG00000000003	1200	14	16	17	47	31	63	32	38	26	34	17	29	vipr1
ENSXETG00000000004	2142	0	0	0	0	0	0	1	0	0	0	0	0	
ENSXETG00000000007	1068	61	98	97	169	131	217	266	287	145	229	161	198	znf37a
ENSXETG00000000009	2242	0	0	0	0	0	0	4	7	1	4	0	3	art1
ENSXETG00000000011	561	5	6	5	26	17	64	104	126	81	54	35	44	ENSXETG00000000011
ENSXETG00000000013	3508	805	1099	1020	1635	1337	2773	3050	3413	2171	4289	3059	3249	nfkb2
ENSXETG00000000014	1034	1	0	0	1	3	1	9	6	7	8	6	3	psd
ENSXETG00000000016	867	7	5	8	2	0	5	72	124	86	13	3	5	ENSXETG00000000016
ENSXETG00000000020	1533	224	276	223	310	235	176	537	704	390	529	342	390	mboat1
ENSXETG00000000021	2123	186	280	256	286	245	193	533	639	400	226	142	171	e2f3
ENSXETG00000000022	1020	98	152	122	287	227	532	715	775	456	671	446	473	cdkal1
ENSXETG00000000026	1570	0	0	1	0	0	0	1	2	0	0	1	2	ddx25
ENSXETG00000000029	3783	90	112	109	684	518	987	1605	1891	1155	773	524	640	cdon
ENSXETG00000000035	2469	640	912	761	849	712	1932	1182	1266	754	2102	1588	1722	mdm4
ENSXETG00000000036	1023	2004	2713	2599	3434	2716	3843	766	864	477	2507	1716	1872	ech1
ENSXETG00000000037	1650	2323	3511	2957	5490	4562	5285	7240	8414	5026	4654	3296	3619	hnrnpl
ENSXETG00000000038	531	1195	1713	1528	991	739	1385	1641	1896	1195	1084	714	805	arf6.2
ENSXETG00000000039	975	3	8	3	2	5	3	19	31	20	6	1	2	lgals4
ENSXETG00000000040	3607	6466	9171	8010	14146	10621	13136	14786	16752	9882	13603	9543	10621	actn4
ENSXETG00000000041	3074	3	3	0	0	0	2	3	3	2	2	2	8	ENSXETG00000000041
ENSXETG00000000042	1143	158	207	180	221	178	341	124	175	89	234	162	166	dnajc28
ENSXETG00000000043	1286	599	935	695	505	400	931	869	1057	640	499	386	401	tmem50b
ENSXETG00000000044	939	245	401	328	357	247	671	671	783	466	608	407	455	ifngr2.1
ENSXETG00000000046	1497	0	0	0	0	0	0	4	6	5	2	0	2	ifnar2.2
ENSXETG00000000048	2451	0	0	1	1	1	2	9	15	8	7	10	3	pla2g4d
ENSXETG00000000049	1227	124	211	160	155	116	317	190	194	115	314	242	243	jmjd7
ENSXETG00000000050	4404	22	30	24	62	36	72	381	415	253	73	74	89	mapkbp1
ENSXETG00000000051	8580	77	121	110	361	248	410	542	643	357	578	438	454	mga
ENSXETG00000000052	4817	1007	1496	1376	2131	1625	5188	1395	1679	968	2608	1792	1999	tyro3
ENSXETG00000000053	7294	0	0	0	0	1	2	2	9	0	8	1	1	tmem151b
ENSXETG00000000054	4170	151	211	175	293	216	359	431	444	251	444	312	308	rpap1
ENSXETG00000000055	3480	3	8	8	63	35	103	92	109	67	101	87	62	ENSXETG00000000055
ENSXETG00000000056	1420	748	1147	910	1083	872	1699	2152	2560	1446	1358	928	1058	snap23
ENSXETG00000000057	1814	474	735	583	673	519	834	1144	1280	763	743	503	575	acot2
ENSXETG00000000060	2694	256	371	297	378	287	429	599	629	384	441	306	359	lrrc57
ENSXETG00000000061	1535	5	16	12	175	148	25	46	69	38	20	20	18	dnal1
ENSXETG00000000062	2985	750	1110	924	1977	1479	2378	2363	2732	1572	2837	1931	2145	elmsan1
ENSXETG00000000063	1419	0	0	0	10	2	0	0	0	0	56	36	53	
ENSXETG00000000066	1599	328	458	390	347	226	868	524	637	364	453	352	362	c1orf159
ENSXETG00000000067	2816	554	707	643	1149	944	1897	899	1014	639	1943	1311	1487	ripk4
ENSXETG00000000068	3456	103	135	122	280	243	290	451	515	298	385	268	274	prdm15
ENSXETG00000000070	7183	466	694	583	1596	1276	2050	2464	2862	1713	1809	1295	1387	rad54l2
ENSXETG00000000071	4377	222	309	253	387	301	405	526	569	358	656	460	513	dcaf1
ENSXETG00000000072	1569	166	234	207	388	301	559	696	784	462	626	455	519	rbm15b
ENSXETG00000000073	987	4474	6454	5417	1125	803	1076	2044	2310	1386	456	327	360	manf
ENSXETG00000000074	7567	3	2	2	8	5	44	253	298	178	322	239	238	dock3
ENSXETG00000000076	2192	3136	4595	3847	5174	3990	8886	6781	7657	4482	10285	7309	7955	mapkapk3
ENSXETG00000000078	1445	180	237	183	321	230	1958	71	72	47	671	464	567	cish
ENSXETG00000000079	891	68	77	78	28	24	42	57	72	35	20	15	15	hemk1
ENSXETG00000000081	2919	6	8	10	27	13	24	54	50	32	61	51	47	cacna2d2
ENSXETG00000000082	1834	820	1168	992	1717	1234	2490	1868	2080	1188	2667	1757	2026	tmem115
ENSXETG00000000083	1566	442	717	589	530	396	661	529	633	328	535	382	387	nprl2
ENSXETG00000000087	1662	1734	2578	2217	2868	2183	2885	1178	1357	803	1368	1016	1102	parp3
ENSXETG00000000088	686	4756	7141	5959	4382	3261	5485	1629	1816	1058	1987	1443	1556	abhd14b
ENSXETG00000000089	1433	987	1490	1293	1422	1050	1381	1411	1639	1003	1577	1090	1245	acy1.1
ENSXETG00000000091	2541	605	869	755	1196	965	1217	2060	2349	1418	1356	930	998	bap1
ENSXETG00000000093	1005	0	0	0	0	0	0	0	0	0	0	0	0	
ENSXETG00000000101	507	645	1002	856	1357	1029	2739	743	768	501	1409	947	1037	tmem37
ENSXETG00000000104	2306	2011	2818	2314	1795	1389	1740	1468	1837	945	2574	1699	2013	vnn1
ENSXETG00000000108	1900	0	0	0	2	2	15	203	258	144	30	10	17	slc18b1
ENSXETG00000000109	396	87564	131389	114416	88482	66681	93170	83858	98018	59413	158425	110366	121529	rps12
ENSXETG00000000110	1869	0	0	0	0	2	5	5	7	2	10	6	5	eya4
ENSXETG00000000111	729	0	0	1	0	0	0	0	0	0	0	0	0	
ENSXETG00000000114	1020	46	61	56	214	167	213	2202	2501	1443	541	405	467	tcf21
ENSXETG00000000116	1217	169	216	156	201	178	366	377	474	277	309	222	198	tbpl1
ENSXETG00000000117	2559	188	249	232	635	486	605	1426	1529	965	1253	911	1018	extl3
ENSXETG00000000118	1581	47	91	62	153	101	262	39	45	18	222	155	166	slc2a12
ENSXETG00000000121	2112	50	84	80	275	182	649	494	547	347	498	405	451	kif13b
ENSXETG00000000124	1475	6044	8932	7563	7133	5329	6745	1479	1707	961	1585	1104	1265	sgk1
ENSXETG00000000128	12471	0	0	0	1	0	0	6	11	10	4	5	6	ENSXETG00000000128
ENSXETG00000000129	408	194	260	212	198	143	350	415	443	292	287	216	246	atg12
ENSXETG00000000130	1120	2	1	2	0	0	0	105	150	94	1	5	3	gsg1l
ENSXETG00000000132	466	19290	28727	24482	29269	21371	34883	32632	37587	22596	79254	55116	60810	rpl28
ENSXETG00000000135	5526	71	98	67	305	260	447	1188	1319	792	697	548	577	crip2
ENSXETG00000000137	932	0	0	0	0	1	1	0	0	0	0	0	0	
ENSXETG00000000138	694	41	53	56	81	48	144	169	186	115	99	46	43	ube2s
ENSXETG00000000139	1134	0	0	0	0	0	0	0	0	0	0	0	0	
ENSXETG00000000140	874	0	2	0	1	0	1	2	4	2	0	2	1	ENSXETG00000000140
ENSXETG00000000142	1341	0	0	0	0	0	0	1	1	1	0	0	0	
ENSXETG00000000143	1093	225	297	218	259	192	319	231	257	178	418	334	301	isoc2
ENSXETG00000000145	1934	126	175	145	180	176	439	351	444	277	379	276	262	gin1
ENSXETG00000000147	2157	9	7	10	43	32	7	5	6	5	4	2	1	XB-GENE-5822220
ENSXETG00000000148	564	0	0	0	0	0	0	1	4	2	0	0	0	
ENSXETG00000000150	3897	283	406	360	521	452	1776	1054	1186	698	900	587	738	ENSXETG00000000150
ENSXETG00000000151	930	678	998	799	753	573	966	1437	1703	997	745	489	564	yif1b
ENSXETG00000000152	2817	401	590	471	530	410	1010	2585	3181	1717	696	471	519	kcnk6
ENSXETG00000000154	1548	47	55	44	133	101	108	258	270	184	118	99	102	mitf
ENSXETG00000000155	2556	4144	5983	5119	7811	6073	11432	9492	11069	6748	9601	6992	7654	spint2
ENSXETG00000000156	2640	7	14	13	77	38	135	261	315	187	107	90	92	rasip1
ENSXETG00000000157	2287	155	203	202	567	514	1314	3165	3642	2151	825	640	699	ppp1r14a
ENSXETG00000000158	4127	4	13	12	36	33	31	221	242	169	60	31	43	ENSXETG00000000158
ENSXETG00000000161	1093	287	434	374	594	373	416	2251	2411	1457	386	258	255	sae1
ENSXETG00000000170	2636	74	108	87	103	71	68	249	286	174	40	20	24	cenpn
ENSXETG00000000172	2819	769	1188	971	958	711	2854	772	867	586	1680	1238	1271	maf
ENSXETG00000000183	2337	80	86	65	104	95	145	224	247	153	313	229	251	atmin
ENSXETG00000000185	1570	99	147	122	143	109	99	214	229	127	138	105	106	cdk10
ENSXETG00000000187	998	8637	12492	10694	6691	5241	9484	3055	3577	2048	3457	2501	2728	gcsh
ENSXETG00000000190	735	4	4	3	10	7	11	72	82	43	33	23	18	tusc3
ENSXETG00000000198	3435	117	185	154	207	138	346	296	324	203	404	292	302	trerf1
ENSXETG00000000199	1080	25	28	16	14	12	25	141	184	94	79	42	42	cxcr3
ENSXETG00000000201	5428	232	328	247	664	486	1023	898	951	536	832	641	681	ubr2
ENSXETG00000000204	3040	1	2	1	1	1	4	44	59	30	26	19	23	syt3
ENSXETG00000000210	6151	1	0	0	0	0	1	5	1	3	3	0	2	
ENSXETG00000000213	1050	0	1	1	1	0	0	9	5	5	4	1	2	prph2
ENSXETG00000000215	1017	178	251	255	251	214	287	532	552	356	197	134	135	tbcc
ENSXETG00000000216	2400	538	778	698	596	458	525	927	968	574	689	506	505	diexf
ENSXETG00000000217	1677	0	0	0	0	2	0	6	3	4	4	0	0	syt14
ENSXETG00000000221	849	0	1	1	0	8	20	158	173	96	24	30	41	sertad4

ENSXETG00000000222	1050	17	27	15	23	20	39	66	62	36	32	28	40	hhat
ENSXETG00000000225	2880	0	1	1	0	2	0	0	0	0	4	3	2	kcnh1
ENSXETG00000000228	1794	45	56	52	154	135	79	476	605	336	165	101	106	SLC6A7
ENSXETG00000000229	1858	909	1352	1146	1188	989	983	1570	1719	1064	1272	903	1008	luc7l2
ENSXETG00000000231	1769	69	108	108	341	281	455	1001	1085	710	610	409	454	rbfox2
ENSXETG00000000232	2063	579	824	653	412	327	930	402	454	268	422	302	348	slc25a38
ENSXETG00000000233	5871	5716	8466	7373	13584	10312	9958	21408	24750	14917	11437	7920	8899	myh9
ENSXETG00000000235	1104	0	0	0	0	0	0	0	0	0	0	0	0	
ENSXETG00000000237	3573	14	29	24	52	33	133	1086	1239	735	267	172	213	zeb2
ENSXETG00000000244	2274	0	2	1	6	6	21	61	72	58	41	20	32	brinp1
ENSXETG00000000246	1476	1513	2121	1837	2191	1847	2927	1490	1847	1087	2155	1471	1722	txn2
ENSXETG00000000247	2221	168	270	231	266	189	355	751	813	466	367	263	306	foxred2
ENSXETG00000000251	858	30	41	49	142	100	413	1622	1943	1149	183	105	134	hmox1
ENSXETG00000000253	3041	702	944	841	1530	1055	304	1552	1860	1053	382	263	320	mcm5
ENSXETG00000000254	1626	70	107	88	136	105	408	188	217	133	304	214	228	gtdc1
ENSXETG00000000256	489	2588	3821	3080	2620	1778	1485	1777	1905	1091	1300	942	1015	ndufa6
ENSXETG00000000260	756	2	1	2	5	4	24	71	69	43	30	23	16	fam109b
ENSXETG00000000261	1053	0	0	0	0	1	3	25	25	6	3	4	2	sept3
ENSXETG00000000264	585	159	256	205	247	179	304	469	527	350	240	198	195	lin7c
ENSXETG00000000265	3702	1	1	3	3	6	7	125	156	83	15	12	12	ppfia3
ENSXETG00000000267	2019	1	2	1	8	2	14	16	13	12	3	2	5	ccndx
ENSXETG00000000274	3582	35	39	39	1307	968	636	692	770	486	1150	839	940	trpm5
ENSXETG00000000279	1968	245	374	341	517	380	386	669	744	488	469	327	354	pak6
ENSXETG00000000286	1635	1733	2588	2177	2556	1937	4764	5999	6576	4107	4858	3306	3574	cdknx
ENSXETG00000000287	3081	66	109	99	212	159	158	420	518	330	69	37	50	bub1b
ENSXETG00000000289	546	80	111	120	470	302	636	1506	1637	1061	383	245	268	bmf
ENSXETG00000000290	671	1815	2610	2355	1272	888	1284	1647	1873	1095	721	482	513	srp14
ENSXETG00000000291	603	2	2	0	41	15	3	11	18	18	36	27	26	xgb
ENSXETG00000000292	3882	715	1067	890	1934	1432	2414	2656	3080	1718	2964	2095	2349	plekhg3
ENSXETG00000000293	1536	208	354	283	353	279	494	1272	1371	883	1331	908	1008	mob2.2
ENSXETG00000000295	2015	37	42	43	52	30	68	162	161	111	108	96	116	XB-GENE-5872932
ENSXETG00000000296	3004	744	1092	937	1574	1208	1891	2821	3283	1885	1812	1270	1441	med25
ENSXETG00000000298	1583	342	531	436	399	284	371	529	551	330	358	239	245	gemin2
ENSXETG00000000299	2442	67	106	107	154	127	69	218	242	150	120	83	96	pla2g4f
ENSXETG00000000300	1233	2	7	11	87	71	231	78	85	52	150	104	112	acpp
ENSXETG00000000301	5175	364	525	479	694	515	1261	2464	2887	1611	870	631	709	vps39
ENSXETG00000000302	3040	380	550	467	2448	1898	4064	301	356	229	1062	754	870	ganc
ENSXETG00000000304	834	1262	1945	1656	216	169	609	976	1177	689	1080	739	819	egln3
ENSXETG00000000305	2914	1	0	1	14	8	26	136	132	81	45	23	25	npas3
ENSXETG00000000307	3472	2101	3077	2579	2879	2139	4611	3266	3627	2183	4114	2822	3230	nr1h2
ENSXETG00000000309	1083	0	0	0	0	0	0	0	0	0	0	0	0	ENSXETG00000000309
ENSXETG00000000310	2839	26	51	23	69	86	80	327	418	241	100	66	85	wdr47
ENSXETG00000000311	1773	104	155	125	151	117	205	204	247	120	94	78	85	ddx59
ENSXETG00000000312	3507	40	51	37	163	130	136	298	374	204	34	24	28	XB-GENE-981231
ENSXETG00000000314	2678	349	538	428	512	374	1281	280	374	239	792	528	628	nr5a2
ENSXETG00000000316	1785	69	85	72	144	83	240	927	1085	610	347	271	309	ptprc
ENSXETG00000000318	390	207	266	259	87	81	137	119	137	86	132	103	106	ENSXETG00000000318
ENSXETG00000000320	1181	966	1476	1191	1307	936	1145	1208	1325	819	897	653	679	adi1
ENSXETG00000000321	1455	56789	79895	66404	29788	22810	35917	22446	27320	14970	14993	10633	11944	calm1
ENSXETG00000000324	1321	1514	2249	1872	1657	1281	2254	2185	2496	1484	1551	1099	1206	ergic2
ENSXETG00000000325	4386	2	2	1	1	0	0	1	1	2	3	0	1	ovch1
ENSXETG00000000326	2736	16	18	15	141	105	199	279	337	187	395	288	332	TMTC1
ENSXETG00000000327	1233	7	14	10	0	2	5	15	26	10	19	20	17	ENSXETG00000000327
ENSXETG00000000331	1305	0	0	0	0	0	0	1	3	1	0	0	1	
ENSXETG00000000332	806	453	629	584	594	437	1053	502	555	339	796	546	576	rchy1
ENSXETG00000000333	2793	2105	2920	2561	3094	2382	4424	2259	2638	1540	3536	2367	2744	uso1
ENSXETG00000000334	1685	260	391	323	357	268	435	751	821	576	340	260	287	g3bp2
ENSXETG00000000335	1689	4	7	5	31	15	82	28	30	16	57	36	36	cdkl2
ENSXETG00000000337	459	44	66	53	73	55	77	1447	1732	1061	1168	820	977	dmp1
ENSXETG00000000338	2855	12	24	12	128	107	277	1022	1223	698	303	226	233	pkd2
ENSXETG00000000340	3210	7384	10700	8866	27742	21071	11759	1907	2336	1320	2942	2138	2270	abcg2
ENSXETG00000000341	1125	16	28	22	70	42	57	94	127	80	164	90	112	ppm1k
ENSXETG00000000342	3093	78	116	90	149	115	442	99	117	72	198	151	161	herc6
ENSXETG00000000343	3351	127	175	143	612	481	602	1374	1571	932	142	77	96	anln
ENSXETG00000000344	1407	40	67	53	121	96	199	254	247	174	165	112	129	zdhhc1
ENSXETG00000000345	2211	41	50	34	1098	822	218	241	289	171	391	277	323	tppp3
ENSXETG00000000346	3357	375	511	423	901	739	1844	1457	1802	1026	1795	1309	1516	rbl2
ENSXETG00000000349	9399	250	381	376	420	389	833	1077	1259	721	1159	847	944	chd9
ENSXETG00000000351	555	0	3	5	10	3	2	23	41	13	21	15	23	epdr1
ENSXETG00000000352	2587	216	259	223	219	148	708	584	700	391	411	279	272	stard3nl
ENSXETG00000000355	387	292	395	294	210	141	237	499	539	325	363	262	300	cck
ENSXETG00000000359	893	6988	9567	7811	7172	5383	12661	9727	11522	7189	14905	9999	11939	eif1b
ENSXETG00000000362	1281	46	79	64	32	23	109	410	510	322	81	62	68	gzma
ENSXETG00000000363	7064	71	98	63	145	114	266	291	383	199	112	80	88	cspg4
ENSXETG00000000364	970	4	2	1	8	7	3	102	112	71	8	2	2	esm1
ENSXETG00000000365	717	80	107	111	84	55	98	1070	1225	676	156	107	114	gzmak
ENSXETG00000000367	4116	656	965	839	823	564	870	1109	1288	739	683	530	603	dhx29
ENSXETG00000000368	4263	1532	2159	1837	2027	1556	1378	2401	2804	1629	1350	906	995	sec31a
ENSXETG00000000369	3258	314	519	415	904	681	949	1259	1415	830	855	561	630	lin54
ENSXETG00000000371	3181	68	99	97	183	134	262	279	328	184	279	236	238	mfhas1
ENSXETG00000000372	1989	115	121	107	245	184	409	539	616	345	413	296	354	cnot6l
ENSXETG00000000373	1843	988	1425	1218	1026	805	2229	946	1157	648	1831	1270	1609	mrpl1
ENSXETG00000000375	1811	783	1176	955	3623	2810	6411	5202	6066	3432	7551	5395	5869	plpp1
ENSXETG00000000377	11934	33	52	37	378	294	279	295	350	193	231	169	211	fras1
ENSXETG00000000380	1564	31466	47601	40636	60790	47126	184612	32337	35502	23207	73600	52021	56485	glul
ENSXETG00000000383	1759	244	385	317	511	388	469	1067	1147	693	504	347	404	nelfb
ENSXETG00000000384	2259	0	2	0	0	0	0	9	15	5	0	0	0	
ENSXETG00000000390	795	1	1	3	0	0	0	21	23	18	2	0	1	
ENSXETG00000000391	1940	0	0	0	0	2	3	1	0	1	3	1	0	
ENSXETG00000000392	1019	1	1	4	2	0	12	0	1	4	0	0	0	ENSXETG00000000392
ENSXETG00000000393	339	0	0	0	0	0	0	0	0	0	0	0	0	
ENSXETG00000000395	1274	0	2	0	1	0	5	0	1	0	1	0	0	
ENSXETG00000000396	2263	8	12	10	64	46	238	689	700	471	84	62	70	MGC108470
ENSXETG00000000399	6045	697	884	760	1211	942	2810	889	1053	629	1864	1272	1484	selenoo
ENSXETG00000000401	3192	5	6	12	89	62	278	2160	2382	1559	417	265	329	pdzrn3
ENSXETG00000000402	2208	220	335	301	770	598	1232	670	704	493	1112	777	875	man1b1
ENSXETG00000000407	1332	0	6	0	2	0	0	4	5	3	2	2	2	ENSXETG00000000407
ENSXETG00000000408	891	42	55	50	34	41	103	226	253	153	83	44	64	ENSXETG00000000408
ENSXETG00000000409	1728	274	395	323	442	320	534	260	291	165	254	187	239	ENSXETG00000000409
ENSXETG00000000413	3363	3	4	9	10	10	8	12	18	12	10	9	6	erbb4
ENSXETG00000000421	1996	212	328	281	184	147	406	572	621	360	308	209	245	smpd2
ENSXETG00000000422	1794	0	0	0	1	0	1	0	0	0	0	0	0	tulp1
ENSXETG00000000426	2413	614	936	836	823	639	749	1341	1504	921	710	472	534	srpk1
ENSXETG00000000436	6062	247	330	281	438	335	733	1182	1488	802	668	430	508	mapk12
ENSXETG00000000438	1089	706	1002	902	795	550	1174	1307	1585	975	695	485	495	mapk14
ENSXETG00000000439	3000	463	674	512	1170	909	1266	1402	1599	877	1988	1411	1544	zc3h11a
ENSXETG00000000441	3771	473	698	590	1614	1307	2851	2539	2782	1659	2610	1812	2075	atp2b2
ENSXETG00000000442	1054	8	14	6	19	18	31	5	3	1	6	7	6	optc
ENSXETG00000000445	1925	578	803	662	600	488	987	478	576	345	343	221	281	adcy7
ENSXETG00000000446	1695	85	128	107	181	129	312	362	436	268	312	186	220	papd5
ENSXETG00000000448	4774	488	693	622	2283	1825	2821	3410	3936	2257	3925	2835	3251	erbin

ENSXETG00000000451	2378	180	246	204	410	337	543	325	369	212	547	415	443	nln
ENSXETG00000000452	921	1	2	0	1	2	0	4	7	5	3	3	3	sgtb
ENSXETG00000000453	1717	471	725	594	681	497	951	876	997	564	972	641	768	trappc13
ENSXETG00000000454	1851	346	433	382	632	478	662	938	1086	656	738	509	597	ppwd1
ENSXETG00000000455	1482	69	81	85	182	132	237	294	340	182	219	158	198	trim23
ENSXETG00000000456	948	48	84	80	129	98	89	237	287	167	100	71	67	cenpk
ENSXETG00000000457	3351	1	1	2	3	3	4	197	238	145	22	18	16	adamts6
ENSXETG00000000459	1682	158	228	189	297	234	518	635	679	442	486	322	357	cwc27
ENSXETG00000000461	2585	203	252	226	291	198	448	553	643	329	357	270	314	srek1ip1
ENSXETG00000000462	486	0	0	0	0	0	0	3	5	4	0	2	2	fam159b
ENSXETG00000000463	762	0	1	0	3	2	2	23	23	14	11	6	13	rgs7bp
ENSXETG00000000465	3074	321	508	416	602	486	678	1238	1318	867	698	502	544	smarcal1
ENSXETG00000000466	1170	0	2	0	2	0	3	11	14	6	3	6	3	march4
ENSXETG00000000469	1897	15	22	18	19	16	50	303	379	236	43	39	48	tmem169
ENSXETG00000000470	1683	1984	2799	2521	2691	2094	3417	779	988	564	1409	1008	1125	pecr
ENSXETG00000000471	1881	947	1399	1199	1067	864	2056	728	868	511	940	717	733	map3k2
ENSXETG00000000472	2462	1005	1517	1340	1343	1011	1763	1527	1788	1094	903	687	751	slc30a5
ENSXETG00000000473	2050	44	50	40	169	124	182	1634	1697	1072	341	286	281	bin1
ENSXETG00000000477	1290	0	0	0	0	0	0	0	6	1	1	0	0	ccnb1
ENSXETG00000000479	2010	42	39	37	80	73	89	95	97	72	166	131	117	zranb3
ENSXETG00000000480	2550	173	282	255	739	582	1534	941	1173	635	1322	952	1007	rab3gap1
ENSXETG00000000481	1257	282	376	308	190	174	151	272	340	190	169	144	125	cdk7
ENSXETG00000000483	954	0	0	0	0	0	0	0	0	0	0	0	0	
ENSXETG00000000484	1398	53	52	39	134	97	125	547	629	353	218	166	179	serinc5
ENSXETG00000000487	1779	405	581	456	873	668	1266	2022	2296	1378	1143	779	928	sh3glb1
ENSXETG00000000488	2172	153	232	209	277	172	375	395	463	301	513	366	404	ccnt2
ENSXETG00000000496	2855	548	770	667	1107	862	2913	1597	2068	1140	1486	1018	1121	ENSXETG00000000496
ENSXETG00000000500	3064	14	20	21	78	59	90	88	115	60	471	339	396	thbs4
ENSXETG00000000501	2388	0	0	0	0	0	0	0	0	0	0	0	0	
ENSXETG00000000502	1062	15	20	21	21	20	25	116	122	78	19	28	25	pbx3
ENSXETG00000000504	1011	486	658	642	736	533	1396	594	664	406	448	317	312	acmsd
ENSXETG00000000505	1602	290	434	356	657	487	1018	1002	1181	705	1114	849	972	mapkap1
ENSXETG00000000506	2769	14592	21163	17810	1581	1257	2279	1429	1644	949	128	65	79	clca1
ENSXETG00000000509	3660	4	2	1	19	22	12	17	27	12	29	10	25	tmem163
ENSXETG00000000510	915	64	91	87	73	62	118	167	221	104	159	122	114	mtx3
ENSXETG00000000511	1590	0	0	1	0	0	0	0	0	0	0	0	0	tubal3
ENSXETG00000000514	2232	434	571	484	941	747	2237	1812	2030	1284	2929	2113	2410	mgat5
ENSXETG00000000516	3591	120	182	158	270	221	411	540	664	410	371	263	288	klhl20
ENSXETG00000000517	4893	318	471	409	701	629	920	1152	1560	788	1131	785	826	ENSXETG00000000517
ENSXETG00000000518	2562	513	677	614	740	584	952	1201	1351	741	1004	741	807	papd4
ENSXETG00000000519	1659	0	1	0	5	0	4	12	16	13	11	5	5	homer1
ENSXETG00000000520	2641	37	45	34	189	147	517	649	736	503	366	278	279	jmy
ENSXETG00000000521	417	0	0	0	0	0	0	0	0	0	0	0	0	
ENSXETG00000000522	3361	80594	115634	98605	26138	19511	35824	8167	9676	5426	3567	2508	2862	clca4
ENSXETG00000000523	1406	14	30	22	85	51	309	3307	3886	2342	2894	2066	2197	bhmt
ENSXETG00000000524	867	9	19	6	40	35	11	32	35	11	5	4	5	ankrd45
ENSXETG00000000525	1305	44868	63428	51940	30787	24040	27862	10810	12657	7325	10984	7839	9011	prdx6
ENSXETG00000000526	1422	256	371	308	455	353	482	518	607	354	416	309	335	tada1
ENSXETG00000000527	2613	0	1	0	7	6	23	546	581	364	174	133	173	dmgdh
ENSXETG00000000529	2219	51	53	41	25	21	49	190	250	99	74	63	60	faslg
ENSXETG00000000531	3291	260	423	338	338	289	336	323	451	230	551	496	560	suco
ENSXETG00000000532	3528	177	278	247	316	234	537	635	778	420	418	283	322	kdm4c
ENSXETG00000000533	2301	0	0	0	0	0	0	0	0	0	0	0	0	
ENSXETG00000000535	1547	180	249	216	223	172	371	300	374	220	240	180	214	pigc
ENSXETG00000000538	2598	0	0	0	0	0	0	4	4	1	7	0	3	dnm3
ENSXETG00000000542	1596	431	589	458	580	416	1473	1257	1377	842	474	330	381	arsb
ENSXETG00000000543	3084	1729	2358	2041	3401	2553	4013	1864	2107	1190	3942	2706	3123	gldc
ENSXETG00000000546	678	60	96	73	131	103	273	242	282	165	322	214	253	lhfpl2
ENSXETG00000000547	2818	93	138	111	178	137	340	786	926	498	293	186	250	scamp1
ENSXETG00000000548	2728	2233	2941	2555	2327	1731	1603	1780	2345	1270	944	691	762	sucla2
ENSXETG00000000549	1466	317	463	412	390	311	262	534	624	388	348	245	254	med4
ENSXETG00000000554	1650	10	9	6	4	1	4	9	12	7	12	7	14	ENSXETG00000000554
ENSXETG00000000556	2079	0	0	0	0	0	0	0	0	0	0	0	0	
ENSXETG00000000557	1032	47	66	47	96	90	126	226	331	179	254	175	214	lpar6
ENSXETG00000000559	2034	701	1088	968	877	665	899	1528	1824	1052	948	653	753	rcbtb2
ENSXETG00000000564	2070	18	20	18	34	26	13	18	22	10	18	15	14	c3orf67
ENSXETG00000000566	1641	845	1384	1154	1504	1084	1775	1990	2149	1345	1662	1232	1260	klhdc3
ENSXETG00000000567	2824	463	669	564	1009	791	908	1145	1337	809	1521	1114	1150	uhrf2
ENSXETG00000000568	2325	0	0	0	0	0	0	0	0	0	0	0	0	
ENSXETG00000000572	2958	58	77	75	90	73	204	319	368	189	207	133	160	acvr1b
ENSXETG00000000574	5666	131	194	155	285	203	480	590	625	355	500	375	462	KIAA2026
ENSXETG00000000577	2631	483	757	609	722	585	759	239	297	177	159	104	126	ermp1
ENSXETG00000000578	3288	288	489	392	349	253	539	2012	2320	1369	875	714	741	grasp
ENSXETG00000000579	2531	338	478	368	716	518	2858	547	632	372	889	625	669	nr4a1
ENSXETG00000000580	2673	4	13	13	59	54	177	348	358	247	350	270	312	prpf40b
ENSXETG00000000581	2916	60	80	53	234	179	294	1503	1743	1095	389	307	324	fmnl3
ENSXETG00000000582	4430	196	258	217	355	286	458	548	647	363	287	223	239	ric1
ENSXETG00000000584	717	17	29	14	10	4	15	74	114	54	15	16	15	cd274
ENSXETG00000000585	1225	851	1257	1053	757	556	1116	436	499	264	416	293	343	plgrkt
ENSXETG00000000586	6185	173	293	241	387	290	588	1127	1305	778	574	427	495	jak2
ENSXETG00000000587	1024	0	0	1	0	0	1	0	0	0	0	0	0	
ENSXETG00000000588	728	657	909	826	951	822	984	615	720	414	737	513	594	ak3
ENSXETG00000000589	1146	3	7	1	89	78	110	566	634	387	185	117	153	foxf2
ENSXETG00000000592	1122	649	920	829	511	414	584	927	1081	610	606	420	478	rcl1
ENSXETG00000000593	2073	43	72	67	123	109	255	591	729	445	345	236	282	pcolce2
ENSXETG00000000594	1488	2	2	2	3	4	3	23	19	13	7	6	6	foxc1
ENSXETG00000000595	1508	176	277	243	185	132	269	206	228	133	421	290	340	cdc37l1
ENSXETG00000000596	2403	13	18	14	63	59	147	321	395	245	194	114	151	trpc1
ENSXETG00000000598	2699	35773	50410	42126	24799	19213	30019	1788	2235	1240	5470	3683	4300	pls1
ENSXETG00000000600	1698	3158	4644	4035	5962	4569	5179	3425	3811	2335	10239	7071	8287	gmds
ENSXETG00000000607	3515	32	36	32	67	66	98	275	331	175	162	128	132	rasgef1a
ENSXETG00000000614	924	1	0	0	0	1	0	5	9	0	3	1	1	
ENSXETG00000000615	4280	105	139	109	174	116	299	424	461	242	254	181	195	csgalnact2
ENSXETG00000000617	7950	109	157	148	204	215	265	271	294	166	191	152	177	atr
ENSXETG00000000619	5308	721	1031	942	2021	1503	2622	1888	2293	1298	2120	1488	1691	xrn1
ENSXETG00000000622	1629	0	4	0	4	2	2	8	9	5	9	5	6	znf585b
ENSXETG00000000623	1596	33	57	37	181	127	206	541	585	340	201	158	140	ENSXETG00000000623
ENSXETG00000000624	1263	5852	8196	6790	11097	8447	15391	11152	13923	7520	12354	8778	9825	serpinb1
ENSXETG00000000625	2234	15	18	16	20	12	10	51	41	28	23	19	18	dpp10
ENSXETG00000000626	312	0	0	0	0	0	0	0	0	0	0	0	0	
ENSXETG00000000627	381	0	1	0	0	0	0	0	0	0	0	0	0	
ENSXETG00000000629	2031	3983	6041	5075	6184	4610	7035	10601	11976	7109	7802	5658	6130	wdr1
ENSXETG00000000635	3432	1751	2682	2214	2399	1868	2874	2935	3570	2056	1884	1345	1405	atp13a1
ENSXETG00000000637	1424	117	167	159	88	64	323	142	159	113	63	49	56	ENSXETG00000000637
ENSXETG00000000638	3086	2140	3037	2542	3176	2522	6982	6369	7644	4135	3410	2630	2831	vps4b
ENSXETG00000000640	285	415	575	487	217	151	163	277	276	170	275	209	179	chchd5
ENSXETG00000000641	1026	0	1	0	0	0	0	2	3	0	0	0	0	
ENSXETG00000000644	399	12	12	6	4	9	12	24	28	18	28	28	43	clec4e
ENSXETG00000000645	1110	47	62	69	96	81	161	132	122	71	183	127	134	fbxl12
ENSXETG00000000646	396	7	7	6	12	13	16	76	76	57	26	11	11	clec4m
ENSXETG00000000649	867	0	0	0	0	0	0	0	0	0	0	0	0	

ENSXETG00000000653	1326	85	100	89	57	39	119	127	146	70	119	92	68	ndufaf7
ENSXETG00000000654	3045	642	882	726	547	452	754	992	1296	721	1099	747	881	cebpz
ENSXETG00000000655	1455	1	0	1	5	4	19	3	0	1	2	2	1	trim50
ENSXETG00000000656	1641	51	65	51	94	56	164	206	207	141	215	174	166	ENSXETG00000000656
ENSXETG00000000658	2037	13	20	23	123	98	151	127	179	122	275	169	235	paqr8
ENSXETG00000000659	1518	6	9	5	374	295	23	7	6	5	27	24	20	efhc1
ENSXETG00000000663	4222	123	152	123	267	225	362	2523	2798	1735	418	294	346	tram2
ENSXETG00000000664	561	218	373	283	326	226	377	191	258	126	266	197	205	tmem220
ENSXETG00000000667	3219	235	314	267	565	435	1262	1409	1531	1028	1121	773	849	DAB2IP
ENSXETG00000000668	2608	8	15	16	35	28	26	135	115	60	22	7	4	xkr5
ENSXETG00000000671	1846	9636	13936	12030	17506	13683	64673	86457	97232	61683	29770	20828	22784	ctsb
ENSXETG00000000673	1580	483	799	689	1829	1342	1715	334	357	228	2036	1330	1558	gata4
ENSXETG00000000679	942	196	288	245	677	489	1506	2504	2950	1834	505	352	413	fgl1
ENSXETG00000000681	4497	274	400	331	1019	736	1530	946	1060	660	1391	1020	1149	mtus1
ENSXETG00000000682	1122	14	19	22	76	71	38	113	126	79	239	166	150	tdh
ENSXETG00000000683	2100	4	12	6	10	6	2	16	17	10	17	27	17	slc7a2.1
ENSXETG00000000685	3481	706	1024	890	1197	853	2061	1884	2125	1231	1441	1080	1161	mtmr9
ENSXETG00000000686	2623	1	2	1	1	0	2	6	3	3	7	3	3	xkr6
ENSXETG00000000688	1231	1862	2819	2354	3766	3004	5985	6307	6880	4295	19366	13693	14972	pinx1
ENSXETG00000000689	1598	57	87	62	99	68	149	379	469	286	148	102	111	pdgfrl
ENSXETG00000000690	1980	4	4	6	13	15	16	58	66	40	31	12	14	mtmr7
ENSXETG00000000693	2032	23	38	27	94	77	253	420	451	265	228	153	195	sox7
ENSXETG00000000694	1645	0	0	0	7	10	45	133	113	71	135	77	94	zdhhc2
ENSXETG00000000696	2916	767	1059	953	2376	1892	3132	5135	5919	3591	1751	1262	1441	rai14
ENSXETG00000000697	1194	529	772	657	579	414	817	1028	1077	652	703	504	563	vps37a
ENSXETG00000000700	1125	1110	1564	1403	1030	848	1785	453	475	302	584	447	487	msra.1
ENSXETG00000000701	2456	414	567	432	317	272	411	688	780	502	483	308	386	cnot7
ENSXETG00000000702	1158	2	0	0	66	44	2	3	2	1	2	3	2	ttc23l
ENSXETG00000000703	1107	1068	1560	1357	886	671	616	1255	1405	822	1031	727	841	brix1
ENSXETG00000000704	897	0	0	0	0	0	0	0	0	0	0	0	0	
ENSXETG00000000705	1514	108	152	137	201	164	404	308	331	200	476	319	383	rad1
ENSXETG00000000706	1608	89	120	114	117	89	141	255	291	219	241	191	211	dnajc21
ENSXETG00000000707	1395	17	8	13	125	101	155	15	9	15	161	153	119	agxt2
ENSXETG00000000708	520	24	44	33	37	45	58	81	93	55	108	55	70	ENSXETG00000000708
ENSXETG00000000709	1812	80	112	106	156	117	399	198	207	128	375	249	289	prlr
ENSXETG00000000715	1819	3	3	2	24	6	13	21	32	18	29	22	22	hoxa1
ENSXETG00000000716	1374	43	66	53	269	194	203	85	129	61	380	249	270	hoxa2
ENSXETG00000000717	2155	41	53	56	285	218	264	377	380	280	501	344	366	hoxa3
ENSXETG00000000719	4685	7	7	10	141	110	495	1943	2226	1342	990	733	820	l1cam
ENSXETG00000000720	1645	9	17	8	66	46	125	490	536	332	239	158	190	hoxa5
ENSXETG00000000722	624	5	11	12	26	22	40	114	105	58	25	29	25	hoxa7
ENSXETG00000000723	2463	5	22	8	106	73	142	1145	1444	855	513	369	409	nacc2
ENSXETG00000000724	786	2	0	2	6	8	19	90	89	67	8	8	7	hoxa9
ENSXETG00000000725	6105	0	0	0	3	1	6	24	28	19	16	20	18	pdzd4
ENSXETG00000000726	1209	0	0	0	1	0	1	3	0	1	0	0	0	lhx3
ENSXETG00000000727	1212	27	40	39	68	64	69	95	109	70	70	49	55	ENSXETG00000000727
ENSXETG00000000728	1080	0	0	0	3	7	2	23	21	10	1	1	1	
ENSXETG00000000729	4184	315	465	393	511	396	882	1663	1832	1211	521	394	452	qsox2
ENSXETG00000000730	618	10521	15464	12990	9461	7242	10173	8570	9725	5572	9581	6802	7384	ssr4
ENSXETG00000000731	1258	1788	2738	2359	1666	1248	1759	1625	1937	1250	1539	1157	1211	idh3g
ENSXETG00000000732	849	0	0	0	1	0	0	35	40	22	3	0	0	
ENSXETG00000000733	2004	49	74	57	116	103	152	452	469	286	308	202	248	gpsm1
ENSXETG00000000734	900	1	0	2	63	61	1	105	104	89	1	0	3	hoxa13
ENSXETG00000000735	2992	72	97	73	119	107	326	335	401	264	546	346	460	srpk2
ENSXETG00000000736	1173	0	0	1	1	0	0	0	0	0	1	0	0	evx1
ENSXETG00000000739	1131	385	621	520	678	508	1034	648	700	413	1399	908	1045	dnlz
ENSXETG00000000740	2164	1560	2237	2050	1647	1311	2184	1441	1554	922	1535	1089	1248	card9
ENSXETG00000000742	879	6	2	1	5	6	2	8	9	12	9	4	9	lmx1b.1
ENSXETG00000000743	4103	50	63	49	177	129	142	335	342	193	176	130	149	snapc4
ENSXETG00000000745	1935	29	46	35	59	48	97	371	393	237	120	100	97	mvb12b
ENSXETG00000000747	2116	247	381	346	271	213	345	498	535	344	508	359	410	sdccag3
ENSXETG00000000748	1609	1062	1663	1329	1709	1340	1251	1719	1951	1244	1697	1278	1311	pmpca
ENSXETG00000000750	2303	757	1145	916	1421	1040	1965	1075	1217	697	1757	1247	1382	hibadh
ENSXETG00000000752	2481	617	846	696	1653	1230	2859	1784	2202	1283	2597	1718	2058	tax1bp1
ENSXETG00000000753	1653	93	105	81	145	90	193	406	439	270	213	128	139	sec16a
ENSXETG00000000754	546	6	9	6	38	24	49	100	77	51	94	66	73	jazf1
ENSXETG00000000757	7616	134	162	159	521	376	674	1237	1365	889	1102	808	823	notch1
ENSXETG00000000758	4853	1261	1849	1503	2458	2015	5493	3355	3846	2252	2725	1996	2217	pcsk5
ENSXETG00000000759	903	579	805	665	310	224	690	626	766	428	197	161	187	rfk
ENSXETG00000000760	1287	1065	1372	1186	851	726	833	632	840	436	568	379	446	gcnt1
ENSXETG00000000762	2043	107	188	151	412	309	399	1496	1679	1053	387	266	284	egfl7
ENSXETG00000000763	984	1121	1614	1433	1773	1302	1731	482	505	329	1119	771	829	agpat2
ENSXETG00000000764	1704	0	0	0	0	0	0	2	1	0	0	2	0	
ENSXETG00000000775	2927	23	32	32	67	47	150	719	747	453	142	95	89	clstn1
ENSXETG00000000776	1831	2511	3532	3104	2560	1949	4812	3465	3976	2264	2502	1746	1900	snx5
ENSXETG00000000777	4553	2416	3549	3041	3631	2657	2170	3837	4263	2462	3252	2249	2576	rrbp1
ENSXETG00000000781	1818	599	952	831	1624	1218	1429	3570	4261	2529	1257	916	1020	ssrp1
ENSXETG00000000782	6048	161	239	204	466	340	351	684	848	485	410	279	334	anapc1
ENSXETG00000000783	2691	7171	10529	9089	5795	4417	5763	5526	6140	3855	2451	1791	2062	sec23b
ENSXETG00000000784	1261	237	377	280	2242	1671	226	47	66	38	94	56	72	hebp2
ENSXETG00000000786	2000	534	809	611	572	438	933	1127	1316	788	909	636	706	dusp12
ENSXETG00000000787	2700	98	147	126	272	221	256	533	672	396	224	124	158	tubgcp2
ENSXETG00000000790	1223	249	325	323	255	177	271	479	489	319	200	152	158	dtd1
ENSXETG00000000793	2371	3	9	7	53	46	175	247	268	206	179	122	138	slc24a3
ENSXETG00000000794	3033	7	6	8	31	16	48	137	130	82	87	64	56	klhl32
ENSXETG00000000796	3702	59	85	67	172	149	56	205	225	144	53	37	30	mms22l
ENSXETG00000000797	1221	1	3	4	5	7	20	39	51	38	22	19	13	faxc
ENSXETG00000000799	1007	201	336	270	207	133	326	308	324	201	193	144	141	coq3
ENSXETG00000000800	2695	760	1101	870	1594	1279	1223	1805	2009	1242	4196	2991	3249	pnisr
ENSXETG00000000802	4437	11	30	11	96	53	171	855	981	530	418	286	341	ror2
ENSXETG00000000804	1389	2810	4081	3305	1278	985	2163	1020	1259	639	1991	1398	1592	nfil3
ENSXETG00000000805	1506	609	853	743	1000	795	1068	818	907	528	661	439	527	auh
ENSXETG00000000807	1842	1220	1729	1527	1422	1021	2338	1776	2120	1164	1531	1061	1361	syk
ENSXETG00000000809	472	194	368	278	310	168	261	287	328	172	633	475	472	ENSXETG00000000809
ENSXETG00000000810	913	2	6	2	4	2	1	12	22	22	12	8	8	diras2
ENSXETG00000000831	687	7	13	4	38	28	13	68	68	27	36	36	26	lurap1l
ENSXETG00000000835	3996	37	56	61	556	398	911	6146	6977	4406	1867	1389	1513	nfib
ENSXETG00000000836	2364	65	106	88	177	145	318	469	501	312	345	289	279	stat5b
ENSXETG00000000838	1347	975	1531	1322	1540	1124	1328	1703	1914	1320	1538	1148	1174	tsn
ENSXETG00000000839	732	956	1312	1065	973	697	1259	1144	1285	720	2711	1902	1996	nifk
ENSXETG00000000840	4902	288	470	395	985	786	1029	1662	1756	1129	1261	885	995	clasp1
ENSXETG00000000843	1497	135	184	186	261	182	308	370	478	248	147	106	122	foxred1
ENSXETG00000000845	3851	0	0	0	0	0	2	0	0	0	0	0	1	
ENSXETG00000000846	1472	217	339	239	288	212	981	627	697	380	178	105	123	asl
ENSXETG00000000847	438	14	27	21	3	2	1	41	43	30	0	1	0	ENSXETG00000000847
ENSXETG00000000848	504	906	1260	1166	1074	794	1185	975	1220	705	725	436	560	echs1
ENSXETG00000000849	1346	764	1059	852	637	518	884	767	894	494	811	571	595	erlin1
ENSXETG00000000852	3336	143	180	169	272	203	542	539	594	365	356	271	286	atp9b
ENSXETG00000000853	5361	151	225	242	257	251	678	882	1054	595	549	391	463	nfatc1
ENSXETG00000000855	1551	0	0	0	0	0	0	0	0	0	0	0	0	
ENSXETG00000000856	3079	125	170	186	461	367	474	541	612	360	709	490	532	ctdp1

ENSXETG00000000858	747	13	15	13	110	82	132	89	93	47	157	115	133	kcng2
ENSXETG00000000864	564	26	42	34	80	69	141	359	364	296	235	166	201	ENSXETG00000000864
ENSXETG00000000866	1383	650	944	858	1172	846	1578	1249	1326	866	1127	775	823	bcl2l13
ENSXETG00000000869	1749	937	1372	1182	1430	1111	4112	5797	6517	3813	1420	1025	1187	atp6v1e1
ENSXETG00000000870	2706	197	264	258	642	523	1695	803	930	472	760	476	558	slc25a18
ENSXETG00000000873	4103	15	17	20	18	23	17	65	60	40	30	27	23	cecr2
ENSXETG00000000874	1410	16	17	13	117	101	162	443	469	310	340	257	276	cecr1
ENSXETG00000000878	1163	132	185	174	174	150	366	600	702	449	218	187	173	cecr5
ENSXETG00000000887	1575	717	1085	924	1188	889	1735	2367	2696	1713	1226	881	1026	rraga
ENSXETG00000000890	921	0	0	0	0	0	0	0	0	0	0	0	0	
ENSXETG00000000896	2577	10	23	21	232	211	575	1472	1576	989	831	595	695	abcc9
ENSXETG00000000899	1659	134	195	165	360	239	346	384	502	267	498	348	393	dcp1b
ENSXETG00000000900	2997	16	18	18	109	117	93	226	274	193	493	389	437	cacna2d4
ENSXETG00000000901	2014	0	0	0	0	0	0	17	20	10	5	12	9	
ENSXETG00000000902	3174	35	50	55	176	138	240	374	458	257	308	223	258	dstyk
ENSXETG00000000903	2223	296	439	351	438	370	615	1187	1319	781	516	390	426	stim2
ENSXETG00000000904	2257	27	27	30	26	13	30	71	83	47	49	34	37	il17rd
ENSXETG00000000906	1584	8	11	4	21	18	11	174	187	106	24	16	12	tbc1d19
ENSXETG00000000907	2324	398	540	489	508	421	600	273	407	234	471	319	385	tmem38b
ENSXETG00000000908	318	0	0	0	0	0	0	0	0	0	0	0	0	
ENSXETG00000000909	1506	13	18	16	16	18	17	41	44	24	20	12	12	fsd1l
ENSXETG00000000910	1145	3	6	3	29	24	48	375	429	251	81	51	68	arl8a
ENSXETG00000000911	1233	1	0	0	2	3	1	1	1	2	2	1	2	cckar
ENSXETG00000000914	2108	12094	16938	14031	5284	4117	5712	383	409	255	313	219	288	msmo1
ENSXETG00000000916	3391	142	189	180	200	158	262	449	519	312	191	140	143	fktn
ENSXETG00000000918	1530	1	0	0	0	0	0	0	0	0	0	0	0	
ENSXETG00000000921	1479	15	17	21	77	59	142	798	883	537	261	175	186	arhgef3.2
ENSXETG00000000923	4798	255	349	301	570	418	961	1250	1406	875	1262	860	1011	FAM208A
ENSXETG00000000924	3014	55	76	67	290	209	226	283	279	200	218	179	174	ccdc66
ENSXETG00000000925	1869	62	93	66	184	176	302	1019	1106	673	512	340	416	slc44a1
ENSXETG00000000927	1467	22	37	47	154	111	161	88	107	56	183	120	136	ENSXETG00000000927
ENSXETG00000000929	2430	0	0	0	4	2	27	5	10	1	5	1	3	erc2
ENSXETG00000000930	1474	458	716	572	212	162	703	51	71	41	1032	740	757	pnlip
ENSXETG00000000931	1407	0	2	1	0	0	0	0	2	1	0	0	0	
ENSXETG00000000933	2559	379	567	476	818	649	824	1586	1935	1087	627	518	495	rbbp5
ENSXETG00000000935	1470	31	64	47	137	82	108	328	390	214	145	74	93	rbpj
ENSXETG00000000936	1063	0	2	0	124	108	25	2	2	0	74	44	54	ccdc172
ENSXETG00000000940	1044	0	0	0	4	3	2	0	0	1	72	44	57	ENSXETG00000000940
ENSXETG00000000941	270	0	1	0	1	2	1	4	4	1	1	2	1	ENSXETG00000000941
ENSXETG00000000947	3039	519	761	616	993	784	1852	661	736	418	1712	1174	1323	sel1l3
ENSXETG00000000950	1995	384	605	470	390	288	496	295	354	184	219	158	165	tmem62
ENSXETG00000000951	6807	1372	2067	1818	3208	2475	4236	5965	7071	4080	2947	2097	2424	abca1
ENSXETG00000000953	2675	538	834	652	389	323	550	350	369	240	424	279	312	ptgr2
ENSXETG00000000954	2040	7376	10951	9203	24408	18538	14634	10953	12636	7434	19639	13556	15444	slc34a2
ENSXETG00000000956	1332	0	0	0	0	0	1	1	0	0	0	0	0	ENSXETG00000000956
ENSXETG00000000957	3067	1017	1438	1304	2010	1404	2056	1920	2225	1292	2176	1478	1699	anapc4
ENSXETG00000000960	1475	188	257	201	338	279	479	395	468	238	539	415	430	zcchc4
ENSXETG00000000961	5223	202	307	250	639	505	777	956	1147	613	790	606	644	ubr1
ENSXETG00000000962	1900	217	281	223	530	399	892	557	648	381	636	439	506	pi4k2b
ENSXETG00000000963	2044	16373	24288	21057	17806	13559	19505	10098	11189	6854	7928	5756	6339	rnpep
ENSXETG00000000967	2316	327	499	403	596	397	1083	636	734	434	856	613	686	nadsyn1
ENSXETG00000000972	2803	67	119	113	216	153	518	516	564	337	553	435	439	dcaf6
ENSXETG00000000973	4440	21	31	28	67	55	76	184	234	124	112	72	89	tmcc2
ENSXETG00000000974	3519	887	1271	1098	2017	1586	2346	1011	1172	641	1466	1030	1117	nuak2
ENSXETG00000000976	2031	23	29	33	104	92	260	455	539	309	317	236	259	klhdc8a
ENSXETG00000000978	4218	0	0	0	0	0	0	0	0	0	0	0	0	
ENSXETG00000000979	3986	13	26	23	51	40	65	168	239	130	108	73	78	ttbk2
ENSXETG00000000980	1395	348	524	431	283	232	621	164	201	122	371	236	300	cdk18
ENSXETG00000000982	840	1520	2292	1904	1620	1232	1729	1512	1751	1048	749	537	580	zbtb8os
ENSXETG00000000983	1095	0	0	0	0	0	0	0	0	0	0	0	0	
ENSXETG00000000984	1516	361	515	447	527	445	296	1139	1364	767	404	238	311	rbbp4
ENSXETG00000000985	2531	72	105	91	97	71	156	95	103	54	202	146	182	mfsd4a
ENSXETG00000000986	3639	62	76	76	147	123	137	189	217	134	250	196	212	cdan1
ENSXETG00000000987	1236	67	103	97	143	128	224	362	377	225	363	249	277	elk4
ENSXETG00000000988	4428	6	8	6	74	55	57	405	478	274	66	58	67	stard9
ENSXETG00000000990	2857	1116	1669	1369	2713	2005	5185	1864	2022	1261	3907	2823	3181	kiaa1522
ENSXETG00000000991	3293	1108	1497	1328	2403	1780	3605	2917	3445	1961	3292	2337	2606	numb
ENSXETG00000000992	885	8	16	21	25	20	75	44	53	52	168	116	125	klf15.2
ENSXETG00000000993	1419	60	80	75	143	99	220	139	157	96	274	196	225	slc45a3
ENSXETG00000000994	2823	36	62	65	197	179	523	896	1017	626	531	367	423	ENSXETG00000000994
ENSXETG00000000997	3634	45	66	55	176	146	499	759	804	556	423	293	372	kiaa1109
ENSXETG00000000998	1121	190	255	227	302	243	290	676	784	432	299	221	198	ENSXETG00000000998
ENSXETG00000001000	1449	1764	2525	2242	4215	3226	3765	7480	8194	4891	5114	3516	3905	nucks1
ENSXETG00000001001	2406	2	2	0	0	3	0	10	11	6	0	1	0	hpca
ENSXETG00000001002	3633	14	19	10	97	75	222	1116	1247	801	241	205	189	papln
ENSXETG00000001003	2634	2	0	0	13	6	22	23	28	18	35	19	19	trpc3
ENSXETG00000001004	603	5	9	4	15	11	12	65	87	54	23	22	15	tmem54
ENSXETG00000001007	765	59	71	60	250	178	347	1627	1926	994	679	511	503	clic5
ENSXETG00000001008	2047	1572	2256	1913	2465	1874	4136	2664	3270	1720	3010	2120	2310	psen1
ENSXETG00000001015	1257	1	0	1	3	0	7	9	27	9	6	7	8	chst1
ENSXETG00000001016	1550	204	267	217	435	306	295	1060	1204	705	76	65	58	ccna2
ENSXETG00000001017	1095	323	440	344	434	356	519	786	847	566	525	351	383	slc35c1
ENSXETG00000001021	4595	32	41	26	106	65	151	932	1070	633	218	158	188	acap3
ENSXETG00000001022	2136	2182	3010	2473	3083	2443	4597	3186	3748	2038	5372	3698	4205	tmem248
ENSXETG00000001023	1656	449	671	650	2250	1721	2521	728	764	511	1615	1169	1241	cry2
ENSXETG00000001025	1956	2	9	2	18	24	22	79	86	57	99	71	76	mapk8ip1
ENSXETG00000001026	1815	1	3	1	2	1	4	2	2	2	3	0	1	scnn1d
ENSXETG00000001027	991	693	1070	908	1074	853	774	611	667	400	426	327	353	pex16
ENSXETG00000001028	2247	1397	1963	1763	4257	3182	4893	2117	2233	1409	5003	3462	3699	large2
ENSXETG00000001029	1353	51	68	62	57	47	186	905	981	624	218	140	147	runx3
ENSXETG00000001030	1653	0	1	4	244	160	16	1	0	0	713	494	511	ENSXETG00000001030
ENSXETG00000001031	1881	64	84	77	253	203	492	682	815	465	506	347	377	phf21a
ENSXETG00000001033	1869	306	384	333	402	296	531	662	713	407	665	453	456	ube2j2
ENSXETG00000001035	1153	4	4	5	39	36	89	989	1045	616	422	262	351	fam132a
ENSXETG00000001036	1032	31	52	38	65	41	103	171	164	119	113	66	88	b3galt6
ENSXETG00000001037	2431	2210	3213	2624	2377	1844	3809	3954	4726	2656	2455	1642	1953	sdf4
ENSXETG00000001040	1521	0	4	0	9	2	2	187	215	132	10	3	3	ENSXETG00000001040
ENSXETG00000001042	2772	132	188	158	139	106	213	350	423	234	187	140	175	XB-GENE-5819923
ENSXETG00000001046	1746	143	251	172	178	130	239	286	355	202	137	100	127	carnmt1
ENSXETG00000001048	5988	238	393	318	517	346	1422	408	509	263	1788	1257	1459	trpm6
ENSXETG00000001053	914	135	221	176	75	73	210	312	329	196	66	55	70	tmem53
ENSXETG00000001054	4334	296	424	401	839	626	739	867	985	610	1206	833	924	rnf220.1
ENSXETG00000001057	1885	317	454	405	737	573	945	1095	1301	784	890	586	687	dmap1
ENSXETG00000001058	2056	173	229	203	591	485	605	409	491	309	1095	802	892	klf17
ENSXETG00000001060	1350	149	210	154	208	180	625	231	344	157	213	178	185	maneal
ENSXETG00000001061	1083	350	484	379	135	114	438	30	32	27	16	12	17	fut9
ENSXETG00000001063	638	1330	1890	1663	1057	825	1921	2526	3020	1739	1133	743	809	cst3
ENSXETG00000001064	2388	398	581	469	649	476	1071	717	894	499	563	426	453	ufl1
ENSXETG00000001065	1801	10	22	14	26	19	34	105	119	47	31	19	24	fhl5
ENSXETG00000001066	1335	0	2	0	1	2	0	8	6	8	3	0	3	gpr63
ENSXETG00000001067	2946	0	0	0	0	0	1	0	0	0	0	2	1	sycp2l

ENSXETG00000001068	1467	0	0	0	0	0	0	0	0	0	0	0	0	
ENSXETG00000001069	1975	10	12	12	22	28	39	47	59	26	8	11	18	mak
ENSXETG00000001070	1506	135	214	162	159	126	179	249	268	161	150	133	94	tbc1d7
ENSXETG00000001071	675	1328	1842	1637	1399	1057	1073	1286	1504	900	546	357	413	tmem14c
ENSXETG00000001072	1228	408	534	514	227	186	453	593	710	428	462	309	339	pak1ip1
ENSXETG00000001073	2089	3	4	4	7	8	52	184	236	140	45	27	25	gcnt2
ENSXETG00000001074	1818	0	1	0	9	8	4	34	31	17	8	4	5	tfap2a
ENSXETG00000001078	2056	628	846	723	612	470	1094	1357	1518	880	422	308	329	adprh
ENSXETG00000001079	1557	9	6	6	3	3	9	25	45	29	7	16	18	ENSXETG00000001079
ENSXETG00000001081	2168	262	401	336	479	396	575	693	905	458	981	675	756	armc8
ENSXETG00000001082	1519	885	1241	1062	970	709	2063	1365	1718	931	1294	887	1038	rab5d
ENSXETG00000001083	3325	82	132	118	360	255	251	24	32	20	77	54	71	nme9
ENSXETG00000001084	910	2775	4164	3480	2077	1506	3323	1813	2169	1232	1244	873	923	dpepe
ENSXETG00000001085	2834	3386	4892	4250	5782	4423	7609	3899	4625	2678	2923	1998	2225	acadl
ENSXETG00000001086	1362	0	0	0	0	0	0	0	0	0	0	0	0	
ENSXETG00000001087	2361	0	0	0	0	0	0	0	0	1	0	0	0	
ENSXETG00000001088	4522	21	14	22	41	31	47	87	97	51	71	51	60	kansl1l
ENSXETG00000001090	2562	0	0	0	0	0	0	0	0	0	0	0	0	
ENSXETG00000001092	2226	0	0	0	0	0	0	0	0	0	0	0	0	
ENSXETG00000001093	519	8	12	9	1	0	1	0	2	2	1	1	0	ENSXETG00000001093
ENSXETG00000001097	2484	0	0	0	0	0	0	0	0	0	0	0	0	ENSXETG00000001097
ENSXETG00000001099	1362	0	0	0	0	0	0	0	0	0	0	0	0	
ENSXETG00000001108	522	420	626	554	261	235	352	662	814	489	216	172	190	polr2j
ENSXETG00000001109	1461	277	421	356	488	371	579	498	662	339	482	330	379	txlng
ENSXETG00000001110	1740	37	36	28	44	28	69	64	83	47	67	47	58	reps2
ENSXETG00000001112	4257	8	8	13	47	30	61	180	199	134	205	126	173	nhs
ENSXETG00000001114	1671	1	9	2	27	16	61	585	698	357	150	103	118	rai2
ENSXETG00000001116	4053	46	51	52	157	96	186	673	789	433	191	137	150	scml2
ENSXETG00000001117	3060	71	145	115	256	201	242	340	398	249	189	165	158	smg8
ENSXETG00000001118	2934	92	134	100	276	167	189	283	315	178	433	322	351	trim37
ENSXETG00000001119	1782	1	5	1	2	3	16	71	72	53	26	24	27	ppm1e
ENSXETG00000001122	1092	197	222	177	87	75	85	135	185	97	68	69	81	rad51c
ENSXETG00000001124	1092	0	0	0	0	0	1	0	0	1	0	0	0	
ENSXETG00000001133	525	0	0	0	3	1	0	0	0	0	2	3	1	
ENSXETG00000001136	1984	479	712	586	620	439	845	1194	1362	763	1253	889	929	nprl3
ENSXETG00000001137	556	0	0	0	0	0	0	0	0	0	0	0	0	
ENSXETG00000001141	598	1026	1566	1378	725	547	5878	2189	2536	1388	1169	780	866	hba4
ENSXETG00000001142	1047	264	375	344	208	168	209	400	409	268	216	168	186	polr3k
ENSXETG00000001143	1212	86	164	175	87	81	76	222	304	161	84	57	65	snrnp25
ENSXETG00000001148	5268	11010	15738	13531	54357	41172	81171	362	453	278	72680	51039	60084	mgam
ENSXETG00000001152	4696	1	0	0	1	1	1	8	11	4	13	12	16	slitrk3
ENSXETG00000001154	4321	15	27	12	72	55	148	376	458	288	202	146	161	gpr153
ENSXETG00000001157	873	19	32	26	65	47	34	13	15	9	166	100	116	cd22
ENSXETG00000001163	862	3	0	1	3	1	2	9	4	8	4	1	3	hes2
ENSXETG00000001165	2520	454	633	588	199	190	319	267	326	182	241	162	164	espn
ENSXETG00000001167	3048	466	726	621	376	278	1140	943	1073	694	631	493	545	plekhg5
ENSXETG00000001170	1529	2886	4252	3735	1912	1365	870	3343	3775	2180	511	389	413	gale
ENSXETG00000001171	657	0	2	0	2	0	8	5	7	2	9	2	2	ENSXETG00000001171
ENSXETG00000001173	753	2	1	3	19	17	11	187	199	148	34	22	27	ENSXETG00000001173
ENSXETG00000001175	2019	74	147	122	390	264	336	665	737	444	446	317	316	TNK1
ENSXETG00000001176	2243	188	278	215	423	327	579	650	694	412	619	467	501	eloa
ENSXETG00000001177	1133	1	1	1	0	0	1	12	16	24	26	26	30	clec10a
ENSXETG00000001178	580	60810	90792	79326	75429	58136	72944	63520	71605	44717	150752	105317	115312	rpl11
ENSXETG00000001179	1282	4	8	5	91	70	196	122	151	94	595	421	403	dlg4
ENSXETG00000001182	1690	369234	542652	466877	468528	355735	706610	534756	604725	363331	1139540	788608	873595	eef1a1
ENSXETG00000001183	909	986	1554	1313	1204	933	2005	956	1099	692	1776	1230	1381	ppt1
ENSXETG00000001184	2155	10081	15082	12229	12833	9740	16750	18499	21259	12382	9513	6746	7398	cap1
ENSXETG00000001185	2216	1073	1542	1292	1193	908	977	716	917	460	1371	966	1062	mfsd2a
ENSXETG00000001192	7771	794	1174	993	2586	1986	4085	4888	5406	3187	3215	2230	2471	ncor2
ENSXETG00000001195	1407	2452	3583	2904	4274	3205	10405	1972	2371	1358	2828	2018	2271	scarb1
ENSXETG00000001197	165	52	67	79	173	125	294	222	273	122	267	172	204	commd7
ENSXETG00000001201	2025	0	0	0	0	1	0	1	1	1	0	0	0	allc
ENSXETG00000001202	2823	164	246	225	221	158	240	445	497	285	485	297	383	dhx37
ENSXETG00000001203	1980	0	0	0	0	0	0	0	0	0	0	0	0	
ENSXETG00000001204	861	1	0	1	17	9	24	84	119	71	42	45	43	colec11
ENSXETG00000001205	1002	0	0	0	0	0	0	0	0	0	0	0	0	
ENSXETG00000001207	1755	103	148	155	170	145	254	436	518	335	193	128	128	bri3bp
ENSXETG00000001209	661	45059	67027	57999	62698	47886	68753	63801	73538	44151	161558	115302	126551	rps7
ENSXETG00000001210	2099	143	210	187	577	453	417	730	831	469	504	373	415	aacs
ENSXETG00000001212	1941	207	319	251	267	168	428	350	410	218	359	255	264	rnaseh1
ENSXETG00000001215	546	3	6	11	12	7	10	5	9	6	5	3	5	
ENSXETG00000001218	777	831	1214	1055	1082	808	1471	966	1042	609	639	489	551	cgref1
ENSXETG00000001220	1050	865	1377	1153	1840	1485	3245	471	577	358	2315	1654	1745	khk
ENSXETG00000001223	3401	146	178	179	1402	1051	2274	6355	7274	4263	3604	2661	2807	emilin1
ENSXETG00000001225	810	111	165	154	235	185	545	9	8	8	390	267	305	mapre3
ENSXETG00000001226	795	838	1295	1203	1276	941	1256	1233	1370	847	1029	772	804	ENSXETG00000001226
ENSXETG00000001227	1689	2	4	5	44	22	28	107	137	81	46	28	24	dpysl5
ENSXETG00000001229	3345	0	0	0	5	5	39	0	0	0	15	8	11	ATP12A
ENSXETG00000001232	2643	46	95	76	294	212	503	1773	1853	1236	699	451	545	dnmt3a
ENSXETG00000001233	1524	19	35	29	118	95	213	281	361	212	344	268	273	dtnb
ENSXETG00000001239	1704	42	45	41	37	23	65	122	142	72	133	82	107	scn3b
ENSXETG00000001240	1956	120	178	152	314	206	914	846	944	543	1653	1126	1386	gramd1b
ENSXETG00000001242	1473	61	100	75	395	299	1211	1256	1401	850	1361	1038	1120	clmp
ENSXETG00000001243	6810	1753	2477	2189	5852	4310	5486	5400	6140	3646	5650	3988	4494	snrnp200
ENSXETG00000001244	3429	340	481	416	528	496	465	910	1058	652	553	381	423	nup133
ENSXETG00000001245	2510	132	202	186	507	359	255	663	807	490	91	74	73	ncaph
ENSXETG00000001246	3597	10	25	24	23	19	38	144	147	102	118	71	97	slk
ENSXETG00000001247	2577	219	303	289	616	480	535	864	932	526	783	551	581	kansl3
ENSXETG00000001248	1605	660	1016	897	825	601	986	1005	1128	720	640	482	482	lman2l
ENSXETG00000001249	4794	200	298	276	1092	844	1104	1092	1170	766	1428	963	997	cnnm2
ENSXETG00000001251	2415	95	164	134	196	139	320	796	912	521	354	332	292	sema4c
ENSXETG00000001252	3546	1	1	0	5	4	15	33	38	26	10	8	5	kirrel2
ENSXETG00000001253	1122	267	344	316	749	575	980	907	1037	693	879	621	662	gna13
ENSXETG00000001255	516	7407	10952	9072	6949	5189	5877	5586	6310	3976	3410	2377	2614	cox5b.1
ENSXETG00000001257	942	121	176	174	358	275	615	727	791	533	479	397	399	purb
ENSXETG00000001258	892	2999	4420	3727	4294	3193	3742	7389	8484	4991	2667	1863	2062	h2afv
ENSXETG00000001259	4642	127	185	145	301	259	260	443	514	315	534	398	413	polm
ENSXETG00000001262	1590	1729	2554	2081	473	322	420	379	407	290	99	68	64	slc16a6
ENSXETG00000001263	1092	12065	17766	15455	3109	2372	1537	2571	2810	1716	3206	2233	2448	mat2a
ENSXETG00000001264	2269	228	313	256	589	510	706	1217	1433	829	497	322	368	ggcx
ENSXETG00000001266	937	926	1371	1153	1082	733	1516	1275	1521	899	1224	918	982	rnf181
ENSXETG00000001267	810	109	158	139	197	158	223	626	723	440	254	182	216	tmem150a
ENSXETG00000001269	1509	728	1038	909	898	709	1031	1749	1912	1168	840	655	673	usp39
ENSXETG00000001272	3833	0	0	0	0	0	0	0	0	0	0	0	0	
ENSXETG00000001273	1201	92	153	117	191	147	263	555	632	381	316	236	236	psd4
ENSXETG00000001276	867	42	64	43	75	57	111	300	360	205	88	48	74	il1b
ENSXETG00000001281	2631	576	796	708	919	666	1561	1385	1543	923	1515	1047	1176	gaa
ENSXETG00000001288	1535	516	830	657	1024	758	1789	1935	2227	1307	1450	1103	1228	csnk1d
ENSXETG00000001289	1279	897	1235	1038	928	711	1391	1571	1871	1062	720	524	602	trappc5
ENSXETG00000001291	2204	170	242	191	268	248	515	614	688	413	428	326	366	mbtd1
ENSXETG00000001293	1824	620	885	770	640	501	622	906	1102	687	857	550	651	utp18

ENSXETG00000001294	3971	209	262	251	353	290	619	601	679	423	578	450	492	crtc3
ENSXETG00000001296	990	0	0	0	0	0	1	14	14	5	4	4	6	ca10
ENSXETG00000001299	5317	243	321	312	818	639	1074	4204	5048	2974	1259	958	1006	iqgap1
ENSXETG00000001300	3846	1	0	0	0	1	0	0	1	0	1	1	0	ENSXETG00000001300
ENSXETG00000001302	5293	0	2	2	56	35	9	2	6	1	6	8	7	noxred1
ENSXETG00000001304	771	0	0	0	0	0	0	0	0	0	0	0	0	
ENSXETG00000001305	1101	30	42	38	73	39	65	88	94	69	68	48	31	spopl
ENSXETG00000001307	531	0	0	0	0	0	0	9	4	5	22	11	11	pthlh
ENSXETG00000001312	1122	959	1372	1146	1564	1246	1877	1165	1336	864	3578	2489	2836	mrps35
ENSXETG00000001314	1671	9	15	11	93	85	75	359	429	229	104	69	79	tulp3
ENSXETG00000001315	2220	38	40	50	207	142	107	336	339	222	28	25	26	foxm1
ENSXETG00000001318	855	0	1	0	0	0	0	3	2	3	0	0	0	
ENSXETG00000001319	1233	5	26	13	44	23	34	23	36	18	25	28	21	ENSXETG00000001319
ENSXETG00000001320	621	0	0	0	0	0	0	0	0	0	2	0	1	
ENSXETG00000001321	513	17	23	16	0	0	1	0	0	0	0	0	0	ENSXETG00000001321
ENSXETG00000001322	2209	3250	4618	3665	3423	2753	7808	5594	6768	3754	5674	4008	4757	syngr2
ENSXETG00000001323	886	108	150	124	183	145	60	515	566	322	47	47	38	tk1
ENSXETG00000001324	1668	14	27	29	48	33	99	130	157	95	55	41	40	dgke
ENSXETG00000001325	669	28	51	40	81	66	204	399	411	269	277	222	250	nog
ENSXETG00000001327	2070	0	0	0	0	0	0	1	0	0	0	0	0	
ENSXETG00000001331	1434	0	1	0	0	0	1	8	12	10	4	3	2	znf804a
ENSXETG00000001335	1144	290	481	389	413	313	504	551	665	365	381	262	265	nup35
ENSXETG00000001336	1876	28	61	58	56	39	62	136	202	75	41	39	45	dusp19
ENSXETG00000001337	3795	1485	2131	1899	3553	2508	5008	4946	6135	3488	5258	3798	4195	nckap1
ENSXETG00000001339	1855	3	12	8	28	19	21	248	306	184	78	76	74	frzb
ENSXETG00000001340	1172	477	829	650	643	481	492	922	949	636	512	341	387	exosc2
ENSXETG00000001343	681	0	0	0	0	1	0	1	2	2	0	0	0	prdm12
ENSXETG00000001344	3600	521	739	619	613	454	516	989	1096	598	515	420	418	fubp3
ENSXETG00000001346	867	0	1	0	1	1	1	0	0	0	0	0	0	
ENSXETG00000001347	1700	2381	3557	3056	1575	1256	1927	3597	3939	2467	214	144	160	ass1
ENSXETG00000001352	5141	1113	1682	1428	1700	1353	2589	1282	1452	863	1990	1385	1538	ssfa2
ENSXETG00000001353	14919	140	231	195	1041	787	419	346	407	232	589	426	473	hmcn2
ENSXETG00000001355	1564	13	21	15	184	135	143	1038	1150	734	319	227	242	ncs1
ENSXETG00000001356	1773	598	906	799	1021	765	1842	2006	2190	1343	1602	1093	1279	gpr107
ENSXETG00000001364	3038	203	290	210	387	302	530	1203	1515	807	428	332	332	stam
ENSXETG00000001366	4341	5	11	13	94	75	189	206	281	160	254	178	223	mrc1
ENSXETG00000001368	2115	0	0	0	0	0	0	0	0	0	0	0	0	
ENSXETG00000001377	756	10	15	10	66	49	109	224	269	154	131	98	117	prkar1a
ENSXETG00000001379	1610	111	178	148	362	265	522	1270	1521	889	639	485	548	pdgfa
ENSXETG00000001382	876	0	0	0	0	0	0	0	1	0	0	0	1	lrrc38
ENSXETG00000001383	657	85	134	100	172	117	46	2	9	7	43	38	48	c1orf158
ENSXETG00000001384	1275	911	1413	1249	611	452	858	75	70	45	119	90	95	aadacl4
ENSXETG00000001388	2225	2238	3258	2872	1611	1171	2697	1217	1516	818	1719	1277	1369	dhrs3
ENSXETG00000001389	1875	61	84	86	136	96	209	144	202	104	174	134	119	fbxl4
ENSXETG00000001390	1320	2	0	1	0	1	0	0	3	1	0	0	0	ENSXETG00000001390
ENSXETG00000001391	1161	145	185	176	290	245	391	264	289	181	446	262	342	vps13d
ENSXETG00000001393	453	0	1	0	0	1	0	1	0	1	2	1	0	ENSXETG00000001393
ENSXETG00000001395	879	18	34	30	61	34	83	219	267	140	138	121	129	fam20c
ENSXETG00000001397	1931	6	8	2	13	10	33	265	272	166	44	43	46	runx2
ENSXETG00000001399	734	672	996	785	446	324	609	609	723	407	310	208	246	mrps16
ENSXETG00000001404	927	69	97	115	110	78	137	340	383	259	190	155	144	supt3h
ENSXETG00000001405	1242	0	0	0	0	0	0	0	0	1	0	0	5	ENSXETG00000001405
ENSXETG00000001407	1755	5	5	3	4	2	5	2	0	2	6	3	1	best3
ENSXETG00000001408	822	0	0	0	1	0	0	0	0	0	4	1	3	
ENSXETG00000001410	1704	2975	4444	3885	3057	2326	3500	8645	10093	5656	2574	1775	2059	cct2
ENSXETG00000001412	2241	159	262	229	290	220	251	2308	2702	1645	619	440	511	gbp4
ENSXETG00000001414	1971	73	119	93	136	104	228	363	474	303	251	150	183	dpy19l1
ENSXETG00000001415	1803	148	223	173	278	224	321	4386	5184	3094	275	190	240	gbp7
ENSXETG00000001416	1215	0	0	0	0	0	0	0	0	0	0	0	0	
ENSXETG00000001420	1161	218	320	252	327	260	470	734	995	586	412	304	346	F2RL1
ENSXETG00000001425	981	0	2	0	0	0	29	3	2	0	48	31	43	ENSXETG00000001425
ENSXETG00000001426	3077	424	595	521	860	649	980	1517	1867	1029	1150	808	889	frs2
ENSXETG00000001428	939	0	0	0	0	0	0	0	0	0	0	0	0	
ENSXETG00000001429	957	0	0	0	0	0	0	0	1	0	0	0	0	
ENSXETG00000001434	2999	441	639	599	527	396	967	1048	1246	738	720	520	559	mdm2
ENSXETG00000001437	748	151	226	166	226	160	198	581	679	422	291	225	258	cdk4
ENSXETG00000001438	1357	35	73	55	82	58	117	220	283	142	147	86	109	cpm
ENSXETG00000001439	1787	287	380	335	589	448	824	1009	1102	771	709	528	594	cpsf6
ENSXETG00000001440	2454	3	0	0	0	0	0	4	8	1	4	0	1	cav3.1
ENSXETG00000001441	1173	4	3	4	16	14	27	44	55	43	33	14	29	oxtr
ENSXETG00000001443	2787	415	569	510	667	594	394	1256	1443	861	518	337	402	nup107
ENSXETG00000001445	3198	12	11	9	38	24	85	219	237	141	105	70	79	rad18
ENSXETG00000001447	2191	660	940	843	609	451	519	609	680	397	664	459	480	thumpd3
ENSXETG00000001448	937	68	95	91	120	102	218	300	347	179	224	173	173	vhl
ENSXETG00000001449	2603	281	366	327	294	229	654	191	194	120	267	184	213	irak2
ENSXETG00000001450	1092	300	519	421	756	560	834	872	975	562	1352	973	1044	dgat1
ENSXETG00000001452	2555	33	46	46	236	194	257	243	279	180	156	100	138	mdm1
ENSXETG00000001453	1778	2920	4283	3620	2838	2144	3372	3398	3716	2199	2258	1532	1786	sec13
ENSXETG00000001455	3702	74	122	118	160	117	287	199	200	128	297	195	249	cand2
ENSXETG00000001457	936	36	74	44	87	56	151	106	100	73	130	96	102	tbc1d20.2
ENSXETG00000001461	495	23007	34670	30562	22137	16048	22036	31326	35035	21931	42734	29977	33090	rpl32
ENSXETG00000001463	3723	11	12	17	254	178	88	292	344	238	81	70	75	ift122
ENSXETG00000001464	3309	84	110	97	274	188	450	680	662	444	396	252	257	abl1
ENSXETG00000001465	1677	0	0	0	0	0	0	2	0	0	0	0	0	
ENSXETG00000001466	889	0	0	0	0	0	0	2	0	0	0	0	0	
ENSXETG00000001467	4562	43	50	61	359	240	568	2211	2491	1428	575	437	455	plxnd1
ENSXETG00000001468	417	0	0	0	0	0	0	0	0	0	0	0	0	
ENSXETG00000001469	1447	0	0	0	0	0	0	2	4	4	1	2	2	
ENSXETG00000001473	1971	102	141	120	308	270	526	1435	1574	1048	743	479	566	tmcc1
ENSXETG00000001474	1324	35	51	44	52	42	50	583	625	397	143	91	121	aif1l
ENSXETG00000001475	6238	384	567	445	1422	1078	1094	1320	1532	934	1375	950	1100	nup214
ENSXETG00000001478	2495	39	40	43	94	66	142	758	835	512	153	98	115	fam78a
ENSXETG00000001479	1878	0	2	1	1	1	30	38	40	26	38	27	24	plpp7
ENSXETG00000001480	3525	388	585	500	1871	1426	2062	3169	3553	2281	3047	2248	2456	prrc2b
ENSXETG00000001481	2691	331	434	411	533	393	588	871	977	619	1093	788	907	pomt1
ENSXETG00000001482	816	467	741	590	973	806	2100	904	930	615	1876	1365	1457	uck1
ENSXETG00000001485	4588	394	600	484	807	653	955	2354	2391	1547	1294	901	970	rapgef1
ENSXETG00000001486	453	58	65	69	116	79	126	218	226	134	132	102	96	med27
ENSXETG00000001494	609	0	0	0	0	0	0	0	0	0	0	0	0	
ENSXETG00000001496	1931	1843	2671	2326	4404	3263	4285	3669	4129	2553	3781	2609	2925	clint1
ENSXETG00000001499	914	57	121	95	158	124	179	184	220	151	125	85	130	lsm11
ENSXETG00000001501	1949	3	1	4	116	91	2	1	2	0	41	47	41	nipal4
ENSXETG00000001502	4564	43	74	59	89	91	141	468	541	330	290	205	237	cyfip2
ENSXETG00000001505	1367	188	313	241	280	216	324	545	669	395	425	303	311	med7
ENSXETG00000001506	405	0	0	0	0	0	0	0	0	0	0	0	0	
ENSXETG00000001509	1600	0	0	0	0	0	0	0	1	0	1	0	0	ENSXETG00000001509
ENSXETG00000001511	1205	536	729	599	1435	1058	2291	1217	1427	873	1915	1397	1519	kiaa1671
ENSXETG00000001512	618	6	11	4	7	5	3	7	11	7	1	2	3	crybb2
ENSXETG00000001517	4644	6	9	9	15	12	21	18	20	16	16	16	20	myo18b
ENSXETG00000001521	1608	216	381	310	482	331	426	680	801	466	651	406	470	tbl1xr1
ENSXETG00000001525	2514	0	0	1	6	3	2	10	14	4	8	0	2	NLGN1

ENSXETG00000001526	2988	41	56	46	125	89	250	754	943	552	672	438	565	smarca1
ENSXETG00000001527	1650	24	27	30	87	61	183	352	399	233	226	156	157	gab3
ENSXETG00000001528	3333	1288	1802	1505	2279	1754	4042	2300	2657	1542	5211	3715	4178	picalm.2
ENSXETG00000001529	1217	925	1275	1042	559	394	535	1265	1567	910	488	351	432	vbp1
ENSXETG00000001531	1858	363	502	440	937	730	1569	980	1207	674	2230	1519	1717	ENSXETG00000001531
ENSXETG00000001533	3118	16	34	19	53	53	75	273	309	177	96	67	64	stox2
ENSXETG00000001534	1317	100	135	120	50	55	65	212	260	160	418	269	295	enpp6
ENSXETG00000001535	1680	7	14	10	53	30	35	57	73	48	29	21	20	primpol
ENSXETG00000001536	2104	266	429	323	1260	1031	2202	1274	1522	878	1839	1257	1403	irf2
ENSXETG00000001537	3300	1027	1454	1096	4482	3452	7444	3345	4304	2361	4757	3388	3830	casp3
ENSXETG00000001539	2814	7670	11455	9672	8311	6509	8192	2100	2456	1360	3325	2265	2585	acsl1
ENSXETG00000001540	1472	26	35	17	35	35	34	77	86	58	32	22	28	c1orf74
ENSXETG00000001541	594	2	7	5	8	1	8	3	2	3	11	10	6	GUCA1B
ENSXETG00000001542	2234	0	1	0	1	0	0	2	2	0	1	0	0	guca1a
ENSXETG00000001547	4932	179	221	221	1588	1216	1778	5801	6293	3852	650	502	518	col4a6
ENSXETG00000001552	1827	0	1	0	0	1	0	1	2	1	2	3	2	lingo2
ENSXETG00000001561	2287	29	46	30	122	89	77	66	97	59	43	37	41	lrp2bp
ENSXETG00000001563	2782	468	693	535	602	482	1802	5752	6781	3960	1760	1215	1322	pdlim3
ENSXETG00000001564	6662	19	18	18	96	74	129	220	256	154	156	106	123	sorbs2
ENSXETG00000001565	5058	506	764	690	4153	3290	4449	12002	13201	8424	1523	1112	1214	col4a5
ENSXETG00000001566	3231	1030	1478	1308	5467	4258	5435	2144	2464	1451	5326	3738	4175	irs4
ENSXETG00000001567	3240	0	0	0	0	0	0	0	0	0	1	0	0	gucy2f
ENSXETG00000001573	960	125	169	140	371	265	602	798	829	540	784	554	585	spry1
ENSXETG00000001582	2040	43	64	47	94	80	233	263	296	148	222	172	188	spata5
ENSXETG00000001584	1203	1755	2592	2235	3262	2478	3891	5763	6476	3936	2362	1658	1764	psmd12
ENSXETG00000001585	903	81	129	127	129	84	200	85	90	67	83	42	76	nudt6
ENSXETG00000001586	1069	11	21	13	40	30	38	294	354	231	87	51	52	XB-GENE-959703
ENSXETG00000001587	4071	4	5	3	33	21	41	181	213	134	97	58	61	fgf2
ENSXETG00000001588	2061	3	2	6	21	14	5	20	19	10	5	7	11	bbs12
ENSXETG00000001591	625	9	14	9	271	220	27	32	34	18	24	11	14	cetn4
ENSXETG00000001592	8775	417	613	548	1729	1348	2681	3074	3338	2056	3588	2450	2831	bptf
ENSXETG00000001593	1923	0	2	1	0	0	0	8	12	5	0	1	2	adad1
ENSXETG00000001594	2315	2015	2988	2525	3483	2624	3770	5864	6686	3987	1642	1196	1215	kpna2
ENSXETG00000001595	843	0	0	0	0	0	0	0	0	0	0	0	0	
ENSXETG00000001596	4711	285	379	335	813	629	1331	2151	2497	1334	1411	995	1135	ENSXETG00000001596
ENSXETG00000001603	1014	0	0	0	0	0	0	0	0	0	0	0	0	
ENSXETG00000001606	1353	0	0	0	0	0	0	0	0	1	0	0	0	
ENSXETG00000001609	6082	213	316	277	551	415	584	770	941	508	786	607	696	smchd1
ENSXETG00000001613	3075	21	38	30	197	180	238	1736	2005	1245	561	361	430	emilin2
ENSXETG00000001616	1758	0	0	0	1	1	5	3	3	2	2	1	2	klhl31
ENSXETG00000001617	2929	911	1337	1123	1216	907	3492	2161	2635	1507	1448	1069	1177	lpin2
ENSXETG00000001618	2349	348	512	417	758	527	1178	692	910	520	810	599	646	lrrc1
ENSXETG00000001619	2607	9	19	17	17	12	11	5	2	10	63	41	49	myom1
ENSXETG00000001621	3006	186	255	192	447	380	521	381	482	254	608	415	496	fam83b
ENSXETG00000001622	1323	27	17	25	43	35	92	12	19	5	139	109	111	hcrtr2
ENSXETG00000001624	6794	0	0	3	19	10	18	129	168	84	120	80	94	map2
ENSXETG00000001625	1494	3	12	6	4	10	25	152	208	102	32	18	19	slc9a9
ENSXETG00000001626	2430	137	216	162	291	207	389	750	819	480	498	338	351	c3orf58
ENSXETG00000001628	2109	15	16	19	118	95	224	811	1045	610	268	209	217	plod2
ENSXETG00000001630	744	3307	4886	4112	5486	4193	6718	1110	1353	827	2745	2017	2216	plscr2
ENSXETG00000001631	606	9	16	6	33	22	29	33	38	41	39	36	28	ENSXETG00000001631
ENSXETG00000001632	1005	6	4	3	172	103	72	29	33	26	194	158	188	rxfp4
ENSXETG00000001633	993	10	11	11	22	32	28	46	44	33	45	31	38	agap3
ENSXETG00000001634	1410	17	27	25	16	9	10	90	100	39	11	9	6	ENSXETG00000001634
ENSXETG00000001636	894	0	1	0	0	0	0	8	7	7	0	0	0	spdyc
ENSXETG00000001637	2493	668	919	808	1331	1004	1553	1645	1844	1065	1110	755	903	vps51
ENSXETG00000001638	1698	403	670	578	918	704	1079	2916	3432	2066	1091	790	816	ehd1
ENSXETG00000001642	885	13	12	9	31	27	86	331	367	216	127	83	99	ENSXETG00000001642
ENSXETG00000001644	2021	903	1442	1251	1623	1249	1201	3206	3525	2175	1779	1312	1445	fkbp4
ENSXETG00000001646	1566	0	0	0	1	0	0	1	0	0	0	0	0	
ENSXETG00000001647	1909	170	273	240	396	242	548	469	498	307	356	237	235	itfg2
ENSXETG00000001648	5019	223	301	263	465	345	675	908	1180	607	827	573	686	dock8
ENSXETG00000001650	2497	0	5	1	18	10	19	97	112	74	36	35	30	ppp2r5b
ENSXETG00000001656	1283	318	473	429	451	343	492	500	579	284	580	432	434	cbwd1.2
ENSXETG00000001660	1518	5	8	3	308	230	579	23	36	6	172	124	145	ENSXETG00000001660
ENSXETG00000001661	860	108	178	145	174	114	248	472	561	332	210	138	154	arl2
ENSXETG00000001662	1594	135	229	197	278	193	450	605	674	415	369	275	284	snx15
ENSXETG00000001666	2414	0	0	1	0	2	0	0	0	0	2	4	2	cbwd1.1
ENSXETG00000001667	1059	0	0	0	0	1	0	0	0	0	0	0	0	
ENSXETG00000001668	1017	0	0	0	0	0	0	0	0	0	0	0	0	
ENSXETG00000001670	1701	29	40	37	243	236	886	2133	2523	1510	838	651	700	pgm5
ENSXETG00000001671	1401	4753	7001	6118	8347	6165	9219	8170	9315	5432	7357	5069	5677	taldo1
ENSXETG00000001672	1358	141	216	162	331	236	371	563	671	415	445	325	358	dpf2
ENSXETG00000001673	1209	3	9	8	33	31	33	27	42	22	54	32	32	CHRM1
ENSXETG00000001676	843	0	0	0	0	0	0	0	0	0	0	0	0	
ENSXETG00000001678	1614	234	306	250	585	425	1044	818	916	505	819	559	679	pip5k1b
ENSXETG00000001682	1597	2306	3464	2923	5971	4563	7677	5116	5665	3591	5311	3659	4044	MGC76064
ENSXETG00000001683	3021	438	698	627	2177	1550	1755	1794	2056	1185	2079	1459	1656	tjp2
ENSXETG00000001688	2152	1	2	6	55	41	101	4485	5638	3232	1077	759	903	mamdc2
ENSXETG00000001690	1093	8088	11934	10273	12256	8956	12045	4473	4842	3096	7886	5706	5913	suclg1
ENSXETG00000001693	948	0	0	0	0	0	0	0	0	0	0	0	0	
ENSXETG00000001695	960	0	0	0	0	0	1	0	0	0	0	1	0	
ENSXETG00000001696	1413	0	0	0	3	0	4	41	48	24	5	6	1	syt6
ENSXETG00000001697	2155	5	13	10	29	36	51	1350	1662	916	307	210	249	olfml3
ENSXETG00000001698	4878	155	215	183	467	361	819	1094	1237	747	1058	765	867	hipk1
ENSXETG00000001699	1541	31	37	25	28	33	73	272	330	190	132	90	96	rab44
ENSXETG00000001701	1435	2252	3169	2773	2317	1775	1408	4010	4596	2961	1413	1016	1053	srsf3
ENSXETG00000001702	1367	40	43	26	83	56	80	144	203	113	55	28	41	adora1
ENSXETG00000001703	1656	87	163	152	444	369	623	747	853	470	333	263	272	neurl1b
ENSXETG00000001704	1260	0	0	0	0	0	0	0	0	0	0	0	0	
ENSXETG00000001710	933	0	0	0	0	0	0	0	0	0	0	0	0	
ENSXETG00000001712	4792	610	862	728	499	433	676	1121	1398	806	901	675	681	pprc1
ENSXETG00000001714	1701	305	472	372	876	658	1128	2148	2377	1498	1537	1126	1234	ldb1
ENSXETG00000001718	1638	2	0	0	31	26	5	47	54	42	7	7	2	ENSXETG00000001718
ENSXETG00000001719	1962	1167	1768	1540	3184	2416	2206	1460	1655	1015	911	714	706	acad9
ENSXETG00000001721	1596	148	247	203	314	222	538	709	736	547	511	348	397	iqsec1
ENSXETG00000001723	711	0	0	0	0	1	1	4	2	1	3	1	3	XB-GENE-5810167
ENSXETG00000001724	1398	770	1210	1024	1615	1182	1967	392	501	294	637	456	505	ankh
ENSXETG00000001725	894	131	163	142	194	121	239	287	340	222	190	105	148	otulin
ENSXETG00000001727	1014	17	27	20	159	115	493	141	178	94	367	299	343	fam105a
ENSXETG00000001728	1872	0	0	0	0	0	0	0	0	0	0	0	0	
ENSXETG00000001729	1461	14	20	16	64	46	88	336	435	214	137	105	113	lpcat1
ENSXETG00000001731	1515	366	549	440	455	329	729	511	592	326	398	262	308	srd5a1
ENSXETG00000001732	2892	886	1227	1114	908	766	781	1309	1547	908	873	624	662	nsun2
ENSXETG00000001734	2234	236	312	230	42	22	92	254	307	186	69	38	48	ube2ql1
ENSXETG00000001738	4177	818	1133	925	1464	1144	1621	1877	2054	1263	2182	1546	1722	nktr
ENSXETG00000001739	939	0	0	0	0	0	0	0	0	0	0	0	0	
ENSXETG00000001740	459	47	87	94	97	57	129	170	179	108	100	73	87	borcs5
ENSXETG00000001742	969	0	0	0	0	0	0	0	0	0	0	0	0	
ENSXETG00000001744	933	0	0	0	0	0	0	0	0	0	0	0	0	
ENSXETG00000001746	933	258	392	303	3725	2855	348	4	3	2	1686	1240	1285	slc39a2

ENSXETG00000001747	1405	115	168	148	149	143	118	226	272	159	155	91	112	mettl17
ENSXETG00000001748	1680	364	476	412	586	456	400	495	566	314	412	312	365	parp2
ENSXETG00000001749	1248	0	0	2	12	9	1	24	30	22	19	10	18	gpr19
ENSXETG00000001751	6297	828	1229	1061	2280	1794	1786	1349	1649	992	2418	1727	1921	tep1
ENSXETG00000001752	1434	2	0	0	1	0	2	4	5	3	10	5	9	tuba8
ENSXETG00000001756	3201	35	73	56	168	121	287	867	919	512	425	306	307	mical3
ENSXETG00000001759	972	0	0	0	0	0	0	1	1	0	0	0	0	
ENSXETG00000001760	2139	235	328	329	433	292	651	925	996	607	557	447	453	ptpn11
ENSXETG00000001762	1218	0	0	0	3	0	1	3	7	4	5	4	0	ENSXETG00000001762
ENSXETG00000001763	5625	193	280	238	1126	863	846	1494	1716	1050	1179	846	979	agrn
ENSXETG00000001764	1044	0	0	0	0	0	0	0	0	2	0	0	0	
ENSXETG00000001765	1420	2	0	0	0	0	0	5	3	4	0	0	1	
ENSXETG00000001766	1215	0	0	0	0	0	0	0	0	0	0	0	1	
ENSXETG00000001769	1342	117	165	119	226	171	796	1520	1533	945	731	513	564	hes4
ENSXETG00000001770	2010	7	9	14	81	60	376	301	337	174	659	422	502	jph2
ENSXETG00000001772	1220	0	1	0	2	2	4	11	9	6	2	2	6	gdap1l1
ENSXETG00000001773	5795	992	1432	1142	670	512	1186	652	757	440	703	450	538	fitm2
ENSXETG00000001774	762	0	0	0	0	0	0	1	1	0	0	0	0	
ENSXETG00000001775	1140	838	1229	1019	2628	1973	3981	1104	1258	738	3224	2366	2618	hnf4a
ENSXETG00000001776	1313	303	475	388	166	136	231	320	379	245	171	99	119	ttpal
ENSXETG00000001777	2010	1041	1522	1408	4653	3575	5332	6148	6914	4295	4756	3303	3639	hnrnpm
ENSXETG00000001778	2048	4624	6428	5621	8244	6413	18016	10512	11581	7028	11123	7670	8639	serinc3
ENSXETG00000001779	3059	856	1326	1104	877	692	783	1193	1424	789	540	390	422	timm44
ENSXETG00000001780	1551	18	19	29	41	62	124	232	275	192	41	23	26	ptgis
ENSXETG00000001781	11088	72	58	87	229	196	515	2657	3125	1933	170	132	142	fbn3
ENSXETG00000001782	927	1	0	0	0	0	0	0	0	0	1	1	1	
ENSXETG00000001783	3388	1563	2312	1892	2758	1985	3800	5657	6502	3822	2393	1674	1840	stau1
ENSXETG00000001784	3588	651	949	750	1565	1193	1350	2844	3183	1925	1253	864	1004	cse1l
ENSXETG00000001785	5061	600	863	741	965	653	1375	1237	1548	946	1163	898	921	arfgef2
ENSXETG00000001791	2162	675	951	816	918	740	1246	1418	1636	958	861	575	636	sppl2b
ENSXETG00000001793	3258	0	0	0	2	0	0	5	9	2	0	0	0	tmprss9
ENSXETG00000001794	1193	1382	1884	1675	976	745	789	999	1153	707	536	372	401	timm13
ENSXETG00000001795	1856	55	66	67	101	76	70	340	409	246	58	50	41	lmnb2
ENSXETG00000001796	1473	3	5	1	4	2	10	5	7	0	8	6	4	lrrc14b
ENSXETG00000001799	774	1	2	7	41	37	16	17	21	7	12	7	8	hoxd4
ENSXETG00000001800	1494	12	17	11	59	54	54	101	105	58	51	24	43	hoxd3
ENSXETG00000001801	1592	0	1	0	0	0	0	0	1	1	0	0	0	hoxd1
ENSXETG00000001802	1039	722	1008	889	616	470	1168	975	1259	691	988	730	731	mtx2
ENSXETG00000001806	1750	4111	5904	5345	13815	10573	14927	15116	17254	10458	11796	8161	9213	hnrnpa3
ENSXETG00000001807	972	0	0	0	1	0	1	7	5	2	0	1	0	ENSXETG00000001807
ENSXETG00000001808	2586	5392	7356	6348	8794	6873	10971	6363	7661	4476	9254	6346	7387	nfe2l2
ENSXETG00000001810	9795	0	1	0	1	2	3	120	129	63	19	21	26	dchs2
ENSXETG00000001811	2631	0	1	0	3	3	4	32	48	22	30	18	21	trpc4
ENSXETG00000001812	2379	64	89	76	263	186	303	1310	1663	984	146	108	146	postn
ENSXETG00000001813	1412	1591	2203	1915	2028	1565	1764	1976	2141	1228	1397	1046	1149	alg5
ENSXETG00000001816	2212	723	1007	888	490	384	654	193	217	115	354	241	301	pex11g
ENSXETG00000001817	2331	26	30	24	65	53	82	479	604	331	91	89	114	tlr2
ENSXETG00000001818	3611	600	841	756	1680	1236	3347	2663	2932	1671	2982	2012	2285	supt20h
ENSXETG00000001819	1061	324	568	491	416	343	402	770	917	600	253	186	203	exosc8
ENSXETG00000001820	3359	92	126	90	340	303	525	915	1055	606	976	659	774	smad9
ENSXETG00000001821	1611	1076	1383	1235	808	579	1025	441	539	284	137	99	116	soat2
ENSXETG00000001822	1233	183	294	240	293	215	367	681	674	455	432	317	343	spryd3
ENSXETG00000001823	5058	16	28	25	185	159	409	476	577	364	422	299	350	tns2
ENSXETG00000001824	2550	99	121	107	227	171	317	574	627	377	317	224	234	rfxap
ENSXETG00000001828	1134	1045	1411	1288	1284	1108	2023	264	347	192	1659	1164	1334	gpat3
ENSXETG00000001829	3134	7480	11130	9732	19820	15471	27014	19510	21944	13550	58594	42124	45824	eif4b
ENSXETG00000001831	2508	292	421	373	356	290	408	432	521	339	509	348	462	helq
ENSXETG00000001833	6581	865	1287	1001	2045	1566	2333	3216	3622	2036	4687	3152	3743	kiaa0922
ENSXETG00000001834	772	381	622	500	747	604	301	979	1056	652	358	244	257	rpa2
ENSXETG00000001835	1632	14	18	12	14	13	58	368	468	262	75	57	63	THEMIS2
ENSXETG00000001836	1579	27	48	28	64	34	39	171	194	155	20	9	18	mnd1
ENSXETG00000001837	3917	1236	1718	1441	1675	1237	2335	822	953	560	1476	1016	1185	trim2
ENSXETG00000001839	3607	62	87	68	77	86	187	506	499	292	189	147	156	fhdc1
ENSXETG00000001840	1389	681	935	833	741	577	966	1341	1530	830	683	507	615	arfip1
ENSXETG00000001845	1026	718	1097	958	480	443	402	704	805	449	385	262	304	mrpl3
ENSXETG00000001846	1740	0	0	0	0	0	0	0	0	0	0	0	0	cpne4
ENSXETG00000001850	6744	737	1068	985	1447	1157	2906	2700	3088	1825	1811	1323	1422	dnajc13
ENSXETG00000001851	774	1	1	0	3	4	7	24	19	14	6	5	7	ENSXETG00000001851
ENSXETG00000001852	715	435	626	578	304	233	292	409	516	288	226	113	151	mrpl32
ENSXETG00000001853	733	3039	4372	3734	3080	2274	2913	6318	7464	4198	1605	1085	1255	psma2
ENSXETG00000001854	4557	380	517	450	579	435	568	883	986	607	640	462	464	topbp1
ENSXETG00000001855	1486	498	700	642	357	277	531	704	837	496	287	221	223	c7orf25
ENSXETG00000001856	4578	6	14	13	77	63	128	699	689	401	135	125	102	gli3
ENSXETG00000001859	1975	2404	3517	2991	1834	1448	2281	3733	4553	2593	1629	1098	1216	cdv3
ENSXETG00000001861	2234	0	2	1	6	5	13	314	360	207	63	36	54	INHBA
ENSXETG00000001863	2412	60	73	66	55	57	97	154	142	80	55	50	41	sfmbt2
ENSXETG00000001865	2823	21	37	35	175	151	325	658	727	442	419	291	335	itih5
ENSXETG00000001866	3216	8	15	15	5	3	1	18	15	10	0	0	1	itih2
ENSXETG00000001868	1115	14844	21551	18479	14385	11100	16285	8814	10942	6206	8748	6047	6911	atp5c1
ENSXETG00000001869	2594	516	634	537	937	696	1248	1980	2196	1236	1364	1010	1138	taf3
ENSXETG00000001873	537	0	0	1	0	0	0	0	1	0	0	0	0	xa-1
ENSXETG00000001875	3229	1019	1452	1295	1831	1338	3905	3885	4558	2789	2736	2008	2153	pcdh1
ENSXETG00000001879	2619	87	129	123	219	186	411	501	593	350	453	348	384	nr3c1
ENSXETG00000001880	4459	246	367	281	677	526	1647	1253	1478	911	2584	1884	2111	arhgap26
ENSXETG00000001882	3650	273	412	329	498	405	763	301	339	211	55	41	62	chst11
ENSXETG00000001883	1770	2713	3793	3293	2518	1929	4145	2025	2566	1446	1131	788	993	slc41a2
ENSXETG00000001884	515	508	684	608	358	286	313	498	571	361	305	174	235	c12orf45
ENSXETG00000001885	1371	173	246	214	235	211	298	497	709	383	340	237	267	actr6
ENSXETG00000001886	2946	232	365	307	511	417	351	474	498	298	352	258	301	aldh1l2
ENSXETG00000001887	3519	321	472	398	661	508	1381	1359	1709	981	1225	803	896	washc4
ENSXETG00000001888	2020	460	634	547	621	491	878	1308	1594	878	1003	741	843	appl2
ENSXETG00000001890	2064	11593	16979	14506	13850	10479	12989	4834	5565	3179	7184	4860	5569	lta4h
ENSXETG00000001891	2926	41	83	58	306	222	501	2106	2468	1413	491	331	440	elk3
ENSXETG00000001892	4340	717	986	776	1194	919	2255	1775	2227	1160	1155	850	995	cdk17
ENSXETG00000001893	2247	5	3	10	20	9	6	5	3	2	0	0	2	cfap54
ENSXETG00000001902	1071	0	0	0	0	0	0	0	0	0	0	0	0	
ENSXETG00000001903	2854	371	554	467	722	598	587	1074	1269	727	549	401	451	tubgcp3
ENSXETG00000001904	2547	0	0	0	0	0	3	3	13	6	4	4	5	ENSXETG00000001904
ENSXETG00000001905	3408	32	50	44	183	172	196	317	382	231	191	119	152	atp11a
ENSXETG00000001907	600	0	1	0	0	0	1	2	4	1	3	0	2	ENSXETG00000001907
ENSXETG00000001908	627	13	16	8	44	29	39	103	131	69	147	82	98	bcl7c
ENSXETG00000001910	2233	121	155	141	293	228	458	664	750	496	392	272	300	znf646
ENSXETG00000001911	1452	104	147	86	347	260	699	376	405	254	825	631	632	znf668
ENSXETG00000001912	718	46	56	48	144	99	181	270	344	208	177	120	118	ENSXETG00000001912
ENSXETG00000001913	3637	2159	3058	2733	1722	1382	7046	1176	1277	794	1555	1043	1127	hsd3b7
ENSXETG00000001920	3453	77	124	90	529	417	447	430	580	292	874	652	691	mcf2l
ENSXETG00000001921	5349	192	247	190	525	387	526	779	848	555	841	571	615	setd1a
ENSXETG00000001922	3323	0	0	1	6	2	1	19	14	10	13	10	14	gria1
ENSXETG00000001923	2046	79	131	98	222	177	553	314	321	232	412	290	308	RNF103
ENSXETG00000001924	2121	795	1134	962	2078	1634	2805	2600	3084	1746	2151	1487	1645	rmnd5a
ENSXETG00000001925	657	1	2	4	1	3	6	11	8	8	12	7	15	ENSXETG00000001925

ENSXETG00000001926	3045	345	540	460	1029	781	948	1053	1118	671	959	700	739	eif2ak3
ENSXETG00000001927	1926	102	139	118	291	181	290	417	420	243	321	253	230	fbxl19
ENSXETG00000001928	672	0	0	0	0	0	0	0	1	0	0	0	0	
ENSXETG00000001929	2259	1	2	3	10	9	12	61	93	63	4	1	1	ENSXETG00000001929
ENSXETG00000001930	948	0	0	0	0	0	0	0	0	0	1	0	0	ENSXETG00000001930
ENSXETG00000001931	1135	375	483	380	220	176	267	464	640	372	157	89	103	pcid2
ENSXETG00000001932	1347	1	2	0	4	6	4	29	44	30	19	9	13	f7
ENSXETG00000001933	2272	2067	2936	2460	7337	5643	7527	8109	9042	5716	6795	4793	5219	xnf7
ENSXETG00000001934	1476	0	0	0	0	0	0	0	0	0	0	0	0	
ENSXETG00000001935	2523	0	0	0	0	0	0	0	0	0	0	0	0	
ENSXETG00000001936	1455	698	1051	944	1568	1179	2921	332	385	211	1934	1394	1565	thnsl2
ENSXETG00000001938	384	54136	78999	69046	51665	39883	15722	2635	2901	1731	8390	5629	6446	fabp1
ENSXETG00000001940	2715	53	71	61	144	117	490	213	238	136	115	88	81	smyd1
ENSXETG00000001943	1907	203	267	243	502	384	875	676	742	485	941	667	734	kat8
ENSXETG00000001947	1194	0	0	0	0	0	0	0	0	0	0	0	0	
ENSXETG00000001949	2343	36	50	46	147	158	329	296	349	197	252	194	221	krcc1
ENSXETG00000001951	1843	2100	3077	2662	5542	4295	3131	8929	10060	6041	4677	3111	3404	fus
ENSXETG00000001952	5322	810	1228	1089	1340	1050	1066	1530	1687	980	1126	814	883	polr1a
ENSXETG00000001956	3294	224	278	281	598	495	734	1334	1534	901	920	656	679	dgkz
ENSXETG00000001957	4061	688	988	856	1432	1210	3455	2016	2499	1354	2159	1492	1665	cul4a
ENSXETG00000001958	1998	8931	12255	10153	10166	7858	40261	18064	23051	13006	15546	10667	12480	lamp1
ENSXETG00000001959	2544	1	0	0	0	1	0	1	4	1	0	0	0	ENSXETG00000001959
ENSXETG00000001963	2592	10	19	20	30	22	226	290	377	182	288	230	266	urgcp
ENSXETG00000001966	1062	0	0	0	0	0	0	0	0	0	0	0	0	
ENSXETG00000001967	927	310	463	378	1108	934	619	17930	18986	11933	820	573	665	mdk
ENSXETG00000001968	3948	77	95	82	31	17	179	644	772	411	278	212	252	ENSXETG00000001968
ENSXETG00000001971	2520	0	0	0	0	0	0	0	0	0	0	0	0	
ENSXETG00000001972	1420	1022	1421	1240	884	650	1307	648	866	534	852	563	660	grtp1
ENSXETG00000001974	993	12	5	7	50	39	41	137	165	94	49	34	46	st3gal5
ENSXETG00000001979	3684	66	83	69	164	146	257	538	613	400	313	214	255	ambra1
ENSXETG00000001980	1131	44	43	40	74	63	142	141	156	92	100	66	78	znf408
ENSXETG00000001981	1044	38	54	54	55	42	120	200	254	114	112	74	89	harbi1
ENSXETG00000001982	1916	6	9	2	1	3	3	16	22	18	4	0	3	f2
ENSXETG00000001983	2622	120	175	113	407	356	883	733	889	528	873	623	726	atg13
ENSXETG00000001985	3051	684	978	757	2018	1554	3192	5176	5979	3577	2890	2128	2377	arhgap1
ENSXETG00000001986	6291	405	540	462	1887	1460	1796	3361	3650	2230	1898	1318	1437	ckap5
ENSXETG00000001990	2589	0	0	0	2	4	1	10	10	11	8	2	0	cdh4
ENSXETG00000001992	674	0	0	0	0	0	0	0	0	0	0	1	0	
ENSXETG00000001994	3300	12	28	19	304	238	356	1069	1237	744	788	522	631	ano1
ENSXETG00000001996	4597	144	233	188	399	287	470	583	632	367	590	398	495	taf4
ENSXETG00000001997	1104	314	517	395	1037	810	1209	1415	1598	945	1173	865	923	lsm14b
ENSXETG00000001998	903	3204	4577	4193	4124	3116	4080	9026	9953	6858	2685	1957	2119	psma7
ENSXETG00000001999	5190	43	43	52	53	43	137	277	267	161	30	21	26	ss18l1
ENSXETG00000002000	3666	0	3	2	12	12	35	49	71	47	83	59	53	amigo1
ENSXETG00000002001	1389	140	222	226	322	249	396	250	258	174	498	314	348	mtg2
ENSXETG00000002003	2183	1015	1433	1138	1422	1080	1753	5231	6334	3702	1248	880	1094	gnai3
ENSXETG00000002004	1182	0	0	0	2	0	0	0	0	0	1	2	1	hrh3
ENSXETG00000002005	1482	89	120	100	189	150	176	166	208	127	398	289	302	gnat2
ENSXETG00000002006	1662	1763	2547	2227	3216	2423	3522	2997	3262	1967	1985	1338	1516	osbpl2
ENSXETG00000002007	1324	1405	2118	1832	2084	1652	1862	3576	3955	2436	1018	737	778	adrm1
ENSXETG00000002008	11344	149	240	223	2708	2090	1964	3258	3624	2329	1687	1195	1348	lama5
ENSXETG00000002009	2400	382	561	488	1064	804	1518	741	871	547	1294	926	1035	ampd2
ENSXETG00000002012	347	24747	37268	31869	28757	21240	33008	31322	34978	22149	65678	46903	49864	rps21
ENSXETG00000002013	2669	175	224	213	258	212	522	816	940	574	315	239	229	cables2
ENSXETG00000002015	2543	0	1	0	26	25	0	15	18	6	3	5	3	rbbp8nl
ENSXETG00000002017	895	8394	11685	9967	8084	6089	11689	5118	6246	3598	6911	4801	5507	gstm1
ENSXETG00000002019	2767	8117	11769	10205	8256	6540	11226	3243	3823	2227	9781	6930	7675	eps8l3
ENSXETG00000002021	3906	0	0	0	1	1	0	1	1	0	0	0	0	ENSXETG00000002021
ENSXETG00000002023	1980	612	854	702	1081	829	1486	1116	1315	773	961	713	803	ahcyl1
ENSXETG00000002025	2178	7	6	5	15	21	13	24	25	18	39	17	29	slc6a17
ENSXETG00000002026	2739	3	3	4	8	3	11	141	169	94	54	51	59	mmp17
ENSXETG00000002027	1635	2	1	0	8	4	5	6	6	5	4	9	8	kcnc4
ENSXETG00000002028	3996	138	181	182	742	529	1301	1486	1678	974	530	380	440	ulk1
ENSXETG00000002029	2511	114	188	162	178	127	271	453	573	326	212	135	177	rbm15
ENSXETG00000002030	1886	126	209	167	81	62	118	207	192	123	195	134	165	pus1
ENSXETG00000002036	1006	4859	7058	5969	7279	5669	7447	3590	4192	2527	5280	3625	4067	dhrs11
ENSXETG00000002038	2316	1168	1586	1328	1076	865	1413	1053	1270	687	1102	742	863	ggnbp2
ENSXETG00000002039	1509	85	100	86	82	62	125	98	134	73	122	82	109	pigw
ENSXETG00000002040	3508	467	664	543	398	329	369	554	696	335	211	124	193	myo19
ENSXETG00000002041	9070	12	30	28	61	47	132	430	456	257	284	189	221	ep400
ENSXETG00000002042	1091	296	450	398	450	326	570	658	729	457	563	416	458	rnf113a
ENSXETG00000002043	1619	531	770	618	561	415	420	741	840	442	655	500	579	noc4l
ENSXETG00000002044	1815	1	0	0	14	2	3	6	7	11	20	13	15	galnt9
ENSXETG00000002045	1416	393	534	459	457	315	683	592	629	345	579	463	465	mina
ENSXETG00000002046	1263	1	1	0	1	2	0	0	0	1	0	0	0	gabrr3
ENSXETG00000002047	1215	1789	2482	2195	1378	987	1231	917	985	714	1010	735	845	cpox
ENSXETG00000002049	1341	30	42	34	50	32	96	193	251	143	161	106	135	c21orf91
ENSXETG00000002052	888	0	1	1	8	2	8	9	12	5	4	6	4	chodl
ENSXETG00000002058	1166	60	97	75	139	115	188	349	400	214	152	100	129	tpra1
ENSXETG00000002059	3253	746	1182	961	1409	1075	198	1979	2314	1310	482	358	452	mcm2
ENSXETG00000002061	3968	440	652	516	1133	853	4296	1977	2340	1326	2310	1577	1803	abtb1
ENSXETG00000002062	4318	134	201	166	550	383	449	1426	1551	908	649	487	556	mgll
ENSXETG00000002064	1487	749	1132	950	1356	1002	894	2452	2830	1630	1049	725	795	ruvbl1
ENSXETG00000002066	1395	182	284	245	315	231	415	437	505	280	189	131	149	eefsec
ENSXETG00000002071	804	295	397	337	308	239	405	446	565	316	200	150	186	slc35e1
ENSXETG00000002072	3787	632	944	834	1455	1130	1666	2080	2414	1458	1455	1030	1117	cherp
ENSXETG00000002073	2252	60	96	67	149	124	134	399	484	254	242	149	163	c19orf44
ENSXETG00000002074	3326	120	174	119	227	176	372	672	802	443	444	338	325	eps15l1
ENSXETG00000002075	2227	55	61	59	555	407	727	559	612	406	1502	1008	1140	klf2
ENSXETG00000002076	1627	1	2	1	11	4	2	4	10	1	28	16	15	gdf3
ENSXETG00000002087	1032	0	3	1	1	4	9	10	13	9	9	7	6	ENSXETG00000002087
ENSXETG00000002095	2630	1439	2017	1780	1121	869	2587	2223	2700	1663	1014	726	767	clcn5
ENSXETG00000002097	1686	0	0	0	1	0	0	2	6	2	0	0	0	ENSXETG00000002097
ENSXETG00000002098	693	9	13	9	16	13	79	124	132	83	72	58	90	ENSXETG00000002098
ENSXETG00000002099	900	0	0	0	0	0	0	0	0	0	0	0	0	
ENSXETG00000002100	1477	112	135	135	190	136	79	290	374	215	37	32	31	mad2l1
ENSXETG00000002101	1896	2	3	7	14	11	25	144	149	105	29	15	29	prdm5
ENSXETG00000002103	2743	3	1	7	15	17	28	136	166	112	118	91	135	ndnf
ENSXETG00000002104	1998	1	1	3	10	16	20	12	11	9	18	19	23	qrfpr
ENSXETG00000002106	2133	3	1	0	1	0	14	2	3	1	15	7	4	cdh10
ENSXETG00000002108	1473	196	269	220	373	290	718	554	657	382	661	477	484	arrdc4
ENSXETG00000002109	588	145	220	182	183	145	267	65	58	40	251	164	178	pgpep1l
ENSXETG00000002112	4077	4	1	1	9	10	30	92	85	53	29	29	34	igf1r
ENSXETG00000002117	2982	801	1004	836	1296	1039	5514	3504	4283	2499	1727	1232	1409	zbed8
ENSXETG00000002119	2174	358	528	458	428	277	514	349	451	261	344	255	248	sepsecs
ENSXETG00000002120	2308	1	1	0	6	1	12	23	25	20	26	6	13	lgi2
ENSXETG00000002121	1503	15	26	20	45	34	81	108	132	72	99	63	66	ccdc149
ENSXETG00000002122	720	9	11	12	88	70	73	71	65	43	217	173	172	sod3
ENSXETG00000002123	2380	1672	2578	2189	3375	2554	4184	4647	5092	3153	5270	3773	4243	dhx15
ENSXETG00000002125	1019	0	1	0	5	7	6	91	108	66	11	16	19	ENSXETG00000002125
ENSXETG00000002126	2308	7505	10323	8470	6205	4596	11791	1414	1622	1014	3843	2789	3150	tmem97

ENSXETG00000002131	1203	9	9	17	13	10	37	46	36	25	27	22	22	rorc
ENSXETG00000002132	1797	0	0	0	0	0	4	2	1	2	3	2	4	lingo4
ENSXETG00000002133	1140	355	614	469	933	747	1409	973	1022	688	1938	1473	1549	creb3l4
ENSXETG00000002134	975	0	0	0	0	0	0	0	0	0	0	0	0	
ENSXETG00000002135	903	0	0	0	0	0	0	0	0	0	0	0	0	
ENSXETG00000002137	1341	0	0	1	7	4	26	608	639	412	185	130	129	scara3
ENSXETG00000002138	525	3	10	10	19	16	89	3114	3531	2123	203	131	147	ENSXETG00000002138
ENSXETG00000002140	3498	0	0	0	0	0	1	3	2	0	1	1	0	
ENSXETG00000002141	8025	48978	69168	59169	52308	39651	37007	5555	6511	3746	20474	14415	16382	zan
ENSXETG00000002143	1287	188	265	197	161	141	209	183	211	97	228	151	172	mb21d2
ENSXETG00000002144	958	3	2	6	13	9	11	15	38	21	30	23	23	fgf12
ENSXETG00000002145	2653	290	380	316	461	320	892	751	971	571	676	480	504	ccdc50
ENSXETG00000002149	857	27	36	20	33	30	61	160	195	97	82	83	62	tpgs2
ENSXETG00000002150	778	252	362	310	645	478	1121	215	227	173	860	569	607	aqp7
ENSXETG00000002151	621	13	8	14	60	35	124	225	222	155	150	103	129	aqp3
ENSXETG00000002153	3414	764	1053	965	1207	916	1167	1260	1493	946	1555	1075	1234	nol6
ENSXETG00000002154	719	57	113	76	113	96	139	283	326	174	126	110	94	ube2r2
ENSXETG00000002157	3552	627	990	783	1464	1167	1964	3466	3713	2434	1920	1401	1444	UBAP2
ENSXETG00000002160	2476	542	758	686	1172	909	1613	2221	2474	1510	1117	787	851	ubap1
ENSXETG00000002161	2316	1915	2833	2459	5011	3857	11092	5237	6153	3551	19237	13620	15078	gnl2
ENSXETG00000002162	2011	2011	2697	2254	2199	1650	4351	3560	4237	2546	2367	1631	1820	nudt2
ENSXETG00000002166	1304	34	43	30	316	229	70	53	55	38	20	16	17	dnali1
ENSXETG00000002167	1326	131	188	166	250	181	214	471	551	298	48	30	37	cdca8
ENSXETG00000002168	6026	0	0	0	0	1	0	1	3	0	2	0	1	epha10
ENSXETG00000002171	1836	46	56	38	361	327	425	897	1013	587	493	392	386	il11ra
ENSXETG00000002173	690	1789	2561	2307	1481	1099	1348	3086	3552	1988	1258	897	953	snrpd2
ENSXETG00000002181	1299	0	0	0	0	0	0	0	0	0	0	0	0	
ENSXETG00000002195	2565	14	22	16	4	2	24	102	145	87	17	22	16	ENSXETG00000002195
ENSXETG00000002201	2598	2	6	7	10	9	11	40	47	25	21	13	18	rab15
ENSXETG00000002203	1632	324	537	422	420	263	231	644	707	424	311	213	224	fntb
ENSXETG00000002204	5234	242	323	311	366	293	545	631	743	477	745	493	576	eif2ak4
ENSXETG00000002206	3100	554	785	621	598	498	1222	702	785	507	748	501	545	bcl11a
ENSXETG00000002208	2626	409	611	526	739	551	1114	1610	1915	1113	1026	700	722	papolg
ENSXETG00000002209	5217	308	421	379	359	266	400	700	801	491	655	476	549	rel
ENSXETG00000002210	1855	175	264	213	186	144	207	211	272	137	229	129	171	pus10
ENSXETG00000002211	1238	13021	17221	14223	18628	14404	7422	11886	13706	8600	15950	11158	12618	srsf5
ENSXETG00000002212	1238	409	544	485	764	556	1391	413	438	277	766	559	599	pex13
ENSXETG00000002215	3534	5	3	2	5	3	11	70	93	37	13	22	20	susd1
ENSXETG00000002216	2751	7	16	13	11	9	22	62	73	35	29	19	27	slc8a3
ENSXETG00000002217	2365	4	9	1	11	10	1	20	35	19	7	7	1	kiaa1841
ENSXETG00000002218	10617	517	717	617	1208	911	1554	1962	2223	1312	2595	1780	2148	usp34
ENSXETG00000002222	1055	259	406	320	342	264	390	699	821	517	360	270	298	med6
ENSXETG00000002224	3347	1212	1596	1369	1435	1081	1234	2010	2573	1324	1533	1104	1221	xpo1
ENSXETG00000002225	2500	399	576	446	864	643	1449	1878	2228	1231	1624	1100	1178	ptbp3
ENSXETG00000002227	1906	3766	5524	4602	5930	4496	6187	10268	11737	7397	5082	3562	4017	cct4
ENSXETG00000002228	1686	3560	5014	4183	4856	3748	4244	2170	2379	1466	1057	709	806	hsdl2
ENSXETG00000002229	4616	52	70	49	82	67	77	209	249	152	126	92	113	kiaa1958
ENSXETG00000002230	2500	297	381	334	516	374	582	1257	1456	808	779	532	627	snx30
ENSXETG00000002231	2731	0	0	0	0	0	0	0	0	1	1	0	0	
ENSXETG00000002232	4548	176	258	210	356	246	353	332	402	222	398	297	341	cep170b
ENSXETG00000002233	2276	10	26	26	109	88	48	100	89	73	54	29	26	fam161a
ENSXETG00000002234	1329	169	220	172	141	101	208	301	358	192	131	94	96	slc46a2
ENSXETG00000002235	756	279	434	330	397	308	746	965	1187	686	374	253	262	commd1
ENSXETG00000002236	2054	171	230	179	258	168	547	341	404	235	435	323	372	mtrr
ENSXETG00000002237	5232	291	438	366	706	569	1508	1459	1827	998	1605	1090	1330	b3gnt2
ENSXETG00000002238	2722	25	39	26	162	114	388	1567	1901	991	563	408	476	akap2
ENSXETG00000002240	432	0	1	2	7	3	7	60	75	25	14	4	19	palm2
ENSXETG00000002243	1931	355	514	383	596	496	697	1118	1343	841	625	475	539	dcaf10
ENSXETG00000002245	4506	1	1	3	10	4	7	37	36	17	27	16	17	frmpd1
ENSXETG00000002247	2412	116	147	135	219	181	230	405	540	296	249	155	185	manba
ENSXETG00000002252	993	7	9	7	18	12	32	44	50	36	39	29	37	ENSXETG00000002252
ENSXETG00000002253	1062	163	268	228	327	252	453	310	355	231	317	199	232	hyi
ENSXETG00000002257	5730	775	1167	968	1839	1359	1566	1766	2038	1203	3407	2354	2666	ptprf
ENSXETG00000002258	4526	53	63	65	165	99	289	449	488	309	253	175	221	mn1
ENSXETG00000002259	899	257	341	261	332	280	405	436	551	316	264	194	227	pitpnb.2
ENSXETG00000002260	3526	1408	2077	1690	1662	1268	1553	1610	1838	1140	855	563	673	tmed1
ENSXETG00000002261	825	0	0	0	0	0	0	0	0	0	0	0	0	
ENSXETG00000002262	5676	57	92	61	91	77	103	166	160	103	243	181	177	ENSXETG00000002262
ENSXETG00000002263	6957	7	4	10	56	36	142	633	773	417	174	141	155	ttc28
ENSXETG00000002264	570	370	573	548	300	191	327	705	821	422	402	308	269	qtrt1
ENSXETG00000002265	1470	0	0	0	0	0	0	1	1	1	0	0	0	ENSXETG00000002265
ENSXETG00000002268	5766	16	23	23	138	126	382	2332	2732	1594	480	334	374	phactr1
ENSXETG00000002270	1594	864	1309	1047	1388	962	1111	1008	1203	695	1362	1041	1132	sirt5
ENSXETG00000002271	1851	432	565	499	1958	1535	4182	2445	2853	1660	2258	1503	1708	ranbp9
ENSXETG00000002272	2142	315	437	344	352	254	700	386	578	285	346	197	272	mcur1
ENSXETG00000002273	2233	0	0	0	96	65	159	4	6	2	34	22	33	ENSXETG00000002273
ENSXETG00000002275	741	0	0	0	0	0	0	4	6	4	3	2	0	rnf182
ENSXETG00000002277	858	0	0	0	0	0	0	0	0	0	0	0	0	
ENSXETG00000002278	5128	822	1170	1020	1016	790	1633	821	933	533	1572	1100	1267	kank1
ENSXETG00000002279	1359	43	66	57	99	99	166	198	257	153	217	139	126	zbtb14
ENSXETG00000002284	2129	25	28	24	98	83	103	853	950	581	158	123	135	p3h4
ENSXETG00000002288	2737	80	100	108	351	261	261	7228	8241	5181	333	245	285	fkbp10
ENSXETG00000002290	771	157	212	197	273	183	191	389	498	255	82	79	99	nt5c3b
ENSXETG00000002292	3700	1113	1660	1505	2747	2173	1392	3360	3702	2288	869	620	726	acly
ENSXETG00000002294	1443	1	4	1	131	110	5	2	6	1	10	6	6	ttc25
ENSXETG00000002295	1529	966	1431	1229	2571	1915	4930	2537	2890	1788	4605	3152	3522	dnajc7
ENSXETG00000002296	573	311	443	361	668	531	1215	981	1081	663	774	569	614	nkiras2
ENSXETG00000002298	1368	0	0	0	1	1	3	2	2	2	2	2	4	znf385c
ENSXETG00000002302	1947	68	87	90	278	195	522	620	759	411	240	155	166	dhx58
ENSXETG00000002303	2526	183	282	217	397	309	571	663	720	463	482	347	391	kat2a
ENSXETG00000002305	1917	1456	2005	1551	1441	1114	1921	2696	3245	1828	1501	1068	1167	rab5a
ENSXETG00000002308	5050	9	12	16	35	39	69	147	188	83	138	104	120	rims1
ENSXETG00000002310	2070	1204	1768	1362	2678	2060	3642	1729	2093	1196	2537	1736	1917	asrgl1
ENSXETG00000002312	4951	93	146	88	80	77	236	339	436	240	256	167	189	ogfrl1
ENSXETG00000002315	1584	17583	25883	22410	22421	16805	20137	13086	14826	9053	12392	8916	9495	ENSXETG00000002315
ENSXETG00000002319	1941	0	0	0	0	1	1	9	8	7	1	1	2	sh3rf2
ENSXETG00000002320	4841	1626	2383	2058	1541	1242	938	1603	2057	1068	966	604	732	lars
ENSXETG00000002322	2958	140	186	180	297	237	278	516	540	327	419	287	334	rbm27
ENSXETG00000002324	4279	508	749	685	1235	912	1267	1609	1827	1069	1447	1033	1215	tcerg1
ENSXETG00000002325	1257	0	1	0	0	0	0	0	0	0	0	0	0	
ENSXETG00000002328	2549	124	216	164	226	182	303	386	463	263	330	238	294	aste1
ENSXETG00000002329	2739	285	371	321	642	495	1028	1411	1788	993	606	441	503	atp2c1
ENSXETG00000002330	4800	341	421	368	690	518	1303	1303	1408	881	615	442	480	PIK3R4
ENSXETG00000002334	855	0	0	0	0	0	0	0	0	0	0	0	0	
ENSXETG00000002336	2000	103	139	116	161	130	276	466	481	291	167	139	164	btbd3
ENSXETG00000002339	3321	6	4	3	39	28	114	225	287	148	146	109	136	znf423
ENSXETG00000002340	3657	135	193	174	226	185	411	422	463	313	256	198	234	jag1
ENSXETG00000002351	990	0	0	0	0	0	0	0	0	0	0	0	0	
ENSXETG00000002359	1821	453	659	544	1006	858	1174	1694	1921	1166	1033	802	919	lsm14a
ENSXETG00000002361	2127	7097	10035	8501	7126	5360	12686	6155	7064	3918	8784	6346	6995	gpi
ENSXETG00000002363	2124	13	12	20	103	78	78	207	233	129	190	142	163	vwa5a.2

ENSXETG00000002364	3818	451	667	577	584	465	847	706	791	471	983	681	738	pigo
ENSXETG00000002370	3000	0	1	1	4	4	18	79	96	41	43	27	33	pcdh11x
ENSXETG00000002371	4170	10	8	7	124	80	43	34	40	27	43	36	40	kif27
ENSXETG00000002374	1884	458	709	561	1256	1010	1836	1971	2328	1219	1218	884	977	uba2
ENSXETG00000002376	2158	445	722	555	1078	795	706	183	233	128	215	167	184	ces2
ENSXETG00000002377	1755	1444	2076	1789	1400	1006	3786	70	83	48	591	396	462	ces3
ENSXETG00000002378	918	0	0	0	6	4	2	1	0	0	3	5	1	ENSXETG00000002378
ENSXETG00000002379	2511	247	322	276	348	263	388	610	782	452	520	406	426	cxorf57
ENSXETG00000002382	3425	943	1395	1166	1003	758	1204	1312	1478	901	817	550	727	TBC1D8B
ENSXETG00000002385	2424	0	0	0	1	0	0	8	14	9	1	0	1	pasd1
ENSXETG00000002387	852	0	0	0	0	0	0	0	0	0	0	1	0	
ENSXETG00000002388	1998	1	1	5	4	3	1	1	0	1	5	7	6	poln
ENSXETG00000002389	2131	0	1	0	0	0	1	0	2	1	1	1	0	nat8l
ENSXETG00000002392	1653	205	260	233	667	491	1443	552	581	392	1041	778	807	nelfa
ENSXETG00000002394	2592	590	870	742	795	620	1371	1335	1536	822	1051	693	818	letm2
ENSXETG00000002396	2866	88	145	116	423	366	728	1046	1215	785	1073	799	965	fgfr3
ENSXETG00000002398	1134	0	0	0	0	0	0	0	0	0	1	0	1	ENSXETG00000002398
ENSXETG00000002403	2166	448	676	564	489	364	543	915	976	611	354	269	294	mogs
ENSXETG00000002406	2295	0	1	0	0	1	0	0	0	0	0	0	0	sim1
ENSXETG00000002407	2436	27	41	40	88	61	117	144	193	129	42	28	31	ascc3
ENSXETG00000002408	1650	720	1070	936	615	412	847	729	901	491	395	301	330	tmem183a
ENSXETG00000002409	1131	303	449	433	588	456	1426	811	919	532	863	576	633	sirt2
ENSXETG00000002410	4106	258	367	308	378	269	377	866	1052	553	274	211	246	gpr161
ENSXETG00000002411	1727	29	31	25	41	43	100	2566	3147	1833	217	156	174	dpt
ENSXETG00000002414	2273	46333	66575	57038	40688	31400	69002	13121	15413	9474	73549	51741	58235	atp1b1
ENSXETG00000002416	1164	221	321	254	508	388	536	709	755	580	591	443	478	ino80b
ENSXETG00000002417	1358	111	146	124	276	191	316	262	312	204	100	75	87	nme7
ENSXETG00000002418	2505	0	0	0	0	0	0	0	0	0	0	0	0	
ENSXETG00000002419	1696	296	511	420	633	500	924	852	979	531	903	661	730	blzf1
ENSXETG00000002421	1567	1	1	1	229	157	13	4	5	5	15	12	10	ccdc181
ENSXETG00000002422	1389	66	80	73	81	48	72	97	152	69	99	73	65	XB-GENE-953257
ENSXETG00000002423	6489	22	42	50	548	434	660	722	915	460	234	151	176	f5
ENSXETG00000002424	1201	1201	1693	1316	1337	1095	1764	2075	2477	1480	1399	950	1142	atg3
ENSXETG00000002426	1242	131	177	171	118	79	132	121	143	100	103	54	74	slc35a5
ENSXETG00000002427	3515	49	63	61	214	149	526	5828	7051	3906	700	526	524	ccdc80
ENSXETG00000002431	7742	88	102	102	258	221	373	516	624	354	280	211	209	prx
ENSXETG00000002437	315	415	611	527	759	533	1188	962	1058	711	979	678	720	vapb
ENSXETG00000002438	1404	317	528	359	234	166	528	308	356	205	629	437	422	apcdd1l
ENSXETG00000002439	929	20	19	24	68	47	129	164	176	121	114	76	83	rab22a
ENSXETG00000002442	3443	523	758	689	915	698	1220	1977	2117	1367	1187	839	882	ppp4r1l
ENSXETG00000002443	2592	0	0	0	0	0	0	0	0	0	0	0	0	
ENSXETG00000002446	867	0	1	0	7	4	3	8	7	9	17	14	17	ENSXETG00000002446
ENSXETG00000002447	1098	35	51	42	107	70	143	212	251	166	180	144	132	vegfc
ENSXETG00000002452	654	0	0	0	0	0	0	0	0	0	1	0	0	
ENSXETG00000002453	3885	2	3	1	7	5	18	40	65	38	12	17	18	wdr17
ENSXETG00000002459	4722	0	3	0	13	10	39	86	117	68	50	37	44	gpm6a
ENSXETG00000002460	1383	0	0	0	0	0	0	0	0	0	0	0	0	
ENSXETG00000002476	4131	184	234	268	390	261	645	989	1209	697	736	500	609	map3k5
ENSXETG00000002479	2034	221	355	291	300	256	622	264	312	211	572	381	352	map7
ENSXETG00000002489	1014	0	0	1	0	1	2	1	2	1	2	2	1	slc35d3
ENSXETG00000002490	2485	131	170	148	285	215	199	494	628	364	135	88	112	dbf4
ENSXETG00000002491	1474	4	12	4	17	12	57	460	521	296	62	40	64	rundc3b
ENSXETG00000002493	3690	42953	62112	54064	162131	124449	359193	28056	32412	19368	323107	226860	254693	abcb1
ENSXETG00000002494	1904	968	1386	1200	1689	1294	2871	720	874	503	1860	1337	1517	crot
ENSXETG00000002496	1086	1668	2466	2175	1687	1338	3333	3417	3940	2435	2310	1673	1771	neu1
ENSXETG00000002497	1833	286	382	303	308	205	339	339	425	257	293	224	240	stk19
ENSXETG00000002498	1472	302	412	374	522	436	527	825	943	579	884	601	645	ddx39b
ENSXETG00000002499	761	764	1098	948	105	56	337	27	36	20	28	21	23	apom
ENSXETG00000002500	3569	1337	1934	1645	3351	2465	3744	3865	4184	2431	3730	2639	2758	bag6
ENSXETG00000002501	6874	1042	1463	1213	3085	2330	3529	4684	4971	2961	4243	2963	3298	prrc2a
ENSXETG00000002503	537	15	20	22	24	14	34	128	131	79	74	52	54	rgs3
ENSXETG00000002504	768	57	72	59	34	20	55	112	133	87	98	97	81	ltb
ENSXETG00000002505	1806	29	54	40	39	31	56	238	252	165	53	34	29	tnf
ENSXETG00000002509	1213	108	145	126	102	103	153	194	218	127	160	118	126	gpank1
ENSXETG00000002510	1872	630	1042	918	1216	937	1777	2159	2191	1398	2103	1485	1640	szrd1
ENSXETG00000002512	3177	2132	3157	2657	3322	2533	2506	2670	3242	1832	1676	1196	1386	atp2a2
ENSXETG00000002514	4287	13	18	10	107	82	78	498	568	303	73	25	59	crb2
ENSXETG00000002515	2643	120	148	136	237	174	391	408	434	245	453	369	338	lrig1
ENSXETG00000002518	1602	24	39	25	53	51	64	96	82	53	85	66	76	kbtbd8
ENSXETG00000002522	1101	3257	4874	4172	7565	5748	7008	1602	1917	1122	3185	2354	2500	suclg2
ENSXETG00000002523	852	17	30	28	25	17	24	38	60	25	28	26	39	wdr25
ENSXETG00000002524	2315	995	1391	1179	1512	1133	1242	1132	1295	722	491	346	368	wars
ENSXETG00000002525	885	6	7	7	60	44	71	20	25	12	60	43	53	slc25a47
ENSXETG00000002526	897	76	101	117	122	90	221	184	166	118	229	158	202	slc25a29
ENSXETG00000002527	2880	495	628	570	717	569	1002	1534	1789	1114	967	741	820	yy1
ENSXETG00000002528	969	4	3	2	2	2	9	4	9	5	2	2	1	degs2
ENSXETG00000002529	2924	105	130	94	138	124	352	808	923	583	584	431	481	evl
ENSXETG00000002532	462	0	1	1	0	0	0	12	6	12	0	5	5	igf1
ENSXETG00000002534	2472	9	13	11	30	24	40	52	60	30	7	5	9	parpbp
ENSXETG00000002535	1641	112	191	134	199	141	218	345	419	224	177	126	156	nup37
ENSXETG00000002536	711	238	367	314	336	246	845	536	639	357	475	339	383	washc3
ENSXETG00000002538	1956	3737	5314	4328	3852	2993	6072	1900	2218	1278	2345	1634	1881	chpt1
ENSXETG00000002540	1213	3246	4600	3780	2339	1723	2332	2450	3019	1671	1503	1054	1180	arl1
ENSXETG00000002542	1875	0	1	3	0	1	6	636	863	478	1172	824	985	slc5a8
ENSXETG00000002548	2307	294	390	365	982	707	631	1019	1150	728	174	130	163	tpx2
ENSXETG00000002549	1242	73	101	84	139	136	165	340	400	268	448	355	351	irf10
ENSXETG00000002551	888	32	55	51	70	53	133	161	175	111	99	78	80	xkr7
ENSXETG00000002553	1650	8	17	11	39	66	53	138	162	101	152	98	96	ccm2l
ENSXETG00000002554	1230	0	2	0	10	6	8	13	8	7	14	8	12	rxfp3
ENSXETG00000002555	1164	1190	1848	1616	2385	1796	2362	2938	3236	2155	2083	1528	1606	csnk2a1
ENSXETG00000002556	1705	1	0	1	12	15	22	25	20	15	13	12	10	slc45a2
ENSXETG00000002557	543	8	11	9	25	18	29	97	90	46	41	23	24	tcf15
ENSXETG00000002559	2051	1651	2428	2069	1608	1244	2085	767	916	473	1329	899	1086	amacr
ENSXETG00000002566	7704	38	76	53	148	127	202	300	379	248	373	286	350	sptbn4
ENSXETG00000002567	624	1	2	2	0	0	0	4	0	1	0	0	1	ENSXETG00000002567
ENSXETG00000002568	1767	10	13	10	53	29	41	520	603	352	88	58	68	numbl
ENSXETG00000002569	1104	872	1316	1146	619	434	525	471	497	341	274	222	231	ephx3
ENSXETG00000002570	7284	88	113	114	551	410	653	1270	1298	859	1136	796	938	notch3
ENSXETG00000002571	1371	2	12	4	31	43	82	155	147	111	47	37	36	sowahc
ENSXETG00000002572	1266	0	0	0	8	2	9	10	13	4	13	6	8	upf3a
ENSXETG00000002573	1104	255	332	256	219	166	249	463	578	326	183	132	154	cdc16
ENSXETG00000002574	1689	303	452	413	246	228	334	353	384	215	209	160	191	adck4
ENSXETG00000002577	1954	29	36	30	74	60	178	170	195	100	83	74	74	fhl2
ENSXETG00000002578	1223	277	455	360	414	281	472	614	717	407	343	246	250	c2orf49
ENSXETG00000002580	2601	1110	1707	1455	1210	851	1387	2017	2483	1511	1535	1123	1269	itpkc
ENSXETG00000002581	2613	196	335	266	269	220	753	554	623	388	470	363	398	tgfbrap1
ENSXETG00000002582	3467	17	18	18	18	12	42	145	176	115	48	17	31	gpr45
ENSXETG00000002583	1155	749	1177	1007	768	603	1180	1091	1184	728	1265	855	967	mrps9
ENSXETG00000002585	852	353	525	429	433	347	314	887	1059	583	498	346	386	snrpa
ENSXETG00000002588	757	14	27	15	10	7	4	24	22	12	20	14	18	mia
ENSXETG00000002589	1754	925	1368	1136	1292	1041	2670	553	692	415	2221	1597	1722	rhou

ENSXETG00000002590	3577	1196	1631	1360	1090	821	1594	1921	2374	1303	1289	957	1106	rab4b
ENSXETG00000002592	605	91	139	116	77	65	183	1579	1758	968	76	50	46	txn
ENSXETG00000002594	588	0	0	0	1	0	0	0	0	1	0	0	0	ENSXETG00000002594
ENSXETG00000002597	1727	1	7	12	24	21	60	300	402	187	60	49	66	lpar1
ENSXETG00000002598	1020	9	11	11	77	55	59	155	165	127	51	31	30	paqr6
ENSXETG00000002600	1365	466	673	583	607	428	1996	967	1069	652	1283	921	1046	egln2
ENSXETG00000002602	1494	0	0	0	0	0	0	0	0	0	0	0	0	
ENSXETG00000002603	942	31	48	46	66	69	15	19	20	23	33	27	22	gas2
ENSXETG00000002604	2841	0	0	1	0	1	3	27	23	18	3	0	3	musk
ENSXETG00000002605	1371	0	0	0	0	0	9	4	4	2	10	2	4	bglap
ENSXETG00000002608	1187	339	447	394	464	361	912	834	964	551	1173	788	903	slc25a44
ENSXETG00000002609	10692	35	45	31	249	191	857	754	912	553	1448	1043	1194	svep1
ENSXETG00000002610	4313	306	409	328	819	646	1263	1480	1615	984	1264	942	998	camsap3
ENSXETG00000002611	2340	349	582	451	737	570	746	1067	1220	747	955	651	755	xab2
ENSXETG00000002616	1797	1342	2010	1734	2783	2182	6201	4129	4547	2640	3209	2181	2296	stxbp2
ENSXETG00000002617	1477	4463	6435	5500	4909	3717	6152	3663	4264	2459	5204	3462	3977	sdhc
ENSXETG00000002619	1401	0	0	0	0	0	0	0	0	0	0	0	0	
ENSXETG00000002620	1146	5453	8026	6952	10488	7986	13778	14815	15814	9956	23804	16633	18098	eif3f
ENSXETG00000002633	2352	0	0	0	0	0	0	0	0	0	0	0	0	
ENSXETG00000002634	1854	0	0	0	0	0	0	0	0	0	0	0	0	
ENSXETG00000002635	4995	26	47	49	528	401	841	2574	2751	1756	535	355	377	col4a2
ENSXETG00000002637	5331	60	59	53	703	530	1207	4556	4924	3135	534	392	469	col4a1
ENSXETG00000002639	4143	374	556	473	1246	852	2503	1571	1814	1076	2744	1945	2149	ENSXETG00000002639
ENSXETG00000002643	4625	284	373	315	600	395	731	986	1138	680	848	596	679	zmym2
ENSXETG00000002644	1533	0	0	0	0	0	0	2	2	0	0	0	0	
ENSXETG00000002645	2116	369	561	465	668	512	1051	1555	1842	1046	821	564	643	cryl1
ENSXETG00000002647	2877	230	333	298	649	521	979	554	588	382	1159	874	900	IRS2
ENSXETG00000002648	5271	5	6	5	26	23	46	29	29	16	13	10	9	myo16
ENSXETG00000002652	1220	409	597	519	450	320	586	541	689	379	413	276	326	abhd13
ENSXETG00000002654	3124	194	270	202	261	208	415	288	410	207	275	198	232	lig4
ENSXETG00000002656	1527	646	827	746	1303	1029	1844	955	1033	582	3389	2516	2646	mtpap
ENSXETG00000002657	1398	293	403	333	278	209	473	906	958	648	406	269	353	map3k8
ENSXETG00000002660	4086	24	33	39	258	224	681	1113	1294	727	411	283	306	JCAD
ENSXETG00000002662	6735	213	345	320	813	606	1709	3001	3351	1959	2588	1814	2058	svil
ENSXETG00000002665	1179	1222	1779	1506	1610	1283	1941	2636	2945	1825	1132	757	903	hars2
ENSXETG00000002668	403	1462	2222	1960	2081	1515	1789	1533	1614	1122	1433	1093	1074	ndufb2
ENSXETG00000002669	2931	133	155	139	206	148	515	753	828	569	1442	1046	1116	kdm7a
ENSXETG00000002672	1164	214	363	282	405	280	568	524	557	388	399	258	280	slc37a3
ENSXETG00000002673	1264	625	896	807	995	818	1654	959	1066	708	613	444	468	rab19
ENSXETG00000002677	1212	0	0	0	0	0	1	3	2	5	1	0	0	XB-GENE-942501
ENSXETG00000002678	1177	34	40	38	130	99	85	148	133	100	219	142	150	ENSXETG00000002678
ENSXETG00000002679	645	25	42	31	36	45	41	153	176	102	125	95	88	fam60a
ENSXETG00000002680	4227	259	346	305	343	281	900	1129	1337	859	579	422	463	dennd5b
ENSXETG00000002681	3789	34	52	38	188	135	293	763	829	475	336	241	298	arap3
ENSXETG00000002682	975	15	27	31	31	43	102	674	914	510	82	48	74	ENSXETG00000002682
ENSXETG00000002686	1807	166	199	215	420	333	606	949	1055	652	525	415	478	atl3
ENSXETG00000002690	1362	70	86	83	169	118	208	183	188	130	130	110	101	ENSXETG00000002690
ENSXETG00000002692	1853	46	66	51	111	105	171	297	304	182	443	287	331	rell2
ENSXETG00000002694	2078	1789	2506	2052	2800	2235	3644	4480	5275	3106	3223	2124	2566	rtn3
ENSXETG00000002695	2667	0	4	2	6	4	9	39	53	22	16	15	6	ENSXETG00000002695
ENSXETG00000002698	2181	26	39	34	89	72	107	323	333	204	187	132	133	fchsd1
ENSXETG00000002699	1458	38	66	72	63	38	63	440	547	325	299	196	225	DUSP10
ENSXETG00000002702	1698	0	1	0	0	3	7	133	144	79	7	6	21	mmp24
ENSXETG00000002703	2766	0	0	0	0	0	0	7	13	8	1	0	0	pcdhgc4
ENSXETG00000002704	3280	777	1132	988	2114	1573	2451	2909	3225	1892	2527	1727	1963	mark2
ENSXETG00000002707	2175	326	513	455	749	584	1293	1227	1431	860	1261	895	940	mtss1.2
ENSXETG00000002708	3070	337	457	368	565	442	700	986	1309	760	627	396	503	hdac3
ENSXETG00000002709	2214	1100	1525	1306	2111	1656	2896	959	1168	662	1226	860	947	bco2
ENSXETG00000002710	3231	26	31	27	93	43	127	985	1123	638	302	188	240	rcor2
ENSXETG00000002711	543	675	1036	932	1757	1311	2653	392	410	254	1384	968	1061	tldc2
ENSXETG00000002712	4254	14	13	9	102	63	237	839	994	573	231	152	182	soga1
ENSXETG00000002713	2116	349	485	419	589	405	649	2155	2482	1375	731	596	569	naa40
ENSXETG00000002714	864	7	3	6	4	0	4	4	6	0	0	0	0	act3
ENSXETG00000002715	4094	401	609	479	1281	995	2233	1574	1944	1151	1549	1129	1300	DIAPH1
ENSXETG00000002717	1962	11	13	11	25	22	59	119	125	69	123	87	87	ENSXETG00000002717
ENSXETG00000002721	1221	4574	6220	5185	4324	3435	7152	2353	2585	1802	4895	3417	3905	slc30a10
ENSXETG00000002724	4592	2161	3063	2713	7947	6268	8589	7064	7861	4801	11449	8126	9062	eprs
ENSXETG00000002725	927	2064	3121	2738	5466	4029	2195	733	861	505	2235	1518	1627	bpnt1
ENSXETG00000002726	3086	922	1296	1124	1758	1386	2722	2176	2413	1508	2171	1657	1744	iars2
ENSXETG00000002728	4047	338	528	417	847	637	1106	1250	1487	914	690	535	489	rab3gap2
ENSXETG00000002733	1011	122	182	169	70	36	243	371	454	281	75	56	56	ENSXETG00000002733
ENSXETG00000002734	1158	25	54	48	392	286	463	1680	1820	1101	825	602	590	hlx
ENSXETG00000002736	1290	0	0	0	0	0	0	0	0	0	0	0	0	
ENSXETG00000002739	2880	0	2	3	6	9	13	32	36	26	16	24	26	pcdhac2
ENSXETG00000002741	1736	192	283	242	309	252	417	237	292	176	524	362	444	nepro
ENSXETG00000002745	7379	340	534	428	536	464	887	1014	1226	734	878	619	689	ercc5
ENSXETG00000002746	1709	603	975	780	1222	985	1967	2439	2833	1726	1682	1193	1282	ppm1b
ENSXETG00000002749	4552	940	1357	1080	2296	1712	4878	4540	5157	3071	2277	1675	1865	tpp2
ENSXETG00000002750	2390	3899	5758	4991	10376	8211	32056	2748	3138	1936	7050	5079	5623	slc3a1
ENSXETG00000002751	2112	40	49	39	88	67	139	106	123	54	235	156	187	prepl
ENSXETG00000002754	1750	58	63	75	109	92	311	231	253	170	193	145	150	camkmt
ENSXETG00000002758	828	0	0	0	2	1	0	2	3	3	3	1	1	six3
ENSXETG00000002760	3063	50	80	78	173	180	197	341	444	261	204	141	138	srbd1
ENSXETG00000002761	435	0	0	0	0	0	0	0	0	0	0	0	1	
ENSXETG00000002765	2304	815	1104	904	1000	779	1372	3460	4006	2489	1464	986	1183	twf2
ENSXETG00000002767	987	246	370	294	298	227	1012	448	507	245	317	225	267	josd2
ENSXETG00000002768	666	1	0	1	1	0	0	781	948	528	176	138	120	ENSXETG00000002768
ENSXETG00000002769	3966	8	17	22	2	3	12	44	68	24	0	1	1	ptx
ENSXETG00000002770	2985	661	916	784	1555	1186	1328	2182	2412	1537	1287	898	993	wbp11
ENSXETG00000002772	969	981	1424	1152	674	531	1662	4898	5803	3154	1433	972	1147	arhgdib
ENSXETG00000002778	756	1010	1533	1372	2192	1679	3031	1878	2017	1382	3063	2191	2244	ENSXETG00000002778
ENSXETG00000002780	7524	14	19	22	100	77	145	252	301	150	359	246	291	clip3
ENSXETG00000002781	888	0	0	0	1	1	0	1	0	0	1	1	0	
ENSXETG00000002782	2254	2942	4097	3753	7802	5945	9773	5177	6058	3866	38267	26665	30092	gltscr2
ENSXETG00000002783	2750	304	483	377	1404	1087	1730	5294	5923	3477	1778	1342	1528	ehd2
ENSXETG00000002784	2103	2621	3938	3342	1168	883	1951	1820	2101	1229	3900	2651	3105	CAPN12
ENSXETG00000002785	1656	0	0	1	4	3	7	69	73	30	12	14	9	slc6a2
ENSXETG00000002787	5164	164	239	203	416	317	667	818	904	569	888	650	708	gltscr1
ENSXETG00000002788	2647	1878	2565	2121	2315	1747	4193	3015	3652	2116	1704	1259	1401	napa
ENSXETG00000002789	1360	144	226	191	179	122	199	290	331	177	236	179	200	kptn
ENSXETG00000002790	2913	9	22	13	45	37	269	37	31	19	45	25	21	slc8a2
ENSXETG00000002794	3077	11	17	13	44	38	86	301	350	219	92	65	93	lpcat2
ENSXETG00000002796	435	342	504	507	325	272	355	408	456	283	222	195	197	ccdc58
ENSXETG00000002801	2598	48	73	47	183	153	582	31285	38234	21450	720	532	616	mmp2
ENSXETG00000002805	1062	204	343	298	383	290	366	343	375	267	362	276	321	qtrt2
ENSXETG00000002807	2390	18	25	25	89	80	154	726	824	527	227	184	188	meis3
ENSXETG00000002816	1881	0	0	2	0	4	1	0	3	1	1	1	2	irx5
ENSXETG00000002818	633	2236	3229	2559	2402	1910	2945	4511	5237	3053	1440	951	1171	psmd8
ENSXETG00000002819	1266	0	3	0	4	3	0	14	20	7	4	3	3	spred3
ENSXETG00000002820	1835	126	186	198	245	178	503	251	276	183	402	269	292	fam98a
ENSXETG00000002821	1743	53	81	74	69	44	93	303	384	202	182	135	140	rasgrp4

ENSXETG00000002822	2403	6	3	3	7	10	15	25	19	18	19	19	11 arhgap11a.2
ENSXETG00000002823	14853	1	0	1	2	3	4	4	2	6	107	59	80 ryr1
ENSXETG00000002831	2925	1071	1521	1311	2583	1987	4035	3190	3638	2112	5201	3641	4152 osbpl9
ENSXETG00000002832	1239	1	0	0	0	1	0	1	2	4	0	0	0
ENSXETG00000002833	5661	827	1090	952	1198	858	1289	1767	2113	1256	1446	1054	1162 eps15
ENSXETG00000002834	1467	0	0	0	0	0	0	0	0	0	0	0	0 ENSXETG00000002834
ENSXETG00000002835	2314	937	1343	1151	512	419	845	552	654	342	868	635	672 ttc39a
ENSXETG00000002836	2334	27	34	39	27	28	69	323	370	229	139	105	96 map4k5
ENSXETG00000002837	1886	255	367	249	296	199	593	1071	1277	668	624	467	453 rnf11
ENSXETG00000002840	2800	658	910	797	1520	1109	2982	2679	3548	2034	3248	2322	2694 tspan15
ENSXETG00000002841	2198	361	454	402	536	394	608	1377	1599	982	412	310	342 faf1
ENSXETG00000002842	1032	63	118	88	169	149	162	171	218	119	294	176	189 alkbh6
ENSXETG00000002843	1081	64234	93471	80645	76165	58709	55307	36129	41397	25851	77625	53847	60513 XB-GENE-992854
ENSXETG00000002846	1344	0	1	0	0	0	0	0	0	0	0	0	0
ENSXETG00000002847	1680	78	125	141	363	243	185	25	39	17	42	42	60 bbof1
ENSXETG00000002849	993	6	4	6	41	21	21	89	104	59	60	45	69 ENSXETG00000002849
ENSXETG00000002852	6556	75	111	79	239	151	309	1026	1137	704	756	498	600 prr12
ENSXETG00000002853	1765	526	735	654	672	539	813	621	724	447	785	539	632 coq6
ENSXETG00000002854	1071	1	1	2	5	3	5	11	18	14	11	7	7 kcnj11
ENSXETG00000002855	759	0	0	0	0	0	0	0	0	0	0	0	0
ENSXETG00000002859	3231	392	579	496	1039	861	1334	2000	2378	1348	1533	1137	1201 scaf1
ENSXETG00000002861	2606	177	251	179	374	259	497	580	672	421	536	389	441 znf410
ENSXETG00000002862	846	0	1	1	2	0	0	3	0	1	2	1	1 ENSXETG00000002862
ENSXETG00000002863	1413	154	231	224	225	185	415	323	398	217	236	162	169 irf3
ENSXETG00000002864	636	0	0	0	0	1	1	1	1	2	1	1	0 ENSXETG00000002864
ENSXETG00000002865	1110	2031	2861	2411	2375	1906	2437	4964	5957	3249	2213	1607	1790 capns2
ENSXETG00000002868	2444	269	405	319	493	366	284	985	1118	649	407	263	259 ric8a
ENSXETG00000002869	2689	150	231	176	535	358	767	654	678	461	1122	830	865 ENSXETG00000002869
ENSXETG00000002871	843	0	0	0	0	0	0	0	0	0	1	2	0
ENSXETG00000002872	501	0	0	0	0	0	0	2	1	0	0	0	0
ENSXETG00000002873	951	2	3	9	10	8	8	38	29	28	14	12	10 tecta.2
ENSXETG00000002874	2133	962	1328	1178	1380	1112	1278	3035	3474	2251	2329	1688	1913 b4galt3
ENSXETG00000002875	3062	0	1	1	2	2	1	13	15	4	1	1	2 dlc
ENSXETG00000002876	2042	42	53	52	5	5	6	1	2	2	2	2	2 igf3
ENSXETG00000002877	2005	4794	6840	6051	4512	3440	2201	9631	11210	6402	2517	1770	2108 prmt1
ENSXETG00000002878	474	845	1278	1043	1011	681	1492	356	382	268	714	502	545 polr2l.2
ENSXETG00000002879	1695	29	51	23	234	161	334	2292	2753	1587	508	326	368 tspan4
ENSXETG00000002881	1020	13	15	13	1	0	1	30	59	29	20	16	14
ENSXETG00000002882	1903	970	1380	1181	573	389	288	950	1050	630	306	200	222 timm50
ENSXETG00000002883	2744	2	3	5	20	11	8	18	30	13	11	6	7 ENSXETG00000002883
ENSXETG00000002884	1152	0	1	0	0	0	0	1	1	1	0	0	1
ENSXETG00000002886	4663	52	69	46	227	153	113	200	254	159	122	62	95 nphp4
ENSXETG00000002887	1221	493	751	669	712	611	1426	1010	1126	672	1193	762	839 akt1s1
ENSXETG00000002888	3829	474	691	646	1247	994	1158	1760	1997	1179	1325	909	1079 supt5h
ENSXETG00000002889	2123	49	62	54	126	66	148	142	165	105	166	101	114 fuz
ENSXETG00000002892	2769	216	366	249	407	310	549	7442	8493	4969	1320	936	1103 napsa
ENSXETG00000002894	1197	18	25	19	17	25	18	67	92	40	24	27	14 kcnab2
ENSXETG00000002899	2691	5211	7535	6129	5089	3969	17778	21059	24159	13784	8484	5928	6671 zfp36
ENSXETG00000002902	1587	624	843	746	1460	1171	1630	1838	1955	1270	1611	1125	1201 paf1
ENSXETG00000002903	2064	412	630	527	735	485	757	1404	1621	1012	987	680	728 samd4b
ENSXETG00000002904	5445	7	6	9	49	42	98	639	773	400	257	158	201 chd5
ENSXETG00000002907	1083	0	0	0	0	0	0	1	3	2	0	0	0
ENSXETG00000002908	1454	0	0	0	0	0	0	0	1	0	0	0	0
ENSXETG00000002909	1041	0	0	0	0	0	0	0	0	0	0	0	0
ENSXETG00000002911	1475	76	109	101	120	89	207	413	461	266	259	166	203 mbip
ENSXETG00000002912	2956	182	288	237	244	205	420	906	1077	595	195	139	149 brms1l
ENSXETG00000002914	467	20948	30866	27530	31554	23321	33978	30060	35899	21826	67084	46856	52220 rpl22
ENSXETG00000002916	1845	11	15	10	5	7	4	29	26	16	16	14	11 ENSXETG00000002916
ENSXETG00000002917	1389	0	0	0	0	0	0	0	1	0	0	0	1
ENSXETG00000002918	1596	62	122	93	179	129	272	285	348	186	188	133	138 icmt
ENSXETG00000002919	1541	370	531	523	547	440	660	847	995	568	699	464	595 eapp
ENSXETG00000002920	6143	179	284	234	604	479	521	646	805	466	520	366	435 znf106
ENSXETG00000002921	906	31	54	53	159	106	248	192	212	114	544	357	381 itpka
ENSXETG00000002931	1758	0	2	2	8	1	0	22	20	7	15	9	10 fzd10
ENSXETG00000002934	3395	1184	1584	1354	1027	762	671	2249	2541	1397	593	405	510 farsb
ENSXETG00000002935	1738	76	137	111	533	402	847	560	639	373	1078	736	825 mogat1
ENSXETG00000002939	3742	266	351	321	917	743	576	1311	1613	886	1261	848	990 scg2
ENSXETG00000002940	954	0	0	0	0	0	0	0	0	0	4	4	1
ENSXETG00000002941	3119	4299	6134	5592	12659	9640	15607	14706	15798	10258	24950	18033	19011 eif3c
ENSXETG00000002942	521	22706	33611	29212	33085	24877	32184	31357	36075	21844	71188	48915	54392 rps15a
ENSXETG00000002943	1104	1112	1555	1290	1294	946	1774	1811	2362	1400	1279	844	962 arl6ip1
ENSXETG00000002944	1965	1066	1496	1269	1125	876	1806	1135	1425	806	1378	1059	1201 ap1s3
ENSXETG00000002945	1197	0	0	0	10	2	0	2	5	1	4	0	2 prodh2
ENSXETG00000002946	1154	510	706	640	576	435	468	547	612	330	422	330	345 coq7
ENSXETG00000002947	855	507	767	632	1107	870	1000	662	754	448	1113	810	917 ENSXETG00000002947
ENSXETG00000002950	429	19	35	33	15	6	6	52	42	28	19	9	19 cabp5
ENSXETG00000002954	2015	275	369	308	790	609	922	1195	1396	767	958	658	736 smg1
ENSXETG00000002955	927	458	648	605	669	540	644	1353	1499	936	587	466	507 polr2d
ENSXETG00000002956	2195	86	147	107	92	72	230	389	498	249	176	129	148 ammecr1l
ENSXETG00000002957	3189	233	363	305	662	488	683	1146	1322	763	704	588	631 sap130
ENSXETG00000002960	5416	512	779	641	1738	1381	1654	2141	2372	1416	1423	1039	1191 wdr33
ENSXETG00000002963	3175	739	1163	983	1155	901	1236	2254	2578	1543	1376	935	1028 iws1
ENSXETG00000002964	1741	1	5	1	32	19	10	24	21	17	95	61	74 proc
ENSXETG00000002965	1209	1	0	0	1	0	0	1	0	0	0	1	0
ENSXETG00000002966	558	4353	6601	5594	3599	2689	2458	4133	4453	2859	1715	1264	1311 ssr2
ENSXETG00000002970	1743	567	833	684	792	632	1896	1418	1735	947	1429	998	1209 pigk
ENSXETG00000002971	1579	0	1	0	0	1	4	76	95	58	10	7	15 st6galnac5
ENSXETG00000002975	432	0	0	1	1	0	1	8	12	12	3	3	3 st6galnac3
ENSXETG00000002976	1280	155	246	213	276	263	380	430	429	246	394	293	297 ing5
ENSXETG00000002978	2562	0	0	0	4	0	1	0	0	0	1	0	1
ENSXETG00000002979	1560	351	503	434	278	245	566	609	751	396	455	269	357 rabggtb
ENSXETG00000002980	2003	329	507	447	597	444	390	822	1029	579	468	357	344 dtymk
ENSXETG00000002981	2027	320	456	387	430	350	920	915	962	540	814	550	612 atg4b
ENSXETG00000002983	1281	4679	6751	5769	7407	5592	7211	3887	4441	2501	5040	3696	4062 acadm
ENSXETG00000002985	3083	483	632	568	902	680	1008	1536	1724	960	823	538	701 ppp6r3
ENSXETG00000002987	2106	115	155	136	717	575	240	194	217	115	646	482	486 slc44a5
ENSXETG00000002989	1683	1044	1458	1279	1279	973	2628	1556	1761	1081	2606	1763	1996 stk25
ENSXETG00000002992	1769	1	0	0	0	0	1	0	0	1	0	0	0
ENSXETG00000002996	941	215	342	277	182	131	201	322	328	181	262	189	180 tyw3
ENSXETG00000002997	1500	888	1198	1059	920	747	1441	510	563	341	524	371	409 cryz
ENSXETG00000002998	1944	0	4	1	168	148	12	1	11	5	31	39	34 erich3
ENSXETG00000003001	1476	1	7	6	5	7	5	85	95	48	17	7	15 notum
ENSXETG00000003002	1461	605	856	707	774	615	863	1063	1237	741	732	526	532 aspscr1
ENSXETG00000003003	2686	204	285	244	447	303	760	623	656	386	851	650	644 lrrc45
ENSXETG00000003004	1737	32217	46157	40200	19825	15275	79593	8254	9769	5923	135208	92684	104677 enpp7
ENSXETG00000003005	1440	119	175	153	307	246	557	680	742	466	725	541	533 cbx2
ENSXETG00000003006	1120	437	629	526	245	183	293	125	103	69	360	235	268 afmid
ENSXETG00000003007	1246	74	95	97	7	10	32	64	73	53	20	10	19 ENSXETG00000003007
ENSXETG00000003010	453	2	2	0	0	0	0	30	28	21	8	0	3 ENSXETG00000003010
ENSXETG00000003014	471	70	89	77	205	146	249	1857	2119	1158	1188	849	930 ENSXETG00000003014
ENSXETG00000003015	345	14	31	21	275	245	41	17	16	9	313	248	287 ENSXETG00000003015

ENSXETG00000003016	1075	240	340	252	802	635	515	3948	4934	2475	2087	1428	1697 dstn
ENSXETG00000003017	1677	2167	2999	2634	2689	2033	4320	687	734	420	1347	969	1058 acoxl
ENSXETG00000003020	948	4	9	7	8	8	41	247	354	171	38	22	36 gpr55
ENSXETG00000003023	942	142	190	141	187	134	214	356	442	234	170	90	112 ENSXETG00000003023
ENSXETG00000003024	930	0	0	0	0	1	0	1	2	2	1	3	2 tmtops.2
ENSXETG00000003028	1878	126	217	192	253	163	70	47	46	26	9	7	7 xrra1
ENSXETG00000003029	1527	158	257	200	547	433	933	633	712	478	902	652	758 rnf169
ENSXETG00000003032	945	9	8	11	16	13	20	55	59	20	39	21	26 st6gal1
ENSXETG00000003033	1187	1	3	0	0	1	0	1	1	5	2	1	0 adipoq
ENSXETG00000003034	1401	50	72	65	74	61	30	74	91	51	35	21	20 gpc5
ENSXETG00000003036	2886	814	1198	1065	1009	708	1012	729	908	522	587	429	455 vars2
ENSXETG00000003037	3050	3	20	8	10	13	28	311	336	190	54	29	35 gpc1
ENSXETG00000003039	3387	0	0	0	0	0	0	0	0	0	0	0	0
ENSXETG00000003040	957	2	2	2	9	6	5	9	20	9	12	7	6 dusp28
ENSXETG00000003043	2418	13	15	5	57	40	72	88	111	61	17	8	16 wdr62
ENSXETG00000003046	1368	16	25	19	35	11	91	111	106	70	96	83	72 fancm
ENSXETG00000003049	750	177	260	250	777	672	716	52	53	41	395	280	272 ENSXETG00000003049
ENSXETG00000003052	804	820	1166	950	1152	891	1152	1588	1844	1076	893	629	743 fkbp3
ENSXETG00000003053	1375	833	1217	1022	2214	1766	2618	2052	2163	1325	4294	2963	3163 nup50
ENSXETG00000003055	2145	1051	1489	1270	1154	873	1685	1353	1642	939	1149	897	902 mvb12a
ENSXETG00000003057	1716	26	38	36	66	39	109	181	222	138	113	88	114 klhl28
ENSXETG00000003060	4188	0	0	0	1	0	0	1	1	0	0	1	0 muc4
ENSXETG00000003062	1324	178	290	253	329	240	996	1460	1657	1124	837	585	630 kiaa0930
ENSXETG00000003063	1173	4	3	2	5	5	12	51	49	32	31	26	16 upk3a
ENSXETG00000003064	3596	0	0	0	0	0	1	5	5	10	1	1	0 smc1b
ENSXETG00000003065	933	40	66	38	107	82	333	73	115	58	253	206	206 c14orf28
ENSXETG00000003066	3178	767	1013	843	954	746	996	1073	1300	756	1591	1091	1262 prpf39.1
ENSXETG00000003068	1172	4	4	5	89	71	11	39	55	47	24	22	19 ribc2
ENSXETG00000003071	1860	22	30	28	156	143	144	595	634	452	496	331	387 fbln1
ENSXETG00000003072	1595	164	228	215	454	333	378	607	686	450	436	269	285 atxn10
ENSXETG00000003073	1579	0	1	0	6	7	1	7	6	4	1	3	3 wnt7b
ENSXETG00000003076	3081	303	475	442	1054	861	1485	1664	1782	1098	1536	1119	1173 tnk2
ENSXETG00000003077	2280	751	1029	846	602	447	395	709	860	520	844	582	669 tfrc
ENSXETG00000003078	987	1	0	0	0	1	0	2	0	2	0	3	0 slc51a
ENSXETG00000003079	1921	5291	7714	6634	3215	2453	3969	3317	3934	2283	2231	1642	1758 pcyt1a
ENSXETG00000003081	804	0	0	0	0	0	0	0	0	0	0	0	0 ENSXETG00000003081
ENSXETG00000003083	1836	237	350	275	429	344	485	592	688	395	452	321	375 e2f4
ENSXETG00000003085	756	11	17	16	20	11	34	100	123	82	29	11	28 lcat
ENSXETG00000003087	1008	343	445	387	1065	821	2483	1514	1583	1048	972	718	815 wipi1
ENSXETG00000003088	2667	1	3	3	268	248	45	1463	1743	992	41	28	38 clca3p
ENSXETG00000003090	1923	48	59	46	111	95	201	218	237	142	149	99	131 odf2l
ENSXETG00000003092	5739	8	18	10	41	39	61	344	372	247	94	73	65 col24a1
ENSXETG00000003094	1968	226	319	273	538	382	1289	2204	2632	1583	788	584	655 cyr61
ENSXETG00000003095	7144	127	200	159	350	260	332	1684	2150	1196	601	403	469 ddah1
ENSXETG00000003096	1854	300	470	371	467	363	662	530	710	340	481	360	424 bcl10
ENSXETG00000003097	1721	469	649	503	518	390	577	930	1063	638	564	417	470 c1orf52
ENSXETG00000003098	879	0	0	0	2	1	4	1	1	1	1	1	2 ENSXETG00000003098
ENSXETG00000003099	1554	0	0	0	1	0	2	6	16	8	3	2	1 ENSXETG00000003099
ENSXETG00000003108	2007	119	162	137	437	350	861	495	488	287	855	681	684 osbpl10
ENSXETG00000003118	303	27	38	27	0	0	0	7	6	6	0	0	0 ENSXETG00000003118
ENSXETG00000003119	1365	542	751	691	1490	1089	2368	1342	1405	923	2354	1745	1802 ccdc6
ENSXETG00000003120	1350	0	0	0	0	0	0	0	1	0	0	0	0 actb
ENSXETG00000003121	12714	167	235	204	768	610	701	564	623	370	1257	854	982 ank3
ENSXETG00000003122	1383	1	0	1	0	0	1	1	1	0	0	0	0 neu4
ENSXETG00000003123	898	380	540	470	1215	889	963	2032	2415	1322	259	173	196 cdk1
ENSXETG00000003124	2239	82	92	76	277	229	449	1428	1598	1051	344	280	246 rhobtb1
ENSXETG00000003125	1371	205	350	258	473	333	532	693	768	421	637	470	500 ubxn7
ENSXETG00000003126	1334	45	41	47	80	61	33	255	274	173	13	19	19 dscc1
ENSXETG00000003127	2009	25	41	31	59	55	60	169	168	108	118	78	102 ikzf2
ENSXETG00000003128	1735	159	232	198	426	287	339	520	555	358	419	244	323 rnf168
ENSXETG00000003129	3180	68	109	86	176	148	66	259	299	149	44	36	41 mtbp
ENSXETG00000003130	3718	194	238	236	418	321	489	1074	1158	701	548	429	446 taf2
ENSXETG00000003133	1703	5	6	4	41	37	78	704	797	472	29	24	27 tmem26
ENSXETG00000003134	3094	427	516	465	4288	3183	4252	15635	17704	10607	5281	3747	4239 enpp2
ENSXETG00000003135	1035	2	2	1	6	2	6	4	7	4	24	13	17 nov
ENSXETG00000003136	1959	3	11	9	11	11	14	41	56	31	555	339	386 ENSXETG00000003136
ENSXETG00000003138	669	74	120	87	42	19	57	197	243	127	108	76	88 ENSXETG00000003138
ENSXETG00000003140	1227	1	0	0	24	9	29	3	5	2	43	19	29 ENSXETG00000003140
ENSXETG00000003141	1734	9	7	10	41	22	67	37	34	17	92	65	83 colec10
ENSXETG00000003142	1044	11	25	15	67	75	129	468	542	304	170	125	147 TNFRSF11B
ENSXETG00000003144	3268	1759	2685	2240	8521	6591	13582	5622	6560	3955	13245	9359	10415 gata6
ENSXETG00000003146	8708	295	380	369	1098	808	1125	1488	1730	1088	1320	993	1160 mib1
ENSXETG00000003147	2037	660	899	773	264	231	92	145	198	92	34	27	26 abhd3
ENSXETG00000003149	6226	6	14	14	9	6	29	253	288	156	60	47	46 greb1l
ENSXETG00000003151	3999	190	308	291	620	515	937	1601	1793	1135	986	681	842 rock1
ENSXETG00000003152	3443	1315	1902	1619	1748	1368	1584	3476	4130	2355	844	622	669 usp14
ENSXETG00000003154	2241	34	30	34	184	112	348	1695	2062	1149	614	429	444 colec12
ENSXETG00000003155	2274	459	673	579	779	567	725	1299	1527	886	659	522	553 thoc1
ENSXETG00000003156	1135	147	212	173	420	321	103	643	713	427	75	57	62 tyms
ENSXETG00000003158	1329	412	645	558	412	388	750	1503	1797	1036	547	324	393 enosf1
ENSXETG00000003160	750	7	12	15	53	48	131	74	68	37	99	79	86 dbp
ENSXETG00000003163	1788	38	79	73	140	98	262	478	536	323	162	120	159 sphk2
ENSXETG00000003164	492	34877	51779	44350	52458	39213	62358	49216	56464	34752	124842	85839	95478 rpl18
ENSXETG00000003165	1241	231	386	366	532	407	761	600	683	381	733	503	543 tfpt
ENSXETG00000003166	4258	576	849	737	1303	1015	1308	2069	2375	1373	1644	1149	1307 cnot3
ENSXETG00000003167	4125	619	860	771	1875	1400	4288	2215	2382	1536	3270	2291	2580 asxl2
ENSXETG00000003168	2462	0	0	0	0	0	0	0	0	0	0	0	0
ENSXETG00000003169	984	0	0	0	0	0	0	0	0	0	0	0	0
ENSXETG00000003171	882	111	167	158	283	231	404	409	450	247	453	315	367 leng1
ENSXETG00000003172	1917	791	1175	1013	1990	1530	5358	3080	3596	2132	5849	3913	4507 tmc4
ENSXETG00000003174	3398	335	527	458	497	391	714	793	934	554	428	359	374 mboat7
ENSXETG00000003175	1314	1	0	2	17	18	40	207	186	115	228	156	180 c1ql1
ENSXETG00000003176	327	919	1407	1230	1802	1297	2339	2237	2451	1669	3243	2289	2424 rab10
ENSXETG00000003177	2949	557	805	729	660	505	699	5404	6475	3636	700	458	582 cd37
ENSXETG00000003179	2174	572	880	733	515	415	454	657	752	453	265	200	241 dcakd
ENSXETG00000003181	2232	1	0	0	2	1	0	9	10	3	2	1	2 ENSXETG00000003181
ENSXETG00000003183	2043	0	0	0	0	0	0	0	0	0	0	0	0
ENSXETG00000003187	2349	0	0	0	0	0	0	0	0	0	0	0	0
ENSXETG00000003191	879	3	11	3	4	1	14	48	57	42	17	12	9 cdk5r1
ENSXETG00000003193	2154	264	366	341	1564	1213	795	940	1046	593	1471	983	1098 eps8l1
ENSXETG00000003194	3024	6415	9429	8207	11213	8547	16290	5198	5824	3433	11258	7895	8942 myo1d
ENSXETG00000003195	2568	0	0	0	0	0	0	0	0	0	0	0	0
ENSXETG00000003197	3417	214	291	262	784	524	775	1121	1327	772	819	592	659 ppp1r12c
ENSXETG00000003199	684	0	1	1	6	8	2	4	5	0	1	1	1 tnnt1
ENSXETG00000003203	1936	4409	6417	5375	8881	6517	6332	5498	5878	3563	3143	2219	2413 hadhb
ENSXETG00000003205	2679	1	0	0	0	0	0	4	7	3	15	7	20
ENSXETG00000003208	801	0	0	0	0	0	0	0	0	0	0	0	0
ENSXETG00000003209	2143	299	443	373	843	598	529	576	691	413	643	500	547 SLC5A6
ENSXETG00000003212	1815	8	14	8	89	72	30	100	142	47	76	37	59 syt5
ENSXETG00000003215	1278	0	0	0	0	0	1	1	2	2	1	0	1 ENSXETG00000003215
ENSXETG00000003217	1965	6	5	6	113	82	15	30	49	34	218	127	133 mylk

ENSXETG00000003219	2532	0	0	0	0	0	0	0	0	0	0	0	0	
ENSXETG00000003220	1749	0	0	0	0	0	0	0	0	0	0	0	0	
ENSXETG00000003221	4420	69	107	77	115	88	243	899	1008	519	363	251	272	fmnl1
ENSXETG00000003222	801	27	19	9	95	59	43	59	46	42	42	35	31	ENSXETG00000003222
ENSXETG00000003223	3002	112	145	152	295	245	269	405	421	248	339	259	233	esyt3
ENSXETG00000003231	2715	0	0	0	0	0	0	0	0	0	1	0	0	
ENSXETG00000003233	929	311	436	393	469	385	563	806	791	582	616	437	454	med21
ENSXETG00000003234	2355	382	553	481	1688	1307	1103	866	968	676	1053	704	782	plcd3
ENSXETG00000003235	2325	0	0	0	0	0	0	0	0	0	0	0	0	ENSXETG00000003235
ENSXETG00000003236	1890	0	0	1	0	0	0	0	0	0	1	2	0	ENSXETG00000003236
ENSXETG00000003237	1476	1233	1669	1469	1801	1405	2468	2830	2984	1961	946	680	703	nmt2
ENSXETG00000003238	2846	319	466	403	655	499	776	1197	1312	805	634	457	492	cdc27
ENSXETG00000003239	2685	139	231	228	663	540	767	963	1064	631	1338	971	1072	ENSXETG00000003239
ENSXETG00000003240	2446	47	60	74	181	130	422	620	636	407	837	560	627	mapt
ENSXETG00000003242	834	0	0	0	0	0	1	2	3	2	0	0	0	
ENSXETG00000003243	1708	3	1	1	23	22	22	13	26	12	70	43	52	crhr1.1
ENSXETG00000003245	3307	551	828	718	1269	998	1481	1931	2137	1303	2110	1538	1688	ddx42
ENSXETG00000003246	2079	1695	2518	2195	2809	2302	3849	3408	3945	2137	2091	1382	1516	ccdc47
ENSXETG00000003247	3102	185	264	212	358	305	551	478	641	368	555	448	475	znf451
ENSXETG00000003248	2629	252	358	311	366	345	752	740	890	571	605	481	524	strada
ENSXETG00000003249	894	230	322	275	255	165	352	364	382	264	217	185	186	taco1
ENSXETG00000003251	2419	408	551	473	311	242	661	3493	3811	2172	667	422	493	limd2
ENSXETG00000003253	2355	9	11	5	2	1	3	101	142	97	6	6	4	pik3r3
ENSXETG00000003254	6170	105	192	177	261	199	342	1165	1257	761	476	351	364	mast2
ENSXETG00000003255	372	7	18	11	10	5	17	10	9	5	11	6	6	ENSXETG00000003255
ENSXETG00000003257	1314	13	16	21	32	18	34	42	49	24	61	36	36	phf21b
ENSXETG00000003259	898	16	23	26	18	17	12	42	54	34	14	16	9	izumo1r
ENSXETG00000003260	1451	798	1099	958	787	568	1708	2195	2538	1500	642	455	565	atp6v0b
ENSXETG00000003264	2163	1271	1853	1550	2962	2297	2222	2331	2626	1638	3204	2204	2414	pacsin2
ENSXETG00000003265	1101	6	15	3	48	33	40	373	468	302	51	25	33	b4galt2
ENSXETG00000003266	2785	6	6	13	17	14	39	392	417	227	29	18	12	vwc2l.2
ENSXETG00000003267	1263	67	109	90	83	56	116	184	229	134	92	56	81	ttll1
ENSXETG00000003268	1902	5	5	13	38	45	23	34	35	17	14	14	17	slc6a9
ENSXETG00000003269	1762	178	258	219	299	226	326	414	378	248	167	131	142	mcat
ENSXETG00000003270	453	812	1193	1073	869	643	1066	948	1059	682	1033	660	745	mrps18a
ENSXETG00000003271	4059	10	29	16	16	16	73	437	519	303	87	59	73	trpm8
ENSXETG00000003272	1098	0	0	0	1	0	1	0	0	0	2	3	0	ENSXETG00000003272
ENSXETG00000003278	1151	2	3	0	6	9	6	47	68	31	36	19	21	efna2
ENSXETG00000003280	2487	302	426	366	769	586	2262	775	839	547	792	531	669	agap1
ENSXETG00000003281	1355	30008	43846	37363	47088	36414	42796	97250	113165	68349	40232	28987	31731	cirbp
ENSXETG00000003282	2633	744	1053	1001	870	664	1776	2392	2621	1640	1300	924	1076	midn
ENSXETG00000003283	589	7388	10795	9178	7177	5449	5093	6569	7439	4241	3499	2567	2657	atp5d
ENSXETG00000003284	2976	15	13	13	22	14	17	164	173	113	18	19	26	cbarp
ENSXETG00000003285	1805	243	390	342	386	314	475	564	630	367	469	339	362	stk11
ENSXETG00000003286	2821	592	898	742	396	303	433	3690	4387	2662	568	439	462	cnn1
ENSXETG00000003287	4047	106	152	135	219	134	371	1562	1665	999	520	399	379	arhgap45
ENSXETG00000003290	1035	0	1	0	0	0	0	1	2	1	1	2	2	gbx2.1
ENSXETG00000003291	1115	1263	1998	1628	1456	1158	2389	2133	2373	1380	1667	1197	1418	polr2e
ENSXETG00000003293	2247	0	0	1	0	0	0	1	0	1	2	0	0	
ENSXETG00000003296	939	28	43	44	146	107	405	1881	2286	1274	321	187	230	ackr3
ENSXETG00000003300	6528	5	14	13	150	112	346	702	861	516	494	304	353	FREM1
ENSXETG00000003301	2195	3448	5018	4154	2498	1941	2245	165	203	112	712	503	547	ttc39b
ENSXETG00000003302	930	155	266	186	207	171	345	773	972	512	284	173	212	snapc3
ENSXETG00000003303	3721	217	350	250	544	414	937	1212	1344	842	1384	1004	1136	psip1
ENSXETG00000003314	2076	0	0	0	1	2	4	1	1	0	19	15	12	ENSXETG00000003314
ENSXETG00000003316	2214	0	0	0	0	0	0	0	0	0	0	0	0	
ENSXETG00000003318	2424	0	0	0	0	0	0	0	0	0	0	0	0	
ENSXETG00000003319	2610	23	17	24	0	0	0	6	8	3	1	1	3	ENSXETG00000003319
ENSXETG00000003320	2553	0	0	0	0	0	0	0	0	0	0	0	0	ENSXETG00000003320
ENSXETG00000003321	414	35577	53107	44998	36839	27107	36618	27876	30736	18720	40935	28723	30756	XB-GENE-5829341
ENSXETG00000003324	2547	2	0	1	1	1	2	1	0	0	1	0	0	ENSXETG00000003324
ENSXETG00000003325	927	0	0	0	0	0	0	0	0	0	0	0	0	
ENSXETG00000003326	957	0	1	4	5	2	9	91	86	50	50	31	39	ENSXETG00000003326
ENSXETG00000003327	462	1	8	4	1225	881	36	2	3	0	4538	3252	3472	ENSXETG00000003327
ENSXETG00000003328	1125	208	288	227	246	200	274	1100	1208	843	193	130	132	selenoi
ENSXETG00000003329	2238	1	6	3	113	79	6	27	16	15	11	7	6	drc1
ENSXETG00000003330	6129	0	0	0	0	0	0	8	3	3	3	2	0	otof
ENSXETG00000003331	1302	294	493	427	830	617	4394	3722	3931	2711	2251	1609	1684	slc35f6
ENSXETG00000003334	1938	0	4	6	7	5	1	1	1	3	4	1	4	ENSXETG00000003334
ENSXETG00000003335	1479	2	5	2	28	23	5	60	96	51	1	1	1	slc7a11
ENSXETG00000003337	641	6300	9092	7616	5487	4025	3636	3577	4005	2350	3049	2281	2329	mgarp
ENSXETG00000003338	1254	98	155	134	356	256	542	831	1011	613	528	342	340	f2r
ENSXETG00000003339	5125	1662	2441	2045	5441	4136	5847	3227	3733	2221	6367	4681	5303	iqgap2
ENSXETG00000003340	2367	61	111	85	465	323	747	1598	1934	1089	499	327	433	f2rl2
ENSXETG00000003345	2030	169	237	184	303	225	403	780	853	558	620	437	450	dtx3
ENSXETG00000003349	2248	44	54	38	260	199	125	148	164	100	42	24	22	poc5
ENSXETG00000003351	1935	224	307	297	727	617	728	1150	1245	842	836	596	638	ptgfrn
ENSXETG00000003353	1755	0	0	0	0	0	0	0	0	0	0	0	0	
ENSXETG00000003356	2197	696	1073	971	3114	2499	3313	4353	4972	2936	4190	2909	3168	ubtf
ENSXETG00000003357	1082	366	499	408	557	407	808	985	1194	725	711	490	521	tmub2
ENSXETG00000003358	483	23	43	35	19	23	49	66	76	45	41	28	31	stmn3
ENSXETG00000003361	1086	0	0	0	2	2	0	0	3	0	8	3	3	asb16
ENSXETG00000003365	3225	8	11	10	156	115	307	287	282	217	419	285	304	HDAC5
ENSXETG00000003366	1922	258	402	358	400	311	161	542	716	370	605	451	525	rab27b
ENSXETG00000003368	3336	0	0	0	1	2	0	0	0	0	0	0	1	ENSXETG00000003368
ENSXETG00000003370	5613	13	9	10	100	56	88	277	318	225	184	118	156	plxna3
ENSXETG00000003371	594	686	994	808	704	521	674	1110	1263	754	573	419	441	lsm12
ENSXETG00000003372	2232	22	26	30	81	78	251	1032	1095	720	317	217	251	ppp1r9b
ENSXETG00000003373	1209	2	1	0	10	7	11	17	15	12	13	4	10	sgca
ENSXETG00000003374	5681	2499	3685	3094	5693	4369	10410	90279	100783	60435	12187	8493	9318	col1a1
ENSXETG00000003375	1727	305	434	364	897	720	1165	1321	1434	874	940	646	740	fam50a
ENSXETG00000003376	1347	72	115	108	210	144	288	1254	1428	867	720	486	569	gdi1
ENSXETG00000003377	1377	1508	2359	1973	2944	2235	5422	4521	5008	3075	2670	1861	2097	atp6ap1.2
ENSXETG00000003378	1406	264	396	338	350	265	502	786	919	536	739	589	621	taz
ENSXETG00000003379	2231	2	0	1	2	0	3	12	13	8	9	2	2	slc25a20
ENSXETG00000003380	1797	363	553	500	482	390	1526	25	48	26	732	540	570	ENSXETG00000003380
ENSXETG00000003381	5559	0	0	4	16	4	8	29	24	32	28	21	15	mpp2
ENSXETG00000003382	630	1610	2416	2038	2116	1679	2563	4267	4653	2778	2273	1642	1750	pyy
ENSXETG00000003384	1476	6	7	3	0	0	85	1	5	3	0	6	4	nags
ENSXETG00000003385	1286	315	508	415	390	280	380	733	865	513	211	118	157	tmem101
ENSXETG00000003386	1187	1012	1402	1281	1019	825	1083	1547	1651	1102	1582	1014	1188	ccdc43
ENSXETG00000003387	4013	74	98	82	118	90	97	247	280	167	160	115	111	dbf4b
ENSXETG00000003388	2687	0	0	1	8	10	7	103	112	74	35	18	26	adam11
ENSXETG00000003389	1219	1	0	1	0	0	2	57	71	39	9	9	14	ttyh1
ENSXETG00000003390	2388	600	939	774	1220	859	1315	1867	2015	1305	4326	3023	3571	leng8
ENSXETG00000003391	1179	0	2	5	30	17	34	242	254	170	66	37	43	gjc1
ENSXETG00000003392	234	0	0	0	0	0	0	12	14	7	7	0	1	higd1b
ENSXETG00000003393	3545	2	1	0	6	3	18	44	31	21	76	67	66	MYBPC2
ENSXETG00000003394	3180	1178	1743	1453	2703	2036	2039	3788	4256	2543	2539	1741	1918	eftud2
ENSXETG00000003395	4088	254	357	290	912	708	1365	1225	1456	898	1151	804	954	tead4
ENSXETG00000003396	1617	2394	3609	3167	3159	2413	4047	4341	4967	2976	2290	1604	1902	ndufv1

ENSXETG00000003398	1326	1386	2038	1684	2152	1677	3031	2894	3211	2023	3295	2296	2430	kras
ENSXETG00000003399	1734	0	0	0	1	0	1	0	0	0	0	0	0	slc17a7
ENSXETG00000003400	669	0	0	0	0	1	2	0	1	2	2	3	1	prl.2
ENSXETG00000003401	894	349	483	402	488	417	478	680	755	462	385	271	314	pih1d1
ENSXETG00000003402	863	17	19	15	77	58	22	74	80	46	15	16	14	ccdc103
ENSXETG00000003403	3362	3807	5506	4562	6689	5117	8118	1917	2019	1293	2612	1905	2061	aldh16a1
ENSXETG00000003405	7119	12	26	24	71	54	400	91	101	68	123	104	121	slit1
ENSXETG00000003406	930	17	43	23	109	63	100	98	112	88	194	152	139	ENSXETG00000003406
ENSXETG00000003407	1584	61	90	66	162	130	198	130	124	78	127	101	113	ENSXETG00000003407
ENSXETG00000003414	2160	6	14	15	21	22	25	49	61	38	10	10	8	ENSXETG00000003414
ENSXETG00000003417	1431	6	15	10	26	26	40	104	104	59	13	6	13	arhgap19
ENSXETG00000003418	1083	2	4	4	1	0	4	2	3	5	0	2	0	ENSXETG00000003418
ENSXETG00000003419	1054	238	331	309	230	200	369	510	559	356	303	181	228	rpp38
ENSXETG00000003420	3935	1118	1674	1435	1249	987	1874	644	801	400	1509	1047	1103	pex1
ENSXETG00000003421	1190	64	87	72	125	89	207	99	128	63	129	75	119	rbm48
ENSXETG00000003423	858	0	0	0	0	1	0	0	0	0	0	0	0	
ENSXETG00000003426	1008	0	0	0	0	0	0	0	0	0	0	0	0	
ENSXETG00000003427	3816	739	925	806	670	511	772	853	1047	624	877	642	715	rhbdd2
ENSXETG00000003430	4027	796	1090	925	1076	810	1035	1423	1616	928	1489	1088	1269	rrp12
ENSXETG00000003432	1401	9261	13490	11413	7846	5985	5690	429	490	314	524	341	451	cyp51a1
ENSXETG00000003433	771	4915	7152	6199	1597	1256	2000	3113	3571	2100	1330	869	934	pgam1
ENSXETG00000003434	1103	521	836	746	396	345	478	821	937	556	340	219	234	exosc1
ENSXETG00000003436	2003	393	587	430	577	469	780	1110	1270	738	683	441	478	zdhhc16
ENSXETG00000003439	3497	537	798	640	995	769	1072	1207	1396	808	986	739	794	mms19
ENSXETG00000003440	12035	1155	1652	1422	2964	2333	4637	3215	3925	2219	7089	5033	5792	akap9
ENSXETG00000003442	1871	6	5	4	1	3	0	2	3	2	2	1	2	ankrd2
ENSXETG00000003443	931	56	94	83	17	22	31	116	140	78	20	14	13	hoga1
ENSXETG00000003446	1311	0	3	1	5	6	7	0	3	2	0	3	4	GP5
ENSXETG00000003447	3654	349	541	457	578	508	1487	1507	1720	1115	892	623	692	atp13a3
ENSXETG00000003448	648	279	454	375	209	178	230	513	668	393	214	143	154	ENSXETG00000003448
ENSXETG00000003449	3648	0	0	1	11	6	78	133	131	81	200	154	159	kcnt1
ENSXETG00000003451	1988	730	1090	952	767	604	892	1399	1557	963	1074	758	886	lsg1
ENSXETG00000003453	951	36	48	39	74	72	215	286	270	171	112	81	93	fam43a
ENSXETG00000003454	1158	82	123	116	116	95	133	366	430	249	183	141	153	xxylt1
ENSXETG00000003455	2469	155	227	197	287	228	591	894	1027	547	427	281	307	acap2
ENSXETG00000003457	1077	14	21	13	35	35	39	53	51	30	84	60	69	ENSXETG00000003457
ENSXETG00000003458	1026	336	561	471	795	617	591	403	458	244	532	364	391	bdh1
ENSXETG00000003459	1071	4433	6436	5529	7648	5881	11115	2535	2982	1766	4367	2909	3298	ada
ENSXETG00000003461	870	0	0	0	0	0	0	0	0	0	0	0	0	ENSXETG00000003461
ENSXETG00000003462	1077	682	1005	851	1692	1302	1846	527	589	364	2388	1730	1819	ada.2
ENSXETG00000003464	3744	299	437	417	951	743	925	1526	1652	948	1186	888	992	dlg1
ENSXETG00000003465	1029	43	51	49	65	48	99	155	196	119	103	74	89	ENSXETG00000003465
ENSXETG00000003466	2289	10	7	10	22	22	51	483	548	313	65	57	58	cdk14
ENSXETG00000003467	3203	283	392	355	769	593	883	1234	1418	839	1168	762	895	arhgap27
ENSXETG00000003468	2949	198	271	230	421	344	1736	1029	1247	747	859	621	648	PLEKHM1
ENSXETG00000003469	837	175	238	203	274	191	413	308	311	208	249	219	230	tp53rk
ENSXETG00000003470	2089	299	397	348	441	323	517	1496	1861	942	679	544	588	pttg1ip.2
ENSXETG00000003471	2661	696	1034	903	717	482	1165	813	1044	577	1562	1159	1301	slc2a10
ENSXETG00000003472	1538	1	0	0	8	10	0	2	0	0	85	88	96	f9
ENSXETG00000003474	2596	0	0	2	2	3	1	57	58	43	19	6	20	eya2
ENSXETG00000003477	2836	4	2	4	11	5	16	78	111	65	77	68	59	fgf13
ENSXETG00000003478	960	0	0	0	1	2	2	24	31	12	15	14	15	gpr101
ENSXETG00000003479	1950	982	1280	1005	671	537	1492	521	711	405	561	371	394	ENSXETG00000003479
ENSXETG00000003480	4348	161	219	204	497	410	1081	2236	2491	1548	1267	912	1041	ncoa3
ENSXETG00000003481	1424	728	1120	964	1026	839	1354	2828	3330	2057	1301	912	1075	rbmx
ENSXETG00000003483	4122	943	1492	1163	2071	1667	2650	3182	3623	2190	2699	1935	2208	kdm4a
ENSXETG00000003484	2631	957	1393	1233	1165	868	1877	1933	2139	1307	618	435	485	sulf2
ENSXETG00000003486	2946	7	19	23	25	25	83	355	461	251	94	65	83	st3gal3
ENSXETG00000003489	697	0	1	0	9	6	6	9	15	12	16	6	14	artn
ENSXETG00000003491	2865	123	195	161	247	209	271	524	634	356	233	170	200	ipo13
ENSXETG00000003492	876	0	0	0	0	0	0	3	5	0	0	1	1	
ENSXETG00000003493	1458	96	136	122	65	46	85	143	135	101	166	105	117	dph2
ENSXETG00000003495	1940	49	67	54	96	82	199	227	245	128	132	91	125	ipp
ENSXETG00000003496	1284	601	823	730	1230	927	1324	1301	1487	838	1616	1125	1232	gpbp1l1
ENSXETG00000003498	1975	576	933	798	1951	1487	975	2459	2875	1653	1165	823	920	nasp
ENSXETG00000003499	1448	1171	1568	1306	1523	1153	2622	2062	2443	1408	1424	1120	1104	akr1a1
ENSXETG00000003501	921	45952	65413	54699	30675	23436	49535	14598	17852	10593	28957	19926	22990	prdx1
ENSXETG00000003502	1269	170	222	162	282	195	265	285	350	191	476	308	357	mmachc
ENSXETG00000003504	2571	625	929	820	1257	875	1825	1759	1977	1211	1196	900	925	hectd3
ENSXETG00000003506	1517	470	776	622	634	481	542	627	760	411	470	307	392	urod
ENSXETG00000003507	879	0	0	0	0	0	0	0	0	0	0	0	0	
ENSXETG00000003508	1455	0	1	0	5	4	8	35	35	26	27	12	9	kcnf1
ENSXETG00000003512	1879	8116	12028	10397	6425	4787	2915	6325	7282	4395	1631	1180	1295	pdia6
ENSXETG00000003513	4399	218	315	232	354	276	469	566	694	378	543	395	422	sh2d4b
ENSXETG00000003514	3356	438	565	480	788	634	1276	2354	3047	1673	920	706	786	tspan14
ENSXETG00000003516	1146	0	0	0	0	0	0	3	2	3	4	2	4	
ENSXETG00000003517	7187	237	408	302	1425	1007	1625	1190	1416	779	1232	869	983	zswim5
ENSXETG00000003518	2073	1116	1485	1308	1324	1032	1772	1890	2055	1186	1788	1215	1392	nol10
ENSXETG00000003519	1965	8497	12550	10503	5348	4181	7498	9997	11791	6933	2946	2107	2338	odc1
ENSXETG00000003521	1448	209	340	272	401	308	607	935	1029	655	586	458	436	hpcal1
ENSXETG00000003522	1153	3	5	4	9	8	14	86	120	69	5	0	1	XB-GENE-5872749
ENSXETG00000003527	1960	138	179	149	681	504	215	806	1007	604	106	69	70	rrm2.1
ENSXETG00000003528	2312	31	61	43	24	24	19	92	109	60	25	12	9	hpdl
ENSXETG00000003529	1982	1881	2635	2213	2912	2326	3805	1179	1510	882	2046	1524	1607	fam213a
ENSXETG00000003530	1439	1	4	4	57	40	76	112	137	78	105	85	93	mat1a
ENSXETG00000003532	2940	0	2	1	5	3	8	2	11	5	12	4	7	c6.2
ENSXETG00000003533	4189	84	116	96	261	175	199	303	308	175	326	240	261	mutyh
ENSXETG00000003534	2011	290	429	371	320	245	339	442	587	342	431	332	331	toe1
ENSXETG00000003535	903	1	10	7	5	2	13	38	48	22	7	4	7	tnip3
ENSXETG00000003537	867	3	5	10	24	12	21	33	33	21	30	23	24	plcxd3
ENSXETG00000003539	510	1	2	3	63	41	22	31	28	14	25	9	13	dydc1
ENSXETG00000003541	1767	0	0	0	0	0	2	5	4	5	0	0	0	sftpd
ENSXETG00000003542	732	0	0	0	1	0	0	1	1	0	0	1	0	ENSXETG00000003542
ENSXETG00000003544	1968	0	0	0	5	0	0	1	0	0	0	0	0	bfsp1
ENSXETG00000003545	2003	4	18	10	121	62	28	41	68	35	116	66	95	pcsk2
ENSXETG00000003548	399	0	0	0	0	0	0	0	1	0	0	1	1	
ENSXETG00000003550	1219	801	1106	947	748	554	699	1428	1653	944	567	347	419	snrpb2
ENSXETG00000003551	3969	214	313	282	318	244	830	816	943	562	532	373	431	kif16b
ENSXETG00000003552	3402	5	6	9	230	190	24	64	71	39	10	11	14	ropn1l
ENSXETG00000003553	2703	532	836	613	830	651	1396	850	1021	595	1087	780	854	march6
ENSXETG00000003560	4488	171	243	233	723	530	2054	109	157	103	374	269	299	znfx1.2
ENSXETG00000003561	1098	54	76	63	102	68	193	1520	1764	1040	286	199	206	irf8
ENSXETG00000003563	1659	293	407	333	424	316	478	105	104	54	317	202	226	mthfsd
ENSXETG00000003565	2432	72	99	94	523	462	789	5098	5673	3509	1312	914	1016	foxf1
ENSXETG00000003569	1892	4792	7078	5913	9200	7006	11324	6968	8568	4699	11827	8015	9543	kars
ENSXETG00000003570	285	161	304	230	458	307	796	751	911	494	783	600	666	cxcl12
ENSXETG00000003571	1639	127	227	194	424	286	1125	293	343	192	1639	1105	1216	adat1
ENSXETG00000003573	845	1906	2711	2261	2858	2261	6354	1523	1772	1081	2947	1990	2279	gabarapl2
ENSXETG00000003575	1633	25	38	41	124	95	239	122	140	68	91	63	69	tmem231
ENSXETG00000003576	1179	9	14	11	30	20	38	53	69	35	689	498	543	chst6
ENSXETG00000003578	3786	2352	3451	2782	3650	2905	9419	2531	2966	1715	3326	2370	2725	plcg2

ENSXETG00000003579	990	290	420	353	133	111	210	295	363	214	79	64	77	hsd17b2
ENSXETG00000003580	1173	328	396	325	171	139	293	135	179	103	95	63	76	sdr42e1
ENSXETG00000003583	1352	156	247	205	85	70	123	244	303	192	192	138	137	wdr89
ENSXETG00000003584	1404	310	469	377	501	381	685	1214	1418	817	649	407	530	ppp2r5e
ENSXETG00000003585	1088	5	5	4	22	35	84	371	389	227	81	56	56	rhoj
ENSXETG00000003588	3024	164	228	216	620	439	763	370	404	270	870	611	701	auts2
ENSXETG00000003591	747	94	119	88	178	148	216	461	508	279	313	212	211	fam168a
ENSXETG00000003598	1230	4	12	6	38	33	62	92	117	68	48	29	31	angpt1
ENSXETG00000003600	1606	2	4	0	25	13	24	69	74	43	77	47	57	rspo2
ENSXETG00000003602	1377	6609	9532	8379	10905	8442	17064	14237	16377	9949	35303	24930	28609	eif3e
ENSXETG00000003603	9324	32	56	61	185	133	711	4652	5535	3300	1977	1355	1631	col12a1
ENSXETG00000003604	2105	974	1360	1151	1153	863	1493	2170	2527	1552	917	574	677	emc2
ENSXETG00000003607	1375	681	871	712	940	718	1211	1600	1956	1088	882	557	693	tmem30a
ENSXETG00000003608	3992	309	457	425	2012	1546	1555	1088	1303	716	2792	1870	2215	filip1
ENSXETG00000003609	3664	235	429	364	796	564	960	1065	1328	759	841	619	697	senp6
ENSXETG00000003610	3812	3447	4974	4409	5795	4420	6184	2763	3247	1834	3611	2520	2798	myo6
ENSXETG00000003611	2642	0	0	0	0	0	0	0	0	0	2	1	2	
ENSXETG00000003613	786	0	0	0	0	0	0	0	0	0	0	0	0	
ENSXETG00000003614	1162	4	4	7	5	6	7	38	29	25	20	8	14	prune2
ENSXETG00000003615	2103	0	0	0	1	0	1	10	11	2	6	4	3	
ENSXETG00000003616	10710	129	169	148	410	312	570	628	728	472	1080	758	885	ENSXETG00000003616
ENSXETG00000003617	4371	68	108	79	311	269	171	270	353	197	144	86	89	fancd2
ENSXETG00000003618	3738	3	1	1	18	14	49	514	584	388	262	197	232	atp2b4
ENSXETG00000003619	3539	462	637	507	764	573	1112	1068	1245	731	1180	890	993	gna14
ENSXETG00000003621	1806	84	108	117	180	129	198	247	303	202	275	185	192	gnaq
ENSXETG00000003622	5399	106	151	119	280	230	405	345	329	203	158	110	126	cep78
ENSXETG00000003623	1783	3564	5174	4389	3912	3019	7519	1350	1629	942	1004	843	922	psat1
ENSXETG00000003625	2452	0	0	0	2	0	1	7	11	6	9	3	9	sorcs3
ENSXETG00000003627	5749	13	21	16	271	200	26	172	192	118	46	20	28	wdr90
ENSXETG00000003628	2901	1381	1986	1712	1479	1110	2648	2026	2161	1324	2123	1440	1511	rhot2
ENSXETG00000003629	2707	54	69	54	143	104	69	183	212	157	27	6	14	ddx11
ENSXETG00000003630	1333	176	238	211	439	349	1308	1007	1140	765	602	458	466	washc1
ENSXETG00000003631	2895	0	1	1	1	1	2	12	13	6	7	5	4	iqsec3
ENSXETG00000003633	1821	0	0	0	0	0	0	0	0	0	0	0	1	slc6a1
ENSXETG00000003634	759	0	0	0	1	0	0	1	1	0	0	0	0	
ENSXETG00000003636	2242	4464	6511	5585	4471	3436	7737	2314	2605	1570	2467	1809	2002	rcan1
ENSXETG00000003637	396	4	2	4	19	18	75	272	372	206	104	78	105	kcne1
ENSXETG00000003641	1542	0	4	4	6	3	5	6	12	6	1	1	4	slc6a13
ENSXETG00000003642	4935	343	446	439	1361	992	1194	1560	1722	1089	1168	796	838	kdm5a
ENSXETG00000003647	1609	613	877	734	1408	1102	1926	2643	2865	1780	1470	1054	1212	wasf2
ENSXETG00000003648	5524	473	702	533	601	452	1012	600	838	460	1200	862	937	ptpn3
ENSXETG00000003649	1856	54	87	74	238	199	214	201	243	149	180	119	142	ccdc77
ENSXETG00000003653	2733	5	4	3	13	11	29	14	23	13	23	29	23	ldb3
ENSXETG00000003655	2058	35	49	45	107	113	155	186	239	139	243	149	181	bmpr1a
ENSXETG00000003657	965	0	3	1	6	7	13	156	153	79	87	64	58	sncg
ENSXETG00000003659	1203	0	0	0	0	1	1	0	0	1	0	0	0	ENSXETG00000003659
ENSXETG00000003663	3849	43	82	61	706	520	1660	1445	1674	946	332	209	243	pcdh10
ENSXETG00000003664	1195	172	292	255	289	240	400	543	609	389	500	324	365	nap1l4
ENSXETG00000003669	5028	44	52	41	40	26	163	369	438	272	135	89	122	dock11
ENSXETG00000003671	5369	1604	1980	1813	3409	2725	6283	2808	3400	2011	3350	2362	2827	wdr44
ENSXETG00000003672	3059	141	194	167	237	183	437	633	781	409	370	259	344	klhl13
ENSXETG00000003675	1668	0	0	0	0	0	0	1	0	0	0	0	0	
ENSXETG00000003677	3928	2559	3501	2953	4504	3475	2475	5095	6214	3424	2328	1595	1837	pls3
ENSXETG00000003678	816	0	0	0	8	5	1	5	8	5	0	0	0	ENSXETG00000003678
ENSXETG00000003680	1035	134	236	176	253	204	351	432	473	268	195	142	134	mfap3
ENSXETG00000003682	539	0	0	0	0	0	0	0	0	0	0	0	0	myl3
ENSXETG00000003683	1452	64	81	66	124	97	138	72	86	36	253	186	180	pth1r
ENSXETG00000003685	378	3	2	1	1	1	4	8	9	7	3	4	2	tmie
ENSXETG00000003686	2890	183	259	211	781	597	1135	370	443	257	1145	858	900	als2cl
ENSXETG00000003687	1206	17	14	14	24	22	27	39	67	34	59	26	50	jmjd4
ENSXETG00000003688	984	22	21	22	43	26	54	62	60	36	48	29	47	snap47
ENSXETG00000003689	1457	5122	6898	5549	3269	2447	3469	3043	3850	2126	1541	1145	1191	XB-GENE-5796437
ENSXETG00000003690	1470	3	7	3	1	0	16	14	12	3	10	6	9	rhd
ENSXETG00000003691	2767	342	485	445	439	334	880	998	1226	718	572	413	508	tmem57
ENSXETG00000003692	1842	0	0	0	0	2	1	2	4	2	1	0	3	man1c1
ENSXETG00000003693	1454	75	118	101	256	193	263	399	464	295	212	160	166	ldlrap1
ENSXETG00000003694	1545	22	29	32	52	39	59	180	195	123	80	45	45	mtfr1l
ENSXETG00000003696	2556	292	353	289	263	223	879	856	1084	618	437	315	379	luzp1
ENSXETG00000003698	3058	196	312	221	535	437	404	1186	1375	810	588	455	476	kdm1a
ENSXETG00000003702	824	3620	5320	4718	3698	2770	3956	2688	3004	1857	3198	2233	2391	ndufs7
ENSXETG00000003703	935	30892	44920	39082	23620	18399	34263	808	866	536	5788	4037	4565	gamt
ENSXETG00000003704	1548	1173	1764	1483	1609	1174	1694	2061	2348	1454	1451	1077	1092	dazap1
ENSXETG00000003706	497	51634	75814	62450	63164	47659	60012	55039	63154	37428	113567	80324	88922	rps15
ENSXETG00000003707	6669	6	20	9	38	35	73	195	219	125	129	78	86	apc2
ENSXETG00000003708	1140	4347	5999	5140	3501	2545	6094	1351	1710	1000	2728	2034	2274	reep6
ENSXETG00000003709	2360	5	10	3	4	3	7	5	0	1	6	4	7	pcsk4
ENSXETG00000003711	1335	0	1	0	0	1	4	12	8	6	5	3	4	ADAMTSL5
ENSXETG00000003712	1854	6	5	5	11	11	10	38	38	25	11	8	7	ENSXETG00000003712
ENSXETG00000003713	2602	1255	1866	1498	1960	1533	2385	1410	1731	1013	1462	987	1172	thop1
ENSXETG00000003714	1709	1689	2485	2115	1533	1164	2019	2104	2473	1377	1325	1004	1063	sgta
ENSXETG00000003715	948	57	71	60	38	32	90	70	94	57	52	33	43	slc39a3
ENSXETG00000003716	1165	0	0	0	0	0	2	0	1	2	1	3	0	diras1
ENSXETG00000003717	477	13	11	14	16	9	30	56	72	40	20	20	26	gadd45b
ENSXETG00000003718	4172	533	838	709	1029	746	1890	1479	1873	1040	1321	865	937	gak
ENSXETG00000003719	1626	242	399	343	423	295	452	830	989	531	516	377	438	cwf19l1
ENSXETG00000003720	2946	88	108	105	70	52	106	125	157	99	85	53	74	pigg
ENSXETG00000003721	1566	1	0	2	0	0	0	1	1	1	0	0	2	ENSXETG00000003721
ENSXETG00000003723	2922	16	26	13	129	90	137	136	221	110	157	111	127	ahi1
ENSXETG00000003725	3483	107	140	105	472	375	214	282	335	199	416	285	329	myb
ENSXETG00000003726	3196	741	1168	1017	1548	1216	2141	1652	1978	1197	2527	1658	1965	hbs1l
ENSXETG00000003727	1464	810	1240	1068	732	557	926	90	98	55	266	225	242	aldh8a1
ENSXETG00000003728	912	51	83	69	71	53	136	87	105	70	33	16	24	slc35g1
ENSXETG00000003729	2583	2	5	2	21	10	38	185	214	142	76	50	66	lgi1
ENSXETG00000003730	2556	1	1	1	0	1	0	0	2	0	0	0	0	pde6c
ENSXETG00000003731	1239	43	58	63	44	38	34	31	30	26	1	0	2	rbp4
ENSXETG00000003732	1080	2	5	4	7	0	6	28	37	22	9	9	5	ffar4
ENSXETG00000003733	693	37	49	37	108	82	77	168	187	100	47	40	40	ENSXETG00000003733
ENSXETG00000003734	6320	62	85	67	404	325	799	3559	4180	2470	1178	836	935	myof
ENSXETG00000003735	2940	0	1	0	0	1	3	3	4	2	3	3	6	pcdh8l
ENSXETG00000003736	3297	181	293	245	721	495	1784	3632	4625	2548	832	602	727	entpd1
ENSXETG00000003737	1479	4	3	3	124	97	43	93	112	53	126	79	101	cpn1
ENSXETG00000003739	4617	163	239	237	629	488	795	817	861	556	826	588	710	dnmbp
ENSXETG00000003740	4641	4412	6483	5650	6544	5111	8886	5841	6829	4109	2399	1656	1923	abcc2
ENSXETG00000003741	6129	55	63	50	70	55	52	195	207	121	92	52	50	dscaml1
ENSXETG00000003742	4275	43	78	64	274	201	196	469	552	305	240	144	170	CEP164
ENSXETG00000003744	1467	283	395	382	243	176	413	265	307	170	423	298	371	cutc
ENSXETG00000003745	1242	515	819	676	513	435	425	692	874	553	786	527	565	cox15
ENSXETG00000003746	3371	1116	1598	1350	569	423	1362	1240	1565	939	461	325	385	entpd7
ENSXETG00000003749	2877	7	11	10	38	13	53	161	178	130	79	59	62	bace1
ENSXETG00000003750	1766	164	200	199	336	238	362	491	553	353	461	344	372	rnf214
ENSXETG00000003753	2668	616	881	738	1038	820	1453	1798	2076	1205	1326	943	1009	orai2

ENSXETG00000003755	2271	631	921	758	1077	822	1360	902	958	586	860	612	678	pcsk7
ENSXETG00000003756	1552	2324	3561	2939	3236	2461	7577	1786	2104	1163	2876	2031	2253	tmem120b
ENSXETG00000003757	1462	11954	17026	14133	16395	13256	49021	65162	76422	43217	59467	42701	47003	tagln
ENSXETG00000003758	1318	188	308	262	367	299	446	1661	2063	1140	163	100	131	rhof
ENSXETG00000003759	2502	868	1237	1035	947	707	1216	1295	1572	930	1172	873	989	sidt2
ENSXETG00000003760	6156	56	63	60	146	111	266	303	321	216	338	286	281	setd1b
ENSXETG00000003761	690	127	199	145	200	155	261	367	517	245	277	167	213	pafah1b2
ENSXETG00000003762	3573	92	137	110	264	202	489	663	756	467	537	384	402	sik3
ENSXETG00000003763	1118	1419026	2083457	1822207	740731	565039	1220222	279670	319150	184333	241249	171714	186858	apoa1
ENSXETG00000003764	1107	81623	117265	100664	31579	24349	64996	14890	17370	9822	15014	10432	11905	apoa4
ENSXETG00000003765	2313	398	544	508	467	403	461	874	1033	624	616	395	566	zpr1
ENSXETG00000003766	1834	1	6	0	14	7	1	1	3	2	312	208	222	hpd
ENSXETG00000003767	2459	386	475	380	429	331	503	690	808	485	519	327	364	bud13
ENSXETG00000003768	914	484	737	646	504	390	549	548	619	336	431	299	328	psmd9
ENSXETG00000003772	1600	145	224	167	194	151	177	634	721	399	162	136	136	bcl7a
ENSXETG00000003773	3381	64	120	92	219	172	362	275	308	187	428	273	349	mlxip
ENSXETG00000003775	1294	1819	2534	1960	1738	1233	2771	1637	2085	1111	1290	980	1092	tmbim4
ENSXETG00000003777	1080	5	5	6	36	23	13	32	54	26	108	98	78	b3gnt4
ENSXETG00000003778	1177	452	626	536	629	445	826	1031	1220	665	643	450	522	diablo
ENSXETG00000003780	4374	592	981	803	1525	1235	3213	1388	1730	1036	1771	1353	1432	clip1
ENSXETG00000003782	3094	605	854	697	821	620	737	928	1074	645	1231	877	929	nat10
ENSXETG00000003783	3173	37	68	66	105	82	128	79	97	60	304	174	200	abtb2
ENSXETG00000003784	2558	217	347	316	389	312	397	648	737	416	556	419	481	tfip11
ENSXETG00000003785	2421	236	369	280	280	211	533	606	686	436	470	370	361	fbxo3
ENSXETG00000003786	726	0	0	0	0	0	0	0	0	0	0	0	0	
ENSXETG00000003788	921	1	1	1	1	0	2	6	9	4	5	2	2	guca1b
ENSXETG00000003793	726	0	0	0	0	0	0	0	1	1	0	1	0	
ENSXETG00000003796	4919	0	0	0	37	45	7	57	57	25	60	53	47	shank2
ENSXETG00000003797	3012	3452	5114	4241	6605	5096	8311	8217	8995	5758	6788	4875	5297	cttn
ENSXETG00000003798	3840	438	580	498	1049	834	1867	1335	1497	908	1600	1087	1233	ppfia1
ENSXETG00000003799	1287	397	557	483	505	373	583	1100	1263	712	605	419	518	fadd
ENSXETG00000003800	928	14	24	31	51	47	53	153	170	113	129	81	88	ccnd1
ENSXETG00000003805	1224	12	25	30	39	19	57	119	144	97	32	26	37	ENSXETG00000003805
ENSXETG00000003807	1272	36	56	41	186	109	318	250	276	191	165	103	138	XB-GENE-996645
ENSXETG00000003810	822	48	68	59	170	170	182	131	151	103	387	252	332	ENSXETG00000003810
ENSXETG00000003811	363	0	0	0	0	0	0	0	0	0	0	0	0	pc.2
ENSXETG00000003815	1291	45	81	62	106	85	184	173	224	167	236	134	176	dedd
ENSXETG00000003816	1176	0	0	0	0	0	1	0	0	0	0	0	0	ENSXETG00000003816
ENSXETG00000003817	6328	528	791	698	1987	1681	1685	1840	1919	1240	1886	1400	1455	znf605
ENSXETG00000003818	1626	1	2	1	2	2	0	3	8	1	10	2	2	klhl10
ENSXETG00000003825	989	3	3	2	125	76	8	44	61	15	3	7	4	nme5
ENSXETG00000003826	1486	0	0	0	0	0	0	1	1	2	0	0	0	
ENSXETG00000003827	2688	20	33	24	54	45	80	250	387	201	132	115	93	fam13b
ENSXETG00000003828	2664	6	14	17	16	13	17	81	102	60	26	13	13	gfpt2
ENSXETG00000003829	3411	12	12	5	42	22	101	614	866	475	146	105	142	adamts2
ENSXETG00000003831	1380	330	501	392	470	383	861	644	835	484	2019	1357	1562	clk1
ENSXETG00000003833	1251	2059	2965	2396	4125	3004	3319	6734	7403	5135	3218	2300	2480	hnrnpa0
ENSXETG00000003836	915	4043	6062	5258	7360	5623	5911	15932	17942	10901	5075	3568	4042	hnrnpab
ENSXETG00000003837	849	493	729	623	1011	791	989	1454	1551	991	879	594	620	pura
ENSXETG00000003838	3195	583	864	701	945	680	1213	1599	2079	1189	1073	846	898	matr3
ENSXETG00000003841	699	0	0	0	0	0	1	0	0	0	1	1	0	
ENSXETG00000003846	1773	37	59	46	10	13	55	35	55	36	46	25	27	chrnb2
ENSXETG00000003847	2047	0	0	0	5	6	9	26	29	24	14	14	22	lrrc4c
ENSXETG00000003848	3300	90	142	116	233	221	278	416	383	239	380	272	315	adar
ENSXETG00000003854	1758	0	0	0	1	1	0	0	3	3	7	4	4	klhl34
ENSXETG00000003857	1059	27	52	44	80	65	50	229	246	143	69	45	49	sms
ENSXETG00000003863	933	1	0	0	0	0	0	0	0	0	0	0	0	
ENSXETG00000003868	438	0	0	0	0	0	0	0	0	0	0	0	1	
ENSXETG00000003869	1847	121	185	153	712	566	1131	206	255	159	515	357	422	nr1d2
ENSXETG00000003870	785	58774	84368	69565	74535	57437	80598	70315	76836	46766	156021	107859	118024	rpl15
ENSXETG00000003871	2637	12	11	10	36	31	77	1084	1288	752	105	72	84	thrb
ENSXETG00000003874	2703	178	280	230	664	542	727	1478	1879	1071	164	144	138	tacc3
ENSXETG00000003875	3398	690	966	853	362	223	781	814	927	542	604	409	484	tmem129
ENSXETG00000003876	3770	1065	1521	1275	1883	1421	1207	2432	2905	1710	1232	785	899	slbp
ENSXETG00000003878	1726	169	244	205	494	345	745	1349	1439	884	694	485	550	fam53a
ENSXETG00000003880	1206	0	0	0	1	1	0	0	0	0	0	0	0	
ENSXETG00000003881	1188	0	0	0	0	0	0	0	0	0	0	0	0	
ENSXETG00000003885	2071	3365	4695	4104	2338	1803	4992	407	537	279	1585	1047	1262	cyp4f2
ENSXETG00000003889	2391	1503	2344	1916	2486	1826	4516	3817	4523	2593	2116	1419	1663	vps35
ENSXETG00000003891	1131	299	485	384	409	395	942	558	602	329	402	259	313	orc6
ENSXETG00000003892	2316	0	0	0	1	0	1	0	0	0	3	3	3	mylk3
ENSXETG00000003894	1012	205	292	243	403	284	739	337	358	223	706	478	582	c16orf87
ENSXETG00000003895	1533	31	57	50	132	102	147	298	348	213	167	100	129	gpt
ENSXETG00000003896	1455	8	4	7	6	4	11	38	76	29	25	14	17	crtam
ENSXETG00000003899	3033	60	94	98	1103	853	674	1066	1296	696	2223	1576	1846	grhl3
ENSXETG00000003900	3147	167	255	220	339	271	472	865	1019	593	485	329	388	ubash3b
ENSXETG00000003906	744	0	0	0	0	0	0	0	1	0	0	0	0	
ENSXETG00000003907	2294	1088	1401	1183	1320	1011	2023	1166	1550	812	949	679	782	nipal3
ENSXETG00000003908	1602	6	9	7	13	6	28	159	195	81	20	11	8	mcoln2
ENSXETG00000003909	1998	48	99	68	52	38	112	45	67	38	52	36	31	tmem229b
ENSXETG00000003911	1774	0	0	1	2	4	4	6	9	6	10	11	12	neto2
ENSXETG00000003912	828	39552	58475	50008	43963	32772	48294	45801	52110	31677	95584	66917	74791	rpl27a
ENSXETG00000003913	1607	22	36	34	162	116	95	102	134	49	107	89	83	ENSXETG00000003913
ENSXETG00000003914	1625	129	206	180	293	289	394	553	639	414	294	264	290	zbtb8b
ENSXETG00000003916	2391	584	790	680	543	409	1023	1586	1837	1079	490	361	368	csf3r
ENSXETG00000003918	3255	86	131	129	300	209	478	496	513	317	418	312	330	phkb
ENSXETG00000003920	1953	1002	1399	1222	707	618	939	1127	1258	741	684	543	584	srpra
ENSXETG00000003921	723	0	0	0	0	0	0	0	0	0	0	0	0	
ENSXETG00000003924	1787	131	185	167	90	61	111	363	393	265	83	76	79	fam118b
ENSXETG00000003925	3465	2765	3880	3311	7831	6177	8654	5988	6764	3978	8123	5714	6371	thrap3
ENSXETG00000003926	1632	2	4	2	1	0	1	0	0	0	0	0	0	nxpe1
ENSXETG00000003928	1377	24	16	16	17	8	16	3	7	3	3	0	3	ENSXETG00000003928
ENSXETG00000003933	1617	87	137	100	28	23	91	25	30	23	22	8	19	PDE9A
ENSXETG00000003934	1755	21	29	30	292	182	260	894	1123	618	1817	1272	1385	slc37a2
ENSXETG00000003936	1254	0	0	0	2	0	2	4	4	1	1	0	2	hepacam
ENSXETG00000003937	2715	26	36	21	85	90	187	392	450	248	132	97	116	robo4
ENSXETG00000003938	4095	9	14	14	62	42	91	179	205	122	65	50	44	robo3
ENSXETG00000003941	2733	582	813	697	1439	1029	2801	2841	3095	2025	1731	1176	1317	fkbp15
ENSXETG00000003943	2160	265	441	394	679	529	1011	1062	1084	663	1039	726	771	lrsam1
ENSXETG00000003944	802	33184	48577	42564	49365	37631	59633	52492	57185	38197	177310	122937	135911	rpl12
ENSXETG00000003945	3739	4032	5953	5262	10125	7820	15060	1793	1788	1152	12978	9304	10285	mamdc4
ENSXETG00000003946	369	214	317	240	303	203	302	481	533	365	273	170	205	phpt1
ENSXETG00000003947	3051	2	0	1	7	1	1	5	16	6	3	7	9	c9orf172
ENSXETG00000003948	2260	320	438	393	937	734	863	1509	1646	1079	927	649	758	rabl6
ENSXETG00000003949	330	157	247	203	164	142	171	179	190	121	125	108	99	tmem141
ENSXETG00000003950	1491	318	431	373	469	366	511	1047	1072	787	659	464	539	traf2
ENSXETG00000003951	1576	296	396	365	648	503	831	587	662	430	766	576	528	fbxw5
ENSXETG00000003952	345	56	44	67	246	170	201	443	441	298	345	249	274	nrarp
ENSXETG00000003954	459	40	62	48	217	122	280	161	167	113	605	447	477	ptgds
ENSXETG00000003957	3754	70	102	74	220	174	258	269	288	179	367	246	256	exd3
ENSXETG00000003959	1441	761	1108	905	1883	1412	1676	2358	2511	1529	2051	1430	1548	noxa1

ENSXETG00000003961	1353	105	183	158	677	532	698	911	996	638	895	631	701	entpd8
ENSXETG00000003966	987	1	1	0	0	0	7	25	31	20	41	18	23	ENSXETG00000003966
ENSXETG00000003968	408	1147	1706	1566	1276	910	1541	1395	1462	1013	1169	869	867	mrpl41
ENSXETG00000003970	657	86	126	105	43	38	92	157	220	152	101	80	71	zmynd19
ENSXETG00000003971	917	1597	2409	1948	3030	2394	4841	3526	3649	2427	4303	2927	3181	edf1
ENSXETG00000003972	1505	571	848	732	618	485	688	494	605	386	614	439	513	coq4
ENSXETG00000003973	1258	310	440	365	311	224	249	363	427	226	218	166	168	trub2
ENSXETG00000003974	1387	1	1	1	12	3	1	13	21	6	1	2	2	clic3
ENSXETG00000003976	663	15	24	18	17	15	24	312	355	206	34	21	22	crp.1
ENSXETG00000003978	7170	162	188	180	305	265	392	958	1036	729	1591	1153	1195	abca2
ENSXETG00000003979	1461	296	430	393	530	413	749	1178	1257	773	466	384	390	acp2
ENSXETG00000003980	2436	101	134	116	276	225	500	566	639	413	577	387	463	ddb2
ENSXETG00000003981	2262	25226	37011	31496	34020	26201	35908	4679	5578	3332	11013	7633	8281	cat.2
ENSXETG00000003984	627	0	0	0	2	0	0	0	0	1	1	1	0	gh2
ENSXETG00000003985	2643	2298	3195	2781	6287	4722	11613	7075	7700	4866	20145	14427	15296	caprin1
ENSXETG00000003986	1360	3	8	8	39	28	104	432	501	289	130	106	107	lmo2
ENSXETG00000003991	2614	0	0	0	0	0	0	0	0	0	0	0	0	
ENSXETG00000003999	2718	426	614	557	1657	1212	563	1866	2054	1333	571	378	397	rrm1
ENSXETG00000004001	1305	0	0	0	3	4	4	0	2	1	2	3	3	stag3
ENSXETG00000004002	1542	2	0	0	3	3	1	65	72	54	1	2	3	gpc2
ENSXETG00000004003	1473	8	10	4	26	20	9	0	1	1	30	15	18	ENSXETG00000004003
ENSXETG00000004005	3382	97	130	114	2075	1615	1431	1731	1924	1228	3342	2313	2625	gal3st4
ENSXETG00000004007	624	16467	24753	21221	16562	12589	108032	23405	25954	16771	36420	25401	28052	ENSXETG00000004007
ENSXETG00000004009	2048	344	496	437	1178	969	1615	1806	2009	1301	1726	1167	1275	taf6
ENSXETG00000004010	1977	99	110	107	461	350	479	1188	1283	837	1656	1156	1235	ENSXETG00000004010
ENSXETG00000004011	1092	0	0	0	1	0	2	4	1	4	0	1	0	
ENSXETG00000004012	1344	9	14	12	25	22	35	64	66	35	86	73	92	mogat2.1
ENSXETG00000004015	3457	0	0	2	3	2	2	82	71	38	14	9	8	nat16
ENSXETG00000004016	999	1	7	3	1	2	6	13	20	8	3	5	5	ENSXETG00000004016
ENSXETG00000004021	1122	0	0	0	0	0	1	0	0	0	1	0	0	
ENSXETG00000004022	1492	2047	3194	2619	2696	2102	3314	4008	4781	2740	5537	3918	4165	drg2
ENSXETG00000004023	1204	184	260	250	173	117	191	604	761	380	167	117	118	zfand2a
ENSXETG00000004024	1557	35	31	45	27	34	48	248	312	164	133	87	91	il21r
ENSXETG00000004025	1812	201	304	250	662	512	797	817	966	571	523	370	407	pdpk1
ENSXETG00000004026	1439	186	299	219	299	261	424	808	932	569	298	231	242	kctd5
ENSXETG00000004027	1389	1785	2510	2037	1744	1355	2066	3342	4050	2322	1901	1378	1529	ube2i
ENSXETG00000004029	1911	2036	3044	2595	3277	2443	3851	4796	5553	3212	2155	1598	1684	vps28
ENSXETG00000004030	192	209	276	261	201	149	198	355	364	244	180	127	159	ENSXETG00000004030
ENSXETG00000004032	1968	122	156	134	141	119	125	254	305	161	72	57	52	gbe1
ENSXETG00000004033	3441	0	0	0	0	1	0	9	12	2	1	0	0	ndst3
ENSXETG00000004035	2550	0	1	2	5	3	36	198	193	133	32	23	26	prss12
ENSXETG00000004036	1026	232	295	256	174	135	197	270	334	195	228	162	194	ctu1
ENSXETG00000004037	1658	183	311	248	404	280	418	1167	1351	791	399	259	309	mettl14
ENSXETG00000004038	1310	1679	2458	2087	1332	1054	1754	1821	2138	1254	1052	695	749	vapa
ENSXETG00000004039	3078	2036	2868	2478	2277	1662	1346	1535	1738	1011	531	339	383	sec24c
ENSXETG00000004042	2914	704	967	788	1441	1130	1568	2035	2369	1334	1449	1032	1158	ppp4r1
ENSXETG00000004043	1023	0	0	0	1	2	2	15	13	13	6	1	7	myoz2
ENSXETG00000004044	2940	79	109	91	451	307	1077	711	949	469	843	577	659	usp53
ENSXETG00000004045	619	217349	313201	263436	147664	115423	118645	3877	4742	2562	41906	28454	32843	fabp2
ENSXETG00000004046	1812	27	54	35	42	68	203	366	451	262	242	194	220	pde5a
ENSXETG00000004047	2054	465	706	641	964	759	1532	2028	2288	1344	1556	1113	1169	ralbp1
ENSXETG00000004048	657	13	20	16	88	52	76	593	663	382	256	185	193	twsg1
ENSXETG00000004049	6488	241	379	304	856	620	769	1088	1252	781	1347	968	1123	ankrd12
ENSXETG00000004050	3630	51	85	74	341	255	215	405	444	300	737	527	633	ENSXETG00000004050
ENSXETG00000004051	862	2242	3203	2721	2256	1638	2589	2390	2869	1615	1901	1351	1479	ndufv2
ENSXETG00000004052	1249	431	648	481	457	355	509	623	747	408	450	344	383	napg
ENSXETG00000004054	6024	1	0	1	50	20	32	410	498	305	109	86	85	PIEZO2
ENSXETG00000004055	888	0	0	4	3	2	4	83	104	65	121	94	93	ENSXETG00000004055
ENSXETG00000004060	990	3	4	2	75	37	22	111	108	65	94	86	92	nkx3-2
ENSXETG00000004063	967	230	451	359	391	295	699	3400	3908	2327	478	346	367	crp.4
ENSXETG00000004065	2487	85	127	120	265	199	389	751	881	548	310	232	234	lrp12
ENSXETG00000004066	1476	7	6	14	26	19	34	126	119	61	35	22	22	dpys
ENSXETG00000004067	1377	13	19	22	75	45	101	116	118	87	117	96	115	bod1l1
ENSXETG00000004068	1420	0	0	0	20	12	28	73	118	54	41	30	33	syt1
ENSXETG00000004069	1293	0	0	0	0	0	2	10	12	9	0	1	0	dcstamp
ENSXETG00000004073	3873	5	4	4	26	27	41	125	152	111	101	74	93	ENSXETG00000004073
ENSXETG00000004074	998	106	144	133	239	165	332	168	223	123	155	95	127	pawr
ENSXETG00000004076	714	0	0	0	0	0	0	2	0	2	0	0	0	hs6st3
ENSXETG00000004078	3918	430	647	532	1074	752	1374	1605	1896	1112	1370	984	1083	nfrkb
ENSXETG00000004079	1350	4597	6706	5427	5319	3952	8023	1884	2271	1291	3176	2187	2437	tmem45b
ENSXETG00000004083	1671	511	783	654	743	581	1211	170	237	129	499	333	414	has1
ENSXETG00000004084	2682	0	0	0	0	0	0	0	0	0	0	0	0	
ENSXETG00000004086	2460	177	268	217	362	267	800	3612	4128	2489	818	618	630	btg5
ENSXETG00000004087	1263	0	0	0	1	0	0	6	9	3	1	0	0	
ENSXETG00000004088	1134	17	23	21	57	51	105	588	669	395	169	135	153	kcnn4
ENSXETG00000004092	1092	423	565	510	538	418	540	666	821	458	819	550	588	dcaf13
ENSXETG00000004096	1418	412	623	484	319	215	298	473	543	322	363	288	264	slc25a32
ENSXETG00000004097	1947	17	36	16	51	45	101	501	582	326	444	298	369	hspa1a
ENSXETG00000004098	3594	377	475	476	626	491	886	865	933	605	661	463	522	smg9
ENSXETG00000004102	444	0	0	0	0	0	0	0	1	0	1	0	0	
ENSXETG00000004104	1463	6	12	10	4	2	37	1650	1876	1104	22	17	21	cthrc1
ENSXETG00000004106	2097	45	75	65	454	333	182	765	802	515	675	431	506	fzd6
ENSXETG00000004115	2070	1107	1535	1322	1491	1164	3491	3108	3746	2170	1617	1100	1317	atp6v1c1
ENSXETG00000004118	2346	13002	18392	15529	6365	4989	10341	7342	8695	5246	3753	2531	3007	azin1
ENSXETG00000004120	1353	1236	1812	1670	800	574	913	2205	2322	1449	1375	968	1029	klf10
ENSXETG00000004121	2438	114	171	149	267	220	391	341	394	228	286	193	198	rrm2b
ENSXETG00000004122	2679	155	212	169	438	311	640	757	981	509	902	655	733	ppp2r3b
ENSXETG00000004123	3012	43	46	47	283	231	529	419	540	322	871	596	686	ENSXETG00000004123
ENSXETG00000004125	1593	147	199	179	144	140	481	225	268	147	252	169	215	gtpbp6
ENSXETG00000004126	909	112	186	135	140	85	117	151	185	106	182	130	122	plcxd1
ENSXETG00000004127	1146	1580	2465	2061	3635	2724	2647	701	781	503	1947	1377	1529	rgn
ENSXETG00000004129	2881	85	130	118	245	184	178	503	584	318	261	163	207	jade3
ENSXETG00000004132	3477	224	349	309	565	413	785	755	844	488	528	339	427	rp2
ENSXETG00000004133	3801	465	659	540	688	516	934	1062	1363	803	608	406	466	slc9a7
ENSXETG00000004136	2763	116	149	117	586	442	1265	540	676	396	427	321	394	chst7
ENSXETG00000004137	2949	305	447	380	612	452	433	526	634	340	435	300	356	tubgcp5
ENSXETG00000004138	3765	1505	2074	1821	3441	2545	5007	5153	6188	3568	5019	3597	4048	cyfip1
ENSXETG00000004143	1173	24	54	53	59	51	87	123	168	96	78	62	72	stradb
ENSXETG00000004144	1969	2352	3365	2806	3360	2588	5964	2637	3133	1850	2995	2064	2329	nipa2
ENSXETG00000004145	1569	418	513	497	520	416	856	563	712	370	437	287	338	casp10
ENSXETG00000004148	14529	361	515	442	1383	941	1819	2061	2300	1424	2029	1466	1633	herc2
ENSXETG00000004151	1705	301	415	341	527	357	702	497	577	350	474	405	418	orc2
ENSXETG00000004152	1219	968	1487	1275	1061	791	2692	458	587	337	1222	850	944	c9orf64
ENSXETG00000004154	3123	983	1484	1236	1598	1147	1503	1721	2020	1114	1122	855	969	cyp20a1
ENSXETG00000004155	1764	18	35	28	127	80	223	897	1050	652	402	272	281	abi2
ENSXETG00000004157	2187	0	2	3	3	0	8	2	1	0	2	2	0	oca2
ENSXETG00000004164	2300	27	41	46	108	102	160	600	658	425	159	127	151	kif3a
ENSXETG00000004165	1086	149	200	188	351	255	521	937	1037	593	469	318	378	ccni2
ENSXETG00000004166	1092	49	57	52	154	139	229	432	485	316	371	289	318	sowaha
ENSXETG00000004167	4299	512	769	652	1644	1234	3006	3284	3889	2198	2929	2020	2263	shroom1
ENSXETG00000004168	1191	0	0	0	0	0	0	2	1	0	0	0	0	

ENSXETG00000004169	3483	129	143	137	253	168	554	381	480	255	473	360	399	aff4
ENSXETG00000004170	2433	0	1	0	0	0	0	8	9	7	2	3	0	XB-GENE-1014282
ENSXETG00000004171	4032	323	400	366	278	212	437	649	732	407	457	319	408	zcchc10
ENSXETG00000004172	1122	2751	3937	3375	1814	1405	7960	6228	7466	4576	1850	1312	1555	gnpda1
ENSXETG00000004176	952	1102	1493	1360	1243	966	967	736	824	484	435	272	378	mocs2
ENSXETG00000004177	3297	78	111	117	446	409	640	2490	3148	1837	1081	761	920	itga2
ENSXETG00000004178	468	404	579	493	367	266	580	367	445	256	364	253	244	rnf185
ENSXETG00000004179	4594	213	297	254	514	413	320	825	1064	596	644	458	543	limk2
ENSXETG00000004180	3486	21	37	39	244	190	550	1484	1789	1086	697	497	596	itga1
ENSXETG00000004181	2803	198	313	247	114	88	341	12	11	9	273	174	181	pnliprp2
ENSXETG00000004188	1224	5	5	7	45	46	15	50	56	41	67	55	54	isl1
ENSXETG00000004189	1268	874	1204	1096	2518	1890	2604	1505	1750	990	2811	1975	2209	pik3ip1
ENSXETG00000004190	1938	48	83	72	348	199	209	897	962	587	454	321	385	patz1
ENSXETG00000004191	999	1220	1729	1412	1753	1342	2529	1877	2158	1341	2725	1893	2247	drg1
ENSXETG00000004192	1188	0	0	0	0	0	0	0	0	0	0	0	0	
ENSXETG00000004193	980	543	785	627	957	730	985	1162	1297	876	909	629	659	stx16
ENSXETG00000004194	3385	657	894	759	1049	791	904	1484	1725	969	1090	771	914	eif4enif1
ENSXETG00000004196	1955	385	597	465	311	238	219	414	424	247	286	195	250	pisd
ENSXETG00000004200	2649	2	7	7	241	177	30	87	98	59	17	10	13	ENSXETG00000004200
ENSXETG00000004201	1446	0	0	1	25	15	4	3	7	4	3	0	1	ENSXETG00000004201
ENSXETG00000004203	1353	115	189	136	158	122	205	318	355	223	195	142	159	prr14l
ENSXETG00000004204	2082	5	5	3	2	3	9	35	41	19	11	8	8	depdc5
ENSXETG00000004205	1696	674	982	845	639	471	374	1247	1484	871	1309	941	983	ywhah
ENSXETG00000004206	1971	23270	33643	29334	39765	30225	33225	1849	1964	1313	29224	20557	22875	ENSXETG00000004206
ENSXETG00000004208	1354	88	120	105	98	82	41	15	20	8	11	9	6	rsph14
ENSXETG00000004209	4902	607	883	767	2303	1762	2306	3015	3253	2013	2520	1922	1991	map4
ENSXETG00000004211	2754	2	0	1	6	3	1	30	36	34	26	12	16	sez6l
ENSXETG00000004212	2247	1	1	0	0	0	0	1	2	0	3	0	1	ENSXETG00000004212
ENSXETG00000004213	4005	578	871	703	423	369	246	568	605	346	382	319	347	dhx30
ENSXETG00000004215	5503	116	192	161	1063	798	795	1081	1226	770	1823	1378	1436	bcr
ENSXETG00000004219	3820	113	160	167	409	391	676	1012	1178	759	778	544	660	specc1l
ENSXETG00000004220	1171	94	128	116	164	120	200	411	462	265	239	184	229	tpst2
ENSXETG00000004221	794	0	1	1	1	1	0	0	1	0	0	0	0	crybb1
ENSXETG00000004222	1860	16	34	27	26	27	50	331	371	207	91	57	87	adora2a
ENSXETG00000004224	3288	72	87	79	177	127	86	209	260	171	93	58	66	trpc2
ENSXETG00000004225	1342	77	123	103	122	87	141	59	56	35	128	108	105	upb1
ENSXETG00000004227	714	0	0	1	0	0	0	0	0	0	0	0	0	
ENSXETG00000004229	960	67	96	84	56	46	77	154	202	114	79	64	66	ENSXETG00000004229
ENSXETG00000004230	1575	820	1198	1050	800	615	857	1128	1275	719	934	692	747	gucd1
ENSXETG00000004231	927	21	36	30	48	35	53	30	41	18	50	39	46	lrrc75b
ENSXETG00000004232	3023	3151	4603	3703	4430	3262	3950	676	733	455	1848	1316	1532	ggt1
ENSXETG00000004234	1833	62	76	84	45	30	77	166	145	107	75	42	56	csrnp1
ENSXETG00000004239	2151	12	20	15	17	7	25	8	12	6	51	29	33	ENSXETG00000004239
ENSXETG00000004245	1298	186	277	219	328	270	304	702	774	471	192	119	130	gorasp1
ENSXETG00000004246	2550	3	0	1	1	2	2	1	5	2	10	8	3	ENSXETG00000004246
ENSXETG00000004247	2907	279	426	407	842	649	803	843	1002	584	832	578	632	wdr48
ENSXETG00000004250	1834	33	44	40	162	132	409	603	765	466	321	253	265	ggt5
ENSXETG00000004251	5978	3	6	2	18	12	12	15	17	10	15	17	23	scn5a
ENSXETG00000004252	2649	560	869	669	1168	943	1077	1561	1684	1019	1086	754	903	smpd4
ENSXETG00000004253	2223	268	390	329	858	625	1076	1213	1324	782	1259	833	974	med15
ENSXETG00000004254	969	77	107	124	132	106	305	175	170	128	198	154	190	sirt4
ENSXETG00000004256	3606	389	577	492	595	449	985	854	1143	690	732	544	570	klhl22
ENSXETG00000004257	2193	0	2	3	0	0	0	2	0	0	0	1	0	ENSXETG00000004257
ENSXETG00000004258	1105	455	707	607	736	573	950	1382	1486	962	936	642	752	crkl
ENSXETG00000004260	2681	0	0	1	0	2	0	2	8	2	0	0	1	vegt
ENSXETG00000004261	357	0	0	0	0	0	0	5	1	3	0	0	0	ENSXETG00000004261
ENSXETG00000004263	7732	435	662	547	1293	1009	1537	2200	2403	1382	2041	1502	1592	cabin1
ENSXETG00000004266	2538	1	1	0	0	0	0	3	2	1	1	1	1	ENSXETG00000004266
ENSXETG00000004276	2499	0	1	0	0	0	0	1	2	2	1	1	0	ENSXETG00000004276
ENSXETG00000004281	1290	37	53	41	31	25	56	69	65	53	40	38	26	mettl15
ENSXETG00000004282	5152	42	72	60	217	173	117	421	461	280	49	25	38	kif18a
ENSXETG00000004284	1209	2	2	3	2	0	5	3	4	4	2	6	3	bdnf
ENSXETG00000004286	705	0	0	0	0	0	0	0	0	0	0	0	0	
ENSXETG00000004287	4751	17	19	19	125	109	272	850	1002	620	399	297	326	ptprm
ENSXETG00000004288	2541	0	0	0	5	6	5	17	15	9	14	12	12	slitrk2
ENSXETG00000004289	2853	79	103	96	121	100	267	598	758	408	345	237	265	rab12
ENSXETG00000004290	5712	8	6	5	9	15	24	336	424	238	53	37	44	mtcl1
ENSXETG00000004291	2121	1267	1936	1633	2108	1606	1911	390	428	234	504	360	418	acss2.2
ENSXETG00000004292	2655	54	72	51	85	71	229	189	205	112	239	182	199	rapgef3
ENSXETG00000004294	858	0	0	0	0	1	0	0	0	0	0	0	0	
ENSXETG00000004295	2019	0	0	0	1	0	0	1	3	0	1	0	0	
ENSXETG00000004296	1227	2	2	2	0	0	5	9	9	3	0	1	3	XB-GENE-987190
ENSXETG00000004297	1341	6	10	11	42	36	49	374	452	212	61	50	47	ppp1r1a
ENSXETG00000004298	1894	4	1	3	23	5	54	104	130	69	60	46	42	pde1b
ENSXETG00000004299	3075	66	67	70	86	64	271	1610	1811	1112	246	166	202	nckap1l
ENSXETG00000004300	645	62	80	51	93	110	423	321	421	221	355	217	262	erp29
ENSXETG00000004301	2930	557	851	696	603	429	640	1177	1404	780	753	540	652	naa25
ENSXETG00000004302	12506	149	204	177	495	403	602	841	1030	583	676	477	562	hectd4
ENSXETG00000004304	627	73989	107879	89201	94913	71321	103926	88632	98556	58274	195803	135656	150060	rpl6
ENSXETG00000004305	1941	183	271	249	303	233	463	737	930	543	378	279	311	PTPN11
ENSXETG00000004309	954	0	0	0	0	0	0	0	0	0	0	0	0	
ENSXETG00000004310	1350	34	43	34	245	183	237	178	228	123	191	131	154	ENSXETG00000004310
ENSXETG00000004314	2091	167	244	210	462	340	580	366	384	219	556	399	451	cdk5rap1
ENSXETG00000004315	987	346	496	427	257	210	524	188	201	116	169	97	104	mmd
ENSXETG00000004316	888	19	29	18	47	34	172	67	79	58	58	41	39	hlf
ENSXETG00000004317	1667	1529	2117	1790	3352	2566	9480	2247	2761	1491	10715	7654	8513	tom1l1
ENSXETG00000004319	3889	872	1208	1093	851	667	1632	1478	1746	987	1236	887	1063	stard7
ENSXETG00000004320	804	2	1	0	0	0	2	0	8	8	5	3	4	ENSXETG00000004320
ENSXETG00000004324	3783	6	7	10	31	18	99	622	686	413	80	71	70	nrk
ENSXETG00000004327	2044	222	314	277	487	368	694	836	944	557	471	329	375	fam199x
ENSXETG00000004328	666	332	470	462	379	286	645	479	569	376	282	205	191	commd5
ENSXETG00000004330	4943	56	73	45	233	172	375	2811	3281	1821	857	638	695	ENSXETG00000004330
ENSXETG00000004333	453	3228	4600	3873	3280	2513	4633	1857	2086	1220	1820	1269	1413	pgrmc1
ENSXETG00000004334	1029	43	75	60	111	83	327	129	117	90	234	155	220	slc25a43
ENSXETG00000004336	2464	914	1386	1146	1364	1069	1953	2004	2267	1285	1197	847	890	ciao1
ENSXETG00000004338	740	287	435	325	425	350	851	1315	1482	910	650	433	462	tmem127
ENSXETG00000004339	1019	636	948	817	947	726	1017	1509	1634	1006	768	537	576	fahd2a
ENSXETG00000004341	771	1	0	0	0	0	1	4	7	1	0	0	0	kcnip3
ENSXETG00000004342	2196	0	0	0	0	0	0	1	0	0	0	0	0	
ENSXETG00000004348	3456	60	86	84	306	256	521	320	369	248	543	391	485	c20orf194
ENSXETG00000004350	1503	0	0	0	0	0	0	0	0	0	0	0	0	
ENSXETG00000004357	4014	766	1174	983	2948	2351	5180	2723	3121	2021	2963	1991	2347	atrn
ENSXETG00000004360	1113	0	0	0	0	0	0	3	0	0	0	0	0	
ENSXETG00000004361	2046	28	29	32	181	138	379	450	526	299	1097	759	865	faah.3
ENSXETG00000004362	1002	3	4	6	12	5	7	14	17	2	2	4	1	gfra4
ENSXETG00000004363	2184	99	155	125	216	153	95	35	38	24	258	204	205	ENSXETG00000004363
ENSXETG00000004364	3058	26	30	39	46	28	77	239	233	164	96	56	66	ago1
ENSXETG00000004365	1866	1817	2531	2159	4020	3279	7309	567	643	343	1965	1454	1578	faah.2
ENSXETG00000004366	312	3	4	5	0	0	5	34	26	17	51	45	49	uqcrh
ENSXETG00000004367	1479	74	95	73	108	74	206	261	278	167	183	129	147	lrrc41
ENSXETG00000004368	1227	0	0	0	0	0	0	0	0	0	0	0	0	

ENSXETG00000004369	2277	58	59	45	142	112	51	195	229	115	17	17	26	rad54l
ENSXETG00000004370	2366	188	292	254	238	181	457	527	639	372	353	269	279	pomgnt1
ENSXETG00000004371	1997	924	1310	1174	2130	1693	1004	2655	3200	1836	3118	2209	2402	tspan1
ENSXETG00000004372	1929	48	57	61	11	12	62	0	0	0	1	1	0	umod.1
ENSXETG00000004373	1595	1	3	5	416	284	13	8	7	5	7	4	7	tekt2
ENSXETG00000004374	1922	399	527	452	605	449	1035	847	1050	614	586	444	478	adprhl2
ENSXETG00000004376	2085	1	3	1	2	2	4	83	113	73	4	9	6	col8a2
ENSXETG00000004378	2738	1352	1932	1679	1156	932	1319	1670	2142	1210	883	610	685	trappc3
ENSXETG00000004379	1497	4	7	2	12	7	16	58	71	55	16	11	14	ANGPT2
ENSXETG00000004380	1836	3	4	0	2	5	4	2	4	3	0	0	0	ENSXETG00000004380
ENSXETG00000004382	3016	67	98	75	373	255	553	2819	3115	1867	1170	812	896	map7d1
ENSXETG00000004387	2389	167	262	204	332	242	443	799	977	540	397	277	321	cept1
ENSXETG00000004388	2962	655	810	736	544	416	966	640	826	462	495	377	421	dennd2d
ENSXETG00000004390	3708	1	0	2	9	7	9	79	83	45	19	26	15	nfasc
ENSXETG00000004391	10125	352	484	485	1865	1442	1764	4558	5184	3123	3971	2774	3128	fryl
ENSXETG00000004406	1820	0	0	0	0	0	1	15	15	9	4	2	2	ENSXETG00000004406
ENSXETG00000004407	2079	20074	29534	25275	31826	24253	36336	43918	49461	29903	125640	87240	96806	naca.2
ENSXETG00000004408	1780	0	0	0	1	0	0	1	0	1	0	0	0	XB-GENE-5902365
ENSXETG00000004409	1210	4	5	5	56	32	6	35	39	12	26	27	30	rdh7.2
ENSXETG00000004411	1075	54	53	51	279	233	261	950	1108	661	684	499	531	hsd17b6
ENSXETG00000004412	1077	16	25	19	78	63	113	71	96	60	27	18	26	gpr182
ENSXETG00000004415	930	1	3	2	2	5	5	17	22	19	2	0	2	ENSXETG00000004415
ENSXETG00000004417	2088	62	81	65	83	98	92	171	198	99	122	76	99	zbtb39
ENSXETG00000004420	1005	15	33	18	82	65	63	232	234	138	110	93	83	ccdc89
ENSXETG00000004422	3515	16509	24149	20879	40285	30322	34856	6777	7833	4504	20952	14984	16782	myo1a
ENSXETG00000004423	561	0	0	0	0	0	0	1	0	0	0	0	1	
ENSXETG00000004424	2964	11	7	11	48	46	65	125	138	91	260	186	205	scube2
ENSXETG00000004425	4701	117	185	164	434	378	911	3154	3652	2223	1299	882	1010	dennd5a
ENSXETG00000004426	2253	141	177	171	222	180	295	347	394	195	158	112	128	nemp1
ENSXETG00000004428	5938	12	15	18	22	22	47	212	211	126	105	79	75	nab2
ENSXETG00000004429	1797	78	113	125	420	313	506	698	706	459	771	563	574	zdhhc13
ENSXETG00000004431	2355	519	787	660	1193	934	1849	2105	2543	1481	2413	1716	1915	stat6
ENSXETG00000004435	1104	7	24	24	14	12	32	231	249	137	61	42	39	csrp3
ENSXETG00000004436	3064	110	152	120	449	360	173	641	705	435	95	86	91	e2f8
ENSXETG00000004449	781	52	85	80	155	116	252	380	449	262	119	92	69	rab23
ENSXETG00000004450	1593	123	191	145	263	208	142	341	360	218	129	90	109	prim2
ENSXETG00000004451	1993	32	44	38	24	15	33	27	33	26	18	15	10	khdrbs2
ENSXETG00000004452	942	646	940	784	1751	1329	1129	43	47	28	77	65	68	ENSXETG00000004452
ENSXETG00000004453	1353	2	4	1	4	3	1	2	1	2	2	1	0	lgsn
ENSXETG00000004454	1936	537	718	591	568	529	839	2085	2570	1439	769	583	591	ptp4a1
ENSXETG00000004455	5855	136	181	156	568	417	591	635	805	422	933	665	745	phf3
ENSXETG00000004456	690	551	821	720	1041	737	648	1682	1796	1120	648	447	490	u2af1
ENSXETG00000004457	962	575	938	776	1015	725	1319	388	448	252	1377	1003	1110	c21orf33
ENSXETG00000004459	1477	1045	1501	1318	1322	1009	1813	1981	2331	1345	1396	1018	1043	fnta
ENSXETG00000004460	1059	102	165	141	96	94	206	168	238	135	150	99	132	pomk
ENSXETG00000004461	2980	76	89	100	137	110	129	214	244	144	57	38	50	hgsnat
ENSXETG00000004462	2136	143	217	181	329	241	349	436	527	309	410	289	352	ints10
ENSXETG00000004463	1596	4	3	1	21	14	14	42	59	39	38	19	25	csgalnact1
ENSXETG00000004464	2579	266	344	298	1142	901	1151	1743	2047	1266	1440	980	1152	sh2d4a
ENSXETG00000004466	1279	12	19	20	35	23	49	116	196	97	64	35	37	psd3
ENSXETG00000004467	1524	101	145	113	464	397	662	113	158	95	1152	757	984	lpl
ENSXETG00000004468	3766	402	609	489	1199	919	1019	2152	2434	1552	1788	1245	1312	gse1
ENSXETG00000004469	1469	112	143	136	265	196	218	304	398	222	150	116	131	gins2
ENSXETG00000004470	1302	6	4	2	219	174	20	35	38	39	22	16	12	cfap157
ENSXETG00000004471	725	1045	1372	1118	897	701	1162	1972	2638	1440	1070	744	884	snrpe
ENSXETG00000004472	809	1341	1981	1626	1320	1019	1407	1782	2178	1312	912	616	780	mcts1
ENSXETG00000004473	668	18187	25508	21222	17686	13245	19160	10544	12423	7334	14653	10367	11297	cox4i1
ENSXETG00000004474	5037	534	681	623	1078	786	1478	1669	2027	1190	1300	902	1028	cul4b
ENSXETG00000004476	3428	3216	4721	4065	5116	3941	13724	6578	7582	4348	6844	4958	5605	lamp2
ENSXETG00000004477	888	0	0	0	0	0	1	2	1	0	0	0	0	
ENSXETG00000004478	1288	0	2	3	4	8	4	10	74	73	67	20	15	17 tmem255a
ENSXETG00000004479	2106	51	67	41	103	74	221	333	410	253	178	161	178	zbtb33
ENSXETG00000004481	664	0	0	0	0	0	0	4	2	2	0	0	0	myl10
ENSXETG00000004483	1931	4	5	2	14	19	21	182	214	140	29	20	21	orai1
ENSXETG00000004486	2901	1	1	0	7	3	13	32	48	23	11	13	10	kif5c
ENSXETG00000004487	756	1028	1416	1343	679	517	477	1709	2043	1281	406	265	332	ENSXETG00000004487
ENSXETG00000004488	1497	54	65	59	24	30	12	52	75	44	4	2	2	acod1
ENSXETG00000004489	2758	46	68	62	95	51	24	293	375	215	8	12	11	ENSXETG00000004489
ENSXETG00000004490	1113	7	18	17	66	42	164	163	213	109	218	140	216	ENSXETG00000004490
ENSXETG00000004491	1506	4	4	4	0	0	0	14	12	7	0	0	0	ENSXETG00000004491
ENSXETG00000004495	744	20	16	17	6	2	2	1	7	7	0	0	0	XB-GENE-5791624
ENSXETG00000004496	792	50	81	69	9	8	3	209	223	149	1	0	0	ENSXETG00000004496
ENSXETG00000004498	1002	1356	2023	1703	1183	890	1484	1444	1685	1080	861	613	717	magt1
ENSXETG00000004499	7480	321	438	371	1144	838	1614	2330	2842	1690	1987	1396	1548	atrx
ENSXETG00000004500	2830	98	140	128	262	194	111	506	513	293	50	33	56	mcm8
ENSXETG00000004501	1790	749	1057	929	976	798	821	1288	1467	835	1280	975	996	trmt6
ENSXETG00000004506	609	0	1	0	3	1	0	2	0	0	2	0	1	fgf16
ENSXETG00000004510	2206	140	173	151	230	184	310	507	520	343	444	337	375	gzf1
ENSXETG00000004516	6109	2	11	9	47	51	199	823	1002	576	161	124	143	ccdc88a
ENSXETG00000004519	468	27729	40480	34209	41887	31800	51826	49561	56309	33057	123028	86232	94925	rps27a
ENSXETG00000004521	4239	8634	12631	10563	9650	7124	14570	9614	10966	6431	12995	9300	10181	rtn4
ENSXETG00000004522	2066	13	12	6	158	142	60	249	315	184	363	246	266	lypd6
ENSXETG00000004524	1236	856	1309	1114	1183	1010	1391	932	979	611	457	309	364	mmadhc
ENSXETG00000004525	1404	356	483	430	551	444	778	1435	1695	1067	522	429	467	nmi
ENSXETG00000004526	1842	1	1	1	0	0	2	1	1	1	1	0	0	ENSXETG00000004526
ENSXETG00000004529	810	9	2	2	2	1	2	27	19	21	14	11	9	tnfaip6
ENSXETG00000004530	6948	341	487	422	733	551	283	953	1204	697	636	431	536	rif1
ENSXETG00000004531	18522	1	0	0	0	0	0	2	1	0	2	0	0	
ENSXETG00000004532	1266	0	4	2	12	5	57	294	380	212	45	27	25	B3GALT2
ENSXETG00000004533	2132	612	903	865	1243	951	1595	1727	1972	1231	2281	1574	1693	cdc73
ENSXETG00000004534	1614	1531	2184	1698	756	602	640	1243	1601	883	479	296	364	glrx2
ENSXETG00000004535	3165	2008	2857	2377	1516	1145	1221	1955	2283	1294	2685	1958	2155	ivns1abp
ENSXETG00000004536	1200	8	11	6	23	10	24	88	126	77	36	27	24	b3galt2
ENSXETG00000004539	1805	91	144	122	211	169	275	365	444	231	239	190	207	trmt1l
ENSXETG00000004541	2870	302	482	413	533	387	806	733	819	485	480	310	382	edem3
ENSXETG00000004542	1385	170	233	211	337	264	424	1170	1341	909	611	483	499	bambi
ENSXETG00000004545	1947	528	715	663	1436	1050	1837	1898	2073	1214	2073	1455	1648	wac
ENSXETG00000004546	2418	22	57	31	148	132	222	308	359	207	250	176	190	mpp7
ENSXETG00000004547	3687	0	4	3	1	2	1	2	1	5	0	0	0	mei1
ENSXETG00000004548	2305	1069	1470	1254	1864	1448	2348	2053	2343	1432	1454	1005	1117	xrcc6
ENSXETG00000004549	1128	1092	1612	1383	6971	5358	9229	2291	2722	1568	4421	2969	3438	pmm2
ENSXETG00000004553	3620	0	5	1	306	206	7	3	5	2	16	7	11	armc4
ENSXETG00000004554	1053	0	0	0	1	0	2	15	19	9	7	4	8	mkx
ENSXETG00000004556	2437	1569	2129	1815	2213	1706	3638	3487	4181	2392	2217	1522	1776	rab18
ENSXETG00000004557	2394	1	11	6	3	10	2	9	13	14	48	34	30	ptchd3
ENSXETG00000004558	2163	886	1316	1151	1425	1120	1501	1729	2022	1206	1115	730	873	yme1l1
ENSXETG00000004564	1068	11	28	16	69	64	87	284	298	178	181	140	143	fut1
ENSXETG00000004570	480	4	4	3	6	10	23	2	3	2	6	2	5	ENSXETG00000004570
ENSXETG00000004571	4438	53	90	71	247	147	404	1817	2077	1253	621	447	453	XB-GENE-986079
ENSXETG00000004572	2067	715	1020	892	641	513	759	611	634	365	436	297	322	abcb7
ENSXETG00000004573	831	18	23	29	87	59	700	1147	1334	737	277	201	242	hsd17b14

ENSXETG00000004575	1200	0	0	0	0	0	0	0	0	0	0	0	0	
ENSXETG00000004577	2802	16	25	29	19	14	21	103	109	68	27	24	27	ENSXETG00000004577
ENSXETG00000004578	1017	53	102	84	132	96	182	166	183	117	183	129	140	zdhhc15
ENSXETG00000004579	738	436	602	498	322	255	465	545	631	367	417	273	294	uprt
ENSXETG00000004580	2140	531	747	668	1377	981	1663	2488	2752	1627	1681	1172	1300	rlim
ENSXETG00000004581	1239	4	1	3	12	5	49	394	504	326	67	43	51	slc16a2
ENSXETG00000004583	1757	0	1	0	0	0	2	6	4	1	3	2	9	ENSXETG00000004583
ENSXETG00000004584	1023	0	0	0	0	0	0	0	0	0	0	0	0	
ENSXETG00000004585	1862	455	632	545	391	312	590	913	1054	613	483	323	366	usp46
ENSXETG00000004589	2366	0	0	0	1	0	0	0	0	0	0	0	0	
ENSXETG00000004590	1212	206	316	256	392	308	768	938	1060	621	589	405	454	cyth1
ENSXETG00000004592	1311	0	0	3	3	3	5	18	16	10	5	1	4	ENSXETG00000004592
ENSXETG00000004593	1599	3	5	4	15	10	14	109	124	81	16	8	7	ENSXETG00000004593
ENSXETG00000004595	4791	1	0	0	0	1	2	5	6	8	3	7	2	lmtk3
ENSXETG00000004596	3564	17	23	27	29	26	37	331	406	243	66	33	61	gprc5c
ENSXETG00000004597	1257	251	356	314	163	133	200	459	539	313	217	162	177	tsen34
ENSXETG00000004598	1901	833	1263	1005	889	672	1186	1395	1590	968	1686	1287	1302	grwd1
ENSXETG00000004603	1227	335	465	416	623	468	421	850	1021	571	167	123	145	pla2g7
ENSXETG00000004606	2487	10855	15509	13366	9341	6867	7881	990	1133	720	885	609	736	bbox1
ENSXETG00000004609	612	10	19	14	43	26	77	431	471	270	204	140	148	fibin
ENSXETG00000004611	1026	20	20	18	31	17	128	423	465	278	33	34	33	ENSXETG00000004611
ENSXETG00000004612	1357	1508	2202	1855	1910	1437	4349	1804	2001	1091	1766	1158	1324	tollip
ENSXETG00000004614	2127	3	2	1	4	4	6	29	41	14	12	11	9	brsk2
ENSXETG00000004621	984	5	21	8	13	3	26	15	11	11	4	2	4	tnni2
ENSXETG00000004623	3126	53	45	68	2941	2443	232	624	733	455	4906	3548	3994	syt8
ENSXETG00000004629	1800	701	1002	845	353	330	925	3133	3462	2116	800	534	645	rac2
ENSXETG00000004630	778	260	413	350	184	129	896	837	1007	580	269	203	197	dcxr
ENSXETG00000004632	1302	86	131	113	156	102	179	211	255	135	281	205	198	usp36
ENSXETG00000004635	2617	1010	1496	1234	4960	3772	4168	6252	7763	4252	3290	2281	2557	XB-GENE-990339
ENSXETG00000004641	2562	2	5	4	0	2	3	10	10	6	4	2	4	cpsf4l
ENSXETG00000004642	2294	522	798	646	273	229	507	1790	2199	1314	706	496	580	cdc42ep4
ENSXETG00000004643	6219	11	19	22	118	101	173	362	486	254	188	147	158	SDK2
ENSXETG00000004647	969	0	0	0	1	0	0	0	0	0	0	0	2	ENSXETG00000004647
ENSXETG00000004649	2421	281	414	364	530	440	1132	1344	1619	912	785	483	613	stat1
ENSXETG00000004650	1704	18352	26131	22225	20582	15501	21813	17257	20562	11798	15395	11010	12043	pdia3
ENSXETG00000004651	1842	603	856	731	517	389	1069	1312	1443	833	530	373	437	slc39a1
ENSXETG00000004653	3414	182	205	181	269	213	570	259	324	184	357	251	309	ppip5k1
ENSXETG00000004660	1187	3403	4695	4203	3213	2509	2221	2738	3473	1983	1265	893	1082	dnajb11
ENSXETG00000004662	1977	29	50	33	19	16	35	139	211	96	83	55	57	stat4
ENSXETG00000004663	1455	0	0	0	0	0	0	0	0	0	0	0	0	
ENSXETG00000004664	878	1051	1688	1315	1820	1322	1191	1209	1220	729	795	575	606	CYC1
ENSXETG00000004666	1629	312	467	401	1001	753	1756	797	876	541	814	591	598	ENSXETG00000004666
ENSXETG00000004668	582	489	659	555	943	646	612	733	770	526	746	517	549	exosc4
ENSXETG00000004669	1485	55	91	77	157	100	147	269	294	184	223	154	171	lrrc14
ENSXETG00000004670	2640	5351	7988	6905	20013	15214	11934	11702	12728	8444	19082	13490	14423	mvp
ENSXETG00000004671	1104	196	313	269	230	180	118	314	313	196	78	67	89	naprt
ENSXETG00000004672	1070	448	732	642	908	656	1384	1292	1430	921	1336	961	1020	pagr1
ENSXETG00000004673	1749	90	150	129	370	282	207	744	803	492	62	37	44	kif22
ENSXETG00000004674	2021	9600	14649	12572	17261	13225	18117	15930	17632	11149	44747	31981	33560	eef1d
ENSXETG00000004675	1224	43	67	53	127	117	219	436	446	327	315	246	272	maz
ENSXETG00000004676	1893	19	32	29	50	33	94	168	179	120	119	65	92	ENSXETG00000004676
ENSXETG00000004677	1341	1737	2619	2140	1906	1467	4741	3117	3717	2033	1755	1195	1497	snx2
ENSXETG00000004679	3273	69	72	65	167	108	220	1008	1264	722	369	278	292	lox
ENSXETG00000004680	1605	6	4	6	16	11	65	101	108	92	76	50	70	tigd5
ENSXETG00000004681	1421	283	367	351	455	397	690	593	683	388	899	614	747	srfbp1
ENSXETG00000004682	2333	823	1066	898	1013	742	1376	1391	1527	998	900	717	683	gpaa1
ENSXETG00000004683	1085	0	1	4	16	6	0	0	0	0	35	29	33	XB-GENE-5830570
ENSXETG00000004684	722	0	0	0	0	0	0	0	0	0	0	0	1	
ENSXETG00000004685	1824	0	0	0	0	0	0	0	0	0	0	0	0	
ENSXETG00000004686	1641	6	8	7	42	28	49	141	176	89	79	64	70	ENSXETG00000004686
ENSXETG00000004687	987	0	0	0	11	5	0	4	3	1	0	0	0	
ENSXETG00000004691	1503	73	113	109	317	230	488	1209	1234	801	655	499	577	trim3
ENSXETG00000004692	7119	4	8	11	91	68	140	728	831	567	210	174	178	DCHS1
ENSXETG00000004693	1386	0	0	0	1	0	0	0	0	0	1	0	0	
ENSXETG00000004697	1437	56	86	73	154	112	151	147	233	150	130	76	76	gtf3c6
ENSXETG00000004698	993	84	143	107	122	96	159	179	208	97	199	139	171	hsdl1
ENSXETG00000004699	2103	228	307	298	460	329	747	876	1020	535	560	407	466	bcar1
ENSXETG00000004700	1143	0	3	7	7	2	6	172	193	123	13	10	8	hpx
ENSXETG00000004702	1317	192	309	236	221	191	330	341	408	268	289	197	248	tmem170a
ENSXETG00000004704	1167	617	856	759	79	76	639	50	67	34	412	317	371	hsd11b2
ENSXETG00000004705	1915	1145	1640	1296	1317	1030	3009	2191	2730	1577	1260	827	1073	atp6v0d1
ENSXETG00000004708	2660	210	304	251	312	251	241	641	744	420	262	146	180	tubgcp4
ENSXETG00000004710	6127	2705	3798	3166	7681	6053	6991	3027	3364	2109	7656	5329	6112	lmo7
ENSXETG00000004711	943	744	1045	881	641	516	886	1820	2133	1160	559	374	451	uchl3
ENSXETG00000004712	1642	753	1017	850	514	435	844	1448	1665	967	285	191	208	commd6
ENSXETG00000004713	3528	102	117	115	238	174	364	283	322	224	544	367	445	tbc1d4
ENSXETG00000004714	1191	0	2	0	5	2	13	17	21	18	9	7	6	klf12
ENSXETG00000004715	1338	184	258	202	2260	1755	1177	682	770	468	1700	1353	1363	klf5
ENSXETG00000004716	2561	63	75	69	263	194	407	300	406	211	304	177	196	pibf1
ENSXETG00000004720	258	263	465	382	318	221	359	398	445	253	489	388	359	etfrf1
ENSXETG00000004721	3275	525	792	650	1549	1225	2045	2342	2732	1597	2335	1781	1966	dsc3
ENSXETG00000004725	631	606	821	709	511	393	193	6	2	2	82	67	66	ttr
ENSXETG00000004726	3877	225	356	278	742	549	613	1010	1164	709	554	394	429	b4galt5
ENSXETG00000004727	495	0	2	3	14	16	8	4	13	2	7	6	10	
ENSXETG00000004728	987	69	100	80	113	86	140	209	242	132	196	119	127	gorab
ENSXETG00000004729	1314	1	0	2	6	4	4	0	0	0	0	0	0	ENSXETG00000004729
ENSXETG00000004731	853	2	0	0	0	0	0	0	0	0	0	0	0	mettl11b
ENSXETG00000004732	1725	2248	3343	2915	3843	2934	6461	1471	1738	1049	2345	1731	1872	ilvbl
ENSXETG00000004733	3403	98	144	164	330	226	375	643	767	434	264	214	239	kifap3
ENSXETG00000004734	4558	560	841	727	1169	802	1622	1585	1876	1076	1124	831	912	trappc8
ENSXETG00000004735	2187	164	229	185	466	304	666	574	671	395	430	295	316	scyl3
ENSXETG00000004737	4930	290	400	346	546	463	948	938	1269	665	1056	683	851	zbtb40
ENSXETG00000004738	2879	99	150	115	188	133	92	190	196	112	112	72	85	c1orf112
ENSXETG00000004739	893	48	48	44	42	37	46	11	10	13	6	0	6	rnf138
ENSXETG00000004740	375	546	797	640	535	416	774	211	267	142	736	517	614	ENSXETG00000004740
ENSXETG00000004741	2199	2	0	1	4	5	1	25	25	15	8	2	5	selp
ENSXETG00000004742	1056	1	1	0	3	3	3	38	41	19	5	2	6	wnt4
ENSXETG00000004743	1945	6486	9310	7644	7913	6109	13570	18217	22122	12602	7891	5654	6404	cdc42
ENSXETG00000004744	2749	115	173	131	109	81	368	319	439	221	190	145	164	ap5b1
ENSXETG00000004745	1621	343	442	326	457	306	484	549	675	383	651	502	568	spout1
ENSXETG00000004746	2805	53	76	75	54	42	101	372	472	225	51	30	48	p3h1
ENSXETG00000004747	1642	477	709	604	938	699	1174	1403	1655	913	572	400	477	atf6
ENSXETG00000004748	2109	5	4	5	112	74	125	616	713	434	219	187	177	olfml2b
ENSXETG00000004749	666	0	0	0	0	0	0	2	4	0	0	0	0	
ENSXETG00000004750	2219	13	16	13	86	65	200	334	368	202	165	104	101	rxrg
ENSXETG00000004751	669	0	0	0	0	0	0	0	0	0	2	0	0	
ENSXETG00000004752	2625	1	5	1	27	22	58	815	1010	614	156	133	147	XB-GENE-983827
ENSXETG00000004753	1718	15	24	19	91	69	70	20	35	17	121	72	79	XB-GENE-5769267
ENSXETG00000004754	1008	1126	1583	1341	868	685	628	272	305	169	304	177	214	hsd17b7
ENSXETG00000004755	867	21	26	31	10	18	55	9	15	9	8	15	7	lrrc52
ENSXETG00000004756	666	43862	64725	53886	25921	19103	24570	14514	16458	9302	18752	13516	14346	mgst3

ENSXETG00000004757	1651	8195	12042	10449	18432	14376	19297	6450	7289	4495	15806	11184	12178	aldh9a1
ENSXETG00000004758	1578	0	0	0	0	0	0	0	0	0	0	0	0	
ENSXETG00000004760	3597	1	1	2	12	9	25	69	89	54	59	38	46	mast1
ENSXETG00000004761	1174	0	0	3	4	2	3	24	35	10	6	5	9	rtbdn
ENSXETG00000004762	1215	435	574	536	1144	892	909	858	938	572	829	626	654	rnaseh2a
ENSXETG00000004763	800	107	206	137	88	69	302	756	948	500	125	73	126	prdx2
ENSXETG00000004765	2166	403	678	563	789	612	974	746	883	559	1239	881	947	hook2
ENSXETG00000004766	924	671	1095	952	3126	2431	11527	5803	6266	4024	5370	3836	4108	junb
ENSXETG00000004767	1040	518	702	673	961	702	895	1506	1707	1043	454	328	395	asna1
ENSXETG00000004768	1460	616	1025	877	1479	1138	1435	2150	2421	1617	1852	1345	1409	c19orf43
ENSXETG00000004769	2112	573	810	703	579	458	1591	1060	1214	698	1044	722	774	XB-GENE-5888819
ENSXETG00000004770	1345	392	585	538	749	594	655	833	939	607	565	357	447	fbxw9
ENSXETG00000004771	2646	353	593	453	1062	895	1648	1649	1818	1118	1204	829	885	tnpo2
ENSXETG00000004772	2637	0	0	0	5	3	3	0	2	3	2	4	2	ENSXETG00000004772
ENSXETG00000004773	5808	2	2	6	2	4	5	8	12	8	4	2	3	col9a2
ENSXETG00000004775	4168	17	29	18	92	75	193	718	791	453	308	181	239	kcnq4
ENSXETG00000004776	1269	322	403	393	695	530	859	2273	2589	1542	764	492	512	smap2
ENSXETG00000004782	684	0	1	1	15	16	30	41	50	34	66	33	43	gdnf
ENSXETG00000004784	3026	177	258	241	406	298	777	593	672	407	566	415	440	wdr70
ENSXETG00000004785	4889	434	583	502	930	716	877	1156	1380	863	826	651	695	nup155
ENSXETG00000004786	465	0	0	0	0	0	0	0	0	1	0	0	0	
ENSXETG00000004787	1182	1544	2139	1863	1885	1458	2546	955	1044	694	587	403	460	slc41a3
ENSXETG00000004788	3381	1	4	12	41	20	21	62	80	49	17	13	13	c5orf42
ENSXETG00000004791	9158	610	856	767	2057	1528	2493	3530	4174	2508	3023	2148	2426	nipbl
ENSXETG00000004792	1419	101	142	136	99	89	150	803	934	510	87	40	59	chst13
ENSXETG00000004794	2877	6615	9718	8317	8556	6423	6703	5761	6734	3965	3987	2815	3061	txnrd3
ENSXETG00000004795	8907	34	50	49	38	26	97	2137	2511	1330	438	321	347	ighx
ENSXETG00000004796	2139	167	218	166	258	184	443	361	393	259	209	132	173	lmbrd2
ENSXETG00000004797	1263	45	82	53	116	74	39	181	197	141	67	65	44	skp2
ENSXETG00000004798	1856	576	846	746	872	699	836	713	801	486	1091	792	949	nadk2
ENSXETG00000004799	2235	0	0	0	1	0	0	1	0	0	0	0	0	ENSXETG00000004799
ENSXETG00000004804	819	5	1	2	25	13	29	22	21	14	36	21	18	fam84b
ENSXETG00000004807	858	0	0	0	0	0	0	0	0	0	0	0	0	
ENSXETG00000004810	4077	0	1	0	0	0	0	81	70	40	2	1	1	
ENSXETG00000004811	2316	76	132	107	225	165	345	471	476	314	295	234	233	rbsn
ENSXETG00000004816	522	537	839	714	433	330	315	428	510	329	229	169	174	mrps25
ENSXETG00000004817	1958	42	42	50	65	47	115	195	205	141	156	97	135	nr2c2
ENSXETG00000004819	4344	20	29	17	214	179	472	650	815	474	534	401	472	fgd5
ENSXETG00000004821	1700	171	230	213	338	241	597	486	545	286	493	357	399	ccdc174
ENSXETG00000004822	5890	301	430	396	794	665	1458	1238	1517	869	1611	1115	1340	kdm6a
ENSXETG00000004828	417	0	0	0	0	0	0	0	0	1	0	0	0	
ENSXETG00000004831	1266	416	564	467	422	359	463	761	862	531	345	257	273	fundc1
ENSXETG00000004834	363	0	0	0	0	1	0	2	7	2	0	0	0	ENSXETG00000004834
ENSXETG00000004835	2417	5	1	4	257	250	17	14	14	6	12	5	10	efhc2
ENSXETG00000004839	1521	500	817	650	301	279	822	405	505	309	441	354	347	maob
ENSXETG00000004842	351	0	0	0	0	0	0	4	1	0	0	0	0	
ENSXETG00000004843	345	0	0	0	1	0	0	2	4	1	2	1	1	ENSXETG00000004843
ENSXETG00000004844	420	0	0	0	0	0	0	4	0	2	1	1	0	
ENSXETG00000004845	2573	173	206	146	382	286	708	723	773	540	675	531	537	braf
ENSXETG00000004847	2602	141	204	150	293	200	279	740	959	539	216	168	169	spidr
ENSXETG00000004849	1971	74	102	77	173	136	656	1534	1789	1032	905	658	787	maoa
ENSXETG00000004850	849	4314	6442	5472	6013	4696	18481	14375	15524	10109	7893	5566	5710	cebpd
ENSXETG00000004851	12249	240	326	270	730	512	555	655	842	498	670	516	523	prkdc
ENSXETG00000004852	4100	777	1189	996	1623	1269	1783	2512	2956	1686	1785	1164	1343	cask
ENSXETG00000004853	593	1142	1717	1495	928	649	1152	1135	1288	830	1096	767	838	mrps33
ENSXETG00000004854	2598	418	624	476	1010	761	172	1409	1552	959	382	260	262	mcm4
ENSXETG00000004856	1288	953	1467	1191	1189	887	1342	1772	2156	1259	1145	820	845	ube2v2
ENSXETG00000004861	750	0	0	0	0	0	0	0	0	0	0	0	0	
ENSXETG00000004863	4710	3	5	3	356	237	9	5	5	2	20	14	12	efcab6
ENSXETG00000004864	855	0	4	3	37	16	28	75	79	48	106	77	73	sult4a1
ENSXETG00000004865	2665	958	1481	1177	1292	970	1505	1168	1366	820	4336	3136	3408	pnpla3
ENSXETG00000004871	1068	1081	1631	1412	2051	1622	1703	1494	1630	1035	1953	1331	1560	samm50
ENSXETG00000004872	1534	18	22	23	53	38	67	559	592	431	61	55	51	parvb
ENSXETG00000004874	888	0	0	0	2	0	0	0	2	0	0	0	2	ENSXETG00000004874
ENSXETG00000004875	3675	170	238	229	410	307	795	556	608	363	851	598	655	usp40
ENSXETG00000004877	5303	32	67	45	474	360	186	566	666	396	221	175	167	traf3ip1
ENSXETG00000004878	614	887	1373	1118	3285	2536	579	3011	3348	2070	2086	1452	1653	ag1
ENSXETG00000004879	2198	242	296	278	438	308	484	350	442	245	451	363	362	asb1
ENSXETG00000004880	3222	8	17	20	60	34	27	74	96	65	110	55	70	hdac4
ENSXETG00000004886	1351	4902	7267	6207	5904	4378	6712	5894	6727	3885	4608	3244	3503	ndufa10
ENSXETG00000004890	2398	330	430	396	346	293	218	3	6	4	65	56	49	ugt1a6
ENSXETG00000004891	846	381	505	445	311	203	376	578	706	394	421	254	348	tma16
ENSXETG00000004893	1894	2	0	2	6	7	15	4	9	9	23	20	15	npy5r
ENSXETG00000004897	1578	42	68	72	59	64	173	92	86	61	86	69	56	ENSXETG00000004897
ENSXETG00000004898	1959	115	170	155	282	218	374	733	743	508	442	329	451	znf347
ENSXETG00000004899	2481	6799	10005	8721	5858	4341	2764	489	510	317	425	289	313	lss
ENSXETG00000004900	1470	0	1	1	0	1	0	4	4	1	2	1	5	CHRNA10
ENSXETG00000004903	1107	0	0	1	2	2	2	4	5	2	8	4	5	NPY1R
ENSXETG00000004904	2884	285	442	336	852	589	897	1157	1204	727	1237	901	947	nup98
ENSXETG00000004906	468	340	533	460	585	422	910	808	970	580	823	526	590	taf10
ENSXETG00000004908	1143	737	1034	864	487	373	1330	402	510	290	459	320	370	b3gnt3
ENSXETG00000004910	3651	123	229	200	410	312	504	1213	1439	852	533	429	473	map1b
ENSXETG00000004912	1823	10	13	6	33	21	32	144	156	106	92	59	65	ENSXETG00000004912
ENSXETG00000004913	4385	74	138	118	182	123	181	242	283	201	244	166	215	dgkq
ENSXETG00000004914	2215	761	1139	900	631	449	2140	1676	1986	1094	293	202	235	mfsd7
ENSXETG00000004916	750	9	12	11	29	12	38	124	128	94	48	29	46	pcgf3
ENSXETG00000004917	2523	0	0	0	0	0	0	0	0	0	0	0	0	
ENSXETG00000004920	2571	0	0	0	0	0	0	0	0	0	0	0	0	
ENSXETG00000004921	954	0	0	0	0	0	0	0	0	0	0	0	0	
ENSXETG00000004924	2361	6	13	19	3	2	22	1	4	3	1	2	2	ENSXETG00000004924
ENSXETG00000004925	2400	0	0	0	0	0	0	0	0	1	0	0	0	ENSXETG00000004925
ENSXETG00000004935	2328	0	0	0	0	0	0	0	0	0	0	0	0	
ENSXETG00000004937	3905	188	323	268	381	268	582	423	496	310	476	301	348	zc3h3
ENSXETG00000004940	12288	0	0	0	0	0	0	0	0	0	0	0	0	
ENSXETG00000004942	1374	29	27	28	31	19	62	518	566	359	60	33	43	ENSXETG00000004942
ENSXETG00000004943	3252	353	477	369	464	400	608	916	1001	579	683	500	549	sh3rf1
ENSXETG00000004944	1599	169	237	230	303	217	422	292	388	184	329	230	240	cbr4
ENSXETG00000004956	9327	271	458	385	502	371	899	1454	1660	999	137	117	123	lama1
ENSXETG00000004957	1845	4	8	10	22	17	25	208	278	149	50	56	56	arhgap28
ENSXETG00000004959	4769	6238	9041	7511	8957	6929	8748	5599	6474	3912	4953	3536	3989	epb41l3
ENSXETG00000004960	4078	60	113	88	178	143	378	835	1084	622	394	315	354	rnf149
ENSXETG00000004961	1563	779	1093	935	1319	963	1162	521	659	355	485	329	388	ddc
ENSXETG00000004962	3020	84	126	111	146	88	288	742	826	452	437	322	331	grb10
ENSXETG00000004971	1905	52	79	66	178	165	407	54	82	45	285	171	231	cobl
ENSXETG00000004974	1122	4	6	5	9	15	48	8	7	5	23	16	21	stac
ENSXETG00000004976	1584	0	0	2	4	5	1	1	3	0	15	6	11	dclk3
ENSXETG00000004981	2210	34	52	40	19	23	132	348	493	279	34	21	21	il1r2
ENSXETG00000004985	2353	109	211	149	128	80	208	326	422	241	188	136	154	MLH1
ENSXETG00000004991	621	1119	1675	1426	1007	782	1883	2277	2745	1642	1254	845	982	chmp5
ENSXETG00000004994	3117	645	973	807	1158	857	1591	1743	2052	1219	1173	780	853	lrrfip2
ENSXETG00000004999	2955	301	396	329	404	328	658	1013	1136	751	688	500	522	tbc1d8b

ENSXETG00000005000	921	78	139	102	115	80	195	185	245	121	115	74	82	prkx
ENSXETG00000005002	1746	194	292	253	421	313	710	108	166	86	348	274	279	arsh
ENSXETG00000005003	1501	340	567	469	372	307	399	523	631	339	1349	889	958	gyg2
ENSXETG00000005005	3781	638	926	816	1936	1477	2498	2140	2432	1452	3664	2749	3013	arhgef16
ENSXETG00000005008	3351	19	42	38	445	344	407	277	303	183	77	57	61	MEGF6
ENSXETG00000005009	831	59242	88729	74935	88287	67990	114834	114253	124763	73824	260503	182957	193486	rpl7a
ENSXETG00000005010	606	180	299	225	182	164	221	732	802	479	308	203	207	med22
ENSXETG00000005012	963	0	0	0	0	0	0	0	0	0	0	0	0	
ENSXETG00000005013	1829	0	2	2	23	12	6	39	51	26	20	9	14	XB-GENE-969944
ENSXETG00000005015	1466	589	672	636	696	545	1392	814	1032	618	773	590	653	tprg1l
ENSXETG00000005016	1719	188	214	188	337	264	256	239	279	175	160	105	139	wrap73
ENSXETG00000005018	1394	304	442	415	600	464	630	743	809	489	843	604	669	surf6
ENSXETG00000005019	1905	0	0	0	29	20	3	7	4	1	4	4	8	tp73
ENSXETG00000005024	891	0	0	0	0	0	0	0	0	0	0	0	1	
ENSXETG00000005025	879	0	0	0	0	0	0	3	0	0	0	0	0	
ENSXETG00000005028	3159	870	1322	1095	2320	1699	3094	1531	1795	1075	3403	2436	2812	per3
ENSXETG00000005030	2923	484	748	649	1121	885	1260	1391	1574	956	1165	795	859	tspyl2
ENSXETG00000005032	834	0	0	0	0	0	0	1	6	0	0	0	0	
ENSXETG00000005033	864	0	0	0	0	0	0	0	0	0	0	0	0	
ENSXETG00000005035	581	76	102	79	70	62	11	197	242	165	17	17	26	XB-GENE-5722383
ENSXETG00000005038	1014	0	0	0	0	0	0	0	0	0	0	0	0	
ENSXETG00000005040	4545	1	3	0	12	10	16	75	89	59	26	19	22	camta1
ENSXETG00000005043	2399	1	5	6	14	10	15	25	19	16	17	7	10	ENSXETG00000005043
ENSXETG00000005046	2439	3	3	7	7	2	7	178	261	171	86	49	72	ENSXETG00000005046
ENSXETG00000005049	2623	0	1	0	10	2	12	22	31	18	12	10	12	phactr3
ENSXETG00000005051	1644	0	1	1	0	1	10	6	14	3	10	8	7	meox2
ENSXETG00000005052	1329	8819	12077	10043	6288	4795	16324	978	1239	712	3837	2712	3092	agmo
ENSXETG00000005053	3884	8	7	14	57	42	271	286	333	189	249	178	215	dgkb
ENSXETG00000005062	4772	185	289	215	1048	799	1075	2114	2647	1484	1431	975	1135	etv1
ENSXETG00000005074	4784	80	128	103	341	252	599	1735	2085	1112	759	530	599	enc1
ENSXETG00000005075	2133	9742	14049	12123	8638	6742	24989	19897	23077	13265	9525	6835	7462	hexb
ENSXETG00000005076	2169	184	268	278	148	125	335	215	279	152	293	219	184	gfm2
ENSXETG00000005077	969	6084	9092	7790	8621	6700	13188	10081	11294	6840	27295	18442	20578	nsa2
ENSXETG00000005079	1848	41	76	55	60	43	68	36	42	29	202	118	153	ENSXETG00000005079
ENSXETG00000005081	1263	8	7	6	172	128	61	73	79	49	280	211	259	gcnt4
ENSXETG00000005082	1200	0	0	0	0	0	0	0	0	0	0	0	0	
ENSXETG00000005083	486	907	1351	1132	880	730	1959	1137	1117	832	1309	908	943	hint2
ENSXETG00000005084	2556	14000	20415	17637	17659	13303	22472	3131	3589	2128	5832	4192	4646	hmgcr
ENSXETG00000005085	2876	1022	1474	1280	2123	1634	3112	3542	3889	2348	2285	1702	1912	col4a3bp
ENSXETG00000005087	2769	78	149	114	151	117	194	316	360	206	150	109	171	polk
ENSXETG00000005089	1176	3	2	3	5	3	3	20	24	16	22	14	11	ar
ENSXETG00000005090	1272	47	81	63	300	235	502	992	1005	691	904	640	702	pacsin3
ENSXETG00000005091	5559	47	74	62	1112	804	774	1278	1392	855	5080	3633	3956	lrp4
ENSXETG00000005092	2379	61	100	82	310	258	115	527	679	391	277	176	204	ophn1
ENSXETG00000005093	3022	1168	1724	1437	1346	1069	2119	1845	2152	1287	1526	1073	1193	yipf6
ENSXETG00000005094	1251	0	0	0	0	0	0	0	0	0	0	0	0	
ENSXETG00000005096	1062	73	110	87	138	105	140	191	240	140	198	157	170	c11orf49
ENSXETG00000005097	3370	798	1249	1115	1343	1078	2073	2589	2775	1825	1333	941	1020	arfgap2
ENSXETG00000005113	948	45	97	88	120	86	118	129	158	107	238	165	181	ipmk
ENSXETG00000005114	1023	1901	2690	2300	2377	1835	3040	1925	2314	1328	2648	1920	2155	cisd1
ENSXETG00000005116	858	217	303	308	292	231	268	490	539	316	322	236	225	tfam
ENSXETG00000005117	2196	1	13	4	85	60	87	436	454	265	124	91	108	bicc1
ENSXETG00000005118	1845	982	1399	1170	837	638	1196	123	121	84	485	311	346	phyhipl
ENSXETG00000005119	1161	73	99	89	191	155	93	133	147	70	174	169	152	fam13c
ENSXETG00000005120	501	0	0	0	7	3	3	0	1	0	8	1	3	ENSXETG00000005120
ENSXETG00000005121	1488	36	41	32	37	19	136	140	155	79	35	33	24	slc16a9
ENSXETG00000005123	1830	117	179	128	215	155	313	304	376	193	340	213	256	reps1
ENSXETG00000005126	1821	0	0	0	0	0	0	1	1	1	0	0	0	rbfox1
ENSXETG00000005127	660	509	704	572	1009	727	2613	1134	1179	745	1688	1209	1263	gata5
ENSXETG00000005142	7780	343	467	390	351	290	561	1355	1641	937	771	553	627	dock10
ENSXETG00000005146	2131	126	170	197	473	369	705	550	695	391	884	601	741	crbn
ENSXETG00000005147	2157	0	0	0	8	9	4	102	128	70	7	7	12	lrrn1-like
ENSXETG00000005149	1249	854	1257	1034	953	688	1383	1233	1417	912	556	418	400	sumf1
ENSXETG00000005150	1965	3	2	4	16	9	24	60	70	42	28	19	23	nyap2
ENSXETG00000005151	708	0	0	0	0	0	0	0	0	0	1	1	1	ENSXETG00000005151
ENSXETG00000005153	8061	60	95	79	212	178	480	1575	1895	1081	397	337	331	itpr1
ENSXETG00000005160	1615	23	25	31	60	38	101	514	562	370	132	74	91	march3
ENSXETG00000005161	1908	797	1150	1029	992	734	1478	1217	1520	880	1089	728	749	crat.2
ENSXETG00000005162	2549	755	1108	929	1883	1457	1147	3772	4367	2626	1597	1174	1248	lmnb1
ENSXETG00000005163	2338	3	3	3	28	21	25	78	115	65	43	22	31	prrt1
ENSXETG00000005164	1500	374	594	425	1110	815	1387	1158	1313	737	1576	1124	1305	atf6b
ENSXETG00000005165	1929	450	651	522	766	594	779	132	172	116	333	216	220	stra6
ENSXETG00000005167	1515	142	223	192	1404	1074	1541	2271	2479	1627	2596	1866	2021	bace2
ENSXETG00000005168	5085	2	5	1	16	18	9	229	261	179	378	266	294	tnxb
ENSXETG00000005172	6154	16	21	15	212	164	25	18	14	9	32	18	43	ccdc33
ENSXETG00000005176	1518	0	1	0	3	0	6	25	31	10	6	3	3	cyp11a1
ENSXETG00000005177	1980	13	10	8	17	10	11	83	94	54	31	21	24	mx1
ENSXETG00000005180	2614	1639	2430	1997	2007	1601	2740	1453	1618	924	2670	1918	2140	fam3b
ENSXETG00000005182	1427	2	0	4	7	5	7	16	23	11	18	16	16	c15orf59
ENSXETG00000005183	1987	68	102	92	156	102	247	1075	1176	629	217	147	163	cd276
ENSXETG00000005185	7426	188	259	227	948	649	834	1843	2081	1230	628	456	503	mki67
ENSXETG00000005187	1542	0	2	2	1	0	3	3	0	0	1	2	1	cyp21a2.1
ENSXETG00000005188	2546	3214	4529	3800	3489	2781	5384	7427	9112	4980	6662	4622	5363	nptn
ENSXETG00000005189	1188	312	492	432	489	385	586	887	951	600	439	300	315	phax
ENSXETG00000005190	2073	218	338	275	463	313	506	918	1114	629	861	613	672	ptpre
ENSXETG00000005191	1626	634	834	753	1245	986	1994	1520	1767	1030	2720	1900	2155	aldh7a1
ENSXETG00000005192	2487	1	0	0	3	2	1	2	3	1	2	2	2	hcn4
ENSXETG00000005193	1378	748	989	875	812	660	1498	498	629	355	455	348	399	gramd3
ENSXETG00000005195	4525	481	659	537	1211	957	1441	2453	2723	1658	2022	1345	1551	neo1
ENSXETG00000005197	1402	137	277	193	266	207	485	497	634	405	299	234	230	cenpa
ENSXETG00000005198	840	68	134	111	81	64	60	1865	2067	1261	232	178	200	psmb7.2
ENSXETG00000005199	1458	0	0	0	0	0	0	0	0	0	0	0	0	
ENSXETG00000005200	1177	26	48	45	65	55	79	103	120	82	62	63	61	bbs4
ENSXETG00000005203	5131	27	43	35	202	149	255	375	406	204	138	111	117	c4a
ENSXETG00000005204	5502	264	418	320	765	602	1271	1773	1986	1215	1407	1013	1117	dock1
ENSXETG00000005205	2809	19	33	25	24	17	24	208	233	134	92	54	60	parp6
ENSXETG00000005206	2307	2601	3634	3095	2381	1721	2262	6103	7069	4287	2064	1451	1702	pkm
ENSXETG00000005207	1839	1031	1580	1400	2096	1645	3908	5320	5970	3415	3368	2320	2577	bhlhe40
ENSXETG00000005208	1289	58	84	58	55	42	126	115	144	77	88	57	65	senp8
ENSXETG00000005211	8423	164	253	230	791	616	1050	1881	2126	1269	2060	1438	1692	myo9a
ENSXETG00000005212	561	58	61	66	80	69	107	214	210	141	109	71	93	arl8b
ENSXETG00000005213	1437	0	0	1	0	0	0	0	2	0	0	3	2	fam196a
ENSXETG00000005214	303	28	34	41	227	156	176	91	119	75	145	107	117	ENSXETG00000005214
ENSXETG00000005217	1785	805	1219	1043	1023	809	1038	917	1076	642	547	449	412	edem1
ENSXETG00000005219	1233	1	6	6	9	9	2	0	1	0	6	2	1	nr2e3
ENSXETG00000005220	1208	9	10	15	59	63	49	145	152	112	331	234	231	egfl8
ENSXETG00000005221	11277	329	460	457	1224	985	1531	1316	1569	948	2152	1574	1771	VPS13C
ENSXETG00000005222	2510	967	1398	1181	736	620	1126	1346	1590	893	803	529	618	agpat1
ENSXETG00000005223	4235	487	711	590	1287	1019	1308	2138	2528	1491	1753	1299	1404	pbx2
ENSXETG00000005224	1212	15	14	8	63	52	22	32	37	17	104	63	78	ENSXETG00000005224
ENSXETG00000005225	813	1	2	0	0	0	0	15	37	13	5	6	4	ENSXETG00000005225

ENSXETG00000005227	4356	29	34	24	86	58	113	352	444	222	196	136	169	camsap2
ENSXETG00000005228	1512	261	386	321	311	221	439	299	368	237	450	327	336	clcc1
ENSXETG00000005230	1965	95	108	119	290	220	329	564	590	350	208	147	179	gpsm2
ENSXETG00000005233	2208	0	0	0	1	1	0	0	0	2	1	2	2	fndc7
ENSXETG00000005234	2985	1126	1427	1250	1467	1047	2066	1798	2423	1325	1325	890	1057	stxbp3
ENSXETG00000005239	1080	936	1234	1082	969	704	1433	1787	2088	1273	1130	793	892	ptp4a2
ENSXETG00000005240	1050	0	2	0	59	55	5	1	1	1	2	2	0	dcdc2b
ENSXETG00000005241	2501	350	503	399	919	720	1044	1300	1518	888	905	666	708	txlna
ENSXETG00000005242	2626	1152	1681	1390	1158	879	1425	1867	2126	1225	1098	776	821	kpna6
ENSXETG00000005243	1455	278	371	342	517	373	796	594	633	399	825	534	622	tmem39b
ENSXETG00000005246	1584	2678	4060	3433	5273	3917	5687	9830	11234	6706	5355	3724	4194	khdrbs1
ENSXETG00000005247	1749	7446	10739	9136	11652	8843	17109	12318	14206	8540	32528	22938	25215	eif3i
ENSXETG00000005248	3465	66	85	69	76	63	199	593	639	394	249	195	196	XB-GENE-5960520
ENSXETG00000005249	1363	73	101	75	229	161	404	715	887	503	403	280	290	fam167b
ENSXETG00000005250	1530	26	39	28	19	19	62	219	277	169	131	106	101	lck
ENSXETG00000005251	1721	556	800	638	712	532	1142	791	852	535	426	298	353	tor2a
ENSXETG00000005253	2228	2327	3475	2929	3020	2360	2971	7136	8485	4743	2525	1825	1959	hdac1
ENSXETG00000005254	2401	1301	1950	1628	2135	1598	3480	2639	3030	1777	2945	2133	2393	bsdc1
ENSXETG00000005257	1001	1068	1533	1298	1129	873	1251	1371	1564	969	836	581	686	mrps15
ENSXETG00000005258	1053	14	22	17	70	62	31	90	114	48	39	24	29	oscp1
ENSXETG00000005259	1755	272	414	322	233	156	512	317	359	214	389	274	345	stk40
ENSXETG00000005260	1556	333	440	415	456	351	745	930	998	609	801	541	618	prpf38b
ENSXETG00000005261	2397	210	308	233	1228	1020	1800	3468	3853	2314	594	396	531	cfh
ENSXETG00000005266	2400	652	880	757	925	670	2959	1972	2506	1341	1189	793	956	rragc
ENSXETG00000005268	1563	0	0	0	0	0	0	0	0	0	0	0	0	
ENSXETG00000005270	1517	15	18	15	22	21	18	132	143	76	34	30	25	rhbdl2
ENSXETG00000005271	2029	362	551	500	531	374	592	891	1041	607	396	304	350	akirin1
ENSXETG00000005272	1041	8	12	6	2	2	6	50	66	25	18	8	17	cnr2
ENSXETG00000005273	4073	6049	8706	7178	8816	6844	14911	13082	15473	8554	9800	7043	7693	pnrc2
ENSXETG00000005274	417	24	41	33	77	50	1	2	3	1	6	6	15	ENSXETG00000005274
ENSXETG00000005275	3753	4	15	9	43	33	20	4	10	4	42	41	43	myom3
ENSXETG00000005283	5864	591	901	723	2349	1805	2903	3113	3437	2175	2388	1714	1960	fam83h
ENSXETG00000005284	465	12	12	14	19	18	30	212	188	166	57	34	57	ENSXETG00000005284
ENSXETG00000005286	741	1	0	2	1	1	1	2	10	1	1	0	0	foxh1.2
ENSXETG00000005288	996	2	0	1	2	2	3	0	0	0	0	0	0	ENSXETG00000005288
ENSXETG00000005296	1290	84	147	135	195	128	275	220	268	154	118	83	80	ENSXETG00000005296
ENSXETG00000005300	4485	4	4	2	42	27	40	234	226	167	106	70	84	rusc2
ENSXETG00000005302	3456	4323	6211	5616	12966	9826	19041	8871	9464	6164	23774	17044	18620	ENSXETG00000005302
ENSXETG00000005305	1248	0	0	0	0	0	0	2	0	1	0	0	0	
ENSXETG00000005309	2172	0	3	1	4	6	8	21	27	9	16	11	7	lrfn1
ENSXETG00000005310	1743	0	1	2	0	0	2	14	16	7	7	3	2	ENSXETG00000005310
ENSXETG00000005311	1618	554	837	633	1299	1006	2082	2636	2956	1773	1615	1122	1278	dcaf7
ENSXETG00000005312	3339	6	1	2	25	19	20	27	20	9	38	26	31	kcnh6
ENSXETG00000005314	5650	463	575	487	804	611	1304	1328	1765	1037	1059	739	854	pak4
ENSXETG00000005315	4216	134922	198217	171764	212890	163719	506365	19018	21913	12844	233020	163807	182706	ace
ENSXETG00000005318	402	0	0	0	0	0	0	0	3	0	1	0	0	
ENSXETG00000005319	681	28	52	26	87	59	67	134	144	87	231	152	149	nccrp1
ENSXETG00000005323	3132	77	93	88	180	131	265	378	469	264	307	208	256	rest
ENSXETG00000005325	1382	691	982	808	1223	885	1651	2221	2484	1522	1836	1342	1513	cyb561
ENSXETG00000005326	1119	1006	1495	1337	849	601	949	1109	1212	741	677	490	556	mrps12
ENSXETG00000005328	4035	22	34	19	139	104	173	597	704	429	323	215	266	tanc2
ENSXETG00000005329	3640	547	772	664	1194	935	1644	2121	2641	1410	1246	858	915	polr2b
ENSXETG00000005331	4613	73	116	95	413	277	599	2302	2516	1564	392	261	280	mrc2
ENSXETG00000005332	2236	766	1103	920	966	787	1235	1272	1552	938	1150	790	873	sars2
ENSXETG00000005334	1778	540	808	676	1813	1362	2151	1414	1669	924	3033	2175	2419	XB-GENE-5961850
ENSXETG00000005336	438	1	1	2	72	40	5	0	0	0	6	6	5	ENSXETG00000005336
ENSXETG00000005337	582	0	0	0	0	0	0	0	0	0	0	0	0	
ENSXETG00000005339	888	124	173	132	74	52	145	61	57	34	77	43	53	ankrd9
ENSXETG00000005341	2682	0	0	0	19	13	3	0	0	0	1	3	1	cfap47
ENSXETG00000005342	2918	10	13	13	82	62	138	684	804	486	384	254	292	arhgap6
ENSXETG00000005343	875	30	42	30	29	15	82	204	207	122	183	101	136	amelx
ENSXETG00000005344	3908	0	0	0	16	9	87	97	97	67	26	18	28	frmpd4
ENSXETG00000005345	837	157	221	199	244	211	612	319	381	203	536	362	443	prps2
ENSXETG00000005347	626	41489	59557	50825	25684	19978	44920	93067	103527	59899	40444	27812	31309	tmsb4x
ENSXETG00000005348	1632	118	158	127	146	104	196	178	268	142	230	159	186	noa1
ENSXETG00000005350	5822	270	386	325	627	445	1152	3196	3671	2197	1611	1124	1356	ptprs
ENSXETG00000005352	1426	56	100	110	123	81	210	234	281	179	241	164	186	kdm4b
ENSXETG00000005356	7578	17	29	24	261	191	644	950	1139	681	320	218	264	stab2
ENSXETG00000005357	1281	194	249	208	237	156	271	622	790	492	369	264	305	nt5dc3
ENSXETG00000005358	3297	50	92	62	87	78	116	151	174	109	79	53	79	ENSXETG00000005358
ENSXETG00000005359	3128	47986	68015	58165	14758	11291	6899	16119	19542	11555	3578	2548	3012	hsp90b1
ENSXETG00000005360	1527	2431	3562	2978	2943	2162	1962	2992	3542	2013	2064	1362	1615	cyp3a5
ENSXETG00000005361	2064	30	26	36	61	61	174	87	125	54	196	142	159	tcaim
ENSXETG00000005362	2445	9	8	8	34	18	23	31	39	15	58	31	53	trim71
ENSXETG00000005363	2643	563	766	706	953	773	964	1029	1348	700	722	524	601	cnot10
ENSXETG00000005364	2341	817	1176	1000	1312	993	1576	1416	1636	952	943	598	683	dync1li1
ENSXETG00000005367	1389	0	0	0	7	11	2	72	93	72	3	2	2	ENSXETG00000005367
ENSXETG00000005368	1487	1	1	2	42	30	6	1	0	0	7	8	5	c6orf118
ENSXETG00000005369	2391	0	3	3	11	8	13	94	87	61	47	27	33	pde10a
ENSXETG00000005370	1306	1	0	1	6	7	0	0	3	0	5	3	5	t
ENSXETG00000005371	2505	0	0	0	0	0	0	0	0	0	0	0	0	
ENSXETG00000005376	2328	0	0	0	0	0	0	0	0	0	0	0	0	
ENSXETG00000005379	921	0	0	0	0	0	0	0	0	0	0	0	0	
ENSXETG00000005380	1371	0	0	0	5	2	4	7	10	4	7	6	12	cnr1
ENSXETG00000005381	2617	348	514	419	564	431	557	1144	1390	761	580	383	449	pycr1
ENSXETG00000005384	3311	142	242	170	342	283	343	660	745	406	659	509	550	rngtt
ENSXETG00000005385	1847	500	761	593	474	363	602	1093	1283	835	717	518	610	srsf12
ENSXETG00000005386	492	5	4	4	10	2	8	10	24	9	10	8	5	mafg
ENSXETG00000005387	1332	543	779	580	780	566	1120	2018	2415	1429	753	523	539	pm20d2
ENSXETG00000005388	1375	291	422	384	737	604	1136	739	814	523	1092	796	881	sirt7
ENSXETG00000005389	1628	7925	11777	10020	5690	4472	8552	2790	3259	1931	6978	4909	5309	pcyt2
ENSXETG00000005391	1009	1835	2562	2397	2813	2146	2465	6394	7262	4554	1982	1356	1499	alyref
ENSXETG00000005392	1287	0	0	0	0	0	1	2	0	0	5	1	3	ENSXETG00000005392
ENSXETG00000005394	2487	0	0	0	0	0	0	0	0	0	0	0	0	
ENSXETG00000005400	1754	40	50	51	153	107	162	426	508	281	196	134	143	gpc4
ENSXETG00000005401	1352	64	60	51	68	72	55	112	132	92	38	40	19	suv39h2
ENSXETG00000005403	2082	111	199	139	166	136	218	242	266	175	442	335	339	dclre1c
ENSXETG00000005405	2853	24444	35048	30635	19361	14884	11215	18117	21286	12227	9807	6880	7865	p4hb
ENSXETG00000005408	735	6	0	5	4	0	5	18	36	26	14	7	9	ENSXETG00000005408
ENSXETG00000005409	1140	15	28	19	31	39	34	124	119	73	78	78	65	mbnl3
ENSXETG00000005411	473	36	43	46	131	108	64	90	120	51	28	14	13	meig1
ENSXETG00000005412	2271	21	55	50	100	69	131	131	139	88	125	94	121	dmtf1
ENSXETG00000005415	1178	282	438	385	287	181	313	640	785	405	244	174	191	rap2c
ENSXETG00000005416	2181	15	11	9	11	11	2	12	18	14	31	18	15	frmd7
ENSXETG00000005417	3165	14	16	30	440	362	82	156	194	91	629	358	498	kiaa1324l
ENSXETG00000005418	1269	819	1207	1056	1044	828	1521	1809	2187	1312	1331	868	1033	stk26
ENSXETG00000005419	450	1	0	0	0	3	2	5	6	0	1	1	1	ppp1r27
ENSXETG00000005420	1274	556	841	641	791	576	1272	1301	1592	967	1331	892	994	mcrip1
ENSXETG00000005421	1452	509	718	605	610	466	950	690	793	490	1496	1073	1151	gcgr
ENSXETG00000005422	3285	24	42	33	127	103	123	257	294	184	187	144	158	ret
ENSXETG00000005423	1359	316	418	341	296	265	1046	1081	1447	778	1626	1096	1235	ENSXETG00000005423

ENSXETG00000005426	2643	0	0	0	0	0	0	0	0	0	0	0	0	
ENSXETG00000005430	3255	14	21	28	71	52	114	226	328	159	89	62	49	slc12a4
ENSXETG00000005431	2340	16	15	11	177	105	147	730	897	532	317	219	239	sema3d
ENSXETG00000005433	1116	10	26	17	24	18	99	149	202	117	89	82	97	dpep2
ENSXETG00000005435	1506	84	115	113	140	86	188	220	279	168	184	117	147	ddx28
ENSXETG00000005436	1491	93	129	108	109	82	274	226	257	133	224	153	199	dus2
ENSXETG00000005438	480	1	1	3	3	5	11	4	5	6	44	20	22	ENSXETG00000005438
ENSXETG00000005440	1975	0	0	0	5	3	3	4	11	3	9	3	5	tcerg1l
ENSXETG00000005442	1459	614	807	707	972	763	1416	1262	1460	878	1106	821	979	ppp2r2d
ENSXETG00000005443	2532	0	0	0	25	13	13	23	17	11	35	27	32	jakmip3
ENSXETG00000005447	747	0	0	0	0	0	0	7	3	2	0	0	0	
ENSXETG00000005449	1339	1519	2228	1842	1894	1417	2502	3689	4089	2366	2200	1500	1802	ostf1
ENSXETG00000005456	456	0	0	0	0	0	0	0	0	0	0	0	0	
ENSXETG00000005457	444	0	0	0	0	0	0	0	0	0	0	0	0	
ENSXETG00000005459	732	0	0	0	1	0	0	0	0	0	0	0	0	
ENSXETG00000005462	1637	0	0	0	1	0	0	0	0	0	0	0	0	dmrta1
ENSXETG00000005465	1495	8	17	7	32	18	91	149	165	100	41	22	28	cdkn2b
ENSXETG00000005467	1188	550	781	663	716	579	1071	971	1153	676	726	535	628	mtap
ENSXETG00000005468	1989	0	0	0	0	0	0	0	0	1	0	0	0	
ENSXETG00000005469	1179	2732	4017	3280	1835	1374	2247	2902	3321	1887	1334	887	1005	yipf5
ENSXETG00000005472	2582	158	224	207	188	159	174	321	406	223	133	103	142	lrwd1
ENSXETG00000005474	885	79	116	99	79	88	193	182	217	132	209	118	145	alkbh4
ENSXETG00000005475	1878	4	8	10	52	33	85	241	267	155	46	42	42	sh2b2
ENSXETG00000005476	4389	341	452	402	462	302	856	1159	1406	884	742	489	569	cux1
ENSXETG00000005477	1506	2644	3935	3449	3990	3115	4266	5844	6573	3811	3242	2315	2505	ubxn1
ENSXETG00000005478	984	907	1206	1089	514	388	384	739	913	471	511	384	426	lbhd1
ENSXETG00000005479	2766	144	209	178	442	310	525	605	632	378	590	404	445	ints5
ENSXETG00000005480	1029	0	0	0	0	0	0	1	6	2	16	9	11	angptl5
ENSXETG00000005481	2634	0	0	0	2	4	16	780	849	525	71	54	74	trpc6
ENSXETG00000005482	2196	0	0	0	1	1	6	34	57	31	52	25	27	pgr
ENSXETG00000005484	2194	145	211	170	178	130	300	1141	1378	728	234	149	171	emc3
ENSXETG00000005485	591	0	0	0	1	0	1	1	2	3	2	3	2	fancd2os
ENSXETG00000005487	2490	104	143	110	354	242	288	517	599	346	365	245	290	ARHGAP42
ENSXETG00000005492	3312	0	0	0	0	0	5	43	35	29	16	7	12	cntn5
ENSXETG00000005493	4404	500	729	641	1282	967	1601	2409	2689	1697	1708	1202	1292	gapvd1
ENSXETG00000005495	1740	1	0	2	2	1	0	2	2	2	2	0	2	hgfac
ENSXETG00000005498	1524	0	0	0	0	0	0	2	4	4	0	1	0	
ENSXETG00000005500	1577	315	478	419	171	139	861	2070	2421	1335	356	268	300	lrpap1
ENSXETG00000005504	1974	60	59	58	74	45	63	155	152	82	111	74	83	slc7a1
ENSXETG00000005505	627	104	172	137	533	411	570	524	599	390	417	298	333	MRAS
ENSXETG00000005513	3249	184	279	207	717	584	527	2279	2710	1584	240	192	206	lrrn4
ENSXETG00000005516	1668	0	0	0	2	0	0	6	4	4	0	1	2	cntnap5
ENSXETG00000005517	2034	567	931	718	1843	1480	2738	3979	4695	2604	2734	1927	2173	fermt1
ENSXETG00000005518	1110	0	1	1	7	9	7	64	56	31	23	7	13	mtus2
ENSXETG00000005519	2643	4	11	9	32	30	42	159	157	102	51	42	47	bmp2
ENSXETG00000005520	1107	0	0	0	0	0	0	0	1	0	0	0	0	
ENSXETG00000005523	861	0	1	0	2	0	0	1	0	1	3	1	2	
ENSXETG00000005524	3792	125	210	181	313	225	494	146	190	96	420	265	319	plcb1
ENSXETG00000005526	2768	1120	1496	1267	1284	930	1301	2644	3302	1878	2826	2013	2227	slc7a3
ENSXETG00000005528	2978	199	305	246	640	484	759	643	852	483	364	248	270	nde1
ENSXETG00000005529	7125	562	831	729	1188	785	2836	2287	2774	1627	2706	1920	2297	kiaa0430
ENSXETG00000005532	683	0	1	0	0	2	0	2	6	1	1	0	0	c16orf45
ENSXETG00000005533	2364	2208	3305	2834	2930	2211	3055	2476	2740	1647	2661	1780	1943	pdxdc1
ENSXETG00000005536	1917	385	569	466	506	363	793	377	510	265	863	617	730	rrn3
ENSXETG00000005539	873	215	331	326	288	201	366	555	634	338	346	204	270	thumpd1
ENSXETG00000005540	1913	2092	3068	2642	2046	1574	2142	2909	3429	2109	1827	1304	1512	gspt1
ENSXETG00000005543	1074	15	23	12	39	25	207	149	189	115	89	61	56	snx29
ENSXETG00000005547	654	0	0	0	0	0	0	0	0	0	0	0	0	
ENSXETG00000005552	2889	726	1093	883	862	724	505	789	907	508	1104	786	840	kif4a
ENSXETG00000005555	948	121	165	173	294	239	412	397	432	262	430	313	333	c2cd5
ENSXETG00000005556	3457	763	1058	918	2287	1668	2690	1774	2021	1202	2273	1624	1688	cc2d1a
ENSXETG00000005560	1728	62	106	81	247	185	381	320	344	213	166	80	114	ghr
ENSXETG00000005567	2700	128	207	147	306	223	653	632	679	440	777	556	610	rfx1
ENSXETG00000005569	1960	651	989	855	770	597	1446	1425	1595	941	716	481	564	fbxo4
ENSXETG00000005570	1712	190	290	265	556	384	556	772	818	570	509	358	358	dcaf15
ENSXETG00000005574	1800	253	359	310	1431	1085	1289	1307	1458	884	1865	1357	1480	ENSXETG00000005574
ENSXETG00000005576	1235	1038	1492	1374	1169	813	957	1418	1555	883	705	521	532	clpp
ENSXETG00000005578	618	43	83	72	79	38	72	80	78	53	50	30	27	alkbh7
ENSXETG00000005579	2437	554	831	718	1008	777	866	1900	2044	1336	677	532	504	gtf2f1
ENSXETG00000005580	2823	2806	4078	3575	4394	3374	4059	3865	4356	2631	1991	1411	1618	ganab
ENSXETG00000005581	1797	160	218	198	135	117	169	484	615	374	228	176	209	b3gat3
ENSXETG00000005582	1044	1	1	1	0	0	1	8	1	3	1	0	1	rom1
ENSXETG00000005583	1822	3164	4485	3741	2662	2023	2345	1675	1888	1087	1516	1108	1223	surf4.1
ENSXETG00000005585	6900	5	18	11	17	9	20	27	38	28	49	21	30	cacna1a
ENSXETG00000005586	1704	0	0	0	0	0	0	0	0	0	0	0	0	
ENSXETG00000005589	1247	324	419	359	371	269	528	1104	1173	743	623	436	489	stx10
ENSXETG00000005590	891	0	0	0	0	0	0	0	0	0	0	0	0	
ENSXETG00000005594	1431	45	49	49	115	91	221	525	531	344	324	267	248	nacc1
ENSXETG00000005595	5441	158	208	246	1594	1175	3270	2894	3053	1887	3827	2675	3048	tns1
ENSXETG00000005597	3146	1349	2008	1677	2277	1730	6669	8661	9830	5662	5725	3961	4508	man2b1
ENSXETG00000005598	1146	498	738	631	709	542	1071	1084	1174	734	1020	680	718	wdr83
ENSXETG00000005599	1424	658	982	851	470	332	624	1022	1196	786	587	416	433	dhps
ENSXETG00000005600	894	0	0	0	0	0	0	0	1	0	0	0	0	pou6f2
ENSXETG00000005611	1098	159	272	233	2227	1661	513	263	240	181	2992	2253	2373	krt18
ENSXETG00000005613	3057	19	27	24	66	49	69	114	134	75	116	80	71	ogdhl
ENSXETG00000005614	3658	221	283	253	511	382	551	428	561	327	790	556	603	aasdh
ENSXETG00000005615	918	191	330	305	649	488	473	1043	1164	707	1010	695	799	paics.1
ENSXETG00000005618	2000	126	188	159	216	150	210	701	806	491	417	298	317	ppat
ENSXETG00000005620	1980	327	471	450	2330	1837	1667	816	905	584	843	590	613	LONRF1
ENSXETG00000005622	1329	6	6	6	5	3	12	28	29	21	4	3	6	kiaa1456l
ENSXETG00000005624	1872	0	0	0	4	2	6	26	26	17	34	20	28	chat
ENSXETG00000005627	750	1	1	2	7	4	10	7	8	4	3	1	6	rgr
ENSXETG00000005628	1980	266	436	376	822	575	819	1414	1556	954	1581	1098	1295	eml3
ENSXETG00000005630	3276	21	32	33	218	175	289	1083	1195	736	389	284	322	dlc1
ENSXETG00000005631	1713	0	0	0	0	0	0	0	0	0	0	0	0	
ENSXETG00000005632	2535	0	0	0	1	0	0	0	0	1	0	0	0	
ENSXETG00000005633	2001	787	1103	989	2727	2016	4288	3734	4205	2456	7142	5203	5578	mta2
ENSXETG00000005634	1038	1048	1588	1315	1605	1151	1637	1216	1388	808	1012	696	737	ghitm
ENSXETG00000005637	2962	0	0	0	1	4	2	5	0	3	3	1	1	nrg3
ENSXETG00000005639	1032	44	58	59	40	38	84	153	203	112	107	70	82	ENSXETG00000005639
ENSXETG00000005640	2641	450	638	557	643	500	1087	932	1058	654	2539	1775	1853	tut1
ENSXETG00000005641	888	0	0	0	97	71	171	1	1	0	108	68	75	sgcz
ENSXETG00000005642	1864	9	9	11	61	49	60	185	180	112	162	108	105	ahnak
ENSXETG00000005647	2770	547	760	634	724	552	1624	542	679	401	1119	740	900	tmem68
ENSXETG00000005648	2550	323	473	418	708	515	882	813	947	529	918	671	734	tgs1
ENSXETG00000005650	1737	765	1098	950	959	821	1895	1956	2236	1448	1937	1328	1540	lyn
ENSXETG00000005652	516	58812	86312	76542	68382	54159	82947	74154	82891	51534	147893	104587	112507	rps20
ENSXETG00000005653	1059	2	1	3	8	2	5	34	27	19	7	8	18	mos
ENSXETG00000005654	1500	7	3	6	32	19	44	20	32	30	43	26	31	plag1
ENSXETG00000005655	1838	8374	12130	10380	7160	5435	11085	1269	1537	897	5030	3719	4040	sdr16c5
ENSXETG00000005657	1357	900	1303	1078	2042	1560	1887	1286	1493	886	1784	1223	1374	penk

ENSXETG00000005659	1709	835	1227	956	1437	1113	1774	2417	2839	1691	1142	767	946	impad1
ENSXETG00000005660	1113	8	15	10	39	19	82	423	489	242	73	56	54	fam110b
ENSXETG00000005661	1545	1820	2662	2234	1472	1122	10214	3	5	2	2171	1484	1685	cyp7a1
ENSXETG00000005662	591	110	154	127	249	158	321	412	496	272	256	167	179	ENSXETG00000005662
ENSXETG00000005663	2030	3740	5344	4446	4091	3061	8099	15933	18876	10757	5669	3967	4546	sdcbp
ENSXETG00000005667	1566	2	0	2	3	2	14	7	13	10	3	1	6	sntg1
ENSXETG00000005672	2268	287	370	339	547	440	669	668	833	434	594	463	484	pcmtd1
ENSXETG00000005673	3078	2	3	3	12	9	20	41	39	43	32	22	36	st18
ENSXETG00000005674	1312	194	260	189	99	85	309	1072	1366	769	441	368	374	rgs1
ENSXETG00000005675	1676	1001	1331	1137	1573	1174	4120	2483	2840	1623	2602	1876	2160	rgs2
ENSXETG00000005678	4962	243	344	235	792	574	1476	1049	1368	766	1020	754	863	RB1CC1
ENSXETG00000005682	909	0	0	0	1	0	1	38	53	20	0	0	1	
ENSXETG00000005683	804	6	11	9	33	19	15	99	98	59	67	51	61	ENSXETG00000005683
ENSXETG00000005684	1185	257	389	375	152	120	120	292	320	185	217	133	136	abt1
ENSXETG00000005685	5029	93	163	130	391	257	1068	953	1067	652	728	522	542	mroh1
ENSXETG00000005692	555	2	2	0	1	7	6	75	96	58	11	3	7	scx
ENSXETG00000005693	1611	147	230	222	444	351	573	711	776	492	585	354	432	hsf1
ENSXETG00000005702	1306	522	797	742	826	663	678	1779	1980	1236	868	638	641	rpia
ENSXETG00000005704	1881	0	0	0	0	0	0	0	0	0	0	0	0	
ENSXETG00000005714	453	181647	265880	244323	146248	112191	104116	34706	39664	25776	18441	13070	14465	fabp6
ENSXETG00000005717	2658	134	229	177	456	349	554	719	841	530	726	536	586	pwwp2a
ENSXETG00000005720	1696	80	91	60	106	101	143	713	905	500	143	84	117	ikbip
ENSXETG00000005721	3470	336	473	408	567	437	507	818	840	509	592	443	464	polr3b
ENSXETG00000005727	1149	1	0	0	13	7	40	356	392	232	71	66	64	c1qtnf1
ENSXETG00000005729	414	1	0	1	3	2	1	5	7	4	0	1	0	ENSXETG00000005729
ENSXETG00000005730	936	4	9	5	43	38	75	522	641	401	104	49	57	ENSXETG00000005730
ENSXETG00000005731	1101	0	3	1	1	0	6	11	14	6	2	1	3	sstr5
ENSXETG00000005733	468	259	392	353	316	217	219	153	182	92	325	196	215	slc25a34
ENSXETG00000005734	1809	1825	2637	2207	1669	1373	1673	1325	1523	942	760	552	618	pemt
ENSXETG00000005738	1366	185	226	203	382	278	1144	1313	1487	921	654	458	498	rasd1
ENSXETG00000005739	1680	57	78	66	220	144	326	278	342	202	587	434	465	klf4
ENSXETG00000005740	444	495	725	584	624	451	651	1015	1064	681	608	423	453	med9
ENSXETG00000005741	522	104	139	113	296	208	543	245	291	159	392	307	319	nt5m
ENSXETG00000005742	1604	1547	2200	1934	1964	1546	2364	2878	3512	1979	1703	1220	1370	cops3
ENSXETG00000005745	2117	232	368	291	780	608	2757	1589	1869	1098	1364	1011	1062	flcn
ENSXETG00000005746	1401	0	0	2	1	0	1	30	27	22	19	11	13	sox8
ENSXETG00000005747	881	1	1	0	3	5	4	12	21	10	6	5	4	pld6
ENSXETG00000005751	2135	2387	3520	2938	4307	3203	5069	5318	5855	3502	3617	2639	2844	rad23b
ENSXETG00000005752	414	0	0	0	0	0	0	1	0	0	0	0	0	
ENSXETG00000005754	1458	3695	5529	4824	2512	1934	5272	1087	1169	762	1780	1253	1319	shmt1
ENSXETG00000005757	1551	816	1323	1104	1132	874	1667	1362	1357	828	1257	911	986	sntb1
ENSXETG00000005758	1620	42	40	34	69	51	60	114	130	64	77	47	74	ENSXETG00000005758
ENSXETG00000005759	5145	257	402	317	1140	861	1186	1321	1648	831	1578	1070	1318	znf462
ENSXETG00000005761	5325	9	12	14	158	119	118	366	454	259	237	170	177	col14a1
ENSXETG00000005762	1050	68	107	75	152	99	167	198	235	153	150	105	113	lmf1
ENSXETG00000005764	1110	47	57	68	345	267	816	145	162	81	349	275	305	XB-GENE-963065
ENSXETG00000005765	3297	119	188	160	293	242	255	532	579	325	338	272	262	top3a
ENSXETG00000005766	2596	306	424	346	979	709	1039	532	629	360	1041	730	818	mief2
ENSXETG00000005767	5392	1060	1493	1262	3175	2461	4207	5162	5433	3397	3388	2465	2666	flii
ENSXETG00000005768	3195	64	109	71	409	315	451	1602	1736	1078	611	488	461	llgl1
ENSXETG00000005769	1068	1330	1952	1662	2164	1699	2578	2109	2363	1536	2594	1848	1870	alkbh5
ENSXETG00000005771	579	76	88	92	91	91	104	129	126	76	80	67	80	gfer
ENSXETG00000005773	2414	114	173	149	85	45	251	18568	21648	13182	53	50	58	noxo1.1
ENSXETG00000005775	1448	30	59	47	71	57	82	101	144	87	71	68	76	gng13
ENSXETG00000005776	2934	168	282	227	548	431	200	694	746	468	167	93	135	chtf18
ENSXETG00000005780	2146	909	1413	1136	3442	2609	3285	4126	4682	2828	3577	2498	2731	cxadr
ENSXETG00000005781	750	139	202	163	319	259	127	374	470	264	212	150	170	btg3
ENSXETG00000005782	2605	0	1	0	2	10	6	48	57	52	29	33	36	col8a1
ENSXETG00000005783	2130	132	195	169	493	362	883	718	845	555	665	510	605	dcbld2
ENSXETG00000005787	1902	540	770	607	896	694	1409	1406	1658	968	948	644	727	akap10
ENSXETG00000005788	2025	0	1	0	1	2	1	0	0	0	1	1	1	trpv3
ENSXETG00000005789	1195	545	709	646	957	734	2412	584	674	387	864	662	763	aspa
ENSXETG00000005790	2517	15	28	25	47	36	46	43	56	40	73	48	60	trpv1
ENSXETG00000005791	882	2485	3442	2958	2409	1840	4085	2675	3267	1798	4002	2826	3334	denr
ENSXETG00000005793	3301	373	512	428	812	595	972	795	932	570	1054	723	904	hip1r
ENSXETG00000005794	1649	1508	2231	1913	1730	1366	4376	673	830	443	2231	1442	1707	shpk
ENSXETG00000005795	1246	822	1158	1007	899	600	2039	1272	1491	847	711	462	532	ctns
ENSXETG00000005797	2814	821	1163	939	1228	921	2722	2284	2636	1470	2220	1559	1777	vps37b
ENSXETG00000005800	414	628	851	767	1188	872	1207	1249	1381	831	949	672	805	tax1bp3
ENSXETG00000005802	1941	1065	1476	1277	699	486	1365	1518	1731	1041	469	348	385	bcl7b
ENSXETG00000005803	1543	217	297	281	158	129	335	147	164	80	222	163	173	ogfod2
ENSXETG00000005805	1487	1493	2159	1897	1591	1324	1176	1043	1245	744	1037	735	839	tbl2
ENSXETG00000005807	930	59	72	63	136	96	148	228	303	153	174	85	107	thap11
ENSXETG00000005808	2883	143	239	210	148	114	320	973	1231	714	472	326	344	itgae
ENSXETG00000005809	4209	531	802	618	1455	1049	1550	2396	2843	1616	1759	1285	1463	edc4
ENSXETG00000005810	1953	369	594	523	518	408	1486	1040	1340	758	509	326	403	pla2g15
ENSXETG00000005811	2265	172	239	226	452	339	569	621	705	395	1040	690	787	trpv2
ENSXETG00000005812	3542	480	716	570	1897	1456	3222	935	1031	640	2152	1474	1831	nfatc3
ENSXETG00000005816	1706	34	49	50	56	43	117	167	170	97	145	98	120	fto
ENSXETG00000005817	1449	171	294	240	315	223	537	767	836	525	192	134	141	cdk2ap1
ENSXETG00000005818	1884	1	1	0	6	9	1	2	2	2	1	1	5	irx3
ENSXETG00000005820	2309	206	265	218	297	246	589	815	889	539	455	314	333	kmt5a
ENSXETG00000005821	1743	647	897	751	528	405	808	967	1260	645	448	330	367	rilpl2
ENSXETG00000005824	2246	4338	6220	5253	3460	2695	3374	4032	4940	2881	2613	1985	2019	tmed2
ENSXETG00000005825	2051	566	695	591	504	377	448	845	966	562	511	408	415	ddx55
ENSXETG00000005830	993	554	808	737	670	485	837	868	1088	578	942	646	768	eif2b1
ENSXETG00000005831	2285	10	18	21	77	63	41	196	252	148	59	41	41	tctn2
ENSXETG00000005832	2605	222	369	291	471	351	618	423	548	307	488	387	393	atp6v0a2
ENSXETG00000005837	3033	4	5	8	62	52	163	381	410	260	214	137	165	sema6c
ENSXETG00000005838	924	2	6	4	36	24	40	295	309	212	128	90	114	mllt11
ENSXETG00000005839	1407	136	182	161	379	283	421	950	1003	652	547	408	467	gabpb2
ENSXETG00000005840	3705	3799	5849	4850	10299	7896	9652	6536	7169	4740	7977	5713	6214	copa
ENSXETG00000005841	2924	0	0	0	0	0	0	2	1	0	0	0	0	nhlh1
ENSXETG00000005842	3611	186	223	211	757	557	790	2231	2362	1540	1452	1017	1149	vangl2
ENSXETG00000005844	831	24	27	12	62	50	31	0	1	1	14	15	12	ENSXETG00000005844
ENSXETG00000005847	1029	88	129	100	130	106	223	234	281	160	298	243	270	rbm11
ENSXETG00000005849	2452	714	1039	903	1994	1540	2108	1125	1361	804	2499	1846	1988	liph
ENSXETG00000005850	1155	1	3	3	19	3	17	132	151	100	56	36	42	olfm2
ENSXETG00000005851	1599	176	237	212	163	109	252	382	452	226	104	90	103	hspa13
ENSXETG00000005855	1616	838	1155	1054	1406	990	1732	2480	2961	1634	2394	1657	1887	pin1
ENSXETG00000005857	2391	0	1	0	0	1	0	0	2	0	0	3	1	
ENSXETG00000005863	2490	1	0	1	0	0	0	1	4	0	1	0	1	gad1.2
ENSXETG00000005865	1170	96	122	114	91	79	231	142	198	92	136	123	115	c5orf22
ENSXETG00000005867	1896	13	22	17	12	14	11	1	3	2	4	2	3	klkb1
ENSXETG00000005868	2605	201	255	246	675	495	1486	438	565	322	2353	1661	1891	cyp4v2
ENSXETG00000005871	1779	41	67	59	224	141	257	11	12	5	604	420	451	fam149a
ENSXETG00000005873	2703	262	296	259	519	402	1093	339	424	197	709	539	647	tlr3
ENSXETG00000005878	909	0	0	0	0	0	0	0	0	0	0	0	0	
ENSXETG00000005880	2976	105	154	116	278	202	592	975	1203	738	732	509	552	hip1
ENSXETG00000005881	1161	7	7	14	14	16	43	34	41	24	110	66	81	ENSXETG00000005881
ENSXETG00000005902	909	80	93	91	120	83	158	487	483	340	60	50	49	prtfdc1

ENSXETG00000005905	5795	672	876	805	1581	1284	2184	2777	3246	1931	3969	2605	3106 arhgap21
ENSXETG00000005906	5400	460	644	547	1282	1042	1551	2103	2441	1500	2617	1783	2081 kiaa1217
ENSXETG00000005911	1887	206	271	206	156	112	180	802	975	586	201	180	195 hes6.1
ENSXETG00000005914	813	0	0	0	4	2	0	0	0	0	18	17	9 ptf1a
ENSXETG00000005916	2604	7	14	11	40	33	39	43	46	24	57	38	42 msrb2
ENSXETG00000005917	2673	1	2	1	132	105	12	9	7	4	16	3	15 armc3
ENSXETG00000005918	2521	800	1244	1009	557	500	1002	1324	1640	845	582	373	467 nampt
ENSXETG00000005919	3555	193	269	227	406	319	557	1339	1573	890	638	464	515 pik3cg
ENSXETG00000005920	1218	6	10	5	7	15	14	54	70	51	29	27	33 prkar2b
ENSXETG00000005922	2595	336	469	391	1531	1204	2779	1971	2318	1292	1768	1113	1325 hbp1
ENSXETG00000005925	855	240	382	336	127	106	155	220	225	149	189	112	129 dus4l
ENSXETG00000005927	2103	302	347	301	1650	1330	449	21	39	15	246	177	206 ENSXETG00000005927
ENSXETG00000005928	3396	735	1070	836	1169	842	2215	2805	3304	1893	1823	1255	1532 dag1
ENSXETG00000005929	2862	492	662	620	1065	831	2321	2400	2770	1610	2186	1570	1672 usp4
ENSXETG00000005930	1122	1	1	0	0	0	2	4	3	0	17	8	18 avpr1b
ENSXETG00000005931	5864	39	41	35	315	213	367	699	837	463	782	577	627 ptpru
ENSXETG00000005932	1708	46	47	42	2544	1967	96	51	37	22	3007	2179	2278 ctse
ENSXETG00000005933	1138	24467	35967	30829	43916	32949	102306	88306	99801	61307	48710	34093	37255 gpx1
ENSXETG00000005934	1643	7319	10693	8921	10377	7873	13905	20619	23723	13895	11213	8057	8624 rhoa
ENSXETG00000005935	1496	265	366	299	419	377	581	648	855	497	552	360	423 nupl2
ENSXETG00000005936	2761	67	115	92	173	132	218	919	1111	674	509	406	375 fam126a
ENSXETG00000005937	1212	2518	3811	3219	3341	2521	3609	2235	2507	1591	2499	1831	1935 amt
ENSXETG00000005939	1086	0	0	0	2	0	0	0	0	0	0	0	1 ENSXETG00000005939
ENSXETG00000005940	1504	0	0	0	2	0	1	3	2	4	3	1	3 matn1
ENSXETG00000005941	1632	0	0	1	0	0	0	6	0	3	0	1	3 ENSXETG00000005941
ENSXETG00000005943	1499	131	210	187	278	204	470	282	299	188	342	218	268 nicn1
ENSXETG00000005944	1234	87	128	116	68	41	33	503	609	329	75	62	74 rab7b
ENSXETG00000005945	2187	504	728	648	1269	986	1626	1204	1393	893	1178	837	878 apeh
ENSXETG00000005947	2391	9	31	12	3872	3013	159	22	26	16	2518	1736	2009 slc26a9
ENSXETG00000005948	2406	511	786	649	411	305	708	1014	1113	664	459	338	374 tcta
ENSXETG00000005949	2169	1	4	3	51	51	81	190	197	130	449	309	356 mst1
ENSXETG00000005950	3667	5304	7736	6256	6783	5181	9170	1078	1165	637	3258	2348	2656 pm20d1
ENSXETG00000005952	4502	499	795	655	1421	1038	2161	1747	1998	1212	1635	1130	1279 rnf123
ENSXETG00000005954	1798	2780	4091	3598	2750	2208	1072	1629	1839	1099	537	372	436 gmppb
ENSXETG00000005955	5168	1259	1935	1571	3498	2607	4697	3022	3675	2098	2749	1969	2207 ip6k1
ENSXETG00000005956	1108	707	1048	930	506	433	471	547	650	416	524	406	378 mrpl39
ENSXETG00000005958	3087	0	1	0	0	0	4	6	8	4	4	3	0 tlr9
ENSXETG00000005959	3290	44	46	49	185	122	74	195	258	143	62	38	40 traip
ENSXETG00000005960	1194	35	44	35	123	90	125	2027	2240	1453	321	235	249 fam212a
ENSXETG00000005961	2730	43	80	70	893	671	881	1738	1936	1209	1445	1037	1079 ENSXETG00000005961
ENSXETG00000005963	2601	0	0	1	0	0	1	0	2	1	0	1	1 tmprss2
ENSXETG00000005966	1798	311	445	397	541	439	584	664	806	471	517	368	392 c12orf4
ENSXETG00000005967	1698	14	12	13	21	18	36	61	75	36	24	19	15 ENSXETG00000005967
ENSXETG00000005968	621	0	0	0	0	0	0	0	0	0	0	0	0
ENSXETG00000005971	2567	200	298	262	204	204	240	885	1078	609	242	164	202 tigar
ENSXETG00000005972	879	137	191	111	157	122	151	306	356	190	348	255	327 ccnd2
ENSXETG00000005976	1179	3	9	6	18	7	20	137	131	125	36	32	22 XB-GENE-5762452
ENSXETG00000005977	969	0	0	0	0	0	0	0	0	0	0	0	0
ENSXETG00000005981	432	0	0	0	0	0	0	0	0	0	0	0	0
ENSXETG00000005982	2424	0	0	0	0	0	0	0	0	0	0	0	0 ENSXETG00000005982
ENSXETG00000005983	2598	0	0	0	0	0	0	0	0	0	0	0	0
ENSXETG00000005986	2424	0	0	0	0	0	0	0	1	0	0	0	0 ENSXETG00000005986
ENSXETG00000005989	1845	96	143	113	165	157	264	305	369	232	390	333	336 rcor3
ENSXETG00000005990	3819	1133	1745	1458	2505	1967	3782	17612	20557	12243	3758	2558	2977 ENSXETG00000005990
ENSXETG00000005992	2556	0	0	0	11	12	3	3	5	1	17	16	14 ENSXETG00000005992
ENSXETG00000005994	2490	109	131	110	122	103	103	184	242	150	437	312	378 ENSXETG00000005994
ENSXETG00000005995	11568	24	26	26	180	153	21	12	10	11	404	264	305 ENSXETG00000005995
ENSXETG00000006000	2739	11	20	10	68	46	113	774	1003	568	344	201	266 pkdcc.2
ENSXETG00000006002	3220	996	1336	1169	3609	2717	5649	4020	4688	2825	4751	3477	3635 eml4
ENSXETG00000006004	2502	0	0	0	2	0	0	1	2	0	4	3	2 ENSXETG00000006004
ENSXETG00000006007	1284	209	316	273	663	461	2371	830	1053	584	1287	755	996 ENSXETG00000006007
ENSXETG00000006008	1176	2831	4085	3416	3331	2647	3575	15207	17675	10591	2293	1688	1917 ENSXETG00000006008
ENSXETG00000006009	680	7530	11316	9908	2901	2165	4350	4040	4683	2953	1862	1306	1447 prdx5
ENSXETG00000006011	2507	3	1	1	7	7	2	15	30	14	11	7	10 slc45a1
ENSXETG00000006013	644	636	889	722	364	297	548	513	607	335	534	372	449 ttc9c
ENSXETG00000006014	4032	36	57	43	79	60	187	1049	1177	775	233	170	170 ENSXETG00000006014
ENSXETG00000006015	2380	581	906	819	1980	1466	1825	2737	3078	1874	1616	1259	1368 hnrnpul2
ENSXETG00000006017	3992	2784	3804	3085	993	765	4214	3044	3759	2119	2452	1770	2032 errfi1
ENSXETG00000006018	1843	1311	1796	1557	1504	1152	2091	1444	1587	1003	1801	1337	1428 park7
ENSXETG00000006019	2135	441	578	489	495	414	732	1086	1418	785	593	434	537 rcn2
ENSXETG00000006020	1243	17	31	15	94	84	314	433	460	262	251	196	177 lrrn4cl
ENSXETG00000006021	990	120	182	120	271	235	465	302	346	208	455	324	323 bscl2
ENSXETG00000006022	958	585	810	660	578	476	771	1342	1536	945	700	476	542 polr2g
ENSXETG00000006024	390	0	0	0	0	0	3	0	2	1	1	0	3 ENSXETG00000006024
ENSXETG00000006025	2855	114	155	144	226	150	345	542	568	389	348	239	268 zbtb3
ENSXETG00000006027	2676	26	35	65	38	44	83	557	634	358	95	80	58 XB-GENE-5818700
ENSXETG00000006028	1754	273	403	382	383	306	411	721	908	493	415	272	297 c15orf40
ENSXETG00000006029	2007	452	657	544	829	594	1389	1145	1291	732	975	687	729 lrrc47
ENSXETG00000006030	1443	88	121	93	537	418	894	640	771	414	872	591	657 irf7
ENSXETG00000006031	1251	2	1	1	2	0	0	20	21	10	2	3	5 ENSXETG00000006031
ENSXETG00000006032	5114	428	661	541	1371	1063	2291	1923	2218	1338	1944	1422	1556 phrf1
ENSXETG00000006033	2909	32	50	52	155	118	239	373	419	255	231	178	206 cep104
ENSXETG00000006035	1848	95	183	134	175	133	224	141	178	111	124	107	117 dffb
ENSXETG00000006036	2429	386	496	434	609	520	784	1898	2282	1263	581	437	494 C1orf174
ENSXETG00000006038	1783	302	447	387	1029	835	1232	1204	1384	899	1509	1097	1128 rassf7
ENSXETG00000006039	1042	0	0	0	22	14	2	4	3	1	10	1	3 lrrc56
ENSXETG00000006040	1743	111	182	126	187	151	200	531	577	354	242	150	186 hras
ENSXETG00000006041	1050	0	0	0	0	0	1	0	2	0	0	2	1 fut6
ENSXETG00000006045	2490	0	0	0	0	0	0	0	0	0	0	0	0
ENSXETG00000006049	864	28	42	42	144	105	127	35	35	24	238	188	168 ENSXETG00000006049
ENSXETG00000006051	2365	346	488	462	463	363	633	924	1077	669	539	341	391 usp22
ENSXETG00000006052	1245	648	974	796	704	553	1070	610	648	387	872	676	694 dhrs7b
ENSXETG00000006053	1096	78	97	74	19	13	30	3	0	0	7	5	10 slc25a45
ENSXETG00000006056	519	357	510	441	524	434	738	655	749	424	685	497	584 tmem11
ENSXETG00000006057	1763	2899	4164	3551	3657	2774	4088	3876	4611	2734	2580	1718	1984 arpc1a
ENSXETG00000006058	1498	6274	9416	7923	6337	4799	8325	17387	19891	12077	10262	7233	7958 arpc1b
ENSXETG00000006061	1680	0	0	0	0	0	0	0	0	0	0	0	0
ENSXETG00000006062	826	1795	2653	2241	2575	2053	2998	3909	4302	2572	4229	2968	3208 pdap1
ENSXETG00000006064	1029	5344	7698	6740	4852	3628	4739	743	964	565	1891	1355	1519 sult1e1
ENSXETG00000006071	1023	44	60	52	226	202	287	310	376	241	552	364	392 cfap36
ENSXETG00000006072	3183	1178	1547	1344	2271	1837	3044	2755	3331	1958	1856	1323	1527 ppp4r3b
ENSXETG00000006073	1930	18	33	27	146	88	100	182	224	118	35	24	26 depdc1b
ENSXETG00000006075	2481	263	423	338	252	219	240	372	462	276	141	139	120 pnpt1
ENSXETG00000006076	1383	19	21	23	139	115	122	194	256	146	392	269	332 efemp1
ENSXETG00000006078	1059	0	0	0	0	0	0	4	5	2	2	1	4
ENSXETG00000006081	2448	0	0	0	0	0	0	0	0	0	0	0	0
ENSXETG00000006082	1515	0	0	0	0	0	0	1	2	1	0	0	0
ENSXETG00000006083	3332	2	0	1	1	0	1	11	7	10	0	1	1 slc1a3
ENSXETG00000006085	1572	0	0	0	0	0	0	1	6	3	1	1	3
ENSXETG00000006087	2015	675	988	885	1714	1276	1542	3332	3980	2320	1220	777	973 znf326
ENSXETG00000006088	2568	255	356	265	377	278	419	451	538	314	426	312	364 lrrc8d

ENSXETG00000006089	4595	1	5	3	14	8	17	105	135	80	41	33	29	lrrc8c
ENSXETG00000006090	1427	332	475	362	466	368	462	375	435	250	337	235	246	kyat3
ENSXETG00000006091	1742	623	871	788	614	501	905	1470	1729	1052	674	496	533	gtf2b
ENSXETG00000006092	2982	122	187	153	254	236	408	592	758	475	411	345	316	pkn2
ENSXETG00000006096	3510	0	0	0	0	0	0	0	0	0	0	0	0	
ENSXETG00000006099	2496	0	2	5	10	9	29	6	9	3	7	2	5	ENSXETG00000006099
ENSXETG00000006103	3711	0	0	0	0	0	0	0	0	0	0	0	0	
ENSXETG00000006104	552	0	0	0	0	0	0	0	0	0	0	0	0	
ENSXETG00000006105	1470	272	470	364	393	276	624	756	882	482	488	390	392	gid8
ENSXETG00000006106	6649	272	439	370	1065	811	1108	1509	1586	1003	1907	1382	1538	DIDO1
ENSXETG00000006108	2043	5	4	5	4	3	1	10	12	11	2	7	6	col9a3
ENSXETG00000006109	1952	55	79	65	190	132	346	233	207	147	322	216	263	ENSXETG00000006109
ENSXETG00000006110	834	55	85	78	258	171	404	523	552	344	357	290	284	ogfr
ENSXETG00000006112	1294	153	220	235	346	258	373	810	956	646	371	272	311	mrgbp
ENSXETG00000006113	768	0	0	0	1	0	0	0	0	0	0	0	0	ENSXETG00000006113
ENSXETG00000006114	801	0	0	1	0	0	0	0	0	0	0	0	0	
ENSXETG00000006115	1461	1796	2477	2071	1811	1381	2391	304	332	189	342	240	263	c14orf1
ENSXETG00000006118	937	0	0	1	0	0	0	0	1	0	0	0	0	XB-GENE-992027
ENSXETG00000006123	966	4	1	2	2	3	6	16	14	10	12	3	10	ENSXETG00000006123
ENSXETG00000006124	987	1654	2306	1991	2364	1890	3030	1426	1714	942	2147	1542	1710	igsf5
ENSXETG00000006126	2345	13	18	15	13	17	21	42	44	19	25	20	22	dscam
ENSXETG00000006128	5133	2	2	8	61	41	147	316	386	245	398	257	266	adamtsl3
ENSXETG00000006135	639	81	124	106	204	193	145	578	675	404	231	180	196	ENSXETG00000006135
ENSXETG00000006139	861	17	26	15	31	19	41	32	23	17	66	47	57	ENSXETG00000006139
ENSXETG00000006144	1305	0	0	0	0	0	0	2	1	0	0	0	0	
ENSXETG00000006145	1324	7147	10690	9029	11290	8678	8920	3541	4011	2292	10752	7539	8352	ppa2
ENSXETG00000006146	1909	1319	1948	1622	1478	1116	2507	916	1047	558	1455	963	1136	tysnd1
ENSXETG00000006147	1414	7	6	10	37	26	44	169	183	136	107	81	57	spock2
ENSXETG00000006149	1343	391	527	496	562	420	901	878	1037	540	712	517	599	ascc1
ENSXETG00000006150	1837	547	825	653	746	606	1156	932	1083	609	1051	732	825	anapc16
ENSXETG00000006151	1683	1341	1796	1618	1914	1435	3384	1806	2015	1218	2272	1657	1796	dnajb12
ENSXETG00000006153	1053	108	157	110	94	95	154	162	177	95	34	19	25	nudt13
ENSXETG00000006154	1608	62	109	80	47	27	65	163	200	122	78	69	61	p4ha1
ENSXETG00000006155	1252	4568	6492	5566	3308	2635	5893	2871	3483	2069	2154	1531	1767	pla2g12b
ENSXETG00000006156	1665	32	56	43	22	28	33	8	23	10	18	11	17	oit3
ENSXETG00000006157	3252	1538	2103	1720	2613	2042	2720	2587	3266	1785	2411	1705	1842	mcu
ENSXETG00000006158	4686	0	0	0	0	0	1	0	0	0	0	1	0	
ENSXETG00000006161	897	464	692	634	100	58	120	357	418	281	137	115	120	sec22c
ENSXETG00000006162	1878	68	106	88	323	249	501	1129	1237	754	777	524	624	zbtb47
ENSXETG00000006163	597	35	73	45	47	42	181	104	140	70	104	63	102	nt5dc1
ENSXETG00000006166	1620	521	787	636	634	479	964	532	638	352	725	506	604	FRK
ENSXETG00000006167	1762	0	0	0	0	1	0	1	4	1	1	3	0	hhatl
ENSXETG00000006168	2703	0	0	0	0	0	0	5	5	6	0	0	0	
ENSXETG00000006170	2139	1	1	0	54	38	6	13	16	16	11	5	7	ccdc13
ENSXETG00000006171	1016	8853	12771	10715	1177	859	1774	5627	6683	3933	519	390	386	higd1a
ENSXETG00000006172	1977	8	10	8	134	78	32	104	90	56	22	16	20	cfap206
ENSXETG00000006173	1536	4201	6127	5248	1291	979	6517	1018	1222	717	1662	1133	1290	cyp8b1
ENSXETG00000006174	1656	0	0	0	0	0	0	0	0	0	0	0	0	
ENSXETG00000006175	2137	18	14	10	43	34	65	403	497	304	85	46	57	gjc2
ENSXETG00000006176	894	39	58	49	75	62	73	88	97	59	67	50	61	iba57
ENSXETG00000006186	2016	18	12	10	54	31	66	86	92	65	27	22	28	ahrr
ENSXETG00000006188	1741	304	458	309	378	274	656	388	484	233	380	260	310	pigm
ENSXETG00000006189	4240	85	104	115	137	117	232	312	394	192	297	166	209	anks6
ENSXETG00000006191	1746	1388	1965	1742	2152	1661	2440	2729	3100	1869	2251	1608	1785	galnt4
ENSXETG00000006194	2497	145	270	205	359	259	602	774	950	531	486	330	381	rab5b
ENSXETG00000006195	2950	601	836	716	1091	790	1421	1897	2175	1274	1044	712	774	exoc3
ENSXETG00000006199	1719	1	0	1	7	3	7	69	88	44	24	9	10	mapk10
ENSXETG00000006200	1620	17	20	15	59	45	146	182	223	124	131	109	122	arhgap24
ENSXETG00000006201	905	19	40	33	155	136	54	283	296	214	249	164	163	stard10
ENSXETG00000006203	4199	2186	3162	2744	2620	2002	3782	1425	1656	939	2447	1664	1913	map7d2
ENSXETG00000006205	1812	24	46	38	53	38	124	858	1006	582	188	158	136	sh3kbp1
ENSXETG00000006210	3831	317	464	380	578	388	638	1215	1434	816	711	472	593	map3k15
ENSXETG00000006212	1814	1541	2244	1941	1929	1478	1977	2873	3320	2006	2037	1312	1529	pdha1
ENSXETG00000006214	1740	61	110	84	117	90	106	186	235	150	114	90	106	klhl15
ENSXETG00000006215	903	0	0	0	0	0	0	0	0	0	0	0	0	ENSXETG00000006215
ENSXETG00000006216	2249	393	609	501	889	704	1007	895	995	574	774	563	593	os9
ENSXETG00000006218	847	852	1192	1029	904	678	835	974	1206	666	931	633	646	apoo
ENSXETG00000006219	450	5228	7533	6277	7071	5472	6606	5444	6468	3685	5761	4245	4367	sat1
ENSXETG00000006221	2303	38	59	64	120	91	89	12	32	13	16	11	6	arsd
ENSXETG00000006222	6000	908	1244	1005	2650	2021	4924	1297	1605	876	2965	2063	2402	b4galnt1
ENSXETG00000006223	1292	272	406	314	521	385	458	385	542	312	770	483	578	acot9
ENSXETG00000006224	1740	20	26	10	43	28	79	300	378	196	57	36	30	slc26a10
ENSXETG00000006225	2297	4757	6943	5872	8352	6373	12816	9207	10626	6289	18310	12741	14176	eif2s3
ENSXETG00000006226	1756	55	99	65	84	66	65	0	0	0	17	9	18	sp5l
ENSXETG00000006227	8906	19	31	14	130	102	397	924	1019	586	493	367	369	arhgef25
ENSXETG00000006229	1077	521	691	585	563	472	769	596	688	376	464	355	410	pip4k2c
ENSXETG00000006230	3250	6	6	10	66	55	57	165	188	114	135	96	100	kif5a
ENSXETG00000006231	1617	30	56	35	106	77	103	184	203	146	142	107	109	POU2F1
ENSXETG00000006234	1992	1422	2069	1736	2130	1630	2480	3906	4502	2556	2182	1585	1798	dctn2
ENSXETG00000006237	5500	103	172	164	263	221	402	606	785	433	510	333	375	nhsl1
ENSXETG00000006239	4529	42	61	49	234	192	401	337	359	223	433	329	365	mbd6
ENSXETG00000006245	1092	1114	1561	1372	807	611	1973	2033	2381	1517	2933	2014	2301	ddit3
ENSXETG00000006252	1566	59	86	90	288	215	71	8	6	3	858	620	609	ENSXETG00000006252
ENSXETG00000006253	1584	0	0	0	0	0	0	12	16	11	7	2	3	ntng2
ENSXETG00000006254	7221	230	318	255	620	467	1156	1511	1695	1033	1431	939	1106	setx
ENSXETG00000006257	984	0	0	0	0	0	1	0	0	0	0	0	0	
ENSXETG00000006258	1845	309	556	409	421	331	760	772	806	494	759	564	611	ddx31
ENSXETG00000006259	2298	180	288	243	317	255	347	484	560	383	664	446	520	gtf3c4
ENSXETG00000006260	1602	8	12	3	202	137	56	100	131	52	35	22	26	ak8
ENSXETG00000006261	785	2	1	1	251	204	16	4	3	2	59	29	43	spaca9
ENSXETG00000006262	2583	123	187	181	379	278	1111	526	507	393	1111	723	806	tsc1
ENSXETG00000006263	1005	0	1	1	1	1	6	17	16	17	10	4	5	gfi1b
ENSXETG00000006264	912	139	164	143	166	117	406	316	396	228	462	329	356	gtf3c5
ENSXETG00000006265	2137	9	15	16	139	99	77	43	40	29	841	620	649	cel
ENSXETG00000006267	5106	231	365	305	933	680	1007	4114	4637	2896	1793	1296	1485	ralgds
ENSXETG00000006269	2070	84	143	122	183	155	448	357	436	250	386	264	293	c10orf76
ENSXETG00000006271	2920	173	268	231	376	276	653	1222	1314	745	819	590	602	hps6
ENSXETG00000006272	2403	266	388	349	722	514	598	1086	1353	758	875	553	698	ENSXETG00000006272
ENSXETG00000006274	3040	64	98	87	219	180	554	414	453	289	560	450	405	mapk7
ENSXETG00000006275	972	494	742	567	755	474	2329	745	974	551	1018	658	782	cds1
ENSXETG00000006277	780	0	0	0	0	0	0	0	0	0	319	208	284	
ENSXETG00000006278	1781	891	1332	1185	2324	1805	4642	2870	3377	1980	3830	2681	2926	rnf112
ENSXETG00000006279	10605	243	356	309	813	663	1156	2182	2612	1563	1266	921	1036	wdfy3
ENSXETG00000006281	843	0	0	0	0	0	0	0	0	1	2	0	0	ENSXETG00000006281
ENSXETG00000006282	960	0	0	0	0	0	0	1	3	0	0	0	0	
ENSXETG00000006284	1704	29	35	31	147	129	96	483	609	304	351	239	306	rnf112.2
ENSXETG00000006285	921	2	2	0	0	0	0	0	0	0	0	0	0	
ENSXETG00000006287	1965	0	1	0	2	1	0	55	60	35	79	52	68	ENSXETG00000006287
ENSXETG00000006293	969	4	12	6	16	18	6	6	5	2	11	11	9	ENSXETG00000006293
ENSXETG00000006294	3160	45	64	41	802	558	92	98	130	75	1410	935	1039	ENSXETG00000006294
ENSXETG00000006296	1157	15	25	14	398	282	107	110	128	78	68	33	66	prss27

ENSXETG00000006297	1554	16	16	24	73	75	1	15	8	3	1	1	0	
ENSXETG00000006299	1140	0	0	0	0	0	1	0	0	0	0	0	0	
ENSXETG00000006301	2337	0	0	0	0	0	0	0	0	0	0	0	0	
ENSXETG00000006302	1795	0	0	0	0	0	1	0	0	0	0	0	0	
ENSXETG00000006304	3064	2	3	2	20	14	9	33	34	18	44	38	22	tbx1
ENSXETG00000006305	1664	47	60	60	62	39	117	50	90	49	55	25	53	gnb1l
ENSXETG00000006306	1402	848	1076	905	871	635	1330	892	1130	693	773	547	608	dffa
ENSXETG00000006307	2408	429	602	522	590	459	930	670	798	436	529	351	412	txnrd2
ENSXETG00000006309	921	1575	2157	2007	1894	1438	3069	830	886	550	1482	1106	1255	pex14
ENSXETG00000006312	6507	294	416	351	819	631	1505	2060	2291	1325	1679	1135	1260	casz1
ENSXETG00000006314	2238	2	2	0	0	1	0	4	11	2	0	0	1	masp2
ENSXETG00000006315	1256	3616	5209	4626	2157	1564	1475	147	170	95	791	543	623	comt
ENSXETG00000006316	2658	848	1250	1082	1150	846	1155	2135	2428	1426	1324	920	1045	arvcf
ENSXETG00000006317	2414	1380	1963	1724	1378	1052	2059	4783	5719	3393	2406	1758	1911	tardbp
ENSXETG00000006318	2595	827	1304	1093	993	744	802	1514	1877	1018	1214	839	951	exosc10
ENSXETG00000006319	1594	135	211	172	275	248	621	587	691	398	486	362	366	tango2
ENSXETG00000006320	8431	1004	1410	1198	1384	1064	1491	2366	2925	1676	2389	1623	1921	dgcr8
ENSXETG00000006322	2619	293	392	370	691	500	779	763	905	513	729	507	544	mtor
ENSXETG00000006323	2650	658	896	765	906	730	842	1196	1342	781	981	726	828	trmt2a
ENSXETG00000006324	690	1977	2829	2407	1797	1412	1186	4489	5106	2843	1878	1310	1433	ranbp1
ENSXETG00000006325	2477	91	116	115	313	278	767	1047	1254	717	675	465	581	zdhhc8
ENSXETG00000006327	2106	41	58	47	86	56	114	187	249	162	87	61	85	tnrc6b
ENSXETG00000006328	1640	1488	2084	1772	1381	1075	1274	1198	1412	871	1483	1094	1111	adsl
ENSXETG00000006329	3590	368	590	458	491	341	421	502	538	304	730	489	537	sgsm3
ENSXETG00000006330	1917	14	21	10	62	57	140	211	226	98	98	39	66	ENSXETG00000006330
ENSXETG00000006331	3356	836	1216	1018	939	676	604	1721	1983	1156	703	465	574	rangap1
ENSXETG00000006332	2901	464	640	580	589	453	546	1022	1172	684	735	578	624	zc3h7b
ENSXETG00000006333	888	1910	2495	2213	5458	4376	4845	1217	1369	791	2107	1526	1675	tef
ENSXETG00000006335	1906	0	1	0	0	0	2	13	5	6	1	0	0	
ENSXETG00000006338	1404	19	24	32	32	30	36	324	296	200	30	17	30	thbs3
ENSXETG00000006339	1419	533	816	637	1107	827	1703	962	1163	694	817	572	654	tom1
ENSXETG00000006340	3993	421	673	606	971	737	1733	1636	1870	1055	1327	951	1017	hmgxb4
ENSXETG00000006341	6648	52	63	69	132	89	158	95	108	62	284	197	205	unc13c
ENSXETG00000006343	2602	56	94	67	184	109	301	645	725	428	411	272	317	micall1
ENSXETG00000006344	3042	0	0	0	2	5	0	2	2	0	2	1	2	wdr72
ENSXETG00000006345	1037	66	118	116	133	113	179	556	624	391	421	325	344	c22orf23
ENSXETG00000006346	603	810	1166	1033	1171	924	1916	1685	1969	1216	2322	1614	1774	polr2f
ENSXETG00000006348	2879	2	2	0	14	16	42	254	298	177	118	86	100	sox10
ENSXETG00000006349	1897	12	19	22	216	164	66	10	19	7	526	418	397	onecut1
ENSXETG00000006351	1857	2824	4027	3451	4219	3183	6915	2147	2469	1455	1893	1301	1581	baiap2l2
ENSXETG00000006353	4764	265	414	345	801	616	493	521	622	414	355	247	285	pla2g6
ENSXETG00000006354	3617	298	443	403	1528	1231	3142	2029	2516	1327	1300	926	986	fam214a
ENSXETG00000006355	447	111	122	130	245	164	423	344	358	232	318	217	243	maff
ENSXETG00000006356	3967	63	96	78	228	200	576	1234	1346	837	234	204	202	tmem184b
ENSXETG00000006357	2643	463	637	537	1395	1113	1655	2663	3016	1731	1890	1261	1520	csnk1e
ENSXETG00000006360	1332	0	0	0	0	0	0	0	0	0	0	0	0	
ENSXETG00000006377	999	0	2	3	0	0	1	3	5	2	0	0	0	mthfd2l
ENSXETG00000006380	944	481	725	586	750	550	767	234	291	162	754	571	667	ereg
ENSXETG00000006382	1236	424	626	467	346	298	1716	1051	1213	699	432	338	396	mfsd12
ENSXETG00000006384	2457	221	267	228	249	200	454	463	601	388	259	202	250	med11
ENSXETG00000006385	3404	125	192	129	259	206	640	1188	1321	793	548	396	411	amotl1
ENSXETG00000006386	2433	308	532	428	767	534	990	1553	1674	1016	1065	699	805	pias4
ENSXETG00000006391	2982	117373	170586	149317	239031	178606	344324	257769	300980	179305	643070	446255	498738	eef2.1
ENSXETG00000006394	1140	3	2	3	5	10	42	70	83	51	55	44	29	atcay
ENSXETG00000006396	1865	393	596	495	717	490	1032	1996	2435	1367	944	664	668	dapk3
ENSXETG00000006397	2807	368	588	449	711	607	868	1587	1886	1080	912	674	756	fzr1
ENSXETG00000006402	1477	105	114	108	428	300	729	535	690	344	368	285	297	ccng2
ENSXETG00000006404	1818	101	133	114	173	122	339	620	702	448	303	210	254	sept11
ENSXETG00000006405	474	0	0	0	3	0	0	0	0	0	0	0	0	
ENSXETG00000006407	2727	609	915	784	1976	1485	1491	2442	2831	1693	2100	1405	1685	tjp3
ENSXETG00000006411	2394	0	0	0	5	3	0	0	0	0	4	0	3	piwil4
ENSXETG00000006415	738	77	130	99	78	65	121	132	152	91	66	53	50	ankrd49
ENSXETG00000006417	3348	217	306	281	424	281	591	732	888	526	717	491	585	mre11
ENSXETG00000006418	2005	5	7	11	10	10	16	72	70	58	24	18	17	pip5k1c
ENSXETG00000006419	1737	78	101	101	126	114	295	258	367	193	254	169	207	klhl8
ENSXETG00000006420	1275	0	0	2	5	3	7	5	8	4	4	2	0	gpr83.2
ENSXETG00000006423	1878	27	31	29	80	64	147	251	334	196	127	114	131	ifnar2.1
ENSXETG00000006425	1306	0	0	0	0	0	0	1	0	2	0	0	0	
ENSXETG00000006428	2972	198	288	249	127	125	225	404	457	261	275	203	242	panx1
ENSXETG00000006429	3264	195	296	250	163	148	24	424	470	292	7	12	9	hephl1
ENSXETG00000006432	2810	598	876	753	1856	1351	2663	1442	1602	965	2931	2099	2275	btbd2
ENSXETG00000006433	1678	582	875	736	526	410	571	199	228	157	664	462	512	GDPD2
ENSXETG00000006434	2574	349	499	427	431	321	428	604	726	408	516	357	403	csnk1g2
ENSXETG00000006435	1446	692	937	811	191	148	606	168	230	138	22	17	27	ENSXETG00000006435
ENSXETG00000006436	1629	30	73	51	168	121	113	248	295	160	47	32	40	ankle1
ENSXETG00000006438	3026	99	141	124	363	273	733	952	1029	615	617	442	493	arhgef18
ENSXETG00000006439	2576	556	867	695	2769	2152	3221	5797	6840	3956	5364	3783	4063	enc1.2
ENSXETG00000006440	855	0	0	1	3	0	1	0	0	0	0	1	1	
ENSXETG00000006441	2547	2099	3046	2575	2119	1703	6768	5102	5994	3636	4337	2981	3432	jund
ENSXETG00000006442	1692	30	28	30	158	102	165	266	302	219	245	164	178	tox3
ENSXETG00000006455	2282	757	1217	1093	1948	1462	2790	2802	3045	1832	2490	1802	1909	camk2g
ENSXETG00000006456	4590	8	17	15	26	18	35	281	323	151	21	8	20	uggt2
ENSXETG00000006458	1527	2177	3214	2705	1505	1158	803	844	991	566	712	460	505	dnajc3
ENSXETG00000006459	903	3	5	4	20	14	40	144	168	112	35	34	34	dzip1
ENSXETG00000006460	7773	4	3	1	11	9	3	220	326	185	8	9	19	camk2a
ENSXETG00000006461	1243	352	532	410	337	287	455	312	302	184	304	205	202	c10orf35
ENSXETG00000006462	1752	0	1	1	8	5	34	29	42	26	19	12	19	ENSXETG00000006462
ENSXETG00000006487	864	0	0	0	0	0	0	0	0	0	0	0	0	
ENSXETG00000006489	2736	61	102	105	85	85	229	276	306	180	221	137	166	ENSXETG00000006489
ENSXETG00000006490	792	0	0	0	2	0	1	3	4	3	3	0	1	znf350
ENSXETG00000006497	1818	1	3	1	13	12	19	32	65	29	75	61	57	XB-GENE-980117
ENSXETG00000006498	2542	0	0	0	4	0	0	0	0	0	0	0	1	abra
ENSXETG00000006499	4152	670	912	727	1148	889	1744	1541	1674	1054	1695	1172	1322	OXR1
ENSXETG00000006505	2919	0	1	2	5	9	32	55	39	32	42	23	23	zfpm2
ENSXETG00000006509	2058	1	2	6	16	31	25	237	238	143	80	47	69	ENSXETG00000006509
ENSXETG00000006517	1056	886	1298	1115	86	58	405	166	179	109	13	6	5	ENSXETG00000006517
ENSXETG00000006527	2352	0	0	0	4	1	3	25	51	20	6	7	9	ENSXETG00000006527
ENSXETG00000006532	2907	7	12	14	26	23	44	201	280	134	51	42	41	fbxo10
ENSXETG00000006533	1273	784	1134	1003	595	402	628	716	839	452	836	567	615	polr1e
ENSXETG00000006534	6699	282	418	343	466	380	806	932	1163	658	629	410	508	zbtb5
ENSXETG00000006535	4046	4	3	3	25	12	48	326	396	235	65	37	47	prickle2
ENSXETG00000006536	1274	0	0	1	2	0	1	0	0	0	2	3	1	grhpr.2
ENSXETG00000006537	1495	1626	2391	2183	3071	2268	2690	5525	6257	4059	1401	994	1096	psmd6
ENSXETG00000006538	2950	141	191	172	551	412	845	842	933	560	1069	725	792	atxn7
ENSXETG00000006539	661	611	929	778	803	625	1133	1265	1412	864	673	483	505	thoc7
ENSXETG00000006540	1024	0	0	0	0	0	0	0	0	0	0	0	0	
ENSXETG00000006541	2593	0	4	0	41	28	17	187	250	137	57	40	52	ENSXETG00000006541
ENSXETG00000006542	1689	237	355	267	188	155	245	300	417	217	296	200	240	zcchc7
ENSXETG00000006543	1080	154	274	234	194	143	214	452	501	302	201	146	167	rnf126
ENSXETG00000006545	3678	340	526	460	471	356	521	468	514	288	776	499	631	polrmt
ENSXETG00000006547	1173	0	0	0	0	0	2	1	5	1	0	0	3	

ENSXETG00000006551	2045	78	136	101	226	206	350	366	414	284	62	40	43	melk
ENSXETG00000006553	1141	2509	3631	3114	2413	1891	4278	3552	4094	2436	2632	1813	2012	bsg
ENSXETG00000006554	1869	63	85	74	216	138	238	382	442	264	328	239	284	rnf38
ENSXETG00000006555	1459	901	1298	1047	1305	940	1611	2734	3197	1841	1370	907	1035	cdc34
ENSXETG00000006556	1305	409	655	580	519	402	815	481	532	291	1139	722	904	trim14
ENSXETG00000006557	828	50	59	46	47	35	46	110	121	77	52	26	30	tpgs1
ENSXETG00000006558	1080	40777	60530	51096	15690	11648	39131	3581	4025	2435	3870	2743	3065	nans
ENSXETG00000006560	2278	1287	1912	1565	1133	839	1746	2280	2657	1553	1550	1080	1149	mfsd14b
ENSXETG00000006562	1854	0	1	1	1	0	6	17	17	10	15	12	11	shc2
ENSXETG00000006568	789	3	6	4	1	1	4	3	3	2	8	6	1	odf3l1
ENSXETG00000006571	6151	9	21	11	42	18	48	59	119	80	58	53	61	c2cd4c
ENSXETG00000006573	975	23	30	32	72	73	113	257	255	133	65	48	34	mier2
ENSXETG00000006574	2542	3	5	4	38	35	68	176	213	139	108	82	72	dpp6
ENSXETG00000006575	3202	64	85	65	161	104	296	246	359	167	248	152	186	znf420
ENSXETG00000006576	1887	0	0	0	0	0	1	0	5	1	3	0	1	lrrc32.2
ENSXETG00000006578	984	6	11	11	65	52	128	1120	1231	777	179	150	135	efnb1
ENSXETG00000006579	2189	211	295	211	1274	1042	1433	469	520	368	1995	1417	1553	tbx3
ENSXETG00000006581	6372	355	525	434	1396	1167	2106	2321	2494	1509	2474	1700	1989	med13l
ENSXETG00000006582	3705	175	241	199	399	301	395	657	835	465	578	404	457	paxip1
ENSXETG00000006584	2088	792	1139	1008	1158	910	1860	1519	1665	1041	1146	818	891	gle1
ENSXETG00000006585	1089	2	4	1	8	11	17	55	50	36	34	26	34	htr5a
ENSXETG00000006598	1352	23	34	33	10	11	15	16	14	12	14	13	10	dkk1
ENSXETG00000006611	1776	3373	4880	4306	5534	4260	11171	1427	1645	1070	5652	3981	4408	a1cf
ENSXETG00000006612	2333	9739	14350	12507	20241	15596	52787	9451	10458	6327	21312	15329	16676	asah2
ENSXETG00000006613	963	0	0	0	0	0	0	0	0	0	0	0	0	
ENSXETG00000006614	1077	268	388	337	513	377	751	1254	1399	827	599	413	461	sgms1
ENSXETG00000006616	1527	119	154	140	131	125	166	321	362	200	236	154	185	c8orf76
ENSXETG00000006617	1032	77	91	68	140	118	292	367	413	246	194	133	145	adat3
ENSXETG00000006618	1422	205	271	282	397	287	722	760	853	517	550	384	424	minpp1
ENSXETG00000006620	1925	4161	6017	5044	6534	5065	7073	10074	11900	7084	5430	3896	4174	mapre1
ENSXETG00000006621	1233	439	616	527	711	527	1437	860	953	566	957	754	763	nol4l
ENSXETG00000006623	1752	3	2	6	3	4	20	1371	1821	956	28	18	23	lrrc15
ENSXETG00000006627	3697	0	0	2	4	3	5	83	71	41	11	14	8	cdh13
ENSXETG00000006631	5677	749	1158	948	1741	1311	3082	2044	2415	1388	2794	1871	2237	ncald
ENSXETG00000006632	2808	68	93	79	439	322	245	409	492	290	645	422	445	grhl2
ENSXETG00000006633	660	1056	1391	1204	1318	1080	1742	2297	2617	1542	2377	1609	1910	znf706
ENSXETG00000006635	2783	23687	34904	29140	45794	35070	72042	44986	51565	30447	92306	64923	71775	pabpc1
ENSXETG00000006636	7602	0	0	0	0	0	0	0	0	0	0	0	0	ENSXETG00000006636
ENSXETG00000006637	486	187	333	298	215	175	242	235	312	196	292	209	193	ctdnep1
ENSXETG00000006639	1093	4861	6932	6272	5341	4291	7860	8906	9992	6268	3782	2626	2962	rab11a
ENSXETG00000006640	1608	105	128	124	130	103	131	129	131	71	331	271	242	znf234
ENSXETG00000006641	5733	94	134	110	310	230	556	837	998	585	598	389	429	dennd4a
ENSXETG00000006644	1650	118	186	166	259	186	243	372	418	260	261	206	214	vwa9
ENSXETG00000006646	2490	729	986	881	1555	1101	1589	2158	2385	1451	1179	851	954	dpp8
ENSXETG00000006647	3321	5	6	7	41	35	111	293	337	230	134	101	99	igdcc4
ENSXETG00000006648	1092	292	339	311	288	168	372	314	355	174	188	149	173	parp16
ENSXETG00000006650	3495	0	0	0	0	0	0	2	6	3	3	2	2	cilp
ENSXETG00000006651	1272	116	180	141	192	143	386	241	285	160	281	192	222	XB-GENE-5959487
ENSXETG00000006652	1173	267	423	387	230	185	285	214	211	109	175	132	136	mtfmt
ENSXETG00000006653	1254	0	0	0	0	0	1	14	9	13	0	1	0	kbtbd13
ENSXETG00000006656	3007	2560	3598	3079	4095	3113	6685	3428	4360	2501	3164	2176	2439	clpx
ENSXETG00000006658	2172	202	236	246	393	271	479	565	697	394	385	279	323	dpy19l4
ENSXETG00000006659	1308	2032	3042	2663	2467	1857	6924	2560	3153	1744	2576	1793	2021	spg21
ENSXETG00000006660	1815	94	120	96	170	121	38	338	390	244	49	30	35	ccne2
ENSXETG00000006661	1389	16	36	23	36	28	55	121	133	75	35	16	38	ankdd1a
ENSXETG00000006662	3008	79	106	106	131	131	341	1273	1504	866	439	313	321	plekho2
ENSXETG00000006663	2775	10	13	5	23	28	40	216	244	148	84	55	68	pcsk6
ENSXETG00000006665	714	27	60	41	224	200	463	530	601	362	201	159	169	tp53inp1
ENSXETG00000006666	3155	151	241	156	337	293	301	558	635	386	365	205	284	ints8
ENSXETG00000006669	1675	191	243	191	308	213	938	598	704	431	686	513	571	plekhf2
ENSXETG00000006675	1980	285	373	336	442	367	656	234	277	190	546	408	432	smpd3
ENSXETG00000006676	2622	701	989	829	743	553	885	2172	2567	1507	692	558	517	prmt7
ENSXETG00000006678	1134	264	380	334	321	249	402	522	625	392	325	231	261	slc7a6os
ENSXETG00000006680	1936	174	209	178	171	147	510	533	667	374	228	123	109	slc7a6
ENSXETG00000006690	2802	196	281	207	308	272	513	505	610	321	336	253	307	scyl2
ENSXETG00000006691	1792	61	96	87	447	337	917	484	625	320	643	492	520	tcp11l2
ENSXETG00000006695	3750	783	1043	915	1273	949	2013	1575	1827	1002	991	666	750	apaf1
ENSXETG00000006696	3096	27	62	61	62	38	100	489	608	346	177	138	135	dagla
ENSXETG00000006697	2812	1021	1365	1200	4136	3297	13656	3068	3258	2002	10130	7089	8039	egr1
ENSXETG00000006700	5244	403	606	507	1970	1514	1734	2071	2451	1417	2069	1555	1604	kdm3b
ENSXETG00000006702	3432	471	723	638	2145	1643	2528	2166	2443	1425	3343	2280	2559	myrf
ENSXETG00000006703	1423	326	539	462	691	533	197	1201	1378	847	197	116	137	fen1
ENSXETG00000006704	1191	425	626	477	890	676	1932	368	464	289	1538	1061	1281	sigirr
ENSXETG00000006705	1780	469	725	613	504	401	646	882	1013	584	410	293	310	ctbp2
ENSXETG00000006706	2463	1196	1747	1454	4037	3039	3186	3156	3460	2130	3809	2707	2811	pkp3
ENSXETG00000006707	3033	17	30	18	31	21	68	247	263	168	67	45	52	b4galnt4
ENSXETG00000006708	4953	77	107	89	233	160	289	313	402	221	240	172	201	fam53c
ENSXETG00000006710	2278	150	235	185	330	249	402	381	448	243	145	89	135	cdc25c
ENSXETG00000006711	1882	75	97	96	266	187	440	2049	2372	1425	743	524	571	ifitm3
ENSXETG00000006712	984	39	49	41	83	75	88	51	65	51	119	68	91	gfra3
ENSXETG00000006714	1923	20	26	21	7	7	2	28	27	18	11	6	6	hspa1l
ENSXETG00000006715	3198	559	725	617	1103	842	1096	1550	1729	1121	1364	958	1010	cpsf7
ENSXETG00000006716	2107	198	313	301	314	244	365	687	773	447	336	220	254	cdc23
ENSXETG00000006717	438	657	898	750	884	685	1298	707	799	436	899	617	726	cenpv
ENSXETG00000006720	8670	1799	2578	2139	5496	4158	6027	8096	9022	5504	6521	4555	5062	ncor1
ENSXETG00000006721	3633	95	162	134	419	358	534	733	835	481	78	54	71	kif20a
ENSXETG00000006722	995	66	124	69	89	52	79	88	134	93	40	35	46	cxorf40a
ENSXETG00000006723	2013	205	269	256	429	301	490	974	1049	645	967	745	824	sytl4
ENSXETG00000006724	396	1120	1595	1367	1214	963	1450	1205	1421	844	786	587	626	sdhaf2
ENSXETG00000006725	1161	1	1	0	78	60	2	12	21	8	5	5	3	ppp1r32
ENSXETG00000006726	2990	360	539	427	1047	843	999	1354	1476	894	1437	1084	1230	brd8
ENSXETG00000006728	1380	3	5	5	17	18	15	71	73	55	37	24	35	srpx2
ENSXETG00000006729	1035	7	13	12	7	7	11	80	84	57	16	18	12	gpr174
ENSXETG00000006730	1521	0	0	0	0	0	0	0	0	0	0	0	0	
ENSXETG00000006731	946	2110	3086	2694	1159	844	1038	2003	2433	1437	1184	762	881	nhp2
ENSXETG00000006732	1317	0	0	0	0	0	0	5	6	7	4	1	0	
ENSXETG00000006733	1638	11	24	17	176	117	75	428	456	279	242	180	222	n4bp3
ENSXETG00000006735	2967	179	271	240	1065	842	623	1276	1390	861	169	137	125	incenp
ENSXETG00000006736	1672	250	334	272	254	189	259	590	691	383	246	177	193	b4galt7
ENSXETG00000006737	710	11766	17422	14780	24438	18323	22369	12305	14273	8203	23630	16657	18272	ftmt
ENSXETG00000006739	1007	1801	2732	2271	1335	1002	1049	2535	2887	1771	801	592	642	tmed9
ENSXETG00000006740	2129	2	3	1	5	7	5	48	59	31	179	138	129	best1
ENSXETG00000006741	1744	1743	2416	2066	1580	1218	3165	1855	2374	1337	1500	1086	1233	lman2
ENSXETG00000006742	1105	78	123	89	342	296	888	331	376	205	713	506	554	ENSXETG00000006742
ENSXETG00000006744	2500	3731	5200	4452	6131	4833	8296	5427	6226	3578	6698	4739	5316	cltb
ENSXETG00000006745	996	2	0	0	5	5	8	16	14	16	13	7	7	adora2b
ENSXETG00000006746	1380	378	537	430	975	737	967	110	109	70	1034	777	811	slc52a3
ENSXETG00000006748	2958	350	488	402	826	598	887	1134	1364	791	727	568	619	specc1
ENSXETG00000006750	1032	2	0	1	1	1	1	0	8	2	6	3	3	ptchd1
ENSXETG00000006754	1980	0	1	0	6	4	8	30	37	18	39	23	18	ppef1
ENSXETG00000006756	3717	62	125	129	170	106	214	585	729	425	195	117	134	phka1p1

ENSXETG00000006758	1333	848	1273	1094	690	528	427	1526	1875	1055	365	267	312	wdr77
ENSXETG00000006760	963	0	0	1	0	0	0	4	0	1	0	0	1	
ENSXETG00000006761	2361	17	38	32	152	125	421	1683	1980	1137	559	407	457	dab2
ENSXETG00000006766	1978	30	49	35	76	49	102	362	391	226	343	224	261	c9
ENSXETG00000006768	2815	29	67	49	112	106	163	235	323	170	168	150	163	ENSXETG00000006768
ENSXETG00000006776	1647	13	33	21	43	25	48	48	54	36	91	47	61	ENSXETG00000006776
ENSXETG00000006790	1658	33	60	53	154	138	107	53	77	31	22	21	25	tctex1d1
ENSXETG00000006793	2316	0	0	2	0	1	0	8	11	5	5	2	5	pdzrn4
ENSXETG00000006794	3421	1404	1843	1639	1918	1429	3211	2099	2692	1395	3392	2395	2779	gxylt1
ENSXETG00000006795	918	55	62	57	66	68	174	216	200	127	127	82	87	yaf2
ENSXETG00000006796	1422	283	380	307	308	259	426	764	958	529	416	328	351	zcrb1
ENSXETG00000006797	4360	4	10	9	89	85	219	961	1142	675	273	201	221	prickle1
ENSXETG00000006798	291	1	0	0	3	4	1	3	5	5	9	7	6	btc
ENSXETG00000006802	5712	0	0	1	1	0	7	11	23	9	4	1	7	adamts20
ENSXETG00000006803	3044	156	190	132	172	140	237	355	530	255	274	205	245	cdca4
ENSXETG00000006809	2159	250	361	290	210	147	205	269	393	248	341	235	274	pus7l
ENSXETG00000006811	2109	134	199	133	161	101	311	474	634	342	288	181	243	irak4
ENSXETG00000006812	2765	1234	1649	1414	1156	963	2771	1598	1765	1065	747	506	635	mcoln1
ENSXETG00000006813	1667	15	19	24	39	26	77	30	43	21	92	61	74	tmem117
ENSXETG00000006816	2064	10	18	11	19	12	29	103	113	65	51	32	39	wscd1
ENSXETG00000006819	1299	0	1	1	3	0	3	2	3	2	5	2	6	ENSXETG00000006819
ENSXETG00000006821	1002	0	1	0	0	2	1	2	2	0	1	0	0	
ENSXETG00000006823	1497	526	821	669	803	642	1454	1200	1315	873	965	774	845	pi4kb
ENSXETG00000006824	6495	0	1	0	17	13	12	15	19	12	34	23	32	cacna1g
ENSXETG00000006825	2619	1	0	0	2	3	4	3	5	6	2	1	3	atp4a
ENSXETG00000006826	1695	879	1238	1109	4206	3142	9318	881	996	693	4197	3005	3255	acsf2
ENSXETG00000006827	531	787	1119	957	1119	871	1093	1236	1456	921	811	587	655	tmem147
ENSXETG00000006828	348	213	273	242	277	211	320	441	478	304	239	163	192	casp9
ENSXETG00000006829	2410	1	6	1	566	415	141	71	95	56	5918	4092	4461	cela2a
ENSXETG00000006830	2140	109	167	135	131	124	274	342	420	240	230	139	166	dnajc16
ENSXETG00000006832	1089	0	0	0	1	0	0	6	7	3	1	3	4	chad
ENSXETG00000006834	633	0	1	0	0	0	1	0	1	2	0	0	0	ENSXETG00000006834
ENSXETG00000006835	1701	2060	2995	2591	2873	2188	2467	1713	1779	1108	1867	1233	1387	lrrc59
ENSXETG00000006836	855	0	2	0	0	0	1	0	0	0	0	0	0	fam43b
ENSXETG00000006837	1065	323	486	413	537	442	846	781	838	566	553	380	385	mul1
ENSXETG00000006838	603	100	180	156	218	169	145	465	501	285	245	151	187	rab37
ENSXETG00000006839	3198	276	375	335	413	302	480	721	822	547	549	356	406	xylt2
ENSXETG00000006840	1589	3431	5267	4311	15053	11650	12742	7340	8182	5213	13265	9392	10077	slc9a3r1
ENSXETG00000006841	11247	9	11	16	110	95	29	85	74	47	90	59	60	ENSXETG00000006841
ENSXETG00000006842	2454	489	770	646	775	575	1898	1783	1938	1264	916	645	725	tmem104
ENSXETG00000006844	2676	1	0	0	0	1	0	9	10	9	1	4	3	grin2c
ENSXETG00000006846	1401	0	6	1	12	2	12	15	32	10	10	3	5	ush1g
ENSXETG00000006848	1068	0	0	0	0	0	0	0	0	1	0	0	1	fads6
ENSXETG00000006849	1665	1	1	1	3	5	3	3	6	2	6	2	1	ENSXETG00000006849
ENSXETG00000006863	3110	1	6	2	11	2	7	86	111	78	38	29	22	has2
ENSXETG00000006865	3068	71	93	94	2337	1782	312	1920	2358	1220	986	705	769	emp1
ENSXETG00000006872	4225	28	71	52	77	72	259	150	203	113	351	283	314	usp45
ENSXETG00000006876	1365	0	2	0	1	3	1	0	0	0	2	0	0	des.2
ENSXETG00000006882	3939	0	0	1	1	0	0	3	6	2	0	2	3	adamts13
ENSXETG00000006883	513	187	276	253	196	172	174	546	618	437	98	60	86	cacfd1
ENSXETG00000006884	1515	24	26	28	97	98	119	785	882	550	103	53	69	slc2a6
ENSXETG00000006887	1287	1	3	2	9	8	13	579	631	416	85	80	77	itih3
ENSXETG00000006888	2354	88	108	89	207	170	289	411	470	251	326	239	251	nek4
ENSXETG00000006889	1176	5	3	6	30	20	38	2	5	1	53	34	41	ENSXETG00000006889
ENSXETG00000006890	884	887	1285	1129	1733	1327	3574	1271	1376	912	1264	912	1058	wbp2nl
ENSXETG00000006892	2456	5204	7494	6407	6350	4668	5808	5511	6445	3712	5174	3634	3963	aco2
ENSXETG00000006893	555	378	563	439	274	219	291	781	877	558	228	172	209	phf5a
ENSXETG00000006894	2736	909	1251	1091	1838	1331	2005	1661	1915	1161	1820	1232	1382	gtpbp1
ENSXETG00000006895	4314	131	159	158	190	150	333	473	676	407	392	290	319	josd1
ENSXETG00000006896	351	302	468	387	276	210	316	437	467	270	297	267	265	tomm22
ENSXETG00000006897	1010	12	18	13	110	63	39	86	111	59	25	16	23	cby1
ENSXETG00000006898	3624	2684	3722	2972	3494	2702	5539	3538	4360	2422	2744	2044	2235	rhoc
ENSXETG00000006900	2408	1017	1460	1178	2765	2111	4051	2745	2990	1860	9583	6657	7576	ddx17
ENSXETG00000006901	2982	31	29	24	107	94	180	980	1140	657	11	6	9	mov10
ENSXETG00000006902	1671	48	78	72	178	132	308	182	270	155	179	122	135	st7l
ENSXETG00000006905	1599	129	191	138	376	289	277	781	948	553	257	158	206	dnal4
ENSXETG00000006906	3676	0	0	2	13	7	2	20	27	14	24	18	17	nptxr
ENSXETG00000006908	993	19	34	14	254	189	338	256	342	183	585	425	447	wnt2b
ENSXETG00000006909	1905	28	25	29	185	178	390	386	439	243	458	308	398	cttnbp2nl
ENSXETG00000006910	1367	84	71	90	113	99	280	435	547	303	123	98	120	cbx6
ENSXETG00000006911	738	8	20	14	24	34	65	30	31	37	191	131	149	cbx7
ENSXETG00000006912	2215	37	49	46	104	70	150	751	849	488	267	192	203	pdgfb
ENSXETG00000006914	1285	98403	147491	128267	158499	121424	179344	164308	187195	110929	369128	258200	283594	rpl3
ENSXETG00000006915	1406	3	7	7	15	12	24	122	145	78	28	33	31	syngr1
ENSXETG00000006916	1503	247	350	285	348	244	439	566	593	377	312	216	244	tab1
ENSXETG00000006917	1181	0	3	1	3	1	2	10	2	10	3	0	0	myl1
ENSXETG00000006918	2031	136	257	199	389	322	580	735	890	462	530	365	403	lancl1
ENSXETG00000006919	4461	0	0	0	0	0	0	11	17	9	0	0	0	cps1
ENSXETG00000006922	1783	788	1199	1031	1469	1081	2465	2574	3031	1700	1805	1214	1411	ss18
ENSXETG00000006924	957	1545	2246	1815	463	315	437	174	195	140	46	43	46	XB-GENE-5798855
ENSXETG00000006926	987	7	5	1	51	32	29	66	49	33	9	10	10	camk4
ENSXETG00000006929	621	1202	1702	1421	923	772	2715	337	397	243	230	158	192	stard4
ENSXETG00000006930	777	5	11	4	47	37	85	510	548	362	159	98	108	kctd1
ENSXETG00000006932	2580	8	9	13	81	50	79	899	1107	644	200	116	130	cdh2
ENSXETG00000006935	2019	758	1110	946	591	464	1034	130	144	79	530	397	403	slc5a9
ENSXETG00000006939	2486	840	1166	1028	1286	954	1533	2688	3039	1797	1487	1035	1250	ftsj3
ENSXETG00000006941	1405	0	0	0	0	0	0	0	0	0	0	0	0	ENSXETG00000006941
ENSXETG00000006944	867	0	0	0	0	0	0	0	0	0	0	0	0	
ENSXETG00000006955	3864	34	57	56	104	64	125	363	372	252	227	131	156	pard3
ENSXETG00000006958	2020	0	0	0	0	0	1	3	4	1	5	5	3	irx1
ENSXETG00000006961	1581	389	541	456	344	275	632	622	851	434	344	218	305	trappc12
ENSXETG00000006962	1402	616	876	762	620	492	909	878	1123	606	428	350	349	tssc1
ENSXETG00000006965	4682	43	39	32	288	212	452	4032	4665	2646	517	352	420	pxdn
ENSXETG00000006966	2235	0	0	0	0	0	0	0	0	0	0	0	0	
ENSXETG00000006969	1411	105	151	136	230	176	333	513	622	369	479	377	455	hs3st1
ENSXETG00000006970	2862	3693	5192	4552	8870	6582	10689	13006	14687	8836	9417	6758	7328	jup
ENSXETG00000006976	963	0	1	0	0	0	0	0	0	0	0	0	0	
ENSXETG00000006978	1266	286	383	315	718	435	380	869	978	565	592	431	489	casp2
ENSXETG00000006979	4275	0	0	0	0	0	1	25	25	15	0	0	0	a2ml1
ENSXETG00000006980	4179	90	126	112	74	67	47	33	36	23	106	68	85	ENSXETG00000006980
ENSXETG00000006986	4592	231	273	292	579	445	549	701	779	496	749	548	589	rad50
ENSXETG00000006987	2326	49	87	74	120	113	143	617	759	408	262	177	204	p4ha2
ENSXETG00000006995	1665	29	45	35	66	40	118	197	243	147	126	86	99	nxpe3
ENSXETG00000006998	1938	142	191	173	152	142	107	143	221	134	693	487	580	ENSXETG00000006998
ENSXETG00000006999	1248	3	9	11	0	0	0	1	6	1	0	0	0	zpld1
ENSXETG00000007010	1676	7012	9839	8120	9126	7157	9191	18728	21798	12532	10570	7248	8033	ywhaq
ENSXETG00000007011	2467	88	129	104	131	115	174	226	291	154	127	99	100	taf1b
ENSXETG00000007012	1916	0	0	0	1	1	2	5	9	3	2	1	0	grhl1
ENSXETG00000007013	3458	1689	2606	2141	2802	2190	2729	3635	4198	2422	2282	1604	1812	klf11
ENSXETG00000007023	366	3	2	6	1	3	11	37	48	33	21	10	14	card11
ENSXETG00000007024	1176	0	0	0	0	0	0	0	0	0	0	0	0	

ENSXETG00000007026	1991	910	1325	1145	1834	1312	2234	2547	2924	1825	1897	1278	1433	prkaa1
ENSXETG00000007027	1215	0	0	0	0	0	0	0	1	0	1	0	1	
ENSXETG00000007029	1865	154	202	197	249	201	607	700	786	482	570	430	454	ttc33
ENSXETG00000007030	4447	1041	1508	1275	1396	1032	1969	2292	2676	1662	1797	1262	1394	ptger4
ENSXETG00000007037	1467	6	6	7	27	29	45	13	14	6	50	35	39	ENSXETG00000007037
ENSXETG00000007038	2606	12	14	9	94	63	147	338	358	200	106	93	102	mef2c
ENSXETG00000007039	837	56	58	45	217	161	163	202	277	149	88	60	59	cetn3
ENSXETG00000007042	1167	5	10	11	56	32	60	73	79	51	13	8	4	ren
ENSXETG00000007043	834	11	20	18	57	50	70	52	70	43	98	69	68	mblac2
ENSXETG00000007044	2405	343	519	472	1263	990	1723	1999	2017	1278	3269	2388	2462	MGC147490
ENSXETG00000007046	3120	178	234	211	371	272	727	551	645	400	831	646	663	plekha6
ENSXETG00000007049	375	73	124	79	54	40	15	140	146	97	22	12	21	XB-GENE-5824086
ENSXETG00000007054	2973	0	3	1	10	8	7	21	22	12	35	20	36	ncam2
ENSXETG00000007058	2532	6	7	10	7	5	9	184	196	101	7	10	5	ENSXETG00000007058
ENSXETG00000007068	1638	3	3	4	0	0	0	33	40	21	0	0	0	prf1
ENSXETG00000007070	1008	1	1	0	2	0	1	1	1	3	1	1	0	hmgcll1
ENSXETG00000007071	1353	2	3	4	54	28	22	226	289	160	87	75	117	bmp5
ENSXETG00000007072	459	6	11	5	15	12	51	31	48	34	68	36	64	ENSXETG00000007072
ENSXETG00000007073	1540	94	151	134	346	268	321	449	460	328	518	383	391	ccdc82
ENSXETG00000007075	3177	11	12	18	108	101	122	494	514	324	221	147	172	maml2
ENSXETG00000007076	939	1075	1584	1445	1462	1176	1621	2519	2729	1727	1257	908	998	cwc15
ENSXETG00000007078	1341	38	87	50	178	127	170	535	555	376	272	187	220	endod1
ENSXETG00000007079	1596	266	391	350	668	490	1223	688	791	475	643	429	523	sesn3
ENSXETG00000007080	4882	399	547	423	885	631	1037	1214	1469	883	1276	923	1105	fam76b
ENSXETG00000007081	1479	86	141	119	232	146	239	348	360	213	212	156	171	cep57
ENSXETG00000007082	1716	270	400	383	355	345	611	661	717	442	429	300	302	mtmr2
ENSXETG00000007084	5053	1155	1690	1535	3028	2247	2434	7584	8809	5256	4484	3182	3616	vcl
ENSXETG00000007086	1014	0	0	0	0	0	0	0	0	0	1	0	0	
ENSXETG00000007088	1664	242	352	304	258	194	322	572	623	353	327	241	247	clp1
ENSXETG00000007089	3218	3136	4408	3771	5768	4320	9079	8980	10572	6254	7131	5019	5741	ctnnd1
ENSXETG00000007091	2037	599	869	760	411	324	368	935	1084	602	417	310	347	heatr3
ENSXETG00000007092	1563	191	235	182	158	122	392	373	477	262	195	116	163	tiprl
ENSXETG00000007094	2290	111	127	89	88	65	107	163	210	108	125	70	81	fastkd3
ENSXETG00000007096	871	3624	5435	4631	7190	5053	5703	1940	2272	1387	5265	3693	3954	tmem35b
ENSXETG00000007097	3508	0	0	1	2	4	7	14	12	12	9	7	10	dlgap3
ENSXETG00000007102	2536	198	288	215	355	298	296	1107	1445	792	412	292	299	hnrnpr
ENSXETG00000007103	487	5366	8050	7051	6386	4778	5766	4931	5464	3353	3576	2577	2785	atpif1
ENSXETG00000007104	1336	15	5	6	110	77	105	883	964	652	178	136	139	sdc3
ENSXETG00000007107	1674	226	279	271	452	297	884	747	843	517	665	429	549	ENSXETG00000007107
ENSXETG00000007110	2595	390	549	456	630	450	4641	1877	2369	1323	1059	734	848	tmem184c
ENSXETG00000007111	3780	20	21	20	186	144	292	999	1225	668	428	269	344	ednra
ENSXETG00000007114	3948	0	0	0	0	1	0	0	2	0	4	3	3	cd163
ENSXETG00000007116	1353	1	1	2	54	35	2	6	2	3	3	2	2	ttc29
ENSXETG00000007119	1029	722	1048	895	486	359	460	678	814	491	404	266	335	slc10a7
ENSXETG00000007124	1110	0	0	0	0	0	0	0	0	0	1	0	0	
ENSXETG00000007126	2295	18	15	15	27	23	24	30	66	19	25	12	21	ENSXETG00000007126
ENSXETG00000007129	1338	0	0	0	1	0	1	3	2	3	2	1	3	ENSXETG00000007129
ENSXETG00000007130	398	278	468	396	307	227	266	324	350	228	292	207	234	c2orf76
ENSXETG00000007136	921	0	0	0	0	0	0	0	2	0	0	0	0	XB-GENE-5794175
ENSXETG00000007137	5331	2	4	3	3	0	3	5	20	7	0	1	2	tdrd15
ENSXETG00000007139	4398	216	342	265	657	498	1049	755	801	502	1883	1289	1441	oplah
ENSXETG00000007140	2839	5233	7782	6457	6560	4841	8928	2231	2352	1480	7609	5493	5976	gpt2
ENSXETG00000007146	774	4	18	7	112	83	90	124	146	85	113	75	80	lrrc18
ENSXETG00000007152	3372	0	0	0	2	0	0	3	7	3	8	7	0	ENSXETG00000007152
ENSXETG00000007155	3963	651	1010	833	1113	862	1344	1707	1903	1099	1762	1240	1366	stk11ip
ENSXETG00000007160	2925	0	1	2	3	0	6	6	5	4	1	1	2	slc4a3
ENSXETG00000007162	3456	61	91	65	341	267	227	418	496	256	68	71	59	ncapg2
ENSXETG00000007163	402	1	2	2	15	10	21	103	121	54	47	25	34	cplx1
ENSXETG00000007164	3965	1055	1582	1287	1268	985	3309	1354	1604	925	1706	1251	1435	gne
ENSXETG00000007165	6087	17	34	23	186	125	247	1298	1369	882	247	163	197	myo10.2
ENSXETG00000007167	3047	912	1339	1190	3001	2243	5072	3426	3910	2221	4060	2961	3231	esyt2
ENSXETG00000007168	1062	10814	15047	13243	6789	5257	10161	7382	8327	5196	4694	3400	3724	clta
ENSXETG00000007169	969	0	0	0	0	1	4	9	7	4	5	1	4	ENSXETG00000007169
ENSXETG00000007171	2607	0	1	2	12	7	4	0	2	0	20	16	20	ENSXETG00000007171
ENSXETG00000007173	2337	0	0	0	0	0	0	0	0	0	0	0	0	
ENSXETG00000007174	252	96	148	145	147	104	230	114	112	85	176	115	146	swi5
ENSXETG00000007175	1494	858	1334	1129	1210	945	1353	1680	2067	1197	806	578	618	ubl4a
ENSXETG00000007176	2332	0	0	1	1	0	0	4	8	2	20	21	8	fcn2
ENSXETG00000007177	3639	70	87	56	270	182	380	466	506	318	622	456	509	iqsec2
ENSXETG00000007178	5546	931	1442	1181	2431	1757	3216	4083	4552	2715	3420	2314	2599	kdm5c
ENSXETG00000007179	3387	60	88	62	348	286	296	1380	1527	966	699	487	515	sema3f
ENSXETG00000007180	2004	0	3	0	2	2	5	13	29	11	17	13	15	flrt1
ENSXETG00000007182	469	59	62	67	51	46	68	65	85	53	109	63	81	macrod1
ENSXETG00000007183	579	14	15	11	42	47	52	156	164	126	71	45	43	vegfb
ENSXETG00000007184	923	43	78	60	58	56	96	122	112	80	79	63	66	rpusd1
ENSXETG00000007187	1407	4	6	9	4	8	8	68	89	43	10	12	6	ENSXETG00000007187
ENSXETG00000007188	1965	0	0	1	225	146	6	0	0	0	64	37	38	thdl20
ENSXETG00000007189	1914	1605	2371	1946	1941	1403	1648	3611	4104	2444	2820	1955	2180	stip1
ENSXETG00000007191	2200	478	632	497	837	588	1409	738	842	508	1285	949	1020	narfl
ENSXETG00000007193	2285	876	1288	1114	1384	1033	1999	2513	2648	1705	1779	1242	1261	ccdc22
ENSXETG00000007195	5877	0	1	1	2	1	5	1	0	0	0	0	0	cacna1f
ENSXETG00000007196	741	63	95	94	143	88	176	102	134	60	82	82	73	trpt1
ENSXETG00000007197	687	359	543	438	377	250	334	355	463	247	251	193	194	dnajc4
ENSXETG00000007198	1106	591	809	684	318	218	256	626	752	414	344	240	241	nudt22
ENSXETG00000007199	632	317	476	449	374	314	604	3161	3547	2273	783	582	644	ppp1r14b
ENSXETG00000007200	1403	237	335	297	422	320	727	363	423	221	679	483	521	haghl
ENSXETG00000007201	3612	409	563	496	936	740	1640	1787	2086	1311	2270	1541	1749	plcb3
ENSXETG00000007202	2253	1486	2220	1882	1740	1388	4097	1614	1846	1122	2174	1590	1662	hagh
ENSXETG00000007204	2796	3	2	0	0	1	1	5	1	2	0	0	0	
ENSXETG00000007206	825	0	0	0	0	0	0	0	0	0	0	0	0	
ENSXETG00000007209	630	99	125	119	151	106	351	176	240	115	169	133	163	XB-GENE-5994048
ENSXETG00000007210	1164	2	2	1	1	0	3	17	20	12	5	3	7	kcnk4
ENSXETG00000007211	2742	1669	2468	1971	997	763	1607	1503	1651	1017	801	569	642	esrra
ENSXETG00000007214	996	0	0	2	9	2	4	80	98	65	16	16	20	hs3st6
ENSXETG00000007216	1453	629	915	743	811	605	2881	1430	1565	982	2891	2032	2339	noxo1
ENSXETG00000007217	2539	539	792	633	751	594	413	1296	1519	875	728	506	544	tbl3
ENSXETG00000007218	490	633	879	783	411	327	323	646	789	446	436	339	334	trmt112
ENSXETG00000007220	895	146124	217563	183884	210479	159457	190737	177491	195281	118919	378944	265719	282823	rps2
ENSXETG00000007221	1319	2	6	6	4	6	12	6	7	2	24	16	25	rpl3l
ENSXETG00000007222	1713	1	2	0	1	3	2	0	0	0	0	0	1	tex2.1
ENSXETG00000007223	2580	1032	1530	1298	1580	1229	2840	1193	1382	791	2210	1476	1712	ENSXETG00000007223
ENSXETG00000007227	6898	20	29	28	138	127	139	215	290	148	400	234	365	syne2
ENSXETG00000007230	2542	415	592	414	314	230	302	667	751	414	424	285	330	znf346
ENSXETG00000007231	3713	570	925	819	795	575	1766	246	262	156	1283	906	980	fgfr4
ENSXETG00000007233	1963	47	77	69	124	87	160	310	380	196	216	149	178	nsd1
ENSXETG00000007234	1027	469	684	586	793	610	706	876	981	590	622	412	524	rab24
ENSXETG00000007235	1095	5795	8336	7131	5850	4397	6089	3900	4402	2554	5462	3649	4223	prelid1
ENSXETG00000007236	2214	190	250	194	637	507	674	945	1073	620	583	432	451	mxd3
ENSXETG00000007237	1744	3	2	0	107	95	27	202	269	132	98	110	102	zfyve28
ENSXETG00000007238	2331	71	124	117	283	253	318	402	496	287	454	320	353	fam193b
ENSXETG00000007240	3952	1575	2117	1709	2266	1814	5497	6516	7528	4499	4917	3421	3940	pdlim7

ENSXETG00000007241	2528	237	347	271	1693	1315	2274	7578	8352	5091	4772	3271	3641	dbn1
ENSXETG00000007243	3486	327	490	425	1228	929	1043	1562	1945	1126	1033	735	800	grk6
ENSXETG00000007245	1800	8	16	9	54	46	23	56	70	47	26	18	22	f12
ENSXETG00000007246	1179	11	18	12	39	28	57	33	47	27	53	52	43	adra2a
ENSXETG00000007251	1455	60	96	70	109	90	165	213	229	150	222	152	175	zmat3
ENSXETG00000007252	3431	66	101	96	51	53	113	207	270	146	96	71	75	pik3ca
ENSXETG00000007253	6462	472	742	571	1203	913	1352	1318	1598	958	2517	1748	1968	syne2.2
ENSXETG00000007254	753	0	0	0	0	3	0	5	3	3	0	0	0	kcnmb3
ENSXETG00000007256	1695	467	616	542	444	305	446	631	728	416	453	354	374	znf639
ENSXETG00000007257	2047	78	125	120	198	143	765	97	113	86	696	505	570	esr2
ENSXETG00000007258	3840	1311	1905	1674	1262	959	1699	1097	1217	696	1215	901	964	mfn1
ENSXETG00000007259	1731	129	188	141	401	293	720	2467	2765	1777	712	456	535	gnb4
ENSXETG00000007260	1290	542	771	749	873	663	714	2780	3256	1984	812	545	589	actl6a
ENSXETG00000007263	1206	287	399	344	460	318	514	530	654	380	337	227	225	gtf2h4
ENSXETG00000007264	657	1727	2460	1948	2108	1527	2239	1593	1878	1056	2149	1526	1698	ndufb5
ENSXETG00000007265	2595	2	3	2	14	15	42	60	66	30	64	63	65	usp13
ENSXETG00000007266	1452	137	220	197	240	178	390	359	412	235	470	341	389	ENSXETG00000007266
ENSXETG00000007268	717	0	0	0	1	0	0	0	1	1	0	0	0	
ENSXETG00000007272	1215	17	33	23	51	32	45	65	65	41	92	50	56	rarb
ENSXETG00000007273	3621	42	98	56	158	148	337	286	371	140	391	242	281	ENSXETG00000007273
ENSXETG00000007274	954	0	0	0	0	0	0	0	0	0	0	0	0	
ENSXETG00000007278	1863	99	122	133	174	155	234	258	354	218	276	189	221	senp1
ENSXETG00000007279	3717	1290	1842	1673	2705	2047	4892	1371	1599	960	2659	1844	2183	pfkm
ENSXETG00000007282	5097	299	363	327	921	734	1174	2643	3093	1717	1382	1018	1185	top2b
ENSXETG00000007283	2240	106	148	132	129	104	154	268	309	188	177	117	134	larp4
ENSXETG00000007284	4602	429	600	518	1269	896	2242	2731	3210	2032	1433	1002	1110	dip2b
ENSXETG00000007286	2260	263	380	319	962	651	726	1288	1588	889	2260	1635	1663	ica1
ENSXETG00000007287	984	25	47	41	89	56	134	223	209	140	126	105	99	c17orf75
ENSXETG00000007288	3534	127	151	134	360	257	587	589	647	351	537	392	416	ENSXETG00000007288
ENSXETG00000007289	1599	153	239	191	262	223	450	447	480	282	568	395	465	glcci1
ENSXETG00000007291	906	295	413	325	280	226	141	491	641	322	159	99	109	rpa3
ENSXETG00000007293	2649	142	190	174	256	176	337	419	538	324	398	282	345	mios
ENSXETG00000007294	1693	9	12	15	7	9	11	0	7	1	12	10	16	tmprss12
ENSXETG00000007295	1071	265	352	347	524	394	811	870	977	664	475	333	412	c1galt1
ENSXETG00000007299	1179	403	659	503	575	378	686	1109	1365	777	966	690	775	atf1
ENSXETG00000007300	852	2267	3409	2844	892	680	1121	549	598	351	486	366	393	ENSXETG00000007300
ENSXETG00000007303	5819	2154	3062	2732	4676	3730	4930	6035	7012	4000	13649	9420	10419	rbm5
ENSXETG00000007304	1492	0	0	0	0	0	0	2	0	0	0	0	0	
ENSXETG00000007305	1635	226	299	228	322	263	496	483	490	296	367	254	302	mon1a
ENSXETG00000007306	4107	882	1343	1148	1898	1438	1726	2845	3225	2033	2221	1567	1752	mst1r
ENSXETG00000007309	1064	3	4	1	1	2	7	20	22	28	6	4	10	ENSXETG00000007309
ENSXETG00000007310	2237	1657	2386	1972	3257	2461	3808	4736	5145	3129	3347	2319	2569	ppp2r1a
ENSXETG00000007311	1164	1	1	1	2	0	1	18	23	10	0	1	4	camkv
ENSXETG00000007312	2316	103	141	119	199	162	245	488	548	336	330	246	240	sema3f
ENSXETG00000007313	975	1	1	1	0	0	0	2	4	1	1	1	2	ENSXETG00000007313
ENSXETG00000007315	2515	0	0	0	0	1	0	0	0	0	0	0	0	
ENSXETG00000007316	3921	134	163	152	2073	1507	632	588	728	446	1284	878	968	slc38a3
ENSXETG00000007317	1285	271	383	339	623	494	1205	2869	3261	2122	1545	1014	1172	gnai2
ENSXETG00000007318	6184	1919	2693	2257	2867	2262	10492	7294	8624	4653	5811	4295	4881	gns
ENSXETG00000007319	1506	5	5	7	31	31	38	205	214	143	60	61	57	apmap
ENSXETG00000007320	4965	1226	1734	1454	2621	1898	2220	6809	7868	4779	3002	2161	2461	sema3b
ENSXETG00000007321	1485	29	51	44	77	66	226	1122	1455	829	190	145	141	ENSXETG00000007321
ENSXETG00000007322	5267	591	855	727	965	782	1320	1436	1657	1046	1371	963	1094	tbk1
ENSXETG00000007323	2916	60	67	49	203	166	263	409	473	294	228	171	205	helb
ENSXETG00000007324	3297	31	45	46	135	108	141	327	328	182	84	77	74	grip1
ENSXETG00000007325	3116	994	1451	1260	1055	723	1122	1240	1376	818	924	645	746	ifrd2
ENSXETG00000007327	723	43	82	64	43	28	70	186	234	136	69	50	51	nat6
ENSXETG00000007328	5552	1097	1561	1295	1829	1371	2641	2708	3268	1882	2147	1432	1745	cand1
ENSXETG00000007329	1347	131	216	177	512	365	704	1088	1343	747	335	260	289	hyal1
ENSXETG00000007330	639	233	344	325	563	414	602	1208	1428	883	504	336	413	hyal2
ENSXETG00000007331	324	247	430	361	276	182	377	365	445	263	214	130	148	tusc2
ENSXETG00000007332	1086	187	264	235	373	261	549	1100	1183	758	578	435	461	rassf1
ENSXETG00000007333	1779	97	170	139	194	145	329	695	797	502	283	199	210	dyrk2
ENSXETG00000007334	1341	2	3	0	98	79	9	12	15	11	4	0	3	zmynd10
ENSXETG00000007335	1526	13	9	15	21	13	12	26	30	18	23	25	19	tube1
ENSXETG00000007336	5277	27	47	37	211	157	359	1610	1948	1109	664	423	553	lama4
ENSXETG00000007339	1974	597	815	735	1093	870	792	2684	3142	1934	1051	683	864	hdac2
ENSXETG00000007345	684	3	8	4	124	90	12	83	82	54	29	20	17	cldn8.3
ENSXETG00000007347	5048	37	68	60	135	137	242	1067	1193	776	637	459	491	tiam1
ENSXETG00000007350	626	10388	15772	13311	15495	11703	16438	5790	6362	4032	15059	10822	11532	sod1
ENSXETG00000007351	6157	333	462	436	618	443	1148	1278	1416	826	1214	848	977	scaf4
ENSXETG00000007352	3245	18	17	27	81	65	51	455	514	316	145	104	90	hunk
ENSXETG00000007353	2196	76	99	79	562	449	243	4	2	4	11	13	16	tgm5
ENSXETG00000007354	2106	0	0	0	0	0	0	1	0	1	0	0	0	
ENSXETG00000007355	1350	0	0	0	0	0	0	0	0	0	0	0	0	
ENSXETG00000007356	2694	0	0	0	0	0	0	0	1	0	0	0	0	
ENSXETG00000007357	2073	0	1	0	0	0	0	4	8	2	0	0	0	
ENSXETG00000007358	2046	72	101	90	855	652	162	117	117	58	201	132	178	ENSXETG00000007358
ENSXETG00000007359	1086	131	183	153	235	182	317	453	510	303	334	270	240	ccndbp1
ENSXETG00000007360	5436	157	233	200	611	484	904	1336	1618	898	790	576	595	znfx1
ENSXETG00000007361	2220	316	418	368	931	683	1091	2592	2986	1767	1377	976	1037	elmo1
ENSXETG00000007362	1312	801	1190	989	619	476	758	454	573	314	752	507	587	slc35c2
ENSXETG00000007363	2261	5	7	14	17	15	33	112	102	52	61	47	49	rundc3a
ENSXETG00000007364	2748	13	18	16	16	10	99	78	108	49	27	23	26	slc4a1
ENSXETG00000007365	339	0	0	0	0	0	0	0	0	0	0	0	0	
ENSXETG00000007366	2550	66	102	100	98	85	181	922	1017	588	184	147	151	vav1
ENSXETG00000007367	21236	664	817	788	3509	2661	5166	4823	5730	3318	4950	3475	3857	macf1
ENSXETG00000007369	1543	23	45	27	143	121	94	93	122	60	44	42	39	dyx1c1
ENSXETG00000007370	3635	274	423	359	1128	877	3326	1653	1900	1152	1979	1363	1492	ccpg1
ENSXETG00000007371	457	2752	3968	3468	2840	2179	3246	2540	2844	1651	2518	1694	1851	ndufs5
ENSXETG00000007373	1904	431	530	528	231	155	442	469	548	338	207	161	170	azin2
ENSXETG00000007375	1904	76	105	95	405	267	215	455	526	315	767	514	633	rab27a
ENSXETG00000007376	486	5183	7225	6288	9036	6835	10589	9683	11222	6554	36674	25963	28179	rsl24d1
ENSXETG00000007381	1599	39	59	38	90	68	114	125	124	73	126	78	85	XB-GENE-5895314
ENSXETG00000007384	1644	43	52	48	437	334	53	177	193	96	118	81	105	spag6
ENSXETG00000007387	2279	187	246	226	412	317	620	856	935	560	943	676	656	bmi1
ENSXETG00000007390	1888	296	415	375	528	425	644	753	857	532	434	311	335	dnajc1
ENSXETG00000007391	5317	605	889	692	1305	916	1933	1330	1598	906	1967	1362	1461	mllt10
ENSXETG00000007392	4036	284	388	313	629	458	723	852	1080	586	669	455	538	klhl7
ENSXETG00000007393	2358	42	47	50	204	153	506	6748	7672	4464	727	459	587	gpnmb
ENSXETG00000007394	2337	19	29	16	20	18	37	104	96	58	54	38	27	skida1
ENSXETG00000007396	2178	280	352	303	463	339	393	156	165	90	277	180	220	igf2bp3
ENSXETG00000007397	1437	1520	2230	1888	1968	1561	2390	3351	3735	2504	2661	1801	2063	tra2a
ENSXETG00000007398	1162	222	322	288	237	196	400	523	604	345	430	333	372	ccdc126
ENSXETG00000007399	834	0	0	0	0	0	0	0	0	0	0	0	0	
ENSXETG00000007400	2727	0	0	0	1	1	0	1	2	1	0	0	0	stk31
ENSXETG00000007404	1812	263	351	364	191	152	264	300	352	225	237	165	194	mpp6
ENSXETG00000007405	1485	306	490	391	686	547	1536	899	1048	641	235	162	183	dfna5
ENSXETG00000007406	3365	189	248	210	339	242	388	398	462	302	288	197	226	osbpl3
ENSXETG00000007407	1723	3511	5070	4159	7226	5357	10986	9055	10595	6355	17711	12498	13838	eif3h
ENSXETG00000007408	660	0	0	0	0	0	0	0	0	0	0	0	0	

ENSXETG00000007409	2463	49	74	84	186	139	338	514	592	354	311	232	261 rapgef5
ENSXETG00000007411	1127	162	225	201	294	237	172	603	678	390	218	179	177 cdca7l
ENSXETG00000007412	13497	3	3	5	610	454	28	18	15	5	97	70	83 dnah11
ENSXETG00000007414	3339	489	767	650	828	597	525	1145	1306	703	767	534	585 rbl1
ENSXETG00000007416	1884	22	22	20	49	54	79	256	303	153	143	96	121 samhd1
ENSXETG00000007417	510	3	11	9	31	19	13	54	53	29	24	18	22 ENSXETG00000007417
ENSXETG00000007419	1720	926	1307	1138	867	585	1327	1327	1466	900	760	490	573 ergic3
ENSXETG00000007422	1586	738	1174	1039	1353	1031	3598	2127	2464	1376	1539	1124	1178 st3gal2.2
ENSXETG00000007423	2100	3	1	7	6	6	9	11	10	10	14	6	7 znf33a
ENSXETG00000007424	4965	3	3	10	377	300	14	79	95	55	601	407	453 fer1l4
ENSXETG00000007426	825	0	0	0	0	0	0	0	0	1	1	0	0 ENSXETG00000007426
ENSXETG00000007428	2834	797	1228	980	1851	1452	1668	7363	8833	5141	2181	1578	1719 cpne1
ENSXETG00000007429	2328	9	8	19	71	42	37	102	111	75	101	65	66 sp4
ENSXETG00000007430	1224	215	368	291	704	554	888	676	806	432	660	490	513 nfs1
ENSXETG00000007431	8972	5	9	4	82	48	149	243	304	170	397	284	322 adam22
ENSXETG00000007433	3798	1	0	2	3	0	2	27	51	18	1	3	6 ENSXETG00000007433
ENSXETG00000007434	2681	2755	3964	3422	5002	3912	4673	5587	6425	3738	8388	6069	6427 rbm39
ENSXETG00000007435	2158	234	327	249	681	515	939	918	1091	614	993	751	756 cldn12
ENSXETG00000007436	1283	151	209	207	198	159	180	207	264	126	187	117	133 gtpbp10
ENSXETG00000007437	459	845	1173	944	1406	1098	2326	1870	2337	1346	1172	815	957 ube2a
ENSXETG00000007438	2513	204	288	262	270	195	403	440	516	287	348	296	311 nkrf
ENSXETG00000007439	1127	494	677	622	1032	785	1322	1521	1853	1041	1107	810	813 nkap
ENSXETG00000007440	1496	286	384	358	618	533	908	979	1187	669	740	563	614 upf3b
ENSXETG00000007441	2679	1	6	3	85	53	17	25	34	14	16	7	10 cfap69
ENSXETG00000007442	3108	83	116	93	248	165	441	751	896	564	495	373	417 arhgap39
ENSXETG00000007443	1541	289	430	380	353	249	650	1691	2004	1241	842	565	635 sept6
ENSXETG00000007447	1324	6009	8603	7478	4016	3070	3766	5230	5876	3392	2082	1419	1556 pgk1
ENSXETG00000007448	1829	969	1466	1142	1102	847	1519	276	355	178	799	549	616 pter
ENSXETG00000007449	2482	817	1155	885	452	352	805	1168	1307	841	319	201	242 edem2
ENSXETG00000007450	774	0	0	1	0	0	5	12	6	2	8	3	3 c1ql3
ENSXETG00000007451	2478	1268	1873	1479	1967	1537	2608	4981	5780	3188	2503	1660	2070 rsu1
ENSXETG00000007453	10950	1710	2452	1978	1925	1454	1022	7	10	6	158	95	100 cubn
ENSXETG00000007454	689	924	1350	1113	1422	1062	2255	1879	2151	1278	1560	1061	1274 smim19
ENSXETG00000007455	2441	322	445	344	879	658	1258	1223	1420	796	912	688	735 trpc4ap
ENSXETG00000007458	1188	3	5	5	9	9	11	21	25	10	8	4	2 st8sia6
ENSXETG00000007460	2187	9761	14613	12667	16305	12312	36956	4906	5782	3357	51797	36666	40973 xpnpep2
ENSXETG00000007461	3689	220	268	217	733	606	832	971	1073	677	1151	842	975 sash3
ENSXETG00000007462	1777	576	868	728	1483	1155	2782	15799	17882	10602	2335	1699	1858 vim
ENSXETG00000007463	7494	786	1043	832	1421	1032	2569	1611	1926	1134	2159	1591	1777 zdhhc9
ENSXETG00000007465	2792	515	683	651	618	447	530	1073	1173	754	640	488	495 utp14a
ENSXETG00000007468	501	0	0	0	2	0	0	2	1	3	0	0	0
ENSXETG00000007469	1803	38	57	47	212	180	259	1073	1167	810	303	237	237 ENSXETG00000007469
ENSXETG00000007470	1543	392	632	578	501	431	506	1148	1231	724	182	131	173 gss
ENSXETG00000007473	2058	128	155	146	232	175	255	296	322	201	311	238	233 acss2.1
ENSXETG00000007480	2976	796	1193	936	1666	1310	1897	1707	2006	1136	1694	1173	1382 micu1
ENSXETG00000007481	1464	305	431	358	371	300	230	501	650	412	196	135	197 dnajc9
ENSXETG00000007482	231	2	5	8	1	1	5	10	14	10	5	1	2 msmb.3
ENSXETG00000007483	3147	117	161	144	261	186	178	228	246	131	124	84	95 dna2
ENSXETG00000007484	4417	471	643	579	1230	965	2877	2355	2761	1655	1626	1018	1217 washc2a
ENSXETG00000007489	3020	100	117	118	259	202	387	420	524	293	416	299	347 zfand4
ENSXETG00000007490	1860	60	65	77	68	49	174	574	817	443	151	112	135 alox5
ENSXETG00000007492	1266	0	0	0	0	1	0	0	2	0	1	0	2
ENSXETG00000007493	3468	20	33	30	35	27	25	15	25	10	19	12	15 tll2
ENSXETG00000007497	1598	0	0	0	0	0	0	0	0	0	0	0	0
ENSXETG00000007498	2268	65	113	93	98	71	144	254	315	188	107	88	98 smurf1
ENSXETG00000007499	975	12	18	11	35	33	42	48	59	40	50	35	46 vegfd
ENSXETG00000007500	2836	225	300	256	335	260	486	486	547	359	433	280	333 rnf216
ENSXETG00000007501	2544	120	158	153	182	159	273	2774	3300	2032	395	332	346 fscn1
ENSXETG00000007502	2079	95	127	104	101	75	187	220	266	158	114	77	100 fbxl18
ENSXETG00000007504	7473	127	175	151	962	655	1462	1677	1984	1143	2084	1471	1647 tnrc18
ENSXETG00000007509	2172	3587	5054	4338	2828	2121	2438	5580	6739	3744	1764	1275	1360 hspd1
ENSXETG00000007510	457	1850	2797	2455	1075	769	1006	2158	2612	1480	777	500	618 hspe1
ENSXETG00000007511	1197	964	1462	1183	934	820	1027	2090	2407	1689	771	571	680 tra2b
ENSXETG00000007512	426	0	0	0	0	0	0	0	0	0	0	0	0
ENSXETG00000007514	2856	123	199	196	280	194	290	658	736	470	297	258	255 ipo8
ENSXETG00000007515	13437	3	5	5	5	5	5	31	19	18	10	9	13 ENSXETG00000007515
ENSXETG00000007516	3088	0	1	1	2	1	0	4	5	3	2	4	5 caprin2
ENSXETG00000007518	2598	924	1315	1163	1690	1312	490	1894	2327	1312	1102	811	936 cdca7
ENSXETG00000007519	2373	224	338	314	255	211	375	1078	1284	740	503	326	385 map3k20
ENSXETG00000007521	3069	16	13	7	49	39	38	102	125	61	80	44	63 rapgef4
ENSXETG00000007522	318	0	0	0	0	0	0	0	0	0	1	0	1
ENSXETG00000007525	3228	517	825	695	1904	1518	2800	2470	2930	1703	2950	2204	2447 ENSXETG00000007525
ENSXETG00000007526	1116	403	562	446	467	326	525	723	862	519	473	313	371 pdk1
ENSXETG00000007527	3267	439	655	567	994	729	1552	1767	2061	1253	1438	1095	1199 itga6
ENSXETG00000007529	1815	346	516	499	455	329	686	944	1188	651	458	331	399 rap1gds1
ENSXETG00000007532	2464	1	0	1	7	13	9	52	52	26	21	20	13 dlx2
ENSXETG00000007533	759	0	0	0	1	1	5	17	20	14	2	8	4 dlx1
ENSXETG00000007534	1011	139	215	169	152	138	277	273	291	157	226	136	193 metap1d
ENSXETG00000007535	1853	409	569	479	1091	829	875	1776	2153	1233	739	584	663 hat1
ENSXETG00000007537	7853	247	341	279	602	499	751	2217	2661	1505	656	473	564 myo10
ENSXETG00000007538	3516	83	128	103	592	478	942	318	410	242	936	775	753 fam134b
ENSXETG00000007539	2022	2334	3284	2810	2004	1594	2489	1784	2069	1174	2151	1579	1683 znf622
ENSXETG00000007540	663	1	0	0	0	0	0	3	7	4	2	0	0 march11
ENSXETG00000007541	1356	10	11	13	20	27	75	238	288	160	100	56	75 fbxl7
ENSXETG00000007544	672	76	99	83	111	75	114	201	200	131	131	86	94 ENSXETG00000007544
ENSXETG00000007545	843	0	0	0	0	0	0	0	0	0	0	0	0
ENSXETG00000007546	2224	671	940	872	1436	1002	2209	2022	2209	1411	1864	1315	1395 ext2
ENSXETG00000007547	1563	0	0	0	1	0	0	4	4	0	0	0	1 rag2
ENSXETG00000007548	2563	0	1	0	0	0	2	1	4	4	0	1	0 rag1
ENSXETG00000007549	2760	968	1542	1219	2566	1906	2057	1323	1621	928	1719	1211	1382 traf6
ENSXETG00000007551	1242	312	443	399	597	445	1224	1181	1307	756	701	511	532 commd9
ENSXETG00000007552	1020	67	105	83	250	206	529	508	503	353	568	425	413 ldlrad3
ENSXETG00000007553	1658	455	632	499	1005	809	1437	1607	1770	1106	1382	962	1104 trim44
ENSXETG00000007554	2169	1	0	3	5	7	73	188	240	154	154	100	132 pamr1
ENSXETG00000007555	1710	0	0	0	6	2	4	11	10	9	8	9	5 slc1a2
ENSXETG00000007556	1335	90	159	123	328	289	906	2976	3394	2098	3834	2697	2941 cd44
ENSXETG00000007557	1767	725	1047	878	1018	882	1255	1306	1433	871	1449	1045	1088 pdhx
ENSXETG00000007558	3094	462	752	623	836	614	1106	1540	1711	971	1051	657	786 apip
ENSXETG00000007559	909	34	48	54	17	15	34	2594	2937	1778	125	106	97 ehf
ENSXETG00000007560	756	0	2	3	3	0	1	12	16	17	6	4	7 elf5
ENSXETG00000007561	1754	8	10	7	5	3	0	128	128	93	7	1	6 cat.1
ENSXETG00000007564	1083	0	3	0	2	0	4	3	6	3	5	3	3 ENSXETG00000007564
ENSXETG00000007566	1479	116	205	155	239	250	473	345	369	187	484	321	345 atf2
ENSXETG00000007569	2027	10	5	6	35	22	85	382	493	287	28	22	34 hapln3
ENSXETG00000007570	1987	298	389	333	198	176	226	489	619	312	261	203	234 lnpk
ENSXETG00000007571	6012	0	2	4	7	8	16	17	27	5	7	2	8 acan
ENSXETG00000007572	1164	0	0	0	1	0	0	0	0	0	0	0	0
ENSXETG00000007574	606	2301	3397	2906	6743	4963	3354	15601	16157	10099	4319	3029	3128 cldn7
ENSXETG00000007575	903	0	0	0	0	1	0	37	42	27	0	0	1
ENSXETG00000007579	4365	14	19	23	175	144	302	542	660	369	521	330	413 pla2r1
ENSXETG00000007580	1733	0	1	0	16	13	3	38	55	45	9	6	3 hoxd10

ENSXETG00000007581	792	0	0	1	11	11	3	17	38	23	9	2	4	hoxd9
ENSXETG00000007582	864	0	0	0	0	0	0	0	0	0	0	0	0	
ENSXETG00000007583	2398	721	1070	872	1288	1053	1630	1396	1656	974	1720	1198	1413	march7
ENSXETG00000007584	6785	202	325	251	741	634	1010	2367	2669	1482	1332	904	1018	baz2b
ENSXETG00000007588	1525	225	354	304	303	222	434	540	672	358	321	211	240	aen
ENSXETG00000007589	1458	1182	1837	1431	1110	868	2758	819	888	518	988	727	779	wdsub1
ENSXETG00000007590	2653	1940	2825	2413	3247	2483	3096	2762	3106	1791	3994	2833	3209	strbp
ENSXETG00000007591	3770	145	215	157	265	210	599	639	782	414	453	341	329	det1
ENSXETG00000007592	5370	78	111	85	585	472	782	1494	1584	1013	1439	1060	1119	tanc1
ENSXETG00000007593	911	858	1268	1004	687	522	623	922	947	645	354	238	254	mrps11
ENSXETG00000007594	1219	637	812	723	319	255	359	463	588	336	204	144	151	mrpl46
ENSXETG00000007599	1938	239	267	257	845	600	502	898	1006	625	662	442	462	cpsf1
ENSXETG00000007601	297	0	0	0	0	1	1	1	3	0	1	0	0	ENSXETG00000007601
ENSXETG00000007602	1539	12	20	21	86	60	108	60	74	60	155	106	104	ENSXETG00000007602
ENSXETG00000007603	1617	36	93	65	252	188	429	302	371	210	270	187	224	ENSXETG00000007603
ENSXETG00000007604	1940	52	77	47	110	91	134	401	438	330	141	114	128	rgs19
ENSXETG00000007607	1627	1	0	1	6	5	2	0	0	0	0	0	0	sox3
ENSXETG00000007608	987	305	452	353	319	211	378	876	953	601	285	208	239	tspan6
ENSXETG00000007609	459	258	355	261	321	224	378	704	829	466	318	224	223	thrsp
ENSXETG00000007610	6284	109	176	132	283	223	467	1131	1223	752	762	561	573	bcorl1
ENSXETG00000007612	2643	1	2	0	0	0	1	1	2	0	0	0	0	ENSXETG00000007612
ENSXETG00000007614	5713	62	94	83	142	112	274	561	777	417	324	255	216	epha4
ENSXETG00000007616	3102	0	0	0	0	0	2	2	3	1	3	2	3	agbl1
ENSXETG00000007619	4122	19	36	18	201	136	368	626	794	479	741	507	549	plekhg1
ENSXETG00000007622	1854	29	27	33	84	63	104	137	181	102	128	68	73	klhl25
ENSXETG00000007623	2646	136	180	153	346	287	403	999	1290	697	714	476	561	akap13
ENSXETG00000007624	705	8	27	16	51	35	58	121	130	64	82	65	54	ENSXETG00000007624
ENSXETG00000007626	2136	1	4	1	18	21	13	18	30	15	27	14	19	sv2b
ENSXETG00000007628	2418	217	316	261	241	163	322	608	804	446	255	193	196	c3orf38
ENSXETG00000007629	3488	435	607	574	408	342	635	739	898	509	808	606	655	znf654
ENSXETG00000007632	1074	0	2	0	8	3	3	3	5	6	8	5	2	htr1f
ENSXETG00000007633	1953	0	2	2	18	14	13	32	27	22	38	26	43	slco3a1
ENSXETG00000007635	3919	666	940	791	1677	1290	3275	3100	3573	2120	2096	1534	1582	atp6v0a1
ENSXETG00000007637	1086	0	0	0	0	0	0	0	0	0	0	0	0	
ENSXETG00000007638	2242	1911	2822	2269	2661	2072	7481	9066	10414	5977	2491	1748	1997	chmp2b
ENSXETG00000007639	2893	331	435	349	2444	1794	4903	10839	12021	7295	6601	4854	5080	ptrf
ENSXETG00000007640	3295	0	0	0	0	1	0	1	1	1	0	1	0	
ENSXETG00000007642	3531	271	369	296	652	477	1105	1210	1305	816	1197	895	966	stat3.1
ENSXETG00000007644	2766	211	385	348	779	529	664	4178	4780	2863	1171	809	874	fut8
ENSXETG00000007649	1621	0	0	0	0	0	0	0	1	1	1	0	0	c11orf87
ENSXETG00000007650	2674	379	524	399	630	515	1277	1645	1771	1055	1524	1074	1191	max
ENSXETG00000007652	408	1	3	1	0	0	2	2	2	4	0	0	0	ENSXETG00000007652
ENSXETG00000007653	7560	78	144	76	331	223	917	662	772	462	521	452	429	zfyve26
ENSXETG00000007658	2148	0	0	0	0	0	0	0	0	0	0	0	0	
ENSXETG00000007664	590	5518	8094	6855	5852	4364	5658	2276	2425	1504	6208	4352	4780	mgst1
ENSXETG00000007666	1056	3	12	5	35	46	103	284	353	212	76	53	59	st3gal2.1
ENSXETG00000007667	1436	335	437	351	422	316	386	488	503	360	450	307	344	exosc6
ENSXETG00000007668	2979	811	1180	1024	1815	1378	1181	2302	2776	1571	2054	1462	1752	aars
ENSXETG00000007669	1197	0	0	3	4	0	2	20	19	15	2	1	0	ENSXETG00000007669
ENSXETG00000007670	2607	225	369	285	491	394	1033	934	1073	668	728	476	574	pdpr
ENSXETG00000007674	1932	201	277	241	465	340	201	713	770	458	278	193	188	rfwd3
ENSXETG00000007675	1954	138	171	136	303	220	331	1231	1477	814	358	247	264	mlkl
ENSXETG00000007676	1600	10010	14109	11835	10208	8093	15025	3171	3593	2064	4994	3672	3968	fa2h
ENSXETG00000007677	2910	228	307	271	578	442	610	520	588	348	847	577	672	wdr59
ENSXETG00000007681	3097	224	294	219	336	256	443	1930	2269	1365	509	402	412	znrf1
ENSXETG00000007683	537	226	357	331	1208	904	949	896	1069	674	700	553	570	pllp
ENSXETG00000007684	7167	787	1149	1027	1949	1525	4269	6359	7063	4529	4820	3387	3674	jmjd1c
ENSXETG00000007686	4754	121	173	147	350	270	392	425	424	267	322	203	266	fhod1
ENSXETG00000007688	1416	0	0	0	0	0	0	0	0	0	0	0	1	
ENSXETG00000007690	771	158	230	189	410	327	493	912	996	655	498	349	379	reep3
ENSXETG00000007693	1530	101	137	122	440	324	615	648	711	426	458	355	390	nrbf2
ENSXETG00000007696	1201	114	164	135	137	89	191	236	271	174	148	130	132	fam58a
ENSXETG00000007698	1101	4	1	4	19	8	33	239	236	148	34	24	24	dusp9
ENSXETG00000007699	1887	89	139	125	200	159	639	970	1173	678	699	460	534	pnck
ENSXETG00000007700	1704	786	1145	1072	1204	936	1772	1708	1856	1058	3066	2243	2331	gnl3l
ENSXETG00000007701	1620	4	4	6	2	0	0	8	8	4	4	0	3	ENSXETG00000007701
ENSXETG00000007703	1929	1259	1852	1594	548	392	1126	947	1102	643	2477	1721	1994	XB-GENE-986840
ENSXETG00000007705	2235	1525	2035	1854	1261	962	3088	1615	1719	1176	1935	1435	1541	abcd1
ENSXETG00000007706	5544	165	218	210	584	464	378	1185	1445	844	1081	726	859	plxnb3
ENSXETG00000007709	4383	0	0	2	1	2	0	18	12	5	0	0	1	myom2
ENSXETG00000007710	9342	0	0	0	0	0	0	2	0	3	1	1	1	
ENSXETG00000007712	887	83	123	105	226	165	399	786	851	595	447	315	352	vgll4
ENSXETG00000007713	1212	0	0	0	0	0	0	0	1	0	0	0	0	
ENSXETG00000007717	1407	0	0	0	0	0	0	0	0	0	0	0	0	
ENSXETG00000007718	1917	18	25	20	87	81	103	388	478	234	263	187	255	syp
ENSXETG00000007719	1053	0	0	0	0	1	0	13	16	9	2	0	1	wnt7c
ENSXETG00000007721	1132	4449	6675	5727	5310	3951	4366	2111	2669	1489	3420	2445	2506	hsd17b10
ENSXETG00000007726	1193	1599	2409	1896	2046	1575	1850	2548	2905	1751	2223	1534	1730	plp2
ENSXETG00000007727	1494	338	456	441	754	615	805	943	1049	665	940	606	652	gpkow
ENSXETG00000007730	1460	80	108	96	282	244	371	306	374	248	296	202	235	naif1
ENSXETG00000007731	2983	3974	5537	4725	3498	2694	4718	1221	1414	854	2455	1784	1902	slc25a25
ENSXETG00000007732	876	0	1	0	11	8	1	29	35	23	29	18	24	prss1
ENSXETG00000007733	753	4	3	1	106	96	47	5	6	5	144	84	95	prss2
ENSXETG00000007734	850	1	0	0	8	4	24	6	1	2	143	82	104	ENSXETG00000007734
ENSXETG00000007735	1074	2	3	4	6	2	1	17	32	12	7	9	5	ENSXETG00000007735
ENSXETG00000007740	1551	7382	10398	9107	8363	6361	8494	16586	19515	11224	5826	4022	4708	hmgb3
ENSXETG00000007741	2619	151	177	156	293	226	333	835	1004	554	413	298	329	mtmr1
ENSXETG00000007743	3048	96	132	103	182	165	350	386	433	262	377	301	325	mamld1
ENSXETG00000007744	2237	562	746	632	776	528	1614	941	1151	649	1445	982	1166	tmem185a
ENSXETG00000007745	1791	168	253	216	311	245	577	392	444	254	585	428	471	ids
ENSXETG00000007747	1116	0	1	2	11	6	24	37	32	17	14	10	13	sh3gl3
ENSXETG00000007748	645	1675	2533	2219	3639	2778	3218	5283	5704	3636	2786	2138	2221	cct3
ENSXETG00000007752	1563	0	0	0	0	0	0	0	0	0	0	0	0	
ENSXETG00000007754	4641	0	0	0	2	1	2	14	14	6	1	0	1	duox1
ENSXETG00000007756	4856	12	10	7	12	13	33	346	361	209	97	91	93	shf
ENSXETG00000007760	549	153	214	211	680	547	1010	2093	2260	1353	829	614	682	rap2b
ENSXETG00000007761	2607	163	256	195	1050	725	1970	1130	1323	700	2145	1468	1712	arhgef26
ENSXETG00000007763	744	1	1	1	5	4	0	2	6	0	1	0	0	stc1
ENSXETG00000007764	1479	0	0	0	0	0	0	0	0	0	0	0	0	
ENSXETG00000007765	2720	0	0	0	0	0	0	0	0	0	0	0	0	
ENSXETG00000007767	2153	282	425	356	206	143	276	196	240	161	218	121	135	slc25a37
ENSXETG00000007768	2991	232	362	291	464	341	684	720	969	504	641	434	504	dhx36
ENSXETG00000007769	2169	0	1	0	9	6	8	3	9	5	20	13	18	gpr149
ENSXETG00000007770	3925	631	890	831	1208	945	2364	2632	3358	1871	3551	2452	2776	entpd4
ENSXETG00000007771	2235	0	0	0	0	0	0	0	0	1	2	0	1	
ENSXETG00000007772	4812	16	34	20	241	203	97	255	321	168	358	263	343	plch1
ENSXETG00000007773	2298	213	303	221	373	279	748	2251	2530	1548	553	387	410	loxl2
ENSXETG00000007774	2310	948	1379	1165	2362	1825	4705	3791	4160	2617	5204	3659	4108	cd2ap
ENSXETG00000007776	1553	132	192	148	533	389	441	395	478	266	636	441	473	sfrp1
ENSXETG00000007777	2109	1659	2378	1907	2311	1662	5209	4398	5169	2730	3870	2710	3034	golga7
ENSXETG00000007779	1428	0	0	0	0	0	1	0	0	0	0	1	2	

ENSXETG00000007782	318	44	69	49	101	93	100	366	392	263	163	93	134	c8orf4
ENSXETG00000007783	1950	1111	1635	1301	1158	829	2268	2235	2428	1403	1389	982	1009	stambp
ENSXETG00000007784	2123	2630	3818	3435	4100	3267	4399	1880	2228	1416	2134	1549	1614	retsat
ENSXETG00000007785	2747	209	316	286	448	398	592	907	1059	604	713	490	538	zfx
ENSXETG00000007786	1398	414	564	546	424	337	750	2269	2545	1565	415	288	340	gdpd1
ENSXETG00000007787	2220	377	607	509	376	273	1700	6724	7779	4634	1453	1015	1207	mpo
ENSXETG00000007789	1410	6	7	5	1	0	1	22	32	21	16	7	14	XB-GENE-986330
ENSXETG00000007790	3426	810	1261	1085	1469	1079	1736	1284	1436	864	3423	2469	2672	mtmr4
ENSXETG00000007792	1209	264	378	333	412	309	454	525	667	386	297	198	251	casp1
ENSXETG00000007793	2749	454	598	557	392	273	344	501	572	330	313	247	234	abhd11
ENSXETG00000007794	642	2148	3081	2533	2878	2251	4196	6466	7799	4608	4086	2916	3252	cldn3
ENSXETG00000007796	878	543	814	712	464	315	650	1258	1463	906	405	316	323	wbscr27
ENSXETG00000007797	3225	220	296	252	504	380	670	938	990	568	627	451	491	zcchc8
ENSXETG00000007798	2091	693	926	869	1177	914	1778	1920	2252	1387	1846	1272	1447	rsrc2
ENSXETG00000007799	6880	64	105	63	195	151	110	341	408	234	44	41	41	kntc1
ENSXETG00000007803	2454	94	138	110	452	334	305	283	329	207	505	351	419	gcfc2
ENSXETG00000007804	1200	26	30	17	122	93	199	971	1099	680	235	171	176	smad3
ENSXETG00000007805	1141	639	912	835	992	789	1141	2385	2728	1699	1174	797	883	aagab
ENSXETG00000007807	3156	23	37	36	117	112	72	105	127	70	45	31	34	iqch
ENSXETG00000007809	1915	274	398	308	577	394	973	1371	1528	888	875	597	684	map2k5
ENSXETG00000007811	2406	0	1	0	4	2	3	15	10	7	3	0	1	skor1
ENSXETG00000007813	1953	66	83	79	167	125	201	254	323	177	271	184	191	pias1
ENSXETG00000007814	1212	8112	11805	10373	6964	5448	10709	3509	3978	2405	3166	2187	2305	calml4
ENSXETG00000007816	3492	12	18	9	25	14	37	512	535	337	58	50	45	itga11
ENSXETG00000007817	1890	179	257	211	286	224	440	460	517	303	326	223	257	fem1b
ENSXETG00000007819	2262	2	4	9	4	1	0	86	113	79	0	0	0	nox5
ENSXETG00000007821	3206	2451	3278	2904	3936	3180	4965	8273	10271	5946	5282	3941	4183	anp32a
ENSXETG00000007822	2298	0	0	0	6	1	3	8	11	5	5	4	2	lrfn2
ENSXETG00000007824	1815	461	709	584	813	616	1157	540	582	359	629	391	443	mocs1
ENSXETG00000007825	1824	101	127	119	164	126	250	637	719	458	177	127	149	glce
ENSXETG00000007827	798	72	105	86	151	121	139	87	113	52	168	113	109	paqr5
ENSXETG00000007829	3519	142	281	228	894	754	781	1156	1371	827	160	93	128	kif23
ENSXETG00000007831	4150	265	362	350	893	711	1443	1356	1525	946	1665	1242	1328	uaca
ENSXETG00000007833	789	7	9	9	28	23	37	87	94	62	108	86	77	kcnk16
ENSXETG00000007834	1111	2358	3432	2772	1407	1141	880	2279	2603	1653	967	686	736	ccdc167
ENSXETG00000007837	1227	323	441	394	481	324	456	1023	1175	708	618	414	530	morf4l1
ENSXETG00000007838	4923	3	6	5	46	29	157	1493	1660	1058	108	75	82	adamts7
ENSXETG00000007840	1748	1492	2253	1911	1698	1281	2059	4329	4880	2707	1490	1001	1203	eif3j
ENSXETG00000007843	2915	739	1098	967	1937	1693	1892	3396	3719	2207	2651	1851	2030	ctdspl2
ENSXETG00000007844	1626	2	0	2	8	6	11	24	18	23	12	3	9	frmd5
ENSXETG00000007845	1293	767	1192	992	1473	1050	1902	2375	2666	1780	1164	848	886	casc4
ENSXETG00000007846	1884	49	64	49	292	229	76	135	149	78	61	53	49	wdr76
ENSXETG00000007847	4951	74	105	91	145	111	82	311	397	226	127	103	94	blm
ENSXETG00000007848	3480	0	0	0	2	0	0	0	0	0	2	1	1	rasgrf1
ENSXETG00000007850	1804	3	8	1	24	8	15	269	295	181	29	34	39	slc13a3
ENSXETG00000007851	1946	46	54	50	103	93	155	1410	1590	999	295	204	227	crtap
ENSXETG00000007852	1630	166	291	215	111	91	205	174	218	135	163	126	153	tfb1m
ENSXETG00000007853	1053	38	63	56	171	127	232	589	596	394	280	242	266	ENSXETG00000007853
ENSXETG00000007854	2933	287	406	307	878	682	963	1281	1481	852	924	609	687	tlk2
ENSXETG00000007856	1578	1090	1555	1286	801	614	917	1031	1191	699	734	509	576	mettl2b
ENSXETG00000007862	1899	24	43	22	75	50	64	208	248	141	93	66	98	zc2hc1a
ENSXETG00000007863	596	37	29	31	79	62	105	663	744	417	165	136	166	stmn2
ENSXETG00000007864	2013	14	31	18	97	84	120	240	259	152	74	42	49	hey1
ENSXETG00000007868	3326	1	2	7	47	42	190	269	351	172	187	139	190	cacna2d1
ENSXETG00000007872	2184	0	2	0	2	2	8	60	57	29	52	28	41	hgf
ENSXETG00000007874	2923	220	337	257	322	215	429	917	1114	635	733	534	588	sema3c
ENSXETG00000007876	1952	6309	9176	7473	9161	6891	13420	412	510	253	7960	5628	6508	cd36
ENSXETG00000007884	1216	1065	1426	1168	2638	2004	5650	910	1128	623	900	658	786	ENSXETG00000007884
ENSXETG00000007885	2493	629	885	822	1647	1249	1975	1988	2009	1292	1931	1337	1418	cdc5l
ENSXETG00000007886	5105	6	14	20	71	50	174	89	130	85	115	79	121	tbc1d32
ENSXETG00000007888	966	27	47	40	145	132	256	52	62	49	111	68	86	ENSXETG00000007888
ENSXETG00000007889	1004	80	172	103	406	318	476	46	50	31	402	299	335	sult6b1
ENSXETG00000007892	3510	0	0	0	0	0	1	1	0	0	2	0	3	grm1
ENSXETG00000007893	2246	982	1336	1118	2011	1510	3659	1023	1261	745	996	739	827	rab32
ENSXETG00000007895	1107	4738	6855	5958	5737	4391	10201	2793	3268	2127	3499	2358	2758	sccpdh.2
ENSXETG00000007897	1823	2102	2994	2589	2957	2312	6429	2036	2328	1373	2100	1498	1648	h2afy
ENSXETG00000007898	1208	0	0	0	0	0	0	1	0	0	0	0	0	
ENSXETG00000007899	2899	1058	1435	1176	5574	4397	10059	8022	10324	5906	8521	6031	6972	cxcl14
ENSXETG00000007900	2105	0	0	0	0	1	2	54	60	23	18	5	8	slc25a48
ENSXETG00000007902	885	413	573	510	371	281	439	457	494	266	401	301	296	thg1l
ENSXETG00000007903	1314	56	72	78	114	90	129	275	370	211	134	86	114	fbxl21
ENSXETG00000007906	3576	103	108	112	385	277	498	577	613	402	86	77	67	hmmr
ENSXETG00000007907	757	288	470	384	400	301	430	443	464	279	273	187	227	nudcd2
ENSXETG00000007909	2744	3579	4886	3992	3281	2541	5516	7714	9794	5433	5618	3940	4449	ccng1
ENSXETG00000007911	948	0	0	1	0	0	0	1	0	0	0	0	1	ENSXETG00000007911
ENSXETG00000007912	3795	5	5	5	4	5	5	4	3	3	5	1	1	mybpc3
ENSXETG00000007913	945	30	65	43	58	39	174	1003	1195	774	219	142	159	spi1
ENSXETG00000007914	948	28	31	33	62	49	63	372	400	241	114	75	108	slc39a13
ENSXETG00000007915	2174	0	2	2	14	7	10	51	72	42	19	17	19	tmem178.2
ENSXETG00000007916	1446	2147	3206	2870	3778	3008	4118	5715	6477	3973	4336	2894	3324	psmc3
ENSXETG00000007917	3780	306	451	403	805	583	502	814	974	595	666	482	524	nup160
ENSXETG00000007920	1386	247	341	296	495	417	469	1142	1273	805	490	329	336	porcn
ENSXETG00000007921	1455	0	0	0	0	1	0	0	0	0	1	0	1	
ENSXETG00000007922	1008	0	1	0	3	8	35	3087	3321	2038	121	82	71	thibz
ENSXETG00000007923	1836	428	630	553	613	464	1207	2939	3118	1932	962	664	762	dnase2
ENSXETG00000007924	1590	3	3	6	7	14	53	43	70	40	61	29	54	epor
ENSXETG00000007925	1074	0	0	0	4	1	2	63	57	42	24	11	18	plppr2
ENSXETG00000007927	992	905	1354	1149	617	456	929	1187	1376	905	663	524	508	tmem205
ENSXETG00000007928	1755	13	22	22	10	6	30	144	165	99	81	65	77	lgals9
ENSXETG00000007930	792	0	0	0	0	0	0	0	0	0	0	0	0	
ENSXETG00000007932	2162	2565	3579	3132	4286	3160	6350	1275	1471	923	3574	2554	2829	gcdh
ENSXETG00000007934	987	0	0	0	0	0	0	0	0	0	0	0	0	
ENSXETG00000007936	2215	990	1524	1263	1097	882	699	1989	2162	1398	804	575	658	farsa
ENSXETG00000007937	2399	46169	67509	59366	44884	34951	17612	34452	38155	23929	10932	7679	8581	calr
ENSXETG00000007938	1692	257	330	290	375	324	160	225	287	153	283	240	229	ENSXETG00000007938
ENSXETG00000007940	1059	581	836	798	808	665	1068	811	831	544	735	566	546	gadd45gip1
ENSXETG00000007942	4609	16	22	18	7	3	9	135	135	74	7	5	11	pzp
ENSXETG00000007948	1558	2038	2846	2435	2196	1721	2064	3599	4283	2456	2514	1716	2057	lbh
ENSXETG00000007949	1197	371	556	441	1368	1114	1819	1806	2131	1305	1425	1054	1083	ypel5
ENSXETG00000007950	849	0	0	0	1	0	0	0	0	0	0	0	0	
ENSXETG00000007957	1602	0	0	0	0	4	1	12	30	27	3	2	4	ENSXETG00000007957
ENSXETG00000007962	309	210	326	244	209	135	189	642	759	518	95	68	55	chrac1
ENSXETG00000007964	3501	166	234	200	242	165	366	577	759	433	326	190	271	heatr6
ENSXETG00000007968	741	0	0	0	0	0	0	0	0	0	0	0	0	
ENSXETG00000007969	912	0	0	0	0	0	0	0	0	0	0	0	0	
ENSXETG00000007972	3445	1182	1637	1365	2064	1584	3495	2367	3028	1668	2039	1486	1697	adipor1
ENSXETG00000007973	4488	1028	1525	1318	1472	1220	2305	2991	3413	2165	2627	1808	2078	kdm5b
ENSXETG00000007974	4451	599	826	655	818	636	733	1266	1527	829	1248	854	1005	ipo9
ENSXETG00000007975	1554	14756	21717	19057	15811	11811	11917	21768	25110	15075	7570	5231	5961	tuba3c
ENSXETG00000007976	1866	76	114	99	348	242	934	3130	3566	2079	2539	1765	2074	lmod1
ENSXETG00000007983	1141	3700	5633	4508	4117	3090	6555	3179	3631	2266	6109	4379	4827	timm17a

ENSXETG00000007985	2846	284	455	413	604	474	661	2038	2318	1441	334	224	264	parp9
ENSXETG00000007986	903	97	182	171	317	254	421	449	529	336	369	249	282	faim
ENSXETG00000007987	2430	74	117	81	275	234	583	767	914	513	1337	1012	1089	arg2
ENSXETG00000007988	726	999	1513	1286	1978	1452	2236	656	721	446	1120	769	891	hacd2
ENSXETG00000007989	639	0	1	0	3	2	5	35	40	36	18	15	19	tvp23a
ENSXETG00000007990	4523	166	218	217	406	320	633	470	613	313	496	353	470	synrg
ENSXETG00000007991	1112	590	932	778	706	523	1101	823	995	557	869	605	748	nubp1
ENSXETG00000007992	3730	1	0	4	32	19	40	407	482	290	152	104	108	osr1
ENSXETG00000007995	1626	0	1	1	1	0	1	2	2	1	0	0	0	ENSXETG00000007995
ENSXETG00000007996	582	3027	4153	3399	1917	1410	1823	4529	5351	2809	1195	843	978	dynll1
ENSXETG00000007997	1713	0	0	0	0	0	0	0	0	0	0	0	0	
ENSXETG00000008000	2138	279	346	345	1033	786	1064	407	418	257	1557	1012	1160	hnf1b
ENSXETG00000008002	3969	3	6	4	32	49	20	1	2	0	12	13	12	grin2a
ENSXETG00000008003	3546	13	11	20	149	97	74	174	227	109	61	58	60	wdr35
ENSXETG00000008004	947	840	1196	1039	595	505	1096	865	976	614	651	454	510	tmem167b
ENSXETG00000008005	807	0	0	0	0	0	0	0	1	1	0	0	1	matn3
ENSXETG00000008007	2507	5	7	5	2	2	2	15	21	12	18	17	13	apobec2
ENSXETG00000008008	2925	233	372	292	1081	890	1152	222	311	173	1907	1318	1514	kiaa1324
ENSXETG00000008009	1672	2298	3233	2765	2032	1562	2055	3094	3461	2002	1969	1360	1439	sars
ENSXETG00000008010	984	4	1	2	3	2	4	21	25	18	7	2	3	ENSXETG00000008010
ENSXETG00000008011	1123	1951	3013	2521	2662	1931	3069	7822	8644	5300	3680	2605	2823	gpa33
ENSXETG00000008012	1121	2084	3047	2626	2271	1751	2685	4435	5016	3152	2597	1748	2111	laptm4a
ENSXETG00000008015	3430	272	415	345	2542	1916	1934	4128	4569	2730	4418	3015	3470	add1
ENSXETG00000008016	2658	121	177	165	619	500	551	797	909	577	606	385	453	mfsd10
ENSXETG00000008017	1374	0	0	0	2	2	0	0	1	0	0	0	0	vim.2
ENSXETG00000008018	2592	3096	4381	3761	3824	3073	2097	1859	2252	1317	672	530	543	abhd4
ENSXETG00000008019	1767	0	5	1	1	4	0	1	1	3	219	149	189	pth2r
ENSXETG00000008021	5293	936	1349	1113	1715	1269	2875	3371	3871	2199	2592	1880	2052	abr
ENSXETG00000008022	1425	41	49	43	13	18	13	109	142	86	89	53	58	trac
ENSXETG00000008026	1071	0	4	0	0	1	4	6	11	3	3	0	4	ENSXETG00000008026
ENSXETG00000008029	936	0	0	0	0	0	0	0	0	0	0	0	0	
ENSXETG00000008030	1698	7	13	6	7	14	10	16	19	4	13	12	18	ENSXETG00000008030
ENSXETG00000008031	933	0	0	0	0	0	0	0	0	0	0	0	0	
ENSXETG00000008032	2676	15218	21922	18433	14568	11067	7413	5602	6627	3884	1446	990	1087	idh1
ENSXETG00000008034	996	23815	31430	25535	27809	22485	38626	220	220	123	3402	2431	2637	ENSXETG00000008034
ENSXETG00000008037	2218	1069	1503	1209	1685	1305	2338	2158	2579	1439	2151	1441	1576	gosr1
ENSXETG00000008043	900	0	0	0	0	0	0	0	0	0	0	0	0	tlx1
ENSXETG00000008044	849	0	0	0	0	0	0	0	0	0	0	0	0	
ENSXETG00000008045	1465	1	4	2	7	5	7	440	511	286	129	95	105	kazald1
ENSXETG00000008052	465	16555	24952	21796	22018	16693	24479	19273	21806	13894	46952	32632	36316	rpl24
ENSXETG00000008054	3174	229	328	292	615	459	617	1322	1530	890	426	301	348	nrdc
ENSXETG00000008055	2302	54	64	59	89	77	258	276	307	179	413	285	315	rab3b
ENSXETG00000008056	902	3333	4827	4256	4130	3335	5021	5188	6026	3325	6894	4972	5440	btf3l4
ENSXETG00000008057	3963	14	31	23	56	33	92	123	126	69	109	75	92	zfyve9
ENSXETG00000008058	2565	249	374	336	706	488	896	910	1098	644	705	487	480	cc2d1b
ENSXETG00000008059	2835	163	196	197	261	197	57	312	343	219	109	65	96	orc1
ENSXETG00000008060	1221	783	1144	942	660	543	558	1192	1291	800	738	511	610	prpf38a
ENSXETG00000008061	4493	440	620	495	1424	1022	1295	1212	1398	818	1498	1057	1223	zcchc11
ENSXETG00000008062	1023	25	31	30	29	22	31	898	1011	592	61	52	54	gpx7
ENSXETG00000008063	2300	586	808	680	132	98	159	365	418	252	148	97	137	coa7
ENSXETG00000008064	2220	35	37	58	142	106	228	178	203	122	194	107	147	zyg11b
ENSXETG00000008065	1167	687	939	867	1594	1145	911	711	786	493	560	362	406	echdc2
ENSXETG00000008068	1701	0	0	0	1	1	2	0	1	0	0	0	0	slc1a7
ENSXETG00000008069	366	2	2	2	3	4	1	48	58	36	8	10	11	fgf11
ENSXETG00000008070	2114	2532	3472	2971	2300	1818	2821	1457	1764	1045	809	527	639	cpt2
ENSXETG00000008071	483	176	274	231	241	207	278	457	549	298	375	254	253	c1orf123
ENSXETG00000008072	587	808	1234	1050	856	648	851	1803	2210	1342	673	455	546	magoh
ENSXETG00000008073	2469	121	180	161	467	402	72	278	308	206	90	62	65	lrp8
ENSXETG00000008075	2939	159	219	190	286	206	244	399	498	328	210	140	185	ndc1
ENSXETG00000008076	1134	54	90	75	195	164	157	273	301	182	286	212	245	ENSXETG00000008076
ENSXETG00000008078	1173	131	222	194	156	101	150	210	253	151	155	100	121	ndufaf5
ENSXETG00000008079	2541	743	1072	948	1066	765	1368	1630	1914	1038	2294	1540	1827	esf1
ENSXETG00000008080	2237	110	155	117	107	72	93	525	633	408	130	74	103	tasp1
ENSXETG00000008081	1380	1	3	3	11	7	17	71	51	33	47	34	37	ism1
ENSXETG00000008083	1533	603	847	799	2092	1578	3300	1007	1071	652	2322	1679	1850	sptlc3
ENSXETG00000008087	2667	1	0	0	11	8	8	2	2	1	2	5	2	slc9a5
ENSXETG00000008088	6319	8	17	14	53	45	118	612	701	413	256	196	224	ENSXETG00000008088
ENSXETG00000008089	3287	1308	1967	1664	2590	1919	4986	479	594	335	1805	1326	1438	lonp2
ENSXETG00000008090	1357	360	499	427	586	424	977	1040	1224	716	835	578	629	siah1
ENSXETG00000008091	2391	410	563	512	525	379	653	816	969	570	501	365	437	aktip
ENSXETG00000008092	1128	41	50	55	98	94	165	474	540	284	85	65	78	oprk1
ENSXETG00000008095	1710	1262	1832	1553	1722	1396	4244	3365	4130	2495	1238	852	940	atp6v1h
ENSXETG00000008098	1824	2	7	5	15	6	16	73	75	39	12	8	15	rgs20
ENSXETG00000008099	1357	966	1420	1172	1738	1322	1752	2292	2705	1589	2379	1749	1883	tcea1
ENSXETG00000008101	1497	723	1041	845	960	704	994	1739	2161	1171	754	536	599	lypla1
ENSXETG00000008102	3333	434	633	581	1726	1313	1662	2240	2469	1560	3900	2781	3053	capn5
ENSXETG00000008103	1233	1105	1565	1403	1003	748	1147	1146	1385	728	1077	731	832	mrpl15
ENSXETG00000008104	1331	0	0	0	0	0	0	0	0	0	0	0	0	
ENSXETG00000008105	5299	0	0	0	36	14	4	0	0	0	22	10	11	ENSXETG00000008105
ENSXETG00000008106	1293	0	0	0	39	38	1	0	0	0	21	14	14	ENSXETG00000008106
ENSXETG00000008109	1860	0	0	0	5	5	10	38	34	23	7	8	14	xkr4
ENSXETG00000008111	1799	237	339	295	784	617	160	71	71	54	144	87	101	cfap45
ENSXETG00000008115	3495	7	12	13	537	397	381	823	953	643	1202	848	982	igsf9
ENSXETG00000008116	456	155	229	206	230	160	442	371	401	267	226	176	177	dusp23
ENSXETG00000008117	1942	452	605	523	1097	816	1356	1354	1533	978	1444	950	1014	fam63a
ENSXETG00000008118	1392	443	626	521	1025	784	1768	600	690	429	994	695	778	prune1
ENSXETG00000008119	1214	0	3	4	14	13	14	2	4	3	10	12	10	bnipl
ENSXETG00000008120	1372	2192	3334	2826	1416	1128	1915	2107	2246	1389	1012	684	799	pex19
ENSXETG00000008121	1815	181	296	237	526	371	694	786	920	623	809	561	577	dcaf8
ENSXETG00000008122	522	10	17	12	37	44	87	349	437	273	99	55	55	pea15
ENSXETG00000008124	2486	0	0	0	1	2	0	10	13	8	0	2	1	casq1
ENSXETG00000008125	3567	5	3	0	27	20	30	163	195	107	255	183	231	atp1a2
ENSXETG00000008127	1278	6	10	12	36	13	39	20	19	6	17	6	13	npffr2
ENSXETG00000008129	3507	21	37	24	53	39	53	393	456	278	97	54	73	adamts3
ENSXETG00000008131	1599	196	218	230	151	116	127	249	330	182	196	170	195	cox18
ENSXETG00000008132	8040	1074	1460	1323	3771	2834	3881	3754	4365	2507	4432	2995	3323	ankrd17
ENSXETG00000008135	3534	0	0	0	0	0	1	0	0	0	0	0	0	
ENSXETG00000008137	483	81	122	100	102	66	203	369	392	318	129	87	102	lysmd1
ENSXETG00000008138	2744	457	604	532	676	535	621	634	755	477	799	572	660	nop9
ENSXETG00000008139	1092	2	3	0	2	2	5	10	6	5	0	3	1	tmod4
ENSXETG00000008141	696	55	83	83	110	90	205	257	279	192	189	166	162	scnm1
ENSXETG00000008142	2600	144	178	143	316	251	448	1118	1229	753	434	345	305	tnfaip8l2
ENSXETG00000008144	1206	0	0	0	0	0	0	0	0	0	0	0	0	
ENSXETG00000008146	3788	243	358	320	492	390	677	704	847	451	677	440	579	rexo1
ENSXETG00000008147	2018	906	1334	1131	1072	810	1269	997	1152	633	805	586	649	abhd17a
ENSXETG00000008148	1407	0	7	2	2	3	3	11	9	6	3	0	3	rorb.2
ENSXETG00000008149	1545	0	2	1	37	33	100	2616	3088	2004	353	252	277	nfic
ENSXETG00000008150	1005	435	637	585	370	289	447	452	503	308	250	179	229	dohh
ENSXETG00000008151	1626	2772	4050	3420	3019	2223	14974	1940	2161	1349	5907	4075	4596	glud1
ENSXETG00000008153	1493	448	686	566	763	610	694	862	1073	605	664	473	508	eri1
ENSXETG00000008154	2802	103	112	79	25	13	20	146	192	109	107	99	87	ppp1r3b

ENSXETG00000008155	3921	235	371	286	783	636	1139	1249	1392	736	1184	868	952 tnks2
ENSXETG00000008156	2506	54	76	81	72	71	112	200	253	152	212	139	153 dusp4
ENSXETG00000008157	1269	4	6	8	2	2	14	15	18	14	21	34	30 syt15
ENSXETG00000008158	3851	786	1043	959	1078	830	891	1541	1794	1088	1626	1171	1224 bms1
ENSXETG00000008160	5029	82	130	120	399	330	800	932	1077	694	1017	761	837 setbp1
ENSXETG00000008162	654	121	218	196	321	254	324	523	563	319	400	298	339 PRRG4
ENSXETG00000008163	3089	5	8	8	56	42	105	925	1062	587	52	43	49 slc13a4
ENSXETG00000008164	768	26	44	29	22	18	24	48	68	31	15	9	13 pnpla4
ENSXETG00000008165	1734	193	262	174	263	243	529	105	120	70	385	242	310 sts
ENSXETG00000008166	474	0	0	0	0	0	0	0	0	0	0	0	0
ENSXETG00000008167	1334	6963	9762	8423	10833	8040	15732	12398	13978	8519	23589	16499	18758 eif3m
ENSXETG00000008168	1644	560	765	668	431	362	642	356	428	240	505	332	395 pudp
ENSXETG00000008170	840	0	2	0	1	0	0	0	2	1	2	0	0 XB-GENE-992380
ENSXETG00000008172	1272	3	6	4	33	30	23	204	202	146	18	7	16 wt1
ENSXETG00000008174	1272	200	303	275	349	249	250	659	737	459	202	163	196 rcn1
ENSXETG00000008175	1291	6	9	6	20	12	37	53	78	34	64	46	45 pax6
ENSXETG00000008176	1101	110	153	139	220	158	279	446	539	340	316	200	247 elp4
ENSXETG00000008178	829	241	376	293	148	114	144	174	222	136	87	46	59 immp1l
ENSXETG00000008179	1265	300	366	307	462	321	552	752	868	521	681	532	503 dnajc24
ENSXETG00000008181	981	0	0	0	0	0	0	0	0	0	0	0	0
ENSXETG00000008182	2303	212	270	246	335	204	3160	1484	1691	956	921	656	750 ENSXETG00000008182
ENSXETG00000008183	2926	857	1058	994	1605	1239	2289	2740	3299	1932	1825	1297	1456 cul3
ENSXETG00000008185	1139	45	74	64	131	106	140	154	202	117	168	117	126 aebp2
ENSXETG00000008186	882	0	0	0	0	0	0	0	0	0	0	0	0
ENSXETG00000008190	1509	2399	3659	3074	4842	3848	9751	3103	3807	2169	3219	2258	2526 sgpl1
ENSXETG00000008191	1105	1130	1670	1365	1297	935	1586	2742	3209	1879	1075	768	889 pcbd1
ENSXETG00000008193	1102	3070	4393	3731	3270	2515	1093	5754	6633	3907	1205	878	981 pcna
ENSXETG00000008194	1335	63	115	100	162	112	233	338	382	242	167	141	174 cds2
ENSXETG00000008195	2874	46	54	50	99	65	77	416	569	330	247	182	209 arhgap25
ENSXETG00000008196	1269	0	0	0	0	0	0	3	10	1	3	0	0 bmp10
ENSXETG00000008197	689	0	1	0	31	20	0	0	0	0	15	13	16
ENSXETG00000008198	1566	2	4	1	10	12	27	527	560	348	132	84	104 antxr1
ENSXETG00000008199	2097	2104	3073	2627	5839	4438	3726	2497	2923	1759	2603	1812	2072 gfpt1
ENSXETG00000008202	1033	555	853	749	913	699	1185	864	1047	580	826	586	652 nfu1
ENSXETG00000008205	4600	194	320	270	333	281	404	927	1003	557	456	332	306 aak1
ENSXETG00000008206	1281	9999	14515	12393	10445	7885	13938	12616	14447	8644	22293	15617	17531 anxa4
ENSXETG00000008207	2219	833	1177	961	1130	855	1870	2198	2478	1487	1623	1232	1359 naxd
ENSXETG00000008208	1407	29	55	46	123	120	412	619	767	459	348	246	237 gmcl1
ENSXETG00000008209	1206	0	0	0	0	0	3	14	19	8	2	0	0 ENSXETG00000008209
ENSXETG00000008210	1719	387	603	474	265	224	504	318	430	214	305	229	245 cars2
ENSXETG00000008211	1763	181	189	200	481	383	391	1970	2347	1330	536	373	411 ankrd10
ENSXETG00000008212	2015	63	90	70	100	71	160	293	383	201	173	107	143 ing1
ENSXETG00000008213	2626	160	209	215	522	404	869	2103	2311	1380	850	565	657 arhgef7
ENSXETG00000008215	2187	0	0	0	0	0	0	1	0	0	0	0	0
ENSXETG00000008218	1995	8550	12080	10263	9236	7135	11044	3580	4230	2547	2357	1674	1808 abcd3
ENSXETG00000008222	5535	195	268	201	552	467	761	1145	1417	831	852	579	632 arhgap29
ENSXETG00000008223	7080	3	3	7	20	15	22	10	23	8	8	1	3 abca4
ENSXETG00000008224	1365	333	534	521	675	503	790	836	1000	573	804	590	656 gclm
ENSXETG00000008225	1758	5	4	11	29	28	6	22	43	15	15	4	14 ENSXETG00000008225
ENSXETG00000008226	2989	202	274	228	359	304	1019	383	428	293	321	245	252 bcar3
ENSXETG00000008227	2551	776	1134	971	2052	1519	3345	3185	3666	2168	2648	1934	2047 fnbp1l
ENSXETG00000008230	4595	288	385	345	115	63	89	67	103	61	144	117	134 ENSXETG00000008230
ENSXETG00000008232	463	7821	11588	9482	7088	5395	7594	5168	5854	3557	5596	3864	4105 ndufa4
ENSXETG00000008233	1885	619	904	813	1716	1285	1821	2432	2669	1767	2296	1620	1696 lsr
ENSXETG00000008234	1896	2	2	1	5	7	21	142	162	113	11	14	12 mag
ENSXETG00000008236	1185	4	8	5	1	0	7	8	3	3	11	6	9 ENSXETG00000008236
ENSXETG00000008237	2961	259	329	338	895	686	780	3311	3779	2193	1838	1319	1488 epb41l2
ENSXETG00000008240	1787	76	110	94	175	136	281	207	254	156	222	147	220 akap7
ENSXETG00000008241	981	10	9	8	20	14	97	168	175	105	19	10	15 arg1
ENSXETG00000008243	4134	348	483	440	430	355	280	1046	1199	678	372	278	311 med23
ENSXETG00000008244	2529	8178	11492	9587	9427	7291	20688	3889	4956	2487	12827	8800	10261 enpp3
ENSXETG00000008245	2343	108	138	126	156	129	377	513	587	335	180	125	152 ctgf
ENSXETG00000008247	4848	0	0	0	3	3	0	4	3	5	16	9	13 sphkap
ENSXETG00000008248	945	0	0	0	0	0	0	0	0	0	0	0	0
ENSXETG00000008249	799	503	670	504	577	437	563	4817	5518	3749	1128	740	860 rbp1
ENSXETG00000008250	3044	8013	11378	10014	14333	10910	14698	13430	15371	9308	9144	6591	7183 copb2
ENSXETG00000008251	891	713	1184	907	742	516	526	754	899	463	485	356	397 mrps22
ENSXETG00000008252	1101	0	0	0	0	0	0	0	0	0	0	0	0
ENSXETG00000008253	912	0	0	0	0	0	0	0	1	2	0	0	0 foxl2
ENSXETG00000008254	3276	514	727	636	975	694	1632	1712	1948	1140	1862	1407	1495 pik3cb
ENSXETG00000008255	1544	0	1	0	6	0	6	37	61	28	14	16	15 vamp1
ENSXETG00000008256	4305	176	253	255	424	298	293	301	309	190	312	241	245 per2
ENSXETG00000008257	1878	101	140	122	193	158	341	480	547	330	306	224	227 XB-GENE-986927
ENSXETG00000008258	933	5	9	9	25	19	17	22	33	19	42	25	31 ENSXETG00000008258
ENSXETG00000008260	1026	156	247	170	387	304	523	671	811	445	468	319	359 cab39
ENSXETG00000008262	5805	497	686	601	1121	859	1393	2902	3329	1848	1398	980	1042 adam17
ENSXETG00000008263	2393	1199	1696	1426	1989	1501	1987	3332	3719	2308	1825	1289	1456 cpsf3
ENSXETG00000008264	615	110	150	134	151	101	268	283	348	225	142	122	113 itgb1bp1
ENSXETG00000008265	3156	309	434	426	1445	1054	2070	981	1100	640	2412	1727	1900 asap2
ENSXETG00000008268	1661	185	251	264	438	315	596	154	165	116	473	311	325 mboat2
ENSXETG00000008271	5563	326	438	359	936	671	2116	1714	1820	1146	1516	1055	1240 kidins220
ENSXETG00000008272	399	633	937	825	2269	1798	2020	3617	3992	2443	2716	1922	2067 id2
ENSXETG00000008275	2096	133	179	168	227	144	260	313	342	204	172	150	137 mzt2b
ENSXETG00000008276	2560	181	249	225	327	272	475	676	738	412	508	371	403 lztr1
ENSXETG00000008277	1853	5467	7982	6911	3951	3000	6509	323	343	208	1839	1233	1440 aifm3
ENSXETG00000008278	548	2494	3492	2963	2263	1828	2769	2419	2718	1666	2703	1852	2138 hint1
ENSXETG00000008281	1135	71	121	77	108	82	156	171	193	107	147	97	106 isoc1
ENSXETG00000008282	1866	179	252	222	896	639	1351	1017	1227	692	1248	910	1102 slc27a6
ENSXETG00000008295	2040	0	0	1	0	0	0	9	8	3	3	2	4 pcdhgb5
ENSXETG00000008299	10629	1	1	2	156	121	6	26	43	24	22	9	12 dnah2
ENSXETG00000008302	1023	0	0	0	0	1	2	0	0	0	0	1	0 ENSXETG00000008302
ENSXETG00000008304	828	0	0	0	0	0	0	0	0	0	0	0	0
ENSXETG00000008309	5724	0	0	0	3	1	0	0	0	0	12	13	10
ENSXETG00000008313	1045	0	0	0	1	1	2	2	14	11	4	2	0 ENSXETG00000008313
ENSXETG00000008317	16152	53	89	57	3578	2684	98	261	289	196	2720	1813	1998 ENSXETG00000008317
ENSXETG00000008323	1542	14	23	31	34	20	32	62	48	43	72	63	68 ENSXETG00000008323
ENSXETG00000008324	762	345	532	443	1832	1465	821	5383	5819	3786	3948	2784	2964 s100a11
ENSXETG00000008325	1995	0	0	0	0	1	2	7	7	8	4	4	4 XB-GENE-961613
ENSXETG00000008327	1263	143	208	169	263	182	187	249	261	166	187	121	133 acad8
ENSXETG00000008333	872	113	116	114	133	94	133	157	184	111	198	144	136 thyn1
ENSXETG00000008335	2661	449	649	534	477	389	846	973	1200	690	467	379	404 vps26b
ENSXETG00000008336	4455	72	124	71	369	267	240	474	589	336	75	57	65 ncapd3
ENSXETG00000008337	1372	305	426	341	309	260	743	753	873	541	580	390	452 cln3
ENSXETG00000008338	1284	789	1158	1013	2535	1893	5583	2055	2191	1483	2099	1427	1658 spns1
ENSXETG00000008342	3669	1	0	0	3	1	5	22	20	18	3	6	5 igsf9b
ENSXETG00000008344	1590	68	97	61	14	10	23	33	31	20	12	7	11 ENSXETG00000008344
ENSXETG00000008346	2373	0	0	0	0	0	0	1	3	0	0	0	1
ENSXETG00000008348	759	0	0	0	0	0	0	0	0	0	0	0	0
ENSXETG00000008349	945	0	0	0	0	0	0	0	0	0	0	0	0
ENSXETG00000008350	666	0	0	0	0	0	0	0	0	0	0	0	0
ENSXETG00000008351	939	0	0	0	0	0	0	0	0	0	0	0	0

ENSXETG00000008356	933	0	0	0	0	0	0	0	0	0	0	0	1	
ENSXETG00000008362	948	0	0	0	0	0	0	0	0	0	0	0	0	
ENSXETG00000008377	2589	0	0	0	0	0	0	2	6	3	0	1	0	ENSXETG00000008377
ENSXETG00000008383	925	1884	2892	2328	1692	1216	1406	2348	2701	1695	945	728	813	golt1b
ENSXETG00000008384	2026	932	1402	1059	713	599	802	894	1109	640	473	336	383	slc30a6
ENSXETG00000008385	1115	598	855	689	225	212	261	304	384	212	275	193	231	rdh13
ENSXETG00000008386	4008	222	357	301	698	537	1143	1066	1300	797	1118	769	876	sos1
ENSXETG00000008388	684	1	3	2	1	2	1	2	5	4	3	1	1	rgs17
ENSXETG00000008390	1877	0	0	0	0	0	0	0	0	0	0	0	0	
ENSXETG00000008392	2565	140	239	187	876	670	1863	397	398	273	610	420	478	ano5
ENSXETG00000008393	1746	0	0	0	0	0	0	0	0	0	0	0	0	
ENSXETG00000008397	2148	31	51	42	501	401	624	588	647	379	610	426	502	ampd3
ENSXETG00000008399	4170	11	18	23	165	107	231	416	400	268	377	228	301	flt4
ENSXETG00000008400	2590	1487	2054	1809	1815	1388	3926	2632	3008	1912	2105	1357	1576	rnf141
ENSXETG00000008401	912	0	2	0	7	5	4	12	15	5	18	7	13	oprm1
ENSXETG00000008402	1743	0	0	0	0	0	1	2	2	0	1	0	0	slc23a1
ENSXETG00000008403	1115	11670	16794	14462	16776	12879	21839	23092	26976	15674	29552	20332	23107	npm1
ENSXETG00000008404	2217	4183	6022	5395	5576	4140	6303	4815	5744	3460	5257	3802	4222	hspa9
ENSXETG00000008405	2721	2348	3434	2872	4689	3531	5225	7816	9223	5481	4059	2897	3236	ctnna1
ENSXETG00000008407	1009	1321	1890	1528	694	534	749	172	221	123	384	265	306	ltc4s.2
ENSXETG00000008408	1855	3336	4931	4381	4712	3600	4071	5573	6358	3692	3574	2442	2810	canx
ENSXETG00000008409	852	230	346	283	296	208	401	551	676	416	397	311	329	cnot8
ENSXETG00000008410	1002	25045	37443	30912	18970	14384	49691	5857	6686	4047	16360	11509	12816	faxdc2
ENSXETG00000008413	5780	1749	2426	2201	2461	1891	2177	3330	3811	2278	3009	2143	2415	larp1
ENSXETG00000008415	3557	419	629	573	2029	1551	1817	918	1117	654	545	430	435	pc.1
ENSXETG00000008416	2562	54	80	87	357	260	761	819	1058	623	695	496	585	pacs1
ENSXETG00000008421	2178	0	0	0	2	0	0	1	2	2	3	2	2	gabra2
ENSXETG00000008422	2502	1469	2234	1850	2784	2131	2629	3938	4307	2714	2889	2099	2353	sf3b2
ENSXETG00000008423	1315	0	0	0	0	0	0	4	6	2	0	0	0	cnih2
ENSXETG00000008424	1017	704	1110	923	594	462	720	1054	1237	754	544	358	420	sssca1
ENSXETG00000008426	3897	452	630	540	1162	917	2389	2288	2918	1595	1979	1407	1541	ehbp1l1
ENSXETG00000008431	3669	34	38	37	208	190	550	2276	2608	1569	751	498	617	ltbp3
ENSXETG00000008432	1851	273	400	370	334	263	285	405	444	258	515	360	368	guf1
ENSXETG00000008433	2229	280	420	298	632	423	544	795	856	559	610	442	512	men1
ENSXETG00000008434	3374	1340	1976	1637	2331	1685	2094	3433	3828	2406	2547	1812	2007	sf1
ENSXETG00000008436	969	0	0	0	0	0	0	0	0	0	0	0	0	
ENSXETG00000008437	896	267	399	351	427	346	1045	764	783	497	342	245	289	gnpda2
ENSXETG00000008440	1215	0	0	0	1	1	3	3	4	2	1	2	2	kctd8
ENSXETG00000008448	792	0	0	0	0	0	0	0	0	0	0	0	0	
ENSXETG00000008462	3402	1188	1781	1427	2060	1654	2736	2812	3431	1973	4037	2877	3099	agps
ENSXETG00000008465	7164	0	0	2	1	4	2	22	18	17	7	4	6	pde11a
ENSXETG00000008469	2195	43	30	21	235	170	73	253	360	188	70	43	34	ttc30a
ENSXETG00000008472	1605	390	594	524	293	256	380	665	807	424	364	232	237	cyct
ENSXETG00000008473	1131	26	46	35	62	53	203	372	412	236	30	30	20	ENSXETG00000008473
ENSXETG00000008476	1491	84	125	107	196	173	470	1939	2070	1298	662	436	509	wipf1
ENSXETG00000008477	4061	30	46	42	94	95	193	504	550	319	138	92	98	nphp3
ENSXETG00000008478	1059	22	34	35	158	116	242	299	371	214	164	112	124	ackr4
ENSXETG00000008479	2491	155	261	210	598	400	1449	1424	1696	990	803	550	589	hand1
ENSXETG00000008480	2587	349	517	411	359	302	657	1223	1400	775	618	432	485	sap30l
ENSXETG00000008481	1989	530	815	708	1027	802	1286	858	920	555	1264	925	1095	galnt10
ENSXETG00000008483	1326	235	355	304	345	247	669	298	324	210	512	399	425	armt1
ENSXETG00000008484	1266	3131	4560	3987	3890	3039	4067	3806	4443	2711	2195	1552	1703	ndufa9
ENSXETG00000008486	2292	166	220	210	252	242	392	511	597	335	205	127	206	gpr155
ENSXETG00000008487	1236	265	385	349	548	434	1113	503	572	349	1058	723	843	scrn3
ENSXETG00000008488	1446	4	3	1	6	8	28	10	9	5	2	6	2	kcna6
ENSXETG00000008490	1470	0	0	0	1	0	2	0	0	0	1	1	2	kcna1
ENSXETG00000008492	1149	141	227	191	406	313	530	536	596	383	548	386	399	cir1
ENSXETG00000008493	1392	0	1	2	10	4	14	17	23	9	8	10	4	kcna5
ENSXETG00000008494	2064	0	3	1	5	0	10	4	3	0	2	5	3	tgm4
ENSXETG00000008495	1179	0	0	1	5	3	5	58	92	63	50	35	39	ntf3
ENSXETG00000008496	2805	1	0	0	1	0	1	2	0	1	0	0	1	ano2
ENSXETG00000008497	4173	81	130	129	432	298	326	565	702	385	96	74	90	kif15
ENSXETG00000008498	956	145	173	164	230	201	314	481	631	411	254	176	191	kiaa1143
ENSXETG00000008500	1680	1528	2219	1929	2478	1822	3281	3586	4073	2526	5183	3618	4037	ola1
ENSXETG00000008503	2460	280	445	389	815	615	929	1576	1931	1096	1069	735	864	sp3
ENSXETG00000008509	2424	173	230	249	850	647	1152	1078	1122	712	1610	1107	1246	uevld
ENSXETG00000008510	2048	99	131	110	249	239	641	625	630	421	795	544	617	foxp1
ENSXETG00000008511	745	1469	2146	1786	632	480	311	829	936	535	323	228	256	fam136a
ENSXETG00000008512	381	772	1037	968	841	657	556	2022	2254	1502	528	379	441	snrpg
ENSXETG00000008513	2023	780	1295	1066	1881	1371	3316	1668	1974	1196	1231	1044	1106	pcyox1
ENSXETG00000008514	1541	1183	1733	1444	1239	968	1644	3777	4354	2549	1261	934	1076	tia1
ENSXETG00000008515	1765	129	165	153	174	134	263	358	433	253	221	148	169	c2orf42
ENSXETG00000008516	1628	1	1	3	54	41	37	239	226	171	95	67	80	nif
ENSXETG00000008518	1010	817	1158	949	829	658	1115	2120	2394	1266	1153	854	864	snrnp27
ENSXETG00000008519	2163	2	3	0	22	5	22	79	106	51	39	35	25	add2
ENSXETG00000008520	2751	43	52	37	137	105	86	831	881	553	123	82	95	hkdc1
ENSXETG00000008521	834	0	1	0	0	0	0	1	0	0	0	0	0	
ENSXETG00000008522	1306	0	0	0	0	0	0	3	7	2	0	0	0	
ENSXETG00000008524	4981	8	18	15	26	41	58	110	135	77	345	257	270	tacr1
ENSXETG00000008525	1359	99	145	142	108	79	212	315	383	214	122	87	111	piga
ENSXETG00000008526	774	0	0	0	0	0	0	0	0	0	0	0	0	
ENSXETG00000008527	3453	9	10	3	27	20	38	376	440	295	56	39	51	ENSXETG00000008527
ENSXETG00000008528	652	656	1015	740	451	322	459	758	810	500	411	298	317	nxt2
ENSXETG00000008529	1146	9	12	7	853	585	38	34	43	16	597	383	394	foxq1
ENSXETG00000008530	1706	1870	2721	2360	1912	1432	340	642	697	453	97	102	80	phgdh
ENSXETG00000008531	888	0	0	0	0	0	0	0	0	0	0	0	0	
ENSXETG00000008533	915	0	0	0	0	0	0	0	0	0	0	0	0	
ENSXETG00000008534	885	0	0	0	0	0	0	0	0	0	0	0	0	
ENSXETG00000008535	999	0	0	0	0	0	0	0	0	1	0	0	0	
ENSXETG00000008541	801	0	1	1	4	1	2	5	10	5	3	2	3	ENSXETG00000008541
ENSXETG00000008544	954	0	0	0	0	0	0	0	0	0	0	0	0	
ENSXETG00000008546	4059	278	423	321	630	470	712	878	1053	581	842	679	662	ahctf1
ENSXETG00000008547	1517	503	729	583	594	456	893	2293	2932	1600	332	199	245	memo1
ENSXETG00000008548	2460	549	823	646	816	599	1681	1679	2192	1207	542	385	451	psen2
ENSXETG00000008549	728	661	924	729	717	602	947	1247	1430	742	748	596	682	dpy30
ENSXETG00000008550	3519	3	3	7	55	51	129	1264	1565	903	142	84	97	thbs2
ENSXETG00000008551	1822	39	51	39	3	3	1	1	2	0	0	0	0	XB-GENE-5767840
ENSXETG00000008552	1546	457	642	555	927	758	1834	1536	1837	1042	1299	849	1047	phf10
ENSXETG00000008555	2194	367	553	490	241	212	384	378	470	255	559	429	483	pdcd2
ENSXETG00000008556	1667	195	262	200	181	146	226	572	631	380	285	181	235	tbp
ENSXETG00000008557	788	2494	3604	3056	2600	1919	3015	5954	7190	4077	1286	933	1149	psmb1
ENSXETG00000008562	1200	0	0	0	0	0	0	0	0	0	0	0	0	
ENSXETG00000008571	2846	1	0	0	0	1	0	1	0	0	2	0	1	brdt
ENSXETG00000008572	2236	194	303	241	555	448	834	603	716	402	747	540	547	dcbld1
ENSXETG00000008575	447	659	1074	959	1341	1046	1365	721	924	565	860	632	625	npepl1
ENSXETG00000008576	1063	0	0	0	0	0	0	0	0	0	1	0	0	
ENSXETG00000008578	1497	0	1	0	2	0	0	4	2	2	0	0	1	nr6a1
ENSXETG00000008581	3364	11	21	23	65	54	116	448	541	303	97	65	88	olfml2a
ENSXETG00000008589	588	0	1	0	0	0	0	0	0	0	0	0	0	ENSXETG00000008589
ENSXETG00000008590	459	1404	2095	1803	431	307	276	730	845	494	381	270	315	mid1ip1
ENSXETG00000008591	1905	19	32	15	41	32	108	392	402	251	139	110	119	tspan7

ENSXETG00000008592	1348	6	6	3	24	13	10	7	6	5	10	1	5	otc
ENSXETG00000008596	1392	28	32	27	209	178	491	607	776	460	203	128	154	srpx
ENSXETG00000008597	2295	14	23	10	167	119	86	107	135	67	302	212	236	sytl5
ENSXETG00000008598	1089	0	0	0	0	0	0	0	0	0	0	0	0	
ENSXETG00000008599	789	27	41	28	68	55	128	657	722	430	169	143	144	frrs1l
ENSXETG00000008600	3846	1650	2505	2000	2641	2028	5247	479	606	307	540	370	370	DPYD
ENSXETG00000008601	1431	34	36	44	124	94	124	114	146	66	130	99	117	znf238.2
ENSXETG00000008602	1740	2285	3212	2681	2124	1641	3127	1937	2341	1358	1712	1245	1443	snx7
ENSXETG00000008603	1458	21	26	26	92	59	138	350	427	209	151	110	135	akt1
ENSXETG00000008604	807	0	0	0	1	0	0	0	0	1	2	2	2	
ENSXETG00000008605	1698	0	0	1	1	0	1	0	0	0	0	0	0	ENSXETG00000008605
ENSXETG00000008607	1260	0	0	0	0	0	2	0	5	1	1	1	0	ENSXETG00000008607
ENSXETG00000008612	2464	25	23	20	60	44	85	504	557	310	137	87	99	slc6a8
ENSXETG00000008613	4477	33	57	51	89	94	81	698	861	481	228	172	186	inf2
ENSXETG00000008615	699	0	0	1	1	1	3	25	34	18	5	5	9	tmem179
ENSXETG00000008619	4916	180	302	252	929	737	1444	1766	2192	1345	1465	1121	1121	usp19
ENSXETG00000008620	2126	28	64	44	304	259	381	1964	2202	1264	760	561	558	bmp4
ENSXETG00000008622	5343	61	118	107	1421	1063	1108	1075	1254	755	1123	791	873	lamb2
ENSXETG00000008628	1579	2758	4101	3380	3838	2908	4969	5959	6963	4233	3081	2136	2387	psmc6
ENSXETG00000008629	2763	49	67	49	186	155	431	512	619	365	436	285	328	syde2
ENSXETG00000008630	2931	0	1	0	86	66	18	10	12	8	6	4	5	wdr63
ENSXETG00000008631	1068	0	0	0	7	14	4	38	57	26	17	9	11	lpar3
ENSXETG00000008633	1868	18	15	18	10	11	127	18	16	5	6	3	5	mcoln3
ENSXETG00000008634	516	9	10	9	39	37	62	30	27	18	211	145	178	ENSXETG00000008634
ENSXETG00000008635	1665	142	257	185	375	317	425	295	301	190	260	203	208	ssx2ip
ENSXETG00000008637	5661	3	8	2	4	6	11	26	23	16	4	8	5	vtga2
ENSXETG00000008638	4162	289	403	382	776	576	253	497	600	342	724	520	540	MGC107780
ENSXETG00000008640	1298	653	967	845	1152	857	2080	1359	1586	921	2744	2022	2199	ctbs
ENSXETG00000008641	681	8	10	6	13	6	0	5	2	2	2	4	1	ENSXETG00000008641
ENSXETG00000008642	1217	765	1112	927	1139	834	1821	1211	1365	808	4309	3055	3441	rpf1
ENSXETG00000008645	1329	41	63	58	436	333	562	147	215	110	533	349	447	spata1
ENSXETG00000008648	991	1	3	2	1	0	0	2	4	0	0	0	0	XB-GENE-5811053
ENSXETG00000008649	3734	346	451	404	546	389	1082	1816	2153	1307	891	627	731	prkacb
ENSXETG00000008651	2869	7	7	11	22	19	22	127	147	93	30	27	15	ttll7
ENSXETG00000008652	534	4	12	8	128	79	35	30	21	19	81	66	61	fam107a
ENSXETG00000008653	1656	443	693	598	1320	958	1374	1344	1555	1048	2142	1631	1585	pde12
ENSXETG00000008654	543	3730	5570	4844	4839	3590	4781	6687	7721	4499	3324	2373	2555	arf4
ENSXETG00000008657	1812	107	172	130	823	596	1131	1040	1096	733	952	693	723	dennd6a
ENSXETG00000008658	2568	810	1243	1091	3275	2527	3991	2980	3395	2113	4066	2957	3134	slmap
ENSXETG00000008663	7830	1576	2338	2102	7448	5842	5691	7163	7874	4916	7641	5376	6092	flnb
ENSXETG00000008665	1420	19	39	49	68	44	83	477	437	287	79	60	80	dnase1l3
ENSXETG00000008666	1668	0	0	0	0	0	0	0	0	0	0	0	0	post
ENSXETG00000008670	1950	3739	5452	4634	3996	3105	5605	1748	1970	1247	2407	1706	1896	abhd6
ENSXETG00000008671	2199	0	2	3	4	5	11	25	24	10	15	12	8	cdh22
ENSXETG00000008672	1569	85	147	115	87	90	191	223	267	162	98	64	89	atg5
ENSXETG00000008674	2566	24	18	26	73	36	144	1111	1230	710	167	102	114	prdm1
ENSXETG00000008675	1752	345	478	412	944	717	1908	1011	1174	728	1388	870	1029	pxk
ENSXETG00000008678	2880	1	1	2	0	0	0	0	1	1	0	0	0	ENSXETG00000008678
ENSXETG00000008680	1569	5	2	2	55	39	76	364	433	259	139	96	133	elavl4
ENSXETG00000008681	2698	130	173	132	202	153	223	366	517	296	319	238	248	kctd6
ENSXETG00000008682	453	5121	7631	7073	9944	7678	19611	2947	3354	2498	9427	6613	7272	ENSXETG00000008682
ENSXETG00000008685	1813	2573	3593	3070	3174	2340	2129	3234	3782	2274	2274	1540	1839	pdhb
ENSXETG00000008687	1245	50	74	62	226	170	283	691	801	526	606	454	536	bend5
ENSXETG00000008688	12258	3	7	5	272	213	28	19	36	14	75	48	37	ENSXETG00000008688
ENSXETG00000008689	636	1	0	2	1	1	4	31	53	21	19	12	20	tmem114
ENSXETG00000008690	3833	78	96	85	72	64	97	99	136	75	128	96	103	mettl22
ENSXETG00000008691	3166	1126	1531	1285	1230	943	1673	1210	1491	766	1329	945	1027	abat
ENSXETG00000008692	756	295	429	337	271	183	147	345	432	245	101	59	74	pmm1
ENSXETG00000008695	2294	517	710	684	1425	1060	1348	2240	2526	1638	1442	1049	1080	ncoa5
ENSXETG00000008696	845	2246	3220	2718	2425	1873	2942	2252	2571	1499	2143	1505	1767	carhsp1
ENSXETG00000008697	3324	826	1218	1000	1791	1345	1837	2578	3145	1725	2241	1537	1801	usp7
ENSXETG00000008699	1648	811	1131	949	1365	1098	2191	2338	2838	1689	1299	921	1010	c16orf72
ENSXETG00000008702	2106	1278	2021	1721	2844	2148	5196	2900	3182	2106	4655	3317	3573	appl1
ENSXETG00000008703	2748	132	170	162	359	311	2115	5928	6893	4080	1242	847	1065	mmp9
ENSXETG00000008705	1776	25	44	33	222	170	91	168	214	118	163	128	121	ENSXETG00000008705
ENSXETG00000008706	2625	54	89	84	291	251	360	169	217	132	229	192	233	flrt3
ENSXETG00000008707	1317	0	0	0	0	1	1	4	3	2	7	8	9	asb14
ENSXETG00000008709	939	0	0	0	0	0	0	0	0	0	0	0	0	
ENSXETG00000008710	835	0	0	0	0	1	0	0	0	0	0	0	0	
ENSXETG00000008714	1125	0	0	0	0	0	1	3	6	1	0	0	2	gna15
ENSXETG00000008715	720	477	661	582	216	204	444	377	409	235	289	196	221	schip1
ENSXETG00000008716	2650	132	214	163	427	320	414	428	510	269	340	238	242	ift80
ENSXETG00000008717	4029	217	317	306	920	771	843	1486	1846	1014	437	325	364	smc4
ENSXETG00000008719	900	30	48	43	62	52	121	2777	2999	1763	324	205	245	gzmh
ENSXETG00000008720	4946	1669	2308	1933	2558	2023	3366	3641	4597	2536	3053	2091	2422	kpna4
ENSXETG00000008722	1371	1	0	0	0	0	1	8	10	10	2	0	0	ENSXETG00000008722
ENSXETG00000008724	966	0	0	0	1	2	1	1	2	1	9	5	2	ENSXETG00000008724
ENSXETG00000008725	1851	1597	2301	2023	801	668	4119	29	39	27	1015	697	779	arl14
ENSXETG00000008726	1394	331	487	386	402	328	795	839	1005	579	681	450	479	ppm1l
ENSXETG00000008727	486	2779	3661	3083	121	114	48	1403	1719	1041	30	25	27	ENSXETG00000008727
ENSXETG00000008728	1719	58	73	76	453	360	479	928	1166	667	287	190	237	sptssb
ENSXETG00000008729	1587	0	0	0	0	0	0	0	0	0	0	0	1	OTOL1
ENSXETG00000008730	819	1	0	0	0	0	0	0	0	0	0	1	1	ENSXETG00000008730
ENSXETG00000008731	1668	150	178	154	21	12	140	270	305	210	631	412	507	ENSXETG00000008731
ENSXETG00000008733	1222	1	0	0	0	0	0	0	0	0	0	0	0	cib3
ENSXETG00000008736	1567	1356	2059	1713	1480	1113	1577	13187	15361	9249	2185	1526	1777	tpm4
ENSXETG00000008740	4454	1671	2286	1840	1676	1349	3625	3578	4499	2520	2105	1488	1732	rab8a
ENSXETG00000008743	2216	0	0	0	0	0	0	0	0	0	0	0	0	
ENSXETG00000008745	1537	5	7	2	9	8	11	206	254	164	70	56	53	XB-GENE-5844396
ENSXETG00000008746	1760	10	9	5	19	12	9	180	204	139	58	41	35	slamf8
ENSXETG00000008752	1379	203	333	284	383	300	345	550	630	375	290	219	217	elavl1
ENSXETG00000008755	3300	798	1153	939	2532	1883	2599	3719	4217	2560	3359	2268	2569	safb
ENSXETG00000008765	528	0	0	0	0	0	0	0	0	0	0	0	0	
ENSXETG00000008771	831	119	167	148	454	382	686	647	708	503	621	434	473	ENSXETG00000008771
ENSXETG00000008777	2850	14	33	25	714	523	251	0	2	0	206	134	173	
ENSXETG00000008779	8445	73	104	115	632	498	601	2495	2796	1707	712	520	591	fbn1
ENSXETG00000008781	5151	49	57	45	321	226	156	217	307	156	141	98	119	cep152
ENSXETG00000008782	1260	2	3	0	8	5	11	40	67	36	21	14	26	shc4
ENSXETG00000008783	3278	618	953	821	961	787	4207	1324	1460	846	1106	788	900	secisbp2l
ENSXETG00000008784	2157	897	1240	1063	1317	927	2272	1524	1911	976	1685	1188	1378	cops2
ENSXETG00000008785	1583	384	583	534	502	414	1632	1086	1288	807	1464	990	1064	galk2
ENSXETG00000008786	585	2	2	1	17	16	16	45	49	31	27	29	12	fgf7
ENSXETG00000008787	1330	92	127	102	199	140	231	179	191	132	167	110	107	dtwd1
ENSXETG00000008788	3108	0	0	0	9	10	1	3	2	1	7	6	2	ENSXETG00000008788
ENSXETG00000008789	4522	81	72	66	124	92	192	276	343	192	209	194	185	atp8b4
ENSXETG00000008792	3009	0	0	0	0	0	0	4	7	3	0	0	0	pcdh8.2
ENSXETG00000008793	784	6218	9165	7943	11836	8954	8462	9534	11246	6616	5162	3670	3889	sri
ENSXETG00000008794	1974	0	1	0	2	0	10	108	141	74	36	17	18	ENSXETG00000008794
ENSXETG00000008795	3624	0	0	0	0	0	1	0	4	0	1	0	1	
ENSXETG00000008796	1926	1134	1674	1318	757	639	1072	62	83	33	209	125	170	cyp2c8.1
ENSXETG00000008798	1962	0	0	0	0	0	0	8	20	5	0	0	0	ENSXETG00000008798

ENSXETG00000008801	2754	145	186	176	405	299	370	709	827	503	622	395	493	znf628
ENSXETG00000008802	2905	0	0	0	1	0	0	0	0	1	1	0	0	
ENSXETG00000008803	2352	8173	11845	9941	3337	2577	5949	685	733	403	760	532	629	atf5
ENSXETG00000008804	2405	4903	6887	5980	2331	1888	2648	2039	2512	1401	3086	2121	2438	atf5.2
ENSXETG00000008805	2799	137	212	179	449	379	176	644	770	447	220	152	168	lig1
ENSXETG00000008809	2055	1	0	0	1	1	3	3	0	2	5	3	1	vstm2b
ENSXETG00000008812	3122	51	75	49	307	235	506	702	762	536	836	646	660	fzd8
ENSXETG00000008813	882	32	35	38	112	65	360	378	490	286	274	174	184	ccny
ENSXETG00000008815	2241	534	669	627	899	674	1088	1317	1524	845	877	573	653	cul2
ENSXETG00000008818	1062	29	40	39	1048	714	70	3	0	0	830	585	681	ENSXETG00000008818
ENSXETG00000008820	3435	1115	1716	1454	3519	2687	3746	3380	4072	2358	2825	2047	2298	ddb1
ENSXETG00000008821	5169	449	597	488	1454	1013	3380	1966	2250	1368	2884	2088	2340	sorbs1
ENSXETG00000008822	2066	142	242	175	494	386	422	218	279	161	522	385	440	tkfc
ENSXETG00000008823	1014	0	0	0	0	0	0	0	0	0	0	0	0	
ENSXETG00000008824	1632	65	104	98	162	114	264	396	429	238	318	213	213	tmem138
ENSXETG00000008825	2170	1952	2727	2249	910	726	1450	1449	1753	1044	1209	808	1000	bet1l
ENSXETG00000008826	1336	5298	7526	6421	7204	5715	10411	8509	9672	5986	8717	6025	6779	pdlim1
ENSXETG00000008827	1439	2115	3127	2638	3406	2482	3357	5389	6107	3611	1874	1264	1374	psmd13
ENSXETG00000008828	1923	160	240	218	48	41	16	126	149	88	25	22	29	hspa1b
ENSXETG00000008830	2846	972	1402	1278	1973	1580	972	1076	1226	705	1085	719	855	hells
ENSXETG00000008831	744	0	0	0	0	0	0	0	0	0	0	0	0	
ENSXETG00000008832	2943	7	13	21	44	22	20	66	79	48	67	51	46	syt7
ENSXETG00000008834	2418	225	358	316	352	253	446	651	801	452	419	321	325	kif1bp
ENSXETG00000008835	852	0	0	1	0	1	0	0	0	0	0	1	2	
ENSXETG00000008836	2459	15971	22881	19297	29977	23138	47045	38821	44026	26848	226122	158233	179857	ddx21
ENSXETG00000008837	2850	0	1	0	4	5	1	23	29	16	7	3	1	stox1
ENSXETG00000008838	2457	0	1	0	14	12	13	2	0	1	14	13	13	ENSXETG00000008838
ENSXETG00000008840	3021	14	27	26	198	165	321	1723	1929	1193	374	214	266	fzd2
ENSXETG00000008843	4182	688	926	835	2247	1755	2205	3481	3778	2274	4222	2902	3221	gpatch8
ENSXETG00000008844	3387	598	861	717	1488	1122	2743	1824	2040	1212	2326	1609	1894	ccar1
ENSXETG00000008847	312	0	0	0	0	0	0	0	0	0	0	0	0	
ENSXETG00000008848	1981	1757	2357	2110	2411	1898	4937	3996	4587	2620	2554	1804	1984	vps26a
ENSXETG00000008851	2541	0	0	0	0	0	0	0	0	0	0	0	0	
ENSXETG00000008852	2181	432	689	503	263	219	239	435	440	275	350	256	270	supv3l1
ENSXETG00000008859	2736	1832	2754	2373	4276	3381	7866	3342	4040	2332	2575	1789	1866	HKDC1
ENSXETG00000008860	4198	26	48	39	341	255	170	545	568	399	417	316	332	hk1
ENSXETG00000008870	4250	0	0	0	0	1	0	0	1	0	0	1	0	
ENSXETG00000008871	1521	36	40	32	46	39	54	175	183	137	67	51	62	nfia
ENSXETG00000008872	2520	0	0	0	3	5	10	0	0	0	0	1	0	
ENSXETG00000008873	5016	9	9	5	42	23	45	158	175	114	74	41	51	kndc1
ENSXETG00000008875	6818	24	34	26	103	111	190	438	516	334	36	37	37	frem1
ENSXETG00000008877	585	1946	2873	2501	1563	1178	1593	4125	4848	2862	711	493	553	psma4
ENSXETG00000008879	1231	0	0	0	0	0	0	0	0	0	0	0	0	
ENSXETG00000008880	2353	527	674	586	841	685	1355	927	1107	657	1108	786	920	hook1
ENSXETG00000008881	945	0	0	1	1	0	5	4	11	6	2	1	1	XB-GENE-5817456
ENSXETG00000008887	2106	150	204	197	86	100	169	196	227	121	70	57	45	fggy
ENSXETG00000008889	477	1173	1740	1525	975	795	1169	1083	1282	721	661	498	498	ndufs6
ENSXETG00000008892	2206	0	0	0	0	0	0	0	0	0	0	0	0	
ENSXETG00000008895	2241	0	0	0	0	1	1	0	0	0	1	2	1	
ENSXETG00000008899	2283	0	0	0	0	0	0	0	0	0	0	0	0	
ENSXETG00000008901	1506	9	9	9	23	27	40	648	767	457	36	20	27	il4i1
ENSXETG00000008902	1392	549	817	671	1032	807	2120	2883	3169	1900	2239	1540	1741	sdc1
ENSXETG00000008903	3510	311	419	411	1156	872	1542	2345	2533	1549	1794	1296	1453	pum2
ENSXETG00000008904	1595	503	749	653	768	586	1017	148	174	99	525	380	388	slc7a15
ENSXETG00000008905	3004	397	605	506	659	450	1075	1353	1617	910	726	562	556	hs1bp3
ENSXETG00000008906	1254	0	0	0	4	0	2	19	9	9	1	2	0	gdf7
ENSXETG00000008907	903	542	836	682	341	282	460	259	287	176	225	149	145	ldah
ENSXETG00000008910	2213	3553	5186	4431	5592	4303	12559	9185	10410	6262	6981	5082	5555	mcl1
ENSXETG00000008911	1254	0	0	0	0	0	0	0	0	0	0	0	0	
ENSXETG00000008912	2765	2363	3369	2773	3809	2857	5868	4291	5054	3035	5115	3465	3909	golph3l
ENSXETG00000008913	1185	1	3	2	3	2	8	15	17	10	8	9	11	hormad1
ENSXETG00000008914	1752	3405	4760	4082	5963	4538	13829	32247	38002	21859	11319	7702	8964	ctss
ENSXETG00000008916	1020	7	15	12	6	4	11	227	243	134	42	24	45	ENSXETG00000008916
ENSXETG00000008917	4842	212	342	278	877	671	1128	1758	1868	1249	1320	950	979	lrp6
ENSXETG00000008919	2031	725	1141	884	878	683	1370	1914	2087	1358	1452	1056	1200	etv6
ENSXETG00000008921	2760	511	728	615	738	526	1826	710	738	458	751	499	582	parp12
ENSXETG00000008923	1848	103	167	180	349	242	493	515	555	340	197	154	155	ENSXETG00000008923
ENSXETG00000008924	849	1	2	3	2	1	2	2	4	2	8	4	8	ENSXETG00000008924
ENSXETG00000008928	951	0	0	0	0	0	0	0	0	0	0	0	0	
ENSXETG00000008929	1149	22	21	26	61	46	52	39	45	30	134	77	86	ENSXETG00000008929
ENSXETG00000008931	1137	1036	1528	1267	1239	963	2507	1214	1314	883	1917	1268	1401	cers2
ENSXETG00000008935	960	0	0	0	0	0	0	0	0	0	0	0	0	
ENSXETG00000008936	3834	280	441	389	1080	902	952	1382	1576	973	1171	812	913	setdb1
ENSXETG00000008937	867	0	0	0	0	0	0	0	0	0	0	0	0	
ENSXETG00000008939	1407	0	0	0	0	0	0	1	3	0	1	0	1	camk1g
ENSXETG00000008940	3841	278	468	429	240	161	140	2893	3372	1991	289	213	260	lamb3
ENSXETG00000008941	879	4512	6450	5437	3502	2716	5361	527	640	352	1589	1105	1196	hsd11b1
ENSXETG00000008942	3189	823	1211	993	1519	1178	2263	2674	3101	1778	2503	1791	1890	arnt
ENSXETG00000008943	399	117	137	163	401	283	247	432	468	319	263	206	208	ENSXETG00000008943
ENSXETG00000008945	1615	5475	7952	6703	8723	6563	14039	2583	2776	1782	3866	2745	3046	dhcr7
ENSXETG00000008946	1582	49	91	59	121	101	130	275	341	181	30	39	24	ska1
ENSXETG00000008949	1135	12	16	8	74	48	144	473	552	303	180	111	156	cuedc1
ENSXETG00000008951	3641	421	609	510	1271	985	1897	2213	2636	1468	2024	1356	1627	vezf1
ENSXETG00000008953	1850	108	139	110	121	90	163	340	409	222	143	121	131	tada2a
ENSXETG00000008954	2036	1986	2992	2481	2021	1612	2186	6275	7452	4550	3252	2344	2555	srsf1
ENSXETG00000008955	7047	80	102	106	364	290	213	261	335	178	490	340	386	acaca
ENSXETG00000008957	1101	795	1065	949	720	538	996	954	1147	645	425	306	304	cpped1
ENSXETG00000008958	7109	10	19	25	21	6	26	28	42	16	36	22	31	scn8a
ENSXETG00000008959	1716	240	381	291	336	283	395	494	579	346	252	201	247	aatf
ENSXETG00000008962	1413	32	47	37	87	49	86	583	662	380	157	133	142	XB-GENE-5957486
ENSXETG00000008963	2640	0	0	0	0	0	0	0	0	0	0	0	0	
ENSXETG00000008965	6407	0	1	2	6	5	11	23	27	26	39	23	13	scn3a
ENSXETG00000008966	972	345	505	441	901	702	1988	1294	1487	897	1345	933	1003	syap1
ENSXETG00000008968	1997	225	316	276	712	557	1273	975	1197	644	1013	705	819	dtx2
ENSXETG00000008969	1572	16	21	25	17	23	22	73	100	75	32	13	24	rad51d
ENSXETG00000008971	3065	53	77	66	42	49	118	409	537	337	237	175	179	ywhag
ENSXETG00000008972	782	0	1	1	0	1	0	2	1	1	0	0	0	hspb1
ENSXETG00000008975	1528	396	537	410	946	674	1620	417	461	269	746	572	702	acy3
ENSXETG00000008976	1305	16533	24203	20654	27502	21355	20109	8749	10233	5789	18154	12931	14106	mdh2
ENSXETG00000008978	1156	18	17	19	89	67	35	19	21	13	11	7	8	styxl1
ENSXETG00000008980	1313	1201	1760	1457	937	694	1951	755	940	618	1580	1168	1248	tmem120a
ENSXETG00000008981	1335	0	1	0	2	3	4	3	7	5	4	3	0	slc38a11
ENSXETG00000008982	1362	7	11	5	82	67	33	99	118	78	151	104	123	grb14
ENSXETG00000008983	2076	5654	8171	7169	5859	4352	14279	5078	5614	3263	4339	2996	3252	por
ENSXETG00000008988	1368	0	0	0	7	4	4	1	1	2	23	24	15	galnt15
ENSXETG00000008989	3653	947	1338	1094	2177	1674	2471	2402	2873	1636	2146	1504	1704	ankrd28
ENSXETG00000008991	1536	289	358	318	197	151	241	175	189	97	77	54	63	btd
ENSXETG00000008992	2100	3696	5361	4596	6184	4760	5480	1041	1252	765	1761	1226	1290	hacl1
ENSXETG00000008997	1566	104	165	123	221	149	302	252	248	163	180	123	151	plekha8
ENSXETG00000008998	1257	180	252	208	182	124	194	774	918	567	284	234	272	fkbp14
ENSXETG00000008999	918	0	0	0	0	0	0	0	0	0	0	0	0	

ENSXETG00000009003	1035	162	215	218	260	204	407	409	434	301	227	179	199 bag4
ENSXETG00000009004	815	839	1239	966	907	654	955	1033	1140	736	721	518	574 lsm1
ENSXETG00000009005	2403	205	291	263	608	455	649	905	987	572	1004	661	787 grk5
ENSXETG00000009006	864	0	0	0	0	0	0	1	4	3	0	1	1 star
ENSXETG00000009007	1752	160	239	192	488	386	426	952	1013	635	345	238	271 ash2l
ENSXETG00000009008	1481	0	0	0	0	0	0	0	0	0	0	0	0
ENSXETG00000009009	1562	0	0	0	0	0	0	0	0	0	0	0	0
ENSXETG00000009012	2919	352	560	423	713	547	798	782	816	525	801	572	647 slc8a1
ENSXETG00000009013	6751	929	1260	1072	1152	848	1243	1289	1471	885	1912	1390	1621 heatr1
ENSXETG00000009015	1572	415	558	460	614	475	1115	972	1076	625	698	504	546 lgals8
ENSXETG00000009022	1262	547	785	693	704	541	795	551	647	404	745	485	593 galm
ENSXETG00000009023	1119	258	391	342	231	173	250	296	357	189	276	185	196 gemin6
ENSXETG00000009025	1515	275	409	387	607	481	728	502	610	351	379	276	280 pkr2
ENSXETG00000009030	676	73331	106495	93611	83791	65400	101197	81642	91972	57536	225447	158936	170983 rps9
ENSXETG00000009033	3004	267	414	348	1208	871	1755	1437	1570	983	1837	1260	1414 ap2a1
ENSXETG00000009034	2097	532	777	686	830	684	1407	2729	3067	1862	952	726	780 spib
ENSXETG00000009036	3421	155	209	207	602	493	347	607	707	432	383	266	280 pold1
ENSXETG00000009037	429	72	85	81	217	192	139	206	241	130	59	51	46 eva1b
ENSXETG00000009042	1784	1212	1764	1495	1698	1258	2575	1568	1687	931	2631	1833	2008 rab3ip
ENSXETG00000009043	1398	4063	6021	5086	3709	2755	1125	132	135	98	143	121	107 fads2
ENSXETG00000009044	1957	257	396	324	599	434	755	1303	1440	798	666	451	466 celf1
ENSXETG00000009045	2712	10	14	12	68	37	129	444	530	277	261	191	221 satb1
ENSXETG00000009046	1515	8	18	10	16	9	27	122	159	83	27	26	26 ENSXETG00000009046
ENSXETG00000009047	2298	100	146	121	239	179	672	549	681	391	424	303	398 tbc1d5
ENSXETG00000009048	1236	1	3	3	7	6	2	2	2	0	3	3	3 rapsn
ENSXETG00000009049	3200	456	715	590	1351	1039	684	2720	3210	1815	1124	773	852 far1
ENSXETG00000009051	3063	148	218	184	298	248	386	424	523	341	461	377	396 plcl2
ENSXETG00000009052	2934	2	0	1	1	0	2	26	33	15	6	1	1 ovch2
ENSXETG00000009053	867	2721	4039	3426	2371	1791	3948	780	824	527	904	622	693 ENSXETG00000009053
ENSXETG00000009054	1358	4099	6037	5448	4338	3155	4219	448	480	295	753	527	573 cyb5r2
ENSXETG00000009055	2010	0	0	0	0	0	0	0	0	0	0	0	0
ENSXETG00000009056	3629	682	999	890	686	546	1061	2447	2905	1634	1402	1031	1062 ppfibp2
ENSXETG00000009057	1200	0	0	0	0	0	0	2	0	2	0	4	1
ENSXETG00000009063	2409	0	6	3	9	15	29	246	248	134	35	25	26 spon1
ENSXETG00000009064	864	35	49	40	89	71	134	157	210	124	121	77	93 ENSXETG00000009064
ENSXETG00000009066	987	10	4	3	9	5	19	126	152	94	28	12	23 kcnab3
ENSXETG00000009068	1023	226	369	309	430	395	968	541	720	432	1547	1144	1257 ENSXETG00000009068
ENSXETG00000009070	1028	718	1106	882	1005	781	1402	1469	1612	967	1040	808	851 trappc1
ENSXETG00000009073	1026	0	0	0	6	9	20	5	17	5	60	55	43 ENSXETG00000009073
ENSXETG00000009074	3171	0	0	0	0	0	0	1	0	0	0	0	0
ENSXETG00000009076	936	10	10	13	550	456	1438	31	46	28	1868	1383	1564 ENSXETG00000009076
ENSXETG00000009077	903	0	0	0	1	3	7	13	20	13	12	8	9 ENSXETG00000009077
ENSXETG00000009079	1276	1	0	0	0	0	0	0	0	0	0	0	0
ENSXETG00000009083	732	0	0	1	1	0	3	2	4	1	0	0	0 hes7.2
ENSXETG00000009086	3153	556	793	702	1676	1292	1139	575	573	386	1157	841	879 per1
ENSXETG00000009089	744	0	0	0	0	1	2	10	7	5	1	5	6 ENSXETG00000009089
ENSXETG00000009094	850	0	2	0	15	14	23	3	4	1	18	17	21 ENSXETG00000009094
ENSXETG00000009096	228	9	15	4	39	36	59	198	242	129	160	98	119 vamp2
ENSXETG00000009097	891	82	122	105	343	275	252	612	750	436	75	60	66 aurkb
ENSXETG00000009098	1191	47	85	59	95	90	164	177	199	122	178	106	128 ENSXETG00000009098
ENSXETG00000009100	597	0	0	0	0	0	0	0	0	0	0	0	0
ENSXETG00000009101	1441	2139	3200	2790	2828	2140	3511	3276	3632	2323	2596	1665	2013 cops6
ENSXETG00000009104	2151	423	669	593	1104	839	318	1569	1780	1113	403	305	327 mcm7
ENSXETG00000009105	1581	180	264	228	281	228	335	377	435	260	164	107	125 ap4m1
ENSXETG00000009108	552	18	23	28	107	54	189	35	21	16	391	279	300 rgs11
ENSXETG00000009109	2016	306	444	373	361	266	676	619	773	455	198	148	171 cdc123
ENSXETG00000009110	870	20	35	18	27	29	65	23	34	30	62	42	48 camk1d
ENSXETG00000009111	1620	827	1126	1018	1191	959	4136	2102	2625	1409	1937	1314	1522 optn
ENSXETG00000009112	2103	619	880	779	632	531	805	1020	1272	725	778	544	592 hspa14
ENSXETG00000009113	3049	95	122	103	287	236	80	219	272	143	71	64	70 mcm10
ENSXETG00000009114	746	52	101	63	184	147	37	4	7	1	11	6	8 ucma
ENSXETG00000009115	1927	45340	65267	55113	26902	20891	62768	3680	4246	2483	17543	12309	14016 phyh
ENSXETG00000009116	1257	164	260	201	286	220	340	928	1080	602	302	226	241 sephs1
ENSXETG00000009117	1047	57	74	64	226	166	402	230	255	134	259	196	209 bend7
ENSXETG00000009118	3639	23	26	30	178	146	274	839	929	536	522	340	449 frmd4a
ENSXETG00000009119	1644	38	70	56	93	58	43	141	154	101	39	32	37 lrr1
ENSXETG00000009120	1665	715	1064	923	577	489	909	1527	1734	1040	807	589	674 fam107b
ENSXETG00000009121	1879	1460	1874	1612	860	697	1249	1531	1900	977	760	543	608 mgat2
ENSXETG00000009122	2983	50	79	73	204	167	225	314	372	236	162	97	137 dnaaf2
ENSXETG00000009123	1733	244	330	261	448	362	205	454	528	315	162	116	144 pole2
ENSXETG00000009124	2726	145	208	162	378	264	1007	1042	1211	799	840	635	625 clcn7
ENSXETG00000009125	1344	269	409	359	502	417	948	694	860	504	927	680	673 klhdc2
ENSXETG00000009126	2874	3	3	3	10	11	28	34	41	37	35	27	33 atl1
ENSXETG00000009127	2706	363	468	453	709	572	1305	1702	2060	1161	1261	890	1007 map4k5
ENSXETG00000009128	1822	1	1	1	3	2	8	17	20	11	6	6	19 ptx4
ENSXETG00000009129	2813	7	15	7	36	23	18	25	23	17	7	3	3 sec14l1
ENSXETG00000009131	3954	204	288	249	609	456	793	882	1055	613	876	624	703 sos2
ENSXETG00000009134	561	0	1	2	24	26	5	0	0	0	3	1	2 tmem212
ENSXETG00000009136	3189	1962	2680	2378	4325	3096	8202	2542	3073	1707	6237	4349	4966 pld1
ENSXETG00000009137	5232	44	62	67	719	512	408	728	767	462	730	495	570 ppl
ENSXETG00000009138	3138	421	586	509	1074	909	1600	1541	1662	1066	1962	1390	1585 ubn1
ENSXETG00000009139	1774	529	770	696	1852	1445	2621	2604	2800	1826	2519	1819	2046 glyr1
ENSXETG00000009142	3852	138	191	156	635	490	933	908	1062	606	545	374	422 tnik
ENSXETG00000009143	1729	172	277	242	621	506	2062	984	1031	611	1125	789	907 rogdi
ENSXETG00000009145	4616	6836	9406	7868	4453	3317	7830	1635	2035	1123	2607	1768	2087 slc2a2
ENSXETG00000009147	1110	0	0	0	0	0	0	202	230	148	14	11	4 XB-GENE-5962512
ENSXETG00000009151	981	7	12	9	32	26	53	644	621	458	105	70	81 sept12
ENSXETG00000009154	410	6227	8806	7308	6040	4744	7033	3840	4936	2768	4946	3514	4104 rpl22l1
ENSXETG00000009157	2289	13	10	13	31	19	71	235	333	182	62	42	58 slc7a14
ENSXETG00000009163	912	38	41	34	49	34	73	304	344	242	135	82	87 ENSXETG00000009163
ENSXETG00000009165	1248	0	0	1	0	0	0	0	1	1	0	0	0
ENSXETG00000009166	1673	1	4	2	36	30	58	157	199	110	40	23	25 hmp19
ENSXETG00000009168	1264	3	3	9	17	13	16	38	28	18	46	19	23 msx2
ENSXETG00000009170	2521	217	263	208	646	540	791	597	610	380	667	484	585 cep97
ENSXETG00000009171	1335	262	365	330	360	289	434	584	671	437	398	292	307 rcc1l
ENSXETG00000009173	2135	209	336	274	187	152	351	622	733	413	515	414	450 dhx33
ENSXETG00000009174	744	58	86	53	118	82	334	332	424	238	296	201	207 aaed1
ENSXETG00000009175	4137	1	1	2	3	9	2	14	13	10	6	2	9 astn1
ENSXETG00000009177	1495	0	0	0	0	0	0	0	1	0	0	0	0
ENSXETG00000009178	4323	29	44	21	175	144	149	194	240	137	219	151	132 ccdc171
ENSXETG00000009181	1104	0	0	0	0	0	0	0	0	1	0	1	0 lrrc2
ENSXETG00000009182	3539	231	338	285	524	410	292	453	548	294	773	544	653 sass6
ENSXETG00000009183	1027	1313	1740	1468	685	582	623	580	789	418	364	219	294 derl2
ENSXETG00000009184	3480	4468	6633	5620	10447	8054	11317	6151	7319	4180	8577	6219	6949 aco1
ENSXETG00000009185	1852	107	149	126	100	88	97	217	275	150	119	66	93 mis12
ENSXETG00000009187	4455	2	0	1	8	7	11	4	6	12	23	16	31 pappa2
ENSXETG00000009188	3342	660	1003	827	1024	750	1459	1367	1679	904	1116	777	905 cnksr1
ENSXETG00000009189	745	2041	2953	2326	3820	2820	4461	3023	3598	2050	17241	12371	13789 znf593
ENSXETG00000009190	1474	263	372	322	451	404	739	673	878	476	605	445	480 tbrg1
ENSXETG00000009191	1470	0	0	0	6	4	3	44	76	52	10	13	9 trim63
ENSXETG00000009192	2908	590	839	744	835	654	1545	1538	1891	1062	1199	845	1020 rfwd2

ENSXETG00000009193	1518	464	652	544	575	455	714	256	333	196	777	523	637	fam110d
ENSXETG00000009194	1503	105	160	141	145	102	184	219	233	167	178	131	125	pdik1l
ENSXETG00000009195	7894	2	1	3	33	25	36	59	62	39	49	26	40	tnr
ENSXETG00000009196	1696	0	0	0	11	3	2	10	12	8	2	4	1	slc30a2
ENSXETG00000009197	921	0	0	0	0	0	0	0	0	0	0	0	0	
ENSXETG00000009198	255	72	97	99	131	94	73	648	777	416	88	68	84	stmn1
ENSXETG00000009199	1313	544	766	634	593	475	725	986	1162	648	519	361	431	abhd10
ENSXETG00000009200	2757	134	135	133	220	168	630	320	447	231	225	152	181	ddx58
ENSXETG00000009201	951	61	84	82	54	34	78	100	154	89	59	34	37	plcxd2
ENSXETG00000009202	1509	91	115	116	90	70	208	215	246	144	174	121	137	nectin3
ENSXETG00000009207	612	0	0	0	0	0	0	1	0	0	0	0	0	
ENSXETG00000009208	2079	27	49	30	74	48	114	251	278	175	212	162	160	skil
ENSXETG00000009209	627	0	0	1	2	3	5	398	473	301	36	39	29	cldn11
ENSXETG00000009210	1889	46	59	63	165	147	227	562	593	378	283	195	229	prkci
ENSXETG00000009212	2925	41	69	53	56	39	94	111	138	80	249	185	213	phc3
ENSXETG00000009213	3411	1224	1946	1630	1424	1116	2175	2844	3326	1934	1474	1017	1061	sec62
ENSXETG00000009215	2120	703	1051	897	1360	1077	2285	1668	1918	1154	1536	1074	1213	lrrc31
ENSXETG00000009216	1689	0	0	1	13	11	1	3	6	4	0	3	4	lrriq4
ENSXETG00000009217	1344	4	7	1	52	67	7	12	13	9	6	7	3	lrrc34
ENSXETG00000009218	3239	76	98	85	75	50	160	156	206	115	170	98	143	mynn
ENSXETG00000009225	622	228	286	258	38	28	89	272	303	171	81	58	61	adm
ENSXETG00000009226	5559	128	212	171	593	417	858	1435	1615	960	1194	919	952	sbf2
ENSXETG00000009227	1249	32	36	26	33	36	39	138	144	111	79	50	74	khdrbs3
ENSXETG00000009229	1216	418	621	554	815	633	1034	933	988	604	629	439	440	metrnl
ENSXETG00000009231	1491	2	2	0	11	11	5	51	48	31	25	15	30	trim16
ENSXETG00000009233	2303	0	1	1	2	1	8	217	251	164	54	32	32	ENSXETG00000009233
ENSXETG00000009235	492	58	95	71	58	38	76	45	54	30	101	74	94	ENSXETG00000009235
ENSXETG00000009237	2328	166	252	212	416	300	607	584	692	371	713	505	550	usp44
ENSXETG00000009238	2453	2265	3311	2900	4862	3715	7964	4932	5635	3277	7569	5260	5681	metap2
ENSXETG00000009239	1797	77	125	79	176	110	194	729	863	507	302	238	242	fgd6
ENSXETG00000009241	944	1709	2546	2087	2851	2180	3618	4585	5000	3232	3254	2359	2495	rhoa.2
ENSXETG00000009243	1488	8	6	6	39	29	52	232	293	161	119	66	65	nectin1
ENSXETG00000009248	1444	144	198	125	93	77	77	383	431	269	16	14	13	trim29
ENSXETG00000009251	5469	33	51	58	118	97	206	488	625	334	240	200	203	dock4
ENSXETG00000009253	1284	298	436	332	359	227	667	569	770	435	600	466	494	znf277
ENSXETG00000009255	2011	940	1355	1152	799	564	1193	2558	2982	1763	682	477	563	ifrd1
ENSXETG00000009256	2100	220	331	275	453	320	641	655	866	470	460	301	338	tmem168
ENSXETG00000009257	1494	66	91	99	150	151	286	309	363	199	169	93	102	bmt2
ENSXETG00000009258	3027	0	0	1	5	2	7	35	42	18	24	10	14	gpr85
ENSXETG00000009263	936	0	0	0	0	0	0	0	0	0	0	0	0	
ENSXETG00000009264	1818	1816	2721	2328	7272	5457	9109	15360	17521	11253	5998	4370	4740	papss2
ENSXETG00000009265	2053	916	1326	1152	1977	1451	1903	798	858	455	1254	844	912	ENSXETG00000009265
ENSXETG00000009266	3598	1643	2326	1980	2028	1580	2210	2581	2953	1675	897	604	763	atad1
ENSXETG00000009267	1430	12	31	27	79	68	41	132	109	60	25	8	21	sult1c2
ENSXETG00000009268	2142	394	580	434	588	464	956	1422	1710	1032	803	603	646	pten
ENSXETG00000009271	2607	793	1159	958	1287	923	890	1738	1946	1110	1469	1024	1147	rbm28
ENSXETG00000009272	1278	278	440	373	321	251	516	214	249	138	257	173	204	rnls
ENSXETG00000009273	1320	1346	2025	1713	2537	1856	2920	5630	6407	4010	1325	945	1016	lipa
ENSXETG00000009274	1976	206	313	252	317	243	505	525	628	352	791	477	612	zfp36l2
ENSXETG00000009275	5796	110	153	125	178	145	181	373	436	229	98	83	93	thada
ENSXETG00000009278	1263	103	146	108	105	71	159	97	88	66	102	65	54	stambpl1
ENSXETG00000009279	1392	16646	23892	20089	38399	28833	125899	95683	102627	65329	148561	102302	114226	acta2
ENSXETG00000009282	4323	2	1	4	15	8	60	297	314	209	99	67	80	plekhh2
ENSXETG00000009287	1068	62	78	55	139	93	119	130	181	85	56	44	53	dync2li1
ENSXETG00000009290	2156	4190	6009	5189	6328	4705	11333	2061	2460	1558	11748	8414	9066	abcg5
ENSXETG00000009293	1980	1887	2662	2248	2807	2117	5425	1773	2161	1225	6226	4404	4871	abcg8
ENSXETG00000009295	945	0	0	0	0	0	0	0	0	0	0	0	0	
ENSXETG00000009296	4387	2942	4185	3721	2886	2186	2294	4270	5026	2838	1573	1171	1277	lrpprc
ENSXETG00000009300	951	0	0	0	0	0	0	0	0	0	0	0	0	
ENSXETG00000009304	2873	4343	6367	5365	4310	3306	2853	2365	2772	1559	2384	1763	1789	adh5
ENSXETG00000009306	1212	80	118	91	125	103	227	216	228	139	181	136	142	dnajb14
ENSXETG00000009307	2652	6601	9456	8122	10271	7922	12465	1616	1831	995	3747	2611	3071	mttp.1
ENSXETG00000009309	1823	151	202	144	275	187	313	653	766	401	392	252	324	dapp1
ENSXETG00000009311	1122	1	0	2	1	0	0	1	0	0	1	2	1	adh7
ENSXETG00000009312	636	0	4	0	0	1	1	1	6	2	4	0	0	ENSXETG00000009312
ENSXETG00000009313	1311	22428	32753	28507	43583	33668	35692	8010	8777	5143	6544	4740	5211	adh1b
ENSXETG00000009314	1131	0	1	0	0	1	1	0	2	2	38	18	20	ENSXETG00000009314
ENSXETG00000009315	1271	163	220	203	217	156	250	350	386	236	274	218	212	metap1
ENSXETG00000009316	1113	1973	2783	2372	1699	1242	1818	2406	2943	1650	1237	807	967	eif4e
ENSXETG00000009317	1920	113	171	143	330	238	347	221	257	165	474	329	346	tspan5
ENSXETG00000009319	2356	295	416	325	496	414	941	859	969	606	412	295	355	nckipsd
ENSXETG00000009324	3485	1913	2770	2285	3263	2565	4572	3551	4283	2453	6804	4873	5396	ip6k2
ENSXETG00000009325	483	0	0	0	0	0	0	0	0	0	0	0	0	
ENSXETG00000009330	1215	1023	1445	1230	1604	1297	2808	2783	3278	1916	1736	1322	1410	prkar2a
ENSXETG00000009331	1450	7167	10190	9024	8272	6298	8149	4432	4976	2920	2265	1633	1728	slc25a20.2
ENSXETG00000009335	999	0	0	0	0	0	9	0	0	0	0	2	1	frzb2
ENSXETG00000009337	1335	2	0	0	0	0	0	1	0	0	0	0	0	ENSXETG00000009337
ENSXETG00000009339	2166	171	251	213	311	244	338	341	426	241	357	256	259	dalrd3
ENSXETG00000009340	3303	165	241	209	186	133	423	329	403	259	435	295	294	wdr6
ENSXETG00000009343	1973	3002	4387	3748	7558	5880	7281	8014	8823	5478	14414	10212	10870	impdh2
ENSXETG00000009344	2520	48	56	53	301	280	711	1277	1358	844	996	717	752	kank2
ENSXETG00000009347	3357	315	419	401	948	709	829	1582	1729	1027	939	633	768	qrich1.1
ENSXETG00000009348	723	3	3	1	9	13	2	48	40	27	13	14	5	adgrd1
ENSXETG00000009350	3028	571	781	719	1127	823	903	1271	1549	827	2250	1562	1786	sfswap
ENSXETG00000009351	2389	2320	3412	3076	4505	3549	7058	5541	6275	3655	9438	6522	7313	qars
ENSXETG00000009352	2697	1553	2238	1955	9048	7240	6124	1489	1670	1032	4749	3140	3649	ldlr
ENSXETG00000009354	2429	1016	1552	1333	1710	1288	2469	3606	4065	2528	3268	2306	2494	pim1
ENSXETG00000009355	5125	90	154	126	218	169	113	2004	2113	1272	173	137	161	smarca4
ENSXETG00000009356	1306	3449	4871	4147	3744	2718	4486	3033	3496	2116	3525	2544	2660	yipf2
ENSXETG00000009357	1829	530	742	618	1283	948	1633	1392	1484	941	1859	1346	1506	carm1
ENSXETG00000009359	2769	1157	1625	1369	3062	2280	3037	3382	3899	2277	3724	2680	3043	prkcd
ENSXETG00000009360	2807	1701	2536	2227	3867	2933	5478	5207	5569	3541	3295	2528	2619	dnm2
ENSXETG00000009362	3439	1509	2218	1892	2100	1526	1385	3027	3411	2011	1666	1137	1317	pfkfb4
ENSXETG00000009368	2929	144	205	193	296	208	473	1016	1282	720	581	410	401	tmem110
ENSXETG00000009373	378	6	9	8	0	0	3	15	11	6	5	4	6	pvalb
ENSXETG00000009374	1968	236	335	257	70	45	198	437	460	293	158	125	163	ncf4
ENSXETG00000009375	1407	49	72	58	101	76	206	904	1100	635	200	149	144	csf2rb
ENSXETG00000009376	1530	433	595	481	449	365	641	831	965	570	517	343	383	c9orf72
ENSXETG00000009377	2167	75	114	89	427	318	1249	459	611	336	1223	889	941	mob3b
ENSXETG00000009381	5286	1	2	1	0	2	0	3	3	7	2	0	1	XB-GENE-5934251
ENSXETG00000009382	3357	58	86	63	248	183	366	746	931	513	423	311	336	tek
ENSXETG00000009383	2337	147	166	170	267	236	332	195	232	120	248	131	190	ift74
ENSXETG00000009385	2900	1562	2257	1887	1980	1382	2965	3427	3995	2348	1540	1056	1196	plaa
ENSXETG00000009386	1196	223	378	282	204	156	315	687	833	460	258	174	202	caap1
ENSXETG00000009387	1903	0	1	0	0	0	0	2	0	0	5	0	1	elavl2
ENSXETG00000009388	3175	109	166	136	280	199	292	320	407	220	444	327	332	fbxw7
ENSXETG00000009392	2628	84	120	88	188	150	434	252	336	220	375	273	337	fam160a1
ENSXETG00000009395	3049	600	891	726	2384	1812	3834	4248	4905	3041	3529	2507	2799	sh3d19
ENSXETG00000009396	840	53306	78160	66717	78993	58986	95157	88279	100259	57465	192260	133995	148934	rps3a
ENSXETG00000009400	4320	0	0	0	0	0	1	0	5	0	4	5	1	
ENSXETG00000009402	3903	1	2	2	54	29	53	76	100	47	168	139	144	ovos2

ENSXETG00000009403	1095	0	0	0	0	0	0	0	0	0	0	0	0	lrtm2
ENSXETG00000009404	1251	2089	3081	2527	3118	2480	4700	1843	2109	1359	4604	3288	3541	adipor2
ENSXETG00000009405	1693	170	246	213	768	582	1619	386	422	272	1442	1007	1115	wnt5b
ENSXETG00000009406	1203	113	169	147	274	218	352	511	618	400	382	282	308	fbxl14
ENSXETG00000009409	5142	87	140	135	260	211	408	792	841	522	476	393	368	erc1
ENSXETG00000009412	1236	0	0	0	0	0	5	0	0	0	4	8	7	
ENSXETG00000009413	1645	49	52	45	77	60	49	113	146	100	73	49	59	rad52
ENSXETG00000009415	1395	0	0	0	0	0	0	0	0	0	0	1	2	
ENSXETG00000009416	7076	299	476	344	1506	1181	2276	3838	3942	2561	2178	1410	1674	wnk1
ENSXETG00000009422	2949	50	38	30	124	102	169	484	590	355	218	183	184	b4galnt3
ENSXETG00000009423	1122	0	0	0	0	0	0	0	0	0	0	0	0	
ENSXETG00000009427	1050	135	169	170	187	175	477	250	288	152	266	213	233	aga
ENSXETG00000009434	3714	279	407	341	893	697	1530	1756	2133	1260	1756	1128	1312	pan2
ENSXETG00000009437	3047	368	522	434	1057	851	984	1668	2065	1212	1354	989	1056	dgka
ENSXETG00000009438	3212	173	290	260	508	376	916	1145	1273	820	935	698	766	taok1
ENSXETG00000009439	2823	29	38	25	18	16	98	74	57	40	33	33	28	pmel
ENSXETG00000009441	1335	2	2	0	9	2	0	2	1	0	1	0	0	ENSXETG00000009441
ENSXETG00000009442	2506	8	15	16	83	60	75	349	411	251	114	79	88	tp53i13
ENSXETG00000009444	1970	285	402	382	471	385	148	652	756	473	105	98	85	cdk2
ENSXETG00000009445	1692	103	139	129	427	334	544	910	992	583	535	364	449	git1
ENSXETG00000009446	1440	49	55	56	261	198	616	56	69	43	9	10	12	suox
ENSXETG00000009447	1941	71	110	93	144	120	248	390	477	270	189	133	139	nufip2
ENSXETG00000009448	816	1	2	2	3	2	7	12	21	14	0	0	1	cryba1
ENSXETG00000009449	1500	23	30	40	54	39	72	132	135	91	81	64	71	ikzf4
ENSXETG00000009450	632	0	1	1	4	2	1	6	6	4	0	1	0	ENSXETG00000009450
ENSXETG00000009451	1278	0	0	1	0	0	3	5	3	3	0	3	3	XB-GENE-5805950
ENSXETG00000009454	7412	306	437	408	1440	1075	1043	2100	2236	1405	2089	1489	1669	myo18a
ENSXETG00000009458	557	21769	32447	28006	41005	30878	47150	39920	44695	26790	85696	60215	64963	rps26
ENSXETG00000009461	1011	0	1	1	8	4	14	34	37	24	24	30	33	ENSXETG00000009461
ENSXETG00000009462	969	1469	2156	1897	1007	689	1308	16	21	16	34	13	30	as3mt.1
ENSXETG00000009463	3888	770	1115	972	3984	3126	4009	2469	2813	1768	4620	3345	3695	erbb3
ENSXETG00000009464	1746	95	154	122	122	103	377	81	79	64	212	141	188	cnnm4
ENSXETG00000009465	2022	231	308	271	675	476	1752	1154	1376	806	1151	882	855	cnnm3
ENSXETG00000009466	1023	0	0	0	2	2	0	8	10	4	7	4	4	
ENSXETG00000009467	1887	5180	7541	6478	7585	5767	10942	1708	1952	1249	6439	4580	4950	pipox
ENSXETG00000009469	999	0	0	0	0	0	0	0	0	0	0	0	0	ENSXETG00000009469
ENSXETG00000009471	1354	6251	9213	7875	8659	6409	14185	9768	11433	6991	16012	11348	12477	pa2g4
ENSXETG00000009473	996	63	91	46	51	42	182	38	33	35	155	93	106	XB-GENE-1012534
ENSXETG00000009475	1629	566	878	755	1847	1406	3434	2287	2517	1450	1770	1272	1372	nt5c2
ENSXETG00000009476	2805	520	728	588	1139	918	1176	1837	2130	1260	986	699	818	zc3h10
ENSXETG00000009478	1179	0	3	4	16	8	19	68	85	45	24	21	28	ina
ENSXETG00000009480	701	135	170	132	152	85	160	193	227	140	143	76	108	pcgf6
ENSXETG00000009481	3108	38	71	47	281	203	387	2078	2446	1381	787	563	584	esyt1
ENSXETG00000009482	5740	1073	1570	1411	1485	1204	824	1587	1780	1136	2018	1431	1683	pdcd11
ENSXETG00000009483	4932	530	728	629	1512	1112	2018	3119	3340	2124	2337	1689	1843	eif4g3
ENSXETG00000009486	3787	237	335	351	903	717	1548	2577	2833	1767	1695	1212	1280	smarcc2
ENSXETG00000009487	1428	1117	1604	1411	2919	2291	3086	3705	3826	2471	3056	2283	2343	hp1bp3
ENSXETG00000009491	1996	67	106	81	66	40	102	316	352	185	180	103	106	zap70
ENSXETG00000009494	2679	19	8	5	35	36	24	167	204	117	29	18	23	phc2
ENSXETG00000009495	1332	7	6	8	24	10	69	348	392	217	46	32	58	ENSXETG00000009495
ENSXETG00000009496	1266	41	61	75	153	85	216	499	530	316	258	211	224	znf362
ENSXETG00000009497	3240	5	3	1	40	38	105	521	652	350	57	50	42	adamts10
ENSXETG00000009498	954	256	348	274	443	345	589	857	985	545	772	514	581	rnf41
ENSXETG00000009499	1938	514	711	592	558	470	1300	47	51	26	1595	1092	1183	slc39a5
ENSXETG00000009500	1619	67	99	64	204	149	354	232	258	164	313	239	234	trim62
ENSXETG00000009501	837	40	75	46	213	145	233	646	741	486	690	511	473	capns1
ENSXETG00000009502	1319	3667	5405	4711	5410	4263	7911	1177	1344	887	4224	3017	3317	ENSXETG00000009502
ENSXETG00000009503	3324	13	28	22	39	26	32	76	116	63	46	35	47	ankrd52
ENSXETG00000009505	2407	730	1013	838	486	399	1100	1140	1402	756	613	431	445	coq10a
ENSXETG00000009506	3201	6318	9169	7738	11410	8610	17332	9061	10316	6070	12092	8445	9643	cs
ENSXETG00000009509	3285	276	407	325	205	182	660	2733	3290	1959	698	499	515	myo1f
ENSXETG00000009510	1208	254	375	284	153	147	245	483	603	344	170	113	115	cnpy2
ENSXETG00000009511	1276	513	786	655	808	647	560	1196	1404	814	552	360	361	wdr61
ENSXETG00000009512	2397	0	0	0	1	0	0	19	22	10	1	0	1	ENSXETG00000009512
ENSXETG00000009516	729	1287	1920	1679	1463	1173	968	3195	3699	2246	1400	1016	1111	anp32c
ENSXETG00000009517	2266	10467	15453	13968	22212	17462	24882	28120	31366	18868	22493	15495	17356	ncl
ENSXETG00000009521	4109	170	222	152	455	382	646	523	652	313	462	287	377	armc9
ENSXETG00000009522	2934	2398	3743	3263	4066	3074	4957	6890	7753	4816	2918	2096	2345	psmd1
ENSXETG00000009523	1362	16	33	24	162	99	229	403	551	286	312	193	209	htr2b
ENSXETG00000009529	2060	168	228	190	148	108	389	299	378	216	200	127	165	paqr3
ENSXETG00000009530	3279	367	574	477	574	417	810	1438	1790	1055	730	463	588	bmp2k
ENSXETG00000009532	2813	119	164	166	318	212	246	340	393	217	402	333	365	slc20a2
ENSXETG00000009533	1833	815	1244	1005	1184	859	1928	2045	2397	1376	1545	1046	1243	kcmf1
ENSXETG00000009534	3990	417	605	522	801	614	944	941	1125	647	1583	1056	1212	ikbkap
ENSXETG00000009541	2236	111	183	154	170	146	209	246	257	143	294	201	186	rab11fip2
ENSXETG00000009543	3042	166	228	209	404	311	450	535	595	383	551	392	444	usp24
ENSXETG00000009544	2119	13	11	11	24	27	45	77	69	63	4	3	6	pif1
ENSXETG00000009545	1554	904	1460	1181	1084	875	1181	2276	2754	1645	1643	1137	1308	ebna1bp2
ENSXETG00000009556	1059	49	81	64	34	39	61	107	114	62	40	40	36	pstk
ENSXETG00000009559	3183	214	302	237	429	338	708	891	1052	604	869	580	662	ikzf5
ENSXETG00000009561	2317	2851	4200	3480	3429	2559	5025	2257	2630	1533	2395	1729	1844	acadsb
ENSXETG00000009562	2173	0	0	0	5	3	0	43	38	21	29	11	24	hmx3
ENSXETG00000009564	1138	465	691	584	817	678	914	1515	1721	1003	650	498	556	bub3
ENSXETG00000009569	1011	0	0	0	0	0	0	0	0	0	0	0	0	
ENSXETG00000009571	2739	10	21	22	77	76	24	112	127	62	89	67	39	cpxm2
ENSXETG00000009576	1868	332	486	432	443	363	618	487	555	312	859	609	656	oat.2
ENSXETG00000009578	2286	0	0	0	0	0	0	0	0	0	0	0	0	
ENSXETG00000009579	651	183	261	231	471	344	596	84	110	55	220	179	161	tmem125
ENSXETG00000009580	2895	65	101	85	423	313	722	1323	1497	931	535	376	423	tie1
ENSXETG00000009581	2490	17533	24593	20114	25561	19395	59515	16174	19753	10899	35085	24894	28044	elovl1
ENSXETG00000009582	7812	333	432	372	455	366	699	711	771	498	609	424	491	med8
ENSXETG00000009586	1491	0	0	2	2	1	4	1	1	4	9	1	2	znf577
ENSXETG00000009588	1182	0	0	0	0	0	0	0	0	0	0	0	0	
ENSXETG00000009592	816	0	0	1	0	0	0	3	5	2	0	0	0	plpp4
ENSXETG00000009595	2518	82	101	81	294	190	224	434	496	235	101	60	79	ska3
ENSXETG00000009596	1107	600	844	783	961	753	1156	923	1107	664	854	670	717	micu2
ENSXETG00000009598	685	531	783	647	540	425	479	1146	1187	812	404	316	317	sap18
ENSXETG00000009599	5586	68	86	85	266	213	360	528	604	373	342	240	242	lats2
ENSXETG00000009602	1203	146	201	187	259	156	365	321	399	251	357	230	248	ankrd13c
ENSXETG00000009605	1218	4	9	6	66	53	83	243	283	219	177	118	144	arrb1
ENSXETG00000009606	1228	36	29	37	69	54	123	100	125	57	122	82	87	fancf
ENSXETG00000009607	1845	0	0	1	5	5	2	4	5	3	1	2	4	lrfn3
ENSXETG00000009615	808	2201	3319	3034	3658	2832	15931	11608	12937	8137	11737	8314	8964	tpm2
ENSXETG00000009616	2694	128	201	185	723	541	547	799	949	561	765	550	549	klf8
ENSXETG00000009617	3006	3113	4508	3554	6189	4749	9428	6598	7822	4496	9425	6658	7780	znf534
ENSXETG00000009620	1206	0	0	0	0	0	0	0	0	0	0	0	0	
ENSXETG00000009622	491	0	0	0	0	0	0	0	0	0	0	0	0	ENSXETG00000009622
ENSXETG00000009623	810	23	31	27	35	28	51	56	62	42	55	34	33	rbmx2
ENSXETG00000009624	1182	0	0	0	0	0	0	0	0	0	0	0	0	
ENSXETG00000009626	1929	45	62	59	113	88	90	214	305	131	61	29	38	cenpi
ENSXETG00000009627	1101	4	4	2	1	0	6	24	32	16	73	62	65	ENSXETG00000009627

ENSXETG00000009628	1382	741	1192	952	865	699	1325	1172	1351	793	1000	670	847	pdzd11
ENSXETG00000009630	1458	0	1	0	1	2	0	5	8	4	5	1	2	ENSXETG00000009630
ENSXETG00000009631	261	0	0	0	0	0	0	0	0	0	0	0	0	
ENSXETG00000009632	1811	451	624	552	917	724	1892	1703	1885	1060	1895	1286	1428	tfcp2
ENSXETG00000009634	2687	45	50	63	123	82	281	482	630	387	130	116	93	csrnp2
ENSXETG00000009635	1152	162	276	211	125	84	126	192	264	154	91	81	78	letmd1
ENSXETG00000009636	1958	302	430	401	292	201	710	448	589	324	349	243	261	slc11a2
ENSXETG00000009637	1810	755	1031	888	1540	1120	1718	1713	1988	1156	1862	1255	1365	nup62
ENSXETG00000009640	657	0	0	0	31	22	23	0	1	1	51	25	45	prrg3
ENSXETG00000009641	1440	26	49	50	78	73	98	135	167	86	94	57	61	trmt12
ENSXETG00000009642	2073	538	742	667	845	623	714	1604	1741	1051	716	541	579	cstf2
ENSXETG00000009643	1762	22	21	26	43	25	77	100	121	73	89	69	69	zbtb43
ENSXETG00000009644	4056	5	1	3	34	27	22	318	340	209	103	73	79	ENSXETG00000009644
ENSXETG00000009646	3638	1094	1444	1241	5374	4235	6880	4505	4952	3027	5696	4078	4393	ctdsp2
ENSXETG00000009647	2610	4	7	6	16	9	8	10	11	10	22	11	15	avil
ENSXETG00000009648	1716	86	133	112	12	15	26	2616	3128	1967	124	95	110	nox1
ENSXETG00000009649	3351	31	45	37	144	119	76	452	611	350	559	405	454	xkrx
ENSXETG00000009650	1299	56	74	60	69	53	71	59	65	40	95	75	75	trmt2b
ENSXETG00000009653	879	886	1369	1182	512	360	363	615	711	455	430	311	357	tsfm
ENSXETG00000009654	1464	191	292	263	290	215	401	383	445	269	474	376	368	mettl21b
ENSXETG00000009655	1701	340	487	422	394	298	485	504	571	329	547	393	526	mettl1
ENSXETG00000009657	2732	7	1	7	3	2	0	11	14	10	0	2	3	cyp27b1
ENSXETG00000009658	2341	0	4	4	7	8	18	86	88	56	24	20	21	march9
ENSXETG00000009659	2429	1136	1672	1338	1199	868	1526	3239	4120	2251	832	570	684	tspan31
ENSXETG00000009660	918	17	26	29	54	44	80	295	342	201	420	283	324	ENSXETG00000009660
ENSXETG00000009661	2352	19	32	22	30	24	61	242	240	148	94	65	67	agap2
ENSXETG00000009662	2849	95	156	122	287	227	43	314	383	217	102	38	58	clspn
ENSXETG00000009664	2535	35	46	54	121	103	99	117	157	87	191	128	144	ago4
ENSXETG00000009665	1491	115	150	127	356	261	356	443	547	280	724	481	574	cyp2a13
ENSXETG00000009666	786	0	0	0	0	0	0	0	0	0	0	0	0	
ENSXETG00000009667	1211	5	3	3	7	13	16	36	47	23	37	15	10	c1orf216
ENSXETG00000009668	606	1882	2790	2415	2304	1733	2962	5199	5957	3573	1773	1177	1346	psmb2
ENSXETG00000009669	2163	196	271	256	227	185	310	567	628	374	442	296	329	ncdn
ENSXETG00000009670	975	0	0	0	0	0	0	0	0	0	0	0	0	
ENSXETG00000009671	1497	0	0	0	1	2	0	1	6	1	8	8	5	ENSXETG00000009671
ENSXETG00000009675	4533	235	381	321	725	596	748	1523	1704	1113	1079	791	878	zmym4
ENSXETG00000009678	942	0	0	0	0	0	0	0	0	0	0	0	0	
ENSXETG00000009679	939	0	0	0	0	0	0	0	0	0	0	0	0	
ENSXETG00000009682	3476	1031	1371	1248	4178	3271	4033	5133	5789	3531	3896	2827	3141	sfpq
ENSXETG00000009683	1023	51	67	59	268	184	688	536	625	371	59	38	46	tpbgl
ENSXETG00000009686	1302	19	39	23	231	207	113	217	211	116	239	167	168	insm1
ENSXETG00000009689	696	1011	1459	1245	2425	1766	2993	3449	3702	2362	1536	1083	1175	ppp1ca
ENSXETG00000009691	1407	58	86	62	179	146	116	218	215	140	140	89	83	ENSXETG00000009691
ENSXETG00000009695	3892	0	2	2	1	4	1	12	11	9	13	6	6	atp2a1
ENSXETG00000009697	3774	1	2	0	158	92	13	10	15	12	16	6	7	cfap61
ENSXETG00000009698	2221	401	557	501	735	594	1043	905	1122	578	1114	782	886	crnkl1
ENSXETG00000009700	3081	97	176	123	334	242	591	1067	1306	780	692	442	551	rin2
ENSXETG00000009704	945	9	12	10	55	39	73	323	387	200	260	183	200	tnfrsf19
ENSXETG00000009705	2136	527	713	616	515	374	602	840	918	546	390	291	277	mipep
ENSXETG00000009707	3252	195	272	227	579	399	1278	446	488	296	689	505	578	spata13
ENSXETG00000009708	726	10	8	3	3	4	7	0	0	0	0	0	0	c1qtnf9b
ENSXETG00000009713	1146	6	7	8	6	13	19	15	26	19	50	32	37	ENSXETG00000009713
ENSXETG00000009717	8245	40	54	54	219	166	197	1039	1186	698	435	312	313	tln2
ENSXETG00000009718	2794	4983	7348	6101	7440	5662	13592	22949	25895	15289	12555	8859	9800	tpm1
ENSXETG00000009720	1392	252	383	312	570	472	900	418	487	318	531	319	416	lactb
ENSXETG00000009721	624	75	116	87	93	57	167	321	357	226	146	91	123	rab8b
ENSXETG00000009722	1014	7	9	9	7	7	14	56	56	34	20	22	17	ca12
ENSXETG00000009724	1035	1443	1974	1582	1434	1156	2435	3217	3991	2115	1533	1097	1254	rheb
ENSXETG00000009726	670	0	1	0	0	0	2	18	26	9	3	1	1	crygn
ENSXETG00000009727	2467	107	158	173	260	193	2245	882	983	563	1269	892	968	prkag1
ENSXETG00000009728	1587	122	209	173	467	299	813	659	690	472	389	272	353	usp3
ENSXETG00000009730	4506	133	195	177	508	399	599	1003	1084	693	1004	687	856	kmt2c
ENSXETG00000009733	681	0	0	1	8	9	11	9	6	7	5	6	3	fbxl22
ENSXETG00000009734	14526	811	1169	1051	3190	2392	3754	2301	2648	1512	4731	3298	3779	herc1
ENSXETG00000009739	879	49	61	53	65	52	104	66	64	40	51	48	44	xrcc2
ENSXETG00000009747	2152	436	618	538	395	281	577	384	432	248	590	412	465	dapk2
ENSXETG00000009751	3147	118	141	117	239	201	502	2806	3240	2084	627	447	487	tbc1d2b
ENSXETG00000009754	1971	1787	2459	2068	1929	1525	2754	1866	2090	1224	3057	2216	2359	idh3a
ENSXETG00000009756	2212	4	2	3	17	10	8	37	40	31	101	69	57	acsbg1
ENSXETG00000009758	975	0	0	0	0	0	0	0	0	0	0	0	0	
ENSXETG00000009759	954	1	4	3	7	10	1	10	6	5	35	19	28	ENSXETG00000009759
ENSXETG00000009763	1375	357	440	384	353	255	378	255	330	186	178	149	143	hddc3
ENSXETG00000009764	1817	130	194	157	194	182	343	267	364	183	360	277	300	hsf3
ENSXETG00000009770	2954	483	647	575	1217	933	2025	10478	11729	7175	2346	1769	1838	msn
ENSXETG00000009773	216	2	8	7	7	2	3	6	9	5	4	1	1	crabp1
ENSXETG00000009774	3189	323	474	414	677	536	1184	1032	1215	670	824	647	712	ireb2
ENSXETG00000009775	2019	16	21	20	17	13	36	76	112	52	40	25	24	sytl3
ENSXETG00000009776	3942	3994	5852	4869	13767	10526	9797	14206	16926	9737	12467	8778	9942	ezr
ENSXETG00000009777	1978	10227	13665	11331	8637	6566	16043	6861	8738	4919	9317	6488	7576	tmbim6
ENSXETG00000009778	1053	30	56	36	343	250	93	218	265	150	59	52	57	rsph3
ENSXETG00000009780	3639	19	31	25	240	164	422	1483	1658	974	528	360	445	nckap5l
ENSXETG00000009781	2434	51	59	45	75	59	93	340	431	254	179	137	147	tagap
ENSXETG00000009782	1665	416	572	475	891	629	864	1108	1336	673	1351	940	1135	kansl2
ENSXETG00000009783	2094	883	1203	1082	851	650	652	1262	1486	826	838	593	690	ltv1
ENSXETG00000009784	1893	97	147	132	203	161	283	264	307	203	315	232	219	ccnt1
ENSXETG00000009785	2384	83	134	130	231	146	488	530	573	353	604	418	462	phactr2
ENSXETG00000009786	1719	1043	1595	1339	1450	1058	3421	2325	2669	1624	3195	2370	2527	fuca2
ENSXETG00000009787	1159	98	144	113	277	219	453	366	404	269	262	179	187	cdk8
ENSXETG00000009788	1582	667	1032	841	719	565	1187	625	811	435	533	372	428	pex3
ENSXETG00000009789	3492	55	91	78	111	102	249	415	431	279	288	184	244	adcy6
ENSXETG00000009790	1037	165	243	217	148	134	254	325	382	205	234	159	190	adat2
ENSXETG00000009792	576	0	0	0	0	0	0	2	3	1	0	1	1	
ENSXETG00000009798	3339	0	0	0	0	0	0	0	0	0	0	0	0	ENSXETG00000009798
ENSXETG00000009799	1068	99	136	140	215	141	311	301	385	182	316	237	273	usp12
ENSXETG00000009800	1542	1	1	0	0	1	5	8	7	2	7	4	7	cacnb3
ENSXETG00000009802	523	29809	44602	38691	50692	38336	60773	46931	53412	34018	108087	75531	83000	rpl21
ENSXETG00000009806	1935	2467	3578	2904	6492	5025	9782	402	503	310	4097	2734	3278	slc28a2
ENSXETG00000009807	3023	611	923	740	1309	1062	1627	1688	1885	1154	1464	1029	1163	ddx23
ENSXETG00000009811	1688	129	188	134	41	33	88	262	270	147	110	63	83	rnd1
ENSXETG00000009814	1531	233	358	295	533	373	978	725	849	516	818	584	613	wdr45
ENSXETG00000009815	1503	1	3	5	68	53	4	6	4	4	9	9	8	ccdc65
ENSXETG00000009816	615	1247	1841	1683	1570	1236	1096	907	991	641	1229	866	930	fkbp11
ENSXETG00000009817	1119	0	0	0	0	0	0	0	0	0	0	0	0	
ENSXETG00000009818	3188	514	830	665	972	743	854	1723	1997	1233	536	418	448	pitrm1
ENSXETG00000009822	5575	14	8	20	5	6	14	83	91	46	16	21	18	alpk3
ENSXETG00000009828	1104	0	0	0	0	0	0	0	0	1	0	1	0	zar1
ENSXETG00000009829	1752	169	224	239	373	234	589	612	758	429	397	296	344	slain2
ENSXETG00000009830	1059	4	2	1	1	1	2	10	17	10	9	4	3	slc10a4
ENSXETG00000009831	1857	93	127	119	98	79	209	269	357	192	185	122	104	tec
ENSXETG00000009832	1209	549	793	773	400	313	2066	1165	1226	805	458	348	357	nipal1
ENSXETG00000009833	1983	13	13	8	9	16	58	8	2	9	4	2	4	cnga1

ENSXETG00000009834	860	0	0	0	0	0	0	0	0	0	0	0	0	
ENSXETG00000009835	1797	69	79	65	100	78	120	763	843	440	115	86	83	cercam
ENSXETG00000009836	3161	598	896	764	769	567	1132	808	880	536	986	664	711	nfxl1
ENSXETG00000009837	2940	0	0	0	5	6	4	17	18	14	70	33	38	corin
ENSXETG00000009839	4131	4	11	5	22	15	18	130	167	103	18	15	20	atp10d
ENSXETG00000009843	1266	623	884	755	469	371	1120	779	1014	575	470	331	345	commd8
ENSXETG00000009845	888	0	0	0	0	0	0	0	0	0	0	0	0	
ENSXETG00000009846	3264	1	3	1	5	3	5	11	14	4	7	3	15	ENSXETG00000009846
ENSXETG00000009858	3674	32	53	42	102	74	55	114	126	86	70	53	33	ENSXETG00000009858
ENSXETG00000009860	918	1779	2651	2414	924	680	1279	1908	2108	1342	1210	829	904	btn2a2
ENSXETG00000009861	1461	50	67	58	65	43	162	102	146	77	35	11	16	ENSXETG00000009861
ENSXETG00000009862	1584	63	102	99	32	36	302	251	245	168	42	22	25	ENSXETG00000009862
ENSXETG00000009863	3882	0	1	4	16	7	17	115	133	89	16	15	23	adamts12
ENSXETG00000009864	3265	484	712	492	470	394	622	573	710	393	547	383	429	samd8
ENSXETG00000009865	1408	19489	28230	23857	21641	16356	22988	14923	18114	10678	23711	16394	18242	vdac2
ENSXETG00000009866	2921	21	25	15	87	74	137	445	490	326	222	144	168	znf503
ENSXETG00000009868	3250	1456	2255	1868	1784	1360	1567	4337	5050	3016	1472	1052	1129	tars
ENSXETG00000009871	435	126	163	154	50	31	216	166	176	100	94	72	77	c10orf11
ENSXETG00000009872	1446	200	275	251	330	249	551	367	464	265	519	370	399	trhd
ENSXETG00000009873	2478	524	811	667	428	351	385	696	868	510	848	609	719	wdr75
ENSXETG00000009874	1747	4449	6378	5623	4243	3385	7665	23330	28048	16494	7949	5571	6283	slc40a1
ENSXETG00000009878	2120	520	693	579	662	473	803	1184	1492	802	712	470	550	asnsd1
ENSXETG00000009880	372	194	265	209	336	227	335	457	465	294	332	199	244	gemin7
ENSXETG00000009881	1261	170	214	206	145	138	235	186	255	149	101	74	84	XB-GENE-5749128
ENSXETG00000009883	1596	43	42	49	108	90	88	153	167	100	64	60	75	osgepl1
ENSXETG00000009884	2388	91	136	111	175	135	339	359	452	233	259	204	198	ormdl1
ENSXETG00000009885	2866	59	81	61	97	74	125	261	270	196	169	131	135	pms1
ENSXETG00000009890	1110	0	0	1	0	1	0	11	6	3	0	0	0	
ENSXETG00000009891	2237	442	607	502	376	315	506	648	814	471	372	254	276	ptcd1
ENSXETG00000009892	1514	279	377	312	388	325	683	580	658	370	644	402	485	cpsf4
ENSXETG00000009893	387	4105	6164	5490	4288	3126	4611	3320	4012	2708	2831	1984	2184	atp5j2
ENSXETG00000009895	7620	210	306	245	417	298	248	322	442	235	556	365	445	urb1
ENSXETG00000009896	1167	31	44	27	29	30	34	75	84	61	102	73	73	eva1c
ENSXETG00000009898	5355	0	0	0	3	2	0	0	0	0	0	0	0	
ENSXETG00000009899	4776	261	367	322	537	449	973	899	987	566	849	574	669	synj1
ENSXETG00000009900	1608	1	1	1	3	3	2	17	19	16	7	2	0	ENSXETG00000009900
ENSXETG00000009902	994	90	126	101	154	106	187	314	345	239	132	86	80	c21orf59
ENSXETG00000009903	3549	475	698	628	804	581	819	931	1105	650	1419	1085	1196	paxbp1
ENSXETG00000009907	2212	365	602	476	677	548	867	1134	1179	817	1237	877	935	man1a2
ENSXETG00000009908	1779	387	539	515	911	718	2458	3669	4095	2386	891	604	682	atp6v1a
ENSXETG00000009909	513	1052	1513	1343	970	809	1273	1745	1932	1222	1306	981	1008	naa50
ENSXETG00000009913	8219	36	44	42	196	159	180	316	356	220	92	72	75	polq
ENSXETG00000009914	795	2	1	1	0	1	1	1	0	2	1	2	2	ENSXETG00000009914
ENSXETG00000009916	1326	3	6	3	18	7	13	27	18	18	54	46	44	hgd
ENSXETG00000009918	1477	64	130	96	274	178	432	406	409	306	429	308	341	gtf2e1
ENSXETG00000009919	276	69	80	66	77	52	81	87	119	75	60	50	43	rabl3
ENSXETG00000009920	3454	939	1371	1212	1555	1191	1368	1462	1653	1032	1651	1245	1285	wdr3
ENSXETG00000009921	1386	296	454	371	608	465	1536	1120	1264	790	1145	863	952	gdap2
ENSXETG00000009936	3543	0	0	0	0	0	0	0	0	0	0	0	0	
ENSXETG00000009939	1023	102	158	123	148	124	145	283	302	186	153	100	124	prmt6
ENSXETG00000009943	1401	0	0	2	9	1	6	27	24	22	26	13	20	ntng1
ENSXETG00000009944	2556	11	19	20	56	34	102	294	388	222	124	86	86	vav3
ENSXETG00000009948	2036	53	77	53	203	136	232	396	413	278	287	244	251	fam102b
ENSXETG00000009949	3396	0	0	0	0	0	0	0	0	0	0	0	0	
ENSXETG00000009951	2370	0	0	0	0	0	0	0	0	0	0	0	0	
ENSXETG00000009952	3787	198	245	237	249	211	420	457	580	315	421	274	353	zbtb41
ENSXETG00000009953	4134	0	0	0	0	0	0	1	0	1	0	0	0	crb1
ENSXETG00000009955	10676	93	161	171	471	367	580	601	807	411	136	77	112	dennd1b
ENSXETG00000009957	1197	0	0	0	0	1	2	1	4	0	0	1	0	lhx9
ENSXETG00000009961	1437	91	151	123	173	128	303	399	503	272	459	350	383	kcnk5
ENSXETG00000009962	915	124	179	150	279	191	513	527	633	348	437	299	318	nek7
ENSXETG00000009963	945	0	0	0	0	0	0	0	0	0	0	2	0	ENSXETG00000009963
ENSXETG00000009964	2511	302	464	424	929	681	951	972	1157	729	1640	1119	1253	cmtr1
ENSXETG00000009966	796	0	0	0	0	0	1	13	19	9	11	4	9	atp6v1g3
ENSXETG00000009971	939	0	0	0	0	0	0	0	0	0	0	0	0	
ENSXETG00000009974	927	0	0	0	0	0	0	0	0	0	0	0	0	
ENSXETG00000009976	839	281	372	331	259	225	398	331	394	245	185	137	140	slc39a11
ENSXETG00000009978	1119	2	3	3	26	21	13	26	35	8	68	56	63	sstr2
ENSXETG00000009979	2934	563	806	727	998	733	1836	1156	1255	759	1201	841	938	cog1
ENSXETG00000009980	561	98	148	140	84	54	213	151	183	84	70	57	57	ptrh1
ENSXETG00000009983	1141	96	135	125	240	177	142	679	730	395	47	40	32	birc5.2
ENSXETG00000009985	1424	1329	1921	1631	1717	1217	1736	1022	1198	735	649	482	499	tha1p
ENSXETG00000009986	1043	103	171	173	186	129	266	214	280	159	180	134	155	psmc3ip
ENSXETG00000009987	1628	313	525	443	398	291	836	679	791	486	419	277	340	pgs1
ENSXETG00000009988	594	4	8	7	64	59	37	27	33	25	28	25	27	ENSXETG00000009988
ENSXETG00000009990	2488	972	1464	1290	2557	1849	3773	9189	10889	6237	1232	865	989	mospd2
ENSXETG00000009993	2503	2324	3362	2899	3162	2397	2070	1541	1761	1063	1692	1192	1334	ndrg4
ENSXETG00000009994	2628	1	12	3	126	83	9	11	12	12	12	5	9	mycbpap
ENSXETG00000009996	1290	204	339	262	291	192	229	245	268	157	200	131	145	rsad1
ENSXETG00000009997	4041	383	532	454	1851	1347	1597	1872	2124	1251	1315	900	1101	ndst2
ENSXETG00000009999	2202	446	715	602	1363	923	2636	782	864	538	1945	1425	1456	spata20
ENSXETG00000010000	5403	270	401	365	817	612	972	2057	2434	1392	1143	808	873	zswim8
ENSXETG00000010001	5173	280	414	330	913	681	1146	1242	1346	851	1179	901	874	znf687
ENSXETG00000010002	2966	10	15	12	112	95	394	410	479	267	202	148	160	ENSXETG00000010002
ENSXETG00000010005	1308	622	909	774	621	487	665	500	552	296	520	346	411	chchd1
ENSXETG00000010007	1816	1095	1517	1317	2887	2285	2383	2486	2635	1799	2575	1798	2033	myo1e.2
ENSXETG00000010009	4797	300	422	388	861	590	1330	1157	1404	792	953	736	811	usp54
ENSXETG00000010012	3549	1420	2018	1710	2228	1643	2816	3017	3591	2113	2395	1775	1964	sec24d
ENSXETG00000010013	1499	1	0	0	0	0	0	0	1	0	0	0	0	myoz1
ENSXETG00000010014	3377	2573	3582	3209	2959	2337	2569	3610	4065	2283	2403	1755	1903	nolc1
ENSXETG00000010015	612	35	58	33	222	167	109	172	185	104	253	218	177	slc37a1
ENSXETG00000010016	1041	0	0	0	2	0	0	0	0	0	0	0	0	
ENSXETG00000010017	795	460	742	661	547	384	1015	178	203	139	144	101	105	elovl3
ENSXETG00000010019	5863	589	832	674	1116	804	995	1200	1488	806	981	702	827	gbf1
ENSXETG00000010020	3058	1693	2273	1978	6611	5100	7594	2660	3175	1754	5638	4060	4489	chka
ENSXETG00000010022	879	0	3	1	3	0	5	4	10	6	3	5	1	pitx3
ENSXETG00000010024	4800	79	139	88	669	549	788	1611	1822	1019	1146	902	952	lrp5
ENSXETG00000010025	2744	359	557	446	913	717	1379	1395	1759	1030	1169	821	878	kmt5b
ENSXETG00000010030	1263	116	129	110	568	396	570	3226	3505	2251	1144	858	915	myadm
ENSXETG00000010034	1761	0	0	3	5	3	9	11	16	14	7	4	4	prkcg
ENSXETG00000010035	2022	3	5	5	2	5	9	35	54	15	7	7	5	tshr
ENSXETG00000010036	4918	556	825	717	1016	787	1803	1666	2067	1178	1474	942	1116	gtf2a1
ENSXETG00000010038	3477	1398	2039	1806	2478	1925	2660	2342	2665	1555	2201	1668	1768	sel1l
ENSXETG00000010042	1893	339	565	476	423	328	1130	1594	1886	1046	378	254	302	gusb
ENSXETG00000010044	5625	0	0	1	0	0	1	3	3	2	0	0	0	
ENSXETG00000010046	1725	0	0	0	1	0	0	9	11	7	0	2	2	
ENSXETG00000010057	1808	2	9	4	50	43	9	243	256	134	47	34	43	pitpnc1
ENSXETG00000010058	2503	36	50	36	280	203	198	636	673	437	612	418	545	axin2
ENSXETG00000010059	2438	906	1281	1063	395	318	499	379	452	274	284	219	227	vkorc1l1
ENSXETG00000010060	2070	386	533	451	662	481	1459	827	1057	589	684	507	522	c18orf8
ENSXETG00000010061	2704	291	375	314	769	571	1003	1031	1195	671	945	585	690	ddx6

ENSXETG00000010062	1471	4802	7081	5863	4666	3609	4489	4706	5995	3296	2714	1886	2088	chchd2
ENSXETG00000010064	1782	7	8	5	2	1	9	42	64	23	95	78	75	phkg1
ENSXETG00000010067	1074	4	3	3	1	1	1	11	11	10	7	1	4	cxcr5
ENSXETG00000010068	1377	210	268	269	362	254	503	445	503	279	234	155	173	sumf2
ENSXETG00000010069	4065	206	345	290	569	459	935	570	611	385	963	651	796	bcl9l
ENSXETG00000010071	1017	113	147	143	198	154	181	201	206	121	264	196	187	ccdc84
ENSXETG00000010072	1044	628	961	772	546	439	868	987	1221	684	716	473	562	trappc4
ENSXETG00000010074	4037	1346	1818	1492	1013	805	787	312	410	242	60	36	53	psph
ENSXETG00000010075	2959	3171	4770	3942	1283	1032	538	1468	1569	969	334	227	253	hyou1
ENSXETG00000010077	1959	4160	6174	5331	5963	4483	6363	13263	15531	8974	8446	5989	6548	cct6a
ENSXETG00000010083	2004	17	19	15	55	33	69	68	74	57	53	48	44	ENSXETG00000010083
ENSXETG00000010084	4263	241	289	252	462	358	790	691	760	452	563	448	483	vps11
ENSXETG00000010085	1399	7343	10560	8637	5342	4243	6579	1159	1504	856	431	313	395	ENSXETG00000010085
ENSXETG00000010086	918	50	60	43	194	155	202	477	536	319	340	223	282	gbas
ENSXETG00000010087	11749	103	162	162	526	416	448	695	767	500	1792	1306	1512	kmt2a
ENSXETG00000010088	924	483	739	624	472	393	725	562	677	375	455	351	397	mrps17
ENSXETG00000010089	2448	0	0	0	0	0	0	0	0	0	0	0	0	
ENSXETG00000010090	1791	334	491	396	273	227	572	397	468	273	417	259	335	ENSXETG00000010090
ENSXETG00000010093	3859	799	1138	965	1117	782	1745	1808	2255	1278	1451	1024	1134	sbds
ENSXETG00000010094	3599	454	678	564	660	506	596	682	855	521	671	471	550	tyw1
ENSXETG00000010095	3038	11	17	18	203	164	347	992	1123	629	491	330	331	nrp2
ENSXETG00000010098	795	17446	25639	21971	28597	21761	32330	29004	32789	18827	64251	44539	49108	eef1b2
ENSXETG00000010108	2457	2285	3507	2918	4230	3236	3762	4125	4689	2913	2496	1785	1975	ndufs1
ENSXETG00000010109	1581	1162	1707	1458	1514	1009	2989	154	174	114	291	242	245	wbscr17
ENSXETG00000010111	2394	4757	7143	5987	4966	3571	4217	3979	4625	2656	3247	2312	2499	atp5l
ENSXETG00000010112	4483	483	681	644	1327	1034	2035	1281	1597	938	1902	1308	1441	ube4a
ENSXETG00000010117	827	47	74	60	39	20	41	204	298	163	145	116	126	cd3g
ENSXETG00000010118	2622	6	8	6	10	4	20	146	179	117	26	23	23	adam23
ENSXETG00000010119	1114	48	58	67	40	29	72	280	334	199	168	117	129	cd3e
ENSXETG00000010120	2598	147	223	189	408	309	508	663	832	444	647	457	530	mpzl2
ENSXETG00000010122	3673	8	5	4	42	35	125	146	163	84	384	291	322	scn2b
ENSXETG00000010123	1886	14	22	17	36	18	25	71	85	37	146	105	139	scn4b
ENSXETG00000010124	2070	402	542	498	751	510	931	465	477	275	726	485	536	tmprss4
ENSXETG00000010125	1107	0	1	0	5	2	2	227	335	196	2	1	3	tmprss13
ENSXETG00000010128	2122	0	1	0	7	7	7	59	85	41	35	25	25	nefm
ENSXETG00000010129	1056	16	17	14	16	13	58	250	343	185	93	81	73	cysltr1
ENSXETG00000010130	657	0	0	0	0	0	0	0	0	0	0	0	0	
ENSXETG00000010131	2251	0	0	0	0	0	0	1	0	0	0	1	1	
ENSXETG00000010132	3734	164	252	227	324	209	419	1374	1476	877	557	378	403	fndc3a.2
ENSXETG00000010133	1053	0	0	1	0	0	0	13	14	13	1	0	2	cers1
ENSXETG00000010134	1211	4340	6296	5199	4887	3805	4143	5460	6390	3751	3026	2006	2288	cope
ENSXETG00000010135	3172	929	1307	1137	631	501	777	872	1000	577	1282	887	1043	ddx49
ENSXETG00000010136	3804	12	15	10	20	24	58	508	549	342	52	27	30	homer3
ENSXETG00000010137	957	0	0	0	0	0	0	0	0	0	0	0	1	
ENSXETG00000010138	809	454	650	517	505	399	951	820	982	534	706	494	539	taf9b
ENSXETG00000010141	2511	5	5	6	15	13	27	107	115	64	46	24	36	lrch2
ENSXETG00000010142	1667	1	3	0	6	2	11	34	30	22	74	50	41	nefl
ENSXETG00000010147	333	0	0	0	2	1	1	3	4	3	3	2	3	gnrh1
ENSXETG00000010149	1761	9	9	4	45	40	8	7	8	4	8	6	4	dthd1
ENSXETG00000010150	5082	0	0	0	4	1	1	7	15	7	5	1	3	NWD2
ENSXETG00000010153	2415	0	0	0	0	0	0	4	2	1	1	1	0	
ENSXETG00000010155	774	3	3	4	9	10	7	21	25	14	16	3	10	pou2af1
ENSXETG00000010159	2344	2355	3344	2969	3259	2533	4087	6209	7081	4328	5300	3722	4098	ppp2r2a
ENSXETG00000010161	1786	107	143	110	178	137	212	395	513	276	286	190	232	parp14.3
ENSXETG00000010162	882	0	0	0	1	0	0	0	0	0	0	0	0	
ENSXETG00000010164	1050	3	4	4	10	3	15	7	8	5	10	18	19	layn
ENSXETG00000010165	3109	36	28	42	99	61	108	630	681	386	219	170	177	sik2
ENSXETG00000010166	1313	6706	9682	8043	4676	3617	14571	6781	7963	4442	12532	8877	9908	adh6
ENSXETG00000010167	1933	1064	1538	1264	1692	1316	1798	2919	3470	1973	1210	847	1013	ppp2r1b
ENSXETG00000010168	1527	400	577	492	935	659	2187	1571	1766	1014	1397	959	1115	bnip3l
ENSXETG00000010169	1698	89	134	129	187	136	253	183	218	130	228	146	173	alg9
ENSXETG00000010171	3523	331	481	424	593	471	823	401	560	268	431	281	321	tdrd7
ENSXETG00000010172	1809	59	77	58	33	23	38	53	53	51	76	41	52	fdxacb1
ENSXETG00000010173	1080	11	11	9	51	37	147	121	150	89	299	193	256	tmod1
ENSXETG00000010174	525	0	0	0	2	0	2	0	0	0	3	0	1	cryab
ENSXETG00000010175	522	0	0	0	0	1	1	2	0	2	4	4	3	hspb2
ENSXETG00000010177	1959	147	240	172	99	79	120	196	278	124	69	58	59	tstd2
ENSXETG00000010179	2418	340	508	383	540	370	464	867	1094	580	495	349	407	ncbp1
ENSXETG00000010181	1012	85	111	93	88	61	117	132	168	106	75	50	67	xpa
ENSXETG00000010182	1233	91	109	90	158	116	287	169	197	123	309	209	264	trmo
ENSXETG00000010183	1708	226	293	270	367	265	391	1003	1254	722	675	471	543	anp32b
ENSXETG00000010185	1905	5	3	7	26	20	26	116	134	80	62	38	34	dixdc1
ENSXETG00000010186	1584	658	994	870	1581	1244	2658	2963	3721	2127	2195	1529	1771	coro2a
ENSXETG00000010187	930	961	1471	1219	1615	1235	3697	751	851	513	2295	1602	1659	rbks
ENSXETG00000010190	2862	50	92	76	81	77	173	167	231	109	134	102	115	prkab2
ENSXETG00000010191	931	68	117	88	148	132	168	331	365	225	223	133	139	c11orf57
ENSXETG00000010192	8919	158	229	196	236	161	506	1206	1309	792	600	393	451	pde4dip
ENSXETG00000010194	2229	11	30	23	42	36	23	62	72	48	10	6	6	pih1d2
ENSXETG00000010195	1854	0	0	1	59	52	7	4	14	5	2	4	2	stkld1
ENSXETG00000010197	1366	119	189	155	186	159	155	277	328	174	141	111	126	thap1
ENSXETG00000010198	2242	1001	1369	1154	1590	1207	1234	2206	2522	1424	1009	730	791	dlat
ENSXETG00000010199	1113	35	57	49	123	110	169	192	225	152	234	152	171	jund.2
ENSXETG00000010200	5674	671	1020	894	1589	1122	2287	2275	2496	1509	1566	1094	1261	kiaa0368
ENSXETG00000010201	1239	419	637	524	536	459	718	781	903	591	448	339	347	bre
ENSXETG00000010202	2985	2926	4298	3692	9200	7144	9238	11867	13089	8457	6416	4579	4810	uba1
ENSXETG00000010205	1940	2412	3537	2997	2416	1832	3249	2879	3494	1990	1643	1156	1366	srp72
ENSXETG00000010208	951	31	71	55	206	139	283	1747	1887	1260	592	484	437	fosl2
ENSXETG00000010209	3618	136	168	194	702	531	505	1140	1421	844	170	103	146	smc2
ENSXETG00000010212	1084	243	330	319	255	244	157	151	190	129	177	118	121	hopx
ENSXETG00000010213	2103	13	22	29	55	48	165	571	640	389	158	114	148	hook3
ENSXETG00000010214	948	638	962	813	1428	1056	1715	1791	2087	1203	1594	1158	1263	ppp1cb
ENSXETG00000010217	1131	973	1470	1171	1122	860	1739	1408	1589	1006	1789	1211	1352	dnajc25
ENSXETG00000010219	4689	1099	1570	1308	934	796	4301	1973	2393	1377	1565	1153	1278	ugcg
ENSXETG00000010224	1737	552	762	665	918	753	1629	1956	2360	1238	1183	835	877	ppp2r5c
ENSXETG00000010225	471	84	114	115	177	144	188	130	136	75	96	74	51	ENSXETG00000010225
ENSXETG00000010226	1002	0	2	0	1	1	7	0	1	1	3	5	2	ENSXETG00000010226
ENSXETG00000010229	561	2368	3595	2996	2710	2051	2369	1280	1437	781	2107	1506	1681	prdx3
ENSXETG00000010230	1722	6	2	5	23	35	138	64	76	46	57	35	44	grk5
ENSXETG00000010231	730	3	12	10	7	7	30	274	295	197	68	66	52	rgs10
ENSXETG00000010233	2751	81	124	98	123	76	325	492	480	312	223	174	188	bag3
ENSXETG00000010234	1284	559	835	694	988	808	1106	1699	1975	1199	1253	900	1025	tial1
ENSXETG00000010235	4313	166	221	176	255	193	330	1071	1169	775	486	365	410	inpp5f
ENSXETG00000010237	2285	242	386	356	860	630	529	726	775	434	805	488	587	mcmbp
ENSXETG00000010238	3030	968	1467	1278	1652	1251	1769	1596	1716	1144	1731	1202	1306	sec23ip
ENSXETG00000010239	1632	0	0	0	0	0	0	0	0	0	0	0	0	
ENSXETG00000010241	4384	3902	5807	5159	16008	12257	16748	14183	15391	9973	32145	22784	24258	eif3a
ENSXETG00000010243	1256	213	321	247	361	278	645	608	745	409	800	619	602	fam204a
ENSXETG00000010244	2464	3	4	2	30	35	64	164	184	114	146	96	107	nova1
ENSXETG00000010247	1215	0	0	0	0	0	0	0	0	0	0	0	0	
ENSXETG00000010248	3820	341	563	444	993	725	991	1708	1985	1250	1230	927	984	prkd1
ENSXETG00000010249	1320	0	0	0	0	0	3	0	2	0	0	0	0	chrna6

ENSXETG00000010250	2067	1	0	0	1	0	0	2	3	3	0	1	2	chrnb3
ENSXETG00000010251	5462	159	253	206	154	118	42	436	565	323	104	80	80	hsp90aa1.1
ENSXETG00000010252	1366	397	616	515	340	251	450	959	1127	656	363	260	288	dctn6
ENSXETG00000010253	2332	811	1167	1014	969	797	1459	1246	1482	882	1298	892	1067	wdr20
ENSXETG00000010255	621	598	855	743	377	273	622	645	798	456	450	265	317	cinp
ENSXETG00000010256	3687	102	133	101	165	133	344	467	494	298	232	163	183	tecpr2
ENSXETG00000010257	4850	205	306	276	923	715	944	627	690	385	1987	1402	1533	znf608
ENSXETG00000010259	4456	648	952	786	880	654	1040	1152	1385	766	947	641	682	csnk1g3
ENSXETG00000010260	3204	32	43	43	217	212	79	189	215	164	111	64	85	cep120
ENSXETG00000010261	1623	0	0	0	2	1	0	29	26	20	2	3	0	prdm6
ENSXETG00000010266	3137	590	916	797	2241	1770	1967	2059	2393	1423	2086	1418	1541	tle4
ENSXETG00000010267	574	7369	10786	9402	8958	6987	8796	15240	18247	11245	4060	2796	3321	ENSXETG00000010267
ENSXETG00000010269	462	74	119	90	195	175	214	356	372	231	252	171	192	ENSXETG00000010269
ENSXETG00000010280	2667	92	156	147	290	223	444	737	820	494	149	109	141	pik3c3
ENSXETG00000010282	3186	2615	3598	3148	3531	2871	6890	6555	7851	4508	5263	3701	4199	cdx1
ENSXETG00000010285	1989	590	770	659	916	676	1275	1720	1945	1152	1000	712	852	rps6ka3
ENSXETG00000010286	3955	1	0	1	0	3	1	6	7	5	3	2	3	cnksr2
ENSXETG00000010288	1952	148	215	174	301	194	731	588	649	376	384	334	340	atxn3
ENSXETG00000010289	2648	774	1224	1017	1075	727	1333	2231	2676	1540	760	506	607	cpsf2
ENSXETG00000010290	1638	0	0	0	0	1	1	5	5	2	3	0	2	slc24a4
ENSXETG00000010291	1536	81	113	93	314	224	300	641	682	463	473	303	368	ENSXETG00000010291
ENSXETG00000010292	2841	71	101	88	159	132	418	2420	2775	1490	554	378	383	rin3
ENSXETG00000010300	2238	9059	13019	10880	9345	7127	55402	45965	55052	30307	17185	11974	13595	lgmn
ENSXETG00000010301	3219	971	1473	1150	1612	1285	2196	2384	2765	1565	2217	1568	1764	golga5
ENSXETG00000010302	2002	818	1206	1021	4128	3253	3216	4621	5099	2952	10114	6823	7835	chga
ENSXETG00000010303	1345	1351	2088	1719	2323	1671	3138	1815	2119	1195	3576	2526	2811	itpk1
ENSXETG00000010304	1873	564	823	703	603	459	1052	1542	1784	1036	652	479	503	taf6l
ENSXETG00000010305	1808	284	453	359	718	561	989	1052	1239	723	877	601	691	ubr7
ENSXETG00000010306	1042	68	108	77	128	113	191	409	529	277	114	91	102	spc25
ENSXETG00000010307	3396	43	54	48	150	114	233	384	437	254	297	209	256	btbd7
ENSXETG00000010308	7901	3	2	8	25	6	21	19	19	21	32	40	29	unc79
ENSXETG00000010311	1923	2	5	5	7	3	38	160	187	102	107	66	72	asb2
ENSXETG00000010313	846	135	213	172	75	45	49	289	353	212	132	131	118	otub2
ENSXETG00000010314	2340	1069	1435	1232	980	838	679	1531	1792	1064	1169	818	941	ddx24
ENSXETG00000010315	2642	3	12	5	122	81	35	257	318	185	100	90	92	ppp4r4
ENSXETG00000010316	1233	0	0	0	2	1	1	46	68	44	13	9	8	serpina10
ENSXETG00000010317	7224	528	755	686	2398	1863	3900	4527	5062	3091	5133	3738	3983	crebbp
ENSXETG00000010319	4419	210	334	276	341	288	317	966	1103	663	359	268	310	slx4
ENSXETG00000010321	2420	63	97	102	311	241	306	399	459	322	240	176	186	cluap1
ENSXETG00000010322	1290	139	184	170	103	76	170	51	63	45	121	113	110	ENSXETG00000010322
ENSXETG00000010323	2390	141	170	142	431	336	386	647	753	462	464	303	367	anks3
ENSXETG00000010324	3299	12	15	9	34	26	50	234	235	181	79	56	52	limk1
ENSXETG00000010325	984	1	0	0	8	2	2	16	21	12	3	0	1	SBK1
ENSXETG00000010326	3378	618	944	807	1547	1151	980	1238	1367	851	1066	771	804	xpo6
ENSXETG00000010327	1397	24	34	31	46	39	58	574	673	427	39	37	42	nme4
ENSXETG00000010329	1043	907	1277	1197	918	702	1134	469	546	327	353	216	250	decr2
ENSXETG00000010330	1831	987	1443	1253	1115	871	1941	1064	1198	756	778	559	639	rab11fip3
ENSXETG00000010331	3334	143	170	171	428	290	469	617	650	461	363	273	321	capn15
ENSXETG00000010332	1473	395	582	492	466	356	748	749	848	523	701	467	463	nsun5
ENSXETG00000010334	4388	440	612	555	1458	1178	1249	2415	2691	1682	1057	685	820	baz1b
ENSXETG00000010335	867	85	107	104	126	88	185	176	208	152	173	108	140	asb8
ENSXETG00000010336	4070	423	550	476	902	686	1028	1214	1450	852	958	651	730	pigq
ENSXETG00000010337	2877	270	417	379	909	677	1032	1113	1245	791	1458	1022	1167	ern2
ENSXETG00000010338	1764	6	7	7	60	39	119	147	152	112	127	106	116	fzd9
ENSXETG00000010339	2224	39	56	56	104	85	118	172	197	101	83	74	72	mks1
ENSXETG00000010343	1311	666	1058	876	816	625	1102	815	935	581	1191	811	931	wbscr22
ENSXETG00000010344	2146	139	226	163	624	462	660	931	1039	621	118	48	83	plk1
ENSXETG00000010345	1434	114	137	126	216	174	110	449	431	272	715	486	464	stx1a
ENSXETG00000010347	1575	8	16	15	299	270	189	907	1029	595	446	338	343	ras-dva
ENSXETG00000010348	891	342	503	370	365	314	614	835	860	527	485	339	431	dctn5
ENSXETG00000010351	1554	668	1037	890	163	133	156	397	430	256	287	184	202	ENSXETG00000010351
ENSXETG00000010353	3413	107	193	134	396	321	383	620	706	470	430	290	330	pom121
ENSXETG00000010354	1808	644	906	800	631	407	914	608	732	417	660	482	536	nubp2
ENSXETG00000010355	1659	108	165	120	286	202	404	449	489	332	414	259	341	spsb3
ENSXETG00000010357	1246	721	1001	907	1454	1085	1914	2801	3275	2201	3595	2492	2757	nme3
ENSXETG00000010358	3592	130	191	175	345	284	598	675	773	481	524	409	398	telo2
ENSXETG00000010360	2156	0	0	0	3	0	0	1	1	1	0	0	0	
ENSXETG00000010361	966	2929	4184	3758	2818	2134	2528	2645	3201	1898	1939	1470	1634	prdx4
ENSXETG00000010363	1376	19	40	23	395	302	149	246	316	175	119	82	78	tekt1
ENSXETG00000010365	975	0	0	0	0	0	0	1	0	0	0	0	0	
ENSXETG00000010368	918	0	1	2	3	4	22	16	20	13	11	10	8	adra1d
ENSXETG00000010370	1194	0	0	0	0	0	0	0	0	0	0	0	0	
ENSXETG00000010371	1003	0	0	0	0	0	0	0	0	0	0	0	0	
ENSXETG00000010373	2610	48	57	41	63	58	109	284	308	191	138	120	118	crim1
ENSXETG00000010376	3739	210	341	274	948	705	778	2795	3125	1940	1045	733	768	pkp4
ENSXETG00000010377	1560	4	4	2	34	10	11	103	115	59	18	16	20	ccdc148
ENSXETG00000010379	2431	95	145	149	345	224	412	761	929	527	1534	1109	1209	upp2
ENSXETG00000010380	3371	300	371	376	604	474	1024	2087	2341	1478	1132	813	875	acvr1
ENSXETG00000010381	1497	43	74	60	72	70	215	80	84	55	43	36	34	acvr1c
ENSXETG00000010382	1844	8	11	6	15	14	58	92	123	57	103	55	64	st6gal2
ENSXETG00000010384	1743	0	0	0	1	4	3	23	31	8	14	7	12	slc5a7
ENSXETG00000010389	4599	1	0	0	0	0	0	0	0	0	0	0	0	
ENSXETG00000010393	14004	6	10	7	9	8	19	1	2	1	8	7	1	ENSXETG00000010393
ENSXETG00000010394	606	1	0	0	0	3	4	36	45	22	13	7	5	ENSXETG00000010394
ENSXETG00000010396	2532	1	0	1	9	10	10	39	66	36	9	9	12	ano3
ENSXETG00000010397	4974	381	539	483	861	685	1537	1545	1887	1136	955	678	885	gcc2
ENSXETG00000010401	2319	0	1	0	7	8	17	172	186	116	14	15	19	pitx1
ENSXETG00000010403	1463	320	449	367	277	231	514	536	650	381	354	228	268	txndc15
ENSXETG00000010404	4115	269	417	330	773	623	1704	1230	1551	836	1762	1246	1465	c5orf24
ENSXETG00000010405	3175	934	1498	1344	2979	2347	3310	3449	3798	2384	3554	2641	2781	ddx46
ENSXETG00000010406	2322	366	512	410	394	275	523	692	864	495	352	231	262	camlg
ENSXETG00000010407	3216	2821	4125	3432	4229	3168	5961	5875	6632	3936	3518	2360	2626	sec24a
ENSXETG00000010408	2654	4962	7019	5776	2095	1545	2239	665	853	457	625	428	494	sar1b
ENSXETG00000010410	3452	77	107	110	384	323	615	412	467	301	1075	769	851	jade2
ENSXETG00000010413	1939	1232	1923	1589	1960	1392	2720	2227	2647	1496	2738	1965	2292	tpd52
ENSXETG00000010415	1947	177	256	191	503	368	709	855	1020	554	664	532	588	zbtb10
ENSXETG00000010417	1137	0	0	0	0	0	0	1	0	0	0	0	0	
ENSXETG00000010418	1983	56	95	78	115	76	195	974	1042	655	263	230	239	pag1
ENSXETG00000010419	405	31	26	37	10	3	14	8	15	18	10	18	8	ENSXETG00000010419
ENSXETG00000010420	637	483	716	673	2992	2281	2112	11619	13819	8314	2767	2009	2223	pmp2
ENSXETG00000010421	1178	658	911	817	788	578	721	1227	1416	840	399	306	314	impa1
ENSXETG00000010422	2159	15	35	16	365	283	12	593	710	426	69	48	61	XB-GENE-5993458
ENSXETG00000010424	984	204	268	224	180	175	266	242	251	162	280	207	216	zfand1
ENSXETG00000010425	717	400	503	459	1209	923	1899	598	743	434	1144	772	841	chmp4c
ENSXETG00000010427	1002	50	49	56	60	50	86	124	173	93	98	88	98	snx16
ENSXETG00000010432	936	1005	1410	1197	1459	1090	2510	681	739	445	869	611	691	tmem135
ENSXETG00000010434	1572	5	5	4	82	54	64	278	315	227	87	63	70	fzd4
ENSXETG00000010435	1697	21	26	29	183	147	169	1349	1553	973	339	234	295	prss23
ENSXETG00000010436	2935	2593	3873	3272	954	683	2152	2825	3197	1917	1130	790	881	me3
ENSXETG00000010437	2151	14	21	17	168	147	55	34	42	32	26	13	14	ccdc81
ENSXETG00000010438	4494	375	580	459	338	258	357	507	595	319	468	341	374	mettl13

ENSXETG00000010439	1046	85	137	119	64	28	109	389	464	292	145	105	121	hikeshi
ENSXETG00000010440	1907	341	481	421	485	404	578	939	1128	672	548	427	414	eed
ENSXETG00000010442	1083	1149	1634	1380	1405	1029	1362	2209	2454	1478	1595	1215	1263	clns1a
ENSXETG00000010444	1791	175	269	235	204	180	365	413	540	258	291	195	220	vamp4
ENSXETG00000010445	3111	118	194	133	538	390	783	1048	1190	690	859	561	601	rsf1
ENSXETG00000010446	1494	1	0	0	4	4	24	0	1	0	4	7	3	myoc
ENSXETG00000010447	2889	169	242	189	475	344	547	652	713	457	458	286	328	ints4
ENSXETG00000010448	8923	659	903	793	2139	1691	1951	3428	3723	2268	2764	1994	2328	prrc2c
ENSXETG00000010449	981	130	209	148	248	182	206	329	439	258	618	409	439	kctd14
ENSXETG00000010450	1657	256	426	343	504	400	627	506	585	389	874	638	689	alg8
ENSXETG00000010451	1632	0	0	0	3	0	0	0	0	1	16	7	6	usp35
ENSXETG00000010452	1968	8	18	8	65	64	173	315	333	214	155	113	139	gab2
ENSXETG00000010455	1590	63	100	84	79	53	84	123	130	80	84	56	62	nars2
ENSXETG00000010456	1865	0	0	0	0	0	1	1	2	0	1	0	1	fmo2
ENSXETG00000010457	2400	25	37	25	42	38	17	417	505	306	52	31	36	MGC89174
ENSXETG00000010459	1995	1414	2085	1745	3356	2533	3232	2579	3039	1881	1345	927	990	uap1
ENSXETG00000010460	5309	1025	1430	1170	1871	1461	2550	2846	3381	1879	1766	1183	1376	nos1ap
ENSXETG00000010461	1991	117	158	126	237	181	703	1476	1866	996	657	532	512	rgs4
ENSXETG00000010462	2900	67	98	68	299	198	1196	1687	2050	1076	1221	866	907	rgs5
ENSXETG00000010463	1610	55	100	75	240	193	197	333	387	229	78	44	51	nuf2
ENSXETG00000010464	1272	78	118	86	233	205	328	1046	1146	652	453	310	332	pbx1
ENSXETG00000010468	984	56	85	96	56	40	167	511	641	364	158	105	142	ENSXETG00000010468
ENSXETG00000010474	1744	485	787	614	354	299	358	704	771	445	171	137	140	c19orf12
ENSXETG00000010475	879	0	0	0	0	0	0	0	0	0	0	0	0	
ENSXETG00000010480	702	0	0	0	0	0	0	7	8	5	0	0	0	ENSXETG00000010480
ENSXETG00000010483	1560	0	0	0	0	0	1	0	0	0	0	1	1	
ENSXETG00000010491	3327	6	8	4	21	7	9	37	30	24	21	22	13	pcbp2
ENSXETG00000010494	1881	444	621	500	728	459	1088	924	1174	632	860	589	667	znf830
ENSXETG00000010495	2256	426	593	512	256	220	544	406	550	292	372	276	328	bag1
ENSXETG00000010497	1946	427	563	510	536	418	611	887	968	677	607	429	477	nudcd1
ENSXETG00000010499	1194	0	0	0	0	0	1	0	0	0	2	0	1	
ENSXETG00000010500	996	8	15	11	40	30	94	282	321	171	99	66	90	pdgfd
ENSXETG00000010505	846	0	1	3	36	24	29	150	168	96	134	93	100	phox2a
ENSXETG00000010507	2043	229	388	305	466	335	475	375	389	264	429	302	318	clpb
ENSXETG00000010509	975	42	65	45	88	67	154	298	385	204	67	47	73	ENSXETG00000010509
ENSXETG00000010510	1202	1291	1918	1625	1877	1436	3715	2357	2658	1626	2256	1601	1756	tnfrsf10b
ENSXETG00000010511	279	5	3	8	45	31	13	31	28	15	13	5	8	lrtomt
ENSXETG00000010512	2284	121	161	151	288	224	477	575	609	381	790	521	565	ENSXETG00000010512
ENSXETG00000010513	2085	315	403	359	733	542	475	57	70	32	465	354	363	rhobtb2
ENSXETG00000010515	3114	51	74	82	280	234	381	779	764	511	610	442	476	ENSXETG00000010515
ENSXETG00000010517	2937	489	732	627	1345	1077	2437	1514	1534	1056	2244	1519	1653	fam160a2
ENSXETG00000010518	762	92	149	125	269	187	534	502	551	299	513	380	418	bin3
ENSXETG00000010525	1545	0	0	0	0	1	0	0	1	1	2	0	0	cnga4
ENSXETG00000010526	1082	2	2	4	7	8	14	783	811	492	73	54	48	XB-GENE-5905274
ENSXETG00000010527	648	1	4	2	126	110	9	13	26	9	8	5	5	rsph1
ENSXETG00000010528	4478	3251	4945	4155	7764	5895	12457	5057	5698	3421	6201	4353	4852	ppp3cb
ENSXETG00000010530	729	185	235	204	420	329	328	7085	7957	5111	1212	837	895	c1qb
ENSXETG00000010532	4440	3291	4981	4147	8747	6548	13103	14226	16393	9628	7348	5335	5785	slc39a14
ENSXETG00000010533	2951	11	16	15	176	133	91	255	275	145	132	101	103	piwil2
ENSXETG00000010534	1777	812	1359	1135	897	652	852	1193	1332	825	1029	738	804	polr3d
ENSXETG00000010535	4677	3	2	1	3	2	4	19	27	10	8	4	5	phyhip
ENSXETG00000010536	3425	101	129	139	724	539	1210	12073	13459	8193	1163	787	968	bmp1
ENSXETG00000010537	3659	218	302	237	1193	929	1454	1529	1764	1067	938	703	748	reep4
ENSXETG00000010538	924	26	48	35	52	47	129	92	138	61	114	66	79	nudt18
ENSXETG00000010539	3368	539	828	755	1704	1263	1733	1662	1978	1163	1610	1108	1224	fam160b2
ENSXETG00000010540	1359	3	4	4	117	91	55	166	191	95	265	160	194	dmtn
ENSXETG00000010542	802	0	1	2	1	1	1	1	0	0	0	0	3	npm2
ENSXETG00000010543	1328	648	898	746	526	445	638	801	960	539	647	497	483	pbdc1
ENSXETG00000010544	3756	970	1437	1155	1681	1351	1871	2261	2531	1495	2366	1611	1900	xpo7
ENSXETG00000010545	1233	16	34	34	54	37	90	463	597	352	83	55	60	dok2
ENSXETG00000010546	1777	17	21	20	121	89	214	452	506	330	316	227	271	gfra2
ENSXETG00000010547	2274	0	0	0	0	0	0	0	0	0	0	0	0	
ENSXETG00000010549	2442	0	0	0	0	0	0	0	0	0	0	0	0	
ENSXETG00000010551	2586	0	0	0	0	0	0	0	0	0	0	0	0	
ENSXETG00000010553	912	0	0	0	0	1	1	0	0	0	0	0	0	
ENSXETG00000010554	2328	0	0	0	0	0	0	0	0	0	0	0	0	
ENSXETG00000010555	648	0	1	0	1	0	0	1	0	1	0	1	0	ubap2
ENSXETG00000010559	2481	0	0	0	0	0	0	0	0	0	0	0	0	
ENSXETG00000010561	11244	269	387	306	236	184	462	429	487	307	426	284	343	DMD
ENSXETG00000010566	2622	0	1	1	0	0	0	3	0	0	0	0	0	ndst4
ENSXETG00000010567	2091	332	461	385	583	421	968	886	1025	555	765	541	608	tab3
ENSXETG00000010570	4292	1529	2026	1770	2730	2133	4289	1453	1853	1044	2310	1662	1805	ugt8
ENSXETG00000010571	2556	0	0	0	0	0	0	0	0	0	0	0	0	
ENSXETG00000010572	2556	0	0	0	0	0	0	0	0	0	0	0	0	
ENSXETG00000010574	2559	0	0	0	0	0	0	0	0	0	0	0	0	
ENSXETG00000010576	1083	0	0	0	0	0	0	0	0	0	0	0	0	
ENSXETG00000010577	1932	0	0	0	0	0	0	0	0	0	0	0	0	
ENSXETG00000010578	2559	0	0	0	0	0	0	0	0	0	0	0	0	
ENSXETG00000010580	2469	0	0	0	0	0	0	0	0	0	0	0	0	
ENSXETG00000010581	2088	210	329	271	420	297	547	805	924	588	542	401	398	rnf8
ENSXETG00000010583	1868	5	16	10	15	25	59	153	168	98	92	56	62	crip3
ENSXETG00000010586	4250	183	274	223	302	225	453	585	624	415	518	304	368	abcc10
ENSXETG00000010587	930	1	0	2	3	3	1	2	1	0	10	9	16	dlk2
ENSXETG00000010588	1323	395	571	470	1199	916	1988	924	987	736	1354	964	929	tjap1
ENSXETG00000010593	3816	0	0	0	1	1	0	6	2	2	7	1	1	grin2d
ENSXETG00000010594	2873	326	444	419	633	460	940	852	918	576	1054	827	951	cnot4
ENSXETG00000010597	6215	667	1037	787	2458	1939	1909	2219	2472	1470	2031	1448	1550	nup205
ENSXETG00000010606	1235	1763	2388	2063	1717	1370	2047	2327	2912	1738	1230	832	1037	kdelr2
ENSXETG00000010608	1869	0	0	0	0	0	0	0	0	0	0	0	0	
ENSXETG00000010609	441	1	2	1	6	3	8	130	159	99	2	1	4	FAM180A
ENSXETG00000010610	357	2440	3658	3216	3730	2695	4222	3138	3223	2049	4732	3380	3559	mtpn
ENSXETG00000010618	1281	14	22	16	21	13	19	36	24	29	42	33	29	tmem143
ENSXETG00000010619	1432	115	179	131	890	752	2115	1633	1927	1141	3085	2177	2462	emp3
ENSXETG00000010621	1929	10	11	10	363	303	110	139	175	96	132	98	87	ccdc63
ENSXETG00000010622	1050	64	94	85	76	54	86	156	162	116	70	43	64	edc3
ENSXETG00000010623	1609	567	695	641	1079	800	1291	943	1112	581	963	705	781	clk3
ENSXETG00000010624	880	4	2	2	13	7	25	43	49	34	18	5	9	rspo3
ENSXETG00000010627	1776	238	363	307	364	297	518	782	961	569	404	291	338	rnf146
ENSXETG00000010628	1070	240	325	266	342	251	680	341	424	239	312	216	218	echdc1
ENSXETG00000010630	2610	4	15	13	25	19	24	169	196	106	71	57	55	soga3
ENSXETG00000010631	1686	8	10	7	5	2	17	41	59	33	40	24	34	themis
ENSXETG00000010633	3667	280	431	334	1277	1014	2152	1821	2213	1247	2014	1475	1686	ptprk
ENSXETG00000010642	1047	1610	2186	1867	1700	1352	1813	1966	2501	1396	1028	755	859	tm9sf3
ENSXETG00000010643	2376	226	348	272	371	263	722	527	672	364	785	580	614	pik3ap1
ENSXETG00000010644	1814	442	664	524	828	636	1461	1359	1652	975	1613	1111	1244	ppp3cc
ENSXETG00000010645	2593	963	1326	1135	4933	3911	5296	9275	11020	6464	8624	5981	6745	anxa7
ENSXETG00000010647	1434	42	74	50	177	133	158	99	108	89	141	100	127	arid3b
ENSXETG00000010648	4296	246	344	327	780	574	1148	1495	1612	984	1013	769	851	znf609
ENSXETG00000010649	2425	619	883	797	1130	851	1496	1128	1362	773	2800	1960	2145	trip4
ENSXETG00000010650	1497	140	216	204	257	193	382	521	512	313	272	219	259	csnk1g1
ENSXETG00000010651	1023	14816	21403	19135	16186	12050	18274	16208	18562	11003	8428	5936	6451	ppib

ENSXETG00000010652	1587	89	103	103	63	53	74	188	246	122	66	46	55	snx22
ENSXETG00000010653	1576	521	740	613	1105	790	1549	3319	3942	2229	1647	1335	1429	snx1
ENSXETG00000010654	1647	254	395	356	315	217	440	589	649	347	236	184	185	ENSXETG00000010654
ENSXETG00000010655	4640	5	10	9	13	8	4	18	20	8	271	203	202	col2a1
ENSXETG00000010656	440	28355	42204	36310	45494	34353	46999	43204	49679	27958	96145	67916	73287	rps17
ENSXETG00000010657	2309	301	416	355	317	284	690	594	803	418	525	362	452	tmem106c
ENSXETG00000010658	1263	247	365	347	390	287	848	128	159	99	448	335	388	vdr
ENSXETG00000010660	3168	14	19	8	9	16	33	165	192	96	34	31	34	cpeb1
ENSXETG00000010661	4259	356	533	441	2073	1663	2069	2416	2960	1666	3205	2297	2545	hdac7
ENSXETG00000010662	4522	8	17	7	42	37	67	418	471	302	68	54	45	ap3b2
ENSXETG00000010663	504	0	0	0	0	0	0	0	0	0	0	0	0	
ENSXETG00000010664	1041	0	0	0	0	0	0	0	0	0	0	0	0	
ENSXETG00000010666	1120	377	549	510	975	780	1971	1996	2255	1484	1224	881	878	oaz2
ENSXETG00000010669	2395	135	235	181	203	178	642	926	1114	718	384	259	321	scamp5
ENSXETG00000010670	642	23	35	33	56	44	71	91	102	66	59	52	48	ppcdc
ENSXETG00000010674	3210	3	11	9	14	8	6	72	108	49	18	17	17	atp8a2
ENSXETG00000010676	1241	844	1248	1121	1227	948	1457	2562	2748	1695	1394	1007	1073	maea
ENSXETG00000010677	2575	149	210	156	321	227	621	588	673	404	490	361	384	uvssa
ENSXETG00000010680	636	1	0	1	1	5	8	158	217	126	61	40	46	shisa2
ENSXETG00000010681	4811	301	408	390	831	594	1172	980	1039	655	1560	1066	1193	nup58
ENSXETG00000010682	1884	145	262	209	320	249	620	689	854	510	459	344	329	mtmr6
ENSXETG00000010684	549	1648	2373	2064	1875	1465	1529	1706	1822	1107	1480	1088	1134	dnajc19
ENSXETG00000010686	1311	3	6	9	25	16	28	204	221	129	31	17	34	selenon
ENSXETG00000010688	2569	32	53	40	196	115	216	304	359	188	191	149	162	fam188b
ENSXETG00000010690	1942	0	0	0	7	9	16	20	27	18	27	11	9	adcyap1r1
ENSXETG00000010691	444	69	112	93	110	83	136	163	189	113	125	98	91	mpg
ENSXETG00000010694	2154	1	1	2	13	9	12	9	7	5	18	7	16	ENSXETG00000010694
ENSXETG00000010700	1275	32	39	40	77	51	91	135	157	88	111	112	108	ENSXETG00000010700
ENSXETG00000010703	1503	3070	4366	4090	3678	2842	3692	3469	3768	2327	4381	3070	3395	pak1
ENSXETG00000010704	1644	242	357	275	206	141	835	132	173	103	196	182	151	ENSXETG00000010704
ENSXETG00000010706	6573	12	22	16	331	294	153	325	395	264	1089	772	870	myo7a
ENSXETG00000010707	1525	2309	3330	2804	3930	3068	7345	58277	65736	36441	5049	3447	3801	sparc
ENSXETG00000010712	2324	3133	4526	3842	3300	2459	2508	5661	6395	3945	3195	2340	2486	g3bp1
ENSXETG00000010713	684	0	0	0	4	0	1	1	0	0	0	0	0	ENSXETG00000010713
ENSXETG00000010716	1815	53	78	90	130	94	78	2357	2800	1645	116	79	109	ckap4
ENSXETG00000010717	3771	29	46	40	17	19	62	162	169	104	53	34	31	sash1
ENSXETG00000010718	1473	0	0	0	0	0	0	1	0	0	0	0	0	
ENSXETG00000010720	2703	56	70	65	164	120	115	299	302	191	142	94	93	brip1
ENSXETG00000010721	3756	153	194	197	323	259	362	570	749	460	348	266	253	ints2
ENSXETG00000010723	2103	1384	2008	1671	1081	823	2176	1113	1220	760	1770	1305	1438	p2ry2
ENSXETG00000010724	969	5	6	11	22	21	12	94	120	54	45	29	38	p2ry6
ENSXETG00000010725	1353	65	81	85	147	117	341	5804	6326	4087	543	405	412	serpinh1
ENSXETG00000010728	6528	241	371	264	692	520	938	1335	1544	893	1161	875	925	med13
ENSXETG00000010729	850	58845	88391	76341	77587	58454	84224	74444	82699	50183	176929	125299	134125	rps3
ENSXETG00000010731	1805	1023	1459	1210	1607	1236	1866	2573	3026	1802	1500	1100	1247	rps6kb1
ENSXETG00000010732	4832	1	2	3	3	2	0	1	1	0	0	1	0	ENSXETG00000010732
ENSXETG00000010734	2307	137	184	181	224	172	537	325	388	241	615	435	498	fchsd2
ENSXETG00000010735	1648	113	134	137	124	111	293	340	433	234	334	223	245	tubd1
ENSXETG00000010736	2373	2660	3612	3184	2328	1833	2240	2135	2638	1461	1622	1113	1305	vmp1
ENSXETG00000010737	714	388	636	498	284	226	298	402	450	268	213	142	171	ptrh2
ENSXETG00000010738	4854	3694	5454	4681	8772	6740	8679	8981	10625	6073	7178	5126	5762	cltc
ENSXETG00000010739	840	1	0	0	5	3	6	33	28	21	22	17	23	fam131b
ENSXETG00000010742	2595	0	0	0	0	0	0	0	0	0	0	0	0	
ENSXETG00000010758	722	308	380	335	396	271	736	585	727	387	377	297	316	glo1
ENSXETG00000010760	3136	133	178	168	279	192	394	542	607	382	391	268	297	btbd9
ENSXETG00000010762	1682	434	644	506	724	590	1348	1824	2026	1230	1292	915	1032	zfand3
ENSXETG00000010768	3264	230	322	242	207	156	735	943	1041	577	572	390	438	plekhf1
ENSXETG00000010769	357	2	0	1	5	1	1	11	16	14	13	3	9	efna3
ENSXETG00000010771	776	1204	1847	1541	1507	1212	1664	1441	1614	1057	925	639	694	tsta3
ENSXETG00000010773	933	177	212	233	294	242	212	358	372	220	140	99	96	dpcd
ENSXETG00000010777	2250	0	0	1	0	0	2	0	3	2	1	3	4	nxph1
ENSXETG00000010779	732	6	17	23	72	57	16	9	10	8	4	8	2	spata4
ENSXETG00000010781	974	7	5	6	196	149	22	14	14	10	15	10	15	pacrg
ENSXETG00000010782	657	0	1	0	2	6	8	60	75	36	20	12	15	gulp1
ENSXETG00000010783	4782	2578	3710	3386	9141	7099	15630	140458	156898	97638	12249	8792	9601	col3a1
ENSXETG00000010784	4428	270	417	347	1759	1360	2289	16674	18911	11463	2238	1580	1782	COL5A2
ENSXETG00000010785	639	1	1	0	0	0	0	2	2	0	0	0	0	
ENSXETG00000010788	744	138	179	174	459	355	699	535	598	397	752	559	641	mbp
ENSXETG00000010789	6274	44	80	84	236	173	381	416	484	270	262	207	237	znf236
ENSXETG00000010791	2198	126	190	144	193	147	248	372	435	244	268	188	221	socs6
ENSXETG00000010797	1209	60	74	69	183	134	249	336	395	257	291	224	217	znf665
ENSXETG00000010799	1575	0	0	0	0	0	0	1	4	0	0	0	0	ENSXETG00000010799
ENSXETG00000010800	887	703	991	859	7414	5616	4801	13421	15015	9283	8038	5561	6153	ENSXETG00000010800
ENSXETG00000010801	1422	0	1	0	0	2	6	38	47	23	8	9	7	ENSXETG00000010801
ENSXETG00000010805	1572	0	0	0	3	2	4	20	20	12	7	6	8	ENSXETG00000010805
ENSXETG00000010810	1404	0	1	1	12	4	30	71	57	46	9	11	17	fbln2
ENSXETG00000010811	541	12209	18469	16104	22770	16507	20899	20474	22812	14342	50263	35906	38695	rpl29
ENSXETG00000010813	1491	14	25	22	140	100	31	70	84	51	30	21	16	poc1a
ENSXETG00000010814	2220	6267	9173	7668	13358	10153	11915	6742	7535	4666	6977	4919	5531	alas1
ENSXETG00000010815	1238	592	872	835	627	436	922	1132	1292	717	766	553	646	pick1
ENSXETG00000010816	517	0	0	0	1	0	0	3	1	0	0	0	0	
ENSXETG00000010820	903	6	10	7	9	10	14	87	102	62	33	22	27	cd200r1
ENSXETG00000010821	1194	20	41	18	21	21	20	143	147	100	17	11	14	ENSXETG00000010821
ENSXETG00000010826	1288	46	70	69	116	86	75	511	504	340	160	116	104	ENSXETG00000010826
ENSXETG00000010827	840	11	7	8	14	14	10	58	61	39	36	14	29	ENSXETG00000010827
ENSXETG00000010829	1578	176	281	252	402	310	550	500	621	334	462	322	352	ildr1
ENSXETG00000010832	2487	0	0	1	11	7	19	31	63	19	43	30	41	ror1
ENSXETG00000010833	3285	1386	1952	1733	1176	837	2221	1058	1272	802	1034	678	745	pgm1
ENSXETG00000010835	1786	260	372	284	185	108	187	231	281	164	95	73	85	alg6
ENSXETG00000010836	1705	1	1	0	7	9	12	117	121	64	43	21	22	foxd3
ENSXETG00000010838	1898	271	370	311	396	330	857	674	987	574	486	372	452	atg4c
ENSXETG00000010839	6970	37	51	35	69	41	62	151	145	66	13	14	13	COL11A1
ENSXETG00000010840	3550	382	567	524	1864	1382	1659	2401	2687	1704	2313	1681	1767	sltm
ENSXETG00000010841	6466	273	368	341	819	576	1053	2333	2608	1583	1106	775	938	dock7
ENSXETG00000010842	3258	482	647	584	1216	960	1352	1246	1370	877	1313	1000	1123	rnf111
ENSXETG00000010843	4364	425	631	558	1230	912	1711	2844	3374	1945	1581	1115	1253	sema4d
ENSXETG00000010844	483	33	58	52	144	98	266	119	147	100	285	189	215	ENSXETG00000010844
ENSXETG00000010845	1359	2	10	3	2	7	2	5	9	4	10	3	7	ENSXETG00000010845
ENSXETG00000010846	1736	1	3	8	6	2	6	8	12	7	6	4	7	amy2b
ENSXETG00000010847	1696	111	157	148	757	590	996	8	4	3	2964	2104	2234	amy2a
ENSXETG00000010848	1380	52	76	75	126	89	387	648	727	438	206	146	171	fam63b
ENSXETG00000010849	2856	10	18	9	29	21	27	15	10	12	44	33	36	angptl3
ENSXETG00000010850	4296	1000	1403	1223	2924	2383	3796	5788	6722	4116	4200	2906	3411	adam10
ENSXETG00000010851	2877	8	10	13	50	35	116	265	355	221	73	48	57	kank4
ENSXETG00000010855	586	121	191	159	244	247	195	333	453	251	45	33	23	cks2
ENSXETG00000010861	885	11	8	4	13	8	17	144	161	91	17	10	17	aqp9
ENSXETG00000010865	3840	58	55	62	265	178	234	5125	6081	3381	560	412	458	aldh1a2
ENSXETG00000010866	1542	0	0	0	0	0	0	0	2	0	0	0	0	
ENSXETG00000010869	474	0	0	0	4	10	1	0	0	0	2	4	2	nxnl2
ENSXETG00000010870	1542	114	151	116	173	117	198	330	334	195	137	130	119	spin1
ENSXETG00000010874	3945	0	0	0	2	0	4	18	41	20	2	4	3	cntnap4

ENSXETG00000010875	3966	481	625	577	2091	1551	1977	800	869	513	2492	1703	1993	myzap
ENSXETG00000010876	2410	32	51	32	379	301	158	430	457	281	397	273	291	cgnl1
ENSXETG00000010878	715	65477	97905	83300	77971	59361	102376	86690	97741	60361	178886	124282	135762	rpl10
ENSXETG00000010879	612	40	51	42	488	350	61	179	217	134	46	38	44	rsph9
ENSXETG00000010880	1095	48	71	61	243	170	253	1192	1229	824	347	265	289	ptk7
ENSXETG00000010882	1578	827	1275	1048	584	440	600	710	793	433	631	512	456	polr1c
ENSXETG00000010883	3148	167	253	220	306	207	281	496	525	345	342	245	254	xpo5
ENSXETG00000010884	2475	63	112	86	100	74	93	51	86	36	75	55	76	ENSXETG00000010884
ENSXETG00000010888	1446	93	172	124	641	491	1086	1479	1595	1130	1506	982	1077	gtpbp2
ENSXETG00000010891	1305	0	0	0	44	37	6	0	2	2	1	7	2	tcte1
ENSXETG00000010892	1697	1583	2328	2042	1916	1391	2957	2593	2775	1790	1866	1309	1449	slc35b2
ENSXETG00000010896	2996	22654	33885	29152	76885	58696	96310	84191	92663	58564	140067	98653	106981	hsp90ab1
ENSXETG00000010898	7497	554	831	702	3483	2691	8514	50155	55764	33894	13363	9334	10150	fn1
ENSXETG00000010902	2031	601	879	757	1530	1067	2105	320	378	234	1489	1084	1214	mreg
ENSXETG00000010905	411	102	155	116	132	92	242	265	340	173	153	106	129	ube2f
ENSXETG00000010906	1822	1	1	1	10	2	12	64	62	35	38	30	36	ramp1
ENSXETG00000010908	1311	44	81	58	20	22	3	0	0	0	0	1	0	ENSXETG00000010908
ENSXETG00000010910	4122	16	21	19	109	95	215	494	525	312	229	177	176	sh3bp4
ENSXETG00000010911	630	7	14	7	1	3	1	7	4	3	3	3	3	ENSXETG00000010911
ENSXETG00000010912	597	0	0	0	0	0	0	0	0	0	0	0	0	ENSXETG00000010912
ENSXETG00000010915	582	3	11	8	35	25	51	372	440	262	62	36	42	arl4c
ENSXETG00000010916	1950	452	645	577	806	644	718	1121	1194	761	723	573	589	prpf31
ENSXETG00000010923	1147	733	994	886	157	114	263	22	47	20	161	133	107	ENSXETG00000010923
ENSXETG00000010924	2121	706	950	839	2481	1904	2582	1690	2086	1181	2723	1788	2212	slc44a4
ENSXETG00000010925	1945	0	2	2	0	2	5	23	24	20	9	4	2	XB-GENE-5794232
ENSXETG00000010927	1660	5	5	4	2	2	11	20	25	13	8	4	3	siglec10
ENSXETG00000010928	1755	350	448	380	726	513	782	1041	1190	695	933	680	780	ehmt2
ENSXETG00000010930	306	3	2	5	3	2	3	34	32	21	18	5	10	c2
ENSXETG00000010931	3207	356	501	467	705	516	1240	1196	1383	760	1030	741	829	nelfe
ENSXETG00000010932	4056	553	871	724	1057	826	1324	1260	1437	867	1172	747	903	skiv2l
ENSXETG00000010934	13596	137	247	189	940	686	906	2084	2510	1400	2270	1544	1753	lrp1
ENSXETG00000010939	2308	55	84	78	108	82	294	222	304	157	255	190	223	dhx40
ENSXETG00000010940	1260	0	6	4	19	14	9	162	191	120	12	11	18	bmp8
ENSXETG00000010942	1634	3	10	5	29	26	47	80	75	42	7	10	12	dct
ENSXETG00000010943	1302	78	131	99	389	275	562	580	767	467	705	558	523	rnf44
ENSXETG00000010944	906	308	424	368	469	356	372	941	1032	591	434	297	344	ppie
ENSXETG00000010945	2460	935	1362	1145	1073	834	795	1530	1667	1001	735	525	580	faf2
ENSXETG00000010946	1881	261	414	374	985	760	1761	1055	1213	649	2374	1702	1829	pabpc4
ENSXETG00000010948	1059	447	652	552	305	215	482	344	429	257	255	188	215	tgds
ENSXETG00000010949	1104	0	0	0	9	6	18	51	63	34	43	18	25	nt5c1b
ENSXETG00000010951	2856	173	189	204	188	142	445	1792	2306	1170	471	392	414	XB-GENE-5901010
ENSXETG00000010952	1663	99	167	124	156	109	316	219	282	184	606	404	447	trit1
ENSXETG00000010953	4969	296	389	351	230	194	141	384	493	272	195	150	150	gpr180
ENSXETG00000010955	816	2	2	3	162	114	7	1	3	1	123	96	81	sox21
ENSXETG00000010956	2038	120	168	138	205	161	122	406	480	273	231	167	217	mycl
ENSXETG00000010958	3912	392	508	454	630	452	1256	344	424	275	355	280	299	abcc4
ENSXETG00000010979	402	0	1	0	5	2	1	0	0	0	1	0	1	tctex1d4
ENSXETG00000010984	8136	3	1	3	1	0	2	5	7	9	2	4	1	tg
ENSXETG00000010985	834	17	32	28	49	38	160	574	696	373	203	127	108	sla
ENSXETG00000010986	606	3	2	2	6	7	2	0	0	0	6	3	2	crisp2-like
ENSXETG00000010987	1098	78	106	88	113	82	255	681	724	500	110	84	102	wisp1
ENSXETG00000010988	1183	2226	3212	2711	6490	4985	14119	6462	7336	4372	13649	9532	10538	ndrg1
ENSXETG00000010989	1008	13	22	17	66	44	134	529	584	341	66	47	40	st3gal1
ENSXETG00000010991	2913	124	177	151	272	217	540	412	503	239	580	410	472	ercc4
ENSXETG00000010993	3538	43	71	52	234	180	548	805	912	552	507	340	399	mkl2
ENSXETG00000010994	3155	451	597	495	511	398	670	913	1105	652	684	479	514	parn
ENSXETG00000010995	2576	3	3	0	1	2	6	0	0	1	8	4	4	ENSXETG00000010995
ENSXETG00000010996	930	0	0	0	0	0	0	0	0	0	0	0	0	
ENSXETG00000010997	447	0	0	0	0	0	0	0	0	0	0	0	0	
ENSXETG00000010999	1191	0	0	0	0	0	0	0	0	0	0	0	0	
ENSXETG00000011004	3085	833	1124	1014	817	657	616	1175	1408	765	713	500	601	wdr36
ENSXETG00000011007	921	0	0	0	0	0	0	0	0	0	0	0	0	
ENSXETG00000011009	924	0	0	0	0	0	0	0	0	0	0	0	0	
ENSXETG00000011010	1920	763	1005	910	680	498	1171	1539	1801	1102	868	623	694	fam192a
ENSXETG00000011033	1473	41	75	79	28	20	26	8	4	4	2	0	3	ENSXETG00000011033
ENSXETG00000011034	1932	428	665	542	1064	787	2709	1168	1345	837	1386	1006	1087	mtmr11
ENSXETG00000011035	2898	11	10	5	73	90	264	1125	1293	762	640	464	499	myocd
ENSXETG00000011036	1392	182	279	205	399	266	481	623	725	425	499	334	412	map2k4
ENSXETG00000011037	1225	443	597	478	640	454	912	1014	1139	625	923	630	679	adprm
ENSXETG00000011038	1601	989	1509	1237	1629	1199	1334	2112	2355	1490	1313	938	998	sf3b4
ENSXETG00000011046	1197	1	3	0	5	8	9	5	8	6	11	5	10	nr2f5
ENSXETG00000011047	1206	0	0	0	0	3	1	2	5	2	0	0	0	hfe2
ENSXETG00000011050	1095	352	483	451	1890	1443	4382	8913	9845	5821	10025	6945	7789	txnip
ENSXETG00000011053	730	176	263	250	249	175	251	415	463	266	224	157	211	polr3gl
ENSXETG00000011054	1611	0	0	0	1	0	2	0	5	1	2	0	0	
ENSXETG00000011057	1872	2	1	1	3	3	4	23	26	16	5	2	4	ENSXETG00000011057
ENSXETG00000011058	1014	34	49	29	68	46	98	138	173	84	158	115	132	znf684
ENSXETG00000011059	3558	56	75	89	236	164	878	731	793	512	559	365	422	spag9
ENSXETG00000011060	1947	877	1363	1084	1516	1113	4301	1051	1170	696	1770	1337	1507	tob1
ENSXETG00000011061	2239	421	577	497	800	565	1614	857	1032	622	837	531	600	ankrd40
ENSXETG00000011062	1346	619	971	844	968	767	1565	1578	1686	955	2698	1988	2206	luc7l3
ENSXETG00000011063	2955	5	5	4	4	7	44	31	42	26	34	19	18	znf850
ENSXETG00000011065	1233	0	6	2	17	13	8	6	2	2	20	18	14	LRRC74A
ENSXETG00000011067	3530	543	872	657	1685	1274	1944	3943	4463	2587	3294	2354	2519	irf2bpl
ENSXETG00000011071	1776	352	488	459	820	629	969	1019	1111	639	833	583	628	cipc
ENSXETG00000011072	6635	60	70	64	118	109	116	322	412	206	313	215	274	tmem63c
ENSXETG00000011079	986	2993	4470	3699	2238	1615	1908	393	435	252	500	311	394	gstz1
ENSXETG00000011080	1659	4	2	5	19	11	25	195	216	121	48	31	38	dtx3
ENSXETG00000011082	1404	42	52	55	59	56	125	259	322	194	117	69	90	tmed8
ENSXETG00000011083	1623	562	848	724	364	290	394	1056	1213	705	277	214	213	ahsa1
ENSXETG00000011084	1463	417	656	618	512	369	685	874	1020	568	549	359	423	vipas39
ENSXETG00000011085	3171	960	1416	1174	1244	951	2258	1893	2211	1296	1576	1115	1253	sptlc2
ENSXETG00000011086	1308	179	270	223	307	276	337	343	399	208	373	320	295	alkbh1
ENSXETG00000011087	2051	853	1216	1135	1830	1351	2319	2776	3119	1976	2268	1583	1758	snw1
ENSXETG00000011088	1871	64	96	94	146	102	130	237	273	169	67	42	56	adck1
ENSXETG00000011090	5285	0	0	0	1	1	5	35	44	29	3	2	0	nrxn3
ENSXETG00000011098	1953	872	1237	1082	1144	907	387	70	111	52	22	18	20	ENSXETG00000011098
ENSXETG00000011102	1725	1074	1487	1393	1305	1018	1493	509	683	377	861	620	654	ttc32
ENSXETG00000011104	1656	1024	1439	1193	1313	966	1915	598	755	431	2399	1699	2018	ENSXETG00000011104
ENSXETG00000011110	1394	3	1	1	0	0	1	16	6	5	2	0	3	hes3.3
ENSXETG00000011111	1847	205	291	215	69	67	349	263	285	177	185	114	167	hes3.1
ENSXETG00000011112	1803	0	0	2	1	0	3	1	3	2	3	2	1	ENSXETG00000011112
ENSXETG00000011113	2064	87	121	95	258	178	278	355	359	217	397	270	314	zbtb48
ENSXETG00000011114	1848	37	34	42	130	88	472	565	653	396	336	235	247	klhl21
ENSXETG00000011117	1488	286	349	354	1207	996	715	44	66	37	236	165	153	XB-GENE-5779150
ENSXETG00000011118	783	683	907	823	348	290	290	670	677	448	258	198	178	mrpl20
ENSXETG00000011119	3000	10	10	8	105	95	449	2492	3054	1693	1123	793	975	mxra8
ENSXETG00000011120	2130	328	505	402	998	774	1170	977	1098	633	542	418	428	dvl1
ENSXETG00000011123	1309	7	8	7	11	8	10	1	1	1	6	3	12	ENSXETG00000011123
ENSXETG00000011124	939	1573	2143	1881	1789	1367	4867	6183	7107	4134	3711	2555	2835	jun
ENSXETG00000011125	1056	12	19	8	7	1	24	92	94	48	40	30	46	ENSXETG00000011125

ENSXETG00000011126	2512	122	173	152	494	365	1255	477	621	367	1087	753	887	mysm1
ENSXETG00000011128	9637	4	4	2	11	5	34	19	21	11	18	17	10	oma1
ENSXETG00000011130	1680	249	366	309	423	327	735	235	262	178	576	383	422	dab1
ENSXETG00000011133	1987	2	5	5	12	4	2	6	6	2	5	4	5	c8b
ENSXETG00000011134	2189	0	0	0	0	0	3	0	1	0	9	2	0	
ENSXETG00000011135	1846	0	0	0	0	0	0	2	3	2	0	0	0	c8a
ENSXETG00000011136	1721	2054	2954	2479	1004	760	1005	566	706	429	254	184	182	cptp
ENSXETG00000011138	6467	89	126	104	86	64	121	74	96	58	137	93	105	prkaa2
ENSXETG00000011140	1269	44	55	35	418	317	526	388	429	237	417	269	337	XB-GENE-983543
ENSXETG00000011141	1799	137	212	188	213	152	430	661	748	443	502	340	362	phf13
ENSXETG00000011143	2952	597	972	792	706	545	641	995	1153	663	611	387	438	dnajc11
ENSXETG00000011144	2777	1357	1897	1519	4073	2976	6114	4594	5586	3006	4205	3088	3463	plpp3
ENSXETG00000011145	681	15	18	6	26	14	40	12	16	9	41	40	26	pusl1
ENSXETG00000011147	1451	0	0	0	0	0	0	0	1	0	0	1	0	
ENSXETG00000011148	937	0	0	0	0	0	0	0	0	2	0	0	0	
ENSXETG00000011150	982	0	0	0	0	0	0	1	0	0	0	0	0	
ENSXETG00000011152	1480	0	0	0	0	0	0	0	0	0	0	0	0	
ENSXETG00000011157	1182	0	1	1	3	1	0	1	10	4	15	12	11	znf585b
ENSXETG00000011158	5580	16	25	13	216	151	278	1614	1933	1073	518	400	436	pcdh18
ENSXETG00000011159	363	2	3	5	3	6	12	49	61	27	18	13	14	bank1
ENSXETG00000011160	1886	249	383	336	588	473	800	1501	1738	1022	787	569	650	ppp3ca
ENSXETG00000011164	1695	3324	4751	3976	5196	3963	6966	2961	3218	1905	2905	2048	2251	adh1c
ENSXETG00000011165	324	2781	4185	3618	4217	3205	3430	6992	7601	4940	3199	2314	2363	ENSXETG00000011165
ENSXETG00000011166	1428	0	8	7	9	4	12	42	58	24	46	34	31	ENSXETG00000011166
ENSXETG00000011167	523	1598	2280	1889	2462	1840	1962	4051	4805	2757	1671	1180	1311	h2afz
ENSXETG00000011168	1042	0	3	3	6	1	4	9	5	2	5	6	3	ENSXETG00000011168
ENSXETG00000011170	2118	39	51	48	182	145	201	894	911	673	294	188	192	ephb4
ENSXETG00000011171	2094	5	4	2	14	11	20	32	43	22	32	28	26	tfr2
ENSXETG00000011173	2594	0	1	0	2	1	0	1	3	0	1	0	0	wnt10b
ENSXETG00000011174	546	47	64	45	117	102	143	432	462	233	133	84	90	arf3
ENSXETG00000011175	1840	1706	2392	2028	1734	1360	2401	2164	2675	1531	1087	797	891	prkag2
ENSXETG00000011176	13368	134	210	161	518	375	629	1146	1317	800	1600	1160	1351	kmt2d
ENSXETG00000011177	1119	29	27	32	52	43	69	85	98	43	66	38	53	ENSXETG00000011177
ENSXETG00000011179	1146	522	748	673	1951	1533	3293	205	216	144	189	123	139	ptger3
ENSXETG00000011180	2013	3	4	0	4	4	0	4	8	3	1	2	1	rhebl1
ENSXETG00000011181	4362	520	739	631	1837	1383	1042	381	447	262	1005	694	785	dhh
ENSXETG00000011183	1449	2893	4303	3573	2870	2172	4898	1401	1618	874	2216	1565	1812	cth
ENSXETG00000011184	1581	266	389	368	624	491	588	628	716	447	292	221	246	lmbr1l
ENSXETG00000011185	1617	606	898	767	556	414	942	8585	9634	5751	843	578	664	tuba4a
ENSXETG00000011186	1529	290	396	352	532	387	831	7199	8015	4882	1663	1152	1198	tuba1b
ENSXETG00000011187	2267	9227	13564	11676	9133	6910	5723	27331	31123	18917	5258	3688	4105	tuba1c
ENSXETG00000011188	2247	21	18	14	107	75	318	1706	1989	1238	364	239	292	prph
ENSXETG00000011189	4266	5	8	3	49	39	138	1269	1348	892	201	166	160	gli2
ENSXETG00000011191	735	0	0	0	1	1	0	2	1	4	0	0	0	c1ql4
ENSXETG00000011192	1451	966	1437	1250	1146	904	1436	1030	1228	760	1457	982	1055	dnajc22
ENSXETG00000011196	3007	1	0	1	16	12	28	81	117	58	62	51	44	tmem59l
ENSXETG00000011197	957	336	519	475	1110	800	1363	1803	1936	1317	1229	908	1032	ENSXETG00000011197
ENSXETG00000011199	2979	50	86	67	132	101	144	287	288	161	193	140	172	crtc1
ENSXETG00000011201	2070	4	5	1	19	18	17	29	29	16	2	2	4	cat1
ENSXETG00000011202	2280	0	0	0	3	0	0	1	3	2	3	1	1	comp
ENSXETG00000011203	840	40	79	46	121	78	154	173	211	133	266	193	220	ENSXETG00000011203
ENSXETG00000011204	1527	354	516	385	486	385	445	741	845	445	439	296	310	katnbl1
ENSXETG00000011206	369	448	706	592	558	380	844	820	905	583	602	459	456	emc7
ENSXETG00000011210	987	0	0	0	0	0	0	0	0	0	0	0	0	
ENSXETG00000011217	5410	2388	3516	3054	9082	6985	12040	22302	25470	15174	10390	7432	7994	thbs1
ENSXETG00000011220	2201	175	250	204	518	438	853	882	1036	683	814	520	655	mpp5
ENSXETG00000011221	1094	1610	2309	2065	2185	1637	2176	3940	4411	2616	2744	1938	2187	eif2s1
ENSXETG00000011222	1070	1097	1690	1407	1581	1265	3889	2739	3039	1829	2645	1922	1962	atp6v1d
ENSXETG00000011223	3797	203	318	269	686	511	1175	690	805	490	865	591	645	gphn
ENSXETG00000011225	1794	307	392	319	475	403	555	816	936	557	545	353	422	zdhhc14
ENSXETG00000011226	4017	23	37	34	136	97	264	688	822	508	375	248	290	arid1b
ENSXETG00000011228	1713	409	576	417	263	180	217	410	461	282	136	105	100	tk2
ENSXETG00000011229	1284	346	569	456	426	331	814	1108	1272	727	703	490	546	parp11
ENSXETG00000011231	1006	21	32	24	40	24	65	406	520	260	99	73	78	cmtm3
ENSXETG00000011232	882	36	52	42	103	82	132	118	124	78	263	185	229	cracr2a
ENSXETG00000011233	624	22	23	19	42	29	80	254	252	156	98	75	82	cmtm4
ENSXETG00000011234	1644	103	167	107	303	238	670	851	940	556	560	380	475	MGAT4B
ENSXETG00000011235	1080	117	166	140	162	138	127	292	287	206	114	81	78	ccdc94
ENSXETG00000011236	1476	177	247	218	378	283	524	1066	1308	777	459	326	362	dync1li2
ENSXETG00000011243	3102	47	101	90	336	268	459	770	862	553	589	430	438	maml1
ENSXETG00000011245	1889	467	706	597	755	565	1023	1323	1623	997	893	615	676	nae1
ENSXETG00000011251	1313	26	37	41	127	85	131	154	203	108	172	112	131	rnf166
ENSXETG00000011252	1876	557	754	646	457	348	392	747	909	499	339	253	293	ctu2
ENSXETG00000011253	3005	70	142	94	321	208	407	491	590	330	614	468	507	piezo1
ENSXETG00000011255	2505	271	463	336	695	507	396	557	578	394	504	342	349	cdt1
ENSXETG00000011256	1563	337	487	385	458	381	2060	704	890	528	703	490	515	galns
ENSXETG00000011257	2145	35	42	36	36	25	36	141	137	109	189	135	139	slco4a1
ENSXETG00000011258	1359	0	0	0	0	0	2	0	1	0	0	1	2	pabpn1l
ENSXETG00000011259	1752	23	18	19	64	40	119	167	187	104	175	126	118	cbfa2t3
ENSXETG00000011260	1461	81	124	101	250	181	248	355	367	235	356	235	258	ENSXETG00000011260
ENSXETG00000011261	2499	275	447	360	489	341	609	455	517	282	528	331	442	acsf3
ENSXETG00000011262	411	0	3	0	27	21	2	51	49	35	1	3	4	c20orf85
ENSXETG00000011263	810	16	25	22	41	27	132	1417	1499	1033	184	118	166	pmepa1
ENSXETG00000011265	2016	0	0	0	0	0	0	1	0	0	0	0	0	
ENSXETG00000011266	2702	14015	21327	18522	64880	48415	48649	50	66	37	25130	18036	19199	pck1
ENSXETG00000011268	1262	15	25	26	224	177	218	723	780	512	285	231	226	rbm38
ENSXETG00000011269	2358	10	22	14	69	50	64	76	98	53	79	49	48	cdh15
ENSXETG00000011270	1329	443	669	541	914	688	966	1507	1564	998	992	747	795	rae1
ENSXETG00000011271	894	0	1	1	1	0	2	9	9	12	2	0	1	spo11
ENSXETG00000011272	2011	24	30	41	126	106	155	2439	2654	1628	294	220	266	bmp7.1
ENSXETG00000011273	510	0	0	0	0	0	0	0	0	0	0	0	0	
ENSXETG00000011276	1308	42	45	35	198	174	139	106	120	77	357	254	287	slc22a31
ENSXETG00000011277	7611	440	600	528	1093	857	1924	2181	2617	1456	1871	1256	1520	ANKRD11
ENSXETG00000011290	606	23	35	31	117	89	206	495	589	374	252	188	198	rbpms
ENSXETG00000011291	864	387	562	479	483	329	528	838	969	591	316	231	301	gtf2e2
ENSXETG00000011292	966	1580	2345	2052	1964	1531	2571	1819	2096	1275	2144	1639	1723	saraf
ENSXETG00000011293	1479	1173	1724	1470	1375	1049	824	1753	1922	1129	643	417	530	gsr
ENSXETG00000011294	3989	8	13	9	22	20	39	309	358	209	305	196	267	trim36
ENSXETG00000011296	1354	245	402	325	332	251	835	800	966	529	391	289	272	pggt1b
ENSXETG00000011297	1599	8	15	11	50	30	63	132	133	69	82	45	51	ccdc112
ENSXETG00000011308	1233	1987	2980	2453	2907	2102	3408	3080	3511	2041	3428	2371	2513	glrx3
ENSXETG00000011309	2001	0	0	0	1	6	6	16	16	16	22	4	12	ebf3
ENSXETG00000011313	2034	1	0	0	4	1	1	5	6	0	6	3	6	ENSXETG00000011313
ENSXETG00000011314	1719	0	0	0	0	0	0	1	0	0	1	1	0	ENSXETG00000011314
ENSXETG00000011315	2403	9	8	7	58	56	78	183	204	121	264	226	200	adgrf1
ENSXETG00000011316	864	0	0	0	0	0	0	0	0	0	0	0	0	
ENSXETG00000011317	1949	48	61	53	247	193	292	1216	1325	838	428	290	323	tnfrsf21
ENSXETG00000011319	2694	332	514	375	755	579	1269	1382	1772	985	732	472	548	rab20
ENSXETG00000011321	1437	0	1	0	1	4	2	14	12	13	10	4	11	adgrf2
ENSXETG00000011322	957	0	0	0	0	1	0	18	21	6	6	4	3	opn5
ENSXETG00000011324	2718	0	0	0	0	1	0	0	0	0	1	1	1	ptchd4

ENSXETG00000011329	4822	2	1	2	15	7	47	49	60	30	20	14	20 thsd7a
ENSXETG00000011330	4639	535	872	616	1105	869	2141	1181	1413	823	1487	1109	1263 tmem106b
ENSXETG00000011332	4707	8	15	10	65	53	166	114	162	113	83	44	54 vwde
ENSXETG00000011333	2145	811	1123	1029	4782	3520	5202	3909	4559	2550	3780	2595	2904 scin
ENSXETG00000011338	594	14	26	16	17	13	27	41	61	39	32	25	19 dusp14
ENSXETG00000011339	2918	3	4	3	26	26	26	92	82	57	55	28	34 sez6l2
ENSXETG00000011340	556	32591	49141	42021	49268	36189	49701	42656	47181	28987	71070	50840	54274 nme2
ENSXETG00000011341	3273	283	380	400	783	617	956	1427	1583	935	1022	809	885 taok2
ENSXETG00000011342	2773	357	476	385	772	646	2172	1832	2156	1275	1374	931	1053 arl4a
ENSXETG00000011349	1119	376	540	496	641	524	969	867	995	649	724	486	527 nlk.2
ENSXETG00000011369	957	786	1158	986	827	643	711	2243	2577	1545	324	234	229 pycard
ENSXETG00000011370	1648	346	492	433	863	661	1251	2642	3007	1822	1151	813	896 XB-GENE-5809894
ENSXETG00000011371	3313	397	602	456	578	432	947	1172	1288	726	577	422	522 bicdl2
ENSXETG00000011373	1017	110	138	120	207	173	314	580	535	320	291	208	233 thoc6
ENSXETG00000011374	7352	1028	1400	1198	3159	2439	3581	3952	4485	2750	4772	3504	3854 srrm2
ENSXETG00000011375	1587	13	13	16	10	4	17	94	97	72	29	18	26 mmp25
ENSXETG00000011378	5961	68	98	92	609	494	874	1566	1896	1116	862	630	670 ptprb
ENSXETG00000011382	1052	6	7	6	21	8	39	70	107	48	65	47	46 kcnmb4
ENSXETG00000011383	1128	247	299	280	279	204	731	715	867	484	868	614	656 iyd
ENSXETG00000011385	1842	433	703	607	806	672	1166	1549	1743	1029	1171	756	920 cnot2
ENSXETG00000011386	2619	2015	2805	2562	5510	4128	7524	8481	9694	6008	7655	5336	6070 hnrnpu
ENSXETG00000011388	2718	723	1045	949	1674	1342	3021	1069	1259	666	2295	1587	1895 myrfl
ENSXETG00000011389	2083	59	86	79	186	139	72	406	428	268	34	30	28 rad51ap1
ENSXETG00000011390	3482	16	34	34	108	92	265	1219	1479	781	422	331	342 cox20
ENSXETG00000011392	2734	485	691	553	942	756	1552	1624	1937	1132	1288	992	1116 spast
ENSXETG00000011394	2428	93	121	126	238	155	334	410	534	296	303	198	199 taf5
ENSXETG00000011395	1056	1	4	4	9	4	3	80	85	42	20	14	20 calhm2
ENSXETG00000011397	5703	191	277	250	396	280	605	641	675	439	515	369	417 neurl1
ENSXETG00000011399	3183	33	60	37	86	72	315	756	867	535	362	243	322 sh3pxd2a
ENSXETG00000011400	3105	2	6	3	39	25	64	176	191	118	132	93	114 casr
ENSXETG00000011402	1765	63	81	71	77	63	116	147	232	92	103	63	79 stn1
ENSXETG00000011407	895	32	72	72	200	136	378	124	154	86	481	348	368 sfr1
ENSXETG00000011409	4287	0	1	3	6	2	8	5	1	3	4	1	4 actn3
ENSXETG00000011411	2787	340	412	404	596	425	992	1316	1603	949	699	506	541 exoc2
ENSXETG00000011413	1353	5	2	4	1	0	5	44	36	18	10	9	9 irf4
ENSXETG00000011414	2113	268	394	308	379	293	683	808	1032	563	472	363	427 dusp22
ENSXETG00000011415	2010	500	800	672	824	592	1053	1536	1851	1076	909	620	712 ripk1
ENSXETG00000011416	2988	556	847	729	1442	1160	1686	1766	2111	1288	2379	1725	1924 prpf4b
ENSXETG00000011423	972	0	0	0	0	0	0	0	0	0	0	0	0
ENSXETG00000011424	751	617	905	777	938	726	1151	501	651	294	716	506	540 ccs
ENSXETG00000011427	2406	50	97	66	203	139	270	969	1282	716	329	245	299 prkd3
ENSXETG00000011428	1132	483	695	573	550	466	918	633	753	441	438	321	400 zdhhc24
ENSXETG00000011429	2319	1088	1564	1370	2669	2076	2813	3549	3954	2391	2555	1809	2019 sart1
ENSXETG00000011430	1096	1241	1743	1608	1486	1215	761	2928	3356	1860	896	661	652 banf1
ENSXETG00000011431	910	335	461	372	352	246	309	577	659	420	390	289	302 eif1ad
ENSXETG00000011434	2820	90	147	113	148	87	265	311	331	203	249	194	223 bcl11b
ENSXETG00000011436	2143	516	754	661	542	396	737	1107	1292	769	547	391	426 setd3
ENSXETG00000011438	1955	736	997	813	967	752	2040	1956	2021	1214	1612	1157	1304 ccnk
ENSXETG00000011440	2642	515	710	638	1804	1381	3732	1162	1405	748	1723	1183	1346 XB-GENE-969052
ENSXETG00000011442	3780	2297	3366	2728	5602	4427	6227	1960	2272	1394	3529	2450	2857 mia2
ENSXETG00000011443	3792	150	251	241	663	487	771	579	677	355	667	508	558 fbxo33
ENSXETG00000011444	642	16	24	24	46	37	37	360	379	224	66	41	47 ppic
ENSXETG00000011446	5694	4	3	3	11	8	22	38	43	17	44	33	38 plxna4
ENSXETG00000011448	2186	81	105	68	207	166	142	219	274	163	111	70	95 ENSXETG00000011448
ENSXETG00000011452	2369	106	131	140	354	231	924	662	759	492	691	500	599 exoc4
ENSXETG00000011453	1533	1	0	1	1	0	0	11	8	0	0	0	1 krt2l
ENSXETG00000011456	1485	0	0	0	0	0	0	0	0	0	0	0	0
ENSXETG00000011460	3522	9	8	4	1	2	4	22	34	18	8	2	6 ADGRD2
ENSXETG00000011461	1989	0	0	0	0	0	0	0	0	0	1	0	0
ENSXETG00000011463	834	1613	2445	2073	2460	1812	3061	4857	5482	3186	1086	753	843 psmb7
ENSXETG00000011464	1966	213	262	207	529	443	750	1276	1519	813	593	415	464 nek6
ENSXETG00000011465	1665	2151	3147	2642	1059	800	1332	856	1016	572	2261	1588	1794 zc3h12a
ENSXETG00000011467	2094	0	1	0	0	1	0	0	0	0	2	0	0 rph3a
ENSXETG00000011472	1978	0	0	0	2	0	0	1	3	1	5	0	1 wscd2
ENSXETG00000011473	1398	175	217	180	500	412	640	1612	1811	1107	782	537	653 meis1
ENSXETG00000011474	1591	19	12	14	32	26	49	450	566	336	45	41	58 cmklr1
ENSXETG00000011475	1299	34	54	33	36	43	106	83	106	58	155	114	123 rnft2
ENSXETG00000011477	1947	40	52	45	101	82	201	251	352	169	324	204	238 lrch1
ENSXETG00000011481	6958	765	1185	982	2749	2138	1693	4914	5727	3415	2188	1576	1698 col18a1
ENSXETG00000011482	1617	132	188	138	77	86	114	150	147	108	114	67	95 slc19a1
ENSXETG00000011483	1020	0	0	0	0	1	0	5	1	3	0	0	1
ENSXETG00000011486	3138	223	299	260	2133	1609	4052	27619	31253	19300	6662	4600	5192 col6a1
ENSXETG00000011488	741	12877	17300	13723	14800	12419	23140	29450	32526	23743	20051	13241	16130 ppdpf
ENSXETG00000011490	3817	453	708	613	2406	1849	4013	40171	45839	27669	5973	4291	4654 col6a2
ENSXETG00000011493	1092	166	234	193	1020	792	1342	1194	1290	858	1413	994	1079 pcmtd2
ENSXETG00000011494	2696	12	13	17	372	296	219	110	107	67	242	182	210 ftcd
ENSXETG00000011496	5898	308	429	362	857	686	964	1041	1195	687	1340	969	1070 mcm3ap
ENSXETG00000011499	3261	2	5	9	54	34	42	143	153	87	62	61	63 myt1
ENSXETG00000011501	543	268	379	303	237	162	155	369	413	224	240	165	188 rpp25l
ENSXETG00000011506	1014	2	5	8	219	170	82	124	150	72	122	101	97 C21orf58
ENSXETG00000011507	1052	70	112	85	172	161	190	302	271	217	202	126	148 ppp1r8
ENSXETG00000011508	2228	5	9	8	18	8	14	53	76	34	13	11	11 manea
ENSXETG00000011509	794	209	294	271	140	99	221	301	379	186	159	121	135 utp11
ENSXETG00000011510	2510	0	0	0	0	0	4	1	1	1	0	0	0
ENSXETG00000011511	2136	6	15	18	162	92	177	420	584	275	224	170	182 ENSXETG00000011511
ENSXETG00000011512	3051	188	271	247	461	374	787	878	1015	630	768	560	616 stard8
ENSXETG00000011514	645	2	4	3	70	41	52	254	285	180	134	86	102 timp3
ENSXETG00000011515	1725	732	1114	945	1054	854	1161	1585	1710	1072	1695	1145	1306 nop2
ENSXETG00000011516	3261	10	9	7	55	39	65	135	172	100	45	29	44 grip2
ENSXETG00000011517	1737	0	1	0	5	5	5	12	12	12	12	7	9 SYN3
ENSXETG00000011518	1842	41	51	35	42	32	98	1509	1736	1124	241	173	170 slc6a6
ENSXETG00000011520	1966	348	518	447	576	455	695	1151	1302	830	616	451	528 fbxo7
ENSXETG00000011522	684	9	13	4	18	19	17	10	18	14	44	17	34 eda2r
ENSXETG00000011523	2411	3028	4291	3723	4549	3380	5143	5027	5526	3491	3201	2280	2375 rtcb
ENSXETG00000011524	990	0	0	0	0	0	2	1	3	1	1	0	1
ENSXETG00000011525	1059	0	0	0	0	0	0	0	0	0	0	0	0
ENSXETG00000011528	3828	120	178	161	740	596	611	992	1135	743	433	314	330 ncapd2
ENSXETG00000011529	2460	52	80	89	92	69	162	234	241	146	200	136	159 prdm4
ENSXETG00000011530	1105	3178	4840	3998	9675	7321	9065	5355	5865	3866	4078	2919	3097 akr1b1
ENSXETG00000011532	1560	206	298	261	341	287	557	803	960	539	770	543	561 znf384
ENSXETG00000011533	984	1056	1534	1269	1558	1206	845	994	1172	654	800	559	599 akr1c8p
ENSXETG00000011534	669	470	709	573	743	543	650	1067	1248	719	627	482	547 lsm3
ENSXETG00000011538	1697	1642	2351	2163	2627	2132	3424	4531	4832	3149	2128	1435	1609 mlf2
ENSXETG00000011540	3231	135	206	197	191	145	182	1064	1233	675	225	165	190 tmem43
ENSXETG00000011541	2076	9	10	15	85	65	142	584	683	449	340	207	279 p3h3
ENSXETG00000011542	1833	6	11	10	7	10	3	31	21	22	94	71	80 NTN4
ENSXETG00000011543	2778	58	96	82	417	357	254	6	6	6	196	135	141 uroc1
ENSXETG00000011544	2417	6	16	17	55	31	33	245	292	163	147	79	108 ENSXETG00000011544
ENSXETG00000011546	921	0	0	1	0	3	2	3	2	4	2	1	2 oprl1
ENSXETG00000011547	2046	186	301	234	438	351	636	624	770	425	723	508	565 zxdc
ENSXETG00000011548	1707	0	0	0	62	53	7	0	0	0	6	3	3 cfap100

ENSXETG00000011549	1185	42	49	52	335	298	568	43	39	30	903	672	700	klf15
ENSXETG00000011551	2523	948	1372	1166	2958	2325	4477	321	331	207	5298	3642	4001	aldh1l1
ENSXETG00000011552	1154	0	5	3	0	2	0	71	112	64	13	11	12	XB-GENE-5836417
ENSXETG00000011556	556	12	15	13	6	8	28	2	1	2	1	0	0	XB-GENE-5745824
ENSXETG00000011560	6689	3	4	4	28	19	48	167	224	129	70	64	106	foxp2
ENSXETG00000011562	1374	667	953	849	859	663	1025	1605	1741	1062	1148	794	874	luc7l
ENSXETG00000011563	3860	1434	2080	1612	1147	891	2186	1797	2334	1228	1025	759	837	fam234a
ENSXETG00000011564	1241	1246	1809	1481	1238	931	1085	787	942	499	649	478	465	mrpl28
ENSXETG00000011565	741	22	31	23	55	48	129	251	297	168	58	36	40	mdfic
ENSXETG00000011566	1104	14	8	11	24	20	24	414	436	276	48	24	31	tfec
ENSXETG00000011567	2931	157	216	160	390	304	500	964	1121	671	761	526	566	trim24
ENSXETG00000011568	2694	603	903	709	642	516	881	3199	3889	2297	1130	757	907	tes
ENSXETG00000011569	1206	0	0	0	0	1	0	0	0	0	0	0	0	svopl
ENSXETG00000011570	1696	300	497	411	797	618	1124	3572	4318	2470	1573	1059	1183	cav2
ENSXETG00000011571	2693	0	0	0	0	0	1	3	1	5	9	6	7	atp6v0a4
ENSXETG00000011572	2156	562	751	648	1976	1475	2928	9645	12087	6945	3788	2835	3140	cav1
ENSXETG00000011574	4011	3	3	1	25	18	29	505	495	296	28	30	32	kiaa1549
ENSXETG00000011576	1672	2821	4049	3239	2613	1862	3505	3131	3635	1908	3023	2084	2308	llph
ENSXETG00000011586	750	579	784	691	454	429	852	873	935	532	609	478	498	rab21
ENSXETG00000011587	1169	105	140	127	113	85	260	214	249	148	116	100	141	tmem19
ENSXETG00000011592	3233	668	899	797	1096	858	1306	1519	1904	1067	1195	872	959	fam172a
ENSXETG00000011594	2353	11	14	12	72	57	83	357	401	210	45	34	56	nr2f1
ENSXETG00000011595	3000	0	2	1	5	5	20	38	64	42	30	27	24	dlgap2
ENSXETG00000011600	1032	1	2	0	8	8	16	59	65	42	12	12	6	crmp1
ENSXETG00000011601	1836	123	126	158	316	245	389	262	294	168	266	174	198	jakmip1
ENSXETG00000011602	942	0	0	0	0	0	0	0	1	0	0	0	0	
ENSXETG00000011603	3582	220	302	274	631	511	590	1916	2182	1313	1040	702	685	wfs1
ENSXETG00000011604	1311	2	2	0	8	5	8	24	24	19	9	13	20	ppp2r2c
ENSXETG00000011608	4038	54	88	70	229	184	217	373	429	276	108	89	91	arhgef10
ENSXETG00000011609	4958	1096	1443	1272	1636	1260	4126	1763	1964	1171	1670	1225	1328	man2b2
ENSXETG00000011610	1935	1	0	2	4	5	1	8	15	13	15	16	16	ablim2
ENSXETG00000011611	2562	5	8	5	37	22	50	419	423	287	85	44	68	afap1
ENSXETG00000011612	363	296	445	363	501	384	463	121	139	80	293	247	237	ENSXETG00000011612
ENSXETG00000011613	1261	353	541	538	2089	1561	1305	1482	1646	1088	6085	4359	4979	slc25a39
ENSXETG00000011614	2839	3268	4846	4281	7963	5965	20968	14516	15956	9556	12123	8707	9160	grn
ENSXETG00000011615	3782	35	44	38	131	109	186	1781	2039	1236	322	211	200	fam171a2
ENSXETG00000011617	3105	14	25	12	116	66	43	188	179	108	42	30	35	itga2b.1
ENSXETG00000011619	1554	50	59	64	191	153	242	434	449	328	300	183	199	ppp1r16a
ENSXETG00000011622	2088	56	111	88	348	279	442	418	500	303	247	157	151	raver1
ENSXETG00000011623	3567	362	569	474	1784	1439	2339	2010	2046	1274	2013	1393	1553	tyk2
ENSXETG00000011624	2739	28	53	47	81	52	111	201	215	119	156	91	113	ENSXETG00000011624
ENSXETG00000011625	1895	1432	2144	1824	2025	1587	2314	4346	5023	3007	1820	1274	1416	cdc37
ENSXETG00000011632	398	0	0	0	0	0	0	0	0	0	0	0	0	
ENSXETG00000011637	2052	9	8	2	26	17	47	221	269	171	88	83	72	pde4a
ENSXETG00000011638	1965	1974	2916	2432	1639	1222	2458	3521	3800	2626	1042	749	779	keap1
ENSXETG00000011640	1152	11	9	10	55	30	89	312	295	197	105	86	94	s1pr5
ENSXETG00000011644	2929	230	330	290	472	370	426	937	1051	669	584	395	475	atg4d
ENSXETG00000011646	2259	376	565	469	811	590	1103	766	858	548	1542	1126	1176	kri1
ENSXETG00000011648	393	0	0	0	0	0	0	26	23	17	1	0	0	
ENSXETG00000011649	486	14	14	13	58	62	97	132	144	100	98	64	71	cdkn2d
ENSXETG00000011650	2121	39	55	54	374	305	507	1511	1678	1019	830	575	613	slc44a2
ENSXETG00000011651	2832	3549	5102	4362	8206	6356	10531	12800	14212	8737	13103	9103	10020	ilf3
ENSXETG00000011652	993	90	146	104	204	160	280	512	607	360	307	197	233	ENSXETG00000011652
ENSXETG00000011653	2331	54	74	52	134	99	189	210	266	176	236	139	189	znf438
ENSXETG00000011655	3604	39	43	34	223	147	521	1596	1741	1087	796	586	607	zeb1
ENSXETG00000011657	2484	229	332	316	781	583	1061	2237	2658	1435	1412	957	1093	arhgap12
ENSXETG00000011658	3103	842	1239	1125	1589	1226	2566	5063	5852	3499	2038	1490	1701	kif5b
ENSXETG00000011660	2256	210	311	272	586	466	736	1170	1345	791	1118	736	848	epc1
ENSXETG00000011662	1350	0	0	0	0	0	0	0	0	0	0	0	0	
ENSXETG00000011663	2121	2	10	8	48	42	18	37	43	21	40	30	39	ankk1
ENSXETG00000011664	1890	0	0	0	43	34	1	3	6	1	5	2	3	ttc12
ENSXETG00000011665	2532	3	4	6	22	19	30	435	580	288	91	64	67	NCAM1
ENSXETG00000011666	1767	1265	1817	1463	1337	974	1396	316	412	241	1355	949	1076	frrs1
ENSXETG00000011667	1590	2844	4263	3474	2808	2123	6764	347	439	260	1796	1140	1343	palmd
ENSXETG00000011668	1507	45	77	65	163	105	255	219	259	155	393	279	313	heca
ENSXETG00000011673	726	1051	1452	1270	841	650	1062	474	538	294	1167	834	972	ociad1
ENSXETG00000011674	634	1363	2017	1797	2323	1729	1745	3971	4490	2595	2177	1620	1795	ociad2
ENSXETG00000011676	2984	87	127	91	219	167	452	1198	1444	780	695	468	542	sgcb
ENSXETG00000011677	1666	349	501	421	589	484	1188	1311	1543	907	1023	678	761	rasl11b
ENSXETG00000011678	1092	33	58	52	199	163	239	110	99	56	201	140	150	scfd2
ENSXETG00000011679	2110	980	1416	1260	3441	2636	6365	3259	3542	2195	7773	5464	5926	fip1l1
ENSXETG00000011680	2229	7	16	11	83	49	62	179	226	136	129	88	108	lnx1
ENSXETG00000011681	1986	222	308	233	288	270	487	557	736	405	352	266	311	chic2
ENSXETG00000011682	1476	78	115	89	131	105	229	415	483	304	166	146	144	mapkapk5
ENSXETG00000011683	929	1	2	0	0	0	2	6	9	6	0	0	0	myl2
ENSXETG00000011684	6745	65	96	72	135	86	118	139	178	93	70	38	40	cux2
ENSXETG00000011685	1578	985	1540	1386	2540	1977	5751	553	704	420	2463	1821	1971	slc1a1
ENSXETG00000011686	6129	545	767	633	475	427	1314	680	841	429	492	346	382	stx2
ENSXETG00000011692	2787	1	3	1	8	1	1	7	7	6	8	9	9	glis3
ENSXETG00000011693	1099	0	0	0	0	0	0	1	2	1	0	0	0	
ENSXETG00000011697	1453	2451	3420	2766	2281	1771	2056	3545	4037	2409	1430	1036	1086	ube2n
ENSXETG00000011698	2296	2	4	3	19	14	18	33	41	21	31	27	17	rfx3
ENSXETG00000011699	2475	32	47	41	171	121	303	459	449	295	365	265	329	ncoa7
ENSXETG00000011700	1437	89	121	113	59	63	98	208	215	138	75	61	70	trmt11
ENSXETG00000011704	2001	18	25	17	72	54	147	887	1027	588	172	117	124	flrt2
ENSXETG00000011705	543	1	1	0	0	0	1	0	0	0	0	0	0	ENSXETG00000011705
ENSXETG00000011707	2199	992	1473	1208	1649	1254	2920	2115	2520	1500	2941	2015	2267	pum3
ENSXETG00000011708	1497	0	1	1	0	0	2	0	1	1	1	2	2	kcnv2
ENSXETG00000011709	3581	512	698	623	549	479	1221	663	826	461	725	531	628	vldlr
ENSXETG00000011714	1626	34	33	40	32	39	93	166	196	115	85	61	57	ENSXETG00000011714
ENSXETG00000011715	1476	0	2	4	4	5	2	3	2	0	8	4	1	ENSXETG00000011715
ENSXETG00000011717	2915	435	547	412	906	681	1304	1243	1445	955	1318	924	1047	rasa1
ENSXETG00000011718	1301	301	367	324	284	210	226	289	348	218	226	154	172	ccnh
ENSXETG00000011719	4294	207	219	196	72	85	133	182	230	137	78	54	62	tmem161b
ENSXETG00000011722	1140	2715	3811	3270	4768	3729	6588	9811	11485	6804	3704	2821	3193	hnrnpk
ENSXETG00000011723	1837	23	30	26	47	44	62	176	196	100	108	49	75	rmi1
ENSXETG00000011724	1869	2	0	1	1	1	14	69	63	41	7	5	3	slc28a3
ENSXETG00000011726	1488	0	0	1	1	2	4	35	34	25	19	14	10	ntrk2
ENSXETG00000011727	865	169	279	218	156	116	297	484	510	327	143	118	108	chordc1
ENSXETG00000011728	3303	21	22	17	112	109	225	839	987	596	240	154	201	naalad2
ENSXETG00000011730	909	0	0	0	0	0	0	0	2	0	0	0	0	
ENSXETG00000011732	4183	121	162	139	235	169	237	310	330	173	281	221	301	agtpbp1
ENSXETG00000011734	1755	0	1	0	3	1	9	250	299	161	13	14	9	nox4
ENSXETG00000011735	3358	255	341	265	619	475	570	664	922	520	495	368	409	kitlg
ENSXETG00000011736	2787	394	537	415	591	471	1040	961	1236	700	765	546	566	tmtc3
ENSXETG00000011738	2232	0	0	1	0	3	14	7	12	8	3	2	0	tyr
ENSXETG00000011740	3561	0	0	0	1	1	3	4	1	6	5	1	0	grm5
ENSXETG00000011742	7632	46	68	55	163	114	214	270	305	179	202	141	137	cep290
ENSXETG00000011743	1158	0	1	0	3	2	3	2	0	1	1	3	2	htr1b
ENSXETG00000011744	1437	0	0	0	0	0	0	0	0	0	0	0	0	
ENSXETG00000011745	1311	0	0	1	4	2	0	7	7	4	7	4	4	rassf9

ENSXETG00000011747	5720	158	217	198	635	469	835	851	998	563	1247	896	998	phip
ENSXETG00000011748	1537	10607	15435	12974	10803	8147	164426	51733	58079	36170	21957	15290	17237	ctsc
ENSXETG00000011749	3813	2	1	0	6	4	3	28	43	18	26	19	23	adgrb3
ENSXETG00000011751	639	0	3	3	2	2	5	32	36	14	12	19	4	rab38
ENSXETG00000011752	882	0	0	0	0	0	0	0	0	0	0	0	0	
ENSXETG00000011753	1694	1021	1315	1085	1057	783	2515	970	1249	710	1596	1043	1331	lmbrd1
ENSXETG00000011756	1561	1893	2543	2225	4318	3271	11235	2148	2655	1473	833	595	691	ENSXETG00000011756
ENSXETG00000011757	4128	3	7	3	60	61	72	841	883	570	123	108	81	COL5A3
ENSXETG00000011758	2786	14	23	11	80	69	255	2386	2664	1749	279	169	223	adam33
ENSXETG00000011761	3921	492	697	648	2017	1461	2355	2588	2899	1791	1775	1348	1371	dctn1
ENSXETG00000011762	1635	66	69	65	75	76	286	106	132	73	110	81	107	ENSXETG00000011762
ENSXETG00000011764	1032	325	487	417	689	502	685	856	912	612	1226	819	902	smyd5
ENSXETG00000011767	2982	5	8	7	10	3	10	63	56	53	15	17	16	ddx43
ENSXETG00000011768	2607	41	58	61	182	134	166	292	402	222	60	39	32	ttk
ENSXETG00000011771	858	931	1299	1084	800	657	1737	643	797	463	2248	1589	1784	nr0b2
ENSXETG00000011773	1146	462	691	544	703	489	1136	783	960	545	831	607	634	bckdhb
ENSXETG00000011775	1248	0	0	0	0	0	0	1	1	1	0	1	0	egr4
ENSXETG00000011776	3469	0	0	0	2	0	1	14	8	7	3	1	2	fbxo41
ENSXETG00000011777	579	103	203	165	85	64	90	167	200	112	94	86	65	pradc1
ENSXETG00000011778	2125	3951	6008	5160	7385	5487	8409	12453	14157	8577	10813	7801	8637	cct7
ENSXETG00000011779	486	638	991	849	798	585	1153	621	725	449	735	487	607	glod5
ENSXETG00000011780	1503	0	0	0	0	0	0	2	0	1	0	0	0	
ENSXETG00000011781	3741	14	14	23	95	68	90	418	413	241	194	148	173	rab11fip5
ENSXETG00000011782	912	209	323	259	142	112	355	225	233	168	221	151	171	sfxn5
ENSXETG00000011784	732	1	0	0	1	0	0	0	0	0	0	0	0	
ENSXETG00000011787	774	13	22	26	409	361	105	68	62	29	1027	715	830	pdx1
ENSXETG00000011788	336	562	776	636	1086	899	1609	593	659	405	1239	830	941	cdx2
ENSXETG00000011791	2070	3	1	5	33	37	39	152	178	108	91	47	65	flt1
ENSXETG00000011794	5145	253	335	274	732	530	826	1105	1184	744	1089	818	858	pan3
ENSXETG00000011802	1591	167	263	210	491	336	900	2039	2192	1455	583	376	417	uap1l1
ENSXETG00000011804	2745	9	14	7	0	4	2	87	93	55	4	4	4	grik1
ENSXETG00000011808	1585	212	326	299	517	385	577	1066	1235	715	692	439	567	pak2
ENSXETG00000011811	4926	442	634	492	539	446	1014	1514	1764	1023	741	517	614	fbxo45
ENSXETG00000011813	798	1	1	0	8	5	7	175	248	144	39	20	34	ENSXETG00000011813
ENSXETG00000011814	3849	2414	3455	2995	1222	872	2118	3241	3784	2176	1207	928	1034	tf
ENSXETG00000011817	2828	717	1103	876	1483	1180	2423	1755	2047	1177	1105	742	888	slco2a1
ENSXETG00000011818	3433	334	452	380	827	644	1338	2133	2328	1401	1456	995	1140	ryk
ENSXETG00000011819	2205	246	376	358	831	656	772	1471	1604	1031	1331	951	1101	amotl2
ENSXETG00000011820	1692	1	2	3	8	7	6	18	17	11	18	19	15	kcnb2
ENSXETG00000011825	1650	243	378	367	399	285	505	2798	3018	1856	419	270	304	rdh10
ENSXETG00000011826	2536	262	370	307	672	517	869	1073	1223	763	1493	1009	1229	stau2
ENSXETG00000011827	522	155	191	170	151	140	251	332	333	212	231	163	209	ube2w
ENSXETG00000011828	627	1409	2086	1622	1153	889	1304	2390	2912	1532	1075	835	848	eloc
ENSXETG00000011833	7643	55	66	68	168	128	453	334	387	220	953	660	711	ptprd
ENSXETG00000011834	1991	16	35	26	182	130	250	280	283	203	437	338	338	jph1
ENSXETG00000011835	1074	0	0	0	0	0	0	4	5	2	1	0	0	
ENSXETG00000011838	2005	685	1052	848	591	477	1213	1471	1636	949	875	641	685	btg2
ENSXETG00000011839	1621	226	333	251	334	267	548	701	866	498	586	435	435	XB-GENE-5763443
ENSXETG00000011842	1515	5	8	14	6	7	8	35	30	14	8	3	1	chia
ENSXETG00000011843	2061	74	125	94	180	151	187	399	480	244	165	105	122	usp49
ENSXETG00000011844	1622	40	52	46	79	61	215	960	1098	663	290	197	238	chit1
ENSXETG00000011845	1513	693	1015	861	1713	1282	2701	1633	1913	1164	3493	2506	2795	taf8
ENSXETG00000011846	1581	1356	1917	1668	1355	1119	1233	1860	2053	1245	1496	1015	1147	bysl
ENSXETG00000011847	468	102	155	128	145	109	248	313	333	211	241	150	185	med20
ENSXETG00000011850	2548	63	66	58	120	85	151	193	236	161	167	111	130	frs3
ENSXETG00000011851	1427	0	1	0	1	0	0	1	0	0	0	0	0	ENSXETG00000011851
ENSXETG00000011852	1146	0	0	0	0	0	0	0	0	0	0	0	0	
ENSXETG00000011854	6368	155	218	183	263	227	377	421	510	268	382	272	299	tfeb
ENSXETG00000011855	1629	18	19	18	34	23	8	5	5	4	5	1	4	zp4.2
ENSXETG00000011856	2356	236	286	237	2523	1933	2125	2160	2369	1476	4489	3124	3451	foxp4
ENSXETG00000011857	1721	8808	12713	10613	9883	7605	17318	7838	9335	5461	11398	8129	9161	tmem30cp
ENSXETG00000011858	1024	385	558	513	448	348	605	883	1086	592	487	365	407	cmss1
ENSXETG00000011861	4038	1163	1646	1389	1708	1326	3908	2163	2720	1503	2331	1605	1828	tbc1d23
ENSXETG00000011863	582	0	1	0	0	0	0	2	0	0	0	0	0	hist1h2am
ENSXETG00000011864	1820	237	321	273	469	357	369	470	561	317	320	222	251	nit2
ENSXETG00000011865	2113	825	1179	1004	1038	826	1304	1630	1799	1148	1218	862	952	nfya
ENSXETG00000011866	564	2	2	1	4	4	2	0	0	1	3	2	2	hist1h2aa
ENSXETG00000011867	2609	2070	2971	2539	2961	2382	3594	4236	5009	3006	3236	2320	2639	tomm70
ENSXETG00000011868	2184	382	529	487	1219	879	1667	796	968	540	1721	1157	1350	sort1
ENSXETG00000011869	1639	31	49	37	50	22	50	14	23	13	51	36	43	sypl2
ENSXETG00000011870	822	1503	2248	1910	901	667	2648	1543	1742	1039	767	587	618	tmem45a
ENSXETG00000011875	1353	1	0	0	0	0	0	2	2	2	5	5	3	
ENSXETG00000011876	2033	3447	4869	4126	4208	3135	3442	5665	6708	3839	1938	1388	1600	etf1
ENSXETG00000011877	914	1966	2893	2480	1460	1157	1451	21084	24007	13868	12157	8390	9412	cd74
ENSXETG00000011879	4837	683	984	808	1345	979	1557	1520	1679	1023	2321	1635	1866	tcof1
ENSXETG00000011881	1722	1	0	1	14	7	15	1006	1151	660	132	92	94	arsi
ENSXETG00000011885	1905	6	5	5	11	8	6	18	34	14	9	5	1	slc6a7
ENSXETG00000011887	4862	378	558	484	615	468	1412	6198	7112	4136	1018	688	721	csf1r
ENSXETG00000011890	3439	177	255	228	625	451	781	1188	1313	860	1181	802	899	rere
ENSXETG00000011897	1767	23710	34814	29767	28945	21717	34343	16079	18952	10928	33647	23783	25809	eno1
ENSXETG00000011901	1634	207	288	259	642	518	734	2	4	1	3328	2441	2594	slc2a5
ENSXETG00000011902	1658	51	64	61	37	33	97	106	121	61	85	68	90	ca6
ENSXETG00000011911	1101	26	26	26	31	26	61	2	1	1	7	3	2	ENSXETG00000011911
ENSXETG00000011917	2530	0	0	0	1	0	1	2	1	0	3	1	1	gabrb2
ENSXETG00000011918	714	0	0	0	0	0	0	0	0	0	0	0	0	
ENSXETG00000011937	1663	113	150	126	121	87	223	830	800	569	234	173	209	was
ENSXETG00000011938	2270	378	551	454	946	604	811	1610	1762	1113	879	595	748	prpf19
ENSXETG00000011939	2636	835	1259	1039	2133	1691	2221	2381	2496	1514	3918	2804	3132	slc38a5
ENSXETG00000011941	2958	17	30	20	111	64	195	1759	2088	1214	265	211	202	tmem132a
ENSXETG00000011942	1203	9	7	17	17	9	14	18	18	17	4	2	4	pga4
ENSXETG00000011944	3547	589	927	751	1150	889	1324	1214	1460	919	1092	811	875	patl1
ENSXETG00000011949	1491	27	48	32	250	190	403	1633	1850	1074	441	312	321	antxrl
ENSXETG00000011951	2950	521	693	669	1081	839	1387	1413	1568	940	1182	892	956	rnf20
ENSXETG00000011955	3681	0	0	0	0	0	0	0	0	0	0	0	0	
ENSXETG00000011956	1125	0	0	0	0	0	0	0	0	0	0	0	0	
ENSXETG00000011958	2645	443	617	576	595	494	501	589	636	424	172	127	131	abcb8
ENSXETG00000011959	1338	0	0	0	0	0	0	4	3	0	1	1	0	gdf10
ENSXETG00000011960	765	78	99	69	170	123	242	56	71	49	255	196	203	ENSXETG00000011960
ENSXETG00000011962	3398	29	57	32	318	245	380	2708	3117	1878	992	672	749	dact1
ENSXETG00000011966	4483	77	104	90	269	188	157	328	348	207	142	102	132	kiaa0586
ENSXETG00000011968	4022	184	252	232	709	508	882	1133	1278	736	1123	811	846	arid4a
ENSXETG00000011970	2052	0	1	0	0	1	0	0	0	0	0	1	1	
ENSXETG00000011972	1608	469	642	534	414	285	629	522	680	387	229	146	168	alg2
ENSXETG00000011974	1983	16	30	17	15	21	35	125	115	66	12	5	13	moxd1
ENSXETG00000011975	1155	936	1313	1128	711	611	938	1020	1300	693	642	419	486	sacm1l
ENSXETG00000011978	956	1944	2797	2344	2390	1803	2443	5619	6791	3817	1427	946	1110	psma3
ENSXETG00000011979	3435	19	32	28	182	155	305	452	496	319	213	155	201	nos3
ENSXETG00000011980	1345	523	790	641	747	625	1115	1388	1582	939	836	613	640	actr10
ENSXETG00000011982	1041	1	0	0	0	0	0	1	0	0	0	1	0	kcnk7
ENSXETG00000011984	1167	0	1	1	3	0	0	0	1	2	4	5	5	slc35f4
ENSXETG00000011985	2226	87	167	134	366	297	842	738	827	472	466	342	408	atg9b

ENSXETG00000011986	1536	45	66	69	98	71	174	90	85	59	117	56	99	ENSXETG00000011986
ENSXETG00000011987	6828	0	0	0	2	0	0	0	0	0	0	0	0	
ENSXETG00000011988	2238	2	2	3	4	0	0	4	0	5	1	2	0	aoc1
ENSXETG00000011990	2487	413	582	474	673	581	1110	983	1147	669	1065	713	793	naa30
ENSXETG00000011991	513	1	2	1	0	0	0	0	0	0	0	0	0	
ENSXETG00000011993	429	672	1026	873	404	351	468	504	598	379	428	284	297	srp19
ENSXETG00000011994	1675	200	246	219	311	273	525	501	669	398	439	302	331	ap5m1
ENSXETG00000011995	2106	415	646	563	512	391	851	919	1191	658	579	447	490	exoc5
ENSXETG00000012001	747	0	0	0	0	0	0	0	0	0	0	0	0	
ENSXETG00000012007	2361	0	0	0	1	2	0	2	0	1	1	0	0	
ENSXETG00000012010	6571	165	245	189	543	491	516	1895	2227	1252	780	577	606	RAPGEF2
ENSXETG00000012011	3213	135	194	182	468	375	3923	1127	1325	803	1850	1458	1573	fnip2
ENSXETG00000012021	1599	7	13	12	91	70	93	221	253	208	126	97	92	sema5b
ENSXETG00000012022	1104	197	298	261	1001	753	2173	1798	1901	1357	1760	1249	1410	dirc2
ENSXETG00000012023	1293	53	54	51	145	111	65	69	67	58	147	79	102	hspbap1
ENSXETG00000012024	2139	34055	49202	42096	81177	62575	102417	4637	5188	3356	23996	16573	18572	slc15a2
ENSXETG00000012025	1993	0	0	0	0	0	0	5	5	3	0	0	2	tfap2c
ENSXETG00000012028	2031	899	1374	1084	1953	1491	2894	2522	2817	1744	2074	1496	1660	rtfdc1
ENSXETG00000012029	2496	13	7	6	29	17	31	262	291	187	57	36	32	cass4
ENSXETG00000012030	1889	273	351	311	502	321	458	813	837	587	404	290	266	cstf1
ENSXETG00000012032	1248	49	82	66	204	147	151	299	312	185	45	24	21	aurka
ENSXETG00000012033	3003	67	104	82	141	86	155	204	253	189	110	78	100	LATS1
ENSXETG00000012035	636	976	1450	1185	1412	1117	1885	2215	2451	1483	2097	1355	1525	mlx
ENSXETG00000012036	1123	855	1247	1136	351	253	946	801	856	506	602	410	445	coq10b
ENSXETG00000012037	1674	306	492	407	852	605	1155	556	568	366	1238	840	940	coasy
ENSXETG00000012041	957	2	5	3	11	3	15	161	146	90	35	35	38	creb5
ENSXETG00000012042	2931	596	847	720	1072	746	2489	1071	1197	713	734	513	598	lipe
ENSXETG00000012043	2358	5	10	7	38	28	41	387	500	271	64	42	55	tril
ENSXETG00000012044	889	0	0	0	0	0	0	1	1	1	0	0	0	XB-GENE-5841027
ENSXETG00000012046	1611	499	783	668	329	231	787	2022	2385	1287	562	411	458	cpvl
ENSXETG00000012047	1368	86	136	108	205	137	315	471	511	316	415	282	310	chn2
ENSXETG00000012050	3381	3	2	5	213	154	30	37	42	18	338	238	298	PRDM16
ENSXETG00000012051	1143	685	967	778	1414	1214	3109	489	548	339	1792	1363	1527	ENSXETG00000012051
ENSXETG00000012053	1545	5	2	1	9	9	22	107	125	77	82	45	78	tmem145
ENSXETG00000012054	3744	675	1030	848	1631	1235	1380	1922	2145	1388	1638	1133	1367	prpf6
ENSXETG00000012057	1227	22	38	17	28	29	35	31	42	27	70	34	57	scrn1
ENSXETG00000012058	1422	1468	2043	1754	547	403	1668	231	247	139	551	395	430	rhag
ENSXETG00000012059	672	0	0	0	0	0	0	0	0	0	0	0	0	
ENSXETG00000012062	504	125	231	172	295	185	388	336	377	216	734	521	582	ENSXETG00000012062
ENSXETG00000012063	1225	885	1292	1066	1142	855	1268	3042	3286	1945	1434	1007	1087	pafah1b3
ENSXETG00000012067	4013	431	694	609	1597	1266	1913	2079	2347	1344	2468	1704	1880	erf
ENSXETG00000012072	1931	753	1097	913	1180	882	1492	1856	2174	1266	1163	949	987	gsk3a
ENSXETG00000012074	1189	1215	1622	1359	1273	967	938	497	649	334	387	244	280	c1orf50
ENSXETG00000012077	2118	0	2	5	4	2	2	1	0	0	3	0	2	adam21
ENSXETG00000012078	1746	636	943	758	934	693	1115	424	527	311	420	288	363	h6pd
ENSXETG00000012079	3004	91	122	107	246	181	299	540	634	365	323	211	259	spsb1
ENSXETG00000012080	1278	0	3	1	0	2	3	20	23	14	2	3	3	hes5.2
ENSXETG00000012081	1606	0	0	0	0	2	3	14	19	10	4	7	2	hes5.1
ENSXETG00000012083	2525	251	311	281	408	309	562	463	495	278	824	527	609	pank4
ENSXETG00000012087	1151	0	0	0	0	0	0	0	0	0	0	0	0	
ENSXETG00000012089	2397	2	1	2	1	1	0	4	3	0	3	0	1	slco1b3
ENSXETG00000012090	5945	137	247	169	355	284	762	1136	1381	831	838	553	687	tet3
ENSXETG00000012091	1440	4	6	11	45	27	55	241	303	173	318	251	247	XB-GENE-5860197
ENSXETG00000012092	4777	112	153	142	370	276	744	361	418	227	754	547	659	plch2
ENSXETG00000012093	3681	736	1043	871	2840	2115	3133	1767	2169	1296	4399	3253	3650	dedd2
ENSXETG00000012094	2136	712	1025	789	3285	2664	4828	5428	6433	3633	11223	7873	8790	mthfd2
ENSXETG00000012095	2658	2042	2828	2451	2358	1855	2811	3955	4695	2718	2009	1448	1591	mob1a
ENSXETG00000012096	984	96	131	122	98	117	224	119	135	87	105	86	85	pex10
ENSXETG00000012097	600	0	1	1	16	16	0	4	5	3	1	3	0	morn1
ENSXETG00000012098	2640	1	2	1	6	0	2	1	2	4	0	0	1	slc4a5
ENSXETG00000012100	804	6	9	9	38	27	63	65	97	50	46	34	39	btnl2
ENSXETG00000012101	1749	15	21	9	112	91	131	1021	1087	784	323	221	265	aplp1
ENSXETG00000012105	1658	0	0	0	0	0	0	0	2	0	2	0	1	
ENSXETG00000012108	3546	171	287	217	583	407	636	877	982	571	880	616	718	fam193a
ENSXETG00000012109	1528	621	931	749	1004	762	1456	934	1102	642	983	729	842	aar2
ENSXETG00000012110	921	1	0	0	4	0	2	1	2	1	24	21	14	rhbdl1
ENSXETG00000012111	5194	721	1097	954	3040	2525	4069	2814	3095	1869	6936	4881	5450	epb41l1
ENSXETG00000012114	3014	403	594	486	465	347	4070	6043	7015	4226	1730	1200	1362	tnip2
ENSXETG00000012115	1647	15	25	28	31	27	20	52	63	42	91	73	66	l3mbtl1
ENSXETG00000012118	3435	0	0	0	4	4	11	43	46	31	21	15	18	klhl30
ENSXETG00000012119	3303	428	596	508	1846	1406	4600	3167	3897	2256	3976	2703	3038	sgk2
ENSXETG00000012120	696	276	401	335	151	104	62	134	142	90	52	44	43	jmjd8
ENSXETG00000012121	2783	5	3	8	33	23	11	56	61	26	61	50	33	XB-GENE-5729976
ENSXETG00000012124	1542	100	161	144	425	302	505	560	680	411	324	226	259	ift52
ENSXETG00000012125	2781	69	97	92	338	230	152	361	414	222	105	103	87	mybl2
ENSXETG00000012126	4320	0	0	0	0	1	3	53	58	24	11	10	10	ptprt
ENSXETG00000012127	2776	5017	7180	5981	7950	6094	9622	13995	16382	9833	10439	7131	8097	efhd1
ENSXETG00000012129	1646	169	240	190	253	190	332	373	432	251	608	422	471	ilkap
ENSXETG00000012130	1269	0	0	1	0	0	1	1	1	1	1	2	0	ENSXETG00000012130
ENSXETG00000012131	2507	389	581	535	1184	836	1412	2362	2590	1642	1222	824	899	tmem8a
ENSXETG00000012132	1265	165	250	229	235	157	192	439	487	290	203	151	186	trmt10a
ENSXETG00000012135	1647	1	0	0	1	0	0	0	0	0	0	0	0	
ENSXETG00000012138	5433	1	3	6	34	32	40	138	151	83	98	48	58	kif1a
ENSXETG00000012139	918	5577	8202	7038	7653	5644	7736	12956	14624	8471	8048	5671	6116	arhgdig
ENSXETG00000012140	1678	0	1	1	33	13	4	6	8	3	310	220	253	pdia2
ENSXETG00000012141	2180	133	199	166	296	197	373	427	458	253	327	208	233	actr5
ENSXETG00000012142	3094	98	110	109	650	534	136	582	666	401	618	453	530	ppp1r16b
ENSXETG00000012143	2634	171	245	222	243	189	515	426	538	316	650	454	503	axin1
ENSXETG00000012144	1236	20	40	20	133	107	121	168	152	104	21	14	17	fam83d
ENSXETG00000012145	2082	142	192	170	261	215	277	461	543	363	182	170	158	dhx35
ENSXETG00000012146	6417	242	358	356	653	478	962	2728	3012	1907	1071	752	775	mprip
ENSXETG00000012147	711	35	38	37	18	10	8	2	5	0	13	7	7	ENSXETG00000012147
ENSXETG00000012148	1233	0	0	1	0	0	0	3	2	1	0	0	0	ENSXETG00000012148
ENSXETG00000012149	1595	2	8	9	6	2	0	4	1	1	5	2	1	agxt
ENSXETG00000012151	1028	169	306	222	267	213	361	807	828	555	405	270	341	gid4
ENSXETG00000012152	2526	0	0	0	0	0	0	0	0	0	0	0	0	
ENSXETG00000012153	1414	932	1408	1121	636	479	540	926	1010	628	634	457	402	atpaf2
ENSXETG00000012154	5886	0	1	2	731	603	18	9	9	4	0	7	2	ENSXETG00000012154
ENSXETG00000012155	1892	2	3	4	89	63	14	9	6	3	8	10	7	drc3
ENSXETG00000012156	1527	113	164	152	350	278	875	419	400	265	459	315	352	tom1l2
ENSXETG00000012160	3321	1745	2705	2381	2005	1567	1993	2061	2226	1395	4337	3050	3395	srebf1
ENSXETG00000012163	1472	1087	1616	1361	1772	1351	2511	3089	3558	1934	1815	1293	1471	ppp1r7
ENSXETG00000012164	5149	18237	26587	23041	33136	25554	36147	28462	32303	19074	27734	19948	22356	hdlbp
ENSXETG00000012165	5418	32	45	38	85	75	143	810	859	509	226	164	199	rai1
ENSXETG00000012166	5455	292	391	318	548	420	805	3630	4367	2361	849	621	727	sept2
ENSXETG00000012168	1545	0	2	0	1	0	0	1	0	0	4	1	2	ENSXETG00000012168
ENSXETG00000012170	1486	2249	3440	3007	5559	4134	6771	12093	13965	8714	6263	4232	4914	hnrnpa2b1
ENSXETG00000012171	2926	9	22	19	94	73	184	302	363	218	274	195	190	boc
ENSXETG00000012173	1836	258	370	339	489	335	550	702	824	455	671	440	513	ccdc9
ENSXETG00000012174	2279	252	371	316	452	340	443	2676	3222	1847	957	695	688	nfe2l3
ENSXETG00000012175	961	793	1150	977	1157	979	1207	3059	3745	2027	1343	937	1001	cbx3

ENSXETG00000012176	3599	111	173	169	291	233	438	501	551	363	482	294	411	dhx34
ENSXETG00000012179	1449	0	0	0	0	0	0	9	4	5	0	3	4	rtn4r
ENSXETG00000012180	1374	221	354	274	228	194	360	614	714	419	301	188	242	snx10
ENSXETG00000012181	1983	142	216	165	187	158	423	2088	2537	1459	494	364	397	skap2
ENSXETG00000012183	6336	114	206	148	520	422	600	683	844	485	216	149	192	cit
ENSXETG00000012184	1737	1458	2212	1911	2029	1582	2445	1271	1406	827	2329	1675	1915	blnk
ENSXETG00000012185	3741	216	274	212	538	448	782	758	935	557	968	722	787	znf518a
ENSXETG00000012186	4213	5	5	6	18	15	20	487	613	363	42	13	32	ccnj
ENSXETG00000012187	2939	283	332	297	420	354	631	828	943	606	898	657	747	dusp11.2
ENSXETG00000012188	5271	170	272	199	543	386	537	865	1009	565	612	438	484	btaf1
ENSXETG00000012189	2814	36	49	29	216	152	218	866	1049	601	220	150	162	bicdl1
ENSXETG00000012190	2269	33	41	51	133	93	108	73	64	41	75	64	82	cpeb3
ENSXETG00000012192	1478	372	517	446	364	293	1000	1241	1505	812	393	341	344	tmem144
ENSXETG00000012193	8316	2032	2809	2338	4196	3315	3802	4483	5258	2998	3436	2366	2884	gcn1
ENSXETG00000012195	726	810	1169	996	609	462	645	833	1010	537	434	286	329	c14orf166
ENSXETG00000012196	3244	0	0	1	0	1	5	18	21	14	26	16	16	slc26a4
ENSXETG00000012197	3122	881	1233	1035	1876	1540	3133	1284	1374	860	1895	1348	1469	march5
ENSXETG00000012198	4572	166	238	196	938	710	1235	4588	5082	2996	568	410	435	nid2
ENSXETG00000012201	2436	586	899	788	1103	940	1721	1751	2087	1227	1824	1311	1498	pxn
ENSXETG00000012202	1119	165	234	198	68	43	113	286	355	196	137	77	87	ptger2
ENSXETG00000012203	3810	447	680	549	605	440	577	852	1040	573	696	448	547	txndc16
ENSXETG00000012204	4844	254	377	285	569	409	907	559	747	391	439	306	328	gpr137c
ENSXETG00000012205	1376	1201	1664	1402	263	213	221	487	557	306	220	142	193	ero1a
ENSXETG00000012207	315	1	1	0	15	12	10	5	3	6	183	106	128	pla2g1b
ENSXETG00000012208	1921	1064	1608	1336	3025	2310	2733	1778	2058	1179	1986	1475	1524	XB-GENE-5804925
ENSXETG00000012209	2133	87	123	115	141	106	228	404	472	280	227	157	180	socs4
ENSXETG00000012210	3378	133	206	151	552	419	242	847	924	578	267	174	177	wdhd1
ENSXETG00000012212	1089	1491	2175	1868	1659	1292	1027	274	327	194	537	351	395	hsd11b1l.2
ENSXETG00000012213	3519	131	189	156	695	561	602	935	1085	634	127	105	82	kif11
ENSXETG00000012214	2141	683	922	836	437	307	569	425	540	302	206	169	176	gch1
ENSXETG00000012215	2225	18	16	21	103	71	95	296	338	215	243	170	216	hhex
ENSXETG00000012216	1095	13	8	12	16	14	5	19	40	24	9	3	9	msi1
ENSXETG00000012217	1923	8	15	15	38	26	140	191	245	137	115	88	116	samd4a
ENSXETG00000012218	2304	0	0	0	0	0	0	0	0	0	0	0	0	
ENSXETG00000012221	1005	74	105	75	57	58	117	157	196	97	113	67	78	cgrrf1
ENSXETG00000012222	949	22124	33246	28638	16171	11892	11144	10551	11905	7268	7585	5574	5859	cox6a1
ENSXETG00000012223	409	1126	1546	1293	565	478	614	728	780	517	492	347	384	triap1
ENSXETG00000012224	1802	752	1057	816	966	784	1226	1736	2177	1217	583	436	438	gmfb
ENSXETG00000012226	3239	4991	7335	6397	9914	7597	15877	8904	10345	6276	10652	7494	8481	rnf10
ENSXETG00000012227	1683	2330	3416	2830	1818	1387	2457	2422	3071	1713	1072	794	890	cnih1
ENSXETG00000012228	936	326	472	451	371	282	403	269	373	238	237	179	221	coq5
ENSXETG00000012229	1131	0	1	0	1	0	7	41	60	26	4	3	5	uts2r
ENSXETG00000012230	1074	0	1	0	1	1	5	6	9	2	2	3	6	gal3st4
ENSXETG00000012231	1556	532	853	688	897	619	927	670	742	482	831	567	624	ogfod3
ENSXETG00000012232	1738	299	496	385	436	317	406	593	601	384	469	311	370	hexdc
ENSXETG00000012233	2268	3694	4768	3804	10708	8331	27530	1178	1578	803	14142	9947	11687	slc26a3.1
ENSXETG00000012234	882	182	274	230	509	342	655	1108	1218	737	655	478	553	ENSXETG00000012234
ENSXETG00000012235	3068	408	598	475	753	566	1215	1769	1909	1156	936	696	758	c17orf62
ENSXETG00000012236	1269	534	733	658	1750	1402	2162	1774	1913	1195	1052	769	775	narf
ENSXETG00000012238	2364	0	0	0	5	3	3	1	4	1	7	2	6	cdh18
ENSXETG00000012239	5158	305	444	410	1813	1428	1437	3363	3883	2310	1812	1317	1440	abcc3
ENSXETG00000012240	2691	2	0	0	0	1	2	8	12	9	3	0	1	veph1
ENSXETG00000012241	447	0	0	0	0	0	0	0	0	0	0	0	0	
ENSXETG00000012243	735	59	81	76	179	155	265	331	356	205	211	144	177	unc119b
ENSXETG00000012244	1269	29	66	41	123	85	265	245	341	173	176	124	119	PTX3
ENSXETG00000012246	2499	153	290	226	659	529	1560	717	841	537	1225	808	890	fgd4
ENSXETG00000012248	3957	24002	35415	29832	61710	47482	125386	5476	6318	3566	61096	43234	48546	plb1
ENSXETG00000012250	1935	564	824	646	831	693	795	1160	1350	833	1111	784	910	ccnl1
ENSXETG00000012252	1869	1071	1549	1328	2421	1911	3263	2576	2837	1914	1806	1321	1444	dnm1l
ENSXETG00000012254	1128	2	2	3	48	27	46	70	106	60	35	18	20	ENSXETG00000012254
ENSXETG00000012255	1004	1	0	6	10	5	8	26	29	16	9	9	13	dhrs7c
ENSXETG00000012256	1212	0	0	0	0	0	0	1	0	0	0	0	0	ANKRD65
ENSXETG00000012260	2371	1928	2631	2320	1515	1213	2727	3343	3880	2241	1416	1020	1125	tiparp
ENSXETG00000012261	1671	2202	3241	2896	4879	3701	5398	7503	8287	5260	4592	3341	3691	sept9
ENSXETG00000012262	1278	331	458	356	771	666	1431	864	954	554	1105	715	893	cbll1
ENSXETG00000012263	1204	0	0	0	2	1	5	20	34	13	12	5	10	kcnab1
ENSXETG00000012264	3023	5	12	18	49	54	111	487	529	291	210	161	159	pde2a
ENSXETG00000012265	300	8	9	4	25	20	27	153	157	108	26	34	29	kcne3
ENSXETG00000012269	1530	2971	4289	3851	6031	4472	5843	4543	5394	3067	4833	3488	3796	dld
ENSXETG00000012271	1131	189	232	220	301	223	372	303	310	199	581	443	456	yars2
ENSXETG00000012272	967	930	1489	1311	809	617	410	1052	1116	679	378	253	250	mlec
ENSXETG00000012273	1817	2991	4325	3801	3499	2584	4956	1729	1952	1203	2992	2141	2380	ttc38
ENSXETG00000012274	1233	0	0	0	0	0	0	4	10	5	0	0	1	ENSXETG00000012274
ENSXETG00000012275	2068	1644	2350	1952	2175	1654	2290	1442	1636	1022	1264	825	904	acads
ENSXETG00000012276	714	4956	7297	6089	9803	7726	4933	2362	2587	1710	2734	1877	2085	tspo
ENSXETG00000012277	2697	3	1	0	27	20	33	404	393	260	134	93	111	scube1
ENSXETG00000012278	1695	269	415	352	854	678	1197	582	683	381	913	659	720	hnf1a
ENSXETG00000012279	5346	156	214	179	647	530	695	4499	5497	3101	781	555	615	lamb1
ENSXETG00000012284	2556	29	46	40	17	23	36	38	42	20	23	21	22	ENSXETG00000012284
ENSXETG00000012286	867	442	654	555	922	677	3160	2951	3127	2033	1403	1020	1093	cebpb
ENSXETG00000012287	1754	182	316	232	278	233	460	564	676	337	461	343	353	ppp2r3c
ENSXETG00000012288	1729	370	459	432	602	431	885	1215	1391	877	976	627	791	ptpn1
ENSXETG00000012289	2994	1304	1948	1705	2072	1556	2759	1673	1918	1052	2558	1753	1982	rps6ka1
ENSXETG00000012290	1669	2121	3055	2674	2269	1722	2318	2611	3095	1884	1470	997	1129	srp54
ENSXETG00000012291	780	0	0	0	0	0	0	0	0	0	0	0	0	ENSXETG00000012291
ENSXETG00000012292	4134	7	9	11	12	3	30	119	118	79	42	32	36	ENSXETG00000012292
ENSXETG00000012293	4569	341	495	392	943	715	1035	1872	2137	1293	1232	887	1065	baz1a
ENSXETG00000012294	561	6	9	10	24	26	65	79	89	71	44	29	37	ENSXETG00000012294
ENSXETG00000012296	417	5	7	5	18	16	13	40	32	18	28	19	20	celf3
ENSXETG00000012297	3163	9	11	12	45	42	79	88	96	60	101	65	64	fam65c
ENSXETG00000012298	1208	116	161	135	355	259	486	781	917	572	542	397	415	pard6b
ENSXETG00000012300	3514	435	605	489	1568	1156	3387	2952	3463	1964	2909	1953	2252	adnp
ENSXETG00000012301	2383	186	295	228	800	606	2113	3308	3932	2258	3644	2542	2941	cfl2
ENSXETG00000012302	2000	2232	3211	2732	2612	2047	4544	4481	5217	3152	3479	2369	2726	snx6
ENSXETG00000012303	1028	970	1428	1224	965	723	1170	1444	1683	975	562	412	417	dpm1
ENSXETG00000012304	2151	5	8	4	6	4	5	70	107	55	21	10	20	capn3
ENSXETG00000012306	1620	953	1340	1162	578	463	557	823	944	551	223	138	166	slc33a1
ENSXETG00000012308	3149	5	6	2	64	44	100	182	197	122	33	11	12	kcng1
ENSXETG00000012311	2535	150	181	164	469	346	399	620	579	382	596	425	463	nfatc2
ENSXETG00000012312	645	1	6	5	21	15	29	10	19	9	34	23	19	c3orf33
ENSXETG00000012313	6460	388	559	479	1854	1509	2925	2320	2706	1622	2661	1836	2193	atp9a
ENSXETG00000012316	1677	76	125	96	52	43	128	158	144	88	190	131	174	slc25a21
ENSXETG00000012322	1500	666	1031	843	741	544	1010	888	1073	662	920	656	669	dhdds
ENSXETG00000012323	892	5484	7877	6853	9702	7367	6985	19040	22506	13608	4312	3025	3451	hmgn2
ENSXETG00000012324	1376	0	0	1	0	0	1	2	2	2	0	0	0	lin28a
ENSXETG00000012325	3825	488	655	642	1136	888	998	543	642	414	976	701	783	aim1l
ENSXETG00000012326	4422	8	11	7	8	3	17	89	93	66	31	29	31	sall4
ENSXETG00000012327	2311	43	51	49	113	83	126	203	210	139	131	102	94	zfp64
ENSXETG00000012328	1445	0	1	7	64	42	11	14	25	18	4	6	9	ubxn11
ENSXETG00000012330	712	888	1302	1047	1855	1499	1533	6129	7793	4347	2553	1831	1984	sh3bgrl3
ENSXETG00000012331	2299	63	90	78	293	229	89	320	367	205	144	90	100	cep85

ENSXETG00000012337	1071	0	0	0	0	0	0	0	0	0	0	0	0 thdl17
ENSXETG00000012340	1293	0	0	0	0	0	0	0	0	0	0	0	0 ENSXETG00000012340
ENSXETG00000012343	1799	38	46	58	51	37	130	942	1052	629	99	68	80 ENSXETG00000012343
ENSXETG00000012344	3225	306	461	354	424	323	552	714	915	455	574	397	438 dis3
ENSXETG00000012345	1538	17	42	38	16	19	84	798	934	539	64	40	31 ENSXETG00000012345
ENSXETG00000012348	786	0	0	0	0	0	0	1	1	0	0	0	0
ENSXETG00000012352	2517	0	0	0	0	0	1	7	13	5	6	4	4 slitrk4
ENSXETG00000012353	1816	37	47	44	144	123	156	181	217	131	45	18	26 bora
ENSXETG00000012355	3794	9	12	15	83	53	111	277	304	168	335	253	276 dach1
ENSXETG00000012362	2168	85	110	93	140	101	265	311	395	214	363	240	262 zbtb2
ENSXETG00000012364	2478	0	0	0	2	1	0	4	1	3	0	2	2 esr1
ENSXETG00000012367	26972	77	173	112	502	431	911	2054	2402	1396	1194	891	946 syne1
ENSXETG00000012371	1550	944	1283	1088	1236	933	1313	2329	2552	1715	2194	1513	1722 srsf11
ENSXETG00000012372	1985	254	411	343	306	239	292	444	498	335	374	293	292 lrrc40
ENSXETG00000012373	4803	0	0	1	1	0	0	1	0	1	1	3	2 lrrc7
ENSXETG00000012374	1143	56831	83507	70205	70526	52756	78797	59872	70513	40757	171824	119825	133593 rpl10a
ENSXETG00000012375	1341	187	241	233	248	187	728	127	160	93	549	391	457 pacsin1
ENSXETG00000012376	3357	61	82	87	327	232	387	395	485	245	63	40	48 depdc1
ENSXETG00000012377	2220	0	1	0	8	8	3	65	87	46	9	7	10 rpe65
ENSXETG00000012378	1887	454	638	534	517	406	602	266	336	189	414	291	343 ENSXETG00000012378
ENSXETG00000012383	1041	34	43	50	103	63	158	179	211	90	183	141	138 uhrf1bp1
ENSXETG00000012387	2586	209	293	262	427	361	689	2158	2839	1652	638	475	588 wls
ENSXETG00000012389	744	6	5	13	28	25	50	166	255	135	129	85	118 diras3
ENSXETG00000012392	1112	1903	2898	2480	726	515	1910	3974	4453	2633	1585	1179	1218 gadd45a
ENSXETG00000012394	3986	10110	14275	12230	28618	22318	38256	30885	35658	20930	52676	37247	40792 serbp1
ENSXETG00000012397	1920	18	22	17	9	12	42	132	155	79	71	43	43 il12rb2
ENSXETG00000012399	2343	22	28	20	53	39	103	47	47	45	74	63	68 ENSXETG00000012399
ENSXETG00000012400	1062	244	374	324	346	279	628	298	392	230	261	175	229 slc35d1
ENSXETG00000012401	1533	215	299	279	379	282	456	718	803	443	452	315	355 mier1
ENSXETG00000012403	2925	0	0	2	2	2	5	8	7	6	11	5	14 trpc5
ENSXETG00000012404	4092	7	16	7	123	88	184	188	190	119	169	91	126 fzd7
ENSXETG00000012405	1462	0	0	0	1	2	2	26	15	19	3	3	6 cdk15
ENSXETG00000012406	5050	371	567	460	772	594	1241	1121	1362	778	1120	775	929 als2
ENSXETG00000012407	2710	85	110	131	387	303	556	848	930	568	806	576	594 pard3b
ENSXETG00000012409	633	0	0	0	0	0	1	2	1	0	3	2	1
ENSXETG00000012416	1110	3	1	1	18	6	6	4	5	1	55	26	38 hs3st2
ENSXETG00000012418	1104	0	0	0	1	2	2	33	33	13	15	5	4 dcx
ENSXETG00000012419	3546	54	95	85	289	217	559	454	520	336	478	330	408 usp31
ENSXETG00000012420	1743	0	1	0	1	0	3	3	3	4	8	1	2 capn6
ENSXETG00000012421	1614	25	27	19	37	35	62	480	564	328	126	71	98 pak3
ENSXETG00000012422	2397	10	13	6	358	319	29	0	0	1	73	68	61 tmprss15
ENSXETG00000012423	1980	0	1	0	1	0	1	1	10	3	0	0	0
ENSXETG00000012424	375	46	90	74	204	182	279	1552	1761	1045	1665	1147	1281 ENSXETG00000012424
ENSXETG00000012426	2091	0	1	0	1	0	0	9	22	3	16	18	16 chrdl1
ENSXETG00000012428	1901	80	120	102	474	369	182	509	644	416	370	272	290 tmem164
ENSXETG00000012429	1542	137	187	159	380	291	292	1145	1402	842	601	408	464 acsl4
ENSXETG00000012431	2656	840	1262	1059	1488	1086	1704	1652	1792	1124	1282	919	991 cog7
ENSXETG00000012432	2042	139	207	142	569	421	646	528	592	359	513	371	397 IGSF8
ENSXETG00000012433	1626	545	755	725	511	401	753	468	506	368	596	385	426 ears2
ENSXETG00000012434	558	95	172	138	272	186	456	561	664	422	331	242	253 rit1
ENSXETG00000012436	599	30	64	34	74	54	24	19	19	13	18	14	12 zg16
ENSXETG00000012437	1335	16	28	15	126	115	153	167	164	116	140	101	99 ENSXETG00000012437
ENSXETG00000012439	381	0	0	0	1	0	0	0	0	1	2	1	0 ENSXETG00000012439
ENSXETG00000012440	1419	1	7	7	18	10	18	98	107	81	31	29	24 ENSXETG00000012440
ENSXETG00000012447	2026	2054	3005	2489	2588	2053	3735	2541	2789	1724	3020	2110	2240 aldh3b2
ENSXETG00000012450	870	83	139	107	109	108	178	217	223	163	140	93	112 lcmt2
ENSXETG00000012454	1344	85	129	109	78	62	104	82	113	63	108	72	101 slc17a9
ENSXETG00000012456	2334	181	269	262	412	339	1978	1297	1421	832	634	441	452 ENSXETG00000012456
ENSXETG00000012458	6510	5	7	5	95	63	238	73	90	47	332	254	277 cacna1e
ENSXETG00000012459	2431	89	129	117	358	266	320	587	592	377	174	136	133 ccnf
ENSXETG00000012460	6408	349	493	408	1928	1461	1180	1496	1855	1055	1503	1107	1223 pcm1
ENSXETG00000012462	1266	1	9	2	23	15	36	13	8	6	47	35	36 gtf2e1.2
ENSXETG00000012463	1200	1337	1965	1747	1525	1053	3654	5090	6049	3651	3328	2333	2669 asah1
ENSXETG00000012465	5841	1132	1734	1505	3443	2715	3561	2901	3051	1990	3381	2398	2687 polr2a
ENSXETG00000012467	1590	596	926	801	612	503	583	480	458	349	118	75	84 slc16a13
ENSXETG00000012468	4350	132	212	165	588	492	878	968	1047	701	1033	765	816 scrib
ENSXETG00000012469	1731	835	1330	1089	1651	1266	1653	2604	2899	1858	1989	1351	1501 puf60
ENSXETG00000012470	999	223	383	331	553	377	898	745	994	585	873	598	707 frg1
ENSXETG00000012482	1464	2	1	2	0	0	0	35	18	16	2	0	3 krt17
ENSXETG00000012487	1660	38	61	64	91	81	147	256	291	154	104	93	106 terf2ip
ENSXETG00000012488	1550	4	2	5	50	45	25	1	3	0	17	17	9 krt
ENSXETG00000012489	3984	4443	6399	5530	9140	6889	14184	4620	5354	3290	21939	15629	17141 hap1
ENSXETG00000012490	309	40	50	50	1098	830	63	2	4	0	764	596	635 gast
ENSXETG00000012497	1182	966	1451	1154	947	774	1941	1391	1806	951	897	606	691 ift22
ENSXETG00000012498	3327	94	124	96	87	68	352	9397	11216	6639	45	26	42 nos2
ENSXETG00000012500	1398	44	82	71	319	223	68	415	443	290	64	48	59 slc16a5
ENSXETG00000012503	1661	75	98	94	245	185	523	242	273	162	321	226	251 nlk
ENSXETG00000012504	1362	8	18	13	32	24	67	345	357	236	129	90	95 cdr2l
ENSXETG00000012505	805	1247	1777	1569	1024	806	1145	1456	1650	1043	907	656	696 mrpl58
ENSXETG00000012506	1809	2	1	2	8	11	32	83	86	46	43	25	32 znf91
ENSXETG00000012507	366	4751	7052	6208	4474	3534	4249	3641	4155	2496	2600	1865	2053 atp5h
ENSXETG00000012508	738	239	384	337	561	427	923	812	914	609	765	529	594 kctd2
ENSXETG00000012510	1097	1462	2262	1882	1872	1413	3007	2127	2583	1442	4209	2946	3232 mrps7
ENSXETG00000012511	3159	362	533	469	666	503	758	832	1013	560	1171	786	857 gga3
ENSXETG00000012512	702	152	252	191	801	677	2862	684	777	493	1263	838	976 XB-GENE-990053
ENSXETG00000012514	1059	251	384	276	581	467	1312	6590	7799	4370	844	546	681 npl
ENSXETG00000012515	4038	718	977	907	2417	1832	2331	2398	2887	1607	2341	1644	1949 dhx9
ENSXETG00000012516	938	1695	2546	2150	1735	1353	2147	4722	5035	3338	2415	1662	1912 sumo2
ENSXETG00000012519	1503	21	30	33	37	32	53	95	85	53	35	30	26 fam84a
ENSXETG00000012520	1724	2790	3989	3391	3426	2507	5171	6402	7119	4372	4046	2798	3170 grb2
ENSXETG00000012521	1086	898	1409	1152	1469	1082	1328	2041	2188	1417	1138	788	817 krtcap3
ENSXETG00000012522	1035	271	439	371	845	701	804	1431	1774	1051	968	705	743 nrbp1
ENSXETG00000012524	3981	231	334	309	619	415	1047	853	994	558	1019	743	803 tmem94
ENSXETG00000012525	4880	647	956	840	2079	1696	2117	4599	5197	3075	2288	1673	1705 lamc1
ENSXETG00000012526	3627	830	1135	1038	1604	1102	3018	2628	2939	1810	3260	2264	2545 caskin2
ENSXETG00000012527	3187	5	13	10	41	33	42	140	167	99	117	55	67 nmnat2
ENSXETG00000012528	3650	494	783	626	1384	1026	1856	1979	2239	1271	1815	1266	1404 smg7
ENSXETG00000012529	3456	2246	3262	2770	5239	4082	6588	3473	3957	2381	5825	4188	4508 llgl2
ENSXETG00000012530	1643	104	172	182	109	105	279	652	784	459	194	108	147 ncf2
ENSXETG00000012531	3369	42	86	60	159	136	236	382	416	281	233	187	196 abl2
ENSXETG00000012532	1943	203	346	284	498	339	528	476	503	320	301	196	225 tsen54
ENSXETG00000012540	1597	341	525	442	152	121	169	210	240	132	210	142	140 tor3a
ENSXETG00000012541	795	10	13	9	157	123	23	27	30	18	21	9	9 dnajb13
ENSXETG00000012544	1136	281	368	312	129	81	156	319	394	246	136	82	118 mettl6
ENSXETG00000012549	3085	691	1005	947	1085	886	1284	1387	1451	900	1025	764	813 sh3bp5
ENSXETG00000012551	921	0	0	0	0	0	0	0	0	0	0	0	0
ENSXETG00000012553	1023	284	443	374	415	327	566	904	997	604	632	419	456 sap30bp
ENSXETG00000012554	2451	921	1429	1205	940	752	1125	1730	2043	1188	858	576	655 capn7
ENSXETG00000012556	5472	299	400	358	1827	1315	1531	1588	1748	1089	3405	2425	2823 itgb4
ENSXETG00000012557	6552	18	26	18	179	161	196	3479	4420	2598	358	257	302 col6a3
ENSXETG00000012558	7410	11	31	15	230	151	275	1027	1152	683	548	399	423 ptpn13

ENSXETG00000012559	741	114	155	135	87	60	130	149	161	95	135	86	95	mrm2
ENSXETG00000012560	7237	67	101	85	356	292	277	361	378	207	347	265	254	c2cd3
ENSXETG00000012561	1137	174	253	252	467	390	764	811	893	561	917	682	727	ppme1
ENSXETG00000012563	561	0	0	0	0	0	0	0	0	0	0	0	0	
ENSXETG00000012564	2542	105	153	138	209	194	179	214	240	142	159	123	137	rasgef1b
ENSXETG00000012567	1377	2	3	0	2	5	4	7	3	2	2	6	3	p4ha3
ENSXETG00000012569	1353	1045	1505	1288	2393	1884	2664	4139	4966	2938	2983	2094	2323	hnrnpd
ENSXETG00000012570	2115	8	22	18	150	103	254	479	490	282	323	232	228	pecam1
ENSXETG00000012572	2598	0	0	0	0	1	0	0	1	0	2	2	3	piwil3
ENSXETG00000012573	1496	356	584	520	607	443	804	583	674	412	655	473	421	galk1
ENSXETG00000012575	411	1592	2310	1960	1489	1188	2546	4066	4574	2616	2777	1922	2175	h3f3b
ENSXETG00000012578	2569	119	152	131	236	151	358	522	637	385	421	318	344	unk
ENSXETG00000012579	1386	696	1104	941	586	376	697	881	971	643	763	563	583	polg2
ENSXETG00000012580	2132	12044	17158	14820	29892	22694	55277	45239	51332	31249	36683	25608	28159	ddx5
ENSXETG00000012581	665	37	68	46	122	94	151	102	114	72	105	77	82	wbp2
ENSXETG00000012582	1087	242	373	300	383	288	488	679	757	367	498	396	473	enoph1
ENSXETG00000012583	1935	30266	42794	35535	42455	32802	56288	588	744	383	13428	9429	10950	g6pc2
ENSXETG00000012584	2511	200	281	257	453	372	763	874	938	592	564	358	424	cep95
ENSXETG00000012585	2181	130	160	135	395	275	493	662	680	451	569	405	489	SMURF2
ENSXETG00000012587	1992	2	1	2	2	3	5	16	24	17	13	4	7	aoc2
ENSXETG00000012588	2422	331	452	430	880	713	1200	5107	6096	3545	1051	782	928	aoc3
ENSXETG00000012591	693	350	461	433	232	188	217	663	831	438	149	120	122	psme3
ENSXETG00000012596	3397	748	1042	938	939	711	1513	963	1218	673	1208	842	919	elac2
ENSXETG00000012598	1524	30	63	37	88	84	69	146	193	111	93	62	68	trim65
ENSXETG00000012599	6381	82	137	132	1271	962	626	620	712	403	1448	1036	1149	evpl
ENSXETG00000012601	2949	24436	36224	31308	32681	25357	21014	5642	6298	3738	13087	9263	10074	acox1
ENSXETG00000012604	1461	0	0	0	0	0	0	3	3	3	1	2	1	ENSXETG00000012604
ENSXETG00000012605	1365	7	9	9	43	27	26	79	99	49	32	21	24	ENSXETG00000012605
ENSXETG00000012606	1389	0	5	5	1	0	5	99	121	52	7	5	6	ENSXETG00000012606
ENSXETG00000012607	2304	983	1376	1180	1590	1187	2328	1765	2148	1211	1400	950	1110	cpt1a
ENSXETG00000012608	1842	0	2	0	5	2	4	23	27	18	6	2	3	ENSXETG00000012608
ENSXETG00000012610	1410	7	3	12	3	1	15	94	99	68	41	28	26	ENSXETG00000012610
ENSXETG00000012613	2079	507	734	620	1347	1021	2808	2153	2506	1532	2416	1743	1962	becn1
ENSXETG00000012615	3483	9	11	13	46	20	77	59	62	47	128	82	119	wnk4
ENSXETG00000012621	1122	1	1	2	12	14	16	254	315	191	89	66	71	galr2
ENSXETG00000012622	450	8	10	17	19	17	27	5	5	2	63	42	34	PLA2G1B
ENSXETG00000012629	5433	557	845	691	585	455	1317	5169	6250	3549	1090	789	852	nrp1
ENSXETG00000012631	2585	2270	3359	2902	2947	2254	3033	3022	3416	2102	3208	2208	2437	srp68
ENSXETG00000012633	426	0	0	0	0	0	0	0	0	0	0	0	0	ENSXETG00000012633
ENSXETG00000012640	1503	3596	4970	4333	1865	1377	3277	1618	1943	1120	2739	1934	2238	cyp2j2
ENSXETG00000012641	3308	92	110	109	242	169	440	468	572	358	476	354	397	atf7
ENSXETG00000012642	1590	9	8	11	118	104	19	14	25	18	155	133	119	ENSXETG00000012642
ENSXETG00000012644	1202	164	289	228	216	170	213	402	453	303	199	171	227	tarbp2
ENSXETG00000012648	1361	180	257	206	210	149	161	503	660	359	160	111	101	rexo2
ENSXETG00000012650	2445	1	0	1	9	7	7	15	24	13	16	10	14	map3k12
ENSXETG00000012651	1461	378	515	445	293	216	108	104	131	68	25	34	33	osgin1
ENSXETG00000012652	935	4662	6666	5823	1750	1352	7564	1363	1725	1006	1557	1132	1255	ENSXETG00000012652
ENSXETG00000012653	2394	3	3	1	20	19	11	12	4	5	51	33	26	irx2
ENSXETG00000012654	986	289	436	359	406	285	642	596	686	399	475	353	389	rbm7
ENSXETG00000012655	1833	87	141	110	135	123	153	215	270	186	1018	680	760	ENSXETG00000012655
ENSXETG00000012656	828	0	0	1	245	200	26	2	0	0	659	437	482	vsig2
ENSXETG00000012658	1874	9536	13821	11505	17667	13737	26581	23893	26159	16002	29802	20991	23864	pcbp3
ENSXETG00000012660	2357	151	203	193	573	439	506	621	829	514	780	560	600	sp1
ENSXETG00000012662	876	10	24	24	133	83	17	78	84	59	24	14	20	ccdc14
ENSXETG00000012663	1260	0	0	0	0	0	0	0	0	0	0	0	0	
ENSXETG00000012664	1578	297	376	343	559	429	334	745	856	511	360	251	228	aaas
ENSXETG00000012666	1263	1289	1840	1581	2185	1621	2586	1969	2215	1413	4061	2948	2969	c12orf10
ENSXETG00000012667	723	1364	1995	1706	2085	1621	2980	2991	3344	2158	4177	2916	3248	pfdn5
ENSXETG00000012668	6417	30	48	27	152	119	119	306	308	182	35	21	29	espl1
ENSXETG00000012669	1824	403	565	466	603	440	843	1033	1138	640	628	431	467	mfsd5
ENSXETG00000012670	1329	39	43	25	139	87	152	428	500	276	139	132	131	rarg
ENSXETG00000012671	2286	146	236	180	186	116	277	1511	1808	1063	499	379	400	itgb7
ENSXETG00000012672	1503	408	559	474	368	295	760	111	141	87	158	101	121	csad
ENSXETG00000012675	1401	0	1	0	3	2	2	13	8	11	8	7	10	ENSXETG00000012675
ENSXETG00000012676	735	10	9	6	445	291	203	2783	3294	1874	890	652	743	ENSXETG00000012676
ENSXETG00000012677	726	28	24	28	290	235	293	1639	1953	1107	3141	2183	2517	XB-GENE-5886929
ENSXETG00000012678	1008	0	0	0	1	0	0	0	0	0	0	0	0	ENSXETG00000012678
ENSXETG00000012689	438	102	145	112	68	95	120	240	305	167	165	92	108	XB-GENE-1011896
ENSXETG00000012691	972	0	0	0	0	0	0	0	0	0	0	0	0	
ENSXETG00000012694	582	0	0	0	0	0	0	0	1	0	0	0	1	
ENSXETG00000012696	3567	1	3	3	13	11	17	120	130	89	65	47	40	vat1l
ENSXETG00000012699	897	152	195	173	229	190	264	165	174	116	197	152	150	nit1
ENSXETG00000012700	708	970	1358	1113	770	571	1439	537	656	340	1185	767	905	nudt7
ENSXETG00000012701	3567	0	0	0	0	0	0	0	1	0	0	0	0	
ENSXETG00000012702	1317	652	963	794	732	596	607	1231	1266	780	720	463	542	pfdn2
ENSXETG00000012703	1020	1	0	1	37	46	30	8	4	2	17	11	11	klhdc9
ENSXETG00000012704	1080	0	1	0	3	3	6	27	31	18	4	3	3	mex3a
ENSXETG00000012706	2596	510	785	677	1165	897	1339	1039	1105	704	1807	1285	1321	adam15
ENSXETG00000012709	1974	17	21	22	6	2	18	1	1	0	3	0	2	pde6a
ENSXETG00000012711	2703	300	413	348	501	396	275	269	336	194	619	466	530	ppargc1b
ENSXETG00000012713	720	1	11	3	21	29	27	70	71	33	23	14	23	stk33
ENSXETG00000012714	1926	315	428	393	764	592	974	292	315	189	984	695	819	arhgef37
ENSXETG00000012715	1143	0	1	0	0	1	1	23	16	6	2	4	2	stk32a
ENSXETG00000012716	2203	75	124	109	383	308	326	890	985	601	381	222	288	ENSXETG00000012716
ENSXETG00000012718	2222	75	96	94	549	431	903	3658	4117	2454	1731	1207	1286	dpysl3
ENSXETG00000012724	5955	1	0	2	8	4	25	10	7	7	34	15	21	col11a2p1
ENSXETG00000012726	2463	0	2	0	7	4	4	23	28	16	17	7	7	jakmip2
ENSXETG00000012727	2688	18	29	27	156	139	169	85	91	50	293	172	239	crebrf
ENSXETG00000012728	2834	455	607	516	362	273	526	519	604	351	328	231	229	bnip1
ENSXETG00000012729	1950	0	0	0	10	5	0	0	0	0	57	44	42	nkx2-5
ENSXETG00000012730	2113	158	248	179	3884	2984	3319	1501	1766	1045	2953	2098	2212	stc2
ENSXETG00000012731	2208	207	288	232	699	510	1371	482	539	325	1099	734	812	cpeb4
ENSXETG00000012733	1383	37	62	57	230	204	484	253	288	172	373	268	282	rxra
ENSXETG00000012737	1338	0	0	0	1	1	0	0	3	2	2	0	0	dlg2
ENSXETG00000012738	868	1121	1646	1363	1404	1107	1088	1044	1121	754	819	548	617	tmem126a
ENSXETG00000012740	3950	479	731	592	1113	851	1701	2258	2478	1499	2832	2039	2210	cdc25b
ENSXETG00000012742	1110	0	0	0	1	1	0	8	16	8	3	4	4	ENSXETG00000012742
ENSXETG00000012743	1460	118	157	150	113	104	312	383	459	286	291	168	209	rcor1
ENSXETG00000012746	1686	114	162	150	154	96	207	468	535	300	207	140	143	traf3
ENSXETG00000012747	1484	595	954	831	705	564	692	1462	1606	992	1541	1144	1251	c20orf27
ENSXETG00000012748	2049	37	50	42	302	232	203	404	426	261	186	127	147	hspa12b
ENSXETG00000012750	547	304	479	418	310	264	387	421	468	287	301	260	277	lyrm2
ENSXETG00000012751	1461	469	620	561	759	552	1957	93	113	68	565	350	439	amn
ENSXETG00000012752	1209	1	1	0	1	0	0	0	5	4	11	5	2	ENSXETG00000012752
ENSXETG00000012753	3308	811	1154	1058	945	678	1781	1369	1523	1003	1523	1131	1227	XB-GENE-5921670
ENSXETG00000012756	1869	5	8	10	100	64	75	1356	1425	827	205	137	159	ebf4
ENSXETG00000012759	1272	521	814	641	388	306	343	481	511	318	305	207	225	XB-GENE-5840789
ENSXETG00000012760	672	2071	3096	2635	975	624	878	12	8	8	3	5	3	ENSXETG00000012760
ENSXETG00000012763	2425	7	8	7	17	19	22	114	149	79	59	41	37	pced1a
ENSXETG00000012764	1848	56	52	59	72	57	107	194	222	111	134	111	109	tnfaip2
ENSXETG00000012766	741	2426	3552	3018	2605	1997	5033	4429	5371	2984	2727	1967	2143	eif5

ENSXETG00000012767	2967	375	500	435	811	649	1352	1969	2349	1415	1384	978	1043 mark3
ENSXETG00000012773	1248	433	637	572	919	746	952	975	1086	685	961	692	790 XB-GENE-955295
ENSXETG00000012775	1112	10	30	26	10	7	48	138	172	102	42	35	35 cst7
ENSXETG00000012776	1467	505	714	609	354	300	807	538	654	316	298	207	228 entpd6
ENSXETG00000012777	1926	104	122	128	686	512	381	249	279	150	1231	906	962 acss1
ENSXETG00000012783	3821	15	15	6	73	59	114	139	197	102	360	251	312 emp2
ENSXETG00000012784	732	306	468	344	682	562	967	63	102	45	43	24	36 ENSXETG00000012784
ENSXETG00000012785	11466	5	6	10	36	31	78	55	71	30	67	47	55 bsn
ENSXETG00000012786	1029	0	0	0	0	0	0	0	0	0	0	0	0
ENSXETG00000012788	2780	147	225	198	417	305	513	1281	1421	835	662	518	498 pygm
ENSXETG00000012789	1905	78	97	82	237	198	262	1090	1388	871	435	357	365 abhd12
ENSXETG00000012790	411	228	286	266	230	186	228	253	303	189	131	82	96 uqcc2
ENSXETG00000012793	3239	116	155	159	1014	818	325	623	678	401	2300	1593	1770 sytl2
ENSXETG00000012798	1557	51	55	53	614	447	500	388	423	284	434	375	388 ENSXETG00000012798
ENSXETG00000012800	669	0	0	0	0	0	0	0	0	0	0	0	0
ENSXETG00000012802	2211	2477	3567	3100	6760	5073	8159	4274	4905	2734	6517	4712	5270 rbm47
ENSXETG00000012803	1491	0	0	0	1	0	0	0	0	0	0	0	0 chrna9
ENSXETG00000012806	1397	331	461	459	351	295	768	452	601	314	675	472	560 mettl18
ENSXETG00000012808	5013	9	6	3	73	61	177	151	210	124	214	122	150 pcdh20
ENSXETG00000012810	1191	8	16	18	34	25	48	59	74	29	61	53	70 n4bp2
ENSXETG00000012812	2988	1185	1763	1493	3400	2601	6221	1095	1282	802	3579	2499	2881 klb
ENSXETG00000012815	2238	257	407	324	489	359	494	896	1113	632	423	299	312 tdrd3
ENSXETG00000012819	480	891	1328	1125	1014	776	905	1415	1494	984	552	418	446 bola2
ENSXETG00000012821	2387	1971	2955	2490	2928	2233	3446	1893	2175	1396	3028	2087	2231 sult1a1
ENSXETG00000012823	3960	481	761	621	1148	888	1241	2093	2282	1313	1457	1089	1183 pds5a
ENSXETG00000012824	1569	264	396	347	654	460	478	1618	1879	1123	726	498	560 tbc1d16
ENSXETG00000012825	1423	118	148	108	213	159	329	1226	1423	810	403	287	317 cbx4
ENSXETG00000012826	1832	145	183	151	205	142	313	335	382	230	253	189	225 cbx8
ENSXETG00000012828	1672	399	567	491	710	543	943	1343	1539	914	495	374	395 ttyh2
ENSXETG00000012829	1770	17	27	20	228	184	30	46	43	41	10	7	12 dnai2
ENSXETG00000012830	3087	16	27	20	492	404	180	207	237	140	316	242	255 kif19
ENSXETG00000012832	444	0	0	0	0	0	0	0	0	0	0	0	1
ENSXETG00000012835	4611	7	10	9	18	15	82	485	628	352	139	78	96 abca9
ENSXETG00000012837	768	521	776	649	665	525	1030	1277	1433	900	825	622	683 ube2k
ENSXETG00000012838	1277	226	313	248	319	315	561	357	436	226	368	253	335 smim14
ENSXETG00000012839	1162	730	1043	850	1180	855	1139	824	1000	580	786	476	562 lias
ENSXETG00000012840	1074	227	306	256	574	432	743	825	994	592	1009	687	762 c9orf78
ENSXETG00000012841	957	1	0	0	1	2	6	1	1	1	2	1	0 mc3r
ENSXETG00000012843	1503	629	965	810	738	489	967	574	638	459	525	342	442 tor1b
ENSXETG00000012844	3450	335	473	377	626	485	966	929	1056	705	1218	959	1021 usp20
ENSXETG00000012845	1659	52	72	65	74	46	199	640	734	448	191	157	144 p3h2
ENSXETG00000012846	2919	8767	12877	10772	5321	3936	4937	3600	4137	2413	2811	1967	2300 ugdh
ENSXETG00000012848	6408	97	159	123	325	234	580	1794	2141	1291	946	686	765 fnbp1
ENSXETG00000012849	1340	1002	1500	1178	1023	759	769	2703	3134	1856	1266	833	999 tprg1
ENSXETG00000012850	2250	305	409	371	1059	783	1984	2986	3504	2017	1515	1081	1173 lpp
ENSXETG00000012851	993	19	24	13	2202	1794	113	5	6	4	4771	3255	3596 vsig1
ENSXETG00000012852	339	0	0	0	0	0	0	0	1	0	0	0	0 ENSXETG00000012852
ENSXETG00000012853	681	173	224	175	131	107	83	345	406	265	111	85	88 psmd10
ENSXETG00000012854	2392	183	208	193	313	257	618	894	1008	585	294	191	243 atg4a
ENSXETG00000012856	657	34555	51709	43837	57569	43488	51201	47972	55172	31073	99980	71380	77302 rpl9
ENSXETG00000012858	2224	2440	3561	3108	2267	1897	4184	1729	2137	1266	2100	1475	1685 degs3
ENSXETG00000012859	2811	116	114	121	312	277	355	764	754	456	533	361	419 amot
ENSXETG00000012860	600	0	0	0	0	1	5	1	2	4	9	6	9 lhfpl1
ENSXETG00000012863	1416	4921	7551	6443	10153	8095	10948	14544	16422	10569	15974	11215	12170 set
ENSXETG00000012865	4821	138	230	191	537	377	951	693	787	466	1026	749	808 zer1
ENSXETG00000012866	3874	441	635	568	1325	1071	1467	1524	1744	1055	1685	1163	1401 rfc1
ENSXETG00000012867	4229	91	133	110	263	194	214	309	374	227	381	262	275 pkn3
ENSXETG00000012868	3354	153	204	159	284	223	112	308	350	203	70	48	57 mcm9
ENSXETG00000012869	7353	3567	5248	4528	10658	8374	7997	6133	6544	4052	7252	5198	5614 sptan1
ENSXETG00000012870	4246	17	39	29	188	151	121	234	272	157	80	48	70 wdr19
ENSXETG00000012874	2148	35	49	40	76	58	159	291	298	183	195	121	140 cep85l
ENSXETG00000012876	1074	2	1	1	3	2	3	20	26	10	1	8	7 slc35f1
ENSXETG00000012880	5044	414	604	488	1065	844	2203	1721	2065	1244	1829	1254	1447 gopc
ENSXETG00000012881	810	0	0	0	0	0	0	0	0	0	0	0	1
ENSXETG00000012882	2765	53	61	73	494	375	433	1148	1287	812	1013	711	750 meis2
ENSXETG00000012883	1296	3710	5301	4512	2376	1827	6184	501	625	351	2075	1411	1713 smpdl3b
ENSXETG00000012885	1125	78	102	105	113	85	170	134	188	108	183	132	174 traf3ip2
ENSXETG00000012888	2698	372	512	467	847	681	1293	993	1206	691	1511	1044	1204 eya3
ENSXETG00000012889	2238	98	155	158	165	137	229	252	273	198	133	95	98 wdr34
ENSXETG00000012892	1596	127	178	114	213	134	1030	1227	1475	906	521	380	402 ptafr
ENSXETG00000012893	1685	519	751	616	961	735	959	1020	1155	667	621	427	448 ptges2
ENSXETG00000012895	2358	149	214	201	441	338	455	2147	2387	1428	716	515	592 sh3glb2
ENSXETG00000012896	1836	55	73	64	96	75	83	220	237	164	57	56	60 c15orf41
ENSXETG00000012897	2058	167	233	215	257	183	242	254	316	195	414	281	309 dph6
ENSXETG00000012899	2439	81	132	115	131	118	233	598	789	431	281	203	216 lrrc8a
ENSXETG00000012900	2094	91	114	79	138	127	253	308	396	192	312	216	230 znf770
ENSXETG00000012901	4449	530	739	593	1046	769	768	1130	1294	807	947	627	744 aqr
ENSXETG00000012902	1087	335	516	408	367	294	501	867	1010	633	483	375	423 dnajc8
ENSXETG00000012903	5615	882	1238	1048	1753	1332	1544	1936	2204	1374	2133	1526	1613 nup188
ENSXETG00000012904	3576	1081	1545	1394	4007	3074	3377	5105	5917	3459	3514	2491	2775 pum1
ENSXETG00000012905	2128	4	6	3	21	17	81	44	67	37	80	59	54 tyrp1
ENSXETG00000012908	4120	6	7	7	10	4	7	102	89	80	3	2	10 nkain1
ENSXETG00000012909	1426	1057	1484	1371	1462	1154	2127	1011	1048	719	1773	1229	1415 kyat1
ENSXETG00000012910	1541	649	911	735	730	567	417	1535	1703	962	478	335	363 snrnp40
ENSXETG00000012911	1329	1623	2421	2129	1609	1299	5968	3253	3700	2179	992	684	760 actc1
ENSXETG00000012912	915	0	0	0	0	0	0	0	5	5	1	0	2 gjd2
ENSXETG00000012915	3057	614	940	799	1255	960	1570	1573	1773	1061	1538	1059	1205 golga2
ENSXETG00000012916	781	18348	26151	22458	12045	9178	13047	983	1066	684	551	446	510 fabp3
ENSXETG00000012917	1199	354	516	441	549	381	660	1068	1230	719	604	463	534 zcchc17
ENSXETG00000012918	2518	45	58	43	594	452	383	1798	2237	1239	1483	1047	1194 serinc2
ENSXETG00000012919	1078	553	825	779	1356	1072	2546	992	1197	622	1981	1456	1592 phyhd1
ENSXETG00000012922	1821	40	71	66	108	81	177	154	172	89	199	122	146 pde4c
ENSXETG00000012923	5619	44	63	57	168	121	273	479	569	333	354	244	305 hivep3
ENSXETG00000012924	1527	151	206	210	234	196	578	420	554	270	637	445	479 uri1
ENSXETG00000012928	1062	0	0	0	11	8	5	1	0	1	119	71	72 dnase1
ENSXETG00000012929	2205	77	94	81	174	121	132	319	361	197	110	61	91 ccne1
ENSXETG00000012930	2117	6	12	9	60	32	48	170	174	92	94	90	59 rab3a
ENSXETG00000012932	1107	1	0	2	2	5	4	9	10	4	4	2	4 ecm2.2
ENSXETG00000012933	2148	158	195	163	193	160	203	644	720	442	311	197	245 pik3r2
ENSXETG00000012934	1452	165	238	211	1004	738	1409	8159	9748	5546	1385	962	1109 bgn
ENSXETG00000012935	4278	70	118	82	160	127	423	704	741	476	538	373	435 mast3
ENSXETG00000012937	2574	0	0	0	0	0	0	0	0	0	0	0	0
ENSXETG00000012939	2475	0	0	0	0	0	0	0	0	0	0	0	0
ENSXETG00000012944	1680	0	0	1	2	0	1	1323	1526	889	10	11	3 foxo6
ENSXETG00000012945	2541	0	0	0	0	0	0	0	0	0	0	0	0
ENSXETG00000012949	3615	479	703	608	333	325	321	901	1028	612	424	324	365 ctps1
ENSXETG00000012950	2408	0	0	0	0	0	0	0	0	0	0	0	1
ENSXETG00000012957	2223	0	0	0	0	0	0	0	0	0	0	0	0
ENSXETG00000012958	1176	45	69	48	272	225	644	526	663	378	735	471	609 vwa1
ENSXETG00000012959	2088	2284	3231	2727	1057	881	573	1302	1505	895	571	462	485 atad3a
ENSXETG00000012961	2550	0	0	0	0	0	0	0	0	0	0	0	0

ENSXETG00000012962	1361	188	249	234	134	89	123	283	349	203	107	54	91	gdpgp1
ENSXETG00000012963	1204	913	1330	1186	1269	1006	1449	2051	2443	1318	942	634	733	ssu72
ENSXETG00000012964	1034	6974	9947	8006	3074	2230	6130	5391	6906	3903	3862	2606	3149	cib1
ENSXETG00000012965	1788	358	535	427	838	672	1261	1667	1893	1120	1455	1026	1133	ncbp3
ENSXETG00000012966	2757	654	948	803	2181	1707	2562	3440	3801	2315	2308	1669	1815	scmh1
ENSXETG00000012967	2391	552	782	676	1241	866	1242	1419	1618	1011	1629	1152	1258	sema4b
ENSXETG00000012969	2001	645	917	862	664	537	448	1062	1300	767	531	420	492	rcc1
ENSXETG00000012970	2013	39	42	46	73	67	93	98	131	72	167	117	159	rap1gap2
ENSXETG00000012971	2001	12	26	17	32	35	69	261	288	173	60	48	75	znf710
ENSXETG00000012972	873	282	431	310	249	187	394	494	612	369	171	126	130	ap3s2
ENSXETG00000012973	2898	68760	99407	84413	168008	127850	182201	28161	31981	18742	71638	49838	56665	anpep
ENSXETG00000012975	1950	72	141	89	616	455	420	238	303	165	1009	722	808	tcea3
ENSXETG00000012976	2559	0	0	0	0	0	0	0	0	0	0	0	0	
ENSXETG00000012978	967	25	42	32	85	71	42	63	94	42	11	3	9	cfap161
ENSXETG00000012979	1259	162	223	215	161	120	253	454	456	322	221	152	173	mesdc1
ENSXETG00000012980	1206	670	979	835	448	366	557	854	924	604	635	450	477	mesdc2
ENSXETG00000012981	3987	14	24	14	463	298	1944	706	740	486	673	448	545	cemip
ENSXETG00000012988	2235	2	4	3	25	12	24	293	341	191	55	44	33	arnt2
ENSXETG00000012991	6158	249	396	337	1110	868	910	4263	4679	2989	2119	1479	1609	chd3
ENSXETG00000012994	804	0	0	0	0	0	6	1	3	1	2	2	4	ENSXETG00000012994
ENSXETG00000012995	411	0	0	0	0	0	0	34	39	31	13	21	10	ENSXETG00000012995
ENSXETG00000012997	3114	132	183	155	500	391	438	796	922	545	523	357	400	peli2
ENSXETG00000012998	432	0	0	1	0	1	2	3	7	0	1	1	1	fcer2
ENSXETG00000012999	672	35	66	53	200	145	145	255	304	172	463	336	401	cbs
ENSXETG00000013000	2937	345	508	408	912	686	1207	1191	1421	857	1027	658	767	vps54
ENSXETG00000013007	2029	10669	15609	13743	5892	4284	5817	2490	3026	1680	3070	2260	2450	ugp2
ENSXETG00000013012	3099	85	113	117	216	184	375	460	538	318	372	253	311	morc3
ENSXETG00000013014	3446	121	187	153	531	431	85	599	647	398	120	76	79	chaf1b
ENSXETG00000013016	1137	9159	13310	11662	11798	8772	11319	7340	8562	4939	4776	3299	3700	mdh1
ENSXETG00000013017	2529	128	189	147	370	294	678	461	572	316	794	513	597	ENSXETG00000013017
ENSXETG00000013018	1499	0	1	0	1	0	0	2	4	6	2	1	0	pglyrp2
ENSXETG00000013019	711	0	0	0	0	0	0	0	0	0	0	0	0	
ENSXETG00000013020	2139	16	12	12	49	44	84	89	100	54	54	28	34	wdpcp
ENSXETG00000013022	2295	3	1	2	20	12	6	1	6	2	7	7	5	
ENSXETG00000013023	2536	39	64	63	36	31	81	401	423	251	212	150	164	rasal3
ENSXETG00000013025	1044	0	0	0	1	0	0	0	0	1	0	0	0	
ENSXETG00000013026	3759	414	650	557	1535	1189	1616	3700	4078	2559	2177	1553	1673	wiz
ENSXETG00000013028	2544	81	85	86	101	74	130	101	154	82	144	105	120	hlcs
ENSXETG00000013029	3534	57	113	80	344	305	933	721	834	505	627	372	490	ehbp1
ENSXETG00000013030	1241	53	64	45	142	121	90	78	84	54	190	140	121	ripply3
ENSXETG00000013033	1542	28	39	36	130	89	169	206	195	129	190	128	124	zfp1
ENSXETG00000013034	798	266	382	347	380	294	495	449	488	364	326	223	219	dnajc5g
ENSXETG00000013035	894	290	407	330	618	477	1502	1114	1223	773	1050	692	809	dscr3
ENSXETG00000013036	2271	724	1071	937	1413	1085	1916	2273	2494	1410	2157	1657	1677	akap8
ENSXETG00000013037	2160	58	73	91	124	119	302	391	406	306	352	227	247	dyrk1a
ENSXETG00000013038	1950	64560	90789	79770	97678	75691	159447	116244	138203	83520	257679	175092	202178	hspa8
ENSXETG00000013039	4153	506	805	630	1878	1393	2388	2604	2749	1731	3567	2621	2847	brd4
ENSXETG00000013041	441	1	0	1	50	29	17	4	5	4	399	279	285	LYZ
ENSXETG00000013042	717	159	226	191	233	169	234	274	341	219	288	222	212	rfxank
ENSXETG00000013044	2114	538	779	666	1255	965	1460	1776	1928	1156	1617	1192	1275	sugp1
ENSXETG00000013046	2771	254	353	294	407	307	528	525	649	347	435	291	347	mau2
ENSXETG00000013049	2377	753	1001	848	635	472	936	767	884	563	937	670	810	mfn2
ENSXETG00000013051	1395	3	3	0	19	18	18	20	21	9	79	43	61	ENSXETG00000013051
ENSXETG00000013053	3319	606	884	776	1152	976	1461	3231	3770	2165	1678	1193	1334	gatad2a
ENSXETG00000013055	2409	31	62	48	17	13	28	300	404	219	45	41	50	plod1
ENSXETG00000013056	2142	0	0	0	2	0	2	3	10	5	0	2	2	ENSXETG00000013056
ENSXETG00000013058	11241	51	67	49	130	113	310	485	598	316	399	307	334	lyst
ENSXETG00000013060	765	350	493	407	417	304	382	648	785	458	380	251	295	exosc3
ENSXETG00000013061	693	5	4	4	11	21	32	304	354	212	89	66	60	yjefn3
ENSXETG00000013064	1650	2	0	1	0	2	0	1	0	2	2	0	1	cilp2
ENSXETG00000013065	1710	96	134	99	158	115	159	371	425	219	135	95	96	b3galnt2
ENSXETG00000013066	1404	149	289	224	256	213	422	411	475	325	369	272	324	gtpbp3
ENSXETG00000013067	1534	399	567	471	364	268	577	461	568	336	343	229	285	tbce
ENSXETG00000013068	1168	279	354	283	168	136	168	347	447	265	145	116	125	ggps1
ENSXETG00000013069	1086	1921	2694	2075	1559	1252	1754	1754	2270	1204	942	623	761	erp44
ENSXETG00000013072	720	60	78	71	54	30	64	113	165	92	105	61	78	ENSXETG00000013072
ENSXETG00000013076	1416	4	2	1	6	4	1	16	15	4	1	1	0	ENSXETG00000013076
ENSXETG00000013077	2985	3	3	7	9	12	22	124	163	85	39	39	34	ano8
ENSXETG00000013078	2446	881	1266	1073	1156	910	1731	1674	2209	1270	1098	760	883	kiaa2013
ENSXETG00000013081	393	0	0	0	0	0	1	274	342	190	410	258	332	ENSXETG00000013081
ENSXETG00000013082	708	1	0	0	0	0	1	122	135	92	67	46	47	nppb
ENSXETG00000013083	2604	121	208	160	220	181	425	232	270	167	254	180	211	clcn6
ENSXETG00000013086	2065	1637	2476	2123	1706	1314	1929	492	616	368	518	397	415	mthfr
ENSXETG00000013088	1371	0	0	1	0	0	0	10	10	8	3	4	3	ENSXETG00000013088
ENSXETG00000013089	1303	745	1022	857	574	438	891	1151	1271	838	1314	885	1034	dda1
ENSXETG00000013090	2145	5	10	12	11	2	40	103	122	75	27	22	21	abhd8
ENSXETG00000013094	1687	229	341	262	161	115	896	641	852	447	126	108	108	agtrap
ENSXETG00000013095	3010	282	415	317	508	387	1011	865	1063	592	824	581	653	mad2l2
ENSXETG00000013098	1238	24	39	29	78	64	229	1300	1615	908	286	216	273	qpct
ENSXETG00000013099	1244	88	163	97	104	104	167	213	272	178	111	86	106	lztfl1
ENSXETG00000013102	1425	0	1	1	0	0	0	5	3	3	3	1	1	trabd2a
ENSXETG00000013105	2175	67	106	79	154	114	56	248	294	181	53	38	43	trip13
ENSXETG00000013106	2084	393	523	462	666	517	1242	1688	2157	1203	869	571	627	brd9
ENSXETG00000013107	3489	688	1005	790	1331	989	1723	1740	1917	1145	1516	1034	1142	cdk13
ENSXETG00000013108	897	192	282	251	222	155	295	613	748	411	335	193	256	rala
ENSXETG00000013109	1312	266	366	325	277	246	472	577	600	372	427	306	315	yae1d1
ENSXETG00000013110	2117	0	0	3	5	6	3	12	16	7	1	0	1	tbx20
ENSXETG00000013111	2703	517	727	606	734	592	1394	1306	1570	909	1130	796	944	herpud2
ENSXETG00000013112	2372	1630	2360	1914	2402	1912	2971	9106	10722	6262	3034	2128	2381	sept7
ENSXETG00000013114	705	237	316	286	238	199	235	852	1059	566	351	265	266	eepd1
ENSXETG00000013115	1539	7	10	3	55	49	7	36	51	29	21	20	18	kiaa0895
ENSXETG00000013118	3693	140	223	164	171	155	210	301	331	218	261	197	224	usp33
ENSXETG00000013123	801	201	310	277	258	216	312	623	673	460	421	260	277	ccdc12
ENSXETG00000013130	4105	585	792	761	2079	1610	3290	1995	2125	1386	2781	1842	2063	magi1
ENSXETG00000013131	1582	447	576	543	609	494	416	865	1116	583	1083	770	901	agpat4
ENSXETG00000013132	5606	366	526	453	1165	776	1514	1459	1677	928	1875	1302	1469	scaf8
ENSXETG00000013134	7642	3	10	2	15	7	30	224	337	172	22	18	18	miga1
ENSXETG00000013137	2960	1282	1920	1531	2421	1826	2256	4781	5311	3363	2738	1819	2012	fubp1
ENSXETG00000013144	2696	4	16	10	31	33	144	258	313	168	223	184	185	dnajb4
ENSXETG00000013147	6028	11	25	15	99	67	116	5789	6472	4239	282	223	249	adamts9
ENSXETG00000013148	2424	7185	10239	8569	7693	6174	13091	8346	10356	5782	7890	5733	6271	gipc2
ENSXETG00000013161	648	199	310	260	651	525	1088	782	790	502	1093	735	835	cdip1
ENSXETG00000013164	1754	4056	5974	5136	4171	3210	4220	3918	4308	2752	4620	3283	3464	pklr
ENSXETG00000013165	2271	0	2	0	1	1	20	11	9	5	28	30	19	fign
ENSXETG00000013171	3276	1	0	1	4	3	8	30	48	21	20	17	14	kcnh7
ENSXETG00000013175	2288	2310	3208	2749	2374	1744	2283	2683	3228	1888	1882	1398	1526	gca
ENSXETG00000013176	3033	246	349	269	451	344	734	674	738	411	401	310	345	ifih1
ENSXETG00000013178	1335	1244	1787	1543	1157	872	1594	2642	2993	1711	1175	861	933	gcg
ENSXETG00000013180	594	0	0	0	8	5	6	10	7	5	16	4	12	ENSXETG00000013180
ENSXETG00000013182	2234	27	44	37	10	4	21	10	5	3	2	5	4	ENSXETG00000013182
ENSXETG00000013183	1140	11	15	12	25	14	32	28	33	22	42	26	34	lmx1a

ENSXETG00000013185	4723	280	405	291	1014	758	1107	1430	1581	968	1050	730	783	agl
ENSXETG00000013186	1677	45	83	54	76	52	175	293	374	159	151	87	132	dclre1b
ENSXETG00000013187	1953	64	90	104	102	73	209	185	244	134	150	123	116	ap4b1
ENSXETG00000013189	2253	39	52	36	32	28	97	301	430	246	138	109	112	ptpn22
ENSXETG00000013190	2468	219	339	270	430	301	426	571	692	391	613	461	484	rsbn1
ENSXETG00000013193	1685	14	16	18	75	55	3	6	8	4	25	25	26	slc35a3.1
ENSXETG00000013194	2118	999	1558	1301	993	754	984	1544	1905	1117	1092	779	881	riok2
ENSXETG00000013195	4890	178	273	244	728	484	1015	808	943	559	896	613	763	magi3
ENSXETG00000013196	774	1	3	0	26	19	57	350	464	254	126	129	104	lix1
ENSXETG00000013197	1806	15	17	10	48	31	10	43	44	35	20	14	12	ENSXETG00000013197
ENSXETG00000013199	3755	749	1116	892	1329	968	2471	3785	4605	2544	2733	1901	2264	lnpep
ENSXETG00000013200	2864	574	797	639	923	738	1173	1182	1413	845	853	606	638	mfsd14a
ENSXETG00000013202	2157	68	97	72	287	251	177	506	526	308	83	53	54	sass6.2
ENSXETG00000013203	2925	145	215	162	312	169	487	504	556	339	399	283	339	lrig2
ENSXETG00000013204	2257	515	652	584	345	298	701	455	556	305	304	254	257	trmt13
ENSXETG00000013205	525	376	538	468	693	577	913	380	457	277	480	350	363	ENSXETG00000013205
ENSXETG00000013206	1268	450	643	542	469	348	673	270	295	204	408	324	359	galt
ENSXETG00000013207	1423	207	313	243	429	370	618	630	634	426	479	314	356	gba2
ENSXETG00000013208	2812	0	0	0	1	0	0	0	0	0	0	0	0	lrrc39
ENSXETG00000013210	2472	496	712	577	644	509	868	1209	1332	772	855	575	679	dbt
ENSXETG00000013212	1059	257	351	341	362	314	497	507	565	369	415	301	335	rgp1
ENSXETG00000013213	642	19	52	42	75	54	113	65	92	64	114	67	106	ENSXETG00000013213
ENSXETG00000013214	3771	101	134	111	169	147	425	4560	5577	3164	884	624	686	vash1
ENSXETG00000013215	5016	160	275	225	447	342	676	711	737	432	688	524	586	rictor
ENSXETG00000013217	1395	0	0	0	0	1	0	0	3	1	1	0	2	
ENSXETG00000013218	1353	72	84	84	267	244	421	283	278	180	303	233	261	ift43
ENSXETG00000013220	3492	21	39	37	99	77	56	164	179	101	69	47	58	ttll5
ENSXETG00000013222	2388	24	38	37	265	179	669	1006	1156	721	581	394	458	osmr
ENSXETG00000013223	3198	20	37	24	147	106	154	478	525	360	260	147	197	lifr
ENSXETG00000013224	1760	0	3	1	42	16	5	6	2	1	2	0	0	ccna1
ENSXETG00000013225	2799	379	579	466	738	574	1115	2055	2274	1318	1245	865	985	erap1
ENSXETG00000013227	2240	3	4	1	0	0	25	6	3	2	8	7	4	nkx6-1
ENSXETG00000013228	2949	15	8	10	82	63	276	982	1168	747	300	217	230	egflam
ENSXETG00000013232	920	0	0	0	0	0	0	0	0	0	0	0	0	
ENSXETG00000013233	2731	460	689	587	283	223	477	308	416	225	275	197	198	mlycd
ENSXETG00000013234	1695	100	153	120	415	344	171	187	262	162	133	86	101	cdc14a
ENSXETG00000013235	348	15	21	26	16	5	28	104	141	75	35	24	23	batf
ENSXETG00000013236	1499	8423	12351	10634	7965	6141	8732	9217	10897	6320	7045	5086	5512	XB-GENE-968508
ENSXETG00000013237	2620	167	240	200	35	21	46	42	73	33	0	4	0	top1.2
ENSXETG00000013238	2130	448	705	575	1196	905	6817	399	518	340	561	425	460	fos
ENSXETG00000013239	4063	1982	2792	2420	2259	1745	2980	4079	4818	2745	3097	2133	2550	nek9
ENSXETG00000013240	966	0	0	0	0	0	0	0	1	1	1	0	0	
ENSXETG00000013241	1893	13	15	19	36	32	55	697	815	488	91	62	78	vcam1
ENSXETG00000013246	1401	482	683	609	484	345	599	593	808	496	467	322	336	extl2
ENSXETG00000013249	1083	3	8	2	31	39	26	53	57	23	45	22	34	zc2hc1c
ENSXETG00000013250	703	8	30	29	35	29	32	123	170	104	29	18	17	acyp1
ENSXETG00000013251	1660	36	45	51	51	34	61	263	305	174	42	24	25	nceh1
ENSXETG00000013252	3807	44	69	58	102	106	171	263	326	182	236	156	157	mlh3
ENSXETG00000013255	1552	396	549	491	248	224	357	683	770	459	359	237	286	dph5
ENSXETG00000013257	1561	418	550	486	366	235	506	422	493	287	162	100	101	XB-GENE-5731324
ENSXETG00000013259	1515	0	0	0	2	0	1	0	0	0	0	0	0	
ENSXETG00000013261	1343	1000	1455	1231	1198	899	1577	1413	1614	995	1309	797	941	eif2b2
ENSXETG00000013262	3118	514	748	637	2269	1754	2376	3866	4437	2513	3735	2758	3050	cast
ENSXETG00000013263	1431	5	5	9	20	20	28	68	101	58	43	30	31	rps6kl1
ENSXETG00000013264	1984	5393	7788	6691	7140	5374	11342	5304	6280	3565	9008	6356	7159	dlst
ENSXETG00000013266	1125	9	9	7	34	13	97	111	158	93	126	88	111	s1pr1
ENSXETG00000013268	1449	0	1	0	0	0	0	5	9	3	4	3	1	
ENSXETG00000013269	2001	275	335	322	703	537	506	472	550	339	496	331	393	ylpm1
ENSXETG00000013271	3260	561	809	715	621	492	1367	1317	1531	889	904	577	712	arel1
ENSXETG00000013276	1728	31	51	47	127	83	87	128	137	81	108	89	91	pcsk1
ENSXETG00000013277	2208	0	0	0	5	1	0	4	5	2	4	4	4	GRIK2
ENSXETG00000013282	3777	256	345	305	309	255	541	718	920	464	531	333	383	hace1
ENSXETG00000013283	701	3	3	8	35	53	33	475	517	359	38	27	31	wfdc1
ENSXETG00000013285	2214	539	800	618	1010	766	2625	1542	1937	1098	1729	1238	1379	PTTG1IP
ENSXETG00000013287	2766	574	840	721	1424	1109	1535	1549	1873	1063	1898	1404	1597	atp2c2
ENSXETG00000013293	1016	1	3	1	28	19	38	5	5	4	40	18	23	lin28b
ENSXETG00000013295	802	3579	5091	4373	4977	3759	9718	17308	20172	11746	7972	5478	6152	npc2
ENSXETG00000013296	1893	863	1250	1049	781	630	1858	1101	1246	733	1018	737	837	ell2
ENSXETG00000013301	1668	16	26	25	38	31	108	268	311	203	125	95	109	bves
ENSXETG00000013302	1014	13	18	15	11	7	15	12	20	7	8	7	17	vsx2
ENSXETG00000013304	1572	1446	2175	1854	3534	2789	4380	1870	2093	1309	4296	3189	3418	aldh6a1
ENSXETG00000013305	2048	434	576	501	732	530	1155	1171	1343	799	562	446	493	ENSXETG00000013305
ENSXETG00000013312	1633	528	723	594	450	342	502	978	1146	626	321	260	266	krr1
ENSXETG00000013313	1232	551	860	728	1577	1106	1446	1319	1465	993	881	611	701	arfgap1
ENSXETG00000013314	1731	168	223	204	205	159	277	312	333	232	81	61	95	cdadc1
ENSXETG00000013316	1491	101	160	136	308	230	219	73	106	59	435	264	304	birc7
ENSXETG00000013317	3374	432	667	512	716	533	1186	1506	1886	1107	706	473	593	itgav
ENSXETG00000013318	1077	0	0	1	1	0	1	0	0	0	2	0	1	
ENSXETG00000013321	1991	214	282	267	384	259	612	1223	1458	851	340	258	315	phlda1
ENSXETG00000013322	1389	134	216	177	169	132	367	283	308	170	343	284	283	tldc1
ENSXETG00000013323	2068	5255	7413	6303	7997	6248	11576	12572	15028	8818	16793	11846	13290	nap1l1
ENSXETG00000013325	2296	7565	10608	9085	3243	2478	2800	8037	9563	5274	3390	2405	2604	cotl1
ENSXETG00000013326	1851	105	109	112	180	132	212	286	418	195	294	196	237	klhl36
ENSXETG00000013330	1843	50	100	85	139	111	243	782	873	500	229	136	148	pitx2
ENSXETG00000013333	2883	16009	23095	19823	38593	29751	59021	16644	20022	11224	59065	41487	47048	enpep
ENSXETG00000013334	3368	20	27	27	72	49	89	380	508	334	165	110	139	osbpl8
ENSXETG00000013335	3495	627	913	768	1291	929	3219	999	1126	694	1817	1213	1366	fndc3a
ENSXETG00000013336	1770	568	791	757	844	629	949	1048	1349	743	810	546	614	zc3h15
ENSXETG00000013339	4000	1755	2495	2159	4135	3184	5832	4831	5886	3444	4416	3091	3413	sf3b1
ENSXETG00000013340	1824	9	10	11	64	36	96	209	268	154	65	63	50	egfl6
ENSXETG00000013342	3643	3544	5094	4451	7034	5597	7132	5593	6479	3835	4579	3394	3815	lima1
ENSXETG00000013343	1837	56	63	66	123	97	190	209	293	143	242	155	202	tceanc
ENSXETG00000013344	1176	850	1198	950	2441	1863	3100	916	1100	627	1617	1129	1258	cers5
ENSXETG00000013345	2098	480	712	599	800	582	1860	1177	1453	774	1070	750	789	rab9a
ENSXETG00000013346	1226	17	24	21	94	65	143	80	104	63	78	42	70	ofd1
ENSXETG00000013347	2816	54	86	98	192	128	213	811	940	542	635	451	484	gpm6b
ENSXETG00000013348	2493	5	9	5	21	15	47	723	889	577	110	81	90	slitrk6
ENSXETG00000013349	1063	144	196	145	187	128	232	360	430	236	170	104	150	gemin8
ENSXETG00000013350	1634	3864	5675	4792	5641	4207	4860	6785	7569	4329	3074	2192	2460	tfg
ENSXETG00000013351	1853	100	167	150	163	100	226	191	280	150	176	128	173	tbccd1
ENSXETG00000013352	1860	2509	3581	3188	2397	1847	3736	6089	6919	4255	2782	1939	2143	gpd1
ENSXETG00000013354	2739	2	0	0	6	4	17	17	29	14	19	18	10	slitrk1
ENSXETG00000013355	894	0	0	0	0	0	0	0	0	0	0	0	0	
ENSXETG00000013357	1545	394	600	489	762	509	861	1939	2268	1283	846	616	711	smarcd1
ENSXETG00000013358	4443	24	34	21	125	95	584	232	256	162	546	381	491	abi3bp
ENSXETG00000013359	1095	0	1	0	9	2	4	4	4	0	15	5	10	gpr150
ENSXETG00000013361	3954	0	1	0	2	0	3	2	3	2	0	1	3	impg2
ENSXETG00000013362	3828	81	105	79	78	52	58	337	371	226	45	31	37	elovl6
ENSXETG00000013364	1593	77	142	117	273	202	158	66	70	59	315	228	225	rhobtb3
ENSXETG00000013365	486	113	187	154	172	162	609	43	52	37	247	200	213	rfesd
ENSXETG00000013366	1581	0	1	3	12	4	11	16	9	22	6	9	7	asic1

ENSXETG00000013367	1485	143	182	168	178	119	377	456	534	296	440	343	321 arsk
ENSXETG00000013368	4808	1	2	1	4	4	2	34	35	17	7	6	11 egf
ENSXETG00000013370	4742	369	521	423	724	525	663	830	974	594	923	658	702 ttc37
ENSXETG00000013371	3510	28	53	32	67	41	163	865	976	574	192	129	134 plcb2
ENSXETG00000013373	1095	0	1	1	134	102	23	42	59	35	236	155	186 fam81b
ENSXETG00000013378	1491	75	111	104	73	61	229	50	56	30	97	88	59 ENSXETG00000013378
ENSXETG00000013380	2523	0	2	2	6	7	6	58	74	52	17	14	16 mctp1
ENSXETG00000013383	2544	169	274	206	546	421	411	908	1037	614	105	53	88 racgap1
ENSXETG00000013385	1018	614	914	835	1549	1218	1599	262	321	193	476	323	386 XB-GENE-5780652
ENSXETG00000013387	3177	34	49	39	63	41	159	251	295	164	95	64	70 slf1
ENSXETG00000013389	813	0	0	0	0	0	0	0	0	0	0	0	0
ENSXETG00000013390	2379	1576	2372	2013	1734	1342	1369	2551	3008	1568	2155	1532	1781 usp10
ENSXETG00000013392	3435	14	20	19	39	35	56	126	156	72	59	32	52 ENSXETG00000013392
ENSXETG00000013393	3960	1	3	3	23	19	9	25	36	18	44	24	28 disp2
ENSXETG00000013394	4679	93	131	138	170	123	447	656	849	423	351	260	311 crispld2
ENSXETG00000013395	1965	0	4	6	64	44	0	5	11	2	0	1	1
ENSXETG00000013400	2520	164	221	189	758	641	1781	1445	1493	1018	1093	795	818 plekhm2
ENSXETG00000013401	2247	0	11	2	4	1	3	13	17	20	17	6	7 unc5d
ENSXETG00000013405	2381	10	17	17	182	155	53	77	92	48	24	29	20 mapk15
ENSXETG00000013407	4020	153	227	215	300	199	1279	1019	1214	767	904	629	721 aatk
ENSXETG00000013408	1365	15	15	15	213	172	63	429	434	247	313	177	211 baiap2
ENSXETG00000013409	3026	1817	2524	2169	1889	1444	2490	3432	3983	2269	1582	1092	1273 chmp6
ENSXETG00000013410	5776	517	693	583	855	662	545	998	1137	689	762	515	604 rptor
ENSXETG00000013412	1816	643	910	817	1262	970	1429	1488	1723	1025	1479	1112	1133 poldip3
ENSXETG00000013413	996	1708	2570	2236	1666	1278	1774	438	487	311	537	392	415 serhl2
ENSXETG00000013414	1023	428	551	519	369	269	344	517	622	361	604	378	428 rrp7a
ENSXETG00000013415	963	230	365	312	340	262	663	146	188	106	678	486	569 serhl
ENSXETG00000013417	5686	394	639	546	1101	878	1589	1811	2104	1171	2083	1478	1680 tcf20
ENSXETG00000013418	667	250	333	276	728	520	996	910	1046	674	810	582	592 necap1
ENSXETG00000013420	2693	30	44	54	138	130	195	427	486	313	181	160	157 rasd2
ENSXETG00000013421	4455	112	200	164	1064	767	451	615	701	419	465	323	291 crocc
ENSXETG00000013423	3813	128	219	170	603	459	879	848	963	535	864	609	685 ttc26
ENSXETG00000013424	180	9	16	11	48	30	68	806	907	582	14	6	10 mfap2
ENSXETG00000013426	2114	0	0	0	2	1	1	11	14	6	7	5	11 nyap1
ENSXETG00000013427	2715	86	134	106	270	227	543	866	966	624	561	426	440 atp13a2
ENSXETG00000013430	3210	907	1331	1139	715	554	1585	661	710	429	913	644	739 gucy2c
ENSXETG00000013431	2184	2	3	3	14	4	10	56	82	43	28	21	25 itgb8
ENSXETG00000013435	1109	5079	7734	6652	7661	5865	5987	3701	3901	2595	3652	2595	2827 sdhb
ENSXETG00000013436	1456	43380	62029	53535	73462	57666	109656	129838	141437	90995	124438	86008	96400 ybx1
ENSXETG00000013439	888	512	808	708	762	608	1049	1284	1648	962	1208	817	899 ppih
ENSXETG00000013440	2424	41	59	48	226	225	466	368	478	285	562	433	473 macc1
ENSXETG00000013441	2237	2	8	6	2	4	8	63	67	34	9	8	10 padi2
ENSXETG00000013442	2598	31	37	37	58	50	65	228	298	206	83	50	59 ccdc30
ENSXETG00000013443	1551	411	594	561	106	99	149	272	297	184	248	185	210 twistnb
ENSXETG00000013445	1755	19	26	24	144	96	113	68	75	37	48	30	48 hdac9
ENSXETG00000013448	4330	509	729	620	1117	936	1322	1469	1688	1025	1050	749	812 ddi2
ENSXETG00000013449	528	14	26	23	44	28	10	12	19	21	9	7	10 rsg1
ENSXETG00000013450	1787	108	132	135	153	125	314	710	799	486	267	203	244 hs2st1
ENSXETG00000013461	2546	348	553	479	527	408	924	1000	1182	678	511	380	433 ppcs
ENSXETG00000013462	1494	5	7	2	12	10	16	172	161	98	77	49	48 mmp28
ENSXETG00000013463	1146	24	44	41	97	99	70	59	62	44	30	21	40 zmynd12
ENSXETG00000013464	1253	1	0	0	1	0	0	1	3	1	2	0	0 rimkla
ENSXETG00000013466	2243	69	96	101	142	107	208	390	454	252	290	161	197 foxj3
ENSXETG00000013467	724	7	10	7	11	5	10	24	17	21	25	21	17 efhd2
ENSXETG00000013468	1979	1451	2075	1755	2688	1980	4841	2326	2597	1483	4709	3423	3700 tmem51
ENSXETG00000013469	2019	331	498	451	391	279	418	680	792	462	467	337	388 kazn
ENSXETG00000013476	1647	609	902	725	963	760	944	947	1044	689	1153	811	801 slc52a1
ENSXETG00000013477	1599	1325	1905	1736	3275	2641	4964	3519	3987	2573	3836	2685	2868 taf15
ENSXETG00000013478	3265	0	0	0	78	90	10	2	0	0	8	9	9 gas2l2
ENSXETG00000013480	3898	1602	2243	1994	3231	2483	3986	4069	4800	2772	2671	1911	2141 ap2b1
ENSXETG00000013481	1229	115	223	183	214	151	296	103	148	77	203	165	170 pex12
ENSXETG00000013482	3249	11	19	9	294	228	17	6	10	6	844	602	681 unc45b
ENSXETG00000013484	2089	10165	14540	12000	12348	9483	14718	4060	5121	2834	3519	2407	2931 aldh3a2
ENSXETG00000013488	1537	489	713	614	796	649	861	746	922	488	750	555	639 nle1
ENSXETG00000013489	846	1	2	0	2	1	1	4	2	0	2	0	1 ca4
ENSXETG00000013492	1597	1867	2640	2255	4166	3003	5906	5156	5947	3578	5470	3939	4494 insig1
ENSXETG00000013496	801	0	0	0	0	0	0	0	0	0	0	0	0
ENSXETG00000013497	534	275	366	356	311	268	302	276	359	220	191	123	157 cnpy1
ENSXETG00000013498	1941	91	92	122	268	251	325	334	409	251	604	435	453 rbm33
ENSXETG00000013500	1122	0	1	0	8	3	18	48	60	33	9	6	9 f2rl3
ENSXETG00000013501	3751	122	183	129	322	259	500	740	899	519	530	377	435 sin3b
ENSXETG00000013503	4797	150	256	195	663	461	1123	483	537	325	950	722	821 usp32
ENSXETG00000013504	315	69	91	89	225	171	213	972	1030	709	634	433	498 shh
ENSXETG00000013506	1092	0	2	0	19	15	1	0	0	1	4	2	3 rnf32
ENSXETG00000013509	1773	322	459	345	487	360	780	503	597	352	377	231	286 appbp2
ENSXETG00000013512	1546	49	60	57	234	157	470	951	1116	645	539	373	412 lmbr1
ENSXETG00000013513	1746	299	441	361	320	231	372	331	360	191	259	183	183 use1
ENSXETG00000013514	1632	29	59	48	120	98	152	241	287	183	136	121	99 ppm1d
ENSXETG00000013516	2209	880	1318	1149	2570	1925	5762	2166	2486	1473	2588	1883	2031 pi4k2a
ENSXETG00000013518	3954	36	67	47	243	181	339	355	402	225	590	404	490 insr
ENSXETG00000013519	3555	352	513	417	875	650	1145	889	1126	618	716	510	522 avpi1
ENSXETG00000013520	1421	160	270	206	557	383	625	441	538	304	399	299	322 pyroxd1
ENSXETG00000013522	969	0	0	0	0	0	0	0	0	0	0	0	0
ENSXETG00000013523	972	0	0	0	0	0	0	0	0	0	0	0	0
ENSXETG00000013524	1512	146	232	221	442	358	300	506	551	367	337	209	266 recql
ENSXETG00000013525	527	0	0	0	0	0	3	0	0	0	0	0	1
ENSXETG00000013529	1750	205	274	244	400	320	820	744	837	498	525	383	430 zfyve27
ENSXETG00000013531	1896	243	350	295	1029	782	1216	1207	1426	844	1302	930	1114 nr2f6
ENSXETG00000013534	1368	0	0	0	0	1	0	3	2	2	5	1	2 rtn4rl1
ENSXETG00000013536	3556	3217	4680	3883	8294	6546	13941	10889	12931	7713	12227	8574	9667 MGC147419
ENSXETG00000013538	2979	672	911	847	1822	1380	668	1848	2318	1331	816	505	586 rpa1
ENSXETG00000013539	2129	374	477	457	955	762	1443	857	968	555	1062	709	825 ocel1
ENSXETG00000013541	2732	72	93	86	115	88	39	152	208	123	61	54	51 smyd4
ENSXETG00000013542	4650	0	1	0	6	1	4	6	6	2	7	7	8 kcnn1
ENSXETG00000013543	1413	450	714	575	565	362	604	1215	1294	775	715	491	499 ccdc124
ENSXETG00000013544	2356	105	143	128	211	157	337	1826	2002	1142	337	246	300 serpinf2
ENSXETG00000013545	2598	38	55	42	85	73	278	250	284	181	242	175	200 wdr81
ENSXETG00000013546	2876	2	2	2	4008	3012	0	17	18	6	2364	1628	1881 slc5a5
ENSXETG00000013548	567	0	0	0	0	0	0	0	0	1	3	1	1
ENSXETG00000013549	1704	0	0	0	3	0	0	0	1	0	14	14	17 ENSXETG00000013549
ENSXETG00000013556	969	9	6	6	11	12	30	185	222	113	27	9	15 XB-GENE-1010848
ENSXETG00000013559	528	3	4	8	20	12	22	66	102	53	16	16	21 ENSXETG00000013559
ENSXETG00000013561	711	15	16	13	3	6	37	17	11	11	9	10	13 rgs9bp
ENSXETG00000013562	1612	857	1250	1051	952	828	1973	4123	5174	2919	1829	1283	1383 rap1a
ENSXETG00000013565	3393	0	2	0	4	2	5	75	84	46	57	27	34 pcdh17
ENSXETG00000013567	1456	1	0	1	3	0	8	40	28	15	11	8	10 fam212b
ENSXETG00000013568	2238	298	508	398	460	352	347	683	834	484	438	317	383 ddx20
ENSXETG00000013573	789	875	1182	1013	632	485	615	1119	1339	749	870	614	686 pdcd5
ENSXETG00000013574	7500	0	0	0	0	0	0	0	1	0	0	1	1 ENSXETG00000013574
ENSXETG00000013577	2879	1	1	6	35	31	94	187	254	149	99	75	77 kcnd3
ENSXETG00000013583	1314	1	0	0	0	1	4	1	4	1	6	6	1 XB-GENE-5994086

ENSXETG00000013584	723	68	92	71	21	13	28	4	6	3	16	17	19	ENSXETG00000013584
ENSXETG00000013589	1971	124	183	156	635	496	430	1461	1669	963	655	457	492	tcf3
ENSXETG00000013590	4062	323	428	356	562	420	1054	955	1108	630	480	365	440	plk3
ENSXETG00000013592	2273	848	1221	1018	987	747	2381	663	751	413	803	542	627	best4
ENSXETG00000013593	2173	859	1266	1019	1420	1084	1417	3895	4639	2594	1626	1116	1262	mbd3
ENSXETG00000013594	1668	39	57	52	77	67	111	178	215	127	86	53	77	mex3d
ENSXETG00000013595	717	0	0	0	0	0	0	0	0	0	0	0	0	
ENSXETG00000013596	677	60590	88198	77394	83827	66758	103013	95047	107217	68192	217606	150310	167610	rps8
ENSXETG00000013597	4836	0	6	4	15	7	12	60	80	47	51	23	34	unc13a
ENSXETG00000013598	2196	74	100	81	217	163	242	388	462	301	59	33	33	kif2c
ENSXETG00000013600	1742	577	836	743	811	640	1255	2466	3084	1724	1021	698	763	ap1m1
ENSXETG00000013602	3540	618	851	766	1527	1178	2343	2150	2587	1551	2405	1769	1888	upf1
ENSXETG00000013603	3088	59	74	86	82	84	196	166	252	153	320	194	253	znf507
ENSXETG00000013605	2413	3	1	0	0	1	1	3	2	0	2	1	2	ENSXETG00000013605
ENSXETG00000013606	2919	320	471	380	1295	954	1973	1634	1847	1004	2375	1694	1849	arrdc2
ENSXETG00000013607	2373	3	7	5	22	38	26	285	292	190	59	61	46	AXIN2
ENSXETG00000013608	2748	27	45	37	234	157	93	245	311	183	52	35	39	cep112
ENSXETG00000013609	1794	38	54	25	392	278	815	1364	1509	984	513	338	416	jam2
ENSXETG00000013610	496	7276	9947	8502	4032	2934	3357	4099	4602	2818	2489	1808	1945	atp5j
ENSXETG00000013611	3556	406	603	464	877	696	1059	1328	1593	891	1269	845	1007	gabpa
ENSXETG00000013612	3313	258	392	308	2578	2064	1510	8429	9048	5897	2820	1991	2179	app
ENSXETG00000013630	945	13	13	7	24	28	71	196	229	134	49	29	47	cyyr1
ENSXETG00000013631	4481	210	307	246	520	389	1776	2155	2479	1470	1274	896	1050	adamts1
ENSXETG00000013633	1354	1149	1661	1459	909	696	627	2419	2629	1668	325	276	280	rab30
ENSXETG00000013634	5317	797	1226	1067	2773	2043	2443	2206	2520	1531	3915	2823	3097	pcf11
ENSXETG00000013637	879	0	0	0	0	0	0	0	0	0	0	0	0	
ENSXETG00000013638	2484	2	2	0	31	13	117	309	365	210	178	107	131	adamts5
ENSXETG00000013639	1811	388	587	492	460	321	623	928	1064	627	617	440	466	ccdc90b
ENSXETG00000013641	1176	26	33	30	2	0	1	2	1	1	6	1	2	g6pc
ENSXETG00000013644	1919	216	346	321	268	202	326	566	673	335	346	233	275	aarsd1
ENSXETG00000013646	1050	0	0	0	25	11	38	7	4	11	49	53	43	ENSXETG00000013646
ENSXETG00000013647	447	32353	48908	42600	43789	33502	56106	44142	51797	31469	94222	64608	72438	rpl27
ENSXETG00000013651	2403	546	830	748	738	560	1692	1092	1282	756	897	595	721	tmc6
ENSXETG00000013652	890	0	0	0	0	0	0	1	0	0	0	0	0	
ENSXETG00000013654	1842	0	0	0	0	1	0	0	0	0	0	0	0	
ENSXETG00000013655	901	20598	28855	24226	18367	14111	29344	30695	36174	20958	12706	9124	10195	oaz1
ENSXETG00000013657	1707	22	41	31	23	12	41	215	285	167	113	94	90	TMC8
ENSXETG00000013658	3690	68	105	97	256	146	345	945	1095	694	380	284	297	dot1l
ENSXETG00000013660	1449	31	56	36	19	18	65	39	39	27	149	99	115	ENSXETG00000013660
ENSXETG00000013662	414	1	0	0	0	0	0	0	0	0	0	0	0	ENSXETG00000013662
ENSXETG00000013663	3698	682	926	815	1705	1258	3042	2449	2736	1673	2214	1578	1728	ap3d1
ENSXETG00000013664	3466	419	559	484	491	417	633	884	1304	676	424	297	342	mob3a
ENSXETG00000013665	1242	0	0	0	0	0	0	0	0	0	0	0	0	
ENSXETG00000013666	3225	35	44	54	163	162	309	646	785	457	468	348	348	ENSXETG00000013666
ENSXETG00000013667	1407	450	623	543	593	389	1379	682	832	457	927	682	725	mknk2
ENSXETG00000013668	449	1331	2163	1823	1520	1193	1495	2279	2496	1680	1114	828	900	spcs1
ENSXETG00000013669	1506	643	910	774	841	694	798	1073	1151	719	1207	867	860	gnl3
ENSXETG00000013670	984	32	41	35	130	104	329	289	339	218	239	186	218	lyve1
ENSXETG00000013671	4559	403	615	497	1539	1163	1892	2555	2779	1766	1691	1170	1253	pbrm1
ENSXETG00000013672	3761	704	1108	896	1350	1094	1313	2179	2539	1424	1678	1219	1357	dhx8
ENSXETG00000013673	3617	550	803	748	1925	1509	3540	2231	2448	1607	2789	2128	2135	ctr9
ENSXETG00000013674	3755	4814	7156	6168	12119	9352	16796	30639	35446	20857	10263	7219	8087	eif4g2
ENSXETG00000013675	1707	93	129	94	252	196	388	247	283	158	289	210	231	pank3
ENSXETG00000013676	5226	45	66	71	369	272	284	855	1097	642	411	265	351	slit3
ENSXETG00000013678	1500	0	1	1	7	4	27	6	2	9	66	45	55	NT5DC2
ENSXETG00000013683	1944	988	1485	1261	1230	934	889	1359	1471	923	1417	1073	1040	rrp1b
ENSXETG00000013684	792	262	341	360	140	124	178	23	29	18	63	62	78	hsf2bp
ENSXETG00000013685	1830	216	307	262	219	140	472	584	641	396	339	245	265	sik1
ENSXETG00000013688	1825	360	541	430	645	514	768	626	706	439	1312	890	1023	mbd2
ENSXETG00000013696	5310	1308	1826	1519	1947	1422	3056	3734	4308	2715	2681	1934	2060	usp47
ENSXETG00000013697	627	1289	1860	1653	1406	1032	1853	1160	1412	804	465	374	371	stard6
ENSXETG00000013698	1717	86	144	114	230	202	242	601	641	349	120	93	100	c18orf54
ENSXETG00000013699	432	1018	1449	1226	121	90	688	18382	21822	12689	22	21	50	ENSXETG00000013699
ENSXETG00000013701	2369	102	138	110	498	356	1464	6629	7346	4801	1949	1437	1445	dkk3
ENSXETG00000013702	3381	216	325	281	607	416	1414	1617	1810	1198	1453	1012	1171	mical2
ENSXETG00000013709	711	14	18	21	14	14	32	181	204	106	37	27	29	ENSXETG00000013709
ENSXETG00000013710	2546	4	7	5	37	21	55	443	550	315	85	50	44	mmp16
ENSXETG00000013712	1737	0	0	0	0	0	0	0	0	0	0	0	0	
ENSXETG00000013714	1590	697	1031	846	2492	1789	2232	2792	3096	1791	2263	1573	1787	cpne3
ENSXETG00000013720	1581	2374	3476	3047	3640	2732	5159	5112	5927	3594	4775	3258	3580	eif4a3
ENSXETG00000013722	3397	33	53	26	94	94	126	346	398	268	299	181	210	ephb1
ENSXETG00000013724	1230	519	737	655	984	756	944	885	1027	636	320	237	249	rmdn1
ENSXETG00000013728	1578	920	1262	1121	1952	1426	2587	2914	3516	2079	1979	1441	1593	csnk2a2
ENSXETG00000013733	1230	0	0	0	0	1	0	0	2	1	1	2	0	
ENSXETG00000013734	1276	46	83	53	81	63	160	808	971	560	123	86	77	atp6v0d2
ENSXETG00000013737	1701	324	449	415	632	528	895	550	629	401	626	479	481	atl2
ENSXETG00000013739	1310	2	3	3	104	80	18	182	220	99	48	48	63	ccdc113
ENSXETG00000013740	1979	1488	2150	1722	18661	14548	4239	736	932	546	6311	4455	4868	ca2
ENSXETG00000013741	1163	43	64	67	86	49	20	159	182	102	20	14	5	gins3
ENSXETG00000013742	3821	42	47	50	445	353	2507	4412	5142	3066	1321	980	993	ndrg3
ENSXETG00000013744	1713	50	73	68	136	108	168	232	273	150	190	124	132	znf319
ENSXETG00000013747	853	36	51	39	1195	893	11	0	1	0	1	0	0	ca1
ENSXETG00000013748	774	124	168	139	363	278	293	2684	2980	1993	411	334	325	cfp
ENSXETG00000013749	3408	432	588	501	350	242	384	501	720	380	685	495	513	polr1b
ENSXETG00000013750	2541	1	1	2	2	2	6	6	4	5	2	2	0	cngb1
ENSXETG00000013751	3443	61	79	73	194	144	313	409	520	268	364	265	275	XB-GENE-5823762
ENSXETG00000013755	3030	339	565	438	360	283	465	501	584	355	513	355	400	kifc3
ENSXETG00000013757	891	6	13	7	19	23	19	39	50	38	34	23	18	rem2
ENSXETG00000013759	2870	1	5	3	107	67	15	43	51	33	12	14	12	drc7
ENSXETG00000013760	1691	33	52	36	49	40	72	391	454	252	83	73	65	ttl
ENSXETG00000013762	4137	235	330	259	578	465	627	983	1209	677	963	725	780	zc3h6
ENSXETG00000013764	1164	164	225	174	305	202	622	373	466	248	384	273	289	adgrg1
ENSXETG00000013767	1139	1	0	0	0	0	0	0	9	4	0	0	0	
ENSXETG00000013772	1323	1	4	1	5	5	4	13	14	8	3	2	4	fbln7
ENSXETG00000013774	969	57	108	95	61	39	67	67	75	35	33	29	34	car1_predicted
ENSXETG00000013776	5164	1745	2507	2152	1940	1451	3453	2339	2817	1651	2536	1787	1964	tmem87b
ENSXETG00000013777	393	1	0	0	2	1	2	7	3	3	2	3	3	ENSXETG00000013777
ENSXETG00000013779	1119	41	74	63	98	81	138	342	395	214	129	99	108	ccdc102a
ENSXETG00000013780	3529	734	1098	881	937	763	1114	1540	1770	1010	1957	1462	1587	e2f5
ENSXETG00000013781	2694	69	92	93	201	152	476	1530	1825	989	520	354	396	mertk
ENSXETG00000013782	951	8	15	15	9	16	34	148	185	127	56	55	43	dok4
ENSXETG00000013784	913	479	726	609	484	382	735	1008	1226	679	619	403	463	polr2c
ENSXETG00000013785	3371	90	85	111	249	190	79	242	296	187	98	73	82	lrrcc1
ENSXETG00000013788	1874	1561	2237	1949	1340	1017	1025	1414	1661	865	692	485	534	ciapin1
ENSXETG00000013792	2019	6	8	5	11	12	32	85	94	58	55	30	43	ENSXETG00000013792
ENSXETG00000013793	1611	388	589	537	415	315	367	767	861	524	599	416	456	setd6
ENSXETG00000013794	2442	12	13	13	251	165	51	89	122	66	55	39	29	dzank1
ENSXETG00000013795	1220	250	359	337	377	258	513	416	481	275	538	402	474	usb1
ENSXETG00000013796	1272	35	75	62	74	61	82	122	153	88	107	71	85	kiaa0895l
ENSXETG00000013798	3026	1301	1895	1596	1080	825	1239	1240	1380	744	1182	820	954	kat14
ENSXETG00000013799	2268	7	9	8	58	32	121	343	382	199	183	145	128	exoc3l1

ENSXETG00000013801	828	0	0	0	0	0	0	0	0	0	0	0	0	
ENSXETG00000013803	2183	327	447	378	586	496	720	492	616	305	615	439	546	mgme1
ENSXETG00000013804	1098	14	33	24	42	42	82	115	115	73	110	86	69	ZNF497
ENSXETG00000013806	879	0	0	0	0	0	0	0	0	0	0	0	0	
ENSXETG00000013814	2967	440	587	507	989	735	1490	1800	2097	1267	1579	1108	1251	med24
ENSXETG00000013818	546	0	4	5	2	0	1	0	0	0	0	0	0	ENSXETG00000013818
ENSXETG00000013819	3273	400	539	462	405	310	522	723	918	506	773	482	572	usp1
ENSXETG00000013820	2455	377	505	390	328	288	695	170	219	172	122	114	133	chst10
ENSXETG00000013823	2148	694	1088	885	1696	1284	2074	1692	1975	1102	2475	1764	1996	ELMO3
ENSXETG00000013825	2651	152	236	209	245	201	444	351	385	198	249	189	190	lonrf2
ENSXETG00000013826	555	0	0	0	0	0	0	0	0	0	0	0	0	
ENSXETG00000013827	993	0	3	1	10	8	6	85	100	45	20	13	26	tmem255b
ENSXETG00000013828	2217	513	759	649	1355	1103	1604	2959	3473	1976	1888	1308	1500	gas6
ENSXETG00000013830	2274	137	218	176	291	207	379	40	45	36	255	139	157	plcd4
ENSXETG00000013833	7017	28	45	30	117	91	161	935	1091	630	332	221	250	rasa3
ENSXETG00000013834	1826	6	2	8	40	20	8	6	4	2	14	12	3	c17orf105
ENSXETG00000013835	666	331	387	333	74	52	123	236	346	179	122	103	92	dph3
ENSXETG00000013836	2537	848	1166	1000	1288	1084	1662	2450	3132	1720	1130	794	903	sept10
ENSXETG00000013837	4374	30	43	41	100	67	133	220	211	176	113	106	111	znf142
ENSXETG00000013841	2821	531	704	579	895	620	1023	1350	1495	917	1170	829	976	lemd3
ENSXETG00000013842	1900	0	2	1	3	3	6	162	180	103	34	32	34	wif1
ENSXETG00000013844	1715	296	379	352	189	147	122	336	437	240	130	93	130	bcs1l
ENSXETG00000013846	1969	4841	7005	6141	4451	3326	3024	864	995	608	2047	1403	1651	ENSXETG00000013846
ENSXETG00000013847	4095	168	218	169	499	371	409	666	692	419	432	300	316	yeats2
ENSXETG00000013848	3414	5062	7366	6258	12054	9216	14476	23672	27590	16604	10938	7694	8508	ctnnb1
ENSXETG00000013849	2933	21	24	17	973	712	1905	1090	1489	774	1226	886	1027	klhl24
ENSXETG00000013850	1923	1639	2332	2012	1972	1446	2963	2475	2932	1853	2087	1493	1620	grsf1
ENSXETG00000013852	3468	38	53	36	92	61	92	167	247	114	102	66	74	ulk4
ENSXETG00000013856	1017	0	0	0	3	0	11	0	0	1	12	4	15	wnt6
ENSXETG00000013857	3060	1171	1669	1512	2687	2035	4354	973	1129	690	2786	1976	2132	trak1
ENSXETG00000013859	1551	23	25	27	29	25	52	140	155	105	61	54	42	klhl6
ENSXETG00000013865	594	0	0	0	2	1	0	4	2	2	1	1	3	cryba2
ENSXETG00000013866	1656	9	12	4	44	28	47	36	48	31	17	10	14	arhgef4
ENSXETG00000013867	1213	154	234	173	285	220	303	477	574	337	280	219	197	fam168b
ENSXETG00000013869	5587	2	9	5	195	160	23	38	43	27	26	21	23	cfap65
ENSXETG00000013870	918	0	0	0	0	0	0	1	0	0	0	0	0	
ENSXETG00000013872	1824	328	548	476	400	310	499	706	730	548	379	308	345	atg16l1
ENSXETG00000013873	684	29	47	43	195	125	222	237	276	180	383	251	328	ENSXETG00000013873
ENSXETG00000013874	2200	487	749	595	704	517	1264	2124	2353	1512	900	630	728	ihh
ENSXETG00000013875	3069	86	104	100	101	69	262	1115	1337	738	238	184	201	inpp5d
ENSXETG00000013877	3654	49	57	63	56	56	294	104	152	74	197	148	180	samd9l
ENSXETG00000013878	1413	0	0	0	0	0	0	0	0	0	0	0	0	
ENSXETG00000013879	2437	1	1	0	4	0	1	7	3	3	3	0	4	prkag3
ENSXETG00000013880	5731	4	6	4	7	7	7	32	44	17	8	5	9	reep2
ENSXETG00000013881	1350	621	882	708	586	469	1421	712	931	515	1127	838	895	hepacam2
ENSXETG00000013882	1536	0	0	0	0	1	0	0	1	0	0	0	2	ENSXETG00000013882
ENSXETG00000013885	4203	300	381	323	777	576	1344	831	1000	549	1013	757	850	vps50
ENSXETG00000013886	1713	891	1274	1109	2128	1593	1837	937	958	613	1109	780	850	ngef
ENSXETG00000013887	1969	174	301	237	361	264	360	555	646	394	392	286	347	ttll4
ENSXETG00000013889	1669	158	228	197	651	462	1008	997	1166	708	694	492	579	cnppd1
ENSXETG00000013890	3546	399	603	539	769	664	964	970	1074	697	1086	785	851	GIGYF2
ENSXETG00000013891	2413	0	4	1	0	2	17	30	45	22	4	10	8	kcnj13
ENSXETG00000013892	5566	579	779	702	2915	2251	1582	1121	1360	799	289	196	239	abcb6
ENSXETG00000013894	795	0	0	0	0	0	0	0	0	0	0	0	0	
ENSXETG00000013895	2656	37	43	38	98	100	336	400	479	279	272	195	219	calcrl
ENSXETG00000013896	3365	288	440	370	748	607	1238	1326	1451	920	861	617	721	fam134a
ENSXETG00000013897	1125	0	0	0	0	0	0	1	5	3	3	0	0	asic4
ENSXETG00000013898	960	0	0	0	0	0	0	0	0	0	0	0	0	
ENSXETG00000013900	2038	418	618	516	1294	985	1527	896	1048	708	1208	887	947	tfpi2
ENSXETG00000013901	638	35	46	35	44	54	111	257	296	172	145	82	107	gngt1
ENSXETG00000013902	1721	1306	1917	1684	1979	1501	3622	1054	1188	669	1269	940	956	XB-GENE-5814061
ENSXETG00000013903	927	0	0	0	0	0	0	0	0	0	0	0	0	
ENSXETG00000013907	963	0	0	0	0	0	0	0	0	0	0	0	0	
ENSXETG00000013909	966	0	0	0	0	0	3	0	0	0	0	0	0	
ENSXETG00000013910	5979	0	2	2	0	1	2	1	0	0	3	2	3	obscn
ENSXETG00000013911	2673	1	0	0	17	8	36	36	72	42	15	12	24	DUSP27
ENSXETG00000013914	558	152	223	162	180	125	210	750	824	489	228	160	216	guk1
ENSXETG00000013916	2267	5872	8218	6581	6532	4920	6477	10922	13019	7532	5097	3511	4157	arf5
ENSXETG00000013919	726	0	0	0	0	0	0	0	0	0	0	0	1	ENSXETG00000013919
ENSXETG00000013921	1356	0	0	1	1	0	1	3	0	1	3	1	2	drd1
ENSXETG00000013923	3071	86	165	125	150	128	187	251	260	153	144	100	93	iqcb1
ENSXETG00000013925	2604	4	8	2	165	108	15	36	57	29	36	25	25	cfap58
ENSXETG00000013926	1602	59	102	88	148	104	340	439	474	265	388	293	315	itprip
ENSXETG00000013927	1088	0	0	0	1	0	0	5	4	6	0	0	0	
ENSXETG00000013928	1094	7	11	6	54	55	133	349	420	263	135	81	116	rdx
ENSXETG00000013930	721	1576	2196	1868	1902	1397	1997	1338	1522	881	1246	971	984	fdx1
ENSXETG00000013931	4291	14	26	18	48	24	131	193	223	115	109	72	90	arhgap20
ENSXETG00000013935	579	21	24	27	107	98	122	230	206	134	126	67	85	cenpj
ENSXETG00000013936	564	80	143	118	127	82	148	284	309	187	153	84	108	elp6
ENSXETG00000013938	4644	128	183	150	392	317	597	1435	1728	988	744	536	614	ENSXETG00000013938
ENSXETG00000013948	1431	312	448	396	674	516	438	1214	1393	824	606	432	444	vrk1
ENSXETG00000013949	2302	418	593	471	865	580	1599	2227	2668	1523	1682	1038	1378	papola
ENSXETG00000013950	2328	6	7	4	203	158	20	2	0	0	10	8	13	ak7
ENSXETG00000013951	423	152	254	200	122	93	186	251	329	180	129	93	96	gskip
ENSXETG00000013953	6544	146	165	167	700	518	1152	706	773	450	1047	779	886	atg2b
ENSXETG00000013954	1065	12	13	10	100	63	125	135	143	75	69	53	49	bdkrb2
ENSXETG00000013956	2979	58	84	70	136	112	227	556	616	364	161	96	126	mthfd1l
ENSXETG00000013957	711	3613	5672	4880	1965	1478	2735	4951	5429	3640	2392	1674	1822	ldha
ENSXETG00000013968	9333	244	353	308	714	499	641	1492	1731	989	1019	688	817	trio
ENSXETG00000013971	378	0	0	0	0	0	0	0	0	0	0	0	0	
ENSXETG00000013972	1470	586	868	741	422	337	938	71	97	39	547	395	472	ENSXETG00000013972
ENSXETG00000013975	780	64	91	92	155	100	421	505	534	376	291	220	236	ENSXETG00000013975
ENSXETG00000013983	3096	251	321	284	239	166	283	696	829	491	391	280	349	msantd3
ENSXETG00000013987	867	0	0	0	0	0	1	0	0	0	0	0	0	
ENSXETG00000013994	1410	0	0	0	2	3	9	0	0	2	3	0	0	chrng
ENSXETG00000013995	1748	5446	7825	6790	6824	5055	6809	14403	17435	10124	3864	2770	3058	cct5
ENSXETG00000013999	2500	190	260	231	386	319	316	543	664	391	439	299	360	tle1
ENSXETG00000014000	2001	114	194	171	414	338	692	1090	1262	740	745	593	631	spred2
ENSXETG00000014001	2503	4939	7240	5926	8712	6769	11297	12645	14709	8630	10372	7293	8131	actr2
ENSXETG00000014005	3261	0	0	0	5	1	2	4	1	0	9	4	5	kcnh8
ENSXETG00000014007	875	602	826	767	764	634	1100	1203	1285	807	704	489	564	rab5c
ENSXETG00000014008	2421	78	112	107	388	244	819	624	701	420	680	441	512	kat2b
ENSXETG00000014009	2039	45	73	54	163	105	108	244	299	180	70	59	57	sgo1
ENSXETG00000014011	2609	5174	7417	6284	4453	3539	6099	8138	9429	5605	3266	2296	2693	rab1a
ENSXETG00000014012	2671	144	191	163	292	219	419	498	515	320	317	194	235	galnt1
ENSXETG00000014014	3020	4	1	3	8	5	14	1542	1887	1160	30	16	18	slc1a4
ENSXETG00000014016	1301	352	569	555	871	629	1466	10582	11869	7210	1734	1201	1367	lsp1
ENSXETG00000014017	1144	773	1056	867	474	396	419	734	881	539	429	284	353	mrpl23
ENSXETG00000014018	1797	22	26	22	68	47	72	98	115	62	82	51	51	ENSXETG00000014018
ENSXETG00000014020	891	6	8	7	39	27	105	305	321	214	39	20	28	igf2
ENSXETG00000014022	948	28	43	32	41	25	90	157	190	100	134	70	99	sertad2

ENSXETG00000014023	2853	684	1060	844	1266	969	1862	1518	1965	1065	1635	1200	1384	aftph
ENSXETG00000014029	843	13	10	20	69	35	32	305	399	249	81	46	63	ins
ENSXETG00000014030	1497	0	0	0	0	0	1	24	19	17	7	7	5	th
ENSXETG00000014032	1586	12	26	21	49	23	41	112	121	91	38	20	27	lrrc24
ENSXETG00000014034	996	17	28	22	132	103	232	1279	1372	939	354	274	329	lix1l
ENSXETG00000014041	2708	0	0	0	0	0	0	1	2	0	0	1	0	
ENSXETG00000014055	3555	100	124	114	809	672	1152	1716	1972	1176	1448	1041	1176	arid5b
ENSXETG00000014056	2245	71	85	77	161	118	148	316	338	223	81	67	57	rtkn2
ENSXETG00000014058	2145	0	2	1	0	0	1	6	0	3	5	2	1	shox2
ENSXETG00000014059	1097	629	933	757	757	596	1117	1278	1421	807	1434	966	1126	rsrc1
ENSXETG00000014063	1188	1	2	4	1	0	3	3	9	1	3	2	2	znf365
ENSXETG00000014065	1038	0	0	0	0	0	0	1	0	0	0	0	0	
ENSXETG00000014066	3606	20	26	23	92	54	178	102	111	65	135	107	108	egr2
ENSXETG00000014068	1437	84	106	111	230	195	131	240	268	205	187	118	135	neurod1
ENSXETG00000014070	1344	0	0	0	22	12	3	10	8	7	12	16	17	cerkl
ENSXETG00000014072	3096	19	36	35	111	93	245	832	1037	615	209	154	190	itga4
ENSXETG00000014073	786	234	395	316	602	432	746	1103	1086	760	644	439	482	ado
ENSXETG00000014074	1915	160	258	208	250	215	154	285	317	201	292	229	241	zufsp
ENSXETG00000014075	958	0	0	0	0	0	0	2	3	0	0	1	0	ENSXETG00000014075
ENSXETG00000014077	960	2573	3684	3105	2279	1756	3060	2900	3639	2094	2331	1632	1895	rwdd1
ENSXETG00000014093	2897	0	0	0	0	0	0	0	3	1	0	0	0	dleu7
ENSXETG00000014097	3701	245	350	268	1453	1166	1067	949	1026	631	2197	1567	1802	rarres1
ENSXETG00000014099	2328	307	490	405	602	466	676	1016	1274	708	745	574	594	cwc22
ENSXETG00000014100	399	0	2	0	6	5	5	34	38	15	7	10	11	cxxc4
ENSXETG00000014101	5349	39	65	42	127	81	185	455	549	310	212	163	177	tet2
ENSXETG00000014102	1051	639	898	809	728	620	828	525	608	360	878	641	671	ppa1
ENSXETG00000014106	2292	89	132	130	148	133	288	172	230	117	129	83	114	arhgef38
ENSXETG00000014107	1638	126	189	165	292	224	284	358	424	248	295	211	252	ints12
ENSXETG00000014108	3268	38	70	66	75	59	119	153	173	98	134	100	101	gstcd
ENSXETG00000014109	897	20	36	29	102	87	148	938	960	591	188	153	140	ier5l
ENSXETG00000014111	1818	90	148	114	671	521	692	531	635	384	436	314	315	npnt
ENSXETG00000014113	5298	2	1	0	5	7	11	52	62	34	31	16	29	akap6
ENSXETG00000014114	2220	0	0	0	1	0	1	1	1	1	0	0	0	gcg.2
ENSXETG00000014115	1308	297	421	352	581	440	479	426	511	296	1289	920	1019	son
ENSXETG00000014119	6898	287	402	315	1006	799	1740	1729	1938	1130	2667	1985	2166	dopey2
ENSXETG00000014123	4470	1024	1463	1360	2934	2148	4260	4329	5129	2974	4093	2901	3214	arhgap5
ENSXETG00000014124	3318	1	0	1	18	19	17	40	63	14	55	22	33	slc4a8
ENSXETG00000014129	1415	258	392	347	421	355	744	492	525	302	592	428	478	nubpl
ENSXETG00000014130	1193	3	3	1	11	8	12	195	247	138	36	24	28	ENSXETG00000014130
ENSXETG00000014131	765	512	705	607	545	417	989	723	785	455	659	438	472	dtd2
ENSXETG00000014132	1806	256	369	305	185	160	226	266	312	172	289	173	200	setd4
ENSXETG00000014133	7156	283	392	336	937	753	1447	1212	1487	916	1345	976	1087	heatr5a
ENSXETG00000014140	1434	4	11	8	34	22	42	481	499	349	91	70	71	runx1
ENSXETG00000014144	790	47516	70007	60751	66396	49490	72663	69949	78142	48662	132936	94755	99740	rpl13a
ENSXETG00000014145	1821	0	1	0	0	2	1	0	0	1	0	0	0	
ENSXETG00000014146	512	23752	35380	29918	34376	26419	34615	38917	42709	24109	79444	57244	59371	rps11
ENSXETG00000014148	2997	192	248	216	261	168	131	510	574	352	359	260	305	gart
ENSXETG00000014149	7749	632	1031	900	2846	2195	4036	4197	4738	2948	3834	2734	3032	hectd1
ENSXETG00000014151	1665	155	217	184	180	126	310	743	783	508	96	96	73	fam214b
ENSXETG00000014152	1005	910	1349	1211	2073	1485	3265	561	578	378	1418	1018	1100	marc1
ENSXETG00000014153	2371	170	201	224	648	482	575	410	459	320	587	427	423	fancg
ENSXETG00000014154	470	152	230	200	189	132	196	252	338	196	117	61	79	ap4s1
ENSXETG00000014155	1053	28	53	36	35	21	62	340	372	220	59	42	37	dnajb5
ENSXETG00000014156	2820	570	896	725	1533	1141	2484	3552	3910	2489	2278	1628	1892	strn3
ENSXETG00000014158	2304	0	0	1	10	4	2	7	19	6	2	3	5	nell1
ENSXETG00000014161	1845	80	124	89	107	87	96	111	123	73	103	68	76	galnt6.1
ENSXETG00000014162	2060	184	252	256	192	135	289	520	594	347	212	135	162	ntmt1
ENSXETG00000014163	1659	0	0	0	0	1	0	2	3	1	4	3	5	coch
ENSXETG00000014164	876	0	2	0	0	0	0	0	2	1	0	0	2	ENSXETG00000014164
ENSXETG00000014165	2289	1789	2480	2167	1876	1461	1588	2358	2707	1576	1076	757	832	scfd1
ENSXETG00000014166	2190	205	298	245	616	407	710	709	797	466	693	509	557	asb6
ENSXETG00000014167	2338	0	0	0	0	0	0	0	0	0	0	0	0	patl2
ENSXETG00000014168	1875	6	8	3	12	15	4	10	8	5	5	5	8	galnt6.2
ENSXETG00000014169	768	4	3	6	13	22	43	345	339	269	110	82	75	prrx2
ENSXETG00000014170	1119	74	106	78	143	107	208	154	182	106	319	223	272	rnf144b
ENSXETG00000014171	2394	0	1	0	1	1	0	1	3	1	1	1	1	slc6a5
ENSXETG00000014173	1511	50	69	57	148	122	296	765	950	524	374	291	292	ptges
ENSXETG00000014175	2613	420	690	558	998	751	983	2484	2759	1667	1511	1041	1107	vwa5a
ENSXETG00000014178	1134	134	197	166	195	168	164	706	789	505	68	70	59	cryzl1
ENSXETG00000014180	2070	31	67	47	193	143	194	245	295	190	152	107	160	g2e3
ENSXETG00000014181	2189	416	562	481	733	579	810	1700	1913	1097	1187	787	910	dek
ENSXETG00000014182	5386	204	288	317	757	572	812	2503	2640	1697	818	568	612	itsn1
ENSXETG00000014183	5088	5	20	14	44	39	63	134	148	83	65	62	60	gmpr
ENSXETG00000014184	2328	127	176	150	326	220	936	374	493	254	462	293	371	atxn1
ENSXETG00000014186	1638	287	445	452	410	337	376	534	597	357	511	349	366	prmt3
ENSXETG00000014191	1437	6	8	11	48	18	123	268	339	195	227	138	162	cap2
ENSXETG00000014192	2422	3115	4388	3837	5258	4203	6833	5619	6382	3991	5001	3503	3867	htatip2
ENSXETG00000014193	987	0	0	0	0	0	0	0	0	0	0	0	0	
ENSXETG00000014194	7837	190	233	234	765	579	923	1982	2031	1358	1387	931	1091	nav2
ENSXETG00000014195	810	0	0	2	75	69	50	43	35	16	333	239	253	ENSXETG00000014195
ENSXETG00000014196	891	202	285	232	186	144	379	227	281	157	275	154	205	fam8a1
ENSXETG00000014197	4269	430	631	483	999	725	964	1437	1653	893	1165	834	1007	nup153
ENSXETG00000014198	829	10094	14644	12341	12078	8596	10074	8244	9104	5759	8201	5680	6213	atp5o
ENSXETG00000014200	2313	5	20	8	62	43	51	114	133	58	61	40	33	pou6f1
ENSXETG00000014202	6574	49	75	62	400	345	480	1255	1472	912	1231	889	903	ENSXETG00000014202
ENSXETG00000014203	3654	662	833	753	1393	1011	1706	1031	1183	664	1884	1344	1487	sec31b
ENSXETG00000014204	1072	443	584	506	558	449	888	795	1007	550	720	574	623	hif1an
ENSXETG00000014206	2208	0	0	0	0	0	0	0	1	0	0	0	0	
ENSXETG00000014207	2640	183	229	187	168	115	190	331	381	230	221	159	189	cuedc2
ENSXETG00000014208	2349	0	0	0	0	0	0	0	0	0	0	0	0	
ENSXETG00000014210	1429	477	753	623	214	145	646	357	467	228	185	139	155	fbxl15
ENSXETG00000014211	630	137	211	170	311	269	411	449	483	299	331	246	243	sft2d3
ENSXETG00000014212	297	81	128	129	173	157	231	235	259	170	164	109	123	ftsj1
ENSXETG00000014216	1530	0	0	0	0	0	0	0	0	0	0	0	0	
ENSXETG00000014217	11835	9	10	5	41	16	18	44	57	34	76	65	61	ENSXETG00000014217
ENSXETG00000014218	1995	0	0	0	0	0	0	0	0	0	0	0	0	
ENSXETG00000014220	699	0	0	0	0	0	0	0	0	0	0	0	0	
ENSXETG00000014221	1593	118	187	194	467	381	1069	375	368	279	690	472	522	klhdc7a
ENSXETG00000014224	1144	2	3	2	26	17	24	200	221	151	95	80	60	igsf21
ENSXETG00000014229	1500	7	17	14	9	9	15	43	53	30	26	21	16	ASIC2
ENSXETG00000014231	3415	602	897	735	1860	1407	2429	2672	3053	1849	2996	2169	2359	smarcd2
ENSXETG00000014232	2223	17	22	12	35	21	58	74	91	74	31	34	29	sema3a
ENSXETG00000014233	1152	1	1	0	4	4	19	109	114	69	26	18	17	avpr2
ENSXETG00000014234	1619	2370	3450	3003	3650	2881	3904	6574	7668	4311	2990	2026	2226	psmc5
ENSXETG00000014235	5418	0	1	1	1	1	2	14	23	7	12	11	14	scn4a
ENSXETG00000014236	2024	0	0	0	0	0	0	0	0	0	0	0	0	
ENSXETG00000014240	522	0	0	0	0	1	0	1	0	0	2	2	0	vwc2l
ENSXETG00000014241	2280	44	48	44	140	82	77	228	278	178	68	41	38	bard1
ENSXETG00000014242	3931	118	190	161	290	247	398	350	456	280	656	480	517	mecp2
ENSXETG00000014244	5784	544	763	647	5683	4368	12578	2168	2706	1530	5237	3588	3972	ABCA12
ENSXETG00000014246	2532	127	150	139	215	173	421	542	595	332	439	300	343	irak1

ENSXETG00000014247	669	36	49	47	581	397	125	479	534	307	2742	1936	1999	ENSXETG00000014247
ENSXETG00000014248	645	0	0	0	0	0	0	0	1	0	0	1	0	
ENSXETG00000014250	2967	14163	20132	16903	21098	16380	26526	26061	30246	17718	13802	9786	11198	arf1
ENSXETG00000014251	5904	396	621	540	695	505	940	1990	2207	1313	1239	926	1009	hcfc1
ENSXETG00000014257	1743	68	89	85	72	62	368	908	1049	616	147	125	138	XB-GENE-5919586
ENSXETG00000014258	979	1019	1503	1316	848	649	1000	1201	1448	887	612	483	457	gosr2
ENSXETG00000014260	3982	85	124	121	910	701	384	2576	2769	1717	1463	1046	1112	fam129b
ENSXETG00000014261	3818	35	58	41	206	153	303	577	691	435	498	380	418	stxbp1
ENSXETG00000014263	943	348	461	416	342	250	412	526	651	406	539	404	427	XB-GENE-5809452
ENSXETG00000014264	2616	4	8	4	24	24	27	35	41	26	35	24	23	whrn
ENSXETG00000014265	452	1826	2550	2134	1355	1139	2743	3920	4387	2575	1033	798	836	atp6v1g1
ENSXETG00000014266	1538	1374	1959	1766	2660	2020	2605	3908	4251	2417	1832	1237	1422	psmc4
ENSXETG00000014268	8830	1797	2553	2226	8703	6609	13598	43255	48918	29501	20967	15301	16509	flna
ENSXETG00000014270	816	0	0	0	2	6	1	0	1	0	81	56	57	ENSXETG00000014270
ENSXETG00000014286	1041	0	0	0	0	1	0	0	0	0	0	0	0	
ENSXETG00000014290	1260	3168	4542	4122	5451	4349	5798	12108	13225	8619	6078	4425	4759	ilf2
ENSXETG00000014291	2463	28	33	34	154	143	213	993	1098	654	410	254	298	npr1
ENSXETG00000014292	1041	94	135	136	166	149	214	267	326	198	307	253	271	ints3
ENSXETG00000014293	2154	391	553	514	484	414	508	947	1156	647	446	320	347	eif2b5
ENSXETG00000014294	1473	0	0	0	0	0	0	1	5	0	0	0	0	
ENSXETG00000014295	4421	614	941	758	1566	1265	1698	4105	4943	2913	3609	2511	2766	ABCC5
ENSXETG00000014297	2193	33	58	52	115	60	67	293	326	207	136	98	116	capn10
ENSXETG00000014298	1776	50	88	72	181	135	274	336	389	212	285	169	203	lrch3
ENSXETG00000014299	669	0	0	0	0	0	0	4	1	1	0	0	0	ENSXETG00000014299
ENSXETG00000014314	1407	0	0	0	0	1	2	6	6	2	7	2	3	PNLIPRP3
ENSXETG00000014315	1902	38	46	51	93	89	109	121	192	89	117	86	85	zdhhc17
ENSXETG00000014317	1995	4	3	5	41	34	45	28	29	21	15	15	18	hspa12a
ENSXETG00000014318	664	597	807	669	910	736	1081	1192	1362	739	398	289	301	csrp2
ENSXETG00000014319	1650	1	1	1	89	64	6	27	35	19	6	2	4	eno4
ENSXETG00000014320	2589	43	64	54	228	161	45	302	358	188	46	33	42	e2f7
ENSXETG00000014321	1227	110	197	148	529	408	828	661	715	439	591	401	494	shtn1
ENSXETG00000014323	6690	1	4	5	10	6	35	243	287	182	88	45	56	NAV3
ENSXETG00000014324	1350	0	1	2	3	0	2	7	9	5	7	8	5	ENSXETG00000014324
ENSXETG00000014326	633	0	0	0	0	0	0	0	0	0	0	0	0	
ENSXETG00000014328	4571	0	1	0	0	0	0	2	5	3	1	0	2	
ENSXETG00000014329	1158	2	2	0	2	0	3	1	1	0	3	2	2	ENSXETG00000014329
ENSXETG00000014330	1080	1	3	2	0	0	2	1	1	3	0	0	0	hapln4
ENSXETG00000014331	2195	3630	5338	4527	2224	1743	2142	1985	2323	1345	1254	887	1029	tm6sf2
ENSXETG00000014335	3267	312	443	408	453	322	653	512	581	333	619	403	465	tmem161a
ENSXETG00000014336	2191	143	184	152	259	236	285	103	113	78	295	189	241	slc25a42
ENSXETG00000014337	1032	0	0	0	0	0	0	0	0	0	0	0	0	
ENSXETG00000014338	2283	269	370	306	342	245	582	1089	1271	705	471	316	361	armc6
ENSXETG00000014342	1594	3693	5322	4447	2822	2192	11874	1052	1267	766	3156	2177	2479	hsd11b1l
ENSXETG00000014343	1953	0	3	1	5	2	5	4	9	2	6	2	4	sez6
ENSXETG00000014344	6237	1	2	0	3	2	0	27	31	19	12	7	7	celf5
ENSXETG00000014345	4457	224	337	250	426	308	516	673	778	461	805	565	630	phf12
ENSXETG00000014346	2364	6680	9752	8342	9849	7568	6235	981	1203	671	1524	1082	1185	dhrs13
ENSXETG00000014348	2246	2635	3773	3322	3494	2721	3153	2351	2802	1614	2074	1456	1680	ncln
ENSXETG00000014349	6125	699	995	764	2405	1814	3360	4574	5428	3038	3223	2348	2549	ENSXETG00000014349
ENSXETG00000014351	1137	5	4	8	3	1	2	17	21	17	14	8	11	s1pr4
ENSXETG00000014352	918	11	10	2	29	18	1	0	0	0	16	16	20	ENSXETG00000014352
ENSXETG00000014353	2611	145	209	182	942	692	410	772	879	549	996	758	796	tle2
ENSXETG00000014356	1750	305	406	355	409	353	441	676	711	403	242	157	182	tmem206
ENSXETG00000014357	1485	121	153	160	187	134	277	278	270	204	231	163	175	angel2
ENSXETG00000014360	3117	205	320	295	524	413	737	877	1016	601	662	465	496	RPS6KC1
ENSXETG00000014363	1137	12	48	33	33	32	70	277	343	216	58	36	43	vash2
ENSXETG00000014365	2160	1506	2076	1734	3023	2331	2684	4038	4577	2568	2587	1849	1972	aes
ENSXETG00000014366	1353	467	708	625	345	278	505	346	352	234	84	66	71	flvcr1
ENSXETG00000014367	1555	730	1009	882	967	813	1391	1702	1967	1151	961	698	727	gna11
ENSXETG00000014368	2794	1022	1438	1140	1450	1109	2525	3499	4340	2392	1646	1141	1349	zfand5
ENSXETG00000014369	867	638	917	794	651	559	1160	495	574	298	816	557	632	TATDN3
ENSXETG00000014370	1356	0	1	1	0	0	1	1	1	1	0	0	1	gda
ENSXETG00000014371	1433	689	1032	817	736	504	593	624	757	432	764	527	570	mecr
ENSXETG00000014375	546	67	108	92	137	97	374	1605	1681	1048	196	122	142	atf3
ENSXETG00000014376	786	189	248	205	142	91	226	399	491	293	159	114	138	c9orf85
ENSXETG00000014379	1606	338	534	437	492	384	787	1346	1540	913	435	324	353	nenf
ENSXETG00000014380	2332	619	956	897	1615	1295	951	1940	1953	1345	2453	1739	1912	srsf4
ENSXETG00000014382	2056	504	748	590	967	760	1308	1525	1773	1016	1037	802	891	ppp2r5a
ENSXETG00000014383	843	0	0	1	0	0	0	4	1	2	1	0	1	ca11
ENSXETG00000014385	1249	3091	4522	3837	1279	1020	4105	153	179	120	366	255	339	sult2b1
ENSXETG00000014390	2408	20	45	28	131	82	154	2677	3103	1809	127	110	115	ntn1
ENSXETG00000014391	1767	0	0	0	1	1	1	1	4	0	3	1	0	
ENSXETG00000014393	3801	140	190	162	671	534	439	866	1000	581	598	440	544	stag1
ENSXETG00000014394	2610	108	123	124	335	246	86	261	260	170	81	57	57	dtl
ENSXETG00000014395	1176	3	6	1	15	11	22	153	170	104	13	10	7	XB-GENE-5905096
ENSXETG00000014396	1667	0	0	2	1	0	1	11	12	5	0	2	1	inha
ENSXETG00000014398	2499	1	2	1	15	5	31	23	30	19	28	26	22	myrip
ENSXETG00000014399	765	1	0	1	0	1	2	3	2	3	6	9	6	XB-GENE-5734593
ENSXETG00000014401	2211	176	281	247	428	315	712	685	833	477	668	448	537	l3mbtl3
ENSXETG00000014404	5202	0	0	0	3	2	2	7	2	7	1	0	1	obsl1
ENSXETG00000014405	2070	186	278	223	564	424	715	642	651	447	689	465	505	ap5z1
ENSXETG00000014406	1947	0	0	0	7	2	27	0	0	0	5	4	7	TMC2
ENSXETG00000014407	3997	289	427	425	748	659	1123	2750	3092	1797	1035	765	847	epb41
ENSXETG00000014408	1101	33	35	33	261	153	364	6411	7055	4840	526	371	373	sox4
ENSXETG00000014409	2901	444	673	524	922	722	665	783	845	485	509	385	417	ints7
ENSXETG00000014410	1979	1182	1634	1369	1350	1075	1063	3127	3378	2242	720	495	523	abcf2
ENSXETG00000014411	3804	293	392	377	237	164	169	538	652	291	229	153	171	lars2
ENSXETG00000014413	2103	28	40	40	77	57	134	181	202	129	87	49	70	chpf2
ENSXETG00000014414	984	2	1	0	1	1	5	7	13	9	0	4	4	TMEM198
ENSXETG00000014415	1428	19	16	17	116	89	101	339	399	218	108	81	90	smarcd3
ENSXETG00000014416	1116	164	233	179	210	141	554	666	767	439	212	145	167	lpgat1
ENSXETG00000014418	2806	3497	5133	4472	3271	2439	4446	1025	1147	753	1017	739	736	XB-GENE-5760633
ENSXETG00000014419	3144	383	549	471	1457	1081	2485	2386	2581	1738	2193	1504	1590	hipk2
ENSXETG00000014420	1498	1056	1578	1274	488	351	706	1175	1268	743	488	327	366	yrdc
ENSXETG00000014421	1748	369	492	417	656	472	1664	1184	1432	815	786	530	637	stx12
ENSXETG00000014422	1059	127	185	171	149	118	163	264	282	189	148	113	106	pole4
ENSXETG00000014423	4113	49	77	68	462	365	295	1706	1845	1180	487	347	356	stk10
ENSXETG00000014424	2560	0	0	0	0	0	0	0	1	1	0	0	0	
ENSXETG00000014427	2225	2632	3908	3400	3278	2438	5645	1020	1235	793	2154	1559	1768	slc30a1
ENSXETG00000014428	1120	35	67	51	116	86	103	518	525	319	148	87	101	ubtd2
ENSXETG00000014429	2151	99	139	123	282	210	234	468	532	293	368	235	265	mtf1
ENSXETG00000014433	1044	295	339	331	341	256	284	342	408	273	352	202	259	tbxas1
ENSXETG00000014435	2100	405	565	479	526	363	1070	899	1049	582	606	458	518	inpp5b
ENSXETG00000014437	1704	958	1409	1227	1767	1363	2048	2081	2413	1513	2219	1702	1694	aamp
ENSXETG00000014439	957	1	2	1	4	4	10	38	37	24	19	21	23	gpbar1
ENSXETG00000014441	1071	456	757	573	3526	2609	551	285	313	164	2768	1931	2028	krt8.2
ENSXETG00000014442	594	8	6	14	6	5	8	11	16	9	4	14	3	rd3
ENSXETG00000014444	1704	141	216	197	170	126	151	187	267	139	439	320	327	traf5
ENSXETG00000014445	1351	6	4	3	3	5	13	328	386	272	26	16	13	XB-GENE-5757686
ENSXETG00000014446	3473	585	838	686	659	511	549	697	883	492	411	289	287	snapc1
ENSXETG00000014449	3798	5673	8196	6831	6373	4802	8562	8474	9919	5639	5533	3938	4378	hif1a

ENSXETG00000014450	3328	154	193	181	130	103	127	272	334	197	217	172	220	prkch
ENSXETG00000014455	1493	212	282	237	156	135	135	258	356	177	183	129	152	trmt5
ENSXETG00000014456	714	0	0	0	0	0	0	0	0	0	0	0	0	
ENSXETG00000014458	2046	149	198	174	240	190	464	442	531	307	597	425	494	ddx10
ENSXETG00000014459	2385	152	241	228	257	191	1102	696	859	518	387	279	342	slc38a6
ENSXETG00000014461	861	0	0	0	0	0	0	0	0	0	0	0	0	
ENSXETG00000014462	747	1	2	2	6	3	12	39	51	22	41	28	39	kcnip2
ENSXETG00000014465	2018	84	108	98	122	87	121	1224	1402	802	90	54	62	kdelc2
ENSXETG00000014468	3868	498	860	711	1579	1188	2600	1838	2168	1304	1986	1404	1634	mgea5
ENSXETG00000014469	906	2	2	2	12	17	3	15	17	6	1	0	2	c11orf65
ENSXETG00000014471	9186	272	384	337	469	329	345	862	931	577	457	317	317	atm
ENSXETG00000014472	2227	1273	1862	1537	816	599	670	917	1126	593	633	479	520	npm3
ENSXETG00000014473	705	0	0	0	0	0	0	0	0	0	0	0	0	
ENSXETG00000014474	1882	2	3	4	15	6	12	5	6	4	3	2	1	fgf8
ENSXETG00000014476	4092	213	339	269	508	418	567	764	825	495	655	463	464	npat
ENSXETG00000014477	1408	1373	2112	1796	1886	1378	2763	1215	1444	815	2735	1885	2104	acat1
ENSXETG00000014480	1566	94	103	79	271	200	606	1218	1509	885	549	383	420	tgfbr2
ENSXETG00000014481	2260	120	161	131	434	361	221	317	393	208	341	230	292	gadl1
ENSXETG00000014482	1216	229	293	246	121	79	104	131	126	75	88	59	74	slc35f2
ENSXETG00000014484	1009	0	2	2	3	1	4	27	22	21	12	9	6	elmod1
ENSXETG00000014487	2636	141	223	199	170	115	223	226	288	151	370	239	287	alkbh8
ENSXETG00000014491	927	497	641	550	671	498	874	459	593	342	401	323	348	ENSXETG00000014491
ENSXETG00000014492	3087	722	993	885	3270	2591	3290	6428	7061	4264	3888	2815	3005	smarcc1
ENSXETG00000014497	1659	412	664	607	196	139	1179	410	471	314	1593	1124	1245	slc22a13
ENSXETG00000014498	1461	0	0	0	0	0	2	5	8	5	10	6	5	fgf20
ENSXETG00000014499	2230	1366	1988	1687	2700	2028	3235	3415	3820	2310	2407	1760	1828	stk39
ENSXETG00000014500	1209	4	12	3	31	18	85	229	246	142	108	68	81	micu3
ENSXETG00000014501	1779	0	1	0	2	1	0	1	0	3	1	0	0	slc9a4
ENSXETG00000014502	8397	0	0	0	0	0	0	1	0	1	0	0	1	
ENSXETG00000014504	2702	0	1	2	0	2	0	4	5	2	2	0	0	pou3f4
ENSXETG00000014506	1959	66	91	77	109	90	142	214	273	154	215	161	178	rps6ka6
ENSXETG00000014509	1245	0	0	0	0	0	0	0	0	0	0	0	0	
ENSXETG00000014518	828	1	5	4	9	13	17	8	7	4	11	10	5	ptprq
ENSXETG00000014519	3711	138	160	157	512	371	768	740	884	519	625	419	538	brd1
ENSXETG00000014520	1136	0	0	0	0	0	0	0	0	0	0	0	0	
ENSXETG00000014521	1053	639	946	781	662	473	1346	769	840	536	520	372	396	ENSXETG00000014521
ENSXETG00000014523	2257	48	62	60	150	125	251	292	349	214	240	158	188	hdx
ENSXETG00000014525	1835	2	4	6	7	3	4	13	31	15	40	20	24	lin7a
ENSXETG00000014526	6043	114	183	140	254	192	348	475	628	335	457	336	383	zbed4
ENSXETG00000014527	1086	1392	2050	1743	699	507	317	666	785	477	110	54	72	creld2
ENSXETG00000014528	1881	1057	1427	1214	4116	3198	4511	348	417	179	1513	1065	1218	acss3
ENSXETG00000014529	972	16	25	17	101	83	149	754	868	511	207	126	169	pim3
ENSXETG00000014530	1671	19	23	26	34	52	4	0	3	0	11	8	8	mlc1
ENSXETG00000014532	3450	5	11	3	7	2	7	25	24	8	3	3	8	PPFIA2
ENSXETG00000014534	1636	308	359	284	270	207	422	679	902	471	286	195	189	fgfr1op
ENSXETG00000014538	1452	1619	2429	2022	2686	2132	3661	2442	3097	1812	3763	2533	2963	adss
ENSXETG00000014539	916	235	313	266	224	174	277	503	536	312	341	211	219	ccdc59
ENSXETG00000014540	2235	47	67	63	93	81	129	162	216	130	115	68	76	mettl25
ENSXETG00000014541	2648	163	262	213	279	208	423	406	524	266	397	311	353	zbtb18
ENSXETG00000014544	1679	53	68	50	123	93	142	167	195	106	253	181	205	sdccag8
ENSXETG00000014546	480	1	0	1	2	1	2	3	6	2	2	1	1	ENSXETG00000014546
ENSXETG00000014548	4920	8	9	16	51	43	110	620	793	407	96	53	92	cep170
ENSXETG00000014552	426	0	0	0	0	0	0	0	0	0	0	0	0	
ENSXETG00000014557	1065	1	0	0	0	0	0	11	9	6	6	6	6	XB-GENE-5888675
ENSXETG00000014559	1022	84	109	72	130	107	213	218	196	119	115	72	77	thtpa
ENSXETG00000014560	3504	14	22	13	9	12	25	78	78	43	27	13	27	dgkh
ENSXETG00000014562	2135	1043	1457	1258	768	569	1058	1157	1295	707	1038	747	784	ngdn
ENSXETG00000014563	1354	0	0	0	0	0	0	0	0	0	0	0	0	
ENSXETG00000014566	444	535	819	650	1069	827	1487	1076	1099	680	1063	727	754	tsg101
ENSXETG00000014567	885	0	2	2	0	1	3	13	10	11	17	9	13	ENSXETG00000014567
ENSXETG00000014570	6508	3	12	9	4	2	0	11	11	11	2	1	0	myh6
ENSXETG00000014571	4080	8	9	11	23	17	20	23	32	16	52	36	38	pcnx2
ENSXETG00000014574	2005	0	0	0	104	77	250	1	2	0	37	28	29	f13b
ENSXETG00000014581	6215	300	461	399	667	470	1346	1019	1340	710	913	639	675	akap11
ENSXETG00000014584	555	281	398	322	229	188	270	252	317	205	92	74	71	ntpcr
ENSXETG00000014585	1224	434	644	595	271	219	307	373	529	294	334	262	271	dnajc15
ENSXETG00000014586	1716	85	128	128	281	206	367	619	771	411	387	271	295	enox1
ENSXETG00000014589	1434	922	1369	1157	1353	1002	2164	1605	1640	1005	1531	1073	1203	wdr13
ENSXETG00000014590	2336	3	4	2	113	87	8	4	1	0	11	8	5	CCDC170
ENSXETG00000014592	1755	2	2	2	14	10	24	98	112	65	41	26	20	runx1t1
ENSXETG00000014594	1983	141	191	165	916	740	714	46	73	28	550	391	420	slc26a7
ENSXETG00000014596	1084	773	1046	867	650	445	868	1055	1215	719	982	703	748	otud6b
ENSXETG00000014597	1642	796	1088	984	1339	981	1749	2348	2705	1614	2120	1476	1641	pabpn1
ENSXETG00000014598	1351	0	1	0	3	2	16	51	57	37	14	6	6	tmem55a
ENSXETG00000014599	1856	363	502	371	581	468	693	1141	1301	798	977	738	777	bcl2l2
ENSXETG00000014601	2785	0	0	0	0	0	0	10	6	3	1	1	0	necab1
ENSXETG00000014602	1192	69	111	75	221	173	627	1194	1318	820	553	338	394	twsg2
ENSXETG00000014603	2700	31163	45315	38431	11758	8844	16395	8469	9824	5851	11462	7875	8990	slc7a8
ENSXETG00000014606	549	142	217	155	82	57	242	117	109	92	125	93	98	tmem64
ENSXETG00000014607	1720	23268	33076	27443	29933	22485	13418	1627	2012	1125	3724	2663	3046	calb1
ENSXETG00000014608	2383	21213	31181	26807	20179	15324	40535	7574	8603	4974	19646	13886	15351	pck2
ENSXETG00000014609	1077	485	725	624	877	695	897	654	752	442	677	448	479	decr1
ENSXETG00000014610	2148	3603	5196	4581	3814	2889	7999	3453	4009	2477	4393	3144	3393	dcaf11
ENSXETG00000014611	2324	186	295	225	489	392	672	609	712	437	841	606	658	nbn
ENSXETG00000014612	819	1465	2062	1761	1277	919	1612	3030	3634	2135	1645	1177	1398	psme1
ENSXETG00000014613	1503	165	214	193	224	176	308	177	207	137	604	382	461	ENSXETG00000014613
ENSXETG00000014614	618	41	32	29	24	30	43	65	80	31	19	16	20	ENSXETG00000014614
ENSXETG00000014616	806	1028	1457	1256	1051	773	1053	3484	4133	2315	539	436	505	psme2
ENSXETG00000014617	4315	69	123	112	122	114	119	499	741	403	82	56	82	osgin2
ENSXETG00000014618	3365	477	737	610	1116	883	769	1257	1378	871	1011	668	807	rnf31
ENSXETG00000014619	1455	0	0	0	0	0	0	2	0	7	0	0	0	
ENSXETG00000014620	2108	3	2	3	9	6	5	11	13	9	1	4	3	rec8
ENSXETG00000014621	3772	1081	1568	1385	1002	667	915	1402	1675	989	628	452	514	ipo4
ENSXETG00000014622	1794	571	788	674	860	605	1536	1033	1190	734	908	604	709	tm9sf1
ENSXETG00000014624	1138	1040	1508	1318	1151	824	1312	1208	1397	801	895	641	697	gmpr2
ENSXETG00000014625	3897	37	44	29	264	224	680	3206	3743	2262	993	655	757	nell2
ENSXETG00000014626	2286	86	105	117	403	316	288	1025	1160	681	389	289	337	tgm1
ENSXETG00000014629	4502	518	728	598	675	504	2016	753	938	447	759	482	595	pgpep1
ENSXETG00000014630	1005	1	5	4	2	0	1	0	0	0	3	0	2	
ENSXETG00000014633	1487	177	235	237	182	141	165	247	291	157	125	97	92	olah
ENSXETG00000014634	3577	332	568	424	861	645	1586	1091	1178	722	1374	943	1030	nmt1
ENSXETG00000014636	3233	1303	1725	1553	2020	1598	4942	3565	4136	2395	5729	3919	4589	prps1
ENSXETG00000014637	5595	722	1091	913	2175	1668	2654	3140	3464	2173	2361	1739	1851	zc3h13
ENSXETG00000014638	2178	8094	12182	10541	20967	16057	38048	38864	44347	26431	24493	17665	19272	aplp2
ENSXETG00000014639	3710	51	88	80	120	96	139	522	697	388	161	102	97	palm
ENSXETG00000014640	2682	0	0	0	2	0	5	0	1	0	0	1	0	slc18a1
ENSXETG00000014643	1053	439	612	554	451	275	540	734	864	529	356	275	293	rabggta
ENSXETG00000014644	3448	1979	2736	2317	2775	2273	5731	2141	2692	1616	2568	1950	2162	sptlc1
ENSXETG00000014645	2535	12	23	26	83	62	195	655	778	467	262	189	215	slc4a11
ENSXETG00000014647	2781	517	724	581	388	283	442	702	787	439	398	267	328	siah3
ENSXETG00000014649	6997	705	1092	926	3779	2774	2967	5419	5694	3489	3229	2387	2467	TLN1

ENSXETG00000014651	2475	352	555	447	758	560	1608	888	1095	576	1261	835	961	cog3
ENSXETG00000014652	1179	0	0	0	0	0	0	2	2	1	3	0	0	panx3
ENSXETG00000014653	1542	720	1095	933	1144	897	1963	2234	2698	1623	1466	1064	1146	siae
ENSXETG00000014656	5460	161	245	189	637	432	908	1395	1694	948	1171	811	964	arid2
ENSXETG00000014657	3463	258	363	301	625	474	863	843	1065	624	994	706	781	slc25a30
ENSXETG00000014661	1156	51508	74338	62923	70724	55428	116358	104034	124798	76552	198470	136850	158012	tpt1
ENSXETG00000014662	4320	965	1269	1012	1428	1124	3492	1353	1971	1065	1441	1019	1208	slc38a2
ENSXETG00000014666	1091	180	261	237	421	322	745	908	1096	578	913	573	636	fam92a
ENSXETG00000014667	2678	375	516	507	937	738	1069	806	833	514	1235	935	1009	esrp1
ENSXETG00000014668	5364	458	712	587	1645	1163	1314	1861	2176	1249	1866	1237	1364	kiaa1429
ENSXETG00000014669	1761	0	0	0	1	0	0	19	18	21	1	3	2	trpm4
ENSXETG00000014674	2584	553	681	623	564	427	991	823	1122	609	812	561	645	rpap3
ENSXETG00000014675	915	0	0	0	0	0	0	0	0	0	0	0	0	
ENSXETG00000014677	1200	583	847	651	357	260	849	340	474	229	403	273	297	atp23
ENSXETG00000014678	2742	43	65	73	248	208	330	824	987	591	650	438	510	gem
ENSXETG00000014681	948	0	0	0	0	0	0	0	0	0	0	0	0	
ENSXETG00000014682	3498	19122	27343	22932	25130	19329	33298	28796	34141	19471	19056	13373	15287	cdh17
ENSXETG00000014684	1773	2	4	1	9	8	3	9	17	10	18	14	11	unc80
ENSXETG00000014685	936	0	0	0	0	0	0	0	0	0	0	0	0	
ENSXETG00000014689	2982	30	44	30	166	107	233	235	319	173	185	113	148	tmem67
ENSXETG00000014690	1404	0	0	0	0	0	0	0	0	0	0	0	0	
ENSXETG00000014693	1810	3	6	3	24	14	16	23	21	16	39	27	27	galr1
ENSXETG00000014694	2429	44	80	60	99	58	81	260	340	191	193	114	134	rbm12b
ENSXETG00000014696	2610	213	293	240	351	219	470	548	667	360	604	423	473	rbm12b
ENSXETG00000014704	2211	0	0	0	1	0	0	5	1	3	1	1	1	GRIK3
ENSXETG00000014706	2352	0	0	0	1	1	1	7	7	6	1	2	4	cdh7
ENSXETG00000014707	1324	398	613	501	393	284	426	730	946	503	384	284	288	rnmt
ENSXETG00000014708	1411	3031	4219	3511	1771	1382	1845	1389	1773	958	1389	987	1117	fam210a
ENSXETG00000014711	2764	599	883	827	781	660	848	1281	1540	926	1293	973	1138	nop14
ENSXETG00000014712	684	0	0	0	0	0	0	0	1	0	0	0	0	ENSXETG00000014712
ENSXETG00000014713	573	2321	3371	2979	3191	2400	3482	5851	6707	4000	2141	1623	1645	psmb3
ENSXETG00000014715	2113	163	284	236	538	426	1230	1343	1519	971	670	484	521	grk4
ENSXETG00000014716	1716	19	28	27	55	57	159	421	475	295	204	129	144	tbkbp1
ENSXETG00000014717	4079	2709	3990	3417	4854	3698	4878	7965	9098	5182	5038	3573	3861	kpnb1
ENSXETG00000014718	1482	79	105	80	212	152	229	434	496	314	386	251	289	shb
ENSXETG00000014720	1554	15	14	16	66	70	80	55	84	39	77	55	66	aldh1b1
ENSXETG00000014721	2203	0	0	0	0	1	0	2	4	1	0	0	1	igfbpl1
ENSXETG00000014723	9637	385	567	497	978	748	1513	1546	1757	1053	1524	1160	1327	htt
ENSXETG00000014724	2529	150	253	203	278	206	345	401	490	299	240	199	210	topors
ENSXETG00000014725	3668	1685	2591	2286	4820	3507	4363	3819	4276	2629	4212	3067	3250	npepps
ENSXETG00000014726	549	1651	2342	2004	2082	1624	1691	2100	2506	1447	1219	851	902	ndufb6
ENSXETG00000014727	660	0	0	0	0	0	0	3	8	4	0	0	0	
ENSXETG00000014729	2496	1	1	0	1	3	0	3	2	5	2	2	1	ENSXETG00000014729
ENSXETG00000014730	1001	4590	6696	5746	6433	4905	5341	2350	2831	1593	3347	2391	2606	ltb4dh
ENSXETG00000014731	1075	525	712	614	511	320	473	657	716	448	280	219	220	mrpl45
ENSXETG00000014736	588	0	0	0	0	0	0	0	0	0	0	0	0	ENSXETG00000014736
ENSXETG00000014740	4218	9	24	15	137	103	208	793	942	550	265	168	180	arhgap23
ENSXETG00000014743	4778	136	184	147	501	356	596	313	365	242	1028	754	816	srcin1
ENSXETG00000014744	3335	330	480	369	576	456	889	709	826	478	1069	765	818	mllt6
ENSXETG00000014746	1056	40	71	49	157	112	196	417	475	287	221	147	179	pcgf2
ENSXETG00000014749	696	552	939	742	697	548	550	981	1049	642	416	279	306	mrpl10
ENSXETG00000014750	2190	74	128	100	97	85	181	604	707	408	247	173	197	tfdp2
ENSXETG00000014751	1028	152	215	189	252	205	430	1854	2381	1211	474	352	374	atp1b3
ENSXETG00000014752	2594	0	0	0	27	26	43	9	12	7	77	53	45	grk7
ENSXETG00000014753	578	892	1340	1168	1067	858	1352	1503	1722	999	1325	960	964	rnf7
ENSXETG00000014755	6098	301	441	348	600	483	867	1613	1935	1153	1077	771	934	rasa2
ENSXETG00000014756	3297	45	47	57	96	94	185	241	332	137	275	198	242	zbtb38
ENSXETG00000014757	6212	758	1107	985	2672	2154	4536	4605	5128	3205	4117	2963	3399	trip12
ENSXETG00000014758	2865	3	5	3	38	39	28	93	101	60	94	59	81	dner
ENSXETG00000014759	654	31	42	35	102	78	168	412	433	251	141	127	139	pid1
ENSXETG00000014760	972	395	539	473	534	357	624	930	1050	611	485	341	406	mff
ENSXETG00000014768	2401	1111	1604	1384	2930	2132	5513	3509	3999	2463	7147	5168	5609	agfg1
ENSXETG00000014770	2231	6993	10250	8887	3914	3126	7810	2250	2569	1461	2070	1505	1704	tm4sf20
ENSXETG00000014771	1239	1129	1686	1481	1182	921	1585	1131	1338	791	1197	884	965	mrpl44
ENSXETG00000014772	1335	618	963	850	1313	962	2858	240	244	139	1678	1139	1262	ENSXETG00000014772
ENSXETG00000014773	3455	0	0	1	0	0	0	0	0	2	2	0	1	ENSXETG00000014773
ENSXETG00000014774	810	0	1	1	0	0	0	0	1	0	1	0	0	
ENSXETG00000014775	1332	96	147	121	89	66	129	444	555	295	167	86	108	lacc1
ENSXETG00000014776	834	24	32	29	25	10	60	205	257	141	33	36	41	tsc22d1
ENSXETG00000014777	1179	0	0	0	0	0	2	1	0	0	0	0	0	
ENSXETG00000014778	2119	387	542	501	425	319	552	558	652	398	677	475	515	nufip1
ENSXETG00000014779	2755	549	820	735	984	747	1590	1155	1301	752	1205	868	995	gpalpp1
ENSXETG00000014780	435	98	149	133	151	120	233	580	654	408	159	121	132	ENSXETG00000014780
ENSXETG00000014782	1731	0	0	1	6	6	31	65	75	46	49	29	45	adgra1
ENSXETG00000014784	4969	1	0	1	27	29	1	1	0	1	2	3	5	cfap46
ENSXETG00000014791	1634	278	415	333	595	482	1078	311	335	168	946	703	718	PRR15
ENSXETG00000014793	1359	264	447	421	655	507	489	2912	2977	2055	515	358	372	rcc2
ENSXETG00000014794	3327	117	174	168	699	534	1159	891	1003	616	1397	1027	1099	arhgef10l
ENSXETG00000014797	1056	18	36	26	23	13	10	19	25	13	8	7	4	mterf2
ENSXETG00000014798	1218	0	0	0	0	1	0	0	0	1	1	0	0	
ENSXETG00000014799	1099	308	444	424	811	627	1075	1446	1493	963	1237	959	933	nfkbie
ENSXETG00000014800	1825	6	4	6	6	8	18	83	84	49	19	20	14	sirt3.2
ENSXETG00000014801	1479	163	237	223	229	205	414	401	471	239	282	197	223	ric8b
ENSXETG00000014805	1563	26	37	23	108	81	250	151	186	114	257	149	185	ENSXETG00000014805
ENSXETG00000014807	560	176	244	200	125	104	122	272	361	212	99	58	88	txnl4a
ENSXETG00000014810	3381	108	147	112	209	171	280	416	530	279	300	198	254	adnp2
ENSXETG00000014811	3149	55	85	67	189	157	376	104	134	53	310	198	257	rnf180
ENSXETG00000014812	1167	0	0	1	0	0	0	0	0	0	1	0	1	
ENSXETG00000014813	2340	1691	2498	2065	2196	1747	4239	2723	3350	1960	2490	1763	1931	c6orf62
ENSXETG00000014814	1032	533	794	654	1259	1010	538	542	632	392	553	359	459	gmnn
ENSXETG00000014815	7241	83	103	82	109	121	244	340	446	220	147	118	100	fam65b
ENSXETG00000014817	1902	7	18	14	46	24	104	32	40	22	59	39	70	cmahp
ENSXETG00000014820	4241	553	764	646	1271	985	1667	2025	2392	1437	1430	1051	1140	carmil1
ENSXETG00000014823	3279	461	652	576	459	353	603	959	1172	643	481	298	424	ipo11
ENSXETG00000014824	890	190	285	230	942	687	652	589	743	426	814	542	597	scgn
ENSXETG00000014825	1155	10	14	12	20	43	73	258	344	171	83	63	59	pard6g
ENSXETG00000014826	3504	110	144	106	648	510	1168	189	210	157	391	305	356	tdp2
ENSXETG00000014827	2949	0	0	2	16	14	7	9	7	4	20	13	8	kiaa0319
ENSXETG00000014830	2313	519	747	638	364	267	477	457	559	327	606	465	437	dimt1
ENSXETG00000014831	2766	277	455	365	494	372	908	929	1045	648	578	381	431	kif2a
ENSXETG00000014834	4311	5	7	12	142	104	244	1230	1481	913	575	412	449	ptch1
ENSXETG00000014835	3509	89	112	97	152	96	171	260	298	183	122	75	94	ercc6l2
ENSXETG00000014837	915	0	0	0	0	1	0	1	8	4	0	0	0	hsd17b3
ENSXETG00000014838	1807	235	347	289	382	310	482	309	354	183	489	357	392	fam175a
ENSXETG00000014839	1353	65	104	98	573	403	663	204	226	156	598	417	550	cdc14b
ENSXETG00000014840	1005	64	99	99	132	93	22	219	242	147	49	35	35	znf367
ENSXETG00000014841	1143	398	609	504	664	551	481	615	685	377	132	105	100	habp4
ENSXETG00000014845	960	0	0	0	0	0	0	0	0	0	0	0	0	
ENSXETG00000014846	1999	4	7	8	114	60	96	144	172	109	162	93	128	or51e1
ENSXETG00000014847	3205	330	502	420	622	424	1623	1212	1436	832	1292	963	1051	foxo3
ENSXETG00000014850	3456	0	0	0	0	0	0	0	0	0	0	0	0	

ENSXETG00000014851	1188	0	0	0	0	0	0	0	0	0	0	0	0	
ENSXETG00000014853	1155	0	0	0	1	0	0	0	0	0	0	0	0	
ENSXETG00000014854	2379	0	0	0	0	0	0	0	0	0	0	0	0	
ENSXETG00000014857	3127	149	197	176	487	345	796	1071	1432	710	881	570	768	ostm1
ENSXETG00000014861	1998	177	289	243	359	294	189	708	840	529	218	144	164	chek1
ENSXETG00000014863	2184	3133	4612	4040	4407	3333	6384	5439	6314	3632	4145	2826	3279	sec63
ENSXETG00000014864	1215	6	21	12	23	19	35	223	253	138	96	72	67	scml4
ENSXETG00000014865	705	0	1	0	6	1	5	1	2	1	4	1	1	ENSXETG00000014865
ENSXETG00000014866	3147	364	507	405	1003	751	1115	1568	1713	1101	1516	1084	1188	pcif1
ENSXETG00000014867	1524	49	50	34	163	143	276	10923	12538	7261	1361	1031	1154	pltp
ENSXETG00000014869	687	12	10	10	42	26	41	239	255	161	77	57	53	efna5
ENSXETG00000014870	2005	5690	8133	6949	6082	4630	15933	12281	14362	8754	9463	6802	7803	ctsa
ENSXETG00000014872	798	0	0	0	0	0	0	1	0	1	0	0	0	neurl2
ENSXETG00000014873	963	1770	2584	2243	1822	1383	3040	1303	1450	907	964	673	680	acot8
ENSXETG00000014875	1695	7819	11389	9718	12330	9387	19878	11308	12463	7491	15058	10872	11754	hnmt
ENSXETG00000014877	1162	1	4	3	7	4	7	43	46	26	74	67	56	tnnc2
ENSXETG00000014878	4689	0	0	0	5	8	2	13	7	3	4	4	2	thsd7b
ENSXETG00000014880	1239	11	15	15	24	18	46	205	194	150	40	37	41	XB-GENE-5936529
ENSXETG00000014881	787	316	424	328	484	381	489	1454	1611	885	129	72	105	ube2c
ENSXETG00000014883	2826	1369	2017	1756	3872	3026	6358	2757	3310	1940	4896	3535	3833	nbr1
ENSXETG00000014890	2199	36	44	42	47	27	143	642	804	476	212	165	169	cxcr4
ENSXETG00000014891	2298	566	873	803	1459	1197	191	1558	1783	1098	463	312	365	mcm6.2
ENSXETG00000014892	3720	0	0	0	0	0	1	1	0	0	0	0	1	ENSXETG00000014892
ENSXETG00000014893	2751	444	670	546	1699	1252	3210	2090	2307	1412	1604	1213	1314	fam134c
ENSXETG00000014898	1353	321	461	342	783	573	914	1334	1601	939	1020	706	779	tubg1
ENSXETG00000014900	1374	65	106	96	339	233	449	2009	2189	1460	360	247	259	vat1
ENSXETG00000014901	2433	0	0	0	0	0	0	0	0	0	0	1	0	
ENSXETG00000014902	1770	883	1254	1142	4046	3044	3498	1019	1205	779	2425	1692	1866	ahcyl2
ENSXETG00000014904	864	198	282	251	272	192	455	880	1060	585	203	149	183	ifi35
ENSXETG00000014905	2580	40	67	54	181	130	142	145	167	77	108	76	72	lama2
ENSXETG00000014908	999	8	10	10	8	4	57	94	132	73	11	12	9	ENSXETG00000014908
ENSXETG00000014909	1915	826	1169	1010	1183	889	2658	2607	3101	1796	1484	1096	1178	arhgap18
ENSXETG00000014910	2602	580	798	701	865	662	1670	1548	1727	964	1189	848	970	dnttip1
ENSXETG00000014911	1842	0	1	0	9	2	6	9	12	7	9	16	6	clgn
ENSXETG00000014912	1467	6	5	5	31	12	34	49	58	32	41	26	30	tmem200a
ENSXETG00000014915	2236	705	995	815	979	736	1286	379	469	290	494	385	403	mgat4a
ENSXETG00000014916	1132	53	104	71	567	418	775	1280	1370	867	1083	754	849	tmbim1
ENSXETG00000014925	2577	390	495	474	381	317	676	624	726	399	265	216	210	elmod2
ENSXETG00000014927	1771	8286	12110	10351	16452	12429	26045	59	60	38	17691	12174	13730	ucp1
ENSXETG00000014931	3125	694	1017	904	2393	1838	4451	1750	2030	1142	2254	1548	1858	pnkd
ENSXETG00000014933	7463	62	75	78	417	290	192	142	180	96	265	173	179	tbc1d9
ENSXETG00000014934	1056	6	7	7	155	115	20	32	39	24	13	10	14	catip
ENSXETG00000014935	564	650	942	805	997	759	1186	1669	1879	1227	1350	948	1006	ralb
ENSXETG00000014937	1641	75	121	104	163	138	337	2053	2250	1367	142	107	106	slc11a1
ENSXETG00000014939	2451	9	32	24	130	74	235	1047	1250	800	257	212	270	smo
ENSXETG00000014940	876	221	391	297	566	443	896	977	1085	737	767	557	529	ccdc93
ENSXETG00000014941	663	109	118	128	138	113	275	282	301	222	267	181	189	insig2
ENSXETG00000014943	1245	96	115	105	182	155	117	236	265	171	157	151	131	wrap53
ENSXETG00000014947	855	15	23	26	37	34	24	124	134	74	63	43	50	tspan33
ENSXETG00000014949	987	1752	2655	2296	2091	1623	1579	362	394	287	416	277	319	rdh11
ENSXETG00000014950	6734	5	3	4	16	12	35	262	352	193	42	27	29	ccdc136
ENSXETG00000014951	732	45	68	69	50	37	92	176	154	149	64	43	57	ENSXETG00000014951
ENSXETG00000014953	2979	1	3	0	6	0	6	57	70	46	4	2	5	zpy1
ENSXETG00000014954	1887	285	470	400	387	304	1453	5984	6882	4130	1103	838	939	lcp1
ENSXETG00000014955	945	750	1150	1025	1100	907	1098	1448	1577	1068	747	490	597	rangrf
ENSXETG00000014956	8193	70	89	69	387	270	878	1352	1545	914	1427	999	1078	flnc
ENSXETG00000014957	1542	12	15	18	1	1	37	69	79	58	53	22	28	chrne
ENSXETG00000014962	1652	3211	4736	4098	5758	4333	9452	4749	5495	3358	8118	5683	6247	nop56
ENSXETG00000014963	1564	308	433	383	992	680	1323	1242	1356	834	2000	1398	1605	ctdsp1
ENSXETG00000014965	2547	47983	68422	59622	78566	60050	66435	11885	12910	7925	40373	28201	31249	vil1
ENSXETG00000014969	1213	828	1377	1137	1688	1242	2027	1558	1806	1135	2610	1835	1922	idh3b
ENSXETG00000014970	1458	612	867	816	635	480	816	1120	1287	767	767	493	582	znf330
ENSXETG00000014976	1332	101	147	137	126	98	210	503	623	338	270	180	209	slc39a6
ENSXETG00000014977	3696	212	324	263	357	325	335	606	760	400	571	452	522	usp37
ENSXETG00000014979	1077	0	1	4	1	3	4	1	0	2	0	1	0	murc
ENSXETG00000014980	1839	11	12	10	0	1	15	46	52	26	12	19	15	ENSXETG00000014980
ENSXETG00000014981	13860	8	5	3	107	78	11	23	29	20	26	22	19	dnah8
ENSXETG00000014982	1197	11	22	13	14	4	29	36	32	17	28	15	22	ENSXETG00000014982
ENSXETG00000014983	969	9170	13431	11485	12641	9583	14598	4718	5226	3283	10392	7077	7918	ENSXETG00000014983
ENSXETG00000014984	1847	880	1200	1040	1632	1180	2359	2450	2829	1714	2119	1553	1781	cnot9
ENSXETG00000014986	959	246	393	329	275	203	525	652	674	420	465	352	368	hmox2
ENSXETG00000014990	3011	440	595	536	799	520	693	1350	1428	842	630	455	512	atxn2l
ENSXETG00000014992	948	844	1226	1029	887	602	766	1234	1352	840	677	537	518	eif4e2
ENSXETG00000014993	7070	17	34	18	333	273	441	805	888	593	171	97	140	kcp
ENSXETG00000014994	534	0	2	3	3	3	6	35	54	36	5	5	4	en1
ENSXETG00000014997	3402	180	261	232	436	327	1241	693	808	487	661	468	519	nfx1
ENSXETG00000015002	3872	0	1	0	0	0	0	1	2	2	0	0	0	MGC69493
ENSXETG00000015003	2145	23	23	21	45	52	102	755	826	496	128	102	99	chn1
ENSXETG00000015004	1737	24	25	23	17	13	57	70	97	60	95	67	75	znf717
ENSXETG00000015005	1906	387	559	479	616	478	1292	993	1103	697	1097	811	889	irf5
ENSXETG00000015007	1473	424	538	453	790	636	1236	1375	1540	944	851	562	695	rbm45
ENSXETG00000015009	2775	989	1389	1186	2299	1801	3952	3592	4003	2278	3878	2785	3085	tnpo3
ENSXETG00000015011	3310	18	20	22	74	61	141	390	430	240	159	116	151	OSBPL6
ENSXETG00000015018	1495	29	36	25	49	41	47	1576	1832	1055	86	64	77	fkbp7
ENSXETG00000015020	1290	169	258	199	205	144	281	341	360	237	214	150	188	plekha3
ENSXETG00000015021	94440	0	0	0	0	0	1	1	0	0	0	0	1	ENSXETG00000015021
ENSXETG00000015022	1937	561	899	699	794	642	821	2163	2614	1474	770	529	618	gpd1l
ENSXETG00000015025	2733	3	8	5	8	2	4	81	84	49	17	7	13	susd4
ENSXETG00000015027	2607	35	54	29	219	149	367	623	752	414	316	241	247	tlr5
ENSXETG00000015028	7455	249	348	306	1608	1198	2277	1990	2227	1330	3125	2168	2388	bahcc1
ENSXETG00000015029	3606	115	169	144	726	539	977	1194	1443	847	1907	1365	1529	disp1
ENSXETG00000015030	2474	1	1	2	0	2	1	14	18	12	6	7	4	fscn2
ENSXETG00000015033	3115	518	773	656	1493	1066	2448	3757	4287	2468	2871	1948	2253	brox
ENSXETG00000015034	2160	178	252	232	819	690	737	2122	2504	1372	2113	1527	1693	aida
ENSXETG00000015035	2538	73	114	112	93	73	127	140	174	102	158	100	124	FAAP100
ENSXETG00000015036	2091	1263	1724	1575	2102	1680	2359	1257	1347	824	1489	1079	1164	ENSXETG00000015036
ENSXETG00000015037	1209	86	120	83	103	97	131	176	183	156	154	89	121	taf1a
ENSXETG00000015038	2417	735	1059	956	904	703	975	1518	1643	992	574	379	447	nploc4
ENSXETG00000015039	2970	1	6	3	8	2	19	20	14	11	19	16	18	hhipl2
ENSXETG00000015042	402	0	0	0	1	0	0	7	0	2	0	0	0	
ENSXETG00000015044	9816	212	265	259	202	156	398	1435	1761	1028	439	316	365	il1rap
ENSXETG00000015045	705	0	1	0	0	0	4	0	0	0	2	2	1	cldn16
ENSXETG00000015048	78	0	0	0	0	0	0	0	0	0	0	0	0	
ENSXETG00000015049	1965	0	1	0	0	3	0	10	3	4	3	0	1	CCDC141
ENSXETG00000015050	2747	0	0	0	3	0	0	101	97	58	3	1	2	cldn1
ENSXETG00000015051	3573	159	238	218	380	318	638	1218	1444	872	1036	747	817	sestd1
ENSXETG00000015052	687	3	9	6	14	15	11	15	25	18	14	14	20	ENSXETG00000015052
ENSXETG00000015053	1699	56	77	76	152	115	222	672	762	454	288	191	224	gdpd5
ENSXETG00000015058	1665	1	0	0	0	0	0	9	4	5	0	1	0	klhl35
ENSXETG00000015059	1140	10	15	11	4	13	19	7	8	4	23	14	17	znf385b
ENSXETG00000015061	8493	4	4	1	59	39	88	447	518	299	175	125	127	map6

ENSXETG00000015062	1014	3758	5333	4605	4684	3434	5405	986	1074	664	4805	3456	3837	mogat2.2
ENSXETG00000015063	1965	138	204	151	402	310	785	581	622	384	557	386	486	uvrag
ENSXETG00000015068	2049	585	832	709	1739	1394	7407	16587	19407	10868	15613	10816	12215	tgfbi
ENSXETG00000015069	2175	73	113	110	95	66	165	215	294	149	277	180	210	ENSXETG00000015069
ENSXETG00000015071	2734	273	402	291	782	567	974	926	1072	608	756	549	607	mat2b
ENSXETG00000015072	1134	0	3	1	2	0	1	4	4	3	1	0	4	ENSXETG00000015072
ENSXETG00000015075	3793	87	165	126	874	638	2147	471	527	318	2007	1417	1645	plcb4
ENSXETG00000015076	1935	86	117	114	293	205	477	449	450	306	613	451	417	ENSXETG00000015076
ENSXETG00000015078	4466	213	278	281	328	305	611	985	1184	675	842	626	693	ENSXETG00000015078
ENSXETG00000015083	705	0	0	0	0	0	0	0	0	0	0	0	0	
ENSXETG00000015084	4233	1	0	0	5	4	1	7	7	3	5	3	9	kiaa2022
ENSXETG00000015086	2067	0	0	0	0	0	0	0	0	0	0	0	0	
ENSXETG00000015090	2354	24	38	39	51	49	100	128	150	87	106	45	81	pak5
ENSXETG00000015091	4275	329	514	415	598	516	1113	1048	1266	683	1142	812	976	osbpl1a
ENSXETG00000015092	1398	33	31	30	41	23	80	679	793	460	72	48	49	gbp1
ENSXETG00000015093	2268	1	4	3	88	61	5	4	10	3	7	3	4	ankef1
ENSXETG00000015094	3044	64	84	90	167	128	203	1238	1421	862	205	173	185	gbp3
ENSXETG00000015096	2024	20	23	26	69	55	60	297	297	195	221	162	170	snap25
ENSXETG00000015100	1883	108	132	111	322	243	250	939	1124	635	295	212	247	ttc39c
ENSXETG00000015102	10863	1048	1456	1229	3817	2840	2101	2691	3121	1827	3124	2115	2484	lama3
ENSXETG00000015105	897	0	0	0	1	1	1	0	1	0	0	0	0	ENSXETG00000015105
ENSXETG00000015106	1248	0	1	0	0	0	0	2	0	1	0	0	1	ENSXETG00000015106
ENSXETG00000015108	1554	5467	7902	6843	6044	4502	8555	1461	1689	1010	4301	3178	3497	rdh7
ENSXETG00000015109	4428	369	532	463	589	458	885	1276	1494	936	783	574	630	anks1a
ENSXETG00000015112	1164	1	2	0	8	8	19	37	44	34	65	38	44	cadm4
ENSXETG00000015117	1791	28	45	37	15	29	69	223	272	175	124	90	107	def6
ENSXETG00000015121	1485	163	241	216	557	397	1131	1196	1354	758	709	485	511	ppard
ENSXETG00000015125	1677	21	29	13	54	34	61	202	215	121	138	91	78	ENSXETG00000015125
ENSXETG00000015126	1232	2236	3270	2873	2503	1832	1787	2017	2208	1319	1273	891	998	slc35b1
ENSXETG00000015127	1465	0	0	0	1	0	0	0	1	1	0	0	0	mkrn3
ENSXETG00000015128	1947	161	259	231	443	345	452	673	667	475	575	419	448	xrcc1
ENSXETG00000015129	546	32946	48795	42450	55437	40591	61786	57735	64139	40377	142677	99163	106916	rps10
ENSXETG00000015130	4220	5	9	10	362	244	18	1	2	5	52	36	42	cacna1s
ENSXETG00000015131	1020	0	4	0	1	1	3	0	2	0	2	3	2	tmprss5
ENSXETG00000015133	1647	81	121	90	165	138	191	312	331	198	156	101	119	phldb3
ENSXETG00000015135	618	0	0	0	0	0	0	0	0	0	0	0	0	
ENSXETG00000015140	1011	0	0	0	0	0	0	0	0	0	0	0	0	
ENSXETG00000015141	2493	460	714	602	998	731	1705	2187	2347	1369	1394	977	1087	stard3
ENSXETG00000015142	1167	145	183	160	224	200	420	306	346	209	282	220	207	pgap3
ENSXETG00000015143	687	0	0	0	1	0	1	0	0	0	1	1	0	
ENSXETG00000015144	1567	0	0	0	1	0	3	4	4	1	1	0	0	tcap
ENSXETG00000015145	3885	433	641	547	4028	3043	4648	3112	3374	2157	6085	4420	4888	ERBB2
ENSXETG00000015146	1598	61	136	117	236	167	295	129	176	103	218	165	210	XB-GENE-5961860
ENSXETG00000015147	1367	600	849	684	665	545	863	1088	1238	742	550	371	447	mien1
ENSXETG00000015148	898	64	96	94	196	140	212	511	631	394	156	93	93	ENSXETG00000015148
ENSXETG00000015149	945	0	4	2	21	6	3	4	7	5	8	9	6	ENSXETG00000015149
ENSXETG00000015151	1569	892	1314	1146	1045	775	1394	1384	1432	929	1340	929	1103	grb7
ENSXETG00000015152	1194	2	2	6	2	2	7	34	55	27	12	10	9	ikzf3
ENSXETG00000015153	2249	236	302	290	684	519	960	2805	3151	1870	2064	1364	1537	plekha2
ENSXETG00000015154	1410	5	6	4	28	20	21	129	170	110	77	51	43	htra4
ENSXETG00000015155	2280	859	1265	1013	919	714	1391	1097	1224	789	712	486	535	tm2d2
ENSXETG00000015156	6000	2872	4457	3547	3864	2799	7665	6652	7543	4612	4010	2986	3141	adam9
ENSXETG00000015157	960	214	288	232	239	154	429	245	276	163	417	316	362	ggh.2
ENSXETG00000015158	1401	0	0	0	0	0	0	0	0	0	1	0	0	
ENSXETG00000015159	1779	2	3	4	110	101	39	39	46	32	43	15	26	eml6
ENSXETG00000015163	951	0	0	0	0	0	0	0	0	0	0	0	0	
ENSXETG00000015168	969	0	0	0	0	0	0	0	0	0	0	0	0	
ENSXETG00000015170	6969	1451	2261	1892	4695	3471	5010	5132	5962	3485	5294	3760	4303	sptbn2
ENSXETG00000015176	1002	0	0	0	0	0	0	0	0	0	0	0	0	
ENSXETG00000015177	891	0	0	0	0	0	0	0	0	0	0	0	0	
ENSXETG00000015178	954	0	0	0	0	0	0	0	0	0	0	0	0	
ENSXETG00000015179	849	0	0	0	0	0	0	0	0	0	0	0	0	
ENSXETG00000015180	960	0	0	0	0	0	0	0	0	0	0	0	0	
ENSXETG00000015184	2118	2715	3864	3300	1594	1212	2149	2245	2622	1459	770	531	581	ddhd2
ENSXETG00000015185	3165	428	698	520	695	480	548	956	1096	634	557	361	426	plpp5
ENSXETG00000015186	4566	110	140	128	290	160	389	532	629	359	397	325	308	NSD3
ENSXETG00000015189	1106	17	32	24	117	70	48	19	30	11	32	17	20	c2orf73
ENSXETG00000015190	3439	0	0	0	0	0	3	6	5	6	0	0	0	atp12a-like
ENSXETG00000015195	5487	658	919	800	1101	826	1504	1819	2194	1276	876	698	721	psme4
ENSXETG00000015196	1512	29	43	27	81	36	113	223	285	169	195	90	140	gpr75
ENSXETG00000015197	1445	1732	2404	2044	1667	1290	1852	1994	2121	1253	1006	697	813	erlec1
ENSXETG00000015198	1123	229	344	294	238	178	148	224	301	152	48	38	48	chac2
ENSXETG00000015199	1569	22	37	47	57	46	119	182	189	141	137	101	109	asb3
ENSXETG00000015200	795	0	0	0	1	0	0	15	4	5	4	2	1	ENSXETG00000015200
ENSXETG00000015201	3986	45	54	65	287	266	373	1613	1777	1042	556	405	488	lzts2
ENSXETG00000015202	828	0	0	0	0	0	0	0	0	0	0	0	0	
ENSXETG00000015205	1935	253	367	297	268	189	199	450	503	264	277	232	233	twnk
ENSXETG00000015206	959	1348	1959	1614	1139	849	1062	1735	1994	1138	664	494	497	mrpl43
ENSXETG00000015207	855	0	0	0	0	0	0	0	0	0	0	0	0	
ENSXETG00000015209	2986	108	120	98	498	404	555	830	987	584	937	705	778	sema4g
ENSXETG00000015214	1720	149	205	186	345	262	435	412	507	300	354	243	259	slf2
ENSXETG00000015216	2187	123	175	158	232	181	443	468	514	355	328	213	270	mfsd13a
ENSXETG00000015217	1682	1277	1780	1512	1871	1336	2418	3919	4629	2767	1753	1209	1335	actr1a
ENSXETG00000015219	1551	65	90	79	151	108	263	446	533	376	323	204	228	sufu
ENSXETG00000015225	549	63	83	71	117	84	151	351	391	240	74	31	45	arl3.1
ENSXETG00000015227	1264	328	522	459	478	345	508	571	753	395	344	237	281	sfxn2
ENSXETG00000015229	2427	0	0	0	2	3	1	1	0	0	0	0	0	
ENSXETG00000015230	1086	0	1	0	25	21	10	23	28	21	8	6	3	c8orf34
ENSXETG00000015231	3451	4	14	14	60	39	68	831	954	597	226	153	169	sulf1
ENSXETG00000015232	2244	0	2	0	2	2	6	53	71	37	7	0	5	slco5a1
ENSXETG00000015233	1332	0	4	0	26	11	7	9	7	6	37	30	23	plekhg7
ENSXETG00000015235	4379	145	180	189	505	366	932	925	1017	674	1199	875	1009	eea1
ENSXETG00000015237	396	3	6	9	18	25	33	119	130	85	65	42	39	ENSXETG00000015237
ENSXETG00000015239	1401	357	594	527	1067	858	1565	2071	2115	1543	1393	924	1033	ENSXETG00000015239
ENSXETG00000015245	1569	1	0	0	10	12	21	4	3	3	13	4	7	ENSXETG00000015245
ENSXETG00000015247	1339	573	876	704	548	382	656	552	690	377	393	325	332	hibch
ENSXETG00000015248	1969	631	817	715	513	435	838	805	904	509	376	270	272	inpp1
ENSXETG00000015249	2139	624	816	686	848	702	1573	1433	1842	961	763	506	593	mfsd6
ENSXETG00000015250	2237	39	55	40	85	49	105	188	219	128	115	92	109	nab1
ENSXETG00000015251	1224	1	5	4	2	5	3	60	100	33	24	14	20	pou2f3
ENSXETG00000015254	1338	0	0	0	0	0	1	1	0	0	1	0	0	ENSXETG00000015254
ENSXETG00000015258	1288	271	399	331	248	195	423	393	486	275	383	253	341	impact
ENSXETG00000015262	252	470	710	646	590	473	586	549	570	411	449	368	366	tmem256
ENSXETG00000015263	3515	324	454	410	596	436	1268	528	594	389	890	657	744	ENSXETG00000015263
ENSXETG00000015264	429	172	300	288	332	291	348	392	435	268	281	223	244	mtx1
ENSXETG00000015266	3059	278	411	345	541	421	933	606	737	458	425	306	362	stoml1
ENSXETG00000015267	1454	452	672	534	910	664	1366	1394	1704	1044	898	630	738	ubl7
ENSXETG00000015268	4321	103	137	117	284	221	480	862	923	593	605	410	445	peak1
ENSXETG00000015269	1377	350	555	462	767	544	1003	1278	1428	846	1206	792	949	hmg20a
ENSXETG00000015270	2687	9	17	17	20	22	40	189	224	125	98	65	72	lingo1
ENSXETG00000015273	6882	2	4	4	30	17	26	319	352	196	121	116	98	XB-GENE-990170

ENSXETG00000015279	2473	274	338	321	811	685	1254	854	1011	615	1147	748	883 snx33
ENSXETG00000015280	1385	349	477	378	380	323	432	615	692	470	621	423	472 snupn
ENSXETG00000015282	3427	32	56	42	250	167	297	546	709	402	810	591	627 XB-GENE-967840
ENSXETG00000015283	3828	550	788	635	1131	934	2204	2885	3380	2026	2088	1500	1648 sin3a
ENSXETG00000015284	2067	2030	2653	2264	2107	1635	3268	1230	1530	877	2411	1756	1969 tspan3
ENSXETG00000015285	1416	0	0	0	0	0	0	0	1	0	0	0	0
ENSXETG00000015287	603	0	0	1	1	0	0	1	2	3	0	0	0 ENSXETG00000015287
ENSXETG00000015288	10720	2	1	1	12	4	25	41	45	18	37	22	17 tenm4
ENSXETG00000015289	1215	21982	31713	27524	31392	23919	43051	42049	49438	29220	35709	24588	27576 anxa2
ENSXETG00000015291	1331	7442	11111	9500	13207	9937	16548	17968	20384	12485	11909	8441	9205 actr3
ENSXETG00000015293	1053	834	1157	1098	764	556	906	2080	2420	1465	708	489	588 sec11a
ENSXETG00000015294	3860	217	382	335	468	385	883	738	878	492	994	692	749 znf592
ENSXETG00000015297	1266	45	48	35	108	101	169	181	209	117	144	105	129 c21orf2
ENSXETG00000015299	5243	1020	1574	1341	3395	2567	5267	4755	5224	3047	5467	3726	4516 smarca2
ENSXETG00000015309	699	29633	42513	35481	25641	20200	31649	42606	45671	31552	21761	15133	16850 pfn1
ENSXETG00000015314	531	513	772	615	930	723	1948	802	978	649	1614	1161	1241 rnf167
ENSXETG00000015319	4035	106	162	113	329	244	640	692	805	485	495	368	372 scaper
ENSXETG00000015321	1852	0	0	0	0	0	2	3	11	4	6	1	1 ajap1
ENSXETG00000015322	1249	5265	7577	6640	4589	3582	3959	3314	3922	2343	1453	1053	1178 etfa
ENSXETG00000015323	1083	0	0	2	5	1	3	21	14	14	56	43	41 isl2
ENSXETG00000015325	3045	1	0	0	3	4	5	21	21	9	22	20	17 cntn2
ENSXETG00000015330	1210	366	533	423	499	382	806	1102	1213	700	819	548	638 ube2q2
ENSXETG00000015336	978	463	684	614	551	442	1204	638	644	453	762	528	508 hmgcl
ENSXETG00000015341	1245	0	0	0	0	0	0	0	0	0	0	0	0
ENSXETG00000015342	2493	830	1237	1062	1735	1349	1625	2467	2639	1740	2249	1556	1700 ENSXETG00000015342
ENSXETG00000015347	1239	1	2	0	8	3	17	125	150	99	13	12	11 tgfb2
ENSXETG00000015353	687	31	28	34	14	17	83	185	301	172	58	28	42 cd53
ENSXETG00000015354	1778	80	103	101	127	118	264	97	150	71	124	69	87 XB-GENE-5945160
ENSXETG00000015359	814	440	592	511	336	241	450	226	219	124	315	238	267 lyplal1
ENSXETG00000015362	813	80	95	91	157	92	218	165	219	127	153	88	127 ENSXETG00000015362
ENSXETG00000015365	2160	872	1275	1025	1171	957	1990	2012	2192	1375	1703	1140	1341 tbc1d22b
ENSXETG00000015370	1700	23	32	21	18	6	44	13	15	5	42	39	32 mybph
ENSXETG00000015374	837	5	6	4	260	192	25	14	13	9	361	242	265 nr0b1
ENSXETG00000015376	3342	42	54	52	15	7	21	69	86	51	67	49	55 guca1a
ENSXETG00000015377	2709	30	39	27	154	129	117	171	222	136	277	157	235 itpr3
ENSXETG00000015382	2235	182	226	206	278	231	427	412	501	273	248	173	181 asmtl
ENSXETG00000015384	1000	27	52	35	42	29	60	52	73	45	49	31	46 ENSXETG00000015384
ENSXETG00000015388	1661	2	2	1	0	2	1	0	3	0	0	3	0 asmt
ENSXETG00000015389	705	286	398	323	300	220	615	420	458	233	636	452	508 dhrsx
ENSXETG00000015391	1095	32	40	48	110	75	157	528	585	323	237	178	191 cadm1
ENSXETG00000015395	5829	318	427	395	1263	954	1355	1476	1524	926	1785	1233	1356 helz
ENSXETG00000015396	447	0	0	0	0	0	0	0	0	0	0	0	0
ENSXETG00000015398	987	0	0	0	0	0	0	2	2	0	1	0	1
ENSXETG00000015399	975	3603	5034	4355	4288	3489	6543	1063	1233	687	1795	1327	1485 ENSXETG00000015399
ENSXETG00000015400	1707	17	30	35	30	25	89	106	108	62	66	47	51 tepsin
ENSXETG00000015401	3159	455	707	581	979	738	1466	1471	1652	976	1122	811	973 slc38a10
ENSXETG00000015402	1968	0	1	3	1	1	11	12	14	11	13	8	18 ENSXETG00000015402
ENSXETG00000015404	1806	331	489	369	1109	889	1282	1800	1974	1202	1243	886	1013 shc1
ENSXETG00000015405	2127	0	0	0	0	0	0	0	0	1	0	1	0
ENSXETG00000015412	1923	307	417	422	392	320	954	3560	4163	2403	1028	703	771 hcls1
ENSXETG00000015415	426	129	211	180	307	251	321	446	560	348	355	223	257 ENSXETG00000015415
ENSXETG00000015416	1020	11	17	7	20	9	47	63	85	49	138	115	138 gpr78
ENSXETG00000015417	1643	22	31	30	56	36	170	4004	4333	2851	1204	821	904 cpz
ENSXETG00000015419	819	0	0	0	0	0	0	0	0	0	0	0	0
ENSXETG00000015420	1146	136	200	176	375	312	706	539	657	413	564	445	512 mon1b
ENSXETG00000015421	592	209	293	245	407	341	309	677	736	450	80	55	62 cks1b
ENSXETG00000015425	1037	1309	1864	1509	1491	1096	1527	943	1216	658	1444	1034	1109 esd
ENSXETG00000015426	2727	62	91	90	124	115	248	203	211	115	200	140	164 cpt1b
ENSXETG00000015427	1302	0	0	0	0	0	0	1	2	0	7	6	4
ENSXETG00000015428	1194	1	0	0	1	3	5	23	27	15	7	6	7 nhlrc1
ENSXETG00000015429	2466	462	700	693	638	490	745	781	926	550	823	594	644 kdm1b
ENSXETG00000015431	2617	181	249	252	357	255	580	771	944	497	1133	819	940 mylip
ENSXETG00000015432	1743	246	385	295	414	310	1040	537	601	376	831	594	721 dtnbp1
ENSXETG00000015433	3630	155	212	201	591	455	918	2339	2696	1592	3935	2720	3031 jarid2
ENSXETG00000015435	4990	187	300	235	404	283	1074	474	594	356	1122	779	859 rreb1
ENSXETG00000015437	3126	2	3	2	16	21	38	306	376	221	136	86	99 fat4
ENSXETG00000015439	1263	0	0	0	0	0	0	0	0	0	0	0	0
ENSXETG00000015440	1519	6	12	12	77	51	3	30	31	16	86	52	75 edn1
ENSXETG00000015441	7662	100	139	112	511	419	718	726	926	517	736	601	668 hivep1
ENSXETG00000015442	1434	2	0	4	10	7	10	37	42	34	27	14	18 syt4
ENSXETG00000015443	2990	249	356	292	400	302	649	550	669	374	466	379	402 vps33b
ENSXETG00000015446	1467	1224	1749	1572	1621	1333	2772	1113	1205	750	1036	704	818 XB-GENE-5892958
ENSXETG00000015447	297	1676	2455	2068	2195	1751	2345	2648	2904	1727	1814	1180	1328 ap1s1
ENSXETG00000015448	1296	74	108	75	27	19	32	229	279	191	140	90	110 serpine1
ENSXETG00000015449	4627	185	249	254	674	535	851	830	981	564	133	92	110 prc1
ENSXETG00000015452	2770	493	732	709	877	648	1080	1836	2070	1235	1060	719	837 unc45a
ENSXETG00000015454	3396	3	6	6	60	44	50	98	108	73	33	23	26 ednrb
ENSXETG00000015456	3392	4	2	6	11	7	12	79	110	52	220	166	182 muc3a
ENSXETG00000015457	2240	6	13	5	3	1	25	11	20	11	41	27	34 slain1
ENSXETG00000015458	3704	140	198	180	391	286	459	415	576	313	531	345	389 man2a2
ENSXETG00000015459	1563	56	57	55	187	140	189	370	397	219	250	207	227 ENSXETG00000015459
ENSXETG00000015460	2469	54	85	73	75	62	185	356	421	241	103	80	88 fes
ENSXETG00000015461	2538	618	935	689	1288	1007	1699	1489	1773	1011	2039	1402	1660 nedd9
ENSXETG00000015462	2674	430	588	532	2535	1932	5050	2342	2644	1537	2395	1629	1868 furin
ENSXETG00000015463	3611	287	405	373	635	523	1334	1365	1587	956	886	640	752 fbxl3
ENSXETG00000015464	904	0	1	0	1	1	0	1	0	0	0	2	0 lhfpl5
ENSXETG00000015465	1142	91	146	134	53	32	366	292	393	212	93	59	67 cln5
ENSXETG00000015466	723	321	438	388	261	177	231	369	477	219	143	102	102 ENSXETG00000015466
ENSXETG00000015467	1338	0	0	0	0	0	0	0	0	0	0	0	1
ENSXETG00000015468	381	26680	39824	32630	39686	30322	41150	42611	48237	27050	90742	63496	68849 rpl37a
ENSXETG00000015470	1748	574	835	729	1308	1001	1654	2340	2655	1639	1355	1005	1109 mfap1
ENSXETG00000015471	3369	110	155	149	443	394	764	1849	2219	1350	780	581	641 kctd12
ENSXETG00000015475	1533	0	0	0	0	0	0	0	0	0	0	0	0
ENSXETG00000015477	4711	5061	7246	5918	10511	7948	11893	13873	17116	9801	14936	10604	12109 ddx3x
ENSXETG00000015478	876	0	0	0	0	1	1	0	0	0	2	3	3 ENSXETG00000015478
ENSXETG00000015481	1596	0	0	0	0	0	0	0	0	0	0	0	0
ENSXETG00000015482	1376	41	51	44	64	46	73	213	244	161	102	67	67 fam49a
ENSXETG00000015483	818	46536	69218	59362	80724	61437	89604	75694	86453	51336	181654	125767	137528 rpl8
ENSXETG00000015484	5937	242	359	276	822	636	1001	821	984	561	1876	1284	1412 focad
ENSXETG00000015485	1663	14	18	17	110	102	27	269	303	185	155	97	111 mycn
ENSXETG00000015486	626	1360	2070	1726	1808	1402	1749	3071	3361	1898	1357	973	966 hypk
ENSXETG00000015488	1626	25	35	33	8	13	22	171	222	124	70	46	49 ENSXETG00000015488
ENSXETG00000015489	8014	723	989	847	1350	1047	2015	2580	3141	1715	2177	1498	1695 usp9x
ENSXETG00000015490	2577	81	131	111	396	293	437	1591	1870	1156	518	421	426 adamtsl5
ENSXETG00000015491	2763	128	211	162	957	665	160	356	380	264	523	367	431 rasef
ENSXETG00000015500	2628	81	126	117	127	111	213	258	255	151	335	228	257 mllt3
ENSXETG00000015501	2547	3	11	5	0	3	2	348	498	254	11	3	2 ENSXETG00000015501
ENSXETG00000015502	4873	333	510	424	925	675	1084	1097	1222	744	1165	850	975 med14
ENSXETG00000015503	2673	18	24	28	105	105	273	1201	1411	791	277	182	214 il6st
ENSXETG00000015504	897	0	0	0	0	0	0	0	0	0	0	0	0
ENSXETG00000015507	408	3568	5038	4409	3953	3026	16077	4218	4825	3099	30614	21239	24112 rpl32

ENSXETG00000015509	2991	4	9	13	3	8	10	74	93	42	21	7	12	ENSXETG00000015509
ENSXETG00000015510	2626	0	0	0	0	1	2	6	6	3	3	4	5	ankrd55
ENSXETG00000015513	5183	100	129	142	631	455	794	1248	1466	872	1190	855	949	map3k1
ENSXETG00000015514	1757	91	113	74	372	270	137	608	775	409	472	361	390	plscr1
ENSXETG00000015515	2529	1	0	0	2	2	10	9	6	5	14	8	14	ENSXETG00000015515
ENSXETG00000015517	913	63	86	90	105	76	178	113	166	91	128	78	85	setd9
ENSXETG00000015520	1825	117	217	166	207	162	352	475	606	350	345	279	297	mier3
ENSXETG00000015523	1047	795	1093	907	804	648	2199	2480	3171	1778	1018	716	792	atp6ap2
ENSXETG00000015525	1725	4	4	2	69	77	61	204	264	140	61	53	61	arsj
ENSXETG00000015527	1795	280	395	335	300	242	341	675	819	438	443	334	349	gpbp1
ENSXETG00000015528	6325	673	960	790	2033	1595	2071	2426	2713	1622	3189	2277	2444	bcor
ENSXETG00000015532	3022	5	0	3	5	8	14	79	101	58	37	38	29	fbxl16
ENSXETG00000015538	1806	0	1	2	5	7	7	25	21	15	2	3	4	igfals
ENSXETG00000015541	1958	306	401	354	529	357	411	951	1006	678	629	446	433	fut11
ENSXETG00000015542	1686	1	0	1	199	134	29	12	17	5	63	37	45	ccdc78
ENSXETG00000015544	7530	122	167	151	434	344	690	795	978	561	1656	1159	1303	lrrk2
ENSXETG00000015547	2282	922	1215	976	1838	1374	2966	1455	1767	1044	3735	2572	2997	camk2d
ENSXETG00000015550	1938	79	133	97	148	107	577	258	270	190	174	124	125	slc2a13
ENSXETG00000015551	4686	1370	1958	1625	3142	2472	4372	4243	4918	3062	3591	2541	2723	mgrn1
ENSXETG00000015555	11442	19	21	18	91	74	90	201	264	143	226	185	185	ank2
ENSXETG00000015556	1032	0	0	0	0	0	0	0	0	1	0	0	0	
ENSXETG00000015557	1766	4	12	2	31	24	29	92	109	75	59	40	38	vsnl1
ENSXETG00000015558	11561	401	517	439	777	592	932	966	1260	693	1231	882	976	kif21a
ENSXETG00000015569	3471	96	147	142	327	229	212	422	511	267	268	168	195	smc6
ENSXETG00000015570	3951	17	13	19	97	67	38	118	122	69	37	21	26	gen1
ENSXETG00000015571	934	0	0	0	0	0	0	0	0	0	0	0	0	
ENSXETG00000015572	3100	0	0	0	0	0	0	0	0	0	0	0	0	
ENSXETG00000015573	1455	0	0	0	0	0	0	13	6	2	2	2	4	kcns3
ENSXETG00000015574	7941	11	14	9	95	71	117	645	791	449	143	96	123	pdzd2
ENSXETG00000015575	5076	3	2	2	30	27	74	190	172	109	185	151	126	nrxn2
ENSXETG00000015577	1074	11	8	13	16	14	21	90	86	72	82	52	61	ENSXETG00000015577
ENSXETG00000015578	1824	129	163	145	514	324	639	383	495	300	429	315	325	rdh14
ENSXETG00000015579	1470	0	0	1	0	0	2	0	1	1	0	2	1	cuzd1.2
ENSXETG00000015580	1679	7	9	7	50	34	94	6573	7168	4512	309	216	220	htra1
ENSXETG00000015581	1326	638	907	803	2106	1607	1661	1462	1676	999	1185	819	949	nt5c1a
ENSXETG00000015583	994	78	124	93	170	128	214	363	422	245	253	177	172	golph3
ENSXETG00000015585	2250	101	158	104	350	259	594	680	745	484	670	483	541	mtmr12
ENSXETG00000015586	2330	476	730	640	602	476	652	1735	1922	1196	811	559	609	plekha1
ENSXETG00000015587	3719	242	384	344	1221	985	1421	1881	2113	1260	2198	1590	1684	tacc2
ENSXETG00000015589	1002	101	169	134	246	193	326	324	402	214	312	252	245	nsmce4a
ENSXETG00000015590	2040	8798	12931	10856	7089	5402	15435	5173	6076	3605	4011	2828	3242	mpp1
ENSXETG00000015591	1554	193	251	265	478	395	507	1184	1229	790	453	333	344	ate1
ENSXETG00000015592	3453	408	569	506	1674	1277	2177	5187	6005	3602	6571	4579	4986	fgfr2
ENSXETG00000015595	1452	1	2	4	13	8	25	21	27	24	109	82	80	npr3
ENSXETG00000015596	1735	1	3	5	119	85	56	9	11	5	1561	1165	1177	ctrb2
ENSXETG00000015598	3255	932	1472	1230	2487	1793	2453	3029	3362	2019	2442	1760	1814	zfr
ENSXETG00000015599	651	211	285	262	154	129	396	41	55	39	117	62	73	sugct
ENSXETG00000015603	3558	288	429	359	902	698	1596	1083	1232	747	903	616	692	wdr11
ENSXETG00000015604	1434	187	274	233	468	374	335	582	635	389	754	547	599	ttll12
ENSXETG00000015605	1138	1920	2959	2531	2416	1792	5263	1338	1549	1010	1325	911	969	sigmar1
ENSXETG00000015609	2329	974	1382	1138	1150	883	4048	2589	3299	1907	1433	987	1134	glb1l
ENSXETG00000015611	963	16	11	9	9	5	44	39	54	28	19	12	20	cxcr6
ENSXETG00000015612	981	15	22	15	14	7	27	86	130	82	65	38	40	ccr2
ENSXETG00000015614	3090	21	12	17	82	82	195	393	509	298	209	127	180	fam65a
ENSXETG00000015615	2808	378	515	423	1123	992	1028	2314	2712	1575	1228	853	925	ctcf
ENSXETG00000015619	2587	69402	95722	78892	98166	75360	236880	18419	23593	13035	80582	56786	65252	slc6a19
ENSXETG00000015620	1150	22	23	29	107	75	80	175	237	129	96	57	82	enkd1
ENSXETG00000015621	1452	191	279	251	346	255	409	604	728	452	365	249	319	gfod2
ENSXETG00000015622	1719	1	1	1	10	7	14	72	91	43	47	36	26	syn2
ENSXETG00000015623	2229	0	0	1	11	10	13	46	64	27	34	26	36	cdh19
ENSXETG00000015624	3477	48	66	41	50	42	95	68	110	69	92	69	99	tert
ENSXETG00000015630	2427	1216	1771	1505	2905	2188	4111	2180	2620	1495	4222	2924	3219	ccdc68
ENSXETG00000015632	3618	1	8	1	11	8	26	70	101	67	40	27	24	dsel
ENSXETG00000015633	629	404	625	546	426	336	477	939	968	616	416	300	338	naa38
ENSXETG00000015634	882	100	122	120	186	113	237	215	239	150	391	292	297	tamm41
ENSXETG00000015636	4965	277	408	337	971	739	1606	5998	6502	4118	3064	2220	2341	kdm6b
ENSXETG00000015637	1365	193	191	198	258	221	409	639	788	477	289	207	237	tmx3
ENSXETG00000015639	1365	0	0	0	0	0	0	1	0	0	0	0	1	
ENSXETG00000015640	393	2	0	2	18	19	36	173	183	103	52	40	37	ninj1
ENSXETG00000015643	804	0	0	0	0	0	1	3	4	5	0	1	0	cd226
ENSXETG00000015645	2608	881	1226	900	915	790	1485	1029	1351	725	620	466	548	clptm1l
ENSXETG00000015646	5004	71	82	87	250	181	341	567	707	405	342	232	289	TNS3
ENSXETG00000015654	2063	2856	4221	3470	10000	7537	5653	8941	9914	6218	13596	9856	10361	tkt
ENSXETG00000015656	2158	671	917	778	3843	3032	2522	3089	3596	2103	1290	985	1033	igfbp1
ENSXETG00000015658	528	79	119	105	233	174	372	240	260	186	401	290	311	micalcl
ENSXETG00000015659	1649	1432	2012	1679	1936	1457	2943	2152	2620	1518	1576	1180	1288	cab39l
ENSXETG00000015660	1026	0	0	0	3	0	0	20	30	19	0	0	0	gal3st2
ENSXETG00000015662	1461	62	117	89	156	122	152	345	348	234	166	117	125	dcp1a
ENSXETG00000015663	3390	0	0	0	1	0	0	2	1	1	7	3	4	adcy1
ENSXETG00000015664	2656	5	4	5	38	37	28	120	122	87	53	38	52	cacna1d
ENSXETG00000015666	4658	123	165	136	247	179	332	262	311	163	271	171	209	setdb2
ENSXETG00000015669	571	7	12	12	5	13	9	120	140	103	21	11	23	ENSXETG00000015669
ENSXETG00000015671	2595	0	0	0	0	0	2	2	1	0	0	0	0	mcm6
ENSXETG00000015673	531	63	87	73	132	113	357	205	227	148	156	103	118	arl11
ENSXETG00000015675	2592	510	704	551	472	289	945	1506	1799	1031	549	405	507	rcbtb1
ENSXETG00000015677	1717	405	630	454	887	698	979	1699	1924	1201	871	640	710	kpna3
ENSXETG00000015678	475	32	40	32	77	40	114	343	438	241	152	129	177	ramp3
ENSXETG00000015679	849	396	563	473	473	328	762	622	821	472	962	709	829	hus1
ENSXETG00000015681	1279	239	383	319	225	167	270	623	755	434	104	77	89	upp1
ENSXETG00000015682	636	2332	3396	2871	2119	1593	2659	769	812	456	525	399	392	ebpl
ENSXETG00000015683	1630	500	689	545	458	347	770	747	978	556	587	386	460	spryd7
ENSXETG00000015684	2425	23	39	39	12	14	36	280	286	182	97	68	69	ikzf1
ENSXETG00000015685	5799	3	5	7	33	32	39	70	83	44	96	51	65	mapk8ip2
ENSXETG00000015687	2020	0	0	0	0	0	0	0	0	0	0	0	0	
ENSXETG00000015689	5061	9	21	18	126	110	204	407	450	241	151	109	130	shank3
ENSXETG00000015691	1227	4	7	6	7	3	15	70	86	50	33	21	38	trim13
ENSXETG00000015692	1863	73	121	109	93	64	447	297	363	219	210	141	136	tmem260
ENSXETG00000015693	2065	124	212	165	91	71	170	295	357	168	139	83	106	tdp1
ENSXETG00000015694	893	0	1	1	82	42	5	0	0	0	3	5	5	kcnrg
ENSXETG00000015696	1899	306	401	375	775	588	1302	822	914	578	1179	857	917	peli1
ENSXETG00000015699	348	1877	2757	2250	2314	1721	3183	2070	2351	1410	3780	2729	2937	cox7a2l
ENSXETG00000015700	4815	2836	4209	3673	8547	6599	11117	6896	7751	4736	11222	7905	8570	ktn1
ENSXETG00000015701	3381	335	514	450	435	354	482	676	900	474	488	332	375	EFL1
ENSXETG00000015703	1551	2843	4149	3545	4222	3153	4331	7306	8693	5019	1880	1307	1494	psmc1
ENSXETG00000015704	2161	72	131	102	66	60	188	291	318	193	233	163	197	fignl1
ENSXETG00000015705	5357	66	68	75	308	243	528	2026	2288	1443	554	387	460	qki
ENSXETG00000015706	915	3	8	5	1	1	3	20	52	22	5	2	9	ntm
ENSXETG00000015707	1521	621	865	766	584	435	2643	1143	1309	786	723	501	608	arsa.1
ENSXETG00000015708	3642	113	200	145	405	296	625	470	550	338	506	360	436	nrde2
ENSXETG00000015710	1113	0	0	0	2	0	0	1	1	0	0	3	0	tbpl2
ENSXETG00000015711	2576	0	0	0	4	0	4	6	6	5	3	3	10	grm7

ENSXETG00000015712	1238	265	355	296	339	247	255	301	324	194	106	65	83	rabl2b
ENSXETG00000015713	1163	25477	36254	30242	19458	14983	15916	14616	16710	9603	13780	9631	10903	calm2
ENSXETG00000015714	832	112	169	147	82	51	102	124	130	90	49	40	54	nme6
ENSXETG00000015715	1818	21	11	17	71	50	187	355	468	245	178	123	137	mid1
ENSXETG00000015716	1881	3	1	4	2	1	3	19	19	12	2	5	3	cbln4
ENSXETG00000015718	3391	246	314	274	696	537	1079	570	716	418	675	476	544	atg14
ENSXETG00000015719	2032	4	11	17	678	515	40	11	19	11	38	13	20	odf3
ENSXETG00000015723	909	0	0	0	0	0	2	20	12	7	4	3	6	dok5
ENSXETG00000015724	2167	13	12	13	16	10	4	7	8	4	47	32	54	tymp
ENSXETG00000015727	2577	0	1	0	5	2	10	21	23	13	19	7	11	clcn4
ENSXETG00000015731	2950	88	133	134	345	287	362	568	607	348	84	60	53	dlgap5
ENSXETG00000015735	954	0	0	0	0	0	0	0	0	0	0	0	0	
ENSXETG00000015741	987	0	0	0	0	2	0	4	2	3	0	1	0	
ENSXETG00000015745	3658	1477	1981	1729	2808	2196	3307	7589	9008	5037	3040	2206	2407	anp32e
ENSXETG00000015746	3539	39	90	61	211	149	535	1031	1194	700	636	429	489	wwc3
ENSXETG00000015748	1780	1	3	6	7	5	13	178	156	92	24	20	25	lmcd1
ENSXETG00000015750	1305	48	71	74	51	46	76	457	516	295	85	55	56	plekho1
ENSXETG00000015752	1042	1241	1707	1410	524	393	442	369	435	246	230	179	234	dnajb9
ENSXETG00000015753	1164	388	556	514	594	430	938	836	1008	543	537	392	413	vps45
ENSXETG00000015756	1218	189	229	187	84	59	172	86	121	45	107	63	94	thap5
ENSXETG00000015757	2898	9	7	13	59	35	77	632	707	456	118	74	108	ENSXETG00000015757
ENSXETG00000015758	2594	540	793	631	1273	950	2277	813	938	552	1152	758	859	pnpla8
ENSXETG00000015759	1461	157	230	215	170	153	363	346	421	238	320	209	236	rad9a
ENSXETG00000015764	1545	269	384	364	1318	998	344	297	363	218	2399	1739	1865	carns1
ENSXETG00000015765	2853	104	158	156	151	130	229	197	195	155	213	198	186	otud7b
ENSXETG00000015766	2639	456	645	510	1025	756	2418	3510	4167	2494	2218	1558	1759	tcirg1
ENSXETG00000015769	7015	9	2	4	13	9	16	30	37	20	49	26	30	nrcam
ENSXETG00000015771	1105	798	1145	960	1203	884	1041	1711	1917	1295	1875	1353	1523	haao
ENSXETG00000015772	884	37	52	41	276	213	62	314	391	210	627	392	442	lgals3
ENSXETG00000015774	2339	826	1241	1033	1452	1190	1164	2300	2520	1515	3001	2089	2253	pnn
ENSXETG00000015777	576	523	782	659	974	722	616	817	935	632	590	446	502	cldn34
ENSXETG00000015780	5832	412	628	554	1411	1101	1506	1875	2215	1265	2545	1739	1934	znf638
ENSXETG00000015781	1669	138	205	155	435	360	692	1391	1654	938	871	600	662	ssuh2
ENSXETG00000015782	2802	370	460	420	359	292	804	526	654	386	483	397	398	trappc6b
ENSXETG00000015784	1805	4	2	4	11	6	45	38	50	24	43	21	29	gpr143
ENSXETG00000015786	1692	60	90	86	243	176	384	1024	1194	707	301	204	260	tbl1x
ENSXETG00000015787	2469	15	8	9	69	53	226	366	424	253	57	34	41	jmjd7-pla2g4b
ENSXETG00000015792	4639	106	148	140	328	227	280	747	800	454	131	101	114	lmnb3
ENSXETG00000015793	2031	120	151	121	331	274	424	674	701	454	419	287	368	kdm2a
ENSXETG00000015794	5828	571	887	730	1250	943	2021	2159	2610	1473	1332	929	1038	ehd4
ENSXETG00000015795	999	0	0	0	0	0	0	0	2	0	0	0	0	gpr68
ENSXETG00000015797	6162	57	95	76	629	444	375	1272	1434	866	1142	768	910	ccdc88c
ENSXETG00000015798	4270	75	131	118	152	134	230	422	437	266	298	196	236	hmgxb3
ENSXETG00000015800	10899	2704	4024	3425	6346	4856	6493	1897	2127	1206	5073	3432	4036	sptbn5
ENSXETG00000015804	4474	227	382	298	706	465	1439	1381	1516	895	1240	881	948	ndst1
ENSXETG00000015806	3996	24	41	56	37	23	31	128	142	85	58	51	57	hecw1
ENSXETG00000015809	630	0	0	0	0	0	0	0	0	0	0	0	0	
ENSXETG00000015810	3060	1	5	1	19	11	30	639	704	444	376	256	296	cntn1
ENSXETG00000015811	2145	545	800	688	1103	877	1351	2612	3257	1863	1464	1030	1151	sh3gl1
ENSXETG00000015812	2571	549	818	727	1128	920	1450	1915	2193	1280	1375	933	1010	ppp4r3a
ENSXETG00000015813	2515	411	612	548	591	444	491	1107	1252	800	572	422	462	stk17a
ENSXETG00000015814	414	159	227	216	137	108	261	286	311	216	156	102	107	coa1
ENSXETG00000015815	678	37157	54277	46325	53387	41194	61828	57475	64346	38690	131834	93621	99971	rps14
ENSXETG00000015816	900	35	35	32	71	61	46	81	93	64	15	10	22	blvra
ENSXETG00000015817	1073	1	1	1	2	2	1	0	0	0	0	0	0	myoz3
ENSXETG00000015818	2645	228	325	268	295	206	374	583	711	416	463	340	355	lancl2
ENSXETG00000015819	1479	9	9	4	17	11	18	713	857	525	59	59	61	tc2n
ENSXETG00000015820	1416	240	365	335	508	371	651	777	906	469	447	326	351	dctn4
ENSXETG00000015822	3761	7	4	4	67	48	67	367	509	278	134	105	123	fbln5
ENSXETG00000015825	450	0	0	0	0	0	0	0	0	0	0	0	0	
ENSXETG00000015826	3633	132	191	156	273	168	411	766	941	569	490	373	382	egfr
ENSXETG00000015828	2721	7	5	6	5	7	9	18	17	7	14	15	9	loxl4
ENSXETG00000015829	1527	12	9	15	161	106	106	3556	4225	2416	160	110	116	ENSXETG00000015829
ENSXETG00000015830	342	92	150	147	263	221	257	1119	1281	772	610	402	443	gpx3
ENSXETG00000015831	2842	1288	1947	1678	2817	2165	3146	4886	5445	3232	2053	1438	1603	tnip1
ENSXETG00000015832	2071	104	138	136	245	216	470	3659	4093	2465	1461	1080	1158	anxa6
ENSXETG00000015833	4563	533	772	630	859	617	986	973	1214	703	935	680	759	trip11
ENSXETG00000015843	201	6487	9367	7537	3544	2678	3718	3867	4633	2555	2456	1787	1873	sec61g
ENSXETG00000015845	4041	210	284	208	338	232	320	430	565	329	279	203	224	tpk1
ENSXETG00000015847	2698	440	627	499	923	707	1276	949	1129	661	946	665	743	cog4
ENSXETG00000015852	3351	1	0	0	3	5	8	22	36	19	6	3	5	megf11
ENSXETG00000015857	2385	0	3	0	20	15	25	77	109	61	35	24	29	mtss1l
ENSXETG00000015859	1913	148	193	167	236	166	216	251	329	191	332	246	255	tex30
ENSXETG00000015861	3600	9	1	2	38	18	76	146	165	100	46	33	36	klhl4
ENSXETG00000015863	4869	229	285	258	515	408	1457	1503	1808	959	682	465	513	vac14
ENSXETG00000015865	3791	144	217	165	465	323	692	551	629	364	611	440	490	dis3l
ENSXETG00000015873	5325	0	0	0	0	0	0	0	0	0	0	0	0	
ENSXETG00000015878	15426	7	12	14	276	235	31	31	37	22	43	30	41	HYDIN
ENSXETG00000015884	2157	66	105	82	130	82	272	147	205	111	174	139	157	cmtr2
ENSXETG00000015887	2589	3673	5362	4533	6624	4879	6373	8098	9239	5353	6172	4517	4889	map2k1
ENSXETG00000015893	1039	379	525	474	356	274	635	466	547	352	404	268	321	snapc5
ENSXETG00000015894	2443	152	204	152	352	290	117	485	540	286	90	47	59	zwilch
ENSXETG00000015896	1453	5742	8595	7230	5508	4116	6496	4765	5638	3309	3805	2722	2959	got2
ENSXETG00000015897	1356	80	148	113	173	131	548	783	884	539	185	113	158	slc38a7
ENSXETG00000015898	1278	75881	112396	95108	122913	93617	146037	134814	151580	89128	330487	230846	255552	rpl4
ENSXETG00000015899	1857	0	0	0	0	0	44	1	1	0	102	80	79	slc7a2.2
ENSXETG00000015900	7867	1536	2187	1773	4299	3084	5150	5857	6869	4026	4843	3425	3943	cnot1
ENSXETG00000015904	1698	10	8	14	1	1	2	0	1	1	6	2	4	lctl
ENSXETG00000015905	1351	0	2	3	17	8	28	49	67	30	61	56	64	dach2
ENSXETG00000015908	501	192	245	221	332	250	404	799	891	526	330	256	235	eif4e3
ENSXETG00000015911	1824	1	0	4	2	3	2	6	3	3	3	1	2	zp4
ENSXETG00000015912	645	0	0	0	0	0	0	1	2	1	0	0	0	
ENSXETG00000015913	1687	2691	3995	3303	16793	12727	44027	24709	27737	16149	7772	5316	5967	sqstm1
ENSXETG00000015914	1876	69	88	87	93	59	232	496	512	306	236	151	159	rybp
ENSXETG00000015915	1515	0	0	0	0	0	0	0	0	0	0	1	0	
ENSXETG00000015918	1163	1480	2141	1762	1850	1374	1780	2496	2749	1747	2046	1348	1507	chtop
ENSXETG00000015920	6427	167	240	208	977	746	1399	1825	2091	1227	951	686	734	tbc1d9b
ENSXETG00000015921	3411	0	0	0	0	0	0	0	0	0	0	0	0	
ENSXETG00000015923	1365	0	0	0	0	0	2	0	1	1	2	1	1	
ENSXETG00000015924	6856	279	414	312	531	432	671	1182	1344	750	935	677	738	lrrk1
ENSXETG00000015926	5948	1046	1403	1281	2428	1819	3597	2811	3156	1990	3306	2252	2602	ncoa2
ENSXETG00000015929	1269	6	7	13	34	23	47	223	219	157	40	23	37	gxylt2
ENSXETG00000015931	5831	164	252	186	906	737	1295	3603	4247	2621	1641	1261	1359	slc9a3r2
ENSXETG00000015932	2858	522	776	678	494	390	757	1142	1451	845	545	437	452	ppp4r2
ENSXETG00000015934	1259	244	371	307	356	297	436	667	700	413	405	282	311	nthl1
ENSXETG00000015935	5899	495	641	595	1020	767	2208	2042	2379	1324	2544	1793	2008	tsc2
ENSXETG00000015936	258	0	0	1	0	0	0	0	0	0	0	0	0	ENSXETG00000015936
ENSXETG00000015937	1248	9	12	7	50	25	46	40	56	25	43	31	33	ENSXETG00000015937
ENSXETG00000015938	1542	1	1	1	5	3	4	96	106	64	1	0	0	cyp1c1
ENSXETG00000015939	2309	55	81	57	287	243	283	863	924	584	750	510	540	smad6
ENSXETG00000015940	1896	77	98	94	206	147	337	136	153	93	299	184	223	ENSXETG00000015940

ENSXETG00000015941	1727	482	682	552	702	551	1060	1299	1511	899	811	543	566	rnf130
ENSXETG00000015944	2343	0	0	0	5	3	1	1	2	0	16	12	5	
ENSXETG00000015945	12714	22	29	35	148	109	265	820	953	565	603	396	474	pkd1
ENSXETG00000015946	1472	70	92	66	131	91	68	205	257	118	76	67	50	orc5
ENSXETG00000015947	1278	0	0	0	2	1	0	0	3	0	5	1	1	ENSXETG00000015947
ENSXETG00000015948	3112	6275	8863	7457	4215	3220	3755	5323	6386	3531	3249	2312	2566	tram1
ENSXETG00000015950	1800	11	18	18	40	40	19	63	77	50	12	8	10	mcph1
ENSXETG00000015951	2263	160	230	168	369	285	99	371	401	240	78	61	42	rad51
ENSXETG00000015952	1692	4098	5925	4934	4281	3311	5760	4258	5118	2676	3978	2908	3198	vdac1
ENSXETG00000015953	876	683	945	848	776	601	1637	504	598	333	583	433	458	lactb2
ENSXETG00000015954	1178	468	734	565	577	443	286	638	759	409	287	188	194	kdelr3
ENSXETG00000015955	1967	0	0	0	5	3	5	21	20	7	15	13	12	eya1
ENSXETG00000015962	1807	328	388	380	233	200	373	322	380	231	315	230	247	agpat5
ENSXETG00000015963	1830	10	20	15	68	57	68	139	151	96	165	114	108	rab26
ENSXETG00000015964	399	849	1245	1032	346	252	713	647	833	441	604	401	450	tmem14a
ENSXETG00000015966	1336	373	533	433	1734	1375	2240	903	1079	632	9216	6514	7010	gcat
ENSXETG00000015967	591	593	910	691	1314	975	2261	2795	3252	1781	3057	2084	2245	h1f0
ENSXETG00000015968	6771	58	80	75	316	190	346	304	401	216	122	98	117	ENSXETG00000015968
ENSXETG00000015970	1991	275	411	348	769	605	1036	1736	1934	1127	1186	863	1002	triobp
ENSXETG00000015971	1785	0	2	0	0	0	0	3	2	1	3	0	2	traf7
ENSXETG00000015973	1398	217	311	292	201	128	390	368	393	259	317	211	240	rpusd2
ENSXETG00000015974	1875	95	155	116	363	301	519	445	532	328	426	249	352	ick
ENSXETG00000015977	1355	0	1	0	0	0	0	0	0	1	8	4	5	
ENSXETG00000015978	2641	87	94	85	134	106	268	1162	1257	792	282	229	213	ski
ENSXETG00000015979	4925	70	106	101	262	251	215	843	1020	601	630	453	548	trpa1
ENSXETG00000015980	1475	1530	2080	1795	2508	1977	4243	2503	2876	1796	3313	2260	2572	fbxo9
ENSXETG00000015984	886	229	338	312	290	230	325	468	504	307	308	203	232	nol12
ENSXETG00000015985	2467	193	277	236	225	150	585	342	359	247	711	476	574	XB-GENE-5997771
ENSXETG00000015986	1870	10	18	11	76	37	91	40	68	29	266	162	189	adap1
ENSXETG00000015989	9330	49	75	50	467	377	244	768	837	497	89	51	64	frem2
ENSXETG00000015990	1494	13	22	31	60	26	64	130	173	97	90	62	67	ENSXETG00000015990
ENSXETG00000015991	942	0	0	0	16	12	5	9	11	5	56	40	40	ENSXETG00000015991
ENSXETG00000015992	1398	236	344	287	66	61	191	198	244	163	302	215	240	slc2a15a
ENSXETG00000015993	1274	457	672	569	870	599	1044	2866	3325	1876	1167	809	854	lims1
ENSXETG00000015994	2759	10056	14011	11721	2929	2271	1083	1110	1288	702	340	238	277	elovl5
ENSXETG00000015995	405	33	59	37	23	28	28	6832	7997	4459	905	680	748	lgals1
ENSXETG00000015996	1259	195	338	282	462	338	628	528	580	349	529	380	373	c15orf57
ENSXETG00000015998	998	2	3	2	1	1	0	31	34	34	7	5	4	XB-GENE-5849758
ENSXETG00000015999	2019	270	451	348	693	555	883	1100	1233	742	717	548	628	prkcz
ENSXETG00000016000	402	4	8	5	5	4	2	5	3	4	1	1	6	ENSXETG00000016000
ENSXETG00000016003	2610	0	2	1	20	4	6	12	7	4	19	7	7	kcnb1
ENSXETG00000016004	1083	0	0	0	0	0	0	0	0	0	0	0	0	
ENSXETG00000016005	1791	424	614	524	963	650	1392	1660	1826	1174	1022	757	807	gga1
ENSXETG00000016006	8629	867	1229	1059	2414	1850	2365	2752	3272	1814	2078	1479	1637	ranbp2
ENSXETG00000016008	6686	401	572	486	911	741	2043	2739	3000	1898	2011	1464	1573	pik3c2a
ENSXETG00000016009	3313	712	1005	830	931	714	1053	1219	1378	817	543	355	431	gclc
ENSXETG00000016010	600	147	288	188	358	256	355	396	459	284	243	169	194	commd2
ENSXETG00000016011	1376	135	194	168	425	320	631	1501	1517	946	737	537	553	wwtr1
ENSXETG00000016012	462	25641	38480	32747	31864	23484	24667	19072	21857	13139	18033	12789	13746	lgals2
ENSXETG00000016013	1110	0	0	0	0	0	0	0	0	0	0	0	0	
ENSXETG00000016014	1667	970	1510	1232	2135	1785	2498	686	761	512	1470	1004	1157	stoml3
ENSXETG00000016016	4948	0	2	1	1	4	3	17	26	10	4	3	4	elfn2
ENSXETG00000016019	1359	118589	167366	138278	57081	44485	102464	17673	21669	12010	34357	23732	27655	tm4sf4
ENSXETG00000016020	2733	173	259	231	518	357	653	753	823	468	743	521	558	proser1
ENSXETG00000016025	1185	79	158	121	116	85	319	678	856	498	239	140	190	cyth4
ENSXETG00000016026	1506	1186	1729	1445	1437	1096	1570	2074	2350	1485	872	611	664	nucb2
ENSXETG00000016028	1719	0	0	0	0	1	0	0	0	0	0	1	0	
ENSXETG00000016030	2827	47	57	39	116	96	151	176	224	126	108	69	83	ccdc138
ENSXETG00000016031	2632	51	85	82	34	21	65	129	124	88	99	64	89	edar
ENSXETG00000016032	1047	156	223	205	219	171	550	369	428	261	379	275	321	nhlrc3
ENSXETG00000016034	2317	12	33	21	144	117	208	885	999	620	388	283	325	lhfp
ENSXETG00000016035	2117	562	870	758	771	608	1269	1612	1900	1091	1084	725	823	cog6
ENSXETG00000016036	501	100	145	111	71	46	117	28	41	26	74	35	62	tmem128
ENSXETG00000016038	1257	198	293	269	245	207	233	419	459	270	279	224	260	lyar
ENSXETG00000016039	1119	6	20	7	30	19	21	19	35	17	35	27	23	sstr3
ENSXETG00000016040	2337	4	4	2	40	30	17	14	16	10	58	38	47	tmprss6
ENSXETG00000016041	2607	227	375	335	336	293	510	587	653	426	478	354	354	zbtb49
ENSXETG00000016042	932	144	204	172	1374	1013	147	92	100	57	2099	1505	1679	tm4sf1
ENSXETG00000016045	423	294	433	350	626	517	677	840	825	535	698	527	539	c11orf58
ENSXETG00000016048	2198	599	873	731	557	406	1006	552	625	382	864	551	639	foxo1
ENSXETG00000016050	432	1	0	2	3	5	6	35	53	35	10	4	8	nsg1
ENSXETG00000016051	1184	365	518	433	382	300	484	555	634	379	558	355	413	stx18
ENSXETG00000016052	1610	0	0	0	0	1	0	2	2	6	0	0	0	
ENSXETG00000016053	570	20	34	32	21	17	24	9	10	6	65	30	55	kctd17
ENSXETG00000016054	1627	5349	7759	6721	3550	2762	6561	2281	2687	1707	5635	4065	4484	mpst
ENSXETG00000016057	1123	1593	2317	1999	4197	3154	2034	1455	1663	992	1751	1236	1331	tst
ENSXETG00000016058	1821	12	14	18	62	40	54	123	119	69	44	50	42	sox6
ENSXETG00000016060	2967	356	541	501	796	549	2149	1417	1827	934	1549	1094	1284	hps3
ENSXETG00000016062	2309	24	33	33	42	25	68	109	161	97	89	50	68	znf184
ENSXETG00000016064	1443	2	6	9	323	238	17	14	27	7	40	20	14	tekt4
ENSXETG00000016071	2055	430	589	499	1381	1057	1509	286	316	180	2358	1559	1760	slc5a11
ENSXETG00000016072	1692	1755	2588	2161	1290	913	3280	1515	1826	970	1739	1156	1362	gyg1
ENSXETG00000016074	1116	11	13	15	15	11	40	175	201	156	62	52	59	chst14
ENSXETG00000016075	2214	52	66	68	164	118	224	229	234	183	347	245	270	bahd1
ENSXETG00000016076	1257	3	0	1	2	1	14	485	667	343	29	24	24	cpa3
ENSXETG00000016077	1056	0	0	0	31	26	54	3	3	1	1088	773	853	cpb1
ENSXETG00000016078	1852	1611	2304	2032	2904	2196	3127	1269	1400	901	2161	1431	1566	ivd
ENSXETG00000016079	4383	9	8	6	56	82	163	111	166	91	72	55	73	agtr1
ENSXETG00000016084	2901	16	18	24	151	103	143	926	1038	626	84	67	82	bnc1
ENSXETG00000016085	3384	0	0	0	0	0	5	4	4	5	3	0	3	grid2ip
ENSXETG00000016086	2365	15	14	11	70	53	117	636	749	429	252	186	186	hdgfrp3
ENSXETG00000016087	1425	0	0	0	0	0	0	3	0	0	1	0	1	
ENSXETG00000016088	1302	6	7	6	9	5	10	91	122	45	19	8	18	tmem173
ENSXETG00000016089	996	41	73	63	94	74	127	107	144	85	138	61	92	zdhhc4
ENSXETG00000016090	533	374	536	371	383	271	589	685	770	442	332	250	260	cdkn2aipnl
ENSXETG00000016091	3128	482	724	630	1495	1119	2460	2639	2810	1724	2117	1601	1670	e4f1
ENSXETG00000016092	1965	3404	4772	4102	4839	3869	7087	7259	8499	5013	5794	4018	4557	ppp2ca
ENSXETG00000016094	2496	10	10	7	4	7	6	31	33	19	5	1	9	homer2
ENSXETG00000016095	750	3594	5084	4377	4649	3393	6148	7556	9364	5193	3700	2547	3063	skp1
ENSXETG00000016096	897	0	1	1	3	0	0	1	2	0	3	2	2	hsd17b1
ENSXETG00000016097	924	409	569	438	233	170	225	353	451	268	127	79	106	pgp
ENSXETG00000016099	2397	663	932	829	1378	1101	2532	2749	3102	1906	1899	1341	1429	whamm
ENSXETG00000016100	1084	2	7	13	18	14	17	83	70	50	64	51	59	tcf7
ENSXETG00000016102	1543	232	407	278	604	434	674	833	886	500	543	360	390	mlst8
ENSXETG00000016103	2277	0	0	1	2	1	1	42	63	24	0	0	0	fsd2
ENSXETG00000016104	2307	175	214	195	368	306	790	539	570	334	658	474	494	pde8a
ENSXETG00000016106	4101	0	2	3	26	18	77	122	144	76	132	79	102	caskin1
ENSXETG00000016109	2052	22916	32774	27156	9838	7713	5129	23	17	12	110	98	119	gatm
ENSXETG00000016110	954	0	0	0	0	0	0	3	2	2	0	0	0	
ENSXETG00000016111	1848	2	1	4	63	64	74	1041	1252	720	419	282	318	slc27a2
ENSXETG00000016114	1715	0	0	0	0	0	2	16	9	6	1	0	2	dnd1

ENSXETG00000016115	1281	207	342	304	169	146	159	303	358	219	133	90	98	hars
ENSXETG00000016116	1947	98	150	131	215	179	192	321	412	218	244	144	207	gabpb1
ENSXETG00000016117	3507	602	809	723	1037	824	1540	1712	2012	1091	1592	1093	1229	usp8
ENSXETG00000016118	702	164	253	227	290	238	390	872	921	506	358	291	269	zmat2
ENSXETG00000016119	3296	0	0	0	1	2	8	10	10	13	0	1	0	pcdha13
ENSXETG00000016123	2376	0	0	0	0	1	0	0	0	0	0	0	0	
ENSXETG00000016124	1723	143	173	156	351	298	558	1648	2106	1187	597	384	464	alcam
ENSXETG00000016127	2774	53	82	54	212	165	375	814	1015	575	707	483	557	CBLB
ENSXETG00000016128	1485	246	353	304	588	400	935	436	524	345	666	449	536	mgat3
ENSXETG00000016131	2727	73	105	76	107	83	201	323	445	222	195	127	157	BBX
ENSXETG00000016132	5180	224	287	241	383	291	572	948	998	628	531	374	438	mief1
ENSXETG00000016133	6216	0	0	0	3	0	2	3	4	1	3	3	5	cacna1i
ENSXETG00000016135	2320	1497	1963	1623	3009	2348	3579	948	1129	622	1929	1330	1495	hhla2
ENSXETG00000016136	5799	0	1	0	1	0	0	1	0	1	0	0	1	myh15
ENSXETG00000016137	990	45	98	74	66	31	145	339	346	228	140	119	137	grap2
ENSXETG00000016139	3438	80	110	78	237	188	100	321	430	243	102	67	95	kiaa1524
ENSXETG00000016140	557	832	1213	972	548	417	605	695	869	467	291	221	260	mrps23
ENSXETG00000016143	1603	650	963	842	700	508	1288	1531	1763	1039	843	602	672	tbc1d10a
ENSXETG00000016144	2406	1530	2146	1863	4233	2966	5360	4776	5353	3122	5284	3934	4078	sf3a1
ENSXETG00000016145	1820	3	16	9	66	38	27	29	27	13	30	31	23	ccdc157
ENSXETG00000016146	2018	4158	6030	4873	7420	5666	9517	1727	2152	1219	4550	3281	3595	sec14l3
ENSXETG00000016147	891	228	281	242	315	227	228	618	692	416	362	265	263	mtfp1
ENSXETG00000016148	1372	98	179	142	59	55	109	12	17	16	30	32	30	zp3.2
ENSXETG00000016149	1287	1209	1761	1486	2118	1620	5311	538	677	382	702	529	605	gal3st1
ENSXETG00000016151	6840	239	328	258	790	559	159	671	711	441	362	246	241	pole
ENSXETG00000016152	1104	12	6	9	20	4	18	47	51	39	41	21	23	p2rx2
ENSXETG00000016153	459	581	736	615	163	129	90	172	218	136	57	37	46	pla2g3
ENSXETG00000016154	2655	128	172	143	108	62	73	159	197	134	96	85	87	inpp5j
ENSXETG00000016155	5763	1081	1509	1286	2674	2128	5851	5960	7068	4170	7929	5709	6272	smtn
ENSXETG00000016156	3825	250	362	284	447	332	463	736	867	528	596	391	448	morc2
ENSXETG00000016157	1248	5	9	5	28	14	39	170	199	105	59	41	49	znf219
ENSXETG00000016158	3990	9	19	20	148	98	328	1728	1987	1208	312	217	263	arhgef40
ENSXETG00000016160	3026	92	168	127	441	323	981	2401	2850	1666	1070	836	859	ndrg2
ENSXETG00000016161	942	0	0	0	0	0	0	0	0	0	0	0	0	
ENSXETG00000016162	1276	2030	2963	2459	2121	1733	2944	4417	5066	3165	3040	2185	2538	hnrnpc
ENSXETG00000016165	1724	67	106	71	85	66	116	500	494	279	191	116	150	eogt
ENSXETG00000016166	3385	908	1446	1240	1969	1495	4450	2907	3173	2010	4258	2940	3267	tmf1
ENSXETG00000016167	1867	739	1104	938	887	693	1262	1583	1857	1019	931	679	716	uba3
ENSXETG00000016168	2654	13	7	23	24	25	9	24	22	19	12	7	4	lmod3
ENSXETG00000016169	3087	292	407	362	974	735	908	2045	2113	1413	1114	813	870	frmd4b
ENSXETG00000016171	1356	0	2	5	11	9	31	56	83	55	36	36	34	gata2
ENSXETG00000016174	728	0	1	0	0	0	0	3	2	3	0	0	0	
ENSXETG00000016175	2626	7203	10765	9228	6965	5314	4770	5842	6924	3908	3311	2353	2585	rpn1
ENSXETG00000016176	2165	4753	6840	5788	7370	5576	14303	12183	13722	8035	7740	5425	5877	rab7a
ENSXETG00000016178	999	1470	2153	1862	2180	1596	2928	3094	3349	2177	2764	2163	2202	XB-GENE-982799
ENSXETG00000016179	381	14	21	16	50	37	53	162	204	114	158	114	123	h2afj
ENSXETG00000016180	2502	5901	8885	7540	8266	6236	9277	8348	9371	5597	5315	3692	4204	copg1
ENSXETG00000016183	4170	1	1	0	4	4	1	157	163	94	16	8	9	otol1
ENSXETG00000016188	2217	0	3	2	1	0	2	1	1	0	0	1	0	slc26a6
ENSXETG00000016189	9006	4	6	3	22	15	26	77	74	31	87	55	50	celsr3
ENSXETG00000016194	2047	208	274	250	542	387	646	623	689	402	909	651	667	xiap
ENSXETG00000016195	4961	942	1437	1279	2268	1668	2464	2971	3507	2000	2927	2047	2204	thoc2
ENSXETG00000016197	2925	13372	19158	16472	28324	21375	63884	14107	16704	9977	44890	31473	35800	dpp4
ENSXETG00000016198	3450	1	0	0	3	0	0	10	9	7	6	1	4	slc4a10
ENSXETG00000016201	3139	0	0	0	1	1	1	0	1	0	0	0	0	
ENSXETG00000016202	1666	1616	2339	2062	2136	1727	2089	5099	5859	3450	872	576	636	psmd14
ENSXETG00000016203	1452	223	318	295	369	297	503	827	990	576	408	305	327	tank
ENSXETG00000016204	1250	47	57	62	94	69	75	424	466	256	100	64	88	rbms1
ENSXETG00000016205	975	0	0	0	1	1	1	14	15	9	1	0	0	b3galt1
ENSXETG00000016206	885	13	20	15	173	134	56	42	48	25	126	85	123	oxsr1
ENSXETG00000016207	1158	1	3	0	2	3	0	5	5	1	9	2	4	cers6
ENSXETG00000016209	1515	542	791	669	1357	1025	1837	867	1091	653	1218	820	976	nostrin
ENSXETG00000016210	3888	1	0	0	1	1	4	0	1	1	0	1	0	abcb11
ENSXETG00000016211	1678	136	188	160	579	396	972	1355	1602	943	430	314	311	dhrs9
ENSXETG00000016213	1378	1	0	1	0	2	1	0	0	0	0	0	0	XB-GENE-5957221
ENSXETG00000016214	13527	10	14	14	62	56	50	94	92	54	8	15	5	lrp2
ENSXETG00000016216	3198	0	0	0	0	0	0	0	0	0	0	0	0	
ENSXETG00000016218	594	0	0	0	6	7	0	0	0	1	1	1	2	ENSXETG00000016218
ENSXETG00000016219	3192	2204	3186	2639	2898	2264	5615	5890	7271	3998	1940	1303	1508	rnaset2
ENSXETG00000016220	3693	35	42	41	497	361	663	1811	2198	1287	508	364	448	nid1
ENSXETG00000016222	1773	588	834	646	1203	912	2067	1351	1695	966	849	632	653	gpr137b
ENSXETG00000016223	3282	84	117	108	206	175	558	516	676	363	416	302	341	ero1b
ENSXETG00000016224	4092	162	233	190	200	150	198	301	424	232	346	237	273	tarbp1
ENSXETG00000016225	1092	7	2	6	1	3	3	12	26	17	9	11	10	ENSXETG00000016225
ENSXETG00000016226	777	286	418	363	137	110	151	170	225	151	64	50	66	coa6
ENSXETG00000016227	1643	402	520	411	441	324	1044	99	138	84	167	112	131	kmo
ENSXETG00000016228	4652	1	4	2	15	3	11	32	56	34	19	15	22	fmn2
ENSXETG00000016229	1725	0	0	0	0	0	0	1	1	0	1	1	0	
ENSXETG00000016232	1587	137	159	151	391	336	427	531	592	401	527	396	424	gmeb2
ENSXETG00000016236	1563	20	42	32	271	192	78	83	102	63	231	172	198	efcab8
ENSXETG00000016238	790	5867	8886	7686	5143	3908	4392	1690	1852	1119	2729	1908	2127	gstk1
ENSXETG00000016239	1798	26	31	31	26	30	31	18	9	7	8	8	11	ybx3
ENSXETG00000016240	2952	8	5	8	51	49	19	299	354	190	32	25	22	styk1
ENSXETG00000016241	699	0	0	0	0	0	0	0	0	0	0	0	0	
ENSXETG00000016242	2733	2638	3867	3168	5274	4125	15573	10054	12084	6853	4364	3121	3581	gabarapl1
ENSXETG00000016245	444	2	2	5	11	1	2	1	1	0	31	25	26	tmem52b
ENSXETG00000016248	5986	0	0	2	3	2	2	3	1	2	39	22	33	myh4
ENSXETG00000016251	2230	94380	135955	116365	269059	207263	334006	14685	16705	9870	123514	85414	95203	mep1a
ENSXETG00000016254	1203	259	398	317	606	480	489	432	439	296	149	100	106	pafah2
ENSXETG00000016255	4950	11	19	11	25	18	14	12	13	10	21	12	11	tdrd6
ENSXETG00000016256	1234	1233	1772	1534	626	507	682	598	638	411	552	397	435	slc25a27
ENSXETG00000016257	1443	3393	4976	4445	1863	1363	1680	719	776	487	857	613	702	cyp39a1
ENSXETG00000016260	839	11	18	11	33	23	34	53	64	39	42	35	23	rcan2
ENSXETG00000016262	1542	436	620	539	673	527	1302	802	873	569	1194	816	905	enpp4
ENSXETG00000016264	2220	7663	11155	9508	14829	11354	18487	1683	1901	1108	10590	7356	8292	ENSXETG00000016264
ENSXETG00000016266	1941	88	134	119	196	147	207	509	614	362	202	141	161	trim69.1
ENSXETG00000016267	6417	0	0	0	0	0	0	0	3	2	0	1	1	
ENSXETG00000016268	2442	2842	4137	3510	10654	8481	14283	3547	3984	2441	10968	7850	8650	clic4
ENSXETG00000016278	1434	97	138	130	287	237	767	547	668	378	589	408	457	ENSXETG00000016278
ENSXETG00000016285	879	332	448	449	208	155	86	321	362	209	125	53	88	hmbs
ENSXETG00000016286	3807	1	4	4	9	3	20	71	65	61	35	11	17	trps1
ENSXETG00000016288	3060	12	25	13	85	52	122	310	345	190	140	98	114	tshz3
ENSXETG00000016289	471	89	141	105	72	49	107	203	255	153	105	105	88	ucn3
ENSXETG00000016290	3105	656	925	785	864	699	1276	1970	2319	1279	982	679	813	copg2
ENSXETG00000016292	1287	1	1	0	1	0	0	8	14	8	1	2	4	ENSXETG00000016292
ENSXETG00000016293	1284	5	5	6	388	256	96	63	85	45	3284	2349	2579	cpa1
ENSXETG00000016294	698	27588	40082	35099	23356	17245	30417	13109	15424	9568	21114	14746	16413	tff1
ENSXETG00000016298	4317	358	509	405	1074	800	1178	1550	1767	1102	1457	952	1102	bcl9
ENSXETG00000016299	1698	398	580	528	826	643	923	853	1009	595	814	554	671	tmem209
ENSXETG00000016301	2685	110	159	142	276	196	401	304	368	184	240	176	163	klhdc10
ENSXETG00000016302	1817	374	567	455	770	536	559	407	440	286	339	232	254	acp6

ENSXETG00000016303	1095	0	6	2	27	24	72	38	47	39	52	44	54	gja5
ENSXETG00000016305	1317	0	0	0	0	0	0	0	0	0	0	0	0	
ENSXETG00000016306	1581	159	278	224	371	286	255	505	550	316	323	256	267	nrf1
ENSXETG00000016307	852	0	4	4	0	4	2	0	0	0	1	0	0	ENSXETG00000016307
ENSXETG00000016308	858	71	102	73	67	42	87	139	158	90	75	48	59	gtpbp8
ENSXETG00000016309	1368	268	382	284	525	369	478	649	639	448	484	356	409	gpr89b
ENSXETG00000016311	1872	17	22	12	61	51	76	75	97	46	375	265	321	ENSXETG00000016311
ENSXETG00000016314	2562	25	51	28	163	106	254	675	811	433	538	370	429	ENSXETG00000016314
ENSXETG00000016315	1710	20013	28813	25380	25660	20136	46220	1701	1774	1220	13446	9587	10627	pdzk1
ENSXETG00000016316	843	41	42	49	228	194	392	512	616	350	306	204	226	ENSXETG00000016316
ENSXETG00000016321	1383	7	16	17	143	109	169	455	564	355	218	129	194	glp2r
ENSXETG00000016323	1572	96	158	145	348	257	758	435	482	287	1235	855	1010	ENSXETG00000016323
ENSXETG00000016324	630	4180	6326	5231	3318	2399	4356	2773	3073	1761	3990	2831	3097	ndufa8
ENSXETG00000016325	1026	1	5	1	3	3	11	7	9	3	11	1	5	lhx6
ENSXETG00000016326	1363	28	39	30	90	64	72	114	158	92	54	31	39	morn5
ENSXETG00000016327	913	312	402	380	292	233	321	345	472	246	174	132	116	mrrf
ENSXETG00000016328	642	103	127	116	107	79	112	171	191	110	102	61	81	rbm18
ENSXETG00000016333	1614	0	0	0	21	15	0	26	22	16	21	21	17	
ENSXETG00000016335	1610	507	762	678	660	540	932	1351	1751	967	588	402	487	pdcl
ENSXETG00000016336	1797	71	125	125	330	249	250	1197	1413	826	122	96	97	ptgs1
ENSXETG00000016337	1047	3	4	4	163	155	45	110	123	78	84	39	54	
ENSXETG00000016338	4056	1	2	4	7	4	5	94	89	74	19	12	6	f8
ENSXETG00000016339	1197	0	0	0	0	0	0	0	0	0	0	0	0	
ENSXETG00000016340	1857	1616	2303	1921	1348	1078	1224	2268	2670	1560	252	165	213	cmpk1
ENSXETG00000016342	1144	136	166	152	288	222	442	861	961	621	499	338	360	ndel1
ENSXETG00000016343	3956	64	83	58	257	214	161	323	398	217	176	127	191	stil
ENSXETG00000016345	6501	108	163	127	789	683	886	4958	5450	3494	756	576	642	myh10
ENSXETG00000016346	3653	6	9	8	15	11	36	55	61	33	18	14	13	tal1
ENSXETG00000016348	1124	0	0	0	0	0	0	0	0	0	0	0	0	
ENSXETG00000016349	1736	9089	13219	11250	5247	4125	6457	585	652	402	2065	1509	1681	cyp4b1
ENSXETG00000016350	1170	1	1	1	0	0	0	0	4	1	1	0	0	nmbr
ENSXETG00000016352	1628	2618	3742	3150	3283	2555	3631	1451	1748	1021	1882	1331	1561	efcab14
ENSXETG00000016354	1341	280	419	306	743	589	791	1459	1726	990	162	91	100	ccnb1.2
ENSXETG00000016355	651	30	44	43	31	20	70	116	133	86	65	53	57	mob3c
ENSXETG00000016356	2313	0	0	0	0	0	0	0	0	0	0	0	0	
ENSXETG00000016357	1365	1133	1693	1514	669	480	782	863	923	590	643	441	478	mknk1
ENSXETG00000016359	519	0	0	0	0	0	0	0	0	0	0	0	0	
ENSXETG00000016360	585	245	400	315	187	215	223	376	429	268	129	83	105	ublcp1
ENSXETG00000016361	3219	843	1214	1030	1121	850	1677	1487	1641	1025	1617	1210	1295	rnf145
ENSXETG00000016362	2562	0	0	0	0	0	0	0	0	0	0	0	0	
ENSXETG00000016364	2649	0	0	0	0	0	0	0	0	0	0	0	0	
ENSXETG00000016366	2439	0	0	0	0	0	0	0	0	0	0	0	0	
ENSXETG00000016367	2343	0	0	0	0	0	0	0	0	0	0	0	0	ENSXETG00000016367
ENSXETG00000016373	1230	308	385	362	502	325	569	778	957	507	535	402	432	nup43
ENSXETG00000016374	291	0	0	0	0	0	0	0	0	0	0	0	0	
ENSXETG00000016375	1638	506	761	648	305	240	500	598	672	405	530	390	377	vegfa
ENSXETG00000016376	2533	862	1277	1085	645	454	693	1107	1368	741	968	684	806	utp4
ENSXETG00000016377	1668	14	25	26	1	1	0	9	9	9	18	5	7	has3
ENSXETG00000016378	3117	150	206	203	283	225	234	443	513	349	283	200	212	tango6
ENSXETG00000016379	3243	8637	12495	10703	17909	13737	24299	35886	41847	23589	23834	16943	18699	cdh1
ENSXETG00000016380	2271	46	49	50	51	48	115	131	145	73	99	90	91	plekhm3
ENSXETG00000016383	1680	260	403	343	709	509	919	507	631	311	1089	719	822	fzd5
ENSXETG00000016384	1929	1	1	0	13	17	57	413	454	261	37	19	26	ENSXETG00000016384
ENSXETG00000016385	2101	309	457	381	382	285	702	688	846	510	593	357	445	ccnyl1
ENSXETG00000016386	1819	0	0	0	0	0	0	0	0	0	0	0	0	
ENSXETG00000016387	2178	0	1	0	2	2	2	49	61	33	14	12	5	foxc2
ENSXETG00000016388	1843	2	2	3	16	14	36	1096	1222	681	173	131	112	foxl1
ENSXETG00000016389	1416	3728	5449	4697	6970	5248	17500	4543	5126	3025	13238	9039	10249	hnf4b
ENSXETG00000016391	899	230	350	295	242	169	369	234	299	189	342	269	286	mettl21a
ENSXETG00000016392	2381	267	366	330	382	277	470	1139	1456	818	554	364	454	creb1
ENSXETG00000016394	3030	13	9	8	12	10	11	100	114	67	32	38	36	klf7
ENSXETG00000016395	1323	19	25	24	1	2	0	35	37	16	6	5	2	chrna7
ENSXETG00000016396	993	0	0	0	2	0	2	9	12	4	4	0	1	ENSXETG00000016396
ENSXETG00000016399	495	270	396	353	316	213	445	577	641	369	323	245	201	atraid
ENSXETG00000016401	5722	434	664	546	1216	904	560	1260	1387	879	1197	874	949	cad
ENSXETG00000016402	3351	301	435	348	405	246	523	803	888	548	419	249	345	myo1e.1
ENSXETG00000016403	1461	9	19	15	13	15	37	62	53	32	54	28	37	ENSXETG00000016403
ENSXETG00000016404	591	0	0	1	1	1	0	0	3	1	1	0	1	fndc4
ENSXETG00000016405	753	0	0	0	0	0	0	0	0	0	0	0	0	
ENSXETG00000016406	1113	368	532	488	688	560	866	1044	1156	699	702	491	562	snx17
ENSXETG00000016407	1895	9	18	23	235	170	47	33	38	23	26	13	8	iqcd
ENSXETG00000016408	990	0	0	0	0	0	0	1	0	0	0	0	1	
ENSXETG00000016409	669	9	12	26	35	23	33	62	74	45	79	40	58	rita1
ENSXETG00000016410	2613	568	830	725	835	646	743	1472	1664	982	1168	879	1000	ddx54
ENSXETG00000016411	1645	0	3	1	23	18	3	5	9	5	2	2	0	cfap73
ENSXETG00000016412	2881	3	4	3	4	3	0	0	2	1	2	2	1	rasal1
ENSXETG00000016413	3212	1	4	0	6	7	12	19	32	21	11	12	10	dtx1
ENSXETG00000016414	2646	216	324	239	188	157	309	2046	2438	1418	301	208	222	emid1
ENSXETG00000016415	1896	40	59	57	191	140	183	326	379	223	275	227	234	gas2l1
ENSXETG00000016416	1717	10	10	7	26	23	18	28	32	21	25	28	20	rasl10a
ENSXETG00000016417	3313	1527	2133	1909	3621	2667	3917	3662	4254	2381	2133	1497	1708	ap1b1
ENSXETG00000016418	3093	0	0	1	0	1	0	5	5	1	1	1	3	nefh
ENSXETG00000016419	2310	735	957	798	1252	907	1424	1683	2021	1174	1272	889	983	thoc5
ENSXETG00000016420	2082	1815	2478	2049	2605	2074	3895	1108	1350	716	1572	1108	1212	nipsnap1
ENSXETG00000016421	1839	269	387	313	504	471	601	1325	1489	914	781	569	593	nf2
ENSXETG00000016422	543	0	0	0	0	0	0	4	2	0	1	0	1	cabp7
ENSXETG00000016423	2368	578	885	782	1096	844	1825	1284	1479	905	1781	1274	1416	ascc2
ENSXETG00000016426	3318	306	473	402	1249	952	668	185	174	120	122	85	94	ca14
ENSXETG00000016428	3606	190	296	242	711	534	974	781	837	520	971	755	775	mtmr3
ENSXETG00000016431	2544	89	119	127	154	126	243	645	783	455	473	338	339	fcho1
ENSXETG00000016434	7593	3	1	2	8	5	7	17	14	5	27	18	14	TENM2
ENSXETG00000016435	930	71	100	69	230	190	344	293	339	244	200	162	177	arfip2
ENSXETG00000016436	2006	11	25	18	206	157	40	1646	1939	1131	222	144	169	XB-GENE-996589
ENSXETG00000016437	474	0	0	0	0	0	0	0	0	0	0	0	0	
ENSXETG00000016438	3650	76	105	81	70	69	110	533	593	321	259	204	213	jak3
ENSXETG00000016439	1567	1211	1782	1491	2362	1880	3627	3701	4179	2606	2214	1646	1774	aph1a
ENSXETG00000016442	2766	1644	2304	1892	2640	1924	4179	1404	1627	986	2982	2088	2286	mgc107841
ENSXETG00000016443	2186	101	146	159	131	104	347	174	211	129	274	192	205	gatsl3
ENSXETG00000016448	6654	166	268	220	868	680	660	833	912	613	876	603	670	itpr2
ENSXETG00000016462	3225	466	667	604	2047	1638	1793	712	822	480	2477	1836	2065	wwc1
ENSXETG00000016463	2475	55	72	64	225	170	503	973	1169	709	1103	816	877	eml2
ENSXETG00000016464	2475	1	0	1	2	4	3	9	19	8	0	1	0	pkdcc.1
ENSXETG00000016466	1032	0	0	0	16	9	18	32	20	16	22	9	23	ENSXETG00000016466
ENSXETG00000016467	6256	22	26	31	171	140	107	652	686	466	145	97	115	nin
ENSXETG00000016468	2525	3	7	8	3	4	0	3	2	4	22	16	24	ENSXETG00000016468
ENSXETG00000016471	939	562	872	689	819	631	990	1958	2102	1361	996	681	800	casp7
ENSXETG00000016474	2952	14	15	19	16	13	22	377	423	221	34	31	27	lzts1
ENSXETG00000016476	3501	2473	3543	3006	3629	2751	11737	10382	11900	6953	3552	2500	2679	atp6v1b2
ENSXETG00000016477	2594	4885	7111	6124	7098	5278	8763	5862	6825	3975	6928	4878	5297	pygl
ENSXETG00000016478	1608	14	41	28	67	45	63	517	628	398	83	74	84	klf13
ENSXETG00000016479	4909	0	1	2	4	2	7	22	21	16	18	14	15	trpm1

ENSXETG00000016480	777	329	552	424	242	218	267	72	70	40	94	66	64	gjb5
ENSXETG00000016481	2027	304	416	310	522	401	667	277	341	201	646	429	486	gjb3
ENSXETG00000016483	1482	0	0	0	0	1	1	0	0	0	0	0	0	
ENSXETG00000016484	930	1	2	0	4	5	1	2	0	1	2	1	2	ENSXETG00000016484
ENSXETG00000016485	2412	1	0	1	1	0	4	0	0	0	0	0	0	slc18a1
ENSXETG00000016486	1520	424	614	533	227	164	255	425	533	309	352	280	275	smim12
ENSXETG00000016487	2139	0	1	0	3	2	4	55	60	47	12	7	8	trim9
ENSXETG00000016489	2315	836	1181	999	1330	999	1799	2532	3067	1667	1900	1341	1426	tmx1
ENSXETG00000016491	1569	8	5	8	37	38	91	225	283	146	91	65	63	FRMD6
ENSXETG00000016492	1017	16	30	29	56	35	85	430	461	251	146	94	88	gng2
ENSXETG00000016493	2342	0	0	0	0	0	0	1	0	0	0	1	0	
ENSXETG00000016494	4788	128	163	122	322	270	268	526	619	366	549	402	431	mtmr10
ENSXETG00000016495	1364	1534	2226	1934	1023	781	1454	2062	2372	1346	1960	1449	1566	mak16
ENSXETG00000016496	3334	210	295	269	333	255	326	325	391	216	325	242	230	fan1
ENSXETG00000016497	2184	858	1209	1064	928	734	1074	1291	1568	952	1456	950	1111	mphosph10
ENSXETG00000016499	549	0	0	0	0	0	0	0	0	0	0	0	0	
ENSXETG00000016500	1572	427	645	499	926	684	904	1334	1519	1004	939	655	644	asun
ENSXETG00000016501	936	0	0	0	0	0	0	0	0	0	0	0	0	
ENSXETG00000016502	1348	590	889	694	660	514	914	1027	1074	669	872	659	733	fgfr1op2
ENSXETG00000016503	2259	1	0	2	9	9	13	33	53	24	12	12	11	apba2
ENSXETG00000016505	1417	130	177	117	226	158	314	498	549	336	310	217	228	endou
ENSXETG00000016509	2592	1157	1816	1539	3367	2444	5517	3841	4091	2497	4323	3053	3250	ik
ENSXETG00000016510	924	0	0	0	0	0	0	0	0	0	0	0	0	
ENSXETG00000016511	3124	0	0	0	0	3	2	23	22	13	7	4	5	fam189a1
ENSXETG00000016512	957	0	0	0	0	0	0	0	0	0	0	0	0	
ENSXETG00000016513	933	500	678	634	1580	1266	1867	1817	1924	1215	2683	1912	2145	cyb5r3
ENSXETG00000016514	948	0	0	0	0	0	0	0	0	0	0	0	0	
ENSXETG00000016515	912	0	0	0	0	0	0	0	0	0	0	0	0	
ENSXETG00000016517	933	0	0	0	0	0	0	0	0	0	0	0	0	
ENSXETG00000016519	945	0	0	0	0	0	0	0	0	0	0	0	0	
ENSXETG00000016521	705	7	8	5	17	13	7	5	7	0	14	10	8	rep15
ENSXETG00000016522	3018	135	183	151	714	597	762	1872	1869	1312	1839	1217	1319	ppfibp1
ENSXETG00000016523	972	0	0	0	0	0	0	0	0	0	0	0	0	
ENSXETG00000016524	960	0	0	0	0	0	0	0	0	0	0	0	0	
ENSXETG00000016526	2018	2	3	0	3	1	3	29	37	34	2	5	2	ugdh.2
ENSXETG00000016536	1777	372	489	429	853	678	1073	1203	1469	921	1103	772	882	slu7
ENSXETG00000016541	870	0	0	0	0	0	0	0	0	0	0	0	0	
ENSXETG00000016542	2484	130	163	128	126	87	57	373	505	309	67	44	75	nudt1
ENSXETG00000016544	1542	4	6	3	228	197	521	2	4	3	233	150	184	ENSXETG00000016544
ENSXETG00000016554	2886	173	245	186	242	175	331	405	518	311	356	227	291	nedd4
ENSXETG00000016556	2128	7	13	20	231	185	32	114	122	72	26	16	27	mns1
ENSXETG00000016560	3150	49	66	75	145	126	198	240	303	165	222	188	196	znf280d
ENSXETG00000016563	2217	122	133	129	295	237	520	1882	2077	1163	783	583	626	tcf12
ENSXETG00000016564	609	41	63	45	0	1	1	31	23	17	2	0	0	ENSXETG00000016564
ENSXETG00000016565	3485	0	0	3	39	39	64	193	189	114	74	67	52	islr2
ENSXETG00000016566	732	0	0	0	9	7	14	3	2	4	105	69	91	ENSXETG00000016566
ENSXETG00000016570	2169	87	149	126	193	148	205	500	547	346	370	253	285	tap2
ENSXETG00000016572	1294	0	1	1	30	15	6	37	40	24	11	10	4	dlx5
ENSXETG00000016573	3418	0	0	0	0	0	0	2	5	4	4	5	2	
ENSXETG00000016574	668	1205	1820	1536	1545	1196	1474	2123	2388	1519	1424	956	1075	tbca
ENSXETG00000016575	441	565	833	699	527	395	558	816	977	621	387	226	358	sem1
ENSXETG00000016576	4444	614	795	712	816	618	835	1367	1526	868	981	722	758	slc25a13
ENSXETG00000016577	2181	84	136	129	101	72	96	437	507	301	216	129	168	tap1
ENSXETG00000016579	2094	637	818	679	3056	2365	5330	7346	7961	4670	4530	3082	3536	pdlim4
ENSXETG00000016581	1653	661	925	795	1026	832	1601	438	526	332	873	642	750	slc22a5
ENSXETG00000016582	4584	33	46	43	103	78	44	175	202	135	64	32	49	recql4
ENSXETG00000016583	2036	112	209	173	345	265	567	533	564	409	527	339	380	znf630
ENSXETG00000016584	3449	557	758	679	859	587	823	625	698	477	664	451	536	slc22a4
ENSXETG00000016585	1542	7	12	5	23	30	30	145	172	108	444	283	314	slc7a5
ENSXETG00000016586	1810	600	861	732	1894	1356	1165	9438	11085	6210	2264	1538	1697	irf1
ENSXETG00000016587	2869	12837	18276	15296	5632	4366	4629	785	936	515	581	403	439	hmgcs1
ENSXETG00000016588	2527	1	3	1	0	1	1	20	26	14	1	0	5	acsl6
ENSXETG00000016590	666	64	102	89	100	80	251	130	144	90	117	58	83	tmem267
ENSXETG00000016591	3285	3404	4995	4142	8158	6355	9021	6924	7540	4538	8000	5835	6197	nnt
ENSXETG00000016592	1119	417	649	532	728	541	550	3836	4529	2620	825	548	615	psmb8
ENSXETG00000016594	2024	268	376	297	1765	1385	2247	332	434	238	1182	812	957	ca5b
ENSXETG00000016596	1512	5	13	8	40	24	60	107	91	57	43	17	30	plxdc2
ENSXETG00000016597	3498	70	101	92	365	285	1335	1538	1772	1047	1527	1091	1231	fnip1
ENSXETG00000016599	2913	2	4	3	13	12	19	33	29	19	26	12	18	nebl
ENSXETG00000016600	1599	247	419	369	614	392	776	1079	1320	802	905	654	705	banp
ENSXETG00000016605	702	1	0	2	0	1	1	4	2	2	0	0	0	ENSXETG00000016605
ENSXETG00000016607	960	0	0	0	0	0	0	0	0	0	0	0	0	
ENSXETG00000016608	2492	1438	2072	1767	2529	1955	5929	4232	4900	2995	2312	1620	1827	galc
ENSXETG00000016610	1611	0	0	0	3	3	2	14	19	11	9	6	3	kcnk10
ENSXETG00000016612	1523	0	2	0	13	7	2	0	1	0	20	5	13	gnrhr
ENSXETG00000016613	1626	436	565	519	521	420	1252	765	902	486	1269	806	965	bfar
ENSXETG00000016614	1869	1556	2292	1845	1958	1532	7547	5659	6876	3956	3391	2490	2818	naga
ENSXETG00000016617	3619	151	207	186	289	193	449	552	673	332	422	238	325	zc3h7a
ENSXETG00000016618	897	3	7	7	17	13	23	52	43	30	27	19	24	ngf
ENSXETG00000016619	1326	0	0	0	0	1	1	0	1	1	0	4	5	ENSXETG00000016619
ENSXETG00000016620	2691	426	532	441	559	413	565	292	338	206	390	282	285	txndc11
ENSXETG00000016621	2661	110	166	143	349	292	739	1876	2312	1254	959	678	756	tspan2
ENSXETG00000016622	1776	84	97	79	74	49	310	943	1083	647	215	124	174	snn
ENSXETG00000016625	2188	212	330	282	375	309	606	769	878	484	521	360	392	stk38
ENSXETG00000016627	6715	159	250	225	38	21	52	45	70	33	23	22	16	EML5
ENSXETG00000016630	453	48	109	61	156	90	128	172	195	127	139	91	101	rmi2
ENSXETG00000016634	1516	306	487	396	453	382	469	801	878	563	399	264	296	pofut1
ENSXETG00000016635	2496	100	129	114	183	142	335	249	326	175	204	149	151	ttc8
ENSXETG00000016636	1788	198	272	243	479	379	466	738	755	471	473	298	391	kif3b
ENSXETG00000016639	2971	3	4	3	41	24	87	209	274	139	298	204	244	lsamp
ENSXETG00000016645	1814	83	121	97	290	166	549	720	866	497	772	558	643	foxn3
ENSXETG00000016646	819	15	29	28	35	20	32	56	66	31	74	49	67	znf805
ENSXETG00000016651	1122	0	3	0	1	0	1	2	1	1	3	1	0	prmt8
ENSXETG00000016655	785	9128	13441	11866	10441	7640	12257	8538	9283	5631	13067	9123	9948	atp5g1
ENSXETG00000016659	2089	37	65	44	95	65	72	236	301	158	31	20	30	eri2
ENSXETG00000016660	1558	375	575	488	509	392	764	896	1051	603	738	462	562	mettl9
ENSXETG00000016661	1380	63	101	106	76	54	161	191	211	134	122	90	107	ENSXETG00000016661
ENSXETG00000016662	2095	175	253	205	108	68	161	141	141	84	78	52	60	cdr2
ENSXETG00000016663	2194	934	1411	1207	1038	793	1336	1128	1427	797	831	631	762	polr3e
ENSXETG00000016664	951	0	0	0	0	0	0	0	0	0	0	0	0	
ENSXETG00000016667	666	0	0	0	0	0	0	0	0	0	0	0	0	
ENSXETG00000016673	942	0	0	0	0	0	0	0	0	0	0	0	0	
ENSXETG00000016677	7095	251	423	305	1146	866	3170	2021	2404	1332	3082	2219	2453	eef2k
ENSXETG00000016679	960	0	0	0	0	0	0	0	0	0	0	0	0	
ENSXETG00000016680	972	0	0	0	0	0	0	0	0	0	0	0	0	
ENSXETG00000016681	2973	164	253	209	597	464	746	845	990	587	757	500	579	zswim6
ENSXETG00000016684	486	64	83	85	60	53	113	78	78	55	88	58	59	ndufaf2
ENSXETG00000016685	3538	83	129	123	241	209	391	1486	1714	1047	335	226	278	hps5
ENSXETG00000016687	2385	56	82	59	658	467	1202	2461	2818	1621	493	380	400	cdh6
ENSXETG00000016688	1594	1384	1965	1602	2173	1563	1789	1758	1989	1117	1102	774	911	elovl7
ENSXETG00000016693	1603	6768	9746	8241	7950	6044	9647	6574	7865	4553	5190	3799	4159	uqcrc2

ENSXETG00000016695	578	32	40	36	46	41	77	200	259	126	79	50	66	c16orf52
ENSXETG00000016698	1171	126	165	147	91	76	34	165	221	129	63	54	63	crym
ENSXETG00000016701	4304	152	238	210	392	279	310	733	885	572	291	202	253	drosha
ENSXETG00000016704	2142	27	35	25	21	19	20	4	11	7	10	6	9	zp2
ENSXETG00000016710	2397	1	5	4	94	73	4	19	20	6	15	3	5	ENSXETG00000016710
ENSXETG00000016712	492	14	25	17	27	12	40	138	195	100	82	70	89	tmem159
ENSXETG00000016718	1200	1	1	1	25	17	30	1760	1845	1157	287	185	231	nfix
ENSXETG00000016721	645	50	78	72	37	24	103	146	198	115	56	37	56	lyl1
ENSXETG00000016725	2474	8216	12054	10232	10316	8049	39783	18447	21356	12741	16600	11551	12796	acp5
ENSXETG00000016727	369	27	36	38	30	19	40	98	109	67	83	64	60	lyrm1
ENSXETG00000016729	1892	124	165	110	212	153	312	536	613	353	295	226	246	dcun1d3
ENSXETG00000016731	186	683	927	781	725	536	766	848	963	590	695	449	498	elof1
ENSXETG00000016732	2847	3659	5078	4288	5433	4431	20707	19871	22876	13218	22983	16098	17931	cnn2
ENSXETG00000016734	1822	3660	5545	4514	4911	3662	7157	414	462	301	2198	1493	1708	ENSXETG00000016734
ENSXETG00000016735	2187	119	185	154	189	151	192	260	304	173	303	227	260	REXO5
ENSXETG00000016736	1200	3911	5621	4862	3780	2747	2008	540	671	386	1519	1100	1258	tmem56.2
ENSXETG00000016738	2093	471	661	565	484	387	688	710	783	490	713	485	531	ecsit
ENSXETG00000016739	1689	38	88	57	118	89	115	262	325	204	175	121	121	znf653
ENSXETG00000016740	1749	240	334	266	550	418	783	206	268	169	983	719	747	acsm3
ENSXETG00000016741	1679	0	0	1	6	3	6	46	55	31	15	20	11	elavl3
ENSXETG00000016743	1890	0	0	0	0	0	0	0	0	0	0	0	0	
ENSXETG00000016744	1998	2691	3975	3426	4154	3323	3169	3785	4294	2653	2219	1612	1756	prkcsh
ENSXETG00000016745	1988	11	15	14	427	325	47	64	87	34	41	23	38	ccdc151
ENSXETG00000016746	2562	0	0	0	0	0	0	0	0	0	0	0	0	
ENSXETG00000016747	2412	30	46	52	142	114	244	599	685	420	325	239	265	rgl3
ENSXETG00000016749	1755	161	257	212	162	130	90	167	180	101	508	383	415	eppk1
ENSXETG00000016751	444	0	0	0	0	0	0	0	1	1	0	0	0	
ENSXETG00000016752	1605	29	40	39	227	186	195	664	803	474	186	142	140	tspan16
ENSXETG00000016758	3077	518	755	655	1304	1040	1975	577	630	386	3074	2082	2390	XB-GENE-1002559
ENSXETG00000016759	1419	302	382	374	422	317	592	740	941	465	555	432	471	gkap1
ENSXETG00000016761	1928	28	34	24	96	105	49	137	167	112	74	61	71	cep19
ENSXETG00000016764	3098	2137	3092	2597	1628	1297	2801	3528	4065	2318	1854	1342	1471	ubqln1
ENSXETG00000016765	1190	319	467	387	260	180	347	304	334	211	298	222	222	sh3yl1
ENSXETG00000016766	978	17	29	27	88	70	129	148	164	110	134	109	101	fam110c
ENSXETG00000016768	2423	1245	1712	1437	3820	3059	8598	1876	2278	1375	5573	3879	4282	fbxo25
ENSXETG00000016769	3370	1553	2281	1955	1968	1500	2127	1983	2267	1391	1540	1054	1224	hs6st1
ENSXETG00000016770	4630	454	727	643	860	655	914	1175	1348	809	527	433	464	uggt1
ENSXETG00000016771	1331	34	59	41	74	54	147	58	50	33	94	75	60	idnk
ENSXETG00000016772	2907	7	7	4	23	10	32	113	122	77	57	27	40	frmd3
ENSXETG00000016774	1230	296	404	390	385	254	578	791	923	509	406	297	328	azi2
ENSXETG00000016775	4102	567	840	708	1713	1270	3036	2245	2620	1606	3033	2082	2342	fgfr1
ENSXETG00000016778	1098	0	0	0	0	0	0	0	0	0	0	0	0	
ENSXETG00000016779	1158	0	0	0	0	0	0	0	0	0	0	0	0	
ENSXETG00000016781	3211	32	63	48	103	82	144	331	389	223	210	153	175	ENSXETG00000016781
ENSXETG00000016782	2562	296	434	384	667	487	1876	1192	1597	823	1341	937	1078	vps41
ENSXETG00000016784	1014	0	0	0	0	0	0	0	0	0	0	0	0	ENSXETG00000016784
ENSXETG00000016786	1728	113	155	127	226	153	495	364	411	238	384	268	274	znf180
ENSXETG00000016788	2537	251	379	319	445	355	611	780	883	527	524	357	406	polb
ENSXETG00000016789	1584	3888	5678	4872	6496	5055	5097	4917	5373	3260	4015	2745	2945	vdac3
ENSXETG00000016790	2970	722	1021	886	1257	947	1576	1485	1606	1039	1651	1119	1268	ikbkb
ENSXETG00000016792	2467	19	44	46	258	185	280	1370	1468	941	330	274	263	plat
ENSXETG00000016795	785	632	966	828	746	600	614	1093	1134	671	1013	809	781	imp4
ENSXETG00000016796	1838	2639	3791	3439	2651	2010	3875	3370	3747	2317	2479	1685	1811	ap3m2
ENSXETG00000016798	7592	54	76	54	63	64	160	190	237	143	170	125	152	ank1
ENSXETG00000016802	774	27	21	23	1235	1068	78	1	2	0	2291	1621	1792	nkx6-3
ENSXETG00000016804	1379	1638	2440	2082	2082	1474	2042	441	525	284	553	420	463	inpp5a.2
ENSXETG00000016807	2863	944	1309	1176	1502	1141	1803	2636	2946	1804	1544	979	1128	gpat4
ENSXETG00000016808	1324	62	65	62	337	263	441	179	204	105	40	22	23	ppp1r3c.2
ENSXETG00000016809	1643	461	663	574	1081	823	493	1238	1547	866	645	464	488	gins4
ENSXETG00000016810	1269	383	595	510	447	327	533	902	1087	599	579	434	482	chmp7
ENSXETG00000016812	2661	270	380	334	549	423	542	856	1015	632	477	371	427	tnpo1
ENSXETG00000016813	780	66	101	98	115	83	173	165	181	121	150	103	121	dcun1d1
ENSXETG00000016814	5006	1218	1777	1470	1383	1163	2638	1941	2309	1368	2108	1551	1674	atp11b
ENSXETG00000016815	6393	89	118	121	250	181	340	416	497	285	305	240	264	fcho2
ENSXETG00000016816	2690	4713	6531	5325	4523	3473	10109	1851	2279	1307	4129	2814	3160	tmem174
ENSXETG00000016818	990	119	184	168	463	344	413	311	302	208	580	438	406	foxd1
ENSXETG00000016819	902	15328	22558	19209	22210	16696	22097	19001	22592	13227	64298	45705	49883	btf3
ENSXETG00000016820	1625	83	127	84	141	136	211	365	469	262	179	121	138	ankra2
ENSXETG00000016821	2203	831	1273	1099	864	621	701	1136	1346	725	1023	676	789	utp15
ENSXETG00000016822	1011	1	2	1	4	7	2	8	7	5	10	2	2	ENSXETG00000016822
ENSXETG00000016823	561	111	133	105	106	71	181	209	268	146	171	114	150	dcun1d2
ENSXETG00000016824	4722	69	105	101	284	228	374	499	587	393	359	266	337	arhgef28
ENSXETG00000016826	2356	758	1115	1000	1406	1058	1643	1237	1497	831	1413	958	1116	mccc1
ENSXETG00000016827	1701	25	33	32	60	49	103	153	200	90	115	105	119	scai
ENSXETG00000016831	4605	863	1249	1026	1528	1118	2357	2955	3637	1972	1637	1124	1168	ppp6c
ENSXETG00000016833	3557	0	0	0	0	0	0	0	0	0	0	0	0	
ENSXETG00000016835	3889	0	0	0	0	0	0	1	1	0	1	0	0	
ENSXETG00000016836	3447	0	0	0	0	0	0	3	4	3	4	0	2	atp13a4
ENSXETG00000016838	1968	23845	35228	31316	6827	5414	5811	12677	14510	8662	2665	1942	2190	hspa5
ENSXETG00000016839	1122	47	90	74	133	111	223	242	256	167	190	106	126	rabepk
ENSXETG00000016840	2508	316	467	422	438	300	713	768	932	552	553	387	449	golga1
ENSXETG00000016841	3391	534	812	726	879	666	1221	1078	1311	800	795	578	590	opa1
ENSXETG00000016842	1227	1867	2678	2203	2071	1604	1499	2257	2560	1404	1519	1130	1199	arpc5l
ENSXETG00000016843	409	32794	47979	41664	43810	32558	43029	40910	47036	27816	81264	58608	63344	rpl35
ENSXETG00000016844	879	0	3	0	32	37	5	20	24	13	4	6	1	wdr38
ENSXETG00000016846	912	0	0	0	0	0	1	0	0	0	0	0	0	ENSXETG00000016846
ENSXETG00000016850	3313	47	66	48	289	209	474	3160	3701	2138	2234	1495	1724	prnp
ENSXETG00000016851	2161	659	932	872	418	321	1026	4116	4800	2804	801	601	639	rassf2
ENSXETG00000016852	1287	179	287	232	1177	911	1836	1741	1973	1115	1411	988	1091	hes1
ENSXETG00000016853	5346	504	721	590	1258	924	819	1759	2034	1214	480	351	375	slc23a2
ENSXETG00000016857	1108	0	0	1	0	0	4	9	13	10	4	3	3	tenm2
ENSXETG00000016861	1508	0	2	1	2	2	2	3	5	1	4	4	4	cbln2
ENSXETG00000016864	1590	0	1	0	0	0	0	23	22	13	0	0	1	
ENSXETG00000016868	2714	2	2	1	4	2	18	47	38	30	7	10	13	kctd15
ENSXETG00000016869	2416	0	2	2	7	6	9	72	91	58	27	20	18	lgi4
ENSXETG00000016870	1740	0	0	0	0	0	0	1	0	1	1	0	1	hpn
ENSXETG00000016872	987	150	209	236	138	99	168	159	164	129	214	140	161	ENSXETG00000016872
ENSXETG00000016876	1443	4092	5806	4934	3069	2373	12892	738	791	567	487	331	381	slc39a4
ENSXETG00000016877	720	1	4	4	0	0	2	5	9	7	1	0	0	siglec12
ENSXETG00000016879	873	0	0	0	0	0	0	0	1	0	0	0	0	
ENSXETG00000016881	1405	175	247	254	361	298	756	361	405	285	660	476	521	mfsd3
ENSXETG00000016882	2992	390	569	478	925	729	1513	1442	1604	1030	964	672	763	larp4b
ENSXETG00000016884	2952	360	549	440	905	721	1730	1884	2124	1220	2286	1552	1802	zmynd11
ENSXETG00000016887	2451	7	8	15	48	39	124	520	627	384	212	137	203	nfatc4
ENSXETG00000016889	3159	572	810	679	769	580	1784	1123	1311	737	1298	875	942	adcy4
ENSXETG00000016896	2991	276	446	364	784	627	887	1197	1324	737	1410	942	1068	elf2
ENSXETG00000016899	1969	549	823	715	267	231	1201	517	510	321	192	143	143	noct
ENSXETG00000016900	2968	1079	1596	1326	1402	1168	2203	1806	2220	1294	2105	1493	1720	naa15
ENSXETG00000016901	1692	56	67	57	156	134	47	5	5	6	23	15	5	ENSXETG00000016901
ENSXETG00000016903	2505	236	310	257	580	418	638	866	1075	653	817	506	600	khnyn
ENSXETG00000016904	1806	18	41	24	153	116	228	2234	2553	1620	453	405	400	hpgd

ENSXETG00000016906	1508	373	425	423	406	326	1022	498	571	344	403	286	320	fbxo8
ENSXETG00000016907	726	0	0	0	0	0	0	14	8	8	2	3	0	ENSXETG00000016907
ENSXETG00000016908	1847	45	71	58	337	238	424	1643	2067	1141	782	613	666	hand2
ENSXETG00000016909	1265	24	36	27	31	33	41	119	129	71	43	30	34	kiaa1456
ENSXETG00000016915	806	120	193	137	163	139	316	400	467	266	269	170	196	sap30
ENSXETG00000016916	435	2464	3634	3201	6064	4520	4896	12082	13601	7963	3866	2781	3065	hmgb2
ENSXETG00000016917	2718	120	212	186	427	295	455	309	392	278	345	261	288	galnt7
ENSXETG00000016918	1071	173	258	207	242	191	149	439	522	305	157	117	136	rfc3
ENSXETG00000016920	2328	108	193	177	210	156	336	246	319	168	175	119	135	rab33b
ENSXETG00000016922	870	0	0	0	0	0	1	25	21	14	2	5	2	ENSXETG00000016922
ENSXETG00000016923	3087	2	4	3	0	0	0	8	4	6	0	0	0	
ENSXETG00000016927	1602	0	2	3	2	4	9	35	49	26	5	1	4	mgc69520
ENSXETG00000016928	3961	148	262	237	689	486	789	1448	1624	1011	907	664	714	stard13
ENSXETG00000016929	1478	283	363	336	363	283	482	294	320	202	367	245	287	sdr39u1
ENSXETG00000016930	3949	69	104	63	470	372	373	796	924	596	480	349	331	cep131
ENSXETG00000016931	624	0	0	0	0	0	0	0	0	0	2	0	0	
ENSXETG00000016932	3539	2782	3781	3194	3932	3065	3026	1106	1308	745	2373	1622	1833	pnp
ENSXETG00000016933	4006	328	430	381	1217	930	1363	2306	2565	1661	2228	1582	1665	ehmt1
ENSXETG00000016934	2118	122	208	165	284	260	376	733	815	449	545	395	424	shkbp1
ENSXETG00000016937	6627	102	158	119	244	185	326	385	437	294	464	293	366	sorl1
ENSXETG00000016938	702	75	92	96	123	86	146	213	224	149	121	93	101	mnat1
ENSXETG00000016939	2114	386	515	524	340	240	293	315	399	201	314	224	215	setd7
ENSXETG00000016941	2220	76	104	88	198	145	256	238	305	163	346	270	256	six4
ENSXETG00000016943	1412	0	0	0	0	1	0	0	1	0	0	2	0	six1
ENSXETG00000016945	1076	0	0	0	0	0	0	0	0	0	0	0	0	
ENSXETG00000016946	1592	644	1022	895	486	395	988	1250	1452	888	546	372	397	dnaja4.1
ENSXETG00000016948	1305	0	0	0	0	0	0	0	0	0	0	0	0	
ENSXETG00000016949	1290	83	142	140	342	257	695	533	597	398	431	281	321	arrdc1
ENSXETG00000016950	2841	0	0	0	0	0	0	0	0	0	0	0	0	
ENSXETG00000016951	3785	1654	2367	2094	4188	3277	1301	1484	1709	1032	3225	2259	2393	cry1
ENSXETG00000016952	3332	4536	6350	5567	5720	4444	7875	6407	7379	4345	5430	3724	4235	ppm1a
ENSXETG00000016954	1669	515	755	633	617	454	743	1032	1175	636	876	591	690	c19orf47
ENSXETG00000016955	2041	1892	2859	2399	2022	1596	4135	698	891	481	1959	1293	1516	dhrs7
ENSXETG00000016956	3549	172	253	205	303	259	416	297	442	211	445	290	340	pcnx4
ENSXETG00000016957	906	1153	1671	1485	1640	1200	1630	2869	3450	2089	1178	837	971	blvrb
ENSXETG00000016961	2313	771	1087	879	1009	856	1766	6831	8495	4783	1749	1222	1486	pld3
ENSXETG00000016963	1435	897	1318	1054	836	670	846	858	1008	626	985	683	722	mrpl38
ENSXETG00000016964	3312	8	16	16	28	33	129	133	131	89	74	52	66	btbd11
ENSXETG00000016965	3604	29	30	33	158	125	334	346	410	250	418	245	285	maml3
ENSXETG00000016967	4317	6	12	8	114	87	138	1223	1379	831	786	505	545	ltbp4
ENSXETG00000016972	2985	177	216	220	815	567	1512	17	18	9	617	472	511	kl
ENSXETG00000016973	312	154	201	154	204	137	371	405	497	297	282	196	223	scoc
ENSXETG00000016974	1150	4693	6822	5980	2277	1771	3380	9719	11359	6598	4583	3336	3597	ldhb
ENSXETG00000016975	1515	673	952	834	614	440	477	785	901	518	742	545	538	pwp1
ENSXETG00000016981	4598	0	2	1	42	42	8	0	2	2	1	2	1	lrrc9
ENSXETG00000016982	912	11	22	21	11	8	41	124	154	79	74	47	65	ctsg
ENSXETG00000016983	3264	7	4	5	37	17	47	361	429	240	164	127	141	rtn1
ENSXETG00000016985	1191	19	15	8	26	26	31	216	206	130	51	33	36	tspan9
ENSXETG00000016986	3262	479	681	572	1013	759	1190	1657	2049	1117	1651	1143	1327	akt2
ENSXETG00000016987	2862	1000	1435	1217	1676	1340	2647	6129	7337	4031	3918	2826	3133	zfp36l1
ENSXETG00000016988	816	2	2	1	1	0	2	0	2	0	0	1	0	ccnb1ip1
ENSXETG00000016989	1831	201	319	270	315	264	601	734	830	531	611	387	449	ttc5
ENSXETG00000016992	603	22	30	19	33	37	121	239	272	140	46	29	38	tmem121
ENSXETG00000016993	510	250	334	270	244	191	419	550	725	417	484	360	438	ENSXETG00000016993
ENSXETG00000016994	1583	203	286	228	536	425	535	710	858	565	527	413	475	lpcat4
ENSXETG00000016996	4942	785	1064	967	1620	1213	2006	3458	3916	2375	2197	1617	1708	slc12a6
ENSXETG00000016998	3009	350	612	462	906	790	1816	1149	1309	784	1130	767	906	map3k10
ENSXETG00000017000	5589	87	125	110	359	278	452	664	829	462	532	407	442	pds5b
ENSXETG00000017003	719	2365	3575	2975	2084	1634	3110	2632	3009	1677	1870	1231	1450	emc4
ENSXETG00000017004	4774	1334	1858	1536	1998	1529	5757	2313	2804	1617	2393	1664	1890	tmem55b
ENSXETG00000017006	1252	523	764	651	669	526	641	959	1031	575	622	425	444	osgep
ENSXETG00000017009	2831	140	220	185	258	221	663	548	599	332	749	544	509	n4bp2l1
ENSXETG00000017011	9944	36	54	41	167	115	46	183	213	112	53	40	60	brca2
ENSXETG00000017014	1959	2403	3422	2951	717	539	1583	2033	2232	1376	1326	916	942	oaf
ENSXETG00000017016	822	2	6	3	4	7	9	32	27	17	7	3	4	XB-GENE-5931039
ENSXETG00000017018	4833	878	1314	1063	1894	1461	2866	2829	3363	2066	1998	1412	1577	arhgef12
ENSXETG00000017024	1527	285	372	321	290	205	474	515	620	362	306	205	276	jkamp
ENSXETG00000017026	3959	114	166	115	200	156	430	801	908	566	414	293	318	l3hypdh
ENSXETG00000017029	1425	0	5	0	10	5	12	40	45	41	20	12	16	gpr135
ENSXETG00000017030	3355	355	492	467	818	678	1697	1180	1422	814	1072	800	892	daam1
ENSXETG00000017031	2823	0	0	0	1	3	0	7	25	10	10	8	8	grik4
ENSXETG00000017032	2406	3	2	2	18	17	44	85	135	63	54	45	41	tll1
ENSXETG00000017033	1455	8	16	22	47	34	39	71	98	45	90	54	68	ENSXETG00000017033
ENSXETG00000017037	1660	506	725	679	846	662	951	3379	3954	2382	769	543	567	cpe
ENSXETG00000017038	1527	7	19	15	130	85	209	31	35	18	287	204	207	ENSXETG00000017038
ENSXETG00000017042	1667	312	490	393	674	558	580	545	670	372	535	397	444	trim45
ENSXETG00000017044	1329	210	322	276	239	188	561	15	20	10	133	84	107	ENSXETG00000017044
ENSXETG00000017046	732	7	17	6	23	12	53	32	24	18	8	11	14	tmem136
ENSXETG00000017048	6429	1	3	2	4	7	4	0	2	2	2	1	0	tecta.1
ENSXETG00000017056	798	0	0	0	0	0	0	6	5	7	0	1	0	acr
ENSXETG00000017061	1986	294	391	284	312	250	836	570	718	421	426	308	333	tmem192
ENSXETG00000017063	780	0	0	0	0	0	0	0	0	0	0	0	0	
ENSXETG00000017065	2107	1545	2273	1938	983	755	929	8063	9358	5396	4406	3226	3501	mhc1a
ENSXETG00000017066	753	320	456	351	80	66	153	1357	1636	862	953	719	783	mhc2a
ENSXETG00000017067	3601	1459	2170	1834	2743	2098	3475	3678	4349	2494	3813	2616	2992	brd2
ENSXETG00000017069	2492	144	222	195	370	295	325	583	731	420	86	48	74	shcbp1
ENSXETG00000017070	4419	43	69	45	21	19	43	155	146	93	8	13	10	a2ml1
ENSXETG00000017073	888	0	0	0	0	0	0	0	0	0	0	0	0	
ENSXETG00000017074	4121	141	212	180	473	379	1003	931	1107	635	585	415	423	wdr41
ENSXETG00000017075	2334	1	2	3	7	4	15	96	116	73	57	48	33	pde8b
ENSXETG00000017077	2190	214	341	301	412	279	521	606	664	389	547	358	423	aggf1
ENSXETG00000017078	303	0	1	0	3	3	1	2	4	4	3	6	7	s100z
ENSXETG00000017080	966	0	0	0	8	5	0	6	10	7	15	13	17	crhbp
ENSXETG00000017085	2181	37	59	32	265	224	236	76	83	41	204	141	140	pkp1
ENSXETG00000017086	819	361	500	487	64	54	167	548	636	392	276	217	235	tnnt2
ENSXETG00000017087	819	16	18	13	5	3	0	45	42	32	3	0	3	tnni1
ENSXETG00000017088	1028	1136	1582	1328	1657	1290	4407	6445	7553	4520	6081	4328	4617	csrp1
ENSXETG00000017089	5217	16	15	27	94	63	161	665	746	491	266	168	210	nav1
ENSXETG00000017090	3879	435	611	499	1169	937	1544	1343	1654	932	1527	1063	1258	prag1
ENSXETG00000017094	1362	0	0	0	2	2	0	9	8	9	2	0	0	syt2
ENSXETG00000017095	3202	141	224	198	378	295	718	1195	1363	815	1071	814	866	ppp1r12b
ENSXETG00000017097	1319	736	1067	856	592	446	410	1113	1399	723	435	298	334	ppil1
ENSXETG00000017098	2044	0	0	1	0	0	0	2	2	0	1	0	2	cpne9
ENSXETG00000017099	4883	40	48	45	61	40	259	605	690	469	207	136	180	kif21b
ENSXETG00000017100	1659	0	4	1	2	0	1	22	20	21	0	0	0	ENSXETG00000017100
ENSXETG00000017102	2091	0	0	1	0	0	0	1	3	5	8	5	10	
ENSXETG00000017103	1425	0	0	0	0	0	0	0	1	0	0	0	0	
ENSXETG00000017105	4215	1223	1745	1451	3042	2424	3134	1511	1866	1045	2592	1883	2240	c1orf116
ENSXETG00000017106	915	21	48	35	47	36	71	102	127	84	75	36	38	yod1
ENSXETG00000017108	2319	244	358	334	198	150	328	237	273	153	240	178	189	pfkfb2
ENSXETG00000017109	1536	248	384	324	732	521	2745	1373	1737	962	661	487	529	ctsf

ENSXETG00000017110	1752	382	498	437	521	385	953	1138	1477	787	1096	760	974	arc3
ENSXETG00000017114	2562	2	4	1	11	7	16	56	43	42	28	13	23	dclk2
ENSXETG00000017115	2433	0	3	1	44	14	50	137	159	79	110	81	108	nbea
ENSXETG00000017116	1536	0	0	0	0	0	0	4	4	0	0	0	0	
ENSXETG00000017117	456	18	12	24	7	5	7	27	38	12	32	21	17	vstm5
ENSXETG00000017118	2295	174	244	222	515	384	838	869	938	549	848	594	663	med17
ENSXETG00000017121	2789	2	2	3	157	97	10	116	95	52	23	4	8	deup1
ENSXETG00000017123	1563	43	82	48	39	33	72	148	164	114	67	50	69	slc36a4
ENSXETG00000017124	1823	1	0	0	21	18	19	99	89	64	26	28	21	mab21l1
ENSXETG00000017133	10002	4	5	5	65	64	103	299	377	212	130	106	102	fat3
ENSXETG00000017134	960	0	0	0	0	0	0	0	0	0	0	0	0	
ENSXETG00000017135	13773	1747	2528	2228	5405	4128	6650	5158	6078	3505	9174	6602	7484	fat1
ENSXETG00000017136	1938	7567	11380	9677	20390	15598	18808	19932	23014	13603	22910	16186	18014	ubc
ENSXETG00000017139	1974	29	43	39	139	120	126	291	310	183	225	189	193	sox13
ENSXETG00000017143	2055	103	187	134	60	59	151	686	850	532	278	209	216	arhgap9
ENSXETG00000017144	3531	5	8	6	105	75	213	499	543	311	306	208	237	gli1
ENSXETG00000017147	2316	0	0	0	0	0	0	11	9	8	1	0	0	krt5.4
ENSXETG00000017148	1875	7	13	12	42	30	117	323	361	252	58	54	30	col13a1
ENSXETG00000017149	1104	1437	2078	1796	6	3	6	33	41	15	2	0	0	inhbc.1
ENSXETG00000017150	2092	752	974	902	1641	1205	1720	3530	4329	2390	1654	1133	1361	h2afy2
ENSXETG00000017151	10497	8	3	3	71	26	65	228	292	181	142	93	104	zfhx4
ENSXETG00000017152	1761	175	249	240	295	222	235	136	184	97	19	17	15	aifm2
ENSXETG00000017153	3752	227	323	263	293	226	838	829	896	584	1122	737	802	r3hdm2
ENSXETG00000017156	3083	0	1	1	5	8	0	1	4	2	0	0	0	stac3
ENSXETG00000017158	1808	10361	15148	12808	15058	11462	44240	34277	40736	22568	23064	15762	17976	psap
ENSXETG00000017162	2058	4724	6879	5835	5731	4235	7197	3716	4354	2514	3349	2410	2610	shmt2
ENSXETG00000017165	3507	347	496	405	444	376	789	539	695	367	564	387	439	pex2
ENSXETG00000017171	1809	67	69	66	167	126	137	186	222	116	145	107	99	znf33b
ENSXETG00000017172	759	0	0	0	4	1	0	8	6	5	25	13	19	barx1
ENSXETG00000017175	2067	3	6	2	11	15	17	68	121	68	24	14	17	ENSXETG00000017175
ENSXETG00000017176	3345	0	0	0	0	2	1	2	2	0	1	0	0	sall2
ENSXETG00000017177	3843	280	373	337	881	695	866	2013	2296	1350	607	435	485	npca1
ENSXETG00000017179	429	187	245	212	125	105	123	130	156	90	83	50	49	tmem241
ENSXETG00000017180	930	88	111	99	262	183	297	143	141	106	340	214	272	cables1
ENSXETG00000017182	1920	277	411	350	535	431	566	1061	1126	681	663	432	525	mettl3
ENSXETG00000017183	3555	405	586	464	752	586	1059	1180	1432	772	924	648	738	tox4
ENSXETG00000017184	1712	197	264	201	248	212	305	871	1131	581	438	373	362	rab2b
ENSXETG00000017189	924	0	0	0	0	0	0	0	0	0	0	1	0	
ENSXETG00000017190	945	0	0	0	0	0	1	0	0	0	0	0	0	
ENSXETG00000017194	7145	337	490	413	856	643	759	1417	1603	902	1118	826	887	CHD8
ENSXETG00000017195	912	0	0	0	0	0	1	0	0	0	0	0	1	
ENSXETG00000017204	3274	1202	1779	1541	2564	2005	3208	4248	4981	2984	3722	2705	2904	supt16h
ENSXETG00000017207	921	0	0	0	0	0	0	0	0	0	0	0	0	
ENSXETG00000017208	915	0	0	0	0	0	0	0	0	0	0	0	0	
ENSXETG00000017212	1734	157	253	217	492	389	664	1103	1197	773	1048	766	751	dvl2
ENSXETG00000017213	2713	2387	3354	2728	479	379	657	2539	3082	1768	543	399	468	slc16a1
ENSXETG00000017214	2363	22	31	16	34	31	126	544	690	379	137	102	85	ppm1j
ENSXETG00000017215	3669	6	9	4	8	5	9	3	2	1	0	0	0	rpgrip1
ENSXETG00000017221	948	0	0	0	0	0	0	0	0	0	0	0	0	
ENSXETG00000017222	1053	2	5	7	6	9	11	41	46	49	4	9	3	ltb4r2
ENSXETG00000017223	1518	327	445	382	624	468	710	1081	1277	789	640	453	509	uchl5
ENSXETG00000017224	1617	340	493	433	646	546	1197	698	741	467	852	606	678	trove2
ENSXETG00000017225	7107	87	119	115	366	262	548	1236	1347	816	646	446	450	notch2
ENSXETG00000017226	1836	115	197	197	148	113	439	218	219	177	382	287	292	ache
ENSXETG00000017227	1094	163	275	208	353	299	509	790	865	537	464	327	367	c17orf49
ENSXETG00000017228	306	846	1169	936	895	647	1799	2042	2139	1274	890	610	667	rnasek
ENSXETG00000017229	3006	278	406	359	602	446	584	707	763	471	810	613	636	pelp1
ENSXETG00000017230	3563	469	657	533	920	721	1309	1556	1774	1100	1559	1109	1220	arrb2
ENSXETG00000017231	2284	604	888	810	1008	717	1101	1445	1562	1037	1595	1033	1178	ddx27
ENSXETG00000017234	249	185	263	219	167	146	385	254	313	216	534	369	442	ENSXETG00000017234
ENSXETG00000017239	981	2288	3332	2814	3338	2539	7892	26599	30127	18496	3886	2811	3111	ENSXETG00000017239
ENSXETG00000017241	3571	969	1477	1333	5947	4541	15814	39673	45048	26829	6912	5050	5520	ENSXETG00000017241
ENSXETG00000017268	1184	12	26	26	184	146	162	576	596	395	226	164	203	XB-GENE-5961370
ENSXETG00000017270	5039	197	203	200	554	432	1242	4314	4826	2766	626	489	521	c3
ENSXETG00000017275	3387	210	290	245	692	520	1061	1384	1490	902	1040	751	827	dvl3
ENSXETG00000017276	2290	5896	8415	7319	8818	6673	11253	15002	17408	9987	9570	6846	7462	ap2m1
ENSXETG00000017277	2544	1109	1655	1438	2834	2129	3973	3393	3944	2321	3306	2372	2664	abcf3
ENSXETG00000017278	3294	2	10	6	49	31	10	42	50	36	9	18	11	ccdc150
ENSXETG00000017279	3801	43	55	48	100	80	99	130	121	62	231	181	183	vwa5b2
ENSXETG00000017280	1519	365	459	424	542	392	611	752	878	534	561	392	445	alg3
ENSXETG00000017281	957	8	22	12	59	27	8	13	16	6	22	6	14	ttc16
ENSXETG00000017282	717	278	401	372	383	258	421	277	349	217	301	241	226	ENSXETG00000017282
ENSXETG00000017283	240	0	1	0	2	1	5	7	9	8	6	2	4	camk2n2
ENSXETG00000017285	2301	77	100	83	218	143	238	195	203	113	325	225	261	ece2
ENSXETG00000017286	2819	1953	2986	2677	4343	3339	3833	7313	8489	5029	2378	1615	1890	psmd2
ENSXETG00000017287	4932	2346	3554	3021	3383	2550	2052	3950	4569	2620	3101	2164	2477	eif4g1
ENSXETG00000017288	2251	13	27	25	36	25	85	54	59	45	200	136	168	fam131a
ENSXETG00000017289	2661	929	1364	1236	3969	3105	9944	185	174	120	5517	3775	4413	clcn2
ENSXETG00000017290	3923	4	15	13	2	2	3	8	11	3	1	2	1	chrd.1
ENSXETG00000017291	560	577	835	693	245	214	296	566	663	445	227	164	191	polr2h
ENSXETG00000017292	849	151	220	180	219	137	752	1134	1287	755	107	66	73	teddm1
ENSXETG00000017293	2925	78	94	84	265	204	358	1717	1981	1256	606	401	461	ephb3
ENSXETG00000017294	3413	33	52	48	198	146	267	1453	1740	1060	335	239	245	prkcb
ENSXETG00000017298	2400	374	550	458	1172	892	2059	2491	2659	1690	1915	1305	1498	arhgap17
ENSXETG00000017302	1176	2474	3613	2995	2973	2262	2477	1373	1582	970	1292	907	1011	eci1
ENSXETG00000017304	1796	73	125	101	161	113	206	274	299	181	337	257	228	c7orf26
ENSXETG00000017308	2964	137	235	148	238	184	321	693	725	414	345	210	258	dlgap4
ENSXETG00000017309	2734	48	83	67	240	184	359	3260	3739	2111	469	311	351	vasn
ENSXETG00000017310	378	202	310	298	123	83	157	159	185	120	229	189	166	coro7
ENSXETG00000017312	924	0	10	1	8	3	4	67	83	42	14	9	12	tgif2
ENSXETG00000017313	3247	0	0	0	0	0	0	0	0	0	0	0	0	srl
ENSXETG00000017314	2414	298	423	418	527	404	628	758	872	550	1147	820	904	lpin3
ENSXETG00000017315	4867	24	53	36	303	285	592	2094	2408	1474	827	602	651	adcy9
ENSXETG00000017316	2139	4	7	2	13	10	29	34	41	25	28	19	18	emilin3
ENSXETG00000017317	2396	9950	14320	12333	12686	9927	9838	11024	12665	7453	7491	5131	5677	rpn2
ENSXETG00000017318	2435	328	539	447	698	529	736	2804	3133	2009	729	516	580	src
ENSXETG00000017319	1900	733	914	898	1335	941	1322	1924	2061	1433	1526	955	1074	ctnnbl1
ENSXETG00000017320	651	1	3	0	9	4	12	43	44	28	27	30	26	vstm2l
ENSXETG00000017321	4247	185	297	221	274	211	404	431	538	327	435	321	352	tti1
ENSXETG00000017322	1491	581	860	699	560	395	862	1069	1290	781	772	572	630	rprd1a
ENSXETG00000017323	1533	523	722	666	1207	898	1337	5567	6406	3941	782	541	568	tgm2
ENSXETG00000017325	807	0	0	0	0	0	0	0	0	0	0	0	0	
ENSXETG00000017326	2526	471	619	519	492	383	1505	3028	3530	2067	869	608	682	bpi
ENSXETG00000017330	957	0	0	0	0	0	0	0	0	0	0	0	0	
ENSXETG00000017331	1597	1369	1953	1606	931	676	1577	639	748	491	1886	1443	1553	bpi
ENSXETG00000017336	1704	18	20	17	34	35	57	57	65	36	52	43	51	ENSXETG00000017336
ENSXETG00000017337	222	0	0	0	0	0	0	0	0	0	0	0	0	
ENSXETG00000017344	801	77	129	111	126	101	283	175	205	122	297	238	244	wdr92
ENSXETG00000017345	1377	34	58	40	76	71	142	2353	2737	1676	228	162	221	msr1
ENSXETG00000017349	1074	3180	4568	3918	4861	3708	7286	4946	5694	3314	23201	16353	18309	pno1
ENSXETG00000017351	1656	1	0	0	1	0	0	0	0	0	0	1	0	ENSXETG00000017351

ENSXETG00000017353	1203	2712	3822	3187	2031	1543	2530	1972	2367	1358	1494	1030	1153	tmco1
ENSXETG00000017355	2478	3011	4446	3824	3235	2396	17492	5006	6159	3595	5868	4226	4679	ppp3r1
ENSXETG00000017356	1737	11	8	18	13	7	5	10	9	11	18	8	11	hbox10
ENSXETG00000017357	2017	79	163	171	244	161	334	502	562	325	599	427	438	uck2
ENSXETG00000017358	2592	16	38	41	28	25	71	263	311	186	40	47	43	plek
ENSXETG00000017359	792	0	1	0	0	1	1	3	8	2	0	2	0	fam78b
ENSXETG00000017360	3485	817	1132	991	953	724	1440	1022	1235	703	956	684	764	plpp6
ENSXETG00000017361	1137	0	0	0	2	1	0	3	5	1	1	0	1	prokr2
ENSXETG00000017362	2070	0	1	0	1	0	37	114	106	72	78	50	34	fmo5
ENSXETG00000017371	4154	349	418	407	465	343	496	1208	1433	776	430	296	339	pla2g4a
ENSXETG00000017372	1633	0	0	0	0	0	1	1	1	0	0	0	1	
ENSXETG00000017373	1134	14	8	11	20	18	31	37	56	33	59	33	31	ENSXETG00000017373
ENSXETG00000017376	1257	0	0	0	0	0	0	0	0	3	4	4	1	zmynd15
ENSXETG00000017377	2244	3	6	9	12	12	25	519	553	339	72	45	44	pcolce
ENSXETG00000017378	3738	123	184	143	394	296	455	1285	1571	949	335	252	255	ptgs2
ENSXETG00000017379	2349	1359	1933	1585	1261	977	1974	2741	3387	1874	1356	986	1202	ccnl2
ENSXETG00000017380	1728	259	411	346	340	282	325	489	579	409	416	283	304	klhdc4
ENSXETG00000017382	522	0	0	0	0	0	0	0	0	0	0	0	0	
ENSXETG00000017385	3643	524	701	689	1859	1394	3810	2648	2967	1929	3310	2469	2621	kif1c
ENSXETG00000017387	690	0	0	0	0	0	0	0	0	0	0	0	0	
ENSXETG00000017388	1116	368	538	432	264	198	482	377	539	246	164	106	146	c1orf27
ENSXETG00000017389	4008	65	110	110	314	261	515	579	699	403	692	487	530	camta2
ENSXETG00000017390	7035	917	1311	1155	2963	2271	2123	2943	3307	1969	2273	1510	1792	tpr
ENSXETG00000017395	678	515	797	692	1051	822	1173	1937	2087	1449	893	602	648	spag7
ENSXETG00000017401	4356	9	16	9	7	9	8	13	21	7	24	14	8	ENSXETG00000017401
ENSXETG00000017402	2271	0	0	1	9	9	7	48	54	28	51	42	32	jph3
ENSXETG00000017403	2271	6	14	8	19	18	39	64	96	52	49	33	32	zcchc14
ENSXETG00000017404	4149	40	54	41	100	55	79	157	184	101	107	80	86	prg4
ENSXETG00000017405	3610	624	808	595	1166	842	2374	2701	3341	1947	2157	1556	1794	map1lc3b
ENSXETG00000017407	3343	715	1051	960	1193	971	1562	887	1083	633	1148	807	935	fbxo31
ENSXETG00000017408	1556	5	7	9	21	24	65	292	361	189	425	360	350	rrad
ENSXETG00000017409	2045	117	127	143	147	123	238	311	318	228	261	204	213	kiaa0513
ENSXETG00000017410	3685	32	47	57	8	4	45	5	3	3	3	1	2	cdh16
ENSXETG00000017411	17303	76	103	70	327	241	663	1167	1286	732	312	233	251	hmcn1
ENSXETG00000017412	3993	0	0	0	2	1	0	5	4	3	1	7	6	disp3.2
ENSXETG00000017414	1134	329	381	346	235	166	380	293	392	223	271	190	225	ubiad1
ENSXETG00000017416	870	1	0	1	108	92	4	44	46	35	4	6	3	fam92b
ENSXETG00000017418	2877	6	11	8	118	101	127	80	72	41	47	44	40	ENSXETG00000017418
ENSXETG00000017419	1599	76	85	72	174	140	314	68	78	59	191	135	153	pdp2
ENSXETG00000017420	1776	2143	2942	2544	1256	1006	2468	1279	1549	914	903	614	668	ca7
ENSXETG00000017422	876	283	427	395	467	420	738	571	686	422	1304	889	1008	pparg
ENSXETG00000017425	1746	4	2	2	1	0	1	2	2	0	1	0	0	angptl7
ENSXETG00000017426	4986	205	258	248	585	479	973	923	1086	622	898	616	759	XB-GENE-994583
ENSXETG00000017427	1212	1576	2284	2075	1589	1189	754	470	498	334	180	145	140	mvd
ENSXETG00000017431	938	400	604	472	341	271	652	2323	2596	1579	926	631	737	cyba
ENSXETG00000017432	1531	0	0	0	0	0	0	1	1	0	0	0	0	
ENSXETG00000017433	603	33	47	37	13	11	22	8	12	4	13	9	9	il17c
ENSXETG00000017437	357	603	827	736	447	384	787	763	810	518	712	513	530	cdk2ap2
ENSXETG00000017438	1396	57	110	77	227	176	326	732	774	465	337	238	245	aip
ENSXETG00000017441	1122	0	0	0	71	58	7	6	7	4	42	28	38	apobec4
ENSXETG00000017442	927	0	0	0	0	0	0	0	0	0	0	0	0	
ENSXETG00000017443	1863	0	0	0	1	1	2	11	15	10	1	1	3	colgalt2
ENSXETG00000017444	711	0	0	0	0	0	0	0	0	0	0	0	0	
ENSXETG00000017446	2508	102	172	126	97	73	102	143	176	98	40	26	43	tsen15
ENSXETG00000017447	3388	525	751	641	915	660	1190	1116	1338	774	1751	1216	1339	c1orf21
ENSXETG00000017448	3564	528	718	569	647	494	1556	1457	1731	933	863	628	672	ccdc134
ENSXETG00000017449	4184	2376	3649	2924	7867	6110	8934	2217	2538	1478	3136	2257	2417	srebf2
ENSXETG00000017450	999	0	0	0	0	0	0	0	0	0	0	0	0	
ENSXETG00000017451	2349	2	8	1	3	4	5	17	14	12	5	4	8	cat2
ENSXETG00000017454	2532	0	0	0	0	0	0	0	0	0	0	0	0	
ENSXETG00000017455	2298	24	34	28	54	42	97	180	190	108	124	70	96	ENSXETG00000017455
ENSXETG00000017458	1368	0	0	0	1	4	7	1	1	3	0	2	0	ENSXETG00000017458
ENSXETG00000017459	1780	314	545	474	364	317	529	375	382	214	402	309	365	nudt12
ENSXETG00000017460	2451	78	109	94	292	244	548	999	1149	688	623	459	534	ENSXETG00000017460
ENSXETG00000017462	2550	83	131	123	486	363	634	1049	1086	652	627	465	490	mtss1.1
ENSXETG00000017463	624	132	190	136	113	89	176	235	277	174	236	154	184	c1orf109
ENSXETG00000017464	792	40	56	55	78	54	109	152	159	109	86	42	73	snip1
ENSXETG00000017465	807	87	116	121	62	49	94	103	110	76	73	56	38	ENSXETG00000017465
ENSXETG00000017466	2619	356	518	398	1214	963	2670	1131	1540	825	3225	2301	2654	meaf6
ENSXETG00000017467	2060	1	1	0	1	1	0	0	1	1	0	0	0	krt15
ENSXETG00000017468	906	52	87	62	138	100	139	233	242	158	179	126	124	ENSXETG00000017468
ENSXETG00000017472	1518	4	4	7	19	17	64	94	110	62	87	66	78	ENSXETG00000017472
ENSXETG00000017473	3813	11	14	16	120	107	128	431	505	312	329	256	274	ENSXETG00000017473
ENSXETG00000017477	897	132	188	177	230	148	133	319	366	200	82	76	77	tatdn1
ENSXETG00000017483	890	306	416	378	455	337	923	661	845	485	683	483	509	bloc1s1
ENSXETG00000017484	756	308	429	381	365	245	642	111	159	86	319	222	265	rdh5
ENSXETG00000017487	2300	2021	2851	2337	1881	1393	2662	1587	1833	1118	2097	1419	1563	rnf139
ENSXETG00000017488	1885	7613	10773	8972	8987	6837	20200	18032	21428	12188	15144	10571	11586	cd63
ENSXETG00000017489	1297	1192	1718	1499	4181	3168	4269	1575	1795	1191	4836	3497	3695	prkca
ENSXETG00000017490	4253	846	1254	1042	1674	1301	4982	3870	4468	2707	2629	1863	2114	washc5
ENSXETG00000017493	936	0	0	0	1	3	11	13	21	8	4	7	5	tmtops
ENSXETG00000017494	831	8	16	21	5	4	10	20	22	18	5	5	2	cd40lg
ENSXETG00000017497	2310	34	63	38	83	83	212	372	464	242	241	121	174	arhgef6
ENSXETG00000017499	1077	239	366	289	472	361	1105	1144	1183	757	895	666	737	trib1
ENSXETG00000017500	1606	669	837	681	780	644	1793	751	954	506	1842	1267	1430	pcmt1
ENSXETG00000017501	1500	0	0	0	0	0	0	1	0	0	0	0	0	
ENSXETG00000017502	3217	59	73	64	138	104	158	263	323	173	187	133	157	fut10
ENSXETG00000017503	1255	23	35	26	46	33	47	126	156	85	78	50	73	lrp11
ENSXETG00000017504	2109	10	16	26	61	62	140	467	507	297	157	145	103	nrg1
ENSXETG00000017509	2460	6	8	5	14	18	20	98	100	55	33	22	22	efr3b
ENSXETG00000017513	3735	4	4	3	5	4	6	17	28	29	9	6	6	nphs1
ENSXETG00000017515	3081	143	216	176	311	220	368	740	845	440	432	320	361	usp38
ENSXETG00000017525	541	1382	2049	1734	1407	1148	1169	1609	1846	1101	777	597	617	pfdn4
ENSXETG00000017526	3024	7	14	10	19	18	43	262	312	186	82	66	46	ENSXETG00000017526
ENSXETG00000017527	1524	95	170	138	1986	1491	50	51	60	36	547	383	414	cyp24a1
ENSXETG00000017530	1090	2	1	3	20	9	58	265	316	191	156	103	114	stmn4
ENSXETG00000017532	1196	0	0	0	0	0	0	0	0	0	0	1	1	pomc
ENSXETG00000017534	2438	18928	27872	23781	20318	15232	24723	6261	7132	4229	5532	3789	4444	hsd17b4
ENSXETG00000017537	1701	149	210	167	385	314	424	422	514	319	328	221	223	c14orf119
ENSXETG00000017538	2888	389	645	510	1049	759	1216	1212	1293	780	1375	992	1098	ptk2b
ENSXETG00000017543	2676	38	73	57	115	70	114	530	574	358	202	168	171	arhgap33
ENSXETG00000017549	2010	101	154	142	257	158	452	610	714	386	384	264	335	gab1
ENSXETG00000017550	3816	100	137	105	184	145	317	733	891	481	262	208	208	znf217
ENSXETG00000017552	4347	400	546	456	1335	1065	1584	1465	1785	1047	1248	876	966	zcchc6
ENSXETG00000017553	1488	0	0	1	1	0	1	44	51	37	6	5	12	chrna2
ENSXETG00000017555	3072	5	7	3	56	43	117	418	553	274	186	166	181	tshz2
ENSXETG00000017559	3675	0	0	0	0	0	0	1	2	0	0	6	5	vwa5b1
ENSXETG00000017560	567	128	202	163	290	212	278	422	459	303	301	223	214	lin37
ENSXETG00000017562	570	66	109	88	108	95	187	545	614	355	164	146	132	tnfaip8
ENSXETG00000017563	330	142	241	209	228	199	888	2808	3011	1933	1435	944	1055	hspb6
ENSXETG00000017565	831	738	984	905	890	693	1135	1320	1555	924	765	549	594	psenen

ENSXETG00000017566	2994	986	1451	1238	1101	825	529	2648	3196	1857	467	331	347	smarca5
ENSXETG00000017567	6827	370	535	448	1428	1096	1492	1743	1926	1147	2124	1547	1668	kmt2b
ENSXETG00000017568	9078	281	407	345	638	487	1273	1500	1812	1084	1672	1103	1310	dmxl1
ENSXETG00000017571	6583	839	1102	1033	1263	984	1569	1363	1760	941	1527	1101	1266	dapk1
ENSXETG00000017573	1173	0	0	0	1	0	0	0	1	0	1	2	1	
ENSXETG00000017576	1371	633	906	790	956	735	1012	729	846	575	792	548	575	otud3
ENSXETG00000017581	1888	4145	6164	5303	5803	4279	5650	1532	1684	1021	4489	3330	3652	ephx2
ENSXETG00000017583	1482	213	349	257	227	175	375	408	440	268	168	110	137	dtwd2
ENSXETG00000017584	2952	0	0	0	4	2	1	11	9	13	5	5	5	epha6
ENSXETG00000017586	2270	4	3	8	5	1	11	3	2	1	24	18	15	fbp2
ENSXETG00000017588	1011	16153	24369	20449	20993	16263	25655	1861	2111	1192	8600	6137	6736	fbp1
ENSXETG00000017590	8349	0	0	0	1	2	0	0	0	0	0	0	1	frem3
ENSXETG00000017591	3613	14	22	25	57	57	182	54	62	38	196	144	161	c9orf3
ENSXETG00000017597	1581	67	87	77	47	33	57	95	115	66	51	31	33	fancc
ENSXETG00000017598	1453	205	260	219	185	149	331	310	369	188	236	152	178	pop4
ENSXETG00000017599	1693	398	604	517	972	759	1427	1435	1570	1084	1274	928	941	rbm42
ENSXETG00000017604	1608	124	189	160	651	471	981	5417	5989	3621	2391	1685	1874	nbl1
ENSXETG00000017606	4944	265	434	344	501	388	587	1010	1237	723	947	662	708	otud4
ENSXETG00000017607	843	1267	1804	1547	2051	1599	2304	4260	4743	2949	2812	1988	2079	capzb
ENSXETG00000017613	872	128	197	167	145	67	113	245	343	182	107	109	90	anapc10
ENSXETG00000017614	2311	0	0	1	4	1	0	8	5	1	1	1	2	XB-GENE-5903348
ENSXETG00000017615	777	2817	4191	3677	4567	3556	4864	1453	1520	1043	3873	2614	2832	akr7a2
ENSXETG00000017616	2097	9	16	21	79	67	289	960	1123	654	423	312	304	hhip
ENSXETG00000017617	1210	182	301	239	199	128	196	232	271	142	255	184	206	rpp40
ENSXETG00000017618	1312	205	291	258	169	120	339	426	526	324	197	159	151	med31
ENSXETG00000017630	2338	0	0	0	7	1	9	25	36	9	16	16	23	rims3
ENSXETG00000017634	1715	796	1163	1007	1430	1139	2265	1561	1703	1000	1527	1021	1239	tinagl1
ENSXETG00000017638	1842	57	70	66	104	70	101	169	186	104	201	134	154	ENSXETG00000017638
ENSXETG00000017639	1350	208	282	247	532	385	644	2333	2647	1725	764	558	573	slc2a1
ENSXETG00000017641	860	25	39	24	35	18	27	2269	2337	1605	127	84	92	rcn3
ENSXETG00000017642	1098	649	976	828	947	757	1028	1411	1547	929	1058	707	832	nosip
ENSXETG00000017643	3141	418	629	533	1022	852	1859	1577	1854	1036	1261	909	986	chuk
ENSXETG00000017645	2825	382	544	498	720	537	644	1360	1411	832	808	548	569	sart3
ENSXETG00000017647	2324	320	491	409	591	455	595	1198	1351	807	922	641	757	prrg4
ENSXETG00000017649	3069	1	2	2	7	6	8	38	42	25	17	17	15	tmem132e
ENSXETG00000017650	2078	609	834	715	860	657	451	1477	1704	983	565	380	386	pef1
ENSXETG00000017651	2475	0	0	0	1	2	2	7	4	4	1	1	5	hcn3
ENSXETG00000017652	2817	261	358	314	222	158	353	375	456	226	248	189	183	ficd
ENSXETG00000017653	780	0	1	0	1	6	10	12	18	9	4	2	3	sds
ENSXETG00000017655	3672	408	508	481	598	507	2078	1380	1683	967	920	627	748	slc8b1
ENSXETG00000017656	1954	733	1122	970	1338	1003	1838	2426	2671	1742	2966	2189	2304	clk2
ENSXETG00000017657	1647	338	476	404	733	570	2384	1636	1761	968	1137	836	900	plbd2
ENSXETG00000017659	1305	2103	3236	2782	2591	2120	4030	666	748	420	1014	779	822	sdsl
ENSXETG00000017662	876	1	2	0	2	4	16	30	30	16	25	14	14	kctd7
ENSXETG00000017663	525	343	507	464	755	610	1654	1447	1496	936	991	711	854	scamp3
ENSXETG00000017664	2618	688	983	777	693	509	1026	1490	1810	952	954	679	799	tpst1
ENSXETG00000017665	3112	0	0	0	1	3	0	0	0	0	0	2	4	
ENSXETG00000017667	840	2355	3452	2794	3340	2531	3976	3388	3697	2254	4374	3037	3398	ufc1
ENSXETG00000017668	2920	315	429	399	579	396	451	731	839	486	634	416	489	rbm19
ENSXETG00000017669	1662	20	54	34	121	86	97	219	242	142	124	112	98	usp21
ENSXETG00000017673	1522	359	513	451	899	663	1131	655	779	493	1349	978	987	ppox
ENSXETG00000017674	974	35	43	38	66	56	82	162	150	91	137	94	88	dnz1
ENSXETG00000017676	2583	15	15	9	38	26	229	3485	3794	2496	130	105	86	adamts4
ENSXETG00000017680	3450	0	0	3	6	6	5	18	31	16	11	17	10	adgrb2
ENSXETG00000017688	1649	0	0	0	0	0	0	0	0	0	0	0	0	
ENSXETG00000017689	2322	11	18	10	15	16	17	30	30	18	37	17	24	ENSXETG00000017689
ENSXETG00000017691	1401	16	23	20	39	19	48	57	62	33	40	23	39	epm2a
ENSXETG00000017697	14415	758	1057	857	2062	1579	2171	2887	3356	1966	2761	1984	2198	birc6
ENSXETG00000017701	2532	341	474	448	522	461	366	962	1175	660	337	313	295	ttc27
ENSXETG00000017706	2388	9	7	7	32	27	46	143	178	106	125	83	93	parp8
ENSXETG00000017708	2197	0	0	0	0	0	0	0	0	0	0	0	0	
ENSXETG00000017710	2645	164	228	181	292	236	603	1019	1262	738	508	324	406	bnip2
ENSXETG00000017712	762	179	247	215	617	464	293	34	39	26	12	8	8	emb
ENSXETG00000017714	2529	0	1	3	2	1	1	8	6	7	6	1	1	hcn1
ENSXETG00000017715	1299	13	15	23	27	16	52	170	201	107	93	77	79	gcnt3
ENSXETG00000017716	1276	993	1476	1289	1110	844	1357	847	970	636	974	649	675	mrps30
ENSXETG00000017718	3808	67	81	72	202	138	246	362	410	249	93	50	73	mtfr2
ENSXETG00000017719	2919	187	199	202	381	256	711	692	801	451	519	402	434	pde7b
ENSXETG00000017722	3101	6	5	14	58	53	166	204	275	168	170	119	130	otud7a
ENSXETG00000017724	4326	9	9	6	120	113	196	176	180	137	177	139	127	plxna2
ENSXETG00000017725	1525	0	0	0	0	0	0	0	0	0	1	0	0	
ENSXETG00000017726	2545	0	0	0	0	0	1	2	0	0	0	0	1	
ENSXETG00000017729	1671	95	131	128	27	20	52	12	16	16	11	7	10	ralyl
ENSXETG00000017730	942	0	3	1	60	33	16	24	29	23	39	17	30	wdr93
ENSXETG00000017731	2331	289	447	347	265	213	749	354	456	254	161	123	130	pex11a
ENSXETG00000017733	2408	0	0	1	1	1	0	3	8	4	1	1	1	plin1
ENSXETG00000017736	675	2	1	4	35	27	42	100	119	56	95	95	93	rergl
ENSXETG00000017737	3240	64	106	88	224	168	393	2077	2343	1388	475	373	365	asap1
ENSXETG00000017738	5658	206	272	218	296	254	420	588	713	427	540	394	437	kif7
ENSXETG00000017742	4450	172	234	204	607	488	202	634	811	461	240	153	153	fanci
ENSXETG00000017744	1561	4	5	7	6	3	9	16	12	9	22	11	16	42Sp50
ENSXETG00000017748	424	28246	42221	37605	39697	28806	46491	39129	45407	27853	113825	79893	87573	XB-GENE-5843131
ENSXETG00000017750	3594	242	294	276	337	280	372	444	519	327	618	366	427	polg
ENSXETG00000017753	1473	0	0	0	2	1	2	5	4	2	4	2	0	kcna3
ENSXETG00000017755	1077	348	468	462	274	172	325	739	852	541	272	177	205	fam49b
ENSXETG00000017758	1500	0	0	0	0	0	0	4	0	0	1	0	1	
ENSXETG00000017761	1389	0	0	0	0	0	0	0	0	0	0	0	0	
ENSXETG00000017762	1965	1	0	0	0	0	0	3	8	2	0	0	1	rhcg
ENSXETG00000017769	5892	73	96	91	167	146	138	231	255	151	80	54	72	ENSXETG00000017769
ENSXETG00000017770	1591	2	3	1	10	7	4	1	2	2	26	14	14	rlbp1
ENSXETG00000017771	7142	1119	1502	1230	2471	1892	3125	2042	2563	1539	2430	1677	1950	abhd2
ENSXETG00000017772	1606	753	1136	1016	1116	795	2398	4695	5333	3001	507	394	447	mfge8
ENSXETG00000017773	1065	0	0	0	0	0	0	0	0	0	0	0	0	
ENSXETG00000017774	2037	418	589	493	734	567	1548	891	1099	687	1363	968	1075	tmco3
ENSXETG00000017775	2419	7094	9946	8294	7970	6093	6192	7084	8538	5022	5298	3672	4266	stt3a
ENSXETG00000017776	1663	215	327	256	298	242	436	711	916	501	316	216	262	tfdp1
ENSXETG00000017777	4932	12	13	12	225	182	45	42	25	18	58	32	28	cfap44
ENSXETG00000017778	1840	0	0	2	0	0	0	0	0	0	0	0	0	atp4b
ENSXETG00000017780	2172	2	2	2	28	27	35	171	204	105	87	73	66	fez1
ENSXETG00000017781	3005	4	3	3	22	28	65	326	389	221	111	76	81	pknox2
ENSXETG00000017782	1683	0	0	2	0	1	3	0	2	0	1	0	1	grk1
ENSXETG00000017786	4005	165	225	208	422	324	594	709	890	528	555	402	414	mtm1
ENSXETG00000017791	1953	0	0	0	0	0	0	0	0	0	0	0	0	
ENSXETG00000017793	1193	222	279	261	529	437	700	1176	1282	754	715	498	513	zc4h2
ENSXETG00000017794	2538	4	10	10	29	19	33	164	178	98	103	75	59	zc3h12b
ENSXETG00000017800	459	0	0	0	2	0	1	0	1	2	4	3	2	fam155a
ENSXETG00000017801	4586	152	200	161	722	583	1332	1517	1753	1055	712	485	501	efnb2
ENSXETG00000017807	1934	44	69	54	331	237	320	441	519	318	793	544	614	spry2
ENSXETG00000017809	2405	1809	2497	2008	1686	1344	2374	1242	1460	838	1941	1369	1529	ndfip2
ENSXETG00000017810	4594	381	567	510	816	628	905	1317	1574	894	1073	763	863	rbm26
ENSXETG00000017812	1389	0	0	0	0	0	0	0	0	0	0	0	0	

ENSXETG00000017813	2311	39	58	37	101	73	124	94	142	61	114	96	117	rnf219
ENSXETG00000017814	1080	0	0	0	0	0	0	0	0	0	0	0	0	
ENSXETG00000017815	1218	0	0	0	0	0	0	0	0	0	0	0	0	
ENSXETG00000017816	1169	675	1062	882	713	545	849	550	692	362	570	417	494	apool
ENSXETG00000017817	2662	116	215	153	167	148	285	359	368	239	184	122	160	znf711
ENSXETG00000017818	1827	739	1170	943	1209	948	1364	361	394	242	1413	1019	1108	pof1b
ENSXETG00000017820	1929	477	699	606	883	662	2675	1860	2212	1216	1619	1191	1304	chm
ENSXETG00000017821	7423	2	6	2	6	5	6	14	19	14	11	13	14	myh2
ENSXETG00000017822	819	2	2	3	22	20	43	89	105	64	88	56	73	tlx2
ENSXETG00000017823	6273	7	5	3	35	23	46	241	283	132	62	48	41	plxnb1
ENSXETG00000017824	1659	13	11	8	34	25	22	368	385	249	57	42	39	ENSXETG00000017824
ENSXETG00000017825	3418	2315	3325	2849	2578	2023	3216	3932	4838	2769	2398	1722	1962	ddx1
ENSXETG00000017826	5676	96	164	139	321	259	355	358	377	220	299	259	234	nbas
ENSXETG00000017827	1269	0	1	0	1	1	0	29	22	20	3	0	5	kcnj9
ENSXETG00000017828	156	8105	11876	10394	6826	5288	6160	4958	5726	3495	5465	3745	4143	cox6b1
ENSXETG00000017830	1599	3348	4855	4179	4481	3506	3131	7284	7962	5196	5499	3867	4295	fbl
ENSXETG00000017833	246	2	2	0	0	0	0	1	2	0	0	0	1	ENSXETG00000017833
ENSXETG00000017834	1644	152	233	211	1561	1167	1102	1196	1318	879	1058	775	807	ENSXETG00000017834
ENSXETG00000017838	2069	1168	1728	1542	1812	1484	2694	3020	3366	1902	2293	1603	1788	vps4a
ENSXETG00000017841	4746	510	826	651	698	523	1065	2000	2229	1311	1305	958	1026	setd5
ENSXETG00000017842	3336	265	398	391	644	443	732	1030	1121	705	602	441	572	neurl4
ENSXETG00000017844	4955	333	448	452	748	539	1112	2072	2484	1356	1115	723	845	stk3
ENSXETG00000017845	1410	439	572	473	1585	1197	5878	759	975	535	4528	3112	3708	hnf4g
ENSXETG00000017847	1749	43	63	54	109	63	186	178	196	120	180	125	139	acap1
ENSXETG00000017850	1699	343	546	395	2786	2078	2618	548	559	389	1912	1332	1468	osr2
ENSXETG00000017851	1689	833	1262	1132	1412	1119	1939	875	972	571	1048	674	797	cog8
ENSXETG00000017853	1224	1117	1559	1280	582	459	860	935	1197	633	769	558	560	nip7
ENSXETG00000017855	12153	242	324	286	840	612	1383	1110	1222	702	1547	1233	1287	vps13b
ENSXETG00000017856	1106	11	16	13	9	15	31	5	18	8	11	12	11	tmed6
ENSXETG00000017857	1171	1	0	0	3	3	4	10	10	14	6	9	3	lhfpl4
ENSXETG00000017858	2437	1966	2803	2427	2147	1716	2120	1595	1889	990	983	664	782	cyb5b
ENSXETG00000017860	2928	852	1249	1032	1154	918	2507	1982	2180	1329	911	658	702	mtmr14
ENSXETG00000017861	4302	129	186	166	368	254	718	577	675	430	546	436	454	nfat5
ENSXETG00000017863	3681	3	3	1	128	88	16	19	30	17	21	16	15	rgs22
ENSXETG00000017864	1651	96	114	95	106	80	156	102	125	62	89	72	69	snx31
ENSXETG00000017865	1326	0	0	0	0	0	0	1	1	0	0	0	0	cpne5
ENSXETG00000017866	1286	224	341	265	306	241	353	490	591	324	270	193	232	aven
ENSXETG00000017868	1578	15	23	24	30	32	95	28	46	28	53	53	39	chrm5
ENSXETG00000017869	828	204	304	230	186	132	120	307	324	196	137	96	114	nqo1
ENSXETG00000017873	3912	1206	1789	1454	2649	1925	3269	4307	4944	3009	3791	2598	2792	rnf19a
ENSXETG00000017874	2156	733	1095	974	679	538	453	947	1042	644	769	538	616	nob1
ENSXETG00000017875	693	101	148	139	433	341	647	405	455	308	497	371	357	pdxk
ENSXETG00000017876	3716	1607	2345	1975	1811	1344	1786	1691	2006	1150	819	520	587	spag1
ENSXETG00000017879	3155	576	820	688	1259	1018	2771	1668	1977	1169	1945	1363	1526	wwp2
ENSXETG00000017880	2967	3	6	6	16	13	26	81	113	71	84	60	77	gpr176
ENSXETG00000017882	2085	8	7	11	75	32	16	45	56	31	9	4	4	fbxo43
ENSXETG00000017883	3619	1678	2323	1882	3142	2295	5319	4928	5890	3383	3490	2366	2737	susd6
ENSXETG00000017884	1110	1515	2173	1835	1635	1184	1738	4066	4409	2675	836	576	616	psmd7
ENSXETG00000017892	69	0	0	0	0	0	0	0	0	0	0	0	0	
ENSXETG00000017893	1371	19	25	21	18	5	10	4	5	4	10	13	11	ENSXETG00000017893
ENSXETG00000017895	1532	121	184	131	87	53	361	837	1021	611	439	277	332	exd1
ENSXETG00000017898	3079	2	0	0	20	9	18	104	122	66	24	15	21	b3gat2
ENSXETG00000017899	3588	1	0	0	1	0	3	10	8	7	6	2	7	fhod3
ENSXETG00000017900	3410	659	917	856	785	626	949	1488	1624	928	644	484	521	smap1
ENSXETG00000017901	1321	2012	2815	2399	1953	1490	1887	5400	6612	3521	2500	1758	1940	chp1
ENSXETG00000017902	1028	392	569	441	272	197	266	248	332	176	244	157	160	ndufaf1
ENSXETG00000017903	2381	726	1123	993	1813	1327	2265	3012	3516	2108	3442	2412	2659	rtf1
ENSXETG00000017906	2631	2644	3827	3266	5435	4123	12010	8743	10527	6060	5267	3545	4268	pdcd6ip
ENSXETG00000017907	4714	394	535	477	665	536	881	1042	1233	699	739	499	588	fam135a
ENSXETG00000017909	1388	653	977	869	898	651	1212	1169	1436	845	937	678	705	slc39a9
ENSXETG00000017911	12474	619	873	781	4462	3360	2814	5503	5805	3859	3646	2442	2719	hspg2
ENSXETG00000017913	1151	332	467	418	201	201	732	288	341	190	156	126	125	st3gal4
ENSXETG00000017914	315	2173	3112	2720	2490	1918	2919	3392	3753	2278	2131	1467	1551	erh
ENSXETG00000017916	3044	15	16	21	52	36	51	379	415	246	130	71	98	galnt16
ENSXETG00000017917	1030	119	182	157	175	125	278	509	585	382	185	139	148	dcps
ENSXETG00000017918	3445	220	299	223	639	477	549	743	827	557	907	728	761	USP48
ENSXETG00000017919	1587	25	24	24	53	51	128	61	80	49	111	93	117	tirap
ENSXETG00000017920	2906	778	1130	980	1960	1440	2374	3150	3656	2075	1885	1433	1565	st14
ENSXETG00000017921	3377	85	92	93	250	196	179	456	530	293	238	157	188	zbtb44
ENSXETG00000017922	1100	378	459	430	228	207	359	494	564	306	281	223	208	sdhaf4
ENSXETG00000017925	2757	6	4	3	43	32	101	57	68	48	144	121	144	adamts8
ENSXETG00000017926	2763	1	2	1	11	4	10	30	22	19	7	5	8	COL9A1
ENSXETG00000017927	2431	0	0	0	0	0	0	0	0	0	3	2	5	
ENSXETG00000017928	1927	207	245	204	273	218	699	1052	1293	754	418	345	389	hpse
ENSXETG00000017929	1935	3203	4551	3946	2440	1924	1002	1947	2344	1272	656	489	549	pdia4
ENSXETG00000017930	2602	225	326	281	281	220	433	338	357	218	307	203	241	exd2
ENSXETG00000017932	2562	199	274	255	376	317	840	3008	3254	2011	916	685	739	pacs2
ENSXETG00000017933	2969	5924	8441	7467	9248	6860	13019	22813	26253	15061	12752	8976	9898	actn1
ENSXETG00000017934	1218	2785	3581	3176	2299	1702	3660	547	664	399	1109	778	904	hsd17b11
ENSXETG00000017935	2314	286	390	389	398	339	30	41	38	10	56	35	40	ugt2a1
ENSXETG00000017936	1083	16	15	17	52	28	34	63	70	35	100	91	67	rap1gap
ENSXETG00000017937	1731	671	989	773	1199	965	1333	1282	1468	828	1429	1024	1185	XB-GENE-5954653
ENSXETG00000017938	1077	10	15	6	55	41	54	409	471	255	82	76	51	ENSXETG00000017938
ENSXETG00000017940	2334	0	0	0	15	7	2	7	10	9	8	10	6	kiaa1549l
ENSXETG00000017941	2466	1	0	0	0	1	2	9	8	8	0	1	3	ENSXETG00000017941
ENSXETG00000017945	432	0	0	0	2	0	2	11	17	11	18	4	10	ENSXETG00000017945
ENSXETG00000017946	1455	35	51	77	173	120	121	145	163	104	152	96	106	ENSXETG00000017946
ENSXETG00000017947	4117	96	155	120	346	217	442	362	355	240	560	384	448	hipk3
ENSXETG00000017948	2157	216	334	250	378	305	484	866	985	559	617	444	483	mta1
ENSXETG00000017950	1593	2120	3032	2613	3560	2864	7094	5837	6674	3920	4158	2763	3126	nfkbia
ENSXETG00000017952	4395	3	1	1	94	63	8	2	6	4	4	2	4	efcab5
ENSXETG00000017954	4059	226	345	257	643	484	1620	2387	2739	1720	1189	863	900	ssh2
ENSXETG00000017955	804	6910	9946	8682	6397	4887	6989	3461	4093	2474	3939	2883	3226	tmigd1
ENSXETG00000017956	3909	350	527	472	1069	871	1054	1395	1582	982	1118	819	873	CPD
ENSXETG00000017957	3070	512	677	545	995	781	1450	1210	1275	811	869	588	703	tcp11l1
ENSXETG00000017958	354	0	0	0	0	0	0	0	0	0	0	1	1	
ENSXETG00000017960	3191	1054	1582	1219	2061	1509	2995	2998	3587	2304	3593	2497	2988	ythdc1
ENSXETG00000017961	1007	218	288	273	317	262	871	264	295	181	593	432	463	fam221a
ENSXETG00000017962	2371	128	173	141	171	129	363	113	114	79	408	296	388	tmprss11f
ENSXETG00000017966	1263	0	4	0	0	4	0	2	3	1	1	0	2	tmprss11a
ENSXETG00000017967	1698	479	591	494	702	536	825	300	334	214	728	540	564	depdc7
ENSXETG00000017968	942	129	176	140	136	79	211	187	262	133	114	81	106	coq2
ENSXETG00000017969	3069	267	399	365	741	601	1727	765	937	543	1843	1321	1457	uba6
ENSXETG00000017970	2344	1495	2183	1847	3201	2452	3626	2412	2785	1639	4535	3207	3484	ptcd3
ENSXETG00000017971	2247	2666	3801	3274	4215	3426	4524	4404	4870	3044	3464	2442	2795	immt
ENSXETG00000017972	3908	62	91	67	359	288	251	880	997	583	687	486	539	REEP1
ENSXETG00000017973	726	151	231	187	106	72	131	137	203	101	250	174	246	stap1
ENSXETG00000017975	4853	1204	1715	1447	3817	2898	3707	5035	5691	3334	5751	4118	4439	kdm3a
ENSXETG00000017977	1893	7	5	11	126	106	78	74	78	51	310	232	244	lzts3
ENSXETG00000017979	676	0	0	0	0	0	0	0	0	0	0	0	0	
ENSXETG00000017980	1659	795	1133	998	1425	1157	2162	2687	3188	1782	1614	1130	1273	chmp3

ENSXETG00000017982	3400	492	812	689	1433	1057	1681	2884	3012	1920	2321	1705	1844	ptpra
ENSXETG00000017985	1177	658	1099	877	1007	782	2069	3866	4757	2634	1391	972	1084	anxa3
ENSXETG00000017986	1086	19	30	25	90	71	180	376	381	207	265	179	197	pank2
ENSXETG00000017988	993	0	0	0	0	0	0	0	0	0	0	0	0	
ENSXETG00000017989	447	6	8	7	13	7	9	65	68	36	31	18	19	rnf24
ENSXETG00000017990	2085	263	338	320	513	393	675	1068	1269	706	729	471	561	ptpn9
ENSXETG00000017992	2053	3614	5030	4349	3142	2442	2955	8629	9633	6468	2177	1569	1730	smox
ENSXETG00000017994	12390	0	0	0	1	0	0	3	3	2	1	1	2	pkhd1l1
ENSXETG00000017998	1080	203	347	272	902	686	1285	1038	1085	718	982	674	786	agpat3
ENSXETG00000018000	3759	361	567	454	1058	840	1386	1277	1419	899	1404	1074	1084	trappc10
ENSXETG00000018002	2036	285	391	370	461	358	459	549	591	413	575	389	434	pwp2
ENSXETG00000018003	1320	2	5	3	8	2	7	36	61	26	7	5	11	ENSXETG00000018003
ENSXETG00000018006	1302	34	59	48	255	188	160	766	975	562	388	287	295	ENSXETG00000018006
ENSXETG00000018007	708	287	430	410	272	199	407	532	562	366	259	191	212	vtcn1
ENSXETG00000018009	1138	140	209	185	626	525	898	7532	8360	5332	776	594	620	fstl1
ENSXETG00000018010	3960	0	1	2	2	2	2	25	22	20	6	6	12	insrr
ENSXETG00000018012	2571	6	5	4	12	8	13	27	44	15	18	10	11	pde6b
ENSXETG00000018013	1041	591	905	745	953	734	1331	794	863	559	819	628	649	lrrc58
ENSXETG00000018015	1247	773	1073	926	763	645	1058	1090	1350	826	678	459	521	ebag9
ENSXETG00000018016	1959	3	7	2	30	43	55	84	88	64	178	125	98	sybu
ENSXETG00000018017	2253	0	0	0	0	0	4	2	5	7	1	0	0	gpr156
ENSXETG00000018018	7170	455	629	502	698	504	922	1158	1395	756	1184	840	1004	znf292
ENSXETG00000018019	1218	78	115	71	138	82	139	341	376	238	190	134	147	gsk3b
ENSXETG00000018020	1132	1	7	3	7	3	11	176	202	121	13	11	19	sh3gl2
ENSXETG00000018022	1020	0	0	0	0	0	0	0	2	0	0	0	0	
ENSXETG00000018024	1945	30	49	39	115	69	272	605	766	429	496	330	386	pxdc1
ENSXETG00000018025	1417	0	1	2	32	16	1	7	1	4	38	36	39	slc22a23
ENSXETG00000018026	1112	491	771	652	725	510	760	758	804	463	595	419	490	sec22a
ENSXETG00000018027	2018	37	57	43	184	134	120	281	302	179	106	106	89	ift27
ENSXETG00000018028	4126	9	16	11	81	65	29	87	114	67	41	27	28	cntln
ENSXETG00000018029	2556	493	701	573	773	558	1042	905	1063	665	790	618	619	nr1i2
ENSXETG00000018031	2662	168	241	211	330	279	535	691	815	484	420	272	321	XB-GENE-5835884
ENSXETG00000018032	1736	15	18	18	45	28	47	1233	1322	808	165	105	118	tubb2b
ENSXETG00000018033	2175	1477	1908	1566	2400	1927	3469	2330	3270	1829	987	713	834	slc26a1
ENSXETG00000018034	864	36	67	56	45	26	70	65	68	41	61	51	61	bphl
ENSXETG00000018036	2973	2	7	5	34	29	52	399	449	231	84	60	58	bnc2
ENSXETG00000018037	1296	2	8	5	8	8	3	197	228	117	28	24	39	serpinb4
ENSXETG00000018039	774	4	2	0	15	12	28	412	440	319	53	48	46	cntfr
ENSXETG00000018040	2196	0	0	0	5	1	3	1	3	2	4	1	1	ntrk1
ENSXETG00000018041	3603	32	53	42	145	93	466	393	452	260	595	454	477	kcnma1
ENSXETG00000018044	3197	80	101	105	159	122	195	236	287	160	114	82	89	gan
ENSXETG00000018046	1149	240	388	373	334	235	409	255	276	183	329	199	284	ano9
ENSXETG00000018051	738	0	0	0	5	13	3	7	10	6	2	4	3	gjb7
ENSXETG00000018052	3267	3	3	6	24	25	36	149	190	110	67	45	53	pear1
ENSXETG00000018054	3118	28	47	39	246	156	163	112	99	90	126	84	101	maats1
ENSXETG00000018057	2686	837	1251	1052	1511	1209	1910	2586	2937	1798	2412	1744	2040	spred1
ENSXETG00000018061	2416	91	128	108	112	83	65	301	371	170	46	23	45	fam98b
ENSXETG00000018063	369	0	3	0	1	0	4	85	98	44	35	28	26	cga
ENSXETG00000018064	1119	25	23	39	117	78	151	318	365	218	396	231	266	popdc2
ENSXETG00000018065	2220	0	0	0	1	1	0	1	0	0	2	0	4	adarb2
ENSXETG00000018066	1316	2017	2897	2453	2126	1634	4075	941	1168	643	1732	1303	1386	eci2
ENSXETG00000018073	837	0	0	0	0	0	0	0	0	0	0	0	0	
ENSXETG00000018074	4716	35	56	31	76	53	153	426	451	289	177	142	157	rasgrp1
ENSXETG00000018075	2444	1165	1705	1579	1877	1389	1627	4221	4936	3010	1957	1374	1622	syncrip
ENSXETG00000018076	4849	93	111	101	365	313	501	452	568	305	478	348	413	etv3
ENSXETG00000018079	1008	0	0	3	0	6	2	0	0	0	41	37	26	hsd3b1
ENSXETG00000018081	2071	78	131	106	288	215	324	470	568	298	43	40	47	prc1.2
ENSXETG00000018082	1236	2452	3639	3217	4063	3021	6246	792	917	620	4701	3477	3554	hao2
ENSXETG00000018083	2209	111	162	108	238	204	399	797	964	599	510	365	426	wdr37
ENSXETG00000018085	2351	993	1456	1250	1368	1164	1770	2648	3256	1805	1844	1270	1436	arrdc3
ENSXETG00000018086	3081	212	326	248	568	367	550	1571	1810	1058	1061	721	837	adamtsl4
ENSXETG00000018088	1363	3900	5734	4820	3286	2566	2104	886	1032	671	877	569	588	idi1
ENSXETG00000018089	3621	71	141	87	211	204	300	613	671	380	219	130	154	arhgap11a.1
ENSXETG00000018090	5811	376	554	532	1131	895	2045	2044	2296	1386	1640	1143	1356	dlg5
ENSXETG00000018091	2935	361	467	385	423	309	717	566	689	389	449	340	412	snx14
ENSXETG00000018094	1043	71	93	100	203	162	148	420	466	274	358	230	245	scg5
ENSXETG00000018095	1293	61	53	62	113	95	384	300	344	202	508	334	390	grem1
ENSXETG00000018096	1749	53	78	63	330	240	567	279	377	194	807	582	716	nt5e
ENSXETG00000018097	1866	4554	6431	5759	12875	9945	18970	12570	13690	8398	76296	53538	59152	gtpbp4
ENSXETG00000018101	5174	374	556	466	683	527	564	699	757	466	815	564	637	polr3a
ENSXETG00000018102	2847	124	181	164	413	284	226	60	57	36	153	120	121	ENSXETG00000018102
ENSXETG00000018104	8412	174	219	209	275	188	459	239	316	184	673	471	515	fmn1
ENSXETG00000018105	4303	378	471	461	1309	1042	1930	3684	4181	2436	1925	1299	1539	micall2
ENSXETG00000018106	2726	16	28	18	8	9	18	87	105	62	47	43	43	prkcq
ENSXETG00000018108	870	18	33	23	35	22	99	105	152	90	91	58	69	ENSXETG00000018108
ENSXETG00000018110	6543	359	530	428	818	642	914	1083	1260	745	992	653	758	INTS1
ENSXETG00000018114	4179	157	235	246	552	424	1171	545	635	394	872	624	661	phka2
ENSXETG00000018118	471	23	18	28	23	19	36	66	62	40	60	43	42	mafk
ENSXETG00000018120	1819	1296	1777	1538	2799	2122	7670	1486	1682	1017	2307	1555	1758	tmem184a
ENSXETG00000018121	4056	896	1248	1012	720	506	1557	2814	3383	1983	1173	859	972	pfkfb3
ENSXETG00000018122	533	148	204	145	139	128	222	323	404	251	184	122	144	psmg3
ENSXETG00000018123	1473	387	562	518	607	482	590	1158	1267	739	649	475	533	rbm17
ENSXETG00000018124	696	716	1100	891	2058	1556	2129	3111	3722	2200	4634	3353	3763	cldn2
ENSXETG00000018125	3081	329	473	422	468	340	970	738	820	476	1333	917	1035	fbxo18
ENSXETG00000018127	1837	21	16	25	46	35	21	34	35	26	11	13	11	IRAK1BP1
ENSXETG00000018128	4788	1	1	2	2	0	4	21	31	21	9	6	4	frmpd3
ENSXETG00000018131	852	0	3	3	0	1	1	23	15	17	8	3	4	slc25a53
ENSXETG00000018132	1575	24	19	20	36	20	72	147	173	105	85	54	67	diaph2
ENSXETG00000018133	1820	9	20	16	28	29	114	341	415	245	74	60	73	rab9b
ENSXETG00000018134	2626	0	1	0	2	2	8	27	34	16	11	4	14	plp1
ENSXETG00000018137	667	28757	42871	37543	37300	28314	36126	36870	42280	27639	78346	54289	61558	rps24
ENSXETG00000018144	3424	20	37	26	93	71	151	220	252	164	24	24	27	DIAPH3
ENSXETG00000018146	1095	474	709	594	604	465	674	918	1049	569	414	310	334	gla
ENSXETG00000018147	1131	251	373	308	251	202	379	355	443	296	428	329	315	ankrd16
ENSXETG00000018148	1734	0	0	0	0	0	0	0	0	0	0	0	0	
ENSXETG00000018149	2617	7144	10254	8374	9516	7163	13222	10864	13125	7514	10129	7019	8032	gdi2
ENSXETG00000018150	397	40305	59838	50527	47421	35299	48263	48467	54530	31228	99555	68041	73596	rpl36a
ENSXETG00000018152	2388	21	29	25	38	16	101	471	544	319	137	70	107	btk
ENSXETG00000018157	612	0	0	0	1	3	5	1	0	0	2	2	0	mrap2
ENSXETG00000018158	1782	106	202	145	120	87	352	149	195	102	304	245	204	asb13
ENSXETG00000018160	3241	2532	3549	3053	2171	1723	4730	3866	4811	2632	3482	2390	2771	net1
ENSXETG00000018162	816	13	14	14	43	24	40	54	39	34	25	20	17	fam151b
ENSXETG00000018164	2220	85	140	98	337	222	396	794	845	566	723	476	539	sema4a
ENSXETG00000018165	1703	175	241	196	249	219	332	380	470	299	550	369	388	ccdc125
ENSXETG00000018166	1711	0	0	0	0	0	0	0	0	0	0	0	0	tuba3e
ENSXETG00000018168	2232	376	515	451	430	365	722	849	1034	558	661	481	536	rad17
ENSXETG00000018169	1471	168	228	190	193	150	419	524	671	369	386	256	276	XB-GENE-5783434
ENSXETG00000018170	1484	1	3	4	3	5	3	15	20	6	3	8	3	XB-GENE-5847737
ENSXETG00000018171	1119	475	712	633	766	605	971	1290	1419	855	696	504	518	taf7
ENSXETG00000018172	1429	740	1066	942	1098	794	1181	846	901	531	1204	832	863	nif3l1
ENSXETG00000018173	2211	306	445	350	1015	772	1405	724	987	600	973	703	749	mkln1

ENSXETG00000018174	1725	521	717	620	289	218	476	625	671	439	492	370	415	ak6
ENSXETG00000018175	4522	94	134	110	116	86	215	57	64	46	94	57	66	drp2
ENSXETG00000018176	1173	102	143	146	104	81	75	175	204	134	45	25	37	ppil3
ENSXETG00000018177	2036	377	566	516	1062	780	1780	975	1038	653	1205	888	1021	marveld2
ENSXETG00000018178	837	2	4	1	1	5	1	2	5	1	12	5	4	ENSXETG00000018178
ENSXETG00000018180	2310	33	41	40	156	88	274	290	331	191	298	209	216	exoc6
ENSXETG00000018182	3022	2438	3346	2889	4564	3553	7535	3824	4609	2677	5304	3601	4155	ocln
ENSXETG00000018183	3232	3	0	2	10	9	10	55	60	24	26	15	24	rab33a
ENSXETG00000018184	1796	2	3	1	35	32	7	538	550	318	41	18	13	cyp26a1
ENSXETG00000018186	2429	2870	4334	3735	5337	4111	4357	2407	2689	1651	3031	2212	2467	aifm1
ENSXETG00000018187	1707	123	158	141	74	86	115	184	165	128	134	86	95	mars2
ENSXETG00000018189	1093	972	1469	1299	1027	752	1042	1520	1709	1040	1001	657	777	smn2
ENSXETG00000018191	1584	3795	5603	4797	3375	2594	4042	1083	1209	724	1550	1112	1186	faah2
ENSXETG00000018194	2120	4	8	8	50	40	52	1101	1218	720	355	235	313	eva1a
ENSXETG00000018197	1086	0	0	0	0	0	0	0	0	0	0	0	0	
ENSXETG00000018198	753	958	1460	1235	558	372	455	776	934	469	554	399	421	mrpl19
ENSXETG00000018202	1435	0	6	4	52	36	3	2	9	0	9	13	4	fam228b
ENSXETG00000018203	1489	213	294	247	442	326	384	108	110	75	528	357	363	tp53i3
ENSXETG00000018204	939	988	1437	1205	1089	874	1572	1715	2162	1135	1134	817	931	sf3b6
ENSXETG00000018208	4917	62	95	60	72	49	106	238	336	173	105	97	100	wdcp
ENSXETG00000018209	1944	7	4	3	8	6	18	535	541	354	20	8	14	mfsd2b
ENSXETG00000018210	1341	2245	3112	2662	2883	2178	5566	5314	6080	3482	5171	3646	3982	ubxn2a
ENSXETG00000018211	4146	108	171	169	286	237	538	1765	2090	1264	424	347	338	atad2b
ENSXETG00000018212	2196	2	0	0	0	4	9	39	46	14	12	16	11	klhl29
ENSXETG00000018213	1792	414	650	550	804	675	774	526	705	398	629	476	538	mccc2
ENSXETG00000018214	4986	94	144	119	133	101	307	485	573	310	300	203	245	mbd5
ENSXETG00000018225	1058	167	233	233	244	203	456	345	388	239	391	264	286	cyb5d2
ENSXETG00000018227	945	0	0	0	0	0	0	0	0	0	0	0	0	
ENSXETG00000018229	2408	191	300	266	402	337	500	593	690	422	576	388	422	ercc2
ENSXETG00000018230	1833	1076	1532	1237	1881	1462	2484	2491	3067	1856	1835	1294	1507	eif4h
ENSXETG00000018234	1082	237	365	291	339	237	181	727	828	457	217	169	164	rfc2
ENSXETG00000018235	3040	387	627	524	597	426	466	683	739	467	542	366	443	ppp1r13l
ENSXETG00000018237	894	5	3	11	36	37	42	25	15	9	19	5	6	fosb
ENSXETG00000018239	2152	1352	1826	1634	2067	1660	3552	8275	9378	5604	3322	2265	2571	vasp
ENSXETG00000018240	456	388	575	455	373	264	417	536	594	413	217	152	189	opa3
ENSXETG00000018242	2755	783	1162	1029	1253	939	1005	1969	2149	1338	1343	934	997	eml1
ENSXETG00000018246	405	0	0	0	0	0	0	0	0	0	0	0	0	
ENSXETG00000018247	1911	1	1	0	0	1	2	4	11	6	7	1	4	kcna4
ENSXETG00000018255	1206	3	1	0	2	5	6	28	19	14	35	27	18	gipr
ENSXETG00000018261	1255	302	426	397	322	264	424	686	880	526	161	128	149	qpctl
ENSXETG00000018262	741	4	4	5	66	53	23	1	3	1	237	142	170	pglyrp1
ENSXETG00000018263	972	1	0	0	26	22	2	1	0	2	18	20	15	ENSXETG00000018263
ENSXETG00000018265	1793	1382	2053	1640	1464	1129	1309	2120	2526	1436	909	628	746	ppp5c
ENSXETG00000018268	1509	6906	9970	8118	8923	6940	25447	3269	4069	2213	3078	2180	2495	slc7a9
ENSXETG00000018269	2193	34	48	24	149	106	258	296	316	178	175	138	140	cep89
ENSXETG00000018270	1029	35	68	53	132	82	302	264	288	183	187	133	161	ptgir
ENSXETG00000018271	3322	462	598	499	1935	1523	2432	3544	4100	2223	2314	1691	1899	rhpn2
ENSXETG00000018272	6585	0	0	0	0	0	0	0	1	1	0	0	0	
ENSXETG00000018274	1790	89	151	131	125	109	171	323	361	206	378	239	282	fkrp
ENSXETG00000018275	1708	170	229	205	579	408	111	595	681	410	198	153	168	slc1a5
ENSXETG00000018276	2919	126	201	148	477	328	610	594	654	358	573	381	466	gpatch1
ENSXETG00000018277	1498	1	1	1	0	0	1	1	4	1	4	1	4	wdr88
ENSXETG00000018279	1431	2	2	0	0	1	0	1	1	0	0	0	0	
ENSXETG00000018280	761	1	4	3	8	6	1	5	5	3	6	2	2	ENSXETG00000018280
ENSXETG00000018283	747	984	1419	1158	962	727	1319	1822	2117	1200	809	602	657	ap2s1
ENSXETG00000018284	4413	368	515	459	1177	935	1747	2225	2480	1503	1758	1232	1422	arhgap35
ENSXETG00000018287	2943	55	63	82	75	58	108	94	78	48	127	89	94	c2orf69
ENSXETG00000018288	1547	32	47	32	23	20	33	52	61	37	30	20	27	XB-GENE-5818975
ENSXETG00000018290	3285	104	121	109	135	126	278	358	504	277	306	232	286	atp11c
ENSXETG00000018291	3206	239	395	309	6012	4705	1657	6096	6885	4144	7936	5390	5911	cebpa
ENSXETG00000018292	1110	4	10	10	38	13	34	55	45	17	47	31	32	arpc1a
ENSXETG00000018293	687	3	5	4	20	12	41	46	50	38	117	83	106	kcnip1
ENSXETG00000018294	603	207	344	301	750	523	1067	1245	1397	953	960	671	738	ENSXETG00000018294
ENSXETG00000018296	1306	9	12	12	30	34	46	92	85	43	68	47	46	fam20a
ENSXETG00000018297	1538	17800	26136	22549	19375	14400	28947	14127	16650	9772	10713	7527	8502	pepd
ENSXETG00000018298	1317	0	0	0	0	0	0	0	0	0	0	0	0	
ENSXETG00000018299	828	2	6	10	9	13	20	47	65	38	21	13	22	purg
ENSXETG00000018300	1236	0	0	0	0	0	0	0	0	0	0	0	0	
ENSXETG00000018301	873	49	70	64	131	80	130	290	320	183	105	92	74	ergic1
ENSXETG00000018302	4393	198	301	229	371	253	560	570	669	392	669	419	502	wrn
ENSXETG00000018304	2102	0	0	0	0	0	0	0	0	0	0	0	0	
ENSXETG00000018306	2811	74	120	107	225	173	366	581	721	429	401	309	341	ENSXETG00000018306
ENSXETG00000018309	1738	2009	3002	2581	3230	2435	4496	3788	4372	2571	5824	4099	4603	ppp2cb
ENSXETG00000018313	554	480	718	564	560	434	763	1069	1165	719	635	523	575	pfdn1
ENSXETG00000018316	2690	535	795	653	876	707	1282	1207	1449	817	1470	1024	1097	rufy1
ENSXETG00000018317	2116	578	859	690	703	620	1723	802	997	517	876	653	698	mpv17l2
ENSXETG00000018319	1425	4935	7034	6055	4200	3444	5429	173	184	130	1525	1057	1244	enpp7-l
ENSXETG00000018320	5763	2726	3713	2986	2266	1851	3841	1896	2338	1284	1523	1049	1199	sfxn1
ENSXETG00000018321	1965	1395	2239	1943	2799	2138	2719	6698	7472	4740	2894	2053	2120	hnrnph1
ENSXETG00000018322	3159	7	6	7	44	23	45	18	17	12	180	131	182	mcf2
ENSXETG00000018326	1061	688	938	822	1093	834	1373	1992	2253	1386	830	592	692	ube2m
ENSXETG00000018328	930	4	6	6	15	13	48	44	47	27	21	20	14	GSG1L
ENSXETG00000018329	1041	0	0	0	0	0	0	0	0	0	0	0	0	
ENSXETG00000018331	1787	78	144	82	223	165	56	209	274	159	45	33	42	dck.2
ENSXETG00000018333	4770	26	36	25	88	61	160	131	191	87	100	64	88	kiaa0556
ENSXETG00000018334	2928	2	2	2	2	2	1	7	6	3	1	1	0	slc4a9
ENSXETG00000018337	6911	497	779	633	1796	1393	2130	1937	2242	1324	2516	1760	2036	ankhd1
ENSXETG00000018341	2238	2	1	1	3	1	1	14	19	17	9	4	6	myot
ENSXETG00000018359	3387	57	95	79	190	156	347	653	748	407	536	344	398	zmiz1
ENSXETG00000018362	360	1422	2147	1768	2359	1867	2679	4649	5097	3263	2277	1634	1744	elob
ENSXETG00000018363	2145	252	391	353	684	517	305	474	513	308	381	295	329	pkmyt1
ENSXETG00000018364	819	2	1	1	7	3	4	48	63	29	6	4	7	paqr4
ENSXETG00000018365	1272	0	0	0	0	0	0	2	2	0	0	1	0	
ENSXETG00000018366	1133	949	1311	1007	678	540	358	475	595	310	227	163	185	ppif
ENSXETG00000018368	988	480	774	625	349	300	460	709	822	501	347	237	239	pelo
ENSXETG00000018372	720	0	1	2	5	5	4	112	123	65	13	7	21	zcchc24
ENSXETG00000018373	318	1754	2604	2327	1761	1237	1385	1147	1257	858	955	671	770	ndufa13
ENSXETG00000018374	1251	0	0	0	0	0	0	0	0	0	0	0	1	
ENSXETG00000018375	1497	7359	10838	9111	12240	9593	18805	12196	13889	8158	11493	8159	8632	anxa11
ENSXETG00000018377	1095	0	0	0	0	0	0	2	1	3	0	0	3	gprin2
ENSXETG00000018379	1437	143	221	160	396	323	341	318	437	204	304	227	244	haus7
ENSXETG00000018380	2466	0	1	0	0	0	0	0	1	0	0	0	1	ENSXETG00000018380
ENSXETG00000018381	1041	13	32	15	6	4	31	94	154	70	26	29	40	ENSXETG00000018381
ENSXETG00000018382	2321	3637	5172	4415	2237	1662	2601	1331	1555	872	1370	935	1050	tmlhe
ENSXETG00000018384	2073	1040	1528	1357	2549	1901	2370	3836	4049	2633	2114	1494	1533	stim1
ENSXETG00000018385	720	37	53	41	36	30	71	96	120	76	60	43	46	ENSXETG00000018385
ENSXETG00000018386	2166	0	0	0	0	0	0	0	0	0	0	0	0	
ENSXETG00000018387	3147	0	1	0	8	3	16	61	87	47	10	10	11	ednrb2
ENSXETG00000018389	663	127	261	199	286	205	588	374	401	249	309	233	231	vamp7
ENSXETG00000018391	5895	12	19	7	187	143	230	862	1001	573	501	337	418	sema6d
ENSXETG00000018392	1833	90	127	115	159	129	312	515	559	439	214	144	146	slc12a9

ENSXETG00000018402	1716	792	1095	917	1142	818	806	256	277	152	323	244	251	pyroxd2
ENSXETG00000018403	1599	8	14	14	22	12	39	62	75	38	23	26	14	ENSXETG00000018403
ENSXETG00000018404	1152	669	984	818	1227	910	2280	5104	5702	3426	1277	923	1000	ENSXETG00000018404
ENSXETG00000018406	1392	0	1	5	3	3	5	2	4	0	1	2	5	slc24a5
ENSXETG00000018409	2264	176	259	221	271	221	412	434	491	285	327	253	273	rspry1
ENSXETG00000018410	1880	129	196	169	156	113	203	329	421	249	161	106	126	arl2bp
ENSXETG00000018412	2413	1604	2370	2000	4107	3152	3112	5740	6415	3891	2425	1687	1832	myef2
ENSXETG00000018415	3696	376	530	473	1023	759	939	921	983	632	1476	984	1028	gigyf1
ENSXETG00000018416	1725	14	30	33	818	643	375	54	67	42	474	380	400	clcnkb
ENSXETG00000018417	4817	6	3	2	6	2	8	291	340	182	50	33	33	sall1
ENSXETG00000018422	1710	1	5	0	11	9	59	81	115	61	63	57	57	hpse2
ENSXETG00000018423	1578	69	103	85	236	167	867	319	340	203	483	362	392	ENSXETG00000018423
ENSXETG00000018427	1806	6	11	16	45	41	77	116	142	84	125	88	81	cnnm1
ENSXETG00000018428	1330	3357	5143	4511	6456	4857	9589	2575	3035	1767	4072	2878	3207	got1
ENSXETG00000018430	987	20	26	34	164	106	292	1578	1760	1012	526	341	435	nkx2-3
ENSXETG00000018432	708	2	0	3	5	2	14	11	12	9	10	12	6	nkx3-3
ENSXETG00000018433	4828	313	407	351	473	388	1025	1181	1474	754	934	615	733	fbxo30
ENSXETG00000018434	1564	1190	1841	1609	1271	990	1878	2732	3105	1869	1672	1208	1220	myd88
ENSXETG00000018435	3906	192	262	230	428	351	582	971	1133	624	710	480	545	gtf2i
ENSXETG00000018441	1870	2979	4333	3559	2305	1795	2330	408	544	268	501	316	342	nsdhl
ENSXETG00000018442	1722	80	162	153	363	254	602	353	360	245	645	462	526	map3k3
ENSXETG00000018443	1221	208	256	197	326	272	522	483	690	343	253	197	260	cetn1
ENSXETG00000018445	1740	2	6	2	29	10	52	106	147	91	60	40	36	lpar4
ENSXETG00000018446	1257	7860	11253	9601	10099	7912	14606	2564	2793	1787	4684	3463	3553	acaa1
ENSXETG00000018447	645	0	0	0	0	0	0	0	1	0	0	0	0	ENSXETG00000018447
ENSXETG00000018448	2005	145	236	192	162	126	342	1486	1719	981	393	276	277	ncf1
ENSXETG00000018449	1562	30	32	30	57	45	219	161	159	107	177	102	145	gatsl2
ENSXETG00000018450	957	232	313	309	279	216	711	594	707	453	307	225	246	c11orf54
ENSXETG00000018451	5028	0	0	1	85	54	5	4	7	8	11	2	5	dlec1
ENSXETG00000018453	3798	792	1179	949	1747	1325	897	425	582	337	653	462	549	mtr
ENSXETG00000018458	2283	214	352	289	1220	946	630	1702	1828	1197	2324	1618	1738	plcd1
ENSXETG00000018460	4089	734	1018	944	1162	887	1744	2380	2881	1513	1709	1227	1363	brd7
ENSXETG00000018461	2608	537	783	669	954	739	877	1803	2088	1192	677	486	529	nup93
ENSXETG00000018462	3393	5	8	2	25	28	87	227	244	136	72	52	69	nkd1
ENSXETG00000018463	765	2	3	3	5	6	12	28	42	16	13	5	18	ENSXETG00000018463
ENSXETG00000018464	1541	130	218	154	169	138	265	411	518	292	243	185	196	pskh1
ENSXETG00000018465	1194	557	826	714	1320	921	3207	1649	1980	1235	1939	1360	1515	rell1
ENSXETG00000018469	1849	546	810	705	1154	859	1014	687	844	435	976	686	817	pgm2
ENSXETG00000018470	2702	101	149	122	1967	1512	539	1407	1416	938	1974	1419	1567	vill
ENSXETG00000018471	822	32	45	34	164	118	167	189	191	107	330	231	239	ctdspl
ENSXETG00000018473	3937	76	83	90	386	278	583	1106	1322	783	443	311	343	tbc1d1
ENSXETG00000018474	2493	2855	4218	3673	6326	4735	7453	8692	9539	5853	5952	4230	4422	gars
ENSXETG00000018476	1041	121	175	161	376	300	1045	1169	1197	741	810	602	660	klf3
ENSXETG00000018477	2349	61	89	85	181	151	185	579	750	365	429	279	334	tlr6
ENSXETG00000018480	1960	719	1052	848	826	622	1814	349	399	208	935	676	729	rmdn2
ENSXETG00000018482	699	0	0	0	18	9	6	3	1	1	13	16	22	ENSXETG00000018482
ENSXETG00000018483	3247	220	334	313	438	363	599	691	803	499	691	477	459	cwf19l2
ENSXETG00000018487	2207	70	79	79	205	132	188	820	930	526	267	196	212	cdc42ep3
ENSXETG00000018489	2352	52	58	52	33	27	39	55	50	23	35	19	35	ENSXETG00000018489
ENSXETG00000018490	3112	64	99	82	151	105	184	131	167	91	235	148	177	nlrc4
ENSXETG00000018492	1137	1	2	0	1	0	1	5	9	4	12	9	3	XB-GENE-5960290
ENSXETG00000018493	2286	0	0	1	6	4	24	76	119	52	16	14	15	gucy1a2
ENSXETG00000018494	1618	293	393	358	106	101	312	321	417	235	138	92	135	thumpd2
ENSXETG00000018496	915	1	1	2	4	1	22	25	17	11	46	35	45	tmem178a
ENSXETG00000018498	1380	265	339	320	409	295	417	501	582	314	200	163	156	fam114a1
ENSXETG00000018499	1395	0	2	1	5	0	3	42	35	24	8	8	14	ENSXETG00000018499
ENSXETG00000018501	4144	188	256	223	581	422	1471	1208	1423	822	1188	861	1007	map4k3
ENSXETG00000018502	1002	69	93	81	82	67	242	87	89	45	136	89	124	crhr2
ENSXETG00000018504	6839	1335	1925	1611	3231	2352	3675	3177	3513	2146	3852	2652	2995	cul9
ENSXETG00000018507	2274	7	10	13	48	35	189	627	670	433	287	200	243	klhl5
ENSXETG00000018511	1458	386	583	440	410	309	508	1248	1289	925	576	414	451	srf
ENSXETG00000018513	2683	598	903	725	546	366	1129	1029	1295	737	721	560	627	fez2
ENSXETG00000018514	845	640	959	790	746	619	865	1035	1066	660	787	558	595	mrpl24
ENSXETG00000018516	3408	904	1348	1111	2357	1821	3005	3957	4391	2665	3051	2144	2367	hdgf
ENSXETG00000018517	3449	485	651	557	1106	912	1386	1781	1994	1255	1682	1297	1339	prcc
ENSXETG00000018519	291	262	428	412	385	302	541	876	1045	651	441	301	346	drap1
ENSXETG00000018525	888	6	5	3	27	18	10	547	542	391	58	47	58	ENSXETG00000018525
ENSXETG00000018527	1569	0	0	1	0	1	0	4	4	0	0	0	0	pax7
ENSXETG00000018528	807	124	163	135	222	152	278	356	445	259	301	222	241	gatad1
ENSXETG00000018529	975	55	53	58	90	75	113	461	500	336	183	123	119	cdk6
ENSXETG00000018531	1781	137	173	162	198	121	371	290	355	203	350	241	266	aldh4a1
ENSXETG00000018532	1410	1	7	8	31	39	66	256	312	207	79	46	60	iffo2
ENSXETG00000018537	783	36	50	31	30	41	85	45	64	38	38	37	46	LRRC3C
ENSXETG00000018541	801	126	211	167	84	68	155	116	140	93	141	104	94	mtrf1l
ENSXETG00000018544	1991	443	601	526	459	356	519	840	971	585	405	278	279	gpatch11
ENSXETG00000018545	1662	31	47	41	77	49	60	129	189	101	17	14	20	fbxo5
ENSXETG00000018546	669	0	0	0	0	0	0	0	0	1	0	0	0	
ENSXETG00000018547	1092	4	1	5	4	3	9	48	46	32	20	18	16	gata1
ENSXETG00000018550	2569	405	616	509	859	702	1336	1336	1495	957	1337	1013	999	enah
ENSXETG00000018551	1945	74	119	105	460	361	486	986	1175	708	466	300	368	lbr
ENSXETG00000018552	1560	2480	3692	3105	3808	2960	5766	5772	6526	3798	3764	2775	3023	psmd3
ENSXETG00000018553	10510	2	1	1	73	50	4	87	83	56	10	11	8	dnah14
ENSXETG00000018563	2271	310	438	415	1177	886	2960	2803	3023	1922	1655	1244	1222	wdr26
ENSXETG00000018564	2097	4412	6425	5317	3075	2425	1183	1552	1931	1006	581	469	404	sdf2l1
ENSXETG00000018565	3629	238	407	333	608	494	743	878	1124	620	557	388	427	top3b
ENSXETG00000018568	420	651	992	875	494	404	634	913	970	696	398	257	314	cnih4
ENSXETG00000018569	2823	404	629	541	570	515	588	713	825	487	784	615	639	nvl
ENSXETG00000018572	1342	144	184	153	283	232	514	1020	1117	660	537	345	431	ppm1f
ENSXETG00000018573	3198	2	4	2	94	69	7	15	3	6	8	7	5	foxn4
ENSXETG00000018576	1106	804	1206	1030	1513	1199	1780	1837	2234	1355	1718	1147	1414	mapk1
ENSXETG00000018580	3114	0	0	0	0	1	0	1	0	0	0	0	1	
ENSXETG00000018586	972	1687	2539	2177	1755	1488	2222	2293	2562	1725	967	670	758	degs1
ENSXETG00000018588	1669	688	1007	867	747	622	1091	1701	2076	1171	856	607	700	kctd10
ENSXETG00000018589	1050	150	236	182	390	303	704	691	736	471	708	502	541	fbxo28
ENSXETG00000018590	3989	146	181	169	427	318	671	700	804	489	477	380	463	ube3b
ENSXETG00000018593	4029	741	1086	905	1108	866	1910	2279	2624	1586	2027	1381	1546	tp53bp2
ENSXETG00000018594	2694	353	540	430	841	628	1318	1259	1423	903	1345	915	1043	ppil2
ENSXETG00000018597	2102	0	0	0	0	0	0	0	2	1	1	0	0	
ENSXETG00000018598	3045	509	810	700	1368	1130	2566	2922	3354	1925	2014	1479	1550	hira
ENSXETG00000018599	2155	89	118	102	700	523	170	2169	2420	1521	1628	1192	1292	capn2.2
ENSXETG00000018600	873	834	1206	1027	779	580	714	1006	1077	686	481	307	341	mrpl40
ENSXETG00000018601	1427	956	1374	1155	1252	933	1949	2613	3113	1787	887	604	710	ufd1l
ENSXETG00000018602	1909	95	146	101	225	165	68	315	363	198	35	38	39	cdc45
ENSXETG00000018603	1197	302	367	339	266	231	291	103	112	58	94	71	66	mvk
ENSXETG00000018604	2559	81	128	101	583	414	854	1924	2232	1254	1278	896	936	cldn5
ENSXETG00000018605	1689	866	1247	1079	1865	1321	1500	5462	6446	3672	1612	1104	1310	sept2-a
ENSXETG00000018606	2779	24	31	26	44	33	67	161	172	103	53	53	38	fam222a
ENSXETG00000018607	2178	20	17	17	679	528	51	79	102	57	768	583	658	capn8
ENSXETG00000018608	3757	24	40	24	147	92	140	540	588	361	360	255	284	atp8a1
ENSXETG00000018609	2607	19	30	24	74	60	178	314	389	190	91	71	67	trpv4
ENSXETG00000018610	3873	3065	4519	3751	4047	3023	9606	640	724	389	3589	2702	2930	gk

ENSXETG00000018612	750	14	12	15	13	14	55	365	386	243	103	79	90	c3orf70
ENSXETG00000018613	4313	397	563	484	763	574	1479	935	1042	638	1069	699	809	vps8
ENSXETG00000018616	1668	1	1	1	0	1	0	0	2	1	2	1	2	habp2
ENSXETG00000018617	564	674	997	909	698	500	993	922	1083	634	1199	818	910	rpe
ENSXETG00000018619	926	1	9	5	7	1	15	44	47	23	16	6	6	cxorf21
ENSXETG00000018620	1192	372	524	403	500	419	1725	1185	1452	817	846	655	720	prrg1
ENSXETG00000018622	777	2	1	0	2	3	6	7	6	1	2	5	3	lrrc3
ENSXETG00000018623	1224	0	0	0	0	0	1	0	0	0	0	0	0	lancl3
ENSXETG00000018624	1953	1	1	0	1	0	7	0	0	0	0	0	0	tspear
ENSXETG00000018625	2107	1208	1737	1505	1243	889	2795	887	1169	589	812	604	678	gltp
ENSXETG00000018626	3096	0	0	0	0	0	7	9	12	7	4	4	4	xk
ENSXETG00000018628	1835	1716	2397	1977	1607	1161	2332	2683	3374	1806	1526	1116	1271	ube2g2
ENSXETG00000018630	1871	195	263	266	232	164	669	1592	1826	1122	393	263	346	cybb
ENSXETG00000018631	1428	0	1	1	11	9	0	11	8	8	1	2	4	nxph3
ENSXETG00000018632	1302	0	0	0	14	6	16	109	124	66	40	28	26	XB-GENE-977685
ENSXETG00000018633	1620	62	81	62	152	123	146	245	239	148	129	84	114	tchp
ENSXETG00000018634	2307	84	118	129	167	172	345	1595	1981	1101	416	233	330	itgb2
ENSXETG00000018635	5843	23	28	32	135	113	217	1475	1611	1046	324	262	251	plxna1
ENSXETG00000018638	2214	88	82	95	157	108	272	1033	1134	657	362	273	298	git2
ENSXETG00000018640	2034	233	302	262	221	178	520	441	509	279	399	331	325	TARS2
ENSXETG00000018641	3228	264	358	316	518	444	619	882	1062	653	713	490	552	znf652
ENSXETG00000018642	1901	45	66	63	124	89	169	380	420	286	191	160	162	pias3
ENSXETG00000018643	717	22	60	41	15	8	3	6	9	8	0	1	0	ENSXETG00000018643
ENSXETG00000018646	2205	1	2	0	10	5	18	62	64	57	30	26	26	SH3RF3
ENSXETG00000018647	441	2	0	0	0	0	0	19	25	18	1	0	0	mgc89221
ENSXETG00000018648	3418	1381	1946	1691	1448	1075	2441	1063	1136	679	1494	1053	1131	ENSXETG00000018648
ENSXETG00000018649	2176	1790	2592	2177	3519	2742	6741	4160	4763	2702	5568	3715	4247	tmem98
ENSXETG00000018650	1719	0	0	0	0	0	0	0	0	0	0	0	0	
ENSXETG00000018651	2472	57	105	85	138	114	205	361	400	248	267	182	220	znf281
ENSXETG00000018652	4134	291	458	384	943	670	1445	798	851	482	958	688	808	rprd2
ENSXETG00000018653	1626	3	3	3	62	44	30	51	50	26	12	12	10	ENSXETG00000018653
ENSXETG00000018654	2239	348	521	429	811	643	1068	1219	1351	801	994	699	783	prpf3
ENSXETG00000018658	1491	2953	4424	3692	6437	4865	20737	1431	1615	942	5559	4089	4594	ENSXETG00000018658
ENSXETG00000018660	1050	13	20	20	41	40	94	242	279	174	227	158	207	tpbg
ENSXETG00000018661	2680	697	1024	856	1302	1101	1518	1792	2152	1208	1619	1161	1294	irf6.2
ENSXETG00000018663	969	1	9	8	78	55	44	89	84	68	163	103	112	ENSXETG00000018663
ENSXETG00000018665	1852	183	269	265	408	323	761	1267	1521	929	597	418	476	ankrd13a
ENSXETG00000018672	3510	3	13	14	60	40	33	55	42	23	85	50	52	sgsm1
ENSXETG00000018674	2393	423	662	549	1019	816	1175	892	1078	608	1126	807	905	irf6
ENSXETG00000018679	4044	496	709	613	1134	929	1505	1514	1832	1067	1077	714	870	ibtk
ENSXETG00000018681	1281	256	379	329	334	266	476	395	456	253	391	312	275	nudt17
ENSXETG00000018682	816	657	937	850	1237	953	1829	860	924	642	877	622	677	rnf115
ENSXETG00000018683	2058	669	965	941	1262	1002	1209	643	677	410	743	570	543	ENSXETG00000018683
ENSXETG00000018684	1903	243	330	283	344	278	591	746	829	463	682	504	524	chchd6
ENSXETG00000018685	1209	89	129	127	193	150	362	480	530	327	317	271	283	fam46a
ENSXETG00000018688	2729	1	4	1	48	38	27	486	536	326	97	97	83	ENSXETG00000018688
ENSXETG00000018689	3750	491	755	662	2441	1809	2299	3526	4083	2375	2570	1936	2011	smc3
ENSXETG00000018690	1034	439	610	490	704	481	841	976	1030	614	1319	877	966	fam207a
ENSXETG00000018692	4618	113	145	120	138	121	183	349	431	229	192	144	141	adarb1
ENSXETG00000018694	2110	3	18	13	5	4	9	61	56	50	11	8	6	alox12b
ENSXETG00000018696	2864	745	995	759	2725	2107	7799	1642	1946	1168	5168	3699	4294	XB-GENE-5744235
ENSXETG00000018700	1311	117	169	157	208	160	181	461	506	272	222	191	192	pofut2
ENSXETG00000018705	2311	79	94	86	189	141	417	787	807	544	251	186	193	dusp5
ENSXETG00000018707	2566	37	48	37	460	323	139	266	344	202	482	355	383	capsl
ENSXETG00000018708	2789	50	52	49	304	224	385	1803	2242	1265	379	242	288	axl
ENSXETG00000018709	1500	0	0	0	0	0	0	0	0	0	0	0	0	
ENSXETG00000018712	2218	547	792	756	1322	883	2844	1045	1127	751	2183	1535	1601	arhgef5
ENSXETG00000018713	4809	0	0	0	0	0	1	0	0	0	0	0	0	
ENSXETG00000018714	600	16	21	19	62	84	200	86	114	59	74	65	78	mxi1
ENSXETG00000018715	1644	53	105	81	351	253	977	1854	2306	1380	70	30	38	olfm4
ENSXETG00000018716	4305	86	116	106	434	301	718	1175	1318	775	976	644	714	ahdc1
ENSXETG00000018718	2184	244	344	317	645	523	1135	1488	1702	1072	1108	726	821	hnrnpul1
ENSXETG00000018719	5481	143	204	166	463	392	299	380	519	288	755	546	605	ENSXETG00000018719
ENSXETG00000018720	3090	77	107	105	402	290	600	1059	1251	790	642	523	610	sema6a
ENSXETG00000018731	3007	408	555	446	370	312	887	1282	1622	849	391	319	366	commd10
ENSXETG00000018732	2601	0	0	0	0	0	0	1	0	0	0	0	0	GRM6
ENSXETG00000018734	2511	2	2	1	10	5	6	39	30	20	22	13	14	lvrn
ENSXETG00000018735	1689	338	468	395	740	589	1211	1519	1683	1091	1613	1109	1139	tcf7l2
ENSXETG00000018736	1522	367	602	499	656	551	891	1079	1247	722	613	393	467	ccdc97
ENSXETG00000018737	2079	808	1111	966	1371	1037	2240	2003	2365	1346	1761	1184	1357	map3k7
ENSXETG00000018738	1191	34	62	42	71	59	133	1657	2035	1156	136	114	109	tgfb1
ENSXETG00000018739	644	12	14	12	928	680	772	4164	4824	2585	3752	2654	2903	cdo1
ENSXETG00000018741	3049	50	71	57	381	314	241	125	176	109	55	37	39	mastl
ENSXETG00000018742	1118	3647	5308	4613	1665	1282	1774	3303	3931	2313	1173	817	886	tmed7
ENSXETG00000018744	3062	58	93	89	135	108	280	403	517	296	292	204	231	chsy1
ENSXETG00000018746	2895	1	3	1	8	5	11	83	126	60	28	24	26	epha7
ENSXETG00000018747	1854	249	303	282	444	361	708	887	1028	613	655	454	508	fem1c
ENSXETG00000018749	1203	7	14	7	80	29	66	193	211	128	25	29	38	b9d2
ENSXETG00000018751	1539	4	8	4	8	21	8	36	54	32	29	28	27	aldh1a3
ENSXETG00000018752	1638	706	1081	785	631	461	698	853	995	527	876	573	649	exosc5
ENSXETG00000018753	1515	504	699	593	936	772	1179	1458	1616	940	1059	751	864	bckdha
ENSXETG00000018756	1191	138	265	190	183	134	216	175	225	126	245	172	178	b3gnt6
ENSXETG00000018759	2134	753	1124	957	1427	1038	1459	2489	3117	1959	1370	913	1131	tmpo
ENSXETG00000018760	624	101	118	112	83	55	136	87	85	67	136	96	117	atp5sl
ENSXETG00000018761	1702	847	1332	1173	993	718	4170	4900	5553	3275	1640	1223	1273	prss16
ENSXETG00000018765	2027	107	178	126	165	160	382	384	453	300	286	207	190	asb7
ENSXETG00000018766	1974	24	20	24	49	22	65	63	85	34	74	48	57	lins1
ENSXETG00000018767	1089	18321	26787	22510	23826	18159	29253	16603	19409	11478	25321	17796	19793	slc25a3
ENSXETG00000018772	1953	5	9	6	37	31	75	229	252	180	51	44	43	nuak1
ENSXETG00000018774	2869	796	1082	913	945	729	943	1657	1817	1084	961	725	716	zdhhc6
ENSXETG00000018775	2253	0	1	0	0	0	0	1	0	0	0	0	0	
ENSXETG00000018777	1496	1322	1924	1748	2135	1654	4444	1083	1256	748	2290	1621	1763	acbd5
ENSXETG00000018778	2049	17261	25151	21848	17054	13004	11638	3197	3418	2063	5592	3911	4311	acsl5
ENSXETG00000018779	2706	1	1	1	0	2	0	916	1012	606	2	0	2	adamts17
ENSXETG00000018781	1209	2	2	1	27	24	53	85	89	57	161	100	123	avpr1a
ENSXETG00000018784	1636	1076	1556	1363	2162	1656	2468	2640	2884	1750	2690	1872	2186	abi1
ENSXETG00000018786	879	108	153	164	147	123	191	229	240	166	187	131	151	lysmd4
ENSXETG00000018788	1668	67	113	80	201	149	286	468	525	340	238	175	158	mef2a
ENSXETG00000018790	2649	13	22	14	54	36	90	2430	2679	1595	242	162	173	sh3pxd2b
ENSXETG00000018791	3457	195	274	242	792	611	2648	3080	3418	1958	4786	3342	3622	tgfb1i1
ENSXETG00000018792	2607	151	176	187	405	352	334	418	393	281	557	395	479	armc5
ENSXETG00000018793	1140	20	37	29	65	37	148	97	120	75	118	72	81	ppm1h
ENSXETG00000018796	5157	661	889	764	1386	1089	2729	2061	2447	1376	2010	1376	1560	mon2
ENSXETG00000018798	1396	767	1125	890	469	347	510	562	659	378	352	272	259	pdss1
ENSXETG00000018799	3086	0	1	1	1	3	4	1	1	1	2	0	0	npr2
ENSXETG00000018800	876	0	0	2	0	0	1	1	3	3	0	1	1	ENSXETG00000018800
ENSXETG00000018803	1462	123	202	137	194	145	274	295	380	218	235	180	201	pigp
ENSXETG00000018804	1418	181	272	230	342	240	583	637	744	392	439	291	343	lrrc28
ENSXETG00000018806	1530	60	81	72	96	76	116	127	181	105	154	107	144	dcaf17
ENSXETG00000018807	3087	51	65	61	105	69	174	592	647	393	190	137	158	apbb1ip
ENSXETG00000018809	509	22838	33223	28136	31175	23131	36898	34197	39147	22651	85029	59542	66309	rps16

ENSXETG00000018810	3158	11	10	15	40	23	92	127	179	99	178	102	134	slc25a12
ENSXETG00000018811	4659	13	13	8	81	84	287	149	213	113	508	332	360	synm
ENSXETG00000018812	1122	0	0	0	0	0	0	0	0	0	0	0	0	
ENSXETG00000018815	1825	770	1009	963	1160	832	2927	912	1071	631	1314	948	1067	rnf25
ENSXETG00000018822	3162	0	0	0	0	0	3	3	5	4	3	0	4	gpr158
ENSXETG00000018823	3891	34	49	32	135	97	157	212	279	163	115	73	99	stk36
ENSXETG00000018824	2034	0	0	0	1	0	0	0	1	1	0	0	0	
ENSXETG00000018825	3024	17	37	38	112	66	181	879	1057	554	408	255	321	cybrd1
ENSXETG00000018827	1305	7646	11116	9661	4677	3528	1233	1181	1316	856	234	188	195	XB-GENE-5897024
ENSXETG00000018831	3943	434	672	596	879	610	664	672	775	460	548	389	419	c16orf58
ENSXETG00000018832	1468	7227	10397	8518	2606	1929	2541	3732	4384	2574	1888	1382	1519	serp1
ENSXETG00000018834	2238	21	36	24	58	29	64	107	120	62	76	59	54	thnsl1
ENSXETG00000018835	2047	1	0	1	6	2	1	19	31	22	56	34	53	nrxn1
ENSXETG00000018837	1700	280	437	403	855	653	958	1506	1715	1066	1409	950	1047	fbrs
ENSXETG00000018839	1143	1977	2760	2339	2314	1753	2351	3313	3566	2224	1526	1111	1181	gorasp2
ENSXETG00000018840	6660	361	512	421	799	634	887	1658	1772	1032	1511	1106	1103	ENSXETG00000018840
ENSXETG00000018842	1097	9	9	9	228	190	37	118	131	70	12	21	18	enkur
ENSXETG00000018844	759	0	0	0	0	2	1	5	11	7	15	9	14	ENSXETG00000018844
ENSXETG00000018847	2679	163	229	192	756	628	1170	1986	2339	1365	2042	1394	1583	plk2
ENSXETG00000018848	1695	0	0	0	0	0	0	1	0	0	0	0	0	
ENSXETG00000018850	3963	0	1	2	3	5	3	3	1	2	2	1	3	myo3b
ENSXETG00000018852	3005	597	860	743	1269	918	2451	1773	2075	1208	1517	1133	1221	arhgap10
ENSXETG00000018853	687	0	1	3	3	3	3	16	10	13	3	9	4	rab3c
ENSXETG00000018854	1977	70	88	77	174	121	269	314	343	213	371	234	312	pde4d
ENSXETG00000018856	2865	206	292	232	487	412	784	451	587	347	569	410	424	ubr3
ENSXETG00000018858	2979	222	291	244	680	492	976	1050	1119	695	898	600	750	rnf40
ENSXETG00000018861	2030	1	0	0	0	1	0	0	1	0	1	0	1	klhl41
ENSXETG00000018862	2076	4	5	1	16	6	28	58	60	51	22	12	24	dclk1
ENSXETG00000018864	2731	0	0	0	4	2	2	0	0	0	0	0	0	zic2
ENSXETG00000018865	3009	0	0	0	0	0	0	1	2	0	1	0	0	pcdh8
ENSXETG00000018866	1110	25	38	30	51	40	93	84	117	52	46	23	41	ercc8
ENSXETG00000018869	3510	0	0	0	19	15	0	0	1	0	39	22	19	muc6
ENSXETG00000018872	1563	0	0	0	0	0	1	0	0	0	0	1	0	
ENSXETG00000018875	948	0	1	0	1	0	1	0	4	3	0	0	0	ccdc42
ENSXETG00000018877	2906	334	449	402	680	501	688	694	856	470	1028	694	775	trim69.2
ENSXETG00000018878	1317	0	2	0	1	0	2	6	6	8	3	4	1	cnmd
ENSXETG00000018884	1131	264	445	354	281	231	218	274	339	188	192	154	151	clybl
ENSXETG00000018885	1538	474	679	566	490	393	495	782	856	473	501	347	400	eif2b3
ENSXETG00000018887	1504	824	1229	955	976	712	1369	1204	1416	773	1391	939	1103	sugt1
ENSXETG00000018892	4272	19	34	9	233	177	473	1760	1918	1137	949	692	785	ptch2
ENSXETG00000018893	3311	786	1115	958	3383	2494	4068	3669	4339	2393	3652	2634	2927	elf1
ENSXETG00000018894	1132	113	109	96	155	124	204	228	257	176	222	176	184	mettl5
ENSXETG00000018895	717	1887	2815	2407	2319	1765	3455	1685	2025	1267	1585	1167	1197	phospho2
ENSXETG00000018896	1307	378	520	512	751	595	1295	1279	1492	829	1048	809	824	wbp4
ENSXETG00000018897	2607	78	119	91	106	67	216	188	247	138	210	167	182	naa16
ENSXETG00000018898	3045	5556	7941	6689	9346	7279	6902	5224	5781	3540	18614	13018	14779	ENSXETG00000018898
ENSXETG00000018900	1346	244	299	267	130	100	91	222	253	143	218	149	179	dctpp1
ENSXETG00000018901	909	280	407	325	291	239	306	33	41	23	23	7	7	qprt
ENSXETG00000018902	2460	0	0	1	0	0	0	1	0	0	0	0	0	ENSXETG00000018902
ENSXETG00000018904	1161	170	237	208	103	61	102	96	114	57	114	81	77	mtrf1
ENSXETG00000018905	1404	259	370	292	563	355	653	903	1008	655	591	400	419	tbc1d10b
ENSXETG00000018908	754	162	235	218	331	236	417	1061	1264	693	254	193	188	rgcc
ENSXETG00000018910	5816	823	1116	965	1327	1070	2185	1133	1379	811	733	556	586	vwa8
ENSXETG00000018912	2568	517	723	637	1148	864	1287	1420	1524	1024	1226	821	948	cd2bp2
ENSXETG00000018913	487	2897	4120	3574	1169	935	671	1704	1919	1247	1646	1094	1296	ENSXETG00000018913
ENSXETG00000018917	2986	1	0	0	4	7	2	1	2	1	2	5	1	gabbr2
ENSXETG00000018918	1901	2549	3700	3219	4587	3450	3408	1839	2129	1262	1237	801	937	pccb
ENSXETG00000018919	2583	676	987	849	1138	876	1213	7389	8900	5004	1664	1258	1311	cnn3
ENSXETG00000018920	3048	459	643	504	361	273	314	564	695	432	314	243	284	elp2
ENSXETG00000018921	390	183	258	304	289	248	279	454	451	308	259	190	215	isy1
ENSXETG00000018922	789	18	37	24	135	110	100	75	119	64	124	117	102	tmem56
ENSXETG00000018924	1117	1131	1664	1302	848	575	722	828	961	528	620	440	481	hccs
ENSXETG00000018926	1620	51	81	64	181	149	245	452	591	307	325	261	241	ptbp2
ENSXETG00000018927	3480	33	67	62	687	530	564	1034	1209	702	565	422	459	ppp2r3a
ENSXETG00000018928	1659	17	29	16	276	211	58	49	59	43	56	52	49	ccdc173
ENSXETG00000018929	2810	470	681	605	169	124	119	324	342	210	222	146	172	fastkd1
ENSXETG00000018933	4464	0	0	0	0	0	0	0	0	0	0	0	0	
ENSXETG00000018934	3380	224	351	298	493	408	868	597	713	404	935	613	823	ctnnal1
ENSXETG00000018935	1566	43	66	64	162	152	198	320	389	220	170	118	141	bbs5
ENSXETG00000018943	2850	1	1	0	3	2	5	24	30	19	6	7	5	ENSXETG00000018943
ENSXETG00000018945	5757	1	1	2	1	2	1	3	2	7	10	4	6	pcdh15
ENSXETG00000018946	909	0	0	0	0	0	0	0	0	0	0	0	0	
ENSXETG00000018949	912	0	0	0	0	0	0	0	0	0	0	0	0	
ENSXETG00000018951	948	0	0	0	0	0	0	0	0	0	0	0	0	
ENSXETG00000018952	957	0	0	0	0	0	0	0	0	0	0	0	0	
ENSXETG00000018953	402	1	0	0	1	0	0	0	0	0	0	0	0	
ENSXETG00000018954	2165	6	11	10	19	12	8	239	282	157	13	18	13	scnn1a
ENSXETG00000018955	1413	4	2	5	9	6	47	28	43	13	15	11	6	nt5dc4
ENSXETG00000018957	3301	1573	2177	1870	2643	2016	6032	4943	5574	3287	8891	6205	6952	slc20a1
ENSXETG00000018958	1215	57	89	68	130	135	170	525	582	333	203	136	153	ENSXETG00000018958
ENSXETG00000018959	1854	421	587	468	494	357	843	1496	1647	1095	860	569	702	lpar5
ENSXETG00000018960	6219	42	43	50	5007	3877	148	12	10	7	5643	3937	4486	ENSXETG00000018960
ENSXETG00000018961	3310	234	341	275	633	530	745	1540	1736	1038	924	661	665	ENSXETG00000018961
ENSXETG00000018962	2289	176	223	239	356	275	598	500	569	362	544	396	418	miga2
ENSXETG00000018963	618	0	0	0	0	0	1	8	7	3	9	3	4	slc15a5
ENSXETG00000018964	823	144	240	167	174	132	184	295	275	179	189	126	142	dolpp1
ENSXETG00000018965	2239	2220	3278	2863	1835	1488	3137	1272	1427	876	1692	1264	1322	crat.1
ENSXETG00000018967	802	1054	1453	1219	863	657	938	1092	1231	760	873	636	745	mrps24
ENSXETG00000018969	2428	0	0	0	0	0	0	0	0	0	0	0	0	
ENSXETG00000018970	1023	460	686	571	609	455	738	990	1096	660	601	471	514	ptpa
ENSXETG00000018971	3002	6	2	4	57	48	67	104	120	71	155	90	118	camk2b
ENSXETG00000018972	579	202	284	209	351	315	713	554	640	367	322	223	217	nudcd3
ENSXETG00000018980	1335	3	5	4	2	1	2	2	6	2	3	1	1	pgam2
ENSXETG00000018981	862	45697	66144	55409	63654	48270	85609	78647	91957	55013	145318	103154	112656	ppia
ENSXETG00000018984	1356	122	161	106	136	108	207	392	403	269	371	296	261	tph1
ENSXETG00000018990	1620	143	251	201	320	275	279	515	548	375	289	184	229	saal1
ENSXETG00000018991	3291	768	1222	970	2280	1725	3207	3453	3778	2191	3773	2699	2816	zmiz2
ENSXETG00000018992	2665	1025	1459	1299	1683	1277	1693	3033	3344	2177	1241	971	980	gtf2h1
ENSXETG00000018997	4535	4888	7401	6348	8031	6029	6224	5745	6387	3922	5194	3545	4009	ogdh
ENSXETG00000018999	2099	3469	4985	4127	3800	2871	5031	3954	4606	2646	4036	2780	2961	tmed4
ENSXETG00000019000	3127	721	1089	852	773	537	723	1437	1636	991	883	646	689	ddx56
ENSXETG00000019001	3879	12393	18131	15618	4077	3103	4534	576	591	407	203	168	171	npc1l1
ENSXETG00000019002	600	742	1110	947	899	742	893	1181	1363	837	631	475	575	ykt6
ENSXETG00000019003	2427	1279	1668	1454	2638	2163	2437	542	586	325	845	578	700	gck
ENSXETG00000019004	565	4	7	5	14	18	7	1	1	1	5	3	1	myl7
ENSXETG00000019005	1560	3	2	4	905	678	50	6	14	7	14	13	12	ENSXETG00000019005
ENSXETG00000019007	1329	68	102	89	170	145	66	377	457	301	153	125	125	pold2
ENSXETG00000019012	2653	135	204	112	211	176	440	426	527	265	280	192	229	elmod3
ENSXETG00000019013	2452	3417	4731	4082	7917	6041	6585	8484	9546	5628	7161	4971	5475	dbnl
ENSXETG00000019014	444	48	65	53	41	34	70	70	81	66	30	26	41	ube2d4
ENSXETG00000019015	2928	275	425	360	810	596	743	1238	1490	890	1135	846	884	tcf7l1

ENSXETG00000019016	1512	542	820	693	487	390	1288	1087	1135	656	1057	714	823	XB-GENE-5812048
ENSXETG00000019019	827	34	31	24	59	61	70	112	125	70	118	82	125	ENSXETG00000019019
ENSXETG00000019020	1395	234	338	265	317	259	377	441	572	339	351	242	271	btn1a1
ENSXETG00000019022	3498	0	0	0	1	0	1	1	3	2	0	0	0	
ENSXETG00000019024	475	23707	35728	31638	41773	31697	40318	36592	41840	26192	75227	53125	57004	rplp2
ENSXETG00000019025	1642	0	1	2	0	0	0	3	0	0	0	0	0	chst4
ENSXETG00000019026	2028	1226	1802	1621	3379	2545	3193	1966	2350	1405	2254	1496	1759	mgat1
ENSXETG00000019027	2510	242	374	300	1255	999	2897	1096	1264	778	2289	1651	1846	pnpla2
ENSXETG00000019028	1790	9	25	11	47	41	150	342	422	252	117	80	118	XB-GENE-5778936
ENSXETG00000019030	672	3	6	6	34	32	130	491	481	314	45	38	41	loxl3
ENSXETG00000019031	918	0	0	0	0	0	0	0	0	0	0	0	0	
ENSXETG00000019032	3034	27	30	30	50	42	20	89	99	56	16	10	20	exo1
ENSXETG00000019033	1677	233	350	346	1110	862	963	428	493	285	761	519	617	cracr2b
ENSXETG00000019035	2889	1089	1611	1345	2946	2226	4436	5370	6361	3809	4115	2920	3113	cd151
ENSXETG00000019036	1074	0	0	0	0	0	0	0	0	0	1	0	0	
ENSXETG00000019038	1806	376	550	394	379	281	848	705	867	459	554	407	431	trappc6a
ENSXETG00000019042	952	32	62	64	85	66	34	187	212	139	50	42	24	dctd
ENSXETG00000019047	2429	657	949	766	1388	1038	1569	2572	2844	1786	1449	976	1103	dok1
ENSXETG00000019049	1723	241	343	316	429	328	470	606	728	384	374	300	288	cdkn2aip
ENSXETG00000019051	1291	97	144	106	293	231	2452	1663	1776	1144	833	615	698	klf9
ENSXETG00000019052	1136	205	268	241	444	351	683	572	708	470	952	643	690	ing2
ENSXETG00000019055	624	3	5	7	7	11	18	11	12	7	28	34	25	TSPAN4
ENSXETG00000019058	5790	3191	4750	4124	10142	8033	12382	5466	6278	3805	7795	5510	6284	myh14
ENSXETG00000019065	711	259	368	309	234	161	343	409	533	286	243	178	210	rwdd4
ENSXETG00000019069	5198	639	939	774	936	677	1747	1518	1823	1045	1059	819	883	trappc11
ENSXETG00000019070	1589	507	826	672	657	506	647	712	765	487	614	473	525	tbcb
ENSXETG00000019071	1840	540	777	635	711	520	726	982	1185	706	1054	700	830	chid1
ENSXETG00000019073	2228	162	258	215	313	261	428	565	641	409	628	453	472	clasrp
ENSXETG00000019075	423	0	0	1	1	1	1	3	7	5	11	5	14	ENSXETG00000019075
ENSXETG00000019079	1340	91	130	117	491	401	596	4635	5199	2995	1421	1002	1139	lgals9b
ENSXETG00000019084	1674	257	392	388	493	390	699	964	1065	644	772	570	624	ppp1r37
ENSXETG00000019086	1485	30	55	33	95	63	143	577	557	402	195	134	131	tcf4
ENSXETG00000019096	2249	48	72	51	592	450	121	262	283	180	83	56	63	rsph6a
ENSXETG00000019099	2300	498	719	539	657	490	946	569	731	404	961	677	732	mypop
ENSXETG00000019101	2929	5	3	10	46	35	117	184	217	118	78	53	71	nova2
ENSXETG00000019104	1851	152	185	154	310	228	533	1156	1227	707	419	295	340	mark4
ENSXETG00000019108	1510	3	5	5	4	4	4	54	48	43	1	1	3	ckm
ENSXETG00000019113	2693	958	1426	1123	1216	982	2328	1475	1852	1033	1046	813	832	ube2j1
ENSXETG00000019114	1218	428	630	504	2415	1813	2600	2196	2472	1489	1937	1334	1468	tfe3
ENSXETG00000019115	1382	0	0	0	11	3	12	18	13	5	67	53	43	rragd
ENSXETG00000019116	1797	6	20	25	21	17	18	25	24	15	32	20	26	ENSXETG00000019116
ENSXETG00000019117	2151	19	27	25	33	21	32	34	39	24	48	40	42	ankrd6
ENSXETG00000019118	3805	67	125	88	157	125	105	232	295	157	317	248	241	mdn1
ENSXETG00000019121	4514	158	216	184	277	200	355	454	565	269	468	350	372	casp8ap2
ENSXETG00000019123	3447	2717	3848	3241	4150	3214	5217	2928	3346	2070	4404	3290	3583	epn2
ENSXETG00000019125	840	0	0	0	1	0	0	0	0	0	0	0	0	
ENSXETG00000019126	2175	3	6	7	12	20	40	104	109	76	54	52	58	bach2
ENSXETG00000019128	2186	6813	9717	8429	11579	8971	11179	15574	17864	10715	11838	8524	9038	u2af2
ENSXETG00000019136	1947	1172	1666	1421	1285	987	2854	2794	3258	1957	1876	1325	1455	ext1
ENSXETG00000019138	3369	37	72	57	127	103	232	264	333	193	284	202	258	znf865
ENSXETG00000019143	912	0	0	0	1	0	0	0	0	0	0	0	0	
ENSXETG00000019145	1584	45	75	76	182	171	513	104	114	78	239	147	162	ENSXETG00000019145
ENSXETG00000019146	2037	150	230	171	573	426	892	682	819	508	790	588	627	kiaa1161
ENSXETG00000019150	4569	1	1	1	7	4	9	26	24	17	46	27	22	adgrb1
ENSXETG00000019153	1065	0	0	0	0	1	0	5	6	3	17	14	10	
ENSXETG00000019157	2582	1	0	0	3	3	4	0	1	0	1	3	3	XB-GENE-5767170
ENSXETG00000019159	2136	0	0	0	0	0	1	0	3	0	0	0	0	
ENSXETG00000019162	339	0	0	0	0	0	0	0	0	0	0	0	0	
ENSXETG00000019163	2379	68	104	92	527	396	1588	1404	1576	970	2015	1452	1649	itgb3
ENSXETG00000019164	1963	0	0	0	0	0	0	1	2	5	16	12	9	
ENSXETG00000019166	1203	5081	7203	6175	7439	5811	7752	17537	20030	11553	4688	3368	3685	hmgb1
ENSXETG00000019167	3030	797	1159	961	1122	786	857	912	1064	591	1510	1004	1134	uspl1
ENSXETG00000019168	1829	124	162	137	459	368	482	16	20	10	64	43	54	tex26
ENSXETG00000019170	816	0	0	0	0	0	0	0	0	0	0	0	0	
ENSXETG00000019171	828	28	24	17	14	7	27	44	34	30	36	25	21	cntd1
ENSXETG00000019172	1326	3	4	2	33	32	8	2	0	2	7	5	7	ENSXETG00000019172
ENSXETG00000019175	3244	829	1157	1052	418	352	252	1654	2002	1168	157	125	145	hsph1
ENSXETG00000019176	3636	1826	2567	2227	2258	1847	3801	3331	3920	2301	3533	2484	2785	yes1
ENSXETG00000019179	2244	0	2	1	9	4	10	20	24	11	12	24	25	adcyap1
ENSXETG00000019180	675	0	0	0	0	0	0	0	0	0	4	1	1	
ENSXETG00000019184	1680	51	75	78	392	294	429	668	794	447	490	342	420	b3glct
ENSXETG00000019186	2208	2	3	0	1	0	1	0	0	1	0	1	0	
ENSXETG00000019195	4173	78	128	88	121	68	260	1072	1196	709	305	185	232	arhgap30
ENSXETG00000019214	2962	227	339	284	323	239	382	791	1017	615	271	207	200	smarcad1
ENSXETG00000019215	795	20524	29474	24868	22133	16768	22962	7692	9344	5314	5068	3615	3951	hpgds
ENSXETG00000019216	1656	0	0	0	0	0	0	0	0	0	1	0	0	ENSXETG00000019216
ENSXETG00000019217	1799	245	394	319	716	529	906	568	669	412	555	394	469	pdlim5
ENSXETG00000019220	2475	4	6	6	53	36	20	83	93	71	193	113	138	bmpr1b
ENSXETG00000019221	2763	6	6	4	19	6	29	90	109	55	18	11	14	unc5c
ENSXETG00000019224	603	718	1144	958	963	716	1088	524	587	355	815	663	607	ADTRP
ENSXETG00000019230	2301	173	227	222	278	207	338	623	723	442	401	276	311	thap12
ENSXETG00000019232	2765	9	12	17	33	39	79	135	138	85	48	30	36	nek8
ENSXETG00000019241	1153	0	0	0	0	0	0	0	0	0	3	3	5	
ENSXETG00000019242	1641	1789	2559	2162	2210	1692	2526	2503	2762	1718	1253	922	953	gmppa
ENSXETG00000019244	1667	1819	2498	2123	3318	2486	7347	6929	8128	4839	4197	2974	3446	chmp2a
ENSXETG00000019245	2211	255	342	337	640	437	525	1426	1513	914	740	519	557	trim28
ENSXETG00000019246	1394	5	7	5	1379	1035	54	6	5	4	36	20	26	ENSXETG00000019246
ENSXETG00000019248	2193	160	213	167	259	229	401	560	645	375	452	344	346	thap7
ENSXETG00000019251	1353	0	0	1	6	1	1	0	0	0	0	0	1	tuba1a
ENSXETG00000019252	1736	30	46	29	68	61	101	115	130	79	80	54	50	fam222b
ENSXETG00000019254	1353	2	0	3	374	305	19	9	7	5	17	11	13	ENSXETG00000019254
ENSXETG00000019257	2346	5	6	4	51	31	67	413	526	274	60	50	40	rab34
ENSXETG00000019259	1147	651	917	830	535	364	379	691	877	516	359	272	308	timm22
ENSXETG00000019260	1158	0	0	1	0	0	0	0	1	0	1	1	1	
ENSXETG00000019261	4146	0	0	2	8	3	20	270	302	170	56	67	47	sobp
ENSXETG00000019263	2434	416	618	453	278	230	400	477	556	355	295	205	251	pdss2
ENSXETG00000019265	6235	981	1463	1234	2449	1950	3711	3404	3830	2262	4077	2722	3096	supt6h
ENSXETG00000019266	2238	26	42	39	55	44	94	202	225	111	104	72	82	bend3
ENSXETG00000019267	1063	175	217	179	245	184	217	261	313	155	201	150	153	c6orf203
ENSXETG00000019268	2991	389	463	429	1195	922	2174	1591	1956	1097	1943	1375	1652	aim1
ENSXETG00000019269	1116	637	899	776	559	454	660	626	695	425	335	214	232	rtn4ip1
ENSXETG00000019275	1377	3033	4527	3891	5602	4376	24481	13818	15306	9612	10783	7445	8156	des.1
ENSXETG00000019276	1555	2461	3607	2955	3620	2830	4081	1399	1625	1069	3211	2262	2446	dnpep
ENSXETG00000019277	6699	513	657	568	1123	885	2370	1118	1293	811	1303	919	1111	KIAA0100
ENSXETG00000019281	753	3	1	2	11	7	2	0	1	0	3	2	1	ENSXETG00000019281
ENSXETG00000019283	3531	19	34	35	150	96	91	253	222	148	249	208	186	ptprn
ENSXETG00000019286	1835	329	475	408	189	137	467	815	957	607	352	262	277	dnajb2
ENSXETG00000019288	1747	6894	10263	8713	10294	7557	8101	8228	9207	5643	7433	5339	5623	tuba4b
ENSXETG00000019290	1440	189	230	233	234	155	398	525	560	369	328	235	245	stk16
ENSXETG00000019291	3385	19	24	24	65	48	171	1056	1206	649	243	147	173	glb1
ENSXETG00000019293	1821	265	413	324	481	412	408	410	452	291	438	314	314	ankzf1

ENSXETG00000019294	2517	451	645	556	1493	1168	2141	1962	2302	1405	1975	1361	1429	atg9a
ENSXETG00000019297	2685	0	0	1	2	0	0	4	2	2	0	0	0	
ENSXETG00000019301	939	0	0	0	0	0	0	0	0	0	0	0	0	
ENSXETG00000019305	1249	4	4	6	1	0	1	16	24	10	12	6	8	klk15
ENSXETG00000019306	5496	62761	93398	80595	183550	139773	20177	64	108	28	329	216	260	lct.2
ENSXETG00000019308	756	0	0	0	0	0	0	1	0	0	0	0	1	upk1a
ENSXETG00000019309	1025	0	0	0	0	0	1	0	0	0	1	1	0	prss1.2
ENSXETG00000019317	880	137723	202313	177209	78992	60275	81765	14705	16687	9337	23011	16241	17770	apoe
ENSXETG00000019319	849	1153	1770	1519	1224	926	547	1614	1758	1140	544	379	429	tomm40
ENSXETG00000019324	4835	236	344	302	1550	1092	625	3779	4176	2564	634	465	466	bcam
ENSXETG00000019330	2925	194	260	223	516	467	667	1123	1178	738	934	684	760	r3hdm1
ENSXETG00000019334	939	0	0	1	0	0	0	1	2	2	0	1	0	ENSXETG00000019334
ENSXETG00000019336	1233	24	40	22	54	59	105	80	85	63	150	106	99	ENSXETG00000019336
ENSXETG00000019347	2705	356	517	382	504	411	848	745	862	506	349	275	299	pigs
ENSXETG00000019348	771	34	42	48	129	96	138	246	288	163	241	156	211	unc119
ENSXETG00000019349	4449	103	142	129	676	543	634	1297	1514	869	382	262	286	ltbp1
ENSXETG00000019350	7143	524	715	575	1204	920	1367	1210	1352	829	1416	943	1103	heatr5b
ENSXETG00000019351	1662	0	0	0	0	1	0	0	2	0	0	0	2	foxn1
ENSXETG00000019353	1302	6	12	9	48	41	68	178	204	108	213	187	192	cadm3
ENSXETG00000019354	2091	2	3	1	18	13	44	113	121	72	31	20	25	rasgrp3
ENSXETG00000019357	1500	680	972	843	653	509	1056	879	1082	662	784	523	591	FAM98A
ENSXETG00000019359	1845	0	0	0	0	0	0	0	0	0	0	0	0	ENSXETG00000019359
ENSXETG00000019362	1606	7	20	14	24	13	9	51	66	40	29	17	28	XB-GENE-5742361
ENSXETG00000019368	918	0	0	0	0	0	0	0	0	0	0	0	0	
ENSXETG00000019369	1610	111	170	176	242	179	198	291	343	199	78	62	56	XB-GENE-5914997
ENSXETG00000019371	4935	244	311	297	541	415	717	953	1090	675	794	566	671	fbxo38
ENSXETG00000019373	1047	7	6	2	20	14	31	76	87	53	50	44	32	htr4
ENSXETG00000019374	615	715	978	910	739	573	880	1432	1782	924	688	458	530	txndc9
ENSXETG00000019375	3759	144	205	190	222	131	368	1143	1489	781	379	259	252	rev1
ENSXETG00000019377	3627	1064	1621	1475	1963	1566	1912	3315	3839	2242	2586	1787	1949	eif5b
ENSXETG00000019379	1215	0	0	0	0	0	0	1	0	0	0	0	0	
ENSXETG00000019380	2585	3899	5666	4742	7086	5338	16686	628	705	477	10971	7613	8945	meltf
ENSXETG00000019383	2436	14	15	16	44	30	183	536	671	370	78	65	93	lyg2
ENSXETG00000019384	700	691	1017	827	822	607	749	1550	1681	1092	574	404	442	ncbp2
ENSXETG00000019385	1173	1	1	2	0	0	0	163	189	122	0	0	0	ahsg
ENSXETG00000019386	1326	41	52	52	65	49	118	114	116	74	90	70	70	mitd1
ENSXETG00000019389	1480	12	8	9	4	5	25	78	80	39	0	3	1	hrg
ENSXETG00000019390	1957	1311	1910	1638	1379	992	1724	2275	2731	1595	3354	2332	2654	eif4a2
ENSXETG00000019391	1257	195	299	237	604	465	313	818	927	516	462	317	360	rfc4
ENSXETG00000019392	3330	3	3	9	9	14	7	50	63	40	37	28	25	mcf2l2
ENSXETG00000019393	1119	99	137	111	83	60	177	97	132	69	164	89	104	lipt1
ENSXETG00000019399	3879	424	606	553	642	501	449	594	637	393	646	428	504	cluh
ENSXETG00000019401	2644	44	48	57	231	176	182	342	447	249	539	384	440	mgat4b
ENSXETG00000019402	1491	1	1	0	6	8	15	124	148	78	104	90	78	ENSXETG00000019402
ENSXETG00000019403	724	654	982	833	661	521	620	1119	1178	720	562	385	403	mrpl22
ENSXETG00000019404	4467	444	589	514	589	487	463	994	1070	672	509	385	390	gemin5
ENSXETG00000019406	3636	0	0	2	0	0	18	73	65	29	15	8	16	ENSXETG00000019406
ENSXETG00000019407	4144	106	165	130	281	181	513	934	1139	593	404	317	345	inpp4a
ENSXETG00000019408	2198	23	28	19	59	48	87	206	248	139	326	251	219	wnt11
ENSXETG00000019409	2246	106	164	154	647	513	1083	2569	2862	1791	1329	861	1047	ccdc69
ENSXETG00000019410	5646	1878	2717	2289	3478	2689	8019	5433	6360	3600	5894	4341	4782	pafah1b1
ENSXETG00000019412	3143	514	789	662	1375	1111	1383	2792	3084	1998	1575	1117	1149	pip5k1a
ENSXETG00000019414	3468	0	0	0	0	0	0	2	0	0	3	1	0	
ENSXETG00000019415	2960	480	691	644	294	253	601	842	1015	602	702	477	500	top1.1
ENSXETG00000019416	1235	246	358	317	268	190	376	217	251	131	99	66	72	XB-GENE-5727515
ENSXETG00000019419	1909	2079	2921	2279	1657	1231	6879	3168	3749	2118	3404	2474	2710	gm2a
ENSXETG00000019422	5575	98	109	83	314	272	401	665	750	460	453	320	382	emsy
ENSXETG00000019423	3822	264	361	354	670	458	829	2371	2544	1640	1070	794	857	plcg1
ENSXETG00000019424	1197	380	599	507	400	297	1945	1119	1226	736	696	489	565	slc36a1
ENSXETG00000019426	282	402	597	525	251	201	366	417	497	337	349	238	277	dpm3
ENSXETG00000019427	606	317	488	432	1001	749	1261	1191	1262	810	1420	1019	1130	slc50a1
ENSXETG00000019428	504	681	1039	830	355	252	593	546	668	379	964	647	701	efna1
ENSXETG00000019429	909	213	290	240	366	315	364	498	552	325	404	330	350	znhit1
ENSXETG00000019430	1896	4	3	1	36	18	72	493	627	409	123	88	109	lrrc32.1
ENSXETG00000019432	2241	364	556	430	420	282	550	671	820	495	485	329	360	mettl16
ENSXETG00000019434	2581	115	170	141	362	293	695	699	770	453	788	566	660	mnt
ENSXETG00000019435	2242	508	755	668	470	356	430	656	742	423	754	522	637	ttc14
ENSXETG00000019436	2904	1	5	0	8	1	2	7	7	6	14	9	18	sgsm2
ENSXETG00000019437	3116	0	0	1	95	72	12	20	29	21	6	9	6	ccdc39
ENSXETG00000019440	1295	185	271	256	258	187	277	399	478	297	351	259	278	wdr74
ENSXETG00000019441	2879	671	970	833	1689	1312	2844	3302	4010	2254	2918	2087	2346	fxr1
ENSXETG00000019442	3478	425	627	483	1115	871	1529	1916	2295	1315	1681	1106	1286	nxf1
ENSXETG00000019443	2260	221	328	293	305	243	607	711	877	561	458	343	348	tnfaip1
ENSXETG00000019444	474	251	401	288	379	275	406	651	772	445	239	162	190	ift20
ENSXETG00000019447	3026	845	1212	1020	244	193	638	228	259	147	306	210	279	ENSXETG00000019447
ENSXETG00000019449	2578	471	732	640	1634	1285	1709	1234	1351	901	972	703	783	tsku
ENSXETG00000019451	603	5	7	8	12	3	21	51	51	42	39	29	25	ENSXETG00000019451
ENSXETG00000019452	1602	0	0	0	2	0	2	7	9	3	8	13	8	or6f1
ENSXETG00000019453	1768	1583	2236	1858	737	584	777	1374	1869	948	504	353	375	stx5
ENSXETG00000019454	1009	693	957	873	1031	831	1734	1107	1314	787	1109	827	870	acer3
ENSXETG00000019456	2149	23777	35019	30550	17514	13419	52851	14582	16658	10067	26090	18318	20350	slc3a2
ENSXETG00000019457	3552	178	248	216	577	434	923	989	1146	679	1141	819	928	zhx3
ENSXETG00000019458	2295	234	381	303	467	360	555	480	543	334	646	469	501	rps6ka4
ENSXETG00000019460	1193	1603	2255	1886	819	643	911	884	981	586	658	440	437	c20orf24
ENSXETG00000019461	1983	9	18	17	40	23	54	48	63	40	76	62	78	ggt7
ENSXETG00000019465	1167	0	0	0	0	0	0	0	0	0	0	0	0	
ENSXETG00000019466	5055	78	138	90	275	212	447	617	651	374	591	394	447	ncoa6
ENSXETG00000019469	960	0	0	0	0	0	0	0	0	0	0	0	0	
ENSXETG00000019470	5010	70	104	75	416	299	638	848	1003	638	831	550	593	tnrc6c
ENSXETG00000019471	660	121	182	169	116	79	392	258	285	192	56	54	48	tp53inp2
ENSXETG00000019472	2914	345	472	381	431	323	538	521	669	382	477	364	394	pigu
ENSXETG00000019473	1456	1048	1481	1221	4196	3160	8821	5483	6293	3657	6474	4489	5064	map1lc3a
ENSXETG00000019474	1051	1106	1565	1319	1124	888	1578	1821	2187	1191	1046	761	798	dynlrb1
ENSXETG00000019475	4933	1551	2312	2018	2900	2247	3828	2644	3123	1868	2890	2060	2221	itch
ENSXETG00000019478	2800	0	0	0	0	0	1	0	1	0	1	0	1	ENSXETG00000019478
ENSXETG00000019480	1908	3	2	3	29	21	25	0	0	1	16	6	9	ENSXETG00000019480
ENSXETG00000019483	630	0	2	1	2	1	5	5	5	6	13	3	3	XB-GENE-5831307
ENSXETG00000019484	1674	752	1038	899	734	569	525	2211	2647	1599	381	255	321	ppid
ENSXETG00000019485	2699	1653	2446	2099	4370	3340	3927	2633	2952	1784	2134	1532	1668	sec14l5
ENSXETG00000019486	2089	44790	65439	56493	43890	33772	47593	10767	12035	7400	28129	20014	22018	ahcy
ENSXETG00000019487	3555	0	0	0	0	0	0	0	0	0	0	0	0	
ENSXETG00000019488	2358	57	95	94	89	84	31	39	35	18	35	36	32	mgat5b
ENSXETG00000019489	1723	2262	3209	2824	2821	2162	2218	1521	1733	1012	812	580	599	etfdh
ENSXETG00000019492	1700	629	904	868	998	759	2635	1766	2050	1221	1009	697	806	mfsd11
ENSXETG00000019493	1863	0	2	1	0	2	1	1	1	2	0	2	2	rxfp1
ENSXETG00000019495	825	6290	9121	8020	5825	4388	7162	4801	5766	3462	3453	2477	2797	uqcrfs1
ENSXETG00000019496	2567	0	0	1	1	0	4	1	3	0	11	1	8	ENSXETG00000019496
ENSXETG00000019498	1527	12	13	8	148	106	73	221	277	159	131	89	103	fam198b
ENSXETG00000019499	705	25	32	37	15	22	47	54	59	39	103	88	77	mettl23
ENSXETG00000019501	2342	1262	1896	1571	644	515	978	1672	2083	1151	1402	975	1108	jmjd6
ENSXETG00000019503	1374	246	371	346	620	508	1065	3549	3791	2380	1706	1207	1310	mxra7

ENSXETG00000019504	2664	0	0	0	0	0	2	4	0	0	0	2	0	
ENSXETG00000019505	1120	0	1	2	4	4	2	8	10	3	3	5	6	ENSXETG00000019505
ENSXETG00000019506	1246	188	306	250	383	338	273	361	410	253	188	148	147	st6galnac2
ENSXETG00000019507	1459	4923	7179	6123	9321	7433	13484	13416	15378	9129	26817	18683	20942	eif2s2
ENSXETG00000019508	2232	366	532	441	536	428	967	503	579	345	977	695	735	mfsd6l
ENSXETG00000019510	399	251	347	250	1055	809	839	236	249	164	154	113	115	cygb
ENSXETG00000019512	2961	68	93	69	443	349	262	752	830	523	839	540	596	pik3r6
ENSXETG00000019514	867	0	0	0	0	0	0	5	6	3	3	2	6	raly
ENSXETG00000019515	3984	86	125	116	221	174	409	2701	3103	1900	628	457	523	pik3r5
ENSXETG00000019519	1821	3	2	7	8	9	11	9	10	7	23	14	16	ntn1
ENSXETG00000019522	6588	0	0	0	0	0	0	1	0	0	1	2	0	ENSXETG00000019522
ENSXETG00000019523	4688	285	429	408	657	501	1923	1617	1853	1100	920	668	730	rhbdf2
ENSXETG00000019524	4420	0	0	0	0	0	0	0	0	0	0	0	0	
ENSXETG00000019530	1953	0	0	0	0	0	0	0	0	0	0	0	0	
ENSXETG00000019532	684	809	1146	960	676	577	1403	1545	1623	1027	891	591	690	stx8
ENSXETG00000019533	2412	61	77	69	108	91	139	92	107	56	166	112	139	glrb
ENSXETG00000019534	606	0	0	0	0	0	0	1	0	1	0	0	0	
ENSXETG00000019537	1653	1	2	0	13	10	15	22	20	8	30	28	17	tbc1d24.2
ENSXETG00000019539	2142	4	7	3	478	365	26	8	3	5	24	19	7	cfap52
ENSXETG00000019540	2186	965	1495	1271	1394	1049	1717	563	638	350	756	533	609	sphk1
ENSXETG00000019542	2360	331	425	359	1053	738	1034	2432	2641	1649	721	519	542	prpsap1
ENSXETG00000019547	2187	5	5	5	20	20	40	57	84	45	38	37	32	arhgap44
ENSXETG00000019548	2025	579	811	702	1272	1080	1701	1245	1435	931	1657	1063	1222	rnf157
ENSXETG00000019549	2589	2	1	1	131	100	12	53	73	40	12	1	4	foxj1
ENSXETG00000019550	732	18	36	38	71	54	100	247	304	154	157	105	127	pdgfc
ENSXETG00000019551	2624	399	593	495	1048	769	1090	1330	1414	845	2037	1405	1495	exoc7
ENSXETG00000019559	1737	1115	1520	1351	3478	2659	1792	937	1036	640	1457	1123	1194	ENSXETG00000019559
ENSXETG00000019566	468	0	0	0	0	0	0	5	7	3	4	2	7	
ENSXETG00000019567	1647	1	0	2	10	8	20	104	146	82	122	95	90	eef1a2
ENSXETG00000019568	894	13	28	26	56	42	76	80	138	59	167	109	154	spry4
ENSXETG00000019569	2527	3049	4237	3565	3985	2969	7040	5415	6288	3955	4583	3197	3572	ndfip1
ENSXETG00000019575	1047	1717	2546	2244	1766	1336	2048	1665	1801	1128	2007	1460	1453	tvp23b
ENSXETG00000019577	1260	10	13	7	222	180	17	28	16	22	16	9	10	tekt3
ENSXETG00000019578	2811	3112	4710	3999	3113	2297	2247	1082	1323	797	2394	1649	1911	vav2
ENSXETG00000019579	1524	179	280	215	1144	832	521	3952	4182	2656	740	524	512	pmp22
ENSXETG00000019581	1185	0	0	1	17	13	31	390	451	270	54	38	48	hs3st3a1
ENSXETG00000019588	1558	790	1145	1026	1144	935	1587	1912	2121	1361	1890	1383	1518	wdr5
ENSXETG00000019589	2615	6	21	15	16	16	25	55	46	36	23	9	10	brd3
ENSXETG00000019594	953	24	37	49	76	62	45	11	11	9	35	29	30	ENSXETG00000019594
ENSXETG00000019595	2094	465	649	632	874	608	1324	884	1046	629	1514	1036	1210	psmd5
ENSXETG00000019597	1575	31	30	32	84	59	25	76	80	44	32	16	20	phf19
ENSXETG00000019599	1200	9	7	10	12	7	13	95	93	65	13	12	21	TRAF1
ENSXETG00000019600	4506	74	72	92	167	156	108	127	127	77	150	108	107	c5
ENSXETG00000019602	1398	15	16	19	45	32	97	217	240	174	52	45	54	slc2a8
ENSXETG00000019603	1718	5426	7951	6450	5245	3904	9305	6140	7426	3954	5331	3799	4096	tmem52
ENSXETG00000019604	1435	1530	2326	1950	3649	2854	4931	5801	6499	3865	4157	2888	3303	gnb1
ENSXETG00000019605	1775	884	1279	1122	1179	956	2890	1148	1243	743	499	431	461	nadk
ENSXETG00000019606	2573	533	716	616	1129	817	1478	2044	2338	1426	1475	1020	1062	cdk11b
ENSXETG00000019607	900	5	5	7	6	2	12	125	153	97	19	13	17	mmp23b
ENSXETG00000019608	3752	133	233	185	166	137	356	203	280	159	306	238	227	mib2
ENSXETG00000019609	2005	1479	1941	1502	2019	1600	5446	259	368	193	1413	1001	1101	XB-GENE-5831291
ENSXETG00000019610	2568	288	409	347	607	424	975	889	1125	647	913	685	704	fbxw2
ENSXETG00000019611	2769	4	16	22	96	47	229	1215	1241	898	658	508	576	garnl3
ENSXETG00000019612	2409	127	204	178	979	692	1211	4908	5293	3274	1849	1311	1453	entpd2
ENSXETG00000019613	1335	102	147	133	240	176	394	1061	1233	725	320	224	213	plod3
ENSXETG00000019615	2683	15568	21335	17988	25333	19543	19222	4603	5399	3005	8370	5774	6622	aldh1a1
ENSXETG00000019616	2136	0	0	0	0	0	1	0	2	0	0	1	0	tmc2
ENSXETG00000019618	3681	0	0	3	6	2	6	74	75	46	6	4	4	nptx1
ENSXETG00000019623	543	55	75	56	87	41	89	129	145	74	116	82	87	endov
ENSXETG00000019624	1641	131	176	142	204	145	558	810	954	591	218	152	167	slc26a11
ENSXETG00000019626	2601	1	2	1	5	7	15	11	9	5	124	78	84	pah
ENSXETG00000019627	1348	77	108	103	66	40	105	121	182	69	75	60	48	lysmd3
ENSXETG00000019628	18480	28	44	34	100	86	185	110	117	67	263	176	212	adgrv1
ENSXETG00000019629	3921	15940	22691	19510	22241	16898	59674	12331	14717	8604	22249	15219	17492	cdhr2
ENSXETG00000019630	3894	0	5	3	11	4	46	46	61	48	52	49	29	aff2
ENSXETG00000019631	759	0	0	0	1	0	0	0	1	0	0	0	0	
ENSXETG00000019632	3846	41	44	41	126	101	271	708	804	481	251	202	221	tspan17
ENSXETG00000019633	1146	502	681	587	551	467	735	658	859	446	694	499	585	eral1
ENSXETG00000019634	3001	268	403	382	863	686	1174	1512	1682	963	699	469	566	lig3
ENSXETG00000019636	1059	0	6	1	3	4	4	245	322	168	21	12	15	glt8d2
ENSXETG00000019639	2460	86	121	103	50	50	227	992	1126	705	390	260	336	hk2
ENSXETG00000019641	999	0	0	0	0	0	0	0	0	0	0	0	0	ENSXETG00000019641
ENSXETG00000019642	786	58	63	58	51	32	126	125	172	77	101	70	90	dguok
ENSXETG00000019643	2416	137	206	170	244	176	376	404	427	261	360	215	263	hcfc2
ENSXETG00000019644	924	17	18	23	71	64	103	108	111	68	210	175	165	fam110a
ENSXETG00000019645	1803	1214	1712	1570	633	452	800	309	390	247	51	26	42	txnrd1
ENSXETG00000019647	2147	384	558	478	1098	900	1608	2030	2453	1425	1489	1017	1210	fmr1
ENSXETG00000019650	5943	2687	3821	3308	16325	12553	56747	30724	35183	20528	68498	47675	54034	myh11
ENSXETG00000019651	1600	88	111	113	157	118	310	405	427	287	276	218	222	sgsh
ENSXETG00000019652	3366	384	565	475	470	353	1180	732	837	545	915	672	721	card14
ENSXETG00000019653	2279	1044	1524	1327	1494	1090	1915	2140	2420	1481	1511	1011	1251	gps1
ENSXETG00000019654	2253	445	684	531	589	448	681	1171	1352	817	494	327	406	dus1l
ENSXETG00000019655	900	0	0	1	0	0	0	0	0	0	1	0	1	ENSXETG00000019655
ENSXETG00000019656	1045	499	772	706	384	297	601	968	1101	695	762	549	581	nfyb
ENSXETG00000019657	1077	245	434	324	514	368	836	857	1051	614	735	518	606	rfng
ENSXETG00000019659	834	1	3	5	14	13	32	371	451	273	39	27	30	ldlrad4
ENSXETG00000019661	4612	18	23	20	134	100	82	334	428	221	96	73	80	abcc1
ENSXETG00000019662	3348	162	213	185	319	245	298	283	360	189	275	178	198	ENSXETG00000019662
ENSXETG00000019663	1485	2	1	6	2	3	9	97	127	69	140	72	106	insc
ENSXETG00000019664	1872	272	428	329	2731	2009	1567	688	743	477	1203	839	918	cyp2r1
ENSXETG00000019666	7071	224	341	290	750	538	280	1401	1586	989	891	624	705	fasn
ENSXETG00000019668	3231	871	1230	1034	713	526	885	1164	1342	776	911	685	772	seh1l
ENSXETG00000019669	1239	482	664	588	673	548	1086	1210	1480	802	1059	728	837	ptpn2
ENSXETG00000019670	1317	712	994	800	503	376	543	1016	1322	730	278	192	215	psmg2
ENSXETG00000019671	2235	67	100	78	310	212	169	338	377	246	143	100	124	cep76
ENSXETG00000019672	1893	69	116	101	242	209	419	289	353	228	565	369	438	SPIRE1
ENSXETG00000019675	336	3	12	12	6	3	6	2	1	0	2	6	1	calca
ENSXETG00000019677	2679	65	98	91	322	244	466	1076	1169	733	734	508	552	pde3b
ENSXETG00000019680	735	1212	1697	1520	1623	1243	1733	3735	4014	2723	1015	720	830	psma1
ENSXETG00000019682	3224	4954	7350	6293	12275	9119	12538	8340	8884	5781	8413	6104	6467	copb1
ENSXETG00000019683	4106	2391	3375	2791	2827	2100	2636	4079	4623	2816	1336	988	1068	nomo3
ENSXETG00000019685	582	68	114	90	134	103	238	314	351	213	245	160	201	ENSXETG00000019685
ENSXETG00000019686	1251	0	0	1	6	6	14	68	73	50	35	25	30	tub
ENSXETG00000019687	1660	82	137	91	192	156	308	2274	2720	1606	845	654	617	tubb6
ENSXETG00000019688	1454	3	0	2	12	5	14	41	65	35	56	33	42	ric3
ENSXETG00000019690	1839	194	267	234	112	69	80	616	682	463	92	73	67	cidea
ENSXETG00000019693	2845	1	1	3	26	11	55	196	231	155	46	37	50	xylt1
ENSXETG00000019694	930	121	132	132	134	102	178	398	563	345	254	163	205	impa2
ENSXETG00000019699	1197	198	261	218	135	139	311	273	392	168	328	246	282	mppe1
ENSXETG00000019700	2227	102	120	118	28	17	69	1389	1697	998	134	73	121	slc16a3
ENSXETG00000019701	3591	988	1458	1222	3119	2349	4431	3458	4001	2351	3607	2687	2934	hgs

ENSXETG00000019702	3538	248	309	255	648	476	919	1153	1403	830	1340	919	1008	gnal
ENSXETG00000019705	2442	5071	7217	6073	11432	8742	5978	2734	3191	1869	5471	3872	4526	slc25a10
ENSXETG00000019706	2376	99	116	115	210	168	178	443	500	288	82	69	77	mis18bp1
ENSXETG00000019709	1059	2	1	0	95	72	29	27	37	31	31	28	27	cdkl1
ENSXETG00000019711	4158	4	15	13	39	37	78	138	129	98	131	87	95	magi2
ENSXETG00000019713	1415	180	235	203	255	147	341	263	299	176	418	279	321	atp5s
ENSXETG00000019714	1650	132	188	154	356	235	999	212	279	158	594	370	442	l2hgdh
ENSXETG00000019718	1958	860	1218	1011	1444	1114	1694	836	1058	603	1498	1028	1097	cited4
ENSXETG00000019720	2260	263	372	357	491	365	369	479	605	362	365	296	306	acot7
ENSXETG00000019722	2283	18	19	17	30	31	86	117	183	102	54	34	37	phtf2
ENSXETG00000019725	2432	201	288	237	509	359	568	861	1019	562	835	580	664	rsbn1l
ENSXETG00000019727	2076	77	102	101	142	102	825	2154	2477	1440	143	99	101	epx
ENSXETG00000019728	3154	188	297	251	302	271	541	1152	1375	822	659	478	503	ptpn12
ENSXETG00000019730	825	5467	8010	6964	5854	4393	10132	2981	3490	2109	3972	2831	3068	cideb
ENSXETG00000019731	1824	1	7	1	1	1	7	14	18	6	2	1	2	slc13a5
ENSXETG00000019733	2703	5	4	3	88	63	9	18	18	16	8	5	6	ccdc146
ENSXETG00000019734	1290	31	49	40	102	70	233	477	619	324	186	136	168	fgl2
ENSXETG00000019736	2409	239	362	268	424	317	853	465	597	308	425	333	350	fam185a
ENSXETG00000019739	1287	1	1	4	15	15	18	105	120	53	42	31	40	lrrc17
ENSXETG00000019740	1206	13	35	19	69	56	110	74	65	33	103	82	57	ENSXETG00000019740
ENSXETG00000019741	1548	175	254	185	371	288	446	381	413	238	379	296	283	ENSXETG00000019741
ENSXETG00000019743	1790	133	176	163	162	118	433	155	198	129	251	196	203	armc10
ENSXETG00000019745	1251	329	553	434	587	415	894	599	674	363	634	422	477	napepld
ENSXETG00000019748	2527	0	0	0	7	2	0	1	2	1	0	0	0	vgll1
ENSXETG00000019749	2101	248	320	268	339	262	625	776	966	568	589	403	495	htatsf1
ENSXETG00000019750	1200	1	0	0	0	0	0	0	0	0	0	0	0	
ENSXETG00000019752	492	30	36	54	97	82	225	2	1	0	52	41	52	ENSXETG00000019752
ENSXETG00000019753	543	15	26	21	69	53	173	33	23	17	58	51	53	rtp3
ENSXETG00000019757	3620	2	8	7	59	30	89	165	198	90	41	40	47	masp1
ENSXETG00000019759	1836	67	126	122	234	203	349	751	843	494	416	280	319	ENSXETG00000019759
ENSXETG00000019760	1421	2114	3007	2506	4014	2937	4760	3865	4587	2622	6219	4326	4900	fhl1
ENSXETG00000019761	2079	232	325	323	393	290	633	631	778	462	469	325	386	slc9a6
ENSXETG00000019762	3772	211	282	221	682	530	872	757	867	507	665	473	534	ints6l
ENSXETG00000019763	1072	171	195	210	353	275	1073	1042	1221	719	598	446	529	mospd1
ENSXETG00000019764	1944	1	3	3	15	6	11	21	32	17	10	6	8	XB-GENE-5949754
ENSXETG00000019765	1597	361	455	407	611	501	633	983	1122	741	593	402	434	fam122a
ENSXETG00000019768	858	420	629	551	446	278	690	891	1080	624	472	331	400	hprt1
ENSXETG00000019769	1776	95	149	117	141	114	269	278	377	212	207	147	178	phf6
ENSXETG00000019777	669	133	188	164	964	721	325	1351	1531	892	3913	2629	2824	sst
ENSXETG00000019779	2198	106	144	147	451	320	205	5132	6016	3489	713	499	504	bcl6
ENSXETG00000019780	1305	5	10	10	66	48	31	226	245	145	32	23	21	rassf10
ENSXETG00000019786	2595	63	113	75	87	67	92	153	169	87	127	115	96	ENSXETG00000019786
ENSXETG00000019787	879	324	481	400	373	303	479	529	600	373	307	240	201	uqcc1
ENSXETG00000019789	2333	12	22	20	299	207	826	1140	1557	878	191	127	167	fam83c
ENSXETG00000019791	1191	0	0	0	0	0	0	0	0	0	0	0	0	
ENSXETG00000019793	2405	3	2	0	3	2	3	58	67	36	19	19	22	edil3
ENSXETG00000019797	996	2	4	5	11	17	42	466	555	382	53	42	43	hapln1
ENSXETG00000019800	1454	550	740	642	771	604	867	1778	2138	1229	1067	741	824	akirin2
ENSXETG00000019801	2190	180	307	225	785	638	679	922	1176	587	1110	806	946	orc3
ENSXETG00000019802	1752	234	310	281	182	116	228	203	262	144	247	192	173	rars2
ENSXETG00000019804	918	0	0	0	0	0	0	2	1	0	0	0	0	
ENSXETG00000019807	7010	166	217	205	514	406	699	1920	2038	1310	842	601	690	cntrl
ENSXETG00000019811	708	895	1383	1206	2183	1639	2096	2932	3215	2074	1816	1364	1402	rab14
ENSXETG00000019813	2464	1355	2011	1707	3130	2535	2981	16598	18416	11465	6922	4926	5517	gsn
ENSXETG00000019824	1757	3	8	6	13	9	28	52	56	31	7	9	3	ttll11
ENSXETG00000019826	1236	7028	10205	8762	11024	8511	9234	7644	8390	5163	6811	4765	4979	ddost
ENSXETG00000019831	1962	1	1	3	54	68	7	22	31	13	141	129	102	hdc
ENSXETG00000019834	2202	585	837	662	1162	823	1295	968	1110	586	1184	835	908	spg7
ENSXETG00000019835	702	39759	58302	49536	54553	40674	68759	66634	75489	44605	155440	110498	118059	rpl13
ENSXETG00000019836	1506	2	1	2	3	0	3	0	2	1	1	1	1	cpne7
ENSXETG00000019839	867	18	29	35	22	13	83	170	218	120	98	77	81	sult5a1
ENSXETG00000019840	1539	17	22	20	49	40	68	68	89	51	87	48	72	ENSXETG00000019840
ENSXETG00000019842	1137	0	0	0	0	0	0	1	1	0	0	0	0	
ENSXETG00000019843	1122	7127	10399	8891	5543	4177	14966	831	996	585	4955	3513	3925	dpep1
ENSXETG00000019844	591	1212	1807	1447	1490	1127	2847	2585	2911	1808	1534	1000	1157	chmp1a
ENSXETG00000019845	2090	67	102	91	204	144	189	255	260	168	225	153	166	vps9d1
ENSXETG00000019846	2828	619	929	812	858	640	694	1195	1436	811	508	346	421	afg3l1p
ENSXETG00000019847	381	1	1	1	7	5	11	59	78	44	7	2	5	dbndd1
ENSXETG00000019848	1534	9	24	25	233	192	41	185	189	127	27	28	32	gas8
ENSXETG00000019849	411	14	16	10	22	31	18	37	26	28	37	23	26	urah
ENSXETG00000019851	2353	58	74	82	574	433	496	804	837	520	618	363	480	XB-GENE-5993343
ENSXETG00000019853	2448	742	1029	913	1711	1316	2381	2187	2561	1517	2104	1459	1615	ap1g1
ENSXETG00000019854	3445	289	389	290	413	317	616	372	472	261	135	85	118	sgms2
ENSXETG00000019855	3427	182	239	183	264	206	252	1345	1585	936	253	200	221	papss1
ENSXETG00000019856	4340	1037	1564	1432	2889	2152	5004	2078	2393	1487	3399	2438	2765	phlpp2
ENSXETG00000019859	768	2	6	7	25	7	33	81	92	57	57	39	46	dkk2
ENSXETG00000019861	2270	279	377	322	623	488	690	768	984	502	920	635	761	marveld3
ENSXETG00000019862	1212	1651	2437	2126	2731	1987	3631	3204	3567	1967	5779	4073	4614	aimp1
ENSXETG00000019863	2184	630	808	721	604	484	1418	694	759	453	1061	722	800	spire2
ENSXETG00000019870	966	0	0	0	0	0	0	0	0	0	0	0	0	
ENSXETG00000019872	1287	0	0	0	0	0	0	0	0	0	0	0	0	ENSXETG00000019872
ENSXETG00000019874	1662	141	199	175	339	247	301	525	587	348	410	295	350	znf276
ENSXETG00000019875	622	0	1	0	2	2	0	6	5	5	7	6	4	
ENSXETG00000019879	3048	6	14	12	49	40	126	1145	1331	824	196	151	180	igfbp5
ENSXETG00000019896	1894	217	394	311	268	189	376	277	344	236	237	158	195	oxsm
ENSXETG00000019897	1590	20	11	24	103	79	183	340	371	246	207	130	169	ENSXETG00000019897
ENSXETG00000019898	1920	599	904	801	1732	1260	1924	2249	2575	1557	1745	1250	1389	rhbg
ENSXETG00000019901	2760	3	4	0	47	31	42	134	140	69	90	74	61	unc5b
ENSXETG00000019902	1572	37	78	55	90	66	175	199	236	162	201	133	167	ENSXETG00000019902
ENSXETG00000019904	780	0	2	0	0	3	1	8	5	7	3	1	0	lrrc3b
ENSXETG00000019906	3477	6	13	10	150	91	163	2	5	6	138	100	106	nek10
ENSXETG00000019908	2679	0	0	0	0	0	0	7	1	3	0	0	0	
ENSXETG00000019909	2336	111	152	114	239	168	484	455	477	274	306	221	254	tdrkh
ENSXETG00000019910	921	0	0	0	0	0	0	1	1	1	0	1	0	
ENSXETG00000019911	3885	136	195	159	1224	938	989	724	891	505	3012	2214	2405	slc4a7
ENSXETG00000019913	1349	0	0	0	0	0	0	0	0	0	0	0	0	
ENSXETG00000019914	1196	97	139	129	200	180	364	924	922	599	382	301	311	slc39a1
ENSXETG00000019915	5247	31	45	29	74	53	200	1175	1429	885	347	231	251	nes
ENSXETG00000019917	1927	540	773	680	530	402	905	1058	1139	688	840	557	603	dnaja4.2
ENSXETG00000019918	4797	176	284	228	450	359	690	800	842	556	608	436	439	dennd4b
ENSXETG00000019919	2655	102	170	155	211	144	209	573	631	378	161	96	116	spdl1
ENSXETG00000019921	1434	14	17	25	128	89	162	398	431	277	159	124	110	gatad2b
ENSXETG00000019922	5499	28	43	37	69	46	129	615	756	452	170	138	151	dock2
ENSXETG00000019924	2842	5	1	1	1	1	3	83	76	44	19	12	13	eomes
ENSXETG00000019927	2556	39	46	38	42	25	53	119	155	92	27	15	16	crabp2
ENSXETG00000019929	988	444	630	561	193	129	147	242	292	181	213	179	181	c16orf91
ENSXETG00000019930	2280	37	38	38	37	30	82	77	91	59	85	58	48	rrnad1
ENSXETG00000019931	408	181	215	201	211	149	250	248	244	175	97	63	65	ptpmt1
ENSXETG00000019932	756	2779	4036	3417	2242	1761	2868	2554	2921	1861	1617	1203	1294	ndufs3
ENSXETG00000019933	1125	2	2	5	5	4	2	6	4	6	12	12	10	c1qtnf4
ENSXETG00000019934	1857	0	0	0	2	2	3	8	10	13	10	6	10	SRRM4

ENSXETG00000019935	1413	5259	7866	6610	5752	4324	9303	4763	5295	3372	5552	3955	4385	mtch2
ENSXETG00000019936	2896	993	1386	1239	1535	1234	3571	184	202	132	1587	1042	1237	susd2
ENSXETG00000019937	2202	25	33	37	61	52	103	42	46	32	31	31	32	agbl2
ENSXETG00000019938	909	286	399	337	528	372	476	643	700	402	1074	831	824	fnbp4
ENSXETG00000019940	2538	7	8	11	86	57	121	265	371	208	125	77	112	kremen1
ENSXETG00000019941	4875	342	528	474	1099	875	3325	1255	1534	830	2518	1595	2001	shroom3
ENSXETG00000019942	3341	42	47	44	149	123	162	235	196	139	277	172	205	znrf3
ENSXETG00000019944	1764	18547	26966	22610	10294	8039	22015	6053	7141	3945	5938	4091	4701	tcn2
ENSXETG00000019945	951	1	0	1	7	2	24	71	94	51	15	10	12	slc35e4
ENSXETG00000019946	564	32	46	26	57	40	137	46	53	29	71	43	59	dusp18
ENSXETG00000019951	1161	0	0	0	0	0	0	0	0	0	0	0	0	
ENSXETG00000019955	3528	136	174	151	243	174	525	220	284	136	389	283	283	aff1
ENSXETG00000019964	1587	236	329	307	650	456	860	976	1055	680	965	663	722	spty2d1
ENSXETG00000019965	1662	0	1	0	132	91	46	99	137	72	73	61	62	kcnq1
ENSXETG00000019972	873	9	17	15	18	24	33	49	44	38	30	12	21	ighmbp2
ENSXETG00000019978	537	571	909	744	333	256	338	491	510	340	247	185	179	mrpl21
ENSXETG00000019980	2430	0	0	0	0	0	0	0	0	0	0	0	0	
ENSXETG00000019981	2523	0	0	0	0	0	0	0	0	0	0	0	0	
ENSXETG00000019982	1684	0	0	0	0	0	1	2	2	0	1	1	1	ENSXETG00000019982
ENSXETG00000019983	909	123	159	135	277	202	799	278	349	182	682	462	574	yipf1
ENSXETG00000019985	2076	0	0	0	0	0	0	0	0	0	0	0	0	
ENSXETG00000019986	1943	71	125	79	164	125	130	200	238	139	136	83	109	lrrc42
ENSXETG00000019988	2190	0	1	0	1	1	0	8	5	2	1	0	0	ENSXETG00000019988
ENSXETG00000019990	1755	4138	5714	4881	4440	3367	8920	4272	5166	3012	4946	3554	3906	tmem59
ENSXETG00000019991	1220	126	148	115	138	85	267	309	405	228	202	174	163	tceanc2
ENSXETG00000019993	1263	679	1056	828	739	533	652	897	990	584	356	274	301	mrpl37
ENSXETG00000019994	2503	409	592	504	695	573	931	1256	1462	861	1082	728	869	ssbp3
ENSXETG00000019997	3120	0	2	1	0	1	8	4	2	0	0	1	2	slc12a3
ENSXETG00000019998	1665	286	474	397	722	558	1192	437	495	317	1274	980	1087	acot11
ENSXETG00000019999	2806	14	17	14	56	35	33	234	257	142	84	53	102	fcrl3
ENSXETG00000020001	2002	4707	6722	5657	7193	5497	13187	200	234	116	3936	2885	3117	fam151a
ENSXETG00000020003	1452	306	413	377	312	205	350	576	695	407	425	362	381	ttc4
ENSXETG00000020004	1854	1	2	0	0	0	0	24	11	10	1	0	0	fcrl2
ENSXETG00000020006	1242	0	0	0	0	0	0	0	0	0	0	0	0	
ENSXETG00000020007	807	110	147	131	109	70	154	127	170	90	139	105	126	ENSXETG00000020007
ENSXETG00000020008	1698	328	430	383	369	242	397	309	350	194	201	125	149	ttc22
ENSXETG00000020009	2316	32	53	43	251	218	498	1148	1546	882	308	216	244	cdh5
ENSXETG00000020010	1657	3026	4355	3658	4675	3644	2907	302	367	183	654	426	513	dhcr24
ENSXETG00000020012	2046	848	1245	1162	4017	3004	3121	142	151	86	3371	2337	2631	pcsk9
ENSXETG00000020013	1775	52	77	51	137	80	120	148	209	107	30	21	25	cenpl
ENSXETG00000020014	1978	151	229	216	258	196	227	333	383	237	116	81	92	dars2
ENSXETG00000020017	1011	695	1016	883	960	724	1114	1742	2006	1142	811	551	590	nudt21
ENSXETG00000020018	1856	533	834	675	585	411	600	761	797	534	588	380	420	ogfod1
ENSXETG00000020019	3893	641	978	830	1188	905	1296	1755	2124	1192	1692	1227	1310	rabgap1l
ENSXETG00000020020	4348	1567	2241	1819	2866	2201	4404	3539	4136	2343	3357	2439	2698	amfr
ENSXETG00000020021	2169	41	70	45	118	108	63	106	141	77	84	56	69	bbs2
ENSXETG00000020022	2729	29	41	39	125	95	151	777	852	481	190	131	157	gnao1
ENSXETG00000020027	2145	125	186	200	244	168	316	2545	2935	1785	673	446	486	cbfb
ENSXETG00000020030	3384	72	80	77	192	172	223	334	334	188	327	228	240	rc3h1
ENSXETG00000020032	2139	262	380	303	494	399	553	246	338	194	835	637	571	pdcd2l
ENSXETG00000020033	1735	11	6	16	13	8	3	15	15	11	1	1	3	serpinc1
ENSXETG00000020037	1299	0	0	0	0	0	0	0	0	0	0	0	0	
ENSXETG00000020040	1737	273	354	280	373	289	553	668	793	488	544	419	445	rhot1
ENSXETG00000020041	1170	202	293	237	559	439	826	759	808	527	888	613	628	zbtb7b
ENSXETG00000020042	1563	58	88	78	148	128	221	397	437	287	292	191	215	zbtb37
ENSXETG00000020043	1062	26	30	25	81	70	101	615	717	418	101	69	77	smad6.2
ENSXETG00000020044	1242	457	682	592	496	353	608	1480	1778	998	952	642	761	cacybp
ENSXETG00000020045	396	430	608	532	241	166	168	426	462	269	179	141	127	mrps14
ENSXETG00000020047	1470	100	163	127	441	300	263	324	335	246	269	223	229	flad1
ENSXETG00000020049	855	1540	2113	1869	2901	2166	2569	4260	4753	2998	1315	892	968	psmd4
ENSXETG00000020052	903	112	151	157	210	160	286	303	304	189	334	244	217	tefm
ENSXETG00000020053	1332	427	663	555	738	561	781	1622	1785	1117	1195	859	892	crlf3
ENSXETG00000020055	2153	445	628	504	843	723	974	2080	2427	1420	999	788	841	suz12
ENSXETG00000020057	1918	672	945	903	873	664	811	1312	1508	945	983	694	755	utp6
ENSXETG00000020061	990	787	1184	1073	914	669	2141	2917	3427	2056	1206	752	873	scpep1
ENSXETG00000020064	4260	1	1	0	1	0	3	2	2	0	0	0	1	tnn
ENSXETG00000020070	785	0	0	0	0	0	0	0	0	0	0	0	0	
ENSXETG00000020072	1002	0	0	0	0	0	0	0	0	0	0	0	0	
ENSXETG00000020074	930	0	0	0	0	0	0	0	0	0	0	0	0	
ENSXETG00000020075	12678	371	489	458	987	774	1114	1729	2085	1221	1248	953	1014	trrap
ENSXETG00000020076	1158	0	2	1	1	3	5	31	38	24	5	3	7	tmem130
ENSXETG00000020086	3255	252	304	302	1776	1417	2117	1784	1973	1157	3034	2220	2556	irs1
ENSXETG00000020088	1710	126	181	136	159	141	238	281	325	184	210	142	184	rhbdd1
ENSXETG00000020089	2326	779	1133	981	1583	1203	1971	3190	3591	2070	2167	1623	1800	rnf13
ENSXETG00000020090	2971	231	331	271	587	423	1199	5840	6985	4059	1556	1142	1257	pfn2
ENSXETG00000020092	3673	215	294	268	723	528	917	832	957	633	1028	724	772	tsc22d2
ENSXETG00000020093	2058	1437	2006	1687	2056	1489	3037	1981	2286	1299	2731	1874	2179	eif2a
ENSXETG00000020094	1293	4386	6345	5252	4570	3689	5597	5208	6145	3758	3187	2147	2252	selenot
ENSXETG00000020096	957	43	54	50	96	74	169	138	123	96	157	126	144	siah2
ENSXETG00000020097	699	5	3	4	6	2	47	14	27	22	63	41	56	clrn1
ENSXETG00000020123	1675	14789	22282	18909	14802	11322	11338	27556	30814	18719	8688	6065	6706	eif4a1
ENSXETG00000020127	1291	396	545	477	953	750	1672	12912	13487	8317	2157	1418	1623	ENSXETG00000020127
ENSXETG00000020131	2373	1	0	0	1	0	0	0	0	0	0	0	1	cdh9
ENSXETG00000020133	1406	451	689	570	750	626	1047	1285	1544	1071	815	592	621	cdc42se1
ENSXETG00000020139	1290	368	498	422	775	557	1100	363	342	235	977	662	749	arhgef39
ENSXETG00000020140	4521	322	438	390	651	520	777	697	864	459	759	456	601	cdcp1
ENSXETG00000020142	1341	0	0	0	0	0	0	7	11	5	0	0	0	ENSXETG00000020142
ENSXETG00000020143	902	0	0	0	0	2	2	1	6	2	2	2	0	clec3b
ENSXETG00000020144	936	755	1082	960	308	246	329	754	886	527	399	258	262	exosc7
ENSXETG00000020145	1644	1944	2685	2280	2102	1661	4237	2663	3306	1911	2704	1897	2151	zdhhc3
ENSXETG00000020146	2199	691	1049	889	1388	1078	1601	3832	4438	2684	1078	722	816	ezh2
ENSXETG00000020148	3103	795	1094	967	1209	961	1526	2498	3033	1752	1407	1043	1147	cul1
ENSXETG00000020157	984	24	17	16	26	16	25	53	40	29	43	23	32	nsun3
ENSXETG00000020159	1692	78	117	79	224	166	254	236	295	155	359	270	293	stx19
ENSXETG00000020160	4521	53	64	59	284	193	807	3598	4077	2354	1031	805	854	pros1
ENSXETG00000020163	2097	222	298	252	665	545	991	1248	1368	860	1157	828	909	raf1
ENSXETG00000020165	951	472	714	563	1287	1027	1704	1001	1082	691	1763	1187	1282	mkrn2
ENSXETG00000020167	1407	75	116	92	343	271	381	559	666	367	621	452	456	tox
ENSXETG00000020173	2889	4	7	5	29	29	51	622	815	490	150	77	100	epha3
ENSXETG00000020174	1331	5	9	9	11	7	21	36	42	19	39	24	40	ca8
ENSXETG00000020175	1413	0	0	0	4	1	12	35	35	34	90	51	59	chrm2
ENSXETG00000020178	1577	4968	7157	6258	4940	3584	5882	6571	7738	4517	3670	2445	2823	rab2a
ENSXETG00000020183	1593	224	343	260	251	235	378	383	429	293	395	257	288	hdhd2
ENSXETG00000020191	2247	12	13	17	74	41	169	260	326	170	274	217	210	ezh1
ENSXETG00000020196	1377	0	0	0	0	0	0	0	0	0	2	0	1	
ENSXETG00000020197	658	1535	2125	1839	1314	1147	1349	1698	2002	1297	1538	1030	1163	ssbp1
ENSXETG00000020198	1405	367	572	497	571	366	526	436	501	317	412	348	309	agk
ENSXETG00000020201	3846	3	3	3	3	4	10	90	117	66	39	39	37	cntnap1
ENSXETG00000020204	1976	242	300	289	1499	1137	1701	3992	4057	2725	3199	2282	2423	add3
ENSXETG00000020205	1821	73	133	101	187	126	226	262	310	189	241	155	184	ENSXETG00000020205
ENSXETG00000020206	2275	96	133	98	322	218	797	17290	20684	11599	1593	1104	1226	timp2

ENSXETG00000020212	1631	1239	1772	1557	1861	1438	2338	1876	2021	1274	1569	1132	1296	cant1
ENSXETG00000020214	2022	135	239	216	439	297	817	943	1037	657	676	465	498	engase
ENSXETG00000020215	1585	23	46	21	31	21	72	102	127	74	84	65	67	znf300
ENSXETG00000020218	2070	104	124	130	285	224	452	1383	1614	992	608	431	478	tmem204
ENSXETG00000020224	3570	144	213	164	359	275	587	659	698	430	491	392	393	cramp1
ENSXETG00000020228	1770	782	1187	974	5372	4103	6109	727	747	513	3498	2516	2799	ENSXETG00000020228
ENSXETG00000020229	1463	915	1291	1064	1085	862	1353	1088	1214	683	1326	927	953	mrps34
ENSXETG00000020230	1685	79	127	85	139	88	99	97	91	55	71	53	51	hs3st5
ENSXETG00000020232	5109	857	1283	1128	2906	2221	5146	2587	2935	1747	3015	2128	2291	abca3
ENSXETG00000020233	1223	116	177	155	172	119	118	462	488	322	87	58	78	wdr73
ENSXETG00000020235	873	3	6	0	13	18	7	1	3	2	9	4	1	stra8
ENSXETG00000020236	1185	16	7	16	21	13	54	86	100	52	38	29	34	rbfox3
ENSXETG00000020237	2909	156	244	202	431	332	1297	1215	1243	819	643	419	518	wdr91
ENSXETG00000020238	2034	1	6	3	5	6	3	8	17	5	9	4	7	agbl3
ENSXETG00000020247	1800	46	63	59	74	57	62	122	138	105	68	49	61	large1
ENSXETG00000020250	5483	696	996	877	1549	1177	1824	1286	1512	895	1363	936	1047	ltn1
ENSXETG00000020252	2169	438	647	545	302	252	424	1032	1058	668	616	502	503	plekhn1
ENSXETG00000020253	1773	15	13	19	33	32	59	91	102	49	48	54	43	klhl17
ENSXETG00000020254	917	255	417	313	405	312	186	129	148	74	491	350	386	rwdd2b
ENSXETG00000020255	3444	290	433	395	400	290	385	632	674	431	534	384	398	usp16
ENSXETG00000020258	699	0	0	0	0	0	1	0	1	2	1	0	0	usp48
ENSXETG00000020259	1266	1068	1581	1367	686	445	746	535	568	370	1174	788	967	mcrip2
ENSXETG00000020260	4429	295	444	372	380	293	549	934	1015	579	344	221	281	mettl26
ENSXETG00000020261	1665	0	5	3	8	4	5	18	18	5	25	11	18	wfikkn1
ENSXETG00000020262	1951	5339	7851	6707	8539	6686	9505	13096	15018	9062	9590	6876	7499	cct8
ENSXETG00000020265	360	2	0	0	0	0	0	0	0	0	0	0	0	
ENSXETG00000020266	5689	634	958	741	828	572	1733	1764	2152	1241	1204	776	925	bach1
ENSXETG00000020273	993	175	251	223	170	127	299	445	505	327	209	156	205	mtfr1
ENSXETG00000020277	1740	0	0	0	0	1	3	27	28	19	18	7	10	dync1i1
ENSXETG00000020279	2618	686	916	779	988	758	3847	1945	2358	1447	621	434	531	amdhd2
ENSXETG00000020280	3825	12	15	26	46	33	93	480	498	284	122	87	98	fam171a1
ENSXETG00000020281	1618	270	337	335	298	214	489	721	876	531	358	290	307	armc1
ENSXETG00000020282	3108	17	26	22	114	94	152	702	858	563	337	213	240	itga8
ENSXETG00000020283	1368	11	16	14	35	25	48	204	241	153	75	47	53	pde7a
ENSXETG00000020284	1710	80	122	138	179	154	370	317	304	203	188	152	152	tbc1d24.1
ENSXETG00000020285	2664	1629	2330	2017	2948	2318	4738	5698	6583	3928	4839	3459	3663	dync1i2
ENSXETG00000020286	1512	1	1	0	4	1	6	23	43	15	1	5	1	trim55
ENSXETG00000020288	1764	4	5	7	66	61	167	1122	1296	738	449	321	391	ntn3
ENSXETG00000020289	3173	4266	6122	5009	5570	4390	7878	952	1120	643	3325	2305	2429	pdk4
ENSXETG00000020292	1913	401	550	491	634	489	960	1764	1968	1189	1091	724	916	smad2
ENSXETG00000020295	1287	5	7	6	7	9	4	1	2	3	0	0	0	asb4
ENSXETG00000020296	5268	735	1047	871	2593	2069	2844	3414	3876	2182	3062	2138	2478	tjp1
ENSXETG00000020297	1399	224	325	257	203	166	457	441	571	301	385	262	319	fam188a
ENSXETG00000020299	2122	5509	7688	6404	4343	3094	5063	1178	1430	829	1954	1346	1529	pon2
ENSXETG00000020301	1122	0	2	7	59	36	73	69	69	43	68	53	54	nog2
ENSXETG00000020302	2076	1	0	0	9	6	1	2	3	4	1	1	2	ankfn1
ENSXETG00000020303	1575	0	1	2	35	28	10	87	92	60	78	47	72	zbtb7c
ENSXETG00000020305	4047	621	882	794	410	354	1470	693	873	474	793	544	584	ppp1r9a
ENSXETG00000020306	1269	117	161	114	79	53	149	588	602	350	395	281	302	sgce
ENSXETG00000020308	618	42658	62935	53851	64095	48029	69233	61621	69546	42815	147723	102402	111468	rpl17
ENSXETG00000020309	6695	475	755	569	1975	1541	1503	942	1042	566	1473	1072	1235	casd1
ENSXETG00000020310	2364	513	698	586	887	699	1437	1595	1798	1049	1466	1060	1060	dym
ENSXETG00000020313	2606	285	380	339	642	466	725	780	940	577	756	535	573	tarsl2
ENSXETG00000020317	1146	46	83	56	215	169	307	1065	1104	661	817	633	695	smad7
ENSXETG00000020318	4166	1094	1649	1409	3401	2652	6796	59157	65275	39703	7627	5373	5769	col1a2
ENSXETG00000020322	1834	4	4	2	304	227	55	37	39	27	39	31	33	cfap53
ENSXETG00000020323	7074	1745	2386	1963	3519	2664	4613	2372	2773	1548	3085	2258	2415	myo5b
ENSXETG00000020324	1665	43	68	59	125	97	172	231	225	138	154	113	127	cpeb2
ENSXETG00000020325	777	0	1	0	0	0	9	6	7	3	3	8	4	cc2d2a
ENSXETG00000020331	2064	1280	1860	1580	1176	855	1327	1110	1315	786	1751	1350	1382	fbxl5
ENSXETG00000020332	1051	27	36	46	40	32	70	443	458	279	53	23	34	cd38
ENSXETG00000020333	2172	5715	8351	6902	5609	4395	6201	2987	3547	2055	2472	1811	2031	acaa2
ENSXETG00000020334	2241	488	698	579	1495	1213	432	1187	1372	860	374	251	291	fgfbp1
ENSXETG00000020335	576	1	0	0	1	1	0	0	2	1	0	0	0	fgfbp2
ENSXETG00000020336	2439	0	0	1	4	1	3	27	27	14	3	3	5	prom1
ENSXETG00000020337	1227	221	355	283	96	85	151	340	376	267	118	78	85	adal
ENSXETG00000020338	1506	361	475	451	510	363	1182	957	1151	672	884	622	695	tapt1
ENSXETG00000020339	894	4	7	4	13	15	40	59	72	47	51	28	54	ldb2
ENSXETG00000020340	2131	35	32	30	83	54	82	380	426	229	127	102	114	larp6
ENSXETG00000020341	897	2307	3645	2996	2248	1690	1484	516	605	352	961	705	773	qdpr
ENSXETG00000020342	2404	2	8	6	66	56	41	155	203	101	29	28	28	lrrc49
ENSXETG00000020343	1790	1872	2703	2344	2389	1766	1548	1114	1249	728	955	716	709	lap3
ENSXETG00000020344	697	362	490	382	433	297	492	644	724	455	349	229	285	med28
ENSXETG00000020346	3155	43	67	58	177	142	247	770	826	515	425	330	366	gabbr1
ENSXETG00000020349	1847	359	514	377	647	441	896	1241	1365	848	949	660	756	sh3bp5l
ENSXETG00000020351	629	3022	4478	3832	3815	2781	4050	2667	2862	1979	2958	2065	2185	ndufb10
ENSXETG00000020352	731	571	837	719	872	681	825	1662	1847	1105	486	338	351	bcas2
ENSXETG00000020353	1096	114	159	146	162	142	188	344	359	227	183	135	164	ENSXETG00000020353
ENSXETG00000020354	3960	1048	1510	1286	2729	2125	4459	2546	2866	1861	2224	1573	1671	scap
ENSXETG00000020355	673	49061	73069	62339	60344	46497	67290	60710	69322	41222	130102	89872	99572	rps5
ENSXETG00000020357	2224	120	182	149	264	213	450	469	558	339	526	345	406	zbtb9
ENSXETG00000020358	3751	5	6	4	95	65	72	259	292	183	165	93	107	syngap1
ENSXETG00000020359	5754	289	497	403	1212	974	2417	1851	2141	1225	1881	1263	1438	ptpn23
ENSXETG00000020362	2957	138	208	145	280	206	398	987	1236	694	672	558	561	phf1
ENSXETG00000020363	1614	65	130	98	186	162	242	511	564	371	294	240	208	slc22a15.1
ENSXETG00000020364	882	0	0	0	2	1	0	6	6	3	2	2	0	ENSXETG00000020364
ENSXETG00000020365	1629	1011	1460	1223	1285	964	1426	605	751	409	728	555	553	hsd17b8
ENSXETG00000020366	3359	55445	82109	70758	115377	88394	133095	30439	33003	20494	98883	69700	76174	atp1a1
ENSXETG00000020367	1576	1210	1836	1580	1388	1017	959	2398	2898	1727	643	452	529	slc39a7
ENSXETG00000020368	2397	16	32	15	138	102	133	45	67	35	31	30	24	wdr78
ENSXETG00000020369	2460	8	2	7	28	24	41	100	100	63	64	58	41	sgip1
ENSXETG00000020370	1044	217	304	238	253	202	353	539	593	374	358	251	244	ring1
ENSXETG00000020372	3062	504	708	628	1175	936	1775	1106	1419	721	1111	825	932	daxx
ENSXETG00000020373	2373	0	1	0	0	0	0	0	0	0	0	0	0	
ENSXETG00000020374	3636	98	139	108	156	126	393	1146	1360	816	707	495	590	pde4b
ENSXETG00000020378	1676	99	141	123	345	257	646	844	922	562	732	494	488	mef2d
ENSXETG00000020384	4908	68	81	95	563	454	357	563	667	396	87	62	68	iqgap3
ENSXETG00000020385	1944	512	677	580	967	761	1550	1009	1193	644	826	612	714	vps52
ENSXETG00000020387	3591	1	0	3	3	3	4	12	5	3	3	2	2	lepr
ENSXETG00000020388	1931	0	0	0	0	0	0	0	2	1	0	0	0	aqp2
ENSXETG00000020389	735	38	44	41	72	58	91	253	290	148	78	49	59	b3galt4
ENSXETG00000020390	6971	3	0	0	15	13	28	43	56	43	33	21	31	dnajc6
ENSXETG00000020391	879	1218	1826	1627	1515	1191	2032	1374	1560	982	1814	1234	1405	naxe
ENSXETG00000020392	696	4	6	5	10	10	27	50	55	35	39	40	48	ak4
ENSXETG00000020393	1768	2	2	2	1	1	5	13	18	4	8	5	9	faim2
ENSXETG00000020394	405	33029	49011	42983	50185	37764	60771	51019	59694	34755	119735	82421	91885	rps18
ENSXETG00000020396	3458	271	361	322	774	680	1473	1859	2346	1331	1756	1175	1460	jak1
ENSXETG00000020397	1508	622	887	752	521	425	1022	712	817	486	1103	704	762	gpatch4
ENSXETG00000020398	1767	616	887	814	680	496	496	862	1056	605	694	503	520	wdr46
ENSXETG00000020399	2390	2073	3063	2485	3850	3020	6340	4052	4417	2642	6424	4587	4753	ubqln4
ENSXETG00000020401	657	1846	2673	2254	1548	1160	1620	2220	2539	1452	1724	1257	1278	pfdn6

ENSXETG00000020402	981	112	163	157	229	191	441	752	917	524	518	416	367	tapbp
ENSXETG00000020404	2057	3104	4523	3738	4342	3252	5750	7977	9091	5480	6384	4631	5029	rab25
ENSXETG00000020405	2022	63	87	92	288	212	203	580	672	370	265	175	200	zbtb22
ENSXETG00000020406	2689	363	537	457	1140	843	2439	1191	1329	852	1713	1200	1377	raver2
ENSXETG00000020409	2492	184	232	183	791	644	454	808	973	529	125	88	104	kifc1
ENSXETG00000020410	3651	95	129	102	148	105	273	384	441	245	435	306	327	cachd1
ENSXETG00000020412	1473	0	0	0	1	0	0	0	0	0	0	0	0	
ENSXETG00000020413	2230	39	47	42	144	121	202	1810	2035	1308	336	222	289	fkbp9
ENSXETG00000020416	3324	1806	2658	2186	3120	2336	4166	4277	4789	2664	2517	1863	2099	rxrb
ENSXETG00000020417	2446	698	866	777	4025	3054	1647	6705	7639	4730	5944	4276	4627	krt5.7
ENSXETG00000020429	2143	1	0	0	0	0	0	20	18	6	6	4	6	krt5.2
ENSXETG00000020431	2067	8963	12261	10505	5719	4683	6024	6218	7373	4295	5993	4129	4833	ssr1
ENSXETG00000020432	1671	0	0	0	1	1	0	0	0	0	0	0	0	
ENSXETG00000020435	1519	1833	2588	2211	2919	2089	5724	3670	4253	2421	4346	3084	3542	rbm22
ENSXETG00000020436	1665	320	427	375	297	202	476	572	642	390	434	320	322	riok1
ENSXETG00000020438	1691	130	165	166	382	316	219	609	675	398	91	46	86	pbk
ENSXETG00000020440	1863	1	2	0	5	2	5	9	10	8	10	5	7	cdh12
ENSXETG00000020442	2238	45	63	44	321	274	171	310	291	187	62	38	59	esco2
ENSXETG00000020444	8874	1621	2202	1922	6938	5425	7250	4962	5881	3331	10936	7798	8988	dsp
ENSXETG00000020447	1847	142	191	211	243	173	313	379	450	284	337	228	267	snrnp48
ENSXETG00000020448	3567	0	0	0	0	0	0	0	0	0	0	0	0	
ENSXETG00000020454	2132	39583	58383	50024	80713	61542	68702	76584	84997	52739	113050	80194	86551	krt8
ENSXETG00000020455	1374	15173	22531	19568	23200	17426	19240	34785	38443	24068	31675	22336	24182	krt18
ENSXETG00000020457	2047	7235	10458	8750	12955	9845	17543	11089	12844	7256	8442	6179	6677	txndc5
ENSXETG00000020459	1006	271	411	347	293	231	309	659	836	445	171	144	164	eef1e1
ENSXETG00000020461	1116	502	749	673	435	347	958	687	860	481	507	345	375	slc35b3
ENSXETG00000020463	2514	0	0	0	0	0	0	0	0	0	0	0	0	
ENSXETG00000020465	2271	0	0	0	0	0	0	0	0	0	2	0	0	
ENSXETG00000020467	2385	19	20	19	77	66	258	1553	1700	1046	280	196	202	cdh11
ENSXETG00000020468	2361	66	93	63	44	29	274	22	36	15	64	44	56	ppargc1a
ENSXETG00000020469	1716	2181	3161	2779	3240	2438	3867	290	365	194	384	330	345	gba3
ENSXETG00000020470	3951	201	275	200	618	433	922	1622	1905	1119	1202	855	904	adgra3
ENSXETG00000020472	886	40	45	48	122	96	67	93	127	60	97	69	81	pacrgl
ENSXETG00000020473	4197	41	47	32	343	274	450	892	946	588	166	107	132	slit2
ENSXETG00000020475	4684	109	197	150	383	325	442	872	923	620	216	191	196	tonsl
ENSXETG00000020477	939	62	69	69	56	48	92	305	397	215	110	85	113	vrk2
ENSXETG00000020478	1972	209	291	260	273	250	423	193	276	127	471	325	444	fn3krp
ENSXETG00000020479	2993	3	5	2	4	3	7	77	84	54	147	124	124	znf750
ENSXETG00000020481	1104	88	126	106	107	93	142	214	244	143	105	75	65	fancl
ENSXETG00000020482	354	453	684	472	935	715	465	519	578	266	1144	918	850	ENSXETG00000020482
ENSXETG00000020483	816	22	29	42	60	54	181	287	349	201	113	84	87	mxd4
ENSXETG00000020484	2865	74	92	92	288	216	523	343	410	235	302	229	295	zfyve28
ENSXETG00000020486	1125	1	1	0	7	4	6	35	42	23	21	15	16	celf4
ENSXETG00000020490	5553	1	0	1	0	0	0	0	1	1	2	2	0	ENSXETG00000020490
ENSXETG00000020491	2253	538	728	727	1929	1547	1757	1025	1098	676	1154	854	947	ENSXETG00000020491
ENSXETG00000020492	2085	70	111	79	56	41	99	142	158	109	64	48	49	slc26a2
ENSXETG00000020495	426	28	39	31	23	20	165	426	467	250	37	40	45	ENSXETG00000020495
ENSXETG00000020496	1758	66	107	82	105	47	161	332	363	250	201	155	188	ENSXETG00000020496
ENSXETG00000020497	1938	24	36	39	37	39	106	96	87	48	98	88	80	ENSXETG00000020497
ENSXETG00000020498	312	1	1	0	1	0	0	0	0	1	0	0	1	
ENSXETG00000020506	1200	38	79	64	337	243	573	188	199	132	439	342	311	ENSXETG00000020506
ENSXETG00000020514	3482	194	304	255	350	289	574	1096	1286	765	508	382	467	stk38l
ENSXETG00000020515	1725	0	0	0	0	0	0	1	0	0	0	0	0	
ENSXETG00000020527	1774	217	290	269	355	281	396	692	785	417	358	238	312	hdac8
ENSXETG00000020530	1984	58	74	55	118	82	207	296	318	199	196	144	167	zbtb6
ENSXETG00000020531	1418	23	30	25	49	29	74	129	150	106	50	46	44	zbtb26
ENSXETG00000020532	1101	0	0	3	2	1	2	25	37	22	7	7	8	krt18l
ENSXETG00000020533	1450	954	1477	1223	1291	1062	1189	616	712	476	1464	906	1095	golm1
ENSXETG00000020534	2500	64911	93588	80092	105701	80033	178541	13914	16552	9378	63520	43605	50830	mep1b
ENSXETG00000020535	960	41	44	40	65	36	115	182	171	121	103	77	76	c11orf68
ENSXETG00000020537	2313	6	11	10	111	65	228	450	465	284	102	72	68	cd248
ENSXETG00000020538	2529	5	5	5	0	1	3	0	0	1	0	4	3	mcm3
ENSXETG00000020540	2529	124	172	140	293	220	427	413	485	286	437	296	353	garem1
ENSXETG00000020541	1168	172	299	212	127	100	240	323	365	234	117	66	77	XB-GENE-5802731
ENSXETG00000020542	1761	0	0	0	0	0	0	1	2	1	0	0	0	
ENSXETG00000020545	2553	0	0	0	0	0	0	0	0	0	0	0	0	
ENSXETG00000020546	981	0	0	0	0	0	0	0	0	0	0	0	0	
ENSXETG00000020547	6806	81	86	80	138	116	192	289	347	224	265	177	223	asxl3
ENSXETG00000020548	1695	2	5	3	6	16	10	29	29	29	15	14	15	nol4
ENSXETG00000020551	2509	0	0	0	0	0	0	9	18	2	12	14	6	grid1
ENSXETG00000020553	2418	37	78	60	83	68	334	269	324	173	165	122	135	bcas3
ENSXETG00000020555	3568	168	219	243	441	379	719	892	1071	569	619	461	584	wapl
ENSXETG00000020557	3510	13	21	20	126	90	172	2046	2189	1407	498	325	396	tbx2
ENSXETG00000020565	2283	147	223	198	263	177	501	322	393	213	419	303	345	rin1
ENSXETG00000020566	3015	49	78	50	48	42	135	503	573	351	199	129	149	gmip
ENSXETG00000020567	4704	525	668	579	836	715	1117	1839	2268	1381	936	715	776	brms1
ENSXETG00000020568	648	0	0	0	0	0	0	2	0	0	0	1	0	nxnl1
ENSXETG00000020569	517	306	531	466	834	664	802	1121	1211	808	698	524	524	pgls
ENSXETG00000020570	1854	136	192	200	215	165	402	766	846	562	357	232	250	colgalt1
ENSXETG00000020571	2886	82	100	81	138	118	295	564	663	370	260	175	270	b4gat1
ENSXETG00000020572	2912	3469	4674	3923	1817	1394	2005	2649	3335	1915	1391	983	1089	slc29a2
ENSXETG00000020574	923	600	859	763	432	322	363	557	664	359	344	225	242	mrpl11
ENSXETG00000020575	897	14	22	25	14	16	29	146	180	101	44	35	43	ENSXETG00000020575
ENSXETG00000020576	3986	6	12	16	3	0	17	16	20	15	20	20	21	npas4
ENSXETG00000020577	2220	1778	2644	2316	3569	2631	3640	2961	3297	2069	3037	2201	2397	dpp3
ENSXETG00000020580	84	0	0	0	0	0	0	0	0	0	0	0	0	
ENSXETG00000020585	1263	162	276	210	291	210	372	203	247	146	129	128	97	chst12
ENSXETG00000020586	1518	131	194	154	369	279	365	452	523	310	445	302	344	kat5
ENSXETG00000020587	798	2	2	0	5	4	6	113	134	75	10	11	5	ovol1
ENSXETG00000020588	2161	123	165	163	151	119	233	224	310	178	268	190	187	mus81
ENSXETG00000020591	1569	0	1	2	0	0	1	0	0	0	2	1	1	ENSXETG00000020591
ENSXETG00000020592	1527	0	3	0	0	0	2	10	14	4	4	1	1	ENSXETG00000020592
ENSXETG00000020593	2670	12271	17354	14938	25866	19909	38650	24765	28548	16599	90074	63664	71500	eif3b
ENSXETG00000020594	1299	3891	5649	4734	4837	3762	4606	1451	1801	1060	2432	1666	1917	tecr
ENSXETG00000020595	3010	3822	5306	4279	3712	2962	4028	1565	1936	1076	1136	824	1039	adk
ENSXETG00000020596	1071	344	459	382	325	249	417	517	650	346	252	208	207	fibp
ENSXETG00000020597	1482	19	35	25	157	116	84	775	839	473	231	158	170	efemp2
ENSXETG00000020598	507	3	8	4	5	8	9	15	12	6	21	21	16	dupd1
ENSXETG00000020601	3015	333	462	357	537	407	911	1298	1481	859	951	657	753	dnajb1
ENSXETG00000020602	2181	105	214	165	369	249	556	1225	1365	848	497	340	355	gipc1
ENSXETG00000020604	633	251	362	316	125	102	125	284	344	192	149	101	120	tmed10
ENSXETG00000020605	2763	166	236	218	495	384	709	1077	1201	703	668	448	510	pkn1
ENSXETG00000020606	1354	33	53	46	116	105	185	1110	1301	738	15	18	20	fosl1
ENSXETG00000020607	1647	6334	9281	8157	9804	7508	9978	16187	17900	11082	10817	7601	8352	ddx39a
ENSXETG00000020609	2019	35	60	38	56	40	121	421	507	317	56	50	57	pgghg
ENSXETG00000020610	2058	15	23	16	75	54	82	528	538	366	197	128	146	adgre5
ENSXETG00000020611	1719	6	13	7	2	5	23	124	152	93	28	24	24	adgre1
ENSXETG00000020613	1167	6	7	3	16	12	35	234	248	157	66	43	50	ENSXETG00000020613
ENSXETG00000020615	1032	15	17	18	27	39	53	315	395	207	49	40	50	p2ry14
ENSXETG00000020616	930	811	1200	963	1039	760	1038	1279	1483	860	667	446	464	taf12
ENSXETG00000020617	1371	130	164	156	150	108	110	74	69	41	87	54	55	ENSXETG00000020617

ENSXETG00000020622	621	0	1	0	0	0	1	3	3	4	0	1	0	rab39a
ENSXETG00000020623	1023	2	2	0	2	8	6	309	417	195	25	37	43	p2ry12
ENSXETG00000020624	7671	1	3	2	10	8	9	32	64	26	39	25	34	igsf10
ENSXETG00000020625	1827	305	452	433	586	415	764	1062	1268	732	623	457	506	gmeb1
ENSXETG00000020627	2527	153	234	199	592	439	289	1853	2204	1293	782	487	570	lpar2
ENSXETG00000020629	1062	4265	6308	5421	4362	3452	5676	598	725	432	1158	851	864	msra.2
ENSXETG00000020631	4108	124	149	155	299	204	586	548	605	372	808	548	653	zfat
ENSXETG00000020636	1491	724	973	900	1239	961	2123	2004	2261	1391	1809	1276	1488	ythdf2
ENSXETG00000020639	1937	947	1317	1137	9051	6793	10636	2201	2696	1473	5412	3745	4218	aadac
ENSXETG00000020644	3285	1380	2061	1819	2881	2298	3310	2143	2494	1410	2693	1875	2090	phactr4
ENSXETG00000020653	1293	133	227	190	2242	1765	1528	3023	3594	1936	3887	2683	3074	id3
ENSXETG00000020654	1179	208	321	240	249	185	319	388	464	239	182	115	165	med18
ENSXETG00000020657	2466	220	324	293	376	260	598	574	671	390	370	236	262	sesn2
ENSXETG00000020659	1584	34	79	72	64	56	288	944	1057	664	266	178	165	fgr
ENSXETG00000020662	2004	320	417	363	431	333	627	856	1043	566	768	531	609	wdtc1
ENSXETG00000020664	744	0	1	0	1	2	3	11	11	1	11	7	9	fgf14
ENSXETG00000020665	1392	0	0	0	2	2	0	11	17	6	34	21	11	itgbl1
ENSXETG00000020670	2135	3195	4652	3969	6278	4849	9954	8243	9142	5707	11560	8326	8902	ppp4c
ENSXETG00000020673	990	949	1326	1139	1225	989	2328	1614	1845	1004	2019	1310	1584	tmem222
ENSXETG00000020674	1478	6181	8676	7346	9181	7209	8171	28655	31134	20155	7174	4890	5422	aldoa
ENSXETG00000020676	3215	36	91	66	503	415	299	263	302	185	1268	874	980	sytl1
ENSXETG00000020677	2741	57	92	70	218	169	509	493	607	300	383	258	286	tmtc4
ENSXETG00000020678	2316	209	335	274	1239	953	597	1198	1313	785	966	682	811	slc9a1
ENSXETG00000020679	2652	0	1	0	15	9	16	53	51	29	15	11	12	pitpnm3
ENSXETG00000020683	2570	98	147	108	197	178	243	262	285	166	418	311	352	fam46b
ENSXETG00000020684	1140	167	217	199	330	237	314	351	375	223	297	199	235	kdf1
ENSXETG00000020685	2136	1355	2018	1628	2416	1856	1751	1439	1766	1015	895	611	709	pcca
ENSXETG00000020686	1058	695	1004	929	958	736	1033	2135	2418	1474	847	593	647	nudc
ENSXETG00000020692	1738	237	373	324	283	226	365	588	661	414	338	269	256	gpn2
ENSXETG00000020693	1416	193	266	235	312	195	440	433	531	295	572	390	436	pigv
ENSXETG00000020695	5742	496	687	595	1660	1239	2304	3011	3221	2048	3342	2268	2525	arid1a
ENSXETG00000020699	1247	780	1188	1016	528	391	439	590	688	404	360	311	280	mrps31
ENSXETG00000020700	1266	1046	1572	1386	928	699	2232	414	474	270	529	424	441	slc25a15
ENSXETG00000020701	1643	73	109	96	112	95	286	508	618	383	199	138	162	tpte2
ENSXETG00000020702	615	1554	2292	1885	2018	1524	3660	1370	1584	892	2797	2056	2248	ENSXETG00000020702
ENSXETG00000020703	759	73	89	80	189	162	294	256	310	186	146	110	112	ENSXETG00000020703
ENSXETG00000020704	937	0	0	0	0	0	0	0	0	0	0	0	0	
ENSXETG00000020706	2547	25	39	24	165	104	306	680	778	428	217	153	190	thsd1
ENSXETG00000020707	1858	1150	1728	1377	1034	831	1817	2072	2408	1409	1175	754	904	vps36
ENSXETG00000020708	1656	164	232	202	513	438	653	765	856	491	91	58	84	ckap2
ENSXETG00000020709	1643	194	290	259	215	165	248	700	726	456	348	242	280	nek3
ENSXETG00000020710	2664	0	6	5	49	45	8	2	5	4	7	11	8	nek5
ENSXETG00000020712	2084	229	384	296	346	274	441	464	581	290	255	212	244	alg11
ENSXETG00000020713	4218	141	218	138	1394	1020	545	306	347	208	756	500	580	atp7b
ENSXETG00000020714	1884	1353	1901	1748	2406	1900	3192	2652	2927	1794	2057	1328	1635	osbp
ENSXETG00000020715	663	510	743	667	1216	916	1512	1329	1550	911	1376	915	1056	zfp91
ENSXETG00000020717	1058	367	531	479	4094	3152	1188	492	509	323	767	576	625	dhrs12
ENSXETG00000020718	1203	33	52	33	75	55	108	223	243	120	57	58	58	wdfy2
ENSXETG00000020719	2634	82	130	98	121	99	262	332	341	213	194	153	163	ints6
ENSXETG00000020720	1048	495	696	566	755	547	993	1197	1428	876	810	547	653	rnaseh2b
ENSXETG00000020722	1565	2470	3840	3245	5576	4287	5264	3969	4323	2649	3483	2478	2581	selenbp1
ENSXETG00000020724	4192	338	535	502	1318	993	1783	1524	1636	1090	1877	1361	1448	pogz
ENSXETG00000020726	4086	2139	3260	2757	12203	9743	18193	6048	6497	4386	14209	10545	11135	cgn
ENSXETG00000020728	1175	197	293	264	560	418	850	951	920	624	632	535	563	snx27
ENSXETG00000020729	1655	498	727	599	1113	824	2069	1421	1582	947	1914	1397	1543	rab13
ENSXETG00000020731	933	66	83	74	129	79	139	178	239	144	111	96	80	slc25a36
ENSXETG00000020732	1119	1596	2285	1931	3023	2323	2995	5927	6422	4174	4128	2959	3111	tpm3
ENSXETG00000020733	765	78	101	88	167	125	248	320	345	218	215	171	185	c1orf43
ENSXETG00000020734	801	167	225	213	378	255	388	507	592	380	481	345	395	ubap2l
ENSXETG00000020735	1299	0	1	0	0	0	0	5	3	1	0	0	0	
ENSXETG00000020736	5571	1	7	5	54	51	19	99	122	70	96	62	81	myo5a
ENSXETG00000020738	2289	2	2	2	2	5	14	21	24	10	35	25	35	clstn2
ENSXETG00000020739	5235	510	682	664	981	737	939	627	747	477	1256	984	1046	MYO5C
ENSXETG00000020741	3340	35	79	44	200	167	285	284	338	180	408	341	340	gnb5
ENSXETG00000020742	1699	0	0	0	0	0	0	1	0	0	0	0	0	
ENSXETG00000020744	2163	1085	1622	1308	537	422	1279	2250	2796	1584	703	482	612	mapk6
ENSXETG00000020746	2260	918	1335	1133	1717	1247	1918	2237	2619	1641	1850	1311	1419	leo1
ENSXETG00000020747	1854	1294	1718	1513	2672	2146	2376	3367	4044	2368	2110	1507	1718	tmod3
ENSXETG00000020749	2832	28	34	21	119	120	203	106	154	93	193	149	132	tmod2
ENSXETG00000020751	741	0	0	0	0	0	0	0	0	0	0	0	0	
ENSXETG00000020754	1199	28	32	30	63	53	83	339	408	244	136	95	93	lysmd2
ENSXETG00000020755	1765	114	168	147	527	368	263	725	886	493	966	613	672	scg3
ENSXETG00000020756	3348	352	486	426	375	275	507	805	890	502	406	313	301	ap4e1
ENSXETG00000020757	576	0	1	0	0	0	0	0	2	2	1	0	2	tnfaip8l3
ENSXETG00000020758	2401	0	0	0	0	0	0	0	0	0	1	0	0	
ENSXETG00000020767	1650	0	1	0	0	0	0	3	4	4	0	0	0	
ENSXETG00000020770	10830	162	235	220	348	252	510	942	1109	618	998	714	826	dmxl2
ENSXETG00000020776	1260	0	0	0	0	0	0	0	0	0	0	0	0	
ENSXETG00000020779	594	7	17	14	34	27	50	93	73	41	61	44	63	dennd1a
ENSXETG00000020781	840	312	505	459	420	307	793	570	650	408	839	631	684	utp23
ENSXETG00000020783	1935	821	1151	1020	2741	2038	3180	4987	5599	3180	2728	1914	2188	rad21
ENSXETG00000020788	808	258	371	336	442	267	427	735	821	475	349	274	270	med30
ENSXETG00000020802	525	0	0	0	0	3	0	5	2	1	0	0	0	cryaa
ENSXETG00000020805	1233	0	0	0	0	0	0	0	0	0	0	0	0	
ENSXETG00000020806	1634	639	845	712	785	538	954	1083	1265	718	686	493	564	cxorf56
ENSXETG00000020813	2697	37	63	52	126	97	241	169	213	123	303	230	245	ENSXETG00000020813
ENSXETG00000020814	561	1424	2175	1825	1865	1329	1893	1583	1824	1105	1999	1443	1547	tmem111
ENSXETG00000020815	2258	217	407	315	687	524	819	1473	1581	975	1162	786	810	cdk16
ENSXETG00000020819	1425	704	1002	897	1280	997	1813	1331	1458	911	2301	1794	1767	ddx18
ENSXETG00000020822	2660	31	35	36	135	107	230	218	238	140	390	243	281	ENSXETG00000020822
ENSXETG00000020823	1761	31	20	17	64	50	129	152	138	96	188	119	122	ENSXETG00000020823
ENSXETG00000020828	1127	1221	1885	1583	964	795	1143	1818	2116	1352	1402	965	1013	mrto4
ENSXETG00000020829	2910	468	685	601	1357	999	1014	1640	1689	1071	812	564	616	emc1
ENSXETG00000020830	8223	222	306	292	1101	875	1170	1087	1142	711	999	710	776	ubr4
ENSXETG00000020831	1297	41	80	55	4	6	4	256	265	174	5	4	12	ceacam8
ENSXETG00000020835	5875	698	1021	859	557	432	1345	936	1211	739	1252	898	973	grpel2
ENSXETG00000020837	1658	96	154	109	223	156	489	449	503	268	684	520	567	kpna1
ENSXETG00000020838	821	1348	1903	1640	1299	1040	1358	2583	2841	1729	1085	775	841	taf13
ENSXETG00000020839	1878	88	122	120	170	127	181	369	414	254	215	140	173	galnt3
ENSXETG00000020840	2104	62	104	85	357	287	236	488	553	346	73	62	69	ndc80
ENSXETG00000020841	699	0	0	1	0	0	0	0	0	0	0	0	0	
ENSXETG00000020842	966	71	102	66	61	44	143	321	318	180	113	77	114	adrb2
ENSXETG00000020843	1618	9	32	26	186	156	104	127	152	106	222	147	140	gramd1c
ENSXETG00000020844	3957	80	135	112	105	96	160	627	759	440	175	144	157	ttc21b
ENSXETG00000020846	6018	0	0	0	2	3	4	17	22	8	13	13	19	scn1a
ENSXETG00000020860	1436	1	5	1	16	6	16	132	156	76	22	14	13	il17b
ENSXETG00000020861	2450	3812	5472	4566	6176	4625	10722	11042	13411	7834	7608	5391	5992	csnk1a1
ENSXETG00000020864	2270	5	2	6	19	13	51	262	306	181	63	41	45	ppp2r2b
ENSXETG00000020868	2027	1134	1601	1359	1685	1276	2082	3291	3784	2375	2223	1467	1751	nono
ENSXETG00000020869	489	0	0	0	0	0	0	0	0	0	0	0	1	
ENSXETG00000020870	2373	18	24	22	21	15	23	32	34	26	14	7	12	itgb1bp2

ENSXETG00000020871	2255	753	1006	851	755	553	669	886	1122	569	765	569	610	sdad1
ENSXETG00000020872	915	3	1	0	3	0	1	4	1	0	3	4	5	ENSXETG00000020872
ENSXETG00000020876	3387	109	175	153	305	190	213	519	615	354	440	307	318	zmym3
ENSXETG00000020877	1327	2	0	1	8	9	22	158	147	88	25	19	24	clcf1
ENSXETG00000020878	3268	10	11	10	143	108	20	523	616	349	118	85	101	atp8b1
ENSXETG00000020879	3397	0	0	1	0	3	0	0	0	0	0	1	2	foxh1
ENSXETG00000020880	3198	2018	2914	2411	14154	10557	10683	1823	2096	1168	22072	15328	17549	slc4a4
ENSXETG00000020882	1342	1678	2580	2107	1575	1229	1144	2070	2339	1537	948	658	716	ndufs8
ENSXETG00000020883	2434	1610	2439	1978	1571	1238	3211	2651	3350	1898	2400	1626	1909	unc93b1
ENSXETG00000020886	9222	2	1	2	1	1	16	17	40	22	10	8	15	xirp2
ENSXETG00000020888	1254	147	249	213	454	368	375	341	408	225	190	154	153	fech
ENSXETG00000020890	1443	0	1	0	2	2	3	1	3	0	4	5	1	ENSXETG00000020890
ENSXETG00000020891	2679	4	5	3	10	17	22	182	220	95	79	57	60	nlgn3
ENSXETG00000020892	3183	5	6	4	7	0	3	3	4	2	4	1	7	gc
ENSXETG00000020893	6668	3	4	1	283	172	25	36	36	27	28	15	22	spag17
ENSXETG00000020897	1227	1	1	0	3	2	4	28	40	15	4	3	7	st8sia3
ENSXETG00000020898	8200	239	363	320	568	428	822	1116	1361	796	615	430	491	wdr7
ENSXETG00000020899	1464	41	84	63	191	141	253	322	345	203	269	201	218	foxo4
ENSXETG00000020901	1164	1975	2807	2387	1894	1319	1915	3468	3933	2385	867	552	636	txnl1
ENSXETG00000020903	4037	1142	1717	1584	2449	1946	4260	2825	3356	1956	5208	3647	4080	iars
ENSXETG00000020904	1831	1662	2360	2044	1907	1502	2741	3857	4482	2663	2240	1612	1860	snx12
ENSXETG00000020907	2424	61	101	68	254	219	443	554	646	402	512	356	407	kctd3
ENSXETG00000020911	1023	23	33	31	38	32	66	202	239	134	75	61	64	sh2b3
ENSXETG00000020912	3177	373	550	432	827	639	1228	1612	1814	1038	1204	886	848	atxn2
ENSXETG00000020913	4019	365	578	481	642	538	617	786	971	577	1409	1022	1139	nol8
ENSXETG00000020916	1761	206	287	242	592	454	1351	809	892	504	944	689	758	brap
ENSXETG00000020918	1962	3	4	2	5	6	13	271	291	147	115	72	89	ecm2.1
ENSXETG00000020919	4320	598	905	796	922	709	1614	484	555	322	971	737	804	acad10
ENSXETG00000020924	5248	3093	4361	3666	2200	1717	2322	1729	2062	1209	1811	1292	1521	ippk
ENSXETG00000020925	13944	0	0	0	0	0	3	0	0	0	0	0	1	USH2A
ENSXETG00000020928	2728	139	209	166	329	257	445	397	485	296	575	394	453	atrip
ENSXETG00000020930	2119	11529	16802	14151	22512	17182	39203	10198	11885	6736	16320	11882	12949	aldh2
ENSXETG00000020932	1986	1	0	1	13	11	5	7	2	4	3	4	6	
ENSXETG00000020934	1051	83	124	94	249	177	440	614	691	421	228	171	158	hvcn1
ENSXETG00000020935	894	128	193	176	98	80	260	209	241	156	162	105	115	pptc7
ENSXETG00000020936	1236	11	13	10	4	5	27	1	3	1	36	18	18	rad9b
ENSXETG00000020939	1522	349	542	469	269	248	506	473	552	340	260	199	202	gpn3
ENSXETG00000020940	986	3083	4442	3805	3762	2876	5318	6714	7956	4546	3196	2262	2454	arpc3
ENSXETG00000020941	2626	275	428	370	516	424	892	809	850	483	695	474	503	anapc7
ENSXETG00000020945	2034	36	43	40	206	160	152	160	198	101	137	95	96	ift81
ENSXETG00000020947	1167	389	532	518	568	430	1399	703	781	531	1036	645	781	p2rx4
ENSXETG00000020949	1200	879	1363	1197	2367	1908	3733	3236	3381	2321	3066	2191	2329	ppm1g
ENSXETG00000020958	2523	0	0	0	0	0	0	0	0	0	0	0	0	
ENSXETG00000020959	1737	477	699	614	1155	883	1016	1183	1268	762	1150	844	850	eif2b4
ENSXETG00000020960	1536	87	111	95	178	123	373	310	377	212	232	202	198	znf513
ENSXETG00000020961	975	0	0	0	1	0	0	1	2	1	3	0	1	
ENSXETG00000020962	1599	68	94	87	229	148	723	799	934	507	543	357	390	camkk2
ENSXETG00000020963	2345	129	192	133	204	164	94	326	378	222	84	68	102	anapc5
ENSXETG00000020964	2454	149	205	177	374	312	426	674	706	447	522	417	390	gtf3c2
ENSXETG00000020965	1298	9	6	5	25	15	57	228	236	144	115	91	87	rnf34
ENSXETG00000020966	4989	427	638	510	1070	863	2059	1797	2111	1335	1988	1477	1579	kdm2b
ENSXETG00000020970	2928	336	453	433	1613	1133	1725	449	610	372	609	429	512	lpin1
ENSXETG00000020971	4968	1	5	2	43	18	51	1162	1230	819	156	117	125	robo1
ENSXETG00000020972	1182	87	97	97	142	102	187	177	233	151	116	104	94	slc22a15.2
ENSXETG00000020975	2418	649	932	863	882	731	1120	972	1174	682	1206	858	949	noc3l
ENSXETG00000020978	4665	218	282	256	391	294	448	637	667	400	482	378	358	ROBO2
ENSXETG00000020979	1398	2	3	2	1	0	0	2	5	0	1	8	3	drd1c
ENSXETG00000020982	1310	0	0	0	0	0	0	0	0	0	0	0	0	
ENSXETG00000020983	3536	187	237	225	452	365	777	935	1108	654	799	524	547	ENSXETG00000020983
ENSXETG00000020984	2997	1114	1491	1245	445	342	234	211	250	172	402	303	348	ppp1r3c.1
ENSXETG00000020986	3308	397	617	488	870	723	1536	2517	2753	1704	1145	850	918	nub1
ENSXETG00000020990	1254	336	491	392	693	573	1008	1179	1368	804	970	744	717	tmem87a
ENSXETG00000020996	213	0	0	0	0	0	0	0	0	0	0	0	0	
ENSXETG00000020999	1543	21	21	18	47	34	51	101	89	83	81	64	56	acvr2b
ENSXETG00000021000	1068	48	62	60	73	52	94	394	452	278	74	36	60	exog
ENSXETG00000021004	4557	0	0	0	11	6	5	32	32	26	40	11	13	scn2a
ENSXETG00000021005	975	0	0	0	0	0	0	0	0	0	0	0	0	
ENSXETG00000021006	930	0	0	0	0	0	0	0	0	0	0	0	0	
ENSXETG00000021009	1548	0	0	0	0	2	1	11	12	10	0	1	0	ENSXETG00000021009
ENSXETG00000021010	966	0	0	0	0	0	0	0	0	0	0	0	0	
ENSXETG00000021011	942	0	0	0	0	0	0	0	0	0	0	0	0	
ENSXETG00000021013	975	0	0	0	0	0	0	0	0	0	0	0	0	
ENSXETG00000021014	966	0	0	0	0	0	0	0	0	0	0	0	0	
ENSXETG00000021016	789	38579	57262	50367	5963	4482	3250	7052	8254	4819	13	15	31	gstp1
ENSXETG00000021017	921	0	0	0	0	0	0	0	0	0	0	0	0	
ENSXETG00000021018	927	0	0	0	0	0	0	0	0	0	0	0	0	
ENSXETG00000021019	2817	10	21	24	45	34	44	462	516	309	120	91	87	pld2
ENSXETG00000021025	969	0	0	0	0	0	0	0	0	0	0	0	0	
ENSXETG00000021029	975	0	0	0	0	0	0	0	0	0	0	0	0	
ENSXETG00000021030	645	0	0	1	0	0	0	0	0	0	0	0	0	
ENSXETG00000021033	918	0	0	0	0	0	0	0	0	0	0	0	0	
ENSXETG00000021035	3547	6	10	11	127	108	250	2549	2899	1700	478	345	379	pdgfra
ENSXETG00000021036	951	0	0	0	0	0	0	0	0	0	0	0	0	
ENSXETG00000021037	945	0	0	0	0	0	0	0	0	0	0	0	0	
ENSXETG00000021039	948	0	0	0	0	0	0	0	0	0	0	0	0	
ENSXETG00000021040	297	785	1181	1030	1384	1154	1470	2141	2195	1651	996	660	737	psmb6
ENSXETG00000021041	1350	274	333	303	351	287	743	849	949	606	471	336	358	dnajc18
ENSXETG00000021042	1930	12	9	4	100	88	163	396	397	263	340	243	269	fgfrl1
ENSXETG00000021043	651	2660	3840	3388	1527	1189	2848	2625	2953	1690	644	416	481	gltpd2
ENSXETG00000021044	1002	0	1	0	0	0	0	1	1	0	10	8	6	spon2
ENSXETG00000021046	579	0	1	0	0	0	0	7	5	2	0	0	0	vmo1
ENSXETG00000021047	1143	380	505	438	748	588	1306	2459	2750	1831	1444	1063	1192	ctbp1
ENSXETG00000021048	603	1	3	0	0	3	2	1	1	0	0	2	0	ENSXETG00000021048
ENSXETG00000021049	2991	23	35	25	215	148	315	687	821	442	496	356	443	kit
ENSXETG00000021051	591	11900	17998	15377	12270	9225	19476	6450	7138	4529	11718	8348	9059	tm4sf5
ENSXETG00000021053	1079	719	1072	945	756	556	757	1165	1256	780	869	571	615	ENSXETG00000021053
ENSXETG00000021054	486	2647	3876	3210	2688	2083	3717	444	520	331	1544	1099	1198	cldn15.1
ENSXETG00000021057	951	0	0	0	0	0	0	0	0	0	0	0	0	
ENSXETG00000021058	966	0	0	0	0	0	0	0	0	0	0	0	0	
ENSXETG00000021059	753	0	0	0	0	0	0	1	0	0	0	0	0	
ENSXETG00000021061	4041	19	17	18	91	67	120	220	228	151	133	102	124	kdr
ENSXETG00000021062	966	0	0	0	0	0	0	0	0	0	0	0	0	
ENSXETG00000021063	1227	197	292	225	164	142	184	335	371	221	177	129	121	srd5a3
ENSXETG00000021064	2837	399	611	535	1005	782	1102	516	598	309	828	623	682	clock
ENSXETG00000021065	906	0	0	0	0	0	0	0	0	0	0	0	0	
ENSXETG00000021067	5313	0	1	0	0	0	0	0	0	0	0	0	0	
ENSXETG00000021068	3479	333	522	427	579	410	662	877	1081	611	707	534	588	exoc1
ENSXETG00000021069	5429	85	103	76	323	231	143	378	404	228	104	66	86	cep135
ENSXETG00000021072	711	0	0	0	0	4	1	8	7	4	6	0	0	shisa3
ENSXETG00000021073	2296	2	4	2	4	4	6	27	35	16	20	16	16	bend4
ENSXETG00000021074	1695	418	603	563	552	403	864	1078	1248	704	396	296	343	slc30a9

ENSXETG00000021075	627	30	33	36	51	55	48	83	79	47	15	14	15	fgf9
ENSXETG00000021077	714	0	0	0	0	0	0	0	0	0	0	0	0	
ENSXETG00000021078	693	0	0	0	0	0	0	0	0	0	0	0	0	
ENSXETG00000021080	1366	1422	1993	1670	1569	1238	2019	1664	2067	1172	1186	896	952	tmem33
ENSXETG00000021081	2611	7	13	12	23	12	89	310	317	203	113	97	108	sgcg
ENSXETG00000021083	2453	3	4	1	44	34	71	211	231	123	94	51	69	phox2b
ENSXETG00000021084	2776	309	436	404	563	462	1462	80	103	65	345	222	308	kiaa1211
ENSXETG00000021086	1175	871	1346	1112	1844	1366	963	1860	2221	1329	2146	1508	1602	mcub
ENSXETG00000021088	3337	15	28	26	157	125	547	963	1073	608	768	478	558	limch1
ENSXETG00000021089	1126	19	29	19	304	255	58	533	664	404	562	359	399	uchl1
ENSXETG00000021090	1377	33	24	43	54	32	101	188	198	134	196	177	152	ENSXETG00000021090
ENSXETG00000021091	2748	265	354	308	317	248	517	596	820	456	395	249	319	sec24b
ENSXETG00000021093	2566	57	58	58	159	90	178	508	608	331	266	186	197	apbb2
ENSXETG00000021094	2079	10	15	6	200	190	87	64	84	51	182	142	157	nsun7
ENSXETG00000021095	420	33862	50806	44608	39273	29510	41897	39379	44858	28784	92024	63189	69203	rpl34
ENSXETG00000021097	1572	0	1	0	3	1	6	136	119	100	8	10	9	COL25A1
ENSXETG00000021098	1550	739	1124	888	3250	2528	12499	14544	16870	9549	13804	9820	10988	etnppl
ENSXETG00000021099	798	5081	7415	6216	4462	3382	4219	7237	8657	4779	3413	2388	2616	ostc
ENSXETG00000021102	1113	3	5	5	11	8	19	169	230	114	29	26	26	lef1
ENSXETG00000021105	1371	5048	7280	6072	6525	4954	8130	3307	3915	2156	5080	3604	4060	hadh
ENSXETG00000021106	2961	1030	1304	1065	1799	1289	4560	203	273	154	2170	1541	1737	cyp2u1
ENSXETG00000021109	729	1220	1754	1514	1567	1201	2991	1624	1803	1107	1353	937	1043	vta1
ENSXETG00000021112	618	1	6	7	8	6	17	0	1	0	0	0	1	gje1
ENSXETG00000021114	1455	600	819	716	1060	829	1526	5462	6399	3718	1890	1432	1579	anxa5
ENSXETG00000021115	2840	221	337	320	462	420	698	751	940	555	672	503	502	avl9
ENSXETG00000021117	4207	138	250	186	257	186	336	705	850	504	322	246	271	kbtbd2
ENSXETG00000021118	1813	561	782	649	504	428	340	787	910	531	570	414	467	exosc9
ENSXETG00000021119	2375	0	0	0	0	0	0	0	0	0	0	1	2	
ENSXETG00000021120	2681	155	202	181	213	175	416	459	605	324	343	216	288	ice2
ENSXETG00000021121	1074	390	549	466	404	303	428	673	806	431	396	259	343	rp9
ENSXETG00000021122	1277	18	15	21	144	127	178	125	161	87	57	27	31	nt5c3a
ENSXETG00000021123	1395	110	178	147	81	59	170	95	115	56	72	40	72	rora
ENSXETG00000021125	2565	43	29	27	98	67	92	192	182	113	65	55	54	bbs9
ENSXETG00000021126	725	17574	26479	22213	14142	10542	17008	10067	11385	6418	11267	7925	8657	cox5a
ENSXETG00000021129	2810	4	6	1	13	10	24	177	202	103	30	26	29	bmper
ENSXETG00000021130	2079	55	73	49	105	88	181	289	382	245	154	118	146	fam219b
ENSXETG00000021131	1848	695	997	787	948	750	1548	1558	1801	1019	864	608	675	mpi
ENSXETG00000021132	2004	297	419	358	469	388	987	1793	2075	1296	724	527	551	phkg2
ENSXETG00000021133	1422	366	562	481	312	212	485	364	429	271	446	328	378	ulk3
ENSXETG00000021134	1347	0	1	0	0	0	0	2	4	1	7	1	2	lman1l
ENSXETG00000021136	1581	0	0	0	10	12	0	1	1	0	12	14	14	cplx3
ENSXETG00000021138	7383	591	845	646	1714	1311	5883	2349	2795	1618	3125	2186	2511	igf2r
ENSXETG00000021139	2890	91	128	120	214	127	298	406	504	297	294	206	252	csk
ENSXETG00000021140	2782	30	33	27	79	59	72	151	190	94	54	34	35	cyp1a1
ENSXETG00000021142	3677	2631	3880	3441	3010	2293	3050	6216	6863	4338	2473	1771	2041	nedd4l
ENSXETG00000021145	807	0	0	0	0	0	0	2	0	0	0	1	0	ENSXETG00000021145
ENSXETG00000021146	2385	186	295	241	446	378	456	559	602	380	557	381	416	malt1
ENSXETG00000021148	3903	1	6	3	21	16	71	296	381	216	81	54	55	znf532
ENSXETG00000021149	1965	0	4	5	31	17	26	71	81	51	39	27	21	fzd3
ENSXETG00000021150	1038	1	1	1	62	46	10	15	11	9	15	8	9	fbxo16
ENSXETG00000021151	1788	271	382	318	527	450	596	567	681	424	541	357	381	elp3
ENSXETG00000021153	459	0	0	0	215	174	8	10	9	3	859	601	635	grp
ENSXETG00000021154	2247	0	0	0	0	0	0	0	0	0	0	0	0	
ENSXETG00000021156	1398	25	39	25	49	32	5	53	99	45	27	18	20	stk35
ENSXETG00000021157	486	0	0	0	0	0	0	0	0	0	0	0	0	
ENSXETG00000021158	1325	66	74	69	37	27	26	97	92	69	3	3	6	pnoc
ENSXETG00000021159	1437	1904	2861	2510	2189	1639	1637	1677	1978	1138	679	515	567	lman1
ENSXETG00000021160	2024	409	692	604	809	596	673	553	596	396	1086	751	858	znf395
ENSXETG00000021161	984	80	108	83	48	50	164	47	74	48	54	35	57	ccbe1
ENSXETG00000021168	642	0	0	0	0	0	0	0	0	0	0	0	0	
ENSXETG00000021169	573	70	83	78	171	124	207	460	488	304	239	183	163	ENSXETG00000021169
ENSXETG00000021173	4518	188	298	248	811	578	1226	1190	1368	807	1440	1055	1058	ncoa1
ENSXETG00000021174	1475	3071	4434	3929	3997	3024	3385	847	982	565	679	492	515	acat2
ENSXETG00000021175	2099	765	1173	1016	1541	1112	1417	1995	2413	1335	1834	1294	1473	wtap
ENSXETG00000021176	1123	148	186	193	160	85	127	256	290	188	63	51	51	ptrhd1
ENSXETG00000021179	3400	14	13	12	93	39	126	477	536	371	150	115	132	adcy3
ENSXETG00000021180	1298	2493	3528	3009	2630	2028	3464	1947	2398	1366	2577	1775	2004	sod2
ENSXETG00000021181	3099	2	0	1	11	14	26	39	56	30	40	19	28	pcdh9
ENSXETG00000021184	819	12	19	9	52	27	80	137	152	90	88	67	68	dnajc27
ENSXETG00000021190	1233	251	347	309	339	249	823	636	735	475	486	335	366	fbxw4
ENSXETG00000021194	1563	86	165	143	286	235	714	672	664	451	530	388	414	gpatch2
ENSXETG00000021195	1401	0	1	1	0	1	2	0	2	0	3	1	1	ENSXETG00000021195
ENSXETG00000021199	2079	174	202	188	451	341	761	1052	1232	724	679	562	583	btrc
ENSXETG00000021205	1425	0	0	0	0	0	0	0	0	0	1	1	0	
ENSXETG00000021208	2484	17	22	23	174	126	59	5232	6002	3659	1275	892	1011	ENSXETG00000021208
ENSXETG00000021211	3033	0	0	0	0	2	0	1	1	2	0	0	0	ENSXETG00000021211
ENSXETG00000021212	525	9	9	10	0	1	0	0	0	0	0	0	0	ENSXETG00000021212
ENSXETG00000021213	1380	1	2	0	2	7	11	3	6	3	18	17	19	ENSXETG00000021213
ENSXETG00000021215	1254	378	549	477	359	310	309	419	482	295	482	326	384	umps
ENSXETG00000021218	1986	0	0	0	0	2	1	1	1	0	3	1	0	grm6
ENSXETG00000021222	2345	70	105	82	158	117	192	158	228	106	160	127	157	pot1
ENSXETG00000021223	1491	0	0	0	0	0	1	0	2	0	1	0	0	
ENSXETG00000021224	2322	0	0	0	0	0	0	0	0	0	3	0	0	
ENSXETG00000021225	1413	0	1	0	1	0	0	9	10	1	3	0	3	ENSXETG00000021225
ENSXETG00000021226	7254	36	63	54	91	56	74	13	0	3	0	0	1	ENSXETG00000021226
ENSXETG00000021227	1518	4	7	11	10	4	12	10	24	11	20	13	14	tulp2
ENSXETG00000021229	2406	1691	2567	2356	2491	1884	2298	2283	2487	1585	2070	1484	1626	nucb1
ENSXETG00000021232	1743	9	26	15	39	27	84	119	142	84	68	53	66	zbtb45
ENSXETG00000021233	915	0	0	0	0	0	0	0	0	0	0	0	0	
ENSXETG00000021234	800	498	774	634	1007	743	76	337	359	250	6	3	5	MGC79725
ENSXETG00000021235	700	1334	2034	1663	2009	1472	2671	1910	2151	1329	1632	1126	1170	XB-GENE-5756459
ENSXETG00000021236	865	1278813	1841050	1593471	1044008	804132	1416132	1012467	1155942	748389	1250801	867033	972137	fth1
ENSXETG00000021237	845	437041	616081	519403	251786	190557	404113	185228	213316	139045	264592	184355	207652	MGC75752
ENSXETG00000021239	2211	1863	2682	2356	3658	2903	2160	565	628	409	1201	834	947	gys1
ENSXETG00000021241	906	17	12	20	11	9	17	0	0	0	7	5	8	ENSXETG00000021241
ENSXETG00000021242	873	2926	4261	3709	14331	10673	3185	1640	1878	1107	570	391	465	fam213b
ENSXETG00000021243	3259	1	2	2	2	4	16	89	98	51	47	50	41	mmel1
ENSXETG00000021244	1689	1	1	1	32	23	1	1	0	0	6	2	3	ttc34
ENSXETG00000021249	405	1	0	0	0	0	0	0	1	0	0	0	0	lhb
ENSXETG00000021250	711	0	0	0	2	2	1	16	14	7	4	4	5	ntf4
ENSXETG00000021253	2510	8	11	15	64	44	35	90	82	58	17	16	17	kif18b
ENSXETG00000021254	4140	24	50	38	329	215	90	213	210	123	193	164	124	ift172
ENSXETG00000021256	1778	291	405	311	449	325	439	295	369	235	401	303	303	slc25a46
ENSXETG00000021258	2030	1	5	3	9	8	8	2	6	1	3	1	13	tmem232
ENSXETG00000021259	2160	0	0	0	2	5	8	117	129	77	40	32	43	cdh8
ENSXETG00000021263	1102	37	47	34	23	18	29	282	365	167	34	18	20	ENSXETG00000021263
ENSXETG00000021265	3114	20	41	24	144	123	292	4648	5424	3276	427	344	331	itga5
ENSXETG00000021269	2631	263	371	333	641	444	557	734	867	473	638	469	477	pja2
ENSXETG00000021270	2655	14	15	9	15	7	22	109	120	74	14	4	13	fer
ENSXETG00000021272	1005	6	19	8	79	67	67	503	507	330	119	88	88	znf385a
ENSXETG00000021273	1536	79	121	92	149	123	240	281	316	188	208	149	162	fbxl17

ENSXETG00000021274	1976	153	195	152	252	197	515	543	705	372	334	222	225	gpr84
ENSXETG00000021277	1608	4471	6525	5593	11945	9638	16679	22500	25026	16470	15112	10117	11632	hnrnpa1
ENSXETG00000021279	1258	249	299	305	251	231	271	723	873	534	245	201	204	cbx5
ENSXETG00000021280	3922	143	189	153	326	262	485	537	628	371	667	447	479	smug1
ENSXETG00000021284	2610	0	5	3	48	44	21	21	21	15	27	18	20	armc2
ENSXETG00000021286	2699	1145	1648	1355	4787	3672	6918	5300	6446	3753	11713	8338	9603	sesn1
ENSXETG00000021289	1508	74	104	82	140	126	66	156	194	124	36	24	22	cep57l1
ENSXETG00000021294	1341	94	121	96	124	103	136	337	385	232	144	117	114	zbtb24
ENSXETG00000021296	1023	1	1	0	88	65	1	1	1	1	5	3	2	ppil6
ENSXETG00000021297	1097	3	5	5	29	23	3	6	5	4	6	0	2	ENSXETG00000021297
ENSXETG00000021301	1587	0	2	0	21	19	0	2	6	1	2	1	3	ak9
ENSXETG00000021302	1074	34	33	31	110	86	182	170	184	113	94	69	81	ENSXETG00000021302
ENSXETG00000021305	8187	84	112	101	620	460	266	897	1074	662	1074	785	813	nbeal2
ENSXETG00000021310	3327	73	112	104	158	137	26	222	201	120	52	35	34	cdc25a
ENSXETG00000021311	3752	712	1017	909	584	466	1063	926	1081	615	778	509	684	wasf1
ENSXETG00000021312	2757	279	391	336	541	391	686	1035	1093	706	688	504	570	cdc40
ENSXETG00000021313	2334	24	48	42	47	31	119	168	260	105	128	112	122	inpp4b
ENSXETG00000021314	2590	92	106	91	183	149	449	393	451	245	263	186	223	mettl24
ENSXETG00000021315	1026	208	286	243	362	267	746	101	129	59	339	246	288	ddo
ENSXETG00000021317	846	0	0	0	2	0	0	5	5	7	3	0	5	
ENSXETG00000021318	1707	1	1	0	3	6	14	48	63	37	53	29	41	slc22a16
ENSXETG00000021319	9697	124	180	148	558	432	434	781	962	549	87	68	59	cenpe
ENSXETG00000021320	738	986	1434	1178	1356	1098	1502	792	1036	503	799	666	734	bdh2
ENSXETG00000021321	1611	81	145	118	125	95	289	122	132	89	84	60	63	slc9b2
ENSXETG00000021322	327	145	239	181	256	225	376	397	437	269	313	214	247	cisd2
ENSXETG00000021323	1101	807	1177	914	957	738	1627	1293	1494	887	1116	828	989	ube2d3
ENSXETG00000021324	1637	111	154	133	191	127	371	264	293	192	351	276	269	cdk19
ENSXETG00000021325	2421	434	673	584	622	456	734	965	1057	598	762	554	662	nfkb1
ENSXETG00000021327	2049	68	78	55	248	191	70	704	793	460	705	467	538	ankrd13b
ENSXETG00000021328	2238	9	4	9	31	26	37	174	193	115	156	104	130	coro6
ENSXETG00000021329	1188	153	202	217	228	182	128	8	14	10	31	16	13	slc39a8
ENSXETG00000021330	1686	1	0	0	19	16	41	40	34	25	13	9	10	ENSXETG00000021330
ENSXETG00000021331	1014	1832	2579	2182	1436	1091	1507	2905	3359	2022	1705	1314	1355	amd1
ENSXETG00000021335	3761	1200	1691	1377	745	601	666	1373	1682	998	819	624	648	rpf2
ENSXETG00000021337	1353	94	170	142	238	197	687	655	795	452	398	289	348	slc16a10
ENSXETG00000021338	1554	352	507	423	616	465	846	522	682	371	536	332	430	mfsd4b
ENSXETG00000021341	1290	6	9	11	26	20	13	4	4	0	4	2	1	krt16
ENSXETG00000021342	1445	591	840	787	829	613	1097	1805	2126	1221	250	185	197	blmh
ENSXETG00000021344	3423	47	63	44	123	122	310	1261	1509	877	479	366	426	fyn
ENSXETG00000021350	7197	77	119	113	198	149	272	599	679	423	501	375	425	nf1
ENSXETG00000021353	2269	1512	2220	1829	4139	3201	5247	3936	4863	2823	7802	5489	6120	wsb1
ENSXETG00000021354	2466	109	148	128	300	249	434	602	737	407	383	267	290	ksr1
ENSXETG00000021355	1713	1956	2791	2471	4624	3499	5690	11093	12218	7814	5250	3733	4008	smarce1
ENSXETG00000021356	2910	490	728	607	795	588	1231	1316	1504	904	1183	859	845	rabep1
ENSXETG00000021358	1101	6	8	10	5	1	13	41	37	22	33	14	21	ccr7
ENSXETG00000021359	2799	500	747	613	969	760	1210	1552	1791	1050	745	554	599	nup88
ENSXETG00000021361	780	6	9	10	17	24	61	505	606	380	250	196	196	igfbp4
ENSXETG00000021362	4591	70	114	111	786	620	477	967	1096	662	147	122	115	top2a
ENSXETG00000021363	852	2708	4062	3438	2149	1625	1834	2578	2857	1795	3014	2169	2419	c1qbp
ENSXETG00000021364	3315	385	506	477	730	524	1103	984	1239	718	1215	840	976	zbtb11
ENSXETG00000021365	552	327	471	394	342	253	494	660	709	366	431	306	308	PCNP
ENSXETG00000021367	748	206	267	256	152	123	215	212	275	149	157	99	106	txnl4b
ENSXETG00000021370	1890	156	243	190	252	165	356	276	292	191	455	340	381	tmtc2
ENSXETG00000021372	2193	1	1	0	6	4	6	31	55	33	13	11	12	slc6a15
ENSXETG00000021373	4078	1	2	0	12	2	8	33	58	28	12	14	13	gria4
ENSXETG00000021375	3585	1	3	2	8	4	2	17	33	20	12	17	18	gria2
ENSXETG00000021376	1007	2	4	3	18	14	20	81	77	55	29	29	31	mpped2
ENSXETG00000021380	1501	379	550	393	835	638	1695	7265	8371	4970	2055	1441	1521	basp1
ENSXETG00000021381	1670	189	264	201	450	313	767	1239	1523	913	724	579	599	msantd4
ENSXETG00000021382	1494	446	632	584	515	380	886	849	952	576	576	389	406	katna1
ENSXETG00000021385	1738	214	319	250	254	214	262	621	716	406	239	139	179	ppil4
ENSXETG00000021386	1824	0	1	0	7	3	1	19	24	10	5	1	4	kbtbd3
ENSXETG00000021389	4560	220	316	256	631	431	821	890	1030	574	714	474	587	tab2
ENSXETG00000021391	1458	0	0	0	2	0	0	17	18	9	4	2	2	pdzd7
ENSXETG00000021393	10749	0	3	2	67	39	12	12	20	11	12	6	15	dnhd1
ENSXETG00000021397	981	0	0	0	1	0	0	0	0	0	0	1	0	alx1
ENSXETG00000021398	2172	3	7	9	24	16	28	102	139	66	77	54	78	ENSXETG00000021398
ENSXETG00000021399	2181	79	147	93	433	329	311	434	454	297	376	259	282	cstf3
ENSXETG00000021402	1510	385	548	466	710	550	986	1748	2048	1199	1152	832	914	ilk
ENSXETG00000021403	1490	22	41	44	207	150	184	781	848	470	478	345	369	sfxn3
ENSXETG00000021408	13914	2	2	4	381	318	20	2	4	1	98	54	91	dnah5
ENSXETG00000021409	261	1208	1682	1576	1440	1025	1419	2237	2500	1642	1292	977	1025	sf3b5
ENSXETG00000021410	2258	300	403	323	464	400	673	695	906	521	549	392	445	exoc8
ENSXETG00000021411	675	0	0	0	0	0	0	0	0	0	0	0	0	
ENSXETG00000021413	1614	484	718	584	1376	1074	3534	3584	3946	2469	1634	1160	1229	tpp1
ENSXETG00000021414	2702	187	275	212	269	215	553	378	502	305	594	425	440	egln1
ENSXETG00000021416	1418	105	192	121	208	142	338	255	374	203	281	180	244	sprtn
ENSXETG00000021417	1436	1	3	2	0	1	0	15	25	11	17	16	15	acta1
ENSXETG00000021421	2377	4	6	1	4	2	15	28	39	15	35	28	40	ENSXETG00000021421
ENSXETG00000021424	738	1209	1754	1633	1902	1434	4228	6197	6598	4357	2075	1421	1649	chmp4b
ENSXETG00000021425	2442	0	0	0	2	3	4	51	49	26	23	15	17	nlgn4x
ENSXETG00000021426	2535	113	177	161	159	145	155	181	247	133	225	155	163	znf341
ENSXETG00000021427	1336	443	658	562	694	506	1242	570	571	368	560	391	433	pxmp4
ENSXETG00000021428	1248	145	204	180	262	238	44	300	306	183	83	85	96	e2f1
ENSXETG00000021431	3298	335	485	413	272	231	437	639	752	483	477	326	319	cbfa2t2
ENSXETG00000021432	2037	978	1551	1271	1617	1244	2332	2100	2346	1409	1583	1085	1210	pigt
ENSXETG00000021433	2255	166	201	178	715	546	722	951	1107	672	1100	821	837	snta1
ENSXETG00000021434	5556	398	615	517	1676	1251	1540	2062	2127	1358	1588	1113	1238	gon4l
ENSXETG00000021435	1732	41	51	36	72	59	158	465	437	276	345	244	246	tox2
ENSXETG00000021436	2207	7	8	8	3	3	11	40	37	24	45	41	49	ENSXETG00000021436
ENSXETG00000021437	2472	30	40	42	38	34	60	326	457	266	187	141	143	ENSXETG00000021437
ENSXETG00000021438	4521	219	319	270	925	698	1264	1189	1356	869	1549	1098	1098	ralgapb
ENSXETG00000021439	1751	263	350	312	381	297	258	556	561	347	270	190	184	msto1
ENSXETG00000021441	1219	590	924	764	827	631	882	923	1018	628	856	621	643	dap3
ENSXETG00000021442	486	47	86	59	188	129	176	187	226	148	333	227	200	ash1l
ENSXETG00000021443	1702	9	10	5	11	11	10	8	12	6	1	3	3	nr1h5
ENSXETG00000021445	7827	46	61	76	126	63	135	560	598	370	307	219	251	chd6
ENSXETG00000021446	1074	809	1170	969	1217	889	2206	1095	1366	698	2541	1902	2026	sike1
ENSXETG00000021448	3784	949	1382	1115	2013	1521	3928	2804	3341	1920	4667	3244	3704	csde1
ENSXETG00000021449	2449	496	744	588	674	508	737	1085	1311	767	635	459	470	nras
ENSXETG00000021450	2319	5	7	10	13	4	9	8	22	9	5	5	9	ampd1
ENSXETG00000021451	2259	119	198	177	297	196	378	523	585	315	468	349	388	med26
ENSXETG00000021454	3330	1	12	6	12	9	11	77	93	50	19	20	17	dennd2c
ENSXETG00000021455	1629	2	4	3	25	24	23	19	35	27	47	29	45	tmem38a
ENSXETG00000021456	798	0	0	0	0	0	0	1	0	1	0	0	0	
ENSXETG00000021457	4181	179	273	231	497	365	661	878	1044	557	885	562	620	trim33
ENSXETG00000021460	1494	83	103	97	168	107	265	269	324	208	353	253	295	srek1
ENSXETG00000021463	3948	22	44	32	130	100	256	272	268	185	247	160	208	mast4
ENSXETG00000021466	2003	1	1	5	7	9	3	15	8	2	44	29	35	ENSXETG00000021466
ENSXETG00000021467	4960	184	267	258	515	367	713	814	946	568	741	520	602	nisch
ENSXETG00000021468	2784	264	362	307	665	525	890	1375	1654	929	1233	789	978	ppp1r12a

ENSXETG00000021470	1514	9077	13581	11371	13291	10180	11353	7758	8373	5217	7435	5241	5602	uqcrc1
ENSXETG00000021472	3109	138	172	156	247	224	362	211	283	161	305	233	282	usp15
ENSXETG00000021474	1059	75	83	94	397	308	547	727	767	524	824	576	663	arhgef15
ENSXETG00000021479	1555	1	1	1	14	5	4	3	4	1	16	10	16	slc16a7
ENSXETG00000021480	2169	1134	1718	1469	1321	963	5763	2755	3168	2008	3221	2273	2562	pik3r1
ENSXETG00000021481	3872	21	33	27	248	180	257	294	389	214	427	292	289	lrig3
ENSXETG00000021492	2328	1	1	3	0	0	1	12	7	5	2	0	0	lrfn5
ENSXETG00000021497	2807	34	48	42	324	239	220	493	580	319	413	264	336	epb41l4a
ENSXETG00000021498	1941	270	367	349	369	301	671	848	1033	592	656	457	479	c18orf25
ENSXETG00000021499	1302	195	276	254	262	178	251	431	482	288	335	230	276	fdx1l
ENSXETG00000021500	993	106	155	109	200	152	60	257	287	166	56	39	28	haus1
ENSXETG00000021501	8696	240	344	276	841	681	1635	1585	1989	1183	1337	894	1024	apc
ENSXETG00000021502	1401	10796	15858	13209	21051	15555	18319	9403	10809	6563	11714	8348	8841	atp5a1
ENSXETG00000021508	2839	139	182	155	1456	1087	1973	3812	4322	2523	17566	12298	13691	eef2.2
ENSXETG00000021513	1038	1788	2409	2037	2541	1884	2334	4848	5440	3512	6575	4466	5227	reep5
ENSXETG00000021514	1912	124	201	127	315	277	653	831	780	530	555	440	478	dcp2
ENSXETG00000021515	5589	135	207	181	483	372	446	898	994	667	491	322	328	mcc
ENSXETG00000021517	4404	289	395	294	636	427	994	806	1004	537	853	623	687	ythdc2
ENSXETG00000021518	2060	2	4	1	19	14	32	38	34	18	28	16	24	kcnn2
ENSXETG00000021519	2221	23	38	48	84	68	86	220	276	167	141	98	120	pias2
ENSXETG00000021523	615	1051	1399	1240	900	699	598	1517	1792	1210	588	415	404	ier3ip1
ENSXETG00000021528	883	908	1285	1146	1169	967	1858	2119	2254	1425	2099	1397	1516	ddrgk1
ENSXETG00000021529	4449	3	6	5	11	7	10	316	346	193	76	54	41	siglec1
ENSXETG00000021530	1481	642	914	780	964	710	1052	967	1186	724	1284	831	1018	tex261
ENSXETG00000021531	4253	25	29	20	176	124	458	2355	2682	1530	546	396	470	antxr2
ENSXETG00000021532	1676	0	0	0	6	5	5	33	42	29	18	14	18	atp6v1b1
ENSXETG00000021533	2652	0	3	1	10	14	16	37	40	19	18	24	26	prdm8
ENSXETG00000021534	738	1	1	0	0	0	0	0	0	0	1	0	0	fgf5
ENSXETG00000021538	1068	4	8	6	37	25	105	197	257	119	122	80	94	bmp3
ENSXETG00000021539	2313	13	26	26	10	10	20	2	6	3	4	5	6	prkg2
ENSXETG00000021541	1041	392	590	533	594	460	1144	827	913	584	855	585	633	nagk
ENSXETG00000021542	2313	147	184	165	355	274	471	677	744	455	585	448	485	exoc6b
ENSXETG00000021546	5834	2601	3913	3285	1114	814	1673	1215	1473	861	1285	909	989	cyp26b1
ENSXETG00000021547	5305	964	1311	1068	511	428	846	106	142	76	379	287	367	usp2
ENSXETG00000021550	1191	267	373	360	244	229	352	319	349	228	341	223	261	rmdn3
ENSXETG00000021551	1353	5637	8341	7056	5397	4025	5518	419	545	308	3132	2305	2531	treh
ENSXETG00000021554	2187	788	1123	985	729	516	643	759	877	532	480	277	349	gchfr
ENSXETG00000021555	1458	657	1039	821	1176	884	1377	2456	2811	1694	1343	975	1041	spint1
ENSXETG00000021556	4231	365	513	438	574	499	739	2864	3114	1864	1248	886	978	phldb1
ENSXETG00000021559	968	165	222	196	512	368	982	498	629	409	605	404	460	dnajc17
ENSXETG00000021560	432	15	17	9	34	17	21	21	28	20	34	15	19	h2afx
ENSXETG00000021561	420	3	3	5	6	2	3	9	9	4	1	3	2	ENSXETG00000021561
ENSXETG00000021564	2775	281	413	343	444	303	674	736	910	480	376	288	327	zfyve19
ENSXETG00000021565	1277	0	0	0	0	0	1	0	0	2	0	0	0	foxr1
ENSXETG00000021566	2129	1	9	2	18	20	17	9	12	7	64	52	41	ttc36
ENSXETG00000021567	2381	685	972	875	821	630	1244	1604	1752	1049	870	604	644	ift46
ENSXETG00000021568	2273	2356	3464	3065	2957	2134	2718	3585	4292	2483	1764	1266	1395	arcn1
ENSXETG00000021572	3482	2	2	5	29	30	8	67	67	41	46	25	40	rhov
ENSXETG00000021574	1227	205	318	216	260	177	328	334	437	235	193	124	151	dpagt1
ENSXETG00000021575	3430	265	418	346	598	478	1128	943	1153	638	582	438	518	vps18
ENSXETG00000021576	3654	56	82	61	232	152	199	342	449	244	455	317	373	c2cd2l
ENSXETG00000021579	3390	210	270	231	567	461	842	1693	1998	1122	1012	705	790	sbno2
ENSXETG00000021580	1627	0	0	0	0	1	6	5	8	7	5	4	5	
ENSXETG00000021581	1872	0	1	1	1	1	2	7	2	4	3	4	4	ABCG4
ENSXETG00000021584	2058	213	299	261	942	770	1790	3193	3788	2235	879	638	714	dll4
ENSXETG00000021585	3228	7	3	2	22	15	20	8	8	8	30	26	25	baiap3
ENSXETG00000021586	2166	86	97	90	123	81	161	948	1070	646	154	116	125	nlrx1
ENSXETG00000021587	1248	1362	1998	1768	1953	1479	3457	787	848	561	1595	1129	1203	pdzd3
ENSXETG00000021588	5710	701	1029	897	1675	1287	3206	5106	5904	3424	2749	1932	2099	cbl
ENSXETG00000021589	1698	410	623	511	473	361	460	480	593	332	632	429	495	tsr3
ENSXETG00000021590	1976	852	1285	1012	1185	944	2131	1151	1327	725	1940	1391	1557	gnptg
ENSXETG00000021591	1695	110	154	149	527	400	815	3370	3816	2300	1327	934	1069	mcam
ENSXETG00000021592	4725	144	217	175	478	345	496	861	933	596	462	324	351	camsap1
ENSXETG00000021595	750	0	1	1	0	0	1	135	152	92	10	4	6	c1qtnf5
ENSXETG00000021596	1005	37	62	48	286	252	549	577	701	398	690	474	558	gpr146
ENSXETG00000021597	1155	0	3	0	7	4	9	28	21	11	26	7	18	gper1
ENSXETG00000021599	2554	7	19	14	111	119	146	115	143	96	270	209	235	unkl
ENSXETG00000021600	3578	917	1358	1099	1325	988	1760	1511	1744	1058	1185	835	1018	tmem259
ENSXETG00000021601	591	28	45	47	190	172	327	72	87	63	51	42	64	CHAC1
ENSXETG00000021602	1308	0	0	0	2	3	1	3	7	6	1	0	3	
ENSXETG00000021603	2192	0	0	1	1	1	13	51	54	30	13	11	8	thy1
ENSXETG00000021606	1518	1	1	1	4	1	14	1	0	0	4	0	4	cyp2w1
ENSXETG00000021607	3006	0	0	0	0	1	0	5	3	5	1	0	0	grin3b
ENSXETG00000021608	1687	182	293	234	233	160	184	427	446	289	132	100	96	chek2
ENSXETG00000021611	2532	425	655	560	759	574	699	970	1069	738	998	749	807	nom1
ENSXETG00000021612	1909	561	730	682	547	458	537	1128	1273	758	455	359	372	wdr18
ENSXETG00000021613	2353	129	172	149	243	172	377	128	116	90	444	331	349	arid3a
ENSXETG00000021614	1080	5	8	3	52	38	7	8	10	6	10	10	11	lrrc74a
ENSXETG00000021615	969	0	0	0	0	0	0	2	0	0	0	0	0	mnx1
ENSXETG00000021616	2293	7	13	10	36	21	15	83	69	48	56	31	44	slc7a4
ENSXETG00000021618	1491	1	1	1	0	0	3	7	5	1	7	4	5	kiss1r
ENSXETG00000021620	3349	408	636	544	882	686	1244	1544	1775	1115	641	482	513	ube3c
ENSXETG00000021621	1610	143	237	228	347	269	465	574	721	434	597	378	477	ubac1
ENSXETG00000021624	1018	888	1349	1129	798	594	1065	2794	3191	2009	1043	721	791	dnajb6
ENSXETG00000021626	1365	12	21	11	13	15	28	191	180	118	78	58	62	BLK
ENSXETG00000021628	1410	0	3	0	6	1	5	3	2	3	12	9	12	ENSXETG00000021628
ENSXETG00000021630	264	831	1263	1108	1162	800	913	2113	2316	1440	959	671	773	snrpf
ENSXETG00000021631	1374	0	0	0	0	0	0	0	0	0	0	0	0	ccdc38
ENSXETG00000021633	6127	1216	1706	1395	1256	1002	2206	2307	2892	1637	1396	987	1204	XB-GENE-5947997
ENSXETG00000021635	3209	0	0	0	11	4	20	32	34	12	20	19	12	clvs1
ENSXETG00000021636	1626	2	2	0	2	0	4	413	500	251	6	6	6	amdhd1
ENSXETG00000021638	2069	1832	2600	2314	1892	1402	2939	2632	3177	1884	2074	1369	1590	get4
ENSXETG00000021641	3694	240	330	298	367	317	479	837	886	533	287	197	228	med16
ENSXETG00000021642	3530	874	1314	1151	1351	1072	1799	1807	2212	1184	2359	1659	1879	sun1
ENSXETG00000021643	2520	97	105	105	155	121	144	232	244	162	159	106	117	dnaaf5
ENSXETG00000021644	2196	1106	1540	1397	1658	1276	1763	2579	2975	1748	1717	1136	1322	ASPH
ENSXETG00000021645	2331	13	25	15	15	22	7	105	108	65	11	8	14	hal.2
ENSXETG00000021646	1452	614	937	744	1473	1113	2611	3606	4189	2534	3249	2313	2549	cfd
ENSXETG00000021647	1680	11	39	10	62	48	169	1495	1741	1041	150	88	96	prtn3
ENSXETG00000021651	1226	0	0	2	2	3	1	35	47	27	12	17	19	nkain3
ENSXETG00000021652	2043	0	1	0	0	0	1	0	0	0	2	1	0	mcmdc2
ENSXETG00000021653	2707	0	2	1	4	4	7	41	36	26	7	5	8	plppr3
ENSXETG00000021654	1358	1294	1703	1389	911	702	1525	1446	1918	1081	1129	795	859	ggh
ENSXETG00000021655	1026	78	112	93	236	197	378	1265	1381	810	522	343	434	mbnl1
ENSXETG00000021656	2263	13	20	22	30	20	7	16	33	19	26	19	15	ttpa
ENSXETG00000021658	1408	90	126	109	112	48	125	302	342	184	176	134	146	dgcr6
ENSXETG00000021659	2542	512	773	710	774	595	1335	1555	1842	1034	805	550	611	ythdf3
ENSXETG00000021660	2448	0	3	4	30	30	102	468	594	354	161	111	123	scarf2
ENSXETG00000021661	1789	1952	2869	2316	4457	3339	2989	3974	4862	2728	3107	2321	2651	ptbp1
ENSXETG00000021662	2001	245	275	279	356	293	437	709	875	488	349	234	300	tmem5
ENSXETG00000021663	3156	72	113	90	165	92	114	476	567	325	383	305	350	srgap1

ENSXETG00000021664	1854	1	1	1	23	26	13	15	9	11	231	150	176	prodh
ENSXETG00000021666	3386	241	313	267	450	306	821	612	717	442	581	437	541	c12orf66
ENSXETG00000021667	718	31212	46325	38412	48406	36276	50675	56314	62991	35290	114919	78815	86197	rpl18a
ENSXETG00000021668	2889	642	896	774	518	412	733	991	1308	659	647	474	555	xpot
ENSXETG00000021669	2030	1116	1573	1345	813	626	2011	1091	1248	637	1192	834	930	nck2
ENSXETG00000021670	1755	0	1	0	0	0	0	0	0	0	0	0	0	c2orf40
ENSXETG00000021671	1095	264	359	360	413	302	704	942	1117	594	710	476	557	uxs1
ENSXETG00000021674	1548	0	0	0	0	0	0	0	2	2	0	0	0	tfap2b
ENSXETG00000021681	11574	3	2	0	2	0	0	0	2	2	1	1	1	
ENSXETG00000021683	3209	216	322	255	320	219	439	574	708	393	401	273	308	nedd1
ENSXETG00000021688	2036	83	95	71	106	88	189	247	248	146	209	158	166	arhgap22
ENSXETG00000021690	414	0	0	0	0	0	0	1	0	0	0	0	0	
ENSXETG00000021691	1402	584	872	731	820	610	1269	1784	1958	1144	1019	721	851	mapk8
ENSXETG00000021692	546	530	778	622	312	223	531	430	522	322	363	249	305	arl5a
ENSXETG00000021693	3573	30	25	19	38	33	20	7	12	5	12	8	3	c12orf63
ENSXETG00000021694	1536	0	0	0	1	0	1	17	7	8	1	2	0	cacnb4
ENSXETG00000021699	453	2	1	3	0	0	1	0	2	0	1	0	0	il17f
ENSXETG00000021700	342	2	8	5	3	1	19	3	3	0	2	0	0	ENSXETG00000021700
ENSXETG00000021701	3107	537	840	693	964	735	143	1262	1394	821	297	207	236	XB-GENE-971646
ENSXETG00000021702	1587	741	1131	921	1928	1542	3962	3503	3946	2429	2288	1586	1848	stam2
ENSXETG00000021703	3063	39	58	58	125	113	224	973	1156	637	497	365	455	fmnl2
ENSXETG00000021705	5667	1	2	1	79	71	3	0	1	1	8	3	6	ENSXETG00000021705
ENSXETG00000021709	3891	911	1327	1124	1669	1265	3343	3132	3760	2277	1799	1284	1435	mbtps1
ENSXETG00000021711	2331	0	0	0	0	0	0	0	0	0	0	0	0	
ENSXETG00000021713	3100	1261	1892	1584	2427	1867	2578	3685	4063	2458	2962	2003	2286	prpf40a
ENSXETG00000021714	1673	39	49	64	254	215	268	727	757	537	437	302	323	rtkn
ENSXETG00000021715	312	0	0	1	1	1	6	162	178	117	18	16	10	rprm
ENSXETG00000021716	2201	0	3	0	4	10	8	59	62	40	33	17	13	galnt13
ENSXETG00000021717	2535	0	1	3	12	6	12	14	15	12	28	19	23	kcnj3
ENSXETG00000021718	1333	265	442	307	349	253	334	1173	1250	714	459	333	295	orc4
ENSXETG00000021720	2317	95	149	112	193	131	358	260	295	205	331	267	294	acvr2a
ENSXETG00000021721	1605	40	68	45	134	124	1527	3215	3823	2148	617	399	504	renbp
ENSXETG00000021723	1029	292	352	339	476	370	595	304	376	235	364	251	302	nudt16
ENSXETG00000021724	1660	2554	3650	3053	1394	1086	1417	3239	3849	2176	1293	856	895	naa10
ENSXETG00000021725	2628	369	554	486	839	655	996	661	839	498	970	688	800	arhgap4
ENSXETG00000021728	1535	0	0	0	0	0	1	0	0	0	0	0	0	cav3.2
ENSXETG00000021730	4896	6	5	5	158	117	169	417	524	324	168	96	100	pappa
ENSXETG00000021731	2416	595	910	753	589	486	744	2374	2511	1587	1181	835	875	epha2
ENSXETG00000021737	2673	0	3	2	7	9	16	51	50	27	72	57	39	astn2
ENSXETG00000021740	3067	122	169	161	293	226	686	664	804	453	402	269	324	c16orf62
ENSXETG00000021741	3062	190	244	225	245	212	368	437	562	333	399	261	289	trim32
ENSXETG00000021745	1362	9	9	9	38	21	22	72	92	53	78	65	54	ENSXETG00000021745
ENSXETG00000021746	1054	172	257	215	161	117	323	252	334	176	85	59	92	gde1
ENSXETG00000021747	2481	136	184	132	235	164	474	229	300	169	314	215	231	brat1
ENSXETG00000021748	2264	223	277	256	155	111	239	179	222	140	159	120	139	kdm8
ENSXETG00000021749	1647	0	0	0	0	0	0	0	0	0	0	0	0	
ENSXETG00000021751	3366	0	0	0	0	1	0	0	0	1	2	1	0	ENSXETG00000021751
ENSXETG00000021752	4164	213	305	240	531	404	672	728	818	510	1123	798	944	met
ENSXETG00000021754	1473	5	14	8	18	13	21	27	18	16	9	16	18	zbtb34
ENSXETG00000021760	960	2285	3259	2675	2500	1889	3618	4006	4872	2728	2108	1524	1731	capza2
ENSXETG00000021761	1722	26	26	32	165	120	202	143	179	97	68	65	74	iqce
ENSXETG00000021762	2836	11	22	14	177	157	438	6829	7541	4817	1228	877	958	angptl2
ENSXETG00000021763	2343	154	256	205	457	351	993	2989	3502	2145	1140	769	868	ttyh3
ENSXETG00000021764	1781	272	351	282	354	283	342	333	382	192	394	284	314	st7
ENSXETG00000021768	1375	27	46	45	214	186	171	1044	1139	689	141	119	118	lfng
ENSXETG00000021769	2430	15	27	21	51	31	47	28	39	25	15	20	13	npas2
ENSXETG00000021772	1764	98	138	116	272	201	407	426	449	283	619	441	496	ralgps1
ENSXETG00000021774	2073	2736	3767	3243	2714	2144	2432	5250	6220	3464	1909	1323	1561	ptges3
ENSXETG00000021779	2064	1350	1884	1684	809	650	975	1831	2122	1238	765	577	645	pdcl3
ENSXETG00000021782	5303	146	197	180	554	454	804	1447	1758	1028	1177	816	903	baz2a
ENSXETG00000021783	2455	0	0	0	0	0	0	0	0	0	0	0	0	ankrd1
ENSXETG00000021785	807	256	390	276	284	231	292	542	705	415	445	318	354	rpp30
ENSXETG00000021786	924	1	1	2	4	1	2	3	9	3	5	2	2	ENSXETG00000021786
ENSXETG00000021787	1095	21	29	21	97	87	104	260	304	194	109	92	109	rbms2
ENSXETG00000021788	1046	0	0	0	0	0	0	0	0	0	0	0	0	
ENSXETG00000021789	1095	0	0	0	0	0	0	0	0	0	0	0	0	
ENSXETG00000021791	1524	2	2	2	1	2	1	2	0	0	1	1	1	asz1
ENSXETG00000021796	4458	222	350	279	368	314	844	47	64	40	405	260	309	cftr
ENSXETG00000021798	1620	1	2	1	16	15	4	28	29	16	12	4	5	gls2
ENSXETG00000021801	777	1	0	0	0	0	0	0	0	0	0	0	0	
ENSXETG00000021803	642	392	546	443	355	287	463	414	428	241	597	427	420	spryd4
ENSXETG00000021804	1297	0	0	0	0	0	0	0	0	0	0	0	0	
ENSXETG00000021807	2773	387	540	404	215	181	252	350	406	237	498	333	356	cdkl5
ENSXETG00000021810	921	2483	3261	2607	2900	2129	5667	432	564	298	730	515	584	ENSXETG00000021810
ENSXETG00000021811	2892	44	38	38	73	64	138	253	281	196	150	106	111	map3k9
ENSXETG00000021812	1637	135	196	161	165	173	387	1059	1218	746	433	317	350	pdk3
ENSXETG00000021813	4927	228	358	267	780	569	2098	951	1186	625	1870	1256	1512	cttnbp2
ENSXETG00000021814	1410	40	60	53	14	12	73	99	114	64	0	0	2	slc16a12
ENSXETG00000021815	6935	544	782	698	1233	927	2461	3420	4018	2370	2266	1681	1870	pcnx1
ENSXETG00000021816	3664	308	416	387	928	765	667	1027	1283	706	782	554	628	timeless
ENSXETG00000021818	1419	2	14	6	16	5	26	66	69	49	11	11	13	ifit5
ENSXETG00000021819	1338	0	0	0	1	1	6	2	1	1	1	0	2	ENSXETG00000021819
ENSXETG00000021820	1110	0	1	0	0	0	3	7	11	6	4	1	3	pcyt1b
ENSXETG00000021822	1470	6	5	2	20	12	42	161	257	124	106	65	79	ifit1
ENSXETG00000021824	4453	138	214	173	726	575	250	841	997	552	300	211	224	pola1
ENSXETG00000021825	5463	243	305	311	683	519	867	1391	1724	986	832	584	658	sipa1l1
ENSXETG00000021829	294	241	345	304	345	254	296	520	670	397	232	180	183	lsm8
ENSXETG00000021830	5482	738	1046	911	1685	1267	3263	1349	1673	1025	1617	1093	1210	stat2
ENSXETG00000021831	5641	87	125	109	205	161	365	445	539	321	375	277	300	ankrd26
ENSXETG00000021832	1503	4	13	14	55	53	64	120	103	81	73	72	73	arx
ENSXETG00000021836	1299	10	13	12	30	28	38	87	95	46	70	52	49	rgs6
ENSXETG00000021839	2808	2	1	1	8	10	17	685	865	459	61	44	50	adamts15
ENSXETG00000021840	1644	94	91	90	144	120	186	167	225	139	256	190	198	rnf217
ENSXETG00000021842	630	44	53	32	22	15	43	41	58	33	44	44	39	tpd52l1
ENSXETG00000021843	726	322	451	375	253	236	315	458	506	304	116	88	99	hddc2
ENSXETG00000021844	960	4	11	6	6	10	22	111	113	60	16	14	18	hey2
ENSXETG00000021845	1101	0	0	0	0	2	0	0	0	0	0	0	0	
ENSXETG00000021846	1263	109	138	143	71	54	99	215	264	159	111	70	88	dpf3
ENSXETG00000021847	2819	38	50	49	78	70	177	11975	15145	8437	1008	727	773	dcn
ENSXETG00000021849	1308	68	117	95	148	112	197	157	187	123	306	199	230	dcaf4
ENSXETG00000021850	988	130	135	115	142	138	162	384	447	252	190	109	143	pym1
ENSXETG00000021851	1544	5	14	7	18	24	27	800	891	585	15	10	16	mmp19
ENSXETG00000021852	2352	401	656	522	1107	807	1743	1317	1520	911	881	683	723	zfyve1
ENSXETG00000021853	3116	9	12	8	43	13	48	460	493	311	66	42	50	tmem198
ENSXETG00000021854	1686	182	278	232	373	324	519	675	820	455	520	403	408	dnajc14
ENSXETG00000021855	4623	3630	5162	4351	4912	3739	4839	3627	4331	2574	5896	4141	4724	ATP2B1
ENSXETG00000021857	3351	1746	2417	2094	2800	2253	2834	2979	3390	2072	6505	4646	5273	rbm25
ENSXETG00000021858	5100	136	175	149	370	271	302	795	923	531	656	453	521	snx19
ENSXETG00000021860	2549	12441	18029	15491	18767	13931	13151	24196	27815	16293	12366	8606	9874	tubb4a
ENSXETG00000021861	1029	1	0	0	0	0	3	4	5	1	9	6	2	gdf11.1
ENSXETG00000021862	2136	784	1227	1034	2266	1816	2204	3519	3826	2396	2261	1592	1755	khsrp

ENSXETG00000021865	1638	68	101	72	92	89	231	139	186	115	83	60	81 poc1b
ENSXETG00000021867	1324	507	787	650	909	695	620	1125	1233	773	539	423	424 mri1
ENSXETG00000021868	2196	772	1132	954	1378	1095	1262	2184	2469	1628	1084	765	798 cars
ENSXETG00000021870	3303	95	144	135	374	264	731	908	975	637	612	426	439 zswim4
ENSXETG00000021871	2574	1685	2497	2062	2683	1964	3291	2044	2483	1446	2820	1991	2244 dusp6
ENSXETG00000021873	1054	216	311	273	480	374	208	505	705	386	645	429	476 acer1
ENSXETG00000021874	447	0	0	0	3	1	0	0	0	0	1	0	0
ENSXETG00000021875	1721	83	117	105	283	217	381	700	756	461	557	375	424 mllt1
ENSXETG00000021876	2327	553	797	690	1391	1032	1066	1376	1603	979	3125	2255	2527 acsbg2
ENSXETG00000021877	3653	120	151	120	648	465	589	700	868	528	850	571	631 rfx2
ENSXETG00000021879	1689	3	4	4	27	11	6	34	52	12	33	33	33 slc1a6
ENSXETG00000021881	1670	116	157	154	97	80	164	92	105	66	149	90	114 caps
ENSXETG00000021884	1912	24	26	15	141	83	141	181	228	110	272	224	254 vmac
ENSXETG00000021885	527	1825	2695	2388	2143	1568	2237	1736	2142	1397	1043	778	846 ndufa11
ENSXETG00000021887	3239	0	0	0	0	0	0	0	0	0	0	0	0
ENSXETG00000021888	2655	696	1086	911	818	627	886	1221	1375	825	786	609	624 lonp1
ENSXETG00000021890	1010	59	63	52	114	70	139	417	488	279	120	101	110 rab11b.2
ENSXETG00000021892	1567	139	176	141	213	169	349	660	768	471	239	166	193 march2
ENSXETG00000021893	1548	1567	2375	1985	1246	953	842	1487	1757	1033	769	506	607 angptl4
ENSXETG00000021895	4270	465	653	514	496	381	1192	1629	1859	1055	743	585	597 kank3
ENSXETG00000021896	1365	3291	4479	3851	5630	4070	5472	2110	2659	1498	1336	936	1064 ugt2b4
ENSXETG00000021897	660	3	0	0	2	2	0	16	18	8	4	1	3 ENSXETG00000021897
ENSXETG00000021898	2361	2	5	4	32	13	31	281	285	143	74	67	73 ENSXETG00000021898
ENSXETG00000021905	361	22302	33159	28149	29550	22115	31316	26745	32125	18404	64956	45923	48983 rps28p9
ENSXETG00000021906	933	0	0	0	0	0	0	0	0	0	0	0	0
ENSXETG00000021908	3448	445	629	490	1068	853	1052	1323	1581	907	1753	1166	1381 kat7
ENSXETG00000021909	2580	0	0	0	0	0	0	0	0	0	0	0	0
ENSXETG00000021913	765	0	0	0	0	0	0	0	0	0	0	0	0
ENSXETG00000021918	1539	1	4	2	8	2	5	38	46	33	12	9	16 RAB11FIP4
ENSXETG00000021919	525	0	0	2	3	0	2	5	4	0	12	10	18 dlx3
ENSXETG00000021920	3132	1052	1572	1297	3590	2725	5434	4449	5010	3051	4281	2968	3392 itga3
ENSXETG00000021921	450	74	128	103	118	91	115	195	209	144	80	59	55 ENSXETG00000021921
ENSXETG00000021922	2223	507	782	661	736	578	1047	1581	1881	1121	1408	1050	1087 pfkp
ENSXETG00000021928	972	0	0	0	0	0	0	0	0	0	0	0	0
ENSXETG00000021930	1299	0	0	1	0	0	1	0	0	0	1	0	0
ENSXETG00000021931	1118	46	86	78	112	65	182	131	189	94	231	174	196 rprd1b
ENSXETG00000021932	963	146	171	139	175	133	408	244	348	215	199	128	182 c18orf21
ENSXETG00000021933	2394	6	5	11	42	43	65	221	173	135	170	103	124 ENSXETG00000021933
ENSXETG00000021938	4723	637	983	880	2213	1672	1986	2378	2505	1794	2762	1943	2101 cxxc1
ENSXETG00000021946	1470	3	2	2	2	0	4	198	211	89	93	78	75 ENSXETG00000021946
ENSXETG00000021947	1617	39	52	49	155	109	163	76	98	61	108	91	108 scrn2
ENSXETG00000021948	2624	59	107	89	85	77	33	307	376	238	44	19	35 chtf8
ENSXETG00000021949	1050	1	0	1	8	4	4	10	13	8	4	0	3 sp6
ENSXETG00000021952	2472	267	468	381	1003	722	1160	1189	1308	760	1126	848	882 sp2
ENSXETG00000021953	1190	854	1236	1104	914	756	1662	1061	1196	700	782	558	635 nudt5
ENSXETG00000021954	2718	428	603	529	992	785	1617	384	521	262	792	570	616 dhtkd1
ENSXETG00000021955	1645	4349	6319	5516	3376	2418	5039	1819	2070	1313	1557	1189	1232 pnpo
ENSXETG00000021957	678	0	8	6	4	5	10	34	36	39	20	17	25 copz2
ENSXETG00000021958	2218	1350	1926	1619	2463	1875	3283	3531	3960	2468	2336	1575	1801 nfe2l1
ENSXETG00000021959	5865	22	39	30	114	92	139	634	768	404	428	329	342 abca8
ENSXETG00000021961	3624	1375	1839	1611	3209	2405	3920	1090	1268	756	2991	2200	2424 aldh5a1
ENSXETG00000021962	1459	142	250	207	279	216	272	1443	1633	1041	581	384	428 cbx1
ENSXETG00000021963	504	29	41	32	56	44	62	112	116	58	87	60	62 snx11
ENSXETG00000021964	915	0	0	0	0	0	0	2	1	0	0	0	0
ENSXETG00000021967	1952	6	5	5	57	47	88	311	377	219	188	127	146 hoxb3
ENSXETG00000021968	2499	1	0	0	0	1	2	2	0	0	1	0	0 gpld1
ENSXETG00000021972	5436	7	6	18	132	85	390	1801	1997	1252	992	625	723 col27a1
ENSXETG00000021973	1382	0	1	2	16	21	21	53	60	33	66	55	58 hoxb4
ENSXETG00000021974	4068	1	0	0	9	9	1	27	20	13	57	36	35 abca10
ENSXETG00000021978	1407	4	10	2	49	42	67	246	264	192	120	70	67 hoxb5
ENSXETG00000021985	1131	71	99	92	51	46	153	152	161	117	142	113	107 mrs2
ENSXETG00000021987	1480	17	35	31	80	63	197	503	608	347	171	119	116 hoxb6
ENSXETG00000021988	1455	7	10	6	65	58	42	14	10	6	165	118	126 dcdc2
ENSXETG00000021989	2739	39	39	58	59	46	150	994	1081	647	232	178	174 st6galnac4
ENSXETG00000021990	3356	16	29	20	81	62	61	200	191	127	160	117	129 nrsn1
ENSXETG00000021991	978	0	0	0	0	0	0	0	0	0	0	0	0
ENSXETG00000021992	2722	6	13	14	19	10	37	150	173	98	66	36	62 celf2
ENSXETG00000021993	732	8	9	6	7	12	55	141	161	101	50	36	31 hoxb8
ENSXETG00000021994	930	0	6	3	42	20	14	27	22	9	14	13	14 pip5kl1
ENSXETG00000021997	942	252	355	324	317	240	224	1022	1125	718	1089	737	862 st6galnac6
ENSXETG00000021999	2269	492	757	705	1202	914	710	373	398	233	737	488	518 ak1
ENSXETG00000022002	330	4	2	2	1	0	1	4	0	0	1	1	3 ENSXETG00000022002
ENSXETG00000022003	2568	72	102	82	245	221	155	183	207	140	89	53	49 TTF2
ENSXETG00000022007	2331	664	1056	860	1224	921	1485	651	713	447	1761	1232	1435 fpgs
ENSXETG00000022008	2547	33	53	45	169	161	241	528	609	392	284	201	242 sh2d3c
ENSXETG00000022009	3977	443	626	568	634	553	809	1224	1620	787	788	592	627 eaf1
ENSXETG00000022010	1859	464	721	591	510	417	727	2575	3019	1734	1057	839	961 mturn
ENSXETG00000022012	2838	9	33	22	40	29	82	100	131	71	88	66	64 nod1
ENSXETG00000022013	1198	7	4	10	146	110	27	38	39	39	8	5	10 efcab1
ENSXETG00000022014	1989	549	813	679	287	225	427	265	346	233	179	141	158 ggct
ENSXETG00000022015	1587	0	0	0	2	2	0	17	23	13	3	2	2 entpd3
ENSXETG00000022016	1275	124	153	110	278	176	97	58	63	34	51	42	49 ribc1
ENSXETG00000022017	2652	92	151	135	162	127	741	850	904	503	637	395	485 alas2
ENSXETG00000022018	414	32157	47349	41291	37040	27189	41366	39107	46197	26732	77954	53754	59978 rpl14
ENSXETG00000022019	2593	0	0	0	1	0	0	0	0	0	0	0	0
ENSXETG00000022020	945	0	0	0	7	8	19	202	231	125	32	17	17 fam198a
ENSXETG00000022021	2608	54	63	63	66	68	61	113	114	45	163	109	129 pomgnt2
ENSXETG00000022022	5419	155	255	209	659	472	747	1143	1414	807	1035	720	834 snrk
ENSXETG00000022024	1845	152	239	218	307	240	398	688	849	526	363	266	335 ano10
ENSXETG00000022025	2016	237	338	298	527	413	735	536	551	306	309	242	294 abhd5
ENSXETG00000022026	1554	73	133	112	200	164	108	156	185	121	156	116	118 apex2
ENSXETG00000022029	1413	1616	2385	2062	4076	3085	3097	878	989	618	2738	1995	2150 pfkfb1
ENSXETG00000022030	732	94	146	89	0	0	0	115	130	75	0	0	0 ENSXETG00000022030
ENSXETG00000022031	708	393	604	469	597	465	285	626	714	471	494	327	396 nsmce3
ENSXETG00000022032	3280	255	363	347	810	590	915	1338	1537	899	1190	831	990 phf8
ENSXETG00000022035	4934	829	1142	1095	2499	1879	612	1800	2075	1294	943	610	682 dnmt1
ENSXETG00000022040	4789	1043	1427	1146	1483	1237	4724	1580	2040	1192	2046	1501	1678 slc30a4
ENSXETG00000022042	2094	489	788	603	1650	1209	1713	1969	2208	1370	2424	1724	1864 c19orf66
ENSXETG00000022043	951	524	756	666	954	732	1549	351	419	252	1143	825	923 rdh8
ENSXETG00000022045	1237	373	591	480	369	303	904	590	740	450	435	297	335 bloc1s6
ENSXETG00000022046	1532	6382	9373	7846	4205	3182	7801	3855	4674	2733	2853	1948	2273 sqrdl
ENSXETG00000022049	970	1938	2872	2496	558	433	507	1556	1890	1149	315	209	245 nmrk2
ENSXETG00000022051	1135	243	316	254	170	138	233	198	251	171	127	91	94 cdc42se2
ENSXETG00000022052	1611	1217	1795	1513	1559	1190	2672	1583	1884	1160	1560	1046	1236 sppl2a
ENSXETG00000022054	5281	1111	1720	1444	2264	1685	3174	2912	3412	2102	2370	1653	1943 lclat1
ENSXETG00000022055	1896	1119	1645	1416	999	759	664	1360	1530	897	1334	981	1073 ppan
ENSXETG00000022056	2160	793	1196	1019	2669	2013	3702	900	1015	631	2615	1793	2004 capn13
ENSXETG00000022057	2982	351	545	390	771	581	2002	1185	1425	790	1044	837	915 trpm7
ENSXETG00000022058	2082	12	19	17	157	119	254	444	490	320	286	218	232 ENSXETG00000022058
ENSXETG00000022059	1469	4	15	4	20	15	21	55	61	35	36	17	19 p2ry11
ENSXETG00000022060	990	818	1204	1025	654	503	658	903	1012	653	746	486	551 mrpl4

ENSXETG00000022063	2543	3	3	2	3	2	2	9	7	4	13	12	8	galnt14
ENSXETG00000022064	1766	10	7	13	32	30	33	285	314	191	68	48	44	ENSXETG00000022064
ENSXETG00000022070	2022	35	56	55	140	104	185	483	477	345	221	142	132	ehd3
ENSXETG00000022072	882	515	677	608	660	505	1133	1050	1304	730	789	574	562	sumo1
ENSXETG00000022073	2054	2894	4345	3692	2922	2322	1885	4505	5333	3101	2624	1914	1975	nop58
ENSXETG00000022074	3132	184	198	197	660	473	1162	1960	2249	1235	1674	1083	1324	bmpr2
ENSXETG00000022076	4077	127	207	168	516	348	933	759	905	566	779	578	704	fam117b
ENSXETG00000022077	1706	876	1334	1216	700	511	505	1150	1262	774	723	575	594	wdr12
ENSXETG00000022078	3987	0	0	0	2	1	2	5	6	5	6	4	4	
ENSXETG00000022080	2206	0	0	0	2	1	0	0	0	0	0	0	0	
ENSXETG00000022081	1644	2	9	4	10	10	3	9	17	7	6	6	9	sema6b
ENSXETG00000022083	3030	512	696	557	466	338	586	918	1050	583	636	440	504	dpp9
ENSXETG00000022086	8127	171	253	179	466	308	529	599	624	394	798	547	605	nbeal1
ENSXETG00000022087	2505	0	0	0	0	0	0	0	0	0	0	0	0	
ENSXETG00000022092	2351	633	888	771	728	577	841	1025	1223	686	676	522	572	hmg20b
ENSXETG00000022093	11706	4	7	5	152	109	15	12	17	8	28	21	23	dnah7
ENSXETG00000022097	2178	0	3	2	9	4	7	27	42	19	14	6	9	gipc3
ENSXETG00000022101	1568	5	5	11	10	6	22	141	210	110	107	50	69	tbxa2r
ENSXETG00000022106	2304	446	631	598	987	781	2058	1726	1893	1115	1725	1213	1249	cactin
ENSXETG00000022111	354	596	850	636	841	651	855	1235	1324	795	913	629	692	fam96b
ENSXETG00000022112	5016	33	59	54	597	456	1610	2506	2847	1862	2255	1605	1874	cdc42bpa
ENSXETG00000022115	399	11	6	6	24	17	42	69	71	38	37	30	32	fam219a
ENSXETG00000022117	3183	36	72	53	145	112	295	479	563	367	350	245	279	brwd3
ENSXETG00000022118	1128	1	4	2	7	5	8	15	13	9	13	5	6	gpr37
ENSXETG00000022119	885	0	0	1	7	2	0	1	0	0	1	1	0	ENSXETG00000022119
ENSXETG00000022122	682	990	1355	1114	1319	1002	3685	3575	4356	2352	1518	1045	1182	chmp1b
ENSXETG00000022124	2380	850	1173	1001	1133	807	5504	2991	3753	2158	1066	815	878	XB-GENE-947799
ENSXETG00000022125	2928	0	1	0	0	0	1	1	0	1	1	0	0	hyal4
ENSXETG00000022127	1362	1027	1466	1239	894	643	1020	904	1005	581	1042	772	782	oxa1l
ENSXETG00000022128	2213	23951	34429	28803	26582	20420	89763	15118	18287	10373	116344	81469	92010	slc7a7
ENSXETG00000022129	2297	940	1302	1086	1621	1275	2235	1912	2053	1217	1638	1152	1324	wasl
ENSXETG00000022130	1938	0	3	0	1	1	3	10	5	10	0	4	1	nrl
ENSXETG00000022131	2127	16	23	12	53	49	68	698	815	475	98	71	68	chst2
ENSXETG00000022132	1713	1	1	0	0	0	0	0	1	0	0	0	0	
ENSXETG00000022133	3103	1111	1532	1339	1803	1425	1851	2118	2628	1533	3636	2614	2894	u2surp
ENSXETG00000022134	2463	150	221	162	273	232	514	668	820	467	331	252	259	slc39a10
ENSXETG00000022136	1764	0	0	0	0	0	0	3	0	0	0	0	0	
ENSXETG00000022137	957	66	108	92	165	105	205	306	371	200	209	131	143	flot2
ENSXETG00000022138	1902	4	9	3	75	76	2	5	3	1	4	1	1	iqub
ENSXETG00000022139	2054	1009	1573	1328	1377	995	3626	1391	1600	859	1983	1496	1495	paox
ENSXETG00000022149	1357	109	146	134	131	90	188	181	208	117	132	113	135	mtg1
ENSXETG00000022150	1024	224	280	235	198	169	255	353	415	269	261	173	220	ap5s1
ENSXETG00000022152	1508	160	235	175	228	162	220	398	495	292	223	111	154	ctnnbip1
ENSXETG00000022153	1637	678	1059	801	772	592	819	1286	1417	863	702	498	510	lzic
ENSXETG00000022154	1236	182	259	208	133	113	235	364	389	204	185	130	132	nmnat1
ENSXETG00000022155	3993	732	986	911	2586	1983	4716	434	520	268	4073	2862	3243	aox1
ENSXETG00000022157	4017	429	576	467	917	696	2098	1602	1871	1076	1343	986	1075	ube4b
ENSXETG00000022163	5662	72	90	82	129	109	256	500	574	334	262	169	209	kif1b
ENSXETG00000022166	693	492	732	536	742	534	433	1221	1443	751	393	318	307	lsm7
ENSXETG00000022167	1443	2106	3125	2569	3468	2590	2340	3406	4063	2293	1261	939	1021	pgd
ENSXETG00000022169	1314	25	42	39	25	11	35	183	276	141	38	22	30	cenps
ENSXETG00000022175	3228	1	0	0	0	2	23	71	75	45	21	15	20	tmem132c
ENSXETG00000022176	1629	12	23	8	63	46	85	106	118	58	92	65	85	ENSXETG00000022176
ENSXETG00000022178	1301	6	3	4	29	21	7	31	27	22	4	3	5	c11orf70
ENSXETG00000022183	2126	1305	1806	1548	1148	974	2876	4321	5332	3051	1790	1284	1455	birc2
ENSXETG00000022185	3456	118	183	173	365	288	900	2930	3615	2159	474	322	389	mmp3
ENSXETG00000022189	1104	0	0	0	1	0	0	0	0	0	1	0	0	ENSXETG00000022189
ENSXETG00000022190	2176	504	744	612	51	31	235	2996	3674	2082	332	213	259	mmp13
ENSXETG00000022195	1425	3	9	7	2	6	2	429	520	307	9	8	11	mmp8
ENSXETG00000022196	1461	0	0	0	0	0	0	0	3	0	0	1	0	
ENSXETG00000022198	1767	160	214	185	44	46	209	759	905	519	2448	1809	2025	mmp1
ENSXETG00000022200	4186	3	1	0	6	5	17	36	42	17	15	10	6	ctnnd2
ENSXETG00000022213	1287	0	2	1	1	3	4	1	6	5	1	1	0	adra1b
ENSXETG00000022215	1419	2	3	2	26	6	77	4630	5437	3171	168	113	147	mmp13l
ENSXETG00000022216	2028	484	635	583	445	306	608	1034	1238	740	404	244	304	dcun1d5
ENSXETG00000022219	912	0	0	0	6	5	7	66	79	49	31	19	32	iglon5
ENSXETG00000022222	1000	2502	3679	3098	3837	2911	3355	3237	3568	2182	2112	1538	1573	etfb
ENSXETG00000022224	1898	0	2	0	1	0	0	2	0	2	0	2	2	
ENSXETG00000022225	813	130	182	190	280	217	654	918	1029	631	552	338	372	usf2
ENSXETG00000022227	1530	390	566	529	784	671	1095	1432	1551	936	1203	778	940	api5
ENSXETG00000022228	3204	514	721	627	899	699	936	782	783	497	876	632	614	ttc17
ENSXETG00000022229	1310	10678	16113	13632	17535	13119	19144	6754	7445	4689	12699	8996	10024	hsd17b12
ENSXETG00000022230	837	66	123	106	152	100	183	165	161	108	267	199	202	alkbh3
ENSXETG00000022231	2583	3	9	1	11	6	8	23	23	11	19	12	9	accs
ENSXETG00000022235	1618	19	25	24	103	89	177	785	876	544	297	213	206	tp53i11
ENSXETG00000022236	1140	29	32	38	272	196	455	1785	1877	1235	477	366	392	tspan18
ENSXETG00000022237	2147	1618	2329	2043	4349	3464	5596	7920	8820	5459	6187	4435	4809	cd82
ENSXETG00000022239	2073	0	0	0	0	0	0	0	0	0	0	0	0	
ENSXETG00000022240	858	0	0	0	0	0	0	1	0	0	0	0	0	
ENSXETG00000022243	4909	14	23	12	51	40	53	150	191	122	148	101	140	cadps
ENSXETG00000022245	468	0	0	0	0	0	0	2	3	0	0	1	0	hes9.1
ENSXETG00000022246	180	136	177	170	148	110	152	240	281	189	158	117	107	arfrp1
ENSXETG00000022248	1945	0	0	0	0	0	0	0	0	0	0	0	0	
ENSXETG00000022249	3594	191	209	224	401	301	613	965	1230	698	608	428	498	phf14
ENSXETG00000022250	3710	61	75	67	89	64	101	189	268	153	102	55	86	znf800
ENSXETG00000022253	6228	1693	2421	1988	3217	2591	4475	5322	6588	3730	4329	3050	3629	PLXNB2
ENSXETG00000022254	2559	187	278	220	313	231	618	1157	1440	802	494	338	397	mapk11
ENSXETG00000022255	914	7420	10699	9260	8779	6554	8300	6833	7990	4518	5845	4113	4535	atp5f1
ENSXETG00000022259	7258	2202	3459	2842	5759	4350	5231	6795	8063	4540	6816	4649	5529	prpf8
ENSXETG00000022260	1223	2	1	0	2	0	1	12	3	6	4	2	1	szl
ENSXETG00000022264	5869	177	249	183	334	224	403	494	573	303	374	243	286	tubgcp6
ENSXETG00000022266	1644	157	227	177	190	132	366	330	301	197	291	195	232	trabd
ENSXETG00000022270	987	0	0	1	2	2	1	1	0	0	2	0	4	ENSXETG00000022270
ENSXETG00000022272	2070	444	706	601	610	424	673	728	885	474	509	331	405	qrich1.2
ENSXETG00000022273	3908	3	13	5	17	10	11	9	33	16	6	4	3	mov10l1
ENSXETG00000022279	4033	35247	50209	42444	26095	20152	53763	20083	24017	14003	25640	18088	20556	slc43a2
ENSXETG00000022281	7905	8	7	3	43	33	158	596	698	438	115	82	95	pcdh7
ENSXETG00000022282	1309	0	0	1	3	0	0	0	1	2	0	0	0	XB-GENE-5872637
ENSXETG00000022285	1188	3	5	4	95	51	38	153	203	114	76	52	57	vit
ENSXETG00000022286	729	12	13	8	59	39	124	860	890	606	182	120	135	fam89a
ENSXETG00000022287	2631	618	877	709	1185	908	1201	719	859	470	1038	719	872	ttc13
ENSXETG00000022288	1162	0	2	5	36	26	120	202	254	134	86	63	71	c1orf198
ENSXETG00000022289	1397	18	29	21	32	17	43	149	193	113	55	40	43	stx11
ENSXETG00000022290	10974	392	565	480	2315	1765	2483	1400	1611	923	2930	2176	2475	utrn
ENSXETG00000022294	7968	2200	3113	2809	3166	2481	8904	2024	2240	1379	2926	2111	2279	acacb
ENSXETG00000022298	2075	150	208	146	355	278	46	247	318	187	130	88	102	ung
ENSXETG00000022299	6423	3	8	3	83	70	166	469	568	310	468	322	372	anos1
ENSXETG00000022300	708	13	11	5	5	16	6	18	24	11	14	9	4	alkbh2
ENSXETG00000022301	2349	427	577	512	496	377	1195	388	457	288	761	523	567	usp30
ENSXETG00000022304	1647	1	1	1	14	6	15	49	54	37	24	23	22	svop
ENSXETG00000022306	2346	144	226	192	403	361	327	854	945	607	377	305	320	uhrf1

ENSXETG00000022308	1044	4939	7361	6403	4801	3716	6715	3121	3726	2093	3574	2624	2866	dao
ENSXETG00000022310	2853	46	57	71	83	66	128	160	201	108	184	139	153	ssh1
ENSXETG00000022312	1560	2239	3317	2846	2994	2289	3072	8205	9087	5418	3259	2208	2504	coro1c
ENSXETG00000022315	1896	107	166	124	193	143	350	139	169	93	263	177	211	cplx2
ENSXETG00000022316	972	716	967	866	1031	748	1249	1502	1795	1013	998	693	778	thoc3
ENSXETG00000022320	1281	0	0	0	1	0	2	18	9	7	3	3	1	fam69b
ENSXETG00000022325	2700	0	0	0	0	0	0	0	0	0	0	0	0	
ENSXETG00000022329	991	554	720	666	357	289	342	527	562	377	570	396	416	mrps2
ENSXETG00000022330	3258	96	212	129	453	364	586	714	796	492	748	533	594	ppp1r26
ENSXETG00000022334	999	0	0	0	0	0	0	0	0	0	0	0	0	
ENSXETG00000022336	2592	3	10	13	37	23	40	156	179	108	121	92	98	olfm1
ENSXETG00000022337	1166	381	493	397	708	525	1251	564	626	388	480	369	403	arl10
ENSXETG00000022341	6159	221	278	290	1865	1319	2218	17580	19557	12267	3059	2299	2383	col5a1
ENSXETG00000022342	1581	2735	3862	3259	2643	2000	3467	3272	3696	2123	3635	2591	2875	nop16
ENSXETG00000022343	732	20	38	32	65	46	47	193	237	140	47	32	45	arl3.2
ENSXETG00000022346	1606	22	36	32	220	153	340	879	910	552	451	309	321	nr2f2
ENSXETG00000022348	1428	12	14	15	157	93	127	233	265	137	453	354	363	sox9
ENSXETG00000022351	1386	92	147	105	124	119	271	354	382	249	252	167	185	fbxw8
ENSXETG00000022354	4284	3	5	3	191	155	213	461	555	286	649	464	539	nos1
ENSXETG00000022355	1860	168	273	200	677	484	829	912	966	610	965	696	735	smad4.1
ENSXETG00000022356	696	0	0	0	0	0	0	14	16	6	1	0	0	ENSXETG00000022356
ENSXETG00000022357	1001	585	861	681	1392	1038	1432	2091	2469	1257	2752	1921	2080	ddt
ENSXETG00000022358	910	32259	47698	40754	33620	25013	29422	3777	4188	2539	2492	1707	1843	gstt1
ENSXETG00000022359	2605	694	1047	893	481	431	550	683	739	442	692	494	542	ddx51
ENSXETG00000022360	1512	34	49	46	54	32	37	141	189	90	12	5	4	slc2a11
ENSXETG00000022361	1266	273	442	344	453	358	777	211	227	126	393	272	287	ENSXETG00000022361
ENSXETG00000022364	1329	0	0	0	0	0	0	0	1	0	0	0	0	
ENSXETG00000022365	1879	557	887	697	878	681	820	1429	1592	942	817	610	667	rfc5
ENSXETG00000022367	2544	183	296	225	431	374	741	2091	2370	1459	764	500	618	mex3c
ENSXETG00000022368	1602	1024	1513	1257	1506	1061	1574	3210	3679	2039	1185	820	943	smarcb1
ENSXETG00000022369	2115	358	514	430	772	632	1035	1149	1408	815	1396	1037	1098	wsb2
ENSXETG00000022371	723	7551	11267	9714	8466	6131	33502	7059	7769	4707	9698	6590	7618	tspan36
ENSXETG00000022372	1437	2	1	1	29	16	109	13633	15259	9196	323	228	240	mmp11
ENSXETG00000022373	1267	80	88	78	169	133	283	234	277	173	281	188	208	vsig10
ENSXETG00000022374	2022	3	5	3	132	105	18	8	10	7	413	317	349	baat
ENSXETG00000022375	1065	2086	3042	2502	1911	1429	1463	958	1053	559	501	413	423	pebp1
ENSXETG00000022376	3223	115	160	126	327	229	457	428	514	319	652	515	496	taok3
ENSXETG00000022377	3458	405	607	482	814	620	624	1180	1418	814	854	582	630	suds3
ENSXETG00000022379	1260	0	6	5	5	3	6	8	18	5	14	15	12	ENSXETG00000022379
ENSXETG00000022383	1734	258	374	373	618	535	851	67	84	49	418	309	362	mapk4
ENSXETG00000022384	2086	0	0	0	0	0	1	1	0	1	0	1	0	ENSXETG00000022384
ENSXETG00000022386	3033	928	1256	1012	1589	1214	2801	2522	3011	1724	1846	1295	1414	dgcr2
ENSXETG00000022387	1365	70	114	79	95	83	184	317	387	203	253	154	173	dgcr14
ENSXETG00000022388	3666	623	963	791	1128	886	2416	2755	3019	1770	1577	1180	1281	me2
ENSXETG00000022389	2105	3511	4967	4306	5011	3911	6032	1820	2142	1309	2577	1923	2062	slc25a1
ENSXETG00000022391	1503	185	297	244	413	309	710	442	512	308	689	488	519	lipg
ENSXETG00000022392	849	145	238	166	282	207	389	227	319	176	461	325	352	ctso
ENSXETG00000022396	2093	4	3	6	1	0	0	3	1	0	0	0	0	tdo2
ENSXETG00000022398	1494	16	15	11	116	90	39	49	60	39	226	169	169	asic5
ENSXETG00000022399	1963	15	15	23	140	123	339	1182	1526	868	563	381	470	gucy1b3
ENSXETG00000022403	1759	233	358	319	275	193	666	889	1011	577	246	142	181	snap29
ENSXETG00000022408	6951	633	898	783	1526	1100	2373	1942	2229	1256	1939	1352	1580	pi4ka
ENSXETG00000022412	1602	2	1	2	1	2	2	4	3	3	0	2	2	rbm46
ENSXETG00000022415	1628	13	20	26	0	0	0	37	54	35	1	0	0	fgg
ENSXETG00000022417	1800	20	33	25	88	50	137	932	1129	627	275	230	238	serpind1
ENSXETG00000022419	1755	35	47	37	79	63	84	50	55	32	68	62	59	hic2
ENSXETG00000022422	1202	3838	5555	4573	4666	3559	5021	6614	7397	4268	3719	2672	2959	ube2l3
ENSXETG00000022425	318	0	0	1	0	2	1	1	0	0	1	1	3	doc2b
ENSXETG00000022426	1313	1441	2005	1752	951	755	911	487	519	301	843	579	633	ydjc
ENSXETG00000022428	1235	44	78	68	94	66	143	329	364	206	198	168	140	rph3al
ENSXETG00000022429	2094	62	68	62	54	43	56	145	172	103	209	171	195	rflnb
ENSXETG00000022431	1945	10	11	8	41	30	11	48	94	36	28	13	29	fgb
ENSXETG00000022432	2496	436	686	476	522	396	909	926	1107	561	753	510	572	vps53
ENSXETG00000022434	1817	691	1020	932	992	787	1058	1819	2099	1228	924	682	712	plrg1
ENSXETG00000022438	1593	2	1	3	2	6	5	2	7	5	0	0	0	slc47a1
ENSXETG00000022439	2298	368	551	504	885	713	763	1018	1214	673	693	490	502	pir
ENSXETG00000022440	1068	590	777	720	772	581	830	784	975	520	805	561	630	mrps5
ENSXETG00000022441	900	109	166	174	234	176	526	413	484	286	318	227	233	ttc19
ENSXETG00000022442	1031	53	73	60	1780	1311	191	508	650	354	2420	1699	1934	mal
ENSXETG00000022445	1059	0	0	0	0	0	0	0	0	1	0	0	0	ENSXETG00000022445
ENSXETG00000022446	2142	63	64	55	235	180	102	329	399	234	122	90	113	nphp1
ENSXETG00000022447	2262	208	319	271	497	382	898	1658	2003	1090	867	654	704	nxn
ENSXETG00000022448	3864	111	190	192	639	476	715	751	986	534	125	99	121	bub1
ENSXETG00000022451	2420	243	350	290	245	213	245	254	332	212	210	174	167	mrm3
ENSXETG00000022452	2580	38915	56864	48131	66395	51119	144282	21171	24985	14498	54968	38671	43397	ace2
ENSXETG00000022456	3627	802	1212	1026	4204	2955	5301	2006	2173	1437	4626	3313	3403	plekha7
ENSXETG00000022458	2006	710	1070	973	763	586	1186	1310	1471	858	840	604	710	glod4
ENSXETG00000022460	3207	98	79	113	107	87	231	376	477	278	229	173	194	gemin4
ENSXETG00000022463	1251	324	487	426	676	552	994	143	175	99	132	106	88	ENSXETG00000022463
ENSXETG00000022464	1317	5	7	5	15	10	9	51	60	42	9	7	3	fam57a
ENSXETG00000022465	4513	1651	2331	1944	1918	1471	4384	2405	2872	1622	3064	2188	2533	rffl
ENSXETG00000022466	2312	44	65	68	126	78	443	350	448	247	4448	3231	3676	tmem27
ENSXETG00000022467	2499	16	21	16	4	0	7	75	82	42	8	2	5	ENSXETG00000022467
ENSXETG00000022468	1213	207	272	245	235	210	538	1134	1430	796	471	333	379	ap1s2
ENSXETG00000022475	2532	135	204	200	379	259	575	1735	1969	1077	718	511	473	ENSXETG00000022475
ENSXETG00000022476	948	0	0	0	0	0	0	0	0	0	0	0	0	
ENSXETG00000022477	2354	56	118	96	280	229	573	1061	1063	683	469	327	354	ENSXETG00000022477
ENSXETG00000022479	2766	233	317	316	467	310	564	787	900	474	727	435	571	ctps2
ENSXETG00000022490	1826	0	0	0	0	0	0	0	0	0	0	0	0	
ENSXETG00000022498	1524	48	89	83	36	25	41	177	193	130	32	22	24	ENSXETG00000022498
ENSXETG00000022502	1516	330	510	428	826	599	1198	991	1090	680	1104	798	884	supt7l
ENSXETG00000022503	1944	230	334	280	598	447	478	886	1001	679	606	406	444	slc4a1ap
ENSXETG00000022505	2181	23	35	17	103	74	58	178	189	113	39	22	37	sclt1
ENSXETG00000022510	7230	191	284	221	419	309	471	446	493	287	746	532	623	jade1
ENSXETG00000022511	1692	4951	7098	6204	6034	4620	14824	1693	1980	1253	5812	4273	4621	mthfd1
ENSXETG00000022512	3541	140	243	205	650	499	847	808	914	534	1001	657	755	larp1b
ENSXETG00000022516	1224	517	718	644	522	411	739	704	830	449	799	527	596	pdrg1
ENSXETG00000022517	1036	42	74	61	185	110	285	327	422	253	372	253	299	rerg
ENSXETG00000022520	2043	885	1334	1114	922	782	1330	1695	1940	1174	1534	1039	1176	pgrmc2
ENSXETG00000022522	2969	66	112	74	298	235	211	318	371	212	74	41	49	plk4
ENSXETG00000022523	4192	0	6	3	17	20	16	145	218	102	123	77	89	ptpro
ENSXETG00000022524	2310	31	45	34	57	46	227	101	107	55	180	110	139	fam120b
ENSXETG00000022525	3404	142	221	183	1226	922	660	1157	1353	843	1361	1007	1091	dll1
ENSXETG00000022526	2447	4206	6270	5292	7375	5537	7697	4068	4584	2860	8960	6580	7096	eps8
ENSXETG00000022527	1168	626	974	899	1268	1008	1484	1645	1845	1163	984	748	727	strap
ENSXETG00000022528	1912	860	1350	980	575	481	768	596	762	425	409	356	372	abhd18
ENSXETG00000022529	1293	32	46	47	94	37	70	97	124	82	122	112	112	zbtb25
ENSXETG00000022532	2058	128	175	170	340	293	434	409	493	287	567	449	416	zbtb1
ENSXETG00000022533	2862	38	46	55	129	110	69	243	261	157	69	53	73	intu
ENSXETG00000022535	3622	948	1250	994	690	550	631	442	524	321	453	316	353	gnpat
ENSXETG00000022536	1902	3	5	2	39	39	32	105	138	72	190	151	185	hspa2

ENSXETG00000022538	2823	0	2	2	9	2	8	282	302	188	5	3	4 adam12
ENSXETG00000022539	2166	0	0	1	3	4	6	13	14	9	11	11	7 trim67
ENSXETG00000022542	1644	5	8	9	45	33	74	94	116	81	92	45	58 ptpdc1
ENSXETG00000022543	1642	513	640	588	1020	755	2312	1362	1581	965	1216	877	901 bod1
ENSXETG00000022545	1682	17	33	21	44	29	11	82	112	70	36	27	30 ppp1r14c
ENSXETG00000022546	2403	346	526	432	953	723	1508	1224	1417	850	998	727	805 strn
ENSXETG00000022548	5987	6	11	7	25	13	18	202	188	141	59	31	37 WNK2
ENSXETG00000022549	6579	199	267	234	357	283	374	698	849	503	340	230	326 brf1
ENSXETG00000022551	1002	15	18	14	28	24	18	16	4	7	12	8	7 fank1
ENSXETG00000022553	3508	905	1294	1107	2926	2263	5016	4708	5098	3214	4675	3440	3573 fam120a
ENSXETG00000022554	2082	357	462	432	777	592	342	320	359	190	205	135	157 dhx32
ENSXETG00000022555	420	2	1	0	0	0	2	3	1	2	2	0	3 ENSXETG00000022555
ENSXETG00000022556	2401	148	258	235	732	559	879	2495	2842	1702	1174	821	951 btbd6
ENSXETG00000022559	2406	28	50	43	128	91	203	266	296	198	298	238	249 phf2
ENSXETG00000022562	6198	45	73	58	294	225	679	601	682	421	819	595	617 zfhx2
ENSXETG00000022563	957	0	0	0	0	1	0	0	0	0	1	1	2 ENSXETG00000022563
ENSXETG00000022564	5119	546	815	712	804	592	645	406	518	302	624	403	481 AP1G2
ENSXETG00000022566	1232	5038	7435	6384	5482	4237	5799	1204	1514	853	2472	1791	1874 dhrs4
ENSXETG00000022571	1272	0	0	0	1	0	0	0	0	0	0	0	0 ENSXETG00000022571
ENSXETG00000022572	1856	6179	8980	7678	5575	4395	7253	4307	4926	2696	6651	4874	5299 tsc22d3
ENSXETG00000022574	3764	70	86	84	238	195	136	415	540	312	29	18	23 ercc6l
ENSXETG00000022577	4458	22	37	26	67	48	112	287	296	254	352	246	263 carmil3
ENSXETG00000022578	2622	721	1135	956	825	586	678	1066	1277	688	761	488	560 pin4
ENSXETG00000022580	2529	3	10	3	21	30	94	388	475	266	145	96	124 nhsl2
ENSXETG00000022581	3448	369	547	467	867	684	1171	1390	1456	908	1695	1222	1326 znf250
ENSXETG00000022583	1152	0	0	0	0	0	0	0	0	0	0	0	0
ENSXETG00000022587	1968	143	192	177	1965	1457	2199	1481	1664	1010	5401	3791	4175 coq8a
ENSXETG00000022588	1815	21	43	25	73	59	60	31	34	21	111	102	99 mmp21
ENSXETG00000022590	993	670	1023	892	1495	1164	2523	6143	7025	4195	3072	2077	2336 tnfsf10l
ENSXETG00000022592	1904	360	523	464	289	231	803	616	736	417	463	339	349 cyb5r4
ENSXETG00000022597	1251	147	214	191	362	275	603	904	1027	628	129	75	115 ccnb3
ENSXETG00000022599	3369	24	22	27	84	33	93	254	269	167	95	63	72 ENSXETG00000022599
ENSXETG00000022600	1263	226	381	290	777	646	1359	317	350	197	598	377	453 man1a1
ENSXETG00000022603	894	39	41	43	186	149	225	465	554	328	314	192	254 itpkb
ENSXETG00000022604	1584	13	22	17	83	40	36	229	269	170	41	26	24 eef1a1o
ENSXETG00000022606	1296	0	0	1	1	0	1742	55	58	30	140	104	115 ENSXETG00000022606
ENSXETG00000022608	3582	74	102	86	138	103	241	314	325	229	160	111	142 edrf1
ENSXETG00000022609	1971	7	10	13	15	11	52	134	160	83	72	49	45 ENSXETG00000022609
ENSXETG00000022610	3000	549	763	682	1874	1373	1822	2976	3252	1976	2118	1486	1560 parp1
ENSXETG00000022612	488	821	1111	901	721	524	750	1035	1161	721	605	386	470 mrpl51
ENSXETG00000022613	3124	541	850	707	1222	915	1140	2405	2636	1590	1113	794	796 aldh18a1
ENSXETG00000022614	1041	1770	2650	2138	7575	5810	6430	22928	26904	15375	8046	5614	6279 epcam
ENSXETG00000022615	2760	83	137	127	476	341	205	855	905	604	339	253	282 msh2
ENSXETG00000022616	3312	7	20	15	65	60	74	40	55	32	32	25	18 fam184a
ENSXETG00000022617	1644	642	1007	861	1833	1405	2656	2352	2516	1595	2118	1455	1631 lrp10
ENSXETG00000022620	1635	159	279	235	373	315	559	811	929	511	640	396	498 lin9
ENSXETG00000022623	3207	300	422	360	620	485	495	947	1074	642	1441	960	1097 rbm23
ENSXETG00000022624	1278	0	0	2	3	7	6	7	9	6	15	6	6 kcnk12
ENSXETG00000022626	2260	700	1011	881	1372	1036	828	3163	3623	2203	1076	722	780 prmt5
ENSXETG00000022627	939	0	0	0	0	0	0	0	0	0	0	0	0 ENSXETG00000022627
ENSXETG00000022629	3124	9	9	14	20	18	22	86	96	62	47	23	38 snap91
ENSXETG00000022630	1107	71	84	71	140	111	50	182	198	127	42	29	30 haus4
ENSXETG00000022632	2276	68	97	88	46	36	83	217	246	138	116	118	91 c14orf93
ENSXETG00000022633	1050	0	0	0	0	0	0	0	0	0	0	0	0 bix1.2
ENSXETG00000022634	4337	1527	2166	1812	1735	1382	2503	3275	3555	2263	2481	1823	1959 acbd3
ENSXETG00000022635	4103	331	458	383	1001	839	630	1243	1419	849	768	621	620 msh6
ENSXETG00000022636	1266	7	20	13	119	81	175	4462	5377	3227	149	118	115 prss35
ENSXETG00000022637	1686	28	35	37	34	33	90	359	415	217	130	111	105 ENSXETG00000022637
ENSXETG00000022638	747	0	0	0	0	0	0	0	0	1	0	0	0
ENSXETG00000022641	525	2434	3531	3032	4389	3232	4926	12655	14182	8259	5001	3641	3745 h3f3b
ENSXETG00000022643	1629	288	450	365	625	472	832	1265	1445	895	1035	755	814 sde2
ENSXETG00000022644	4091	740	1075	878	1763	1381	2445	2836	3329	1988	2682	1825	2113 fbxo11
ENSXETG00000022646	6843	5	12	10	102	73	96	434	432	283	269	214	247 cacna1h
ENSXETG00000022647	1671	3102	4724	4008	2464	1964	7650	1197	1433	896	1632	1238	1378 me1
ENSXETG00000022648	2166	0	0	0	1	0	1	21	27	10	2	1	1 cdh24
ENSXETG00000022649	519	0	0	0	0	1	0	0	0	0	2	2	1 lefty
ENSXETG00000022650	3001	54	70	73	306	242	471	546	672	372	616	472	451 tmem63a
ENSXETG00000022651	1487	31	56	49	73	55	93	152	183	81	118	97	83 foxn2
ENSXETG00000022652	2337	505	669	600	1107	869	1649	1252	1376	790	1185	736	878 ppp1r21
ENSXETG00000022654	2860	907	1342	1170	1459	1121	2341	1145	1307	811	3406	2422	2636 ephx1
ENSXETG00000022658	3890	10	24	28	157	127	460	664	815	490	365	251	288 gtf2a1l
ENSXETG00000022659	1377	6	19	11	107	74	52	325	408	224	88	54	61 b9d1
ENSXETG00000022660	948	0	0	0	0	0	0	0	0	0	0	0	0
ENSXETG00000022662	1764	26	37	17	114	73	206	715	777	487	305	247	261 epn2
ENSXETG00000022663	1842	0	0	0	0	1	1	7	12	7	0	0	1 ENSXETG00000022663
ENSXETG00000022665	2025	2	2	3	4	8	8	55	57	41	12	8	15 slc5a10
ENSXETG00000022667	2750	417	570	481	872	692	1862	807	878	529	993	696	778 fam83g
ENSXETG00000022668	1520	17	29	19	21	20	65	242	310	204	101	85	77 grap
ENSXETG00000022669	4176	91	127	132	676	546	1908	31979	37607	21836	4092	2987	3409 mmp14
ENSXETG00000022670	684	3	4	5	9	13	15	208	256	131	47	34	41 syngr3
ENSXETG00000022671	3772	645	953	773	908	707	1048	1544	1673	1032	1060	690	820 znf598
ENSXETG00000022672	1008	29	42	34	62	51	60	84	118	59	65	47	79 rwdd2a
ENSXETG00000022673	2018	900	1383	1171	1048	771	1539	1322	1504	878	1029	742	826 pgm3
ENSXETG00000022674	780	501	675	526	320	217	429	422	494	285	254	180	191 ndufaf4
ENSXETG00000022675	7336	389	508	458	843	622	1623	740	918	550	1290	851	956 dopey1
ENSXETG00000022677	1170	62	98	93	121	92	207	83	88	59	138	100	113 ube3d
ENSXETG00000022685	279	0	0	0	0	0	0	0	0	0	0	0	0 RPS25
ENSXETG00000022687	4256	5	16	8	30	30	21	32	41	26	46	43	33 slc35g2
ENSXETG00000022688	1956	160	249	186	478	307	507	1395	1584	897	779	552	606 nck1
ENSXETG00000022689	720	0	0	0	0	0	1	0	0	1	1	0	0
ENSXETG00000022690	783	1	0	1	1765	1449	128	2	3	6	3105	2181	2455 cldn18
ENSXETG00000022691	2470	1	5	4	247	202	12	12	10	12	27	16	9 dzip1l
ENSXETG00000022694	4607	1465	2113	1790	2718	1975	4803	2570	2954	1779	3351	2211	2603 uhrf1bp1l
ENSXETG00000022706	1005	0	0	0	0	0	0	0	0	0	0	0	0
ENSXETG00000022707	1002	0	0	0	0	0	0	0	0	0	0	0	1
ENSXETG00000022722	1481	423	709	515	886	715	1244	807	1074	601	1194	781	937 ing3
ENSXETG00000022726	2553	0	0	0	0	0	1	0	0	0	0	0	0 ENSXETG00000022726
ENSXETG00000022727	1265	0	0	0	4	1	30	4	5	4	6	2	2 wnt16
ENSXETG00000022729	2342	534	714	676	554	446	883	633	711	436	514	336	373 pdf
ENSXETG00000022730	1043	112	161	149	227	162	325	305	449	241	273	182	211 fam3c
ENSXETG00000022733	477	3	6	7	9	6	18	169	174	125	28	32	19 rhoq
ENSXETG00000022735	6932	1	0	0	5	5	8	98	134	88	49	39	46 ptprz1
ENSXETG00000022737	653	238	347	276	826	681	1218	985	1131	656	1093	792	844 cript
ENSXETG00000022741	702	0	1	0	2	1	4	1	3	2	0	0	0 ENSXETG00000022741
ENSXETG00000022744	2240	99	128	106	169	127	292	587	697	403	232	139	176 socs5
ENSXETG00000022745	465	394	554	498	338	258	317	910	1065	654	115	84	102 mcfd2
ENSXETG00000022746	1794	352	531	494	658	489	1053	1102	1281	731	875	678	718 ttc7a
ENSXETG00000022747	3448	205	301	222	368	292	688	667	753	444	1017	657	758 aass
ENSXETG00000022748	2231	0	0	0	0	0	0	0	0	0	0	0	0
ENSXETG00000022749	3888	159	227	170	277	243	468	512	634	346	414	284	320 cadps2
ENSXETG00000022751	1833	582	870	645	924	709	1322	1150	1439	853	346	276	300 slc13a1

ENSXETG00000022755	1896	2	8	4	64	49	124	300	304	197	226	125	151	cacnb2
ENSXETG00000022756	2874	0	1	0	0	0	0	4	7	1	1	1	3	
ENSXETG00000022757	1395	9210	13442	11227	8773	7219	11869	32570	36303	21987	17051	12120	13570	ptma
ENSXETG00000022759	447	22	27	17	27	20	87	96	114	73	66	63	66	pde6d
ENSXETG00000022761	795	631	874	768	765	597	908	1058	1242	734	860	597	679	cops7b
ENSXETG00000022762	1788	148	193	190	191	140	221	239	323	160	316	233	237	nsun6
ENSXETG00000022765	4195	88	119	106	141	115	170	412	501	312	232	172	198	arl5b
ENSXETG00000022768	1967	127	176	149	173	134	218	135	160	91	2862	2033	2143	alpi.1
ENSXETG00000022769	21453	2051	2963	2506	4060	3116	6672	3066	3622	2108	3135	2253	2495	dst
ENSXETG00000022772	2295	0	0	0	2	3	0	2	1	3	0	0	1	ecel1
ENSXETG00000022775	5917	510	770	629	840	667	959	1763	2082	1103	1081	786	914	arhgap32
ENSXETG00000022779	1119	0	0	0	0	0	0	0	0	0	0	0	0	
ENSXETG00000022780	4507	27	40	26	158	110	305	580	798	443	288	202	238	fli1
ENSXETG00000022781	3864	75	110	86	155	123	312	1298	1622	973	364	285	315	ets1
ENSXETG00000022783	2898	97	125	96	252	189	442	843	1089	604	375	286	271	snx13
ENSXETG00000022784	2565	65	94	100	268	191	373	757	1022	562	624	451	502	ahr
ENSXETG00000022789	1464	0	0	0	0	0	0	0	0	0	2	0	0	ENSXETG00000022789
ENSXETG00000022793	1039	994	1305	1119	1090	763	1232	1085	1363	748	1853	1325	1445	agr3
ENSXETG00000022794	606	691	896	762	437	356	818	751	878	513	556	418	480	tspan13
ENSXETG00000022795	1266	178	289	248	251	180	325	277	326	197	401	250	295	bzw2
ENSXETG00000022796	1098	167	250	203	269	173	455	388	427	272	361	249	286	ankmy2
ENSXETG00000022797	1068	751	1134	937	1126	843	1511	1851	2177	1288	1273	964	1021	syf2
ENSXETG00000022798	2043	20	40	38	47	57	75	293	412	186	174	97	119	sostdc1
ENSXETG00000022799	2150	38	59	44	74	56	77	136	179	98	189	124	146	ispd
ENSXETG00000022803	1938	206	309	259	1451	1134	3364	1471	1503	1038	5054	3540	3984	foxk2
ENSXETG00000022804	1866	602	901	737	803	628	796	1888	2129	1316	911	678	716	wdr45b
ENSXETG00000022805	1830	89	147	128	192	102	323	194	217	143	352	285	276	pgm2l1
ENSXETG00000022806	3661	1543	2207	1976	2495	1777	2778	4403	4829	2928	2892	2110	2316	calcoco2
ENSXETG00000022807	1155	43	69	60	530	408	639	301	343	199	1096	820	883	rab40b
ENSXETG00000022808	753	1133	1653	1420	1311	972	1523	811	904	521	1227	856	952	kcnk1
ENSXETG00000022810	912	18	38	14	5	14	22	109	129	86	64	29	38	gpr171
ENSXETG00000022814	1035	476	689	553	385	278	491	520	603	386	279	195	239	fam20b
ENSXETG00000022815	1758	10	16	16	53	32	137	520	557	335	251	181	194	ralgps2
ENSXETG00000022817	1047	139	174	137	225	190	338	569	590	370	370	280	262	ube2z
ENSXETG00000022819	774	226	344	297	383	328	712	454	533	304	546	334	400	snf8
ENSXETG00000022820	891	0	0	0	0	0	0	0	0	1	0	0	1	ENSXETG00000022820
ENSXETG00000022821	4073	236	354	302	708	502	1006	1207	1380	896	1525	1075	1275	rasal2
ENSXETG00000022822	816	313	413	352	801	626	1012	150	205	94	154	91	118	ENSXETG00000022822
ENSXETG00000022827	1771	0	1	0	4	2	4	10	27	15	5	4	4	brinp2
ENSXETG00000022828	1119	2	0	0	5	6	6	3	7	1	10	10	5	kcnk15
ENSXETG00000022830	983	4622	6692	5707	9301	7101	11865	11267	13143	8001	7626	5399	5711	ywhab
ENSXETG00000022831	2034	1	0	2	8	3	0	1	1	0	1	0	0	pabpc1l
ENSXETG00000022832	2268	709	996	811	1486	1148	1897	3131	3597	2110	2331	1624	1821	stk4
ENSXETG00000022834	2736	4	1	1	11	11	9	0	1	2	3	4	1	
ENSXETG00000022838	660	482	648	559	1633	1309	2601	1368	1525	981	3446	2418	2771	sdc4
ENSXETG00000022839	3162	31	33	36	317	235	310	2065	2478	1486	829	578	645	mecom
ENSXETG00000022840	4374	1	0	2	16	21	49	141	150	97	97	66	80	adgrl1
ENSXETG00000022848	1932	336	419	399	675	513	913	1523	1611	995	918	693	689	golim4
ENSXETG00000022849	933	719	1041	941	1511	1128	1773	2636	2858	1756	1297	925	1013	prkaca
ENSXETG00000022850	895	680	1022	835	316	291	280	296	334	192	184	156	156	bag2
ENSXETG00000022852	1152	4	8	4	5	2	4	1	0	0	2	1	0	ENSXETG00000022852
ENSXETG00000022855	897	0	0	0	0	0	0	0	0	0	0	0	0	ENSXETG00000022855
ENSXETG00000022857	1764	0	0	0	0	0	0	0	0	0	0	0	0	
ENSXETG00000022859	4003	20	27	23	220	171	75	134	200	127	198	135	151	lrrc8e
ENSXETG00000022865	3227	264	397	322	888	669	1122	1196	1355	859	1382	992	1206	map2k7
ENSXETG00000022866	1613	205	340	296	243	176	186	314	376	202	309	177	267	serpini1
ENSXETG00000022868	2409	65	122	94	158	117	271	323	376	219	286	203	240	evi5l
ENSXETG00000022874	2577	21	29	27	136	117	27	18	11	4	49	37	50	wdr49
ENSXETG00000022875	585	2251	3060	2578	1533	1191	2226	2278	2719	1483	1206	867	928	pdcd10
ENSXETG00000022877	1440	0	1	1	12	3	0	0	1	0	9	5	18	serpini2
ENSXETG00000022881	5399	326	449	361	686	548	1811	834	1078	582	899	633	750	inpp5k
ENSXETG00000022882	3213	216	294	237	615	481	796	1387	1630	965	931	710	742	myo1c
ENSXETG00000022884	546	666	1000	858	1034	768	850	568	633	398	874	651	745	sat2
ENSXETG00000022887	1299	481	707	652	495	415	687	959	973	614	518	349	326	abcb10
ENSXETG00000022889	963	0	0	2	23	15	1	4	3	2	5	2	1	zbbx
ENSXETG00000022892	2691	1	0	0	29	10	30	51	53	31	77	49	49	sorcs2
ENSXETG00000022893	717	6012	8574	7435	9304	7457	10962	14621	16340	9801	8854	6008	6874	ywhae
ENSXETG00000022894	431	6	9	8	1	3	0	141	236	120	13	8	2	
ENSXETG00000022898	2187	0	0	0	0	0	0	0	0	0	0	0	0	ENSXETG00000022898
ENSXETG00000022900	1465	1432	2224	1829	538	424	395	880	1012	590	441	294	335	grpel1
ENSXETG00000022903	1263	33	33	37	122	69	96	150	152	105	139	81	90	tada2b
ENSXETG00000022904	1479	261	378	342	391	298	633	1014	1159	669	491	340	397	crk
ENSXETG00000022907	1425	166	235	190	159	133	803	712	900	450	395	287	339	nagpa
ENSXETG00000022908	2073	807	1210	1078	4363	3339	8689	1452	1568	996	5753	4060	4547	tbc1d14
ENSXETG00000022909	4410	402	613	528	1109	856	2156	3434	4159	2547	2220	1574	1816	kiaa0232
ENSXETG00000022910	1491	35	66	46	50	49	59	236	264	138	57	34	47	adpgk
ENSXETG00000022912	1892	728	983	833	401	291	638	2862	3392	1973	1474	1018	1199	fhl3
ENSXETG00000022916	1470	0	0	0	0	0	0	0	1	0	1	0	0	
ENSXETG00000022919	2101	2451	3552	3033	3232	2505	2491	5192	5592	3475	7714	5471	5852	snrnp70
ENSXETG00000022920	3747	1974	2884	2401	4842	3808	6635	2563	2964	1817	5243	3734	4314	ptprh
ENSXETG00000022923	1666	1216	1653	1460	2690	2022	4140	3618	4124	2536	6309	4469	5023	sf3a3
ENSXETG00000022924	2178	1	3	1	8	7	8	31	34	17	16	14	16	brsk1
ENSXETG00000022926	1394	265	414	372	254	162	261	676	746	476	264	183	194	hspbp1
ENSXETG00000022927	2187	505	731	599	1200	914	2376	1529	1717	941	2544	1732	2023	ppp6r1
ENSXETG00000022930	1887	232	336	308	434	318	421	500	535	326	297	202	219	ndor1
ENSXETG00000022931	1536	34	42	54	111	92	198	267	293	199	546	386	430	slc47a2
ENSXETG00000022932	2463	219	263	253	549	407	553	774	815	564	411	258	336	anapc2
ENSXETG00000022933	4146	19	40	30	24	24	38	136	132	86	60	49	47	grin1
ENSXETG00000022934	1562	2934	4235	3489	5853	4293	11323	3976	4265	2697	6230	4323	4674	XB-GENE-5992704
ENSXETG00000022935	2734	78	121	88	184	123	203	602	663	391	468	286	363	znf618
ENSXETG00000022936	1314	164	229	192	476	366	581	595	580	451	650	460	454	araf
ENSXETG00000022938	1834	15720	23092	19747	11522	8640	3401	2208	2612	1455	468	309	414	isyna1
ENSXETG00000022939	702	1156	1702	1367	1207	899	1578	869	964	597	1363	968	1067	dhfr
ENSXETG00000022940	888	9	2	6	38	28	67	237	251	161	80	58	58	ssbp4
ENSXETG00000022941	2751	84	129	116	129	103	146	264	322	188	76	47	68	msh3
ENSXETG00000022942	3696	6	4	5	24	8	58	52	50	42	42	50	30	rasgrf2
ENSXETG00000022948	1722	299	394	372	541	376	370	305	441	203	140	82	111	ENSXETG00000022948
ENSXETG00000022950	801	0	0	0	1	0	0	0	2	2	0	0	0	
ENSXETG00000022951	6283	8	18	12	120	86	127	443	425	298	263	204	254	prkce
ENSXETG00000022954	2801	407	589	452	613	472	788	1344	1604	955	826	614	630	map2k6
ENSXETG00000022956	596	1444	2123	1727	1164	888	1105	485	574	297	697	453	499	chchd10
ENSXETG00000022958	3162	84	96	92	231	154	247	548	664	365	317	224	240	usp28
ENSXETG00000022959	765	18	15	11	17	7	47	188	203	125	20	14	8	XB-GENE-5764783
ENSXETG00000022960	903	49	59	60	230	162	33	2	2	1	901	612	733	aqp10
ENSXETG00000022961	2787	955	1333	1107	1765	1320	2281	452	534	329	1189	839	997	kcnj16
ENSXETG00000022962	1278	1	6	3	10	10	23	84	104	56	20	19	15	kcnj2
ENSXETG00000022963	4636	1110	1596	1471	3911	2897	6727	5847	6686	3748	8524	5951	6562	mkl1
ENSXETG00000022966	1353	0	0	0	0	0	0	0	0	0	0	0	0	
ENSXETG00000022967	1395	20	42	35	171	124	251	374	414	262	331	257	248	she
ENSXETG00000022968	1366	681	1032	879	472	404	717	1205	1433	845	612	500	531	slc25a17
ENSXETG00000022969	782	43	91	81	29	23	80	146	196	109	32	22	23	fhit

ENSXETG00000022970	2171	8	7	11	46	20	68	531	589	344	81	47	64	fstl3
ENSXETG00000022971	1922	3245	4691	4235	7148	5395	8391	7735	8789	5169	14400	10354	11073	st13
ENSXETG00000022972	957	0	0	0	2	6	4	2	2	4	1	2	2	tdrd10
ENSXETG00000022973	1813	116	187	148	130	88	162	216	289	152	222	194	216	xpnpep3
ENSXETG00000022974	6924	299	496	396	1374	1023	1624	1853	1952	1206	2242	1585	1798	EP300
ENSXETG00000022975	1128	0	0	0	0	0	0	0	1	0	0	0	0	
ENSXETG00000022977	2401	272	398	347	567	393	760	700	890	445	761	511	625	zw10
ENSXETG00000022980	4248	36	55	38	119	64	269	969	1287	710	294	240	266	ankrd50
ENSXETG00000022981	1557	52	76	87	118	97	153	193	231	119	266	152	200	l3mbtl2
ENSXETG00000022982	2115	0	0	0	0	0	0	5	3	1	2	2	2	chadl
ENSXETG00000022983	1026	5	12	9	117	93	6	1312	1483	870	68	31	43	gprc5a
ENSXETG00000022984	1650	841	1129	1030	873	712	1165	1277	1517	812	1476	986	1167	ddx47
ENSXETG00000022986	1683	0	0	0	2	3	3	11	17	4	10	2	12	brinp3
ENSXETG00000022989	1847	110	163	130	162	129	341	556	604	341	240	176	184	dr1
ENSXETG00000022991	2311	0	0	0	0	0	0	1	0	0	0	0	0	
ENSXETG00000022993	1764	51	61	53	205	159	380	889	1060	597	388	281	298	rnf112.1
ENSXETG00000022994	2388	0	2	1	3	0	1	2	3	4	5	0	1	elfn1
ENSXETG00000022997	1014	87	111	110	295	200	289	456	531	340	358	271	312	mad1l1
ENSXETG00000022999	2168	16	28	19	54	42	129	110	111	53	114	71	100	ccdc18
ENSXETG00000023000	681	448	646	516	265	263	495	306	384	274	467	319	400	tmed5
ENSXETG00000023001	2758	177	223	204	309	279	375	811	932	526	249	193	214	mtf2
ENSXETG00000023002	1960	112	142	128	123	91	199	153	211	142	67	65	62	fam69a
ENSXETG00000023003	3172	0	0	0	0	0	0	0	0	0	0	0	0	
ENSXETG00000023004	1010	54688	80760	70938	83323	62342	101857	89470	102466	62273	229621	159936	176091	rpl5
ENSXETG00000023005	2515	151	204	165	333	277	566	791	880	524	619	433	444	evi5
ENSXETG00000023006	1191	16	22	14	81	55	95	88	98	54	110	102	90	gfi1
ENSXETG00000023007	1398	35	41	33	74	57	169	103	109	56	140	72	85	rpap2
ENSXETG00000023009	2356	76	129	105	89	76	232	230	282	176	156	122	124	glmn
ENSXETG00000023010	600	296	460	402	641	510	1581	3482	3637	2517	1478	1001	1151	creg1
ENSXETG00000023012	1047	16	27	13	82	64	271	577	609	414	253	197	211	rcsd1
ENSXETG00000023013	3852	186	258	246	190	163	338	201	287	140	319	215	213	btbd8
ENSXETG00000023014	738	209	287	246	754	540	1402	1550	1689	1108	1334	854	1059	mpzl1
ENSXETG00000023015	1467	436	585	547	569	381	519	731	753	484	594	407	430	tmem39a
ENSXETG00000023016	1005	122	209	167	343	280	979	2263	2477	1770	1291	922	1035	b4galt4
ENSXETG00000023017	831	107	172	135	639	519	275	364	339	195	758	487	578	XB-GENE-988039
ENSXETG00000023018	1592	3	1	4	29	22	16	86	73	37	92	43	73	b3gat1
ENSXETG00000023020	1077	0	2	0	1	1	2	14	15	13	12	7	10	ephx4
ENSXETG00000023026	1259	3650	5291	4597	10230	7879	12473	10099	11651	6858	8068	5809	6285	hnrnpdl
ENSXETG00000023029	1235	0	1	0	0	1	0	0	0	1	0	1	0	upk1b
ENSXETG00000023030	2292	6	10	6	77	75	247	226	306	149	339	196	247	tgfbr3
ENSXETG00000023031	5888	326	420	395	729	496	851	1447	1724	1015	797	595	635	efr3a
ENSXETG00000023033	1083	0	0	0	2	0	0	8	4	2	3	4	2	igsf11
ENSXETG00000023034	1724	106	155	128	216	183	173	235	298	197	249	143	162	cdc7
ENSXETG00000023036	2256	0	0	0	0	3	2	24	19	6	2	4	2	kcnq3
ENSXETG00000023038	1545	32443	47725	40636	45135	34268	35879	15409	17971	10447	15631	11145	12201	ckb
ENSXETG00000023039	1939	2	6	9	37	22	19	24	24	27	11	6	8	lrrc6
ENSXETG00000023043	2720	3689	5448	4817	9309	7289	11816	8663	9886	5809	9906	7230	7745	snd1
ENSXETG00000023046	1734	105	159	117	222	211	358	492	580	327	353	264	291	phf20l1
ENSXETG00000023047	2867	652	960	865	1173	858	1370	2990	3384	1910	1815	1289	1480	impdh1
ENSXETG00000023048	963	80	136	125	176	158	241	365	426	251	291	216	259	ENSXETG00000023048
ENSXETG00000023050	1686	1599	2384	2069	1051	812	779	59	75	37	13	7	10	sqle
ENSXETG00000023051	1849	122	153	134	49	53	146	117	137	112	145	104	105	bag5
ENSXETG00000023054	3469	1096	1443	1246	1182	876	1573	2322	2585	1611	1683	1198	1303	klc1
ENSXETG00000023057	2192	0	2	3	3	2	1	2	3	5	2	2	1	opn1sw
ENSXETG00000023058	3015	5	13	14	44	21	58	88	108	61	64	52	46	invs
ENSXETG00000023059	2523	1394	1894	1695	1666	1288	1711	5801	6877	3979	1120	768	843	calu
ENSXETG00000023061	1445	263	409	292	353	253	647	261	335	182	479	357	342	xrcc3
ENSXETG00000023062	2686	1845	2588	2144	2168	1709	3978	3228	4003	2121	4229	3006	3392	zfyve21
ENSXETG00000023063	2550	191	302	265	361	303	538	615	747	438	520	393	372	gcc1
ENSXETG00000023064	5034	321	430	366	978	747	948	1216	1252	821	1502	1053	1234	ppp1r13b
ENSXETG00000023066	1671	3	7	2	8	9	13	58	55	32	46	25	42	dennd6b
ENSXETG00000023067	1556	378	527	460	632	471	1007	1237	1587	945	791	532	609	fam3a
ENSXETG00000023068	2878	161	225	193	431	353	684	756	849	475	934	689	745	ppp6r2
ENSXETG00000023069	3809	1	0	1	5	2	1	2	3	1	8	5	11	tdrd9
ENSXETG00000023071	1261	368	595	519	1059	831	1663	696	716	461	1009	683	770	fam102a
ENSXETG00000023076	3611	539	718	663	1038	794	1952	810	907	515	988	703	796	aspg
ENSXETG00000023077	615	152	216	168	97	81	156	261	270	173	94	93	73	ENSXETG00000023077
ENSXETG00000023079	8240	756	1147	965	1673	1234	2372	3956	4671	2644	2478	1815	1948	sbf1
ENSXETG00000023080	5391	49	66	62	175	139	407	1686	1961	1145	219	159	196	kif26a
ENSXETG00000023082	6050	19	41	33	46	27	27	136	185	104	44	45	51	wnk3
ENSXETG00000023084	3144	182	302	278	719	562	846	1588	1712	1010	1127	839	895	fam120c
ENSXETG00000023087	1020	0	0	0	0	1	1	0	0	1	0	0	0	miox
ENSXETG00000023089	2899	277	389	327	544	430	404	1129	1429	787	292	203	220	lmf2
ENSXETG00000023090	2632	147	189	142	493	369	312	773	966	519	141	90	87	ncaph2
ENSXETG00000023092	456	359	541	468	333	249	326	610	742	474	265	169	183	surf4.2
ENSXETG00000023094	2042	40	57	63	126	112	61	313	373	220	49	30	44	cdca5
ENSXETG00000023095	1358	739	1037	846	699	557	786	977	1154	606	758	545	559	zfpl1
ENSXETG00000023096	1032	510	710	605	685	575	823	746	889	619	1880	1456	1499	rexo4
ENSXETG00000023097	4806	418	642	536	1058	759	888	394	515	319	1095	698	836	CDC42BPA
ENSXETG00000023098	2730	40	74	70	152	117	201	126	148	92	546	389	397	sardh
ENSXETG00000023099	2010	1	0	0	0	0	0	0	0	1	0	0	2	slc24a2
ENSXETG00000023100	821	72109	105144	92259	117696	90504	119972	103919	115872	72932	242367	173316	186651	rps6
ENSXETG00000023101	1619	28417	41216	34698	4232	3130	9418	31749	37178	20931	2700	1940	2007	plin2
ENSXETG00000023103	1572	4	1	4	338	277	20	3	3	0	16	12	13	spata18
ENSXETG00000023104	5865	227	376	304	795	642	1095	1233	1334	841	1352	971	1033	dennd4c
ENSXETG00000023106	1536	1330	1807	1519	1316	1067	1539	1565	1861	1046	867	569	692	pmpcb
ENSXETG00000023108	2047	1149	1661	1396	955	743	831	2044	2430	1373	895	694	681	dnajc2
ENSXETG00000023109	5280	4	1	10	51	47	40	192	201	127	55	38	54	adamtsl1
ENSXETG00000023110	1845	0	0	0	0	0	4	0	0	1	2	2	0	dbh
ENSXETG00000023111	2397	93	129	114	299	202	278	684	873	488	273	185	226	slc26a5
ENSXETG00000023113	2835	490	777	645	1274	953	2197	1926	2088	1299	1711	1205	1348	scyl1
ENSXETG00000023114	10564	464	704	568	1202	935	1356	2059	2341	1402	2630	1863	2054	SPEN
ENSXETG00000023115	468	0	0	1	0	0	0	7	2	0	2	0	2	fam163b
ENSXETG00000023116	1140	3	1	0	4	6	9	60	57	34	5	8	5	ENSXETG00000023116
ENSXETG00000023118	1809	246	321	255	459	365	381	475	507	314	418	292	290	zbtb17
ENSXETG00000023119	3439	1904	2585	2096	1937	1542	4045	1984	2388	1252	9748	6857	7687	frmd8
ENSXETG00000023120	1239	0	1	0	2	7	6	332	368	212	52	26	33	kcnk2
ENSXETG00000023124	8145	45	62	69	410	296	324	574	674	381	82	61	79	cenpf
ENSXETG00000023126	1098	30	37	38	18	22	68	230	260	153	90	44	62	samsn1
ENSXETG00000023127	1459	1453	2176	1863	2320	1759	2853	4052	4519	2775	1515	979	1186	psmc2
ENSXETG00000023129	876	476	689	614	928	737	782	1822	1982	1246	1256	928	939	ier5
ENSXETG00000023130	5498	396	521	462	806	665	972	1011	1310	703	1002	680	817	nrip1
ENSXETG00000023131	906	10	9	11	13	18	12	410	542	246	30	31	31	ENSXETG00000023131
ENSXETG00000023132	866	157	209	189	252	205	266	442	533	341	338	219	237	stx6
ENSXETG00000023134	5209	615	849	802	1880	1467	4016	2420	2796	1630	2348	1560	1695	xpr1
ENSXETG00000023135	3944	124	220	213	400	335	501	1660	1922	1176	815	551	696	usp25
ENSXETG00000023137	5514	289	367	331	779	550	1026	1162	1253	748	1065	744	814	taf1
ENSXETG00000023140	2820	453	678	563	1971	1503	1469	1837	2015	1379	3244	2373	2489	plekha5
ENSXETG00000023141	2795	231	338	290	356	255	575	850	983	615	325	224	243	acbd6
ENSXETG00000023143	1816	1245	1771	1514	2699	2132	3639	1001	1192	650	2241	1485	1725	aadat
ENSXETG00000023144	1696	110	168	119	207	141	240	1409	1744	971	165	133	131	mfap3l

ENSXETG00000023145	1041	336	493	379	283	222	170	676	843	475	193	141	144	hpf1
ENSXETG00000023146	2719	3107	4590	4038	3772	2840	7644	3040	3596	2123	2791	2100	2283	clcn3
ENSXETG00000023151	1562	631	883	714	621	436	1012	839	1146	622	525	385	445	fam173b
ENSXETG00000023153	4347	3373	4852	4125	6812	5120	6480	6066	6984	4196	14723	10540	12012	ogt
ENSXETG00000023154	2298	37	59	57	1207	921	189	216	289	169	2638	1849	2142	slc9a3
ENSXETG00000023155	7161	47	53	41	268	173	157	541	606	353	160	90	149	nek1
ENSXETG00000023156	2360	48	94	80	81	65	144	76	81	57	344	209	259	qsox1
ENSXETG00000023157	3354	10	15	10	80	63	118	505	526	322	142	126	127	ptpn14
ENSXETG00000023159	969	0	0	0	0	0	0	0	0	0	0	0	0	
ENSXETG00000023169	738	0	0	0	0	1	1	0	0	1	2	2	0	ENSXETG00000023169
ENSXETG00000023173	2058	163	271	184	408	318	836	966	1164	640	591	439	480	srpk3
ENSXETG00000023175	3402	876	1301	1119	2017	1558	5052	9706	10944	6597	2924	2051	2272	smyd2
ENSXETG00000023176	2214	15	27	20	35	21	48	80	90	64	72	39	52	prox1
ENSXETG00000023178	915	0	0	0	0	0	0	0	0	0	0	0	0	ENSXETG00000023178
ENSXETG00000023182	2845	208	341	289	494	324	1025	1123	1362	842	1006	735	795	zhx2
ENSXETG00000023186	1397	3753	5238	4487	3650	2939	3956	3165	3495	2488	2606	1911	2070	derl1
ENSXETG00000023188	975	426	574	513	769	576	713	969	1046	616	782	545	629	igbp1
ENSXETG00000023189	3642	436	605	504	794	588	781	786	863	541	665	478	564	tbc1d31
ENSXETG00000023190	1457	0	3	2	5	7	10	7	13	10	6	5	5	dgat2l6
ENSXETG00000023191	1726	0	1	0	3	3	6	8	3	3	0	1	0	fam83a
ENSXETG00000023192	2643	221	320	273	695	535	1238	1412	1589	998	1439	1023	1160	zhx1
ENSXETG00000023193	2283	13	12	6	8	10	19	158	184	116	50	21	23	p2ry4
ENSXETG00000023197	546	0	0	0	0	0	0	0	0	0	0	0	0	
ENSXETG00000023198	1155	0	0	0	0	0	0	0	0	0	0	0	0	crlf1
ENSXETG00000023199	854	414	647	513	416	333	296	728	775	453	259	170	201	mrpl48
ENSXETG00000023200	676	987	1509	1236	1335	996	2079	2359	2744	1801	1388	965	1078	rab6a
ENSXETG00000023201	876	470	654	578	464	363	713	578	699	401	523	393	484	c19orf60
ENSXETG00000023202	455	22857	33817	29099	34265	25249	36857	40088	46492	27429	78677	55223	60080	uba52
ENSXETG00000023203	1401	128	178	168	127	82	222	299	370	198	144	89	102	kxd1
ENSXETG00000023204	1267	0	0	1	0	2	1	0	0	0	0	0	0	arr3
ENSXETG00000023205	2183	567	820	777	1305	964	2257	916	1056	612	1096	718	850	fkbp8
ENSXETG00000023207	2589	832	1157	948	684	520	813	891	1162	586	804	506	632	pus7
ENSXETG00000023209	1122	0	1	0	0	0	0	34	22	22	0	0	0	ENSXETG00000023209
ENSXETG00000023211	1247	4	1	4	2	2	3	18	15	15	10	11	7	capg
ENSXETG00000023213	1828	136	225	191	249	181	402	337	382	265	372	244	286	ell
ENSXETG00000023215	4107	320	456	382	1251	982	320	1211	1394	787	312	213	237	atad2
ENSXETG00000023220	1914	4828	7053	6016	4928	3715	8560	4239	4865	2929	4038	2726	3140	ENSXETG00000023220
ENSXETG00000023224	1733	0	0	0	0	0	0	0	2	1	1	0	0	gabrg2
ENSXETG00000023226	2256	922	1334	1176	1042	776	1044	876	1133	575	982	725	845	rint1
ENSXETG00000023227	2090	2	1	0	17	16	28	24	34	19	38	33	29	wnt11b
ENSXETG00000023228	1062	43	56	54	639	532	4326	1917	2106	1335	2699	1809	2089	fbxo32
ENSXETG00000023230	1743	24	40	38	178	140	155	44	31	23	307	211	241	klhl38
ENSXETG00000023232	2808	45	42	48	98	79	173	195	260	145	309	218	243	atxn7l1
ENSXETG00000023234	1659	2315	3222	2817	3132	2334	5656	4110	4944	3012	3739	2639	3044	soat1
ENSXETG00000023235	1635	408	617	499	3623	2770	4756	3741	4032	2641	5571	4142	4367	ece1
ENSXETG00000023236	1662	19477	28352	24546	18778	14045	16221	8297	9973	5668	7720	5453	6101	anxa13
ENSXETG00000023239	1659	0	0	0	0	0	0	0	0	1	0	0	0	slc22a6
ENSXETG00000023240	2373	1	4	1	153	133	22	9	12	4	22	21	24	cdhr3
ENSXETG00000023241	1802	482	680	534	660	517	919	1180	1452	836	926	688	737	mcrs1
ENSXETG00000023243	2686	480	717	594	1069	806	1282	1439	1506	961	1106	781	859	fam91a1
ENSXETG00000023244	1611	10	18	17	49	40	41	411	493	304	27	20	22	alpl
ENSXETG00000023247	5514	57	89	60	696	539	355	383	500	282	1597	1176	1254	fer1l6
ENSXETG00000023248	2355	310	474	405	584	417	865	1036	1138	752	717	544	543	fbxo42
ENSXETG00000023250	210	1	3	0	22	17	20	6	12	12	122	74	69	ENSXETG00000023250
ENSXETG00000023251	1616	34	28	38	49	36	112	127	105	73	99	61	70	adhfe1
ENSXETG00000023254	1137	0	5	2	5	4	7	1	1	2	5	0	11	zfp36l2.2
ENSXETG00000023255	1151	59	72	89	359	264	278	65	76	53	197	148	141	zfp36l2.1
ENSXETG00000023262	1681	1	1	2	39	34	2	5	5	7	4	5	6	ppp1r42
ENSXETG00000023263	1224	1368	1979	1726	1752	1373	2216	3100	3695	2075	1146	844	868	cops5
ENSXETG00000023264	2422	32	38	30	213	156	196	143	176	118	170	126	152	cspp1
ENSXETG00000023265	1512	21	13	22	80	61	148	235	268	147	163	144	113	SLC6A4
ENSXETG00000023270	803	3	1	2	0	0	0	1	1	0	0	0	0	sycp3
ENSXETG00000023276	585	0	0	0	0	0	0	0	0	0	0	0	0	
ENSXETG00000023277	3258	19	18	15	67	76	127	458	515	344	238	170	190	znf385c
ENSXETG00000023279	720	103	173	129	206	136	273	110	120	86	577	399	413	atoh1
ENSXETG00000023280	2292	1	1	1	2	0	1	5	1	3	4	3	4	grid2
ENSXETG00000023284	7077	345	475	439	1238	929	1371	1238	1586	936	1160	845	995	hivep2
ENSXETG00000023288	1319	151	243	189	307	246	346	522	540	367	394	254	321	cdc42ep2
ENSXETG00000023289	2705	176	290	244	654	548	287	806	878	578	402	332	335	pola2
ENSXETG00000023290	3095	266	364	308	463	387	625	853	1081	595	840	602	652	hsf2
ENSXETG00000023292	2572	437	645	509	1116	845	1935	3542	4518	2521	2371	1591	1881	serinc1
ENSXETG00000023295	1427	40	58	55	127	104	82	273	292	164	259	182	199	lmo4.1
ENSXETG00000023296	1487	476	670	544	597	463	1689	2329	2930	1685	931	666	778	smpdl3a
ENSXETG00000023297	679	1	4	2	24	10	9	106	134	86	26	14	14	fabp7
ENSXETG00000023298	993	0	0	0	1	1	0	5	7	4	3	1	3	clvs2
ENSXETG00000023299	2650	528	744	673	4558	3447	2671	4501	5189	3066	3152	2215	2556	capn1
ENSXETG00000023300	1217	178	234	178	183	151	201	229	304	170	165	121	116	tprkb
ENSXETG00000023301	2443	109	150	142	535	435	883	434	499	293	110	85	94	gtse1
ENSXETG00000023302	4935	52	64	55	172	123	363	1028	1276	709	672	452	524	cnksr3
ENSXETG00000023303	1383	436	550	453	186	138	349	49	105	43	150	101	132	unc93a
ENSXETG00000023304	1278	216	299	235	228	175	256	223	253	144	291	201	242	trmu
ENSXETG00000023305	5367	903	1259	1051	2324	1770	2132	2500	2938	1721	2361	1613	1879	afdn
ENSXETG00000023306	8307	12	24	7	568	411	87	345	351	232	731	486	598	celsr1
ENSXETG00000023308	2281	210	264	256	443	299	547	435	508	336	398	276	354	thul16
ENSXETG00000023310	2554	167	262	202	388	299	400	167	227	116	522	350	444	capn9
ENSXETG00000023311	3112	68	86	85	112	86	162	387	468	281	246	153	174	syne3
ENSXETG00000023313	3406	118	162	122	133	109	250	580	691	396	252	198	200	gramd4
ENSXETG00000023315	5700	144	190	160	293	228	384	566	654	382	477	261	370	dicer1
ENSXETG00000023319	2899	2231	3206	2674	3597	2732	3445	977	1234	640	1163	831	1074	tm7sf2
ENSXETG00000023320	1707	621	937	724	863	703	1818	923	1110	645	1114	786	856	cerk
ENSXETG00000023322	1629	139	174	156	204	143	487	380	443	246	345	238	290	tbc1d22a
ENSXETG00000023323	1662	144	188	153	170	150	289	421	478	268	388	279	312	znhit2
ENSXETG00000023327	2679	1565	2183	1824	2637	2003	3632	1822	2132	1245	3011	2042	2349	syvn1
ENSXETG00000023328	1093	0	1	0	0	0	0	0	0	2	0	0	0	
ENSXETG00000023329	1356	47	81	59	55	58	25	66	70	34	0	0	0	serpina1
ENSXETG00000023330	2088	231	413	294	315	223	193	481	581	322	223	161	193	taf5l
ENSXETG00000023331	4581	330	452	375	223	183	214	347	437	232	349	277	281	urb2
ENSXETG00000023332	1659	177	221	207	204	178	421	382	449	266	288	214	246	galnt2
ENSXETG00000023334	804	286	442	403	201	150	285	407	499	264	162	99	125	slc25a22
ENSXETG00000023336	1251	5	1	4	14	9	14	49	47	27	21	9	19	pgbd5
ENSXETG00000023338	1131	2	4	3	32	22	23	59	70	41	10	9	1	pdgfrl.2
ENSXETG00000023340	2208	766	1094	933	844	646	1591	775	1012	563	829	598	763	cog2
ENSXETG00000023341	4005	2310	3311	2684	1952	1490	2817	1005	1158	660	1374	1038	1181	tmem181
ENSXETG00000023342	1414	394	519	386	165	120	2000	645	936	488	582	375	448	agt
ENSXETG00000023344	2881	375	525	487	385	377	582	596	723	347	545	402	404	serac1
ENSXETG00000023345	547	321	458	377	267	215	265	450	578	314	215	152	171	gtf2h5
ENSXETG00000023346	1749	553	882	650	944	695	3363	1451	1860	1053	1588	1142	1257	snx9
ENSXETG00000023348	2883	13	23	32	55	35	74	52	74	34	78	43	60	ENSXETG00000023348
ENSXETG00000023355	3498	652	870	743	1984	1521	1571	1786	1876	1210	3290	2362	2424	hdac6
ENSXETG00000023361	939	0	0	0	0	0	0	0	0	0	0	0	0	
ENSXETG00000023362	924	0	0	0	0	0	0	0	0	0	0	0	0	

ENSXETG00000023368	1641	9	13	15	7	5	14	60	80	44	28	17	36	ENSXETG00000023368
ENSXETG00000023370	945	0	0	0	0	0	0	0	0	0	0	0	0	
ENSXETG00000023372	972	0	0	0	0	0	0	0	0	0	0	0	0	
ENSXETG00000023376	1751	156	266	217	578	489	1056	1950	2013	1400	839	649	637	snx18
ENSXETG00000023377	438	0	0	0	0	0	0	3	1	0	2	0	0	hspb3
ENSXETG00000023378	2533	187	223	207	344	247	710	970	1244	695	430	332	358	arl15
ENSXETG00000023380	498	13	7	8	22	15	12	36	51	26	40	27	22	heg1
ENSXETG00000023381	2781	243	364	261	365	300	470	553	718	415	311	234	274	tex10
ENSXETG00000023383	2358	63	112	82	365	260	465	783	873	562	519	356	382	znf148
ENSXETG00000023384	773	1720	2377	2049	1492	1175	1629	1504	1627	904	960	698	745	ndufs4
ENSXETG00000023385	2792	3	5	1	131	93	144	396	462	288	291	248	251	fst
ENSXETG00000023388	624	27	36	39	198	148	355	299	362	234	317	257	254	ENSXETG00000023388
ENSXETG00000023389	1299	394	578	470	930	688	1235	1725	1850	1199	872	639	663	snx4
ENSXETG00000023392	1808	739	992	766	1363	1075	2122	888	1131	558	1882	1422	1459	rassf6
ENSXETG00000023393	2748	931	1403	1258	3131	2433	4023	2778	2977	1870	3817	2791	3010	osbpl11
ENSXETG00000023394	2082	592	894	714	1089	849	1763	1922	2457	1395	1701	1134	1250	tgfbr1
ENSXETG00000023395	1979	42	77	53	1	0	0	219	220	118	4	2	2	alb
ENSXETG00000023397	1041	4634	6840	5820	7328	5390	10299	576	614	382	7763	5669	6032	GPD1
ENSXETG00000023399	5433	50	61	44	92	77	131	1036	1110	653	120	74	86	parp14.2
ENSXETG00000023400	2151	302	429	402	572	445	673	2428	2674	1657	385	295	307	ENSXETG00000023400
ENSXETG00000023401	4575	202	309	235	1006	809	1763	1357	1465	885	1907	1367	1484	ENSXETG00000023401
ENSXETG00000023402	738	46	76	62	149	90	143	243	264	160	115	88	103	ENSXETG00000023402
ENSXETG00000023408	2137	2436	3659	3082	5558	4010	4592	2364	2730	1614	2810	1887	2255	sdha
ENSXETG00000023412	1332	117	170	140	141	107	263	329	384	244	242	178	194	def8
ENSXETG00000023413	2552	21	18	20	58	48	83	904	1159	649	236	171	164	tubb3
ENSXETG00000023414	2052	870	1258	1093	1476	1208	2182	1722	2032	1205	1878	1272	1420	tcf25
ENSXETG00000023416	2686	7	15	14	1	3	22	1	8	3	14	15	19	tat
ENSXETG00000023417	2155	346	487	448	464	380	672	330	375	259	401	287	328	ccdc127
ENSXETG00000023418	663	329	536	413	329	273	308	386	445	272	137	73	113	alg13
ENSXETG00000023419	6123	646	916	810	1175	964	2538	1581	1992	1148	1678	1207	1320	golga4
ENSXETG00000023421	1690	190	278	261	310	261	549	677	750	454	416	279	338	znf821
ENSXETG00000023422	3090	11	9	9	106	75	319	392	485	319	252	176	205	itga9
ENSXETG00000023423	1834	1352	1844	1553	2697	2093	5595	5409	6360	3709	2671	1928	2137	ist1
ENSXETG00000023424	1003	79	172	145	526	385	294	191	205	103	197	134	159	akr1c8p
ENSXETG00000023425	1136	6	14	4	134	97	11	30	26	12	2	1	2	akr1c2
ENSXETG00000023427	1443	0	0	0	0	0	0	0	0	0	0	0	0	neurod6
ENSXETG00000023430	1992	0	0	1	5	3	7	12	16	9	12	8	11	pde1c
ENSXETG00000023433	834	3	6	7	14	9	20	51	52	41	37	23	36	rnf144a
ENSXETG00000023435	1086	7	7	7	57	46	148	4	5	3	39	17	23	rsad2
ENSXETG00000023437	1272	16	25	25	120	90	237	54	74	44	69	38	46	cmpk2
ENSXETG00000023439	1098	2	2	2	16	9	43	166	205	135	43	35	33	sox11
ENSXETG00000023441	477	1	5	4	6	5	2	0	3	2	6	7	4	ppp1r17
ENSXETG00000023444	1514	709	1001	949	548	466	744	742	846	474	613	500	521	dhodh
ENSXETG00000023447	3606	452	610	565	996	808	1322	2029	2233	1377	1164	855	887	dhx38
ENSXETG00000023449	750	5	15	8	22	11	21	269	278	180	49	54	49	rnf208
ENSXETG00000023451	1493	130	180	179	242	203	300	1356	1500	948	545	373	477	tor4a
ENSXETG00000023452	4067	113	140	123	924	763	583	2643	3002	1910	1280	897	911	ddr2
ENSXETG00000023454	954	175	262	202	311	256	681	334	416	294	612	456	506	ppara
ENSXETG00000023455	2760	17	22	23	261	202	220	1234	1253	877	705	482	511	glipr2
ENSXETG00000023462	837	20	19	17	66	51	68	101	84	56	153	105	102	ENSXETG00000023462
ENSXETG00000023465	2388	1172	1830	1477	867	582	3092	1955	2202	1376	3151	2319	2448	lmo4.2
ENSXETG00000023469	1797	19642	28999	24701	29119	21887	275100	3491	3827	2352	103967	73387	79146	slc34a3
ENSXETG00000023470	792	0	0	0	0	0	0	0	0	0	0	0	0	
ENSXETG00000023471	882	0	0	0	0	0	0	2	1	3	0	0	0	
ENSXETG00000023472	1056	0	0	1	9	7	10	92	80	34	10	5	6	
ENSXETG00000023473	1522	2350	3488	2908	13312	10148	3566	6900	7419	4833	2056	1278	1515	tubb4b
ENSXETG00000023474	831	875	1394	1183	1147	853	1464	1019	1066	632	1263	954	929	alad
ENSXETG00000023475	1422	1	8	6	23	17	47	154	203	111	34	22	13	hoxc9
ENSXETG00000023476	2243	30	69	27	96	72	212	790	1061	614	202	134	141	hoxc8
ENSXETG00000023477	741	28	58	60	54	52	56	81	58	43	46	45	40	hdhd3
ENSXETG00000023479	705	10	10	11	8	14	89	214	239	135	37	24	24	hoxc6
ENSXETG00000023481	762	3	2	1	3	6	2	39	53	31	14	5	6	st8sia1
ENSXETG00000023482	2478	10	16	9	29	15	46	265	321	203	99	99	99	bhlhe41
ENSXETG00000023484	795	11	12	15	49	29	72	241	280	132	167	113	119	hoxc4
ENSXETG00000023485	1884	201	318	256	636	470	764	824	941	603	714	521	606	tmem63b
ENSXETG00000023486	664	1002	1485	1229	936	662	1451	1564	1628	1043	893	630	616	mrpl14
ENSXETG00000023490	1134	11	32	28	129	124	212	802	930	600	339	281	293	rassf8
ENSXETG00000023492	537	0	3	3	4	2	5	6	12	6	11	5	6	rnf183
ENSXETG00000023495	1465	45	48	43	62	60	105	842	1104	630	155	123	126	rap2a
ENSXETG00000023496	4117	2658	3688	3220	6228	4784	10102	7654	8875	5364	14554	10289	11622	ipo5
ENSXETG00000023497	2335	0	0	0	0	0	0	0	1	0	0	0	0	
ENSXETG00000023499	1726	41	63	43	84	57	113	49	66	36	147	97	109	nkx2-2
ENSXETG00000023500	1032	0	0	0	0	0	1	0	0	0	0	0	0	
ENSXETG00000023501	2914	1097	1608	1387	2509	1983	3356	3267	3837	2208	3158	2273	2542	xrn2
ENSXETG00000023506	2315	122	177	158	347	272	507	370	437	240	341	255	306	kiz
ENSXETG00000023508	861	0	0	0	0	0	0	0	0	0	0	0	0	
ENSXETG00000023509	10530	66	120	87	299	231	596	683	684	445	898	609	725	zfhx3
ENSXETG00000023514	1373	0	0	0	0	1	0	18	21	12	2	2	3	ENSXETG00000023514
ENSXETG00000023516	1065	0	1	0	5	3	23	166	225	140	47	27	24	klhdc8b
ENSXETG00000023522	1728	0	4	5	32	25	128	149	168	94	81	64	72	ENSXETG00000023522
ENSXETG00000023523	2683	214	309	229	300	227	383	1065	1277	759	286	212	226	bax
ENSXETG00000023524	2191	464	659	567	674	476	1250	1266	1477	832	799	513	599	stk24
ENSXETG00000023526	1779	55	85	83	147	114	77	160	173	116	235	157	187	slc6a16
ENSXETG00000023532	1233	67	83	92	72	48	127	186	233	174	130	102	100	creld1
ENSXETG00000023534	4429	101	136	88	324	261	623	715	903	529	787	566	611	dock9
ENSXETG00000023538	1911	621	883	774	416	320	797	453	601	406	419	316	370	ubac2
ENSXETG00000023542	2695	0	1	0	72	40	3	3	0	0	4	1	4	ENSXETG00000023542
ENSXETG00000023543	1851	3578	4947	4193	5302	4006	6140	4027	5009	2725	3105	2237	2546	tm9sf2
ENSXETG00000023544	459	1	0	0	0	0	0	0	1	0	2	0	2	
ENSXETG00000023545	891	0	0	0	0	0	0	0	0	0	0	0	0	
ENSXETG00000023546	1094	299	395	349	220	176	181	248	293	178	259	173	208	rpusd3
ENSXETG00000023547	1878	3520	4872	4083	1295	1105	2512	2841	3345	1912	1306	944	1050	cidec
ENSXETG00000023548	2082	27	43	37	105	93	160	198	223	137	171	137	129	ENSXETG00000023548
ENSXETG00000023554	2770	56	81	59	46	48	135	349	370	251	114	60	102	rassf4
ENSXETG00000023556	1110	687	932	762	1793	1351	2499	327	381	207	1313	996	1122	kcnj15
ENSXETG00000023557	957	0	0	0	3	0	2	3	0	1	1	2	2	KCNJ6
ENSXETG00000023558	2721	65	98	69	152	136	310	130	170	102	131	85	102	fbxo15
ENSXETG00000023559	927	835	1202	988	710	552	661	591	691	419	650	421	511	timm21
ENSXETG00000023560	1843	5515	7559	6010	4023	3134	6328	3642	4548	2506	1452	1081	1207	cyb5a
ENSXETG00000023561	1581	17141	25216	22085	22664	17302	22418	12110	13738	8254	9144	6513	7234	cndp2
ENSXETG00000023563	1056	0	6	0	2	2	2	31	33	21	19	9	11	fam69c
ENSXETG00000023564	1844	332	489	429	518	394	774	702	948	534	288	203	213	cndp1
ENSXETG00000023565	2116	6	5	4	11	6	19	81	133	68	29	30	20	rufy2
ENSXETG00000023567	2070	1854	2840	2374	4361	3369	5691	7376	8441	5177	5620	3773	4136	hnrnph3
ENSXETG00000023568	693	868	1233	956	832	612	1760	855	1044	555	547	369	435	bloc1s2
ENSXETG00000023570	3258	171	262	191	443	384	610	588	762	405	575	403	474	herc4
ENSXETG00000023571	723	0	0	0	0	0	0	10	12	2	2	2	3	ENSXETG00000023571
ENSXETG00000023572	1275	491	741	601	962	768	708	262	318	184	332	211	256	gk5
ENSXETG00000023575	420	0	0	2	0	3	0	3	0	0	3	5	0	atoh7
ENSXETG00000023578	807	1	0	1	0	0	0	0	0	0	0	0	0	
ENSXETG00000023585	3521	3	2	1	33	26	54	448	549	318	228	175	169	dpysl4

ENSXETG00000023586	1935	216	336	270	430	321	376	851	1060	565	551	373	430	ecd
ENSXETG00000023588	2250	275	371	360	728	588	928	827	970	644	669	498	499	sirt1
ENSXETG00000023590	3504	1	1	0	2	2	0	3	3	4	0	3	1	ENSXETG00000023590
ENSXETG00000023591	8433	38	66	42	322	243	513	574	679	386	814	563	631	vwf
ENSXETG00000023595	2575	906	1411	1188	1305	1016	1409	1480	1706	972	774	543	603	tor1a
ENSXETG00000023596	3357	92	130	119	238	181	326	565	662	363	64	46	37	ect2
ENSXETG00000023597	2325	27	52	38	104	75	217	273	327	189	389	284	322	ENSXETG00000023597
ENSXETG00000023608	1315	295	473	425	623	530	631	1661	1951	1169	676	505	514	inpp5a.1
ENSXETG00000023609	1103	1815	2649	2266	2859	2105	2972	4658	5406	3332	8472	6100	6709	cd9
ENSXETG00000023610	1101	1	3	1	23	9	14	45	51	40	84	67	74	ghsr
ENSXETG00000023613	1225	0	0	0	0	0	0	0	0	0	0	0	0	tnni1.2
ENSXETG00000023614	1691	21	28	18	1605	1198	36	2	0	1	1373	956	1085	nkx6-2
ENSXETG00000023615	3633	4	8	7	100	56	111	1256	1486	844	230	163	164	fndc3b
ENSXETG00000023616	3908	23	32	20	118	82	233	328	387	241	237	183	215	bpgm
ENSXETG00000023618	1727	49	82	77	462	354	996	3556	3970	2433	2391	1673	1845	cald1
ENSXETG00000023620	741	0	0	0	0	0	0	0	0	0	0	0	0	
ENSXETG00000023627	1262	354	581	434	503	390	570	696	808	480	614	426	422	fam175b
ENSXETG00000023629	2278	407	526	463	1185	871	1869	1419	1515	957	1181	808	908	zranb1
ENSXETG00000023631	3118	32	46	40	49	32	76	40	50	32	29	23	20	ctbp2
ENSXETG00000023632	876	274	366	342	432	346	681	755	854	491	670	451	548	tsnare1
ENSXETG00000023638	648	466	692	618	375	272	479	638	748	437	407	285	318	cox11
ENSXETG00000023640	2640	3	6	1	48	38	157	128	142	62	178	139	165	mmrn1
ENSXETG00000023641	300	0	0	1	1	0	1	12	8	2	4	6	8	snca
ENSXETG00000023642	3267	34165	50082	42893	43057	33168	80339	421	491	253	18885	13372	14993	gprin3
ENSXETG00000023643	3618	55	83	57	100	84	121	214	227	129	161	107	113	znf484
ENSXETG00000023647	2295	94	130	116	261	179	378	761	838	503	510	345	345	rufy3
ENSXETG00000023648	1023	178	267	216	146	123	315	836	1067	575	223	161	159	mob1b
ENSXETG00000023649	3871	127	207	173	305	246	125	945	1140	626	81	78	77	dck.1
ENSXETG00000023652	3123	795	1177	977	1649	1155	3657	1757	2082	1203	4546	3282	3654	herc3
ENSXETG00000023654	1466	152	230	184	497	387	415	493	575	310	618	386	418	il17re
ENSXETG00000023658	3776	100	153	103	275	173	348	483	493	301	381	308	328	brpf1
ENSXETG00000023661	3066	322	405	382	1685	1337	2388	696	818	503	3043	2247	2396	fam13a
ENSXETG00000023668	1217	35	52	48	81	54	95	155	166	104	50	25	27	ogg1
ENSXETG00000023670	531	4	4	5	0	2	0	2	5	3	0	0	1	il10
ENSXETG00000023671	2475	2	2	2	7	3	13	306	349	198	8	8	13	XB-GENE-5901104
ENSXETG00000023672	2658	246	333	281	405	330	690	5688	6725	3916	1326	901	991	camk1
ENSXETG00000023673	2779	597	859	766	1133	842	1868	1805	2239	1282	1207	898	958	mapkapk2
ENSXETG00000023674	1038	93	154	146	286	240	289	613	641	383	377	244	276	tada3
ENSXETG00000023675	1414	6940	9908	8351	6162	4727	8883	12449	14640	8686	6278	4488	5075	arpc4
ENSXETG00000023676	2398	4828	7016	6149	7755	5854	13936	2475	2833	1632	4896	3368	3803	acox2
ENSXETG00000023677	3661	77	115	111	128	126	345	507	622	361	332	269	321	dyrk3
ENSXETG00000023678	2125	280	375	323	348	264	302	715	864	503	358	240	282	eif2d
ENSXETG00000023679	890	15	11	20	11	6	35	134	163	109	51	30	37	rassf5
ENSXETG00000023683	574	362	570	467	567	381	508	969	1082	666	341	237	221	lsm6
ENSXETG00000023684	3174	117	169	134	155	117	295	358	437	232	246	160	184	znf827
ENSXETG00000023685	1077	93	133	121	104	80	254	147	209	101	105	91	85	mmaa
ENSXETG00000023687	3611	16	18	14	79	49	98	546	574	360	120	103	122	srgap2
ENSXETG00000023689	1556	0	0	0	0	0	0	0	0	0	0	0	0	SMAD9
ENSXETG00000023691	7146	3	9	4	60	51	36	65	59	34	86	81	101	ENSXETG00000023691
ENSXETG00000023695	4604	849	1146	1034	1522	1187	1570	2092	2300	1516	1849	1329	1451	cdk12
ENSXETG00000023696	4909	573	772	666	1269	944	1098	1543	1680	1052	1662	1203	1268	med1
ENSXETG00000023700	1293	74	121	91	400	287	619	523	632	417	416	298	297	fbxl20
ENSXETG00000023707	2229	0	2	0	0	3	4	42	71	42	8	6	7	ubp1
ENSXETG00000023709	1149	0	1	0	1	2	2	99	64	56	5	7	6	stac2
ENSXETG00000023710	1138	0	0	0	0	0	0	1	2	0	0	0	0	
ENSXETG00000023711	692	41127	61151	52551	65064	51651	71148	70764	79505	46716	170996	121356	128158	rpl19
ENSXETG00000023713	585	47	72	62	45	48	248	410	450	293	119	72	83	arl5c
ENSXETG00000023714	4080	94	131	92	279	200	311	454	500	378	475	297	376	ENSXETG00000023714
ENSXETG00000023716	869	1162	1709	1384	2396	1839	2523	3545	3930	2498	2043	1381	1505	lasp1
ENSXETG00000023717	2594	11	9	25	93	63	229	582	659	391	198	115	136	erg
ENSXETG00000023718	1755	152	223	186	745	478	1191	1031	1061	635	1022	727	730	shoc2
ENSXETG00000023719	327	27442	41472	36386	43497	33831	45546	44130	47660	29756	110003	76854	82502	rpl23
ENSXETG00000023720	588	4	3	6	4	0	11	5	3	6	8	8	4	c17orf98
ENSXETG00000023721	1285	481	697	586	493	339	498	786	950	582	680	502	531	rbm34
ENSXETG00000023722	1964	460	613	523	483	414	738	1081	1249	673	750	531	559	cwc25
ENSXETG00000023724	2787	2073	2922	2537	3023	2321	3157	7161	8092	5094	5029	3465	3979	ets2
ENSXETG00000023725	3760	177	247	239	410	358	661	2101	2496	1497	893	684	702	pip4k2a
ENSXETG00000023728	5031	195	290	237	395	297	492	407	504	301	542	425	444	brwd1
ENSXETG00000023730	1493	340	461	386	829	680	2098	1737	2103	1155	1358	930	1069	stx3
ENSXETG00000023735	1026	938	1288	1082	1235	865	1208	2091	2282	1335	1054	723	844	psmg1
ENSXETG00000023736	2442	0	0	0	1	4	2	11	18	8	6	5	6	tmem132d
ENSXETG00000023738	696	0	0	1	1	2	3	2	3	3	0	4	1	casq2
ENSXETG00000023739	718	362	502	470	329	288	750	675	792	524	600	423	482	wrb
ENSXETG00000023741	1093	74	84	89	117	95	119	119	160	87	153	122	128	glt1d1
ENSXETG00000023742	1698	89	128	110	201	206	605	883	1083	617	356	226	287	slc15a4
ENSXETG00000023744	2961	44	58	53	191	151	154	1027	1229	735	252	170	227	ptp4a3
ENSXETG00000023746	1236	0	0	0	0	0	0	2	1	3	0	0	0	
ENSXETG00000023747	4773	0	0	0	4	7	11	120	133	87	17	10	15	ENSXETG00000023747
ENSXETG00000023748	1197	1	0	0	0	0	0	2	1	0	0	0	0	
ENSXETG00000023749	891	4	3	6	69	44	153	916	1046	560	285	230	242	gpr20
ENSXETG00000023750	2028	223	327	310	320	254	951	2332	2623	1670	558	355	436	slc45a4
ENSXETG00000023751	3103	31	50	38	147	122	368	1237	1392	827	371	270	284	pald1
ENSXETG00000023752	3264	1	3	0	17	10	12	41	46	21	8	4	4	adamts14
ENSXETG00000023753	3741	75	127	104	211	123	385	566	591	332	328	231	250	dennd3
ENSXETG00000023754	438	50808	76445	65580	83153	62470	88682	94504	104622	67590	201251	143313	149148	rplp1
ENSXETG00000023755	3652	137	193	199	227	165	432	503	571	347	333	207	225	ago2
ENSXETG00000023757	3567	17918	25108	21040	10852	8312	12120	4896	6278	3537	13271	9293	10854	scd
ENSXETG00000023760	1296	0	0	0	0	0	0	0	0	0	0	0	0	
ENSXETG00000023763	1302	1	1	2	0	0	1	0	0	1	0	0	0	
ENSXETG00000023764	3069	286	442	314	741	602	1093	1312	1511	954	1110	766	854	ptk2
ENSXETG00000023767	3036	108	179	155	307	236	393	508	525	364	336	262	293	trappc9
ENSXETG00000023771	1899	1	2	1	2	0	0	15	17	6	2	1	2	kcnd1
ENSXETG00000023776	2522	5711	7979	6805	13322	10124	50735	12757	14802	8788	73040	50068	57360	slc13a2
ENSXETG00000023781	752	6	10	15	262	182	76	77	77	63	35	35	36	c2orf50
ENSXETG00000023782	4215	654	879	844	1363	1052	2038	2203	2533	1425	1701	1157	1347	rock2
ENSXETG00000023785	2176	2069	2926	2453	1724	1211	3462	1551	1879	1084	1343	912	1042	slc46a1
ENSXETG00000023789	1457	5	6	6	0	0	1	9	18	7	2	5	3	vtn
ENSXETG00000023792	1410	881	1295	1103	1932	1554	3392	1098	1344	761	2995	2144	2407	poldip2
ENSXETG00000023793	7583	0	2	2	4	4	14	56	64	48	11	9	22	greb1
ENSXETG00000023794	576	0	0	0	0	0	0	0	0	0	0	0	0	
ENSXETG00000023795	784	709	951	865	621	472	936	916	1070	650	680	449	512	tmem199
ENSXETG00000023796	1509	201	305	257	400	280	512	279	274	187	367	238	267	srr
ENSXETG00000023797	2590	750	1101	963	894	689	1212	1456	1723	1025	1522	1116	1231	tsr1
ENSXETG00000023798	816	3	5	3	30	23	96	4450	4831	3142	284	197	222	clu
ENSXETG00000023799	4080	82	146	124	586	454	1045	673	828	467	923	715	724	SMG6
ENSXETG00000023803	1929	39	56	61	93	72	218	1698	1998	1151	298	238	227	hic1
ENSXETG00000023804	1496	18	40	22	149	121	166	303	361	253	259	163	201	gfra1
ENSXETG00000023806	2859	0	1	1	15	11	25	241	263	175	30	17	20	atrnl1
ENSXETG00000023808	4491	228	350	373	896	755	1092	1567	1736	1077	1657	1084	1215	arhgef11
ENSXETG00000023811	3145	4	7	4	73	36	37	178	239	125	114	113	136	atp1a3
ENSXETG00000023813	1620	1	1	1	0	0	3	1	0	0	2	0	0	XB-GENE-5875042

ENSXETG00000023814	999	0	0	0	1	0	0	1	1	0	0	0	0	angpt4
ENSXETG00000023815	788	43	57	51	106	65	508	37	32	18	272	193	245	socs2
ENSXETG00000023817	4020	28	34	33	37	35	120	694	961	527	127	80	95	plxnc1
ENSXETG00000023819	760	832	1118	997	521	397	487	535	661	436	291	214	227	ovca2
ENSXETG00000023822	1019	1116	1604	1363	1826	1360	1037	861	967	564	589	434	432	ENSXETG00000023822
ENSXETG00000023826	3597	129	209	147	565	520	364	736	949	510	172	120	137	ncapg
ENSXETG00000023828	1366	501	732	615	560	372	398	843	926	569	441	394	352	trub1
ENSXETG00000023829	2573	57	67	65	350	281	202	296	363	225	110	80	85	cep83
ENSXETG00000023831	2488	131	176	153	411	327	533	796	862	497	724	464	519	fam160b1
ENSXETG00000023832	3393	94	147	110	303	216	561	728	816	498	791	554	662	tmcc3
ENSXETG00000023834	1173	0	0	0	0	0	0	0	0	0	0	0	0	
ENSXETG00000023839	528	1035	1595	1272	1159	808	913	1217	1406	815	601	407	428	ndufa12
ENSXETG00000023840	3474	128	172	158	269	172	451	434	577	308	463	313	359	nr2c1
ENSXETG00000023845	2232	273	347	292	574	409	576	784	911	507	846	635	675	pgam5
ENSXETG00000023848	2739	185	231	182	418	350	531	765	873	517	749	587	613	ankle2
ENSXETG00000023849	4188	309	380	359	433	316	528	1006	1195	693	584	412	419	golga3
ENSXETG00000023850	2481	0	1	0	1	1	3	51	51	27	16	4	9	mdga2
ENSXETG00000023851	2922	480	691	610	1682	1353	2220	1294	1367	846	2745	1955	2100	fbrsl1
ENSXETG00000023853	3220	628	874	782	691	577	1010	1097	1217	700	688	484	585	vezt
ENSXETG00000023854	1587	633	918	747	597	432	465	1661	1970	1102	546	394	418	rabac1
ENSXETG00000023857	816	27	40	34	21	20	71	19	16	9	14	5	10	ENSXETG00000023857
ENSXETG00000023859	1416	175	269	226	352	256	397	517	655	394	422	332	312	ENSXETG00000023859
ENSXETG00000023861	5456	83	108	106	137	124	120	256	312	174	114	76	82	c5orf30
ENSXETG00000023862	5748	129	161	124	431	355	373	814	1025	537	573	383	449	ppip5k2
ENSXETG00000023863	3917	3249	4879	4195	4878	3757	7654	7681	8641	4942	7442	5367	5809	pam
ENSXETG00000023865	1911	2111	2895	2469	1776	1403	4838	49	75	40	1334	968	1044	slco4c1
ENSXETG00000023866	1951	1858	2752	2315	1358	1083	1579	1331	1411	864	1005	728	766	tbrg4
ENSXETG00000023867	910	5	10	6	5	8	10	69	65	40	26	13	18	naca
ENSXETG00000023870	1080	4	17	9	15	6	27	119	170	98	46	19	29	st8sia4
ENSXETG00000023871	1166	209	333	265	271	198	365	604	729	436	336	237	297	anapc15
ENSXETG00000023872	2774	8	17	5	72	49	128	2325	2569	1608	161	96	128	ngfr
ENSXETG00000023873	717	0	0	0	0	0	0	0	0	0	0	0	0	
ENSXETG00000023875	5518	373	553	403	913	687	1194	1326	1569	889	1341	935	981	chd1
ENSXETG00000023876	7620	339	485	438	961	724	1119	1938	2194	1368	1645	1250	1414	setd2
ENSXETG00000023878	1269	55	86	50	248	183	78	161	184	110	160	126	130	tubz1
ENSXETG00000023880	1940	20	22	20	72	60	109	312	327	222	147	109	124	rgmb
ENSXETG00000023881	2406	22	28	28	284	215	100	91	105	57	62	50	40	kif9
ENSXETG00000023885	1431	496	758	616	819	670	1766	1461	1751	965	1001	678	801	commd4
ENSXETG00000023886	1327	433	583	581	845	633	665	562	591	348	712	547	581	neil1
ENSXETG00000023887	3222	366	491	421	861	587	1069	681	770	425	862	635	651	man2c1
ENSXETG00000023888	1953	3192	4519	3892	3746	2952	6815	245	300	175	1149	719	886	slc27a3
ENSXETG00000023889	2281	552	735	601	434	351	1531	387	453	264	621	463	521	hykk
ENSXETG00000023890	1904	23	22	28	34	30	70	1800	2285	1258	128	71	118	ctsl
ENSXETG00000023891	975	0	1	0	1	0	1	9	12	4	15	9	12	mas1l
ENSXETG00000023893	983	1137	1624	1409	925	731	927	1040	1293	737	648	488	513	mrpl18
ENSXETG00000023896	1730	18	19	17	17	16	26	57	66	45	50	35	31	chrna5
ENSXETG00000023897	1198	1226	1785	1531	1330	987	1445	636	829	461	1046	733	825	sord
ENSXETG00000023898	4650	4181	5941	4937	803	559	3679	1242	1622	875	507	364	374	duox2
ENSXETG00000023900	2277	1	0	0	5	4	3	23	22	16	2	0	0	duoxa1
ENSXETG00000023902	1485	27	36	26	26	19	143	434	475	303	118	83	88	slc2a3
ENSXETG00000023904	8612	21	38	38	121	92	174	404	437	285	391	287	275	ENSXETG00000023904
ENSXETG00000023905	606	0	0	0	0	0	1	1	2	0	2	1	1	aicda
ENSXETG00000023907	1095	8	19	19	30	30	35	280	327	220	46	34	38	c3ar1
ENSXETG00000023908	246	115	166	119	188	162	233	427	489	306	349	259	273	c12orf57
ENSXETG00000023910	2029	826	1167	1010	1441	1103	2354	1001	1023	668	1096	764	817	pex5
ENSXETG00000023912	2540	116	139	134	345	235	653	1962	2435	1379	803	573	579	c1s
ENSXETG00000023913	1464	2601	3912	3159	3360	2483	2763	2472	2887	1823	1937	1409	1579	lpcat3
ENSXETG00000023914	2151	40	68	58	244	198	392	1540	1655	994	394	256	304	ENSXETG00000023914
ENSXETG00000023915	408	8	14	11	17	8	11	6	4	5	32	18	27	rbp5
ENSXETG00000023916	4064	10	6	11	78	52	97	216	223	155	159	131	120	clstn3
ENSXETG00000023917	618	545	827	675	475	381	451	554	648	396	590	426	455	emg1
ENSXETG00000023918	2283	3318	4861	4078	5312	4181	9396	5062	5676	3369	7552	5433	5881	ptpn6
ENSXETG00000023919	899	3191	4739	4220	4074	3224	3739	3655	4227	2616	4158	2953	3096	phb2
ENSXETG00000023920	990	0	0	0	1	0	0	1	1	0	0	0	0	
ENSXETG00000023922	3333	33	57	27	175	132	350	586	584	353	436	339	331	atn1
ENSXETG00000023923	1555	125	179	160	572	437	403	254	262	184	199	97	107	lrrc23
ENSXETG00000023924	1006	8157	11977	10543	8989	6848	8524	10709	11779	7482	6395	4657	5015	tpi1
ENSXETG00000023925	3094	1352	1983	1667	2966	2259	2395	4434	5015	3126	1679	1190	1291	usp5
ENSXETG00000023927	1842	35	49	36	113	86	214	318	354	216	263	200	205	ENSXETG00000023927
ENSXETG00000023928	4053	71	117	97	217	195	436	507	498	303	539	399	430	ENSXETG00000023928
ENSXETG00000023930	1365	20	44	32	61	43	39	30	27	17	139	117	154	ENSXETG00000023930
ENSXETG00000023932	4687	135	178	169	380	301	1150	633	731	395	1160	882	864	xlcof28
ENSXETG00000023933	2505	14	40	29	126	90	245	162	193	102	229	158	196	zbed6
ENSXETG00000023935	1804	2709	4058	3425	3657	2851	2994	3493	3872	2640	2103	1442	1576	rnf121
ENSXETG00000023937	1209	2	0	0	0	0	0	0	2	0	0	0	0	
ENSXETG00000023938	5148	114	144	127	817	663	1534	5491	6365	3690	4710	3290	3682	tnc
ENSXETG00000023940	5568	0	0	0	0	1	1	2	0	1	2	0	0	ENSXETG00000023940
ENSXETG00000023941	2453	148	171	150	322	246	565	659	782	497	216	150	171	ENSXETG00000023941
ENSXETG00000023942	1881	174	270	211	594	444	1236	2388	2715	1629	1317	992	1000	nt5dc2
ENSXETG00000023958	1566	0	0	0	6	1	10	65	67	33	53	34	57	lrrtm1
ENSXETG00000023960	705	0	1	0	1	0	2	4	6	1	1	0	0	ENSXETG00000023960
ENSXETG00000023963	432	41	73	57	112	92	99	191	175	147	52	42	43	tmem216
ENSXETG00000023966	1875	72	130	91	228	204	321	784	806	521	252	173	194	sfrpx
ENSXETG00000023967	801	5	3	7	17	11	15	27	28	20	48	32	29	LRTOMT
ENSXETG00000023968	1338	5766	8849	7559	7054	5162	11207	1220	1370	824	9608	6951	7394	folr1
ENSXETG00000023969	2131	62	114	76	303	274	167	754	875	544	63	41	51	ENSXETG00000023969
ENSXETG00000023970	2424	83	155	122	144	110	497	541	647	383	563	395	466	ENSXETG00000023970
ENSXETG00000023971	1968	1	1	1	2	4	4	12	21	10	6	5	9	nlgn2
ENSXETG00000023972	2325	374	544	482	765	576	2465	1035	1153	716	920	770	801	ENSXETG00000023972
ENSXETG00000023975	2770	61	65	70	94	84	404	368	425	245	203	136	161	ENSXETG00000023975
ENSXETG00000023978	1124	540	794	688	524	401	593	1208	1357	776	566	412	463	arf6
ENSXETG00000023980	768	5	6	1	16	3	16	65	81	43	11	5	8	ENSXETG00000023980
ENSXETG00000023985	3624	221	277	226	380	311	633	1059	1328	761	594	398	476	znf644
ENSXETG00000023986	1086	0	0	0	1	0	0	0	0	0	0	0	0	
ENSXETG00000023987	2595	0	1	0	4	9	1	12	7	9	2	5	3	slitrk5
ENSXETG00000023988	3546	121	170	183	283	199	500	332	346	220	754	536	571	zfpm1
ENSXETG00000023989	1203	4	3	5	2	1	3	1	3	3	0	0	0	trhr2
ENSXETG00000023990	939	0	0	0	0	0	1	1	0	0	2	1	0	
ENSXETG00000023995	1592	74	83	73	169	99	133	295	338	212	174	141	151	znf84
ENSXETG00000023996	1209	0	0	0	0	0	0	0	0	0	0	0	0	
ENSXETG00000023997	3024	0	0	0	0	0	0	1	0	0	1	0	0	ENSXETG00000023997
ENSXETG00000023998	663	1	3	2	1	0	4	3	3	4	3	1	2	ENSXETG00000023998
ENSXETG00000024001	4776	40	57	64	102	65	253	955	1049	670	328	228	244	prex1
ENSXETG00000024002	1152	86	129	91	89	61	153	231	245	171	334	215	203	b4galt6
ENSXETG00000024003	1887	217	298	240	352	275	628	298	315	188	428	297	340	slc9a8
ENSXETG00000024004	870	23	46	36	77	75	65	262	348	227	148	103	119	tmem189
ENSXETG00000024006	1773	7	8	8	53	49	85	2073	2409	1373	268	206	226	snai2
ENSXETG00000024007	873	269	424	342	300	252	547	1018	1230	673	493	310	323	rnf114
ENSXETG00000024008	1932	409	596	504	890	689	1318	1109	1306	827	1018	679	813	spata2
ENSXETG00000024009	1434	9	12	7	13	14	40	152	196	117	38	26	25	HCK
ENSXETG00000024010	1289	345	533	454	455	369	833	701	829	508	602	434	424	tbc1d20.1

ENSXETG00000024011	735	0	0	0	1	2	0	14	30	14	5	6	1	sox12
ENSXETG00000024013	1502	42	54	47	268	193	529	422	484	294	933	630	607	rem1
ENSXETG00000024014	1053	2700	3784	3096	2329	1752	1707	2825	3135	1801	1350	934	968	hm13
ENSXETG00000024015	2232	515	830	663	898	608	1178	938	1050	644	762	543	589	gpd2
ENSXETG00000024016	1572	0	0	0	0	0	0	0	0	1	0	0	0	nr4a2
ENSXETG00000024034	1316	179	231	203	150	111	193	311	361	189	240	145	168	cnep1r1
ENSXETG00000024035	3189	0	1	1	2	3	9	17	22	16	3	3	7	nptx2
ENSXETG00000024036	1626	751	1122	896	1932	1558	2636	2160	2259	1439	3325	2227	2697	baiap2l1
ENSXETG00000024037	1137	6030	8884	7926	6755	5139	28275	7664	8659	5405	6397	4364	4947	bri3
ENSXETG00000024038	7050	139	230	202	637	474	1685	964	1035	663	762	540	663	tecpr1
ENSXETG00000024039	4161	380	507	453	892	674	1916	1266	1519	856	2092	1467	1768	lmtk2
ENSXETG00000024043	671	0	0	2	1	0	0	10	21	8	2	0	0	pvalb.1
ENSXETG00000024046	767	2	1	0	0	0	1	1	1	1	0	0	0	XB-GENE-5936750
ENSXETG00000024049	1032	0	0	0	1	0	0	0	0	0	0	0	0	ocm
ENSXETG00000024051	753	0	0	0	0	0	0	0	0	0	0	0	0	
ENSXETG00000024053	1786	404	597	485	325	284	488	490	579	318	296	220	234	ccz1b
ENSXETG00000024054	2514	2	3	4	139	87	13	22	16	8	16	11	9	rsph10b
ENSXETG00000024055	1764	224	357	310	505	381	818	602	678	363	1144	842	882	eif2ak1
ENSXETG00000024057	1038	236	332	314	291	247	518	568	626	376	500	351	364	prpf18
ENSXETG00000024058	1559	29624	42369	36069	44218	33339	68685	6455	7914	4489	33814	24107	26393	aldob
ENSXETG00000024063	4219	64	62	88	257	185	402	593	689	389	579	425	484	cyth3
ENSXETG00000024065	1770	77	102	84	154	102	247	334	394	232	321	267	331	usp42
ENSXETG00000024066	2553	114	141	109	201	151	197	350	357	219	198	153	161	pms2
ENSXETG00000024067	3522	3	4	5	82	58	39	7	1	1	104	82	62	otoa
ENSXETG00000024069	3228	294	433	386	581	472	612	849	971	556	587	421	507	dhx16
ENSXETG00000024070	2038	476	726	543	960	779	1442	1017	1290	692	762	539	633	abhd16a
ENSXETG00000024072	2433	0	1	0	2	0	3	4	2	0	1	0	0	msh5
ENSXETG00000024073	1408	1666	2337	1960	2622	2050	2902	5827	7132	4029	3935	2745	3163	clic1
ENSXETG00000024074	1200	37	40	50	54	42	87	613	702	412	134	111	105	ddah2
ENSXETG00000024076	3765	1451	2189	1783	2550	1997	1937	3029	3396	1953	2665	1898	2096	vars
ENSXETG00000024077	635	488	688	548	354	314	307	804	917	499	185	134	153	lsm2
ENSXETG00000024078	2938	163	257	219	141	107	37	173	225	111	29	28	42	hsp70
ENSXETG00000024079	999	14	16	13	35	27	8	56	50	29	17	8	13	tcf19
ENSXETG00000024080	3036	100	145	112	204	174	279	359	477	245	322	215	265	cchcr1
ENSXETG00000024084	2445	5481	7919	6719	6134	4610	4427	24840	29495	17521	4394	3114	3483	tubb
ENSXETG00000024085	3371	970	1357	1177	2374	1816	3024	4578	5345	3037	3137	2289	2563	flot1
ENSXETG00000024086	2709	322	495	476	1221	946	1198	1281	1472	871	1563	1152	1205	ddr1
ENSXETG00000024087	3661	360	492	393	480	311	846	976	1112	738	715	535	594	gnl1
ENSXETG00000024088	2571	1238	1923	1589	1748	1256	2157	4544	5065	3064	1499	1043	1129	abcf1
ENSXETG00000024089	1669	24	44	28	70	76	130	390	491	253	244	176	187	atat1
ENSXETG00000024094	2598	24	25	22	24	20	29	237	283	152	263	179	212	ciita
ENSXETG00000024095	3134	275	448	367	631	445	1213	1223	1399	811	748	578	687	clec16a
ENSXETG00000024097	2264	134	207	165	237	192	188	511	637	342	327	230	261	larp7
ENSXETG00000024098	2508	9	9	10	22	14	18	57	42	39	22	17	17	zgrf1
ENSXETG00000024099	2508	0	0	0	0	0	0	3	1	3	0	0	0	
ENSXETG00000024100	642	0	0	0	1	1	0	1	0	1	1	1	1	
ENSXETG00000024103	4510	66	88	77	180	158	623	414	469	301	613	416	485	alpk1
ENSXETG00000024105	2533	3	6	5	17	11	37	200	223	122	52	36	41	negr1
ENSXETG00000024108	2143	478	664	568	626	456	826	1325	1531	830	981	621	713	zranb2
ENSXETG00000024109	4398	31	46	31	82	83	122	432	539	317	181	132	153	tifa
ENSXETG00000024110	1311	120	157	165	484	379	724	1192	1379	761	368	260	266	cfi
ENSXETG00000024111	3093	3703	5309	4402	2634	2159	5178	4319	5183	2916	3558	2620	3002	casp6
ENSXETG00000024112	468	371	512	409	483	325	893	531	557	329	385	269	288	pla2g12a
ENSXETG00000024113	1137	1	2	1	16	13	12	6	9	3	65	42	41	rrh
ENSXETG00000024114	1181	699	1042	840	901	705	842	1432	1719	953	774	566	629	gar1
ENSXETG00000024115	1926	5	8	4	5	1	26	2	5	1	14	6	6	lrit3
ENSXETG00000024116	2179	277	341	323	405	344	903	997	1249	733	491	318	396	scarb2
ENSXETG00000024117	2004	462	660	517	568	447	535	993	1237	720	472	311	357	nup54
ENSXETG00000024118	974	19	24	30	56	33	63	147	149	80	106	93	83	ENSXETG00000024118
ENSXETG00000024121	1059	72	90	96	162	148	288	292	318	225	296	194	248	kdsr
ENSXETG00000024124	573	4	1	4	5	8	14	79	102	62	47	18	39	bcl2
ENSXETG00000024127	2859	21	19	17	9	5	20	26	21	22	32	24	31	st8sia2
ENSXETG00000024129	4785	366	511	425	1177	932	1111	1673	1994	1161	1793	1231	1351	chd2
ENSXETG00000024133	3897	540	748	628	1502	1105	2293	1231	1473	804	1623	1190	1291	kiaa1468
ENSXETG00000024138	1489	270	413	353	901	708	1519	1865	1990	1228	1486	1071	1176	rgma
ENSXETG00000024139	2157	0	0	0	0	0	0	0	0	0	1	0	0	
ENSXETG00000024140	2643	264	386	340	402	302	521	489	617	333	856	587	660	mctp2
ENSXETG00000024143	1539	177	257	204	136	99	213	204	234	132	95	88	111	aptx
ENSXETG00000024144	1614	2062	3064	2714	2794	2048	3241	4085	4835	2689	2493	1901	2128	dnaja1
ENSXETG00000024145	1700	715	1007	905	1185	910	1428	1548	1936	1008	1089	800	889	smu1
ENSXETG00000024146	1954	296	415	345	225	157	524	147	185	104	247	184	191	b4galt1.2
ENSXETG00000024147	4028	1266	1703	1338	1373	1117	1738	1929	2374	1318	1310	1043	1060	b4galt1.1
ENSXETG00000024149	678	19	23	30	41	30	76	446	532	309	80	46	74	ebi3
ENSXETG00000024150	2868	1	1	0	0	2	1	1	2	1	0	0	0	ankrd24
ENSXETG00000024151	1910	0	0	0	0	0	0	5	3	2	0	1	1	shd
ENSXETG00000024154	870	1323	1908	1741	3151	2324	5191	1336	1575	946	5653	4041	4673	stap2
ENSXETG00000024155	3520	63	107	82	38	48	150	9	11	6	103	79	88	mpnd
ENSXETG00000024157	2691	315	486	415	849	669	259	1170	1333	815	327	226	219	chaf1a
ENSXETG00000024158	1824	571	822	692	833	693	1661	1253	1472	897	1398	962	1102	ubxn6
ENSXETG00000024159	2263	396	598	501	665	534	844	1543	1787	1010	804	648	642	hdgfrp2
ENSXETG00000024160	1636	1314	1861	1720	1023	754	1641	1049	1155	731	863	607	621	pex11b
ENSXETG00000024161	2179	418	613	536	2108	1607	2779	7762	8362	5650	3022	2169	2235	lmna
ENSXETG00000024163	2070	35	67	46	110	100	118	683	766	537	298	258	248	XB-GENE-5994092
ENSXETG00000024167	1260	4104	5623	4882	2992	2411	3357	3411	3918	2362	1928	1336	1517	plin3
ENSXETG00000024172	1290	10	21	19	127	99	35	83	109	64	25	20	24	ENSXETG00000024172
ENSXETG00000024177	4541	722	977	874	1800	1422	4513	1685	1906	1279	2658	1779	2030	kalrn
ENSXETG00000024182	2573	254	398	364	815	608	1089	905	1014	605	940	675	734	epb41l5
ENSXETG00000024183	2677	313	472	367	1132	806	1853	1263	1423	936	2226	1493	1655	ptpn4
ENSXETG00000024184	786	1367	2085	1801	2205	1711	5787	6525	7010	5155	2924	1958	2200	ctsz
ENSXETG00000024186	1395	2	6	5	69	42	135	146	117	101	120	92	88	TUBB1
ENSXETG00000024187	960	340	419	353	580	415	510	776	827	540	539	343	363	nelfcd
ENSXETG00000024188	1315	273	395	339	637	515	690	1212	1360	814	806	554	592	gnas
ENSXETG00000024189	708	1	1	1	0	0	1	0	0	0	1	0	0	ENSXETG00000024189
ENSXETG00000024193	1263	11	13	12	53	50	75	140	151	102	177	144	117	elk1
ENSXETG00000024194	1824	4	9	16	34	34	83	830	871	572	91	42	44	ENSXETG00000024194
ENSXETG00000024196	1755	503	653	600	1215	963	2082	3070	3249	2074	2098	1464	1663	swap70
ENSXETG00000024197	885	0	0	0	0	0	0	0	0	0	0	0	0	
ENSXETG00000024199	3162	369	616	552	773	569	845	1515	1660	1022	1106	852	868	znf143
ENSXETG00000024201	3144	2351	3187	2860	6151	4549	9321	6199	6964	4304	12951	9156	9968	ipo7
ENSXETG00000024202	2142	86	112	95	424	337	429	650	763	416	834	613	662	ENSXETG00000024202
ENSXETG00000024203	2044	439	655	536	1137	944	1356	2145	2391	1479	1409	1021	1056	btbd10
ENSXETG00000024204	1305	224	380	303	244	150	176	593	657	392	141	95	127	arntl
ENSXETG00000024209	1361	2188	3208	2809	2320	1735	2366	3355	3764	2217	1005	750	805	chchd3
ENSXETG00000024210	882	475	721	630	398	295	398	630	762	475	258	194	222	mrpl16
ENSXETG00000024212	4780	398	581	480	1336	1069	2539	1263	1507	844	2306	1578	1860	chfr
ENSXETG00000024215	2124	90	131	103	237	181	190	379	427	311	354	254	264	ENSXETG00000024215
ENSXETG00000024217	504	0	0	0	0	0	0	1	1	2	0	1	1	ENSXETG00000024217
ENSXETG00000024218	1293	0	0	0	0	0	0	0	0	0	0	0	1	
ENSXETG00000024219	4977	4	4	6	34	17	26	54	50	45	65	58	58	a2m
ENSXETG00000024221	1338	825	1201	995	2044	1547	1685	1840	1950	1249	1377	985	1125	creb3
ENSXETG00000024224	2733	307	402	405	758	550	1196	1558	1767	993	1536	1043	1137	phc1

ENSXETG00000024225	708	521	656	607	1051	830	1342	1581	1721	1117	1603	1153	1189	m6pr
ENSXETG00000024227	4359	1	7	4	14	11	1	16	18	12	66	54	60	pzp
ENSXETG00000024231	1065	0	0	0	0	0	0	1	1	1	0	0	0	
ENSXETG00000024232	513	2	2	2	13	9	5	6	5	3	36	19	22	ENSXETG00000024232
ENSXETG00000024242	2334	23	38	38	47	43	70	64	80	50	113	56	72	ENSXETG00000024242
ENSXETG00000024245	1758	1	8	6	4	4	17	25	26	8	15	14	12	ENSXETG00000024245
ENSXETG00000024246	1266	15	21	20	43	25	35	74	76	41	69	58	51	ENSXETG00000024246
ENSXETG00000024252	564	0	0	0	0	0	0	0	0	0	0	1	0	ENSXETG00000024252
ENSXETG00000024253	2277	43	47	39	66	56	124	114	151	66	125	90	95	ENSXETG00000024253
ENSXETG00000024254	2550	57	66	89	134	123	266	223	328	168	271	189	238	ENSXETG00000024254
ENSXETG00000024263	1161	30	35	26	24	21	64	85	97	56	99	47	57	znf484
ENSXETG00000024279	1260	29	52	39	33	25	37	97	100	55	56	32	59	zkscan7
ENSXETG00000024280	957	0	0	0	0	0	0	0	0	0	0	0	0	
ENSXETG00000024286	933	0	0	0	0	0	0	0	0	0	0	0	0	
ENSXETG00000024287	1527	55	104	97	244	202	198	328	369	229	95	81	82	fah
ENSXETG00000024288	7174	0	0	1	1	1	0	6	8	5	1	0	1	
ENSXETG00000024290	951	0	0	0	0	0	0	0	0	0	0	0	0	
ENSXETG00000024293	525	75	118	103	91	68	185	65	54	31	103	64	78	mthfs
ENSXETG00000024302	3894	4	12	14	30	22	49	253	365	189	62	38	27	atp10a
ENSXETG00000024306	2523	0	0	0	0	0	0	0	0	0	0	0	0	
ENSXETG00000024313	2334	0	0	0	0	0	0	0	0	0	0	0	0	
ENSXETG00000024316	1056	0	0	0	0	1	0	0	0	0	0	0	0	
ENSXETG00000024318	2316	0	0	0	0	0	0	0	0	0	0	0	0	
ENSXETG00000024320	1599	50	68	60	34	31	63	125	157	98	82	58	45	ENSXETG00000024320
ENSXETG00000024321	5455	685	905	715	2710	1975	3634	3811	4420	2563	3764	2661	3005	rab11fip1
ENSXETG00000024325	3663	8	16	9	129	110	346	778	897	557	368	277	271	adgra2
ENSXETG00000024326	2926	413	516	443	593	501	941	1128	1326	802	685	506	585	ube3a
ENSXETG00000024337	2559	0	0	0	1	1	2	5	7	2	4	1	0	znf470
ENSXETG00000024338	2205	1	0	0	1	0	0	13	17	9	10	8	11	cnga3
ENSXETG00000024339	2436	0	0	0	0	0	0	0	0	0	0	0	0	
ENSXETG00000024342	2352	0	0	1	0	0	0	1	0	0	1	0	0	ENSXETG00000024342
ENSXETG00000024343	1762	1358	2000	1718	2393	1781	3462	1721	2035	1249	1760	1305	1525	erlin2
ENSXETG00000024347	2197	84	102	97	546	423	499	1021	1136	730	518	408	402	znf703
ENSXETG00000024348	2337	0	1	0	54	51	11	2	1	0	7	7	7	vwa3b
ENSXETG00000024349	2271	0	0	0	0	0	0	0	0	0	0	0	0	
ENSXETG00000024355	2406	0	0	0	0	0	0	0	0	0	0	0	0	
ENSXETG00000024362	3666	56	100	78	173	116	442	306	357	192	445	303	347	znf568
ENSXETG00000024374	531	2	3	6	15	15	29	29	39	33	21	17	17	ENSXETG00000024374
ENSXETG00000024376	1329	33	31	44	191	140	240	280	295	153	359	231	260	ENSXETG00000024376
ENSXETG00000024377	2535	30	51	63	131	120	268	326	399	201	296	230	221	XB-GENE-5822524
ENSXETG00000024379	1945	0	0	0	0	0	0	0	0	0	0	0	0	
ENSXETG00000024385	2933	0	1	0	0	0	0	5	4	0	0	0	1	uvs.2
ENSXETG00000024390	1918	655	878	746	983	728	1532	2965	3382	2071	1156	793	875	rara
ENSXETG00000024391	774	32	45	36	19	17	62	102	102	76	77	41	66	gjd3
ENSXETG00000024393	2223	460	589	502	704	473	1332	1491	1883	1078	796	570	650	snx3
ENSXETG00000024394	2213	222	354	247	747	555	102	992	1159	593	246	207	189	cdc6
ENSXETG00000024395	2667	494	729	634	1259	933	1406	2081	2372	1503	1115	794	818	casc3
ENSXETG00000024396	2390	211	313	273	1786	1318	1470	1144	1268	764	1687	1126	1263	rapgefl1
ENSXETG00000024397	2939	569	819	642	1204	845	4733	1222	1311	786	2104	1521	1642	nr1d1
ENSXETG00000024399	1909	40	49	39	56	35	78	193	197	116	65	61	65	thra
ENSXETG00000024403	534	0	0	0	2	0	0	4	11	9	0	0	0	csf3
ENSXETG00000024411	2964	203	296	247	588	446	916	1265	1450	878	722	487	537	ammecr1
ENSXETG00000024414	411	1	0	0	1	2	1	179	184	124	1	0	0	dio3
ENSXETG00000024416	1260	0	0	0	0	0	0	0	0	0	0	0	0	
ENSXETG00000024418	741	988	1408	1289	2329	1822	3471	2505	2801	1872	2701	1794	2020	f11r
ENSXETG00000024421	15531	0	0	0	0	0	0	0	0	0	0	0	0	
ENSXETG00000024423	640	36965	54611	46405	38392	28788	31980	23425	25893	16194	22122	15785	16823	atp5g3
ENSXETG00000024424	728	2	3	2	20	14	14	25	24	13	6	7	7	hoxd8
ENSXETG00000024425	489	0	0	0	0	0	0	0	0	0	0	0	0	
ENSXETG00000024426	552	0	0	0	0	0	0	0	0	0	0	0	0	
ENSXETG00000024427	990	0	0	0	0	0	0	0	0	0	0	0	0	
ENSXETG00000024429	5531	476	729	629	1830	1317	1315	1673	2028	1237	1782	1243	1333	rbbp6
ENSXETG00000024430	788	2955	4437	3846	3347	2500	3561	3230	3668	2367	2396	1718	1870	ndufab1
ENSXETG00000024431	1960	1	2	4	157	123	20	5	2	0	17	13	13	foxj1.2
ENSXETG00000024432	447	6	12	14	125	65	124	529	573	334	456	325	317	glis2
ENSXETG00000024434	4655	24	35	21	199	162	218	225	284	159	174	122	147	ift140
ENSXETG00000024437	1043	3	1	4	3	5	4	2	3	2	20	14	9	ENSXETG00000024437
ENSXETG00000024438	1876	291	452	355	489	405	712	1549	1613	1021	858	589	671	pqlc3
ENSXETG00000024439	1462	665	953	882	1094	813	746	1709	1965	1202	969	621	737	rnps1
ENSXETG00000024441	1202	277	382	347	544	476	902	754	805	549	512	316	378	c8orf33
ENSXETG00000024442	1369	4121	5807	4856	4824	3579	13270	11787	13735	8421	6725	4679	5299	atp6v0c
ENSXETG00000024444	801	0	0	0	1	2	0	2	2	0	3	1	2	ENSXETG00000024444
ENSXETG00000024447	1109	63254	96937	83416	120556	91486	146096	120661	136303	83690	304232	210760	232405	rack1
ENSXETG00000024449	1527	1109	1553	1332	1154	924	1708	1989	2293	1335	1118	805	919	ppp1r11
ENSXETG00000024450	552	207	294	248	109	85	278	292	336	194	185	114	147	bloc1s3
ENSXETG00000024451	1620	0	0	0	0	0	0	0	0	0	0	0	0	
ENSXETG00000024452	1552	138	258	199	538	428	373	540	563	339	362	274	276	ccdc61
ENSXETG00000024453	3744	964	1377	1232	2104	1664	4023	2319	2622	1499	3206	2254	2561	sympk
ENSXETG00000024454	1030	27	48	33	86	58	163	877	986	600	364	293	265	rtn2
ENSXETG00000024456	1702	325	472	417	385	348	675	765	931	555	551	453	520	fbxo46
ENSXETG00000024458	2380	0	0	0	0	0	0	0	0	0	0	0	0	
ENSXETG00000024459	1048	1221	1768	1502	1000	801	1164	1380	1507	848	851	608	659	mmgt1
ENSXETG00000024460	252	0	6	3	7	7	2	10	8	4	5	5	1	
ENSXETG00000024466	2391	6	11	3	7	5	31	88	76	48	62	45	32	popdc3
ENSXETG00000024468	681	126	171	135	660	506	960	5506	6064	3767	2223	1516	1855	mfap4
ENSXETG00000024469	729	0	0	0	0	0	0	24	19	10	0	3	0	ENSXETG00000024469
ENSXETG00000024470	762	0	3	0	0	1	0	13	37	18	22	9	12	ENSXETG00000024470
ENSXETG00000024472	1763	6	15	15	19	11	61	151	195	96	36	31	27	c5ar1
ENSXETG00000024473	1098	0	0	0	0	0	0	0	0	0	0	0	0	
ENSXETG00000024476	833	894	1274	1054	1060	854	2138	1593	1955	1069	856	581	625	vps29
ENSXETG00000024477	1386	9	15	18	6	10	39	26	26	12	56	38	44	ENSXETG00000024477
ENSXETG00000024478	567	20072	28769	23124	12380	9020	12087	10628	12464	6838	11092	7979	8460	crip1
ENSXETG00000024479	2410	138	140	142	444	330	1068	737	748	405	575	401	491	tctn1
ENSXETG00000024480	1992	4840	7052	5926	5599	4335	7932	7382	8988	5267	4371	3067	3436	ppp1cc
ENSXETG00000024486	3987	95	138	99	128	100	66	220	293	170	110	92	84	haus6
ENSXETG00000024487	2089	41	52	51	100	85	200	757	880	554	226	155	166	acer2
ENSXETG00000024488	1251	4	2	1	58	42	3	15	14	11	5	3	1	lexm
ENSXETG00000024489	1092	34	49	29	135	93	52	83	111	63	50	33	26	hspb11
ENSXETG00000024490	2265	51064	73916	61944	53731	41109	56824	7526	8971	5034	19049	13500	15007	scp2
ENSXETG00000024492	1197	0	0	0	0	0	0	0	0	0	0	0	0	
ENSXETG00000024496	1329	4154	5968	5134	2732	1996	7337	236	258	146	1455	1055	1129	ENSXETG00000024496
ENSXETG00000024497	1545	1993	2921	2632	2573	1958	3266	30	37	28	410	261	297	ENSXETG00000024497
ENSXETG00000024498	2167	5140	7184	6288	2657	1911	5568	920	1016	592	1585	1162	1227	cyp4b1.2
ENSXETG00000024499	1268	662	1016	825	894	684	995	946	1132	654	1103	777	891	atpaf1
ENSXETG00000024500	1899	21	46	25	42	43	31	5	1	1	15	9	15	faah.1
ENSXETG00000024501	873	203	258	195	101	83	81	103	115	63	65	47	54	nsun4
ENSXETG00000024502	1230	322	461	440	293	220	365	109	118	65	264	196	227	lurap1
ENSXETG00000024505	1458	52	89	79	321	237	259	111	102	67	347	281	264	kbtbd11
ENSXETG00000024506	1049	16	22	31	65	57	106	416	489	271	117	84	107	tdrp
ENSXETG00000024508	1542	0	1	0	8	5	1	4	4	5	0	1	1	ENSXETG00000024508
ENSXETG00000024509	1446	68	81	67	210	136	227	405	422	291	212	147	163	slc25a23

ENSXETG00000024510	794	303	459	388	580	417	1041	1148	1317	757	869	659	683	crb3
ENSXETG00000024511	2154	42	73	67	58	62	124	620	731	434	184	144	148	dennd1c
ENSXETG00000024512	2317	211	286	263	267	201	330	347	436	239	226	179	171	c4orf33
ENSXETG00000024516	1899	522	812	611	737	493	837	1128	1229	756	621	466	498	fyttd1
ENSXETG00000024517	1589	493	684	598	746	598	1581	428	547	317	1175	766	962	XB-GENE-5734925
ENSXETG00000024518	2870	9	6	12	82	73	292	790	988	525	439	332	383	pcdhga3
ENSXETG00000024519	2442	3	4	1	12	2	45	148	241	113	45	39	42	ENSXETG00000024519
ENSXETG00000024520	2346	0	0	0	1	1	0	5	5	2	1	2	0	ENSXETG00000024520
ENSXETG00000024522	11842	0	0	2	2	3	1	17	22	10	1	2	3	ENSXETG00000024522
ENSXETG00000024523	1689	0	0	0	0	0	0	0	0	0	0	0	0	
ENSXETG00000024527	1392	7	7	5	13	11	20	63	82	52	64	45	68	cfap70
ENSXETG00000024528	3321	3	1	3	213	159	36	189	171	109	72	42	36	CFAP70
ENSXETG00000024529	1165	618	926	812	1067	797	1278	483	571	363	641	419	521	cyp7b1
ENSXETG00000024532	2095	288	446	378	384	321	539	620	692	422	575	391	449	pus3
ENSXETG00000024534	1002	2	1	4	1	1	4	21	18	15	2	1	0	mylpf
ENSXETG00000024537	505	0	0	0	0	0	0	0	0	0	0	0	1	ENSXETG00000024537
ENSXETG00000024538	499	1	1	0	0	1	0	1	0	1	0	0	1	ENSXETG00000024538
ENSXETG00000024541	912	215	286	277	224	161	282	326	393	230	224	201	169	mzt1
ENSXETG00000024543	1149	13	18	9	56	48	108	67	72	54	74	62	68	tex9
ENSXETG00000024544	6349	87	98	111	332	268	567	578	698	413	624	413	483	rfx7
ENSXETG00000024550	1077	9	15	13	43	40	67	124	142	93	69	45	99	mycbp2
ENSXETG00000024551	1989	17	22	18	520	389	37	715	868	501	548	370	408	scel
ENSXETG00000024564	6509	103	162	117	458	390	169	294	381	236	145	97	128	brca1
ENSXETG00000024565	617	0	0	0	0	0	0	0	0	0	0	0	0	
ENSXETG00000024567	711	36	58	61	67	48	66	107	108	75	104	75	94	snx21
ENSXETG00000024568	1924	250	340	274	563	455	1139	753	875	539	862	591	697	zswim1
ENSXETG00000024571	1251	0	1	0	2	0	0	1	2	0	1	0	1	ENSXETG00000024571
ENSXETG00000024576	843	0	1	0	0	0	0	0	0	0	0	0	0	
ENSXETG00000024577	1341	63	98	70	169	143	230	416	478	316	370	280	280	cyth2
ENSXETG00000024580	795	0	0	0	0	0	0	0	0	0	0	0	0	
ENSXETG00000024581	804	3	2	1	6	2	15	2	6	1	3	5	1	ENSXETG00000024581
ENSXETG00000024582	1736	1056	1521	1257	976	787	1670	681	800	438	365	261	277	ggact
ENSXETG00000024584	5214	1	12	3	34	21	30	46	74	45	51	35	50	nalcn
ENSXETG00000024586	3973	1	1	2	0	0	1	43	69	37	5	1	2	
ENSXETG00000024588	2124	2055	2804	2348	1116	903	1897	147	175	111	64	36	43	g6pc
ENSXETG00000024589	2293	0	0	0	1	1	0	1	1	0	0	1	0	
ENSXETG00000024594	1056	6	4	2	12	7	18	91	123	55	29	12	28	GPR1
ENSXETG00000024597	862	1079	1707	1403	1099	865	1109	1320	1722	924	919	696	764	XB-GENE-5749774
ENSXETG00000024599	818	792	1193	1045	969	803	1340	1472	1690	927	1249	936	1066	vps25
ENSXETG00000024603	1105	1	1	3	2	2	7	25	29	11	40	27	15	ENSXETG00000024603
ENSXETG00000024605	1206	144	221	204	387	286	591	861	972	558	545	424	397	ENSXETG00000024605
ENSXETG00000024607	675	3	2	1	3	2	5	20	12	16	4	0	4	ENSXETG00000024607
ENSXETG00000024610	522	191	303	275	300	223	417	576	607	356	324	209	228	prkrip1
ENSXETG00000024611	2136	269	416	341	921	707	1045	1152	1381	722	1347	908	1015	epc2
ENSXETG00000024613	927	0	0	0	0	0	0	0	0	0	0	0	0	
ENSXETG00000024614	1275	55	100	75	86	52	165	182	195	119	125	83	101	tbcel
ENSXETG00000024618	2541	58	82	77	86	81	366	378	456	253	206	152	176	rbm24
ENSXETG00000024624	1644	46	43	41	86	52	173	56	98	55	137	128	121	kif13a
ENSXETG00000024625	1102	2423	3393	2917	2361	1793	2545	1982	2567	1422	289	214	279	tpmt
ENSXETG00000024626	608	2966	4498	3752	2484	1823	17680	4661	5386	3130	2275	1602	1693	hbd
ENSXETG00000024627	496	0	0	1	2	1	0	0	0	0	1	0	0	hbe1
ENSXETG00000024628	613	2766	4051	3424	200	147	3955	5267	6376	3682	1358	903	1020	hbg2
ENSXETG00000024630	609	0	5	2	34	21	2	11	15	5	3	1	6	ccdc153
ENSXETG00000024631	1146	97	103	108	115	101	186	293	286	250	203	163	152	hinfp
ENSXETG00000024632	1802	71	116	99	193	174	2275	764	875	487	269	177	213	socs3
ENSXETG00000024633	1014	1209	1849	1560	1155	866	1457	275	350	196	359	250	282	ENSXETG00000024633
ENSXETG00000024634	936	444	667	612	557	398	700	297	347	237	231	176	148	XB-GENE-941700
ENSXETG00000024635	2341	4	11	8	26	20	39	472	542	318	139	94	120	bmp6
ENSXETG00000024640	1107	112	183	170	189	165	509	1994	2216	1378	260	191	220	ENSXETG00000024640
ENSXETG00000024641	1617	453	635	478	653	520	314	6	10	2	81	41	43	elovl2
ENSXETG00000024642	927	1	1	1	6	6	15	147	150	96	32	30	24	fjx1
ENSXETG00000024645	870	0	0	0	0	0	0	0	0	0	0	0	0	
ENSXETG00000024646	714	341	490	387	373	278	408	441	545	328	417	338	310	smco4
ENSXETG00000024648	1248	459	668	574	330	275	761	373	443	265	360	272	319	mocs3
ENSXETG00000024649	2562	1742	2564	2274	4827	3719	4982	4089	4607	2833	4789	3308	3639	irf2bp2
ENSXETG00000024650	886	1431	2044	1784	1916	1384	2370	2071	2412	1356	2981	2116	2240	tomm20
ENSXETG00000024651	4239	146	222	186	264	202	700	776	990	591	622	436	512	arid4b
ENSXETG00000024652	432	170	196	206	210	176	322	463	515	292	379	256	305	nabp2
ENSXETG00000024654	866	30373	45040	38211	31688	24063	62757	60672	69359	40322	49583	34790	38043	myl6
ENSXETG00000024657	1085	1489	2015	1683	1528	1142	1441	2792	2998	1845	1484	1005	1129	sarnp
ENSXETG00000024658	2001	2417	3246	2750	3131	2497	5078	3561	4179	2311	3194	2263	2494	ormdl2
ENSXETG00000024659	525	0	0	0	0	0	0	0	0	0	0	0	0	
ENSXETG00000024660	525	0	0	0	0	0	0	0	0	0	0	0	0	
ENSXETG00000024662	525	0	0	0	0	0	0	0	0	0	0	0	0	
ENSXETG00000024663	510	0	0	0	0	0	0	0	0	0	0	0	0	ENSXETG00000024663
ENSXETG00000024664	1787	7063	9926	7905	5557	4309	9767	12718	15598	8601	5725	4016	4545	arpc5
ENSXETG00000024665	1851	46281	67275	58649	63442	47926	55495	44947	51167	30234	41845	29444	32081	atp5b
ENSXETG00000024667	419	24120	35148	29851	37432	28155	37496	36277	42228	24139	85849	58550	65038	rpl31
ENSXETG00000024668	3456	11	20	20	182	122	193	839	963	545	680	472	546	lamc2
ENSXETG00000024669	1855	0	11	4	51	39	33	23	30	19	61	45	34	rgs16
ENSXETG00000024675	1555	0	0	0	7	5	3	2	2	3	0	1	1	dnaaf3
ENSXETG00000024676	2714	1	4	0	2	3	2	10	8	12	7	2	5	tnni3
ENSXETG00000024677	458	1690	2434	2024	1585	1107	1393	1718	1977	1124	1355	1007	1098	ndufa3
ENSXETG00000024679	3626	629	850	761	1459	1151	3068	707	874	510	1742	1218	1438	spats2
ENSXETG00000024682	2013	211	314	284	637	534	1043	2023	2180	1400	871	656	744	ENSXETG00000024682
ENSXETG00000024683	1247	51	87	77	94	55	118	1531	1799	982	136	82	84	ptgr1.1
ENSXETG00000024684	2103	7383	10356	8457	8269	6427	13784	9657	11726	6710	9569	6757	7446	itm2b
ENSXETG00000024695	1620	13	17	20	51	31	116	537	661	442	703	453	541	adssl1
ENSXETG00000024696	1447	0	2	2	1	1	4	19	29	24	11	4	8	agtr2
ENSXETG00000024697	605	128	204	185	190	140	215	279	317	174	410	239	298	siva1
ENSXETG00000024699	1902	1496	2076	1807	391	295	254	396	437	247	573	379	428	sfrp5
ENSXETG00000024701	1146	0	0	0	0	0	0	0	0	0	0	0	0	
ENSXETG00000024706	1336	0	0	0	0	0	0	0	0	0	0	0	0	
ENSXETG00000024707	930	2	0	1	1	2	0	13	15	9	4	3	6	p2ry8
ENSXETG00000024708	1419	0	0	0	0	0	1	0	0	0	1	0	0	ENSXETG00000024708
ENSXETG00000024709	4982	187	267	222	375	244	525	755	889	483	386	309	344	ENSXETG00000024709
ENSXETG00000024712	1636	3553	5133	4210	5981	4593	7444	4124	5039	2910	6314	4440	5095	XB-GENE-5838770
ENSXETG00000024716	1692	0	0	1	3	0	0	3	0	1	0	0	0	
ENSXETG00000024720	552	194	334	292	374	271	604	723	781	529	819	610	578	usf1
ENSXETG00000024721	1616	1892	2715	2231	1862	1529	1902	2426	2750	1680	1368	949	1087	snrpc
ENSXETG00000024723	609	6017	8977	7654	9133	6933	11356	10229	11899	6742	33372	23617	26216	eif3k
ENSXETG00000024727	960	0	0	0	0	0	0	0	0	0	0	0	0	
ENSXETG00000024728	732	558	836	662	604	412	815	1388	1540	840	870	498	606	ccdc25
ENSXETG00000024732	3984	26	37	24	144	106	255	691	726	457	234	160	196	ENSXETG00000024732
ENSXETG00000024734	1610	1344	1966	1647	1024	788	988	1169	1457	878	508	358	381	mcee
ENSXETG00000024735	1455	11	15	16	190	125	315	429	496	289	1138	756	837	ENSXETG00000024735
ENSXETG00000024739	1354	89	135	99	106	91	224	255	260	173	235	170	179	zdhhc12
ENSXETG00000024742	1758	480	769	635	1516	1165	1977	1803	2119	1298	2009	1378	1562	cdk9
ENSXETG00000024743	2355	12755	18399	15970	10202	7529	13921	8825	9944	5843	11299	7879	8802	sec61a1
ENSXETG00000024744	1014	662	1024	820	500	376	916	781	1000	615	693	495	567	dpm2
ENSXETG00000024745	639	2434	3687	2985	3880	3018	4848	3191	3567	2084	5209	3698	3890	h1fx

ENSXETG00000024746	1625	345	577	443	868	776	542	499	559	290	638	432	521	hmces
ENSXETG00000024747	3708	861	1275	1144	2992	2215	2663	4522	5082	3197	2942	2071	2377	smc1a
ENSXETG00000024748	2281	1185	1738	1532	763	527	1179	624	704	451	329	234	262	ENSXETG00000024748
ENSXETG00000024749	1321	5157	7569	6428	5463	4043	6519	6455	7346	4383	5246	3780	4093	bcap31
ENSXETG00000024752	1175	286	458	370	615	442	407	787	883	496	537	391	396	tipin
ENSXETG00000024753	1643	270	412	337	443	330	619	790	967	563	526	397	479	arih2
ENSXETG00000024754	2074	281	413	325	379	229	717	688	699	398	294	265	255	dolk
ENSXETG00000024755	1135	577	838	760	370	262	269	356	401	280	245	179	197	ndufaf3
ENSXETG00000024756	2642	45	57	50	41	33	42	767	824	502	140	120	119	dnm1
ENSXETG00000024758	2607	27	55	45	250	186	254	251	316	184	95	74	97	odf2
ENSXETG00000024763	999	1	0	0	0	0	0	0	0	1	1	0	0	col20a1
ENSXETG00000024765	1477	343	480	365	769	590	1456	579	673	323	582	445	441	dynlrb2
ENSXETG00000024766	162	491	729	648	364	281	335	546	603	357	271	192	219	cmc2
ENSXETG00000024768	912	0	0	0	0	0	0	0	0	0	0	0	0	
ENSXETG00000024769	942	0	0	0	0	0	0	0	0	0	0	0	0	
ENSXETG00000024770	918	0	0	0	0	0	0	0	0	0	0	0	0	
ENSXETG00000024771	599	1117	1628	1226	643	530	690	1345	1698	942	821	596	660	nop10
ENSXETG00000024773	2120	462	638	544	446	360	489	1879	2329	1292	304	250	236	apex1
ENSXETG00000024774	3245	131	185	179	248	208	285	235	300	160	329	232	266	ENSXETG00000024774
ENSXETG00000024780	901	207	300	261	411	342	450	196	248	162	236	197	207	pxmp2
ENSXETG00000024781	862	1399	1859	1627	884	660	2600	1865	2389	1286	1108	818	915	atp6v0e1
ENSXETG00000024782	508	34097	50340	43179	47161	35722	54287	52539	60182	34525	124798	85610	93344	rpl26
ENSXETG00000024783	609	2	3	0	1	1	4	19	28	11	12	9	9	lif
ENSXETG00000024784	447	3522	5004	4264	2801	2140	2359	2562	2951	1821	1651	1166	1270	uqcr10
ENSXETG00000024785	393	236	336	302	284	196	273	436	456	252	302	218	253	zmat5
ENSXETG00000024786	1197	3	0	3	2	3	8	55	76	48	25	18	15	il13ra2
ENSXETG00000024787	1422	2	1	0	0	0	3	10	18	9	1	0	0	htr2c
ENSXETG00000024790	2262	2	2	0	19	14	39	200	212	144	70	56	55	kif3c
ENSXETG00000024793	960	25	15	19	9	8	28	100	117	77	33	38	24	p2ry10
ENSXETG00000024794	3009	261	320	290	364	280	644	864	998	579	709	501	556	XB-GENE-5831456
ENSXETG00000024796	2639	2378	3272	2840	6150	4849	6406	7690	8341	5088	4982	3433	3745	ewsr1
ENSXETG00000024797	1483	62	99	84	99	70	115	96	95	61	77	45	68	rhbdd3
ENSXETG00000024799	963	0	0	0	0	0	0	0	0	0	0	0	0	
ENSXETG00000024802	1569	11	5	9	32	18	23	37	42	15	30	26	23	ENSXETG00000024802
ENSXETG00000024804	2492	6457	8980	7834	5376	4168	12600	2030	2414	1425	3732	2701	2937	cyp2d6
ENSXETG00000024805	960	0	0	0	0	0	0	0	0	0	0	0	0	
ENSXETG00000024806	1467	7915	11510	10177	14412	10940	13302	26869	30123	17628	22606	16205	17612	atf4
ENSXETG00000024807	231	122	181	126	95	51	118	215	257	141	135	60	86	rps19bp1
ENSXETG00000024810	504	38	56	49	92	79	212	305	296	184	209	158	130	ENSXETG00000024810
ENSXETG00000024814	940	577	880	715	920	647	1594	1014	1117	792	1080	724	880	tmem41b
ENSXETG00000024817	2284	48	42	48	31	21	103	225	285	153	174	126	141	laptm5
ENSXETG00000024818	486	0	0	0	0	0	0	0	0	0	0	0	0	
ENSXETG00000024823	1116	0	0	0	0	0	0	0	0	0	2	0	0	
ENSXETG00000024827	978	0	0	0	1	1	1	0	2	0	2	0	0	ENSXETG00000024827
ENSXETG00000024834	877	177	283	210	233	174	343	424	500	316	411	278	291	kti12
ENSXETG00000024835	375	0	0	0	0	0	0	0	0	0	0	0	0	nipsnap3a
ENSXETG00000024836	494	2313	3170	2697	2103	1566	2321	1146	1381	786	2662	1838	2032	rps27
ENSXETG00000024839	867	0	0	0	0	0	0	0	0	0	0	0	0	
ENSXETG00000024840	1063	47	69	62	215	186	876	519	542	319	515	304	326	c21orf62
ENSXETG00000024842	1098	5	26	6	18	20	13	23	30	13	36	28	35	ENSXETG00000024842
ENSXETG00000024850	1092	0	0	0	0	0	0	0	0	0	0	0	0	
ENSXETG00000024857	1281	0	0	0	0	0	0	0	0	0	0	0	0	
ENSXETG00000024858	1750	2562	3668	3105	1596	1310	5696	2547	3138	1743	2109	1551	1788	cyp2c18
ENSXETG00000024859	842	165	239	212	491	429	487	763	764	523	892	615	677	stxbp6
ENSXETG00000024861	2299	87	160	115	369	286	680	1427	1724	970	852	593	639	loxl1
ENSXETG00000024863	474	0	0	0	0	0	0	0	0	0	0	0	0	ENSXETG00000024863
ENSXETG00000024864	438	0	0	0	0	0	0	0	0	0	1	0	0	
ENSXETG00000024867	934	438	619	468	402	301	370	499	514	315	292	193	197	timm29
ENSXETG00000024869	720	1327	2066	1713	4117	3189	3982	1050	1165	731	1644	1189	1310	sptssa
ENSXETG00000024870	929	0	0	0	0	0	0	0	0	0	2	0	0	
ENSXETG00000024872	631	1029	1554	1250	959	777	1005	1922	2173	1285	789	545	607	bud31
ENSXETG00000024874	2824	1011	1410	1181	3303	2484	3752	7124	7871	4878	3191	2212	2597	nectin2
ENSXETG00000024875	1755	398	542	482	550	383	549	728	983	528	618	479	456	mplkip
ENSXETG00000024880	2174	291	379	337	371	281	985	811	1001	535	889	752	770	natd1
ENSXETG00000024882	967	13	28	38	147	139	310	775	885	508	173	114	138	XB-GENE-5809273
ENSXETG00000024884	309	195	281	234	341	245	325	596	665	379	300	220	235	inip
ENSXETG00000024887	2043	243	324	306	493	379	1439	2223	2695	1617	923	652	765	cdkn1a
ENSXETG00000024889	1907	3	4	4	2	0	1	30	28	15	1	4	3	foxe1
ENSXETG00000024892	1677	159	243	218	956	730	380	168	175	116	154	125	145	clcn1
ENSXETG00000024894	876	0	0	0	0	0	0	0	0	1	0	0	0	
ENSXETG00000024895	1245	71	109	97	71	47	146	240	257	162	143	98	103	socs1
ENSXETG00000024896	1791	378	597	439	521	386	748	823	975	545	419	300	323	litaf
ENSXETG00000024897	2705	1040	1610	1413	1693	1269	1568	853	972	612	1543	1055	1170	ovol2
ENSXETG00000024898	1349	127	193	141	179	136	255	334	393	207	140	99	160	polr3f
ENSXETG00000024901	1986	0	0	0	0	1	1	1	1	0	3	3	3	ENSXETG00000024901
ENSXETG00000024902	1102	533	807	593	328	188	458	250	318	203	108	97	89	nanp
ENSXETG00000024903	660	1	0	1	5	3	1	1036	1321	725	133	86	111	crp.2
ENSXETG00000024904	717	0	0	0	0	0	0	1	4	2	166	119	167	
ENSXETG00000024906	651	164	221	227	140	122	185	205	265	144	148	102	113	c12orf43
ENSXETG00000024908	2082	0	0	0	0	0	0	0	0	0	0	0	0	
ENSXETG00000024909	1031	1148	1681	1474	964	766	1287	1057	1115	662	957	717	742	tmem230
ENSXETG00000024910	1414	0	0	0	9	12	3	2	1	1	40	42	24	fcn3
ENSXETG00000024911	480	72	84	95	45	33	79	176	193	119	61	52	57	tgfa
ENSXETG00000024912	831	0	0	0	0	0	0	0	0	0	0	0	0	
ENSXETG00000024914	919	9	13	9	50	39	23	23	9	6	23	13	12	rab36
ENSXETG00000024916	366	62	88	67	77	74	219	101	162	81	48	35	35	cmtm7
ENSXETG00000024917	864	1	0	0	0	0	0	1	3	1	1	0	1	figla
ENSXETG00000024918	387	1001	1518	1329	988	818	887	2078	2454	1312	1034	728	810	snrpd3
ENSXETG00000024919	354	0	0	0	0	0	0	6	4	1	0	0	0	
ENSXETG00000024920	357	0	0	0	0	0	0	4	3	0	0	0	0	
ENSXETG00000024921	417	0	0	1	1	0	0	2	6	3	1	2	0	ighv4-59
ENSXETG00000024922	351	0	0	0	0	1	1	0	0	0	3	0	1	ENSXETG00000024922
ENSXETG00000024924	1120	87	136	90	360	269	340	1653	1902	1148	390	286	294	XB-GENE-980731
ENSXETG00000024925	1495	0	0	0	3	3	1	7	3	6	2	0	1	ENSXETG00000024925
ENSXETG00000024927	363	0	0	0	0	0	0	20	21	15	20	7	7	ENSXETG00000024927
ENSXETG00000024929	342	0	0	0	0	0	0	29	19	21	3	1	1	XB-GENE-5734556
ENSXETG00000024930	402	0	0	0	0	0	0	9	13	7	2	0	1	ENSXETG00000024930
ENSXETG00000024931	1641	2	1	0	1	2	4	122	127	84	12	11	17	igl
ENSXETG00000024932	685	0	1	0	0	0	0	114	134	75	21	9	17	ENSXETG00000024932
ENSXETG00000024933	1727	895	1398	1096	1400	1116	1360	2642	3066	1817	1253	877	982	lsm4
ENSXETG00000024935	2262	1	4	2	15	16	26	314	335	221	25	16	19	XB-GENE-5772074
ENSXETG00000024937	561	0	0	0	0	0	0	0	0	0	0	0	0	
ENSXETG00000024938	552	2708	4102	3517	3606	2860	2927	4787	5514	3406	3381	2516	2721	tma7
ENSXETG00000024940	1610	64	111	88	519	351	525	1430	1860	1076	611	477	497	ypel1
ENSXETG00000024941	2375	1368	1965	1603	1360	1033	1745	1581	1907	1072	1346	953	974	plekhj1
ENSXETG00000024943	1666	745	1184	1001	1621	1231	1786	2158	2286	1417	1930	1389	1532	sf3a2
ENSXETG00000024946	1623	13	11	11	41	16	48	27	21	12	19	9	12	comt.2
ENSXETG00000024947	1219	369	633	483	825	704	786	1293	1460	814	1207	842	897	tssc4
ENSXETG00000024949	1401	332	441	372	1285	1042	1798	6185	6912	4296	2539	1790	1965	cd81
ENSXETG00000024951	3606	7	15	18	129	103	115	756	842	477	154	109	128	ENSXETG00000024951
ENSXETG00000024952	1111	2	4	8	6	6	8	21	19	11	1	0	1	tnnt3

ENSXETG00000024953	981	263	414	346	470	377	2854	8394	9782	5955	747	554	629	ENSXETG00000024953
ENSXETG00000024954	999	3	0	5	0	0	3	10	19	10	4	2	2	ENSXETG00000024954
ENSXETG00000024956	1656	3407	4935	4331	5610	4310	12385	6619	7433	4467	8090	5664	6310	ctsd
ENSXETG00000024957	1479	1326	1945	1585	3225	2554	4221	2766	3075	1881	4395	3161	3284	mob2.1
ENSXETG00000024962	1206	0	0	0	0	0	0	0	0	0	0	0	0	
ENSXETG00000024963	1608	2272	3408	2875	3876	3114	6239	6094	6799	4348	5936	4214	4438	lamtor1
ENSXETG00000024964	1461	0	0	0	0	0	0	0	0	0	0	1	0	
ENSXETG00000024965	1227	0	0	0	0	0	0	0	0	0	1	0	0	ENSXETG00000024965
ENSXETG00000024966	950	70	97	79	94	70	260	817	942	537	291	171	193	vsir
ENSXETG00000024985	1277	1660	2357	2003	1746	1354	2642	2960	3531	2014	2146	1546	1648	emc10
ENSXETG00000024988	357	8	15	14	16	10	24	31	49	38	5	1	3	fam72a
ENSXETG00000024989	1421	9	25	14	9	7	34	100	144	92	50	30	39	cxcr3
ENSXETG00000024990	1241	0	0	0	0	0	0	3	2	1	0	0	0	
ENSXETG00000024994	1551	2019	3016	2488	3887	3000	6722	3840	4301	2591	3748	2711	2903	ubxn4
ENSXETG00000024999	1858	6235	9181	7851	12476	9482	21769	13816	15833	9895	32087	22387	25023	dnaja2
ENSXETG00000025004	2014	547	777	709	845	622	1366	1336	1692	925	805	556	689	itfg1
ENSXETG00000025005	1616	7	6	7	51	45	84	326	363	234	174	121	151	sox17a
ENSXETG00000025007	3978	238	299	267	439	362	580	766	907	522	511	329	430	n4bp1
ENSXETG00000025008	1398	43	49	63	152	175	147	184	229	149	38	33	26	nek2
ENSXETG00000025009	1397	10823	15549	13250	8749	6702	10616	26183	30935	17686	11666	8256	9290	myl12b
ENSXETG00000025010	1997	126	154	127	369	255	364	2136	2307	1400	904	688	778	tgif1
ENSXETG00000025012	1130	99	171	116	178	140	176	215	227	140	188	127	138	nhej1
ENSXETG00000025014	1416	1	0	0	3	1	3	11	27	12	5	1	6	tmem200c
ENSXETG00000025015	1053	0	0	0	1	3	0	0	0	0	1	1	1	ENSXETG00000025015
ENSXETG00000025020	969	0	0	0	6	3	2	19	24	14	9	11	8	cdk5r2
ENSXETG00000025023	687	1	4	2	0	1	2	1	1	3	1	0	0	lrrc30
ENSXETG00000025024	2529	3834	5742	5093	4524	3680	4604	642	745	460	2000	1415	1637	cyp27a1
ENSXETG00000025025	3393	1150	1750	1498	2532	1933	2948	1590	1881	1125	3293	2368	2662	glg1
ENSXETG00000025027	1697	158	220	202	374	276	213	291	331	185	812	575	675	rnpc3
ENSXETG00000025028	435	227	352	268	217	184	389	467	584	320	477	339	422	crcp
ENSXETG00000025029	228	5	17	13	3	1	0	11	9	3	1	1	0	ENSXETG00000025029
ENSXETG00000025031	5664	4792	6848	5936	10591	8047	20377	5522	5962	3667	13765	9812	10812	lct.1
ENSXETG00000025032	3616	318	484	393	1115	866	1487	2706	3148	1769	2949	2104	2333	mmp15
ENSXETG00000025033	2024	2879	4285	3693	6664	5148	6903	5780	6372	4089	9871	7066	7695	dars
ENSXETG00000025034	564	0	0	0	0	0	0	0	0	0	0	0	0	
ENSXETG00000025035	345	0	0	0	0	0	0	7	7	4	0	0	0	ENSXETG00000025035
ENSXETG00000025036	363	0	0	0	0	0	0	42	29	25	2	0	1	ENSXETG00000025036
ENSXETG00000025037	261	2	1	3	1	5	0	2	3	2	0	0	1	tepp
ENSXETG00000025038	348	0	0	0	0	0	0	4	3	1	1	0	0	
ENSXETG00000025040	7734	3	4	9	95	74	39	101	82	47	208	155	185	celsr2
ENSXETG00000025041	3047	176	257	194	317	234	367	620	718	388	291	221	227	katnb1
ENSXETG00000025042	982	916	1256	1080	831	620	773	1210	1363	836	589	408	441	coq9
ENSXETG00000025043	1282	397	539	464	990	748	1809	2222	2473	1550	2159	1508	1760	ube2e3
ENSXETG00000025045	1641	0	1	1	0	0	0	0	0	1	4	1	1	ENSXETG00000025045
ENSXETG00000025047	2606	3422	4868	3800	2494	1905	5203	8656	11093	6034	6099	4217	4914	c4bpa
ENSXETG00000025050	2439	71	86	90	47	42	48	135	162	113	51	37	37	DNAJC10
ENSXETG00000025051	980	14914	21650	18163	26124	19746	35843	18000	20596	12564	35378	24420	27536	eif1
ENSXETG00000025052	1555	2673	4038	3455	16030	12502	9317	21288	23587	15175	37026	25731	28251	krt19
ENSXETG00000025055	2381	2097	3160	2607	4233	3332	3311	6955	7607	4605	4616	3322	3557	tp53
ENSXETG00000025056	1414	0	0	1	3	1	1	1	1	1	2	2	1	atp1b2
ENSXETG00000025060	5046	48	68	61	103	86	187	260	304	191	211	144	180	shprh
ENSXETG00000025061	2605	2171	3001	2468	3086	2338	4124	5227	6331	3581	3120	2179	2493	capza1
ENSXETG00000025063	603	141	216	180	172	126	176	101	129	81	70	45	58	ENSXETG00000025063
ENSXETG00000025064	1098	0	0	0	0	0	0	1	0	0	2	1	0	ENSXETG00000025064
ENSXETG00000025065	1097	1	1	1	1	3	3	1	1	1	8	4	8	ENSXETG00000025065
ENSXETG00000025068	570	76	129	111	65	54	188	749	852	602	211	127	145	coro1a
ENSXETG00000025071	760	191	310	247	96	77	120	207	219	129	99	85	92	c1orf131
ENSXETG00000025072	4986	0	1	1	1	1	4	3	3	0	3	4	0	ENSXETG00000025072
ENSXETG00000025073	494	2356	3412	2712	2887	2168	3171	3815	4785	2501	2450	1781	1806	gng5
ENSXETG00000025074	615	16	9	16	27	18	39	82	113	61	40	39	30	asf1a
ENSXETG00000025075	741	1	1	1	10	13	26	2	15	9	29	30	24	ENSXETG00000025075
ENSXETG00000025076	1651	26	37	30	42	39	100	541	726	392	95	70	65	dnase2b
ENSXETG00000025077	1128	840	1198	1021	535	457	611	597	749	433	326	171	216	nus1
ENSXETG00000025078	3109	148	205	158	118	114	173	622	776	419	212	160	170	samd13
ENSXETG00000025079	1452	799	1097	941	843	593	1763	702	881	494	866	632	729	c6orf120
ENSXETG00000025081	469	10696	15917	13406	8523	6554	7607	6303	7651	4571	4551	3159	3502	cox7c
ENSXETG00000025082	1714	10	18	9	24	9	6	74	92	59	7	2	0	srd5a2
ENSXETG00000025083	806	78	124	84	85	66	122	163	186	98	114	84	102	tmem242
ENSXETG00000025084	1692	850	1251	1091	1006	802	2021	1151	1338	786	796	548	643	slc35a1
ENSXETG00000025086	1014	197	273	224	262	192	379	256	284	129	325	222	273	smim8
ENSXETG00000025087	1944	498	751	643	1445	1049	1089	3210	3617	2128	5230	3752	3968	atic
ENSXETG00000025090	1677	10005	14269	12116	9495	7076	56030	16365	20068	11115	10961	7659	8458	cyp3a4
ENSXETG00000025091	1622	657	1008	843	761	563	1286	1072	1172	706	877	644	698	trnt1
ENSXETG00000025093	939	0	0	0	0	0	0	0	0	0	0	0	0	
ENSXETG00000025095	974	0	1	0	6	4	2	6	2	1	3	6	7	sh3bgr
ENSXETG00000025097	1304	11893	16871	14209	16104	12542	12444	20719	23562	14072	8458	6093	6648	hmgn1
ENSXETG00000025105	1780	764	1238	1046	1437	1132	957	2329	2689	1538	1226	891	1003	ruvbl2
ENSXETG00000025107	504	130	150	138	101	65	153	31	52	28	98	83	84	ENSXETG00000025107
ENSXETG00000025109	4062	0	0	0	44	21	6	5	8	2	5	2	2	ankar
ENSXETG00000025116	1616	92096	131596	112530	83311	63170	52136	133611	156186	91209	52913	37178	40755	actb
ENSXETG00000025118	1085	1008	1417	1207	935	731	1409	1419	1672	945	640	447	510	mob4
ENSXETG00000025120	591	103	125	100	56	51	32	1944	2035	1507	280	197	193	c1qa
ENSXETG00000025123	771	11	7	10	37	28	46	115	142	93	82	72	72	ENSXETG00000025123
ENSXETG00000025124	2791	920	1317	1198	3253	2458	3668	3095	3410	2128	6711	4700	4982	srrt
ENSXETG00000025128	918	251	330	293	220	170	228	466	506	306	136	126	113	maip1
ENSXETG00000025129	2528	1	8	2	380	303	22	2	2	1	18	16	14	saxo2
ENSXETG00000025130	1478	1	4	0	28	23	15	85	93	52	80	53	70	fgf10
ENSXETG00000025131	675	84	103	75	110	69	216	155	203	118	118	114	90	ENSXETG00000025131
ENSXETG00000025132	1764	36	67	60	77	61	159	173	230	138	257	162	201	tm6sf1
ENSXETG00000025135	1077	0	0	0	0	0	0	9	6	8	1	2	2	wnt9b
ENSXETG00000025136	567	3113	4583	4018	3515	2685	3924	417	523	320	1581	1086	1216	XB-GENE-5813137
ENSXETG00000025139	390	40	86	71	103	84	174	444	525	333	180	118	151	arl6ip5
ENSXETG00000025140	1493	630	884	718	504	414	1116	1331	1541	940	654	454	501	ube2d1
ENSXETG00000025141	2117	0	0	0	3	2	3	12	11	12	9	3	9	fmo1
ENSXETG00000025142	1693	3	0	0	1	5	4	27	25	15	3	2	1	fmo3
ENSXETG00000025145	1602	1794	2703	2224	3477	2485	4008	368	405	278	657	509	566	ENSXETG00000025145
ENSXETG00000025146	1239	147	152	154	118	85	142	110	103	74	268	169	195	tsen2
ENSXETG00000025147	716	2173	3340	2853	4641	3449	3149	5275	5935	3833	4552	3188	3506	cnbp
ENSXETG00000025150	2789	657	974	825	858	640	923	787	1038	545	659	479	513	ENSXETG00000025150
ENSXETG00000025152	695	107	169	145	170	136	298	203	256	137	189	127	126	dusp22a
ENSXETG00000025153	1720	1085	1453	1278	677	504	823	1159	1338	751	389	289	319	emc8
ENSXETG00000025155	1737	0	1	0	4	11	1	4	4	0	20	12	16	igkv3-20
ENSXETG00000025156	351	0	0	0	0	0	0	0	0	0	0	0	0	
ENSXETG00000025157	1829	0	0	0	33	31	14	13	4	3	267	186	211	ENSXETG00000025157
ENSXETG00000025160	387	44	50	55	94	71	51	206	244	160	152	74	114	smagp
ENSXETG00000025161	1925	3617	4990	4034	5364	4183	9589	5428	6324	3664	5700	4117	4542	dazap2
ENSXETG00000025164	3417	1628	2274	1930	1419	1130	1920	2716	3282	1846	1514	1035	1166	slc30a7
ENSXETG00000025166	1042	2530	3512	2772	1056	831	1951	567	657	432	925	664	738	higd1c
ENSXETG00000025168	2333	4513	6420	5316	5690	4130	6971	8167	9827	5603	8901	6097	7111	abce1
ENSXETG00000025170	1119	0	0	1	5	3	3	15	27	13	7	11	2	gpr173
ENSXETG00000025171	804	0	2	2	0	0	0	0	0	0	0	0	0	ENSXETG00000025171

ENSXETG00000025174	1996	921	1299	1086	825	617	556	1279	1440	850	625	403	474	mars
ENSXETG00000025175	606	398	609	511	913	760	1120	991	1048	670	1174	836	879	bcl2l1
ENSXETG00000025177	1527	218	268	247	273	229	488	764	885	484	513	398	463	znf740
ENSXETG00000025180	1401	2	0	0	0	0	1	0	1	2	0	0	0	
ENSXETG00000025181	1577	0	2	1	3	3	7	10	17	15	9	8	11	hoxc3
ENSXETG00000025183	678	3	12	3	20	11	31	121	153	85	28	19	16	hoxc5
ENSXETG00000025184	921	0	0	0	0	0	2	3	2	0	5	1	1	hoxc13
ENSXETG00000025190	2388	43	49	42	66	46	270	270	437	246	101	83	84	lrrc8b
ENSXETG00000025196	659	181	261	222	160	135	341	204	238	112	232	154	199	uros
ENSXETG00000025198	762	1491	2237	1938	2190	1748	2656	2143	2381	1393	2458	1745	1976	bccip
ENSXETG00000025199	1812	0	0	0	0	0	0	1	1	0	0	0	0	
ENSXETG00000025205	827	1	1	2	8	11	0	1	3	0	3	0	0	XB-GENE-5922677
ENSXETG00000025206	1283	331	580	468	272	211	350	509	544	305	204	152	172	cyb561d2
ENSXETG00000025208	1206	0	0	0	1	0	0	1	1	0	0	1	0	
ENSXETG00000025209	1478	362	491	385	457	303	543	749	915	507	811	526	566	mgmt
ENSXETG00000025211	1381	0	0	0	0	0	0	4	0	2	1	0	2	
ENSXETG00000025212	1297	150	174	156	60	50	183	5540	6591	3746	148	126	145	XB-GENE-5780408
ENSXETG00000025213	318	1	0	1	4	4	1	9	16	10	8	1	8	mgp
ENSXETG00000025214	3030	3706	5355	4347	19164	15069	70309	18889	22110	13131	23255	16509	18700	bnip3
ENSXETG00000025215	264	0	1	0	1	0	1	0	0	0	1	3	2	ENSXETG00000025215
ENSXETG00000025220	288	0	0	0	2	1	2	0	0	0	2	1	0	ENSXETG00000025220
ENSXETG00000025222	927	0	0	0	0	0	0	0	0	0	0	0	0	
ENSXETG00000025225	968	0	0	0	0	0	0	0	0	0	0	0	0	
ENSXETG00000025226	567	80	125	76	99	76	133	220	270	131	80	70	62	ENSXETG00000025226
ENSXETG00000025233	1431	0	1	1	2	0	0	4	1	3	2	0	2	ENSXETG00000025233
ENSXETG00000025234	1412	0	0	0	0	0	0	0	0	0	0	0	0	
ENSXETG00000025235	1989	0	0	0	0	0	0	0	0	0	0	0	0	
ENSXETG00000025236	2617	0	0	0	0	0	0	0	0	0	0	0	0	
ENSXETG00000025238	2448	0	0	0	0	0	0	0	0	0	0	0	0	
ENSXETG00000025239	1486	212	282	281	248	165	221	725	823	514	157	134	133	abhd1
ENSXETG00000025242	5736	0	0	0	0	0	0	2	3	1	0	1	0	
ENSXETG00000025245	3204	38	61	48	25	19	156	7	10	7	4	6	7	rbm20
ENSXETG00000025246	2582	2	3	2	0	1	3	0	0	0	0	0	0	ENSXETG00000025246
ENSXETG00000025248	2037	2941	4225	3703	5736	4368	7727	3459	4047	2457	8021	5840	6399	pdcd4
ENSXETG00000025249	903	0	0	0	0	0	0	0	2	0	1	3	1	ENSXETG00000025249
ENSXETG00000025251	10695	1	1	0	136	108	4	29	29	15	13	13	13	ENSXETG00000025251
ENSXETG00000025253	690	0	0	0	5	5	0	0	0	0	57	50	61	ENSXETG00000025253
ENSXETG00000025254	1515	0	1	1	8	5	0	1	2	0	1	0	1	CCDC60
ENSXETG00000025255	2484	33	35	47	64	62	206	920	1153	641	293	241	245	hspb8
ENSXETG00000025262	787	0	3	2	5	2	9	2	5	0	22	14	5	apoh
ENSXETG00000025264	1470	0	0	0	0	0	0	0	0	0	0	0	0	
ENSXETG00000025265	1616	546	769	758	1714	1285	3001	1788	1932	1203	2228	1614	1766	ubald2
ENSXETG00000025266	1613	1112	1582	1412	1156	911	1106	1475	1642	924	924	628	682	uba5
ENSXETG00000025267	588	33	57	36	59	27	17	29	28	28	9	2	3	ybx2
ENSXETG00000025271	1723	5728	8157	6822	9008	6954	6734	12579	13707	8587	9383	6620	7146	srsf2
ENSXETG00000025273	1246	49	70	73	99	83	158	37	65	32	66	59	51	ENSXETG00000025273
ENSXETG00000025274	852	0	0	0	0	0	0	0	0	0	0	0	0	ENSXETG00000025274
ENSXETG00000025275	1704	0	0	0	0	0	0	0	0	1	0	0	0	wfikkn2
ENSXETG00000025278	1287	46	46	43	104	96	127	158	208	103	126	90	79	ENSXETG00000025278
ENSXETG00000025280	531	0	0	0	0	0	0	0	0	0	0	0	0	
ENSXETG00000025282	1470	9	12	8	28	19	37	97	160	78	48	29	30	ENSXETG00000025282
ENSXETG00000025283	648	0	0	0	0	0	0	0	0	0	0	0	0	
ENSXETG00000025286	817	595	771	649	507	389	550	559	632	338	408	334	334	bola3
ENSXETG00000025288	588	0	2	0	0	0	0	0	0	0	0	0	0	
ENSXETG00000025292	1092	45	65	50	60	51	102	218	272	153	179	145	147	XB-GENE-979293
ENSXETG00000025293	1699	575	784	707	760	552	1098	1359	1536	942	731	474	566	zfand6
ENSXETG00000025296	3006	1	2	0	1	1	0	1	4	2	2	3	2	actn2
ENSXETG00000025297	6876	510	763	615	1478	1092	2794	2555	2944	1660	2645	1877	2054	SPG11
ENSXETG00000025300	1263	3276	4507	3716	2945	2308	1996	4838	5567	3459	4387	3077	3154	srsf7
ENSXETG00000025302	1611	409	529	414	333	247	681	486	612	346	417	237	311	eif2ak2
ENSXETG00000025303	1557	3	7	2	165	124	109	11	8	2	545	401	488	cyp1b1
ENSXETG00000025304	1365	0	1	0	1	1	0	0	1	1	1	1	0	ENSXETG00000025304
ENSXETG00000025305	1509	30	55	50	214	154	156	90	144	71	25	15	21	ENSXETG00000025305
ENSXETG00000025307	537	2	0	3	0	0	2	3	5	5	7	6	4	ENSXETG00000025307
ENSXETG00000025308	2361	9	10	10	39	26	45	280	352	205	114	97	120	coro2b
ENSXETG00000025311	1134	1308	1895	1649	486	389	396	831	1001	597	350	246	266	chchd4
ENSXETG00000025313	1074	0	2	1	3	2	5	2	5	2	3	0	3	ENSXETG00000025313
ENSXETG00000025314	705	0	0	0	0	0	0	0	0	0	0	0	0	
ENSXETG00000025315	705	0	0	0	0	0	0	0	0	0	0	0	0	
ENSXETG00000025316	482	2251	3393	2932	2393	1928	1732	2231	2561	1606	1111	777	816	ndufb7
ENSXETG00000025317	3062	5869	8315	6758	14301	10821	24631	3969	4831	2682	10895	7683	8665	dgat2
ENSXETG00000025318	1080	0	0	0	0	0	0	1	2	0	0	0	5	gpr25
ENSXETG00000025319	300	22114	32488	27984	33465	24790	30237	31661	36256	21203	72089	50711	54776	rpl38
ENSXETG00000025321	1828	0	0	0	1	0	0	2	3	3	0	0	1	pde6g
ENSXETG00000025324	1029	0	3	0	11	8	6	44	37	27	11	0	8	cyp27c1
ENSXETG00000025326	444	0	0	0	1	1	1	0	3	0	2	3	2	pla2g2e
ENSXETG00000025329	2332	520	719	637	3459	2625	3069	16545	19583	11748	2969	2027	2395	cldn4
ENSXETG00000025330	936	0	0	0	0	0	0	0	0	0	0	0	0	
ENSXETG00000025331	354	505	814	667	582	425	929	1031	1148	754	706	494	512	supt4h1
ENSXETG00000025333	939	0	0	0	0	0	0	0	0	0	0	0	0	
ENSXETG00000025334	969	0	0	0	0	0	0	0	0	0	0	0	0	
ENSXETG00000025335	3138	71	91	88	402	305	825	3126	3511	1944	1061	732	787	rnd3
ENSXETG00000025336	1167	1	3	4	14	20	9	9	19	16	9	11	13	cxorf58
ENSXETG00000025338	1362	0	0	0	1	0	0	2	4	5	0	1	0	
ENSXETG00000025339	1200	0	0	0	2	0	0	3	10	4	0	1	0	
ENSXETG00000025340	1640	0	0	0	0	0	0	11	9	5	1	0	0	
ENSXETG00000025341	1466	3	0	3	15	7	4	23	42	14	1	4	1	krt12
ENSXETG00000025345	1481	0	0	0	4	3	1	1	3	0	0	0	0	ENSXETG00000025345
ENSXETG00000025347	1031	3278	4597	3831	3111	2395	4200	5716	7123	4239	2901	2086	2354	eif1ax
ENSXETG00000025348	1177	469	573	531	750	554	929	782	946	626	1284	849	994	arl14ep
ENSXETG00000025356	324	1166	1794	1467	1390	1047	1194	1678	1851	1130	1224	858	963	spcs2
ENSXETG00000025357	806	0	0	0	0	0	0	2	2	1	0	1	2	
ENSXETG00000025359	4947	3	6	7	213	165	14	15	11	13	14	7	15	spef2
ENSXETG00000025360	519	0	0	0	0	0	1	1	0	0	0	0	0	
ENSXETG00000025361	780	0	0	0	0	0	0	0	0	0	0	0	0	
ENSXETG00000025363	1993	1409	2048	1778	2222	1647	2255	4311	5004	2818	2618	1863	2024	nfyc
ENSXETG00000025364	1260	0	0	0	0	0	0	0	1	0	0	0	0	
ENSXETG00000025365	1400	6	14	11	12	10	27	86	106	68	58	39	48	tnfsf11
ENSXETG00000025366	894	28	25	21	34	22	89	258	355	210	45	29	23	XB-GENE-5776175
ENSXETG00000025367	956	16	25	26	78	69	111	131	146	96	213	159	162	ENSXETG00000025367
ENSXETG00000025368	1389	0	0	0	0	0	0	0	0	0	0	0	0	
ENSXETG00000025371	1989	143	222	193	366	299	541	617	700	447	455	312	350	kbtbd7
ENSXETG00000025372	2402	1959	2874	2510	2453	1874	4544	7169	8148	5190	8787	6096	6671	myc
ENSXETG00000025376	4830	1499	2363	1988	4578	3531	5860	6007	6654	3969	6226	4478	4835	acin1
ENSXETG00000025377	1773	15	16	20	60	48	34	47	46	36	190	117	115	galnt11
ENSXETG00000025379	1357	0	0	0	0	0	0	6	4	1	0	0	0	
ENSXETG00000025381	909	0	0	0	0	1	0	0	0	1	1	3	1	ENSXETG00000025381
ENSXETG00000025382	6612	0	0	1	0	0	0	0	0	0	0	0	0	
ENSXETG00000025388	732	2	5	5	61	58	118	146	154	106	89	67	67	ENSXETG00000025388
ENSXETG00000025390	312	0	0	0	0	0	0	0	0	0	0	0	0	
ENSXETG00000025391	393	0	0	0	0	0	0	0	1	0	1	0	0	hist1h2ad

ENSXETG00000025393	478	2	0	1	3	0	1	9	8	7	3	1	1	ENSXETG00000025393
ENSXETG00000025394	1269	0	0	0	0	0	0	0	0	0	0	0	1	ENSXETG00000025394
ENSXETG00000025395	381	0	0	0	0	0	0	0	0	0	0	0	0	ENSXETG00000025395
ENSXETG00000025396	312	0	0	0	0	0	0	0	0	0	0	0	0	
ENSXETG00000025397	864	1301	1613	1433	2012	1637	1605	2397	2583	1753	2592	1843	1999	cystm1
ENSXETG00000025398	1314	2	13	14	39	11	27	97	93	83	148	106	109	gata3
ENSXETG00000025399	2451	1003	1510	1273	2144	1740	2657	2098	2440	1539	2406	1745	1960	PPIG
ENSXETG00000025400	1677	5	14	8	24	16	28	78	71	44	22	17	26	klhl23
ENSXETG00000025401	2106	327	482	364	903	712	951	426	482	261	553	390	376	gfod1
ENSXETG00000025402	1632	2521	3641	3008	4067	3104	4371	6087	7194	4190	5159	3670	4084	ssb
ENSXETG00000025403	702	0	1	1	0	0	0	0	1	0	1	0	0	tmem110l
ENSXETG00000025404	1845	3	5	1	5	3	11	63	74	42	11	8	6	foxi1
ENSXETG00000025405	1506	0	0	1	2	0	3	9	10	7	8	5	8	FAM196B
ENSXETG00000025406	978	0	0	0	0	0	0	0	0	0	1	0	0	
ENSXETG00000025407	1665	0	0	0	0	0	0	2	0	0	3	2	4	sp5
ENSXETG00000025408	1488	0	1	0	2	0	0	2	1	0	5	3	1	cyp2c8.2
ENSXETG00000025410	1551	0	3	4	9	0	4	9	17	12	5	0	3	ENSXETG00000025410
ENSXETG00000025411	1140	12	25	22	18	13	94	284	337	172	76	71	64	gja1
ENSXETG00000025413	2202	1819	2618	2316	1879	1498	3812	355	438	241	597	434	535	mest
ENSXETG00000025415	1715	472	631	539	748	574	1708	1668	2011	1156	802	548	680	ube2h
ENSXETG00000025416	1974	474	668	575	753	594	876	659	740	425	1358	991	1096	rdh16
ENSXETG00000025417	1249	24	40	25	104	83	66	25	27	22	107	79	85	XB-GENE-5807237
ENSXETG00000025418	1877	0	0	0	0	0	1	6	6	2	3	3	3	
ENSXETG00000025420	2286	1	1	0	1	0	4	4	4	5	2	0	0	chrna1
ENSXETG00000025421	1184	202	279	258	471	378	309	593	685	368	267	203	212	prim1
ENSXETG00000025422	1013	1380	2011	1503	1280	980	2895	2417	2920	1726	1226	746	867	atp6v1f
ENSXETG00000025424	1705	21	54	40	104	81	151	240	327	149	203	170	175	ENSXETG00000025424
ENSXETG00000025425	1065	19	25	18	53	48	88	98	87	53	98	74	92	ENSXETG00000025425
ENSXETG00000025427	1343	1213	1779	1431	1713	1293	1964	2683	3126	1817	1439	1062	1188	cops8
ENSXETG00000025433	1419	0	0	0	0	0	0	0	2	1	0	0	0	
ENSXETG00000025434	1317	0	0	0	0	0	0	0	0	0	0	0	0	
ENSXETG00000025435	629	3031	4556	3831	1755	1272	2430	640	759	422	1855	1439	1452	cox5b.2
ENSXETG00000025436	945	0	0	0	0	0	0	0	0	0	0	0	0	
ENSXETG00000025437	951	0	0	0	0	0	0	0	0	0	0	0	0	
ENSXETG00000025439	945	0	0	0	0	0	0	0	0	0	0	0	0	
ENSXETG00000025440	954	0	0	0	0	0	0	0	0	0	0	0	0	
ENSXETG00000025441	933	9	8	3	16	20	39	227	301	164	48	35	46	ENSXETG00000025441
ENSXETG00000025443	1441	112	158	140	196	149	192	279	313	190	134	78	97	nsmce1
ENSXETG00000025446	2148	0	0	0	0	0	0	0	0	0	0	0	0	
ENSXETG00000025449	1047	0	0	0	0	0	0	1	1	0	0	0	0	
ENSXETG00000025450	942	0	0	0	0	0	0	0	0	0	0	0	0	
ENSXETG00000025453	768	1	0	0	0	0	1	14	16	8	5	5	10	ENSXETG00000025453
ENSXETG00000025457	797	58813	88314	76324	82548	62036	95531	79745	91762	55787	211332	148803	163963	rpl7
ENSXETG00000025460	841	1288	1862	1545	1211	972	2149	1608	1922	1067	967	722	770	vma21
ENSXETG00000025461	1200	194	279	212	263	226	230	508	520	354	131	84	102	anapc13.1
ENSXETG00000025462	918	52	95	58	62	46	96	406	442	272	324	251	232	arhgef3.1
ENSXETG00000025463	3676	9870	14207	12417	13056	9941	23683	7048	8336	4930	14267	9900	11484	mgc108077
ENSXETG00000025464	1790	347	479	424	566	421	862	410	440	289	739	482	510	ccdc85c
ENSXETG00000025466	1317	88987	130574	111806	100885	77009	155039	56335	64568	37851	111013	76753	83910	slc25a5
ENSXETG00000025468	1098	30	48	51	46	27	57	101	114	87	51	31	33	XB-GENE-5936142
ENSXETG00000025471	2033	829	1284	1013	1082	895	1180	1702	1942	1164	2933	1948	2209	arglu1
ENSXETG00000025472	3669	434	601	512	858	681	882	1724	1833	1158	1757	1281	1443	phf20
ENSXETG00000025473	261	749	1255	1010	608	499	690	801	925	568	413	330	318	selenok
ENSXETG00000025474	358	1289	1825	1593	1293	1052	1102	1451	1668	1071	1128	740	754	romo1
ENSXETG00000025475	2526	482	633	531	668	497	666	1215	1291	781	1027	715	869	ranbp3
ENSXETG00000025476	2455	102	121	103	173	98	303	482	682	374	322	188	236	slc25a19
ENSXETG00000025477	3403	432	604	456	653	447	731	883	1083	630	525	405	447	arhgdia
ENSXETG00000025478	2084	1	3	0	4	0	7	8507	11271	6083	214	152	180	lum
ENSXETG00000025479	1056	0	0	1	1	0	2	35	26	18	4	2	0	kera
ENSXETG00000025480	1637	1	1	1	0	0	0	927	1026	613	5	6	8	epyc
ENSXETG00000025484	2146	3493	4877	3984	3832	2900	4707	4657	5575	3118	2876	2027	2298	rab11b.1
ENSXETG00000025485	1180	132	158	168	155	107	252	359	441	260	221	160	177	arl16
ENSXETG00000025486	2000	3006	4289	3615	3569	2857	8596	4244	5179	3019	3488	2384	2711	scamp2
ENSXETG00000025487	519	0	0	1	0	0	0	0	1	0	0	0	0	rpp25
ENSXETG00000025489	648	57	104	99	68	31	157	879	1018	655	244	178	234	fgf19
ENSXETG00000025492	1909	6	9	6	30	40	92	389	423	290	196	152	130	f10
ENSXETG00000025493	1345	0	0	1	0	1	0	0	2	0	0	0	0	proz
ENSXETG00000025497	2726	0	0	0	0	0	0	9	9	9	9	7	4	
ENSXETG00000025498	735	0	0	0	0	0	0	1	0	0	0	0	0	XB-GENE-5733005
ENSXETG00000025500	1506	0	0	0	0	0	0	1	0	0	0	0	0	
ENSXETG00000025501	1983	40	67	54	281	203	96	591	618	396	247	163	205	bcat2
ENSXETG00000025503	1683	1082	1635	1446	2087	1658	1853	3300	3693	2396	3975	2905	3114	srsf6
ENSXETG00000025504	713	197	314	254	283	236	352	1198	1265	775	266	201	242	tmem150b
ENSXETG00000025505	351	5463	8217	6407	7288	5816	17170	14404	16102	10247	18600	12574	15001	myl9
ENSXETG00000025507	1140	5920	8748	7376	9712	7319	2843	4428	5011	2997	14724	10305	11476	ethe1
ENSXETG00000025510	473	32403	48708	41680	40182	29942	42908	44695	50471	30708	94749	67221	72323	rps19
ENSXETG00000025511	1112	668	975	859	1152	892	1851	1615	1777	1045	1506	1130	1168	blcap
ENSXETG00000025512	682	715	1002	835	738	590	1094	5788	7005	3837	1743	1246	1536	ifi30
ENSXETG00000025513	1257	655	877	745	408	305	489	500	629	322	393	267	324	gnpnat1
ENSXETG00000025514	600	141	227	170	157	127	166	229	299	148	142	95	117	styx
ENSXETG00000025515	1539	667	876	775	660	539	1434	1749	1939	1080	790	562	683	vamp3
ENSXETG00000025516	3338	190	274	230	286	195	402	321	367	192	299	215	248	alg10
ENSXETG00000025518	924	0	2	2	2	1	0	3	7	2	4	2	3	ENSXETG00000025518
ENSXETG00000025519	792	113	164	141	138	91	195	216	277	135	115	87	98	vcpkmt
ENSXETG00000025521	1064	0	1	0	0	0	0	2	0	0	0	0	1	
ENSXETG00000025522	462	0	1	1	2	2	4	8	17	10	1	0	0	esr10
ENSXETG00000025523	990	12	19	16	11	4	11	255	254	153	200	133	174	ENSXETG00000025523
ENSXETG00000025524	1005	0	1	0	7	3	8	78	83	41	26	35	26	ENSXETG00000025524
ENSXETG00000025525	2532	0	0	1	0	0	1	0	0	1	0	0	0	pnhd
ENSXETG00000025527	920	197	244	208	88	62	433	420	531	307	140	109	143	ENSXETG00000025527
ENSXETG00000025533	2017	2062	2915	2463	3304	2480	4522	6228	7340	3966	2993	2113	2366	lypla2
ENSXETG00000025540	519	0	1	1	0	0	1	0	0	0	0	0	0	
ENSXETG00000025541	3513	3	3	3	3	0	3	9	17	9	2	0	1	fam19a5
ENSXETG00000025543	567	0	3	4	2	1	0	0	0	0	0	1	1	ENSXETG00000025543
ENSXETG00000025544	1510	9595	13438	11100	9884	7692	14673	402	500	294	6564	4573	5346	cpo
ENSXETG00000025545	1919	364	559	381	368	326	287	342	370	224	114	70	104	alg12
ENSXETG00000025547	528	1	0	0	0	0	0	0	0	0	0	0	0	ENSXETG00000025547
ENSXETG00000025548	4001	60	80	72	126	101	429	333	415	243	294	160	236	pikfyve
ENSXETG00000025551	2121	4109	5777	4870	4854	3665	7804	5944	7116	4189	4913	3459	3895	rars
ENSXETG00000025552	2368	0	0	1	0	0	0	4	5	2	2	0	2	
ENSXETG00000025558	1242	0	1	0	0	0	0	0	0	0	0	0	0	
ENSXETG00000025559	1050	0	0	0	0	0	0	0	0	0	0	0	1	
ENSXETG00000025560	486	197	300	259	202	166	570	252	276	166	207	155	170	XB-GENE-5931223
ENSXETG00000025564	1272	0	0	0	0	0	0	2	4	1	1	0	0	ENSXETG00000025564
ENSXETG00000025567	1267	1419	1844	1511	825	604	1384	646	825	391	656	473	526	nudt19
ENSXETG00000025568	1018	4	0	2	2	0	4	0	1	1	0	2	1	ENSXETG00000025568
ENSXETG00000025571	1262	738	1036	941	954	719	1149	1355	1585	867	855	548	671	babam1
ENSXETG00000025574	2273	457	710	610	1460	1152	1597	2011	2435	1337	1275	977	986	slc31a2
ENSXETG00000025575	548	938	1429	1244	725	512	958	826	998	598	894	620	685	cuta
ENSXETG00000025578	1631	865	1239	1042	1400	1122	1930	387	478	260	1519	1099	1163	tmem82
ENSXETG00000025579	1089	1	0	0	1	0	0	0	0	1	0	0	0	ENSXETG00000025579

ENSXETG00000025580	1576	1289	1795	1543	1214	1005	1440	1279	1580	935	823	588	652	smim7
ENSXETG00000025586	1465	786	1132	890	726	571	789	8391	9971	5700	4719	3260	3728	hla-drb1
ENSXETG00000025588	723	2	0	1	4	5	8	22	27	12	14	5	8	ENSXETG00000025588
ENSXETG00000025590	396	35	52	47	26	11	21	403	429	230	147	92	114	hla-dqa2
ENSXETG00000025592	1161	0	0	0	0	0	0	6	5	4	0	0	0	
ENSXETG00000025593	1197	0	0	0	1	0	1	1	0	0	2	1	0	pou3f3
ENSXETG00000025594	2336	38	42	50	62	37	82	115	158	90	72	59	63	XB-GENE-5799682
ENSXETG00000025595	1146	0	0	0	3	1	2	2	0	0	5	4	2	ENSXETG00000025595
ENSXETG00000025597	822	0	0	0	0	0	0	0	0	0	0	1	0	ENSXETG00000025597
ENSXETG00000025598	1820	0	0	0	0	0	0	0	0	0	0	0	0	
ENSXETG00000025599	404	13495	20053	16133	11003	8380	11421	8484	9550	5093	7258	5083	5775	cox7b
ENSXETG00000025601	1752	27430	39276	33690	21208	15730	56506	1199	1445	822	17610	12263	13847	slc5a12
ENSXETG00000025603	996	1113	1562	1342	1100	862	2498	2168	2657	1470	1573	1093	1219	leprot
ENSXETG00000025604	1017	0	0	1	1	3	1	4	5	5	8	1	5	ENSXETG00000025604
ENSXETG00000025606	3507	636	864	730	910	663	965	1078	1313	714	1146	744	841	c1galt1c1
ENSXETG00000025608	2230	0	0	0	2	0	0	0	0	0	0	1	1	shisa1.1
ENSXETG00000025609	771	0	0	0	0	0	0	0	0	0	0	0	0	
ENSXETG00000025610	2395	40	60	50	77	75	120	181	188	112	149	135	136	asb12.2
ENSXETG00000025611	1292	98	187	154	222	149	225	284	303	176	204	148	164	asb12
ENSXETG00000025614	1126	468	635	581	147	112	170	267	311	211	120	68	76	endog
ENSXETG00000025615	1134	0	0	0	0	0	0	0	0	0	0	0	0	
ENSXETG00000025621	1476	1447	2071	1700	1632	1192	1990	1792	2142	1191	2049	1342	1514	lrrc19
ENSXETG00000025623	1990	179	235	218	520	364	591	576	675	423	107	89	75	cdc20
ENSXETG00000025625	721	2444	3466	2978	5549	4266	1833	3278	3946	2409	2670	1884	2122	agr2
ENSXETG00000025626	1078	288	389	342	136	93	243	262	364	177	256	188	233	tmem69
ENSXETG00000025628	1905	187	235	205	240	161	424	431	530	272	298	209	260	ENSXETG00000025628
ENSXETG00000025629	987	5472	7854	6676	3261	2524	7712	802	1033	588	1868	1344	1529	cmbl
ENSXETG00000025630	1320	8	13	7	42	34	52	656	760	440	190	128	135	twist1
ENSXETG00000025632	924	120	166	142	152	110	131	6	13	9	56	36	47	ENSXETG00000025632
ENSXETG00000025633	2057	774	1159	1020	996	704	1332	1012	1116	678	762	534	594	dap
ENSXETG00000025634	669	0	0	0	0	0	0	0	0	0	0	0	0	
ENSXETG00000025635	870	0	0	0	0	0	0	0	0	0	0	0	0	
ENSXETG00000025636	702	0	0	0	0	0	0	0	0	0	0	0	0	
ENSXETG00000025641	914	737	1120	932	649	489	645	611	742	382	508	334	401	pigy
ENSXETG00000025642	553	117	147	117	135	88	212	131	173	113	171	128	139	c4orf32
ENSXETG00000025643	672	1276	2064	1802	1374	999	2016	2157	2669	1474	1190	855	948	XB-GENE-5918131
ENSXETG00000025645	753	419	659	586	546	404	893	912	1147	723	609	398	508	ap1ar
ENSXETG00000025648	1441	0	0	0	0	0	0	0	0	0	0	0	0	
ENSXETG00000025649	516	895	1500	1175	1949	1407	2928	866	1034	631	1859	1284	1385	mal2
ENSXETG00000025650	186	0	2	1	1	1	4	25	18	13	4	0	3	ENSXETG00000025650
ENSXETG00000025651	1691	592	923	798	1308	1000	2703	1597	1884	1093	1406	1013	1164	eif4ebp2
ENSXETG00000025653	1048	119	194	179	101	58	64	280	307	203	59	38	37	lrrc20
ENSXETG00000025656	717	0	0	0	1	3	0	0	0	0	3	5	5	ENSXETG00000025656
ENSXETG00000025658	1044	989	1550	1306	1077	862	1146	1851	2152	1368	1146	866	902	mrpl13
ENSXETG00000025659	3104	62	106	56	261	222	431	339	427	263	1275	902	1050	DNAJC12
ENSXETG00000025660	2286	1068	1437	1147	3580	2810	3750	3325	4120	2336	3655	2637	2990	slc26a3.2
ENSXETG00000025663	712	9620	14622	12336	3641	2750	22707	17039	19756	11262	5696	3922	4231	hba3
ENSXETG00000025664	584	74	106	68	8	5	27	7614	8687	5164	5941	3931	4597	hba1
ENSXETG00000025667	581	47	73	61	5	4	7	6360	7459	3990	6253	4273	4796	hbg1
ENSXETG00000025668	3614	176	270	222	1580	1226	1435	1573	1755	1140	3389	2389	2540	ST5
ENSXETG00000025669	2488	388	554	430	357	275	403	546	679	381	321	208	287	bcap29
ENSXETG00000025674	1805	492	612	532	428	388	777	419	554	308	769	519	606	alg1
ENSXETG00000025675	5347	932	1301	1116	2457	1821	3672	4319	4965	2859	4429	3040	3463	cdc42bpb
ENSXETG00000025676	1904	1306	1885	1562	1891	1468	2471	2439	2721	1599	2450	1640	1858	paip2b
ENSXETG00000025677	1582	3105	4224	3242	2682	1869	5243	3685	4811	2573	2128	1535	1730	sypl1
ENSXETG00000025679	5968	429	620	503	1302	987	1587	2019	2335	1361	2444	1794	1922	kmt2e
ENSXETG00000025680	1055	1	0	1	0	1	3	18	27	16	12	14	10	lhfpl3
ENSXETG00000025681	10398	2	3	2	61	42	60	150	157	105	245	177	175	reln
ENSXETG00000025686	312	0	0	0	0	0	0	0	0	0	0	0	0	hist1h4c
ENSXETG00000025687	393	1	1	2	6	5	2	12	9	4	7	6	5	ENSXETG00000025687
ENSXETG00000025688	706	0	1	0	1	3	0	24	25	15	6	4	2	TMEM269
ENSXETG00000025689	279	106	136	114	110	72	98	151	169	122	94	89	81	rpp14
ENSXETG00000025690	1437	0	3	0	11	5	19	56	47	36	27	21	28	ENSXETG00000025690
ENSXETG00000025693	804	45	59	59	41	41	96	170	249	125	69	45	63	tmem60
ENSXETG00000025696	2896	325	452	393	859	640	1177	1697	2155	1146	1101	802	870	gnai1
ENSXETG00000025697	492	16	27	14	38	41	81	150	195	110	245	154	178	sspn
ENSXETG00000025698	1728	2497	3652	3089	1754	1389	1560	1803	2121	1328	1588	1025	1254	fam3d
ENSXETG00000025702	2346	0	0	0	0	0	0	0	0	0	0	0	0	
ENSXETG00000025704	2493	0	0	0	0	0	0	0	0	0	0	0	0	
ENSXETG00000025707	1853	984	1472	1204	736	575	3725	319	342	214	683	486	510	asns
ENSXETG00000025708	510	0	0	0	0	0	0	0	0	0	0	0	0	
ENSXETG00000025709	1010	1	1	0	6	3	8	231	307	151	120	109	122	tac1
ENSXETG00000025713	839	320	496	443	272	225	442	490	599	319	389	287	308	znhit3
ENSXETG00000025714	1482	20	31	11	37	32	56	79	116	61	112	66	82	ENSXETG00000025714
ENSXETG00000025716	598	2093	3049	2553	1986	1512	2014	1196	1379	776	1272	905	984	mpc2
ENSXETG00000025722	621	1042	1431	1198	682	534	1341	1113	1302	745	860	549	612	lamtor5
ENSXETG00000025727	639	1239	1868	1656	1599	1179	1712	3719	4364	2632	819	592	631	psma5
ENSXETG00000025729	1378	0	1	0	0	0	0	0	0	0	1	1	1	
ENSXETG00000025730	1504	2	1	3	3	3	1	13	22	11	33	20	21	pgc
ENSXETG00000025732	1787	0	0	0	0	0	0	0	0	0	0	0	0	
ENSXETG00000025735	978	0	0	1	1	0	0	0	0	0	2	0	0	P2RY6
ENSXETG00000025736	2918	313	506	357	510	373	568	1448	1702	1038	1219	827	916	id4
ENSXETG00000025737	1175	2	3	1	118	67	9	8	6	9	15	1	6	c4orf47
ENSXETG00000025738	764	491	614	604	38	23	145	1025	1166	708	392	271	278	ankrd37
ENSXETG00000025739	885	112	158	107	444	310	472	317	365	211	1457	1002	1204	ENSXETG00000025739
ENSXETG00000025740	897	2	3	3	3	1	4	2	7	0	15	12	5	slc25a4
ENSXETG00000025742	1296	4	2	4	13	8	6	1	3	0	18	13	17	ENSXETG00000025742
ENSXETG00000025743	1017	9	9	6	20	15	21	10	11	8	49	29	34	ENSXETG00000025743
ENSXETG00000025749	2456	0	0	0	0	0	0	4	2	0	0	0	0	
ENSXETG00000025752	496	118	123	117	103	76	160	191	254	153	108	68	77	rnf4
ENSXETG00000025753	3845	138	246	203	738	484	681	337	319	220	551	374	444	wwc2
ENSXETG00000025754	1065	351	470	391	617	502	846	428	422	310	438	307	351	ENSXETG00000025754
ENSXETG00000025755	1350	507	780	648	2866	2205	4385	224	279	165	2264	1566	1773	ENSXETG00000025755
ENSXETG00000025761	534	39	82	62	343	242	433	219	287	172	867	605	658	ENSXETG00000025761
ENSXETG00000025765	3066	1097	1594	1304	3285	2495	2527	1724	2040	1199	2039	1420	1603	ap2a2
ENSXETG00000025768	450	647	949	801	496	368	452	998	1142	726	399	256	292	polr2l.1
ENSXETG00000025770	2656	0	0	0	0	0	0	0	0	0	0	0	0	
ENSXETG00000025771	1688	702	1061	834	771	537	949	751	931	555	712	511	566	mbtps2
ENSXETG00000025773	1353	11	16	19	57	44	19	13	14	14	25	19	12	cfap74
ENSXETG00000025775	510	0	0	0	0	0	0	0	0	0	0	0	0	
ENSXETG00000025777	1403	3413	5118	4309	2818	2057	2825	1677	1934	1122	1554	1098	1126	sec22b
ENSXETG00000025778	1233	318	518	410	162	133	185	258	339	219	136	87	114	oxnad1
ENSXETG00000025781	1743	85	117	109	195	144	252	247	262	159	286	228	197	fam46c
ENSXETG00000025783	1307	7	10	4	14	16	16	249	283	170	16	17	10	ido1
ENSXETG00000025787	8448	0	0	0	2	1	3	4	1	3	10	8	5	CSMD3
ENSXETG00000025788	2986	5952	8768	7652	15017	11579	14228	16465	17928	11403	12547	9058	9657	vcp
ENSXETG00000025791	1214	1643	2423	2009	2439	1822	2348	3590	3984	2426	1592	1109	1248	stoml2
ENSXETG00000025794	1051	6	8	6	2	2	6	19	32	13	13	12	13	ENSXETG00000025794
ENSXETG00000025798	513	0	0	0	0	0	0	0	0	0	0	0	0	
ENSXETG00000025799	924	0	0	0	0	0	0	0	0	0	0	0	0	
ENSXETG00000025801	594	0	0	0	0	1	0	0	0	0	2	0	1	cradd.2

ENSXETG00000025802	456	119	212	190	205	152	260	217	258	136	241	191	182	c2orf68
ENSXETG00000025807	949	452	597	479	318	227	453	432	490	286	385	264	285	sys1
ENSXETG00000025808	1748	269	457	358	427	337	590	385	508	330	898	657	718	lgalsl
ENSXETG00000025809	483	0	0	0	2	0	1	4	6	7	1	1	1	adora3
ENSXETG00000025810	585	0	0	0	0	0	0	0	0	0	0	0	0	
ENSXETG00000025811	1290	7	4	8	12	9	66	138	168	109	120	93	86	adra2b
ENSXETG00000025813	609	2	2	1	5	5	13	2	0	0	4	5	2	pebp4
ENSXETG00000025814	1107	6	9	8	17	9	18	32	33	23	10	13	6	ENSXETG00000025814
ENSXETG00000025815	1332	0	0	1	0	0	0	0	0	0	0	0	0	
ENSXETG00000025816	1547	26	57	32	60	41	84	527	650	360	106	81	85	gpr34
ENSXETG00000025818	1593	95	144	106	328	230	50	215	292	168	96	68	65	atad5
ENSXETG00000025823	753	0	0	0	0	0	0	0	0	0	0	0	0	
ENSXETG00000025826	432	1902	2655	2216	1909	1394	2093	2469	2636	1607	1657	1176	1317	wdr83os
ENSXETG00000025827	1782	0	0	0	0	0	7	18	18	10	8	5	5	fshr
ENSXETG00000025830	1711	133	189	158	207	165	287	297	310	204	470	279	339	ccdc130
ENSXETG00000025833	915	0	0	0	0	0	0	0	0	0	0	0	0	
ENSXETG00000025834	933	0	0	0	0	0	0	0	0	0	0	0	0	
ENSXETG00000025835	909	0	0	0	0	0	0	0	0	0	0	0	0	
ENSXETG00000025842	1180	1071	1503	1326	1696	1392	2051	1188	1366	776	1365	1030	1130	grhpr.1
ENSXETG00000025843	1567	223	313	279	175	148	219	212	251	134	133	114	116	rwdd3
ENSXETG00000025844	2274	15	15	13	37	42	31	79	81	40	82	54	78	ENSXETG00000025844
ENSXETG00000025846	1440	72	108	111	109	75	212	117	134	80	109	112	95	ENSXETG00000025846
ENSXETG00000025847	962	207	278	213	302	225	356	560	620	368	331	254	223	cdk5
ENSXETG00000025848	1073	105	193	159	336	263	734	1872	2258	1258	934	629	669	sh3bgrl2
ENSXETG00000025849	1275	103	139	131	130	100	248	342	394	245	346	234	241	ripk3
ENSXETG00000025850	788	1131	1599	1465	1565	1174	1396	4052	4588	2837	1806	1274	1358	hmgn3
ENSXETG00000025853	427	12329	18765	15750	9671	7188	8058	7794	8905	5234	8885	6082	6862	cox7a2
ENSXETG00000025857	795	0	1	0	0	0	9	30	41	20	23	17	28	chst9
ENSXETG00000025861	7905	119	177	164	540	419	993	1265	1492	856	1264	946	984	ralgapa1
ENSXETG00000025864	982	2046	2977	2694	2443	1829	2678	4848	5449	3178	2237	1529	1702	hsbp1
ENSXETG00000025867	1539	393	572	501	1992	1556	1697	4180	4729	2863	3521	2615	2750	foxa1
ENSXETG00000025877	1437	0	0	0	0	0	0	0	0	0	0	0	0	
ENSXETG00000025878	1981	415	576	512	357	259	304	541	633	429	432	279	317	mphosph6
ENSXETG00000025884	873	3326	4611	3987	3757	2806	4416	7119	8241	4957	2926	2048	2236	psma6
ENSXETG00000025885	2409	0	0	0	0	0	0	0	0	0	0	0	0	
ENSXETG00000025887	813	204	294	259	218	141	289	138	154	77	117	80	89	ENSXETG00000025887
ENSXETG00000025889	2856	0	0	0	0	0	0	4	1	0	0	2	1	serpina3m
ENSXETG00000025891	1337	1	1	1	0	0	0	14	19	10	0	0	0	serpina3
ENSXETG00000025892	1352	2	2	1	0	0	0	25	38	14	0	0	0	serpina3k
ENSXETG00000025898	975	0	0	0	1	0	1	2	1	1	1	0	0	ENSXETG00000025898
ENSXETG00000025899	656	225	277	235	114	89	68	324	399	224	218	145	170	dynll2
ENSXETG00000025900	1457	463	595	552	386	297	478	426	553	273	298	230	228	vti1b
ENSXETG00000025901	778	1822	2691	2294	1588	1229	1811	1529	1782	1077	930	708	749	glrx5
ENSXETG00000025903	2063	282	425	367	316	275	691	617	761	470	1072	709	862	ddx52
ENSXETG00000025905	832	1054	1548	1290	736	557	1632	611	792	464	668	452	504	sft2d2
ENSXETG00000025907	1104	45	65	75	112	89	148	153	183	80	149	96	111	klhl12
ENSXETG00000025908	1635	377	481	402	248	201	442	783	873	459	195	139	153	rabif
ENSXETG00000025909	909	137	171	144	296	226	898	558	610	454	777	629	572	c1orf35
ENSXETG00000025910	981	0	0	0	0	0	0	0	0	0	0	0	0	
ENSXETG00000025913	1053	6	9	8	16	9	10	0	4	0	4	4	5	lrrc69
ENSXETG00000025918	7020	199	316	289	411	363	1040	621	755	397	1640	1293	1453	ENSXETG00000025918
ENSXETG00000025920	3097	186	280	201	257	202	378	436	530	257	320	219	265	ENSXETG00000025920
ENSXETG00000025921	2249	46	66	55	252	192	210	224	260	187	232	162	194	pdp1
ENSXETG00000025922	1350	0	0	0	0	0	0	0	0	1	1	0	0	ENSXETG00000025922
ENSXETG00000025923	1393	0	0	0	0	0	0	0	0	0	0	0	0	
ENSXETG00000025924	2938	14	33	30	93	68	61	139	159	96	27	20	24	rad54b
ENSXETG00000025925	1557	3766	5586	5070	5404	4075	6081	4480	4945	3018	5267	3800	4086	ndufs2
ENSXETG00000025926	1476	0	0	0	0	0	0	3	4	1	0	0	0	
ENSXETG00000025927	4524	520	678	590	751	581	920	1807	2219	1259	567	384	494	tdg
ENSXETG00000025929	462	347	466	433	324	220	231	557	593	335	314	194	223	polr2k
ENSXETG00000025930	414	0	0	0	0	0	0	0	0	0	0	0	0	
ENSXETG00000025931	312	0	0	0	0	0	0	0	0	0	0	0	0	
ENSXETG00000025932	2908	278	387	373	403	328	511	574	674	358	402	277	344	etaa1
ENSXETG00000025934	532	287	391	341	172	89	228	564	684	351	209	119	183	c1d
ENSXETG00000025936	309	11	17	3	6	10	15	75	85	53	48	42	33	cnrip1
ENSXETG00000025937	416	9107	13652	11769	9573	7295	8311	6434	7350	4414	4424	3224	3433	cox6c
ENSXETG00000025938	915	67	105	97	160	136	534	282	322	171	290	225	245	aplf
ENSXETG00000025940	695	369	522	442	740	530	1020	266	298	188	238	141	159	acot13
ENSXETG00000025941	939	363	513	445	434	373	764	769	826	530	451	316	332	bloc1s4
ENSXETG00000025944	1059	0	0	0	0	0	0	0	0	0	0	0	0	
ENSXETG00000025945	2370	0	0	0	0	0	0	0	0	0	0	0	0	
ENSXETG00000025946	1334	172	193	162	156	121	205	291	325	198	97	73	95	pqlc1.1
ENSXETG00000025952	1749	11	15	8	14	14	27	46	49	19	26	26	22	ENSXETG00000025952
ENSXETG00000025954	1188	12	11	10	26	32	62	36	69	30	76	48	50	ENSXETG00000025954
ENSXETG00000025955	2450	50	74	67	186	148	285	320	390	217	417	221	253	znf527
ENSXETG00000025956	2340	48	76	51	122	91	173	209	243	146	250	161	203	ENSXETG00000025956
ENSXETG00000025961	312	0	0	1	0	0	0	0	0	0	0	0	0	
ENSXETG00000025963	2565	1	2	1	1	1	0	0	0	0	0	0	0	ENSXETG00000025963
ENSXETG00000025968	1533	63	64	78	291	205	476	572	593	332	363	212	245	XB-GENE-5945738
ENSXETG00000025969	1247	11	14	11	20	13	33	99	114	65	22	22	18	XB-GENE-5846549
ENSXETG00000025970	1590	22	23	21	25	26	49	97	86	69	29	23	26	ENSXETG00000025970
ENSXETG00000025971	873	0	0	0	1	2	0	2	0	0	2	1	0	
ENSXETG00000025974	2785	129	198	159	440	387	349	1006	1275	748	1059	714	763	b3gnt5
ENSXETG00000025978	1176	158	252	180	238	156	216	400	387	254	299	165	236	dcaf12
ENSXETG00000025981	2147	16	30	18	59	53	86	172	194	113	117	77	83	KIAA1328
ENSXETG00000025990	732	1275	1849	1441	926	731	1295	504	763	405	608	410	515	mpc1
ENSXETG00000025991	915	258	382	329	174	92	243	190	195	123	135	77	97	zdhhc7
ENSXETG00000025992	2646	82	122	105	256	195	242	1273	1375	850	242	170	204	cdh3
ENSXETG00000025993	1305	210	342	312	465	354	569	501	510	361	502	319	351	XB-GENE-5919443
ENSXETG00000025994	2106	0	0	0	1	0	4	9	8	2	14	6	6	rps6ka2
ENSXETG00000025995	10786	405	543	492	544	405	681	558	678	407	656	476	540	lcor
ENSXETG00000025998	1409	302	392	374	456	375	892	604	721	409	719	523	548	polr1d.1
ENSXETG00000025999	1823	173	252	222	393	277	307	741	783	455	615	433	491	agmat
ENSXETG00000026000	1250	619	871	666	2357	1808	2676	2273	2643	1612	3577	2509	2694	ubl3
ENSXETG00000026001	2280	187	258	209	219	158	426	1524	1675	1071	391	258	270	alox5ap
ENSXETG00000026002	878	1	0	3	0	0	2	2	1	0	0	0	0	ENSXETG00000026002
ENSXETG00000026003	744	132	193	172	95	81	222	274	385	233	149	97	103	sertad1
ENSXETG00000026004	8733	56	77	77	296	220	306	602	694	379	554	354	423	chd8
ENSXETG00000026005	2748	837	1170	1023	1055	756	1909	1142	1402	782	863	605	682	nsmaf
ENSXETG00000026006	2640	0	0	0	0	0	0	0	2	0	0	0	0	
ENSXETG00000026007	1308	598	880	809	2290	1735	2513	2746	2961	1758	2265	1584	1784	slc25a24
ENSXETG00000026010	801	1420	2011	1747	3147	2474	4925	56	76	45	3512	2557	2643	ca3
ENSXETG00000026012	1668	2	1	1	0	0	1	2	0	0	1	2	4	
ENSXETG00000026013	1611	11	10	7	22	26	60	33	28	14	45	29	31	ENSXETG00000026013
ENSXETG00000026014	2306	132	195	160	138	108	159	221	259	139	75	57	65	emc6
ENSXETG00000026016	4698	8	14	12	130	108	145	487	519	353	395	291	284	XB-GENE-1001170
ENSXETG00000026017	702	0	0	0	0	0	0	0	0	0	0	0	0	
ENSXETG00000026019	960	2	4	1	2	1	2	2	4	2	4	2	4	ENSXETG00000026019
ENSXETG00000026021	201	10	14	12	15	12	26	105	112	58	16	12	16	ENSXETG00000026021
ENSXETG00000026022	1930	213	284	256	674	549	968	1060	1164	624	1020	690	826	rnf2
ENSXETG00000026023	156	44	48	58	159	136	83	348	404	244	107	65	84	ENSXETG00000026023

ENSXETG00000026024	813	3029	4370	3714	2214	1689	2008	5149	6032	3571	2543	1728	2016	snu13
ENSXETG00000026025	2591	292	420	350	838	606	1343	1129	1306	726	405	307	314	desi1
ENSXETG00000026026	1552	300	383	314	328	257	446	490	581	279	229	161	186	ccnc
ENSXETG00000026029	531	120	134	124	103	94	104	1224	1353	750	252	159	196	psmb9
ENSXETG00000026030	1667	2	3	1	3	2	17	636	687	416	239	130	155	ENSXETG00000026030
ENSXETG00000026031	564	0	0	0	0	0	0	9	8	5	2	1	2	
ENSXETG00000026033	973	48	85	87	135	118	223	675	723	441	227	168	180	phlda2
ENSXETG00000026035	1101	34	38	42	44	34	77	90	108	68	50	40	31	tmx4
ENSXETG00000026037	2545	1372	1997	1648	1362	1004	1732	1600	2000	1207	1401	961	1180	crls1
ENSXETG00000026042	4345	76	120	104	314	257	470	602	690	438	442	303	356	RGS12
ENSXETG00000026045	1004	694	1035	856	1118	827	1537	1133	1266	770	1141	865	870	XB-GENE-5820599
ENSXETG00000026046	997	1	0	0	0	2	0	12	2	3	3	1	1	ENSXETG00000026046
ENSXETG00000026047	3115	487	663	561	1474	1158	2238	4819	5545	3344	2109	1521	1728	smad1
ENSXETG00000026049	1579	1622	2434	2012	1905	1425	3257	2618	3025	1794	2326	1695	1730	lamtor2
ENSXETG00000026053	999	0	0	0	0	0	0	0	0	0	0	0	0	
ENSXETG00000026058	1417	651	967	761	6275	4864	1042	5872	7177	4224	2553	1863	2063	cnfn.1
ENSXETG00000026059	1437	72	74	61	211	133	170	280	372	200	36	24	23	ccnb2
ENSXETG00000026061	2943	256	362	333	630	482	930	1442	1709	975	771	550	619	nr3c2
ENSXETG00000026062	9759	732	1069	885	2010	1469	2082	2211	2609	1412	1608	1094	1312	LRBA
ENSXETG00000026068	2550	0	0	0	0	0	0	0	0	0	0	0	0	
ENSXETG00000026079	549	0	0	1	1	1	0	4	2	2	1	1	0	ENSXETG00000026079
ENSXETG00000026080	909	2	0	1	1	0	0	0	1	1	0	0	0	ENSXETG00000026080
ENSXETG00000026081	2427	0	0	0	0	0	0	0	0	0	0	0	0	
ENSXETG00000026087	1476	4	7	6	578	412	72	145	160	107	400	291	303	sfrp2
ENSXETG00000026093	2310	21	21	28	0	0	1	67	88	52	1	0	1	fga
ENSXETG00000026094	1116	185	320	275	276	207	321	414	528	315	789	534	626	ercc1
ENSXETG00000026097	1716	1	1	0	0	0	0	13	31	19	2	0	1	lrat
ENSXETG00000026098	4242	144	246	195	758	567	1206	1059	1190	756	1251	876	961	itsn2
ENSXETG00000026099	1621	3470	5153	4235	5119	3795	3526	1957	2356	1323	2256	1551	1765	herpud1
ENSXETG00000026100	6309	21	21	34	184	154	74	263	271	167	59	44	50	ENSXETG00000026100
ENSXETG00000026101	1786	2000	2841	2373	2218	1755	2351	5866	6653	3899	1907	1348	1434	fkbp1b
ENSXETG00000026102	978	0	0	0	1	0	0	0	0	0	0	0	0	ENSXETG00000026102
ENSXETG00000026103	300	138	217	181	187	136	285	301	339	210	178	129	130	ENSXETG00000026103
ENSXETG00000026104	1900	495	769	589	977	747	1951	1085	1282	729	830	576	614	st3gal6
ENSXETG00000026107	885	0	0	0	0	0	0	0	0	0	0	0	0	
ENSXETG00000026108	960	0	0	0	0	2	0	0	0	0	0	0	1	ENSXETG00000026108
ENSXETG00000026112	2589	33	81	64	167	144	307	423	518	329	282	208	265	gjb2
ENSXETG00000026113	381	0	0	0	0	0	0	0	0	0	0	0	0	
ENSXETG00000026114	381	0	0	0	0	0	0	0	0	0	0	0	0	
ENSXETG00000026117	1257	0	0	0	0	0	0	1	0	0	3	0	0	
ENSXETG00000026118	312	0	0	0	0	0	0	0	0	0	0	0	0	
ENSXETG00000026123	1710	0	0	0	0	0	0	0	0	0	0	0	0	
ENSXETG00000026124	1467	3	4	3	1	2	1	1	3	2	0	0	1	cpb2
ENSXETG00000026125	1047	0	0	0	0	0	0	0	0	0	0	0	0	
ENSXETG00000026131	1149	0	0	0	0	0	0	0	0	0	0	0	0	
ENSXETG00000026137	975	0	0	0	0	0	0	0	0	0	0	0	0	
ENSXETG00000026140	969	0	0	0	0	0	0	0	0	0	0	0	0	
ENSXETG00000026142	924	0	0	0	0	0	0	0	0	0	0	0	0	
ENSXETG00000026146	1341	544	804	741	692	568	1312	336	409	240	498	384	445	ENSXETG00000026146
ENSXETG00000026147	1362	490	751	618	866	612	1794	305	383	203	519	351	445	rnf14
ENSXETG00000026148	744	0	2	4	18	8	33	5	5	2	59	31	49	ENSXETG00000026148
ENSXETG00000026149	906	0	0	0	0	0	0	0	0	0	0	0	0	
ENSXETG00000026151	609	180	205	188	220	177	203	270	331	230	196	171	146	zgpat
ENSXETG00000026152	1755	692	988	855	806	693	765	940	1243	687	514	339	419	tsnax
ENSXETG00000026153	1107	6	4	5	0	1	9	60	115	54	25	10	21	ctsw
ENSXETG00000026154	1756	22	35	30	79	67	142	295	369	159	111	107	118	ccdc85b
ENSXETG00000026157	2583	254	334	279	393	347	664	871	1146	632	569	392	448	ENSXETG00000026157
ENSXETG00000026158	1795	0	0	0	9	5	0	2	5	3	94	69	65	six2
ENSXETG00000026160	2241	9	26	9	55	35	118	301	407	212	128	90	105	ENSXETG00000026160
ENSXETG00000026161	1520	37660	54055	45197	52755	41101	81273	70913	85189	49166	83905	57615	66659	cfl1
ENSXETG00000026163	1144	209	324	269	135	125	285	262	297	174	251	186	174	taf1d
ENSXETG00000026165	2963	60	98	91	165	129	134	240	274	159	74	79	76	bbs1
ENSXETG00000026166	1389	19	28	22	1	0	2	131	139	93	4	4	2	arl13a
ENSXETG00000026167	2592	37	45	44	349	240	390	1247	1405	872	693	466	556	epas1
ENSXETG00000026168	1007	541	787	689	643	530	820	805	925	510	806	582	621	pigf
ENSXETG00000026169	2124	43355	63600	54459	8328	6464	3841	463	519	308	745	525	638	XB-GENE-5904605
ENSXETG00000026171	1841	0	0	0	0	0	0	0	0	0	0	0	0	
ENSXETG00000026172	2142	3097	4466	3645	3078	2343	3664	3864	4534	2586	2261	1608	1832	ube2g1
ENSXETG00000026173	1787	327	495	402	893	700	1046	1520	1799	1058	1310	891	956	rela
ENSXETG00000026175	695	1691	2669	2250	2810	2103	4355	1797	2156	1329	6320	4532	4913	timm23
ENSXETG00000026176	1329	167	233	201	186	132	422	1316	1623	1020	316	221	229	ENSXETG00000026176
ENSXETG00000026177	1401	0	0	0	0	0	0	6	6	7	2	1	3	gabra3
ENSXETG00000026178	1450	1696	2254	1914	1325	1028	2228	898	1026	652	552	420	476	tmem254
ENSXETG00000026179	2167	75	97	76	95	77	95	128	163	89	116	82	83	ENSXETG00000026179
ENSXETG00000026182	1641	2127	2998	2666	1609	1302	1819	627	768	477	1075	734	891	tmem171
ENSXETG00000026184	2251	199	320	268	295	231	360	347	451	245	265	200	199	brcc3
ENSXETG00000026185	1549	76	136	114	277	177	202	387	455	269	237	184	212	ift57
ENSXETG00000026187	1338	147	180	162	205	139	174	251	292	185	221	155	128	gtf2h2
ENSXETG00000026188	360	5	7	3	42	19	44	45	82	42	76	62	60	ypel2
ENSXETG00000026189	528	41732	61368	52931	58495	45252	67812	63358	70816	41417	133129	90898	103175	rpl23a
ENSXETG00000026193	1362	1	0	0	0	0	2	22	37	17	24	9	13	ENSXETG00000026193
ENSXETG00000026196	1743	65	113	111	209	130	230	464	492	274	368	259	283	msl2
ENSXETG00000026198	1068	3	2	4	2	0	0	4	6	5	0	0	1	gpha2
ENSXETG00000026210	1804	536	762	649	492	350	762	805	892	508	504	338	409	rnft1
ENSXETG00000026214	1662	12	12	15	0	2	1	0	0	0	1	0	0	
ENSXETG00000026217	1510	1	0	0	0	0	0	0	0	0	0	0	0	
ENSXETG00000026218	2313	0	0	0	0	0	0	0	0	0	0	0	0	
ENSXETG00000026219	481	30039	45222	39021	34028	25422	37578	30772	34566	22117	66834	47666	50592	fau
ENSXETG00000026220	930	0	0	0	0	0	0	0	0	0	0	0	0	
ENSXETG00000026221	915	0	0	0	0	0	0	0	0	0	0	0	0	
ENSXETG00000026223	817	47664	69868	59063	19962	15023	35694	6342	7343	4105	14484	9936	11167	ENSXETG00000026223
ENSXETG00000026224	823	0	0	0	0	0	0	0	0	0	0	0	0	
ENSXETG00000026230	898	150	194	180	194	144	314	207	288	152	392	261	276	rpp21
ENSXETG00000026231	1377	242	338	305	524	406	406	601	631	435	605	438	432	prpf4
ENSXETG00000026232	372	5009	7415	6348	4339	3232	7091	765	917	523	1681	1161	1250	slc31a1
ENSXETG00000026233	1947	0	0	0	0	0	0	0	0	0	2	2	0	
ENSXETG00000026234	815	34	44	45	95	71	119	144	182	104	106	59	71	rpain
ENSXETG00000026235	3863	456	620	569	1162	912	1984	1750	1997	1169	1375	994	1047	trafd1
ENSXETG00000026236	1008	1812	2569	2064	1668	1303	3139	2214	2732	1525	1449	1054	1147	ube2b
ENSXETG00000026240	1470	2	19	7	8	6	31	123	134	87	31	21	27	omg
ENSXETG00000026242	615	15	26	18	41	26	53	196	265	156	62	45	51	fam109a
ENSXETG00000026245	1060	157	263	255	194	157	200	143	169	109	157	117	143	fam174a
ENSXETG00000026258	873	0	0	0	0	0	0	3	5	3	0	0	0	ENSXETG00000026258
ENSXETG00000026259	2764	0	0	0	0	0	0	0	0	0	0	0	0	
ENSXETG00000026261	510	0	0	0	0	0	0	0	0	0	0	0	0	
ENSXETG00000026266	1203	0	0	0	1	0	1	2	1	0	5	1	1	ENSXETG00000026266
ENSXETG00000026267	561	138	174	161	766	581	216	1206	1371	851	253	183	216	glrx
ENSXETG00000026269	1453	2216	3117	2757	2794	2174	4180	334	362	232	3112	2213	2361	akr1d1
ENSXETG00000026270	593	1177	1730	1423	703	513	405	1081	1327	659	340	255	264	timm9
ENSXETG00000026271	994	5	2	8	9	1	2	8	3	11	2	0	2	akr1c1
ENSXETG00000026272	477	1	2	1	0	0	0	6	6	2	1	1	5	ascl3

ENSXETG00000026274	1584	0	0	0	0	0	0	8	5	2	7	0	6	ENSXETG00000026274
ENSXETG00000026276	1920	24	38	31	20	19	71	43	66	47	35	33	30	rhog.2
ENSXETG00000026277	1923	11	12	10	15	12	31	17	27	11	14	15	15	ENSXETG00000026277
ENSXETG00000026278	1599	17	16	12	32	19	37	42	64	43	82	55	69	ENSXETG00000026278
ENSXETG00000026279	568	90	139	118	316	235	404	1420	1450	999	609	420	494	plscr3
ENSXETG00000026282	1481	1	3	5	11	6	1	0	0	1	12	10	8	MGC69466
ENSXETG00000026283	1359	993	1560	1296	3059	2318	1110	131	145	98	2089	1393	1547	gjb1
ENSXETG00000026284	908	106	170	135	816	613	517	2736	2958	1884	2268	1590	1635	GDPD3
ENSXETG00000026292	936	3	1	7	215	177	22	5	0	4	140	106	110	sox2
ENSXETG00000026296	1236	0	0	0	0	0	0	0	0	0	0	0	0	
ENSXETG00000026298	1603	3553	5193	4609	6419	4904	6829	6336	7121	4470	5830	4114	4425	pitpnb
ENSXETG00000026303	1779	0	3	0	1	0	2	10	7	5	1	2	0	tp63
ENSXETG00000026305	312	0	0	0	0	0	0	2	0	1	0	0	0	hist1h4c
ENSXETG00000026306	645	2	2	1	5	0	0	1	7	2	1	1	1	hist1h1d
ENSXETG00000026307	951	0	0	0	0	1	0	0	0	0	0	1	0	
ENSXETG00000026308	2199	32	36	23	48	53	36	67	108	71	34	24	21	haus2
ENSXETG00000026316	528	0	0	0	0	0	0	0	0	0	0	0	0	
ENSXETG00000026317	528	0	0	0	0	0	0	0	0	0	0	0	0	
ENSXETG00000026334	3726	9	9	16	47	31	56	157	197	111	121	85	100	cacnb1
ENSXETG00000026336	89	0	0	0	0	0	0	0	0	0	0	0	0	
ENSXETG00000026339	360	0	0	0	0	0	0	0	1	0	0	0	1	
ENSXETG00000026340	363	0	1	0	0	0	0	6	7	5	1	0	1	XB-GENE-5877002
ENSXETG00000026342	759	54	68	46	91	83	415	251	322	183	196	161	146	ENSXETG00000026342
ENSXETG00000026343	640	8	5	6	50	42	75	535	566	374	174	120	127	gap43
ENSXETG00000026344	1155	0	0	0	0	0	0	0	0	0	0	0	0	
ENSXETG00000026347	2877	2023	3000	2492	3781	3112	3293	2607	3325	1830	7426	5296	6098	slc25a28
ENSXETG00000026348	141	10	15	19	7	6	17	70	62	56	21	16	17	atp6v0e2
ENSXETG00000026349	249	0	0	0	0	0	0	0	0	0	0	0	1	
ENSXETG00000026351	930	2261	3476	2986	3922	3012	3444	5967	6726	4187	1871	1420	1498	psmb5
ENSXETG00000026353	624	0	0	0	0	0	0	0	0	0	0	0	0	
ENSXETG00000026355	4262	39	65	61	394	307	572	1382	1585	930	712	503	543	akap12
ENSXETG00000026358	597	0	0	0	0	0	0	0	0	0	0	0	0	
ENSXETG00000026359	1263	344	484	403	238	153	201	196	259	151	194	114	150	arv1
ENSXETG00000026360	4329	14	31	23	163	121	153	387	432	267	228	183	176	abcc6
ENSXETG00000026362	1797	784	1143	987	2582	1908	3831	1114	1143	704	2673	1810	1920	ENSXETG00000026362
ENSXETG00000026367	837	115	172	140	186	133	297	182	204	128	370	262	287	fra10ac1
ENSXETG00000026372	1574	51105	76118	66704	91242	69322	98302	102829	115465	71128	256750	182597	199353	eef1g
ENSXETG00000026373	5196	108	129	129	489	361	252	663	795	449	117	72	81	KIF20B
ENSXETG00000026376	4036	145	239	199	476	406	182	654	758	431	257	218	252	nsd2
ENSXETG00000026378	2753	75	146	88	234	161	450	347	470	261	464	322	344	XB-GENE-5729272
ENSXETG00000026389	465	0	0	0	0	0	0	0	0	0	0	0	0	
ENSXETG00000026391	1287	2	0	1	0	2	5	66	59	44	29	21	13	XB-GENE-5872721
ENSXETG00000026395	372	150	231	221	828	658	82	361	400	220	247	181	209	nmral1
ENSXETG00000026397	1488	0	0	0	0	0	0	1	0	0	0	0	0	
ENSXETG00000026399	832	37	42	40	127	100	97	187	233	141	201	165	192	dsn1
ENSXETG00000026404	1186	362	536	388	336	224	520	632	777	390	422	281	333	med10
ENSXETG00000026406	1338	79	101	125	158	124	239	329	430	251	305	169	248	ENSXETG00000026406
ENSXETG00000026410	3739	718	1035	892	1319	1030	1235	2725	3094	1892	1615	1083	1183	chd4
ENSXETG00000026411	1546	30	44	44	122	96	204	454	505	287	162	127	136	iffo1
ENSXETG00000026412	2298	0	0	0	0	0	1	4	2	1	0	0	0	
ENSXETG00000026414	2379	538	746	660	703	550	701	904	1092	646	541	384	420	XB-GENE-1017156
ENSXETG00000026415	477	29611	44913	40374	44823	34044	49816	45171	50970	31905	108704	72706	80362	rps25
ENSXETG00000026417	1349	1174	1731	1521	1228	904	1760	997	1280	771	807	566	666	tmx2
ENSXETG00000026419	939	0	0	0	0	0	0	0	0	0	0	0	0	
ENSXETG00000026421	255	70	97	107	249	222	534	1394	1464	1160	1362	917	1140	ENSXETG00000026421
ENSXETG00000026423	1308	12	19	8	47	36	24	108	108	62	26	14	19	neurog3
ENSXETG00000026426	1173	0	0	1	23	15	0	2	3	1	4	1	3	fn3k
ENSXETG00000026430	2802	0	0	0	0	0	0	2	1	2	0	0	0	
ENSXETG00000026432	366	273	389	357	465	355	844	194	248	168	284	189	239	ENSXETG00000026432
ENSXETG00000026434	1084	233	362	279	187	143	228	294	378	223	172	116	129	chchd7
ENSXETG00000026435	546	0	0	0	0	0	0	0	0	0	0	0	0	
ENSXETG00000026436	3005	17448	24388	20441	22738	17866	26548	19512	23571	12943	24134	16919	18699	eif5a
ENSXETG00000026438	1361	0	0	0	0	0	0	0	0	0	0	0	0	sox17b.1
ENSXETG00000026439	1587	4	7	5	4	0	0	1	2	2	0	1	0	samd7
ENSXETG00000026441	399	0	0	0	0	0	0	0	7	0	0	0	0	
ENSXETG00000026442	342	0	0	1	0	0	0	1	0	0	0	0	0	
ENSXETG00000026447	1981	43265	59017	51682	47085	36769	108467	15199	16093	11058	79772	55347	61787	ucp2
ENSXETG00000026450	1471	157	252	209	514	379	621	1051	1212	670	915	630	680	pold3
ENSXETG00000026454	327	7775	11872	10773	11413	8905	14035	10897	11964	8019	25380	18008	19181	rps13
ENSXETG00000026455	942	0	0	0	0	0	0	0	0	0	0	0	0	
ENSXETG00000026456	1341	0	2	4	18	13	25	77	84	45	53	41	29	rims2
ENSXETG00000026457	2675	6216	9336	8140	16318	12679	11158	8727	9259	5758	8124	5809	6140	hadha
ENSXETG00000026458	1479	46	60	62	144	113	255	179	227	150	263	212	231	sox5
ENSXETG00000026459	2177	6727	9771	8246	6196	4814	6332	13788	15586	9401	5261	3659	4034	ywhaz
ENSXETG00000026460	2574	104	203	164	34	37	103	78	84	66	173	115	144	cdh26
ENSXETG00000026463	1176	73	108	77	83	64	83	119	158	98	75	50	57	zdhhc21
ENSXETG00000026465	246	131	172	147	112	83	103	261	288	217	106	67	79	lsm5
ENSXETG00000026468	360	0	0	0	0	0	0	3	0	0	0	0	0	XB-GENE-5941156
ENSXETG00000026470	2343	0	0	0	0	0	0	0	0	0	0	0	0	
ENSXETG00000026471	1823	201	270	274	282	225	428	567	628	375	415	294	314	rabgef1
ENSXETG00000026473	943	42	80	56	139	126	84	535	567	328	21	15	9	fam64a
ENSXETG00000026475	1338	0	0	0	1	0	0	0	0	0	0	0	1	
ENSXETG00000026476	252	22	29	22	38	49	47	82	102	46	20	18	18	arl6
ENSXETG00000026477	1242	0	0	0	0	0	0	0	0	0	2	1	1	ENSXETG00000026477
ENSXETG00000026478	1212	0	0	0	0	0	0	1	0	0	0	0	0	
ENSXETG00000026482	1428	0	0	0	0	0	0	0	0	0	0	0	0	
ENSXETG00000026483	573	3065	4462	3800	2521	1954	2590	2752	3191	1985	1391	1023	987	dad1
ENSXETG00000026484	1425	0	0	0	0	0	0	0	0	0	0	0	0	
ENSXETG00000026487	2021	1	6	4	6	2	1	50	77	30	101	72	72	ENSXETG00000026487
ENSXETG00000026488	1884	30	31	38	48	52	89	450	537	321	69	29	62	p2ry13
ENSXETG00000026489	1303	0	1	1	1	0	0	2	3	4	3	1	2	cadm2
ENSXETG00000026490	960	0	0	0	0	1	1	1	0	0	0	0	0	ENSXETG00000026490
ENSXETG00000026492	951	0	0	0	0	0	0	0	0	0	0	0	0	
ENSXETG00000026496	1308	0	0	0	5	1	6	14	9	7	14	9	8	drd5
ENSXETG00000026497	2091	0	0	0	0	0	0	0	0	0	0	0	0	
ENSXETG00000026500	1437	0	1	0	2	0	4	10	1	4	0	4	0	gjd4
ENSXETG00000026501	1645	488	685	568	1095	852	1592	1777	2023	1193	1081	739	877	XB-GENE-5885309
ENSXETG00000026503	390	0	0	0	0	0	0	0	0	0	0	0	1	
ENSXETG00000026506	735	314	437	338	394	300	488	436	528	296	225	151	173	iah1
ENSXETG00000026511	4805	158	193	203	364	269	639	1414	1592	952	750	551	648	ENSXETG00000026511
ENSXETG00000026514	240	6	19	16	73	58	200	374	450	265	410	276	306	fxyd1
ENSXETG00000026515	869	490	713	633	706	577	1246	1170	1306	816	863	593	726	stx7
ENSXETG00000026517	1767	0	0	0	0	0	0	0	0	2	9	1	6	gad2
ENSXETG00000026518	753	0	0	0	0	0	0	0	0	1	0	0	0	
ENSXETG00000026520	573	119	153	145	66	52	72	53	56	33	63	33	43	rmnd1
ENSXETG00000026528	1350	9	5	11	54	39	81	570	598	368	173	107	122	havcr1
ENSXETG00000026530	3965	673	899	766	1073	775	1436	1132	1475	930	1162	841	939	man2a1
ENSXETG00000026537	1245	1138	1635	1473	1205	950	2201	2184	2657	1560	1012	751	841	gadd45g
ENSXETG00000026538	1209	0	0	0	0	0	0	0	0	0	0	0	0	ENSXETG00000026538
ENSXETG00000026542	1068	0	0	0	1	0	0	0	0	0	0	0	0	
ENSXETG00000026544	510	0	0	0	0	0	0	0	0	0	0	0	0	

ENSXETG00000026547	1674	0	1	0	4	1	0	8	7	12	25	10	18	ENSXETG00000026547
ENSXETG00000026548	921	0	0	0	0	0	0	0	0	0	0	0	0	
ENSXETG00000026549	885	0	0	0	0	0	0	0	0	0	0	0	0	
ENSXETG00000026552	939	0	0	0	0	0	0	0	0	0	0	0	0	
ENSXETG00000026554	991	0	0	0	0	0	0	0	0	0	0	0	0	
ENSXETG00000026555	954	0	0	0	0	0	0	0	0	0	0	0	0	
ENSXETG00000026556	942	0	0	0	0	0	0	0	0	0	0	0	0	
ENSXETG00000026557	963	0	0	1	1	0	0	0	0	0	0	0	0	
ENSXETG00000026558	930	0	0	0	0	0	0	0	0	0	0	0	0	
ENSXETG00000026559	3209	146	238	173	271	204	587	1078	1266	718	483	364	378	gpr157
ENSXETG00000026561	954	0	0	0	0	0	0	0	0	0	0	0	0	
ENSXETG00000026562	625	332	490	380	292	239	219	391	532	311	286	174	208	coa5
ENSXETG00000026564	915	548	792	629	583	430	732	646	801	487	536	382	475	unc50
ENSXETG00000026565	725	1132	1717	1411	1040	731	1169	1067	1315	743	965	662	724	atox1
ENSXETG00000026569	555	8	9	5	153	106	141	331	361	223	405	250	319	nmu
ENSXETG00000026570	939	0	0	0	0	0	0	0	0	0	0	0	0	
ENSXETG00000026571	546	777	1152	958	492	387	502	674	727	418	485	312	381	mrpl30
ENSXETG00000026572	2038	0	0	0	0	0	0	0	0	0	0	0	0	
ENSXETG00000026574	774	337	474	380	290	214	288	377	427	294	194	177	157	tmem165
ENSXETG00000026575	957	0	0	0	0	0	0	0	0	0	0	0	0	
ENSXETG00000026576	1260	285	488	356	224	177	354	350	427	216	290	176	235	tm2d3
ENSXETG00000026577	915	0	0	0	0	0	0	0	0	0	0	0	0	
ENSXETG00000026578	1038	580	760	618	421	347	697	672	864	489	538	369	410	sft2d1
ENSXETG00000026579	414	0	0	0	0	0	0	0	1	0	0	0	0	ENSXETG00000026579
ENSXETG00000026581	2604	0	0	0	0	0	0	0	0	0	0	0	0	
ENSXETG00000026582	939	0	0	0	0	0	0	0	0	0	0	0	0	
ENSXETG00000026583	330	242	353	312	494	319	550	598	714	423	414	316	336	yipf4
ENSXETG00000026585	2568	351	516	433	792	563	1168	522	636	363	1051	780	859	nudt4
ENSXETG00000026587	4722	7	7	10	207	150	58	78	98	82	67	40	44	ENSXETG00000026587
ENSXETG00000026592	1440	687	891	801	315	212	746	81	101	70	172	104	155	ENSXETG00000026592
ENSXETG00000026594	907	3225	4634	4006	4944	3905	10081	6440	7534	4302	7965	5537	6178	btg1
ENSXETG00000026595	264	0	0	0	1	0	0	1	1	0	4	1	1	pde6h
ENSXETG00000026596	1551	0	0	1	2	2	5	2	2	0	10	8	6	XB-GENE-5799769
ENSXETG00000026599	1188	30	29	30	11	11	29	10	11	7	7	10	13	ENSXETG00000026599
ENSXETG00000026600	2413	0	2	1	32	23	5	2	5	2	2	4	1	ENSXETG00000026600
ENSXETG00000026601	836	4	8	5	189	145	20	19	24	11	21	6	9	gsto1
ENSXETG00000026602	920	3357	5053	4157	5859	4541	4780	212	204	94	1078	741	810	gsto2
ENSXETG00000026605	936	0	0	0	0	0	0	0	0	0	0	0	0	
ENSXETG00000026606	417	0	0	0	0	0	0	2	5	2	17	3	12	
ENSXETG00000026608	942	0	0	0	0	0	0	0	0	0	0	0	0	
ENSXETG00000026609	951	0	0	0	0	0	0	0	0	0	0	0	0	
ENSXETG00000026611	960	0	0	0	0	0	0	0	0	0	0	0	0	
ENSXETG00000026612	525	0	0	0	0	0	0	0	0	0	0	0	0	
ENSXETG00000026622	1649	106	121	107	196	174	282	266	322	176	314	245	244	mbd4
ENSXETG00000026627	2448	1	1	0	3	0	0	28	40	28	114	72	98	ENSXETG00000026627
ENSXETG00000026629	3450	0	7	2	5	4	2	22	30	14	10	6	4	itgam
ENSXETG00000026630	1255	2	6	4	21	15	3	276	339	185	59	41	31	
ENSXETG00000026634	1008	0	0	0	0	0	0	0	0	0	0	0	0	
ENSXETG00000026635	2050	1311	1946	1631	3353	2695	3960	5341	6075	3711	2798	1834	2174	znf207
ENSXETG00000026639	5214	183	302	277	621	491	910	963	1000	619	1237	870	962	qser1
ENSXETG00000026640	579	24	44	32	45	32	52	63	66	32	48	32	57	ENSXETG00000026640
ENSXETG00000026642	1449	88	120	110	58	45	111	110	138	82	90	47	79	rce1
ENSXETG00000026647	1080	0	0	0	1	0	1	0	2	2	1	0	1	c17orf58
ENSXETG00000026649	1754	359	483	410	904	674	1147	1391	1611	918	1312	973	949	rbm4b
ENSXETG00000026650	2541	197	309	243	507	371	1234	1936	2203	1339	1102	707	784	rbm14
ENSXETG00000026651	5954	0	1	0	15	7	0	1	0	1	4	1	1	myh1
ENSXETG00000026656	2140	4632	6751	5636	8916	6832	7130	5501	6334	3841	5522	3922	4404	tktl2
ENSXETG00000026658	450	59	72	61	54	40	56	21	33	16	82	71	84	tnfsf15
ENSXETG00000026659	966	0	0	1	0	0	0	2	1	0	1	1	0	
ENSXETG00000026661	975	0	2	0	1	4	8	7	6	3	6	10	7	ENSXETG00000026661
ENSXETG00000026662	1561	31	35	30	32	25	54	458	614	305	90	52	65	rgs18
ENSXETG00000026664	4369	290	443	385	683	482	779	898	1013	575	939	684	758	ERCC6
ENSXETG00000026666	1413	1	0	0	2	0	0	1	6	7	2	0	2	ENSXETG00000026666
ENSXETG00000026667	1797	1	2	1	2	1	1	18	22	19	15	8	10	ENSXETG00000026667
ENSXETG00000026669	381	0	0	0	0	0	0	0	0	0	0	0	0	
ENSXETG00000026672	2412	0	0	0	0	0	1	0	0	0	0	0	0	
ENSXETG00000026673	1590	57	75	61	251	164	297	471	481	327	513	406	404	ENSXETG00000026673
ENSXETG00000026675	360	1	2	0	0	1	0	0	0	0	0	0	0	hist1h2aj
ENSXETG00000026676	970	0	0	0	0	0	0	0	0	0	0	0	0	
ENSXETG00000026677	752	36	43	56	49	40	78	106	115	71	62	27	47	rab28
ENSXETG00000026678	381	0	2	0	2	1	2	1	3	2	0	2	1	ENSXETG00000026678
ENSXETG00000026679	312	0	0	0	0	0	0	0	0	0	0	0	0	
ENSXETG00000026680	381	0	0	0	0	0	0	0	0	0	0	0	0	ENSXETG00000026680
ENSXETG00000026681	1577	7	2	6	3	0	2	2	2	1	0	0	0	darmin
ENSXETG00000026682	393	0	0	0	0	0	0	0	0	0	0	0	0	ENSXETG00000026682
ENSXETG00000026684	2027	3397	4843	4011	4163	3112	6889	7001	8055	4736	4425	2996	3397	ensa
ENSXETG00000026686	939	0	0	0	0	0	0	0	0	0	0	0	0	
ENSXETG00000026687	1629	10	18	10	8	6	30	47	59	46	38	9	17	ENSXETG00000026687
ENSXETG00000026688	1509	0	0	3	1	0	1	197	282	163	58	41	52	ENSXETG00000026688
ENSXETG00000026690	924	108	156	148	187	120	251	447	532	291	391	279	290	XB-GENE-5897958
ENSXETG00000026691	942	0	0	0	0	0	0	0	0	0	0	0	0	
ENSXETG00000026694	921	0	0	0	0	0	0	0	0	0	0	0	0	
ENSXETG00000026695	942	0	0	0	0	0	0	0	0	0	0	0	0	
ENSXETG00000026697	894	0	0	0	0	0	0	0	0	0	0	0	0	
ENSXETG00000026702	948	0	0	0	0	0	0	0	0	0	0	0	0	
ENSXETG00000026703	849	54	81	68	94	69	160	142	145	114	164	88	112	cldn23
ENSXETG00000026704	678	2454	3601	3046	1742	1323	1487	397	467	239	733	514	625	XB-GENE-5780700
ENSXETG00000026706	1497	5	11	6	11	10	12	10	13	7	4	5	2	XB-GENE-5771028
ENSXETG00000026707	1296	1	0	0	0	0	0	8	1	1	7	2	3	ENSXETG00000026707
ENSXETG00000026711	303	0	0	0	0	0	0	0	0	0	0	0	0	ENSXETG00000026711
ENSXETG00000026712	399	0	0	0	0	1	0	0	0	1	0	0	0	
ENSXETG00000026713	411	0	0	0	0	0	1	0	1	0	0	1	0	
ENSXETG00000026715	2496	145	215	164	400	308	203	1692	2139	1112	314	188	254	spef1
ENSXETG00000026716	3547	3327	4944	4196	9507	7127	17015	39404	45412	27184	14637	10202	11389	itgb1
ENSXETG00000026722	1058	14	25	20	1	0	0	28	35	14	0	0	0	ambp
ENSXETG00000026726	399	0	0	0	0	0	0	2	2	1	0	0	0	
ENSXETG00000026729	363	0	0	0	1	1	0	1	5	3	0	0	0	
ENSXETG00000026734	1047	20	27	18	12	4	14	1071	1234	709	127	94	101	ENSXETG00000026734
ENSXETG00000026741	525	0	0	0	0	0	0	0	0	0	0	0	1	
ENSXETG00000026743	528	0	0	0	0	0	0	0	0	0	0	0	0	
ENSXETG00000026745	586	0	0	0	0	0	0	0	0	0	1	0	0	crygb
ENSXETG00000026752	1772	2	3	0	0	0	4	1	1	0	0	1	1	trim72
ENSXETG00000026753	2586	0	0	0	0	0	0	0	0	0	0	0	0	
ENSXETG00000026755	1772	4	3	2	6	4	6	1	4	0	0	0	2	ENSXETG00000026755
ENSXETG00000026762	928	0	0	0	0	0	0	0	0	0	0	0	0	
ENSXETG00000026763	2564	1816	2656	2234	701	601	1945	1354	1697	962	1128	765	839	cdipt
ENSXETG00000026765	709	1112	1452	1265	883	666	1588	1580	1728	1048	1097	785	854	lamtor3-like
ENSXETG00000026767	2088	1604	2305	1970	6451	4888	12041	5109	5229	3305	19944	13801	14855	XB-GENE-5854345
ENSXETG00000026768	13368	123451	173735	146283	195153	149565	223628	62256	77084	42667	47160	33312	38921	APOB
ENSXETG00000026772	2024	1814	2660	2332	1751	1359	2656	7775	8837	5588	3576	2524	2789	rhob
ENSXETG00000026776	540	1986	2774	2290	2726	2349	3723	4658	5434	3164	3474	2444	2804	rap1b

ENSXETG00000026777	3761	184	246	231	260	162	479	343	408	245	305	191	225	scly
ENSXETG00000026783	990	399	571	469	383	312	506	844	1089	649	376	291	296	yeats4
ENSXETG00000026787	1443	2	2	7	4	2	3	16	12	5	5	1	2	sag
ENSXETG00000026788	3381	35	48	31	151	121	348	830	924	560	321	249	243	plekhb2
ENSXETG00000026789	1059	20	34	23	192	145	315	482	571	314	231	153	173	gja4
ENSXETG00000026791	1958	1264	1769	1387	1273	909	1115	1952	2403	1316	488	328	371	zmpste24
ENSXETG00000026793	1485	0	1	1	68	46	5	4	2	2	27	20	16	tmem30b
ENSXETG00000026794	4275	270	354	302	361	282	513	572	728	409	736	531	606	rlf
ENSXETG00000026796	1008	0	0	0	0	0	0	0	0	0	0	0	0	
ENSXETG00000026797	672	8998	12876	11200	7063	5122	13619	6819	8462	4954	7014	4958	5683	cldn15.2
ENSXETG00000026798	1276	0	0	0	0	0	0	0	0	0	0	0	0	
ENSXETG00000026801	948	0	0	0	0	0	0	0	0	0	0	0	0	
ENSXETG00000026802	701	860	1346	1069	1277	971	2667	3175	3864	2215	2134	1595	1715	tmem50a
ENSXETG00000026803	781	20	46	36	11	10	14	65	81	49	1	1	3	otos
ENSXETG00000026804	2519	67	63	69	107	87	196	409	506	304	239	190	209	ptprr
ENSXETG00000026806	586	613	842	722	564	472	596	989	1140	728	414	272	335	cops9
ENSXETG00000026807	1108	5721	7942	6682	9783	7370	8477	5487	7035	3771	7926	5630	6588	tspan8
ENSXETG00000026808	198	1	0	0	1	0	2	0	0	0	1	0	4	serp2
ENSXETG00000026816	1449	146	192	154	78	49	385	19	14	8	175	119	121	ENSXETG00000026816
ENSXETG00000026817	1245	0	1	1	0	0	1	0	1	1	0	0	0	slc7a13
ENSXETG00000026818	454	51915	77052	66965	38397	29120	17604	739	916	505	14098	9904	10980	rbp2
ENSXETG00000026821	1497	3	1	2	81	42	9	31	35	16	8	5	6	daw1
ENSXETG00000026822	1659	1	1	6	0	1	4	96	121	59	14	7	7	slc19a3
ENSXETG00000026823	1701	607	877	700	708	511	869	1057	1280	766	688	541	537	pphln1
ENSXETG00000026827	1701	76	108	65	176	144	173	182	232	157	121	87	98	fbxo36
ENSXETG00000026830	1808	960	1396	1238	897	681	1348	1321	1608	899	633	524	540	twf1
ENSXETG00000026832	978	0	0	0	0	0	0	0	0	0	0	0	0	ENSXETG00000026832
ENSXETG00000026833	975	0	0	0	0	0	0	0	0	0	0	0	0	
ENSXETG00000026834	909	0	0	0	0	0	0	0	0	0	0	0	0	
ENSXETG00000026835	981	0	0	0	0	0	0	0	1	0	1	1	0	or52r1
ENSXETG00000026836	1797	23	30	39	98	61	189	142	145	125	171	92	132	ctif
ENSXETG00000026838	423	2669	3962	3306	3527	2591	3132	2599	3010	1739	2057	1467	1547	atp5i
ENSXETG00000026840	684	1185	1816	1480	1218	920	1734	1419	1649	856	998	672	695	c18orf32
ENSXETG00000026841	954	0	0	0	0	0	0	0	0	0	0	0	0	
ENSXETG00000026842	1528	68	97	78	96	118	144	574	660	390	227	160	189	gng7
ENSXETG00000026845	1499	9	4	3	4	2	4	1	0	0	1	0	1	ENSXETG00000026845
ENSXETG00000026846	1510	1	1	0	21	16	15	85	131	62	27	26	27	sncb
ENSXETG00000026847	1377	582	806	711	748	577	957	835	1006	565	809	522	568	c19orf25
ENSXETG00000026853	348	0	0	0	0	0	0	2	3	0	0	1	0	
ENSXETG00000026854	360	0	0	0	0	0	0	1	10	2	1	1	1	
ENSXETG00000026857	1040	566	883	728	755	598	1122	997	1026	670	780	579	571	mospd3
ENSXETG00000026858	813	304	399	357	215	174	375	317	401	245	294	194	234	slc25a26
ENSXETG00000026859	1513	775	1096	939	983	750	896	1378	1563	916	1520	1050	1202	utp3
ENSXETG00000026864	1626	11	16	13	79	68	293	297	406	232	358	234	270	ms4a1
ENSXETG00000026865	5159	364	484	433	590	449	926	1151	1389	756	984	707	796	prdm2
ENSXETG00000026866	486	0	0	0	0	0	0	0	0	0	0	0	0	ENSXETG00000026866
ENSXETG00000026867	399	936	1429	1198	1087	735	1064	1882	2187	1425	942	652	665	eif6
ENSXETG00000026872	1158	0	0	0	0	0	0	2	4	4	1	0	2	ENSXETG00000026872
ENSXETG00000026873	3552	0	0	0	0	0	0	0	0	0	0	0	1	ENSXETG00000026873
ENSXETG00000026876	13485	1103	1599	1418	8528	6379	9042	13343	14781	9153	17389	12142	13849	plec
ENSXETG00000026877	489	0	0	0	0	0	0	0	0	0	0	0	0	
ENSXETG00000026881	507	225	405	308	201	185	335	437	503	310	322	244	240	nr2c2ap
ENSXETG00000026886	2046	2427	3347	2728	3714	2829	5428	4238	5178	3114	8842	6171	6781	plpp2
ENSXETG00000026888	942	0	3	1	0	0	0	17	10	8	1	0	4	ENSXETG00000026888
ENSXETG00000026892	1303	882	1245	1135	1111	928	948	2071	2293	1505	1226	831	906	slc35a3.2
ENSXETG00000026893	2405	85	127	128	17	24	25	55	74	37	13	10	15	apcdd1
ENSXETG00000026897	888	1863	2625	2019	1534	1209	1655	3010	3526	1939	1253	922	1057	nutf2
ENSXETG00000026898	372	506	773	602	949	670	792	738	806	504	748	522	485	fam162a
ENSXETG00000026899	1779	0	0	2	1	1	1	1	0	0	1	0	0	thdl18
ENSXETG00000026901	2272	417	511	473	698	509	1095	490	562	325	1182	833	940	vrk3
ENSXETG00000026902	898	0	1	2	257	206	66	11	9	10	2157	1518	1691	ctrb1
ENSXETG00000026907	1236	669	946	791	274	207	293	522	633	325	147	136	134	trmt61a
ENSXETG00000026908	738	12	25	21	32	27	65	82	96	53	88	71	57	znf721
ENSXETG00000026914	462	85	121	104	7	5	23	19	25	10	15	18	15	ENSXETG00000026914
ENSXETG00000026919	1953	54	91	95	81	67	253	226	221	148	269	145	184	shisa4
ENSXETG00000026920	2340	0	0	0	0	0	0	0	0	0	0	0	0	
ENSXETG00000026921	5287	32	50	38	240	223	234	767	856	548	413	282	319	DIP2C
ENSXETG00000026924	861	0	0	0	0	0	0	0	0	0	0	0	0	ENSXETG00000026924
ENSXETG00000026925	2156	0	0	0	1	0	1	5	0	0	0	0	1	ENSXETG00000026925
ENSXETG00000026926	1702	0	0	0	0	0	0	0	1	0	0	0	0	
ENSXETG00000026927	1913	215	313	295	244	218	230	9	16	7	92	77	49	ENSXETG00000026927
ENSXETG00000026928	1488	630	945	763	821	594	1022	82	95	55	747	554	652	ENSXETG00000026928
ENSXETG00000026930	1461	33	40	33	54	50	64	121	155	76	67	44	54	XB-GENE-5768835
ENSXETG00000026931	1400	336	548	464	409	322	1055	209	252	137	335	254	279	XB-GENE-5870754
ENSXETG00000026932	993	22	56	39	79	62	96	73	100	49	76	91	92	ENSXETG00000026932
ENSXETG00000026933	562	120	165	166	0	0	0	349	354	281	0	0	0	bid
ENSXETG00000026934	562	102	149	124	175	139	276	179	195	135	169	117	110	bid
ENSXETG00000026938	2359	691	993	747	1451	1099	1792	2337	2778	1595	2283	1584	1707	sppl3
ENSXETG00000026940	1297	3492	5100	4361	2781	2016	2925	4147	4590	2779	2526	1979	2047	srsf9
ENSXETG00000026941	1914	0	1	3	2	0	0	1	4	2	1	1	0	ENSXETG00000026941
ENSXETG00000026942	1111	72393	104483	88887	109908	83151	124739	105611	119279	70978	218066	153221	166657	rplp0
ENSXETG00000026943	1445	1221	1757	1469	1536	1221	2552	2634	3174	1912	2032	1300	1568	rab35
ENSXETG00000026944	719	370	525	410	294	243	508	631	821	409	403	277	307	gtf2a2
ENSXETG00000026945	1034	1415	2084	1738	1376	1007	2020	1807	2128	1197	1072	740	839	prkab1
ENSXETG00000026948	980	196	210	165	132	104	221	143	183	110	192	109	122	hscb
ENSXETG00000026950	1095	391	513	434	608	474	943	1202	1367	776	944	663	773	ifngr2.2
ENSXETG00000026953	636	5	5	2	17	15	4	2	2	0	3	1	3	crybb3
ENSXETG00000026955	696	387	640	532	769	589	1138	1303	1395	981	856	614	656	ENSXETG00000026955
ENSXETG00000026961	2404	460	667	579	499	423	1250	1769	2023	1227	1089	798	896	march8
ENSXETG00000026962	393	0	0	0	0	0	0	0	0	0	0	0	0	
ENSXETG00000026963	381	0	0	0	0	0	0	0	0	0	0	0	0	ENSXETG00000026963
ENSXETG00000026964	367	13	17	14	6	3	32	39	59	28	235	184	193	msmb.1
ENSXETG00000026965	383	0	0	0	0	0	0	1	0	0	0	0	0	hist1h4c
ENSXETG00000026966	312	0	0	0	0	0	0	0	0	0	0	0	0	hist1h4c
ENSXETG00000026967	381	0	0	1	0	0	0	0	0	0	0	0	0	
ENSXETG00000026968	393	0	0	0	1	0	1	0	0	0	1	0	0	hist1h2ah
ENSXETG00000026971	381	0	0	0	0	0	0	0	0	0	0	0	0	
ENSXETG00000026973	360	1021	1491	1321	1030	818	1109	1766	1971	1221	1184	801	866	ubl5
ENSXETG00000026974	384	168	234	206	342	265	606	456	498	272	494	356	395	ube2e2
ENSXETG00000026975	1660	270	347	282	428	321	657	1032	1104	687	627	434	499	ube2e1
ENSXETG00000026976	1600	4723	6912	5868	1391	1014	3249	2988	3542	1980	3100	2201	2432	ddit4
ENSXETG00000026977	2073	40	68	42	26	11	22	8	9	4	29	26	28	med1
ENSXETG00000026979	2487	5474	7796	6503	5935	4605	9334	8394	9734	5619	8089	5926	6578	sar1a
ENSXETG00000026980	2157	1521	2175	1945	1073	856	781	145	200	112	1126	734	895	ENSXETG00000026980
ENSXETG00000026982	1109	0	0	0	5	4	0	1	3	1	7	6	4	cela3b
ENSXETG00000026983	1559	2027	2972	2466	3798	2892	3148	5861	6976	4023	3713	2573	2905	rbbp7
ENSXETG00000026984	1026	0	1	2	1	0	0	1	0	0	2	2	2	
ENSXETG00000026985	461	394	552	426	186	148	256	320	386	195	235	152	175	cmc1
ENSXETG00000026988	1463	44	55	59	138	102	187	193	222	134	55	26	35	prr11
ENSXETG00000026990	738	1839	2868	2430	2206	1722	2816	4872	5504	3234	1720	1211	1323	pomp
ENSXETG00000026998	1784	113	194	142	511	400	297	206	211	146	1021	715	762	foxa2

ENSXETG00000027007	969	901	1326	1152	576	433	526	1108	1276	749	309	243	244	sdf2
ENSXETG00000027008	1243	4060	5966	5304	6729	5306	9034	7425	7987	5225	19687	13924	16089	eif3g
ENSXETG00000027010	2076	422	579	541	672	482	960	111	106	56	631	424	491	XB-GENE-5852427
ENSXETG00000027011	1050	35	69	48	80	41	145	462	613	335	181	132	163	s1pr2
ENSXETG00000027013	1276	497	723	602	748	570	1003	584	581	368	728	525	532	lcmt1
ENSXETG00000027014	2615	125	213	170	513	397	795	1364	1572	956	949	731	809	traf4
ENSXETG00000027021	2168	1088	1633	1343	900	674	1429	2001	2506	1466	918	647	809	pdcd6
ENSXETG00000027024	318	29449	44280	37594	39687	29542	41301	42343	46583	28723	92019	65199	68276	rpl37
ENSXETG00000027025	1242	372	507	448	660	475	477	403	482	285	643	467	615	galnt12
ENSXETG00000027032	944	208	289	223	198	151	260	309	408	189	181	148	165	gins1
ENSXETG00000027034	407	0	0	0	0	0	0	0	0	0	0	0	0	
ENSXETG00000027035	1573	314	376	344	381	332	682	689	860	536	473	364	412	tmem251
ENSXETG00000027037	1248	0	0	0	0	0	0	0	0	1	0	0	0	
ENSXETG00000027041	2376	0	0	0	0	0	0	0	0	0	0	0	0	
ENSXETG00000027042	2577	34	66	38	28	28	106	170	215	130	136	104	91	ago3
ENSXETG00000027050	1403	80	130	103	171	125	269	274	315	199	259	147	211	ENSXETG00000027050
ENSXETG00000027052	552	979	1310	1111	447	322	536	589	750	376	449	310	338	mrps10
ENSXETG00000027055	4438	4	2	8	6	9	9	12	10	10	6	3	5	umodl1
ENSXETG00000027057	3057	153	189	173	210	192	362	391	472	269	395	302	294	zbtb21
ENSXETG00000027058	1910	1035	1360	1102	771	547	1020	792	1052	512	357	233	240	hint3
ENSXETG00000027059	540	0	0	0	0	0	0	4	2	2	1	2	3	ENSXETG00000027059
ENSXETG00000027060	2046	646	957	823	1468	1177	1125	1664	1920	1092	1112	750	813	nup85
ENSXETG00000027061	3655	300	531	439	765	603	1280	1383	1560	949	897	658	706	vps16
ENSXETG00000027063	1734	511	731	600	780	615	633	1269	1422	804	864	620	662	mif4gd
ENSXETG00000027064	678	0	0	0	0	0	1	1	1	0	0	1	1	
ENSXETG00000027066	2842	474	783	613	894	687	798	1561	1712	1075	1152	762	880	hid1
ENSXETG00000027068	2168	9	12	14	24	19	9	4	10	1	7	6	12	otop3
ENSXETG00000027069	1013	4	12	10	43	28	120	228	253	189	140	109	128	tmem47
ENSXETG00000027071	579	837	1121	961	795	642	769	993	1063	653	1134	841	851	ENSXETG00000027071
ENSXETG00000027073	1110	55876	83700	71445	96494	71927	112835	93504	106667	63788	224972	155941	173326	rps4x
ENSXETG00000027075	1686	188	229	201	237	163	444	591	748	369	492	312	378	cradd.1
ENSXETG00000027076	3563	23	23	26	36	32	89	182	249	125	37	32	41	tlr7
ENSXETG00000027078	4024	1354	1955	1617	392	315	197	419	542	305	170	117	158	ptdss2
ENSXETG00000027080	1968	699	894	732	555	480	691	644	860	451	363	289	302	trappc2
ENSXETG00000027081	420	172	264	245	198	130	282	398	443	261	223	161	145	trappc2l
ENSXETG00000027084	1185	1691	2343	2091	1082	851	2203	655	777	441	1534	1049	1193	aprt
ENSXETG00000027086	1698	799	1208	944	1498	1177	2005	1737	1872	1206	1945	1356	1525	ythdf1
ENSXETG00000027088	1050	206	313	279	541	446	239	404	430	247	318	254	267	fut4
ENSXETG00000027089	1271	0	2	0	6	6	0	1	0	0	1	1	1	fut3
ENSXETG00000027090	2399	137	209	179	442	294	870	8002	9189	5472	1385	892	1044	ENSXETG00000027090
ENSXETG00000027092	966	0	0	0	0	0	0	0	0	0	0	0	0	
ENSXETG00000027093	1536	25	53	39	374	280	244	1007	1107	659	330	207	268	ENSXETG00000027093
ENSXETG00000027097	1835	2336	3235	2687	1588	1329	1734	2584	3145	1838	1762	1287	1497	arpp19
ENSXETG00000027098	2403	0	0	0	0	0	0	0	0	0	1	0	0	
ENSXETG00000027099	3475	654	906	823	1754	1358	2490	1185	1328	775	1449	1037	1129	ubtd1
ENSXETG00000027100	1859	11	6	12	99	61	79	155	216	128	188	136	172	ism2
ENSXETG00000027101	2493	0	0	0	0	0	0	0	0	0	0	0	0	
ENSXETG00000027103	1773	0	0	0	1	1	0	1	0	0	71	34	49	
ENSXETG00000027105	1314	10	7	10	3	1	13	5	9	7	12	19	29	ENSXETG00000027105
ENSXETG00000027106	504	35	33	26	51	24	73	254	268	162	170	116	117	ngb
ENSXETG00000027107	687	0	0	0	114	77	4	0	0	1	231	169	178	ENSXETG00000027107
ENSXETG00000027108	2028	2	5	0	112	101	9	5	3	1	165	139	130	ENSXETG00000027108
ENSXETG00000027109	2249	1537	2229	1875	1753	1356	2558	649	737	426	1249	924	1028	cyp4f22
ENSXETG00000027112	606	524	718	608	527	420	329	712	792	553	539	381	458	srsf10
ENSXETG00000027114	522	527	701	645	708	548	766	665	745	413	344	205	257	mycbp
ENSXETG00000027116	975	0	0	0	0	0	0	0	0	0	0	0	0	
ENSXETG00000027117	1155	1295	1970	1684	2709	2079	4831	2305	2709	1659	4275	3142	3318	mkrn1
ENSXETG00000027121	1535	61	111	96	212	171	56	186	217	136	150	103	126	adh1
ENSXETG00000027124	1330	292	365	334	198	143	207	279	366	187	271	193	223	prelid2
ENSXETG00000027126	1773	128	157	141	167	120	211	213	222	140	121	73	100	elac1
ENSXETG00000027128	404	4589	6568	5511	4235	3239	4633	3333	3850	2202	4339	2938	3242	uqcrq
ENSXETG00000027130	1260	16	44	29	75	39	28	8	7	2	18	22	13	ENSXETG00000027130
ENSXETG00000027131	381	0	0	0	0	0	0	0	0	0	0	0	0	
ENSXETG00000027136	1176	5	11	6	42	53	61	80	90	78	56	43	44	ENSXETG00000027136
ENSXETG00000027138	3999	2595	3623	3076	4208	3265	4336	8030	9641	5364	3031	2130	2343	hspa4
ENSXETG00000027140	312	0	0	0	0	0	0	0	0	0	0	0	0	hist1h4c
ENSXETG00000027142	1380	372	541	453	402	306	570	495	550	374	355	297	290	ENSXETG00000027142
ENSXETG00000027144	1349	134	218	163	141	119	396	116	123	58	231	156	185	ENSXETG00000027144
ENSXETG00000027145	1836	35	53	51	87	56	111	194	232	137	174	121	116	ENSXETG00000027145
ENSXETG00000027146	381	0	2	0	0	0	0	1	0	0	0	1	1	
ENSXETG00000027148	381	0	0	0	0	0	0	0	0	1	0	0	0	ENSXETG00000027148
ENSXETG00000027149	3042	150	180	151	295	244	434	560	671	410	570	380	479	ENSXETG00000027149
ENSXETG00000027151	1725	28	26	34	53	34	99	123	153	95	88	65	76	ENSXETG00000027151
ENSXETG00000027152	5680	87	125	84	382	294	690	2129	2484	1426	1199	811	966	ENSXETG00000027152
ENSXETG00000027157	2166	1	0	0	6	2	1	11	18	10	7	6	6	ENSXETG00000027157
ENSXETG00000027158	1571	25	34	35	47	33	62	126	137	87	82	60	71	ENSXETG00000027158
ENSXETG00000027160	2322	56	78	48	66	58	136	336	360	248	130	95	98	ENSXETG00000027160
ENSXETG00000027161	2222	137	201	178	185	143	247	239	337	172	256	194	229	znf160
ENSXETG00000027163	1362	78	101	103	85	85	167	131	134	73	398	280	354	znf383
ENSXETG00000027164	312	0	0	0	0	0	1	0	0	1	0	0	0	
ENSXETG00000027165	2916	125	143	103	362	284	893	860	913	558	504	336	343	agbl5
ENSXETG00000027166	387	0	0	0	0	0	0	0	0	0	0	0	0	hist1h4c
ENSXETG00000027167	498	37	84	44	72	62	120	151	176	104	242	190	182	ENSXETG00000027167
ENSXETG00000027168	2948	2	2	1	6	3	8	61	102	56	9	4	19	fam124a
ENSXETG00000027170	1680	1098	1546	1409	1780	1315	1335	2250	2454	1494	1294	922	1048	nars
ENSXETG00000027171	249	3	4	2	15	7	19	18	14	10	15	8	13	ENSXETG00000027171
ENSXETG00000027173	2580	40	43	44	138	87	255	195	279	151	374	211	278	ENSXETG00000027173
ENSXETG00000027175	312	0	0	0	0	0	0	0	0	0	0	0	0	
ENSXETG00000027178	312	0	0	0	0	0	0	0	0	0	1	0	0	hist1h4c
ENSXETG00000027179	1548	0	0	0	3	0	1	0	0	0	0	0	0	ENSXETG00000027179
ENSXETG00000027181	312	0	0	0	0	0	0	0	0	0	0	0	0	
ENSXETG00000027182	414	0	0	0	0	0	0	0	0	0	0	0	0	
ENSXETG00000027185	1152	109	124	117	202	179	257	488	693	424	305	215	212	zdhhc20
ENSXETG00000027187	312	0	0	0	0	0	0	2	0	0	0	0	0	
ENSXETG00000027192	939	0	0	0	0	0	0	1	1	1	0	0	0	
ENSXETG00000027193	1077	0	0	0	1	1	0	2	2	0	0	0	0	wnt9b.2
ENSXETG00000027197	312	0	0	0	0	0	0	0	0	0	0	0	0	
ENSXETG00000027198	408	0	0	0	0	0	0	0	0	0	0	0	0	ENSXETG00000027198
ENSXETG00000027199	405	0	0	0	0	0	0	0	0	0	0	0	0	ENSXETG00000027199
ENSXETG00000027200	312	0	0	0	0	0	0	0	0	0	0	0	0	
ENSXETG00000027202	1382	0	5	2	0	0	28	5	9	5	5	4	2	MGC108450
ENSXETG00000027205	394	0	0	0	0	0	0	0	1	0	0	0	0	
ENSXETG00000027207	881	1215	1719	1372	678	521	637	1110	1376	806	422	312	347	ufm1
ENSXETG00000027208	2195	113	151	142	141	120	232	3737	3994	2496	267	165	192	gbp6
ENSXETG00000027213	1285	1	3	1	0	0	1	2	2	4	0	0	0	
ENSXETG00000027217	441	1022	1519	1254	1192	886	798	1922	2149	1282	605	393	464	nedd8
ENSXETG00000027218	2266	2595	3619	3244	3431	2836	2657	8030	9120	5675	3092	2187	2426	sub1
ENSXETG00000027219	1520	231	305	271	765	582	337	1255	1429	887	1247	956	1014	rab3d
ENSXETG00000027220	1005	1	0	0	0	1	0	0	0	0	0	0	0	ENSXETG00000027220
ENSXETG00000027221	507	28	37	31	80	68	192	92	108	63	266	211	222	ENSXETG00000027221
ENSXETG00000027222	1038	1	1	0	0	0	0	1	1	3	0	0	0	ENSXETG00000027222

ENSXETG00000027223	2111	306	480	386	619	482	1270	1087	1295	795	1408	1007	1038	efs
ENSXETG00000027224	1611	735	1073	892	597	475	848	1129	1452	728	911	594	670	ENSXETG00000027224
ENSXETG00000027225	1798	7882	11310	9649	20046	15575	18429	22072	25060	14686	17349	11728	13223	tagln2
ENSXETG00000027226	946	1666	2586	2223	1608	1228	1191	1578	1764	1099	820	566	649	sec11c
ENSXETG00000027230	3043	86	108	89	139	98	191	274	356	202	134	122	144	znf518b
ENSXETG00000027231	1149	741	1045	893	521	356	693	1070	1119	682	268	192	233	timm10
ENSXETG00000027232	888	688	913	839	1363	988	1513	964	1039	647	1655	1181	1297	ENSXETG00000027232
ENSXETG00000027233	381	0	0	0	0	0	0	0	0	0	0	0	0	
ENSXETG00000027234	312	0	0	0	0	0	0	1	0	0	0	0	0	
ENSXETG00000027236	1881	6908	9851	8679	5477	4248	8686	1732	2083	1242	5421	3871	4368	ENSXETG00000027236
ENSXETG00000027237	876	171	248	222	367	267	518	427	489	315	437	298	343	hexim1
ENSXETG00000027239	1497	0	0	0	0	0	0	0	0	0	0	0	0	
ENSXETG00000027241	743	0	0	0	0	0	0	0	0	0	0	0	0	ENSXETG00000027241
ENSXETG00000027242	6078	174	298	270	697	537	832	785	895	545	990	681	785	dock6
ENSXETG00000027243	2487	166	272	216	578	477	476	1048	1184	744	1309	910	1048	map3k14
ENSXETG00000027245	885	1	1	1	12	2	9	349	499	254	114	62	94	ogn
ENSXETG00000027246	1440	0	0	0	1	0	2	11	26	20	14	5	6	aspn
ENSXETG00000027247	1527	527	778	723	1126	913	1497	1695	1736	1090	1025	746	799	ncstn
ENSXETG00000027249	870	1476	2327	1853	3813	2819	8331	5542	6193	3531	6466	4710	5077	mgc75753
ENSXETG00000027251	590	234	331	288	620	490	1398	4927	5634	3590	1886	1309	1451	fabp4
ENSXETG00000027252	1224	0	0	0	0	0	0	0	0	0	0	0	0	
ENSXETG00000027253	1359	127	198	165	357	310	492	427	448	287	566	359	377	GGT6
ENSXETG00000027254	1077	1134	1588	1392	818	568	893	853	1027	601	765	546	614	mrpl53
ENSXETG00000027255	345	50	74	84	159	129	347	330	387	216	235	142	159	ENSXETG00000027255
ENSXETG00000027257	336	5860	8497	7324	5315	4014	6378	11281	13424	7589	3036	2177	2289	gpx4
ENSXETG00000027259	1858	11861	17424	15362	15820	12217	26050	20336	22518	14274	59436	41404	45842	eif3d
ENSXETG00000027261	1259	625	828	663	518	374	653	977	1242	693	680	470	556	c19orf24
ENSXETG00000027262	1547	5	10	8	19	10	59	502	628	348	144	108	101	pkia
ENSXETG00000027265	1032	110	165	136	191	163	204	434	454	250	294	217	231	atxn7l3
ENSXETG00000027266	1886	3113	4618	3828	5484	4175	5338	10352	12011	7235	7807	5408	5621	snrpb
ENSXETG00000027268	741	0	0	0	0	0	0	3	3	2	0	0	0	nog4
ENSXETG00000027269	2373	0	1	1	2	3	0	0	3	0	1	1	1	
ENSXETG00000027271	1245	9	16	6	6	6	67	16	27	12	152	110	145	XB-GENE-5856185
ENSXETG00000027272	1165	1	0	0	0	1	4	0	0	0	3	2	1	apcs
ENSXETG00000027273	1079	376	554	497	569	417	421	18	23	19	1567	1057	1215	XB-GENE-5761426
ENSXETG00000027274	1903	6716	9650	8161	12609	9668	16356	13938	16336	9320	33321	23351	26186	eif3l
ENSXETG00000027277	1020	0	0	1	0	0	0	0	0	0	0	0	0	
ENSXETG00000027280	1310	6938	9688	8188	7201	5476	13373	11310	14534	8014	10957	7662	8892	itm2a
ENSXETG00000027281	351	0	0	0	0	0	0	0	1	0	0	0	0	
ENSXETG00000027282	2226	5173	7310	6203	3716	2757	5703	8855	10674	6317	4083	2920	3156	flvcr2
ENSXETG00000027283	4215	7	9	4	19	16	47	392	406	291	64	51	58	ENSXETG00000027283
ENSXETG00000027285	1511	220	288	236	65	62	90	160	210	137	44	20	33	tmem177
ENSXETG00000027286	375	965	1494	1286	1164	856	1010	865	940	603	345	262	324	mpdu1
ENSXETG00000027287	387	151	186	173	242	187	328	478	507	318	377	261	277	senp3
ENSXETG00000027288	1417	1263	1695	1373	903	714	1094	1078	1372	771	947	692	814	fcf1
ENSXETG00000027289	1573	4	12	4	23	30	10	57	74	33	41	30	31	prelid3a
ENSXETG00000027291	1782	315	543	438	584	453	1176	452	464	298	550	394	424	abcd4
ENSXETG00000027292	198	81	116	99	136	81	176	190	236	147	154	107	99	lin52
ENSXETG00000027293	756	234	387	346	491	391	563	92	99	61	348	236	253	mogat3
ENSXETG00000027295	498	367	561	514	273	242	438	503	559	326	289	212	240	mrpl17
ENSXETG00000027297	1982	0	0	0	0	0	0	0	1	0	0	0	0	
ENSXETG00000027298	408	183	232	200	225	150	427	433	534	259	340	260	268	med29
ENSXETG00000027302	1455	0	0	0	0	0	1	2	3	2	6	4	5	gabra5
ENSXETG00000027304	1834	469	657	550	527	355	879	1285	1463	886	509	380	404	cldnd1
ENSXETG00000027309	2739	73	124	91	50	27	206	63	78	45	324	220	281	apold1
ENSXETG00000027311	984	103	140	123	536	392	370	1658	1885	1059	384	268	327	hebp1
ENSXETG00000027324	2469	0	0	0	0	0	0	0	0	0	0	0	0	
ENSXETG00000027327	381	0	0	0	0	0	0	0	0	0	0	0	0	
ENSXETG00000027328	2163	91	144	130	117	91	165	240	238	160	185	127	119	fastkd5
ENSXETG00000027329	1260	12	11	13	6	9	35	36	27	25	39	18	30	ENSXETG00000027329
ENSXETG00000027330	2145	200	321	260	291	193	406	437	448	289	299	210	250	ubox5
ENSXETG00000027331	2010	82	110	104	100	62	122	193	239	133	68	69	79	cenpm
ENSXETG00000027332	2727	67	96	101	115	87	259	297	353	197	293	230	239	znf208
ENSXETG00000027334	2401	21	39	32	64	34	73	97	106	57	82	56	61	ENSXETG00000027334
ENSXETG00000027337	2811	1651	2413	1922	1401	1146	2087	2754	3200	1788	1573	1190	1196	cebpg
ENSXETG00000027338	821	2723	4172	3586	3456	2460	4696	3397	3957	2435	4161	2964	3286	smdt1
ENSXETG00000027339	2700	106	135	126	272	206	237	278	343	207	498	347	397	ENSXETG00000027339
ENSXETG00000027340	1164	0	0	0	0	0	0	0	0	0	0	0	0	
ENSXETG00000027344	1043	68884	100072	85759	99084	75025	120867	101860	113845	68271	249124	175501	189554	rpsa
ENSXETG00000027345	1014	0	0	0	1	2	0	0	0	0	0	0	0	
ENSXETG00000027346	606	0	0	0	1	2	1	12	14	9	2	0	1	kcnip4
ENSXETG00000027347	1846	511	732	608	733	536	1054	1072	1251	753	1018	723	794	oser1
ENSXETG00000027348	408	0	0	0	0	0	0	0	0	0	0	0	0	
ENSXETG00000027349	492	18	20	35	33	24	74	104	135	78	70	51	59	ENSXETG00000027349
ENSXETG00000027350	1747	0	0	0	2	1	0	15	14	12	7	2	0	hoxb9
ENSXETG00000027351	714	1	2	1	9	5	7	37	60	46	20	6	16	hoxb7
ENSXETG00000027353	1044	5	13	3	30	17	38	150	169	88	65	42	54	hoxb2
ENSXETG00000027354	1400	1531	2296	1843	1306	1011	1317	2767	3471	2027	1029	706	846	snrpd1
ENSXETG00000027356	751	7280	10404	8631	5055	3921	5610	5278	6356	3394	4934	3445	3623	sec61b
ENSXETG00000027357	1441	17	34	31	25	18	37	131	161	115	96	57	64	skap1
ENSXETG00000027359	1576	810	1221	1094	1529	1132	1663	1635	1795	1060	1396	1071	1066	cdk5rap3
ENSXETG00000027360	1509	197	286	268	602	461	561	689	810	482	554	389	473	ENSXETG00000027360
ENSXETG00000027362	1038	3	7	9	22	22	27	20	19	18	14	10	9	wisp2
ENSXETG00000027363	270	169	283	271	201	130	267	293	380	246	140	107	115	churc1
ENSXETG00000027365	454	469	597	508	473	369	480	755	919	572	478	334	386	ube2v1
ENSXETG00000027366	1913	8	9	9	50	32	58	993	1135	714	64	52	60	snai1
ENSXETG00000027370	2810	2067	2909	2301	4163	3241	6559	4711	5787	3384	2861	1962	2154	riok3
ENSXETG00000027373	1542	3	5	3	14	12	1	15	17	9	3	2	0	ttll2
ENSXETG00000027374	1271	704	1035	879	758	568	1359	1513	1762	1072	685	544	589	ap3s1
ENSXETG00000027375	2162	7	15	6	34	33	22	5	4	0	4	0	3	gcm1
ENSXETG00000027376	1285	292	447	349	221	143	481	410	558	261	290	202	252	rnf170
ENSXETG00000027377	1418	218	331	272	341	215	476	483	580	322	455	334	364	arl9
ENSXETG00000027380	863	750	1169	944	217	151	282	594	723	417	149	108	128	bet1
ENSXETG00000027387	930	0	0	0	0	0	0	0	0	0	0	0	0	
ENSXETG00000027390	1236	0	0	1	134	84	8	0	0	1	16	6	5	znf474
ENSXETG00000027391	630	52	69	64	84	65	102	144	151	87	143	137	125	fam133b
ENSXETG00000027392	3333	5	16	9	52	25	54	308	310	173	135	85	92	sncaip
ENSXETG00000027393	1464	30	43	27	47	39	217	231	272	156	71	53	47	snx24
ENSXETG00000027398	1137	42	81	51	81	58	134	149	190	110	125	84	103	mterf1
ENSXETG00000027400	3373	7	8	11	5	1	28	79	84	46	16	11	8	nfe2
ENSXETG00000027401	4345	498	720	632	687	553	1495	821	938	562	1151	788	848	sowahb
ENSXETG00000027403	1281	4299	6114	5070	3420	2600	4334	4099	4870	2683	4431	2994	3439	copz1
ENSXETG00000027405	2071	667	996	854	1068	791	1106	1040	1210	745	1273	910	1016	dus3l
ENSXETG00000027406	1532	844	1193	1001	1435	1082	2383	2830	2996	1727	3079	2100	2373	ccni
ENSXETG00000027407	3536	12	17	5	106	69	452	394	554	288	368	256	301	steap4
ENSXETG00000027410	2628	1473	2121	1762	1600	1287	1378	2570	2928	1655	925	611	696	prrc1
ENSXETG00000027411	855	137	219	182	31	19	77	143	186	114	99	56	78	sla2
ENSXETG00000027414	3158	160	221	198	768	532	200	177	135	108	280	228	252	epn3
ENSXETG00000027416	2666	5194	7353	6552	6107	4892	12376	7452	8579	5052	7259	5340	5735	xbp1
ENSXETG00000027418	1422	349	485	407	249	190	620	826	1051	580	338	236	265	slc48a1
ENSXETG00000027419	831	0	0	0	0	1	0	0	0	0	0	1	0	a4galt

ENSXETG00000027421	309	0	0	0	0	0	0	0	0	0	0	0	0	
ENSXETG00000027422	454	343	536	458	384	312	332	596	647	443	225	157	169	mrpl27
ENSXETG00000027428	1435	1433	2021	1645	1632	1199	2009	1648	1890	1091	1358	996	1114	iscu
ENSXETG00000027429	1820	522	742	643	532	429	401	347	400	306	174	136	125	fdxr
ENSXETG00000027430	1380	12	13	7	9	0	26	226	292	155	52	46	38	ENSXETG00000027430
ENSXETG00000027431	1362	18	31	20	55	49	105	45	57	31	76	44	52	ENSXETG00000027431
ENSXETG00000027433	1521	949	1443	1216	756	578	1704	684	805	459	459	357	433	otop2
ENSXETG00000027434	1134	4	2	1	0	1	0	1	9	6	0	0	2	ENSXETG00000027434
ENSXETG00000027435	651	0	0	0	0	0	0	0	0	0	0	0	0	
ENSXETG00000027436	945	2	3	1	0	0	2	3	2	2	9	14	12	ENSXETG00000027436
ENSXETG00000027437	1581	2	1	2	2	3	11	30	32	26	14	7	8	ENSXETG00000027437
ENSXETG00000027438	921	0	0	0	0	0	0	0	0	0	0	0	0	
ENSXETG00000027440	945	0	0	0	0	0	0	1	0	0	0	0	0	
ENSXETG00000027441	939	0	1	0	0	0	0	0	0	0	0	0	0	
ENSXETG00000027454	1644	57	102	80	461	368	747	384	481	280	703	498	563	irs2
ENSXETG00000027455	1357	1	2	0	4	3	19	3934	4844	2672	279	215	237	ctsh
ENSXETG00000027459	1273	298	434	349	468	342	1325	814	964	536	708	482	551	tmem243
ENSXETG00000027460	492	294	529	404	310	248	109	733	805	453	107	66	79	pclaf
ENSXETG00000027463	909	2	6	2	5	3	6	125	149	103	1	7	5	ENSXETG00000027463
ENSXETG00000027465	483	82	107	99	31	16	5	76	68	53	16	8	4	hsp30d
ENSXETG00000027466	1470	7	11	15	9	10	46	83	99	54	43	35	40	ccdc3
ENSXETG00000027467	2675	130	161	151	205	168	261	294	406	191	189	137	129	kbtbd4
ENSXETG00000027469	2601	12	9	8	10	11	17	28	27	23	22	14	12	gtf3c3
ENSXETG00000027471	789	0	0	0	0	0	0	0	0	0	0	0	0	
ENSXETG00000027472	3252	441	613	518	791	644	1315	1953	2267	1349	910	591	740	abhd17b
ENSXETG00000027473	1380	344	510	405	216	159	516	334	350	190	283	229	260	tmem218
ENSXETG00000027476	1284	377	582	537	1321	1030	3540	1583	1816	1120	1212	830	896	leng9
ENSXETG00000027477	321	28	46	41	21	13	14	2	6	2	0	0	0	ENSXETG00000027477
ENSXETG00000027478	2404	455	574	491	1330	994	1209	10694	13060	7126	2230	1595	1760	anxa1
ENSXETG00000027479	1522	0	0	1	1	0	5	14	38	8	2	0	0	anxa8
ENSXETG00000027480	1132	1514	2156	1897	1752	1362	2091	586	650	389	1882	1346	1592	XB-GENE-968486
ENSXETG00000027482	633	0	0	0	0	0	0	0	0	0	0	0	0	
ENSXETG00000027483	488	412	555	492	415	352	375	1305	1457	884	214	153	175	eny2
ENSXETG00000027484	1251	15	38	31	77	49	90	153	138	75	113	83	97	znf432
ENSXETG00000027485	828	3	0	0	2	3	4	36	41	25	12	5	4	tmem74
ENSXETG00000027487	1697	2781	3952	3286	3277	2475	3258	5066	6100	3450	3837	2717	2997	sumo3
ENSXETG00000027491	2112	853	1291	1217	1041	836	1711	1515	1918	1179	1214	869	1017	dbr1
ENSXETG00000027492	452	4513	6632	5616	6139	4478	10869	3149	3611	1984	10174	7521	7937	dapl1
ENSXETG00000027496	411	2089	3152	2809	2280	1773	2710	1918	2095	1380	1698	1242	1343	ndufb3
ENSXETG00000027498	690	10646	15916	13249	21413	16306	27098	20883	23542	12928	10461	7037	7935	UBB
ENSXETG00000027499	2493	140	183	173	682	493	607	958	1141	676	160	120	147	nusap1
ENSXETG00000027500	1347	16	16	20	140	132	137	358	439	242	162	99	145	tmem237
ENSXETG00000027501	741	0	0	0	0	0	0	0	0	0	0	0	0	
ENSXETG00000027505	660	35	44	35	66	64	88	70	83	44	51	33	48	rflna
ENSXETG00000027506	1395	48	59	67	115	87	133	312	343	203	180	144	141	ENSXETG00000027506
ENSXETG00000027508	632	0	0	0	0	0	0	0	0	0	1	0	0	
ENSXETG00000027510	2588	51	78	69	462	337	592	617	681	379	979	677	819	itgb6
ENSXETG00000027514	312	0	0	0	0	0	0	0	0	0	0	0	0	
ENSXETG00000027519	855	0	4	0	1	1	0	6	7	2	3	1	1	ENSXETG00000027519
ENSXETG00000027521	150	182	279	242	237	162	194	282	317	161	164	100	113	tomm7
ENSXETG00000027522	1119	1	1	2	1	2	8	21	14	9	9	11	9	ENSXETG00000027522
ENSXETG00000027524	770	708	963	827	857	624	844	1208	1337	855	1219	872	951	surf2
ENSXETG00000027525	1385	101	173	116	157	120	196	441	502	317	210	142	156	sirt6
ENSXETG00000027526	351	415	648	566	521	385	819	343	401	254	659	444	478	surf1
ENSXETG00000027527	1044	628	857	723	478	334	555	556	699	384	386	283	337	malsu1
ENSXETG00000027531	720	7775	11599	9913	4203	3197	2482	5515	6645	3783	1006	659	830	cycs
ENSXETG00000027532	1164	125	184	150	95	92	214	264	338	187	276	193	229	ENSXETG00000027532
ENSXETG00000027534	744	12	10	10	59	54	77	192	178	115	152	111	133	hoxa4
ENSXETG00000027536	3127	298	413	331	689	522	893	872	1002	642	1226	853	1001	znf574
ENSXETG00000027540	486	5362	7817	6900	4105	3047	3592	3705	4147	2679	2454	1709	1855	tmem258
ENSXETG00000027541	1430	115	170	159	506	412	603	698	688	441	711	538	540	rfx5
ENSXETG00000027542	1562	96	168	125	142	96	305	150	200	102	180	111	140	faap24
ENSXETG00000027543	348	0	0	0	0	0	0	2	1	1	0	0	0	
ENSXETG00000027544	541	0	0	0	0	0	0	0	1	0	0	0	0	cox6a2
ENSXETG00000027545	877	5	6	5	10	7	11	295	313	167	1	0	1	cldn6.1
ENSXETG00000027546	792	3	3	6	18	7	8	45	34	28	6	5	3	deptor
ENSXETG00000027547	645	23	21	19	1	2	2	120	124	76	4	2	6	cldn6.2
ENSXETG00000027548	3758	1	1	0	5	2	4	19	18	11	10	4	7	chst8
ENSXETG00000027549	1126	140	221	188	268	204	305	484	604	307	395	290	330	ss18l2
ENSXETG00000027551	2222	6021	8518	7106	7394	5751	8759	1282	1650	818	3454	2460	2870	ENSXETG00000027551
ENSXETG00000027556	1494	28	37	31	61	46	138	1451	1716	987	20	12	13	cyp2g1p
ENSXETG00000027557	2835	36	45	36	31	15	36	159	189	128	100	107	84	mttp.2
ENSXETG00000027558	1470	2602	3712	3135	5268	4062	16035	1036	1232	727	5038	3516	3994	XB-GENE-5903934
ENSXETG00000027560	950	0	4	3	1	0	2	3	2	1	0	0	1	ENSXETG00000027560
ENSXETG00000027561	2252	6978	10022	8469	10700	8083	22834	6513	7781	4304	9296	6351	7537	ENSXETG00000027561
ENSXETG00000027563	1479	232	340	274	1855	1360	2979	97	122	71	1922	1329	1451	ENSXETG00000027563
ENSXETG00000027564	2378	0	0	0	0	1	0	0	0	0	1	0	2	
ENSXETG00000027566	2653	0	0	0	9	8	5	19	30	8	28	11	20	cyp2a6
ENSXETG00000027567	2170	1	7	6	28	21	4	7	8	7	3	7	4	scnn1b
ENSXETG00000027571	969	0	0	0	7	2	0	1	0	0	2	3	3	ENSXETG00000027571
ENSXETG00000027573	1323	1	1	2	0	1	0	5	8	3	10	8	4	pdilt
ENSXETG00000027577	1020	0	0	0	0	0	0	0	0	0	0	0	0	
ENSXETG00000027581	1290	11160	15857	13599	24269	18360	160019	38681	45372	25770	13451	9333	10418	ctsv
ENSXETG00000027582	1026	0	1	0	1	0	0	0	0	0	0	0	0	
ENSXETG00000027585	2053	160	207	180	194	152	390	490	569	326	284	200	247	dxo
ENSXETG00000027586	1729	67	137	89	121	81	103	93	121	63	57	48	41	polr3e
ENSXETG00000027588	1533	8966	12480	10104	10341	7755	16380	12020	15287	8345	6280	4340	5057	serpinb6
ENSXETG00000027589	1673	5172	7333	6093	4701	3467	4695	6157	6937	3980	4047	2826	3162	kdelr1
ENSXETG00000027590	1316	0	1	0	9	4	2	16	18	10	37	26	34	serpinb5
ENSXETG00000027592	601	12407	18268	15283	5758	4263	6716	4354	4819	2993	5922	4134	4575	cox4i2
ENSXETG00000027593	2644	10786	15318	13045	12554	9529	16771	22228	25867	15037	10030	6986	7656	rac1
ENSXETG00000027594	1863	387	484	452	999	780	1707	1543	1650	1048	1293	889	995	msl1
ENSXETG00000027596	1500	1447	2204	1774	1224	962	1260	2121	2506	1473	1067	771	734	aimp2
ENSXETG00000027597	714	442	617	490	354	228	371	376	438	270	387	278	346	mrps18c
ENSXETG00000027599	1284	198	258	246	616	502	710	1198	1261	758	911	677	660	wipf2
ENSXETG00000027601	1488	9	17	17	45	46	103	974	1095	709	132	96	132	mex3b
ENSXETG00000027604	798	0	0	1	0	1	0	0	0	0	0	0	0	
ENSXETG00000027605	1593	5827	8556	7200	8680	6676	13929	222	273	159	5152	3724	4105	cyp4v2
ENSXETG00000027606	779	5	3	4	16	7	3	10	11	5	1	0	0	pvalb
ENSXETG00000027607	1326	29	33	35	282	173	709	678	817	436	720	501	575	vopp1
ENSXETG00000027610	1270	4340	6169	5262	3197	2385	9600	1026	1259	762	3441	2476	2807	naaa
ENSXETG00000027611	747	1	4	5	4	6	1	5	6	9	3	2	2	wnt7a
ENSXETG00000027612	1534	37683	56052	48699	22569	16962	21071	11627	13347	8082	24196	17096	18577	ckmt1b
ENSXETG00000027613	1677	236	335	304	785	680	1442	558	630	373	1460	1037	1094	ell3
ENSXETG00000027617	5926	249	370	317	527	379	866	1159	1329	812	726	517	552	ralgapa2
ENSXETG00000027618	1086	0	0	0	0	0	0	0	0	0	0	0	0	
ENSXETG00000027621	1173	1	1	0	0	0	0	5	5	5	2	7	3	ENSXETG00000027621
ENSXETG00000027622	1774	11887	17592	14990	18031	13402	26392	14191	16813	9836	15330	10976	12147	idh2
ENSXETG00000027623	1112	494	812	681	888	687	943	1265	1471	852	777	573	612	arpin
ENSXETG00000027626	1452	2	0	1	20	12	35	179	177	85	15	8	12	ENSXETG00000027626
ENSXETG00000027627	2429	721	1003	873	1026	756	1741	1286	1461	872	972	757	770	abhd17c

ENSXETG00000027628	1167	0	0	0	0	0	0	0	0	0	0	0	0	
ENSXETG00000027630	1287	0	0	0	1	0	0	2	2	0	0	2	0	ENSXETG00000027630
ENSXETG00000027631	685	324	418	351	268	209	415	508	688	355	309	226	243	lsm10
ENSXETG00000027636	1801	5	5	13	10	9	17	122	125	81	9	12	12	c1r
ENSXETG00000027637	630	0	0	0	1	2	4	0	0	1	1	1	0	olig4
ENSXETG00000027638	1607	225	339	266	697	613	906	6829	7648	4473	871	595	640	marcksl1
ENSXETG00000027639	2462	356	597	474	785	583	1020	1149	1260	763	1296	881	1012	foxj2
ENSXETG00000027641	2018	190765	279966	240653	239693	180584	471258	508030	583529	348896	879009	599084	676062	actg1
ENSXETG00000027646	2824	596	821	658	471	381	728	836	1007	556	624	453	535	XB-GENE-974141
ENSXETG00000027648	493	1	5	1	5	1	3	20	30	16	12	14	11	acbd7
ENSXETG00000027651	573	24	26	24	52	44	44	538	588	380	49	55	34	ccdc28b
ENSXETG00000027655	2048	418	561	461	387	337	284	1281	1433	930	296	203	247	slc25a40
ENSXETG00000027656	2141	369	646	492	640	452	998	411	474	255	511	357	377	pex7
ENSXETG00000027658	637	921	1329	1092	718	530	832	991	1159	660	666	373	464	mrps36
ENSXETG00000027666	2807	247	370	356	600	455	1096	972	1053	610	455	314	339	tmeff1
ENSXETG00000027667	1206	531	756	574	306	297	542	413	469	282	218	175	211	fam206a
ENSXETG00000027669	1467	1	0	1	10	8	9	61	70	54	45	23	36	steap2
ENSXETG00000027671	1440	125	200	177	120	95	124	214	239	129	61	53	63	trdmt1
ENSXETG00000027672	2292	0	0	0	2	1	2	0	0	0	1	1	5	mixer
ENSXETG00000027673	2105	67	90	63	161	104	126	38	44	22	52	29	36	ENSXETG00000027673
ENSXETG00000027674	2439	0	0	0	0	0	0	0	0	0	0	0	0	
ENSXETG00000027675	2343	0	0	0	0	0	0	0	0	0	0	0	0	
ENSXETG00000027677	1859	122	163	144	303	247	228	1120	1334	855	724	484	556	aldoc
ENSXETG00000027678	2872	29	41	39	136	114	160	160	206	122	231	153	190	gnaz
ENSXETG00000027679	963	3	9	3	1	0	2	2	1	2	5	1	0	ENSXETG00000027679
ENSXETG00000027681	988	847	1244	1058	697	545	777	846	965	595	571	442	438	timm8b
ENSXETG00000027683	1126	3872	5445	4371	3598	2742	4498	2666	3308	1748	2716	1927	2106	sdhd
ENSXETG00000027684	1272	843	1229	1085	1309	1043	1345	1408	1641	987	1092	841	891	ap1m2
ENSXETG00000027686	1481	172	313	272	333	246	813	830	973	591	223	152	166	serpinf1
ENSXETG00000027687	1061	156	243	207	217	138	137	572	664	385	79	57	49	spc24
ENSXETG00000027688	480	1230	1853	1658	988	703	766	1092	1271	738	604	446	488	ndufa5
ENSXETG00000027689	960	1	3	1	7	3	2	1	1	1	1	0	0	tmem229a
ENSXETG00000027690	1421	105	183	157	715	549	1561	2135	2432	1532	3221	2277	2486	icam5
ENSXETG00000027691	381	12	10	19	13	9	27	26	29	10	18	12	10	ENSXETG00000027691
ENSXETG00000027692	2790	142	213	175	183	125	531	2086	2324	1380	433	291	330	fermt3
ENSXETG00000027697	796	617	817	671	1018	816	2123	1537	1962	1172	1082	776	1027	cmtm6
ENSXETG00000027698	1188	0	1	0	2	1	0	8	5	2	13	3	3	sctr
ENSXETG00000027700	168	59	74	65	94	62	131	146	155	83	96	61	96	snapin
ENSXETG00000027701	522	16	38	20	84	62	151	329	341	236	301	188	208	smad4.2
ENSXETG00000027703	348	0	0	0	0	0	0	15	24	15	3	3	3	
ENSXETG00000027712	813	1	3	9	13	9	9	26	14	8	6	5	9	ENSXETG00000027712
ENSXETG00000027713	2805	79	121	87	143	133	194	486	511	325	709	475	602	p2ry1
ENSXETG00000027716	426	579	906	766	601	461	553	944	1147	716	761	610	553	cox17
ENSXETG00000027717	1647	450	661	597	329	229	404	502	563	315	261	201	221	qrsl1
ENSXETG00000027719	2860	0	2	0	0	0	2	0	5	0	0	1	0	tfap2e
ENSXETG00000027720	2727	0	0	0	0	0	0	0	0	0	0	0	0	
ENSXETG00000027721	1218	0	0	0	0	0	0	0	0	0	0	0	1	
ENSXETG00000027722	1814	2223	3187	2533	1845	1481	2524	3716	4310	2631	2175	1556	1641	znf706.2
ENSXETG00000027723	793	5531	7794	6384	3074	2329	2182	3318	3996	2235	1394	966	1096	ssr3
ENSXETG00000027727	1769	1531	2177	1936	1592	1120	1971	1530	1765	1105	2296	1671	1913	nmd3
ENSXETG00000027728	1295	1044	1528	1346	1102	872	2123	1449	1595	954	1720	1232	1391	rnf5
ENSXETG00000027734	387	24895	35953	29537	31776	24482	31682	31236	35341	20227	66151	45691	50460	rps29
ENSXETG00000027735	1269	6	15	8	5	7	16	27	50	24	29	22	18	znf41
ENSXETG00000027737	2240	3	0	1	4	1	14	18	18	17	29	19	12	klhdc1
ENSXETG00000027739	3210	588	852	778	1003	824	1265	1400	1581	892	1048	717	806	nemf
ENSXETG00000027740	1982	148	232	238	425	331	537	1388	1502	910	750	514	594	sav1
ENSXETG00000027741	3726	223	269	267	573	447	1276	1312	1602	927	875	592	713	ppt2
ENSXETG00000027742	1034	43	65	44	102	59	94	103	111	69	49	32	38	rhno1
ENSXETG00000027743	1730	168	269	235	297	228	313	275	356	186	153	99	117	XB-GENE-5727065
ENSXETG00000027748	1641	0	0	0	0	0	0	1	0	0	0	0	0	
ENSXETG00000027750	2331	0	0	0	0	0	0	0	0	0	0	0	0	
ENSXETG00000027751	2259	248	327	306	1873	1516	1271	5092	5863	3394	5036	3533	3796	marcks
ENSXETG00000027753	1717	2842	4182	3505	2600	1925	3149	2288	2703	1507	2090	1596	1679	traf3ip3
ENSXETG00000027754	10059	314	455	354	838	656	1794	1781	2193	1182	1946	1306	1422	rev3l
ENSXETG00000027759	2626	7	3	4	0	2	4	13	18	11	12	15	15	acyp2
ENSXETG00000027760	1131	0	1	1	1	2	0	2	0	0	1	3	4	sptbn1
ENSXETG00000027762	1027	0	0	0	0	0	0	0	0	0	0	0	0	
ENSXETG00000027764	939	0	0	0	0	0	0	0	0	0	0	0	0	
ENSXETG00000027765	882	1	0	2	0	1	0	2	2	0	1	0	1	C2CD4D
ENSXETG00000027769	939	0	0	0	0	0	0	0	0	0	0	0	0	
ENSXETG00000027770	933	0	0	0	0	0	0	0	0	0	0	0	0	
ENSXETG00000027771	823	0	0	0	1	2	2	6	9	3	2	1	2	hmx2
ENSXETG00000027772	195	4	8	5	34	19	74	114	136	99	47	37	41	hmga2
ENSXETG00000027778	3824	662	922	820	1779	1365	2930	2287	2475	1535	2664	1906	2133	rassf3
ENSXETG00000027781	1128	0	0	0	0	1	0	1	2	0	1	0	1	
ENSXETG00000027782	739	969	1391	1142	1763	1442	2657	2582	2998	1618	3367	2358	2618	perp
ENSXETG00000027787	678	258	376	302	871	617	1270	689	689	483	1324	897	973	gps2
ENSXETG00000027792	528	0	0	0	0	0	0	0	0	0	0	0	0	
ENSXETG00000027796	465	0	0	0	0	0	0	0	0	0	0	0	0	
ENSXETG00000027798	786	0	0	0	0	0	1	0	0	0	0	0	0	
ENSXETG00000027799	2050	451	676	547	717	570	966	1353	1475	950	1005	713	774	spop
ENSXETG00000027802	1218	5053	7244	6089	4936	3839	3559	4300	4866	2940	3556	2468	2656	phb
ENSXETG00000027804	1231	604	899	713	803	583	929	994	1127	662	993	622	665	zcchc9
ENSXETG00000027806	2514	1180	1723	1459	2355	1783	2937	747	812	493	2179	1554	1735	acot12
ENSXETG00000027807	1500	92	113	87	161	125	281	309	350	215	205	151	156	cited2
ENSXETG00000027809	1744	186	231	207	368	254	441	728	853	457	665	439	586	ssbp2
ENSXETG00000027811	485	56640	84463	72162	69744	52734	71172	64910	73671	44421	136801	95032	102628	rps23
ENSXETG00000027813	1071	684	927	757	652	460	757	764	1069	564	502	325	403	tmem167a
ENSXETG00000027814	1389	161	238	209	120	80	180	246	319	155	97	86	87	xrcc4
ENSXETG00000027815	11256	7	11	16	34	39	67	1652	2007	1067	229	167	172	vcan
ENSXETG00000027817	1269	16	22	21	58	40	56	175	186	87	84	56	63	ENSXETG00000027817
ENSXETG00000027823	936	0	0	0	0	0	0	0	0	0	0	0	0	
ENSXETG00000027824	921	0	0	0	0	0	0	0	0	0	0	0	0	
ENSXETG00000027825	939	0	0	0	0	0	0	0	0	0	0	0	0	
ENSXETG00000027828	1170	174	222	205	280	210	431	181	178	100	371	264	298	pygo1
ENSXETG00000027829	1338	0	0	0	0	0	0	1	2	1	1	0	2	
ENSXETG00000027832	1781	8861	12470	10451	9421	7031	9988	14714	17624	9978	9234	6448	7003	ran
ENSXETG00000027833	1030	0	0	0	0	0	0	0	0	0	0	0	0	
ENSXETG00000027834	2401	1	0	0	1	0	4	3	1	2	0	0	1	MAEL
ENSXETG00000027836	867	37	64	54	212	152	171	72	68	48	42	25	41	ENSXETG00000027836
ENSXETG00000027840	405	29933	44738	40021	37760	28515	39408	34480	39907	25848	86815	61150	66105	rpl36
ENSXETG00000027841	1560	39	65	50	121	83	116	343	369	205	192	123	145	ENSXETG00000027841
ENSXETG00000027843	411	0	0	0	0	0	0	0	0	0	0	0	0	hist2h3c
ENSXETG00000027849	426	0	0	0	0	0	0	0	0	0	0	0	0	
ENSXETG00000027852	3750	0	0	0	0	0	0	0	0	0	0	0	0	
ENSXETG00000027853	411	0	0	0	0	0	0	0	0	0	0	0	0	ENSXETG00000027853
ENSXETG00000027857	768	142	226	183	234	176	308	568	647	451	251	183	205	ENSXETG00000027857
ENSXETG00000027865	1908	1900	2814	2454	1201	926	837	2098	2281	1397	726	502	596	yars
ENSXETG00000027866	1663	38	39	39	84	46	102	123	135	64	78	62	59	zbtb8a.2
ENSXETG00000027867	1251	3594	4929	4250	3359	2510	2200	3092	3448	2077	1879	1346	1362	ak2
ENSXETG00000027870	2046	55	87	54	154	112	352	373	479	261	176	111	110	tbck

ENSXETG00000027871	435	17761	24970	20910	21395	16323	28884	25899	29629	16901	53303	36666	41342	rpl35a
ENSXETG00000027874	477	0	0	0	0	0	0	0	0	0	0	0	0	
ENSXETG00000027875	1626	0	0	0	0	0	0	0	0	0	0	0	0	
ENSXETG00000027880	2011	5398	7864	6490	6663	5051	18276	6950	8127	4601	10209	7224	7878	dusp1
ENSXETG00000027881	942	0	0	0	0	0	0	0	0	0	0	0	0	
ENSXETG00000027882	933	0	0	0	0	0	0	0	0	0	0	0	0	
ENSXETG00000027883	682	1180	1542	1307	892	634	1343	2115	2437	1419	963	686	768	brk1
ENSXETG00000027884	1838	0	1	1	0	0	0	3	4	4	1	0	0	chrnd
ENSXETG00000027885	1391	310	449	382	608	453	695	1248	1409	911	1163	780	904	XB-GENE-973579
ENSXETG00000027887	4082	45	92	68	249	194	274	484	517	300	244	164	176	trib2
ENSXETG00000027888	1095	0	0	0	0	0	0	0	0	0	0	0	0	
ENSXETG00000027891	768	0	1	0	0	0	0	18	36	20	7	2	4	ENSXETG00000027891
ENSXETG00000027892	1605	0	0	0	0	0	1	125	157	84	29	10	14	ENSXETG00000027892
ENSXETG00000027894	570	3	0	1	2	0	1	1	5	6	0	1	0	ENSXETG00000027894
ENSXETG00000027901	1996	220	309	257	391	258	468	858	984	580	652	459	498	stxbp4
ENSXETG00000027904	1331	1575	2124	1795	1732	1332	2016	1623	2038	1137	776	560	641	rer1
ENSXETG00000027906	519	3	10	17	255	173	222	540	646	367	706	482	564	vip
ENSXETG00000027913	321	0	0	0	0	0	0	0	0	0	0	0	0	
ENSXETG00000027914	351	0	0	0	0	0	0	33	21	20	0	0	1	
ENSXETG00000027916	1494	22	44	39	41	23	56	47	56	22	26	15	16	asb5
ENSXETG00000027917	560	842	1213	1004	726	561	843	1361	1610	826	617	443	513	spcs3
ENSXETG00000027919	327	677	1071	899	1203	937	1267	552	655	418	1156	802	877	dera
ENSXETG00000027920	828	5756	8374	7419	9900	7866	12489	15169	16881	10726	13939	9779	10361	arpc2
ENSXETG00000027921	1117	0	0	0	0	0	0	0	0	0	0	0	0	
ENSXETG00000027925	2382	145	202	193	479	400	155	938	1128	618	104	73	66	donson
ENSXETG00000027926	1711	0	0	0	0	2	0	0	2	0	0	0	0	etv2
ENSXETG00000027927	1800	2447	3425	3061	3707	2908	3879	8764	10253	6212	3088	2182	2503	hist2h3d
ENSXETG00000027928	996	0	0	1	0	0	0	0	0	0	0	0	0	
ENSXETG00000027931	3091	153	240	223	527	424	583	877	1006	553	960	608	749	ppp1r10
ENSXETG00000027932	1146	942	1336	1118	446	327	954	684	863	511	521	377	394	c6orf136
ENSXETG00000027934	1609	140	216	186	692	516	1016	892	984	564	904	603	648	crebl2
ENSXETG00000027935	776	2685	3937	3158	3418	2599	4366	6011	7098	3799	3245	2147	2455	csnk2b
ENSXETG00000027937	967	0	0	0	0	0	0	0	0	0	0	0	0	
ENSXETG00000027940	342	0	0	0	0	0	0	18	18	11	1	0	1	
ENSXETG00000027943	1458	0	0	0	0	0	0	0	0	0	0	0	0	
ENSXETG00000027944	651	4	12	4	3	5	5	4	4	3	0	0	0	ENSXETG00000027944
ENSXETG00000027945	1209	0	0	0	31	22	9	0	2	0	5	6	11	slc30a8
ENSXETG00000027949	1395	0	0	0	0	0	0	0	0	0	0	0	0	
ENSXETG00000027953	1545	17	21	22	109	66	68	57	76	54	66	71	67	ENSXETG00000027953
ENSXETG00000027954	1155	4	9	15	19	14	52	252	283	182	49	21	30	adap2
ENSXETG00000027956	318	964	1325	1111	1326	1062	1447	2191	2256	1307	1747	1213	1318	rbm8a
ENSXETG00000027958	381	0	0	0	0	0	0	0	0	1	0	0	0	
ENSXETG00000027959	1212	0	0	1	0	0	0	0	0	0	0	0	0	
ENSXETG00000027960	1176	0	0	0	0	0	0	0	0	0	0	0	0	
ENSXETG00000027962	741	0	0	0	0	0	0	0	0	0	0	0	0	ENSXETG00000027962
ENSXETG00000027965	381	0	0	0	0	0	0	0	0	0	0	0	0	
ENSXETG00000027969	1901	0	2	0	0	0	0	0	0	1	0	0	0	
ENSXETG00000027971	285	1032	1562	1306	1099	802	1134	1265	1452	885	930	628	685	higd2a
ENSXETG00000027973	1563	0	0	0	1	0	0	0	0	0	0	0	0	ENSXETG00000027973
ENSXETG00000027977	219	36	46	53	152	121	108	508	590	302	266	190	214	ENSXETG00000027977
ENSXETG00000027979	1119	0	0	1	5	3	1	35	35	19	36	10	29	npy2r
ENSXETG00000027982	1167	0	0	0	0	0	0	0	0	0	0	0	0	
ENSXETG00000027983	3489	1	2	0	7	2	1	28	35	18	10	4	7	myt1l
ENSXETG00000027985	261	123	169	122	72	64	72	145	167	106	74	49	70	tmem18
ENSXETG00000027987	714	1377	2020	1720	1354	1077	1758	1853	2289	1352	612	421	500	acp1
ENSXETG00000027988	1413	64	71	65	69	67	48	107	100	56	41	28	37	depdc4
ENSXETG00000027990	2838	2736	3806	3205	1054	831	2945	5441	6442	3573	1471	1067	1102	dram1
ENSXETG00000027991	1911	0	0	0	1	1	0	6	14	7	1	0	2	sacs
ENSXETG00000027993	1682	287	417	363	861	641	1119	1316	1471	949	986	754	842	bcl2l14
ENSXETG00000027994	2612	2708	4024	3394	2156	1761	5035	5872	6886	3853	5543	3811	4333	cers3
ENSXETG00000027995	2465	840	1256	1088	1118	881	1748	1900	2371	1349	1292	940	1012	CFDP1
ENSXETG00000027997	564	2053	3101	2652	2146	1659	3129	1913	2145	1407	2051	1434	1540	selenos
ENSXETG00000027998	990	4291	6472	5425	9286	7023	14263	8432	9286	6013	8318	5875	6414	prss8
ENSXETG00000028002	1170	171	264	254	293	205	324	1102	1173	812	425	338	359	bckdk
ENSXETG00000028008	720	29	44	37	45	47	66	392	428	277	117	72	74	XB-GENE-5720006
ENSXETG00000028009	2113	23	31	29	215	161	112	89	85	78	46	33	46	spa17
ENSXETG00000028014	1329	0	0	0	0	0	0	0	1	1	1	0	0	ENSXETG00000028014
ENSXETG00000028015	291	0	0	0	0	0	0	2	3	2	2	0	0	
ENSXETG00000028016	1634	25	57	36	71	56	125	114	166	86	324	228	224	znf333
ENSXETG00000028017	1146	0	1	0	0	0	4	9	11	9	5	2	1	ENSXETG00000028017
ENSXETG00000028019	1234	647	1033	832	994	772	698	1096	1206	713	682	499	508	TUFM
ENSXETG00000028021	1776	109	155	126	296	210	344	463	523	360	1196	898	1044	slc6a12
ENSXETG00000028023	960	7	8	5	25	22	77	156	180	102	95	61	63	ptger1
ENSXETG00000028025	870	234	353	275	250	166	156	564	630	351	117	96	128	srm
ENSXETG00000028029	1890	642	845	769	1240	915	794	1809	1937	1207	1191	880	911	asf1b
ENSXETG00000028030	387	1	0	0	2	2	0	1	1	0	0	0	1	asgr1
ENSXETG00000028031	924	0	0	0	0	0	0	0	0	0	0	0	0	
ENSXETG00000028032	480	0	0	0	0	0	1	2	4	2	0	1	0	ENSXETG00000028032
ENSXETG00000028033	441	19	15	18	36	25	121	13	8	9	54	30	32	ENSXETG00000028033
ENSXETG00000028035	1657	169	333	245	421	324	656	478	473	312	1199	804	892	akap8l
ENSXETG00000028037	1628	103	143	133	261	192	334	657	720	428	465	336	341	ENSXETG00000028037
ENSXETG00000028038	518	634	1000	905	635	503	618	768	853	550	392	295	309	mrps21
ENSXETG00000028044	1506	160	248	169	204	133	307	394	482	256	203	155	170	c12orf49
ENSXETG00000028047	817	0	0	0	0	0	0	24	30	15	0	1	3	
ENSXETG00000028048	807	0	0	0	0	0	0	6	13	4	0	1	0	ENSXETG00000028048
ENSXETG00000028049	982	0	0	1	3	1	1	149	189	119	57	35	39	nnmt
ENSXETG00000028050	1315	6	12	8	0	0	0	576	544	353	5	4	4	inmt
ENSXETG00000028051	2292	3	3	3	3	3	18	250	348	192	144	98	114	XB-GENE-5907649
ENSXETG00000028052	1783	0	0	0	3	0	3	18	30	19	19	15	12	ENSXETG00000028052
ENSXETG00000028057	849	0	0	0	0	0	0	0	0	0	0	0	0	
ENSXETG00000028061	786	43	25	33	33	29	80	214	248	162	119	94	88	ENSXETG00000028061
ENSXETG00000028065	1479	24	39	36	83	92	90	178	196	110	149	108	114	ENSXETG00000028065
ENSXETG00000028066	6527	505	748	657	2009	1532	2523	2440	2659	1749	3062	2169	2420	numa1
ENSXETG00000028069	1110	0	0	0	9	2	0	1	0	4	3	1	3	ENSXETG00000028069
ENSXETG00000028070	591	1	2	1	2	2	4	19	17	3	6	2	2	nat14
ENSXETG00000028075	147	55	74	44	109	69	225	874	1047	592	309	235	230	gmfg
ENSXETG00000028079	2085	18	18	22	20	18	26	184	274	123	42	29	34	aloxe3
ENSXETG00000028080	1582	6	5	4	29	14	32	38	67	31	27	12	18	fam124b
ENSXETG00000028081	2556	582	870	724	389	313	734	1739	1992	1140	401	288	299	serpine2
ENSXETG00000028082	566	461	661	544	632	497	588	948	1121	710	376	281	285	ssna1
ENSXETG00000028090	1960	115	180	153	290	231	759	399	459	253	292	197	233	slc38a9
ENSXETG00000028091	1569	5	0	1	210	168	21	23	23	17	6	3	5	ccno
ENSXETG00000028092	2531	7	16	10	29	18	58	557	650	349	64	48	39	gpx8
ENSXETG00000028096	738	0	0	0	2	1	2	38	41	23	9	10	8	XB-GENE-5947742
ENSXETG00000028097	1440	941	1411	1143	2310	1600	2654	3188	3311	2167	2341	1692	1815	ddx19b
ENSXETG00000028098	1535	1763	2342	2039	4285	3489	8526	1999	2487	1382	4537	3031	3690	clrn3
ENSXETG00000028100	952	1293	1949	1628	1313	977	1348	1116	1243	791	896	691	700	mrpl35
ENSXETG00000028102	486	1	8	8	27	17	33	157	170	111	59	47	41	ENSXETG00000028102
ENSXETG00000028105	115	0	0	0	0	0	0	0	0	0	0	0	0	
ENSXETG00000028111	76	3	3	1	2	0	3	2	1	1	2	0	2	SNORD36
ENSXETG00000028112	119	0	0	0	0	0	0	0	0	0	0	0	0	

ENSXETG00000028122	115	0	0	0	0	0	0	0	0	0	0	0	0	
ENSXETG00000028127	109	0	0	0	0	0	0	0	0	0	0	0	0	
ENSXETG00000028128	115	0	0	0	0	0	0	0	0	0	0	0	0	
ENSXETG00000028134	101	0	0	0	0	0	1	0	1	0	3	0	0	
ENSXETG00000028135	115	0	0	0	0	0	0	0	0	0	0	0	0	
ENSXETG00000028137	119	0	0	0	0	0	0	0	0	0	0	0	0	
ENSXETG00000028139	74	1	4	2	0	3	0	3	1	1	2	1	1	SNORD24
ENSXETG00000028141	119	0	0	0	0	0	0	0	0	0	0	0	0	
ENSXETG00000028143	164	0	0	0	0	0	0	0	0	0	0	0	0	
ENSXETG00000028150	147	2	8	8	3	3	5	4	10	5	3	0	7	SNORD15
ENSXETG00000028156	115	0	0	0	0	0	0	0	0	0	0	0	0	
ENSXETG00000028161	115	0	0	0	0	0	0	0	0	0	0	0	0	
ENSXETG00000028162	164	0	0	0	0	0	0	0	0	0	1	0	0	U1
ENSXETG00000028165	141	0	2	0	0	0	0	1	0	0	0	0	0	U4
ENSXETG00000028167	70	2	0	1	1	0	1	0	0	0	0	1	0	SNORD18
ENSXETG00000028168	132	3	0	0	0	0	0	0	2	1	0	1	0	SNORA71
ENSXETG00000028174	116	0	0	0	0	0	0	0	0	0	0	0	0	
ENSXETG00000028176	147	1	4	3	3	3	1	7	2	2	5	1	3	SNORD15
ENSXETG00000028179	108	0	0	0	0	0	0	0	0	0	0	0	1	5S_rRNA
ENSXETG00000028182	219	0	0	0	0	0	0	0	0	0	0	0	0	U3
ENSXETG00000028184	222	4	2	4	2	0	3	1	4	1	1	2	2	SNORA73
ENSXETG00000028186	115	0	0	0	0	0	0	0	0	0	0	0	0	
ENSXETG00000028188	146	13	24	21	7	5	8	15	18	8	15	7	9	SNORD15
ENSXETG00000028189	109	0	0	0	0	0	0	0	0	0	0	0	0	
ENSXETG00000028192	141	0	0	0	0	0	0	0	0	0	0	0	0	
ENSXETG00000028193	164	0	1	2	1	0	1	0	0	2	3	0	0	U1
ENSXETG00000028196	164	0	0	1	0	0	0	0	0	0	0	0	0	
ENSXETG00000028197	141	0	0	0	0	0	0	0	0	0	0	0	0	
ENSXETG00000028199	119	0	0	0	0	0	0	0	0	0	0	0	0	
ENSXETG00000028202	119	0	0	0	0	0	2	0	0	0	0	0	0	5S_rRNA
ENSXETG00000028209	119	0	0	0	0	0	0	0	0	0	0	0	0	
ENSXETG00000028210	155	2	5	7	6	3	3	5	2	3	7	4	9	SNORA62
ENSXETG00000028212	139	0	0	0	0	0	0	0	0	0	0	0	0	
ENSXETG00000028214	115	0	0	0	0	0	0	0	0	0	0	0	0	
ENSXETG00000028216	70	1	2	0	0	0	0	0	0	0	0	0	0	SNORD18
ENSXETG00000028217	77	1	3	2	0	0	0	0	1	0	0	0	2	
ENSXETG00000028221	119	0	0	0	0	0	0	0	0	0	0	0	0	
ENSXETG00000028223	141	0	0	0	0	0	0	0	0	0	0	0	0	
ENSXETG00000028226	109	0	0	0	0	0	0	0	0	0	0	0	0	
ENSXETG00000028234	115	0	0	0	0	0	0	0	0	0	0	0	0	
ENSXETG00000028239	115	0	0	0	0	0	0	0	0	0	0	0	0	
ENSXETG00000028242	191	0	0	0	0	0	0	0	0	0	0	0	0	
ENSXETG00000028244	149	0	0	0	0	0	0	0	0	0	0	0	0	
ENSXETG00000028246	125	0	0	0	0	0	0	0	0	0	0	0	0	
ENSXETG00000028249	115	0	0	0	0	0	0	0	0	0	0	0	0	
ENSXETG00000028251	131	31	38	28	12	9	10	30	47	19	28	12	24	SNORA63
ENSXETG00000028255	141	0	0	0	0	0	0	0	0	0	0	0	0	
ENSXETG00000028263	115	0	0	0	0	0	0	0	0	0	0	0	0	
ENSXETG00000028264	164	0	0	0	0	0	0	0	0	0	0	0	0	
ENSXETG00000028266	72	7	11	2	1	1	1	1	1	0	6	4	7	SNORD27
ENSXETG00000028267	141	0	0	0	0	0	0	0	0	0	0	0	0	
ENSXETG00000028273	143	2	2	3	1	4	3	0	1	1	3	3	0	SNORD15
ENSXETG00000028289	69	32	31	28	12	12	7	14	19	7	39	18	23	SNORD31
ENSXETG00000028297	119	0	0	0	0	0	0	0	0	0	0	0	0	
ENSXETG00000028299	115	0	0	0	0	0	0	0	0	0	0	0	0	
ENSXETG00000028302	305	1	1	1	0	0	0	1	0	1	2	2	2	7SK
ENSXETG00000028318	126	0	0	0	0	0	0	0	0	0	0	0	0	
ENSXETG00000028327	107	0	0	0	0	0	0	0	0	0	0	0	0	
ENSXETG00000028329	96	0	0	0	0	0	0	0	0	0	0	0	0	
ENSXETG00000028331	164	0	0	0	0	0	0	0	0	0	0	0	0	
ENSXETG00000028336	108	0	0	0	0	0	0	0	0	0	0	0	0	
ENSXETG00000028350	164	0	0	0	0	0	0	0	0	0	0	0	0	
ENSXETG00000028353	141	0	0	0	0	0	0	0	0	0	0	0	0	
ENSXETG00000028354	141	0	0	0	0	0	0	0	0	0	0	0	0	U4
ENSXETG00000028355	100	0	0	0	0	0	0	0	0	0	0	0	0	
ENSXETG00000028356	187	0	0	0	0	0	0	0	0	0	0	0	0	
ENSXETG00000028358	86	0	0	0	0	0	0	0	0	0	0	0	0	
ENSXETG00000028362	119	0	0	0	0	0	0	0	0	0	0	0	0	
ENSXETG00000028364	119	0	0	0	0	0	0	0	0	0	0	0	0	
ENSXETG00000028366	138	0	0	0	0	0	0	0	0	0	0	0	0	
ENSXETG00000028372	115	0	0	0	0	0	0	0	0	0	0	0	0	
ENSXETG00000028373	115	0	0	0	0	0	0	0	0	0	0	0	0	
ENSXETG00000028375	107	0	0	0	0	0	0	0	0	0	0	0	0	U6
ENSXETG00000028376	141	0	0	0	0	0	0	0	0	0	0	0	0	
ENSXETG00000028380	109	0	0	0	0	0	0	0	0	0	0	0	0	
ENSXETG00000028385	115	0	0	0	0	0	0	0	0	0	0	0	0	
ENSXETG00000028392	119	0	0	0	0	0	0	0	0	0	0	0	0	
ENSXETG00000028394	107	0	0	0	0	0	0	0	0	0	0	0	0	
ENSXETG00000028398	119	0	0	0	0	0	0	0	0	0	0	0	0	
ENSXETG00000028399	222	6	8	5	1	0	0	5	0	2	5	3	4	SNORA73
ENSXETG00000028407	119	0	0	0	0	0	0	1	2	0	1	1	0	5S_rRNA
ENSXETG00000028413	116	0	0	0	0	0	0	0	0	0	0	0	0	
ENSXETG00000028418	115	0	0	0	0	0	0	0	0	0	0	0	0	
ENSXETG00000028427	92	0	0	0	0	0	0	0	0	0	0	0	0	
ENSXETG00000028430	119	0	0	0	0	0	0	0	0	0	0	0	0	
ENSXETG00000028432	222	3	2	8	7	3	1	1	5	3	8	8	4	SNORA73
ENSXETG00000028435	100	2	5	7	3	4	3	8	3	1	7	0	2	SNORD16
ENSXETG00000028437	119	0	0	0	0	0	0	0	0	0	0	0	0	
ENSXETG00000028447	164	1	1	0	0	1	0	3	1	0	8	0	1	U1
ENSXETG00000028450	119	0	0	0	0	0	0	0	0	0	0	0	0	
ENSXETG00000028456	119	0	0	0	0	0	0	0	0	0	0	0	0	
ENSXETG00000028457	100	0	0	0	0	0	0	0	0	0	0	0	0	
ENSXETG00000028461	119	0	0	0	0	0	0	0	0	0	0	0	0	
ENSXETG00000028467	114	0	0	0	0	0	0	0	0	0	0	0	0	
ENSXETG00000028477	115	0	0	0	0	0	0	0	0	0	0	0	0	
ENSXETG00000028478	119	0	0	0	0	0	0	0	0	0	0	0	0	
ENSXETG00000028492	164	0	0	0	0	0	0	0	0	0	0	0	0	
ENSXETG00000028498	115	0	0	0	0	0	0	0	0	0	0	0	0	
ENSXETG00000028500	115	0	0	0	0	0	0	0	0	0	0	0	0	
ENSXETG00000028506	119	0	0	0	0	0	0	0	0	0	0	0	0	
ENSXETG00000028507	164	0	0	0	0	0	1	0	0	0	1	0	0	U1
ENSXETG00000028521	299	0	0	0	0	0	0	0	0	0	0	0	0	
ENSXETG00000028524	115	0	0	0	0	0	0	0	0	0	0	0	0	
ENSXETG00000028525	141	0	0	0	0	0	0	0	0	0	0	0	0	
ENSXETG00000028527	115	0	0	0	0	0	0	0	0	0	0	0	0	
ENSXETG00000028529	119	0	0	0	0	0	0	0	0	0	0	0	0	
ENSXETG00000028532	119	0	0	0	0	0	0	0	0	0	0	0	0	
ENSXETG00000028543	115	0	0	0	0	0	0	0	0	0	0	0	0	
ENSXETG00000028544	115	0	0	0	0	0	0	0	0	0	0	0	0	
ENSXETG00000028547	115	0	0	0	0	0	0	0	0	0	0	0	0	
ENSXETG00000028551	107	1	3	0	0	1	2	8	17	14	2	1	1	5S_rRNA

ENSXETG00000028556	115	0	0	0	0	0	0	0	0	0	0	0	0	
ENSXETG00000028563	72	3	4	5	0	0	1	3	3	6	1	1	2	SNORD18
ENSXETG00000028564	115	0	0	0	0	0	0	0	0	0	0	0	0	
ENSXETG00000028565	164	0	0	0	0	0	0	3	1	0	0	0	0	U1
ENSXETG00000028573	115	0	0	0	0	0	0	0	0	0	0	0	0	
ENSXETG00000028575	164	0	0	0	0	0	0	0	0	0	0	0	0	
ENSXETG00000028577	107	0	0	0	0	1	0	0	0	1	0	0	0	U6
ENSXETG00000028579	119	0	0	0	0	0	0	0	0	0	0	0	0	
ENSXETG00000028581	119	0	0	0	0	0	0	0	0	0	0	0	0	
ENSXETG00000028586	115	0	0	0	0	0	0	0	0	0	0	0	0	
ENSXETG00000028593	131	3	1	7	2	3	4	4	3	2	2	1	0	SNORA63
ENSXETG00000028606	91	0	0	0	0	0	0	0	0	0	0	0	0	SNORD14
ENSXETG00000028609	222	14	19	18	6	5	8	11	12	12	10	8	6	SNORA73
ENSXETG00000028618	443	0	0	0	0	0	1	0	2	2	0	1	1	
ENSXETG00000028621	115	0	0	0	0	0	0	0	0	0	0	0	0	
ENSXETG00000028626	115	0	0	0	0	0	0	0	0	0	0	0	0	
ENSXETG00000028627	119	0	0	0	0	0	0	0	0	0	0	0	0	
ENSXETG00000028628	115	0	0	0	0	0	0	0	0	0	0	0	0	
ENSXETG00000028629	115	0	0	0	0	0	0	0	0	0	0	0	0	
ENSXETG00000028633	111	0	0	0	0	0	0	0	0	0	0	0	0	
ENSXETG00000028635	109	0	0	0	0	0	0	0	0	0	0	0	0	
ENSXETG00000028639	115	0	0	0	0	0	0	0	0	0	0	0	0	
ENSXETG00000028651	119	0	0	0	0	0	0	0	0	0	0	0	0	
ENSXETG00000028657	201	0	0	0	0	0	0	0	0	0	0	0	0	
ENSXETG00000028664	141	0	0	0	0	0	0	0	0	0	0	0	0	
ENSXETG00000028665	164	0	0	0	0	0	0	0	0	0	0	0	0	
ENSXETG00000028666	115	0	0	0	0	0	0	0	0	0	0	0	0	
ENSXETG00000028667	65	140	178	173	53	52	39	60	78	38	45	43	43	SNORD29
ENSXETG00000028668	115	0	0	0	0	0	0	0	0	0	0	0	0	
ENSXETG00000028678	147	0	0	0	0	0	0	0	0	0	0	0	0	
ENSXETG00000028693	45	0	0	0	0	0	0	0	0	0	0	0	0	
ENSXETG00000028709	117	0	0	0	0	0	0	0	0	0	0	0	0	
ENSXETG00000028712	119	0	0	0	0	0	1	0	0	0	0	0	0	5S_rRNA
ENSXETG00000028717	141	0	0	0	0	0	0	0	0	0	0	0	0	
ENSXETG00000028734	192	0	0	0	0	0	0	0	0	0	0	0	0	
ENSXETG00000028737	119	0	0	0	0	0	0	0	0	0	0	0	0	
ENSXETG00000028739	115	0	0	0	1	0	0	0	0	0	0	0	0	
ENSXETG00000028742	115	0	0	0	0	0	0	0	0	0	0	0	0	
ENSXETG00000028745	119	0	0	0	0	0	0	0	0	0	0	0	0	
ENSXETG00000028750	119	0	0	0	0	0	0	0	0	0	0	0	0	
ENSXETG00000028751	115	0	0	0	0	0	0	0	0	0	0	0	0	
ENSXETG00000028760	163	0	0	0	0	0	0	0	0	0	0	0	0	
ENSXETG00000028764	330	57	71	56	67	59	61	166	72	93	203	90	95	7SK
ENSXETG00000028765	141	0	0	0	0	0	0	0	0	0	0	0	0	
ENSXETG00000028767	109	0	0	0	0	0	0	0	0	0	0	0	0	
ENSXETG00000028777	109	0	0	0	0	0	0	0	0	0	0	0	0	
ENSXETG00000028790	115	0	0	0	0	0	0	0	0	0	0	0	0	
ENSXETG00000028791	164	0	0	0	0	0	0	0	0	0	0	0	0	
ENSXETG00000028792	100	5	10	5	8	4	1	4	5	0	0	0	2	SNORD16
ENSXETG00000028794	164	0	0	0	0	0	0	0	0	0	0	0	0	
ENSXETG00000028800	115	0	0	0	0	0	0	0	0	0	0	0	0	
ENSXETG00000028815	119	0	0	0	0	0	0	0	0	0	0	0	0	
ENSXETG00000028816	148	0	0	0	0	0	0	0	0	0	0	0	0	
ENSXETG00000028825	115	0	0	0	0	0	0	0	0	0	0	0	0	
ENSXETG00000028828	119	0	0	0	0	0	0	0	0	0	0	0	0	
ENSXETG00000028837	193	0	0	0	0	0	0	0	0	0	0	0	0	
ENSXETG00000028841	164	0	0	0	0	0	0	0	0	0	0	0	0	
ENSXETG00000028843	115	0	0	0	0	0	0	0	0	0	0	0	0	
ENSXETG00000028853	62	10	5	8	8	2	2	4	6	2	2	3	1	snR47
ENSXETG00000028855	119	0	0	0	0	0	0	0	0	0	0	0	0	
ENSXETG00000028856	377	0	0	0	1	0	0	0	0	0	0	0	0	
ENSXETG00000028865	115	0	0	0	0	0	0	0	0	0	0	0	0	
ENSXETG00000028866	219	0	0	0	0	0	0	2	3	0	0	0	0	U3
ENSXETG00000028868	160	0	0	0	0	0	0	0	0	0	0	0	0	
ENSXETG00000028872	222	9	4	9	10	4	3	6	12	3	4	5	2	SNORA73
ENSXETG00000028882	133	2	9	3	10	8	4	4	5	9	0	1	3	SNORA2
ENSXETG00000028885	107	1	2	0	1	0	0	0	1	0	0	0	0	U6
ENSXETG00000028886	69	8	4	5	1	1	1	7	6	5	6	4	1	SNORD18
ENSXETG00000028895	119	0	0	0	0	0	0	0	0	0	0	0	0	
ENSXETG00000028905	149	0	0	0	0	0	0	0	0	0	0	0	0	
ENSXETG00000028911	141	0	0	0	0	0	0	0	0	0	0	0	0	
ENSXETG00000028914	96	0	0	0	0	0	2	7	3	7	1	2	3	xtr-mir-199a
ENSXETG00000028915	81	0	0	0	0	0	0	0	0	0	0	1	0	
ENSXETG00000028916	94	0	1	2	0	0	0	1	0	1	0	1	1	xtr-mir-140
ENSXETG00000028917	89	0	2	0	0	0	0	2	1	1	4	1	3	xtr-mir-200a
ENSXETG00000028918	84	0	0	0	0	0	0	0	0	0	0	0	1	
ENSXETG00000028969	88	0	0	0	0	0	0	0	0	0	0	0	0	
ENSXETG00000028970	108	2	2	4	4	0	2	2	5	5	0	1	2	xtr-mir-7-1
ENSXETG00000028971	97	0	0	0	0	0	0	0	0	0	0	0	0	
ENSXETG00000028972	86	0	0	0	0	0	0	0	0	0	0	0	0	
ENSXETG00000028973	111	0	0	0	1	1	0	1	0	0	0	0	0	ENSXETG00000028973
ENSXETG00000028974	74	0	0	0	0	0	0	0	0	0	0	0	0	
ENSXETG00000028975	75	0	0	0	0	0	0	0	0	0	0	0	0	
ENSXETG00000028976	88	0	0	0	0	0	0	0	0	0	0	0	0	
ENSXETG00000028977	78	0	1	1	1	0	1	0	0	0	0	0	1	xtr-mir-30c-2
ENSXETG00000028978	78	0	0	0	0	0	0	0	0	0	0	0	0	
ENSXETG00000028979	73	0	0	0	0	0	0	0	0	0	0	0	0	xtr-mir-107
ENSXETG00000028980	87	0	0	0	0	0	0	0	0	0	0	0	1	
ENSXETG00000028981	96	1	0	3	8	6	6	17	11	11	7	10	3	xtr-mir-27b
ENSXETG00000028982	71	0	0	0	0	0	0	0	0	0	0	0	0	
ENSXETG00000028983	86	29	41	29	32	22	34	5	6	9	5	9	7	xtr-mir-365-1
ENSXETG00000028984	83	0	0	2	0	1	0	1	1	0	7	4	5	xtr-mir-101a-2
ENSXETG00000028985	69	0	0	0	0	0	0	0	0	0	0	0	0	
ENSXETG00000028986	84	0	0	0	0	0	0	0	0	0	1	0	0	
ENSXETG00000028987	107	0	0	0	0	0	0	3	5	5	0	0	2	xtr-mir-214
ENSXETG00000028988	86	0	0	0	0	0	0	0	1	0	0	0	0	
ENSXETG00000028989	88	0	0	0	0	0	0	0	1	2	0	0	0	
ENSXETG00000028990	83	0	0	1	0	0	0	0	0	1	0	0	1	xtr-mir-103-2
ENSXETG00000028991	88	0	0	0	0	0	0	0	0	0	0	0	0	
ENSXETG00000028992	85	0	0	0	0	0	0	0	0	0	0	0	0	
ENSXETG00000028993	80	0	0	0	0	0	0	0	0	0	0	0	0	
ENSXETG00000028994	88	0	0	0	0	0	0	0	0	0	0	0	0	
ENSXETG00000028995	71	0	0	0	0	0	0	0	0	0	0	0	0	
ENSXETG00000028996	87	0	0	0	0	0	0	0	0	0	0	0	0	
ENSXETG00000028997	101	0	0	0	0	0	0	0	0	0	0	0	0	
ENSXETG00000028998	101	0	0	0	0	0	0	0	0	0	0	0	0	
ENSXETG00000028999	101	0	0	0	0	0	0	0	0	0	0	0	0	xtr-mir-137-3
ENSXETG00000029000	94	0	0	0	0	0	0	0	0	0	0	0	0	
ENSXETG00000029003	85	0	2	0	0	0	1	0	0	0	0	1	0	xtr-mir-103-1
ENSXETG00000029004	73	0	0	0	0	0	0	0	0	0	0	0	0	
ENSXETG00000029005	91	1	2	2	0	1	1	2	1	0	0	0	0	

ENSXETG00000029006	86	0	0	0	0	0	0	0	0	0	0	0	0	
ENSXETG00000029007	100	0	0	0	1	0	0	0	0	0	1	0	0	
ENSXETG00000029008	84	0	0	0	0	0	0	0	0	0	0	0	0	
ENSXETG00000029009	70	0	0	0	0	0	0	0	0	0	0	0	0	
ENSXETG00000029010	73	0	0	0	0	0	0	0	0	0	0	0	0	
ENSXETG00000029011	71	0	0	0	0	0	0	0	0	0	0	0	0	
ENSXETG00000029012	83	0	1	0	1	0	0	2	0	1	5	0	4	
ENSXETG00000029013	85	1	5	3	3	2	3	31	43	28	9	14	11	xtr-mir-125b-2
ENSXETG00000029014	76	0	0	0	0	0	0	0	0	0	0	0	0	
ENSXETG00000029015	91	0	0	0	0	0	0	0	0	0	0	0	0	
ENSXETG00000029016	87	0	0	0	0	0	0	0	0	0	0	1	0	
ENSXETG00000029017	85	10	14	11	10	5	4	11	4	0	25	13	15	xtr-let-7i
ENSXETG00000029018	62	1	2	0	0	0	0	3	0	3	1	1	3	xtr-mir-451
ENSXETG00000029030	153	9	19	12	9	10	3	7	7	3	14	9	10	SNORA62
ENSXETG00000029035	115	0	0	0	0	0	0	0	0	0	0	0	0	
ENSXETG00000029040	119	0	0	0	0	0	0	0	0	0	0	0	0	
ENSXETG00000029041	121	0	0	0	0	0	0	0	0	0	0	0	0	
ENSXETG00000029042	131	3	5	3	5	2	2	0	1	1	2	1	1	SNORA71
ENSXETG00000029043	87	0	0	0	0	0	0	0	0	0	0	0	0	
ENSXETG00000029044	115	0	0	0	0	0	0	0	0	0	0	0	0	
ENSXETG00000029045	137	0	0	0	0	0	0	0	0	0	0	0	0	
ENSXETG00000029059	121	0	0	0	0	0	0	0	0	0	0	0	0	
ENSXETG00000029062	115	0	0	0	0	0	0	0	0	0	0	0	0	
ENSXETG00000029063	219	0	0	0	0	0	0	0	0	0	0	0	1	
ENSXETG00000029069	120	0	0	0	0	0	0	0	0	0	0	0	0	
ENSXETG00000029070	107	0	0	0	0	0	0	0	0	0	0	2	0	
ENSXETG00000029072	119	0	0	0	0	0	0	0	0	0	0	0	0	
ENSXETG00000029073	121	0	0	0	0	0	0	0	0	0	0	0	0	
ENSXETG00000029080	114	0	0	0	0	0	0	0	0	0	0	0	0	
ENSXETG00000029084	108	0	0	0	0	0	0	0	0	0	0	0	0	
ENSXETG00000029085	93	0	0	0	2	2	0	2	5	2	2	4	4	xtr-let-7e-2
ENSXETG00000029086	86	0	1	0	1	0	1	1	5	0	1	0	1	xtr-mir-223
ENSXETG00000029087	89	0	0	0	0	0	0	0	0	0	0	1	0	
ENSXETG00000029088	62	0	0	0	0	0	0	0	0	0	0	0	0	
ENSXETG00000029089	83	0	0	0	0	1	0	0	0	0	0	0	0	xtr-mir-23a-2
ENSXETG00000029090	79	0	0	0	0	0	0	0	0	0	0	1	0	
ENSXETG00000029091	103	0	0	0	0	0	0	2	1	0	0	2	0	xtr-mir-7-2
ENSXETG00000029092	83	0	0	0	0	0	0	5	4	6	1	0	0	xtr-mir-181b-2
ENSXETG00000029093	74	0	0	0	2	1	1	0	0	0	0	2	0	xtr-mir-15c
ENSXETG00000029094	84	0	0	0	0	0	0	1	0	0	3	0	0	xtr-let-7g
ENSXETG00000029095	86	5	3	7	6	5	3	29	37	21	48	43	52	xtr-mir-145
ENSXETG00000029096	90	0	0	0	0	0	0	0	0	0	0	0	0	
ENSXETG00000029097	83	0	0	0	0	0	0	0	0	0	0	0	0	
ENSXETG00000029098	63	0	0	0	0	0	0	0	0	0	0	0	0	
ENSXETG00000029099	84	0	0	0	0	0	0	0	0	0	0	0	0	
ENSXETG00000029100	90	0	0	0	0	0	0	1	0	0	0	0	0	
ENSXETG00000029101	80	0	0	0	0	0	1	0	0	0	0	1	0	xtr-mir-29b
ENSXETG00000029102	68	0	0	0	0	0	0	0	0	0	0	0	0	
ENSXETG00000029103	69	0	0	0	0	0	0	0	0	0	0	0	0	
ENSXETG00000029104	87	3	6	5	6	7	8	2	0	1	18	11	13	xtr-mir-429
ENSXETG00000029105	76	0	1	0	0	0	1	4	7	6	5	5	2	xtr-mir-133d
ENSXETG00000029106	80	0	0	0	0	0	0	2	0	0	0	0	2	
ENSXETG00000029107	78	0	0	0	0	0	0	0	0	0	0	0	0	
ENSXETG00000029108	78	0	1	0	0	0	0	0	0	0	3	3	1	xtr-mir-92a-1
ENSXETG00000029109	73	0	0	1	0	0	0	0	0	0	0	0	1	xtr-mir-92a-2
ENSXETG00000029110	62	0	0	0	0	0	0	0	0	0	0	0	0	
ENSXETG00000029111	86	0	0	0	0	0	0	0	0	0	0	0	0	
ENSXETG00000029112	73	0	0	0	0	0	0	0	0	0	0	0	0	
ENSXETG00000029113	74	0	0	0	0	0	0	0	0	0	0	0	0	
ENSXETG00000029114	88	0	0	0	0	0	0	0	0	0	0	0	0	
ENSXETG00000029115	83	0	0	0	0	0	0	0	0	0	0	0	0	
ENSXETG00000029116	68	2	2	0	0	0	13	20	9	6	33	17	17	xtr-mir-24b
ENSXETG00000029117	85	0	0	0	0	2	0	0	0	0	0	0	0	
ENSXETG00000029118	85	0	0	0	0	0	0	0	0	0	0	0	0	
ENSXETG00000029119	97	5	9	16	2	5	5	3	4	2	9	6	4	xtr-mir-215
ENSXETG00000029120	77	0	0	0	1	1	0	3	1	2	0	2	1	xtr-mir-425
ENSXETG00000029121	85	0	0	0	0	1	0	0	2	1	1	0	2	
ENSXETG00000029122	81	0	0	0	0	0	0	0	0	0	0	0	0	
ENSXETG00000029123	87	0	0	0	0	0	0	0	0	0	0	0	0	
ENSXETG00000029124	84	0	0	0	0	0	0	0	0	0	0	0	0	
ENSXETG00000029125	76	0	0	0	0	0	3	1	0	0	3	3	3	xtr-mir-27a
ENSXETG00000029126	82	0	0	0	0	0	0	0	0	0	0	0	0	
ENSXETG00000029127	69	0	0	0	0	0	0	0	0	0	0	0	0	
ENSXETG00000029128	71	0	0	0	0	0	0	0	0	0	0	0	0	
ENSXETG00000029129	84	0	0	0	0	0	0	0	0	0	0	0	0	
ENSXETG00000029130	82	1	1	2	0	0	0	0	0	0	1	1	1	xtr-let-7b
ENSXETG00000029131	89	0	0	0	1	0	0	0	0	0	6	3	2	xtr-mir-200b
ENSXETG00000029132	95	0	0	0	0	0	0	9	5	5	1	1	1	xtr-mir-181a-2
ENSXETG00000029133	83	0	0	0	0	0	0	0	0	0	0	0	0	
ENSXETG00000029134	73	0	0	0	0	0	0	0	0	0	0	0	0	
ENSXETG00000029135	64	0	0	0	0	0	0	0	0	0	0	0	0	
ENSXETG00000029136	95	0	0	0	0	0	0	0	0	0	0	0	0	
ENSXETG00000029139	87	0	0	0	0	0	0	0	0	0	0	0	0	
ENSXETG00000029140	71	0	0	0	0	0	0	0	0	0	0	0	0	
ENSXETG00000029141	89	0	0	1	0	0	0	0	0	0	0	0	0	
ENSXETG00000029142	78	1	0	0	3	1	0	0	2	0	7	2	2	xtr-mir-23a-1
ENSXETG00000029143	64	0	1	0	0	0	0	1	0	1	0	0	0	xtr-mir-150
ENSXETG00000029144	69	0	0	0	0	0	0	0	1	0	2	0	2	xtr-mir-126
ENSXETG00000029145	90	6	9	5	1	1	0	1	2	1	5	2	1	xtr-mir-146a
ENSXETG00000029146	93	0	1	2	0	0	0	0	3	1	0	2	0	xtr-mir-221
ENSXETG00000029147	86	0	0	0	0	0	0	0	0	0	0	0	0	
ENSXETG00000029148	105	0	0	0	0	0	0	0	0	0	0	0	0	
ENSXETG00000029149	87	0	0	0	0	0	0	0	0	0	0	0	0	
ENSXETG00000029150	85	0	0	0	0	0	0	0	0	0	1	1	0	xtr-mir-26-1
ENSXETG00000029151	87	0	0	2	0	0	0	3	3	1	0	0	0	xtr-mir-363
ENSXETG00000029152	88	0	0	0	0	0	0	0	0	0	0	0	0	
ENSXETG00000029153	75	0	0	0	0	0	0	0	0	0	0	0	0	
ENSXETG00000029154	90	2	1	2	3	3	1	1	0	1	4	7	2	xtr-mir-29c
ENSXETG00000029155	67	7	9	6	12	19	3	41	40	30	39	31	28	xtr-mir-24a
ENSXETG00000029156	82	0	0	0	0	0	0	0	0	0	0	0	0	
ENSXETG00000029157	91	21	42	43	6	4	7	22	17	11	42	23	25	xtr-mir-210
ENSXETG00000029158	86	2	5	1	1	0	3	0	1	0	3	1	1	xtr-mir-194-2
ENSXETG00000029159	90	0	0	0	0	0	0	0	0	0	0	0	0	
ENSXETG00000029160	82	0	0	0	0	0	0	0	0	0	0	0	0	
ENSXETG00000029161	82	0	0	0	0	0	0	0	0	0	0	0	0	
ENSXETG00000029162	83	0	0	0	0	0	0	0	1	0	2	0	0	
ENSXETG00000029163	64	0	0	1	1	0	0	0	0	1	1	0	1	xtr-mir-222
ENSXETG00000029164	105	0	0	0	0	0	0	0	0	0	0	0	0	
ENSXETG00000029165	96	0	0	0	0	0	0	0	0	0	0	0	0	
ENSXETG00000029166	110	0	0	0	0	0	1	5	3	7	0	1	0	xtr-mir-199b
ENSXETG00000029167	76	0	0	0	0	0	0	0	0	0	0	0	0	

ENSXETG00000029168	63	0	0	0	0	0	0	0	0	0	0	0	0	
ENSXETG00000029169	81	1	0	0	0	0	0	0	1	0	0	0	0	xtr-mir-30d
ENSXETG00000029170	83	0	0	0	0	0	0	4	8	4	0	0	0	xtr-mir-181b-1
ENSXETG00000029171	90	0	0	0	0	0	0	0	0	0	0	0	0	
ENSXETG00000029172	67	0	0	0	0	0	0	0	0	0	0	0	0	
ENSXETG00000029173	83	0	0	0	0	0	0	0	0	0	0	0	0	
ENSXETG00000029174	83	0	0	0	0	0	0	0	0	0	0	0	0	
ENSXETG00000029175	92	6	4	0	5	2	4	3	7	5	1	0	1	xtr-mir-22
ENSXETG00000029176	72	0	0	0	0	0	0	0	0	0	0	0	0	
ENSXETG00000029177	73	0	0	0	0	0	0	0	0	0	0	0	0	
ENSXETG00000029178	88	0	0	0	0	0	0	0	0	0	0	0	0	
ENSXETG00000029179	88	1	0	0	0	0	0	0	0	0	0	0	1	
ENSXETG00000029180	60	1	0	0	0	0	0	0	0	0	0	0	3	xtr-mir-338-2
ENSXETG00000029181	106	0	0	0	0	0	0	0	0	0	0	0	0	
ENSXETG00000029182	75	0	0	0	0	0	0	0	0	0	0	0	0	
ENSXETG00000029183	88	0	0	0	0	0	0	0	0	0	0	0	0	
ENSXETG00000029186	80	0	0	1	0	0	0	0	0	0	0	0	0	
ENSXETG00000029187	87	37	73	61	44	40	34	17	21	10	44	36	36	xtr-mir-192
ENSXETG00000029188	82	1	1	2	5	1	0	4	3	4	2	1	2	xtr-mir-16a
ENSXETG00000029189	76	0	0	0	0	0	0	0	0	0	0	0	0	
ENSXETG00000029190	70	0	0	0	0	1	0	0	1	0	0	1	0	xtr-mir-16c
ENSXETG00000029191	87	0	0	0	0	0	0	0	0	0	0	0	0	
ENSXETG00000029192	97	9	10	8	2	6	2	0	6	4	15	4	8	xtr-mir-148b
ENSXETG00000029193	69	0	0	0	0	0	0	0	0	0	0	0	1	
ENSXETG00000029194	87	3	3	5	5	1	3	2	10	4	8	3	8	xtr-mir-29a
ENSXETG00000029195	86	0	0	1	0	0	0	0	0	0	0	1	1	xtr-mir-148a
ENSXETG00000029196	80	7	6	6	3	4	1	2	3	0	5	3	7	xtr-mir-194-1
ENSXETG00000029197	87	0	0	0	0	0	0	0	0	0	0	0	0	
ENSXETG00000029200	100	0	0	0	1	0	0	0	0	0	0	0	0	
ENSXETG00000029201	87	0	0	0	0	0	0	0	0	0	0	0	0	
ENSXETG00000029202	83	0	0	0	0	0	0	0	0	0	0	0	0	
ENSXETG00000029203	62	0	0	0	0	0	0	0	0	0	2	0	0	xtr-mir-144
ENSXETG00000029204	74	0	0	0	0	0	0	0	0	0	0	0	0	
ENSXETG00000029205	87	0	0	0	0	0	0	0	0	0	0	0	0	
ENSXETG00000029206	71	0	0	0	0	0	0	0	0	0	0	0	0	
ENSXETG00000029207	96	5	5	2	10	10	28	50	64	24	26	18	19	xtr-mir-34a
ENSXETG00000029208	97	0	0	0	0	0	0	0	0	0	0	0	0	
ENSXETG00000029209	85	0	0	0	0	0	0	0	0	0	0	0	0	
ENSXETG00000029211	108	0	0	0	0	0	0	0	0	0	0	0	0	
ENSXETG00000029213	101	0	0	0	0	0	0	0	0	0	0	0	0	
ENSXETG00000029214	70	0	2	0	1	0	0	1	0	1	0	1	0	xtr-mir-375
ENSXETG00000029217	90	0	0	0	0	0	0	0	0	0	0	0	0	
ENSXETG00000029218	67	0	0	0	0	0	0	0	0	0	0	0	0	
ENSXETG00000029219	97	0	0	0	0	0	0	0	0	0	0	0	0	
ENSXETG00000029220	67	0	0	0	0	0	0	0	0	0	0	0	0	
ENSXETG00000029221	92	2	1	0	0	0	1	2	5	2	4	1	3	ENSXETG00000029221
ENSXETG00000029223	85	0	0	0	0	0	0	0	0	0	0	0	0	
ENSXETG00000029224	84	0	0	0	0	0	0	0	0	0	0	0	0	
ENSXETG00000029225	90	0	0	0	0	0	0	0	0	0	0	0	0	
ENSXETG00000029228	91	0	0	0	0	0	0	0	0	0	0	0	0	
ENSXETG00000029229	88	2	2	0	1	1	5	0	1	0	1	0	0	ENSXETG00000029229
ENSXETG00000029230	100	0	1	1	0	0	0	2	3	1	4	1	5	xtr-mir-146b
ENSXETG00000029233	89	0	0	0	0	0	0	1	0	0	0	0	0	SNORD70
ENSXETG00000029234	140	7	16	3	7	6	4	10	7	8	9	3	3	SNORA8
ENSXETG00000029242	134	1	3	0	1	1	2	0	1	0	1	0	0	SNORA22
ENSXETG00000029243	79	0	2	2	4	2	0	2	3	0	2	1	1	SNORD74
ENSXETG00000029246	91	19	18	13	4	2	4	12	11	5	8	0	3	SNORD12
ENSXETG00000029250	92	4	7	1	3	4	3	1	0	2	2	0	1	SNORD35
ENSXETG00000029253	92	0	0	0	0	0	0	0	0	0	0	0	0	
ENSXETG00000029259	132	0	1	0	0	0	0	0	0	0	0	0	0	
ENSXETG00000029264	80	0	0	1	0	0	0	3	0	0	1	0	2	SNORD103
ENSXETG00000029265	132	0	0	0	0	1	1	0	1	0	1	0	2	SNORA3
ENSXETG00000029268	129	5	7	7	2	4	5	4	4	5	2	4	3	SNORA69
ENSXETG00000029269	71	13	11	10	1	2	2	6	12	1	6	6	7	SNORD26
ENSXETG00000029270	84	5	5	5	0	0	1	4	1	0	3	3	2	SNORD35
ENSXETG00000029272	126	52	63	55	34	29	12	57	47	25	38	28	37	SNORD22
ENSXETG00000029278	86	0	0	0	0	0	0	0	0	0	0	0	0	
ENSXETG00000029279	92	20	31	20	3	3	8	13	26	7	7	3	5	SNORD12
ENSXETG00000029283	73	82	92	85	16	20	9	36	48	19	31	25	20	SNORD30
ENSXETG00000029286	180	0	0	1	3	0	1	0	1	1	1	1	0	SNORA23
ENSXETG00000029287	94	2	3	1	0	0	0	0	1	1	0	1	1	snoZ30
ENSXETG00000029288	132	1	2	1	0	0	0	0	0	0	0	0	0	
ENSXETG00000029289	180	0	5	1	1	0	0	2	2	3	1	2	2	SNORA23
ENSXETG00000029293	133	9	11	15	2	1	5	6	10	4	13	5	8	SNORA21
ENSXETG00000029301	118	0	0	0	0	0	0	0	0	0	0	0	0	
ENSXETG00000029302	132	2	3	1	0	0	0	0	2	0	0	0	0	SNORA3
ENSXETG00000029304	119	0	0	0	0	0	0	0	0	0	0	0	0	
ENSXETG00000029307	133	2	5	3	0	0	0	2	0	2	3	0	3	SNORA21
ENSXETG00000029309	90	16	30	13	0	3	3	4	10	11	8	2	8	SNORD12
ENSXETG00000029311	91	15	22	22	2	1	7	5	16	3	13	6	7	SNORD35
ENSXETG00000029312	105	0	0	0	0	0	0	0	0	0	0	0	0	
ENSXETG00000029314	242	12	20	11	8	4	2	0	10	5	16	4	7	SNORA53
ENSXETG00000029320	92	0	0	0	0	0	0	0	0	0	0	0	0	
ENSXETG00000029323	92	0	0	0	0	0	0	0	0	0	0	0	0	
ENSXETG00000029325	90	0	0	0	0	0	0	0	0	0	0	0	0	
ENSXETG00000029327	82	0	0	0	0	0	0	0	0	0	0	0	0	
ENSXETG00000029328	74	0	0	0	0	0	0	0	0	0	0	0	0	
ENSXETG00000029332	58	0	0	0	0	0	0	0	0	0	0	0	0	
ENSXETG00000029345	67	0	0	0	0	0	0	0	0	0	0	0	0	
ENSXETG00000029348	81	0	0	0	0	0	0	0	0	0	0	1	0	
ENSXETG00000029357	84	0	0	0	0	0	0	0	0	0	0	0	0	
ENSXETG00000029360	69	0	0	0	0	0	0	0	0	0	0	0	0	
ENSXETG00000029361	79	0	0	0	0	0	0	0	0	0	0	1	0	ENSXETG00000029361
ENSXETG00000029362	71	2	19	4	5	8	1	6	6	8	7	4	4	SNORD99
ENSXETG00000029364	71	8	6	9	3	1	2	3	5	5	9	3	2	SNORD99
ENSXETG00000029367	147	0	0	0	0	0	0	0	0	0	0	0	0	
ENSXETG00000029370	101	0	0	0	0	0	0	0	0	0	0	0	0	
ENSXETG00000029374	146	0	0	0	0	0	0	0	0	0	0	0	0	
ENSXETG00000029380	85	0	0	0	0	0	0	0	0	0	0	0	0	
ENSXETG00000029382	141	0	2	0	0	0	0	1	1	0	0	0	0	SNORD94
ENSXETG00000029383	83	0	1	0	0	0	0	0	0	0	0	0	0	SNORD100
ENSXETG00000029389	135	10	18	21	6	2	5	8	16	4	11	2	12	SNORA42
ENSXETG00000029391	69	0	0	0	0	0	0	0	0	0	0	0	0	
ENSXETG00000029392	121	0	0	0	0	0	0	0	0	0	0	0	0	
ENSXETG00000029393	146	0	0	0	0	0	0	0	0	0	0	0	0	
ENSXETG00000029395	67	0	0	0	0	0	0	0	0	0	0	0	0	
ENSXETG00000029396	137	0	0	0	0	0	0	0	0	1	0	0	0	U11
ENSXETG00000029398	71	16	20	21	15	7	5	12	18	6	11	18	12	SNORD99
ENSXETG00000029399	140	5	4	2	1	1	0	2	4	1	4	1	6	SNORD94
ENSXETG00000029400	91	19	9	5	3	1	3	4	5	1	4	3	5	SNORD100
ENSXETG00000029402	83	0	3	0	0	0	0	0	0	0	0	0	1	SNORD100

ENSXETG00000029404	120	0	0	0	0	0	0	0	0	0	0	0	0	
ENSXETG00000029405	111	0	0	0	0	0	0	0	0	0	0	0	0	
ENSXETG00000029406	86	0	0	0	0	0	0	0	0	0	0	0	0	
ENSXETG00000029407	159	0	0	0	0	0	0	0	0	0	0	0	0	
ENSXETG00000029409	153	0	0	0	0	0	0	0	0	0	0	0	0	
ENSXETG00000029410	165	0	1	0	0	0	0	0	0	0	0	0	0	U4
ENSXETG00000029412	214	0	0	0	0	0	0	0	0	0	0	0	0	
ENSXETG00000029413	133	0	0	0	0	0	0	0	0	0	0	0	0	
ENSXETG00000029415	131	1	2	2	5	3	2	0	3	1	0	3	0	SNORA68
ENSXETG00000029417	74	0	0	0	0	0	0	0	0	0	0	0	0	SNORD101
ENSXETG00000029418	105	0	0	0	0	0	0	0	0	0	0	0	0	
ENSXETG00000029419	184	0	0	0	0	0	0	0	0	0	0	0	0	
ENSXETG00000029422	150	0	0	0	0	0	0	0	0	0	2	1	0	
ENSXETG00000029423	118	0	0	0	0	0	0	0	0	0	0	0	0	
ENSXETG00000029424	135	0	0	0	0	0	0	0	0	0	0	0	0	
ENSXETG00000029425	133	0	0	0	0	0	0	0	0	0	0	0	0	
ENSXETG00000029426	134	1	5	4	0	0	3	3	3	2	5	3	5	SNORA9
ENSXETG00000029430	146	0	0	0	0	0	0	0	0	0	0	0	0	
ENSXETG00000029432	160	0	0	0	0	0	0	0	0	0	0	0	0	
ENSXETG00000029433	137	4	3	0	1	1	0	0	0	0	3	1	3	SNORA42
ENSXETG00000029434	191	0	0	0	0	0	0	0	0	0	0	0	0	
ENSXETG00000029436	152	0	0	0	0	0	0	0	0	0	0	0	0	
ENSXETG00000029437	134	16	21	21	16	8	8	19	20	4	34	25	20	SNORA9
ENSXETG00000029438	147	0	0	0	0	0	0	0	0	0	0	0	0	
ENSXETG00000029439	135	0	0	0	0	0	0	0	0	0	0	0	0	
ENSXETG00000029441	111	0	0	0	0	0	0	0	0	0	0	0	0	
ENSXETG00000029442	82	0	0	0	0	0	0	0	0	0	0	0	0	
ENSXETG00000029443	175	0	0	0	0	0	0	0	0	0	0	0	0	
ENSXETG00000029444	115	0	0	0	0	0	0	0	0	0	0	0	0	
ENSXETG00000029446	273	3	12	11	11	3	36	13	12	10	68	14	17	RNase_MRP
ENSXETG00000029447	176	0	0	0	0	0	0	0	0	0	0	0	0	
ENSXETG00000029449	133	9	21	10	5	0	4	10	6	11	4	1	2	SNORA17
ENSXETG00000029450	158	0	0	0	0	0	0	0	0	0	0	0	0	
ENSXETG00000029456	167	0	0	0	0	0	0	0	0	0	0	0	0	
ENSXETG00000029462	158	0	0	0	0	0	0	0	0	0	0	0	0	
ENSXETG00000029463	158	0	0	0	0	0	0	0	0	0	0	0	0	
ENSXETG00000029464	175	0	0	0	0	0	0	0	0	0	0	0	0	
ENSXETG00000029465	131	2	6	2	2	2	2	1	2	4	2	0	3	SNORA68
ENSXETG00000029467	164	0	0	0	0	0	0	0	0	0	0	0	0	
ENSXETG00000029469	167	0	0	0	0	0	0	0	0	0	0	0	0	
ENSXETG00000029470	186	0	0	0	0	0	0	0	0	0	0	0	0	
ENSXETG00000029471	186	0	0	0	0	0	0	0	0	0	0	0	0	
ENSXETG00000029472	184	0	0	0	0	0	0	0	0	0	0	0	0	
ENSXETG00000029473	130	32	51	32	16	14	6	27	35	21	36	19	25	SNORA61
ENSXETG00000029474	134	1	1	4	3	2	1	6	5	0	6	2	1	SNORA9
ENSXETG00000029477	86	0	0	0	0	0	0	0	0	0	0	0	0	
ENSXETG00000029478	66	0	0	0	0	0	0	0	0	0	0	0	0	
ENSXETG00000029479	158	0	0	0	0	0	0	0	0	0	0	0	0	
ENSXETG00000029480	181	0	0	0	0	0	0	0	0	0	0	0	0	
ENSXETG00000029482	73	0	0	2	1	0	0	0	0	0	0	0	1	
ENSXETG00000029483	158	0	0	0	0	0	0	0	0	0	0	0	0	
ENSXETG00000029484	145	0	0	0	0	0	0	0	0	0	0	0	0	
ENSXETG00000029485	86	0	0	0	0	0	0	0	0	0	0	0	0	
ENSXETG00000029486	176	0	0	0	0	0	0	0	0	0	0	0	0	
ENSXETG00000029487	175	0	0	0	0	0	0	0	0	0	0	0	0	
ENSXETG00000029488	83	0	0	0	0	0	0	0	0	0	0	1	0	U2
ENSXETG00000029490	146	0	0	0	0	0	0	0	0	0	0	0	0	
ENSXETG00000029493	83	0	0	0	0	0	0	0	0	0	0	0	0	
ENSXETG00000029494	179	0	0	0	0	0	0	0	0	0	0	0	0	
ENSXETG00000029496	179	0	0	0	0	0	0	0	0	0	0	0	0	
ENSXETG00000029498	117	0	0	0	0	0	0	0	0	0	0	0	0	
ENSXETG00000029500	148	0	0	0	0	0	0	0	0	0	0	0	0	
ENSXETG00000029503	158	0	0	0	0	0	0	0	0	0	0	0	0	U4
ENSXETG00000029504	149	0	0	0	0	0	0	0	0	0	0	0	0	
ENSXETG00000029506	146	0	0	0	0	0	0	0	0	0	0	0	0	
ENSXETG00000029507	105	0	0	0	0	0	0	0	0	0	0	0	0	
ENSXETG00000029508	181	0	0	0	0	0	0	0	0	0	0	0	0	
ENSXETG00000029509	185	0	0	0	0	0	0	0	0	0	0	0	0	
ENSXETG00000029510	156	0	0	0	0	0	0	0	0	0	0	0	0	
ENSXETG00000029512	158	0	0	0	0	0	0	0	0	0	0	0	0	
ENSXETG00000029516	189	0	0	0	0	0	0	0	0	0	0	0	0	
ENSXETG00000029517	147	0	0	0	0	0	0	0	0	0	0	0	0	
ENSXETG00000029518	158	0	0	0	0	0	0	1	0	0	0	0	0	
ENSXETG00000029519	135	0	0	0	0	0	0	0	0	0	0	0	0	
ENSXETG00000029520	78	0	0	0	0	0	0	0	0	0	0	0	0	
ENSXETG00000029521	157	0	0	0	0	0	0	0	0	0	0	0	0	
ENSXETG00000029523	152	0	0	0	0	0	0	0	0	0	0	0	0	
ENSXETG00000029524	149	0	0	0	0	0	0	0	0	0	0	0	0	
ENSXETG00000029527	133	0	0	0	1	1	0	0	2	2	0	1	0	SNORA66
ENSXETG00000029528	147	0	0	0	0	0	0	0	0	0	0	0	0	
ENSXETG00000029529	148	0	0	0	0	0	0	0	0	0	0	0	0	
ENSXETG00000029531	147	0	0	0	0	0	0	0	0	0	0	0	0	
ENSXETG00000029535	133	0	0	0	0	0	0	0	0	0	0	0	0	
ENSXETG00000029536	158	0	0	0	0	0	0	0	0	0	0	0	0	
ENSXETG00000029537	167	0	0	0	0	0	0	0	0	0	0	0	0	
ENSXETG00000029539	201	6	1	2	1	1	0	1	1	0	2	3	1	SNORA74
ENSXETG00000029540	185	0	0	0	0	0	0	0	0	0	0	0	0	
ENSXETG00000029541	78	3	2	1	1	0	7	1	4	2	2	0	5	SNORD72
ENSXETG00000029542	190	0	0	0	0	0	0	0	0	0	0	0	0	
ENSXETG00000029544	107	23	31	34	14	13	7	16	24	15	17	13	10	snoU83B
ENSXETG00000029545	146	0	0	0	0	0	0	0	0	0	0	0	0	
ENSXETG00000029546	191	0	0	0	0	0	0	0	0	0	0	0	0	
ENSXETG00000029547	82	0	0	0	0	0	0	0	0	0	0	0	0	
ENSXETG00000029549	132	0	0	0	0	0	0	0	0	0	0	0	0	
ENSXETG00000029552	192	0	0	0	0	0	0	0	0	0	0	0	0	
ENSXETG00000029553	158	0	0	0	0	0	0	0	0	0	0	0	0	
ENSXETG00000029554	135	4	5	3	5	2	1	6	9	8	11	6	6	SNORA2
ENSXETG00000029555	127	3	3	1	0	2	0	1	2	1	2	0	1	SNORA65
ENSXETG00000029556	145	0	0	0	0	0	0	0	0	0	0	0	0	
ENSXETG00000029557	311	0	0	0	0	0	0	0	0	0	0	0	0	
ENSXETG00000029558	147	0	0	0	0	0	0	0	0	0	0	0	0	
ENSXETG00000029559	97	0	0	0	0	0	0	0	0	0	0	0	0	
ENSXETG00000029560	196	0	0	0	0	0	0	0	0	0	0	0	0	
ENSXETG00000029562	158	0	0	0	0	0	0	0	0	0	0	0	0	
ENSXETG00000029564	176	0	0	0	0	0	0	0	0	0	0	0	0	
ENSXETG00000029566	166	0	0	0	0	0	0	0	0	0	0	0	0	
ENSXETG00000029568	135	9	9	6	3	3	2	2	3	4	2	2	0	SNORA42
ENSXETG00000029570	134	0	0	0	0	1	0	0	0	0	0	0	0	
ENSXETG00000029576	114	0	0	0	0	0	0	0	0	0	0	0	0	
ENSXETG00000029577	88	0	0	0	0	0	0	0	0	0	0	0	0	
ENSXETG00000029578	141	12	13	16	12	19	20	20	23	12	19	10	16	SNORD10

ENSXETG00000029579	96	0	0	0	0	0	0	0	0	0	0	0	0	
ENSXETG00000029580	117	0	0	0	0	0	0	0	0	0	0	0	0	
ENSXETG00000029581	137	6	4	7	3	3	5	4	8	6	10	11	1	SNORA16
ENSXETG00000029587	113	0	0	0	0	0	0	0	0	0	0	0	0	
ENSXETG00000029588	121	0	1	0	0	0	0	0	1	0	0	0	0	SNORA54
ENSXETG00000029589	73	1	0	0	0	0	0	0	0	0	0	0	0	
ENSXETG00000029592	103	0	0	0	0	0	0	0	0	0	0	0	0	
ENSXETG00000029594	96	0	0	0	0	0	0	0	0	0	0	0	0	
ENSXETG00000029595	136	0	0	0	1	3	0	0	0	0	11	4	5	U2
ENSXETG00000029596	129	18	22	19	5	6	3	19	25	13	4	3	6	SNORA44
ENSXETG00000029598	114	0	0	0	0	0	0	0	0	0	0	0	0	
ENSXETG00000029599	205	1	3	0	1	1	1	0	0	0	2	0	1	SNORA74
ENSXETG00000029600	158	0	0	0	0	0	0	0	0	0	0	0	0	
ENSXETG00000029601	158	0	0	0	0	0	0	0	0	0	0	0	0	
ENSXETG00000029604	203	0	0	0	0	0	0	0	1	0	1	0	0	
ENSXETG00000029605	132	197	277	244	126	88	95	195	261	131	131	99	130	SNORA35
ENSXETG00000029606	133	1	12	3	7	8	1	5	5	2	13	6	9	SCARNA14
ENSXETG00000029607	115	0	0	0	0	0	0	0	0	0	0	0	0	
ENSXETG00000029609	123	0	0	0	0	1	0	0	0	0	0	0	2	ENSXETG00000029609
ENSXETG00000029610	107	0	0	0	0	0	0	0	0	0	0	0	0	
ENSXETG00000029611	137	0	1	0	0	0	0	2	1	1	1	0	3	SNORA75
ENSXETG00000029612	156	0	0	0	0	0	0	0	0	0	0	0	0	
ENSXETG00000029613	105	0	0	0	0	0	0	0	0	0	0	0	0	
ENSXETG00000029614	103	0	0	0	0	0	0	0	0	0	0	0	0	
ENSXETG00000029615	80	0	0	0	0	0	0	0	0	0	0	0	0	
ENSXETG00000029616	104	0	0	0	0	0	0	0	0	0	0	0	0	
ENSXETG00000029617	70	2	0	2	0	0	0	0	1	5	1	1	1	SNORD2
ENSXETG00000029621	183	0	0	0	0	0	0	0	0	0	0	0	0	
ENSXETG00000029623	136	0	0	0	0	0	0	0	0	0	0	0	0	
ENSXETG00000029624	151	0	0	0	0	0	0	0	0	0	0	0	0	
ENSXETG00000029625	135	19	23	16	9	6	6	8	14	7	9	7	7	SNORA27
ENSXETG00000029626	135	5	3	3	0	1	0	1	2	0	3	3	2	SNORA5
ENSXETG00000029628	129	6	6	5	1	2	3	2	5	3	0	2	1	SNORA41
ENSXETG00000029631	96	0	0	0	0	0	0	0	0	0	0	0	0	
ENSXETG00000029632	104	0	0	0	0	0	0	0	0	0	0	0	0	
ENSXETG00000029633	115	0	0	0	0	0	0	0	0	0	0	0	0	
ENSXETG00000029637	119	0	0	0	0	0	1	0	0	0	1	0	0	
ENSXETG00000029638	156	0	0	0	0	0	0	0	0	0	0	0	0	
ENSXETG00000029639	178	0	0	0	0	0	0	0	0	0	0	0	0	
ENSXETG00000029642	214	0	0	0	0	0	0	0	0	0	0	0	0	
ENSXETG00000029643	198	0	0	0	0	0	0	0	0	0	0	0	0	
ENSXETG00000029644	141	0	0	0	0	0	0	0	0	0	0	0	0	
ENSXETG00000029645	141	0	0	0	0	0	0	0	0	0	0	0	0	snoU109
ENSXETG00000029646	115	0	0	0	0	0	0	0	0	0	0	0	0	
ENSXETG00000029649	102	0	0	0	0	0	0	0	0	0	0	0	0	
ENSXETG00000029650	90	0	0	0	0	0	0	0	0	0	0	0	0	
ENSXETG00000029651	138	2	1	3	2	0	2	1	0	0	2	0	1	SNORA29
ENSXETG00000029652	119	0	0	0	0	0	0	0	0	0	0	0	0	
ENSXETG00000029653	137	0	0	0	1	0	0	0	1	0	0	0	0	
ENSXETG00000029654	96	0	0	0	0	0	0	0	0	0	0	0	0	
ENSXETG00000029655	129	3	5	4	4	0	1	0	2	0	3	1	3	SNORA44
ENSXETG00000029658	97	0	0	0	0	0	0	0	0	0	0	0	0	
ENSXETG00000029662	83	0	0	0	0	0	0	0	0	0	0	0	0	
ENSXETG00000029663	125	0	0	0	0	0	0	0	0	0	0	0	0	
ENSXETG00000029664	167	0	0	0	0	0	0	0	0	0	0	0	0	
ENSXETG00000029665	262	0	0	0	0	0	0	0	0	0	0	0	0	
ENSXETG00000029666	126	0	0	0	0	0	0	1	0	1	0	0	0	
ENSXETG00000029667	83	0	0	0	0	0	0	0	0	0	0	0	0	
ENSXETG00000029668	158	0	1	0	3	0	0	7	6	4	0	0	1	U4
ENSXETG00000029669	110	0	0	0	0	0	0	0	0	0	0	0	0	
ENSXETG00000029670	166	1	0	4	1	2	5	1	5	5	10	4	3	SNORD97
ENSXETG00000029671	131	2	5	0	2	1	1	2	1	1	3	4	3	SCARNA15
ENSXETG00000029673	132	0	0	0	0	0	0	0	0	0	0	1	0	
ENSXETG00000029674	214	0	0	0	0	0	0	0	0	0	0	0	0	
ENSXETG00000029675	129	0	0	0	1	2	0	0	0	0	0	0	2	
ENSXETG00000029676	206	0	0	0	0	0	0	0	0	0	0	0	0	
ENSXETG00000029677	88	0	0	1	1	0	0	0	0	0	1	1	0	SNORD127
ENSXETG00000029678	152	1	0	0	0	0	0	1	0	0	0	0	0	U4
ENSXETG00000029679	150	0	0	0	0	0	0	0	1	0	0	0	0	
ENSXETG00000029680	176	8	3	6	2	2	1	5	5	1	4	2	8	SNORA81
ENSXETG00000029682	156	0	0	0	0	0	0	0	0	0	0	0	0	
ENSXETG00000029683	94	0	0	0	0	0	0	0	0	0	0	0	0	
ENSXETG00000029684	211	0	0	0	0	0	0	0	0	0	0	0	0	
ENSXETG00000029685	97	0	0	0	0	0	0	0	0	0	0	0	0	
ENSXETG00000029686	181	0	0	0	0	0	0	0	0	0	0	0	0	
ENSXETG00000029687	86	0	0	0	0	0	0	0	0	0	0	0	0	
ENSXETG00000029688	119	5	5	4	4	1	2	3	2	4	5	1	1	SNORA36
ENSXETG00000029689	78	0	0	0	0	0	0	0	0	0	0	0	0	
ENSXETG00000029691	134	1	0	0	0	0	0	0	0	0	0	0	0	
ENSXETG00000029692	158	0	0	0	0	0	0	0	0	0	0	0	0	
ENSXETG00000029693	125	0	0	0	0	0	0	0	0	0	0	0	0	
ENSXETG00000029694	96	0	0	0	0	0	0	0	0	0	0	0	0	
ENSXETG00000029695	121	0	0	0	0	0	0	0	0	0	0	0	0	
ENSXETG00000029696	116	20	39	22	6	8	7	10	16	9	11	5	11	SNORA32
ENSXETG00000029697	174	0	0	0	0	0	0	0	0	0	0	0	0	
ENSXETG00000029698	312	0	0	0	0	0	0	0	0	0	0	0	0	
ENSXETG00000029699	89	0	0	0	0	0	0	0	0	0	0	0	0	
ENSXETG00000029700	297	0	1	0	0	0	1	0	0	0	1	1	0	
ENSXETG00000029701	84	0	0	0	0	0	0	0	0	0	0	0	0	
ENSXETG00000029703	178	4	10	10	1	6	1	7	14	8	19	12	14	SCARNA16
ENSXETG00000029704	99	0	0	0	0	0	0	0	0	0	0	0	0	
ENSXETG00000029705	133	0	0	0	0	0	0	0	0	0	0	0	0	
ENSXETG00000029706	73	0	0	0	0	0	0	0	0	0	0	0	0	
ENSXETG00000029707	130	0	0	0	0	0	0	0	0	0	0	0	0	
ENSXETG00000029710	111	0	0	0	0	0	0	0	0	0	0	1	0	
ENSXETG00000029711	122	0	0	0	0	0	0	0	0	0	0	0	0	
ENSXETG00000029714	82	0	0	0	0	0	0	1	2	2	2	3	4	
ENSXETG00000029715	81	0	2	0	1	0	0	0	0	1	4	1	1	SNORD24
ENSXETG00000029719	202	9	5	5	4	3	2	1	1	4	1	0	9	SNORA74
ENSXETG00000029720	152	0	0	0	0	0	0	0	0	0	0	0	0	
ENSXETG00000029721	148	0	0	0	1	0	0	2	4	1	0	0	0	
ENSXETG00000029722	35	0	0	0	0	0	0	0	0	0	0	0	0	
ENSXETG00000029723	125	17	36	14	11	9	9	18	20	14	25	20	23	SNORA65
ENSXETG00000029724	118	0	0	0	0	0	0	0	0	0	0	0	0	
ENSXETG00000029725	135	41	53	43	37	30	13	20	22	15	36	22	17	SNORA51
ENSXETG00000029726	158	0	0	0	0	0	0	0	0	0	0	0	0	
ENSXETG00000029727	141	10	7	17	10	3	9	8	12	14	13	11	10	SNORA64
ENSXETG00000029728	69	7	16	8	7	5	0	8	12	4	5	3	5	snoU25
ENSXETG00000029729	167	0	0	0	0	0	0	0	0	0	0	0	0	
ENSXETG00000029730	155	0	0	0	0	0	0	0	0	0	0	0	0	
ENSXETG00000029731	214	0	0	0	0	0	0	0	0	0	0	0	0	

ENSXETG00000029732	95	0	0	0	0	0	0	0	0	0	0	0	0	
ENSXETG00000029733	175	0	0	0	0	0	0	0	0	0	0	0	0	
ENSXETG00000029734	132	0	0	0	0	0	0	0	0	0	0	0	0	
ENSXETG00000029735	278	0	0	0	2	0	1	0	0	0	4	0	1	SCARNA6
ENSXETG00000029736	159	0	0	0	0	0	0	0	0	0	0	0	0	
ENSXETG00000029738	127	4	3	6	4	4	2	6	4	0	3	1	1	SNORA65
ENSXETG00000029739	136	0	0	0	1	0	0	0	0	0	0	0	1	snoU109
ENSXETG00000029740	100	1	0	0	0	0	0	0	0	0	1	1	0	
ENSXETG00000029743	119	0	0	0	0	0	0	0	0	0	0	0	0	
ENSXETG00000029744	135	10	12	13	11	5	3	4	7	5	6	2	3	SNORA16
ENSXETG00000029745	116	3	4	0	4	3	1	7	3	4	4	1	0	SNORD89
ENSXETG00000029746	125	18	36	38	11	14	7	17	20	8	13	9	11	SNORA18
ENSXETG00000029747	116	0	0	0	0	0	0	0	0	0	0	0	0	
ENSXETG00000029748	132	0	0	0	0	0	0	0	0	0	0	0	0	
ENSXETG00000029749	85	0	0	0	0	0	0	0	0	0	0	0	0	
ENSXETG00000029750	122	0	0	0	0	0	1	0	0	0	0	0	0	SNORA28
ENSXETG00000029751	159	0	0	0	0	0	0	0	0	0	0	0	0	
ENSXETG00000029752	205	0	0	0	0	0	0	0	0	0	0	0	0	
ENSXETG00000029753	177	0	0	0	0	0	0	0	0	0	0	0	0	
ENSXETG00000029754	123	0	0	0	0	0	0	0	0	0	0	0	0	ENSXETG00000029754
ENSXETG00000029755	107	0	0	0	0	0	0	0	0	0	0	0	0	
ENSXETG00000029757	136	11	14	15	3	7	1	3	4	5	14	6	8	SNORA5
ENSXETG00000029759	158	0	0	0	0	0	0	0	0	0	0	0	0	
ENSXETG00000029760	74	4	6	6	6	5	6	4	9	3	13	5	12	SNORD24
ENSXETG00000029761	137	1	8	3	0	0	0	1	4	2	1	3	4	SNORA16
ENSXETG00000029763	127	18	17	22	5	4	3	12	15	8	15	10	7	SNORA25
ENSXETG00000029766	79	1	1	3	0	0	1	2	1	1	1	0	3	SNORD79
ENSXETG00000029768	133	0	0	0	0	0	0	0	0	0	0	0	0	
ENSXETG00000029769	131	1	0	2	0	1	3	1	4	1	0	1	1	SCARNA8
ENSXETG00000029770	86	0	0	0	0	0	0	0	0	0	0	0	0	
ENSXETG00000029771	189	0	0	0	0	0	0	0	0	0	0	0	0	
ENSXETG00000029772	96	0	0	0	0	0	0	0	0	0	0	0	0	
ENSXETG00000029774	153	0	0	0	1	4	5	0	0	0	2	2	5	U4
ENSXETG00000029775	134	8	12	6	2	3	4	9	8	3	7	0	2	SNORA70
ENSXETG00000029776	133	18	18	15	2	6	3	8	12	11	10	9	12	SNORA71
ENSXETG00000029778	126	0	0	0	0	0	0	0	0	0	0	0	0	
ENSXETG00000029779	97	0	0	0	0	0	0	0	0	0	0	0	0	
ENSXETG00000029780	118	0	0	0	0	0	0	0	0	0	0	0	0	
ENSXETG00000029782	99	0	0	0	0	0	0	0	0	0	0	0	0	
ENSXETG00000029783	152	0	0	0	0	0	0	0	0	0	0	0	0	
ENSXETG00000029784	52	0	0	0	0	0	0	0	0	0	0	0	0	
ENSXETG00000029785	97	0	0	0	0	0	0	0	0	0	0	0	0	
ENSXETG00000029788	127	1	0	1	1	0	0	0	1	0	0	1	0	SNORA19
ENSXETG00000029789	158	0	0	0	0	0	0	0	0	0	0	0	0	
ENSXETG00000029791	158	1	0	0	0	0	2	3	4	2	1	1	1	U4
ENSXETG00000029792	312	0	0	0	0	0	0	0	0	0	0	0	0	
ENSXETG00000029793	72	4	0	0	0	0	0	1	1	0	1	0	0	SNORD2
ENSXETG00000029794	158	0	0	0	0	0	0	0	0	0	0	0	0	
ENSXETG00000029795	96	0	0	0	0	0	0	0	0	0	0	0	0	
ENSXETG00000029796	72	0	0	0	0	0	0	0	0	0	0	0	0	
ENSXETG00000029797	81	14	36	25	8	6	5	12	17	11	21	17	11	SNORA56
ENSXETG00000029798	158	0	0	0	0	0	0	0	0	0	0	0	0	
ENSXETG00000029799	159	0	0	0	0	0	0	0	0	0	0	0	0	
ENSXETG00000029800	129	1	0	0	0	0	0	1	0	0	0	0	0	
ENSXETG00000029802	174	0	0	0	0	0	0	0	0	0	0	0	0	
ENSXETG00000029804	165	0	0	0	0	0	0	0	0	0	0	0	0	
ENSXETG00000029805	130	16	24	4	2	4	5	14	15	13	17	9	8	SNORA27
ENSXETG00000029807	137	0	1	0	1	0	1	0	0	0	3	2	2	SCARNA11
ENSXETG00000029808	164	0	0	0	0	0	0	0	0	0	0	0	0	
ENSXETG00000029809	157	0	0	0	0	0	0	0	0	0	0	0	0	
ENSXETG00000029813	144	6	5	12	2	0	2	3	7	3	2	2	5	SNORA79
ENSXETG00000029815	154	0	0	0	0	0	0	0	0	0	0	0	0	
ENSXETG00000029817	112	0	0	0	0	0	0	0	0	0	0	0	0	
ENSXETG00000029819	84	0	0	0	0	0	0	0	0	0	0	0	0	
ENSXETG00000029825	155	0	0	0	0	0	0	0	0	1	0	0	0	
ENSXETG00000029826	111	0	0	0	0	0	0	0	0	0	0	0	0	
ENSXETG00000029828	178	1	1	3	0	1	0	0	1	0	1	0	0	SCARNA16
ENSXETG00000029829	119	0	0	0	0	0	0	0	0	0	0	0	0	
ENSXETG00000029831	128	2	11	5	1	2	9	3	4	1	4	1	1	SNORA40
ENSXETG00000029832	203	0	0	1	0	0	1	0	0	0	0	1	0	U2
ENSXETG00000029834	82	0	0	0	0	0	0	0	0	0	0	0	0	
ENSXETG00000029835	136	0	0	0	0	0	0	0	0	0	0	0	0	
ENSXETG00000029836	98	0	0	0	0	0	0	0	0	0	0	0	0	
ENSXETG00000029837	158	0	0	0	0	0	0	0	0	0	0	0	0	
ENSXETG00000029839	302	0	3	3	2	1	2	1	1	0	3	2	1	SCARNA7
ENSXETG00000029840	156	0	0	0	0	0	0	0	0	0	0	0	0	
ENSXETG00000029841	136	0	0	0	0	0	0	0	0	0	0	0	0	
ENSXETG00000029843	158	0	0	0	0	0	0	0	0	0	0	0	0	
ENSXETG00000029846	142	0	0	0	0	0	0	1	0	0	0	1	0	U4
ENSXETG00000029847	124	0	0	0	0	0	0	0	0	0	0	0	0	
ENSXETG00000029849	98	1	1	0	0	0	0	0	0	0	0	0	0	
ENSXETG00000029850	113	0	0	0	0	0	0	1	0	1	0	0	0	
ENSXETG00000029851	136	0	0	0	0	1	0	0	0	0	0	0	0	
ENSXETG00000029852	72	0	0	0	0	0	0	0	0	0	0	0	0	
ENSXETG00000029854	310	1	1	0	0	0	2	0	0	1	0	0	1	snoU85
ENSXETG00000029856	83	0	0	0	0	0	0	0	1	0	0	0	0	
ENSXETG00000029857	101	0	0	0	0	0	0	0	0	0	0	0	0	
ENSXETG00000029858	119	0	0	0	0	0	0	0	0	0	0	0	0	
ENSXETG00000029860	135	0	0	1	0	0	1	0	0	0	0	0	0	
ENSXETG00000029861	136	0	0	0	0	0	0	0	0	0	0	0	0	
ENSXETG00000029862	57	0	0	0	0	0	0	0	0	0	0	0	0	
ENSXETG00000029864	163	0	0	0	0	0	0	0	0	0	0	0	0	
ENSXETG00000029865	158	0	0	0	0	0	0	0	0	0	0	0	0	
ENSXETG00000029866	129	9	24	12	3	2	2	14	12	8	4	2	0	ACA64
ENSXETG00000029867	84	0	0	0	0	0	0	0	0	0	0	0	0	
ENSXETG00000029868	74	0	0	0	0	0	0	0	0	0	0	0	0	
ENSXETG00000029869	114	0	0	0	0	0	0	0	0	0	0	0	0	
ENSXETG00000029870	123	0	0	0	0	0	0	0	0	0	0	0	0	
ENSXETG00000029871	130	1	1	0	0	1	1	1	3	1	1	1	3	SCARNA4
ENSXETG00000029872	96	0	0	0	0	0	0	0	0	0	0	0	0	
ENSXETG00000029873	159	0	0	0	0	0	0	1	0	0	0	0	0	
ENSXETG00000029875	413	15	18	24	11	11	37	18	15	11	31	8	10	RNaseP_nuc
ENSXETG00000029878	57	0	0	0	0	0	0	0	0	0	0	0	0	
ENSXETG00000029879	173	0	0	0	0	0	0	0	0	0	0	0	0	
ENSXETG00000029880	137	38	71	60	49	35	11	43	37	33	24	19	17	SNORA57
ENSXETG00000029882	90	0	0	0	0	0	0	0	0	0	0	0	0	
ENSXETG00000029883	78	0	0	0	0	0	0	0	0	0	0	0	0	
ENSXETG00000029884	135	20	34	21	9	5	2	15	7	8	10	8	9	SNORA17
ENSXETG00000029886	269	0	1	0	1	1	0	2	0	0	0	0	1	SCARNA6
ENSXETG00000029889	186	0	0	0	0	0	0	0	0	0	0	0	0	
ENSXETG00000029891	80	73	89	95	20	15	15	33	39	25	54	39	29	SNORD28

ENSXETG00000029894	109	0	0	0	0	0	0	0	0	0	0	0	1	
ENSXETG00000029895	307	0	0	0	0	0	0	0	0	0	0	0	0	
ENSXETG00000029896	135	2	11	5	2	4	3	21	9	10	10	3	8	SNORA16
ENSXETG00000029898	158	0	0	0	0	0	0	0	0	0	0	0	0	
ENSXETG00000029899	214	0	0	0	0	0	0	0	0	0	0	0	0	
ENSXETG00000029903	104	0	0	0	0	0	0	0	0	0	0	0	0	
ENSXETG00000029905	78	0	0	0	0	0	0	0	0	0	0	0	0	
ENSXETG00000029906	176	5	8	4	16	13	12	36	14	15	74	41	49	SNORD97
ENSXETG00000029907	115	0	0	0	0	0	0	0	0	0	0	0	0	
ENSXETG00000029908	131	14	27	15	7	5	25	20	20	15	15	12	11	SNORA31
ENSXETG00000029909	159	0	0	0	0	0	0	0	0	0	0	0	0	
ENSXETG00000029910	101	0	0	0	0	0	0	0	0	0	0	0	0	
ENSXETG00000029911	167	0	0	0	0	0	0	0	0	0	0	0	0	
ENSXETG00000029912	100	0	0	0	0	0	0	0	0	0	0	0	0	
ENSXETG00000029913	197	0	0	0	0	0	0	0	0	0	0	0	0	
ENSXETG00000029914	158	0	0	0	0	0	0	0	0	0	0	0	0	
ENSXETG00000029915	109	0	0	0	0	0	0	0	0	0	0	0	0	
ENSXETG00000029916	115	0	0	0	0	0	0	0	0	0	0	0	0	
ENSXETG00000029917	3342	362	524	458	759	518	1404	933	1076	609	1311	927	1003	HLTF
ENSXETG00000029918	642	0	2	1	5	4	2	7	4	3	7	7	10	BARX2
ENSXETG00000029919	252	1	3	1	1	0	7	86	116	58	40	16	15	ENSXETG00000029919
ENSXETG00000029920	3435	0	1	0	3	3	3	0	1	0	8	4	3	GRIN3A
ENSXETG00000029921	1998	260	379	333	181	132	281	252	333	162	425	324	346	trmt10c
ENSXETG00000029922	792	0	0	0	0	0	0	0	0	0	0	0	0	ENSXETG00000029922
ENSXETG00000029923	3585	11	7	19	113	93	141	662	731	415	209	170	176	EVC2
ENSXETG00000029924	3195	87	126	113	116	88	409	460	555	315	329	226	244	STXBP5
ENSXETG00000029925	1305	165	282	219	312	238	358	363	354	272	349	262	286	ENSXETG00000029925
ENSXETG00000029926	393	0	0	0	0	0	0	0	0	0	0	0	0	hist2h2ab
ENSXETG00000029927	2493	0	0	0	0	0	0	0	0	0	0	1	1	ENSXETG00000029927
ENSXETG00000029928	870	26	33	27	382	369	527	1564	1693	1070	747	471	483	gas1
ENSXETG00000029929	501	0	0	3	0	0	0	5	4	0	0	0	1	ENSXETG00000029929
ENSXETG00000029930	408	327	457	425	267	166	176	356	465	233	194	133	182	ENSXETG00000029930
ENSXETG00000029931	3510	24	26	27	37	31	28	199	235	123	140	130	143	ENSXETG00000029931
ENSXETG00000029932	2431	187	302	256	389	277	420	619	848	435	328	246	250	cln6
ENSXETG00000029933	1344	62	78	77	348	278	325	113	121	66	282	235	243	ENSXETG00000029933
ENSXETG00000029934	2181	4	5	4	7	13	32	27	53	27	73	54	57	MYBL1
ENSXETG00000029935	1225	289	457	377	303	237	293	441	477	290	283	180	229	DUSP11
ENSXETG00000029936	1116	0	0	0	0	0	0	0	1	0	0	1	0	
ENSXETG00000029937	1275	1337	2123	1765	2361	1775	2622	3811	4437	2579	1558	1119	1203	PSMD11
ENSXETG00000029938	195	0	0	0	0	0	0	0	0	0	0	0	0	ENSXETG00000029938
ENSXETG00000029939	2259	643	898	824	1382	1012	1616	975	1097	647	1184	852	919	MUT
ENSXETG00000029940	990	0	0	0	0	0	0	0	0	0	0	0	0	
ENSXETG00000029941	1753	106	133	127	283	236	361	634	781	393	1032	758	848	ENSXETG00000029941
ENSXETG00000029942	3697	10	7	2	7	1	7	15	11	7	25	17	13	TDRD5
ENSXETG00000029943	119	0	0	0	0	0	0	0	0	0	0	0	0	
ENSXETG00000029944	522	4	2	0	13	5	17	18	19	18	9	12	13	ENSXETG00000029944
ENSXETG00000029945	612	222	310	280	321	263	418	293	309	176	422	316	308	C1orf53
ENSXETG00000029946	2564	79	124	107	573	405	131	186	264	143	572	384	479	SYT16
ENSXETG00000029947	2401	124	176	118	398	289	716	537	602	325	1602	1100	1183	POLI
ENSXETG00000029948	2243	75	102	84	281	181	468	408	451	282	332	262	250	ENSXETG00000029948
ENSXETG00000029949	510	95	147	120	164	116	144	187	196	79	190	106	138	ENSXETG00000029949
ENSXETG00000029950	678	0	0	0	0	1	0	0	0	0	0	0	0	ENSXETG00000029950
ENSXETG00000029951	1038	0	0	0	0	0	0	0	0	0	1	0	0	
ENSXETG00000029952	499	0	0	0	0	0	0	0	0	0	0	0	0	ENSXETG00000029952
ENSXETG00000029953	501	0	0	0	0	0	0	0	0	0	0	0	0	
ENSXETG00000029954	2492	0	0	1	4	0	1	1	5	1	1	0	1	ENSXETG00000029954
ENSXETG00000029955	1836	315	435	369	505	357	825	777	898	549	802	544	604	secisbp2
ENSXETG00000029956	489	47	76	98	76	40	229	44	43	46	114	76	103	ENSXETG00000029956
ENSXETG00000029957	1617	29	58	48	221	160	741	1189	1439	879	235	164	178	bcl2l11
ENSXETG00000029958	1578	123	179	143	254	210	392	543	663	398	350	232	254	FAM126B
ENSXETG00000029959	2028	66	117	114	286	185	766	1019	1204	663	791	574	609	SUN2
ENSXETG00000029960	578	14	25	28	35	25	51	67	96	57	47	36	48	C8G
ENSXETG00000029961	468	424	599	571	489	433	656	681	798	523	345	218	281	ENSXETG00000029961
ENSXETG00000029962	5372	1	0	0	0	0	0	0	1	0	1	1	0	ENSXETG00000029962
ENSXETG00000029963	2766	549	768	706	1110	890	1618	1171	1369	795	1209	772	858	WWP1
ENSXETG00000029964	849	3	4	2	8	4	11	323	396	186	39	28	28	ENSXETG00000029964
ENSXETG00000029965	927	0	0	0	0	0	0	0	0	0	0	0	0	
ENSXETG00000029966	513	0	0	0	0	0	0	0	0	0	0	0	0	
ENSXETG00000029967	900	0	0	0	0	0	0	0	0	0	0	0	0	ENSXETG00000029967
ENSXETG00000029968	5699	6	8	6	59	46	81	597	616	342	312	237	282	KIF26B
ENSXETG00000029969	1029	0	2	2	1	0	0	0	1	0	3	0	2	mchr2
ENSXETG00000029970	387	0	0	0	0	0	0	1	0	0	0	0	0	
ENSXETG00000029971	9984	0	1	1	1	6	4	151	172	107	2	1	3	DPF1
ENSXETG00000029972	585	0	0	0	0	0	0	0	0	0	0	0	0	
ENSXETG00000029973	3558	60	102	91	60	46	32	218	226	172	96	62	78	STXBP5L
ENSXETG00000029974	588	93	122	120	229	170	437	426	446	311	214	146	183	ENSXETG00000029974
ENSXETG00000029975	1044	25	37	33	71	50	187	137	113	92	207	134	133	ENSXETG00000029975
ENSXETG00000029976	2469	0	0	0	0	0	0	0	0	0	0	0	0	ENSXETG00000029976
ENSXETG00000029977	2448	0	0	0	0	0	0	0	0	0	0	0	0	
ENSXETG00000029978	930	0	0	0	0	0	0	0	0	0	0	0	0	
ENSXETG00000029979	164	0	0	0	0	0	0	0	0	0	0	0	0	
ENSXETG00000029980	1181	180	253	200	371	292	567	415	451	243	527	340	414	ENSXETG00000029980
ENSXETG00000029981	1179	0	0	0	0	0	0	1	0	0	1	1	0	
ENSXETG00000029982	666	0	0	0	0	0	0	0	0	0	0	0	0	
ENSXETG00000029983	3007	2	1	7	8	6	4	342	451	230	19	10	13	ENSXETG00000029983
ENSXETG00000029984	1470	11	24	15	8	13	23	169	215	90	29	15	17	ENSXETG00000029984
ENSXETG00000029985	1284	0	0	0	1	3	0	0	2	0	15	8	9	ENSXETG00000029985
ENSXETG00000029986	2345	23	41	39	159	108	252	190	226	132	178	126	157	ENSXETG00000029986
ENSXETG00000029987	8730	368	494	438	953	730	956	888	1141	670	1447	998	1224	ZZEF1
ENSXETG00000029988	642	1	0	1	2	0	8	0	0	0	0	0	0	hba-l3
ENSXETG00000029989	981	0	0	0	0	0	0	0	0	0	0	0	0	
ENSXETG00000029990	495	0	0	0	0	0	0	0	0	0	0	0	0	
ENSXETG00000029991	1275	418	590	508	379	260	1501	602	690	422	892	612	725	ACP7
ENSXETG00000029992	324	1	0	0	0	0	0	1	6	2	0	0	1	ENSXETG00000029992
ENSXETG00000029993	1527	108	150	146	298	266	212	526	618	358	284	212	245	ARL6IP6
ENSXETG00000029994	276	8	9	3	0	0	1	4	9	4	11	3	7	C2orf88
ENSXETG00000029995	7555	1227	1911	1602	4858	3810	3175	4786	5313	3173	3595	2679	2823	sptbn1
ENSXETG00000029996	1392	0	0	0	0	0	2	1	0	1	4	1	2	ASB18
ENSXETG00000029997	957	5	6	2	5	14	4	5	3	1	8	7	9	HRH2
ENSXETG00000029998	572	0	0	0	0	0	0	0	0	0	1	0	0	ENSXETG00000029998
ENSXETG00000029999	2298	0	0	0	0	0	0	0	0	0	0	0	0	
ENSXETG00000030000	2541	0	0	0	0	0	0	0	0	0	0	0	0	
ENSXETG00000030001	1220	521	782	617	486	346	440	634	720	446	534	383	396	ENSXETG00000030001
ENSXETG00000030002	969	1	0	0	0	2	1	0	0	0	1	0	0	
ENSXETG00000030003	1428	2	0	3	100	93	6	5	5	3	16	16	18	ENSXETG00000030003
ENSXETG00000030004	351	1367	2055	1820	1730	1397	1566	1504	1647	1057	1148	856	901	ndufa1
ENSXETG00000030005	1833	0	0	0	1	0	0	19	21	13	1	4	1	ENSXETG00000030005
ENSXETG00000030006	90	0	0	0	0	0	0	0	0	0	0	0	0	
ENSXETG00000030007	1533	1	12	6	12	7	15	10	5	3	17	15	7	HSF4
ENSXETG00000030008	889	0	0	0	0	0	0	2	2	0	0	1	0	
ENSXETG00000030009	915	0	0	0	0	0	0	0	0	0	0	0	0	

ENSXETG00000030010	513	0	0	0	1	0	0	0	0	1	0	0	0	ENSXETG00000030010
ENSXETG00000030011	13253	1281	1911	1582	4423	3411	3381	5780	6299	3881	5605	4068	4548	HUWE1
ENSXETG00000030012	948	0	0	0	0	0	0	0	0	0	0	0	0	
ENSXETG00000030013	167	0	0	0	0	0	0	0	0	0	0	0	0	
ENSXETG00000030014	982	14	16	18	16	10	15	0	0	0	6	2	1	ENSXETG00000030014
ENSXETG00000030015	966	42	64	74	89	61	82	216	227	144	137	95	108	ENSXETG00000030015
ENSXETG00000030016	2688	15	18	18	37	34	43	66	56	27	44	20	27	ENSXETG00000030016
ENSXETG00000030017	119	0	0	0	0	0	0	0	0	0	0	0	0	
ENSXETG00000030018	582	0	0	1	0	0	1	1	1	0	1	3	0	ENSXETG00000030018
ENSXETG00000030019	2326	494	712	576	969	690	948	1565	1729	1029	1094	802	919	las1l
ENSXETG00000030020	119	0	0	0	0	0	0	0	0	0	0	0	0	
ENSXETG00000030021	1884	439	620	502	810	625	593	331	418	231	246	187	193	c14orf159
ENSXETG00000030022	1275	0	1	1	1	1	1	1	2	1	4	2	1	
ENSXETG00000030023	1143	0	0	0	0	0	1	0	0	0	0	0	0	
ENSXETG00000030024	1614	1	7	2	2	2	4	36	30	13	42	28	21	ENSXETG00000030024
ENSXETG00000030025	960	0	0	0	0	0	0	0	0	0	0	0	0	
ENSXETG00000030026	153	0	1	0	0	0	0	0	0	0	1	0	1	
ENSXETG00000030027	780	43	84	74	190	142	355	273	287	167	362	236	297	ENSXETG00000030027
ENSXETG00000030028	1653	0	0	0	1	1	1	10	6	4	3	5	0	ENSXETG00000030028
ENSXETG00000030029	12717	2	3	4	204	147	18	13	10	5	22	38	22	DNAH7
ENSXETG00000030030	882	2	1	0	3	10	2	22	23	11	5	5	3	KCTD16
ENSXETG00000030031	1197	4	4	2	9	12	6	1	2	1	4	4	5	ETV5
ENSXETG00000030032	933	0	4	1	2	2	2	23	11	7	3	0	0	OVOL3
ENSXETG00000030033	1329	12	17	16	21	15	31	453	533	310	170	140	131	ENSXETG00000030033
ENSXETG00000030034	354	2	5	4	34	30	7	5	6	5	13	3	4	ENSXETG00000030034
ENSXETG00000030035	2319	5	4	3	29	24	9	26	26	16	29	9	16	ABCB9
ENSXETG00000030036	1075	47	79	66	60	43	103	50	80	53	84	59	53	ENSXETG00000030036
ENSXETG00000030037	1104	3	3	3	6	4	1	2	2	3	10	8	17	ENSXETG00000030037
ENSXETG00000030038	2370	0	0	0	0	0	0	1	0	0	1	1	0	
ENSXETG00000030039	70	0	0	0	0	0	0	0	0	0	0	0	0	
ENSXETG00000030040	510	0	0	0	0	0	0	0	0	0	0	0	0	ENSXETG00000030040
ENSXETG00000030041	1982	1121	1547	1361	1127	799	1581	2261	2812	1604	733	539	578	HACD3
ENSXETG00000030042	1708	1	1	0	3	0	5	18	27	21	2	4	1	rab11fip4l
ENSXETG00000030043	2582	63	114	84	136	91	124	187	200	111	247	188	195	ENPP1
ENSXETG00000030044	1626	49	68	49	31	27	52	111	147	70	50	18	29	ENSXETG00000030044
ENSXETG00000030045	642	0	0	0	0	0	2	4	2	3	2	3	1	ENSXETG00000030045
ENSXETG00000030046	816	0	0	0	0	0	0	1	0	0	0	0	0	
ENSXETG00000030047	717	0	0	0	0	0	0	0	1	0	0	0	0	
ENSXETG00000030048	1080	0	0	0	0	0	0	0	0	0	0	0	0	
ENSXETG00000030049	2019	3	3	4	6	0	3	2	3	1	6	5	1	CNGA2
ENSXETG00000030050	1110	0	0	0	0	0	0	0	0	0	0	0	0	
ENSXETG00000030051	1002	0	0	0	0	0	0	0	0	0	0	0	0	
ENSXETG00000030052	3189	11	23	19	141	100	66	25	18	14	24	7	15	ENSXETG00000030052
ENSXETG00000030053	999	0	0	0	0	0	0	0	0	0	0	0	0	
ENSXETG00000030054	4215	311	405	406	755	563	1052	1379	1577	964	1183	840	909	SLK
ENSXETG00000030055	1349	412	567	414	365	293	512	395	557	302	451	298	375	btn1a1
ENSXETG00000030056	903	68	80	77	39	29	13	1206	1505	769	134	96	125	ENSXETG00000030056
ENSXETG00000030057	1835	0	0	0	0	0	0	0	0	0	0	0	0	
ENSXETG00000030058	1298	13	19	17	32	21	40	322	391	184	93	75	83	pip4k2b
ENSXETG00000030059	1290	2	3	6	4	5	18	128	151	66	47	40	36	ENSXETG00000030059
ENSXETG00000030060	978	0	0	0	0	0	0	0	0	0	0	0	0	ENSXETG00000030060
ENSXETG00000030061	1479	211	277	271	2898	2277	5166	128	125	89	365	244	278	ENSXETG00000030061
ENSXETG00000030062	1767	112	163	155	151	111	190	178	258	137	218	168	137	KLHL26
ENSXETG00000030063	1197	1	1	0	7	8	9	33	41	27	18	8	23	STK32B
ENSXETG00000030064	2298	24	49	41	83	79	147	111	158	85	191	156	160	RPS6KA5
ENSXETG00000030065	164	0	0	0	0	0	0	0	0	0	0	0	0	
ENSXETG00000030066	149	0	0	0	0	0	0	0	0	0	0	0	0	
ENSXETG00000030067	214	0	0	0	0	0	0	0	0	0	0	0	0	
ENSXETG00000030068	1185	0	0	0	0	0	0	0	0	0	0	0	0	
ENSXETG00000030069	936	195	307	220	186	129	228	386	460	288	123	106	100	TEX264
ENSXETG00000030070	1764	2	3	4	14	14	17	22	25	14	61	37	47	CUZD1
ENSXETG00000030071	3850	82	116	106	613	543	413	1504	1686	958	732	545	642	SLC12A2
ENSXETG00000030072	1254	126	205	156	321	263	463	487	489	303	443	322	350	MGC146454
ENSXETG00000030073	1398	19	8	15	18	14	26	233	254	160	75	42	52	ENSXETG00000030073
ENSXETG00000030074	119	0	0	0	0	0	0	0	0	0	0	0	0	
ENSXETG00000030075	119	0	0	0	0	0	0	0	0	0	0	0	0	
ENSXETG00000030076	2475	0	0	0	0	0	0	0	0	0	0	0	0	
ENSXETG00000030077	2304	0	0	0	0	0	0	0	0	0	0	0	0	
ENSXETG00000030078	1188	0	0	0	0	0	1	0	2	0	1	0	0	
ENSXETG00000030079	1563	0	0	1	0	0	0	0	3	0	6	0	4	ENSXETG00000030079
ENSXETG00000030080	377	2190	3384	2630	2318	1693	5769	426	477	306	2855	2054	2274	leap2
ENSXETG00000030081	252	120	169	136	199	188	195	146	175	117	117	89	114	COA4
ENSXETG00000030082	2583	0	0	0	0	1	0	0	0	0	0	0	0	ENSXETG00000030082
ENSXETG00000030083	1062	230	293	277	246	190	452	453	604	317	368	276	287	ENSXETG00000030083
ENSXETG00000030084	995	391	604	485	716	578	1849	804	928	545	972	660	772	ENSXETG00000030084
ENSXETG00000030085	3050	10	10	14	63	35	45	759	834	560	505	345	385	SMOC1
ENSXETG00000030086	119	0	0	0	0	0	0	0	0	0	0	0	0	
ENSXETG00000030087	939	0	0	0	0	0	0	0	0	0	0	0	0	
ENSXETG00000030088	510	0	0	0	0	0	0	0	0	0	0	0	0	
ENSXETG00000030089	603	9	16	8	4	6	11	8	7	3	20	15	12	RASL10B
ENSXETG00000030090	1036	0	0	0	0	0	0	0	0	0	0	0	0	
ENSXETG00000030091	3343	70	94	72	544	465	423	311	379	206	1048	724	841	ENSXETG00000030091
ENSXETG00000030092	1221	24	34	27	70	59	57	303	360	206	61	59	53	ENSXETG00000030092
ENSXETG00000030093	951	0	0	0	0	0	0	0	0	0	0	0	0	
ENSXETG00000030094	918	0	0	0	0	0	0	0	0	0	0	0	0	
ENSXETG00000030095	1290	307	475	389	546	352	853	1836	2027	1060	2186	1453	1703	ENSXETG00000030095
ENSXETG00000030096	1035	1	5	3	0	0	10	36	53	15	3	2	4	ccr3
ENSXETG00000030097	1752	1738	2595	2271	2591	2042	3207	2440	2813	1631	2941	2027	2328	PES1
ENSXETG00000030098	4074	20	37	44	95	50	93	164	186	113	196	118	156	RIMBP2
ENSXETG00000030099	70	0	0	0	0	0	0	0	0	0	0	0	0	
ENSXETG00000030100	843	3	2	0	10	6	2	0	0	0	7	11	4	ENSXETG00000030100
ENSXETG00000030101	930	0	0	0	0	0	0	0	0	0	0	0	0	
ENSXETG00000030102	383	1	0	0	0	1	0	0	0	0	0	0	0	
ENSXETG00000030103	958	0	2	7	2	0	2	31	24	16	4	3	2	ENSXETG00000030103
ENSXETG00000030104	717	4	3	6	31	30	13	47	50	32	32	21	20	ENSXETG00000030104
ENSXETG00000030105	1944	186	277	249	543	415	1681	1138	1221	906	1371	984	1013	dusp16
ENSXETG00000030106	1188	0	0	0	0	0	0	0	0	0	0	0	0	
ENSXETG00000030107	2409	43	90	68	127	72	235	377	419	271	253	155	187	MGC145685
ENSXETG00000030108	312	0	0	0	0	0	0	0	0	0	0	0	0	
ENSXETG00000030109	639	0	0	0	0	0	0	0	0	0	0	0	0	
ENSXETG00000030110	1182	54	86	89	261	203	362	120	130	73	233	112	140	FAM83E
ENSXETG00000030111	1098	0	0	0	2	0	0	0	0	0	1	0	2	itln2
ENSXETG00000030112	1497	46	50	76	145	90	142	156	173	90	232	153	172	ENSXETG00000030112
ENSXETG00000030113	3000	0	0	0	0	0	0	0	0	0	0	0	0	
ENSXETG00000030114	2119	126	168	148	146	117	110	216	317	176	168	126	144	ENSXETG00000030114
ENSXETG00000030115	1323	0	2	1	0	2	3	1	2	1	2	3	4	ENSXETG00000030115
ENSXETG00000030116	636	186	252	200	298	248	395	436	459	271	344	276	292	FAM167A
ENSXETG00000030117	1265	228	337	296	659	473	470	891	985	617	90	80	79	ENSXETG00000030117
ENSXETG00000030118	558	14	34	19	68	47	63	64	65	52	65	65	50	ENSXETG00000030118
ENSXETG00000030119	1310	0	0	0	0	0	0	0	0	1	0	0	0	fgfbp3
ENSXETG00000030120	1104	1	0	0	1	4	4	22	30	8	28	16	16	LGALS12

ENSXETG00000030121	513	0	0	0	0	0	0	0	0	0	0	0	0	
ENSXETG00000030122	1299	0	0	0	0	0	0	0	0	0	0	0	0	
ENSXETG00000030123	2334	0	0	0	0	0	0	0	0	0	0	0	0	
ENSXETG00000030124	2466	0	0	0	0	0	0	0	0	0	0	0	0	
ENSXETG00000030125	1170	59	88	74	208	117	162	230	271	147	124	99	99	TRIM59
ENSXETG00000030126	1023	4	1	5	13	11	20	55	60	32	30	19	18	ENSXETG00000030126
ENSXETG00000030127	119	0	0	0	0	0	0	0	0	0	0	0	0	
ENSXETG00000030128	1686	30	43	40	70	49	95	217	277	161	93	58	72	ENSXETG00000030128
ENSXETG00000030129	876	218	338	286	218	159	111	59	90	50	113	81	98	ENSXETG00000030129
ENSXETG00000030130	1508	4	4	8	26	13	15	79	80	41	20	9	18	ENSXETG00000030130
ENSXETG00000030131	3447	3	3	1	13	6	6	15	24	15	11	6	3	KCNT2
ENSXETG00000030132	873	286	444	373	315	229	418	111	137	88	120	95	85	ENSXETG00000030132
ENSXETG00000030133	312	0	0	0	0	0	0	0	0	0	0	0	0	
ENSXETG00000030134	396	101	154	113	94	80	149	236	262	135	117	80	104	armc7
ENSXETG00000030135	1059	1	1	0	0	0	0	1	1	0	2	0	0	ENSXETG00000030135
ENSXETG00000030136	933	0	0	0	0	0	0	0	0	0	0	0	0	
ENSXETG00000030137	2406	0	0	0	0	0	0	0	0	0	1	1	0	
ENSXETG00000030138	1065	5	10	13	11	12	13	11	12	14	3	0	4	DFNB59
ENSXETG00000030139	150	0	0	0	0	0	0	0	0	0	0	0	0	
ENSXETG00000030140	867	0	0	1	8	5	8	28	22	20	6	6	10	stx1b
ENSXETG00000030141	974	26	22	28	42	25	19	72	68	52	24	21	12	RDM1
ENSXETG00000030142	2034	532	727	666	1227	831	3510	1389	1611	964	2427	1782	1980	C2CD2
ENSXETG00000030143	798	3	5	0	53	45	18	131	157	89	76	66	69	SBSPON
ENSXETG00000030144	510	0	0	0	0	0	0	0	0	0	0	0	0	
ENSXETG00000030145	70	0	0	0	0	0	0	0	0	0	0	0	0	
ENSXETG00000030146	915	0	0	0	0	0	0	0	0	0	0	0	0	
ENSXETG00000030147	1203	196	243	240	560	425	433	1019	1132	628	994	665	723	ENSXETG00000030147
ENSXETG00000030148	357	0	0	0	0	0	0	0	0	0	0	0	0	
ENSXETG00000030149	939	0	0	0	0	0	0	0	0	0	0	0	0	
ENSXETG00000030150	1485	0	0	0	0	0	0	2	1	0	1	0	2	
ENSXETG00000030151	118	0	0	0	0	0	0	0	0	0	0	0	0	
ENSXETG00000030152	1587	17	16	15	20	11	15	39	39	28	30	17	25	ENSXETG00000030152
ENSXETG00000030153	70	0	0	0	0	0	0	0	0	0	0	0	0	
ENSXETG00000030154	507	0	0	0	0	0	0	0	0	0	0	0	0	ENSXETG00000030154
ENSXETG00000030155	501	0	1	0	0	0	0	0	0	0	0	1	0	ENSXETG00000030155
ENSXETG00000030156	390	2	0	0	18	8	3	4	2	1	2	3	1	ENSXETG00000030156
ENSXETG00000030157	3192	65	100	58	191	176	201	294	372	197	226	170	197	SMC5
ENSXETG00000030158	927	0	0	0	0	0	0	0	0	0	0	0	0	
ENSXETG00000030159	1242	1859	2640	2196	2474	1883	4896	670	799	472	1958	1469	1504	grina
ENSXETG00000030160	70	0	0	0	0	0	0	0	0	0	0	0	0	
ENSXETG00000030161	846	0	0	0	0	0	0	0	0	0	0	0	0	
ENSXETG00000030162	148	0	0	0	0	0	0	0	0	0	0	0	0	
ENSXETG00000030163	3654	376	562	485	1030	786	2067	863	1093	659	2089	1445	1723	ADGRG6
ENSXETG00000030164	810	1	0	4	2	2	1	36	53	17	3	1	8	ENSXETG00000030164
ENSXETG00000030165	534	0	0	0	0	0	0	0	0	0	0	0	0	
ENSXETG00000030166	1065	0	3	4	3	0	2	18	16	18	5	4	4	ENSXETG00000030166
ENSXETG00000030167	2410	15	20	13	86	51	88	323	345	205	108	63	109	trim47
ENSXETG00000030168	3006	1	0	0	0	0	0	0	0	0	0	0	2	
ENSXETG00000030169	1236	0	0	0	0	0	0	0	0	0	0	0	0	
ENSXETG00000030170	1094	0	0	0	0	0	0	0	1	0	0	1	0	
ENSXETG00000030171	2616	0	0	0	0	0	0	0	0	0	0	0	0	ENSXETG00000030171
ENSXETG00000030172	2676	1	2	1	2	2	8	30	34	20	11	13	14	KSR2
ENSXETG00000030173	1447	5	8	5	0	0	1	0	0	0	0	0	0	
ENSXETG00000030174	3333	28	24	24	139	82	145	196	217	136	131	112	130	WDR60
ENSXETG00000030175	119	0	0	0	0	0	0	0	0	0	0	0	0	
ENSXETG00000030176	1536	1	1	0	0	2	0	1	2	2	0	0	0	ENSXETG00000030176
ENSXETG00000030177	95	0	0	0	0	0	0	0	0	0	0	0	0	
ENSXETG00000030178	885	0	0	0	1	0	0	0	0	0	0	0	0	
ENSXETG00000030179	119	0	0	0	0	0	0	0	0	0	0	0	0	
ENSXETG00000030180	741	0	0	0	0	0	0	0	0	0	0	0	0	ENSXETG00000030180
ENSXETG00000030181	1017	0	0	0	0	0	0	0	2	0	0	0	0	
ENSXETG00000030182	930	0	0	0	0	0	0	0	0	0	0	0	0	
ENSXETG00000030183	2009	0	0	0	1	0	0	0	0	0	0	0	0	eln2
ENSXETG00000030184	837	0	0	0	0	0	2	2	8	3	0	0	0	MSANTD1
ENSXETG00000030185	1181	0	0	0	1	2	2	0	0	2	8	4	2	GPX2
ENSXETG00000030186	119	0	0	0	0	0	0	0	0	0	0	0	0	
ENSXETG00000030187	119	0	0	0	0	0	0	0	0	0	0	0	0	
ENSXETG00000030188	10455	1	2	3	191	134	11	4	5	2	9	5	7	DNAH10
ENSXETG00000030189	957	1	1	3	3	8	25	342	382	223	17	16	15	ENSXETG00000030189
ENSXETG00000030190	749	1133	1678	1383	762	529	747	991	1169	674	407	317	337	mydgf
ENSXETG00000030191	70	0	0	0	0	0	0	0	0	0	0	0	0	
ENSXETG00000030192	1152	43	42	32	77	48	102	103	141	53	73	40	67	MFSD9
ENSXETG00000030193	1768	1	1	2	3	3	0	4	0	1	0	0	0	ENSXETG00000030193
ENSXETG00000030194	296	0	0	0	0	0	0	0	0	0	0	0	0	
ENSXETG00000030195	296	5	4	5	1	1	9	2	1	0	1	0	0	Metazoa_SRP
ENSXETG00000030196	834	181	266	221	459	357	613	679	793	453	513	337	345	ENSXETG00000030196
ENSXETG00000030197	813	4	4	3	16	13	16	370	442	238	38	31	46	ENSXETG00000030197
ENSXETG00000030198	732	11	25	23	10	4	19	11	9	5	6	2	7	ENSXETG00000030198
ENSXETG00000030199	141	0	0	0	0	0	0	0	0	0	0	0	0	
ENSXETG00000030200	510	0	0	0	0	0	0	0	0	0	0	0	0	
ENSXETG00000030201	1332	2	3	6	2	2	94	35	31	16	10	3	5	ANGPTL6
ENSXETG00000030202	2142	0	1	0	2	1	2	23	29	12	6	7	8	ENSXETG00000030202
ENSXETG00000030203	1149	96	146	103	155	116	220	317	403	233	169	108	147	ENSXETG00000030203
ENSXETG00000030204	4145	44	65	64	231	218	661	1358	1653	958	386	254	279	spock3
ENSXETG00000030205	119	0	0	0	0	0	0	0	0	0	0	0	0	
ENSXETG00000030206	528	0	0	0	0	0	0	0	0	0	0	0	0	
ENSXETG00000030207	668	75	107	93	131	115	70	240	250	161	71	55	54	ENSXETG00000030207
ENSXETG00000030208	2526	0	0	0	0	0	0	0	0	0	0	0	0	
ENSXETG00000030209	936	0	0	0	0	0	0	1	3	2	0	0	0	ENSXETG00000030209
ENSXETG00000030210	4502	1024	1501	1318	2051	1534	2762	3147	3787	2134	1866	1336	1462	ANKFY1
ENSXETG00000030211	312	0	0	0	0	0	0	0	0	0	0	0	0	
ENSXETG00000030212	2486	1982	2874	2562	1940	1508	3246	2475	3275	1894	2681	1862	2109	taf11
ENSXETG00000030213	162	1	1	1	10	2	20	33	34	14	24	14	18	ENSXETG00000030213
ENSXETG00000030214	2619	0	0	0	0	0	0	0	0	0	0	0	0	
ENSXETG00000030215	119	0	0	0	0	0	0	0	0	0	0	0	0	
ENSXETG00000030216	1170	3	5	7	73	52	126	1260	1377	931	599	424	509	ENSXETG00000030216
ENSXETG00000030217	1008	4	7	6	21	17	27	52	48	40	23	20	15	GPR17
ENSXETG00000030218	877	293	445	343	412	292	391	472	497	318	411	246	255	RANGRF
ENSXETG00000030219	119	0	0	0	0	0	0	0	0	0	0	0	0	
ENSXETG00000030220	5222	210	265	228	433	362	247	889	954	595	438	374	363	pfas
ENSXETG00000030221	312	0	0	0	0	0	0	0	0	0	0	0	0	
ENSXETG00000030222	405	0	0	0	0	0	0	7	5	5	0	0	0	ENSXETG00000030222
ENSXETG00000030223	1866	26	50	32	97	61	123	127	162	87	78	68	73	ENSXETG00000030223
ENSXETG00000030224	525	0	0	0	33	10	12	11	18	7	10	10	15	ENSXETG00000030224
ENSXETG00000030225	1764	101	138	140	231	176	372	459	588	316	323	231	234	KLHL18
ENSXETG00000030226	486	0	0	0	0	0	0	0	0	0	0	0	0	
ENSXETG00000030227	4343	136	169	149	267	220	294	586	656	433	457	322	344	krit1
ENSXETG00000030228	510	0	0	0	0	0	0	0	0	0	0	0	0	
ENSXETG00000030229	119	0	0	0	0	0	0	0	0	0	0	0	0	
ENSXETG00000030230	1283	34	43	32	160	117	75	286	368	209	245	162	144	tesc
ENSXETG00000030231	1767	1	4	0	1	3	6	16	9	9	16	8	8	ENSXETG00000030231

ENSXETG00000030232	164	0	0	0	0	0	0	0	0	0	0	0	0	
ENSXETG00000030233	507	0	0	0	0	0	0	0	0	0	0	0	0	
ENSXETG00000030234	1338	203	320	252	282	218	1042	1098	1265	689	428	300	321	ENSXETG00000030234
ENSXETG00000030235	492	0	0	0	0	0	0	0	0	0	0	0	0	
ENSXETG00000030236	657	0	0	0	0	0	0	0	0	0	0	0	0	ENSXETG00000030236
ENSXETG00000030237	786	0	0	0	0	0	0	0	0	0	0	0	0	
ENSXETG00000030238	2412	0	0	0	0	0	0	0	0	0	0	0	0	
ENSXETG00000030239	1013	603	868	829	682	563	489	800	878	591	830	534	595	ENSXETG00000030239
ENSXETG00000030240	141	0	0	0	0	0	0	0	0	0	0	0	0	
ENSXETG00000030241	1185	1	0	1	1	2	1	2	1	0	0	1	1	ENSXETG00000030241
ENSXETG00000030242	933	2	4	3	16	17	50	195	234	160	97	63	70	ENSXETG00000030242
ENSXETG00000030243	534	2	4	5	2	4	0	1	2	2	0	1	2	ENSXETG00000030243
ENSXETG00000030244	182	0	0	0	0	0	0	0	0	0	0	0	0	
ENSXETG00000030245	981	0	0	0	0	0	0	0	0	0	0	0	0	
ENSXETG00000030246	1005	792	1192	983	271	230	65	121	148	105	5	0	0	ENSXETG00000030246
ENSXETG00000030247	2865	258	369	310	548	352	924	1600	1868	1022	1591	1077	1278	FARP1
ENSXETG00000030248	939	0	0	0	0	0	0	0	0	0	0	0	0	
ENSXETG00000030249	975	9	20	29	9	3	8	22	17	11	11	3	10	ENSXETG00000030249
ENSXETG00000030250	1557	29	39	38	192	151	119	135	134	94	156	97	109	ENSXETG00000030250
ENSXETG00000030251	191	0	0	0	0	0	0	0	0	0	0	0	0	
ENSXETG00000030252	1509	0	0	0	0	2	0	9	12	4	1	0	0	
ENSXETG00000030253	537	2155	3010	2750	1588	1242	3227	198	230	150	822	553	599	ENSXETG00000030253
ENSXETG00000030254	2931	61	86	77	123	85	182	305	329	204	280	208	211	ENSXETG00000030254
ENSXETG00000030255	2325	0	0	0	0	0	0	0	0	0	0	0	0	
ENSXETG00000030256	872	14	20	20	16	13	31	96	122	62	46	18	28	lta
ENSXETG00000030257	106	0	0	0	0	0	0	0	0	0	0	0	0	
ENSXETG00000030258	2490	52	99	93	155	133	215	108	132	80	166	87	124	pqlc1.2
ENSXETG00000030259	119	0	0	0	0	0	0	0	0	0	0	0	0	
ENSXETG00000030260	897	25	31	20	34	38	29	124	120	66	21	12	19	ENSXETG00000030260
ENSXETG00000030261	930	0	0	0	0	0	0	0	0	0	0	0	0	
ENSXETG00000030262	1170	0	0	0	0	0	0	0	0	0	0	0	0	
ENSXETG00000030263	1728	851	1295	1098	2260	1652	1320	489	575	356	846	641	696	ENSXETG00000030263
ENSXETG00000030264	1461	0	0	0	0	0	0	0	0	0	0	0	0	
ENSXETG00000030265	1554	1	2	1	3	3	9	17	20	14	19	9	16	mchr1.2
ENSXETG00000030266	2694	0	0	1	49	42	4	1	1	2	4	2	1	WDR66
ENSXETG00000030267	1296	188	278	244	315	243	334	93	108	74	145	117	140	ENSXETG00000030267
ENSXETG00000030268	654	2	0	0	18	12	2	0	2	0	1	0	1	CCDC169
ENSXETG00000030269	936	0	0	0	0	0	0	0	0	0	0	0	0	
ENSXETG00000030270	1323	301	382	357	833	675	2485	900	893	641	1844	1260	1432	ENSXETG00000030270
ENSXETG00000030271	2010	439	660	581	1056	921	1503	1167	1329	841	727	461	524	ENSXETG00000030271
ENSXETG00000030272	1137	137	202	170	218	142	336	333	384	226	255	199	207	ENSXETG00000030272
ENSXETG00000030273	164	0	0	0	0	0	0	0	0	0	0	0	0	
ENSXETG00000030274	1431	21	24	27	61	52	129	89	103	55	160	113	120	ENSXETG00000030274
ENSXETG00000030275	249	1510	2186	1801	1953	1448	2512	1849	2258	1181	2732	1987	2240	MIF
ENSXETG00000030276	693	0	0	0	0	0	0	0	0	0	0	0	0	
ENSXETG00000030277	1083	0	0	0	0	0	0	0	0	0	0	0	0	
ENSXETG00000030278	2430	227	340	281	982	696	1494	1976	2211	1215	1630	1160	1271	adam7
ENSXETG00000030279	119	0	0	0	0	0	0	0	0	0	0	0	0	
ENSXETG00000030280	888	3	11	5	9	7	8	42	65	32	18	22	19	t2r38
ENSXETG00000030281	1245	133	227	151	282	256	424	634	662	411	524	397	394	SNTB2
ENSXETG00000030282	1632	5	11	7	14	7	24	28	40	27	28	10	15	ENSXETG00000030282
ENSXETG00000030283	2463	57	96	79	219	139	279	1168	1417	866	470	317	360	FGD1
ENSXETG00000030284	1356	0	0	0	2	0	1	2	1	0	2	0	0	ENSXETG00000030284
ENSXETG00000030285	1734	70	77	76	132	76	367	275	295	196	243	187	230	PWWP2B
ENSXETG00000030286	3579	284	480	402	1998	1512	2130	645	821	474	1703	1292	1428	DGKD
ENSXETG00000030287	207	0	0	0	0	0	0	0	0	0	0	0	0	ENSXETG00000030287
ENSXETG00000030288	1182	0	0	0	0	0	0	0	0	0	0	1	0	
ENSXETG00000030289	321	0	0	0	0	0	0	0	0	0	0	0	0	ENSXETG00000030289
ENSXETG00000030290	924	3	8	7	0	0	0	0	0	0	0	0	0	
ENSXETG00000030291	100	0	0	0	0	0	0	0	0	0	0	0	0	
ENSXETG00000030292	921	0	0	0	0	0	0	0	0	0	0	0	0	
ENSXETG00000030293	1233	0	0	0	0	0	0	0	0	0	0	0	0	
ENSXETG00000030294	1653	1	0	0	4	0	5	58	65	42	6	10	4	EBF4
ENSXETG00000030295	119	0	0	0	0	0	0	0	0	0	0	0	0	
ENSXETG00000030296	1191	0	0	0	0	0	0	0	0	0	0	0	0	
ENSXETG00000030297	2082	34	49	39	97	70	201	135	185	97	220	126	141	ENSXETG00000030297
ENSXETG00000030298	582	18	32	31	104	79	36	16	15	7	23	13	19	PIFO
ENSXETG00000030299	948	0	0	0	0	0	0	0	0	0	0	0	0	
ENSXETG00000030300	1245	91	161	128	547	443	875	2511	3140	1731	852	638	681	ENSXETG00000030300
ENSXETG00000030301	1110	16	22	13	0	0	0	6	10	7	0	0	2	
ENSXETG00000030302	2640	0	0	0	0	0	0	0	0	0	0	0	0	
ENSXETG00000030303	2760	201	294	247	512	452	840	830	862	507	648	486	480	DCAF5
ENSXETG00000030304	795	0	0	0	0	0	0	2	1	0	0	0	0	
ENSXETG00000030305	2007	2	1	3	18	14	22	59	86	36	49	42	34	AMPH
ENSXETG00000030306	158	0	0	0	0	0	0	0	0	0	0	0	0	
ENSXETG00000030307	2958	55	94	78	212	174	312	374	422	229	464	349	416	ENSXETG00000030307
ENSXETG00000030308	1185	0	0	0	0	0	0	0	0	0	1	0	0	
ENSXETG00000030309	2388	0	0	0	0	0	0	0	0	0	0	0	0	
ENSXETG00000030310	2445	385	524	413	1419	1043	2542	471	494	283	1189	885	956	MLXIPL
ENSXETG00000030311	981	0	0	0	0	0	0	0	0	0	0	0	0	
ENSXETG00000030312	936	0	0	0	0	0	0	0	0	0	0	0	0	
ENSXETG00000030313	1494	91	140	122	494	340	353	302	332	203	578	391	437	TFCP2L1
ENSXETG00000030314	1122	22	22	30	80	47	124	133	153	96	147	117	137	ENSXETG00000030314
ENSXETG00000030315	984	0	0	0	0	0	0	0	0	0	0	0	0	
ENSXETG00000030316	528	0	0	0	0	0	0	0	0	0	0	0	0	
ENSXETG00000030317	828	2	2	6	1	0	1	5	6	0	2	2	3	ENSXETG00000030317
ENSXETG00000030318	312	0	0	0	0	0	0	0	0	0	0	0	0	
ENSXETG00000030319	1188	0	0	0	1	1	0	0	0	0	1	0	0	
ENSXETG00000030320	2358	0	0	0	0	0	0	0	0	0	0	0	0	
ENSXETG00000030321	1350	1	10	3	10	6	23	249	336	172	38	44	43	ENSXETG00000030321
ENSXETG00000030322	522	0	0	0	0	0	0	0	0	0	0	0	0	ENSXETG00000030322
ENSXETG00000030323	954	0	0	0	4	2	0	0	0	0	0	0	0	
ENSXETG00000030324	1185	2	1	1	6	1	2	3	4	0	11	2	1	ENSXETG00000030324
ENSXETG00000030325	2142	0	0	0	0	0	0	0	0	0	0	0	0	
ENSXETG00000030326	5016	41	98	88	293	246	392	734	909	562	674	503	561	ENSXETG00000030326
ENSXETG00000030327	564	0	0	0	0	0	0	1	1	0	0	0	0	
ENSXETG00000030328	119	0	0	0	0	0	0	0	0	0	0	0	0	
ENSXETG00000030329	336	762	1141	1012	751	623	925	711	852	477	498	348	420	ENSXETG00000030329
ENSXETG00000030330	2265	19	21	21	26	12	10	102	112	68	28	27	40	ENSXETG00000030330
ENSXETG00000030331	1305	0	0	0	0	0	0	0	0	0	0	0	0	
ENSXETG00000030332	2694	32	44	42	93	63	106	96	94	55	70	40	53	CCDC191
ENSXETG00000030333	882	60	104	77	66	61	133	36	43	28	127	93	109	ENSXETG00000030333
ENSXETG00000030334	1557	39	58	72	124	113	212	385	364	215	293	225	188	ENSXETG00000030334
ENSXETG00000030335	2205	1	2	4	15	15	7	6	6	1	15	19	15	PKD2L1
ENSXETG00000030336	3822	422	582	476	1093	838	1964	1164	1319	764	2841	1969	2296	fbxo34
ENSXETG00000030337	276	24	27	36	45	23	60	67	82	51	48	41	38	ENSXETG00000030337
ENSXETG00000030338	2106	0	0	0	0	0	0	0	0	0	1	0	0	
ENSXETG00000030339	5472	273	463	430	1320	970	753	1946	2150	1385	1151	865	938	ENSXETG00000030339
ENSXETG00000030340	2338	51	72	60	36	32	87	292	324	207	107	68	73	rhoh
ENSXETG00000030341	1215	8	7	13	28	16	67	360	411	261	73	33	52	RAB3IL1
ENSXETG00000030342	214	0	0	0	0	0	0	0	0	0	0	0	0	

ENSXETG00000030343	267	999	1482	1143	705	528	795	768	959	568	599	365	450	SLIRP
ENSXETG00000030344	108	7	0	0	20	21	3	2	2	1	0	2	1	PK-G12rRNA
ENSXETG00000030345	1449	167	205	178	327	236	557	456	519	351	416	316	319	gpatch3
ENSXETG00000030346	1167	1	0	1	4	1	2	2	5	5	9	9	5	FAM46D
ENSXETG00000030347	789	0	1	0	0	0	0	1	1	0	1	1	0	GSG1L2
ENSXETG00000030348	981	0	0	0	1	1	0	2	0	1	1	2	0	GPR12
ENSXETG00000030349	3252	1	1	0	9	9	26	5	6	7	7	7	4	ENSXETG00000030349
ENSXETG00000030350	393	0	0	0	0	0	1	6	7	6	2	0	0	ENSXETG00000030350
ENSXETG00000030351	657	0	0	0	0	0	0	0	0	0	0	0	0	
ENSXETG00000030352	119	0	0	0	0	0	0	0	0	0	0	0	0	5S_rRNA
ENSXETG00000030353	684	0	0	0	0	0	1	0	0	0	0	0	0	
ENSXETG00000030354	690	56	86	65	102	91	216	560	604	355	183	124	135	ENSXETG00000030354
ENSXETG00000030355	477	4	5	9	41	25	11	13	13	8	3	2	5	CYB5D1
ENSXETG00000030356	1020	1	0	0	0	0	0	2	0	0	0	0	0	
ENSXETG00000030357	324	2	8	5	14	10	45	182	208	139	22	10	18	ENSXETG00000030357
ENSXETG00000030358	1871	152	207	187	193	149	360	213	207	133	241	196	201	GLYCTK
ENSXETG00000030359	3897	2	0	1	0	0	0	5	1	1	0	0	0	ENSXETG00000030359
ENSXETG00000030360	2161	1180	1662	1464	1574	1168	2694	387	499	279	782	579	602	slc10a3
ENSXETG00000030361	396	133	220	141	58	49	160	109	140	73	83	73	78	ENSXETG00000030361
ENSXETG00000030362	681	0	0	0	0	0	0	0	0	0	0	0	0	
ENSXETG00000030363	750	14	22	21	40	51	67	149	161	110	164	98	114	ENSXETG00000030363
ENSXETG00000030364	2385	0	0	0	0	0	0	0	0	0	0	0	0	
ENSXETG00000030365	264	23	23	30	34	35	82	43	58	28	101	70	87	ENSXETG00000030365
ENSXETG00000030366	2331	2	2	0	0	0	1	0	0	0	1	1	0	ENSXETG00000030366
ENSXETG00000030367	1625	39	30	31	91	49	77	234	263	149	91	71	78	naip
ENSXETG00000030368	777	0	0	0	0	0	0	53	78	47	0	0	2	PI15
ENSXETG00000030369	1289	283	401	298	211	161	205	562	624	367	240	143	158	fam103a1
ENSXETG00000030370	107	0	0	0	0	0	0	0	0	0	0	0	0	
ENSXETG00000030371	1117	3	0	0	1	1	1	11	14	5	2	3	0	ENSXETG00000030371
ENSXETG00000030372	1293	1069	1589	1388	1585	1305	2489	443	492	295	1988	1419	1569	NR1H4
ENSXETG00000030373	91	7	11	8	2	5	3	7	10	2	7	8	10	SNORD14
ENSXETG00000030374	1851	0	0	0	0	0	0	0	0	0	0	0	0	
ENSXETG00000030375	164	0	0	0	0	0	0	0	0	0	0	0	0	
ENSXETG00000030376	1557	4	7	9	29	29	26	47	54	30	57	41	38	AMIGO2
ENSXETG00000030377	546	0	0	0	0	0	0	1	1	2	0	1	0	ATP6AP1L
ENSXETG00000030378	2881	100	125	111	324	233	226	322	397	217	625	472	501	tbc1d30
ENSXETG00000030379	7155	472	707	611	1458	1132	2523	1530	1905	1057	2503	1901	2041	PATJ
ENSXETG00000030380	501	0	0	0	1	1	2	1	1	2	11	5	5	ENSXETG00000030380
ENSXETG00000030381	581	2	1	0	3	0	3	0	1	0	5	2	2	ENSXETG00000030381
ENSXETG00000030382	960	0	0	0	0	0	0	0	0	0	0	0	0	
ENSXETG00000030383	1091	0	0	0	0	0	0	0	0	0	0	0	0	
ENSXETG00000030384	975	22	35	24	36	22	11	171	191	101	13	5	7	ENSXETG00000030384
ENSXETG00000030385	1467	114	203	188	399	289	298	821	942	597	282	262	274	ENSXETG00000030385
ENSXETG00000030386	81	0	0	0	0	0	0	0	0	0	0	0	0	
ENSXETG00000030387	678	2	0	1	36	39	4	1	3	1	4	2	3	ENSXETG00000030387
ENSXETG00000030388	1546	0	4	5	5	3	18	109	123	67	25	21	27	ENSXETG00000030388
ENSXETG00000030389	5829	1	2	0	4	2	1	3	1	1	2	0	2	MYH6
ENSXETG00000030390	2496	209	335	269	481	375	2619	78	109	67	1687	1197	1325	HHIPL1
ENSXETG00000030391	1716	737	1085	866	1652	1131	2415	1449	1682	1009	1764	1303	1460	ENSXETG00000030391
ENSXETG00000030392	669	29	34	26	22	17	35	48	42	32	58	43	50	ENSXETG00000030392
ENSXETG00000030393	513	0	0	0	0	0	0	0	0	0	0	0	0	
ENSXETG00000030394	495	1	4	3	0	1	0	34	71	56	3	3	2	xt6l
ENSXETG00000030395	1041	0	0	0	5	4	0	1	3	1	0	0	0	
ENSXETG00000030396	2226	0	0	0	0	0	0	1	0	0	0	0	0	
ENSXETG00000030397	819	100	159	149	151	126	231	620	666	378	288	200	175	ERI3
ENSXETG00000030398	930	98	145	113	329	214	502	506	559	359	508	383	437	ENSXETG00000030398
ENSXETG00000030399	933	0	0	0	0	0	0	0	0	0	0	0	0	
ENSXETG00000030400	1311	0	0	0	1	0	0	6	7	7	4	0	1	ENSXETG00000030400
ENSXETG00000030401	1059	0	0	0	0	0	0	15	10	1	0	1	0	ENSXETG00000030401
ENSXETG00000030402	2166	0	0	0	0	0	0	0	0	0	0	0	0	
ENSXETG00000030403	6248	545	726	615	2646	1958	2546	2565	3038	1695	5166	3701	4190	lgr4
ENSXETG00000030404	2182	72	95	86	214	163	403	914	1061	645	295	237	233	tbc1d12
ENSXETG00000030405	663	0	0	0	0	0	0	0	0	0	0	0	0	
ENSXETG00000030406	1259	12	27	21	28	20	32	48	62	39	28	20	21	ENSXETG00000030406
ENSXETG00000030407	1188	0	0	0	0	0	0	0	0	0	0	0	0	
ENSXETG00000030408	1170	0	0	0	0	1	0	0	1	1	3	1	1	
ENSXETG00000030409	918	0	0	0	0	0	0	0	0	0	0	0	0	
ENSXETG00000030410	1926	1	5	0	2	0	2	20	20	12	6	7	6	LRRC4
ENSXETG00000030411	783	26	19	14	44	37	40	25	19	11	24	33	26	
ENSXETG00000030412	918	0	0	0	0	0	0	3	0	0	0	0	0	
ENSXETG00000030413	1393	3	1	3	10	10	4	9	6	6	17	15	12	KCNK9
ENSXETG00000030414	735	0	0	0	0	0	0	0	0	0	0	0	0	
ENSXETG00000030415	933	0	0	0	0	0	0	0	0	0	0	0	0	
ENSXETG00000030416	2583	0	0	0	0	0	0	0	0	0	0	0	0	
ENSXETG00000030417	2109	0	0	0	0	0	0	0	0	0	0	0	0	
ENSXETG00000030418	1323	0	0	0	0	0	0	0	0	0	0	0	0	
ENSXETG00000030419	528	0	0	0	0	0	0	0	0	0	0	0	0	
ENSXETG00000030420	1481	0	2	0	3	2	2	0	1	1	19	11	12	ENSXETG00000030420
ENSXETG00000030421	900	0	0	0	0	0	0	0	0	0	0	0	0	
ENSXETG00000030422	495	0	0	0	0	0	0	0	0	0	0	0	0	
ENSXETG00000030423	1089	0	0	0	0	0	0	0	0	0	0	0	0	
ENSXETG00000030424	855	0	0	0	0	0	0	0	0	0	0	0	0	
ENSXETG00000030425	696	18	23	17	8	7	4	28	18	17	0	0	0	ENSXETG00000030425
ENSXETG00000030426	1119	49	77	48	86	71	55	75	76	46	202	160	163	ENSXETG00000030426
ENSXETG00000030427	1005	0	0	0	0	0	0	0	0	0	0	0	0	
ENSXETG00000030428	3096	165	245	204	327	255	696	1405	1534	908	413	301	311	ENSXETG00000030428
ENSXETG00000030429	2517	0	0	0	0	0	0	0	1	0	0	0	0	ENSXETG00000030429
ENSXETG00000030430	119	0	0	0	0	0	0	0	0	0	0	0	0	
ENSXETG00000030431	783	0	0	0	0	0	0	0	0	0	0	0	0	
ENSXETG00000030432	657	0	0	0	0	0	1	0	0	0	0	0	0	
ENSXETG00000030433	4243	31	25	28	350	299	73	740	893	560	167	123	141	proser2
ENSXETG00000030434	897	0	0	0	0	0	0	0	0	0	0	0	0	
ENSXETG00000030435	366	0	0	0	0	0	0	0	0	0	0	0	0	
ENSXETG00000030436	1566	0	0	0	0	0	0	0	0	0	0	0	0	
ENSXETG00000030437	5647	189	326	253	1365	994	2556	1402	1629	972	2468	1751	2026	TNRC6A
ENSXETG00000030438	2388	0	0	0	0	0	0	0	0	0	0	0	0	ENSXETG00000030438
ENSXETG00000030439	135	0	0	0	0	1	1	0	2	1	1	2	1	SNORA66
ENSXETG00000030440	1518	0	0	0	0	0	0	0	0	0	0	0	0	
ENSXETG00000030441	531	0	0	0	0	0	0	0	0	0	0	0	0	
ENSXETG00000030442	1623	70	74	66	199	130	322	223	261	166	251	189	194	GHDC
ENSXETG00000030443	3774	2	2	2	6	11	35	16	29	8	202	112	142	ITIH6
ENSXETG00000030444	501	0	0	0	0	0	0	0	0	0	0	0	0	
ENSXETG00000030445	984	34	50	48	28	30	43	419	463	256	72	54	46	ENSXETG00000030445
ENSXETG00000030446	1959	0	1	1	31	27	138	235	257	148	85	69	81	ENSXETG00000030446
ENSXETG00000030447	3922	278	331	303	211	183	737	370	473	298	816	543	633	ENSXETG00000030447
ENSXETG00000030448	693	0	0	0	0	0	0	6	10	3	1	1	2	ENSXETG00000030448
ENSXETG00000030449	57	0	0	0	0	0	0	0	0	0	0	1	0	
ENSXETG00000030450	2790	432	562	517	730	558	856	1354	1572	918	1093	769	842	ENSXETG00000030450
ENSXETG00000030451	3282	0	0	1	3	1	2	11	8	6	7	5	6	DCC
ENSXETG00000030452	885	19	21	19	167	125	31	127	174	77	82	30	69	ENSXETG00000030452
ENSXETG00000030453	903	1	3	2	1	0	0	2	3	5	1	2	3	

ENSXETG00000030454	783	526	738	637	515	406	513	567	632	353	354	231	248	PLPBP
ENSXETG00000030455	516	0	0	0	0	0	0	0	0	0	0	0	0	
ENSXETG00000030456	909	1435	1966	1607	926	706	1240	156	218	121	447	275	316	MGST2
ENSXETG00000030457	513	0	0	0	0	0	0	0	0	0	0	0	0	
ENSXETG00000030458	5010	13	15	18	17	9	23	226	283	167	115	88	101	ENSXETG00000030458
ENSXETG00000030459	119	0	0	0	0	0	0	0	0	0	0	0	0	
ENSXETG00000030460	57	0	0	0	0	0	0	0	0	0	0	0	0	
ENSXETG00000030461	996	2	1	3	20	18	49	152	201	103	59	38	46	ENSXETG00000030461
ENSXETG00000030462	2568	0	0	0	0	0	0	0	0	0	0	0	0	
ENSXETG00000030463	1161	0	0	0	0	0	0	0	0	0	0	0	0	
ENSXETG00000030464	1506	0	0	0	0	0	0	1	5	0	0	0	1	ENSXETG00000030464
ENSXETG00000030465	801	528	803	663	354	281	510	1292	1497	912	659	455	546	F3
ENSXETG00000030466	1395	4	9	15	13	16	36	14	16	16	35	20	17	ENSXETG00000030466
ENSXETG00000030467	396	0	0	2	0	0	0	3	5	7	1	0	1	ENSXETG00000030467
ENSXETG00000030468	786	0	0	0	0	0	1	1	1	1	1	0	2	
ENSXETG00000030469	2115	4	9	7	17	10	42	31	28	11	24	24	23	ABLIM3
ENSXETG00000030470	780	0	0	0	0	0	0	0	0	0	0	0	0	
ENSXETG00000030471	685	0	0	0	1	0	0	6	10	1	4	3	3	ENSXETG00000030471
ENSXETG00000030472	1602	1	0	1	21	14	14	12	8	5	105	70	92	ENSXETG00000030472
ENSXETG00000030473	2061	0	0	0	0	0	0	0	0	0	0	0	0	
ENSXETG00000030474	130	0	0	0	0	0	0	0	0	0	0	0	0	U6atac
ENSXETG00000030475	8325	715	1099	1008	1257	989	757	1338	1467	865	2024	1376	1640	ENSXETG00000030475
ENSXETG00000030476	1035	0	0	0	0	0	0	0	0	0	0	0	0	
ENSXETG00000030477	939	150	215	162	549	434	899	1308	1461	906	1027	744	846	ifitm1
ENSXETG00000030478	1113	626	886	824	934	691	2078	1239	1382	825	1300	942	1045	TUFT1
ENSXETG00000030479	1665	281	417	375	573	452	1039	1600	1710	1110	1023	709	714	SMPD1
ENSXETG00000030480	447	0	0	0	0	0	0	0	0	0	0	0	0	
ENSXETG00000030481	2795	11	21	17	33	30	76	105	121	78	170	101	130	angptl1
ENSXETG00000030482	119	0	0	0	0	0	0	0	0	0	0	0	0	
ENSXETG00000030483	1278	4	9	3	174	119	19	9	24	16	16	13	18	ENSXETG00000030483
ENSXETG00000030484	1113	0	0	0	0	0	0	0	0	0	0	0	0	
ENSXETG00000030485	678	0	0	6	0	0	0	1	3	6	4	0	2	ENSXETG00000030485
ENSXETG00000030486	115	0	0	0	0	0	0	0	0	0	0	0	0	
ENSXETG00000030487	127	0	0	0	0	0	0	0	0	0	0	0	0	
ENSXETG00000030488	1632	0	0	0	0	0	0	0	0	0	0	0	0	ENSXETG00000030488
ENSXETG00000030489	399	12	17	19	38	15	33	42	33	23	28	16	21	ENSXETG00000030489
ENSXETG00000030490	393	0	0	0	0	0	0	0	0	0	0	0	0	
ENSXETG00000030491	1854	3	0	0	1	0	0	0	0	0	4	2	1	ENSXETG00000030491
ENSXETG00000030492	1293	0	0	0	1	0	0	4	1	1	1	0	0	TMPRSS3
ENSXETG00000030493	474	105	124	123	49	34	124	215	245	116	99	63	74	C15orf61
ENSXETG00000030494	119	0	0	0	0	0	0	0	0	0	0	0	0	
ENSXETG00000030495	686	150	221	168	325	261	149	109	118	84	313	229	279	ENSXETG00000030495
ENSXETG00000030496	1310	3	2	1	14	7	25	40	48	33	30	14	17	SCARA5
ENSXETG00000030497	119	0	0	0	0	0	0	0	0	0	0	0	0	
ENSXETG00000030498	1917	0	0	0	0	0	0	0	0	0	0	0	0	
ENSXETG00000030499	119	0	0	0	0	0	0	0	0	0	0	0	0	
ENSXETG00000030500	915	0	0	0	0	0	0	0	0	0	0	0	0	
ENSXETG00000030501	1110	4	4	6	4	3	8	5	2	4	1	1	0	henmt1
ENSXETG00000030502	1572	79	119	102	169	131	254	126	129	77	183	132	135	ENSXETG00000030502
ENSXETG00000030503	921	0	0	0	0	0	0	0	0	0	1	1	2	
ENSXETG00000030504	801	34	40	48	103	75	81	121	126	82	62	55	51	ENSXETG00000030504
ENSXETG00000030505	1824	0	0	0	0	0	0	0	1	0	0	0	0	ENSXETG00000030505
ENSXETG00000030506	966	7	10	7	14	5	42	122	169	102	42	36	29	UST
ENSXETG00000030507	1170	0	0	1	0	0	1	1	0	1	0	2	0	ENSXETG00000030507
ENSXETG00000030508	2021	0	0	0	0	0	0	18	19	10	3	2	1	crtac1
ENSXETG00000030509	936	0	0	0	0	0	0	0	0	0	0	0	0	
ENSXETG00000030510	1395	0	0	0	0	0	0	0	0	0	0	0	0	
ENSXETG00000030511	993	10076	14181	12165	15566	11789	20722	9845	10972	6748	23438	15856	18069	ENSXETG00000030511
ENSXETG00000030512	1020	0	0	0	0	0	0	0	0	0	0	0	0	
ENSXETG00000030513	141	0	0	0	0	0	0	0	0	0	0	0	0	
ENSXETG00000030514	5424	2	5	5	134	128	216	335	443	262	244	149	216	PLCE1
ENSXETG00000030515	600	112	186	134	83	59	259	22	32	4	0	0	0	ENSXETG00000030515
ENSXETG00000030516	687	0	0	0	0	0	0	0	0	0	0	0	0	
ENSXETG00000030517	411	0	0	0	0	0	0	0	0	0	0	0	0	hist2h3c
ENSXETG00000030518	1011	0	0	0	0	0	0	0	0	0	0	0	0	
ENSXETG00000030519	70	0	0	0	0	0	0	0	0	0	0	0	0	
ENSXETG00000030520	2601	0	0	0	0	0	0	0	0	0	0	0	0	
ENSXETG00000030521	963	0	0	0	0	0	0	0	0	0	0	0	0	ENSXETG00000030521
ENSXETG00000030522	966	0	0	0	0	0	0	0	0	0	0	0	0	
ENSXETG00000030523	2397	0	0	0	0	0	0	0	0	0	0	0	0	
ENSXETG00000030524	816	0	0	1	0	3	2	2	2	0	4	2	5	ENSXETG00000030524
ENSXETG00000030525	2018	345	471	420	243	162	340	464	455	308	437	350	330	ENSXETG00000030525
ENSXETG00000030526	2990	1	4	3	591	410	34	4	5	1	13	6	12	tsga10
ENSXETG00000030527	383	0	0	0	0	0	0	0	0	0	3	1	3	
ENSXETG00000030528	191	0	0	0	0	0	0	0	0	0	0	0	0	
ENSXETG00000030529	546	11	20	11	152	128	50	92	122	54	364	273	266	LYPD6B
ENSXETG00000030530	648	3	0	1	1	2	4	2	1	1	7	4	2	ENSXETG00000030530
ENSXETG00000030531	1284	49	79	76	517	445	196	296	330	193	1355	928	1054	ENSXETG00000030531
ENSXETG00000030532	876	2166	3330	2769	3187	2440	7143	1000	1038	679	1165	825	919	ENSXETG00000030532
ENSXETG00000030533	1188	0	0	1	1	0	0	0	0	0	0	0	0	
ENSXETG00000030534	513	0	0	0	0	0	0	0	0	0	0	0	0	
ENSXETG00000030535	2463	0	0	0	1	1	5	11	8	5	3	6	7	ENSXETG00000030535
ENSXETG00000030536	927	111	155	148	203	156	305	413	429	234	249	174	204	CACUL1
ENSXETG00000030537	119	0	0	0	0	0	0	0	0	0	0	0	0	5S_rRNA
ENSXETG00000030538	591	0	0	0	0	0	0	0	0	0	0	0	0	
ENSXETG00000030539	1185	0	0	0	0	0	0	0	0	0	3	2	0	ENSXETG00000030539
ENSXETG00000030540	564	472	619	579	565	404	758	462	497	290	391	302	313	ENSXETG00000030540
ENSXETG00000030541	1665	157	239	196	437	298	1078	90	104	54	36	26	47	ENSXETG00000030541
ENSXETG00000030542	1673	163	194	196	257	165	421	492	509	313	346	214	247	fbxo22
ENSXETG00000030543	57	0	0	0	0	0	0	0	0	0	0	0	0	
ENSXETG00000030544	804	0	0	0	2	8	4	25	26	18	14	3	8	ENSXETG00000030544
ENSXETG00000030545	91	0	0	0	0	0	0	0	0	0	0	0	0	
ENSXETG00000030546	486	0	0	0	0	0	0	0	0	0	0	0	0	
ENSXETG00000030547	70	0	0	0	0	0	0	0	0	0	0	0	0	
ENSXETG00000030548	774	0	0	0	0	0	0	0	0	0	0	0	0	
ENSXETG00000030549	969	57	75	43	65	64	122	139	154	94	122	91	72	ENSXETG00000030549
ENSXETG00000030550	312	0	2	2	1	3	3	4	8	4	11	6	12	ENSXETG00000030550
ENSXETG00000030551	2535	0	0	0	0	0	0	0	0	0	0	0	0	
ENSXETG00000030552	2385	0	0	0	3	0	3	49	54	28	6	4	3	ENSXETG00000030552
ENSXETG00000030553	441	641	977	850	970	717	1060	1081	1182	743	780	538	594	mea1
ENSXETG00000030554	141	0	0	0	0	0	0	0	0	0	0	0	0	
ENSXETG00000030555	804	0	0	0	0	0	0	0	0	0	0	0	0	
ENSXETG00000030556	1872	0	1	0	0	0	0	0	0	0	0	0	0	
ENSXETG00000030557	1454	55	64	62	115	88	223	1467	1829	1018	293	186	206	ENSXETG00000030557
ENSXETG00000030558	477	0	0	0	0	0	0	0	0	0	0	0	0	
ENSXETG00000030559	459	0	0	0	0	0	0	0	0	0	0	0	1	
ENSXETG00000030560	1317	0	0	0	0	0	0	0	0	0	0	0	0	
ENSXETG00000030561	1014	0	1	0	1	0	0	0	1	1	0	0	0	ENSXETG00000030561
ENSXETG00000030562	627	31	55	43	81	65	221	427	606	354	103	69	87	slc29a3
ENSXETG00000030563	1017	0	1	1	0	0	0	0	0	0	0	0	0	
ENSXETG00000030564	543	1	1	1	7	2	7	0	0	0	0	2	0	hba-l5

ENSXETG00000030565	792	0	0	0	1	0	0	2	4	1	1	1	1	DRGX
ENSXETG00000030566	393	1	0	0	0	0	0	0	0	1	0	0	0	ENSXETG00000030566
ENSXETG00000030567	4007	238	295	272	716	600	1370	4502	5300	3118	2431	1663	1898	FERMT2
ENSXETG00000030568	1290	0	0	0	2	0	3	1	0	0	0	0	0	ENSXETG00000030568
ENSXETG00000030569	810	0	0	0	0	0	0	0	0	0	0	0	0	
ENSXETG00000030570	2770	0	0	0	0	0	0	0	0	0	2	3	0	
ENSXETG00000030571	130	0	0	0	0	0	0	0	0	0	0	0	0	
ENSXETG00000030572	753	83	122	77	84	40	102	208	239	132	60	38	68	CENPH
ENSXETG00000030573	299	23988	35594	29864	30657	23548	30814	31165	34070	20975	66226	46175	50304	rpl39
ENSXETG00000030574	1239	12	20	21	65	68	46	53	80	30	29	19	16	ENSXETG00000030574
ENSXETG00000030575	654	0	0	0	1	0	1	8	11	4	10	5	2	ENSXETG00000030575
ENSXETG00000030576	1596	5	7	7	31	23	20	10	14	10	47	33	46	ENSXETG00000030576
ENSXETG00000030577	1155	0	0	0	0	0	0	0	0	0	0	0	0	
ENSXETG00000030578	948	0	0	0	0	0	0	0	0	0	0	0	0	
ENSXETG00000030579	3129	35	42	25	18	20	34	14	15	7	25	22	28	TLR8
ENSXETG00000030580	2508	2	0	0	3	4	8	272	348	218	32	25	32	CHSY3
ENSXETG00000030581	2613	0	0	0	0	0	0	0	0	0	0	0	0	
ENSXETG00000030582	112	0	0	0	0	0	0	0	0	0	0	0	0	
ENSXETG00000030583	1311	0	0	0	0	0	0	0	0	0	0	0	0	
ENSXETG00000030584	1155	9	7	9	12	5	6	21	32	32	19	13	29	GRPR
ENSXETG00000030585	702	4	3	7	3	2	11	37	47	27	17	15	15	CD79A
ENSXETG00000030586	2316	0	0	0	0	0	0	0	0	0	0	0	0	
ENSXETG00000030587	4035	1580	2202	1927	3912	2831	4019	2327	2766	1649	1565	1088	1260	ATP10B
ENSXETG00000030588	1200	1	0	0	0	0	0	0	0	0	0	0	0	
ENSXETG00000030589	2400	83	123	109	143	98	541	304	363	185	247	171	192	CCSER2
ENSXETG00000030590	903	1	1	1	7	4	4	4	3	1	9	3	8	ENSXETG00000030590
ENSXETG00000030591	1407	2298	3218	2732	1702	1326	2789	220	198	131	1456	1029	1141	entpd5
ENSXETG00000030592	528	0	0	0	0	0	0	0	0	0	0	0	0	
ENSXETG00000030593	507	2	2	1	5	2	0	0	0	1	0	0	0	ENSXETG00000030593
ENSXETG00000030594	139	0	0	0	0	0	0	0	0	0	0	0	0	
ENSXETG00000030595	867	564	792	673	459	325	818	816	991	528	592	488	512	TNFSF10
ENSXETG00000030596	1528	135	195	179	464	308	427	3710	4269	2421	3369	2390	2613	ENSXETG00000030596
ENSXETG00000030597	5991	99	170	110	351	273	299	1006	1135	642	640	447	495	dip2a
ENSXETG00000030598	825	0	0	0	0	0	0	5	7	7	12	11	9	ENSXETG00000030598
ENSXETG00000030599	3765	229	297	232	233	208	478	465	553	298	436	290	334	pxylp1
ENSXETG00000030600	1230	32	48	32	95	77	171	136	194	100	136	89	121	ENSXETG00000030600
ENSXETG00000030601	1335	0	2	2	2	1	0	100	142	69	3	0	1	cpa6
ENSXETG00000030602	3948	0	0	0	0	0	0	4	2	0	1	1	2	
ENSXETG00000030603	2088	19	28	30	83	84	156	231	297	179	179	124	164	ENSXETG00000030603
ENSXETG00000030604	927	0	0	0	0	0	0	0	0	0	0	0	0	
ENSXETG00000030605	5823	0	0	0	85	86	4	49	77	39	5	6	8	ENSXETG00000030605
ENSXETG00000030606	1836	1	0	0	0	0	0	16	28	24	0	1	0	
ENSXETG00000030607	966	0	0	0	0	0	0	0	0	0	0	0	0	
ENSXETG00000030608	7986	314	536	426	739	643	1358	1099	1337	836	3111	2227	2472	ENSXETG00000030608
ENSXETG00000030609	2769	228	303	264	247	179	498	506	664	346	410	291	366	ENSXETG00000030609
ENSXETG00000030610	1359	0	0	0	0	0	1	0	5	0	0	1	0	SERINC4
ENSXETG00000030611	2165	1	1	1	27	38	3	1	0	0	1	3	1	LRRD1
ENSXETG00000030612	1143	0	0	0	0	1	0	1	0	0	1	0	0	ENSXETG00000030612
ENSXETG00000030613	684	67	124	96	171	147	343	228	293	143	248	179	186	ANKRD54
ENSXETG00000030614	1029	0	0	0	0	0	0	0	0	0	0	0	0	
ENSXETG00000030615	1203	0	1	0	0	0	0	0	0	0	1	2	0	ENSXETG00000030615
ENSXETG00000030616	924	0	0	0	0	0	0	0	0	0	0	0	0	
ENSXETG00000030617	1452	272	363	352	512	406	539	1815	2099	1234	528	340	414	sept8
ENSXETG00000030618	2382	0	0	0	0	0	0	0	0	0	0	0	1	
ENSXETG00000030619	119	0	0	0	0	0	0	0	0	0	0	0	0	
ENSXETG00000030620	855	0	0	0	0	0	0	4	6	3	2	0	2	
ENSXETG00000030621	942	0	0	0	0	0	0	0	0	0	0	0	0	
ENSXETG00000030622	1078	24	28	33	212	154	280	908	963	664	416	293	323	ENSXETG00000030622
ENSXETG00000030623	1317	3	8	3	33	21	19	53	60	35	27	24	25	abo
ENSXETG00000030624	703	357	521	385	241	235	216	6	7	4	8	4	2	gngt2.1
ENSXETG00000030625	1149	6	3	4	18	11	2	22	9	6	47	34	33	sept4
ENSXETG00000030626	1214	30	40	32	78	68	124	585	758	428	125	84	107	apln
ENSXETG00000030627	1806	89	153	117	221	159	225	270	324	176	210	143	163	ENSXETG00000030627
ENSXETG00000030628	1104	11	19	16	89	89	17	78	95	51	15	15	8	cep41
ENSXETG00000030629	513	0	0	0	0	0	0	0	0	0	0	0	0	
ENSXETG00000030630	372	0	0	0	0	0	0	6	6	1	1	3	4	ENSXETG00000030630
ENSXETG00000030631	762	402	559	478	682	491	1265	759	832	484	752	562	577	AMN1
ENSXETG00000030632	1121	46	49	47	147	105	61	162	218	123	113	100	91	ankrd22
ENSXETG00000030633	1221	0	0	0	0	0	0	0	0	0	0	0	0	
ENSXETG00000030634	1002	0	1	0	1	0	2	0	1	0	0	0	0	ENSXETG00000030634
ENSXETG00000030635	960	0	0	0	0	0	0	0	0	0	0	0	0	
ENSXETG00000030636	3219	0	0	0	0	0	0	0	0	0	0	0	0	
ENSXETG00000030637	2556	0	0	0	0	0	0	0	0	0	0	0	0	
ENSXETG00000030638	2064	4	0	5	15	7	41	65	77	66	102	68	70	FBXO40
ENSXETG00000030639	936	0	0	0	0	0	0	0	0	0	0	0	0	
ENSXETG00000030640	948	0	0	0	0	0	0	0	0	0	0	0	0	
ENSXETG00000030641	1788	99	146	163	256	175	429	395	502	307	885	627	684	ENSXETG00000030641
ENSXETG00000030642	1203	292	357	333	437	317	500	72	91	64	238	144	195	SGPP2
ENSXETG00000030643	939	0	0	0	1	0	0	1	1	0	0	1	1	
ENSXETG00000030644	2610	2480	3707	3216	4972	3828	6281	2017	2187	1433	3482	2466	2724	USH1C
ENSXETG00000030645	393	0	0	0	0	1	0	0	1	1	0	0	0	
ENSXETG00000030646	2298	0	0	0	0	0	0	0	0	0	0	0	0	
ENSXETG00000030647	1824	0	2	1	1	1	0	0	1	1	0	0	0	ENSXETG00000030647
ENSXETG00000030648	1681	0	0	0	1	1	3	91	127	60	26	15	22	ENSXETG00000030648
ENSXETG00000030649	1671	25	27	23	41	32	67	576	583	352	28	24	25	fcn1
ENSXETG00000030650	2348	0	1	0	3	4	0	0	1	0	29	25	27	npy
ENSXETG00000030651	3323	1	0	0	0	0	0	0	0	0	0	0	0	
ENSXETG00000030652	792	2	4	2	5	4	8	17	18	10	7	6	5	ENSXETG00000030652
ENSXETG00000030653	2783	0	0	0	1	1	2	0	0	0	1	0	0	
ENSXETG00000030654	1329	8	10	9	30	28	32	19	16	9	50	27	38	ENSXETG00000030654
ENSXETG00000030655	4007	2	3	1	10	4	22	573	724	397	232	144	197	prelp
ENSXETG00000030656	2903	105	160	140	191	140	202	347	408	238	209	129	184	ENSXETG00000030656
ENSXETG00000030657	3438	124	150	124	352	273	514	526	692	374	395	311	319	MPHOSPH9
ENSXETG00000030658	119	0	0	0	0	0	0	0	0	0	0	0	0	
ENSXETG00000030659	2043	3	10	4	110	82	68	361	422	255	159	114	148	ENSXETG00000030659
ENSXETG00000030660	393	0	1	0	0	0	0	3	0	0	0	0	0	ENSXETG00000030660
ENSXETG00000030661	1581	0	0	0	5	5	0	1	3	1	3	0	1	ENSXETG00000030661
ENSXETG00000030662	2562	0	1	0	0	0	0	0	0	0	0	0	0	
ENSXETG00000030663	1478	146	216	183	154	110	106	579	718	423	99	96	84	ENSXETG00000030663
ENSXETG00000030664	528	0	0	0	0	0	0	0	0	0	0	0	0	
ENSXETG00000030665	1149	0	0	0	0	0	0	0	0	0	0	0	0	
ENSXETG00000030666	70	0	0	0	0	0	0	0	0	0	0	0	0	
ENSXETG00000030667	495	0	0	0	0	0	0	0	0	0	0	0	0	
ENSXETG00000030668	930	0	0	0	0	0	0	0	0	0	0	0	0	
ENSXETG00000030669	294	0	0	0	0	0	0	0	0	0	0	0	0	
ENSXETG00000030670	2538	0	0	0	0	0	0	0	0	0	0	0	0	
ENSXETG00000030671	1440	610	928	756	499	395	454	642	785	483	701	592	551	RRP9
ENSXETG00000030672	1287	93	173	118	228	182	317	614	696	415	525	338	362	ZBTB12
ENSXETG00000030673	4812	29	41	36	148	93	260	608	743	454	271	207	222	PREX2
ENSXETG00000030674	945	0	0	0	0	0	0	0	0	0	0	0	0	
ENSXETG00000030675	757	140	223	160	3303	2435	1302	5722	7157	3866	3822	2723	3137	mt4

ENSXETG00000030676	312	0	0	0	0	0	0	0	0	0	0	0	0	
ENSXETG00000030677	1056	0	0	0	0	0	0	0	0	0	0	0	0	
ENSXETG00000030678	1001	778	1162	930	1677	1347	1027	1335	1637	869	297	219	246	ENSXETG00000030678
ENSXETG00000030679	2629	13	11	10	53	47	73	485	561	354	139	75	111	pde1a
ENSXETG00000030680	351	0	0	0	0	0	0	0	0	0	0	0	0	
ENSXETG00000030681	1896	0	0	0	3	3	6	2	7	1	7	8	1	ENSXETG00000030681
ENSXETG00000030682	1272	1678	2453	1970	638	433	114	52	60	34	112	58	80	ENSXETG00000030682
ENSXETG00000030683	1280	127	201	175	11	2	85	146	140	70	20	9	12	ENSXETG00000030683
ENSXETG00000030684	805	5	5	5	78	45	8	10	16	6	0	0	0	ENSXETG00000030684
ENSXETG00000030685	154	0	0	0	0	0	3	1	3	2	8	1	3	5_8S_rRNA
ENSXETG00000030686	558	243	330	288	229	200	464	559	646	454	389	317	300	ENSXETG00000030686
ENSXETG00000030687	1122	274	384	335	163	142	290	346	350	277	430	324	352	ENSXETG00000030687
ENSXETG00000030688	1329	0	0	0	0	0	0	0	0	0	0	0	0	
ENSXETG00000030689	1005	0	0	0	16	9	32	1	2	0	39	40	35	
ENSXETG00000030690	264	23	25	12	24	13	35	31	31	16	45	29	24	ENSXETG00000030690
ENSXETG00000030691	1053	0	0	0	0	0	0	0	0	0	0	0	0	ENSXETG00000030691
ENSXETG00000030692	3657	97	142	139	530	378	705	239	314	180	611	479	522	SH3TC2
ENSXETG00000030693	5001	3	9	5	58	42	17	24	21	22	7	7	7	NRAP
ENSXETG00000030694	141	0	0	0	0	0	0	0	0	0	0	0	0	
ENSXETG00000030695	489	0	0	0	0	0	0	0	0	0	0	0	0	
ENSXETG00000030696	1992	71	99	89	66	67	129	108	106	45	147	104	127	ERMARD
ENSXETG00000030697	119	0	0	0	0	0	0	0	0	0	0	0	0	
ENSXETG00000030698	1065	0	0	0	0	0	0	0	0	0	0	0	0	
ENSXETG00000030699	1614	4	7	5	11	4	12	31	27	17	12	15	10	ENSXETG00000030699
ENSXETG00000030700	1095	0	0	0	0	0	0	0	0	0	0	0	0	
ENSXETG00000030701	1563	0	0	1	1	0	1	1	4	0	4	3	2	ENSXETG00000030701
ENSXETG00000030702	119	0	0	0	0	0	0	0	0	0	0	0	0	
ENSXETG00000030703	1176	0	0	0	0	0	0	4	1	3	3	2	0	ENSXETG00000030703
ENSXETG00000030704	84	0	0	0	0	0	0	0	0	0	0	0	0	
ENSXETG00000030705	1581	765	1141	1003	223	153	419	326	416	238	214	133	147	ENSXETG00000030705
ENSXETG00000030706	513	0	0	0	0	0	0	0	0	0	0	0	0	
ENSXETG00000030707	936	0	0	0	0	0	0	0	0	0	0	0	0	
ENSXETG00000030708	772	39	45	35	76	71	47	67	91	49	28	13	16	ENSXETG00000030708
ENSXETG00000030709	1887	124	180	149	252	170	377	395	464	274	306	233	233	ZSWIM3
ENSXETG00000030710	1519	0	0	0	2	0	0	2	1	0	0	0	0	
ENSXETG00000030711	930	0	0	0	0	0	0	0	1	0	0	0	0	
ENSXETG00000030712	708	0	0	0	0	0	0	0	0	0	0	0	0	
ENSXETG00000030713	1071	0	0	0	0	0	0	0	0	0	0	0	0	
ENSXETG00000030714	1239	0	0	0	1	0	0	1	0	0	0	1	1	
ENSXETG00000030715	70	0	0	0	0	0	0	0	0	0	0	0	0	
ENSXETG00000030716	2326	645	970	808	674	523	898	860	1079	629	621	416	485	INTS11
ENSXETG00000030717	501	0	0	0	0	0	0	0	0	0	0	0	0	
ENSXETG00000030718	1062	0	0	1	3	5	3	0	0	0	2	0	2	ENSXETG00000030718
ENSXETG00000030719	616	0	0	0	0	0	0	17	22	17	3	0	0	
ENSXETG00000030720	1425	1	0	1	64	58	7	0	2	0	12	11	9	MINDY4B
ENSXETG00000030721	657	132	156	169	87	72	104	243	251	131	85	65	51	ENSXETG00000030721
ENSXETG00000030722	2394	0	0	0	0	1	0	0	0	0	0	0	0	
ENSXETG00000030723	897	50	96	68	33	29	126	37	49	24	38	33	31	ENSXETG00000030723
ENSXETG00000030724	381	0	0	0	0	0	0	2	2	2	0	1	0	ENSXETG00000030724
ENSXETG00000030725	1338	98	155	134	143	127	99	308	352	207	188	145	162	TMEM44
ENSXETG00000030726	1356	72	106	71	187	126	109	180	177	120	79	60	59	RCCD1
ENSXETG00000030727	882	1	2	1	3	0	1	0	1	0	2	1	0	ENSXETG00000030727
ENSXETG00000030728	164	0	0	0	0	0	0	0	0	0	0	0	0	
ENSXETG00000030729	2573	495	769	624	543	408	465	877	1070	566	683	495	544	ENSXETG00000030729
ENSXETG00000030730	844	0	0	0	0	0	0	0	0	0	0	0	0	
ENSXETG00000030731	933	0	0	0	0	0	0	0	0	0	0	0	0	
ENSXETG00000030732	1311	0	0	0	0	0	0	0	0	0	0	0	0	
ENSXETG00000030733	1758	2935	4091	3494	4114	3169	8080	6857	8606	4772	6612	4703	5475	BZW1
ENSXETG00000030734	606	0	0	0	0	0	0	0	0	0	0	0	0	
ENSXETG00000030735	119	0	0	0	0	0	0	0	0	0	0	0	0	
ENSXETG00000030736	867	0	0	0	0	0	0	0	0	0	0	0	0	
ENSXETG00000030737	498	180	267	265	618	463	516	667	748	527	605	409	509	ENSXETG00000030737
ENSXETG00000030738	1435	0	0	0	0	0	2	25	21	12	14	7	14	c17orf67
ENSXETG00000030739	951	0	0	0	1	0	0	1	0	0	0	0	0	
ENSXETG00000030740	2238	19796	28360	23649	19020	14645	15134	2818	3192	1968	1792	1292	1445	ENSXETG00000030740
ENSXETG00000030741	1071	57	94	80	120	98	150	211	230	150	271	159	159	ENSXETG00000030741
ENSXETG00000030742	948	0	0	0	0	0	0	0	0	0	0	0	0	
ENSXETG00000030743	664	1826	2470	2007	4821	3683	2769	1032	1359	789	1657	1121	1291	ENSXETG00000030743
ENSXETG00000030744	2075	1	0	0	2	1	1	3	1	0	1	0	0	MGC145330
ENSXETG00000030745	795	0	0	0	0	0	0	0	0	0	0	0	0	ENSXETG00000030745
ENSXETG00000030746	1476	0	1	0	0	0	0	2	3	0	0	0	0	
ENSXETG00000030747	474	0	0	0	0	0	0	0	0	0	0	0	0	
ENSXETG00000030748	891	0	0	0	0	0	0	0	0	0	0	0	0	
ENSXETG00000030749	995	184	295	248	342	333	757	420	486	297	617	422	495	ENSXETG00000030749
ENSXETG00000030750	771	0	0	0	88	56	324	0	2	2	224	137	174	ENSXETG00000030750
ENSXETG00000030751	119	0	0	0	0	0	0	0	0	0	0	0	0	
ENSXETG00000030752	498	0	0	0	0	0	0	0	0	0	0	0	0	
ENSXETG00000030753	141	0	0	0	0	0	0	0	0	0	0	0	0	
ENSXETG00000030754	507	34	56	44	0	0	0	0	2	0	0	0	0	
ENSXETG00000030755	1191	24	43	45	108	88	174	746	875	525	246	158	189	RIMKLB
ENSXETG00000030756	837	2	10	4	5	1	7	27	54	39	15	3	3	ENSXETG00000030756
ENSXETG00000030757	567	249	390	305	386	241	386	93	92	60	422	255	324	ENSXETG00000030757
ENSXETG00000030758	1611	1071	1434	1190	1434	1144	462	214	243	137	246	166	205	ENSXETG00000030758
ENSXETG00000030759	1339	12	19	9	276	229	114	56	55	34	1051	720	849	rtp3
ENSXETG00000030760	2616	0	0	0	0	0	0	0	0	0	0	0	0	
ENSXETG00000030761	1022	250	444	369	831	641	1543	1137	1322	736	1874	1294	1458	KIAA1191
ENSXETG00000030762	1611	111	140	146	444	286	711	452	478	294	784	596	615	ENSXETG00000030762
ENSXETG00000030763	1005	0	0	0	0	0	0	0	0	0	0	0	0	
ENSXETG00000030764	1621	1693	2544	2171	10697	8122	3479	7121	8249	4677	1627	1156	1308	lgals9c
ENSXETG00000030765	441	0	0	0	0	0	0	0	0	0	0	0	0	
ENSXETG00000030766	186	0	0	0	15	11	0	1	0	0	1	2	2	ENSXETG00000030766
ENSXETG00000030767	1299	0	0	0	0	0	0	0	0	0	0	0	0	
ENSXETG00000030768	1872	75	101	82	190	164	284	276	306	176	413	293	312	ENSXETG00000030768
ENSXETG00000030769	831	1	0	1	3	2	25	19	33	23	22	22	27	CREG2
ENSXETG00000030770	1137	0	1	1	0	0	1	13	16	7	1	0	4	ENSXETG00000030770
ENSXETG00000030771	639	5	18	14	56	39	26	25	30	23	36	36	26	PLAC8
ENSXETG00000030772	525	15	24	22	68	66	11	49	55	42	73	47	51	BRICD5
ENSXETG00000030773	510	0	0	0	0	0	0	0	0	0	0	0	0	
ENSXETG00000030774	1095	0	0	0	0	0	0	0	2	0	0	1	0	ENSXETG00000030774
ENSXETG00000030775	1605	10	28	15	17	12	52	267	319	194	54	27	26	ENSXETG00000030775
ENSXETG00000030776	2112	404	543	496	610	466	679	690	797	449	527	385	487	pnkp
ENSXETG00000030777	7331	78	128	102	527	391	1042	1545	1709	1032	1254	869	1033	shroom4
ENSXETG00000030778	699	1	0	2	1	4	2	0	0	1	0	1	1	CLRN2
ENSXETG00000030779	2313	0	0	0	0	0	0	1	5	1	2	1	2	ENSXETG00000030779
ENSXETG00000030780	702	4	6	1	142	124	117	72	61	50	110	92	76	UBXN10
ENSXETG00000030781	1897	20	23	19	86	80	39	39	45	27	29	35	30	IQCG
ENSXETG00000030782	1206	0	0	0	0	0	0	0	0	0	0	0	0	
ENSXETG00000030783	411	14	26	28	40	43	47	39	42	13	65	59	61	ENSXETG00000030783
ENSXETG00000030784	902	73	103	80	48	43	57	59	95	37	57	40	43	lyrm7
ENSXETG00000030785	786	5	8	6	1	1	4	18	20	6	1	0	0	ENSXETG00000030785
ENSXETG00000030786	1752	0	0	0	0	0	0	0	0	0	0	0	0	

ENSXETG00000030787	1888	507	748	606	809	648	1742	1172	1475	795	875	593	642	mpzl3
ENSXETG00000030788	1889	12	12	16	235	174	274	1736	2051	1261	1023	726	783	vipr2
ENSXETG00000030789	1511	3	6	3	0	4	3	11	12	9	1	1	3	susd3
ENSXETG00000030790	1131	6	3	2	7	10	19	293	314	180	36	27	21	ADRB3
ENSXETG00000030791	2736	20	28	24	88	88	249	442	613	281	220	178	232	MMRN2
ENSXETG00000030792	2178	0	0	0	9	10	2	21	26	11	18	22	18	SV2C
ENSXETG00000030793	3038	0	0	0	0	0	0	0	0	0	0	0	0	
ENSXETG00000030794	711	0	0	0	0	0	0	0	0	0	0	0	0	
ENSXETG00000030795	330	0	0	0	0	1	0	0	0	0	0	0	0	
ENSXETG00000030796	214	0	0	0	0	0	0	0	0	0	0	0	0	
ENSXETG00000030797	2328	0	0	0	0	0	0	0	0	0	0	0	0	
ENSXETG00000030798	507	0	0	0	0	0	0	0	0	0	0	0	0	
ENSXETG00000030799	2244	588	923	830	752	550	1210	941	1053	611	1307	950	1065	ENSXETG00000030799
ENSXETG00000030800	119	0	0	0	0	0	0	0	0	0	0	0	0	5S_rRNA
ENSXETG00000030801	954	2	2	5	0	1	5	2	2	3	7	3	3	ENSXETG00000030801
ENSXETG00000030802	948	0	0	0	0	0	0	0	0	0	0	0	0	
ENSXETG00000030803	1461	1	0	4	7	7	7	118	150	64	34	20	24	tmeff2
ENSXETG00000030804	498	0	0	0	0	0	0	0	0	0	0	0	0	
ENSXETG00000030805	2435	0	0	0	0	0	0	0	0	0	0	0	0	
ENSXETG00000030806	921	0	0	0	0	0	0	0	0	0	0	0	0	
ENSXETG00000030807	119	0	0	0	0	0	0	0	0	0	0	0	0	
ENSXETG00000030808	1667	3	1	0	29	34	61	28	27	9	8	3	14	ENSXETG00000030808
ENSXETG00000030809	492	0	0	0	0	0	0	0	0	0	0	0	0	
ENSXETG00000030810	84	2	2	0	0	0	0	0	1	0	2	0	0	SNORD103
ENSXETG00000030811	936	0	0	0	0	0	0	0	0	0	0	0	0	
ENSXETG00000030812	1182	1	3	5	18	10	5	3	3	1	2	1	3	ENSXETG00000030812
ENSXETG00000030813	6765	321	505	410	2170	1471	4384	4806	5224	3141	4348	3093	3303	ENSXETG00000030813
ENSXETG00000030814	1422	0	0	0	0	0	1	0	0	0	0	0	0	
ENSXETG00000030815	465	0	0	0	0	0	0	1	1	0	0	0	0	
ENSXETG00000030816	606	0	0	0	0	0	0	0	0	0	0	0	0	
ENSXETG00000030817	1524	0	0	0	0	0	0	3	0	0	2	1	1	ENSXETG00000030817
ENSXETG00000030818	714	1	1	3	0	0	1	2	4	1	0	0	0	
ENSXETG00000030819	528	0	0	0	0	0	0	0	0	0	0	0	0	
ENSXETG00000030820	119	0	0	0	0	0	0	0	0	0	0	0	0	
ENSXETG00000030821	1554	0	0	0	0	1	0	0	1	0	5	5	2	ENSXETG00000030821
ENSXETG00000030822	4586	458	663	545	1503	1124	2111	2003	2421	1417	1780	1266	1352	ankrd46
ENSXETG00000030823	2023	73	83	71	180	147	309	639	721	423	515	381	384	rspo1
ENSXETG00000030824	681	137	176	128	82	66	49	43	28	15	196	115	135	ENSXETG00000030824
ENSXETG00000030825	1365	33459	45790	36842	38896	30353	56737	356	468	236	2959	2020	2436	ENSXETG00000030825
ENSXETG00000030826	486	0	0	0	0	3	0	0	1	1	2	8	1	TMEM182
ENSXETG00000030827	717	0	0	0	1	0	0	0	0	0	0	0	0	
ENSXETG00000030828	522	0	1	0	0	0	0	1	0	0	0	0	2	ENSXETG00000030828
ENSXETG00000030829	1236	221	332	239	160	124	339	550	660	365	283	195	203	KIAA1147
ENSXETG00000030830	738	1	1	1	4	1	0	2	1	1	1	2	1	ENSXETG00000030830
ENSXETG00000030831	119	0	0	0	0	0	0	0	0	0	0	0	0	
ENSXETG00000030832	2119	2	2	3	0	1	1	12	14	3	11	4	9	ddx4
ENSXETG00000030833	96	0	1	0	0	0	0	1	0	0	0	0	0	SNORD14
ENSXETG00000030834	2397	4	5	6	0	0	19	11	16	7	47	25	43	ARHGEF33
ENSXETG00000030835	384	0	0	0	0	0	0	35	27	12	4	3	6	ENSXETG00000030835
ENSXETG00000030836	70	0	0	0	0	0	0	0	0	0	0	0	0	
ENSXETG00000030837	118	0	0	0	0	0	0	0	0	0	0	0	0	
ENSXETG00000030838	438	2	1	0	2	4	0	2	0	1	83	59	50	
ENSXETG00000030839	566	222	267	226	247	212	325	402	515	263	237	181	217	ISCA1
ENSXETG00000030840	519	0	0	0	0	0	0	0	0	0	0	0	0	
ENSXETG00000030841	3511	48	111	71	120	91	179	377	485	291	243	138	204	GRK3
ENSXETG00000030842	1404	0	1	0	2	1	1	8	9	6	4	3	2	ENSXETG00000030842
ENSXETG00000030843	654	370	492	438	1194	830	2585	139	168	106	707	498	549	ENSXETG00000030843
ENSXETG00000030844	2428	586	670	626	765	613	1002	1357	1575	964	1519	1087	1155	ENSXETG00000030844
ENSXETG00000030845	942	0	0	0	0	0	0	0	0	0	0	0	0	
ENSXETG00000030846	585	1	1	1	0	2	0	0	2	1	0	0	0	ENSXETG00000030846
ENSXETG00000030847	1185	6	12	6	0	0	8	25	30	7	31	14	12	
ENSXETG00000030848	1677	180	271	216	1195	976	2828	2214	2603	1476	3110	2159	2333	ENSXETG00000030848
ENSXETG00000030849	2392	419	614	504	509	408	962	646	813	496	559	451	540	UBXN8
ENSXETG00000030850	1406	144	194	186	224	173	448	258	330	187	303	198	205	ENSXETG00000030850
ENSXETG00000030851	1626	365	448	423	615	408	593	702	786	508	383	261	275	ENSXETG00000030851
ENSXETG00000030852	164	0	0	0	0	0	0	0	0	0	0	0	0	
ENSXETG00000030853	813	0	1	0	0	0	0	0	0	0	0	1	0	
ENSXETG00000030854	1962	11	10	11	17	13	39	44	55	39	66	43	49	ENSXETG00000030854
ENSXETG00000030855	1413	0	0	1	1	0	1	0	0	0	0	0	0	
ENSXETG00000030856	660	523	760	636	121	81	249	639	790	469	68	55	66	ENSXETG00000030856
ENSXETG00000030857	70	0	0	0	0	0	0	0	0	0	0	0	0	
ENSXETG00000030858	1170	0	1	0	1	1	0	0	1	0	0	0	0	
ENSXETG00000030859	1206	6	8	7	37	18	27	139	166	91	8	9	10	ACOD1
ENSXETG00000030860	3336	1	1	0	3	1	14	0	0	0	1	1	0	ENSXETG00000030860
ENSXETG00000030861	1686	16	23	21	14	16	21	38	39	34	32	44	29	ENSXETG00000030861
ENSXETG00000030862	528	0	0	0	0	0	0	0	0	0	0	0	0	
ENSXETG00000030863	420	56	84	66	403	318	1077	622	727	451	991	680	764	ENSXETG00000030863
ENSXETG00000030864	456	0	0	0	0	0	0	0	0	0	0	0	0	
ENSXETG00000030865	996	6	16	10	5	4	4	53	61	38	5	2	8	ENSXETG00000030865
ENSXETG00000030866	2804	282	402	389	1248	939	2638	2424	2528	1616	3182	2188	2575	ENSXETG00000030866
ENSXETG00000030867	1026	4	6	8	2	4	13	25	13	13	16	11	10	ENSXETG00000030867
ENSXETG00000030868	107	0	0	0	0	0	0	0	0	0	0	0	0	
ENSXETG00000030869	1629	99	153	147	276	250	178	221	262	134	326	223	249	PRICKLE3
ENSXETG00000030870	459	0	0	0	0	0	0	0	0	0	0	0	0	
ENSXETG00000030871	1554	11	14	22	72	56	148	228	275	158	259	172	191	ENSXETG00000030871
ENSXETG00000030872	1122	0	0	0	0	0	0	1	0	2	1	3	0	ENSXETG00000030872
ENSXETG00000030873	1026	0	0	0	1	0	0	0	0	0	0	0	0	
ENSXETG00000030874	1627	33	47	39	73	69	101	173	204	97	162	113	137	xkr8
ENSXETG00000030875	4101	179	276	211	167	134	35	82	113	84	117	98	118	ENSXETG00000030875
ENSXETG00000030876	115	0	0	0	0	0	0	0	0	0	0	0	0	
ENSXETG00000030877	1179	10	4	10	33	29	67	304	346	221	70	52	60	ENSXETG00000030877
ENSXETG00000030878	424	0	0	3	45	24	0	3	2	0	10	7	2	NHLRC4
ENSXETG00000030879	598	45	59	36	50	43	40	61	64	44	18	14	11	efcab11
ENSXETG00000030880	435	27	25	20	13	11	55	204	219	119	126	70	88	CD247
ENSXETG00000030881	513	0	0	0	0	0	0	0	0	0	0	0	0	
ENSXETG00000030882	2371	215	276	227	233	195	238	323	404	233	214	185	178	TRMT44
ENSXETG00000030883	5411	10	13	10	177	96	34	103	116	53	23	15	18	ankmy1
ENSXETG00000030884	1344	189	311	218	300	235	701	417	458	264	700	520	567	C19orf54
ENSXETG00000030885	1428	668	1032	779	861	684	1504	108	130	82	407	309	318	ENSXETG00000030885
ENSXETG00000030886	1582	28	55	46	76	69	58	144	126	88	97	59	81	NSL1
ENSXETG00000030887	954	0	0	0	0	0	0	0	0	0	0	0	0	
ENSXETG00000030888	1784	141	199	152	325	208	618	825	919	564	533	367	414	sept5
ENSXETG00000030889	891	152	253	166	362	245	426	172	209	124	370	300	315	ENSXETG00000030889
ENSXETG00000030890	1509	0	0	0	0	0	0	308	390	208	311	242	279	
ENSXETG00000030891	945	0	0	0	0	0	0	0	0	0	0	0	0	
ENSXETG00000030892	119	0	0	0	0	0	0	0	0	0	0	0	0	
ENSXETG00000030893	1491	42	70	47	168	117	234	262	293	180	144	115	138	ENSXETG00000030893
ENSXETG00000030894	252	1	0	0	1	0	0	0	1	0	0	0	0	ENSXETG00000030894
ENSXETG00000030895	1188	1	3	3	2	4	3	4	9	3	7	5	6	ENSXETG00000030895
ENSXETG00000030896	522	0	1	0	0	1	1	7	6	4	0	0	0	TMEM240
ENSXETG00000030897	96	4	7	9	1	1	5	25	12	7	9	5	3	SNORD14

ENSXETG00000030898	417	0	0	0	0	0	0	0	3	1	3	2	2	
ENSXETG00000030899	743	3261	4818	4075	1098	820	1952	3954	4291	2683	520	378	470	hn1
ENSXETG00000030900	348	0	0	0	0	0	0	2	1	2	0	0	0	ENSXETG00000030900
ENSXETG00000030901	572	195	247	233	152	110	240	176	219	143	231	170	160	COX16
ENSXETG00000030902	1695	445	657	556	90	62	68	235	246	158	50	38	37	ENSXETG00000030902
ENSXETG00000030903	2804	188	290	234	815	640	1359	546	644	347	824	629	705	slc25a16
ENSXETG00000030904	846	5	10	12	41	26	14	37	41	28	24	20	7	ENSXETG00000030904
ENSXETG00000030905	921	0	0	0	0	0	0	0	1	0	1	0	0	
ENSXETG00000030906	73	0	0	0	0	0	0	0	0	0	0	0	0	
ENSXETG00000030907	997	2	2	0	1	1	1	23	18	10	8	4	2	ENSXETG00000030907
ENSXETG00000030908	672	4	6	2	45	41	88	278	290	163	97	70	71	ENSXETG00000030908
ENSXETG00000030909	1746	33	59	57	167	124	371	276	283	206	202	146	169	fpgt
ENSXETG00000030910	312	0	0	0	0	0	1	0	1	3	0	0	1	
ENSXETG00000030911	567	0	0	0	0	0	0	0	1	0	0	0	0	
ENSXETG00000030912	852	1	0	0	0	0	0	0	0	0	0	0	0	
ENSXETG00000030913	119	0	0	0	0	0	0	0	0	0	0	0	0	
ENSXETG00000030914	177	4	2	3	0	0	1	0	1	0	2	3	1	SNORA53
ENSXETG00000030915	825	0	0	0	0	0	1	0	0	0	1	0	0	ENSXETG00000030915
ENSXETG00000030916	141	0	0	0	0	0	0	0	0	0	0	0	0	
ENSXETG00000030917	906	0	0	0	0	0	0	0	1	0	0	0	0	
ENSXETG00000030918	67	2	5	10	3	4	0	6	7	1	10	2	8	SNORD58
ENSXETG00000030919	948	0	0	0	0	0	0	0	0	0	0	0	0	
ENSXETG00000030920	4348	1	0	1	9	7	28	39	55	37	49	32	33	ATP2B3
ENSXETG00000030921	2253	4	2	3	29	18	30	306	341	226	56	43	35	KIRREL1
ENSXETG00000030922	540	0	0	0	0	0	0	0	0	0	0	0	0	
ENSXETG00000030923	1202	1	1	0	0	1	7	19	21	11	8	13	18	ENSXETG00000030923
ENSXETG00000030924	1477	65	92	87	137	145	280	278	289	186	366	249	302	ENSXETG00000030924
ENSXETG00000030925	3300	1140	1645	1395	1674	1360	3688	2308	2613	1625	3076	2219	2315	TEX2
ENSXETG00000030926	654	0	0	0	1	2	1	1	4	0	4	7	2	ENSXETG00000030926
ENSXETG00000030927	921	0	0	0	0	0	0	0	0	0	0	0	0	
ENSXETG00000030928	468	0	0	0	0	0	0	0	0	1	0	0	0	
ENSXETG00000030929	1320	1	1	1	0	0	0	1	5	5	0	0	0	ENSXETG00000030929
ENSXETG00000030930	1044	0	0	0	0	0	0	0	0	0	0	0	0	
ENSXETG00000030931	717	0	0	0	0	0	0	0	0	0	0	0	0	
ENSXETG00000030932	2421	1	2	1	10	7	12	46	52	31	31	20	25	ptgfr
ENSXETG00000030933	141	0	0	0	0	0	0	0	0	0	0	0	0	
ENSXETG00000030934	504	0	0	0	0	0	0	1	0	0	0	0	0	
ENSXETG00000030935	702	0	0	0	0	0	0	0	0	0	0	0	0	
ENSXETG00000030936	312	0	0	0	0	0	0	0	0	0	0	0	0	
ENSXETG00000030937	2056	3	11	7	56	56	50	33	48	24	48	25	40	ENSXETG00000030937
ENSXETG00000030938	2011	91	110	81	197	151	146	313	354	194	328	208	245	rab41
ENSXETG00000030939	1083	141	195	149	159	116	236	227	217	141	168	128	148	ENSXETG00000030939
ENSXETG00000030940	528	2	2	4	0	0	0	0	1	1	1	0	1	il22
ENSXETG00000030941	1725	384	569	389	484	354	735	907	950	612	633	442	478	zdhhc5
ENSXETG00000030942	447	42	57	56	29	17	111	86	91	68	4	9	11	ENSXETG00000030942
ENSXETG00000030943	2166	2	1	3	4	2	6	23	18	10	10	4	8	ENSXETG00000030943
ENSXETG00000030944	498	0	0	0	0	0	0	0	0	0	0	0	0	
ENSXETG00000030945	1574	262	364	325	316	249	511	702	849	490	427	277	342	ENSXETG00000030945
ENSXETG00000030946	2325	0	0	0	0	0	0	1	0	0	0	0	0	ENSXETG00000030946
ENSXETG00000030947	1812	561	784	656	666	553	873	1393	1577	880	601	431	487	cops4
ENSXETG00000030948	270	0	0	0	0	1	0	0	0	0	1	2	2	ENSXETG00000030948
ENSXETG00000030949	636	2892	4087	3637	3512	2846	5140	3431	3733	2503	4618	3313	3828	ENSXETG00000030949
ENSXETG00000030950	918	0	0	0	0	0	0	0	0	0	0	0	0	
ENSXETG00000030951	214	0	0	0	0	0	0	0	0	0	0	0	0	
ENSXETG00000030952	1126	2576	3496	2951	4438	3431	5410	421	549	279	1376	968	1088	ENSXETG00000030952
ENSXETG00000030953	3360	176	257	223	909	661	1397	2599	3002	1775	2666	1830	2026	FILIP1L
ENSXETG00000030954	1188	0	0	0	0	0	0	0	0	0	0	0	0	
ENSXETG00000030955	83	1	2	0	0	0	1	1	1	0	1	0	1	ENSXETG00000030955
ENSXETG00000030956	444	3127	4542	3973	4066	3063	3439	3146	3483	2121	3561	2596	2634	NDUFB9
ENSXETG00000030957	1494	32	57	51	42	32	33	29	40	16	49	30	33	ENSXETG00000030957
ENSXETG00000030958	3434	0	0	0	0	1	1	5	9	2	0	0	0	
ENSXETG00000030959	393	0	0	0	0	0	0	1	2	0	0	0	0	ENSXETG00000030959
ENSXETG00000030960	119	0	0	0	0	0	0	0	0	0	0	0	0	
ENSXETG00000030961	1656	43	80	67	213	155	274	348	417	292	289	229	265	TBC1D25
ENSXETG00000030962	1977	258	425	369	1826	1437	367	89	108	66	91	49	63	LONRF3
ENSXETG00000030963	1605	0	0	0	0	0	0	26	35	19	2	0	0	SLCO1C1
ENSXETG00000030964	70	0	0	0	0	0	0	0	0	0	0	0	0	
ENSXETG00000030965	1786	245	352	279	1021	736	690	17923	18908	11246	308	192	245	ENSXETG00000030965
ENSXETG00000030966	654	0	1	1	3	2	2	4	2	3	6	1	6	ENSXETG00000030966
ENSXETG00000030967	747	25	44	39	60	47	62	153	131	89	118	91	86	ENSXETG00000030967
ENSXETG00000030968	3570	53	80	82	97	66	236	130	177	104	151	117	102	slx4ip
ENSXETG00000030969	1404	8	17	8	1575	1126	549	7	7	12	680	498	588	ENSXETG00000030969
ENSXETG00000030970	1479	0	0	0	0	0	1	0	0	0	0	0	0	ENSXETG00000030970
ENSXETG00000030971	513	0	1	0	0	0	0	3	2	0	0	0	1	ENSXETG00000030971
ENSXETG00000030972	119	0	0	0	0	0	0	0	0	0	0	0	0	
ENSXETG00000030973	107	0	0	0	0	0	0	0	0	0	0	0	0	
ENSXETG00000030974	294	3	2	7	1	3	3	7	13	5	7	4	6	ENSXETG00000030974
ENSXETG00000030975	492	3	4	1	3	1	2	4	5	2	1	0	1	ENSXETG00000030975
ENSXETG00000030976	5133	236	302	302	474	328	631	861	1126	581	665	489	590	USP6NL
ENSXETG00000030977	375	6	9	12	17	13	28	46	50	31	50	32	44	ENSXETG00000030977
ENSXETG00000030978	3726	13	9	13	231	154	50	24	19	7	20	14	13	CFAP57
ENSXETG00000030979	930	0	0	0	0	0	0	0	0	0	0	0	0	
ENSXETG00000030980	296	0	0	0	0	0	0	0	0	0	0	0	0	
ENSXETG00000030981	714	5	8	7	4	5	1	0	0	0	4	1	3	HELT
ENSXETG00000030982	945	0	0	0	0	0	0	0	0	0	0	0	0	
ENSXETG00000030983	735	14	12	10	34	34	48	106	136	77	35	28	32	RASL11A
ENSXETG00000030984	729	274	428	334	339	258	381	460	479	250	295	218	232	ENSXETG00000030984
ENSXETG00000030985	2421	0	0	0	0	0	0	0	0	0	0	0	0	
ENSXETG00000030986	4503	92	107	92	129	88	208	249	288	150	303	210	229	ENSXETG00000030986
ENSXETG00000030987	2060	1	0	0	0	0	0	1	1	0	0	0	0	
ENSXETG00000030988	684	5	13	5	12	3	13	11	10	4	8	4	9	ENSXETG00000030988
ENSXETG00000030989	2349	0	0	0	1	0	1	0	0	1	0	0	0	ENSXETG00000030989
ENSXETG00000030990	606	0	0	0	0	0	0	0	0	0	0	0	0	
ENSXETG00000030991	1201	140	194	195	120	103	124	139	195	104	121	73	89	ENSXETG00000030991
ENSXETG00000030992	1523	0	2	0	2	1	4	14	25	9	3	3	3	ENSXETG00000030992
ENSXETG00000030993	453	0	0	0	0	0	0	0	0	0	0	0	0	
ENSXETG00000030994	3383	247	356	301	348	283	1215	715	785	461	745	597	620	RUBCN
ENSXETG00000030995	645	29	44	35	74	50	36	70	82	49	21	14	19	STARD5
ENSXETG00000030996	1380	1	0	1	20	9	2	16	10	7	14	9	7	ENSXETG00000030996
ENSXETG00000030997	2529	0	0	0	0	0	0	0	0	0	0	0	0	
ENSXETG00000030998	387	1	1	0	1	1	1	6	9	4	1	0	1	ENSXETG00000030998
ENSXETG00000030999	1388	18	26	31	320	242	507	80	74	45	658	469	522	ENSXETG00000030999
ENSXETG00000031000	3062	84	116	98	227	150	52	398	494	332	55	31	33	ENSXETG00000031000
ENSXETG00000031001	119	0	0	0	0	0	0	0	0	0	0	0	0	
ENSXETG00000031002	591	0	0	0	0	0	0	0	0	1	1	1	0	
ENSXETG00000031003	582	0	0	0	0	0	0	0	0	0	0	0	0	
ENSXETG00000031004	4458	0	3	2	1	0	2	55	65	26	13	7	14	CD109
ENSXETG00000031005	966	0	0	0	0	0	0	0	0	0	0	0	0	
ENSXETG00000031006	1053	5	12	10	11	17	20	2	0	0	1	1	4	ENSXETG00000031006
ENSXETG00000031007	1270	95	131	114	113	79	225	282	294	214	127	91	113	ENSXETG00000031007
ENSXETG00000031008	942	0	0	0	0	0	0	0	0	0	0	0	0	

ENSXETG00000031009	1886	1	1	0	1	2	1	6	13	7	5	1	4	ENSXETG00000031009
ENSXETG00000031010	963	0	0	0	0	0	0	0	0	0	0	0	0	
ENSXETG00000031011	164	0	0	0	0	0	0	0	0	0	0	0	0	
ENSXETG00000031012	618	0	1	1	0	1	3	25	28	19	4	1	12	ENSXETG00000031012
ENSXETG00000031013	711	0	0	0	0	0	0	0	0	0	0	0	1	
ENSXETG00000031014	507	0	0	0	17	19	77	0	0	1	98	69	81	
ENSXETG00000031015	391	0	0	0	0	0	0	1	3	2	1	1	1	
ENSXETG00000031016	1716	8987	13095	11155	9292	7394	11332	10728	12092	7583	4876	3359	3775	SEPHS2
ENSXETG00000031017	1353	2194	3264	2831	2914	2248	5522	4278	4881	2809	5565	3804	4192	PTK6
ENSXETG00000031018	4593	589	858	699	693	521	1020	1729	2061	1220	511	349	377	pitpna
ENSXETG00000031019	2610	4	13	5	2	2	0	5	12	9	1	1	0	ENSXETG00000031019
ENSXETG00000031020	119	0	0	0	0	0	0	0	0	0	0	0	0	
ENSXETG00000031021	1334	35	51	43	73	45	116	1063	1297	746	174	131	133	ENSXETG00000031021
ENSXETG00000031022	1335	1	0	0	0	0	0	1	0	0	0	1	1	HTR3B
ENSXETG00000031023	135	0	0	0	1	0	1	0	0	1	2	0	1	SNORA66
ENSXETG00000031024	119	0	0	0	0	0	0	0	0	0	0	0	0	
ENSXETG00000031025	1182	0	0	0	0	0	0	0	0	0	0	0	0	
ENSXETG00000031026	1032	36	68	63	897	643	347	262	282	190	2251	1566	1733	ANXA9
ENSXETG00000031027	1140	0	0	0	0	0	0	1	0	0	1	0	1	tmem211
ENSXETG00000031028	966	0	0	0	0	0	0	0	0	0	0	0	0	
ENSXETG00000031029	1005	0	0	0	0	0	0	0	0	0	0	0	0	
ENSXETG00000031030	1389	0	2	0	1	1	0	7	3	2	2	0	2	ENSXETG00000031030
ENSXETG00000031031	1115	30	55	44	44	33	95	134	154	100	70	56	52	ENSXETG00000031031
ENSXETG00000031032	3381	1	0	1	121	87	24	14	17	7	26	19	29	VWA3A
ENSXETG00000031033	474	0	0	0	0	0	0	0	0	0	0	0	0	
ENSXETG00000031034	471	0	0	0	0	0	0	0	0	0	0	0	0	
ENSXETG00000031035	1272	1	6	4	2	4	2	14	15	7	21	11	10	ENSXETG00000031035
ENSXETG00000031036	435	1	2	2	5	6	5	266	315	168	5331	3798	4166	ENSXETG00000031036
ENSXETG00000031037	1587	42	62	43	99	59	149	137	163	86	122	96	102	ENSXETG00000031037
ENSXETG00000031038	1200	0	0	0	1	3	2	0	0	0	3	1	3	ENSXETG00000031038
ENSXETG00000031039	2211	9	11	9	17	18	79	196	248	146	27	17	21	F13A1
ENSXETG00000031040	2091	55	74	50	242	200	351	178	191	109	276	214	211	TMC7
ENSXETG00000031041	1683	20	39	52	120	97	40	97	113	83	81	43	39	ENSXETG00000031041
ENSXETG00000031042	954	8	8	3	16	6	16	13	20	7	30	15	28	FFAR1
ENSXETG00000031043	547	1354	1938	1527	1141	872	1494	1579	1779	1008	1176	844	991	fam32a
ENSXETG00000031044	1518	9	18	13	15	14	10	26	28	23	16	9	12	ENSXETG00000031044
ENSXETG00000031045	615	194771	286859	253369	113417	87338	174684	44070	50731	31113	23284	16506	18062	ENSXETG00000031045
ENSXETG00000031046	792	90	136	120	94	53	29	427	498	281	13	6	7	ENSXETG00000031046
ENSXETG00000031047	741	1	3	3	20	26	11	4	7	2	60	31	41	ENSXETG00000031047
ENSXETG00000031048	414	0	0	0	0	0	0	1	0	1	1	0	0	
ENSXETG00000031049	119	0	0	0	0	0	0	0	0	0	0	0	0	
ENSXETG00000031050	786	93	144	112	254	170	254	516	601	303	314	179	257	ENSXETG00000031050
ENSXETG00000031051	591	0	0	1	0	0	0	0	0	0	0	0	0	
ENSXETG00000031052	528	0	0	0	0	0	0	0	0	0	0	0	0	
ENSXETG00000031053	1818	0	0	0	33	34	2	3	6	7	5	1	0	TTC6
ENSXETG00000031054	2283	923	1370	1184	917	764	675	1745	1970	1099	693	472	551	NOC2L
ENSXETG00000031055	966	412	575	465	358	246	678	495	622	376	354	249	305	pqlc2l
ENSXETG00000031056	119	0	0	0	0	0	0	0	0	0	0	0	0	
ENSXETG00000031057	1620	0	0	0	0	0	0	0	0	0	0	0	0	
ENSXETG00000031058	2475	0	0	0	0	0	0	0	0	0	0	0	0	
ENSXETG00000031059	119	0	0	0	0	0	0	0	0	0	0	0	0	
ENSXETG00000031060	930	0	0	0	0	0	0	0	0	0	0	0	0	
ENSXETG00000031061	951	0	0	0	0	0	0	0	0	0	0	0	0	
ENSXETG00000031062	411	0	0	0	0	0	0	0	1	0	0	0	0	hist2h3c
ENSXETG00000031063	939	0	0	0	0	0	0	0	0	0	0	0	0	
ENSXETG00000031064	951	0	0	0	0	0	0	0	0	0	0	0	0	
ENSXETG00000031065	296	0	0	0	0	0	0	0	0	0	0	0	0	
ENSXETG00000031066	570	84	146	125	218	160	235	448	503	295	239	180	180	TMEM65
ENSXETG00000031067	1179	0	0	0	0	0	0	0	0	0	0	0	0	
ENSXETG00000031068	2277	5	9	0	0	0	0	0	0	1	0	0	0	ENSXETG00000031068
ENSXETG00000031069	537	0	0	0	0	0	0	0	0	0	0	0	0	
ENSXETG00000031070	1053	0	0	0	0	0	0	0	0	0	0	0	0	
ENSXETG00000031071	1143	31	56	43	123	83	218	133	129	76	122	95	117	ENSXETG00000031071
ENSXETG00000031072	296	0	0	0	0	0	0	0	0	0	0	0	0	
ENSXETG00000031073	1464	3	2	0	77	68	8	34	34	23	9	6	10	KATNAL1
ENSXETG00000031074	2781	136	215	191	220	159	278	282	337	206	310	199	262	PIGN
ENSXETG00000031075	1203	0	1	4	5	1	1	42	47	38	2	3	2	CREB3L1
ENSXETG00000031076	70	0	0	0	0	0	0	0	0	0	0	0	0	
ENSXETG00000031077	119	0	0	0	0	0	0	0	0	0	0	0	0	
ENSXETG00000031078	2400	137	211	181	199	148	276	398	479	273	321	222	249	akap17a
ENSXETG00000031079	57	0	0	0	0	0	0	0	0	0	0	0	0	
ENSXETG00000031080	3002	112	165	158	234	154	240	238	325	159	447	320	357	ENSXETG00000031080
ENSXETG00000031081	2373	0	0	0	0	0	0	0	0	0	0	0	0	
ENSXETG00000031082	948	0	0	0	0	0	1	0	0	0	0	0	0	
ENSXETG00000031083	119	0	0	0	0	0	0	0	0	0	0	0	0	
ENSXETG00000031084	585	6	8	4	29	15	33	193	172	123	86	71	56	fzd1
ENSXETG00000031085	1122	0	0	0	0	0	0	0	2	1	0	0	1	ENSXETG00000031085
ENSXETG00000031086	1959	68	91	92	69	62	94	240	292	160	69	59	66	NOL9
ENSXETG00000031087	393	0	1	0	1	0	0	1	0	0	0	0	0	ENSXETG00000031087
ENSXETG00000031088	119	0	0	0	0	0	0	0	0	0	0	0	0	
ENSXETG00000031089	1398	3	3	3	3	5	9	47	49	23	8	10	8	ENSXETG00000031089
ENSXETG00000031090	1572	79	117	127	458	345	498	317	357	205	468	379	367	ENSXETG00000031090
ENSXETG00000031091	545	68	116	84	119	99	114	314	340	185	115	75	68	ENSXETG00000031091
ENSXETG00000031092	576	4	2	0	9	6	11	41	33	26	11	10	6	ENSXETG00000031092
ENSXETG00000031093	534	3	1	3	15	10	15	16	14	12	42	41	32	ENSXETG00000031093
ENSXETG00000031094	4083	0	0	0	0	0	0	0	0	0	0	0	0	
ENSXETG00000031095	510	0	0	0	0	0	0	0	0	0	0	0	0	
ENSXETG00000031096	1092	4	9	3	33	26	24	112	98	48	57	55	39	PRR5L
ENSXETG00000031097	1917	459	607	545	387	318	614	348	386	219	305	174	204	ccdc51
ENSXETG00000031098	108	0	0	0	0	0	0	0	0	0	0	1	0	PK-G12rRNA
ENSXETG00000031099	3459	23	30	40	96	82	101	106	169	93	183	155	183	ENSXETG00000031099
ENSXETG00000031100	70	0	0	0	0	0	0	0	0	0	0	0	0	
ENSXETG00000031101	495	0	0	0	0	0	0	0	0	0	0	0	0	
ENSXETG00000031102	1599	110	151	146	264	181	345	359	429	258	468	322	349	ptar1
ENSXETG00000031103	978	0	0	0	0	0	0	0	0	0	0	0	0	
ENSXETG00000031104	756	0	2	1	4	5	1	2	7	5	2	1	2	ENSXETG00000031104
ENSXETG00000031105	963	36	62	55	46	65	108	548	551	334	168	111	99	PARVG
ENSXETG00000031106	498	0	0	0	2	1	0	0	0	0	1	1	0	
ENSXETG00000031107	720	0	0	0	0	0	0	0	0	0	0	0	0	
ENSXETG00000031108	1389	0	0	0	0	0	0	0	0	1	0	0	0	
ENSXETG00000031109	1230	12	8	9	38	29	48	93	123	84	94	63	78	ENSXETG00000031109
ENSXETG00000031110	1054	0	0	0	0	0	0	0	0	0	0	0	0	
ENSXETG00000031111	987	2	5	2	18	12	17	755	815	501	68	45	59	ENSXETG00000031111
ENSXETG00000031112	2445	0	0	0	0	0	0	0	0	0	0	0	0	
ENSXETG00000031113	975	0	0	0	0	0	0	0	0	0	0	0	0	
ENSXETG00000031114	1032	0	0	0	0	0	0	1	0	0	0	0	0	
ENSXETG00000031115	1434	0	0	0	0	0	0	7	9	4	0	0	0	
ENSXETG00000031116	1155	0	0	0	0	0	0	0	0	0	0	0	0	
ENSXETG00000031117	1122	0	0	0	1	0	1	9	10	6	1	2	1	GPR27
ENSXETG00000031118	528	0	0	0	0	0	0	0	0	1	0	0	0	
ENSXETG00000031119	87	0	0	0	0	0	0	0	0	0	0	0	0	

ENSXETG00000031120	822	41	73	67	208	169	382	588	654	399	333	269	237	CH25H
ENSXETG00000031121	167	0	0	0	0	0	0	0	0	0	0	0	0	
ENSXETG00000031122	1656	597	941	773	1007	711	1838	1426	1549	942	1215	828	959	RNF19B
ENSXETG00000031123	657	269	415	364	527	401	386	645	767	458	1055	770	820	ENSXETG00000031123
ENSXETG00000031124	525	0	0	0	0	0	0	0	0	0	0	0	0	
ENSXETG00000031125	1847	0	0	1	0	0	0	0	0	0	1	0	0	
ENSXETG00000031126	2362	0	0	0	0	0	0	0	0	0	0	0	0	
ENSXETG00000031127	1014	6	6	7	24	6	1	19	13	9	180	103	130	ENSXETG00000031127
ENSXETG00000031128	1011	0	0	0	0	0	0	2	0	0	1	0	0	PTGDR
ENSXETG00000031129	885	0	0	0	1	0	0	0	0	0	0	1	0	ENSXETG00000031129
ENSXETG00000031130	1086	60	82	62	66	65	98	273	292	187	84	75	85	ENSXETG00000031130
ENSXETG00000031131	942	0	0	0	0	0	0	0	0	0	0	0	0	
ENSXETG00000031132	1359	720	1055	877	603	414	688	567	748	438	489	323	330	ENSXETG00000031132
ENSXETG00000031133	2307	0	0	1	5	10	17	15	32	17	25	16	10	SEMA3E
ENSXETG00000031134	870	2	4	1	1	1	8	15	17	5	12	5	3	NFKBID
ENSXETG00000031135	402	0	0	0	0	0	0	0	0	0	0	0	0	
ENSXETG00000031136	2104	2	3	4	11	11	12	340	424	227	36	16	23	tmem35a
ENSXETG00000031137	978	1	0	0	0	0	0	0	1	0	0	1	1	ENSXETG00000031137
ENSXETG00000031138	1036	1340	1963	1673	1461	1083	10996	9763	11156	6694	4008	2921	3043	ENSXETG00000031138
ENSXETG00000031139	1464	0	1	1	89	80	15	1	1	1	40	23	34	ANKUB1
ENSXETG00000031140	786	339	472	361	157	139	118	828	970	571	48	28	39	ENSXETG00000031140
ENSXETG00000031141	1152	0	0	0	0	0	0	0	0	0	0	0	0	
ENSXETG00000031142	3474	0	3	0	7	5	3	12	11	8	9	3	5	ALK
ENSXETG00000031143	1653	9	7	3	11	6	1	0	2	0	7	5	4	ENSXETG00000031143
ENSXETG00000031144	2262	50	86	61	101	86	127	115	156	77	95	78	89	ENSXETG00000031144
ENSXETG00000031145	2703	11	14	17	5	5	4	6	5	2	19	12	14	ENSXETG00000031145
ENSXETG00000031146	576	0	0	0	0	0	0	0	0	0	0	0	0	
ENSXETG00000031147	1197	0	0	0	0	0	0	2	0	2	0	0	0	
ENSXETG00000031148	119	0	0	0	0	0	0	0	0	0	0	0	0	
ENSXETG00000031149	4146	0	1	0	0	0	2	0	0	0	1	0	0	ENSXETG00000031149
ENSXETG00000031150	945	0	0	0	0	0	0	0	0	0	0	0	0	
ENSXETG00000031151	978	0	0	0	0	0	0	0	0	0	0	0	0	
ENSXETG00000031152	921	0	0	0	0	0	0	0	0	0	0	0	0	
ENSXETG00000031153	141	0	0	0	0	0	0	0	0	0	0	0	0	
ENSXETG00000031154	456	0	1	0	4	3	0	0	0	0	8	5	6	ENSXETG00000031154
ENSXETG00000031155	579	2	0	5	76	46	22	1	1	0	320	223	251	ENSXETG00000031155
ENSXETG00000031156	2065	83	145	94	124	89	205	341	349	196	225	145	196	ENSXETG00000031156
ENSXETG00000031157	501	0	0	0	0	0	0	3	0	1	1	3	1	
ENSXETG00000031158	2949	73	111	87	206	171	343	244	313	192	154	135	134	KIAA0753
ENSXETG00000031159	3186	3	3	3	9	3	10	198	268	160	135	96	92	CP
ENSXETG00000031160	981	43	59	58	51	32	32	131	152	76	54	45	36	ENSXETG00000031160
ENSXETG00000031161	1419	0	0	0	0	0	0	0	0	0	0	0	0	
ENSXETG00000031162	528	0	0	0	0	0	0	0	0	0	0	0	0	
ENSXETG00000031163	807	0	0	0	0	0	0	0	0	0	0	0	0	
ENSXETG00000031164	1657	590	791	673	623	526	897	344	361	237	1080	769	786	thap4
ENSXETG00000031165	3393	0	1	0	1	4	13	28	32	16	5	13	14	ENSXETG00000031165
ENSXETG00000031166	1938	0	0	0	0	1	1	1	4	0	2	1	1	
ENSXETG00000031167	720	0	1	0	4	0	6	14	18	8	1	1	3	ENSXETG00000031167
ENSXETG00000031168	1758	37	53	50	112	98	234	412	502	305	214	145	171	ELMO1
ENSXETG00000031169	2373	0	0	0	0	0	1	0	0	0	0	0	0	
ENSXETG00000031170	2346	0	0	0	0	0	0	0	0	0	0	0	0	
ENSXETG00000031171	1167	1	0	0	1	0	0	1	0	0	8	2	0	ENSXETG00000031171
ENSXETG00000031172	969	0	0	0	0	0	0	0	0	0	0	0	0	
ENSXETG00000031173	2695	3	5	10	7	5	9	2	3	1	21	8	11	MORC1
ENSXETG00000031174	942	1	2	2	1	3	0	2	2	1	7	4	3	ENSXETG00000031174
ENSXETG00000031175	888	22	39	28	41	37	89	69	77	34	53	40	52	FAM26E
ENSXETG00000031176	1707	1703	2508	2178	2276	1713	2096	1534	1736	1008	2341	1663	1893	ENSXETG00000031176
ENSXETG00000031177	1317	0	0	0	0	0	0	3	17	19	0	0	0	
ENSXETG00000031178	561	0	1	0	0	1	1	12	11	10	2	9	2	ENSXETG00000031178
ENSXETG00000031179	3727	4	2	0	28	15	10	14	17	11	36	20	25	slc12a5
ENSXETG00000031180	3459	203	309	237	445	358	464	551	649	381	405	279	327	FAM149B1
ENSXETG00000031181	690	10	18	12	24	12	24	30	46	16	20	12	11	ENSXETG00000031181
ENSXETG00000031182	1284	0	0	0	6	2	1	0	1	0	30	28	27	ENSXETG00000031182
ENSXETG00000031183	906	0	0	0	0	0	0	0	0	0	0	0	0	
ENSXETG00000031184	1611	106	165	136	71	62	120	66	85	50	57	27	30	XYLB
ENSXETG00000031185	948	0	0	0	0	0	0	0	0	0	0	0	0	
ENSXETG00000031186	336	0	0	0	0	0	0	1	0	0	0	0	0	
ENSXETG00000031187	158	0	0	0	0	0	0	0	0	0	0	0	0	
ENSXETG00000031188	1308	20	27	25	36	31	103	169	171	91	77	63	84	ENSXETG00000031188
ENSXETG00000031189	4464	367	499	448	595	462	1069	2234	2765	1591	1392	1004	1095	ADGRL2
ENSXETG00000031190	671	316	377	337	228	162	309	345	365	219	219	180	187	cox19
ENSXETG00000031191	1002	0	0	0	0	0	0	0	0	0	0	0	0	
ENSXETG00000031192	1260	48	77	66	65	60	77	111	127	74	107	63	89	ENSXETG00000031192
ENSXETG00000031193	70	0	0	0	0	0	0	0	0	0	0	0	0	
ENSXETG00000031194	5187	228	320	283	656	514	736	1106	1263	712	936	626	706	shroom2
ENSXETG00000031195	423	10	11	15	59	47	17	1281	1384	808	405	312	307	ENSXETG00000031195
ENSXETG00000031196	1280	1	2	0	1	2	2	12	3	5	4	5	2	ENSXETG00000031196
ENSXETG00000031197	1905	1317	2050	1755	2978	2193	3299	2941	3225	2074	2385	1777	1981	TM9SF4
ENSXETG00000031198	645	0	2	1	0	1	0	1	0	0	8	4	4	ENSXETG00000031198
ENSXETG00000031199	528	0	0	0	0	0	0	0	0	0	0	0	0	ENSXETG00000031199
ENSXETG00000031200	411	1	0	0	1	2	1	41	44	23	9	13	11	ENSXETG00000031200
ENSXETG00000031201	810	215	344	282	328	249	174	407	403	253	260	220	213	ENSXETG00000031201
ENSXETG00000031202	3432	4	2	0	2	1	1	2	4	4	8	2	0	ENSXETG00000031202
ENSXETG00000031203	2897	164	242	195	321	270	747	618	793	473	484	398	416	MFSD1
ENSXETG00000031204	115	0	0	0	1	0	0	0	0	0	0	0	0	
ENSXETG00000031205	912	0	0	0	0	0	0	0	0	0	0	0	0	
ENSXETG00000031206	918	0	0	0	0	0	0	0	0	0	0	0	0	
ENSXETG00000031207	2179	52	74	71	232	164	201	712	816	479	168	139	169	cep68
ENSXETG00000031208	888	0	0	0	0	0	0	0	0	0	0	0	0	
ENSXETG00000031209	393	0	0	0	2	0	0	0	0	0	1	0	0	hist1h2aj
ENSXETG00000031210	1020	0	0	0	0	0	0	0	0	0	0	0	0	
ENSXETG00000031211	942	0	0	0	0	0	0	0	0	0	0	0	0	
ENSXETG00000031212	783	26	30	29	67	48	87	153	146	120	96	64	65	lrrc61
ENSXETG00000031213	504	0	0	0	0	0	0	0	2	0	2	1	1	
ENSXETG00000031214	381	0	0	0	0	0	0	0	0	0	0	0	0	
ENSXETG00000031215	119	0	0	0	0	0	0	0	0	0	0	0	0	
ENSXETG00000031216	158	0	0	0	0	0	0	0	0	0	0	0	0	
ENSXETG00000031217	1056	0	0	0	0	0	0	0	0	0	0	0	0	
ENSXETG00000031218	675	73	125	114	192	143	265	553	626	394	246	197	172	ENSXETG00000031218
ENSXETG00000031219	312	0	0	0	0	0	1	0	0	0	0	0	0	
ENSXETG00000031220	504	0	0	0	0	0	0	0	0	0	0	0	0	
ENSXETG00000031221	119	0	0	0	0	0	0	0	0	0	0	0	0	
ENSXETG00000031222	528	0	0	0	0	0	0	0	0	1	0	0	0	
ENSXETG00000031223	1414	48	92	77	33	31	224	314	306	222	2967	2122	2441	mustn1
ENSXETG00000031224	1773	4	6	7	525	432	32	3	2	2	97	73	71	ENSXETG00000031224
ENSXETG00000031225	1641	0	0	0	0	0	0	0	0	0	1	0	1	ENSXETG00000031225
ENSXETG00000031226	70	0	0	0	0	0	0	0	0	0	0	0	0	
ENSXETG00000031227	214	0	0	0	0	0	0	0	0	0	0	0	0	
ENSXETG00000031228	1694	0	2	0	0	1	10	17	23	8	10	6	10	MGC147314
ENSXETG00000031229	8733	233	349	274	1661	1266	2342	17695	20083	12189	3109	2240	2502	COL6A3
ENSXETG00000031230	813	272	374	345	273	213	457	360	428	263	210	129	141	LHPP

ENSXETG00000031231	3604	16	23	21	102	99	67	199	213	152	95	68	68	FBF1
ENSXETG00000031232	3120	1	8	4	12	6	11	114	136	87	25	10	11	ENSXETG00000031232
ENSXETG00000031233	2067	0	0	0	0	0	0	0	0	0	0	0	0	
ENSXETG00000031234	114	39	54	55	109	98	74	313	327	223	75	64	74	ENSXETG00000031234
ENSXETG00000031235	582	0	0	0	0	0	0	0	0	0	0	0	0	ENSXETG00000031235
ENSXETG00000031236	930	0	0	0	0	0	0	0	1	0	0	0	0	
ENSXETG00000031237	1419	0	0	0	0	0	0	0	0	0	0	0	0	
ENSXETG00000031238	1947	0	2	3	70	54	6	16	14	10	3	5	6	LRRC43
ENSXETG00000031239	111	0	0	0	0	0	0	0	0	0	0	0	0	
ENSXETG00000031240	139	0	0	0	0	0	0	0	0	0	0	0	0	U8
ENSXETG00000031241	1050	621	914	776	641	501	1293	2966	3251	2048	1003	701	829	rras
ENSXETG00000031242	1682	601	940	751	989	732	1559	1907	2222	1268	1061	758	838	ubfd1
ENSXETG00000031243	1188	0	0	0	0	0	0	0	0	0	0	0	0	
ENSXETG00000031244	2753	193	285	242	195	165	405	349	430	239	156	85	122	rft1
ENSXETG00000031245	483	0	0	0	0	0	0	0	0	0	0	0	0	
ENSXETG00000031246	1452	331	511	402	433	316	843	708	896	507	592	410	474	ENSXETG00000031246
ENSXETG00000031247	168	0	0	0	1	1	0	0	0	0	1	1	0	
ENSXETG00000031248	504	0	0	0	0	0	0	0	0	0	0	0	0	
ENSXETG00000031249	405	0	0	0	0	0	0	0	0	0	1	1	1	
ENSXETG00000031250	3052	306	475	409	559	454	810	1098	1337	792	786	591	641	cyld
ENSXETG00000031251	1410	0	0	0	0	0	0	0	0	0	1	0	0	
ENSXETG00000031252	1167	0	0	0	0	0	0	0	0	0	0	0	0	
ENSXETG00000031253	855	0	0	0	0	0	0	1	0	0	0	0	0	
ENSXETG00000031254	540	260	351	298	182	135	145	257	291	177	83	57	69	ENSXETG00000031254
ENSXETG00000031255	1254	0	0	0	0	0	0	0	0	0	0	0	1	
ENSXETG00000031256	989	0	0	0	0	0	0	2	1	0	0	0	0	ENSXETG00000031256
ENSXETG00000031257	336	0	1	0	1	0	1	1	0	0	1	0	0	
ENSXETG00000031258	2721	161	229	213	278	228	350	394	450	291	289	190	204	ENSXETG00000031258
ENSXETG00000031259	507	0	0	0	0	0	0	0	0	0	0	0	0	
ENSXETG00000031260	3551	52	104	84	164	112	198	243	286	186	205	127	152	sertad3
ENSXETG00000031261	1188	0	0	0	0	0	0	0	0	0	0	0	0	
ENSXETG00000031262	107	0	0	0	0	0	0	0	0	0	0	0	0	
ENSXETG00000031263	3526	510	740	627	873	746	1436	2400	2917	1673	2085	1532	1602	traf7
ENSXETG00000031264	1491	0	0	0	0	1	5	0	2	0	9	8	4	ENSXETG00000031264
ENSXETG00000031265	3708	255	348	304	723	551	953	1230	1273	773	830	626	726	RAPH1
ENSXETG00000031266	837	1	2	0	2	1	4	19	14	8	16	27	20	CCDC106
ENSXETG00000031267	567	0	0	0	0	0	0	0	0	0	0	0	0	ENSXETG00000031267
ENSXETG00000031268	1485	17	29	34	41	37	98	141	119	88	109	67	77	ZNF513
ENSXETG00000031269	959	0	0	0	0	0	0	0	0	0	0	0	0	
ENSXETG00000031270	969	1	0	0	1	2	1	0	0	0	2	0	2	ENSXETG00000031270
ENSXETG00000031271	1341	1841	2625	2282	2950	2196	3148	2536	2690	1725	1739	1180	1326	ENSXETG00000031271
ENSXETG00000031272	2343	0	0	0	0	0	0	0	0	0	0	0	0	
ENSXETG00000031273	786	0	0	2	0	0	3	10	9	6	0	4	3	ENSXETG00000031273
ENSXETG00000031274	1818	0	0	0	0	0	0	0	0	0	0	0	0	ENSXETG00000031274
ENSXETG00000031275	2868	210	311	280	487	394	622	510	561	345	1592	1151	1293	ENSXETG00000031275
ENSXETG00000031276	1709	18	26	28	85	80	35	85	92	58	102	92	82	MGC147302
ENSXETG00000031277	1967	4	1	3	49	32	97	246	292	193	86	68	79	spry3
ENSXETG00000031278	1165	2	1	0	0	0	0	0	1	0	0	0	0	
ENSXETG00000031279	2787	59	77	70	169	137	193	242	273	175	91	55	59	BBS7
ENSXETG00000031280	969	62	102	80	100	77	129	192	204	134	125	105	110	ENSXETG00000031280
ENSXETG00000031281	1396	6515	9586	8297	12171	9134	30889	197	230	121	17971	12858	14121	aqp8
ENSXETG00000031282	600	1	0	0	0	0	0	0	0	0	1	0	0	ENSXETG00000031282
ENSXETG00000031283	1656	440	656	553	281	179	261	470	630	348	295	219	229	ENSXETG00000031283
ENSXETG00000031284	1269	22	35	27	0	0	0	50	46	33	0	0	0	ENSXETG00000031284
ENSXETG00000031285	657	0	0	0	0	0	0	0	0	0	0	0	0	ENSXETG00000031285
ENSXETG00000031286	4795	137	219	192	226	181	413	1362	1472	896	602	461	459	ENSXETG00000031286
ENSXETG00000031287	420	0	0	0	0	0	0	0	0	0	1	0	0	
ENSXETG00000031288	2529	0	0	0	0	0	1	0	2	0	2	0	0	
ENSXETG00000031289	2247	177	267	247	759	643	204	630	718	435	492	352	390	ENSXETG00000031289
ENSXETG00000031290	666	0	0	0	0	0	0	0	0	0	0	0	0	
ENSXETG00000031291	507	3	1	1	9	4	4	1	0	5	1	1	1	ENSXETG00000031291
ENSXETG00000031292	918	0	0	0	0	0	0	0	0	0	0	0	0	
ENSXETG00000031293	107	0	0	0	0	0	0	0	0	0	0	0	0	
ENSXETG00000031294	660	0	0	0	0	0	0	0	0	0	0	0	0	
ENSXETG00000031295	927	0	0	0	0	0	0	0	0	0	0	0	0	
ENSXETG00000031296	1830	0	1	0	0	0	0	0	0	1	0	0	0	
ENSXETG00000031297	849	0	0	0	0	0	0	2	3	1	0	0	0	
ENSXETG00000031298	687	0	0	0	0	0	0	0	0	0	0	0	0	
ENSXETG00000031299	945	0	0	0	0	0	0	0	0	0	0	0	0	
ENSXETG00000031300	363	79	98	78	179	150	314	197	211	137	328	266	258	ENSXETG00000031300
ENSXETG00000031301	1545	0	3	0	96	65	3	0	0	0	1	2	1	CDC20B
ENSXETG00000031302	1002	24	35	24	79	64	85	94	128	49	96	72	93	ENSXETG00000031302
ENSXETG00000031303	1458	61	88	66	22	16	64	203	162	118	148	90	97	ENSXETG00000031303
ENSXETG00000031304	624	2	4	1	2	0	1	4	4	5	0	4	3	ENSXETG00000031304
ENSXETG00000031305	534	0	0	0	0	0	0	0	0	0	0	0	0	
ENSXETG00000031306	141	0	0	0	0	0	0	0	0	0	0	0	0	
ENSXETG00000031307	593	0	0	0	0	0	0	0	0	0	0	0	1	ifnl3
ENSXETG00000031308	639	0	0	0	0	0	0	0	0	0	1	0	0	
ENSXETG00000031309	670	7	15	7	65	46	192	1463	1810	1070	220	174	176	rab31
ENSXETG00000031310	1734	32	42	36	72	50	160	191	197	113	155	110	124	ENSXETG00000031310
ENSXETG00000031311	3006	0	4	2	176	124	14	11	10	11	33	16	26	ENSXETG00000031311
ENSXETG00000031312	4881	1	6	1	14	1	6	60	74	31	20	16	14	ENSXETG00000031312
ENSXETG00000031313	495	0	0	0	0	0	0	0	0	0	0	0	0	
ENSXETG00000031314	1259	0	0	0	2	1	1	0	0	0	0	0	0	sftpc
ENSXETG00000031315	552	142	195	122	175	131	190	372	422	247	292	209	205	ENSXETG00000031315
ENSXETG00000031316	1947	19	38	23	158	145	69	156	211	109	189	127	155	RGS14
ENSXETG00000031317	1560	0	0	0	0	0	0	0	0	0	0	0	1	
ENSXETG00000031318	1983	0	0	0	0	0	2	0	0	0	0	0	0	
ENSXETG00000031319	951	0	0	0	0	0	0	0	0	0	0	0	0	
ENSXETG00000031320	567	5	12	10	44	38	40	32	38	20	165	102	124	ENSXETG00000031320
ENSXETG00000031321	1866	0	0	0	0	0	0	4	4	2	1	0	1	cacng2
ENSXETG00000031322	780	1	2	1	1	3	0	2	1	0	5	2	2	ENSXETG00000031322
ENSXETG00000031323	1245	0	0	0	0	0	0	0	0	0	0	0	0	ENSXETG00000031323
ENSXETG00000031324	119	0	0	0	0	0	0	0	0	0	0	0	0	
ENSXETG00000031325	337	417	672	563	8813	6617	695	946	1123	622	5664	4020	4347	S100P
ENSXETG00000031326	474	7	6	12	77	54	44	84	97	65	66	64	47	ENSXETG00000031326
ENSXETG00000031327	1437	0	0	0	0	0	0	0	0	0	0	0	0	
ENSXETG00000031328	1203	0	0	0	0	0	0	3	2	2	0	0	0	
ENSXETG00000031329	109	0	0	0	0	0	0	0	0	0	0	0	0	
ENSXETG00000031330	720	0	0	0	0	0	0	0	0	0	0	0	1	
ENSXETG00000031331	1499	103	144	147	260	170	257	471	558	353	435	305	353	ENSXETG00000031331
ENSXETG00000031332	1820	39	41	40	39	41	91	198	218	122	135	126	123	CPNE8
ENSXETG00000031333	705	0	0	0	0	0	0	0	0	0	0	0	0	
ENSXETG00000031334	591	0	0	0	0	0	0	0	0	0	0	0	0	
ENSXETG00000031335	1323	0	0	0	3	1	0	0	0	0	0	0	0	ENSXETG00000031335
ENSXETG00000031336	3693	2	2	1	10	5	3	10	7	5	49	23	34	MYPN
ENSXETG00000031337	3463	0	0	0	0	0	0	0	0	0	0	0	0	
ENSXETG00000031338	3009	1	1	1	14	1	16	42	59	31	19	10	8	cntn4
ENSXETG00000031339	1416	72	119	83	149	138	273	191	264	133	234	204	182	terf2
ENSXETG00000031340	1164	8	4	5	2	2	12	25	26	23	20	17	16	ENSXETG00000031340
ENSXETG00000031341	990	0	0	0	0	0	0	0	0	0	0	0	0	

ENSXETG00000031342	2947	161	273	221	369	271	769	552	740	425	339	277	292	ENSXETG00000031342
ENSXETG00000031343	2451	0	0	0	0	0	0	0	0	0	0	0	0	
ENSXETG00000031344	119	0	0	0	0	0	0	0	0	0	0	0	0	
ENSXETG00000031345	501	0	0	0	0	0	0	0	0	0	0	0	0	
ENSXETG00000031346	1644	110	152	147	288	203	308	208	236	159	263	163	189	ENSXETG00000031346
ENSXETG00000031347	119	0	0	0	0	0	0	0	0	0	0	0	0	
ENSXETG00000031348	1120	0	0	0	0	4	0	1	1	0	1	0	0	hes6.2
ENSXETG00000031349	807	0	0	0	0	0	0	0	0	0	0	0	0	
ENSXETG00000031350	636	3	4	2	2	3	4	2	2	0	4	0	4	ENSXETG00000031350
ENSXETG00000031351	807	0	0	0	0	0	0	0	1	0	0	0	0	ENSXETG00000031351
ENSXETG00000031352	70	0	0	0	0	0	0	0	0	0	0	0	0	
ENSXETG00000031353	312	0	0	0	0	0	0	0	0	0	0	1	0	
ENSXETG00000031354	1104	0	0	0	0	0	0	0	0	0	0	0	0	
ENSXETG00000031355	802	0	0	1	1	0	1	2	0	1	0	0	1	ENSXETG00000031355
ENSXETG00000031356	1328	104	217	172	222	193	485	591	599	376	377	226	261	naa60
ENSXETG00000031357	1617	63	81	93	125	104	106	288	288	195	113	92	75	ADCK2
ENSXETG00000031358	2010	0	0	0	0	0	0	0	1	0	0	0	0	ENSXETG00000031358
ENSXETG00000031359	255	4	3	6	52	38	177	239	258	153	88	51	76	ENSXETG00000031359
ENSXETG00000031360	513	0	0	0	0	0	0	0	0	1	0	0	0	
ENSXETG00000031361	1275	0	0	0	0	0	0	0	0	0	0	0	0	
ENSXETG00000031362	2604	51	66	50	151	94	85	213	286	168	144	114	111	RBBP8
ENSXETG00000031363	711	76	114	85	100	93	249	56	93	42	89	59	52	ENSXETG00000031363
ENSXETG00000031364	2624	1574	2450	2093	3212	2447	4133	2536	2855	1702	2467	1772	1901	nsf
ENSXETG00000031365	444	0	0	0	2	2	0	15	19	10	3	4	4	ENSXETG00000031365
ENSXETG00000031366	918	0	0	0	0	0	0	0	0	0	0	0	0	
ENSXETG00000031367	107	0	0	0	0	0	0	0	0	0	0	0	0	
ENSXETG00000031368	2031	337	514	452	484	361	920	745	909	540	818	524	616	DAGLB
ENSXETG00000031369	741	0	0	0	0	0	0	0	0	0	0	0	0	
ENSXETG00000031370	1596	16	22	23	15	13	76	186	220	117	107	80	88	ENSXETG00000031370
ENSXETG00000031371	1188	0	1	0	1	0	1	0	1	0	0	1	0	ENSXETG00000031371
ENSXETG00000031372	983	0	0	0	0	0	0	1	0	1	0	0	0	
ENSXETG00000031373	423	58	95	78	207	146	119	136	178	97	162	126	134	ENSXETG00000031373
ENSXETG00000031374	2310	0	0	0	0	0	0	0	0	0	0	0	0	
ENSXETG00000031375	1662	0	0	0	0	0	0	0	0	0	0	0	0	
ENSXETG00000031376	1302	77	116	100	160	104	336	67	59	38	146	90	106	ENSXETG00000031376
ENSXETG00000031377	2277	17	18	22	27	21	64	57	74	52	65	47	51	KCNQ5
ENSXETG00000031378	2550	0	0	0	0	0	0	0	0	0	0	0	0	
ENSXETG00000031379	1853	38	40	33	93	56	121	746	878	496	216	184	183	ENSXETG00000031379
ENSXETG00000031380	583	30	25	27	114	78	100	547	646	407	314	216	316	gng3
ENSXETG00000031381	468	0	0	0	0	0	0	0	0	0	0	0	0	
ENSXETG00000031382	507	0	0	0	0	0	0	0	0	0	0	0	0	
ENSXETG00000031383	5553	471	746	612	1512	1195	1693	1314	1481	900	2479	1784	2012	DOCK5
ENSXETG00000031384	1158	0	0	0	0	0	0	0	0	0	0	0	0	
ENSXETG00000031385	1050	6	3	11	39	32	52	72	68	52	35	34	28	ENSXETG00000031385
ENSXETG00000031386	1272	2	0	0	29	16	4	2	1	1	4	2	1	gpr83
ENSXETG00000031387	1326	0	0	0	0	0	0	0	0	0	0	0	0	ENSXETG00000031387
ENSXETG00000031388	119	0	0	0	0	0	0	0	0	0	0	0	0	
ENSXETG00000031389	1905	75	58	60	63	60	152	152	169	100	176	128	125	BBS10
ENSXETG00000031390	2136	0	0	0	0	0	0	0	0	0	0	0	0	
ENSXETG00000031391	2166	3	0	2	5	9	5	15	19	15	17	16	21	ENSXETG00000031391
ENSXETG00000031392	1188	0	0	0	0	0	0	0	0	0	0	0	0	
ENSXETG00000031393	918	29	42	29	32	33	78	53	69	33	11	13	13	ENSXETG00000031393
ENSXETG00000031394	1314	44	55	57	35	26	11	180	207	116	16	11	10	ENSXETG00000031394
ENSXETG00000031395	393	0	0	0	0	0	0	0	0	0	0	0	0	hist1h2ah
ENSXETG00000031396	1154	154	239	207	265	176	408	379	404	292	534	412	467	DUSP7
ENSXETG00000031397	491	816	1194	970	800	617	760	994	1183	673	656	454	467	ndufa2
ENSXETG00000031398	3220	8	20	19	115	97	89	328	368	217	81	64	76	CCP110
ENSXETG00000031399	522	0	0	0	0	0	0	0	0	0	0	0	0	
ENSXETG00000031400	119	0	0	0	0	0	0	0	0	0	2	0	2	5S_rRNA
ENSXETG00000031401	1632	288	354	349	387	308	678	583	652	397	417	317	350	mtif3
ENSXETG00000031402	258	0	0	0	4	1	0	0	2	0	0	0	0	CTXN1
ENSXETG00000031403	711	0	0	0	0	0	0	0	0	0	0	0	0	
ENSXETG00000031404	1041	4	4	5	10	11	29	20	31	11	32	28	23	DRAXIN
ENSXETG00000031405	1419	0	0	0	0	0	0	0	0	0	0	0	0	
ENSXETG00000031406	1470	57	97	80	63	48	84	236	250	179	228	154	158	LNX2
ENSXETG00000031407	1233	326	462	366	865	665	1169	622	752	431	967	735	829	ENSXETG00000031407
ENSXETG00000031408	1083	2	1	5	1	0	1	2	2	0	0	1	1	ENSXETG00000031408
ENSXETG00000031409	119	0	0	0	0	0	0	0	0	0	0	0	0	
ENSXETG00000031410	8652	7	11	7	6	9	25	78	88	56	31	22	20	ENSXETG00000031410
ENSXETG00000031411	486	150	190	165	150	111	363	206	241	135	576	428	502	il15
ENSXETG00000031412	651	0	0	0	0	0	0	2	0	0	0	0	0	
ENSXETG00000031413	139	1	0	0	0	0	0	2	5	2	0	1	3	U8
ENSXETG00000031414	1452	1	0	0	1	0	0	26	23	15	0	0	0	
ENSXETG00000031415	3246	146	207	157	891	699	1296	2019	2514	1390	949	683	780	ENSXETG00000031415
ENSXETG00000031416	333	425	646	540	785	640	1290	330	353	244	1334	983	968	ENSXETG00000031416
ENSXETG00000031417	612	2	3	3	2	0	0	9	7	9	1	1	0	ENSXETG00000031417
ENSXETG00000031418	751	1	2	1	0	0	0	0	2	3	1	0	0	CARTPT
ENSXETG00000031419	792	21	35	26	21	24	3	3	2	2	6	1	4	ENSXETG00000031419
ENSXETG00000031420	486	1	5	3	2	0	1	59	58	42	1	2	3	ENSXETG00000031420
ENSXETG00000031421	2058	2	3	1	11	4	15	23	21	17	21	14	15	EPB42
ENSXETG00000031422	1425	2	5	4	29	26	8	45	55	45	24	12	16	ENSXETG00000031422
ENSXETG00000031423	519	4	4	3	14	11	135	221	248	150	59	48	49	ENSXETG00000031423
ENSXETG00000031424	1467	10082	14050	12108	10819	8271	59112	1957	2342	1321	5547	3921	4389	ENSXETG00000031424
ENSXETG00000031425	2061	252	387	312	411	300	792	834	1010	560	739	505	541	ENSXETG00000031425
ENSXETG00000031426	378	152	180	148	136	107	115	249	348	192	104	89	91	ENSXETG00000031426
ENSXETG00000031427	4219	163	245	202	203	156	302	273	304	176	226	138	151	FAM169A
ENSXETG00000031428	948	157	181	173	125	78	155	243	342	182	131	107	99	TYW5
ENSXETG00000031429	1195	11	19	10	71	59	71	22	36	17	256	165	188	ENSXETG00000031429
ENSXETG00000031430	1272	147	215	200	232	165	345	458	589	320	293	185	254	C16orf70
ENSXETG00000031431	463	1617	2457	2017	2465	1879	1320	1357	1543	868	1242	910	998	ENSXETG00000031431
ENSXETG00000031432	936	0	0	0	0	0	0	0	0	0	0	0	0	
ENSXETG00000031433	2082	0	3	3	0	6	15	10	2	3	13	7	8	ENSXETG00000031433
ENSXETG00000031434	516	0	0	0	0	0	0	0	0	0	0	0	0	
ENSXETG00000031435	1080	8	7	6	6	6	4	12	15	10	7	8	9	ENSXETG00000031435
ENSXETG00000031436	885	0	0	0	0	0	0	0	0	0	0	0	0	
ENSXETG00000031437	2547	6	3	4	1	3	2	9	9	4	6	1	5	ENSXETG00000031437
ENSXETG00000031438	1086	0	0	0	0	0	0	0	1	0	0	0	0	
ENSXETG00000031439	510	0	0	0	0	0	0	0	0	0	0	0	0	
ENSXETG00000031440	816	0	0	0	4	3	2	81	86	56	22	12	18	ENSXETG00000031440
ENSXETG00000031441	141	0	0	0	0	0	0	0	0	0	0	0	0	
ENSXETG00000031442	312	0	0	0	0	0	0	0	0	0	0	0	0	
ENSXETG00000031443	678	0	0	0	0	0	0	0	0	0	1	0	0	ENSXETG00000031443
ENSXETG00000031444	2664	3	5	1	34	19	59	134	177	112	52	38	51	ANO6
ENSXETG00000031445	312	0	0	0	0	0	0	0	1	1	0	0	0	hist1h4c
ENSXETG00000031446	119	0	0	0	0	0	0	0	0	0	0	0	0	
ENSXETG00000031447	2169	535	817	690	645	493	785	417	495	317	640	488	519	ENSXETG00000031447
ENSXETG00000031448	864	1	2	0	0	0	2	1	0	0	2	2	1	ENSXETG00000031448
ENSXETG00000031449	582	207	337	270	289	207	291	400	429	279	584	470	449	ENSXETG00000031449
ENSXETG00000031450	1170	0	0	0	0	0	0	0	0	0	0	0	0	ENSXETG00000031450
ENSXETG00000031451	85	0	0	0	0	0	0	0	0	0	0	0	0	
ENSXETG00000031452	119	0	0	0	0	0	0	0	0	0	0	0	0	

ENSXETG00000031453	510	0	0	0	0	0	0	0	0	0	0	0	0	
ENSXETG00000031454	1062	75	116	95	91	58	168	33	48	24	203	141	147	ENSXETG00000031454
ENSXETG00000031455	2508	32	58	33	343	300	227	497	576	302	173	118	181	IFT88
ENSXETG00000031456	957	0	0	0	0	0	0	0	0	0	0	0	0	ENSXETG00000031456
ENSXETG00000031457	561	413	699	654	740	530	878	578	649	407	1277	997	1031	ENSXETG00000031457
ENSXETG00000031458	44	0	0	0	0	0	0	0	0	0	0	0	0	
ENSXETG00000031459	924	0	0	0	0	0	0	0	0	0	0	0	0	
ENSXETG00000031460	1551	207	312	248	187	142	340	672	803	427	777	567	633	ENSXETG00000031460
ENSXETG00000031461	981	0	0	0	0	0	0	1	0	1	0	0	0	
ENSXETG00000031462	1014	2	1	0	0	0	0	0	0	0	1	0	1	NECAB3
ENSXETG00000031463	717	0	0	0	0	0	0	0	0	0	0	0	0	ENSXETG00000031463
ENSXETG00000031464	1089	0	0	0	0	0	0	0	0	0	0	0	0	ENSXETG00000031464
ENSXETG00000031465	1580	216	332	271	488	367	675	1081	1222	747	401	282	303	wbp1l
ENSXETG00000031466	1989	4760	7195	5912	7943	6110	4458	84	86	72	427	316	341	ENSXETG00000031466
ENSXETG00000031467	1467	16	17	18	38	29	89	81	137	81	39	29	43	pianp
ENSXETG00000031468	585	0	0	0	0	0	0	0	0	0	0	0	0	
ENSXETG00000031469	681	12	27	17	10	13	11	0	0	0	0	0	0	ENSXETG00000031469
ENSXETG00000031470	501	0	0	0	0	0	0	0	0	0	0	0	0	ENSXETG00000031470
ENSXETG00000031471	969	0	0	0	0	0	0	0	0	0	1	0	0	
ENSXETG00000031472	6114	25	33	24	64	46	91	696	849	502	107	94	77	ENSXETG00000031472
ENSXETG00000031473	1599	0	0	0	0	0	0	1	1	0	0	0	0	ENSXETG00000031473
ENSXETG00000031474	2034	167	239	190	212	147	208	286	340	207	313	203	251	HDAC10
ENSXETG00000031475	3519	26	39	36	20	14	50	67	93	44	66	52	38	CARMIL2
ENSXETG00000031476	1065	40	99	81	80	63	184	1033	1114	592	154	123	133	ENSXETG00000031476
ENSXETG00000031477	1554	24	49	39	22	22	43	477	528	352	82	44	64	ENSXETG00000031477
ENSXETG00000031478	1830	25	29	29	67	33	163	501	598	349	258	172	211	ENSXETG00000031478
ENSXETG00000031479	1525	24	25	19	197	164	153	458	505	300	4067	2843	3225	ENSXETG00000031479
ENSXETG00000031480	453	159	245	179	202	143	190	257	289	197	203	140	137	ANKRD39
ENSXETG00000031481	933	0	1	0	0	3	2	2	2	4	2	2	2	GPR142
ENSXETG00000031482	996	0	0	0	0	0	0	0	0	0	0	0	0	
ENSXETG00000031483	6804	0	3	0	89	80	5	12	13	8	7	4	11	ENSXETG00000031483
ENSXETG00000031484	3495	367	544	477	851	676	1494	1192	1375	844	1028	705	737	pdzd8
ENSXETG00000031485	860	11	7	15	9	3	8	15	27	8	22	19	23	ENSXETG00000031485
ENSXETG00000031486	742	0	1	0	0	0	0	0	0	0	2	0	1	ENSXETG00000031486
ENSXETG00000031487	624	161	233	245	597	454	707	962	1059	695	1068	738	874	ENSXETG00000031487
ENSXETG00000031488	3282	73	120	107	805	608	765	1166	1288	782	404	311	309	PCDH19
ENSXETG00000031489	993	0	0	0	0	0	0	0	0	0	0	0	0	
ENSXETG00000031490	214	0	0	0	0	0	0	0	0	0	0	0	0	
ENSXETG00000031491	1047	0	0	0	0	0	0	0	0	0	0	0	0	ENSXETG00000031491
ENSXETG00000031492	113	0	0	0	0	0	0	0	0	0	0	0	0	
ENSXETG00000031493	1650	35	73	43	119	103	138	208	246	153	86	53	62	ENSXETG00000031493
ENSXETG00000031494	2403	203	305	248	185	154	321	489	500	319	436	260	315	PRMT2
ENSXETG00000031495	2268	1656	2233	1937	2009	1514	3252	367	417	218	1061	773	884	ACOX3
ENSXETG00000031496	663	0	0	0	0	0	1	1	4	4	0	0	1	ENSXETG00000031496
ENSXETG00000031497	888	0	0	0	0	0	0	0	0	0	0	0	0	ENSXETG00000031497
ENSXETG00000031498	1353	15	34	25	7	5	12	147	188	107	54	34	38	FOXP3
ENSXETG00000031499	507	0	0	0	0	0	0	0	0	0	0	0	2	
ENSXETG00000031500	1191	1	6	2	8	4	2	26	15	11	21	12	16	ENSXETG00000031500
ENSXETG00000031501	513	0	0	0	0	0	0	0	0	0	0	0	0	
ENSXETG00000031502	1293	0	0	0	0	0	0	0	0	0	0	0	0	ENSXETG00000031502
ENSXETG00000031503	2853	0	1	1	6	1	7	20	17	13	39	26	26	GRIK5
ENSXETG00000031504	1582	6	6	4	32	34	10	2	5	3	51	39	39	neil2
ENSXETG00000031505	510	262	308	279	144	110	376	82	107	43	176	113	131	NMRK1
ENSXETG00000031506	2009	1213	1814	1599	2426	1819	2897	952	1139	612	2565	1765	1925	ENSXETG00000031506
ENSXETG00000031507	1644	0	0	0	0	0	0	0	0	0	0	0	0	
ENSXETG00000031508	786	0	0	0	0	0	0	0	4	1	0	0	0	
ENSXETG00000031509	1722	91	116	87	103	79	288	219	253	120	136	108	124	LCORL
ENSXETG00000031510	1160	3750	5408	4850	3671	2817	4301	6271	7332	4382	6228	4334	4834	ELF3
ENSXETG00000031511	70	0	0	0	0	0	0	0	0	0	0	0	0	
ENSXETG00000031512	119	0	0	0	0	0	0	0	0	0	0	0	0	
ENSXETG00000031513	414	0	0	0	0	0	1	6	6	3	0	2	1	
ENSXETG00000031514	313	969	1469	1203	799	624	656	624	786	473	316	250	211	NDUFC1
ENSXETG00000031515	2073	342	488	364	353	282	356	436	557	305	407	266	313	POMT2
ENSXETG00000031516	1296	0	0	0	0	0	0	0	0	0	0	0	0	ENSXETG00000031516
ENSXETG00000031517	597	0	0	0	0	0	0	0	0	0	0	0	0	
ENSXETG00000031518	420	0	0	0	0	0	0	6	6	3	1	1	0	ENSXETG00000031518
ENSXETG00000031519	513	0	0	0	0	0	0	0	0	0	1	0	0	
ENSXETG00000031520	435	70	121	98	154	137	175	1256	1479	799	1180	859	927	ENSXETG00000031520
ENSXETG00000031521	1506	127	174	148	226	175	303	474	593	357	365	251	304	RELB
ENSXETG00000031522	918	0	0	0	0	0	0	0	0	0	0	0	0	
ENSXETG00000031523	972	0	0	0	1	2	2	1	1	0	0	4	2	ENSXETG00000031523
ENSXETG00000031524	1002	0	0	0	0	0	0	0	0	0	0	0	0	
ENSXETG00000031525	2397	0	0	0	0	0	0	0	0	0	1	0	0	
ENSXETG00000031526	825	23	33	21	34	29	86	58	61	32	120	93	117	ENSXETG00000031526
ENSXETG00000031527	2103	1	1	4	48	29	3	6	2	0	9	4	6	ENSXETG00000031527
ENSXETG00000031528	969	0	0	0	0	0	0	0	0	0	0	0	0	
ENSXETG00000031529	1254	5	4	6	7	7	14	24	37	18	15	7	11	CALCR
ENSXETG00000031530	3308	64	90	52	218	202	429	1781	2163	1305	538	392	427	ENSXETG00000031530
ENSXETG00000031531	2241	0	0	0	0	0	0	0	0	0	0	0	0	
ENSXETG00000031532	2103	4	5	4	7	5	3	0	0	0	0	0	0	ENSXETG00000031532
ENSXETG00000031533	510	0	0	0	0	0	0	0	0	0	0	0	0	
ENSXETG00000031534	2390	0	1	4	17	3	16	58	81	50	38	20	29	hectd2
ENSXETG00000031535	1323	0	0	4	0	0	1	0	0	0	0	0	0	ENSXETG00000031535
ENSXETG00000031536	113	0	0	0	0	0	0	0	0	0	0	0	0	5S_rRNA
ENSXETG00000031537	3507	1	7	7	55	37	18	38	23	23	18	17	5	ENSXETG00000031537
ENSXETG00000031538	3735	123	166	150	654	478	684	1155	1379	827	1096	760	858	atp2a3
ENSXETG00000031539	564	0	0	0	0	0	0	0	0	0	0	0	0	
ENSXETG00000031540	2559	0	0	0	0	0	0	0	0	0	0	0	1	
ENSXETG00000031541	1541	6	8	8	48	52	211	230	264	176	160	90	126	ENSXETG00000031541
ENSXETG00000031542	2046	119	183	140	301	254	372	432	531	326	579	526	511	ENSXETG00000031542
ENSXETG00000031543	666	0	0	0	0	0	0	0	0	0	0	0	0	
ENSXETG00000031544	375	0	0	0	0	0	0	0	0	0	0	0	0	
ENSXETG00000031545	846	0	0	0	0	0	0	0	0	0	0	0	0	
ENSXETG00000031546	5016	0	3	2	11	11	19	51	62	37	22	18	18	TRPM3
ENSXETG00000031547	1167	0	0	0	0	0	0	2	2	0	2	0	1	ENSXETG00000031547
ENSXETG00000031548	519	50	85	64	83	51	118	191	207	116	119	69	83	ENSXETG00000031548
ENSXETG00000031549	924	5	12	8	18	14	18	63	49	42	16	11	16	TOMM40L
ENSXETG00000031550	119	0	0	0	0	0	0	0	0	0	0	0	0	5S_rRNA
ENSXETG00000031551	366	5	27	13	22	19	63	57	81	42	102	69	75	ENSXETG00000031551
ENSXETG00000031552	957	0	0	0	0	0	0	0	0	0	0	0	0	
ENSXETG00000031553	1016	0	0	0	0	0	0	0	0	0	0	0	0	
ENSXETG00000031554	567	218	355	247	35	32	297	36	25	14	83	49	45	ENSXETG00000031554
ENSXETG00000031555	939	0	0	0	0	0	0	0	0	0	0	0	0	
ENSXETG00000031556	1393	2	6	6	15	10	27	52	51	25	23	20	35	erfe
ENSXETG00000031557	963	41	39	32	107	53	83	33	44	20	49	41	38	ENSXETG00000031557
ENSXETG00000031558	1386	6	18	9	16	8	39	64	79	47	22	21	25	ENSXETG00000031558
ENSXETG00000031559	3072	77	112	108	248	172	128	342	420	249	240	184	185	cenpu
ENSXETG00000031560	3498	531	767	648	1359	1088	1365	866	960	598	1625	1132	1227	PHLPP1
ENSXETG00000031561	513	0	0	0	0	0	0	0	0	0	0	0	1	
ENSXETG00000031562	1008	0	0	0	0	0	0	0	0	0	0	0	0	
ENSXETG00000031563	600	0	2	1	8	1	3	48	56	38	6	9	11	ENSXETG00000031563

ENSXETG00000031564	101	0	0	0	0	0	0	0	0	0	0	0	0	
ENSXETG00000031565	1128	23	24	41	61	48	157	130	175	81	102	68	78	ENSXETG00000031565
ENSXETG00000031566	2391	340	514	471	429	363	267	312	348	230	558	419	445	HCN2
ENSXETG00000031567	1425	29	25	25	7	6	30	97	129	78	31	31	32	IL18RAP
ENSXETG00000031568	969	0	0	0	5	3	11	162	202	116	8	6	5	ASB11
ENSXETG00000031569	2319	718	1032	850	938	771	1054	1322	1526	821	996	620	810	naa35
ENSXETG00000031570	3816	494	735	566	1490	1180	1946	1001	1243	667	1856	1347	1480	ENSXETG00000031570
ENSXETG00000031571	351	0	0	0	0	0	0	0	0	1	0	0	0	
ENSXETG00000031572	707	7	8	4	1	0	1	12	25	6	3	6	3	kiss1
ENSXETG00000031573	1326	0	0	0	0	0	0	6	6	3	0	0	0	
ENSXETG00000031574	4515	760	1132	966	2234	1664	2869	2491	2893	1771	2957	2080	2350	ARFGEF1
ENSXETG00000031575	1044	0	0	0	0	0	0	0	0	0	0	0	0	
ENSXETG00000031576	1428	98	116	96	225	180	273	449	508	312	272	189	206	KLHL42
ENSXETG00000031577	990	57	77	70	116	74	179	632	695	402	292	215	246	ENSXETG00000031577
ENSXETG00000031578	2169	0	2	1	0	0	0	0	0	0	0	0	0	ENSXETG00000031578
ENSXETG00000031579	1425	0	0	0	0	0	0	0	0	0	0	0	0	
ENSXETG00000031580	606	2	5	6	22	12	44	74	70	51	75	42	37	ENSXETG00000031580
ENSXETG00000031581	113	0	0	0	0	0	0	0	0	0	0	0	0	
ENSXETG00000031582	528	0	0	0	0	0	0	0	0	0	0	0	0	
ENSXETG00000031583	119	0	0	0	0	0	0	0	0	0	0	0	0	
ENSXETG00000031584	414	903	1391	1154	689	492	726	667	760	482	704	480	507	MRPL49
ENSXETG00000031585	513	0	0	1	1	0	0	0	0	0	0	0	0	ENSXETG00000031585
ENSXETG00000031586	411	0	0	0	0	0	0	0	0	0	0	0	0	hist2h3c
ENSXETG00000031587	214	0	0	0	0	0	0	0	0	0	0	0	0	
ENSXETG00000031588	139	0	0	0	0	0	0	0	0	0	0	0	0	U8
ENSXETG00000031589	1185	0	0	0	0	0	0	0	0	0	0	0	0	
ENSXETG00000031590	876	0	0	0	0	0	0	0	0	0	0	0	0	
ENSXETG00000031591	945	0	0	0	0	0	0	0	0	0	0	0	0	
ENSXETG00000031592	1332	34	46	39	64	40	133	134	186	110	160	106	105	ENSXETG00000031592
ENSXETG00000031593	6537	550	723	648	2015	1483	2494	2361	2636	1672	1921	1317	1422	MED12
ENSXETG00000031594	70	0	0	0	0	0	0	0	0	0	0	0	0	
ENSXETG00000031595	119	0	0	0	0	0	0	0	0	0	0	0	0	
ENSXETG00000031596	611	3753	5547	4662	3046	2208	3542	3624	4250	2497	2175	1489	1691	fkbp2
ENSXETG00000031597	5156	27	41	24	142	93	533	96	107	60	261	157	210	ENSXETG00000031597
ENSXETG00000031598	252	19	19	15	23	23	41	63	49	45	28	27	24	ENSXETG00000031598
ENSXETG00000031599	3252	7	12	12	59	41	29	90	108	53	132	94	119	rhpn1
ENSXETG00000031600	381	0	0	0	0	0	0	0	0	0	0	0	0	ENSXETG00000031600
ENSXETG00000031601	1017	0	0	0	0	0	0	0	1	0	0	0	0	CALHM1
ENSXETG00000031602	930	0	0	0	0	0	0	0	0	0	0	0	0	
ENSXETG00000031603	107	0	0	0	0	0	0	0	0	0	0	0	0	
ENSXETG00000031604	1929	85	139	76	182	135	305	175	238	113	288	206	233	CARF
ENSXETG00000031605	2814	27	32	37	188	140	211	431	452	296	317	198	235	ENSXETG00000031605
ENSXETG00000031606	786	0	0	0	0	0	0	0	1	0	0	1	0	
ENSXETG00000031607	992	201	294	285	303	254	408	435	536	325	363	235	275	C6orf106
ENSXETG00000031608	70	0	0	0	0	0	0	0	0	0	0	0	0	
ENSXETG00000031609	1170	51	73	67	127	126	810	1176	1318	778	1193	889	898	ENSXETG00000031609
ENSXETG00000031610	375	1	4	1	47	37	45	276	310	195	107	81	55	ENSXETG00000031610
ENSXETG00000031611	912	0	0	0	0	0	0	0	0	0	0	0	0	
ENSXETG00000031612	1638	31	27	26	237	153	96	0	0	0	177	134	132	ENSXETG00000031612
ENSXETG00000031613	531	0	0	0	0	0	0	0	0	0	0	0	0	
ENSXETG00000031614	296	0	0	0	0	0	0	0	0	0	0	0	0	
ENSXETG00000031615	2493	129	149	131	292	233	245	300	352	208	304	208	210	ENSXETG00000031615
ENSXETG00000031616	1611	7	15	6	4	3	22	40	22	19	12	8	9	ENSXETG00000031616
ENSXETG00000031617	2568	533	765	679	714	551	1138	989	1132	665	906	681	728	RHBDF1
ENSXETG00000031618	141	0	0	0	0	0	0	0	0	0	0	0	0	
ENSXETG00000031619	1029	0	1	1	2	0	6	15	12	2	11	6	6	ENSXETG00000031619
ENSXETG00000031620	70	0	0	0	0	0	0	0	0	0	0	0	0	
ENSXETG00000031621	745	4	8	8	212	181	36	53	52	44	30	28	33	c2orf70
ENSXETG00000031622	2057	13	13	18	35	36	85	259	288	191	104	67	67	ENSXETG00000031622
ENSXETG00000031623	1125	2	1	1	0	1	23	0	0	0	0	0	0	WNT10A
ENSXETG00000031624	4653	0	0	1	1	0	2	10	12	4	5	3	4	ENSXETG00000031624
ENSXETG00000031625	132	7	4	2	0	0	0	2	1	1	0	0	0	SNORA3
ENSXETG00000031626	1053	107	112	118	122	96	126	450	483	301	139	89	95	ENSXETG00000031626
ENSXETG00000031627	1188	0	0	0	0	0	0	0	0	0	0	0	0	
ENSXETG00000031628	1485	0	0	0	0	0	1	0	1	0	6	4	5	ENSXETG00000031628
ENSXETG00000031629	948	7	3	0	8	3	16	68	72	53	36	12	28	ENSXETG00000031629
ENSXETG00000031630	92	0	0	0	0	0	0	0	1	0	3	3	1	ENSXETG00000031630
ENSXETG00000031631	927	0	0	0	0	0	0	0	0	0	0	0	0	
ENSXETG00000031632	2738	1227	1874	1631	867	663	630	348	452	253	1103	744	916	ENSXETG00000031632
ENSXETG00000031633	960	0	0	0	0	0	0	0	0	0	0	0	0	ENSXETG00000031633
ENSXETG00000031634	807	0	0	0	0	0	0	3	4	0	1	1	1	ENSXETG00000031634
ENSXETG00000031635	978	3	4	4	4	3	10	65	69	40	6	3	10	ENSXETG00000031635
ENSXETG00000031636	1207	1	4	0	11	8	50	48	55	41	17	13	21	MGC147231
ENSXETG00000031637	1047	188	310	235	431	307	759	1422	1542	895	424	278	360	ENSXETG00000031637
ENSXETG00000031638	1299	0	0	0	0	0	0	0	0	0	0	0	0	ENSXETG00000031638
ENSXETG00000031639	1464	0	0	0	0	0	0	0	0	0	0	0	0	
ENSXETG00000031640	489	16	33	29	16	17	36	40	48	21	53	29	46	ENSXETG00000031640
ENSXETG00000031641	852	0	0	0	0	1	1	1	0	0	1	0	1	
ENSXETG00000031642	822	0	0	1	0	1	1	22	18	19	16	6	5	ENSXETG00000031642
ENSXETG00000031643	924	0	0	0	0	0	0	0	0	0	0	0	0	
ENSXETG00000031644	1161	182	233	194	268	217	503	805	854	558	524	360	416	MGC147402
ENSXETG00000031645	615	1	1	0	5	3	7	46	61	28	24	16	18	ENSXETG00000031645
ENSXETG00000031646	567	0	0	0	0	0	0	0	0	1	0	0	1	
ENSXETG00000031647	1353	9	12	5	1	0	2	0	2	1	2	1	1	MGC147172
ENSXETG00000031648	3102	64	102	79	107	56	135	142	155	101	312	176	233	BICRAL
ENSXETG00000031649	510	0	0	0	0	0	0	0	0	0	0	0	0	
ENSXETG00000031650	927	0	0	0	0	0	0	0	0	0	0	0	0	
ENSXETG00000031651	1293	0	1	1	0	0	0	0	0	0	0	1	1	
ENSXETG00000031652	1149	260	407	274	142	108	135	189	248	121	154	117	111	PTCD2
ENSXETG00000031653	1640	1180	1735	1450	1880	1557	3621	2957	3420	2022	2182	1533	1662	rilp
ENSXETG00000031654	914	1	0	1	2	2	1	2	1	2	2	4	1	ENSXETG00000031654
ENSXETG00000031655	1110	0	0	0	0	0	0	0	0	0	0	0	0	
ENSXETG00000031656	2364	0	0	0	0	0	0	0	0	0	0	0	0	
ENSXETG00000031657	129	0	0	0	0	0	0	0	0	0	0	0	0	
ENSXETG00000031658	2193	0	0	0	0	0	0	0	0	0	0	0	0	
ENSXETG00000031659	441	8	7	4	24	15	14	3	7	7	9	8	7	TEX36
ENSXETG00000031660	2862	30	50	37	98	84	188	1017	1010	715	192	118	148	DENND2A
ENSXETG00000031661	1204	285	350	322	324	208	212	473	525	334	123	102	88	RTCA
ENSXETG00000031662	507	0	0	0	0	0	0	0	1	0	0	0	0	ENSXETG00000031662
ENSXETG00000031663	1083	0	3	2	0	0	0	1	0	0	0	0	0	ENSXETG00000031663
ENSXETG00000031664	1122	1	3	1	18	26	19	27	35	16	48	30	50	SSTR4
ENSXETG00000031665	4047	194	256	228	412	294	782	387	431	248	518	375	401	hps4
ENSXETG00000031666	578	0	1	0	0	0	1	3	2	0	1	1	2	
ENSXETG00000031667	573	0	0	0	0	0	0	0	1	2	0	1	2	ENSXETG00000031667
ENSXETG00000031668	1206	0	0	0	0	0	0	0	0	0	0	0	0	
ENSXETG00000031669	1416	2	2	3	2	2	1	3	1	3	3	4	1	ENSXETG00000031669
ENSXETG00000031670	3357	63	110	66	229	213	179	316	380	220	122	77	110	cenpo
ENSXETG00000031671	2514	0	0	0	0	0	0	0	0	0	0	0	0	
ENSXETG00000031672	798	31	63	41	73	58	95	230	237	142	49	40	50	ENSXETG00000031672
ENSXETG00000031673	1075	0	0	0	1	1	5	1	0	0	4	0	2	ENSXETG00000031673
ENSXETG00000031674	2746	153	221	188	439	337	326	564	681	384	591	446	497	ENSXETG00000031674

ENSXETG00000031675	2475	14	18	12	116	86	199	802	884	568	343	243	274	MRVI1
ENSXETG00000031676	2749	5	6	6	6	2	21	71	82	53	23	19	7	cib2
ENSXETG00000031677	1847	0	0	0	46	28	4	2	0	0	6	4	1	ENSXETG00000031677
ENSXETG00000031678	119	0	0	0	0	0	0	0	0	0	0	0	0	
ENSXETG00000031679	214	0	0	0	0	0	0	0	0	0	0	0	0	
ENSXETG00000031680	2754	16	26	17	94	90	131	493	565	310	418	263	331	ENSXETG00000031680
ENSXETG00000031681	498	0	0	0	0	0	0	0	0	0	0	0	0	
ENSXETG00000031682	846	0	0	0	0	0	1	0	0	0	0	0	1	ENSXETG00000031682
ENSXETG00000031683	516	0	0	0	0	0	0	0	0	0	0	0	0	
ENSXETG00000031684	408	0	0	0	0	0	0	0	1	0	1	0	0	ENSXETG00000031684
ENSXETG00000031685	1419	2	0	2	3	0	2	3	2	2	2	0	1	ENSXETG00000031685
ENSXETG00000031686	465	0	0	0	7	5	8	10	10	7	19	6	9	ALKAL2
ENSXETG00000031687	2280	122	197	153	352	224	419	558	579	322	380	270	287	ENSXETG00000031687
ENSXETG00000031688	1173	0	0	0	1	0	0	0	0	0	0	0	0	
ENSXETG00000031689	4183	7966	11039	9392	12326	9568	17065	1429	1645	947	2801	2055	2280	EHHADH
ENSXETG00000031690	154	128	207	158	191	168	389	640	680	479	980	614	606	5_8S_rRNA
ENSXETG00000031691	909	69	103	73	61	39	109	193	220	135	153	102	124	BSPRY
ENSXETG00000031692	513	0	0	1	1	0	0	0	0	0	0	0	0	
ENSXETG00000031693	1857	3	6	2	10	6	10	13	17	11	23	15	19	ENSXETG00000031693
ENSXETG00000031694	501	0	0	0	0	1	0	0	0	0	1	0	1	ENSXETG00000031694
ENSXETG00000031695	70	0	0	0	0	0	0	0	0	0	0	0	0	
ENSXETG00000031696	507	0	0	0	0	0	0	0	0	0	0	0	0	
ENSXETG00000031697	3771	8	11	14	19	18	49	97	113	62	67	52	45	SH3TC1
ENSXETG00000031698	2545	16	24	19	35	22	55	628	694	387	86	57	53	NEXN
ENSXETG00000031699	888	0	0	0	0	1	1	1	0	0	0	0	0	ENSXETG00000031699
ENSXETG00000031700	1317	0	0	0	0	0	0	0	0	0	0	0	0	
ENSXETG00000031701	164	0	0	0	0	0	0	0	0	0	0	0	0	
ENSXETG00000031702	384	0	0	0	0	0	0	0	0	0	2	1	1	ENSXETG00000031702
ENSXETG00000031703	3344	58	75	71	169	124	281	458	504	331	390	252	284	SNX25
ENSXETG00000031704	70	0	0	0	0	0	0	0	0	0	0	0	0	
ENSXETG00000031705	507	0	0	0	0	0	0	0	0	0	0	0	0	
ENSXETG00000031706	510	0	0	0	0	0	0	0	0	0	0	0	0	
ENSXETG00000031707	939	0	0	0	0	0	0	0	0	0	0	0	0	
ENSXETG00000031708	57	0	0	0	0	0	0	0	0	0	0	0	0	
ENSXETG00000031709	774	3	3	3	48	33	4	17	18	17	5	3	10	c7orf57
ENSXETG00000031710	164	0	0	0	0	0	0	0	0	0	0	0	0	
ENSXETG00000031711	582	0	0	0	3	7	1	0	0	0	1	2	2	ENSXETG00000031711
ENSXETG00000031712	1416	1	0	0	16	8	23	25	17	12	12	12	21	ADRA1A
ENSXETG00000031713	1377	0	0	0	0	2	0	2	1	0	0	1	0	ENSXETG00000031713
ENSXETG00000031714	390	0	0	0	0	0	0	0	0	0	0	0	0	ENSXETG00000031714
ENSXETG00000031715	148	0	0	0	0	0	0	0	0	0	0	0	0	
ENSXETG00000031716	924	0	0	0	0	0	0	0	0	0	0	0	0	
ENSXETG00000031717	119	0	0	0	0	0	0	0	0	0	0	0	0	
ENSXETG00000031718	963	4467	6652	5815	4399	3163	5732	1462	1530	948	2641	1914	1930	ENSXETG00000031718
ENSXETG00000031719	2085	7	10	15	15	18	33	318	417	235	76	73	60	DPY19L3
ENSXETG00000031720	978	0	0	0	0	1	0	0	0	0	1	1	0	ENSXETG00000031720
ENSXETG00000031721	606	3	4	2	2	2	1	32	42	27	4	5	6	ENSXETG00000031721
ENSXETG00000031722	2343	0	0	0	0	0	0	0	0	0	0	0	0	
ENSXETG00000031723	1479	0	1	1	8	5	12	72	49	39	47	23	35	CHRNA3
ENSXETG00000031724	312	0	0	0	0	0	0	0	0	0	0	0	0	
ENSXETG00000031725	219	13	18	11	15	6	30	14	15	7	40	14	16	U3
ENSXETG00000031726	1080	0	1	1	1	0	2	20	15	11	5	0	1	ENSXETG00000031726
ENSXETG00000031727	2278	58	88	78	186	156	157	272	315	167	145	105	102	ttc26
ENSXETG00000031728	1977	6	4	3	18	10	19	6	2	2	2	2	2	F13B
ENSXETG00000031729	2296	843	1151	1014	605	460	1578	918	1156	639	996	716	833	phtf1
ENSXETG00000031730	1440	262	383	353	819	588	1554	634	701	393	1405	932	1049	ENSXETG00000031730
ENSXETG00000031731	1833	141	230	174	884	638	1104	1071	1118	728	875	584	643	ENSXETG00000031731
ENSXETG00000031732	2160	0	1	0	7	0	1	1	1	1	1	2	1	ENSXETG00000031732
ENSXETG00000031733	498	0	0	0	0	0	0	0	0	0	0	0	0	
ENSXETG00000031734	510	0	0	0	0	0	0	0	0	0	0	0	0	
ENSXETG00000031735	2381	44	41	46	160	130	264	514	660	420	208	159	176	myct1
ENSXETG00000031736	2688	0	0	0	0	0	0	0	0	0	0	0	0	
ENSXETG00000031737	2571	97	139	107	59	48	101	858	1056	620	347	222	247	ENSXETG00000031737
ENSXETG00000031738	2273	186	302	242	759	547	284	996	1079	635	443	297	299	ENSXETG00000031738
ENSXETG00000031739	2781	0	0	0	0	0	0	0	2	0	2	1	0	ANO4
ENSXETG00000031740	873	0	0	0	1	0	0	0	0	1	5	0	2	ENSXETG00000031740
ENSXETG00000031741	420	0	0	0	0	0	0	0	0	0	0	0	0	
ENSXETG00000031742	1767	26	30	34	216	122	203	406	530	282	308	213	235	CALCOCO1
ENSXETG00000031743	750	0	0	0	0	0	0	1	1	0	0	0	0	ENSXETG00000031743
ENSXETG00000031744	1173	5	1	7	1	5	2	15	14	7	6	8	5	ENSXETG00000031744
ENSXETG00000031745	189	0	0	0	0	0	0	0	0	0	0	0	0	
ENSXETG00000031746	783	0	1	0	3	1	1	333	409	245	17	19	18	ENSXETG00000031746
ENSXETG00000031747	3399	0	0	0	77	60	6	2	4	6	8	8	7	ENSXETG00000031747
ENSXETG00000031748	2580	0	0	0	0	0	0	2	0	0	0	1	0	
ENSXETG00000031749	5788	487	737	593	1444	1102	1503	2015	2313	1404	2152	1549	1690	madd
ENSXETG00000031750	393	0	0	0	0	0	0	0	2	0	0	0	0	hist1h2ad
ENSXETG00000031751	1240	0	0	0	0	0	0	0	0	0	0	0	0	ferd3l
ENSXETG00000031752	725	0	0	0	0	1	0	11	16	15	2	0	0	ENSXETG00000031752
ENSXETG00000031753	3058	30	59	43	81	66	247	164	221	126	333	253	202	nr4a2
ENSXETG00000031754	1293	0	0	0	2	0	1	0	0	0	2	1	0	ENSXETG00000031754
ENSXETG00000031755	164	0	0	0	0	0	0	0	0	0	0	0	0	
ENSXETG00000031756	1149	0	0	0	1	1	1	0	0	0	1	2	3	ENSXETG00000031756
ENSXETG00000031757	1506	0	0	0	20	15	7	2	5	1	22	14	20	ENSXETG00000031757
ENSXETG00000031758	567	720	940	818	1294	972	1083	97	118	74	670	493	528	ENSXETG00000031758
ENSXETG00000031759	1353	828	1255	1018	824	646	1451	194	210	150	578	383	429	NR1I3
ENSXETG00000031760	2331	647	963	817	769	595	1313	744	857	498	1237	966	992	nadsyn1
ENSXETG00000031761	133	2	0	1	0	0	0	12	8	7	1	1	0	ENSXETG00000031761
ENSXETG00000031762	1299	0	0	0	0	0	0	0	0	0	0	0	0	
ENSXETG00000031763	918	0	0	0	0	0	0	0	0	0	0	0	0	
ENSXETG00000031764	648	24	25	17	49	35	75	98	73	67	126	93	105	ENSXETG00000031764
ENSXETG00000031765	2764	137	201	162	188	129	110	208	261	151	208	132	165	asphd2
ENSXETG00000031766	115	0	0	0	0	0	0	0	0	0	0	0	0	
ENSXETG00000031767	1813	7	20	12	15	16	5	51	46	40	20	7	2	zgrf1
ENSXETG00000031768	792	133	189	154	327	262	661	584	639	410	448	339	351	ENSXETG00000031768
ENSXETG00000031769	149	0	0	0	0	0	0	0	0	0	0	0	0	
ENSXETG00000031770	2573	535	668	546	484	396	798	887	1108	619	392	291	306	SENP5
ENSXETG00000031771	1273	295	407	322	337	206	454	476	578	321	395	284	304	KIN
ENSXETG00000031772	1119	15	24	22	45	38	35	59	60	46	39	33	33	ENSXETG00000031772
ENSXETG00000031773	750	113	166	139	89	48	151	2171	2544	1647	243	192	190	TIMP1
ENSXETG00000031774	975	0	0	0	0	0	0	0	0	0	0	0	0	
ENSXETG00000031775	3648	2	1	0	2	0	4	0	6	2	7	6	10	ENSXETG00000031775
ENSXETG00000031776	708	50	59	68	36	31	70	69	121	68	49	17	32	ENSXETG00000031776
ENSXETG00000031777	6096	14	38	31	115	121	210	287	305	208	186	151	177	NOTCH4
ENSXETG00000031778	2181	1	2	0	17	22	66	93	89	60	134	88	115	VWCE
ENSXETG00000031779	1380	0	0	0	0	0	0	0	0	0	0	0	0	
ENSXETG00000031780	519	0	0	0	0	0	0	0	0	0	0	0	0	
ENSXETG00000031781	1770	52	91	72	118	83	225	282	381	171	272	194	235	limd1
ENSXETG00000031782	1854	0	0	0	0	2	0	4	4	3	0	0	1	KBTBD12
ENSXETG00000031783	1362	337	490	409	211	161	253	636	677	456	156	112	103	ENSXETG00000031783
ENSXETG00000031784	70	0	0	0	0	0	0	0	0	0	0	0	0	
ENSXETG00000031785	921	0	0	0	0	0	0	3	2	2	1	0	0	ENSXETG00000031785

ENSXETG00000031786	549	0	0	0	4	0	0	3	2	1	1	0	0	HPCAL4
ENSXETG00000031787	1671	0	1	0	0	1	0	2	4	2	2	3	6	ENSXETG00000031787
ENSXETG00000031788	1137	0	0	0	0	0	0	0	0	0	1	1	1	ENSXETG00000031788
ENSXETG00000031789	1188	0	0	0	0	0	0	0	0	0	0	0	0	
ENSXETG00000031790	478	0	0	0	0	0	0	0	0	0	0	0	0	ENSXETG00000031790
ENSXETG00000031791	912	0	0	0	0	0	0	0	0	0	0	1	0	ENSXETG00000031791
ENSXETG00000031792	957	1	0	0	1	0	1	3	5	4	1	1	1	ENSXETG00000031792
ENSXETG00000031793	345	105	172	127	88	52	129	86	102	60	99	90	74	ENSXETG00000031793
ENSXETG00000031794	912	484	685	561	266	209	581	539	722	370	284	245	268	TRADD
ENSXETG00000031795	5706	2	9	1	22	15	79	171	229	123	141	95	110	abca5
ENSXETG00000031796	1305	0	0	0	0	0	0	0	0	0	0	0	0	
ENSXETG00000031797	1524	0	0	0	0	0	1	0	0	0	0	0	0	
ENSXETG00000031798	1143	0	0	0	1	0	0	0	0	0	3	1	1	ENSXETG00000031798
ENSXETG00000031799	498	0	1	3	1	3	2	0	2	2	2	2	3	ENSXETG00000031799
ENSXETG00000031800	2949	85	138	132	316	204	455	696	784	479	494	354	407	ENSXETG00000031800
ENSXETG00000031801	375	0	0	0	4	1	0	0	0	1	0	0	0	ENSXETG00000031801
ENSXETG00000031802	2574	0	0	0	0	0	1	4	3	3	2	1	3	KIAA1024
ENSXETG00000031803	960	0	0	0	0	0	1	8	8	6	0	0	0	ENSXETG00000031803
ENSXETG00000031804	783	11	22	16	35	27	39	40	59	26	47	31	38	ENSXETG00000031804
ENSXETG00000031805	714	0	0	0	0	0	0	0	0	0	0	0	0	
ENSXETG00000031806	70	0	0	0	0	0	0	0	0	0	0	0	0	
ENSXETG00000031807	1107	0	0	0	0	0	0	0	0	0	0	0	0	
ENSXETG00000031808	164	0	0	0	0	0	0	0	0	0	0	0	0	
ENSXETG00000031809	1259	984	1468	1306	1044	860	1588	1085	1237	750	1234	972	1022	DNAJA3
ENSXETG00000031810	825	0	0	0	0	0	0	0	0	0	0	0	0	
ENSXETG00000031811	1059	13	12	16	62	50	13	8	21	6	8	5	4	STPG1
ENSXETG00000031812	3766	324	488	391	861	715	1075	1483	1720	1092	1341	992	1110	ATF7IP
ENSXETG00000031813	546	9	14	13	72	40	54	37	41	19	57	43	33	ENSXETG00000031813
ENSXETG00000031814	1311	0	0	0	0	0	0	0	0	0	0	0	0	
ENSXETG00000031815	1827	0	0	0	0	0	0	0	0	0	0	0	0	
ENSXETG00000031816	348	0	0	0	6	5	6	16	18	6	25	17	19	ENSXETG00000031816
ENSXETG00000031817	2277	557	787	722	1245	959	1318	1481	1620	994	1472	966	1165	ikbkg
ENSXETG00000031818	6064	395	592	531	1320	955	1725	1432	1625	924	1647	1253	1288	ATG2A
ENSXETG00000031819	2319	0	0	0	0	0	0	0	0	0	0	0	0	
ENSXETG00000031820	549	0	0	0	7	8	4	21	24	11	17	5	12	TTC9B
ENSXETG00000031821	1113	0	0	0	0	0	0	0	0	0	0	0	0	
ENSXETG00000031822	315	0	1	0	0	3	1	2	1	3	2	2	0	ENSXETG00000031822
ENSXETG00000031823	3258	9	5	2	28	37	85	658	727	442	123	82	108	PDGFRB
ENSXETG00000031824	945	12	11	11	8	5	17	67	86	45	23	36	30	ENSXETG00000031824
ENSXETG00000031825	624	0	0	0	0	0	0	0	0	0	0	0	0	
ENSXETG00000031826	2475	0	0	0	0	0	0	1	1	1	0	2	0	ENSXETG00000031826
ENSXETG00000031827	2559	0	0	0	0	0	0	0	0	0	0	0	0	
ENSXETG00000031828	942	0	0	0	0	0	0	0	0	0	0	0	0	
ENSXETG00000031829	651	5	6	8	5	3	6	70	99	57	19	19	8	ENSXETG00000031829
ENSXETG00000031830	507	0	0	0	1	0	0	0	2	0	1	1	0	ENSXETG00000031830
ENSXETG00000031831	510	0	0	0	0	0	0	0	0	0	0	0	0	
ENSXETG00000031832	2211	391	582	449	1100	788	1872	1862	2045	1234	1619	1247	1266	CMIP
ENSXETG00000031833	534	0	0	0	0	2	0	0	0	0	0	1	0	
ENSXETG00000031834	528	0	0	0	0	0	0	0	0	0	0	0	0	
ENSXETG00000031835	1191	0	0	0	0	0	0	0	0	0	0	0	0	
ENSXETG00000031836	945	0	0	0	1	0	0	0	0	0	1	2	1	ENSXETG00000031836
ENSXETG00000031837	73	0	0	0	0	0	0	0	0	0	0	0	0	
ENSXETG00000031838	1308	1	0	0	2	3	3	4	7	5	2	2	6	ENSXETG00000031838
ENSXETG00000031839	599	38	53	47	17	13	17	28	44	18	11	12	11	ENSXETG00000031839
ENSXETG00000031840	969	0	0	0	0	0	0	1	2	0	0	0	0	
ENSXETG00000031841	3664	840	1290	1075	1801	1276	4918	1739	1971	1243	5468	3900	4301	MGC145244
ENSXETG00000031842	1104	7	10	7	31	26	35	16	19	15	37	22	42	SSTR1
ENSXETG00000031843	1889	0	2	3	0	1	1	6	2	4	4	2	1	SSC4D
ENSXETG00000031844	1212	0	0	0	0	0	0	0	0	0	0	0	0	
ENSXETG00000031845	1203	0	0	0	0	0	0	0	1	0	0	0	0	
ENSXETG00000031846	1848	439	548	472	423	341	558	536	613	395	454	334	347	RUNDC1
ENSXETG00000031847	3600	129	150	148	225	128	415	354	399	235	287	191	243	VCPIP1
ENSXETG00000031848	6276	17	19	23	119	86	232	268	344	213	365	260	265	CACNA1C
ENSXETG00000031849	2202	69	111	94	224	177	45	9	3	3	22	12	32	PPEF2
ENSXETG00000031850	405	0	1	1	0	1	0	10	31	20	1	0	0	ENSXETG00000031850
ENSXETG00000031851	1155	0	0	0	1	1	1	0	0	0	3	3	4	ENSXETG00000031851
ENSXETG00000031852	70	0	0	0	0	0	0	0	0	0	0	0	0	
ENSXETG00000031853	726	0	0	0	0	0	1	14	9	1	1	1	2	CD302
ENSXETG00000031854	2902	1	1	2	1	1	0	0	0	1	2	1	1	megs
ENSXETG00000031855	1578	92	131	111	233	200	289	750	893	514	449	299	330	ENSXETG00000031855
ENSXETG00000031856	2262	520	729	665	902	634	2370	3735	4157	2678	961	702	776	NAGLU
ENSXETG00000031857	324	0	0	0	0	0	0	0	0	0	0	0	0	
ENSXETG00000031858	2685	81	98	96	194	137	245	253	252	180	386	296	318	ENSXETG00000031858
ENSXETG00000031859	4613	0	0	0	1	0	2	4	3	0	11	6	6	ENSXETG00000031859
ENSXETG00000031860	387	0	0	0	1	0	1	1	0	0	1	0	0	ENSXETG00000031860
ENSXETG00000031861	486	0	0	0	0	0	1	0	0	0	0	0	0	ENSXETG00000031861
ENSXETG00000031862	279	62	84	90	204	161	228	240	229	156	261	161	174	ENSXETG00000031862
ENSXETG00000031863	513	0	0	0	0	0	0	3	2	2	0	0	0	
ENSXETG00000031864	7071	1081	1594	1298	1544	1164	1531	1810	1986	1221	1242	880	982	SEC16A
ENSXETG00000031865	1146	0	0	0	3	1	0	0	0	0	1	0	0	ENSXETG00000031865
ENSXETG00000031866	2277	0	0	0	0	0	0	0	0	0	0	0	0	
ENSXETG00000031867	141	0	0	0	0	0	0	0	0	0	0	0	0	
ENSXETG00000031868	795	0	0	0	0	0	0	0	0	0	0	0	0	
ENSXETG00000031869	107	0	0	0	0	0	0	0	0	0	0	0	0	
ENSXETG00000031870	555	0	3	1	1	1	2	6	9	5	4	2	3	TRAPPC3L
ENSXETG00000031871	721	196	314	278	215	148	210	234	295	153	236	194	207	ISCA2
ENSXETG00000031872	816	96	121	84	104	65	147	102	147	83	145	77	107	ENSXETG00000031872
ENSXETG00000031873	2808	0	1	0	20	16	94	638	648	424	330	235	259	ZBTB20
ENSXETG00000031874	534	0	0	0	0	0	0	0	0	0	0	0	0	
ENSXETG00000031875	456	429	646	575	642	474	1004	733	818	533	552	441	443	BAD
ENSXETG00000031876	804	0	0	0	0	0	0	0	0	0	0	0	0	
ENSXETG00000031877	296	0	0	0	0	0	0	0	0	0	0	0	0	
ENSXETG00000031878	2463	0	0	0	0	0	0	0	0	0	0	0	0	
ENSXETG00000031879	1167	0	0	0	0	0	0	0	0	0	0	0	0	ENSXETG00000031879
ENSXETG00000031880	417	1943	2723	2446	2077	1556	2785	2559	2969	1922	2318	1719	1824	fis1
ENSXETG00000031881	1143	0	0	0	1	1	0	3	2	2	1	1	0	EGR3
ENSXETG00000031882	531	0	0	0	0	0	0	0	0	0	0	0	0	
ENSXETG00000031883	1777	2717	4240	3588	918	675	847	346	444	298	296	246	216	tmem86a
ENSXETG00000031884	2705	98	152	143	331	263	434	1534	1689	1074	1249	852	857	PKP2
ENSXETG00000031885	114	0	0	0	0	0	0	0	0	0	0	0	0	
ENSXETG00000031886	463	0	0	0	0	0	0	2	3	3	0	1	1	
ENSXETG00000031887	4200	0	0	0	0	0	0	0	0	1	1	0	0	ENSXETG00000031887
ENSXETG00000031888	561	0	0	0	0	0	0	0	0	0	0	0	0	
ENSXETG00000031889	1107	105	147	140	369	272	493	462	520	287	562	372	388	ENSXETG00000031889
ENSXETG00000031890	516	0	2	0	30	26	1	3	6	1	1	0	1	LDLRAD1
ENSXETG00000031891	393	1	0	0	0	0	0	0	0	0	0	0	0	ENSXETG00000031891
ENSXETG00000031892	921	0	0	0	0	0	0	0	0	0	0	0	0	
ENSXETG00000031893	1209	0	0	0	0	0	0	0	0	0	0	0	0	
ENSXETG00000031894	1146	0	0	0	0	0	0	0	2	0	0	1	0	
ENSXETG00000031895	12594	16	16	22	40	26	58	145	184	119	48	34	32	ENSXETG00000031895
ENSXETG00000031896	6791	338	499	389	1132	818	1885	1703	1865	1258	2113	1488	1580	usf3

ENSXETG00000031897	1422	8	14	13	64	49	119	556	697	383	113	86	101	ACVRL1
ENSXETG00000031898	1557	4204	5911	4988	12014	9481	23694	3869	4511	2557	10655	7323	8376	ENSXETG00000031898
ENSXETG00000031899	109	0	0	0	0	0	0	0	0	0	0	0	0	
ENSXETG00000031900	1185	0	2	0	0	0	0	0	0	0	1	0	0	
ENSXETG00000031901	115	0	0	0	0	0	0	0	0	0	0	0	0	
ENSXETG00000031902	405	0	0	0	0	0	0	0	0	0	0	0	0	ENSXETG00000031902
ENSXETG00000031903	96	3	4	4	4	2	3	6	17	0	3	0	0	SNORD14
ENSXETG00000031904	1169	0	2	2	0	0	1	1	1	1	0	1	0	EPGN
ENSXETG00000031905	2328	0	0	0	0	0	0	0	0	0	0	0	0	
ENSXETG00000031906	1158	1	0	0	0	0	1	0	0	0	0	0	0	
ENSXETG00000031907	513	0	0	0	0	0	0	0	0	0	0	0	0	
ENSXETG00000031908	771	153	233	188	463	322	537	809	817	542	634	411	463	ENSXETG00000031908
ENSXETG00000031909	534	0	0	0	0	0	0	0	0	0	0	0	0	
ENSXETG00000031910	119	0	0	0	0	0	0	0	0	0	0	0	0	
ENSXETG00000031911	396	0	0	0	0	0	0	0	0	0	0	0	0	
ENSXETG00000031912	1570	3	4	5	52	35	3	9	3	1	8	3	3	MOK
ENSXETG00000031913	1557	28	50	45	64	34	105	177	231	125	161	123	116	IDUA
ENSXETG00000031914	2061	152	234	152	193	136	308	261	245	184	328	243	285	WDR24
ENSXETG00000031915	2601	1	1	1	0	0	0	0	0	0	0	0	0	ENSXETG00000031915
ENSXETG00000031916	2596	760	1248	1037	1052	881	1814	3760	4386	2572	430	308	333	fas
ENSXETG00000031917	2469	64	83	69	234	166	286	456	451	297	445	308	307	ENSXETG00000031917
ENSXETG00000031918	2884	42	77	61	107	100	196	1093	1282	727	196	147	152	adam28
ENSXETG00000031919	717	0	0	0	1	0	0	0	1	0	0	0	0	ENSXETG00000031919
ENSXETG00000031920	531	0	0	0	0	0	0	0	0	0	0	0	0	
ENSXETG00000031921	1233	0	0	0	0	0	0	0	0	0	0	0	0	
ENSXETG00000031922	1759	3279	4608	3681	2788	2171	6004	3726	4675	2501	1845	1294	1542	mapk13
ENSXETG00000031923	1143	2	0	0	1	2	0	0	2	0	0	0	0	ENSXETG00000031923
ENSXETG00000031924	1014	0	0	0	0	0	0	0	0	0	0	0	0	
ENSXETG00000031925	588	250	368	299	135	102	32	557	590	367	195	129	165	RNF223
ENSXETG00000031926	1173	0	0	1	0	0	0	0	0	0	0	0	0	
ENSXETG00000031927	1623	0	0	0	3	2	3	21	11	12	5	9	5	GPR162
ENSXETG00000031928	564	3	0	0	3	0	2	147	150	66	19	12	19	ENSXETG00000031928
ENSXETG00000031929	1182	0	0	0	1	0	0	0	0	0	0	0	0	
ENSXETG00000031930	4477	684	1015	794	1357	1065	2300	2267	2671	1519	2075	1474	1731	SBNO1
ENSXETG00000031931	764	0	1	0	14	10	21	0	0	0	41	34	33	ENSXETG00000031931
ENSXETG00000031932	540	0	1	1	1	0	0	1	0	0	7	0	0	ifnl3
ENSXETG00000031933	489	0	0	0	0	0	0	0	0	0	0	0	0	
ENSXETG00000031934	159	0	0	0	0	0	0	0	0	0	0	0	0	
ENSXETG00000031935	1056	28	52	37	64	52	71	2	7	1	63	39	31	ENSXETG00000031935
ENSXETG00000031936	2196	0	1	1	2	0	0	4	2	0	1	0	0	
ENSXETG00000031937	2163	0	0	0	0	0	0	0	0	0	0	0	0	ENSXETG00000031937
ENSXETG00000031938	115	0	0	0	0	0	0	0	0	0	0	0	0	
ENSXETG00000031939	900	0	0	0	0	0	0	0	0	0	0	0	0	
ENSXETG00000031940	1114	431	670	562	780	645	845	859	902	540	940	654	773	ENSXETG00000031940
ENSXETG00000031941	741	91	112	106	119	74	207	131	152	83	141	114	137	ENSXETG00000031941
ENSXETG00000031942	1645	41	48	27	122	73	94	168	233	120	34	12	20	ENSXETG00000031942
ENSXETG00000031943	555	37	48	37	23	20	50	59	63	32	47	26	40	TRAT1
ENSXETG00000031944	1167	0	0	0	0	0	0	0	0	0	0	0	0	
ENSXETG00000031945	2625	3	2	4	1	4	12	2	4	3	5	2	6	ENSXETG00000031945
ENSXETG00000031946	1884	38	56	46	99	73	168	220	243	159	117	93	124	DENND1B
ENSXETG00000031947	1572	307	440	384	222	168	279	283	381	210	193	143	156	ifnlr1
ENSXETG00000031948	3414	240	403	376	768	573	1062	944	1159	679	838	557	621	strip1
ENSXETG00000031949	4069	122	184	164	219	160	302	541	622	350	157	96	135	DHX57
ENSXETG00000031950	1554	40	61	54	94	74	152	111	124	63	179	111	133	ENSXETG00000031950
ENSXETG00000031951	648	0	0	0	0	0	0	0	0	0	0	0	0	ENSXETG00000031951
ENSXETG00000031952	894	0	0	0	0	0	0	0	0	0	0	0	0	
ENSXETG00000031953	119	0	0	0	0	0	0	0	0	0	0	0	0	
ENSXETG00000031954	6234	32	53	38	184	129	149	909	1094	665	432	282	319	MYO9B
ENSXETG00000031955	717	0	0	0	0	0	0	0	0	0	0	0	0	
ENSXETG00000031956	645	61	90	96	59	41	143	350	433	237	137	115	110	BOK
ENSXETG00000031957	606	1329	2030	1850	5533	4004	1301	347	367	225	5386	3732	4130	ENSXETG00000031957
ENSXETG00000031958	375	0	0	0	0	0	0	0	0	0	0	0	0	ENSXETG00000031958
ENSXETG00000031959	3172	335	531	443	887	710	983	1336	1676	931	1215	863	1017	ZZZ3
ENSXETG00000031960	936	0	0	0	0	0	0	0	0	0	0	0	0	
ENSXETG00000031961	1329	3	6	5	35	28	31	21	30	8	24	17	22	HTR7
ENSXETG00000031962	492	0	0	0	0	0	0	0	0	0	0	0	0	
ENSXETG00000031963	312	0	0	0	0	0	0	0	0	0	0	0	0	hist1h4c
ENSXETG00000031964	2481	0	0	0	0	0	0	0	0	0	0	0	0	
ENSXETG00000031965	3810	126	165	145	442	308	524	2419	2607	1621	642	436	532	MAP4K4
ENSXETG00000031966	296	0	0	0	0	0	1	0	0	0	0	0	0	
ENSXETG00000031967	139	2	1	1	2	0	22	44	64	33	76	50	65	U8
ENSXETG00000031968	711	0	0	0	0	1	3	0	0	3	5	2	6	ENSXETG00000031968
ENSXETG00000031969	528	0	0	0	0	0	0	0	0	0	0	0	0	
ENSXETG00000031970	1017	0	0	0	0	0	0	0	0	0	0	0	0	
ENSXETG00000031971	653	6	2	7	44	26	78	137	157	106	73	51	53	ENSXETG00000031971
ENSXETG00000031972	960	0	0	0	0	0	0	0	0	0	0	0	0	
ENSXETG00000031973	459	0	0	0	0	0	0	0	0	0	0	0	0	
ENSXETG00000031974	2334	7	16	10	16	6	38	466	505	289	27	12	14	ENSXETG00000031974
ENSXETG00000031975	870	6369	8988	8027	2916	2322	2953	2888	3613	1961	1152	783	933	ENSXETG00000031975
ENSXETG00000031976	963	0	0	0	0	0	0	0	0	0	0	0	0	
ENSXETG00000031977	88	0	0	0	0	0	0	0	0	0	0	0	0	
ENSXETG00000031978	1119	0	0	0	0	0	0	0	0	0	0	0	0	
ENSXETG00000031979	119	0	0	0	0	0	0	0	0	0	0	0	0	
ENSXETG00000031980	1467	815	1261	1071	707	591	1146	387	379	238	474	339	355	LDHD
ENSXETG00000031981	2348	96	158	107	186	139	299	542	602	345	176	90	122	eme2
ENSXETG00000031982	1623	0	0	0	1	0	0	0	0	0	0	0	0	
ENSXETG00000031983	513	0	1	0	0	1	1	15	11	11	0	1	0	ENSXETG00000031983
ENSXETG00000031984	2181	0	0	0	0	0	0	0	0	0	0	0	0	
ENSXETG00000031985	1694	0	0	0	0	0	0	29	32	35	4	3	0	ENSXETG00000031985
ENSXETG00000031986	1562	1	1	0	1	0	0	1	1	0	0	1	1	c3h12orf40
ENSXETG00000031987	978	0	0	0	0	0	0	0	0	0	0	0	0	
ENSXETG00000031988	1692	2	5	6	5	2	17	9	6	9	15	1	7	wee2
ENSXETG00000031989	927	0	0	0	0	0	0	0	0	0	0	0	0	
ENSXETG00000031990	532	4	9	2	64	31	2	21	22	5	10	7	9	EFCAB2
ENSXETG00000031991	1989	0	2	0	0	0	0	1	1	0	0	0	0	ENSXETG00000031991
ENSXETG00000031992	3420	327	475	398	593	492	627	1084	1248	668	819	549	629	ZNF335
ENSXETG00000031993	951	0	0	0	0	0	0	0	0	0	0	0	0	
ENSXETG00000031994	1119	0	0	0	0	0	0	0	1	0	0	0	1	
ENSXETG00000031995	8373	798	1053	988	2573	2030	2061	3238	3630	2219	2923	2036	2166	UBR5
ENSXETG00000031996	1071	1449	2137	1926	1553	1156	1750	822	1069	563	904	638	660	ENSXETG00000031996
ENSXETG00000031997	2166	266	349	291	395	321	453	503	620	318	409	270	316	ZC3H14
ENSXETG00000031998	1611	300	446	395	261	188	481	499	565	318	435	325	346	ENSXETG00000031998
ENSXETG00000031999	214	0	0	0	0	0	0	0	0	0	0	0	0	
ENSXETG00000032000	483	99	150	135	656	504	1334	1251	1377	902	1047	728	792	ENSXETG00000032000
ENSXETG00000032001	1983	5	9	9	14	7	15	70	72	51	31	15	28	BICD1
ENSXETG00000032002	1440	1	1	2	2	4	5	3	1	0	9	2	2	ENSXETG00000032002
ENSXETG00000032003	107	0	0	0	0	0	0	0	0	0	0	0	0	
ENSXETG00000032004	465	3	5	7	13	10	19	9	6	5	21	9	14	ENSXETG00000032004
ENSXETG00000032005	741	0	0	0	0	0	0	0	0	0	0	0	0	GRXCR2
ENSXETG00000032006	381	0	0	0	0	0	0	0	0	1	0	1	0	ENSXETG00000032006
ENSXETG00000032007	1899	21	26	29	55	34	95	150	203	110	102	59	79	ENSXETG00000032007

ENSXETG00000032008	1863	487	677	583	397	263	729	409	514	310	873	605	676 PIGB
ENSXETG00000032009	2562	3	2	0	1	4	3	72	67	54	14	3	5 KCNQ2
ENSXETG00000032010	306	0	1	1	2	0	0	0	1	0	2	1	0 ENSXETG00000032010
ENSXETG00000032011	2295	0	0	0	0	0	0	0	0	0	0	0	0
ENSXETG00000032012	1091	0	3	0	7	6	9	6	15	9	37	9	15 SHISA8
ENSXETG00000032013	1710	0	0	0	0	0	0	0	0	0	0	0	2 ENSXETG00000032013
ENSXETG00000032014	852	1	0	0	0	0	0	0	0	1	3	0	1 ENSXETG00000032014
ENSXETG00000032015	1221	0	0	1	0	0	3	1	1	0	0	0	0 ENSXETG00000032015
ENSXETG00000032016	912	1	0	0	0	0	0	0	0	0	0	0	0
ENSXETG00000032017	1323	0	0	0	0	0	0	0	0	0	0	0	0
ENSXETG00000032018	698	113	175	166	146	109	328	167	189	123	152	101	97 c11orf74
ENSXETG00000032019	214	0	0	0	0	0	0	0	0	0	0	0	0
ENSXETG00000032020	167	0	0	0	0	0	0	0	0	0	0	0	0
ENSXETG00000032021	945	0	0	0	0	0	0	0	0	0	0	0	0
ENSXETG00000032022	483	0	0	0	0	0	0	0	0	0	0	0	0
ENSXETG00000032023	2706	38	53	64	159	138	315	284	316	213	214	151	177 SMCR8
ENSXETG00000032024	2736	1	3	1	6	11	15	4	6	4	17	13	12 ENSXETG00000032024
ENSXETG00000032025	164	0	0	0	0	0	0	0	0	0	0	0	0
ENSXETG00000032026	597	1	0	1	0	1	0	1	0	0	0	1	1 ENSXETG00000032026
ENSXETG00000032027	1476	0	0	0	0	0	0	0	1	0	0	0	1 RNF207
ENSXETG00000032028	708	0	0	0	0	0	0	0	0	0	0	0	0
ENSXETG00000032029	1188	0	0	0	0	0	0	0	0	0	0	0	0
ENSXETG00000032030	1635	149	217	147	283	182	383	92	114	52	478	298	315 ENSXETG00000032030
ENSXETG00000032031	1545	742	1123	951	635	488	948	1464	1754	1008	1257	872	887 ZDHHC23
ENSXETG00000032032	1874	29	38	37	106	84	190	336	359	176	218	152	177 C11orf95
ENSXETG00000032033	1044	0	0	0	0	0	0	0	0	0	0	0	0
ENSXETG00000032034	522	271	433	359	316	267	520	36	50	19	580	410	487 ENSXETG00000032034
ENSXETG00000032035	1188	0	0	0	1	1	1	2	0	1	3	1	2 ENSXETG00000032035
ENSXETG00000032036	552	0	0	0	1	0	0	0	0	0	0	0	0
ENSXETG00000032037	3216	326	512	457	2303	1768	3126	1620	1833	1087	6216	4339	4992 ENSXETG00000032037
ENSXETG00000032038	807	2	5	9	6	3	3	47	69	27	24	8	9 ENSXETG00000032038
ENSXETG00000032039	2016	130	188	144	197	140	359	429	459	310	365	265	284 GRAMD1A
ENSXETG00000032040	119	0	0	0	0	0	0	0	0	0	0	0	0
ENSXETG00000032041	914	4	5	8	7	14	16	416	496	300	51	31	43 trem2
ENSXETG00000032042	417	1	3	3	11	5	13	76	104	69	65	45	36 STUM
ENSXETG00000032043	762	48	60	55	65	58	48	49	70	37	117	61	106 PCGF5
ENSXETG00000032044	119	0	0	0	0	0	0	0	0	0	0	0	0
ENSXETG00000032045	2088	1	2	0	2	1	0	6	6	4	0	0	0
ENSXETG00000032046	348	0	0	0	0	0	0	4	11	4	0	3	3 ENSXETG00000032046
ENSXETG00000032047	516	39	61	43	92	82	126	224	284	165	146	107	101 ENSXETG00000032047
ENSXETG00000032048	1020	0	0	0	0	0	0	1	0	0	0	0	0 ENSXETG00000032048
ENSXETG00000032049	1011	1	4	2	14	5	35	206	249	155	45	32	26 ENSXETG00000032049
ENSXETG00000032050	933	0	0	0	0	0	0	0	0	0	0	0	0
ENSXETG00000032051	1188	0	0	0	2	0	0	0	0	0	0	1	0 ENSXETG00000032051
ENSXETG00000032052	1542	0	0	0	0	0	0	0	0	0	0	0	0
ENSXETG00000032053	429	1	1	0	1	0	0	1	1	2	0	0	0 ENSXETG00000032053
ENSXETG00000032054	1456	25	20	18	29	21	11	0	0	0	3	4	1 TMEM17
ENSXETG00000032055	924	2	0	1	13	14	36	188	271	142	66	36	42 RBMS3
ENSXETG00000032056	591	0	0	0	0	0	0	0	2	1	1	0	1 ENSXETG00000032056
ENSXETG00000032057	990	0	0	0	0	0	0	0	0	0	0	0	0
ENSXETG00000032058	853	1	1	0	2	5	4	190	228	147	9	9	7 ENSXETG00000032058
ENSXETG00000032059	1605	5	6	13	48	25	39	56	66	41	42	43	48 ENSXETG00000032059
ENSXETG00000032060	717	0	0	0	0	0	0	1	0	0	0	0	0
ENSXETG00000032061	966	0	0	0	0	0	0	0	0	0	0	0	0
ENSXETG00000032062	1299	124	168	152	337	294	559	597	734	421	689	445	543 ENSXETG00000032062
ENSXETG00000032063	1913	64333	94642	80360	48103	36787	78848	1263	1312	743	3850	2627	2910 alpi.2
ENSXETG00000032064	789	2	3	6	4	1	9	88	107	59	11	11	11 ENSXETG00000032064
ENSXETG00000032065	606	0	0	0	0	0	0	0	0	0	0	0	0
ENSXETG00000032066	2886	65	106	104	290	250	484	918	1045	680	590	407	414 sfmbt1
ENSXETG00000032067	1053	0	0	0	0	0	1	1	0	1	0	0	0 ENSXETG00000032067
ENSXETG00000032068	1509	47	67	60	68	57	149	510	604	359	90	54	78 ENSXETG00000032068
ENSXETG00000032069	528	0	0	0	0	0	0	0	0	0	0	0	0
ENSXETG00000032070	897	2	1	4	6	5	6	116	114	79	36	36	31 ANKRD29
ENSXETG00000032071	7408	697	1029	869	1236	938	1813	1549	1947	1090	1377	973	1113 OCRL
ENSXETG00000032072	792	0	0	0	0	0	0	0	0	0	0	0	1
ENSXETG00000032073	1248	1	1	0	4	5	11	0	3	1	1	1	2 ENSXETG00000032073
ENSXETG00000032074	1734	0	0	0	0	0	0	0	0	0	0	0	0
ENSXETG00000032075	1266	5	7	9	10	14	22	7	12	3	18	13	11 ENSXETG00000032075
ENSXETG00000032076	2578	152	256	206	688	552	916	1096	1236	749	1713	1213	1373 ENSXETG00000032076
ENSXETG00000032077	720	526	699	668	526	388	932	520	657	362	528	385	420 ENSXETG00000032077
ENSXETG00000032078	1088	211	333	279	345	287	561	420	442	281	531	342	409 ENSXETG00000032078
ENSXETG00000032079	9741	201	315	250	666	527	907	2743	3287	1883	712	522	550 ENSXETG00000032079
ENSXETG00000032080	107	0	0	0	0	0	0	0	0	0	0	0	0
ENSXETG00000032081	1188	0	0	0	0	0	0	0	0	0	0	0	0
ENSXETG00000032082	2469	0	0	0	0	0	0	0	0	0	0	0	0
ENSXETG00000032083	1488	1	5	0	23	12	68	88	106	79	241	162	185 P4HTM
ENSXETG00000032084	126	0	0	0	0	0	0	0	0	0	0	0	0
ENSXETG00000032085	4066	209	265	240	525	431	1489	737	927	483	996	628	733 GNPTAB
ENSXETG00000032086	1026	0	0	0	0	1	0	0	0	0	0	0	0
ENSXETG00000032087	1416	3	4	5	17	25	38	27	23	20	42	45	27 ENSXETG00000032087
ENSXETG00000032088	2037	0	0	0	0	0	0	0	0	0	0	0	0
ENSXETG00000032089	720	2	4	6	165	125	14	29	25	16	12	2	7 SPEF1
ENSXETG00000032090	1431	0	0	0	1	1	1	1	0	0	0	1	0
ENSXETG00000032091	5172	0	0	2	18	20	65	184	169	110	51	30	41 CPAMD8
ENSXETG00000032092	213	0	0	0	0	0	1	1	2	1	1	0	0 GNG8
ENSXETG00000032093	366	0	0	0	0	0	0	0	0	0	0	0	0 ENSXETG00000032093
ENSXETG00000032094	1230	0	0	0	1	3	1	1	1	0	1	1	0 ENSXETG00000032094
ENSXETG00000032095	83	0	0	0	0	0	0	1	0	0	0	0	0
ENSXETG00000032096	1959	43	42	42	88	90	202	523	628	360	207	150	149 ENSXETG00000032096
ENSXETG00000032097	1419	435	663	541	525	381	1005	936	1021	632	770	536	594 ENSXETG00000032097
ENSXETG00000032098	1314	0	0	0	0	0	1	0	0	0	0	0	0
ENSXETG00000032099	993	0	0	0	0	0	0	0	0	0	0	0	0
ENSXETG00000032100	921	0	0	0	0	0	0	0	0	0	0	0	0
ENSXETG00000032101	873	0	0	0	0	0	0	0	0	0	0	0	0
ENSXETG00000032102	70	0	0	0	0	0	0	0	0	0	0	0	0
ENSXETG00000032103	113	0	0	0	0	0	0	0	0	0	0	0	0
ENSXETG00000032104	2700	600	911	742	2310	1754	4217	5103	5654	3523	6556	4554	4970 ENSXETG00000032104
ENSXETG00000032105	1182	1	2	1	0	0	0	0	0	1	0	0	0
ENSXETG00000032106	1202	173	231	196	208	182	491	415	520	246	453	329	371 ENSXETG00000032106
ENSXETG00000032107	2640	13	21	11	37	32	57	40	40	21	32	20	30 GPRC6A
ENSXETG00000032108	312	0	0	0	0	0	0	0	0	0	0	0	0
ENSXETG00000032109	114	0	0	0	0	0	0	0	0	0	0	0	0
ENSXETG00000032110	816	123	151	140	195	128	214	209	265	154	146	98	114 ENSXETG00000032110
ENSXETG00000032111	285	170	260	215	178	168	302	146	175	84	174	99	125 ENSXETG00000032111
ENSXETG00000032112	1530	1624	2418	1991	2079	1632	2939	1543	1898	1083	3346	2351	2810 FH
ENSXETG00000032113	388	336	471	449	116	83	426	3	4	2	0	0	0 ENSXETG00000032113
ENSXETG00000032114	807	0	0	0	0	0	0	0	0	0	0	0	0
ENSXETG00000032115	399	0	0	0	0	0	0	5	1	2	0	1	0 ENSXETG00000032115
ENSXETG00000032116	531	0	1	1	0	1	0	2	0	0	20	16	11 ENSXETG00000032116
ENSXETG00000032117	2696	2126	3008	2592	2578	1954	6316	2680	3087	1729	4159	2920	3280 il5ra
ENSXETG00000032118	1242	426	608	553	884	630	1481	3219	3855	2244	1263	874	967 tnfrsf1a

ENSXETG00000032119	718	3	3	3	7	6	5	5	8	6	4	2	0	ENSXETG00000032119
ENSXETG00000032120	1230	1	0	0	0	0	1	0	2	2	0	0	0	ENSXETG00000032120
ENSXETG00000032121	70	0	0	0	0	0	0	0	0	0	0	0	0	
ENSXETG00000032122	164	0	0	0	0	0	0	0	0	0	0	0	0	
ENSXETG00000032123	759	5	10	1	4	7	4	23	17	11	22	14	18	ENSXETG00000032123
ENSXETG00000032124	3414	0	0	1	0	1	2	3	7	4	0	0	0	enam
ENSXETG00000032125	945	0	0	0	0	0	0	0	0	0	0	0	0	
ENSXETG00000032126	672	664	988	801	1187	853	1117	1031	1222	694	736	518	578	LXN
ENSXETG00000032127	1560	9	19	13	110	97	131	191	225	122	146	115	111	ENSXETG00000032127
ENSXETG00000032128	70	0	0	0	0	0	0	0	0	0	0	0	0	
ENSXETG00000032129	774	0	0	0	0	0	0	14	24	10	10	10	12	kctd4
ENSXETG00000032130	699	4	2	1	0	0	0	37	42	18	1	1	1	ENSXETG00000032130
ENSXETG00000032131	873	0	0	0	0	0	0	0	0	0	0	0	0	
ENSXETG00000032132	1722	0	0	0	0	0	0	0	0	0	0	0	0	
ENSXETG00000032133	2040	8	23	9	12	12	29	171	223	125	49	45	32	nrros
ENSXETG00000032134	933	0	0	0	0	0	0	0	0	0	0	0	0	
ENSXETG00000032135	2028	0	0	0	0	0	0	0	0	0	0	0	0	
ENSXETG00000032136	70	0	0	0	0	0	0	0	0	0	0	0	0	
ENSXETG00000032137	1383	93	153	140	705	524	1360	562	730	431	579	382	458	SLC46A3
ENSXETG00000032138	2451	0	0	0	0	0	0	0	0	0	0	0	0	
ENSXETG00000032139	656	1543	2241	1884	1040	809	791	1691	1715	1095	508	385	420	mrps6
ENSXETG00000032140	1230	0	0	1	0	2	0	0	1	0	1	4	1	ENSXETG00000032140
ENSXETG00000032141	1491	0	0	0	0	0	0	0	0	0	0	0	0	
ENSXETG00000032142	1380	8	10	9	2	2	2	8	7	8	1	0	0	ENSXETG00000032142
ENSXETG00000032143	1005	0	0	0	1	4	2	0	0	1	3	1	1	ENSXETG00000032143
ENSXETG00000032144	2313	7	23	6	407	336	29	159	183	112	1239	850	947	ankdd1b
ENSXETG00000032145	963	0	0	0	0	0	0	0	1	0	0	0	0	
ENSXETG00000032146	1007	96	123	94	149	108	139	246	261	179	480	351	397	ppp1r1b
ENSXETG00000032147	70	0	0	0	0	0	0	0	0	0	0	0	0	
ENSXETG00000032148	1247	1	0	0	0	0	0	1	3	0	0	0	2	dppa2/4
ENSXETG00000032149	119	0	0	0	0	0	0	0	0	0	0	0	0	
ENSXETG00000032150	641	253	368	362	288	242	441	815	873	484	440	308	378	MED19
ENSXETG00000032151	1434	251	380	312	325	296	678	164	223	136	264	202	226	ENSXETG00000032151
ENSXETG00000032152	70	0	0	0	0	0	0	0	0	0	0	0	0	
ENSXETG00000032153	462	113	187	149	188	141	243	951	1093	664	309	199	223	CARD19
ENSXETG00000032154	972	0	0	0	0	0	0	0	0	0	0	0	0	
ENSXETG00000032155	639	0	0	0	5	3	0	2	4	0	1	0	0	ENSXETG00000032155
ENSXETG00000032156	1185	8	7	7	0	0	12	29	22	13	17	15	18	
ENSXETG00000032157	513	0	0	0	0	0	0	0	0	0	0	0	0	
ENSXETG00000032158	3787	7	6	10	39	25	100	472	520	291	138	113	120	wasf3
ENSXETG00000032159	759	0	0	1	0	0	0	0	0	0	0	0	0	
ENSXETG00000032160	834	0	0	1	2	0	1	1	0	0	0	0	0	ENSXETG00000032160
ENSXETG00000032161	660	1	2	7	23	26	72	12	14	10	1	2	4	MORN3
ENSXETG00000032162	67	4	9	5	2	1	0	6	9	4	2	3	10	SNORD58
ENSXETG00000032163	1062	4	15	12	4	3	5	94	125	66	5	2	9	ENSXETG00000032163
ENSXETG00000032164	528	0	0	0	0	0	0	4	3	2	1	0	0	BHLHA15
ENSXETG00000032165	1011	77	77	75	101	88	146	178	184	138	166	118	131	SWSAP1
ENSXETG00000032166	1032	3	0	3	1	5	4	0	2	1	2	1	1	ENSXETG00000032166
ENSXETG00000032167	588	0	0	0	0	0	0	0	0	0	0	0	0	
ENSXETG00000032168	1233	0	0	0	0	0	0	0	0	0	0	0	0	
ENSXETG00000032169	1590	23	26	16	80	46	123	180	198	128	122	91	97	ENSXETG00000032169
ENSXETG00000032170	228	0	0	0	1	1	1	0	0	0	1	0	0	CTXN2
ENSXETG00000032171	2892	182	283	227	492	361	1158	494	614	377	394	288	368	MAP3K13
ENSXETG00000032172	1188	0	0	0	0	0	0	0	0	0	0	0	0	
ENSXETG00000032173	3516	69	123	96	184	167	217	346	409	233	218	175	161	prdm10
ENSXETG00000032174	363	0	0	0	0	0	0	1	3	1	1	0	2	ENSXETG00000032174
ENSXETG00000032175	1188	0	3	0	0	0	0	0	0	0	2	0	0	ENSXETG00000032175
ENSXETG00000032176	1021	0	0	0	0	0	0	0	0	0	0	0	0	
ENSXETG00000032177	1134	1645	2502	2170	5324	4100	1526	1409	1584	932	882	574	713	cnfn.2
ENSXETG00000032178	717	0	0	0	0	0	0	0	0	0	0	0	0	
ENSXETG00000032179	972	0	0	0	0	0	0	0	0	0	0	0	0	
ENSXETG00000032180	1098	0	0	1	4	0	3	0	1	1	8	5	2	ENSXETG00000032180
ENSXETG00000032181	2840	11	10	12	30	20	11	20	32	14	18	10	20	tmem25
ENSXETG00000032182	6138	7	17	4	61	51	70	329	361	214	104	59	95	MPDZ
ENSXETG00000032183	119	0	0	0	0	0	0	0	0	0	0	0	0	
ENSXETG00000032184	513	0	0	0	0	0	0	0	0	0	0	0	0	
ENSXETG00000032185	1728	8	14	9	68	37	120	126	160	74	90	41	55	ENSXETG00000032185
ENSXETG00000032186	153	0	0	0	0	0	0	0	0	0	0	0	0	
ENSXETG00000032187	1083	62	85	81	177	139	298	356	390	225	390	327	383	ENSXETG00000032187
ENSXETG00000032188	660	21	36	27	52	42	50	161	174	124	154	99	113	ENSXETG00000032188
ENSXETG00000032189	858	2	2	3	29	23	40	230	255	169	107	60	81	VSTM4
ENSXETG00000032190	296	0	0	0	0	0	0	0	0	0	0	0	0	
ENSXETG00000032191	2364	78	87	85	273	185	358	436	535	322	398	269	318	ENSXETG00000032191
ENSXETG00000032192	573	2	0	5	1	4	3	0	0	0	4	7	5	hamp2
ENSXETG00000032193	525	0	0	0	0	0	0	0	0	0	0	0	0	
ENSXETG00000032194	663	23	25	30	69	48	35	28	32	27	69	48	59	ENSXETG00000032194
ENSXETG00000032195	1188	8	13	4	36	28	101	84	119	58	78	57	54	GPRC5B
ENSXETG00000032196	2823	0	6	4	2	2	12	4	1	3	7	0	4	ENSXETG00000032196
ENSXETG00000032197	1347	0	0	0	0	0	0	0	2	1	8	0	4	DRD4
ENSXETG00000032198	1284	2	3	4	19	11	21	87	100	56	26	23	26	rnf150
ENSXETG00000032199	1035	0	0	0	0	0	0	0	0	0	0	0	0	
ENSXETG00000032200	1062	2	6	4	3	2	1	42	93	46	0	1	2	ENSXETG00000032200
ENSXETG00000032201	531	0	0	0	0	0	0	0	0	0	0	0	0	
ENSXETG00000032202	1911	6	6	7	19	21	24	32	34	14	41	22	24	ENSXETG00000032202
ENSXETG00000032203	5610	6	17	7	97	62	166	196	238	133	346	259	308	ENSXETG00000032203
ENSXETG00000032204	3060	9	16	13	21	17	48	199	217	106	64	49	44	PLCL1
ENSXETG00000032205	1293	0	2	0	19	11	0	0	1	1	42	26	22	ONECUT3
ENSXETG00000032206	219	5	3	8	9	6	10	8	6	5	13	7	5	U3
ENSXETG00000032207	1878	105	152	131	299	222	573	333	377	250	438	336	340	ZNF131
ENSXETG00000032208	3789	0	0	0	0	0	0	0	0	0	0	0	0	
ENSXETG00000032209	762	1	0	0	4	7	2	2	7	2	0	2	0	GJB5
ENSXETG00000032210	816	0	1	1	0	0	0	80	99	66	1	1	1	ENSXETG00000032210
ENSXETG00000032211	618	0	0	0	0	0	0	0	0	0	0	0	0	ENSXETG00000032211
ENSXETG00000032212	6375	220	332	296	526	374	465	604	696	431	163	119	149	ENSXETG00000032212
ENSXETG00000032213	2580	0	0	0	0	0	0	0	0	0	0	0	0	
ENSXETG00000032214	1545	0	0	0	2	5	6	42	57	27	49	29	49	LRRTM3
ENSXETG00000032215	2371	207	301	202	249	212	202	323	387	244	360	238	268	shq1
ENSXETG00000032216	141	0	0	0	0	0	0	0	0	0	0	0	0	
ENSXETG00000032217	1839	3	2	5	20	14	16	11	22	20	59	46	70	ENSXETG00000032217
ENSXETG00000032218	3066	78	136	106	277	209	376	645	764	415	492	401	407	KIAA0355
ENSXETG00000032219	296	0	0	0	0	0	0	0	0	0	0	0	0	
ENSXETG00000032220	141	0	0	0	0	0	0	0	0	0	0	0	0	
ENSXETG00000032221	70	0	0	0	0	0	0	0	0	0	0	0	0	
ENSXETG00000032222	2408	0	0	0	0	0	0	0	0	0	0	0	0	
ENSXETG00000032223	5052	11	24	15	114	106	162	886	942	587	173	153	130	CLTCL1
ENSXETG00000032224	984	14	15	16	4	0	15	53	84	39	13	10	12	GPR18
ENSXETG00000032225	3153	64	77	68	148	127	302	118	158	105	276	213	230	CAPS2
ENSXETG00000032226	3585	287	481	406	838	645	1564	1926	2314	1315	1587	1163	1282	RABGAP1
ENSXETG00000032227	768	0	0	0	0	0	0	0	0	0	0	0	0	
ENSXETG00000032228	1095	5	6	6	1	0	0	4	6	6	1	2	1	ENSXETG00000032228
ENSXETG00000032229	591	17	20	24	3	2	12	17	23	10	12	8	4	ENSXETG00000032229

ENSXETG00000032230	2394	0	0	0	0	0	0	0	0	0	0	0	0	
ENSXETG00000032231	1059	0	0	0	0	0	0	0	0	0	0	0	0	
ENSXETG00000032232	2178	92	141	91	254	212	276	201	213	142	858	610	731	mlph
ENSXETG00000032233	951	0	0	0	0	0	0	0	0	0	0	0	0	
ENSXETG00000032234	2204	2	2	2	1	4	1	7	9	5	2	0	2	ENSXETG00000032234
ENSXETG00000032235	939	0	0	0	0	0	0	0	0	0	0	0	0	
ENSXETG00000032236	303	3045	4593	3734	3440	2607	1124	11765	13298	8315	649	384	489	ENSXETG00000032236
ENSXETG00000032237	396	0	0	0	0	0	0	7	4	6	2	0	0	
ENSXETG00000032238	107	0	0	0	0	0	0	0	0	0	0	0	0	
ENSXETG00000032239	528	0	0	0	0	0	0	0	0	0	0	0	0	
ENSXETG00000032240	119	0	0	0	0	0	0	0	0	0	0	0	0	
ENSXETG00000032241	660	60	96	104	210	168	251	292	335	210	352	235	246	ENSXETG00000032241
ENSXETG00000032242	819	0	0	0	0	0	0	0	0	0	0	0	0	ENSXETG00000032242
ENSXETG00000032243	882	1	11	11	21	20	31	46	41	19	92	73	76	ENSXETG00000032243
ENSXETG00000032244	414	0	0	0	0	0	0	0	0	0	0	0	0	
ENSXETG00000032245	1110	5	7	9	17	9	24	68	76	41	30	27	24	CRISPLD1
ENSXETG00000032246	1074	2805	4013	3324	1917	1495	5208	18	18	7	813	525	527	
ENSXETG00000032247	1425	0	0	0	4	2	0	0	0	0	0	0	0	MEIOB
ENSXETG00000032248	296	0	0	0	0	0	0	0	0	0	0	0	0	
ENSXETG00000032249	2199	183	280	216	127	91	278	359	346	247	237	150	191	MTIF2
ENSXETG00000032250	70	0	0	0	0	0	0	0	0	0	0	0	0	
ENSXETG00000032251	1167	0	1	0	0	0	0	0	0	0	0	0	0	
ENSXETG00000032252	963	366	513	381	465	313	458	221	282	155	464	370	361	B3GNT7
ENSXETG00000032253	1188	0	0	0	0	0	0	0	0	0	0	0	0	
ENSXETG00000032254	528	0	0	0	0	0	0	0	0	0	0	0	0	
ENSXETG00000032255	696	0	0	0	0	0	0	0	0	1	0	0	0	
ENSXETG00000032256	3146	110	139	112	275	215	495	562	694	410	500	302	366	rilpl1
ENSXETG00000032257	921	0	0	0	0	0	0	0	0	0	0	0	0	
ENSXETG00000032258	1044	98	119	87	112	71	148	128	214	101	118	97	98	ENSXETG00000032258
ENSXETG00000032259	1017	116	176	158	194	140	278	332	400	216	272	197	270	ENSXETG00000032259
ENSXETG00000032260	648	86	149	120	27	23	14	91	106	67	15	9	10	ENSXETG00000032260
ENSXETG00000032261	3555	0	0	0	0	0	1	0	1	0	0	0	0	ENSXETG00000032261
ENSXETG00000032262	1896	119	196	156	270	210	458	426	464	287	565	420	458	ENSXETG00000032262
ENSXETG00000032263	231	2	2	2	2	0	1	47	60	31	1	0	1	
ENSXETG00000032264	138	0	0	0	0	0	0	0	0	0	0	0	0	
ENSXETG00000032265	948	0	0	0	0	0	0	0	0	0	0	0	0	
ENSXETG00000032266	158	0	0	0	0	0	0	0	0	0	0	0	0	
ENSXETG00000032267	510	0	0	0	0	0	0	0	0	0	0	0	0	
ENSXETG00000032268	513	0	0	0	0	0	0	0	0	0	0	0	0	
ENSXETG00000032269	2073	188	292	268	509	398	787	864	938	628	852	604	640	ENSXETG00000032269
ENSXETG00000032270	1033	2323	3418	2845	1410	1107	1108	230	265	149	345	230	263	mettl7a
ENSXETG00000032271	618	0	0	0	0	0	0	0	0	0	1	0	0	ENSXETG00000032271
ENSXETG00000032272	2478	0	0	0	0	0	0	0	0	0	0	0	0	
ENSXETG00000032273	1224	0	0	0	0	0	0	0	0	0	0	0	0	
ENSXETG00000032274	1449	2	8	10	23	13	42	541	662	420	523	368	420	HTRA3
ENSXETG00000032275	5754	1	3	2	12	5	3	10	5	5	12	9	18	MYH3
ENSXETG00000032276	360	0	0	0	0	0	0	0	0	1	0	0	0	ENSXETG00000032276
ENSXETG00000032277	119	0	0	0	0	0	0	0	0	0	0	0	0	
ENSXETG00000032278	83	0	0	0	0	0	0	0	0	0	0	0	0	
ENSXETG00000032279	1056	0	0	0	1	0	2	1	6	3	4	2	2	ENSXETG00000032279
ENSXETG00000032280	1701	0	0	0	0	0	0	1	0	0	0	0	0	ENSXETG00000032280
ENSXETG00000032281	820	1381	1869	1537	2201	1774	3105	938	1112	635	1578	1005	1230	cisd3
ENSXETG00000032282	1622	0	2	1	3	3	12	116	149	91	23	17	23	ENSXETG00000032282
ENSXETG00000032283	12183	0	0	0	91	67	4	7	9	6	16	20	17	DNAH3
ENSXETG00000032284	1350	0	0	0	0	0	0	0	1	0	0	0	0	
ENSXETG00000032285	324	74	91	72	71	61	107	1398	1545	993	993	622	747	ENSXETG00000032285
ENSXETG00000032286	624	149	208	185	304	268	300	350	378	256	357	247	249	ENSXETG00000032286
ENSXETG00000032287	2040	320	467	401	524	405	1356	679	921	491	853	599	722	TBC1D15
ENSXETG00000032288	119	0	0	0	0	0	0	0	0	0	0	0	0	
ENSXETG00000032289	4482	11	14	14	4	2	26	149	173	107	43	34	40	ENSXETG00000032289
ENSXETG00000032290	509	0	0	0	4	5	3	0	1	0	62	47	44	clps
ENSXETG00000032291	1215	0	1	0	0	0	3	31	30	21	3	4	3	HTR6
ENSXETG00000032292	3825	908	1263	1052	2863	2164	3307	3246	3522	2293	4673	3210	3501	MINK1
ENSXETG00000032293	1269	0	0	0	0	0	0	0	0	0	0	0	0	
ENSXETG00000032294	3509	7	22	13	84	73	150	460	493	285	142	73	99	MGC145260
ENSXETG00000032295	954	0	0	0	0	0	0	0	0	0	0	0	0	
ENSXETG00000032296	3419	298	450	368	422	336	858	1304	1438	881	784	584	671	unc13d
ENSXETG00000032297	603	0	1	0	0	0	0	0	3	3	0	1	0	ENSXETG00000032297
ENSXETG00000032298	774	0	0	0	0	0	0	0	0	0	0	0	0	
ENSXETG00000032299	1293	0	0	0	0	0	0	0	0	0	0	0	0	ENSXETG00000032299
ENSXETG00000032300	840	2	1	0	102	68	8	12	18	7	11	9	6	STMND1
ENSXETG00000032301	561	650	995	814	560	465	739	619	662	433	445	320	363	fmc1
ENSXETG00000032302	1160	1	0	3	0	2	3	41	55	27	25	23	19	ENSXETG00000032302
ENSXETG00000032303	70	0	0	0	0	0	0	0	0	0	0	0	0	
ENSXETG00000032304	2589	0	0	0	0	0	0	0	0	0	0	0	0	
ENSXETG00000032305	2122	45	71	76	105	69	197	199	215	149	254	195	198	ENSXETG00000032305
ENSXETG00000032306	630	88	148	97	85	65	100	132	165	77	105	74	97	ENSXETG00000032306
ENSXETG00000032307	492	0	0	0	0	0	0	0	0	0	0	0	0	
ENSXETG00000032308	3195	1	0	0	1	0	1	1	4	2	3	2	6	ENSXETG00000032308
ENSXETG00000032309	119	0	0	0	0	0	0	0	0	0	0	0	0	
ENSXETG00000032310	2367	0	0	0	0	0	0	0	0	0	0	0	0	
ENSXETG00000032311	1026	0	0	0	0	0	0	0	0	0	0	0	0	
ENSXETG00000032312	1176	0	0	0	0	0	0	0	0	0	0	0	0	
ENSXETG00000032313	1188	0	0	2	4	0	1	0	1	0	2	2	1	ENSXETG00000032313
ENSXETG00000032314	727	1747	2413	2146	4133	3114	4398	1232	1446	941	2080	1514	1663	ENSXETG00000032314
ENSXETG00000032315	1350	6	11	8	6	5	9	66	111	60	38	42	38	MATK
ENSXETG00000032316	1329	0	0	0	0	0	0	0	0	0	0	0	0	
ENSXETG00000032317	1422	83	139	116	113	75	147	281	298	214	124	84	104	TMCO4
ENSXETG00000032318	1188	1	0	0	0	0	0	0	0	0	0	0	0	
ENSXETG00000032319	2268	67	93	81	204	177	272	423	473	302	367	258	268	ENSXETG00000032319
ENSXETG00000032320	832	2	5	5	0	2	7	12	15	13	5	7	7	ENSXETG00000032320
ENSXETG00000032321	918	0	0	0	0	0	0	0	0	0	1	0	0	
ENSXETG00000032322	780	101	136	131	111	112	171	259	308	196	119	81	101	ENSXETG00000032322
ENSXETG00000032323	82	0	0	0	0	0	0	0	0	0	0	0	0	xtr-mir-27c-2
ENSXETG00000032324	1494	18	19	17	34	28	52	142	155	95	46	30	34	KDELC1
ENSXETG00000032325	900	0	0	0	0	0	0	0	0	0	0	0	0	
ENSXETG00000032326	1223	985	1397	1142	872	614	1055	1204	1369	879	375	277	311	sra1
ENSXETG00000032327	2942	35	58	34	131	118	189	251	295	166	221	143	189	ccdc92
ENSXETG00000032328	471	0	0	0	0	0	0	0	0	0	0	0	0	
ENSXETG00000032329	4009	93	129	126	285	231	603	1359	1549	925	1124	794	826	ENSXETG00000032329
ENSXETG00000032330	936	1	3	2	0	0	3	0	0	0	10	12	15	ENSXETG00000032330
ENSXETG00000032331	1131	0	0	0	0	0	0	0	0	0	0	0	0	
ENSXETG00000032332	916	2	2	0	4	5	2	1	2	1	2	1	1	ENSXETG00000032332
ENSXETG00000032333	1410	0	0	0	9	9	8	20	33	14	24	13	23	ENSXETG00000032333
ENSXETG00000032334	984	0	0	0	0	0	0	0	0	0	0	0	0	
ENSXETG00000032335	86	4	10	1	4	1	0	15	18	7	12	10	12	xtr-mir-142-2
ENSXETG00000032336	70	0	0	0	0	0	0	0	0	0	0	0	0	
ENSXETG00000032337	1437	1	0	0	11	6	13	0	1	0	6	2	10	ENSXETG00000032337
ENSXETG00000032338	621	0	0	0	0	0	0	0	0	0	0	0	0	
ENSXETG00000032339	1248	0	0	0	0	0	0	0	0	0	0	0	0	
ENSXETG00000032340	966	0	0	0	0	0	0	0	0	0	0	0	0	ENSXETG00000032340

ENSXETG00000032341	3157	0	2	2	3	0	0	3	5	1	5	0	3	cdcp2
ENSXETG00000032342	155	0	0	0	0	0	0	0	0	0	0	0	0	
ENSXETG00000032343	525	0	0	0	0	0	0	0	0	0	0	1	0	
ENSXETG00000032344	1602	0	0	0	0	0	0	2	1	0	0	1	0	ENSXETG00000032344
ENSXETG00000032345	939	0	0	0	0	0	0	0	0	0	0	0	0	
ENSXETG00000032346	8337	521	703	635	741	545	426	753	967	531	1062	775	818	UTP20
ENSXETG00000032347	678	0	1	0	0	0	1	2	2	2	0	0	0	ENSXETG00000032347
ENSXETG00000032348	756	38	39	38	269	249	254	16	10	18	125	105	92	ETFBKMT
ENSXETG00000032349	303	0	0	0	1	0	0	0	1	1	0	0	2	ENSXETG00000032349
ENSXETG00000032350	296	0	0	0	0	0	0	0	0	0	0	0	0	
ENSXETG00000032351	1122	118	183	149	166	122	126	192	240	108	210	159	173	ENSXETG00000032351
ENSXETG00000032352	708	7	7	7	30	18	25	18	34	21	67	36	44	ENSXETG00000032352
ENSXETG00000032353	960	0	0	0	0	0	0	0	0	0	0	0	0	
ENSXETG00000032354	141	0	0	0	0	0	0	0	0	0	0	0	0	
ENSXETG00000032355	70	0	0	0	0	0	0	0	0	0	0	0	0	
ENSXETG00000032356	296	0	0	0	0	0	0	0	0	0	0	0	0	
ENSXETG00000032357	2369	1	0	1	2	6	0	1	1	2	11	2	5	ENSXETG00000032357
ENSXETG00000032358	936	4	3	3	12	11	12	10	12	10	15	7	9	ENSXETG00000032358
ENSXETG00000032359	1066	0	1	2	0	0	2	5	1	3	2	5	0	spdya
ENSXETG00000032360	1170	9	16	6	19	13	23	274	331	185	24	19	11	NPHS2
ENSXETG00000032361	528	0	0	0	0	0	0	0	0	0	0	0	0	
ENSXETG00000032362	395	0	1	0	0	0	1	4	5	4	1	2	4	ENSXETG00000032362
ENSXETG00000032363	1014	0	0	0	0	0	0	0	0	0	0	0	0	POU4F3
ENSXETG00000032364	750	0	0	0	0	0	0	0	1	0	0	0	0	MAFA
ENSXETG00000032365	393	0	0	0	0	0	0	0	0	0	0	0	0	hist1h2aj
ENSXETG00000032366	2385	18	19	21	204	146	141	243	283	134	494	329	408	LGR5
ENSXETG00000032367	456	0	2	1	7	5	12	129	124	86	17	13	14	ENSXETG00000032367
ENSXETG00000032368	1518	0	0	0	0	0	0	0	0	0	2	2	3	ENSXETG00000032368
ENSXETG00000032369	1333	1	1	1	5	2	8	0	4	0	25	14	8	ENSXETG00000032369
ENSXETG00000032370	729	2	1	0	3	2	0	6	9	7	6	3	5	ENSXETG00000032370
ENSXETG00000032371	492	0	0	0	0	0	0	1	0	0	0	0	0	
ENSXETG00000032372	296	0	0	0	0	0	0	0	0	0	0	0	0	Metazoa_SRP
ENSXETG00000032373	375	0	0	0	0	0	0	0	0	0	0	0	0	
ENSXETG00000032374	876	0	0	0	0	0	0	0	0	0	0	0	0	
ENSXETG00000032375	5589	297	470	365	891	677	1339	945	1153	701	1223	901	1070	ENSXETG00000032375
ENSXETG00000032376	1774	147	242	191	169	124	30	487	519	315	47	32	36	ENSXETG00000032376
ENSXETG00000032377	1188	0	0	3	0	0	0	0	0	0	0	2	1	ENSXETG00000032377
ENSXETG00000032378	936	0	0	0	0	0	0	0	0	0	0	0	0	
ENSXETG00000032379	1299	0	0	0	3	1	1	0	4	2	9	3	5	GPR22
ENSXETG00000032380	2349	0	0	0	0	0	0	0	0	0	0	0	0	
ENSXETG00000032381	1746	0	0	0	0	1	0	0	0	0	0	0	0	ENSXETG00000032381
ENSXETG00000032382	214	0	0	0	0	0	0	0	0	0	0	0	0	
ENSXETG00000032383	1615	16	19	9	66	53	101	149	171	110	80	58	66	arl13b
ENSXETG00000032384	951	0	0	0	0	0	0	0	0	0	0	0	0	
ENSXETG00000032385	1080	0	0	0	1	0	1	0	4	0	2	3	1	GLP1R
ENSXETG00000032386	318	1	0	2	78	76	2	0	0	0	146	103	120	KCNE2
ENSXETG00000032387	993	14	18	13	28	35	23	127	165	85	42	33	26	ENSXETG00000032387
ENSXETG00000032388	2415	0	0	0	0	0	0	0	0	0	0	0	0	
ENSXETG00000032389	537	144	194	152	227	194	237	261	235	168	247	210	229	ENSXETG00000032389
ENSXETG00000032390	109	0	0	0	0	0	0	0	0	0	0	0	0	
ENSXETG00000032391	1726	521	859	700	541	377	556	571	690	376	445	335	408	cxorf38
ENSXETG00000032392	2795	99	159	134	211	162	345	294	323	214	432	326	312	MGC145518
ENSXETG00000032393	119	0	0	0	0	0	0	0	0	0	0	0	0	
ENSXETG00000032394	2661	59	82	72	156	135	202	364	400	230	254	207	212	ENSXETG00000032394
ENSXETG00000032395	1542	1	5	3	5	1	22	29	42	24	24	24	20	ENSXETG00000032395
ENSXETG00000032396	1782	60	80	78	208	149	363	1095	1240	708	219	188	197	CD93
ENSXETG00000032397	912	1	3	2	12	11	8	2	3	2	3	3	6	t2r53
ENSXETG00000032398	1568	278	358	333	382	268	289	30	47	31	339	243	272	ENSXETG00000032398
ENSXETG00000032399	1398	442	647	546	656	548	2032	541	630	382	715	475	555	KYNU
ENSXETG00000032400	1467	12	23	21	82	60	189	205	249	130	151	113	119	ENSXETG00000032400
ENSXETG00000032401	699	0	0	0	0	0	0	0	0	0	0	0	0	ENSXETG00000032401
ENSXETG00000032402	603	1	0	2	1	0	0	0	0	1	1	0	0	ENSXETG00000032402
ENSXETG00000032403	825	0	0	0	2	0	0	11	9	8	29	23	26	ERP27
ENSXETG00000032404	2166	16	20	32	241	144	85	242	259	182	55	41	54	ENSXETG00000032404
ENSXETG00000032405	993	0	0	0	0	0	0	0	0	0	0	0	0	
ENSXETG00000032406	1140	23	48	43	8	5	6	33	34	23	13	5	14	ENSXETG00000032406
ENSXETG00000032407	504	0	0	0	0	0	0	0	0	0	0	0	0	
ENSXETG00000032408	411	0	0	0	1	1	1	0	1	0	3	0	0	CYTL1
ENSXETG00000032409	909	29	25	24	62	72	332	237	351	197	74	29	57	IL17REL
ENSXETG00000032410	984	53	58	53	82	68	106	314	360	210	58	55	41	ENSXETG00000032410
ENSXETG00000032411	2631	0	0	0	0	0	0	0	0	0	0	0	0	ENSXETG00000032411
ENSXETG00000032412	3549	32	47	29	133	94	110	293	347	194	97	48	53	PASK
ENSXETG00000032413	1487	2	1	6	0	0	5	6	0	1	1	0	0	ENSXETG00000032413
ENSXETG00000032414	531	0	0	0	0	0	0	0	0	0	0	0	0	
ENSXETG00000032415	606	0	0	4	5	4	12	89	95	46	12	10	16	EDARADD
ENSXETG00000032416	828	0	0	0	1	0	0	46	74	51	0	0	0	HOXD11
ENSXETG00000032417	1245	0	0	0	0	0	0	0	0	0	0	0	0	SERPINE3
ENSXETG00000032418	954	0	0	0	0	0	0	0	0	0	0	0	0	
ENSXETG00000032419	1044	182	251	197	162	123	375	117	142	73	115	77	75	ENSXETG00000032419
ENSXETG00000032420	864	0	0	0	0	0	0	0	0	0	0	0	0	
ENSXETG00000032421	813	6	6	5	7	2	0	3	5	5	16	12	16	ENSXETG00000032421
ENSXETG00000032422	603	0	0	0	1	0	0	0	0	0	0	0	0	
ENSXETG00000032423	2028	0	5	1	2	4	8	26	27	21	13	3	5	ENSXETG00000032423
ENSXETG00000032424	414	0	0	0	0	0	3	0	0	0	4	1	1	ENSXETG00000032424
ENSXETG00000032425	510	0	0	0	0	0	0	0	0	0	0	0	0	
ENSXETG00000032426	936	0	0	0	0	0	0	0	0	0	0	0	0	
ENSXETG00000032427	70	0	0	0	0	0	0	0	0	0	0	0	0	
ENSXETG00000032428	735	0	2	0	10	4	3	29	42	21	22	15	27	ENSXETG00000032428
ENSXETG00000032429	2757	25	28	37	196	141	81	235	318	174	77	50	49	spice1
ENSXETG00000032430	648	0	0	0	0	1	0	0	0	0	8	5	5	ENSXETG00000032430
ENSXETG00000032431	2663	900	1275	1125	1024	832	1530	1053	1263	706	784	543	627	AFG3L2
ENSXETG00000032432	414	0	0	0	0	0	0	10	14	4	1	0	0	ENSXETG00000032432
ENSXETG00000032433	1122	0	0	0	0	0	0	1	2	1	5	3	2	ENSXETG00000032433
ENSXETG00000032434	975	0	0	0	0	0	0	0	0	0	0	0	0	
ENSXETG00000032435	1518	239	310	275	507	393	997	875	1017	610	1199	833	875	ENSXETG00000032435
ENSXETG00000032436	7284	3	4	2	4	1	10	2	1	0	12	9	14	ENSXETG00000032436
ENSXETG00000032437	894	13	13	7	24	13	64	61	70	34	38	25	33	ENSXETG00000032437
ENSXETG00000032438	1686	78	120	81	188	122	371	326	396	220	295	231	247	ENSXETG00000032438
ENSXETG00000032439	1146	22	32	30	24	19	45	133	146	86	72	54	71	ENSXETG00000032439
ENSXETG00000032440	375	0	0	1	0	0	0	0	0	0	1	0	1	ENSXETG00000032440
ENSXETG00000032441	2358	7	16	8	35	31	42	81	111	73	79	59	70	syt17
ENSXETG00000032442	2481	12	22	15	7	10	13	47	57	33	66	50	39	ENSXETG00000032442
ENSXETG00000032443	642	4	0	0	4	4	8	27	44	35	16	14	11	RAB39B
ENSXETG00000032444	846	1	0	0	1	0	0	0	0	0	0	0	0	ENSXETG00000032444
ENSXETG00000032445	1797	0	0	0	0	1	0	0	1	4	2	0	0	PEX5L
ENSXETG00000032446	2496	0	0	0	0	0	2	0	0	0	0	0	0	
ENSXETG00000032447	393	0	1	2	0	0	0	1	3	2	0	0	0	THRSP
ENSXETG00000032448	501	0	0	0	0	0	0	0	0	0	0	0	0	
ENSXETG00000032449	492	0	0	0	0	0	0	0	0	0	0	0	0	
ENSXETG00000032450	837	0	0	0	0	0	0	0	0	0	0	0	0	ENSXETG00000032450
ENSXETG00000032451	135	0	0	0	0	2	0	0	0	0	0	0	0	SCARNA3

ENSXETG00000032452	2770	6	3	10	41	27	20	100	117	77	69	46	53	ENSXETG00000032452
ENSXETG00000032453	765	4	4	8	21	22	24	97	94	59	46	25	22	ENSXETG00000032453
ENSXETG00000032454	939	0	0	0	0	0	0	0	0	0	0	0	0	
ENSXETG00000032455	1229	0	0	1	1	0	0	0	1	0	0	1	0	ENSXETG00000032455
ENSXETG00000032456	1653	46	78	70	59	30	110	230	293	158	138	82	114	ENSXETG00000032456
ENSXETG00000032457	1188	0	0	0	4	7	4	0	6	3	10	12	14	ENSXETG00000032457
ENSXETG00000032458	1041	1	0	0	2	0	1	0	0	1	5	3	1	ENSXETG00000032458
ENSXETG00000032459	119	0	0	0	0	0	0	0	0	0	0	0	0	
ENSXETG00000032460	375	21	27	17	6	2	12	7	8	9	13	8	10	ENSXETG00000032460
ENSXETG00000032461	408	2	0	0	0	0	0	2	0	2	3	2	2	ENSXETG00000032461
ENSXETG00000032462	1103	57	82	62	127	91	181	162	186	114	344	249	272	smpx
ENSXETG00000032463	5424	17	25	25	21	24	44	236	269	155	119	86	76	NLRC3
ENSXETG00000032464	909	0	0	0	0	0	0	0	0	0	0	0	0	
ENSXETG00000032465	57	0	0	0	0	0	0	0	0	0	0	0	0	
ENSXETG00000032466	1124	607	825	697	786	580	811	1284	1528	906	650	449	500	RNASEH2C
ENSXETG00000032467	1311	59	119	75	94	69	255	1015	1134	654	579	420	465	ENSXETG00000032467
ENSXETG00000032468	119	0	0	0	0	0	0	0	0	0	0	0	0	5S_rRNA
ENSXETG00000032469	363	0	0	0	0	0	0	10	11	4	0	0	0	ENSXETG00000032469
ENSXETG00000032470	1473	0	1	2	3	0	1	6	9	1	61	49	40	ENSXETG00000032470
ENSXETG00000032471	6996	4	5	8	6	8	44	36	38	20	26	21	10	SPTB
ENSXETG00000032472	420	0	0	1	2	2	3	38	36	22	11	7	15	ENSXETG00000032472
ENSXETG00000032473	751	569	859	784	285	179	997	10	4	5	0	0	0	ENSXETG00000032473
ENSXETG00000032474	1221	0	0	0	0	0	0	0	0	0	0	0	0	ENSXETG00000032474
ENSXETG00000032475	979	0	0	0	0	0	0	0	0	0	0	0	0	
ENSXETG00000032476	2004	104	137	133	218	180	337	353	372	260	317	264	269	KLHL11
ENSXETG00000032477	1830	0	0	0	4	0	7	3	3	3	3	4	3	ENSXETG00000032477
ENSXETG00000032478	1023	4	2	4	2	1	0	7	15	5	5	5	5	ENSXETG00000032478
ENSXETG00000032479	504	0	0	0	0	0	0	0	0	0	0	0	0	
ENSXETG00000032480	1068	1	3	1	11	0	13	14	15	5	20	22	25	GDAP1
ENSXETG00000032481	1083	0	0	0	0	0	0	0	1	0	0	0	2	ENSXETG00000032481
ENSXETG00000032482	849	555	867	686	818	642	1152	4880	5651	3261	1111	700	771	ENSXETG00000032482
ENSXETG00000032483	975	0	0	0	0	0	0	0	0	0	0	0	0	ENSXETG00000032483
ENSXETG00000032484	1185	6	3	10	0	0	1	0	0	0	1	1	1	ENSXETG00000032484
ENSXETG00000032485	513	0	0	1	0	0	0	0	0	0	0	0	0	ENSXETG00000032485
ENSXETG00000032486	1533	40	54	46	164	129	294	334	378	190	156	129	153	ENSXETG00000032486
ENSXETG00000032487	119	0	0	0	0	0	0	0	0	0	0	0	0	
ENSXETG00000032488	921	2	3	1	18	15	6	1	5	1	5	6	5	ENSXETG00000032488
ENSXETG00000032489	2421	0	0	0	0	0	0	0	0	0	0	0	0	
ENSXETG00000032490	558	0	0	0	22	23	10	2	5	2	86	39	52	ENSXETG00000032490
ENSXETG00000032491	1287	17	23	21	189	171	51	47	40	22	490	378	443	ENSXETG00000032491
ENSXETG00000032492	393	0	0	0	0	0	0	0	0	0	0	0	0	hist2h2ab
ENSXETG00000032493	119	0	0	0	0	0	0	0	0	0	0	0	0	
ENSXETG00000032494	70	0	0	0	0	0	0	0	0	0	0	0	0	
ENSXETG00000032495	1030	0	0	0	0	0	0	0	0	0	0	0	0	
ENSXETG00000032496	1311	98	163	140	71	58	139	168	234	95	44	26	39	AFG1L
ENSXETG00000032497	96	0	0	0	0	0	0	0	0	0	0	0	0	
ENSXETG00000032498	1428	0	0	0	0	0	0	1	0	0	1	0	0	ENSXETG00000032498
ENSXETG00000032499	498	27	32	29	16	13	65	12	10	6	23	19	26	ENSXETG00000032499
ENSXETG00000032500	1107	0	1	1	20	30	1	1	2	1	10	5	2	ENSXETG00000032500
ENSXETG00000032501	1941	20	48	39	15	13	19	236	247	168	32	26	41	ENSXETG00000032501
ENSXETG00000032502	948	0	0	0	0	0	0	0	0	0	0	0	0	
ENSXETG00000032503	669	1111	1556	1346	1032	788	1534	1356	1452	800	1282	841	1001	MRPS18B
ENSXETG00000032504	3193	4	12	6	117	94	88	85	93	60	129	61	95	ENSXETG00000032504
ENSXETG00000032505	2337	0	0	0	0	0	0	0	0	0	0	0	0	
ENSXETG00000032506	510	0	0	0	0	0	0	0	0	0	1	0	0	
ENSXETG00000032507	600	0	0	0	0	0	0	0	0	0	0	0	0	
ENSXETG00000032508	1095	0	0	0	0	0	0	0	0	0	0	0	0	
ENSXETG00000032509	1086	7	26	13	32	13	59	27	39	16	45	43	39	NMUR1
ENSXETG00000032510	2652	5	8	7	1	3	4	265	400	230	26	24	16	ENSXETG00000032510
ENSXETG00000032511	711	1185	1881	1676	2055	1620	2241	1552	1709	1150	1363	954	1112	ENSXETG00000032511
ENSXETG00000032512	822	101	158	128	99	73	140	154	222	139	162	115	116	ENSXETG00000032512
ENSXETG00000032513	1827	0	4	3	4	1	0	17	26	19	6	1	5	ENSXETG00000032513
ENSXETG00000032514	2875	3787	5472	4717	2706	2113	2311	1712	2015	1167	6832	5021	5460	nfkbiz
ENSXETG00000032515	270	1	1	0	2	1	5	7	4	3	1	3	2	ENSXETG00000032515
ENSXETG00000032516	318	2	3	1	6	2	3	6	5	1	4	5	3	ENSXETG00000032516
ENSXETG00000032517	1594	4022	5508	4913	4782	3697	7785	5261	6291	3680	6029	4142	4686	DKC1
ENSXETG00000032518	312	0	0	0	0	0	0	0	0	0	0	0	0	hist1h4c
ENSXETG00000032519	639	0	0	0	0	0	0	1	0	0	2	0	1	ENSXETG00000032519
ENSXETG00000032520	1398	0	0	0	0	0	0	0	0	0	0	0	0	
ENSXETG00000032521	1827	0	0	0	0	0	1	5	5	4	3	3	3	ENSXETG00000032521
ENSXETG00000032522	2190	0	1	0	10	10	6	12	14	5	14	9	12	SV2A
ENSXETG00000032523	504	0	0	0	0	0	0	0	0	0	0	0	0	
ENSXETG00000032524	1077	74	112	90	150	135	516	282	271	209	321	230	263	trim25
ENSXETG00000032525	1758	2	4	5	2	4	3	179	262	137	11	8	9	P2RX7
ENSXETG00000032526	1014	0	0	0	0	0	0	0	1	0	2	3	2	ENSXETG00000032526
ENSXETG00000032527	822	83	124	118	216	140	254	204	233	143	216	149	198	ENSXETG00000032527
ENSXETG00000032528	15426	0	0	1	0	0	3	64	49	42	4	1	8	SSPO
ENSXETG00000032529	1722	486	700	582	585	397	645	386	484	284	407	338	356	MKKS
ENSXETG00000032530	825	0	0	0	0	0	0	0	1	0	0	0	0	ENSXETG00000032530
ENSXETG00000032531	3381	1	0	5	14	9	20	81	114	59	43	25	33	ENSXETG00000032531
ENSXETG00000032532	119	0	0	0	0	0	0	0	0	0	0	0	0	
ENSXETG00000032533	1140	18	20	27	40	37	54	557	601	376	119	98	99	ENSXETG00000032533
ENSXETG00000032534	846	1	1	0	1	1	1	4	2	0	1	0	0	ENSXETG00000032534
ENSXETG00000032535	8451	8	7	6	118	65	178	4730	5649	3368	367	258	309	FBN2
ENSXETG00000032536	627	0	1	0	0	0	0	0	0	0	0	0	0	
ENSXETG00000032537	7643	0	0	0	3	0	1	24	40	19	5	2	1	PKD1L1
ENSXETG00000032538	2422	362	464	439	526	398	1318	1247	1364	839	755	543	602	ATG7
ENSXETG00000032539	3564	1	0	0	0	0	1	0	1	0	0	0	0	GRM2
ENSXETG00000032540	381	0	0	0	0	0	0	0	0	0	0	0	0	
ENSXETG00000032541	528	4	4	3	5	3	4	9	13	2	13	10	4	SYNDIG1
ENSXETG00000032542	1167	0	0	0	1	0	2	71	82	40	75	52	41	ENSXETG00000032542
ENSXETG00000032543	1413	0	0	0	0	0	0	0	1	0	3	3	4	ENSXETG00000032543
ENSXETG00000032544	1038	13081	19275	16255	4771	3738	6287	313	367	207	204	142	151	apoa5
ENSXETG00000032545	141	0	0	0	0	0	0	0	0	0	0	0	0	
ENSXETG00000032546	849	0	1	3	0	1	0	0	0	0	0	0	1	C6orf58
ENSXETG00000032547	1379	30	55	58	54	46	90	1091	1323	760	180	112	143	PKIB
ENSXETG00000032548	687	213	324	293	217	206	487	709	791	502	157	100	115	GATD1
ENSXETG00000032549	1551	0	1	4	2	1	0	0	0	1	0	0	0	ENSXETG00000032549
ENSXETG00000032550	1584	31	45	33	124	81	186	85	102	51	140	92	105	ENSXETG00000032550
ENSXETG00000032551	2259	500	742	588	239	171	155	561	688	371	124	98	103	GFM1
ENSXETG00000032552	1545	45	70	56	39	36	96	464	500	271	121	99	111	lcp2
ENSXETG00000032553	408	0	0	0	0	0	0	0	0	0	0	0	0	ENSXETG00000032553
ENSXETG00000032554	2307	356	527	448	444	304	286	658	827	461	188	133	128	sec23a
ENSXETG00000032555	516	0	0	0	0	0	0	0	0	0	0	0	0	
ENSXETG00000032556	693	0	0	0	0	0	0	0	0	0	0	0	0	
ENSXETG00000032557	1118	226	290	255	179	136	180	296	283	193	238	174	168	DPH1
ENSXETG00000032558	2195	0	1	2	68	59	6	11	15	0	7	2	7	ENSXETG00000032558
ENSXETG00000032559	783	19	25	20	16	14	30	114	151	91	26	24	24	CEBPE
ENSXETG00000032560	1101	0	0	0	0	0	0	0	0	0	0	0	0	
ENSXETG00000032561	957	0	0	0	0	0	0	0	0	0	0	0	0	
ENSXETG00000032562	119	0	0	0	0	0	0	0	0	0	0	0	0	

ENSXETG00000032563	4641	2387	3516	3042	3419	2659	5885	4457	4800	2900	6709	4736	5152	picalm.2
ENSXETG00000032564	567	1	0	0	0	0	0	0	0	0	0	0	0	ENSXETG00000032564
ENSXETG00000032565	927	0	0	0	0	0	0	0	0	0	0	0	0	
ENSXETG00000032566	141	0	0	0	0	0	0	0	0	0	0	0	0	
ENSXETG00000032567	846	54	72	71	72	64	95	97	112	80	68	56	66	SIRT3
ENSXETG00000032568	630	16	24	23	27	15	21	381	391	231	48	26	44	ENSXETG00000032568
ENSXETG00000032569	951	0	0	0	0	0	0	0	0	0	0	0	0	
ENSXETG00000032570	984	20	35	19	37	31	65	273	384	245	50	25	37	ENSXETG00000032570
ENSXETG00000032571	447	0	0	0	2	0	1	1	2	3	0	0	0	ENSXETG00000032571
ENSXETG00000032572	70	0	0	0	0	0	0	0	0	0	0	0	0	
ENSXETG00000032573	954	0	0	0	0	0	0	0	0	0	0	0	0	
ENSXETG00000032574	537	0	0	0	2	1	0	6	1	3	4	7	6	ENSXETG00000032574
ENSXETG00000032575	924	0	0	0	0	0	0	0	0	0	0	0	0	
ENSXETG00000032576	1182	0	0	0	0	0	0	0	0	0	1	0	0	
ENSXETG00000032577	2792	916	1413	1267	1258	1041	3717	1119	1340	775	2587	1863	1994	spg20
ENSXETG00000032578	495	0	0	0	0	0	0	1	0	0	0	0	0	ENSXETG00000032578
ENSXETG00000032579	4293	1593	2409	2065	6317	4856	10664	6174	6477	4386	6764	4832	5308	pnpla6
ENSXETG00000032580	2190	443	702	619	967	764	909	1182	1343	802	711	487	523	XRCC5
ENSXETG00000032581	445	0	0	1	0	0	1	6	6	1	4	0	1	ENSXETG00000032581
ENSXETG00000032582	510	0	0	0	0	0	0	0	0	0	0	0	0	
ENSXETG00000032583	2304	0	0	0	0	0	0	0	0	0	0	0	0	
ENSXETG00000032584	1254	0	5	2	3	0	17	24	37	23	9	8	9	ENSXETG00000032584
ENSXETG00000032585	834	11	19	12	37	37	50	85	88	55	63	36	46	MLF1
ENSXETG00000032586	57	0	0	0	0	0	0	0	0	0	0	0	0	
ENSXETG00000032587	2053	68444	98492	82702	90605	69275	297929	20444	24484	14036	157998	108738	124573	SLC15A1
ENSXETG00000032588	870	0	0	0	0	0	1	0	0	1	2	1	1	ENSXETG00000032588
ENSXETG00000032589	591	15	13	11	4	4	5	28	37	18	10	6	5	ENSXETG00000032589
ENSXETG00000032590	653	56	107	118	102	88	201	237	258	149	188	152	146	ENSXETG00000032590
ENSXETG00000032591	606	0	0	0	0	0	0	0	0	0	0	0	0	
ENSXETG00000032592	1034	74	110	79	134	95	91	86	108	63	37	26	45	setmar
ENSXETG00000032593	930	0	0	0	0	0	0	0	0	0	0	0	0	
ENSXETG00000032594	2184	31	42	30	42	33	64	330	396	254	127	82	96	ENSXETG00000032594
ENSXETG00000032595	3126	454	685	607	2475	1942	5971	879	986	606	2159	1497	1746	ULK2
ENSXETG00000032596	1032	0	4	2	2	5	2	5	6	3	5	2	5	ppp1r1c
ENSXETG00000032597	942	0	0	0	0	0	0	0	0	0	0	0	0	
ENSXETG00000032598	6787	0	2	3	15	14	24	164	187	129	60	46	44	MAP1B
ENSXETG00000032599	744	79	116	89	251	170	457	123	150	90	629	459	545	METTL12
ENSXETG00000032600	2484	1	0	0	0	0	1	0	0	0	1	0	1	ENSXETG00000032600
ENSXETG00000032601	1320	15	24	16	131	97	126	341	392	227	146	104	91	ENSXETG00000032601
ENSXETG00000032602	764	2	0	0	0	1	0	0	0	0	0	0	0	
ENSXETG00000032603	1038	0	0	0	0	0	0	0	0	0	0	0	0	
ENSXETG00000032604	1703	4	2	8	14	10	2	21	27	12	12	3	4	lipc
ENSXETG00000032605	1015	1	0	1	0	3	3	7	2	1	7	3	4	ENSXETG00000032605
ENSXETG00000032606	930	0	0	0	0	0	0	0	0	0	0	0	0	
ENSXETG00000032607	441	9	18	23	31	15	25	16	24	11	33	24	20	ENSXETG00000032607
ENSXETG00000032608	939	13	19	7	130	82	117	36	47	29	78	39	55	ENSXETG00000032608
ENSXETG00000032609	924	0	0	0	0	0	0	0	0	0	0	0	0	
ENSXETG00000032610	387	2	4	1	0	0	1	6	3	4	1	0	0	ENSXETG00000032610
ENSXETG00000032611	918	0	0	0	2	0	1	4	3	0	5	2	2	GSG1
ENSXETG00000032612	4749	232	368	281	889	689	1414	827	939	532	1874	1359	1476	DEPDC5
ENSXETG00000032613	3588	0	2	0	6	3	0	116	159	63	26	17	26	ADAMTS19
ENSXETG00000032614	637	0	0	0	14	17	14	5	4	0	45	33	44	ENSXETG00000032614
ENSXETG00000032615	888	1	1	4	5	5	7	333	332	233	24	20	23	C1QTNF2
ENSXETG00000032616	5808	238	352	306	579	411	900	1142	1347	722	1456	1043	1059	TP53BP1
ENSXETG00000032617	528	0	0	0	0	0	0	0	0	0	0	0	0	
ENSXETG00000032618	70	0	0	0	0	0	0	0	0	0	0	0	0	
ENSXETG00000032619	116	0	0	0	0	0	0	0	0	0	0	0	0	
ENSXETG00000032620	1116	0	0	0	0	0	0	0	0	0	0	0	0	
ENSXETG00000032621	675	0	0	0	0	0	0	0	0	0	0	0	0	
ENSXETG00000032622	786	0	0	0	0	0	0	0	0	0	0	0	0	
ENSXETG00000032623	1392	0	0	1	2	2	2	1	5	3	3	1	3	HTR2A
ENSXETG00000032624	2576	0	0	0	0	0	0	0	0	0	0	0	0	
ENSXETG00000032625	1305	534	797	648	1624	1172	2411	89	120	55	741	502	612	PANK1
ENSXETG00000032626	6075	0	0	2	2	4	4	11	12	6	18	7	16	ENSXETG00000032626
ENSXETG00000032627	363	0	0	0	0	0	0	0	0	0	0	0	1	ENSXETG00000032627
ENSXETG00000032628	2634	7	14	2	5	10	13	115	164	78	49	35	51	FLT3
ENSXETG00000032629	119	0	0	0	0	0	0	0	0	0	0	0	0	
ENSXETG00000032630	408	9	7	7	6	5	20	66	58	35	13	4	9	ENSXETG00000032630
ENSXETG00000032631	1602	8	21	12	37	29	86	59	72	31	138	116	124	ENSXETG00000032631
ENSXETG00000032632	549	0	0	0	0	0	0	0	0	0	0	0	0	
ENSXETG00000032633	1971	185	288	245	300	253	398	732	864	484	426	331	354	ENSXETG00000032633
ENSXETG00000032634	1603	53	68	78	121	60	82	78	105	74	114	72	103	xkr9
ENSXETG00000032635	2349	2632	3750	3297	3156	2466	5192	976	1178	711	2741	1983	2166	ACAD11
ENSXETG00000032636	2735	0	0	0	0	0	0	0	0	0	0	0	0	
ENSXETG00000032637	2229	2629	3947	3389	5185	3869	8508	3397	3870	2344	5756	4002	4489	EPS8L2
ENSXETG00000032638	999	5	9	13	4	3	4	11	17	13	12	13	8	ENSXETG00000032638
ENSXETG00000032639	1689	3	4	7	7	9	3	2	4	2	3	1	1	ENSXETG00000032639
ENSXETG00000032640	1269	32	40	33	154	126	229	85	101	48	195	132	163	ENSXETG00000032640
ENSXETG00000032641	636	1	0	0	0	0	0	1	0	0	0	0	0	ENSXETG00000032641
ENSXETG00000032642	714	0	0	0	0	0	0	0	0	0	0	0	0	PDYN
ENSXETG00000032643	510	0	0	0	0	0	0	0	0	0	0	0	0	
ENSXETG00000032644	70	0	0	0	0	0	0	0	0	0	0	0	0	
ENSXETG00000032645	729	0	0	0	0	0	0	0	0	0	0	0	0	ENSXETG00000032645
ENSXETG00000032646	70	0	0	0	0	0	0	0	0	0	0	0	0	
ENSXETG00000032647	648	92	155	151	40	53	14	373	419	269	25	18	15	ENSXETG00000032647
ENSXETG00000032648	155	0	0	0	0	0	0	0	0	0	0	0	0	
ENSXETG00000032649	279	0	1	0	1	0	0	0	1	1	0	0	0	
ENSXETG00000032650	1782	0	0	0	1	0	0	114	147	85	0	0	0	ENSXETG00000032650
ENSXETG00000032651	513	0	0	0	0	0	0	0	0	0	0	0	0	
ENSXETG00000032652	1025	0	0	0	0	0	0	0	0	0	0	0	0	
ENSXETG00000032653	993	0	0	0	0	0	0	0	0	0	0	0	0	
ENSXETG00000032654	537	11	18	16	57	56	38	493	551	344	105	82	71	ENSXETG00000032654
ENSXETG00000032655	492	0	0	0	0	0	0	0	0	0	0	0	0	
ENSXETG00000032656	501	0	0	0	0	0	0	0	0	0	0	0	1	
ENSXETG00000032657	1143	0	0	0	0	0	0	0	0	0	0	0	1	
ENSXETG00000032658	921	0	0	0	0	0	0	0	0	0	0	0	0	ENSXETG00000032658
ENSXETG00000032659	2377	13096	18729	15689	17754	13923	13902	1061	1233	693	2661	1814	2182	ces3
ENSXETG00000032660	1734	243	395	341	883	640	946	709	759	444	886	628	695	SH3BP1
ENSXETG00000032661	114	0	0	0	0	0	0	0	0	0	0	0	0	
ENSXETG00000032662	1464	0	0	0	0	0	0	0	0	0	0	0	0	
ENSXETG00000032663	1070	85	127	103	113	99	201	238	282	167	229	137	166	ENSXETG00000032663
ENSXETG00000032664	70	0	0	0	0	0	0	0	0	0	0	0	0	
ENSXETG00000032665	312	0	0	0	0	0	0	0	0	0	0	0	0	hist1h4c
ENSXETG00000032666	2583	44	55	46	69	59	96	249	289	178	159	107	96	ENSXETG00000032666
ENSXETG00000032667	1188	41	65	58	148	119	58	70	91	67	158	128	145	ENSXETG00000032667
ENSXETG00000032668	933	0	0	0	0	0	0	0	0	0	0	0	0	
ENSXETG00000032669	1912	137	193	158	182	153	408	443	595	324	372	300	303	sp110
ENSXETG00000032670	847	3	6	1	3	2	4	8	12	8	6	4	5	ENSXETG00000032670
ENSXETG00000032671	930	0	0	0	0	0	0	0	0	0	0	0	0	
ENSXETG00000032672	191	0	0	0	0	0	0	0	0	0	0	0	0	
ENSXETG00000032673	1461	0	0	0	1	0	4	4	0	2	3	4	1	ENSXETG00000032673

ENSXETG00000032674	2211	0	0	0	0	0	0	0	0	0	0	0	0	
ENSXETG00000032675	2586	0	0	0	0	0	0	0	0	0	0	0	0	ENSXETG00000032675
ENSXETG00000032676	1185	0	0	0	0	0	0	0	0	0	0	0	0	
ENSXETG00000032677	1821	500	702	570	473	387	788	278	315	179	1193	888	927	ENSXETG00000032677
ENSXETG00000032678	5101	11	19	25	78	64	73	275	326	212	274	182	211	pitpnm2
ENSXETG00000032679	645	32	58	31	27	19	102	43	34	28	68	58	53	ENSXETG00000032679
ENSXETG00000032680	1818	72	93	76	147	124	313	369	412	228	433	302	295	CEP63
ENSXETG00000032681	2562	0	0	0	0	0	0	0	0	0	0	0	0	
ENSXETG00000032682	1545	0	0	0	0	0	0	1	0	0	0	0	0	
ENSXETG00000032683	1188	0	0	0	0	0	0	0	0	0	0	0	0	
ENSXETG00000032684	4894	51	86	72	169	157	448	383	419	256	422	336	365	ENSXETG00000032684
ENSXETG00000032685	528	0	0	0	0	0	0	0	0	0	0	0	0	
ENSXETG00000032686	312	0	1	0	0	0	0	0	0	0	0	0	0	
ENSXETG00000032687	1185	0	0	0	0	0	0	0	0	0	0	0	0	
ENSXETG00000032688	2106	20	32	37	78	57	108	163	187	121	168	119	129	ENSXETG00000032688
ENSXETG00000032689	1371	62	79	78	194	169	344	662	713	475	389	266	307	yap1
ENSXETG00000032690	561	12	22	18	54	48	112	113	121	79	61	52	58	TTC9
ENSXETG00000032691	2382	0	0	0	0	0	0	0	2	0	0	0	0	ENSXETG00000032691
ENSXETG00000032692	2366	389	582	423	507	369	1221	1053	1353	704	1155	903	960	ENSXETG00000032692
ENSXETG00000032693	663	1523	2173	1894	1872	1468	2500	820	970	537	1200	816	937	ENSXETG00000032693
ENSXETG00000032694	1506	7	11	3	7	8	12	5	9	3	8	1	3	ENSXETG00000032694
ENSXETG00000032695	966	0	0	0	0	0	0	0	0	0	0	0	0	
ENSXETG00000032696	891	0	0	0	0	0	0	0	0	0	0	0	0	
ENSXETG00000032697	2245	347	467	388	703	495	1010	1018	1158	687	810	551	587	stub1
ENSXETG00000032698	3315	8	13	11	59	47	36	1102	1167	774	337	230	248	AEBP1
ENSXETG00000032699	7554	118	208	170	7067	5419	3062	1003	1113	783	6887	5020	5460	EPG5
ENSXETG00000032700	164	0	0	0	0	0	0	0	0	0	0	0	0	
ENSXETG00000032701	999	0	0	0	1	2	6	1	1	0	8	2	3	ENSXETG00000032701
ENSXETG00000032702	909	0	0	0	0	0	0	0	0	0	0	0	0	
ENSXETG00000032703	164	0	0	0	0	0	0	0	0	0	0	0	0	
ENSXETG00000032704	1170	0	0	0	0	0	0	1	0	0	0	0	0	
ENSXETG00000032705	468	0	0	0	0	0	0	0	0	0	2	2	0	
ENSXETG00000032706	819	11	9	12	60	60	117	340	367	221	187	150	127	ENSXETG00000032706
ENSXETG00000032707	1905	0	0	0	1	2	1	0	1	0	0	1	1	ENSXETG00000032707
ENSXETG00000032708	381	0	0	0	0	0	0	0	0	0	0	0	0	
ENSXETG00000032709	119	0	0	0	0	0	0	0	0	0	0	0	0	
ENSXETG00000032710	1848	70	80	77	194	130	243	255	264	149	264	166	202	ENSXETG00000032710
ENSXETG00000032711	2944	153	237	204	320	230	530	662	803	509	434	342	354	tbc1d13
ENSXETG00000032712	468	0	0	0	0	0	0	0	0	0	0	0	0	
ENSXETG00000032713	1389	4	4	6	7	4	25	70	89	50	21	10	34	CHST3
ENSXETG00000032714	1038	3	3	5	8	8	8	112	152	82	13	12	9	fut7
ENSXETG00000032715	1079	47	102	65	105	62	790	24	23	16	116	90	91	ENSXETG00000032715
ENSXETG00000032716	654	1	0	0	3	1	3	21	33	13	20	15	13	METTL21C
ENSXETG00000032717	1428	0	0	0	0	0	0	3	3	2	0	0	1	
ENSXETG00000032718	458	2	1	0	2	0	5	71	70	57	4	1	5	ENSXETG00000032718
ENSXETG00000032719	1929	0	0	0	0	0	0	0	0	0	0	0	0	PRDM13
ENSXETG00000032720	780	0	0	0	0	0	0	0	0	0	0	0	0	
ENSXETG00000032721	2796	68	104	71	131	96	244	234	254	167	118	80	117	RECQL5
ENSXETG00000032722	2352	0	22	17	3	1	10	33	27	17	4	3	9	TAF4B
ENSXETG00000032723	402	0	0	0	0	0	0	0	0	0	0	0	0	GPHB5
ENSXETG00000032724	525	0	0	0	0	0	0	0	0	0	0	0	0	
ENSXETG00000032725	567	0	0	0	0	0	0	0	0	0	0	0	0	
ENSXETG00000032726	291	360	539	472	749	614	1631	442	510	328	1092	752	870	PRR15L
ENSXETG00000032727	119	0	0	0	0	0	0	0	0	0	0	0	0	
ENSXETG00000032728	2421	0	0	0	0	0	0	0	0	0	0	0	0	
ENSXETG00000032729	2155	713	1129	896	1283	1000	2085	1591	1821	1080	1915	1327	1520	arih1
ENSXETG00000032730	806	0	0	0	0	0	0	0	0	0	0	0	0	
ENSXETG00000032731	1221	0	0	0	0	0	0	0	0	0	0	0	0	
ENSXETG00000032732	109	0	0	0	0	0	0	0	0	0	0	0	0	
ENSXETG00000032733	2313	0	0	0	0	0	0	0	0	0	0	0	0	
ENSXETG00000032734	774	4	4	2	12	6	13	31	33	17	11	9	6	ENSXETG00000032734
ENSXETG00000032735	921	0	0	0	0	0	0	0	0	0	0	0	0	
ENSXETG00000032736	1497	9	8	5	14	10	18	69	134	66	28	29	26	FSD1
ENSXETG00000032737	570	0	1	0	11	12	0	3	1	0	0	1	0	ENSXETG00000032737
ENSXETG00000032738	828	0	0	0	0	0	0	2	0	0	0	0	0	ENSXETG00000032738
ENSXETG00000032739	1647	47	60	61	99	76	207	74	75	35	173	124	168	ENSXETG00000032739
ENSXETG00000032740	978	0	0	0	0	0	0	0	0	0	0	2	0	
ENSXETG00000032741	678	0	0	1	1	0	0	0	0	0	1	0	0	
ENSXETG00000032742	897	0	0	0	0	0	0	0	0	0	0	1	0	ENSXETG00000032742
ENSXETG00000032743	1275	7	12	11	11	17	26	178	184	109	37	25	27	ENSXETG00000032743
ENSXETG00000032744	141	0	0	0	0	0	0	0	0	0	0	0	0	
ENSXETG00000032745	1373	3	4	2	1	6	13	9	9	9	8	2	6	ENSXETG00000032745
ENSXETG00000032746	858	910	1302	1110	1597	1282	2977	4439	4573	2934	2103	1487	1654	MAFB
ENSXETG00000032747	1818	2	0	3	6	2	6	16	15	12	13	11	18	ENSXETG00000032747
ENSXETG00000032748	1518	179	234	198	198	172	337	264	351	195	195	130	130	ENSXETG00000032748
ENSXETG00000032749	381	0	0	0	0	0	0	0	0	0	2	0	0	ENSXETG00000032749
ENSXETG00000032750	474	0	2	1	31	26	59	365	417	256	85	66	59	ENSXETG00000032750
ENSXETG00000032751	1872	1086	1528	1280	1558	1257	4093	1787	2039	1212	1703	1175	1434	NCOA4
ENSXETG00000032752	84	1	3	1	1	2	0	0	2	0	3	2	0	SNORD103
ENSXETG00000032753	1278	0	0	0	0	0	0	0	0	0	0	0	0	ENSXETG00000032753
ENSXETG00000032754	312	0	0	0	0	0	0	0	0	0	1	0	0	
ENSXETG00000032755	1776	113	155	126	176	182	327	305	350	261	303	211	248	poll
ENSXETG00000032756	1299	3	1	3	40	41	5	41	55	27	63	36	52	ENSXETG00000032756
ENSXETG00000032757	936	0	0	0	0	0	0	0	0	0	0	0	0	
ENSXETG00000032758	1158	0	0	0	0	0	0	0	0	0	0	0	0	
ENSXETG00000032759	1698	481	694	664	711	565	1807	812	910	585	1185	805	928	tmco6
ENSXETG00000032760	119	0	0	0	0	0	0	0	0	0	0	0	0	
ENSXETG00000032761	1119	0	0	0	0	0	0	0	0	0	0	0	0	
ENSXETG00000032762	1071	0	0	0	0	0	0	0	0	0	0	0	0	
ENSXETG00000032763	82	0	0	0	0	0	0	0	0	0	0	0	0	xtr-mir-27c-2
ENSXETG00000032764	86	7	4	4	11	7	5	18	24	14	25	11	10	xtr-mir-142-2
ENSXETG00000032765	864	0	0	0	0	0	0	0	0	0	0	0	0	ENSXETG00000032765
ENSXETG00000032766	1099	6	3	3	5	3	21	54	66	35	15	11	11	ENSXETG00000032766
ENSXETG00000032767	666	0	0	0	2	1	0	5	5	3	2	0	0	ENSXETG00000032767
ENSXETG00000032768	2711	0	0	0	0	0	0	0	0	0	0	0	0	ENSXETG00000032768
ENSXETG00000032769	1206	704	1047	903	1031	798	1442	1198	1345	913	862	607	687	ENSXETG00000032769
ENSXETG00000032770	1581	8	18	13	92	56	232	501	557	342	142	89	117	ENSXETG00000032770
ENSXETG00000032771	357	7	5	13	12	6	6	21	14	15	22	9	15	ENSXETG00000032771
ENSXETG00000032772	70	0	0	0	0	0	0	0	0	0	0	0	0	
ENSXETG00000032773	119	0	0	0	0	0	0	0	0	0	0	0	0	
ENSXETG00000032774	96	0	0	0	0	0	0	0	0	0	0	0	0	
ENSXETG00000032775	1119	411	547	478	84	61	97	301	382	218	212	172	178	ENSXETG00000032775
ENSXETG00000032776	3394	7257	10173	8849	7759	5783	20955	1585	1919	1035	3867	2728	3094	dse
ENSXETG00000032777	119	0	0	0	0	0	0	0	0	0	0	0	0	
ENSXETG00000032778	1548	117	146	148	215	156	330	548	645	406	320	278	298	ENSXETG00000032778
ENSXETG00000032779	1764	1	1	3	0	1	0	1	1	0	0	1	3	ENSXETG00000032779
ENSXETG00000032780	1566	4	18	16	15	16	21	90	97	56	16	16	14	ENSXETG00000032780
ENSXETG00000032781	980	0	0	0	0	0	0	1	0	0	0	0	0	ENSXETG00000032781
ENSXETG00000032782	164	0	0	0	0	0	0	0	0	0	0	0	0	
ENSXETG00000032783	936	0	0	0	0	0	0	0	0	0	0	0	0	
ENSXETG00000032784	2385	0	0	0	0	0	0	0	0	0	0	0	0	

ENSXETG00000032785	1157	0	0	0	0	1	0	0	1	0	2	1	0	ENSXETG00000032785
ENSXETG00000032786	1149	0	0	0	0	0	0	0	0	0	0	0	0	
ENSXETG00000032787	516	330	465	356	488	336	623	648	740	417	531	333	415	FAM177A1
ENSXETG00000032788	675	283	427	337	313	249	225	232	275	184	250	157	177	ENSXETG00000032788
ENSXETG00000032789	510	0	0	0	0	0	0	0	0	0	0	0	0	
ENSXETG00000032790	2583	0	0	0	0	0	0	0	0	0	0	0	0	
ENSXETG00000032791	891	0	0	0	0	0	0	0	0	0	0	0	0	
ENSXETG00000032792	437	0	0	0	0	0	0	0	0	0	0	0	0	
ENSXETG00000032793	864	0	4	2	2	2	10	12	26	12	23	17	10	ENSXETG00000032793
ENSXETG00000032794	1188	0	0	0	2	2	0	0	0	2	0	1	0	
ENSXETG00000032795	804	0	0	0	0	0	0	1	1	0	0	1	0	
ENSXETG00000032796	2547	0	0	0	0	0	0	0	0	0	0	0	0	
ENSXETG00000032797	152	0	0	0	0	0	0	0	0	0	0	0	0	
ENSXETG00000032798	3096	713	1073	933	1196	837	1006	1636	1819	1061	868	624	724	SKIV2L2
ENSXETG00000032799	296	0	0	0	0	0	0	0	0	0	0	0	0	Metazoa_SRP
ENSXETG00000032800	930	5	6	9	9	5	2	9	22	4	10	10	10	ENSXETG00000032800
ENSXETG00000032801	1062	8	19	13	10	6	66	308	389	265	70	34	47	P2RY8
ENSXETG00000032802	1452	0	0	0	0	0	0	0	0	0	0	0	0	ANKRD34B
ENSXETG00000032803	150	0	0	0	0	0	0	0	0	0	0	0	0	U12
ENSXETG00000032804	465	0	0	0	0	0	0	1	0	0	0	0	1	
ENSXETG00000032805	1629	19	35	29	152	143	187	313	390	224	258	199	216	ENSXETG00000032805
ENSXETG00000032806	2325	0	0	0	0	0	0	0	0	0	0	0	0	
ENSXETG00000032807	1806	0	0	0	0	0	0	3	1	1	0	0	0	TBX15
ENSXETG00000032808	1324	2	1	2	0	0	2	0	0	0	0	1	0	ENSXETG00000032808
ENSXETG00000032809	12234	55	85	63	259	198	178	409	494	271	195	163	204	DYNC2H1
ENSXETG00000032810	453	0	0	0	1	0	0	1	0	0	0	0	0	
ENSXETG00000032811	119	0	0	0	0	0	0	0	0	0	0	0	0	
ENSXETG00000032812	681	91	146	121	275	154	349	65	68	36	70	71	60	ENSXETG00000032812
ENSXETG00000032813	1635	0	0	0	0	0	0	0	0	0	0	0	0	
ENSXETG00000032814	2519	5	7	7	80	68	16	41	34	16	12	14	10	clhc1
ENSXETG00000032815	4175	2035	2938	2406	3019	2245	3227	2853	3454	2016	1792	1304	1473	gpcpd1
ENSXETG00000032816	495	0	0	0	0	0	0	0	0	0	0	0	0	
ENSXETG00000032817	378	0	0	0	0	0	0	0	0	0	0	0	0	
ENSXETG00000032818	2362	0	0	0	0	0	0	0	0	0	0	0	0	
ENSXETG00000032819	4983	6	18	10	14	24	37	161	177	107	49	21	38	ENSXETG00000032819
ENSXETG00000032820	1362	0	1	0	1	2	2	0	2	0	2	1	3	ENSXETG00000032820
ENSXETG00000032821	1017	0	0	0	0	0	0	0	0	0	0	0	0	
ENSXETG00000032822	513	0	0	0	0	0	0	0	0	0	0	0	0	
ENSXETG00000032823	810	22	30	23	41	24	62	81	88	55	73	46	60	ENSXETG00000032823
ENSXETG00000032824	1025	29	54	48	54	29	49	75	123	74	40	33	22	G6PC3
ENSXETG00000032825	441	0	0	0	0	0	0	0	0	0	0	0	0	
ENSXETG00000032826	996	0	0	0	0	0	0	0	1	0	0	0	0	
ENSXETG00000032827	921	13	15	14	16	19	7	0	2	1	95	70	68	ENSXETG00000032827
ENSXETG00000032828	519	0	0	0	0	0	0	0	0	0	0	0	0	
ENSXETG00000032829	552	0	0	0	0	0	0	0	0	0	0	0	0	
ENSXETG00000032830	171	0	0	0	0	0	0	0	0	0	0	0	0	
ENSXETG00000032831	912	2475	3353	2708	1681	1294	2967	217	327	177	1152	831	1006	ENSXETG00000032831
ENSXETG00000032832	2526	0	0	0	0	0	0	0	0	0	0	0	0	
ENSXETG00000032833	2892	1	2	1	1	0	1	1	2	1	10	3	1	ENSXETG00000032833
ENSXETG00000032834	1206	0	0	0	0	0	0	0	1	0	1	0	0	
ENSXETG00000032835	1450	884	1312	1058	971	722	1084	841	954	588	895	641	723	ufsp2
ENSXETG00000032836	504	0	0	0	0	0	0	0	0	0	0	0	0	
ENSXETG00000032837	918	1101	1474	1180	1050	777	2432	655	888	518	840	627	716	ENSXETG00000032837
ENSXETG00000032838	780	93	172	131	206	181	394	231	307	181	247	165	182	ZFAND2B
ENSXETG00000032839	1401	427	602	568	737	617	875	778	877	503	928	643	754	RIOX1
ENSXETG00000032840	141	0	0	0	0	0	0	0	0	0	0	0	0	
ENSXETG00000032841	1737	3	5	8	58	36	144	287	324	196	93	42	57	ENSXETG00000032841
ENSXETG00000032842	933	0	0	0	0	0	0	0	0	0	0	0	0	
ENSXETG00000032843	7245	0	3	5	20	21	21	62	70	40	62	39	52	TENM1
ENSXETG00000032844	345	0	0	0	0	0	0	1	0	0	0	2	2	
ENSXETG00000032845	1824	10	13	13	32	22	37	33	64	29	28	22	20	GAS2L3
ENSXETG00000032846	119	0	0	0	0	0	0	0	0	0	0	0	0	
ENSXETG00000032847	1638	20	16	25	44	42	94	1204	1637	828	74	43	54	ENSXETG00000032847
ENSXETG00000032848	2817	5	7	3	9	6	12	88	104	52	39	36	41	ENSXETG00000032848
ENSXETG00000032849	942	0	0	0	0	0	0	0	0	0	0	0	0	
ENSXETG00000032850	3272	0	2	3	4	5	4	13	17	10	16	8	10	lrrn3
ENSXETG00000032851	1496	12	17	8	80	56	53	50	58	42	72	50	60	fam47e
ENSXETG00000032852	1206	0	0	0	0	0	0	0	0	1	0	0	0	
ENSXETG00000032853	383	0	0	0	0	0	0	0	1	0	0	0	0	hist1h4c
ENSXETG00000032854	1986	0	0	0	1	1	3	0	0	1	0	0	3	ENSXETG00000032854
ENSXETG00000032855	534	0	0	0	3	0	6	4	9	5	0	0	1	tmem196
ENSXETG00000032856	2987	97	133	113	216	145	401	247	314	159	372	230	306	ENSXETG00000032856
ENSXETG00000032857	486	0	1	0	1	2	11	53	64	39	24	20	19	PTN
ENSXETG00000032858	717	9	3	3	108	68	6	44	56	37	172	132	158	ENSXETG00000032858
ENSXETG00000032859	1038	5209	7742	6696	5587	4175	7596	1549	1653	979	3139	2368	2430	ENSXETG00000032859
ENSXETG00000032860	2331	0	0	0	0	0	0	0	0	0	0	0	0	
ENSXETG00000032861	1660	576	795	729	4089	3113	3947	1650	1944	1029	2551	1784	1999	cyp46a1
ENSXETG00000032862	735	7	6	6	13	13	18	54	60	40	40	34	24	TMEM150C
ENSXETG00000032863	3466	262	416	301	533	405	935	1590	1927	1062	861	544	667	trim8
ENSXETG00000032864	4778	297	433	379	1280	1008	1386	1461	1560	956	1571	1088	1229	ZC3H4
ENSXETG00000032865	2190	895	1233	1041	1213	927	1329	215	273	150	536	383	461	ENSXETG00000032865
ENSXETG00000032866	2170	42	61	53	74	54	134	152	154	97	140	109	109	ENSXETG00000032866
ENSXETG00000032867	930	0	0	0	0	0	0	0	0	0	0	0	0	
ENSXETG00000032868	845	0	0	0	0	0	0	0	0	1	0	0	0	
ENSXETG00000032869	4586	1209	1698	1512	2147	1679	1338	3041	3596	2066	2007	1363	1535	SF3B3
ENSXETG00000032870	996	0	1	0	0	0	0	0	0	0	0	2	0	ENSXETG00000032870
ENSXETG00000032871	1035	0	0	0	0	0	0	0	0	0	0	0	0	
ENSXETG00000032872	366	29	49	46	89	60	104	255	244	160	92	68	68	ENSXETG00000032872
ENSXETG00000032873	1627	3	5	6	8	7	17	155	202	120	26	17	17	ENSXETG00000032873
ENSXETG00000032874	843	0	0	0	0	0	0	0	1	0	0	0	0	
ENSXETG00000032875	798	0	0	0	1	0	0	2	4	6	2	1	2	ENSXETG00000032875
ENSXETG00000032876	1194	0	0	0	0	0	0	0	0	0	0	0	0	
ENSXETG00000032877	963	0	0	0	0	0	0	0	0	0	0	0	0	
ENSXETG00000032878	4759	187	304	261	699	493	1123	1161	1355	798	1097	788	836	TULP4
ENSXETG00000032879	303	36	53	44	94	85	152	1681	1944	1143	1418	1024	1111	ENSXETG00000032879
ENSXETG00000032880	2349	3	2	3	3	2	4	0	1	0	0	1	2	ENSXETG00000032880
ENSXETG00000032881	1172	125	166	162	3049	2335	134	5	0	3	2899	2071	2312	anxa10
ENSXETG00000032882	975	1	2	0	5	5	3	1	1	0	1	1	2	GPR61
ENSXETG00000032883	1146	0	0	0	1	0	1	0	0	0	0	0	0	
ENSXETG00000032884	119	0	0	0	0	0	0	0	0	0	0	0	0	
ENSXETG00000032885	864	0	0	0	0	0	0	0	0	0	0	0	0	
ENSXETG00000032886	1479	161	228	214	261	201	327	4	5	1	59	45	30	ENSXETG00000032886
ENSXETG00000032887	390	0	0	0	0	0	0	0	1	2	0	1	0	ENSXETG00000032887
ENSXETG00000032888	1036	0	0	0	0	0	0	0	0	0	0	0	0	
ENSXETG00000032889	1137	2	0	0	3	1	0	1	0	0	0	1	0	
ENSXETG00000032890	1566	64	96	81	218	179	259	235	238	158	327	205	215	ENSXETG00000032890
ENSXETG00000032891	4812	61	71	77	229	171	250	496	540	329	234	168	159	CC2D2A
ENSXETG00000032892	1328	17	30	17	31	26	26	175	174	120	55	45	37	awat1
ENSXETG00000032893	2694	0	5	1	2	3	2	32	30	14	4	1	5	ENSXETG00000032893
ENSXETG00000032894	510	9	13	12	21	31	32	24	28	17	38	16	19	ENSXETG00000032894
ENSXETG00000032895	960	7	11	6	4	7	13	22	19	7	0	1	2	ENSXETG00000032895

ENSXETG00000032896	1188	0	0	0	0	0	0	0	0	0	0	0	0	
ENSXETG00000032897	2535	0	0	0	0	0	0	0	0	0	0	0	0	
ENSXETG00000032898	6579	3405	4926	4079	8410	6314	13370	3317	3926	2261	5101	3632	4118	MYO7B
ENSXETG00000032899	1718	5	2	3	66	61	10	70	100	38	15	7	11	ENSXETG00000032899
ENSXETG00000032900	70	0	0	0	0	0	0	0	0	0	0	0	0	
ENSXETG00000032901	762	45	82	64	85	71	160	70	91	41	86	49	43	AASDHPPT
ENSXETG00000032902	540	611	924	787	393	304	518	638	837	475	567	373	377	mrpl42
ENSXETG00000032903	942	0	0	0	0	0	0	0	0	0	0	0	0	
ENSXETG00000032904	3059	802	1298	1041	8212	6152	6421	1627	1783	1080	6724	4627	5170	TRAK2
ENSXETG00000032905	396	0	0	0	0	0	0	3	2	1	1	0	2	ENSXETG00000032905
ENSXETG00000032906	1004	1979	2683	2311	2022	1610	1686	2226	2500	1540	1361	971	1070	smim15
ENSXETG00000032907	1632	0	0	0	3	1	1	1	4	2	0	1	0	ENSXETG00000032907
ENSXETG00000032908	6156	1	5	3	548	388	202	942	1113	658	481	377	419	ENSXETG00000032908
ENSXETG00000032909	953	0	0	0	17	24	8	2	3	2	190	129	176	ENSXETG00000032909
ENSXETG00000032910	945	0	0	0	0	0	0	0	0	0	0	0	0	
ENSXETG00000032911	1608	80	120	118	76	69	124	101	119	70	57	62	67	MGC89056
ENSXETG00000032912	846	156	220	207	315	252	540	23	26	8	549	375	420	ENSXETG00000032912
ENSXETG00000032913	888	1	0	0	0	0	0	0	0	1	0	0	0	
ENSXETG00000032914	531	0	0	0	0	0	0	0	0	0	0	0	0	
ENSXETG00000032915	2028	0	0	1	0	0	0	0	1	0	0	0	0	
ENSXETG00000032916	1781	248	435	314	395	292	804	563	730	405	605	433	489	ifngr1
ENSXETG00000032917	744	0	0	0	0	0	0	0	0	0	0	0	0	
ENSXETG00000032918	620	2	4	1	0	0	0	0	1	0	0	0	1	hamp
ENSXETG00000032919	2412	0	0	0	0	0	0	0	0	0	0	0	0	
ENSXETG00000032920	1329	0	0	0	0	0	0	0	0	0	0	0	0	
ENSXETG00000032921	384	1	0	0	0	0	0	0	0	0	0	0	0	ENSXETG00000032921
ENSXETG00000032922	2024	56	90	71	454	330	22	530	676	363	107	72	76	ENSXETG00000032922
ENSXETG00000032923	969	0	0	0	0	0	0	1	0	2	0	1	0	ENSXETG00000032923
ENSXETG00000032924	4121	605	828	658	1789	1432	2113	2456	2925	1753	2349	1602	1842	STAG2
ENSXETG00000032925	393	0	0	0	0	1	0	1	0	0	0	0	0	ENSXETG00000032925
ENSXETG00000032926	1737	51	55	64	48	32	92	116	104	89	116	85	102	ENSXETG00000032926
ENSXETG00000032927	978	0	0	0	0	0	0	0	0	0	0	0	0	
ENSXETG00000032928	504	0	0	0	0	0	0	0	0	0	0	0	0	
ENSXETG00000032929	166	0	0	0	0	0	0	0	0	0	0	0	0	
ENSXETG00000032930	1361	196	250	223	170	146	209	292	396	206	252	151	217	GTF3A
ENSXETG00000032931	1543	114	143	150	248	168	263	272	257	160	109	88	107	ENSXETG00000032931
ENSXETG00000032932	70	0	0	0	0	0	0	0	0	0	0	0	0	
ENSXETG00000032933	119	0	0	0	0	0	0	0	0	0	0	0	0	
ENSXETG00000032934	1325	0	0	0	0	1	5	14	22	9	3	3	3	TMEM72
ENSXETG00000032935	2664	0	0	0	0	0	0	0	0	0	1	0	0	
ENSXETG00000032936	1371	0	1	0	0	0	0	0	0	0	3	1	1	ENSXETG00000032936
ENSXETG00000032937	88	0	0	0	0	0	0	0	0	0	0	0	0	
ENSXETG00000032938	1227	1	0	0	4	2	6	39	45	24	7	3	4	SYT12
ENSXETG00000032939	1320	0	0	0	0	0	0	0	0	0	0	0	0	
ENSXETG00000032940	4515	169	240	206	431	346	367	510	684	367	456	328	342	esam
ENSXETG00000032941	2541	0	0	0	0	0	0	0	0	0	0	0	0	
ENSXETG00000032942	2277	28	55	47	195	141	234	519	601	365	256	212	218	AFAP1L1
ENSXETG00000032943	957	0	0	0	0	0	0	0	0	0	0	0	0	
ENSXETG00000032944	1239	42	64	65	89	55	620	63	92	49	59	44	46	ENSXETG00000032944
ENSXETG00000032945	504	0	0	0	0	0	0	0	0	0	0	0	0	
ENSXETG00000032946	70	0	0	0	0	0	0	0	0	0	0	0	0	
ENSXETG00000032947	119	0	0	0	0	0	0	0	0	0	0	0	0	
ENSXETG00000032948	783	5	2	2	0	0	0	0	0	0	0	1	0	CA3
ENSXETG00000032949	805	0	0	0	0	0	0	0	1	0	0	0	0	
ENSXETG00000032950	1323	53	73	84	32	18	66	96	128	89	87	48	38	MFSD11
ENSXETG00000032951	1626	13	15	14	120	78	179	371	410	223	278	172	204	ENSXETG00000032951
ENSXETG00000032952	510	0	0	0	0	0	0	0	0	0	0	0	0	ENSXETG00000032952
ENSXETG00000032953	855	474	641	530	444	343	398	532	657	345	315	225	256	triqk
ENSXETG00000032954	1311	0	0	0	6	3	13	42	74	50	18	11	8	NIM1K
ENSXETG00000032955	158	0	0	0	0	0	0	0	0	0	0	0	0	
ENSXETG00000032956	1518	213	349	279	435	348	384	318	362	222	365	278	283	cep44
ENSXETG00000032957	1014	0	0	0	0	0	0	0	0	0	0	0	0	
ENSXETG00000032958	885	0	0	0	0	0	0	0	0	0	0	0	0	
ENSXETG00000032959	1122	146	216	202	238	226	635	387	498	233	837	586	684	P2RX1
ENSXETG00000032960	927	0	0	0	0	0	0	0	0	0	0	0	0	
ENSXETG00000032961	2034	264	431	365	440	336	733	1378	1568	933	678	469	484	DYRK1B
ENSXETG00000032962	426	0	0	0	0	0	0	0	0	0	0	0	0	
ENSXETG00000032963	1566	17	14	15	10	5	15	79	107	57	37	26	37	ENSXETG00000032963
ENSXETG00000032964	2736	103	148	106	207	169	638	699	734	450	554	428	483	ENSXETG00000032964
ENSXETG00000032965	858	0	0	0	0	0	0	0	0	0	0	0	0	
ENSXETG00000032966	2535	0	0	0	0	0	0	0	0	0	0	0	0	
ENSXETG00000032967	585	0	0	0	0	0	0	0	0	0	0	0	0	
ENSXETG00000032968	1342	743	973	795	482	442	797	783	1008	522	625	449	493	PIGH
ENSXETG00000032969	795	0	0	0	0	0	0	0	2	0	0	0	0	
ENSXETG00000032970	3998	235	322	277	424	338	439	900	1055	596	420	291	310	ttc7b
ENSXETG00000032971	1148	310	477	427	394	292	707	180	215	112	468	362	374	SAYSD1
ENSXETG00000032972	1092	19	40	46	107	65	105	255	259	163	82	44	65	ENSXETG00000032972
ENSXETG00000032973	2644	4	3	7	126	111	30	36	47	38	40	27	35	IQCA1
ENSXETG00000032974	1050	0	0	0	0	0	0	0	0	0	0	0	0	
ENSXETG00000032975	1200	0	0	0	0	0	0	0	0	0	0	0	0	
ENSXETG00000032976	5679	396	562	506	1530	1239	2482	1332	1555	965	2499	1747	1913	ENSXETG00000032976
ENSXETG00000032977	519	0	0	0	0	0	0	0	0	0	0	0	0	
ENSXETG00000032978	1194	4499	6652	5590	7177	5413	11657	2028	2282	1379	5693	4154	4364	ENSXETG00000032978
ENSXETG00000032979	2148	3	4	7	19	14	19	42	46	32	31	36	32	CLIP4
ENSXETG00000032980	3528	102	143	141	260	162	189	395	450	262	340	214	272	zcchc2
ENSXETG00000032981	369	0	0	0	0	0	0	6	11	3	0	0	0	
ENSXETG00000032982	3357	0	0	0	0	1	1	4	9	4	15	12	11	MYBPC1
ENSXETG00000032983	573	2	0	1	9	3	8	29	38	31	33	22	27	ENSXETG00000032983
ENSXETG00000032984	669	53	77	61	53	42	77	191	252	115	623	492	506	ENSXETG00000032984
ENSXETG00000032985	1497	115	154	126	136	97	250	255	292	172	248	174	189	CDYL2
ENSXETG00000032986	972	9	22	11	1926	1544	85	4	5	4	49	29	40	ENSXETG00000032986
ENSXETG00000032987	1134	0	0	0	0	2	6	13	21	8	19	8	8	ENSXETG00000032987
ENSXETG00000032988	607	407	653	590	377	300	293	547	621	376	425	333	334	ENSXETG00000032988
ENSXETG00000032989	408	1	0	1	0	0	0	1	0	0	0	0	0	hist1h2bk
ENSXETG00000032990	214	0	0	0	0	0	0	0	0	0	0	0	0	
ENSXETG00000032991	1627	218	282	264	273	232	440	781	884	494	554	377	408	arl6ip4
ENSXETG00000032992	5249	304	429	402	2336	1805	2690	6116	6873	4372	1632	1199	1243	ENSXETG00000032992
ENSXETG00000032993	3747	20	31	34	162	119	232	931	1070	700	448	310	342	samd11
ENSXETG00000032994	5961	1775	2590	2329	8277	6135	5364	3994	4421	2621	7884	5440	6119	ENSXETG00000032994
ENSXETG00000032995	4491	41	77	57	161	154	349	124	140	78	520	342	391	ENSXETG00000032995
ENSXETG00000032996	474	0	0	0	0	0	0	0	0	0	0	0	0	
ENSXETG00000032997	1359	0	0	2	0	0	0	0	0	0	0	0	0	TFAP2D
ENSXETG00000032998	1341	0	0	0	0	0	1	0	0	0	0	0	0	
ENSXETG00000032999	3402	374	550	485	1077	924	1904	727	797	456	1182	906	1045	COBLL1
ENSXETG00000033000	1533	460	776	628	1330	953	2118	1002	1092	643	2121	1518	1554	sult1b1
ENSXETG00000033001	930	0	0	0	0	0	0	0	0	0	0	0	0	
ENSXETG00000033002	2441	0	0	0	0	0	0	0	0	0	0	0	0	
ENSXETG00000033003	837	6	1	7	20	10	24	54	60	43	18	14	19	ENSXETG00000033003
ENSXETG00000033004	957	0	0	0	0	0	0	0	0	0	0	0	0	
ENSXETG00000033005	1244	0	0	1	0	0	0	0	0	0	0	0	1	
ENSXETG00000033006	519	0	0	0	0	0	0	0	0	0	0	0	0	

ENSXETG00000033007	798	6	3	5	2	1	1	7	4	5	1	1	2	ENSXETG00000033007
ENSXETG00000033008	393	1	0	0	0	0	0	6	0	1	0	0	0	ENSXETG00000033008
ENSXETG00000033009	921	0	0	0	0	0	0	0	0	0	0	0	0	
ENSXETG00000033010	837	0	0	1	0	0	0	0	0	1	4	0	1	ENSXETG00000033010
ENSXETG00000033011	296	0	0	0	0	0	0	0	0	0	0	0	0	
ENSXETG00000033012	348	1	2	0	5	1	3	6	2	2	2	0	0	ENSXETG00000033012
ENSXETG00000033013	858	0	0	0	2	1	1	0	1	0	1	0	3	ENSXETG00000033013
ENSXETG00000033014	107	0	0	0	0	0	0	0	0	0	0	0	0	
ENSXETG00000033015	1131	0	1	0	0	0	0	0	0	0	0	0	0	
ENSXETG00000033016	381	2	1	0	0	2	1	3	2	1	2	1	1	ENSXETG00000033016
ENSXETG00000033017	1299	0	0	0	1	0	2	123	160	84	1	1	1	ENSXETG00000033017
ENSXETG00000033018	891	0	0	0	0	0	0	0	0	0	0	0	0	odam
ENSXETG00000033019	966	0	0	0	0	0	0	0	0	0	0	0	0	
ENSXETG00000033020	1551	9	9	6	4	3	10	4	6	4	12	10	5	ENSXETG00000033020
ENSXETG00000033021	2334	111	159	129	389	290	620	916	969	567	542	403	414	ENSXETG00000033021
ENSXETG00000033022	942	1	1	0	1	0	3	12	12	9	6	1	1	ENSXETG00000033022
ENSXETG00000033023	1221	2	5	0	4	2	7	202	277	141	19	18	26	ENSXETG00000033023
ENSXETG00000033024	927	0	0	0	0	0	0	0	0	0	0	0	0	
ENSXETG00000033025	690	1	0	0	0	0	2	1	3	2	0	0	0	CCDC172
ENSXETG00000033026	119	0	0	0	0	0	0	0	0	0	0	0	0	
ENSXETG00000033027	1092	20	17	13	32	24	44	30	48	28	36	33	27	ENSXETG00000033027
ENSXETG00000033028	70	0	0	0	0	0	0	0	0	0	0	0	0	
ENSXETG00000033029	10344	16	18	16	157	105	18	3	6	1	226	184	191	MYO15A
ENSXETG00000033030	1128	0	2	0	3	1	1	0	0	0	1	0	1	ENSXETG00000033030
ENSXETG00000033031	1848	0	0	0	0	0	0	16	15	12	0	0	0	COL10A1
ENSXETG00000033032	301	0	0	1	0	0	0	0	0	0	0	0	0	
ENSXETG00000033033	2535	3	4	3	122	93	6	50	61	35	4	4	3	ENSXETG00000033033
ENSXETG00000033034	119	0	0	0	0	0	0	0	0	0	0	0	0	
ENSXETG00000033035	612	0	0	0	0	0	0	0	0	0	0	0	0	
ENSXETG00000033036	2410	0	0	0	2	2	6	25	29	16	6	6	6	ENSXETG00000033036
ENSXETG00000033037	1746	428	585	533	226	209	200	8	5	11	1	1	4	ENSXETG00000033037
ENSXETG00000033038	107	0	0	0	0	0	0	0	0	0	0	0	0	
ENSXETG00000033039	1128	705	917	733	936	719	1405	108	120	70	553	391	441	ENSXETG00000033039
ENSXETG00000033040	1104	66	82	81	174	105	82	275	358	185	155	113	122	HAUS8
ENSXETG00000033041	2342	34	63	44	138	90	312	839	993	568	363	257	298	ENSXETG00000033041
ENSXETG00000033042	2265	0	0	0	0	0	0	0	0	0	0	0	0	
ENSXETG00000033043	2322	0	0	1	0	0	0	0	0	0	0	0	0	
ENSXETG00000033044	1895	17	27	19	26	21	94	662	836	449	146	102	116	gal3st4
ENSXETG00000033045	582	264	395	379	492	395	541	114	121	77	240	157	211	ENSXETG00000033045
ENSXETG00000033046	1569	1	2	0	0	0	1	2	1	0	2	0	1	ENSXETG00000033046
ENSXETG00000033047	1515	0	0	0	0	0	2	10	17	3	1	1	1	ENSXETG00000033047
ENSXETG00000033048	1425	0	0	0	0	0	0	0	0	0	0	0	0	
ENSXETG00000033049	2121	903	1257	1066	1322	991	1269	2283	2627	1609	1218	822	980	GMPS
ENSXETG00000033050	1866	0	1	0	196	188	89	28	42	21	2841	1908	2059	ela1
ENSXETG00000033051	528	0	0	0	0	0	0	0	0	0	0	0	0	
ENSXETG00000033052	2352	0	0	0	0	0	0	0	0	0	0	0	0	
ENSXETG00000033053	510	0	0	0	0	0	0	0	0	0	0	0	0	
ENSXETG00000033054	954	0	0	0	1	1	0	0	0	0	0	0	0	
ENSXETG00000033055	1140	28	33	27	56	46	90	82	118	59	88	61	82	ENSXETG00000033055
ENSXETG00000033056	1254	0	0	0	0	0	0	0	0	0	0	0	0	
ENSXETG00000033057	57	0	0	0	0	0	0	0	0	0	0	1	0	
ENSXETG00000033058	705	0	0	0	0	0	0	0	1	1	0	0	0	
ENSXETG00000033059	741	0	0	0	0	0	0	0	0	0	0	0	0	ENSXETG00000033059
ENSXETG00000033060	57	0	0	0	0	0	0	0	0	0	0	0	0	
ENSXETG00000033061	1896	0	0	0	0	0	0	3	3	2	2	2	0	CTXN3
ENSXETG00000033062	2427	0	0	0	0	0	0	0	0	0	0	0	0	
ENSXETG00000033063	513	13	14	20	2	3	4	6	9	3	19	14	11	ENSXETG00000033063
ENSXETG00000033064	57	0	0	0	0	0	0	0	0	0	0	0	0	
ENSXETG00000033065	771	48	74	79	112	91	235	2762	3384	1878	139	101	140	ENSXETG00000033065
ENSXETG00000033066	1140	0	0	0	0	0	0	0	0	0	0	0	0	OPN3
ENSXETG00000033067	214	0	0	0	0	0	0	0	0	0	0	0	0	
ENSXETG00000033068	2001	0	0	0	0	0	0	1	5	2	1	5	1	ABCG1
ENSXETG00000033069	2637	0	0	1	11	6	8	21	24	10	14	8	15	UNC5A
ENSXETG00000033070	2514	172	275	223	601	461	556	555	638	407	731	495	595	DLG3
ENSXETG00000033071	234	12	20	10	24	19	12	14	26	14	38	14	14	ENSXETG00000033071
ENSXETG00000033072	714	0	0	0	0	1	0	0	2	0	0	0	0	
ENSXETG00000033073	4374	58	94	89	259	211	539	649	756	474	648	482	499	ptpn21
ENSXETG00000033074	119	0	0	0	0	0	0	0	0	0	0	0	0	
ENSXETG00000033075	7848	5	4	6	1	0	0	0	4	2	0	0	0	ENSXETG00000033075
ENSXETG00000033076	2635	0	1	0	3	1	0	5	11	6	1	4	2	il1rapl2
ENSXETG00000033077	1695	0	0	0	107	78	6	0	0	0	39	29	28	ENSXETG00000033077
ENSXETG00000033078	1566	202	284	237	152	125	267	446	527	287	477	332	365	ENSXETG00000033078
ENSXETG00000033079	699	0	0	0	0	0	0	0	0	0	0	0	0	
ENSXETG00000033080	936	1	1	1	0	0	0	2	0	1	0	0	0	
ENSXETG00000033081	2164	131	239	181	310	264	406	583	771	402	324	208	210	ENSXETG00000033081
ENSXETG00000033082	2044	4	10	10	28	31	59	211	224	132	84	57	68	RFTN1
ENSXETG00000033083	2031	1	1	1	0	4	13	12	12	7	3	2	2	PANX2
ENSXETG00000033084	119	0	0	0	0	0	0	0	0	0	0	0	0	
ENSXETG00000033085	712	0	0	0	0	0	0	0	0	0	0	0	0	rln3
ENSXETG00000033086	2234	3	4	2	0	2	9	35	48	27	27	12	10	ENSXETG00000033086
ENSXETG00000033087	393	0	0	0	0	0	0	0	0	0	0	0	0	
ENSXETG00000033088	119	0	0	0	0	0	0	0	0	0	0	0	0	
ENSXETG00000033089	906	259	333	271	233	183	570	173	227	124	398	258	324	NDUFAF6
ENSXETG00000033090	1218	0	0	0	2	1	0	0	1	0	3	2	1	ENSXETG00000033090
ENSXETG00000033091	1536	435	519	517	290	215	290	252	293	163	85	50	77	SLC16A14
ENSXETG00000033092	945	0	0	0	0	0	0	0	0	0	0	0	0	
ENSXETG00000033093	1494	87	149	116	153	97	217	767	850	462	399	264	303	CREB3L2
ENSXETG00000033094	990	0	0	0	3	3	2	14	15	8	2	9	12	STK32C
ENSXETG00000033095	972	0	0	0	0	0	0	0	0	0	0	0	0	
ENSXETG00000033096	1818	0	0	0	0	0	2	6	7	4	1	4	3	CCDC177
ENSXETG00000033097	1197	0	0	0	0	0	0	0	0	0	0	0	0	
ENSXETG00000033098	1854	22	21	28	79	59	99	337	423	241	110	106	120	ZNF366
ENSXETG00000033099	3603	49	71	53	230	199	250	454	526	326	380	286	303	JAG2
ENSXETG00000033100	1087	41	61	52	12	13	13	18	24	19	16	8	7	ENSXETG00000033100
ENSXETG00000033101	729	174	243	192	225	205	259	255	274	194	290	206	232	ENSXETG00000033101
ENSXETG00000033102	1110	24	43	38	74	52	170	182	215	123	281	210	220	ENSXETG00000033102
ENSXETG00000033103	4744	442	656	522	1473	1113	2632	1743	2026	1198	2276	1512	1831	UPF2
ENSXETG00000033104	1188	0	0	0	0	0	0	0	0	0	0	0	0	ENSXETG00000033104
ENSXETG00000033105	588	0	0	0	2	1	1	3	9	4	5	7	4	ENSXETG00000033105
ENSXETG00000033106	678	0	0	0	1	1	9	0	0	0	8	3	2	ENSXETG00000033106
ENSXETG00000033107	930	0	0	0	0	0	0	0	0	0	0	0	0	
ENSXETG00000033108	1665	13	14	14	27	17	31	51	68	50	43	37	40	ENSXETG00000033108
ENSXETG00000033109	2349	1	2	1	3	7	4	31	17	11	6	1	3	ENSXETG00000033109
ENSXETG00000033110	516	0	0	0	0	0	0	0	0	0	0	0	0	ENSXETG00000033110
ENSXETG00000033111	495	2	3	4	36	15	13	27	25	20	5	1	5	ENSXETG00000033111
ENSXETG00000033112	804	1	0	1	0	3	2	7	10	9	7	4	6	ENSXETG00000033112
ENSXETG00000033113	1135	426	659	531	360	284	482	190	225	131	271	170	178	PEX26
ENSXETG00000033114	2487	0	0	0	0	0	0	1	0	0	0	0	0	
ENSXETG00000033115	2893	134	191	170	301	206	235	250	293	143	326	257	248	D2HGDH
ENSXETG00000033116	852	0	0	0	0	0	0	0	2	2	1	0	0	DOK6
ENSXETG00000033117	214	0	0	0	0	0	0	0	0	0	0	0	0	

ENSXETG00000033118	1419	0	0	0	0	0	0	0	0	0	0	0	0	
ENSXETG00000033119	1131	0	0	0	0	0	0	0	0	0	0	0	0	
ENSXETG00000033120	2481	10	16	9	73	58	53	14	20	18	115	76	97	ENSXETG00000033120
ENSXETG00000033121	3140	20	34	31	207	191	287	1175	1367	749	358	283	284	evc
ENSXETG00000033122	1620	24	18	26	163	129	429	351	421	248	299	185	205	ENSXETG00000033122
ENSXETG00000033123	2769	14	24	13	107	74	169	97	115	56	478	337	415	ENSXETG00000033123
ENSXETG00000033124	456	484	689	633	577	439	680	765	751	480	793	529	552	MRPL52
ENSXETG00000033125	1188	0	0	0	0	0	0	0	0	0	0	0	0	
ENSXETG00000033126	2183	138	223	195	362	268	281	521	619	378	580	366	418	acsl3
ENSXETG00000033127	480	404	661	551	406	305	430	596	712	392	373	259	309	ENSXETG00000033127
ENSXETG00000033128	1546	0	0	0	0	0	0	0	0	0	0	0	0	
ENSXETG00000033129	867	0	0	0	1	2	2	13	4	7	0	1	3	PAQR9
ENSXETG00000033130	1221	2815	4068	3538	3130	2302	4633	1601	1842	1123	2058	1382	1638	anks4b
ENSXETG00000033131	270	1	3	5	5	2	4	6	0	1	5	7	4	ENSXETG00000033131
ENSXETG00000033132	1709	0	0	0	6	7	7	194	286	149	58	29	49	ENSXETG00000033132
ENSXETG00000033133	958	238	327	290	373	259	396	760	930	551	456	341	406	MGC147242
ENSXETG00000033134	798	0	0	0	0	0	0	0	0	0	0	0	0	
ENSXETG00000033135	1002	0	0	1	0	0	1	0	0	0	0	0	0	ENSXETG00000033135
ENSXETG00000033136	507	3	1	4	3	2	0	9	5	1	1	1	0	ENSXETG00000033136
ENSXETG00000033137	2604	179	244	220	302	244	225	549	708	396	172	112	130	FANCB
ENSXETG00000033138	737	0	0	1	1	0	9	15	23	4	11	4	5	ENSXETG00000033138
ENSXETG00000033139	1986	4	6	7	38	23	123	263	286	155	205	175	187	ZBTB16
ENSXETG00000033140	1569	1613	2318	1988	2070	1495	1596	1529	1823	1064	939	689	739	CLPTM1
ENSXETG00000033141	1613	0	5	9	15	9	6	82	115	59	127	86	100	ENSXETG00000033141
ENSXETG00000033142	1523	6	3	7	8	9	29	204	261	121	16	6	17	ENSXETG00000033142
ENSXETG00000033143	834	0	0	0	1	1	0	0	1	0	0	1	0	ENSXETG00000033143
ENSXETG00000033144	1373	8	7	8	7	4	11	7	6	0	0	0	2	ENSXETG00000033144
ENSXETG00000033145	1956	5	8	3	13	11	91	410	454	300	30	17	28	ENSXETG00000033145
ENSXETG00000033146	119	0	0	0	0	0	0	0	0	0	0	0	0	
ENSXETG00000033147	119	0	0	0	0	0	0	0	0	0	0	0	0	
ENSXETG00000033148	1887	8721	12656	10853	11297	8698	31405	5767	6719	3980	14910	10353	11996	ENSXETG00000033148
ENSXETG00000033149	2295	0	0	0	0	0	0	0	0	0	0	0	0	
ENSXETG00000033150	1020	0	0	0	0	0	0	0	0	0	0	0	0	
ENSXETG00000033151	1611	0	0	0	0	0	0	0	0	0	0	0	0	
ENSXETG00000033152	363	44	60	58	69	64	61	216	257	137	39	29	38	ENSXETG00000033152
ENSXETG00000033153	657	0	0	0	0	0	0	0	0	0	0	0	0	ENSXETG00000033153
ENSXETG00000033154	115	0	0	0	0	0	0	0	0	0	0	0	0	
ENSXETG00000033155	750	914	1359	1178	886	689	1238	1346	1527	963	749	526	564	BOLA1
ENSXETG00000033156	1890	1	2	0	0	1	5	23	42	19	9	3	8	ENSXETG00000033156
ENSXETG00000033157	84	1	0	0	0	1	1	0	0	0	0	0	0	SNORD103
ENSXETG00000033158	828	0	0	0	0	0	0	0	0	0	0	0	0	
ENSXETG00000033159	381	0	0	0	0	0	0	0	0	0	0	0	0	
ENSXETG00000033160	2574	0	0	0	0	0	0	0	0	0	0	0	0	
ENSXETG00000033161	1350	3	0	1	1	2	1	2	2	0	3	0	0	ENSXETG00000033161
ENSXETG00000033162	783	0	0	0	0	0	0	0	0	0	0	0	0	ENSXETG00000033162
ENSXETG00000033163	1272	0	0	0	25	11	46	1	0	3	119	47	74	ENSXETG00000033163
ENSXETG00000033164	164	0	0	0	0	0	0	0	0	0	0	0	0	
ENSXETG00000033165	1179	0	2	1	1	1	1	1	3	1	4	3	2	ENSXETG00000033165
ENSXETG00000033166	1896	1	2	0	120	107	12	12	15	8	305	228	262	SLC9A4
ENSXETG00000033167	462	0	0	0	0	0	0	0	0	0	0	0	0	
ENSXETG00000033168	4350	0	1	0	13	8	5	8	5	6	15	9	9	FAM135B
ENSXETG00000033169	3975	778	1127	938	1672	1317	4203	3695	4571	2593	2252	1563	1829	WDFY1
ENSXETG00000033170	579	0	0	0	0	0	1	6	4	0	1	1	0	CCDC152
ENSXETG00000033171	1863	1	2	3	91	71	9	5	3	1	7	2	13	LRGUK
ENSXETG00000033172	909	625	864	713	5	0	65	92	79	61	15	11	11	ENSXETG00000033172
ENSXETG00000033173	660	9	15	11	86	72	65	324	353	224	314	226	220	ENSXETG00000033173
ENSXETG00000033174	1620	0	0	0	0	0	0	0	6	4	0	0	0	ENSXETG00000033174
ENSXETG00000033175	375	0	1	0	0	0	0	2	0	0	1	0	0	
ENSXETG00000033176	1149	1313	1966	1744	2621	2088	6850	8944	10039	6437	7701	5376	6218	ENSXETG00000033176
ENSXETG00000033177	453	0	0	0	0	0	1	1	3	1	0	0	0	
ENSXETG00000033178	3013	0	0	0	0	0	0	0	0	0	0	0	0	ENSXETG00000033178
ENSXETG00000033179	70	0	0	0	0	0	0	0	0	0	0	0	0	
ENSXETG00000033180	2142	8	16	10	54	54	151	378	463	234	77	58	57	SCARF1
ENSXETG00000033181	1428	102	121	98	129	97	277	141	178	109	126	86	110	ENSXETG00000033181
ENSXETG00000033182	966	15	16	18	67	56	162	908	1014	528	206	150	152	ENSXETG00000033182
ENSXETG00000033183	2274	0	1	0	4	3	1	5	3	5	11	8	8	CAPN14
ENSXETG00000033184	942	2	3	2	6	1	5	13	7	10	5	6	2	ENSXETG00000033184
ENSXETG00000033185	1166	159	244	214	507	382	577	790	782	524	700	480	534	sgf29
ENSXETG00000033186	1311	0	0	0	0	0	0	2	2	1	0	1	1	ENSXETG00000033186
ENSXETG00000033187	2260	109	187	176	351	279	585	853	959	538	465	356	421	tbc1d17
ENSXETG00000033188	119	0	0	0	0	0	0	0	0	0	0	0	0	
ENSXETG00000033189	1497	488	669	571	623	457	961	487	581	322	1552	1132	1124	SRMS
ENSXETG00000033190	1164	0	0	0	0	0	0	0	0	0	0	0	0	
ENSXETG00000033191	984	0	0	0	0	0	0	0	0	0	0	2	0	
ENSXETG00000033192	783	0	0	0	0	0	0	0	0	0	0	0	0	
ENSXETG00000033193	408	0	0	0	1	0	1	6	6	2	1	0	0	
ENSXETG00000033194	312	0	0	0	0	0	0	1	0	0	0	0	0	
ENSXETG00000033195	432	0	0	0	0	0	0	0	0	0	0	0	0	hist1h2ah
ENSXETG00000033196	729	20	34	31	23	18	15	39	52	29	13	2	10	ENSXETG00000033196
ENSXETG00000033197	2497	0	0	0	0	0	0	0	0	0	2	1	1	ENSXETG00000033197
ENSXETG00000033198	1272	49	84	68	78	60	69	105	114	63	126	105	90	ENSXETG00000033198
ENSXETG00000033199	726	0	0	0	0	0	0	0	0	0	0	0	0	ENSXETG00000033199
ENSXETG00000033200	738	0	0	0	3	0	0	3	6	3	5	3	3	ENSXETG00000033200
ENSXETG00000033201	3795	3	2	2	34	9	17	477	555	326	51	38	54	ENSXETG00000033201
ENSXETG00000033202	1060	24	42	31	24	17	55	209	255	165	78	66	62	ENSXETG00000033202
ENSXETG00000033203	192	0	0	0	0	0	0	0	0	0	0	0	0	
ENSXETG00000033204	2547	0	0	0	2	2	0	2	0	1	1	0	0	KCNH4
ENSXETG00000033205	1326	0	0	0	0	0	4	0	0	0	0	3	1	ENSXETG00000033205
ENSXETG00000033206	141	0	0	0	0	0	0	0	0	0	0	0	0	
ENSXETG00000033207	2550	12	30	28	105	85	146	125	131	89	123	81	79	ENSXETG00000033207
ENSXETG00000033208	1188	0	0	0	0	0	0	0	0	0	0	0	0	
ENSXETG00000033209	5277	0	0	0	1	0	0	1	1	0	1	0	1	LAMB4
ENSXETG00000033210	1832	1032	1425	1202	2118	1650	4751	3315	3794	2148	3672	2470	2848	lad1
ENSXETG00000033211	912	327	456	396	493	347	421	778	785	540	540	434	447	INO80E
ENSXETG00000033212	930	0	0	0	0	0	0	0	0	0	0	0	0	
ENSXETG00000033213	954	0	0	0	1	0	2	3	0	0	1	2	2	ENSXETG00000033213
ENSXETG00000033214	408	0	0	0	0	0	0	0	0	0	0	0	0	
ENSXETG00000033215	717	0	0	0	0	0	0	0	1	0	0	0	0	
ENSXETG00000033216	1035	143	226	146	348	265	324	514	641	341	380	240	301	ENSXETG00000033216
ENSXETG00000033217	3093	56	94	100	177	154	308	458	517	320	320	232	269	ENSXETG00000033217
ENSXETG00000033218	933	0	0	0	0	0	0	0	0	0	0	0	0	
ENSXETG00000033219	957	0	0	0	0	1	1	0	0	0	0	0	0	ENSXETG00000033219
ENSXETG00000033220	507	19	21	19	28	28	59	139	179	91	88	49	76	cdkn2c
ENSXETG00000033221	933	5	3	4	1	1	3	3	5	0	0	0	0	ENSXETG00000033221
ENSXETG00000033222	564	14	22	22	39	34	56	57	52	40	58	30	34	ENSXETG00000033222
ENSXETG00000033223	759	1	5	6	8	8	51	107	127	70	24	17	25	ENSXETG00000033223
ENSXETG00000033224	942	0	0	0	0	0	0	0	0	0	0	0	0	
ENSXETG00000033225	1026	5	6	12	15	8	31	8	5	2	20	13	14	ENSXETG00000033225
ENSXETG00000033226	3313	0	0	0	4	3	1	12	16	6	8	8	2	ak5
ENSXETG00000033227	366	42	55	55	158	127	141	196	191	138	204	126	152	ENSXETG00000033227
ENSXETG00000033228	1341	8	17	7	41	23	60	170	232	105	50	36	62	SPNS2

ENSXETG00000033229	939	0	0	0	0	0	0	0	0	0	0	0	0	
ENSXETG00000033230	786	0	0	0	0	0	0	0	0	0	0	0	0	
ENSXETG00000033231	1659	0	2	1	2	2	15	0	0	0	3	1	3	ENSXETG00000033231
ENSXETG00000033232	588	5	3	1	2	0	3	37	41	28	0	1	0	ENSXETG00000033232
ENSXETG00000033233	3426	0	3	2	6	6	3	57	54	40	4	8	6	ENSXETG00000033233
ENSXETG00000033234	318	0	0	0	0	0	0	0	0	0	0	0	0	
ENSXETG00000033235	1884	26	30	23	22	18	54	183	216	151	77	66	80	ITK
ENSXETG00000033236	937	1646	2430	1861	1022	732	1563	808	1020	549	529	417	444	ENSXETG00000033236
ENSXETG00000033237	2553	0	0	0	0	0	0	0	0	0	0	0	0	
ENSXETG00000033238	456	54	86	61	148	105	332	199	208	121	242	175	196	csdc2
ENSXETG00000033239	1980	2020	3000	2584	1822	1364	946	2343	2781	1607	738	540	561	TRAP1
ENSXETG00000033240	843	0	0	0	6	7	1	1	5	2	12	8	9	ENSXETG00000033240
ENSXETG00000033241	2126	92	174	132	601	426	326	267	339	176	287	186	192	ENSXETG00000033241
ENSXETG00000033242	3932	100	159	108	160	116	232	327	357	242	355	280	278	napb
ENSXETG00000033243	528	0	0	0	0	0	0	0	0	0	0	0	0	
ENSXETG00000033244	3367	198	267	211	1668	1329	1575	1031	1182	754	1654	1290	1286	XPC
ENSXETG00000033245	2757	19	24	20	223	195	190	1827	2003	1227	902	682	695	ENSXETG00000033245
ENSXETG00000033246	348	842	1188	989	1298	997	1803	5070	5682	3430	3847	2795	3014	B2M
ENSXETG00000033247	2598	0	0	0	0	0	0	0	0	0	0	0	0	ENSXETG00000033247
ENSXETG00000033248	630	0	0	0	0	0	0	0	0	0	0	0	1	
ENSXETG00000033249	1072	1	0	1	7	9	14	23	15	9	14	17	8	ENSXETG00000033249
ENSXETG00000033250	402	642	1003	853	346	249	352	590	644	416	256	208	208	MRPL54
ENSXETG00000033251	2569	318	450	369	641	509	1727	426	516	290	664	420	490	ENSXETG00000033251
ENSXETG00000033252	723	432	592	527	446	336	474	643	729	403	437	309	365	CCDC137
ENSXETG00000033253	119	0	0	0	0	0	0	0	0	0	0	0	0	
ENSXETG00000033254	3450	380	507	465	1033	835	1575	1137	1336	823	1574	1106	1163	TBCD
ENSXETG00000033255	477	0	0	0	0	1	0	1	2	1	0	1	1	RNF224
ENSXETG00000033256	1182	0	0	0	0	0	0	0	0	1	0	0	0	
ENSXETG00000033257	504	2	0	1	1	0	2	6	2	4	0	0	0	
ENSXETG00000033258	585	16	42	37	21	9	104	80	90	63	54	29	52	ENSXETG00000033258
ENSXETG00000033259	1320	41	56	46	87	50	131	143	177	87	168	106	122	ENSXETG00000033259
ENSXETG00000033260	1047	0	0	0	0	0	0	0	0	0	0	0	0	
ENSXETG00000033261	1539	0	0	0	0	0	0	0	0	0	0	0	0	
ENSXETG00000033262	2364	124	177	169	219	164	218	963	1083	628	466	341	407	ENSXETG00000033262
ENSXETG00000033263	840	0	0	2	2	3	0	11	19	6	14	6	18	ENSXETG00000033263
ENSXETG00000033264	3087	273	339	292	408	284	485	509	572	333	562	400	379	ENSXETG00000033264
ENSXETG00000033265	545	1	2	0	1	0	0	8	7	4	3	1	0	ENSXETG00000033265
ENSXETG00000033266	864	5	4	4	37	19	99	3634	3956	2411	306	211	229	ENSXETG00000033266
ENSXETG00000033267	639	0	1	0	0	0	2	0	0	0	2	5	0	ENSXETG00000033267
ENSXETG00000033268	1455	370	577	510	838	610	969	1474	1634	991	850	603	669	PAIP1
ENSXETG00000033269	1485	61	89	72	78	57	72	331	352	204	95	66	61	PCYOX1L
ENSXETG00000033270	1188	0	0	0	0	0	0	0	1	0	0	0	1	
ENSXETG00000033271	1056	26	25	24	35	15	48	96	75	67	30	39	38	ENSXETG00000033271
ENSXETG00000033272	588	271	344	304	336	250	315	123	132	79	317	220	194	ENSXETG00000033272
ENSXETG00000033273	2607	0	0	0	0	0	0	0	0	0	0	0	0	
ENSXETG00000033274	1170	11	9	5	305	205	20	0	0	0	2	1	1	MCIDAS
ENSXETG00000033275	1089	0	0	0	0	0	0	0	0	0	0	0	0	ENSXETG00000033275
ENSXETG00000033276	2703	151	237	211	238	176	167	218	248	144	533	442	433	ENSXETG00000033276
ENSXETG00000033277	1074	0	0	0	0	0	0	0	0	0	0	0	0	
ENSXETG00000033278	183	0	0	0	0	0	0	0	0	0	0	0	0	
ENSXETG00000033279	924	43	85	68	72	48	301	137	150	82	139	116	120	ENSXETG00000033279
ENSXETG00000033280	1956	0	0	3	5	4	2	11	10	10	7	7	13	ENSXETG00000033280
ENSXETG00000033281	1011	0	0	0	0	0	0	0	0	0	0	0	0	
ENSXETG00000033282	489	12	15	2	94	76	113	511	594	394	184	122	151	ENSXETG00000033282
ENSXETG00000033283	822	12	24	33	46	43	124	159	168	115	83	42	53	BTBD19
ENSXETG00000033284	2355	1	1	0	0	0	0	0	0	1	0	0	0	
ENSXETG00000033285	366	3	9	6	0	1	0	0	0	0	2	0	2	ENSXETG00000033285
ENSXETG00000033286	1498	271	377	330	467	376	885	863	963	579	647	499	506	ENSXETG00000033286
ENSXETG00000033287	1245	4998	7190	6078	2643	2089	3194	115	154	84	95	61	88	ENSXETG00000033287
ENSXETG00000033288	2370	0	1	0	0	0	0	0	0	0	0	0	0	
ENSXETG00000033289	1224	0	0	0	0	0	0	0	0	0	0	0	0	ENSXETG00000033289
ENSXETG00000033290	6102	0	0	0	0	0	0	0	0	0	0	0	0	
ENSXETG00000033291	552	0	4	0	2	0	3	0	0	0	3	0	0	
ENSXETG00000033292	1224	0	0	0	0	0	0	0	0	0	0	0	0	
ENSXETG00000033293	531	0	0	0	0	0	0	0	0	0	0	0	0	
ENSXETG00000033294	1327	187	286	288	268	199	263	416	465	285	190	140	158	PSTPIP2
ENSXETG00000033295	751	644	948	697	293	203	497	357	438	208	416	301	344	TMEM70
ENSXETG00000033296	405	0	0	0	0	0	0	0	0	0	0	0	0	ENSXETG00000033296
ENSXETG00000033297	267	0	0	0	3	4	10	1	0	2	6	9	4	ENSXETG00000033297
ENSXETG00000033298	4341	0	0	0	135	108	1	1	0	1	35	16	20	ENSXETG00000033298
ENSXETG00000033299	1242	0	0	0	0	0	0	0	0	0	0	0	0	
ENSXETG00000033300	1284	1	0	0	0	0	0	0	0	0	0	0	0	ENSXETG00000033300
ENSXETG00000033301	1596	0	0	0	1	0	0	4	6	1	0	0	0	ENSXETG00000033301
ENSXETG00000033302	5943	297	438	338	517	413	729	1226	1423	831	1735	1152	1355	ZFC3H1
ENSXETG00000033303	1188	0	0	0	0	0	0	0	0	0	1	0	1	
ENSXETG00000033304	927	547	826	649	608	439	1518	1019	1167	771	1291	853	953	ENSXETG00000033304
ENSXETG00000033305	381	0	0	1	2	3	0	5	9	9	3	0	3	ENSXETG00000033305
ENSXETG00000033306	2187	10	31	17	4	2	3	12	12	11	1	2	2	ENSXETG00000033306
ENSXETG00000033307	828	389	555	459	492	405	293	120	133	75	193	119	112	MGC147188
ENSXETG00000033308	804	2	3	0	19	17	3	0	2	2	3	1	1	ENSXETG00000033308
ENSXETG00000033309	2013	8	10	4	34	15	47	230	257	149	79	82	68	FAM189A2
ENSXETG00000033310	2319	32	32	25	73	63	91	386	476	279	30	30	31	fap
ENSXETG00000033311	660	1	0	0	0	0	0	0	0	0	0	0	0	
ENSXETG00000033312	1818	0	0	0	0	0	0	0	0	0	0	0	0	
ENSXETG00000033313	1848	0	0	0	0	1	3	0	0	0	3	3	0	ENSXETG00000033313
ENSXETG00000033314	2660	227	281	268	406	308	397	651	768	510	521	349	360	znf684
ENSXETG00000033315	970	26	43	35	4	4	9	0	2	3	10	11	15	ENSXETG00000033315
ENSXETG00000033316	825	0	0	0	0	0	0	0	0	0	0	0	0	
ENSXETG00000033317	1449	47	63	50	88	56	63	273	349	190	47	29	48	terf1
ENSXETG00000033318	2330	0	0	0	0	0	0	0	0	0	0	0	0	
ENSXETG00000033319	492	0	0	0	0	0	0	0	0	0	0	0	0	
ENSXETG00000033320	119	0	0	0	0	0	0	0	0	0	0	0	0	
ENSXETG00000033321	1047	535	762	603	633	448	540	807	978	564	95	58	75	ENSXETG00000033321
ENSXETG00000033322	1086	35	66	40	38	32	38	463	573	334	340	242	262	ENSXETG00000033322
ENSXETG00000033323	1215	0	0	0	14	11	8	48	54	27	22	14	12	ENSXETG00000033323
ENSXETG00000033324	714	0	0	0	0	0	1	0	0	0	0	0	1	ENSXETG00000033324
ENSXETG00000033325	2356	18	35	31	100	83	109	283	310	184	74	57	66	katnal2
ENSXETG00000033326	101	0	0	0	0	0	0	0	0	0	0	0	0	
ENSXETG00000033327	855	0	0	0	0	0	0	0	1	0	0	0	0	ENSXETG00000033327
ENSXETG00000033328	930	22	31	25	96	72	86	214	188	122	168	128	120	GPR4
ENSXETG00000033329	119	0	0	0	0	0	0	0	0	0	0	0	0	
ENSXETG00000033330	119	0	0	0	0	0	0	0	0	0	0	0	0	
ENSXETG00000033331	1149	0	0	0	0	0	1	1	1	0	0	0	0	ENSXETG00000033331
ENSXETG00000033332	1656	70	103	85	18	18	61	3	4	2	22	15	18	ENSXETG00000033332
ENSXETG00000033333	705	0	0	0	0	0	0	3	9	7	5	2	4	ENSXETG00000033333
ENSXETG00000033334	2324	3	4	0	14	10	41	44	53	22	22	9	10	il17d
ENSXETG00000033335	70	0	0	0	0	0	0	0	0	0	0	0	0	
ENSXETG00000033336	1461	0	0	0	0	0	0	0	0	0	0	0	0	
ENSXETG00000033337	534	1	4	4	3	0	3	17	26	7	3	5	3	ENSXETG00000033337
ENSXETG00000033338	810	0	1	3	0	0	2	1	2	1	8	2	3	ENSXETG00000033338
ENSXETG00000033339	1446	1	1	3	8	2	13	23	24	17	11	7	5	ENSXETG00000033339

ENSXETG00000033340	9405	741	1051	911	2281	1729	2734	2246	2668	1553	2758	1985	2211	GOLGB1
ENSXETG00000033341	1398	0	0	0	0	0	0	0	0	1	0	0	0	
ENSXETG00000033342	164	0	0	0	0	0	0	0	0	0	0	0	0	
ENSXETG00000033343	1618	1484	2104	1778	1016	694	1188	748	928	541	1392	926	1070	TFB2M
ENSXETG00000033344	903	0	0	0	0	0	0	0	0	0	0	0	0	
ENSXETG00000033345	2114	2164	3148	2752	3914	3020	4482	2077	2319	1456	1379	972	1048	oxct1
ENSXETG00000033346	990	0	0	0	0	0	0	0	0	0	0	0	0	
ENSXETG00000033347	2318	4	4	1	11	6	31	65	84	36	40	33	42	CPED1
ENSXETG00000033348	825	23	30	21	109	78	181	374	426	296	236	147	177	ENSXETG00000033348
ENSXETG00000033349	5921	868	1198	973	1377	1103	1391	2102	2438	1473	2313	1578	1920	MBNL2
ENSXETG00000033350	1599	160	228	179	320	269	390	648	773	428	444	331	371	hnrnpll
ENSXETG00000033351	1152	0	0	0	0	0	0	0	0	0	0	0	0	
ENSXETG00000033352	570	0	0	0	0	0	0	0	0	0	0	0	0	
ENSXETG00000033353	3321	3	1	1	3	9	14	16	23	13	20	13	10	TMC3
ENSXETG00000033354	1685	14	10	17	14	14	61	96	128	64	50	37	46	gpr132
ENSXETG00000033355	2255	204	289	247	847	670	698	1443	1721	1026	1377	967	1104	ENSXETG00000033355
ENSXETG00000033356	1356	128	172	146	238	206	220	667	653	415	425	304	316	CCM2
ENSXETG00000033357	2142	31	39	31	49	53	100	139	188	93	242	146	192	ENSXETG00000033357
ENSXETG00000033358	1122	0	0	0	0	0	0	0	0	0	0	0	0	
ENSXETG00000033359	867	0	0	0	0	0	0	0	0	0	0	0	0	
ENSXETG00000033360	990	0	0	0	0	1	0	1	5	7	18	9	12	GPR87
ENSXETG00000033361	119	0	0	0	0	0	0	0	0	0	0	0	0	
ENSXETG00000033362	840	80	117	100	127	85	144	525	579	353	184	123	161	cd40
ENSXETG00000033363	1116	0	1	0	0	1	1	1	2	0	4	4	2	
ENSXETG00000033364	1668	1	2	1	0	1	2	2	5	1	3	0	0	SLC17A8
ENSXETG00000033365	1630	1145	1711	1347	761	624	738	1548	1776	876	1106	787	935	RSL1D1
ENSXETG00000033366	609	58	62	50	128	106	138	212	224	138	265	199	214	ENSXETG00000033366
ENSXETG00000033367	4542	1816	2700	2210	2449	1956	3698	2784	3293	1912	2605	1816	2048	TMEM2
ENSXETG00000033368	510	10	13	14	22	9	22	45	62	46	31	25	26	ENSXETG00000033368
ENSXETG00000033369	657	0	0	0	0	0	0	0	0	0	0	0	0	ENSXETG00000033369
ENSXETG00000033370	804	57	85	86	235	193	1136	1036	1160	791	708	542	643	ENSXETG00000033370
ENSXETG00000033371	513	0	0	0	0	0	0	0	0	0	0	0	0	
ENSXETG00000033372	1602	7	6	3	83	46	116	208	259	138	154	143	129	ENSXETG00000033372
ENSXETG00000033373	1188	2	0	3	4	5	17	1	1	0	35	23	23	ENSXETG00000033373
ENSXETG00000033374	1061	19	36	27	31	15	27	120	133	87	39	25	39	MANSC4
ENSXETG00000033375	296	0	0	0	0	0	0	0	0	0	0	0	0	
ENSXETG00000033376	1326	619	919	746	446	337	36	72	96	55	622	426	513	B4GALNT2
ENSXETG00000033377	432	0	0	0	40	30	0	1	0	0	0	0	2	ENSXETG00000033377
ENSXETG00000033378	495	0	0	0	0	0	2	8	8	12	7	7	4	ENSXETG00000033378
ENSXETG00000033379	990	10	11	9	35	27	22	53	50	35	80	66	63	STEAP3
ENSXETG00000033380	2054	8492	12512	10455	3000	2315	2285	342	407	225	331	244	243	cyp2e1
ENSXETG00000033381	119	0	0	0	0	0	0	0	0	0	0	0	0	
ENSXETG00000033382	1572	14	31	30	117	76	164	749	784	523	248	161	188	ENSXETG00000033382
ENSXETG00000033383	486	10631	14880	12926	19927	15508	17901	9437	10813	6613	18248	12430	14119	ENSXETG00000033383
ENSXETG00000033384	57	0	0	0	0	0	0	0	0	0	0	0	0	
ENSXETG00000033385	930	0	0	0	0	0	0	0	0	0	0	0	0	
ENSXETG00000033386	2586	0	0	0	0	0	0	0	0	0	0	0	0	
ENSXETG00000033387	354	0	0	0	0	0	0	0	0	1	0	0	2	ENSXETG00000033387
ENSXETG00000033388	3348	169	243	215	482	337	491	679	773	483	637	454	461	ENSXETG00000033388
ENSXETG00000033389	708	0	0	0	0	0	0	0	0	0	0	0	0	
ENSXETG00000033390	2343	249	358	376	320	244	455	402	492	316	361	270	327	STRIP2
ENSXETG00000033391	119	0	0	0	0	0	0	0	0	0	0	0	0	
ENSXETG00000033392	1221	48	50	67	242	174	337	442	507	287	278	198	213	ENSXETG00000033392
ENSXETG00000033393	861	0	0	0	0	0	0	0	0	0	0	0	0	
ENSXETG00000033394	2457	0	0	0	0	0	0	0	0	0	0	0	0	
ENSXETG00000033395	1305	0	0	0	0	0	0	0	0	0	0	0	0	
ENSXETG00000033396	141	0	0	0	0	0	0	0	0	0	0	0	0	
ENSXETG00000033397	1296	0	0	0	2	2	2	6	14	4	4	0	3	ENSXETG00000033397
ENSXETG00000033398	2514	110	164	163	494	351	997	2621	2865	1956	1441	948	1097	BICD2
ENSXETG00000033399	894	0	0	0	0	0	0	0	1	0	0	0	0	ENSXETG00000033399
ENSXETG00000033400	2450	0	0	0	0	0	0	0	0	0	0	0	0	
ENSXETG00000033401	1467	25	57	49	48	59	78	170	256	116	138	105	135	ITPRIPL2
ENSXETG00000033402	510	0	0	0	0	0	0	0	0	0	0	0	0	
ENSXETG00000033403	1188	0	0	0	0	0	0	0	0	0	0	0	0	
ENSXETG00000033404	810	0	0	0	0	0	0	0	0	1	0	0	0	ENSXETG00000033404
ENSXETG00000033405	994	2510	3909	3329	3457	2573	1835	3547	4233	2381	891	636	651	cbr1
ENSXETG00000033406	1128	0	0	0	0	2	2	1	0	0	0	0	0	ENSXETG00000033406
ENSXETG00000033407	846	242	314	341	182	147	190	231	273	179	258	169	210	ENSXETG00000033407
ENSXETG00000033408	2457	0	0	0	0	0	0	0	0	0	0	0	0	
ENSXETG00000033409	3312	76	86	89	255	182	201	403	448	234	52	31	32	ENSXETG00000033409
ENSXETG00000033410	831	13	21	13	3	7	29	278	334	181	35	29	28	ENSXETG00000033410
ENSXETG00000033411	119	0	0	0	0	0	0	0	0	0	0	0	0	
ENSXETG00000033412	1350	0	0	0	1	0	1	0	0	0	0	1	1	
ENSXETG00000033413	57	0	0	0	0	0	0	0	0	0	0	0	0	U7
ENSXETG00000033414	119	0	0	0	0	0	0	0	0	0	0	0	0	
ENSXETG00000033415	6329	45	62	55	306	232	144	149	149	96	202	133	138	ENSXETG00000033415
ENSXETG00000033416	101	0	0	0	0	0	0	0	0	0	0	0	0	
ENSXETG00000033417	3750	64	76	83	1182	939	99	6	7	10	28	15	15	ENSXETG00000033417
ENSXETG00000033418	756	0	1	0	1	0	1	3	3	1	2	1	1	ENSXETG00000033418
ENSXETG00000033419	70	0	0	0	0	0	0	0	0	0	0	0	0	
ENSXETG00000033420	477	10	19	17	37	38	29	54	60	35	21	19	15	ENSXETG00000033420
ENSXETG00000033421	750	0	0	0	0	0	0	0	0	0	0	0	0	
ENSXETG00000033422	119	0	0	0	0	0	0	0	0	0	0	0	0	
ENSXETG00000033423	992	150	161	150	153	132	403	204	268	118	8806	6422	7017	ENSXETG00000033423
ENSXETG00000033424	633	12	20	14	38	41	41	48	57	22	48	43	38	TMEM74B
ENSXETG00000033425	296	116	132	100	40	32	337	105	115	89	167	19	33	Metazoa_SRP
ENSXETG00000033426	444	0	0	0	0	0	0	0	0	0	0	0	1	
ENSXETG00000033427	2478	38	46	50	206	124	378	522	587	364	513	366	444	APBA1
ENSXETG00000033428	4810	524	788	691	1866	1422	2925	5199	5954	3583	3728	2569	2901	ARHGEF1
ENSXETG00000033429	120	0	0	0	0	0	0	0	0	0	0	0	0	
ENSXETG00000033430	1945	28	37	31	245	169	137	468	462	272	637	471	537	ENSXETG00000033430
ENSXETG00000033431	615	0	0	0	0	0	0	0	0	0	0	0	0	
ENSXETG00000033432	435	0	0	0	0	0	0	0	0	5	0	0	0	
ENSXETG00000033433	756	0	0	0	0	0	0	6	4	4	0	0	0	ENSXETG00000033433
ENSXETG00000033434	3255	3	3	3	20	14	15	46	44	21	36	35	25	ENSXETG00000033434
ENSXETG00000033435	1305	0	0	0	0	0	0	0	0	0	0	0	0	
ENSXETG00000033436	489	67	112	100	70	54	83	80	105	82	67	40	53	ENSXETG00000033436
ENSXETG00000033437	641	157	217	198	62	32	14	6	7	4	16	15	20	ENSXETG00000033437
ENSXETG00000033438	115	0	0	0	0	0	0	0	0	0	0	0	0	
ENSXETG00000033439	1341	42	53	60	76	85	166	543	601	407	215	162	168	ENSXETG00000033439
ENSXETG00000033440	2472	17	33	35	189	147	395	492	539	303	385	251	357	C7
ENSXETG00000033441	1371	95	125	134	323	288	617	489	561	298	618	411	493	ENSXETG00000033441
ENSXETG00000033442	744	2	2	3	18	16	33	73	76	56	40	33	31	ENSXETG00000033442
ENSXETG00000033443	164	0	0	0	0	0	0	0	0	0	0	0	0	
ENSXETG00000033444	3621	20	21	16	16	12	35	55	32	26	22	16	18	TRIM66
ENSXETG00000033445	660	0	0	0	0	0	0	0	0	0	0	0	0	ENSXETG00000033445
ENSXETG00000033446	2301	15	35	28	48	39	80	122	138	64	85	55	70	ENSXETG00000033446
ENSXETG00000033447	510	0	0	0	0	0	0	0	0	0	0	0	0	
ENSXETG00000033448	1950	297	378	306	239	197	326	271	352	205	234	153	156	c14orf79
ENSXETG00000033449	2646	0	0	0	0	0	0	0	0	0	0	0	1	
ENSXETG00000033450	2637	0	0	0	0	0	0	0	0	0	0	0	0	

ENSXETG00000033451	849	18	44	34	16	8	30	72	110	57	57	54	42	atp6v1g2
ENSXETG00000033452	933	0	0	0	0	0	0	0	0	0	0	0	0	
ENSXETG00000033453	158	0	0	0	0	0	0	0	0	0	0	0	0	
ENSXETG00000033454	1026	0	0	0	0	0	0	0	0	0	0	0	0	
ENSXETG00000033455	927	323	479	388	331	220	114	38	59	33	3	2	1	ENSXETG00000033455
ENSXETG00000033456	141	0	0	0	0	0	0	0	0	0	0	0	0	
ENSXETG00000033457	765	0	0	0	0	0	0	0	0	0	0	0	0	
ENSXETG00000033458	975	0	0	0	0	0	0	0	0	0	0	0	0	
ENSXETG00000033459	2810	100	215	175	234	197	489	451	531	286	363	278	314	znf554
ENSXETG00000033460	818	14	10	12	0	1	0	18	26	11	1	0	2	ENSXETG00000033460
ENSXETG00000033461	3724	0	0	0	0	0	0	0	0	0	0	0	0	
ENSXETG00000033462	57	0	0	0	0	0	0	0	0	0	0	0	0	
ENSXETG00000033463	92	0	8	2	1	2	8	1	10	2	19	5	3	ENSXETG00000033463
ENSXETG00000033464	214	0	0	0	0	0	0	0	0	0	0	0	0	
ENSXETG00000033465	5336	90	107	106	195	151	241	439	520	304	291	205	236	ENSXETG00000033465
ENSXETG00000033466	1524	4724	7134	6166	6810	5367	2263	37	45	22	1873	1312	1474	ENSXETG00000033466
ENSXETG00000033467	1701	358	556	470	1004	771	1195	605	653	411	702	548	560	SLCO2B1
ENSXETG00000033468	312	0	0	0	0	0	0	0	0	0	0	0	0	
ENSXETG00000033469	729	0	0	0	0	0	0	0	0	0	0	0	0	ENSXETG00000033469
ENSXETG00000033470	927	0	0	0	0	0	0	0	0	0	0	0	0	
ENSXETG00000033471	2367	0	0	0	0	0	0	0	0	0	0	0	0	
ENSXETG00000033472	4585	0	0	0	0	0	0	0	0	0	0	0	0	
ENSXETG00000033473	1086	0	0	0	0	0	0	0	0	0	0	0	0	
ENSXETG00000033474	957	109	163	120	74	63	140	147	179	109	225	145	178	ngrn
ENSXETG00000033475	528	0	0	0	0	0	0	0	1	1	0	0	0	
ENSXETG00000033476	957	0	0	0	0	0	0	0	0	0	0	0	0	
ENSXETG00000033477	924	0	0	0	0	0	0	0	0	0	0	0	0	
ENSXETG00000033478	70	0	0	0	0	0	0	0	0	0	0	0	0	
ENSXETG00000033479	870	0	0	0	0	0	0	0	0	0	0	0	0	
ENSXETG00000033480	1080	10	3	3	12	5	22	11	20	20	19	12	10	ENSXETG00000033480
ENSXETG00000033481	771	1	0	0	1	0	1	3	6	1	2	1	1	ENSXETG00000033481
ENSXETG00000033482	2739	85	100	94	177	117	131	349	439	233	305	233	242	lemd1
ENSXETG00000033483	492	0	0	0	0	0	0	0	0	0	0	0	0	
ENSXETG00000033484	510	0	0	0	0	1	0	0	1	0	2	3	4	ENSXETG00000033484
ENSXETG00000033485	1074	367	526	440	268	191	446	463	433	296	401	269	309	eef1akmt1
ENSXETG00000033486	2875	240	371	329	392	271	396	626	662	435	743	484	554	znf526
ENSXETG00000033487	897	0	0	1	10	8	17	12	11	6	17	9	15	ENSXETG00000033487
ENSXETG00000033488	510	0	0	0	0	0	0	0	0	0	0	0	0	
ENSXETG00000033489	576	1	2	1	0	0	0	0	0	0	0	0	0	
ENSXETG00000033490	4683	28	43	30	376	271	211	661	842	451	695	564	635	ENSXETG00000033490
ENSXETG00000033491	103	0	0	0	0	0	0	0	0	0	0	0	0	ENSXETG00000033491
ENSXETG00000033492	3639	300	414	321	542	471	890	1491	1646	1058	1113	783	879	ZMYND8
ENSXETG00000033493	1263	344	426	396	303	230	414	759	931	550	275	197	208	synj2bp
ENSXETG00000033494	1083	12	14	19	6	4	11	99	124	71	72	48	58	ENSXETG00000033494
ENSXETG00000033495	585	1	1	0	0	0	0	0	0	0	1	0	0	ENSXETG00000033495
ENSXETG00000033496	4953	4	7	6	14	4	18	24	33	19	13	6	21	SIPA1L2
ENSXETG00000033497	555	199	300	247	264	178	296	577	676	372	311	267	300	CCDC28A
ENSXETG00000033498	107	0	0	0	0	0	0	0	0	0	0	0	0	
ENSXETG00000033499	1314	0	0	0	0	0	0	0	0	0	0	0	0	
ENSXETG00000033500	876	12	13	14	6	7	9	38	44	29	1	1	0	ENSXETG00000033500
ENSXETG00000033501	1926	111	188	137	99	42	60	60	84	43	57	40	39	ENSXETG00000033501
ENSXETG00000033502	1645	0	2	0	1	4	3	57	53	25	10	9	10	MGC108497
ENSXETG00000033503	783	0	0	0	0	0	0	2	1	2	0	0	0	
ENSXETG00000033504	1011	1	3	3	0	1	1	7	7	3	0	0	3	ENSXETG00000033504
ENSXETG00000033505	1491	242	363	317	666	493	1180	774	1039	608	613	417	491	ENSXETG00000033505
ENSXETG00000033506	675	1	9	2	6	4	36	64	69	27	9	7	2	avd
ENSXETG00000033507	546	0	0	0	0	0	0	1	2	0	1	1	1	ENSXETG00000033507
ENSXETG00000033508	909	10	14	12	33	26	22	36	49	22	27	19	33	ENSXETG00000033508
ENSXETG00000033509	1233	0	0	1	2	3	1	2	6	5	0	0	1	st8sia5
ENSXETG00000033510	411	0	0	0	0	0	0	0	0	2	1	0	0	ENSXETG00000033510
ENSXETG00000033511	915	1	0	0	0	0	0	0	0	0	0	0	0	
ENSXETG00000033512	342	0	0	0	0	0	0	0	0	0	0	0	0	ENSXETG00000033512
ENSXETG00000033513	840	0	0	0	0	0	0	0	0	0	0	0	0	
ENSXETG00000033514	987	0	0	2	0	2	2	1	1	1	13	5	8	ENSXETG00000033514
ENSXETG00000033515	117	140	183	149	201	143	190	252	251	167	306	236	201	ENSXETG00000033515
ENSXETG00000033516	1376	12	12	16	36	34	11	23	30	23	39	17	28	SLC38A8
ENSXETG00000033517	1389	79	136	99	87	55	146	98	110	60	87	71	62	DEAF1
ENSXETG00000033518	1848	318	465	381	729	538	897	1071	1305	743	786	563	594	DDX41
ENSXETG00000033519	917	219	255	237	156	134	247	264	298	187	296	189	236	lyrm9
ENSXETG00000033520	880	1033	1461	1171	800	591	796	659	815	499	409	293	329	tmem208
ENSXETG00000033521	115	0	0	0	0	0	0	0	0	0	0	0	0	
ENSXETG00000033522	1032	677	1065	911	676	451	936	209	268	133	254	195	207	acbd4
ENSXETG00000033523	1084	19648	29345	24340	10871	8270	18844	5634	6673	3815	11800	8821	9184	ENSXETG00000033523
ENSXETG00000033524	510	0	0	0	0	0	0	0	0	0	0	0	0	
ENSXETG00000033525	798	0	0	0	0	0	0	0	3	0	0	0	0	
ENSXETG00000033526	735	0	0	0	0	0	1	0	0	0	0	0	0	ENSXETG00000033526
ENSXETG00000033527	390	0	0	0	0	0	0	0	0	0	0	0	0	ENSXETG00000033527
ENSXETG00000033528	405	0	0	0	0	2	0	2	4	0	2	1	3	ENSXETG00000033528
ENSXETG00000033529	465	0	0	0	0	0	0	0	0	0	0	0	0	
ENSXETG00000033530	164	0	0	0	0	0	0	0	0	0	0	0	0	
ENSXETG00000033531	339	8	18	5	46	50	102	63	66	44	76	69	78	ENSXETG00000033531
ENSXETG00000033532	495	0	0	0	0	0	0	0	0	0	0	0	0	
ENSXETG00000033533	2097	46	71	63	216	153	297	454	504	315	292	205	247	ATXN1L
ENSXETG00000033534	1303	0	2	1	0	0	9	53	65	39	8	9	8	ENSXETG00000033534
ENSXETG00000033535	1536	522	757	682	1649	1346	737	2014	2208	1375	829	621	631	G6PD
ENSXETG00000033536	1830	0	0	0	0	0	0	0	0	0	0	0	0	
ENSXETG00000033537	1299	0	0	0	0	0	0	0	0	0	0	0	0	
ENSXETG00000033538	933	0	0	0	0	0	0	0	0	0	0	0	0	
ENSXETG00000033539	381	0	0	0	0	0	0	0	0	0	0	0	0	ENSXETG00000033539
ENSXETG00000033540	516	1	5	4	31	15	15	12	8	13	219	131	166	sycn
ENSXETG00000033541	3624	164	215	171	290	228	207	436	505	264	196	131	156	RBM12
ENSXETG00000033542	2157	724	1022	900	1058	803	1765	1931	2229	1342	1003	697	861	FOLH1
ENSXETG00000033543	708	0	0	0	0	0	0	0	0	0	0	0	0	
ENSXETG00000033544	214	0	0	0	0	0	0	0	0	0	0	0	0	
ENSXETG00000033545	2321	5	4	2	4	5	8	1	3	2	0	2	1	ENSXETG00000033545
ENSXETG00000033546	930	0	0	1	0	0	0	4	16	5	0	0	0	gtsf1
ENSXETG00000033547	2274	100	156	134	465	348	1078	817	923	517	893	636	699	ENSXETG00000033547
ENSXETG00000033548	510	0	0	0	0	0	0	0	0	0	0	0	0	
ENSXETG00000033549	1173	0	0	0	0	1	1	0	0	0	0	0	0	
ENSXETG00000033550	107	0	0	0	0	0	0	1	0	0	0	0	0	U6
ENSXETG00000033551	480	0	0	0	0	0	0	0	0	0	0	1	0	
ENSXETG00000033552	513	0	0	0	0	0	0	0	0	0	0	0	0	
ENSXETG00000033553	567	0	0	0	0	0	0	0	0	0	0	0	0	
ENSXETG00000033554	1580	0	0	0	0	0	0	1	0	0	2	1	0	ENSXETG00000033554
ENSXETG00000033555	882	0	0	0	0	0	0	0	0	0	0	0	0	
ENSXETG00000033556	114	0	0	0	0	0	0	0	0	0	0	0	0	
ENSXETG00000033557	1188	2	1	1	1	2	0	4	4	2	3	4	2	ENSXETG00000033557
ENSXETG00000033558	960	0	1	0	0	1	0	0	0	0	23	21	14	ENSXETG00000033558
ENSXETG00000033559	528	0	0	0	0	0	0	0	0	0	0	0	0	
ENSXETG00000033560	1851	77	121	81	206	157	277	561	607	346	347	222	262	ENSXETG00000033560
ENSXETG00000033561	2361	0	0	0	0	0	0	0	1	2	0	0	0	

ENSXETG00000033562	936	0	0	0	0	0	0	0	0	0	0	0	0	
ENSXETG00000033563	4380	0	1	0	49	25	1	28	19	11	138	85	105	COL17A1
ENSXETG00000033564	457	0	0	0	0	0	0	0	0	0	0	0	0	il9
ENSXETG00000033565	711	0	0	0	0	0	0	0	0	0	0	0	1	
ENSXETG00000033566	567	0	0	0	0	0	0	0	0	0	0	0	0	ENSXETG00000033566
ENSXETG00000033567	2358	7	18	10	30	12	53	7	14	11	24	17	27	GPAT2
ENSXETG00000033568	119	0	0	0	0	0	0	0	0	0	0	0	0	
ENSXETG00000033569	1542	39	44	47	119	90	143	80	97	48	221	148	175	ENSXETG00000033569
ENSXETG00000033570	468	0	0	0	0	0	0	0	1	0	0	0	0	ENSXETG00000033570
ENSXETG00000033571	315	0	5	0	2	1	4	0	0	2	0	0	1	PROK1
ENSXETG00000033572	977	45	51	50	75	49	95	859	1049	609	161	91	126	cd28
ENSXETG00000033573	480	239	300	318	1363	956	1190	739	920	501	2438	1723	1881	ENSXETG00000033573
ENSXETG00000033574	576	367	550	475	237	195	224	375	407	247	375	294	293	TSR2
ENSXETG00000033575	119	0	0	0	0	0	0	0	0	0	0	0	0	
ENSXETG00000033576	6288	4	3	1	10	2	5	0	7	0	12	4	4	ENSXETG00000033576
ENSXETG00000033577	1896	56	65	97	454	388	93	489	528	316	418	253	314	ENSXETG00000033577
ENSXETG00000033578	774	0	0	0	0	1	0	4	3	0	1	2	0	TNMD
ENSXETG00000033579	2094	0	0	1	3	6	14	20	9	16	28	17	20	amer2
ENSXETG00000033580	3575	27	20	21	6	14	7	27	40	26	4	5	9	ENSXETG00000033580
ENSXETG00000033581	612	12	21	24	18	16	29	37	41	25	55	41	30	ENSXETG00000033581
ENSXETG00000033582	969	0	0	0	0	0	0	0	0	0	0	0	0	
ENSXETG00000033583	594	2	5	1	1	2	0	1	4	5	1	0	1	ENSXETG00000033583
ENSXETG00000033584	381	0	0	0	0	0	0	0	0	0	0	0	0	ENSXETG00000033584
ENSXETG00000033585	441	0	0	0	0	0	0	0	0	0	0	0	0	tdgf1.1
ENSXETG00000033586	864	4	7	7	11	13	52	122	156	83	53	34	52	ENSXETG00000033586
ENSXETG00000033587	1268	1602	2341	2081	2360	1696	1344	436	516	304	607	476	501	fdft1
ENSXETG00000033588	723	21	30	21	4	5	0	4	2	1	0	0	0	ENSXETG00000033588
ENSXETG00000033589	888	0	0	0	0	0	0	0	1	0	0	0	0	
ENSXETG00000033590	3851	78	106	84	289	172	588	673	809	492	567	397	452	ENSXETG00000033590
ENSXETG00000033591	66	1	1	1	0	0	0	2	1	1	0	1	2	ENSXETG00000033591
ENSXETG00000033592	1959	0	0	0	0	1	1	0	1	0	0	0	0	
ENSXETG00000033593	1470	13	15	13	4	0	1	2	1	0	5	3	3	ENSXETG00000033593
ENSXETG00000033594	833	0	0	0	0	0	0	0	0	0	0	0	0	ENSXETG00000033594
ENSXETG00000033595	699	0	0	0	0	0	0	0	0	0	0	0	1	ENSXETG00000033595
ENSXETG00000033596	1947	0	0	0	0	1	1	15	9	4	0	5	3	ENSXETG00000033596
ENSXETG00000033597	1089	0	0	0	0	0	0	0	0	0	0	0	0	
ENSXETG00000033598	2765	0	0	0	0	0	0	0	0	0	0	0	0	
ENSXETG00000033599	312	0	0	0	19	7	4	3	0	0	77	58	48	ENSXETG00000033599
ENSXETG00000033600	672	4	6	9	12	21	14	40	43	27	68	65	51	ENSXETG00000033600
ENSXETG00000033601	807	0	0	0	0	0	0	0	0	0	0	0	0	
ENSXETG00000033602	4473	0	0	0	0	0	2	2	3	4	2	1	0	ENSXETG00000033602
ENSXETG00000033603	510	0	0	0	0	0	1	0	1	0	1	0	1	ENSXETG00000033603
ENSXETG00000033604	219	0	0	0	0	0	0	1	1	0	0	0	0	U3
ENSXETG00000033605	2328	0	0	0	0	0	0	0	0	0	0	0	0	
ENSXETG00000033606	426	0	0	0	0	0	0	0	0	0	0	0	0	
ENSXETG00000033607	2861	668	926	807	1609	1189	1415	2617	2833	1774	1955	1395	1474	ENSXETG00000033607
ENSXETG00000033608	411	123	207	174	0	0	1	32	29	15	0	2	1	ENSXETG00000033608
ENSXETG00000033609	97	0	0	0	0	0	0	0	0	0	0	0	0	
ENSXETG00000033610	1536	151	207	173	1084	911	82	221	284	161	392	252	272	ENSXETG00000033610
ENSXETG00000033611	471	0	0	0	0	0	0	0	0	0	1	0	0	SYCE2
ENSXETG00000033612	3081	10	7	10	33	26	44	208	230	133	54	45	52	ENSXETG00000033612
ENSXETG00000033613	2084	639	925	786	929	669	1672	122	144	75	1497	1078	1235	dhdh
ENSXETG00000033614	214	0	0	0	0	0	0	0	0	0	0	0	0	
ENSXETG00000033615	158	0	0	0	0	0	0	0	0	0	0	0	0	
ENSXETG00000033616	1620	0	0	0	0	0	0	0	0	0	0	0	0	
ENSXETG00000033617	3726	237	323	286	1150	897	1825	456	490	353	831	601	657	ENSXETG00000033617
ENSXETG00000033618	474	0	0	0	0	0	0	0	0	0	0	0	0	
ENSXETG00000033619	1158	0	0	2	0	0	0	0	0	0	0	0	0	
ENSXETG00000033620	1401	29	29	22	50	40	72	75	104	53	50	34	53	ENSXETG00000033620
ENSXETG00000033621	1017	0	0	0	0	0	0	0	0	0	0	0	0	
ENSXETG00000033622	1020	0	0	0	0	0	0	0	0	0	0	0	0	
ENSXETG00000033623	3450	5	12	12	22	23	25	116	153	86	63	58	62	COL28A1
ENSXETG00000033624	818	1099	1552	1407	1143	790	2151	1294	1464	971	929	694	676	R3HDM4
ENSXETG00000033625	1233	6	8	3	8	7	22	39	40	19	48	28	30	KCNG3
ENSXETG00000033626	960	0	0	0	0	0	0	0	0	0	0	0	0	
ENSXETG00000033627	3924	0	0	0	0	0	0	0	0	0	0	0	0	
ENSXETG00000033628	919	305	473	393	541	411	645	925	1057	642	712	523	510	ENSXETG00000033628
ENSXETG00000033629	3051	33	47	30	50	38	54	97	129	72	70	49	76	INO80D
ENSXETG00000033630	957	0	0	0	3	0	1	0	0	0	2	1	0	ENSXETG00000033630
ENSXETG00000033631	285	5	5	5	29	28	60	127	191	98	126	70	113	fxyd6
ENSXETG00000033632	510	0	0	0	0	0	0	0	0	0	0	0	0	
ENSXETG00000033633	1068	212	289	244	123	81	212	194	267	152	56	45	41	ENSXETG00000033633
ENSXETG00000033634	1434	0	1	0	9	10	2	3	0	3	8	9	7	ENSXETG00000033634
ENSXETG00000033635	936	0	0	0	0	0	0	0	0	0	0	0	0	
ENSXETG00000033636	119	0	0	0	0	0	0	0	0	0	0	0	0	
ENSXETG00000033637	214	0	0	0	0	0	0	0	0	0	0	0	0	
ENSXETG00000033638	166	0	0	0	0	0	0	0	0	0	0	0	0	
ENSXETG00000033639	312	0	0	0	0	0	0	0	0	0	1	0	2	hist1h4c
ENSXETG00000033640	963	35	38	44	26	22	85	40	75	45	36	30	32	ENSXETG00000033640
ENSXETG00000033641	474	0	0	0	0	0	0	0	0	0	0	0	0	ENSXETG00000033641
ENSXETG00000033642	312	0	0	0	0	0	0	0	0	0	0	0	0	
ENSXETG00000033643	1479	3	1	5	7	13	6	3	2	0	4	1	3	ENSXETG00000033643
ENSXETG00000033644	957	2	16	5	6	8	46	108	117	64	42	32	27	ENSXETG00000033644
ENSXETG00000033645	513	0	0	0	0	0	0	0	0	0	0	0	0	
ENSXETG00000033646	3611	424	599	572	998	740	1074	1427	1502	947	1158	821	930	ercc3
ENSXETG00000033647	2340	532	770	691	1269	916	2483	1596	1768	1052	964	684	735	dis3l2
ENSXETG00000033648	768	16	25	23	24	29	7	51	40	22	49	27	28	ENSXETG00000033648
ENSXETG00000033649	912	14	16	10	6	12	15	73	83	51	27	15	31	ENSXETG00000033649
ENSXETG00000033650	2748	6	4	7	7	3	4	2	0	0	5	4	4	ENSXETG00000033650
ENSXETG00000033651	1710	103	128	104	82	55	143	161	209	122	82	75	97	slc25a51
ENSXETG00000033652	2028	9	14	13	67	60	146	480	623	366	166	145	173	GUCY1A3
ENSXETG00000033653	684	0	0	0	0	0	0	0	1	0	0	0	0	ENSXETG00000033653
ENSXETG00000033654	119	0	0	0	1	0	0	0	0	0	0	0	0	5S_rRNA
ENSXETG00000033655	774	1	0	1	14	11	8	21	13	17	20	12	10	AQP4
ENSXETG00000033656	522	0	0	0	0	0	0	0	0	0	0	0	0	
ENSXETG00000033657	1986	56	97	77	191	145	314	462	520	337	458	340	397	ENSXETG00000033657
ENSXETG00000033658	645	0	0	0	0	1	0	0	1	0	0	2	3	ENSXETG00000033658
ENSXETG00000033659	296	0	0	0	0	0	0	0	0	0	0	0	0	
ENSXETG00000033660	777	0	0	0	0	0	0	2	3	2	0	0	0	
ENSXETG00000033661	567	0	1	1	2	0	0	0	0	0	0	0	0	ENSXETG00000033661
ENSXETG00000033662	1839	0	0	0	0	1	0	0	0	0	0	0	0	TERB1
ENSXETG00000033663	3129	25	37	31	110	85	192	443	493	246	374	272	270	GTF2IRD1
ENSXETG00000033664	5394	0	7	1	65	51	121	689	754	512	312	224	234	SNED1
ENSXETG00000033665	672	0	0	0	0	0	0	0	0	0	0	0	0	
ENSXETG00000033666	1488	55	110	81	174	114	322	115	126	69	98	52	76	ENSXETG00000033666
ENSXETG00000033667	951	129	169	164	116	87	150	247	317	169	135	110	102	ENSXETG00000033667
ENSXETG00000033668	1137	8	11	12	43	54	105	280	290	179	162	126	145	ENSXETG00000033668
ENSXETG00000033669	2577	0	0	0	0	0	0	0	0	0	0	0	0	
ENSXETG00000033670	119	0	0	0	0	0	0	0	0	0	0	0	0	
ENSXETG00000033671	3888	1	1	0	25	15	20	495	560	315	64	27	56	ZNF536
ENSXETG00000033672	1509	17	19	19	46	31	165	22	17	14	73	48	66	ENSXETG00000033672

ENSXETG00000033673	3426	0	0	1	7	2	4	24	32	26	14	8	10	fam155b
ENSXETG00000033674	1059	0	0	0	0	2	0	0	1	1	0	0	0	
ENSXETG00000033675	930	0	0	0	0	0	0	0	0	0	0	0	0	
ENSXETG00000033676	694	0	1	0	0	0	3	14	25	8	24	21	18	ENSXETG00000033676
ENSXETG00000033677	1039	25	45	33	225	167	62	1385	1661	940	1504	1077	1153	ENSXETG00000033677
ENSXETG00000033678	1849	1	1	4	10	6	2	3	9	2	48	46	55	npy4r
[truncated: 5,522,212 more chars]
